# A Chemoselective Polarity‐Mismatched Photocatalytic C(sp^3^)−C(sp^2^) Cross‐Coupling Enabled by Synergistic Boron Activation**

**DOI:** 10.1002/anie.202310462

**Published:** 2023-09-13

**Authors:** Jeremy Brals, Thomas M. McGuire, Allan J. B. Watson

**Affiliations:** ^1^ EaStCHEM School of Chemistry University of St Andrews Purdie Building, North Haugh St Andrews KY16 9ST UK; ^2^ AstraZeneca Darwin Building, Unit 310 Cambridge Science Park, Milton Road Cambridge CB4 0WG UK

**Keywords:** Boron, Chemoselectivity, Cross-Coupling, Crossover, Photocatalysis

## Abstract

We report the development of a C(sp^3^)−C(sp^2^) coupling reaction using styrene boronic acids and redox‐active esters under photoredox catalysis. The reaction proceeds through an unusual polarity‐mismatched radical addition mechanism that is orthogonal to established processes. Synergistic activation of the radical precursor and organoboron are critical mechanistic events. Activation of an *N*‐hydroxyphthalimide (NHPI) ester by coordination to boron enables electron transfer, with decomposition leading to a nucleofuge rebound, activating the organoboron to radical addition. The unique mechanism enables chemoselective coupling of styrene boronic acids in the presence of other alkene radical acceptors. The scope and limitations of the reaction, and a detailed mechanistic investigation are presented.

## Graphical abstract

We report a rare polarity‐mismatched radical addition reaction using styrene boronic acids and redox‐active *N*‐hydroxyphthalimide (NHPI) esters under photoredox catalysis. The reaction displays broad scope with mechanistic investigations supporting a unique synergistic activation pathway that displays chemoselectivity for styrene boronic acids in the presence of other SOMOphiles. SOMO=singly occupied molecular orbital.

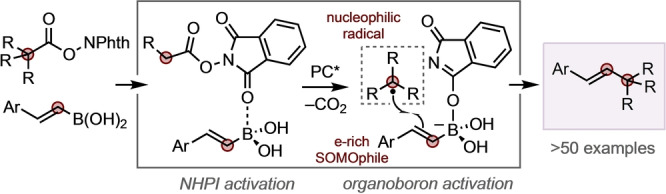

Radical addition to alkenes is a powerful platform for C−C bond formation.[^1^, ^2^, ^3^] Classic Giese‐type reactions^[4]^ using stoichiometric radical initiators have seen significant innovation through the development of catalytic approaches under contemporary photoredox[^5^, ^6^, ^7^, ^8^] and electrochemical conditions.[^9^, ^10^, ^11^] The mechanistic requirements of these processes are relatively well understood, specifically with respect to radical philicity (Scheme 1a):[^12^, ^13^] aligning radical and alkene polarity is key to successful bond formation. Generally, nucleophilic radicals engage electrophilic alkene SOMOphiles and vice versa,[^12^, ^13^, ^14^] with the main exception arising from ambiphilic radicals, which can engage both electron‐rich and electron‐poor alkene SOMOphiles.[^12^, ^13^] Pertinent examples of these reactivity principles can be seen in radical additions to well‐known electron‐rich π‐systems, such as vinyl boron compounds where *ipso*‐substitution takes place using the electrophilic radicals generated from α‐halocarbonyls, as developed by Leonori,^[15]^ or the Togni reagent, as developed by Koike and Akita (Scheme 1b).[^16^, ^17^]

**Scheme 1:**
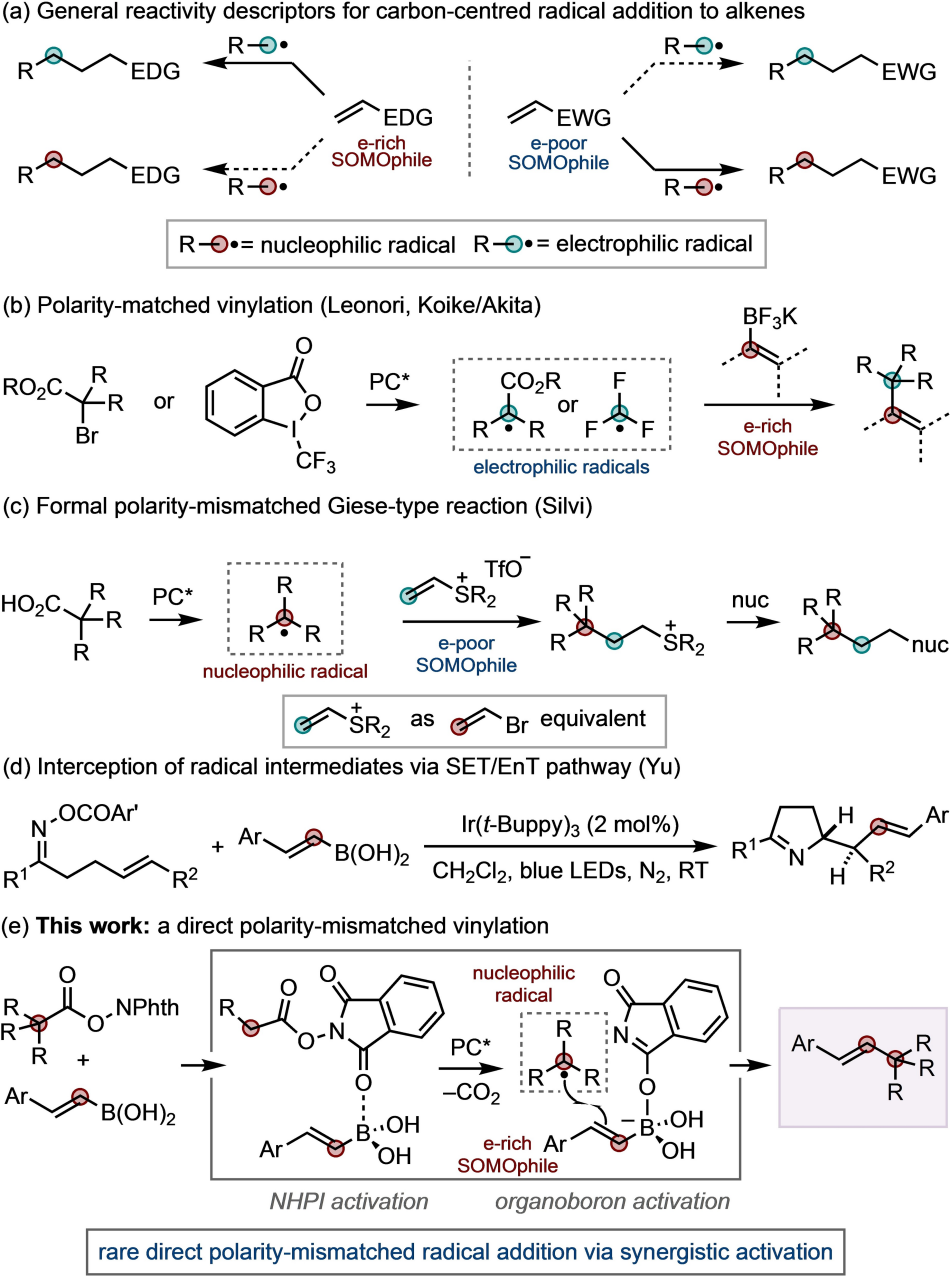
Radical additions to alkenes via polarity‐matched and ‐mismatched pathways. (a) General description of polarity requirements. (b) Examples of polarity‐matched vinylation using organoboron reagents. (c) An approach to formal polarity‐mismatched radical addition. (d) Interception of radical intermediates using organoboron reagents. (e) This work ‐ a direct polarity mismatched C(sp^3^)−C(sp^2^) coupling.

Examples of polarity‐mismatched radical addition to alkenes are rare. This has inspired development of strategies for formal polarity‐mismatched bond formations. For example, a recent approach by Silvi and co‐workers used vinyl sulfonium salts as vinyl halide equivalents, allowing preparation of products that display the same downstream reactivity profiles (i.e., use as an electrophile for subsequent alkylations) but without the reactivity issues of, for example, vinyl bromide (Scheme 1c).^[18]^ This innovative approach allows formal access to the polarity‐mismatched products; however, from a radical philicity perspective, this reaction remains a polarity‐matched addition. Yu has developed cascade processes based on single electron transfer (SET) and energy transfer (EnT, e.g., Scheme 1d) and hydrogen atom transfer (HAT) where radical intermediates are intercepted by organoboron reagents.^[19]^ Alternative approaches have been developed using transition metals, for example, the Pd(I)‐mediated methods developed by Wu and Loh^[20]^ and Gevorgyan.^[21]^

Here we show a direct polarity‐mismatched radical addition of a nucleophilic radical to an electron‐rich SOMOphile (Scheme 1d).

The developed reaction is a C(sp^3^)−C(sp^2^) radical‐polar crossover coupling[^22^, ^23^] (arylalkenylation) reaction of nucleophilic alkyl radicals generated from photoredox‐induced decomposition of redox‐active *N*‐hydroxyphthalimide (NHPI) esters[^24^, ^25^, ^26^] using styrene boronic acids as a electron‐rich π‐system.^[27]^ The key to this overall process was control of boron speciation:^[28]^ synergistic activation of the NHPI ester by the organoboron and activation of the organoboron by nucleofuge rebound.

Based on our previous work in nucleophile‐nucleophile couplings using organoboron compounds under transition metal catalysis,[^29^, ^30^, ^31^, ^32^, ^33^] we were intrigued by the prospect of identifying a nucleophile‐nucleophile coupling using organoboron compounds with nucleophilic radicals. We selected NHPI ester **2** as a precursor to a nucleophilic alkyl radical. A directed organoboron screening campaign identified a hit reaction where commercial styrene boronic acid **1 a** delivered the desired product **3** in 63 % yield upon treatment with Ru(bpy)_3_(PF_6_)_2_ under irradiation with blue LEDs (Scheme 2); however, while noteworthy, this result was found to be irreproducible. Thorough purification of **1 a** gave consistent results but very low yields. The main impurity in commercial **1 a** is catechol; addition of catalytic quantities of catechol to the reaction restored reactivity and importantly, gave consistent and reproducible results.

**Scheme 2:**
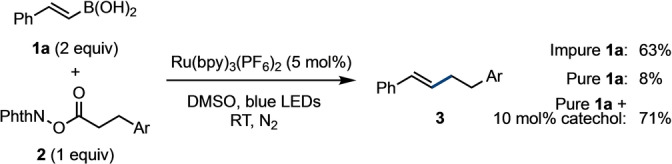
Hit reaction from screening campaign. Ar=*p*‐F−C_6_H_4_. Blue LED wavelength=456 nm. Yields determined by ^1^H NMR using an internal standard. bpy=2,2′‐bipyridine, DMSO=dimethylsulfoxide, LED=light emitting diode, PhthN=phthalimide.

Since catechol has an oxidation potential similar to some common additives used as electron shuttles in photoredox reactions,[^34^, ^35^, ^36^] while unusual, we hypothesized that catechol had the same function. Indeed, based on this initial hit reaction, the system was modified to use the cyclohexyl NHPI ester **4** and optimized to deliver the standard conditions shown in Table 1, which delivered excellent conversion to the expected cross‐coupled product **5** (entry 1).

**Table 1:** Reaction development.^[a]^

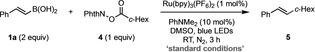		
Entry	Deviation from “standard conditions”	Yield 5 [%]^[b]^
1	None	96, 80^[c]^
2	Remove Ru(bpy)_3_(PF_6_)_2_	0
3	Run in dark	0
4	Remove PhNMe_2_	16
5	Using styrenyl Bpin (**1 b**)	28
6	Using styrenyl Bcat (**1 c**)	46
7	Using styrenyl BF_3_K (**1 d**)	0
8	Using styrenyl BMIDA (**1 e**)	0

[a] Reactions performed on 0.2 mmol scale. [b] Determined by ^1^H NMR analysis using an internal standard. [c] Isolated yield. Ar (aryl)=4‐(MeO)−C_6_H_4_.

Selected optimization data are worth noting. Firstly, the reaction operates under photoredox conditions ‐ removal of the photocatalyst or light source completely inhibits the reaction (entries 2 and 3). Next, the reaction requires an electron shuttle, with PhNMe_2_ the most effective (see ESI for full details of the additive screen). Removal of the electron shuttle significantly diminished efficiency, although a small background reaction was noted. Finally, boron speciation was a key component ‐ the boronic acid was the most effective, with Bpin and Bcat esters less effective (entries 5 and 6). Contrary to previous radical additions using vinyl organoboron reagents,[^15^, ^16^, ^17^] the BF_3_K and BMIDA were completely unreactive (entries 7 and 8). This, along with other evidence, revealed important information on the mechanism of the reaction (vide infra). Finally, the reaction was incompatible with arylboronic acids (see ESI).

The generality of the reaction was explored by application to a panel of substrates (Scheme 3). A broad scope was observed in the NHPI component, with primary, secondary, and tertiary alkyl radicals, including well‐known nucleophilic α‐amino radicals (e.g., **23**, **24**, **30**, **31**) and α‐oxo radicals (e.g., **33**, **34**) accommodated. Functional groups that are potentially reactive with radical species were compatible, including an alkyl bromide (**19**), alkenes (**20**, **21**), and alkynes (**16**, **22**). Importantly, in contrast to existing polarity‐matched reactions, electrophilic radicals (e.g., α‐carboxy) as well as benzylic radicals were unsuccessful (see ESI for a full description of limitations).[^16^, ^17^, ^18^] Allyl radicals were unsuccessful (**41**). Similarly, the styrene component was broadly tolerant of functional group electronic and regiochemical variation. A dienyl example underwent the expected *ipso*‐substitution in low yield (**58**). Interception of the putative benzylic radical and/or carbocation (vide infra) was not observed (e.g., **51**, **59**, **60**). Lastly, the styrene boronic acid starting materials were pure *E*‐stereoisomers and products were generally isolated as the pure *E*‐stereoisomer; however, several products (e.g., **48**, **49**, **55**) displayed a lower *E*/*Z* ratio, possibly due to photocatalytic isomerization.[^37^, ^38^, ^39^] Control experiments also suggested in situ isomerization (see ESI).

**Scheme 3:**
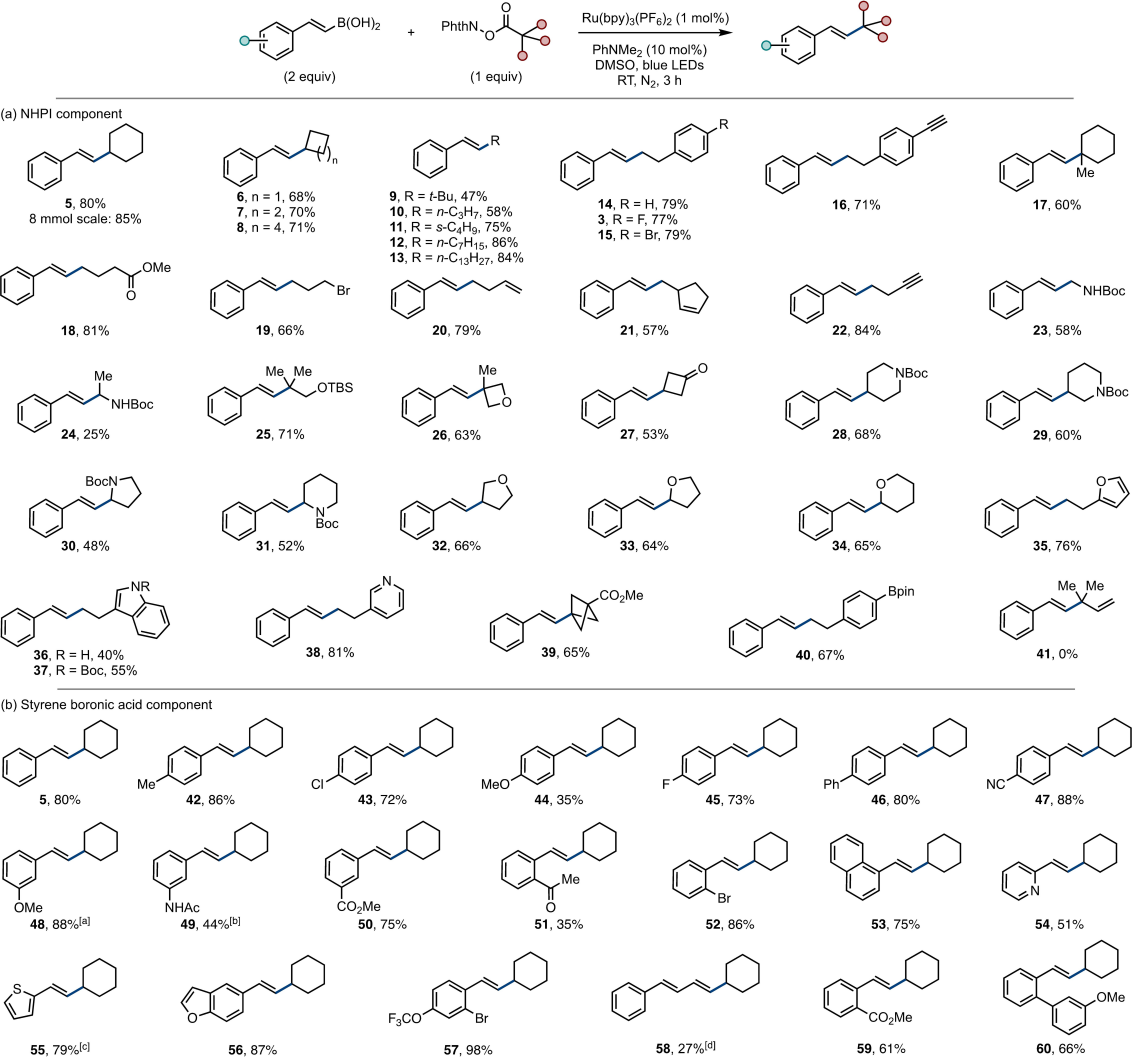
Example scope of the polarity‐mismatched radical coupling. Isolated yields. *E : Z* >20 : 1 unless indicated. [a] *E : Z*=16.5 : 1. [b] *E : Z*=12.5 : 1. [c] *E : Z*=7.3 : 1. [d] *(E,E)*:*(Z,E):(E,Z):(Z,Z)*=1 : 1:0.1:0.1. Boc=*tert*‐butoxycarbonyl, pin=pinacolato, TBS=*tert*‐butyldimethylsilyl.

The unusual reactivity of this system compelled deeper investigation (Scheme 4). Firstly, support for the formation of the expected alkyl radical from the precursor NHPI ester was achieved by using radical clock NHPIs (Scheme 4a). Under standard conditions, use of alkene substituted NHPI **61** delivered a ca. 1 : 3 mixture of **62** and ring closed analogue **63**. Likewise, cyclopropyl NHPI **64** led exclusively to ring opened product **20**.

**Scheme 4:**
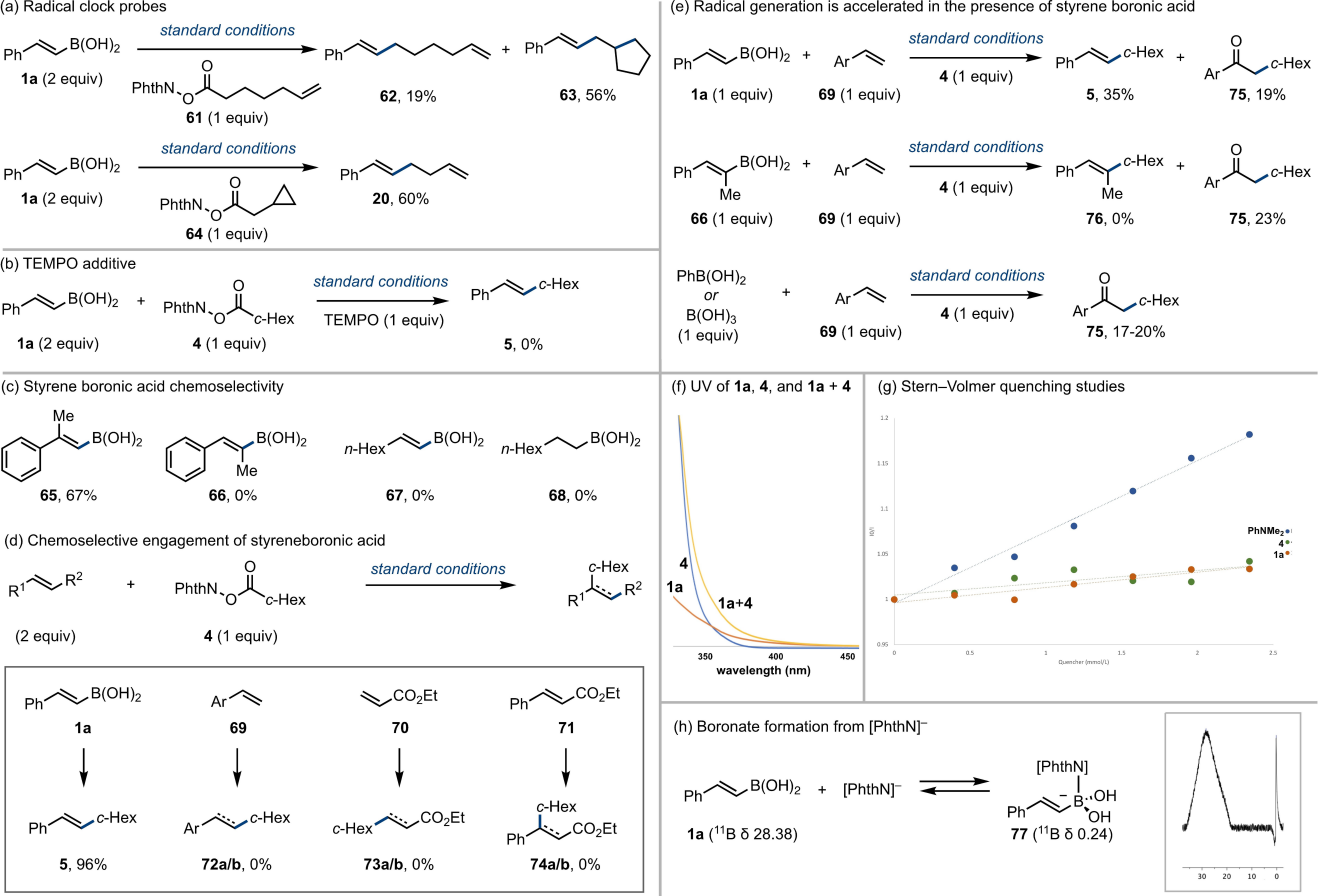
Mechanistic investigations. (a) Radical clock experiments. (b) TEMPO additive. (c) Reactivity of alkenyl and alkylboronic acids. (d) Comparison between reactivity differences of alkene radical acceptors. (e) Promotion of NHPI decomposition and the reaction with styrene in the presence of boronic or boric acid. (f) Stern–Volmer quenching studies. (g) UV of **1 a**, **4**, and **1 a+4** showing bathochromic shift. (h) Boronate formation from [PhthN]^−^. Yields determined by ^1^H NMR using an internal standard. Ar=*p*‐F−C_6_H_4_.

Addition of TEMPO to the standard reaction completely inhibited the reaction (Scheme 4b). Greater insight was found in the reactivity profiles of the organoboron component (Scheme 4c). Styrene boronic acid **1 a** undergoes the reaction to deliver **5** in high conversion. Substitution of the alkene was tolerated but only in the α‐position. Styrene **65** delivered the expected product in 67 % yield; however, substitution on the β‐carbon (i.e., **66**) gave no conversion. The origin of this reactivity arises from an increase in LUMO energy for **66** due to a sterically induced deconjugation of the π‐system. These effects have been exploited for contra‐thermodynamic alkene isomerization.[^37^, ^38^, ^39^] In contrast to polarity‐matched processes,[^15^, ^16^, ^17^] this leads to exclusive selectivity for styrene boronic acids, with alkyl substituted alkenyl boronic acids, such as **67**, unreactive, and establishes a previously unknown chemoselectivity element in these radical addition processes. Alkylboronic acids (e.g., **68**) were also unreactive.

The unique chemoselectivity characteristics of this reaction were further demonstrated by variation of the alkene (Scheme 4d). The nucleophilic alkyl radical liberated from NHPI ester **4** would be expected to undergo effective reaction with ambiphilic radical acceptors, such as styrenes, and electrophilic acceptors, such as acrylate esters;[^12^, ^13^] however, this was not the case. While styrene boronic acid **1 a** delivers **5** in good yield, styrene **69**, ethyl acrylate **70**, and ethyl cinnamate **71** were completely unreactive.

In the reactions above where no product was observed (i.e., using alkenes **66**–**71**), the majority of the NHPI ester was recovered, suggesting the reaction failed to initiate or turnover in the absence of the styrene boronic acid. Increased quantities of photocatalyst and PhNMe_2_ did not induce any product formation with **66**–**71** (see ESI). We therefore hypothesized that the styrene boronic acid was involved in radical generation from the NHPI ester.

The role of the styrene boronic acid in radical initiation was established in a series of competition experiments (Scheme 4e). While styrene **69** is unreactive under standard conditions, in the competition reaction between **1 a** and **69**, a ca. 2 : 1 ratio of **5** and **75** was observed. Conversion of styrenes to ketones such as **75** is a known reaction under photoredox conditions using nucleophilic radicals, proceeding via radical addition to the styrene, oxidation of the benzylic radical to the carbocation, and interception by DMSO leading to Kornblum‐type oxidation;[^40^, ^41^] however, under the developed conditions, the styrene is only reactive in the presence of the styrene boronic acid. The same effect was observed using styrene boronic acid **66** (Scheme 4c), which was unreactive to the cross‐coupling, but enabled similar levels of conversion of **69** to ketone **75**. Boric acid and arylboronic acids (which were inert to the coupling process) also promoted formation of **75**; however, the same experiments using **70** and **71** did not lead to **75**, **73 a/b**, **74 a/b**, or any oxidized derivatives (see ESI), surprisingly indicating that the nucleophilic radical does not react with these electron‐poor alkenes. Similarly, electron‐deficient styrenes did not react with the nucleophilic radical in the presence and absence of B(OH)_3_ or PhB(OH)_2_ (see ESI). Collectively, these data suggested that the boronic acid activated the NHPI ester to photocatalytic reduction, similar to the activation of B_2_Cat_2_ proposed by Aggarwal for decarboxylative borylation,^[42]^ and Chen on radical addition to ketoacids,^[43]^ and Indeed, UV studies suggested a Lewis pairing interaction between **1 a** and **4** (Scheme 4f). Since the reaction works moderately with the equivalent styrenyl Bpin and Bcat (see Table 1, entries 5 and 6), H‐bonding activation of the NHPI is unlikely.^[19]^ In addition, since the reaction does not operate with the equivalent styrenyl BF_3_K and BMIDA, there is a clear requirement for a neutral organoboron reagent (see Table 1, entries 7 and 8). Accordingly, a Lewis pairing interaction between NHPI is proposed, which is consistent with both Aggarwal's and Chen's proposed activation model.[^42^, ^43^]

Stern–Volmer studies confirmed that only PhNMe_2_ quenches the excited photocatalyst (Scheme 4g). A range of other photocatalysts were assessed, including those capable of energy transfer, but delivered no reaction (see ESI). ^11^B NMR studies supported boronate formation from **1 a** and phthalimide anion (Scheme 4h). Light ON/OFF experiments supported a lack of chain process and the role of PhNMe_2_ as catalytic electron shuttle (see ESI).

Based on all the above data, we propose a reaction mechanism as shown in Scheme 5. Consistent with established oxidation/reduction potentials (indicated),[^5^, ^34^, ^36^, ^44^, ^45^] excitation of [Ru(bpy)_3_]^2+^ (**78**) leads to [Ru(bpy)_3_]^2+^* (**79**), which is reduced by PhNMe_2_ to give radical anion **80** and aminium radical **81**. Activation of NHPI **82** by coordination to **83** facilitates reduction by **80** to regenerate the ground state photocatalyst **78**. Decarboxylation of **84** and nucleofuge rebound of phthalimide anion leads to alkyl radical **85** and simultaneous activation of **84** as boronate **86**, which is now primed for radical addition giving benzylic radical **87**. Radical‐polar crossover, via oxidation of **87** with radical cation **81**, regenerates the electron shuttle and benzyl carbocation **88**. Elimination then delivers the cross‐coupled product **89**.

**Scheme 5:**
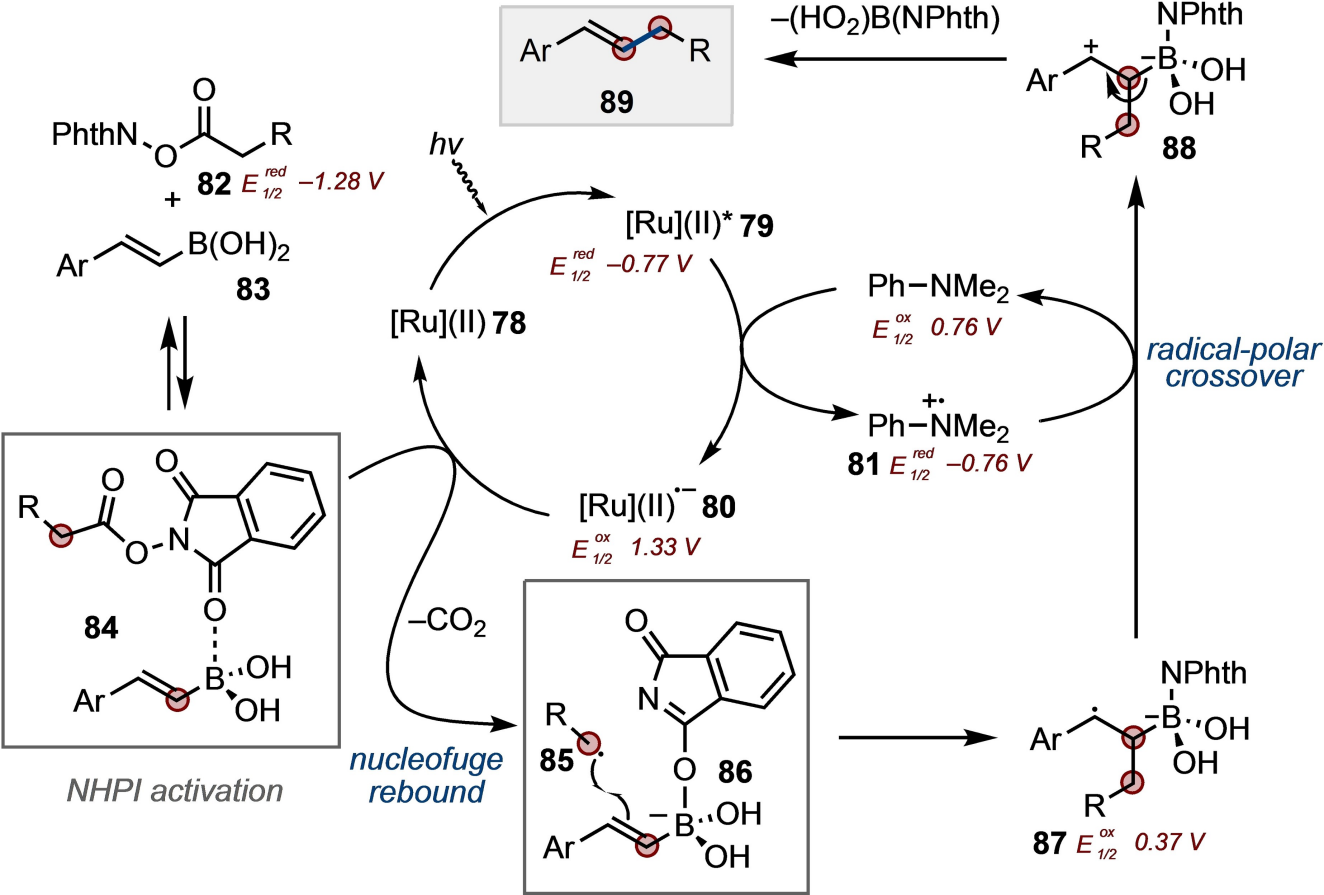
Proposed mechanism. [Ru](II)=[Ru(bpy)_3_]^2+^ (counteranions not shown for clarity).

The nucleofuge rebound removes the possibility of uncontrolled activation of the boronic acid as the boronate derivative (i.e., **86**). Interception of the benzylic carbocation **88** with free boronate, such as the alkene difunctionalization reactions developed by Molander, Rueping, and others[^46^, ^47^, ^48^, ^49^] is therefore avoided, further highlighting the distinct mechanism of this polarity‐mismatched coupling.

In summary, we have developed a rare polarity‐mismatched radical addition reaction. The developed conditions allow the straightforward arylalkenylation of nucleophilic radicals with nucleophilic styrene boronic acids under photoredox conditions using an electron shuttle to mediate a radical‐polar crossover process. Mechanistic investigations support a synergistic activation pathway where the NHPI ester is activated by the organoboron towards electron transfer. The resulting decomposition allows a nucleofuge rebound of phthalimide anion, which activates the boronic acid to radical addition. This unique mechanism displays unusual reactivity, enabling chemoselective coupling of styrene boronic acids in the presence of other well‐known alkene radical acceptors.

## Supporting Information

The research data supporting this publication can be accessed at https://doi.org/10.17630/33380f7f‐28cd‐45b8‐b55b‐dd6b83651e89. The authors have cited additional references within the Supporting Information.[^50^, ^51^, ^52^, ^53^, ^54^, ^55^, ^56^, ^57^, ^58^, ^59^, ^60^, ^61^, ^62^, ^63^, ^64^, ^65^, ^66^, ^67^, ^68^, ^69^, ^70^, ^71^, ^72^, ^73^, ^74^, ^75^, ^76^, ^77^, ^78^, ^79^, ^80^, ^81^, ^82^, ^83^, ^84^, ^85^, ^86^, ^87^, ^88^, ^89^, ^90^, ^91^, ^92^, ^93^, ^94^, ^95^, ^96^, ^97^, ^98^, ^99^, ^100^, ^101^, ^102^, ^103^, ^104^, ^105^, ^106^, ^107^, ^108^, ^109^, ^110^, ^111^, ^112^, ^113^, ^114^, ^115^, ^116^, ^117^, ^118^, ^119^, ^120^, ^121^, ^122^, ^123^, ^124^, ^125^, ^126^, ^127^, ^128^, ^129^, ^130^, ^131^, ^132^, ^133^, ^134^, ^135^, ^136^, ^137^, ^138^, ^139^, ^140^, ^141^, ^142^, ^143^, ^144^, ^145^, ^146^, ^147^, ^148^, ^149^, ^150^, ^151^, ^152^, ^153^, ^154^, ^155^, ^156^, ^157^]

## Conflict of interest

The authors declare no conflict of interest.

## Supporting information

As a service to our authors and readers, this journal provides supporting information supplied by the authors. Such materials are peer reviewed and may be re‐organized for online delivery, but are not copy‐edited or typeset. Technical support issues arising from supporting information (other than missing files) should be addressed to the authors.

Supporting Information

## Data Availability

The data that support the findings of this study are openly available in University of St Andrews at https://doi.org/10.17630/33380f7f‐28cd‐45b8‐b55b‐dd6b83651e89, reference number 1.
