# Supplementary material for: A Chemoselective Polarity‐Mismatched Photocatalytic C(sp3)−C(sp2) Cross‐Coupling Enabled by Synergistic Boron Activation
Source: Angew Chem Int Ed Engl. 2023 Sep 13;62(42):e202310462. doi: 10.1002/anie.202310462 (PMC10952440; doi:10.1002/anie.202310462)

Angewandte Chemie
Eine Zeitschrift der Gesellschaft Deutscher Chemiker

Supporting Information

A Chemoselective Polarity-Mismatched Photocatalytic C(sp^3)–C(sp^2) Cross-Coupling Enabled by Synergistic Boron Activation

*J. Brals, T. M. McGuire, A. J. B. Watson**

Table of contents

1. GENERAL INFORMATION **3**

2. REACTION OPTIMIZATION **4**

2.1 REACTION OPTIMIZATION WITH **2** 4

2.2 IMPURE BATCH OF **1A** 7

2.3 REACTION OPTIMIZATION WITH **4** 11

3. GENERAL PROCEDURES **14**

3.1 SYNTHESIS OF NHPI ESTERS 14

3.2 SYNTHESIS OF STYRENYL BORONIC ACIDS 16

3.3 PROCEDURE FOR THE DEVELOPED REACTION 18

4. CONTROL REACTIONS AND MECHANISTIC INVESTIGATIONS **20**

4.1 GENERAL COMMENTS 20

4.2 REACTION WITH (*Z*)-STYRENYL BORONIC ACID 20

4.3 REACTION WITH A- AND B-SUBSTITUTED STYRENE BORONIC ACIDS. 20

4.4 COMPETITION REACTIONS 21

4.5 RADICAL CLOCK EXPERIMENTS 25

4.6 TEMPO EXPERIMENT 25

4.7 ON/OFF EXPERIMENTS 25

4.8 REACTION SCALE-UP 26

4.9 EMISSION QUENCHING AND STERN-VOLMER LINEARIZATION 27

4.10 UV-VIS ABSORPTION 29

4.11 ^{1}H AND ^{11}B NMR: FORMATION OF BORONATE *IN SITU*. 30

5. UNSUCCESSFUL SUBSTRATES **31**

5.1 LOW YIELDING SUBSTRATE 31

5.2 UNSUCCESSFUL STARTING MATERIALS 32

6. CHARACTERIZATION **33**

6.1 NHPI ESTERS 33

6.2 BORONIC ACID STARTING MATERIALS 62

6.3 PRODUCTS FROM THE DEVELOPED COUPLING REACTION 105

7. REFERENCES **135**

8. NMR SPECTRA **140**

1. General information

Reagents and solvents were obtained from commercial suppliers and were not purified further unless specified. Purification (where specified) was performed following the standard procedures. Chlorotrimethylsilane was dried and distilled over CaH_2. Dry solvents (THF, DCM, Et_2O) were provided by a PureSolv SPS-400-5 solvent purification system and stored over 4 Å molecular sieves.

Reactions were carried out in standard borosilicate glassware microwave vials with septum caps. Glassware was either flame-dried under vacuum or allowed to dry in a 180 °C oven for 24 h before use and then sparged with nitrogen. Room temperature was approximately 18 °C. Reactions at elevated temperatures were heated using a sand bath using a temperature probe where the temperature indicated is the temperature of the sand bath. Reactions at low temperature were performed using an ice/water bath (0 °C) or dry ice/acetone bath (–78 °C). Water and/or oxygen-sensitive reactions were carried out in oven-dried glassware under inert atmosphere (N_2) using standard vacuum lines techniques. The light source used was a Kessil LED PR160L-456 nm, referred to as blue LEDs throughout.

TLC was carried out using Merck aluminium-backed silica plates coated with F_{254} fluorescent indicator, analysed under UV light, and developed using aqueous $KMnO_4$ or ethanolic vanillin solutions, where appropriate. Flash column chromatography performed using silica gel (40–62 μm, Fluorochem).

^{1}H, ^{13}C {^{1}H}, ^{19}F {^{1}H} NMR spectra were recorded by either a Bruker AVII 400 (BBFO probe) or AVIII-HD 500 or AVIII 500 with BBFO+ and Prodigy BBFO probes, respectively, at 400-101-376 MHz or at 500-126-377 MHz respectively. ^{11}B NMR spectra were recorded on a Bruker AV300 spectrometer at 96 MHz or on a Bruker AVII 400 spectrometer at 128 MHz. All spectra were recorded at room temperature with the deuterated solvents used as a lock for spectra and internal reference (*d*-chloroform: ^{1}H, 7.26 ppm; ^{13}C, 77.2 ppm; d_6-acetone: ^{1}H, 2.05 ppm, ^{13}C, 29.8 ppm; d_6-dimethylsulfoxide: ^{1}H, 2.50 ppm, ^{13}C, 39.5 ppm; d_3-acetonitrile: ^{1}H, 1.94 ppm, ^{13}C, 1.3 ppm). For ^{11}B NMR, samples were run using a standard borosilicate tube and the spectra baselines corrected during processing unless the sample size prevented an acceptable signal-to-noise ratio, where a quartz NMR tube was used. All ^{11}B NMR spectra were externally referenced to $F_3B{\bullet}OEt_2$ in $CDCl_3$. All chemical shifts (δ) are reported in parts per million (ppm) relative to the residual solvent peak, all coupling constants, *J*, are quoted in Hz and refer to $^{3}J_{HH}$ unless otherwise stated. NMR spectra are reported as follows: chemical shift/ppm (multiplicity, coupling constant(s), number of nuclei). Multiplicity given as app. (apparent), br (broad), s (singlet), d (doublet), t (triplet), q (quartet), quint (quintet), h (hextet), m (multiplet), and combinations thereof. ^{13}C signals adjacent to boron are, in some cases, not observed or reported as a broad signal. Signals which overlap with one another are described as multiplets.

IR spectra were recorded using a Shimadzu IT Affinity-1 Fourier transform IR spectrophotometer with a Specac Quest ATR (diamond puck). Spectra were recorded as films (using $CDCl_3$), as solids, or as neat liquids, as specified. Transmittance is recorded with maximal absorption wavenumbers given as cm^{-1}. Steady-state emission, excitation spectra, and time-resolved emission spectra were recorded at 298 K using an Edinburgh Instruments FS5. Samples were irradiated at 468 nm for both steady-state measurements and time-resolved measurements. UV-Vis absorption spectra were recorded using an Agilent Technologies Cary 3500 Series UV-Vis spectrometer. Mass spectra were recorded on a Bruker micrOTOF benchtop ESI with

either positive or negative electrospray ionisation or EI using a Thermo Mat 900XP, Double Focussing Hi-resolution mass spectrometer at the University of Edinburgh mass spectrometry facility (SIRCAMS).

2. Reaction optimization

2.1 Reaction optimization with **2**

Table 1: Solvent screening.

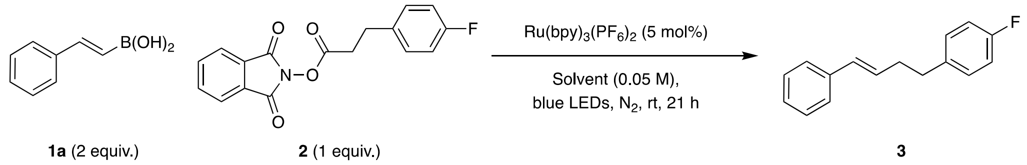

Entry	Solvent	**3** (%)[a]	**2** (%)[a]
1	DMSO	60	0
2[b]	DMSO	59	0
3	MeCN	14	57
4	Acetone	40	9
5	THF	0	88
6	DMF	35	13
7	HFIP	0	74
8	Trifluoroethanol	0	83
9	DCM	11	82
10	DCE	0	88
11	Toluene	0	97
12	TBME	0	92
13	$PhCF_3$	0	90

Reaction run on 0.1 mmol scale in dry and degassed solvents. [a] Determined by ^{19}F NMR using 2-fluoro-4-nitrotoluene as internal standard (added after work up). [b] Reaction time = 3 hours.

Table 2: Control experiments: light source.

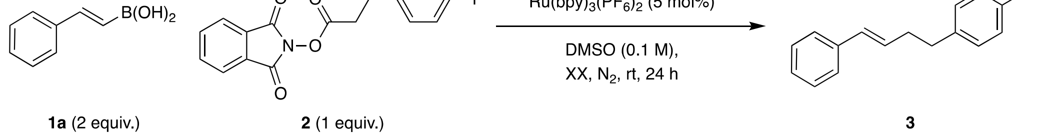

Entry	Conditions	**3** (%)[a]	**2** (%)[a]
1	Daylight	0	97

2	Dark	0	100

Reaction run on 0.1 mmol scale in dry and degassed solvents. [a] Determined by ^{19}F NMR using 2-fluoro-4-nitrotoluene as internal standard (added after work up).

A time study was carried out where the reaction was performed in a J. Youngs NMR tube. An internal standard ($PhCF_3$) was added to allow monitoring of the reaction by ^{19}F {^{1}H} NMR. Time represents the sample irradiation time. It was assumed no reaction was occurring under daylight (see table 2). Note: Reactions were not stirred.

Graph 1: Time study.

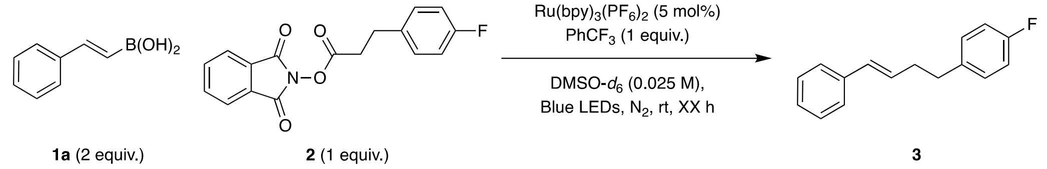

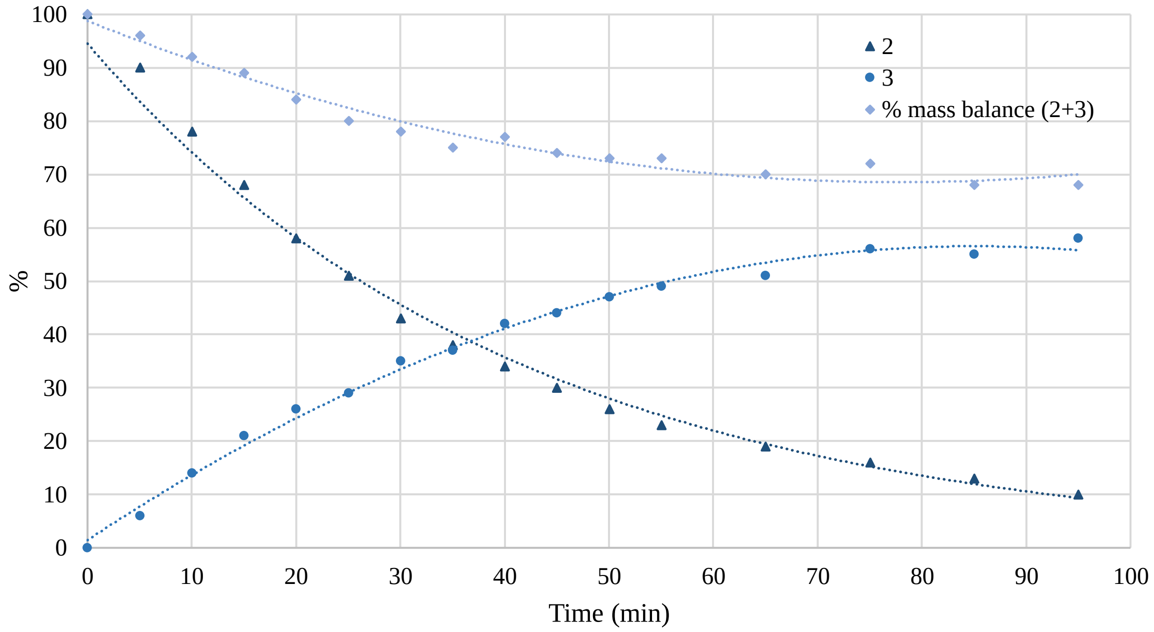

Table 3: Concentration of reaction.

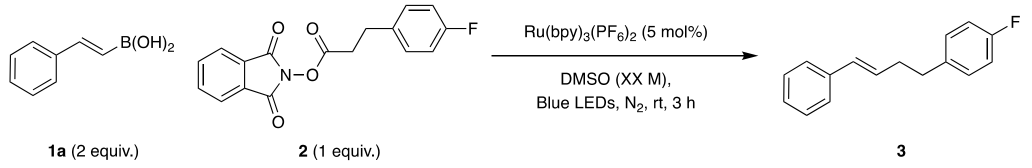

Entry	Concentration	**3** (%)[a]	**2** (%)[a]
1	0.2 M	62	2
2	0.1 M	63 (60)[b]	0
3	0.05 M	59	0

4	0.025 M	57	1

Reaction run on 0.1 mmol scale in dry and degassed DMSO. [a] Determined by ^{19}F NMR using 2-fluoro-4-nitrotoluene as internal standard (added after work up).

Table 4: Stoichiometry of reaction.

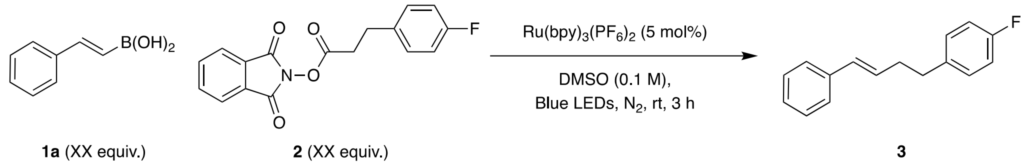

Entry	**1a** (equiv.)	**2** (equiv.)	**3** (%)[a]	**2** (%)[a]
1	1	1	31	48
2	1.5	1	45	25
3	2	1	63 (60)[b]	0
4	3	1	66	0
5	4	1	67	0
6	5	1	67	0
7	1	2	17	57
8	1	2	24	76

Reaction run on 0.1 mmol scale in dry and degassed DMSO. [a] Determined by ^{19}F NMR using 2-fluoro-4-nitrotoluene as internal standard (added after work up). [b] Isolated yield.

Table 5: Control reactions: Reaction atmosphere.

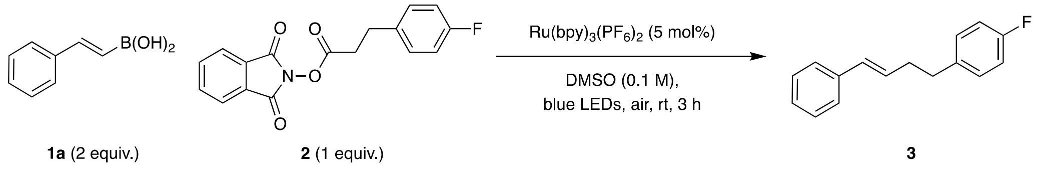

Entry	Atmosphere	**3** (%)[a]	**2** (%)[a]
1	Run under air	21	51
2	Run under air<br>Air bubbled through	23	52

Reaction run on 0.1 mmol scale in dry and degassed DMSO. [a] Determined by ^{1}H NMR using 2-fluoro-4-nitrotoluene as internal standard (added after work up).

2.2 Impure batch of **1a**

The optimization started with a bottle of (*E*)-2-phenylvinylboronic (**1a**) from Sigma Aldrich (S1) that had been in lab storage. Two new bottles were also purchased: one from Sigma Aldrich (S2) and one from Fluorochem (F3).

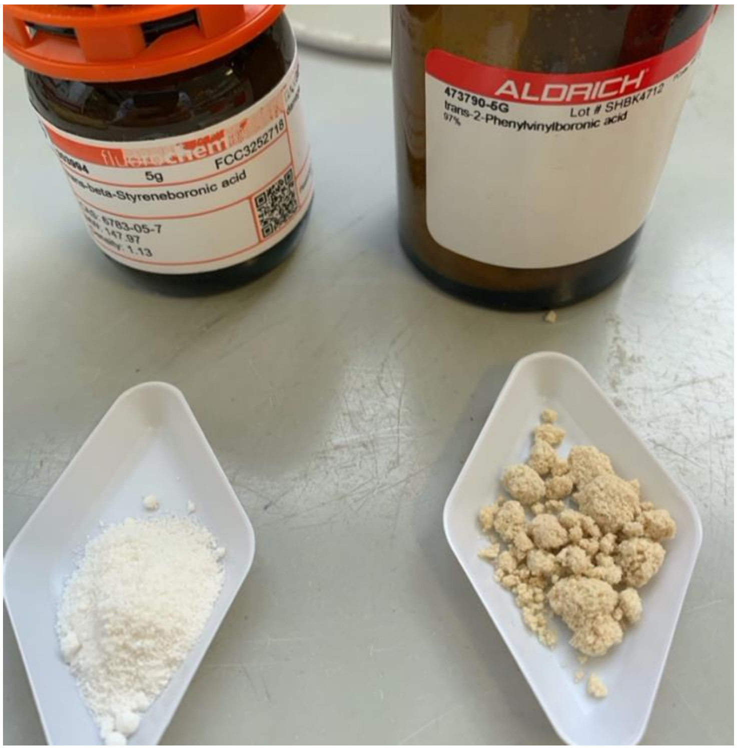

Picture 1: (*E*)-2-phenylvinylboronic acid (**1a**) from F3 (left) and S1 (right).

Table 6: Reaction with different batches of (*E*)-2-phenylvinylboronic acid (**1a**).

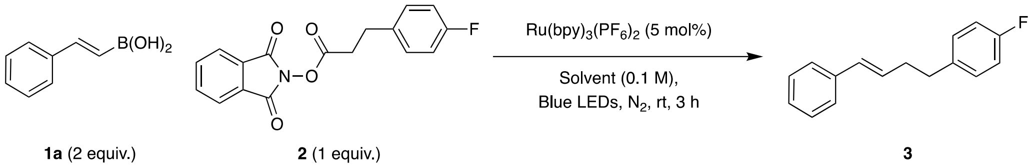

Entry	Batch (**1a**)	Solvent	**3** (%)[a]	**2** (%)[a]
1	S1	DMSO	63 (60)[b]	0
2	S2	DMSO	3	92
3	F3	DMSO	8	84
4[c]	F3	DMSO	7	77
5[d]	F3	DMSO	31	49
6	F3	MeCN	0	94
7[e]	F3	DMSO-d_6	11	82

8	S1 (1 equiv.)<br>F3 (1 equiv.)	DMSO	49	21

Reaction run on 0.1 mmol scale in dry and degassed solvents. [a] Determined by ^{19}F NMR using 2-fluoro-4-nitrotoluene as internal standard (added after work up). [b] Isolated yield. [c] 20 mol% of photocatalyst used. [d] Reaction time = 24 hours. [e] Determined by ^{1}H NMR using 2-fluoro-4-nitrotoluene as internal standard (added to the reaction mixture).

Table 7: First additive screening.

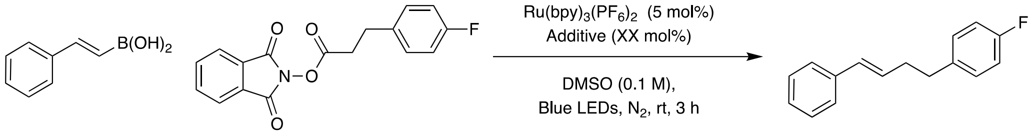

1a (2 equiv.) **2** (1 equiv.) **3**

Entry	Additives	**3** (%)[a]	**2** (%)[a]
1	None	8	84
2	Phenylacetylene (20 mol%)	16	78
3	Catechol (20 mol%)	64	0
4[b]	Catechol (20 mol%)	64	0
5	Catechol (10 mol%)	71	0
6	Catechol (5 mol%)	70	0

Reaction run on 0.1 mmol scale in dry and degassed DMSO. [a] Determined by ^{19}F NMR using 2-fluoro-4-nitrotoluene as internal standard (added after work up). [b] Reaction time = 24 hours.

Table 8: Photocatalyst screening.

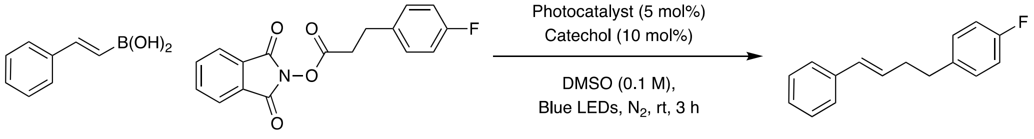

1a (2 equiv.) **2** (1 equiv.) **3**

Entry	Photocatalyst	**3** (%)[a]	**2** (%)[a]
1	None	0	98
2	$Ru(bpy)_3(PF_6)_2$	70	0
3	4-CzIPN	37	0
4	$(Ir(dF(CF_3)ppy)_2(dtbpy))PF_6$	8	33
5	10-phenylphenothiazine	1	93
6	$Ir(ppy)_3$	63	0

7	Eosin Y	0	94
8	Methylene blue	0	95
9	Rose bengal	27	42
10	Thioxanthen-9-one	0	94
11	Safranine O	0	98

Reaction run on 0.1 mmol scale in dry and degassed DMSO. [a] Determined by ^{19}F NMR using 2-fluoro-4-nitrotoluene as internal standard (added after work up).

Table 9: Stoichiometry of reactants.

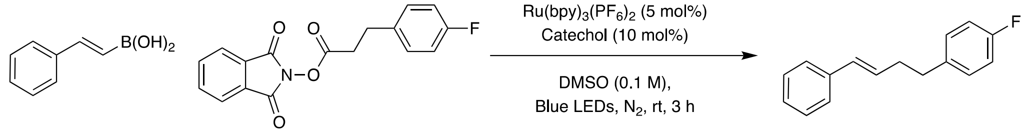

1a (XX equiv.) **2** (XX equiv.) **3**

Entry	**1a** (equiv.)	**2** (equiv.)	**3** (%)[a]	**2** (%)[a]
1	1	1	49	0
2	2	1	69	0
3	3	1	67	0
4	4	1	70	0
5	5	1	71	0
6	1	2	62	36
7	1	3	58	48

Reaction run on 0.1 mmol scale in dry and degassed DMSO. [a] Determined by ^{1}H NMR using 2-fluoro-4-nitrotoluene as internal standard (added after work up).

Table 10: Additive screening.

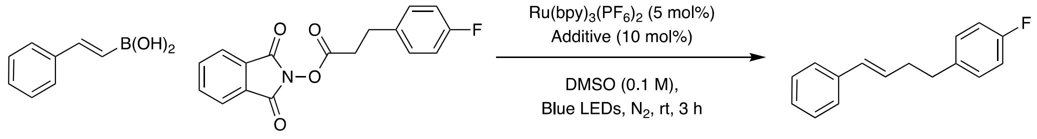

1a (2 equiv.) **2** (1 equiv.) **3**

Entry	Additive	**3** (%)[a]	**2** (%)[a]
1	Catechol	70	0
2	4-Methylcatechol	55	4

3	*tert*-Butylcatechol	68	0
4	4-Nitrocatechol	6	77
5	Ethyl 3,4-dihydrobenzylate	3	87
6	3-Methylcatechol	59	2
7	3-Methoxycatechol	52	0
8	Naphtalene-2,3-diol	5	77
9	1,3-Dihydroxybenzene	12	78
10	1,4-Dihydroxybenzene	21	51
11	Phenol	14	72
12	*p*-Benzoquinone	0	90
13	2-Methoxyphenol	21	65
14	1,2-Dimethoxybenzene	12	81
15	1,3-Dimethoxybenzene	15	75
16	1,3,5-Trimethoxybenzene	16	76
17	Anisole	7	56
18	2-Hydroxybenzylalcohol	15	76
19	Salicylic acid	2	90
20	2-Aminophenol	59	0
21	1,2-Phenyldiamine	62	0
22	Naphtalene-2,3-diamine	23	44
23	1,2-Benzenedithiol	14	65
24	2-Aminothiophenol	46	12
25	Aniline	62	12
26	*N*-Methylaniline	72	0
27	*N*,*N*-Dimethylaniline	75	0
28	4-Nitroaniline	13	74
29	4-Methoxyaniline	70	0

30	Pinacol	10	79
31	Urea	10	72
32	Thiourea	16	72
33	Ethylene glycol	9	78
34	Ethylenediamine	20	20
35	Diphenylamine	64	0
36	Triphenylamine	15	79
37	Pyridine	21	72
38	DMAP	17	74
39	(L)-Tartaric acid	4	91
40	Boric acid	8	93

Reaction run on 0.1 mmol scale in dry and degassed DMSO. [a] Determined by ^{19}F NMR using 2-fluoro-4-nitrotoluene as internal standard (added after work up).

2.3 Reaction optimization with **4**

Table 11: Catalyst loading and additives screen.

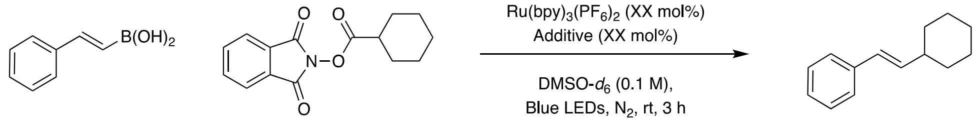

Entry	Photocatalyst loading	Additive (mol%)	**5** (%)[a]	**4** (%)[a]
1	5 mol%	Catechol (10)	82	0
2	5 mol%	*N,N*-Dimethylaniline (10)	92	0
3	2.5 mol%	*N,N*-Dimethylaniline (10)	97	0
4	1 mol%	None	16	89
5	1 mol%	Catechol (10)	77	0
6	1 mol%	*N,N*-Dimethylaniline (10)	96 (72)[b]	0
7	1 mol%	*N,N*-Dimethylaniline (5)	85	10
8	0	*N,N*-Dimethylaniline (10)	0	100

Reaction run on 0.2 mmol scale in dry and degassed DMSO-d_6. [a] Determined by ^1H NMR using 1,3,5-trimethoxybenzene as internal standard (added to the reaction mixture). [b] Isolated yield.

Table 12: Time study.

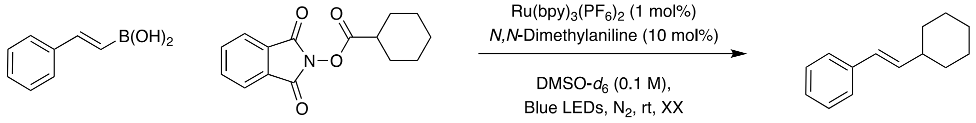

1a (2 equiv.) **4** (1 equiv.) **5**

Entry	Time	**5** (%)[a]	**4** (%)[a]
1	30 min	60	35
2	1 h	81	5
3	2 h	95	0
4	3 h	96	0

Reaction run on 0.2 mmol scale in dry and degassed DMSO-d_6. [a] Determined by ^{1}H NMR using 1,3,5-trimethoxybenzene as internal standard (added to the reaction mixture).

Table 13: Organoboron screening.

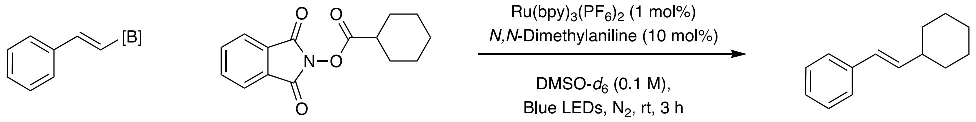

1 (2 equiv.) **4** (1 equiv.) **5**

Entry	[B]	**5** (%)[a]	**4** (%)[a]
1	$B(OH)_2$ (**1a**)	96	0
2	BPin (**1b**)	28	0
3	BCat (**1c**)	46	0
4	BF_3K (**1d**)	Traces	Traces
5	BMIDA (**1e**)	0	23
6	*p*-MeOPhB$(OH)_2$ instead of **1**	0	69

Reaction run on 0.2 mmol scale in dry and degassed DMSO-d_6. [a] Determined by ^{1}H NMR using 1,3,5-trimethoxybenzene as internal standard (added to the reaction mixture).

Table 14: Concentration screening.

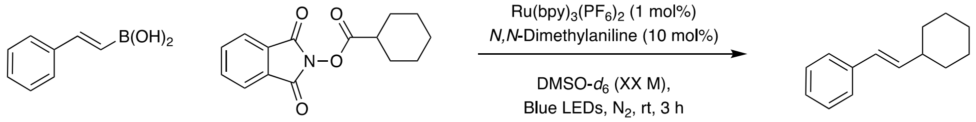

1a (2 equiv.) **4** (1 equiv.) **5**

Entry	Scale and Concentration	**5** (%)[a]	**4** (%)[a]

1	0.1 mmol in 1 mL (0.1 M)	94	0
2	0.2 mmol in 2 mL (0.1 M)	96 (72)[b]	0
3	0.2 mmol in 1 mL (0.2 M)	94 (80)[b]	0

Reaction run in dry and degassed DMSO-d_6. [a] Determined by ^{1}H NMR using 1,3,5-trimethoxybenzene as internal standard (added to the reaction mixture). [b] Isolated yield.

Table 15: Control experiments: Role of NHPI esters.

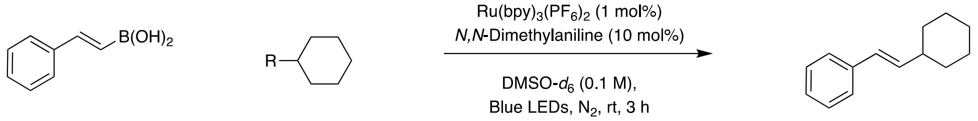

1a (2 equiv.) (1 equiv.) **5**

Entry	R	**5** (%)[a]	**SM** (%)[a]
1	COOH	0	95
2	Br	0	100

Reaction run in 0.2 mmol scale in dry and degassed DMSO-d_6. [a] Determined by ^{1}H NMR using 1,3,5-trimethoxybenzene as internal standard (added to the reaction mixture).

Graph 2:Stability of the product under photoredox conditions.

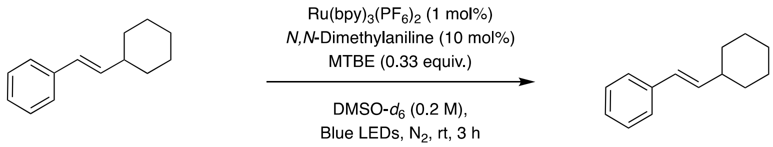

5 **5**

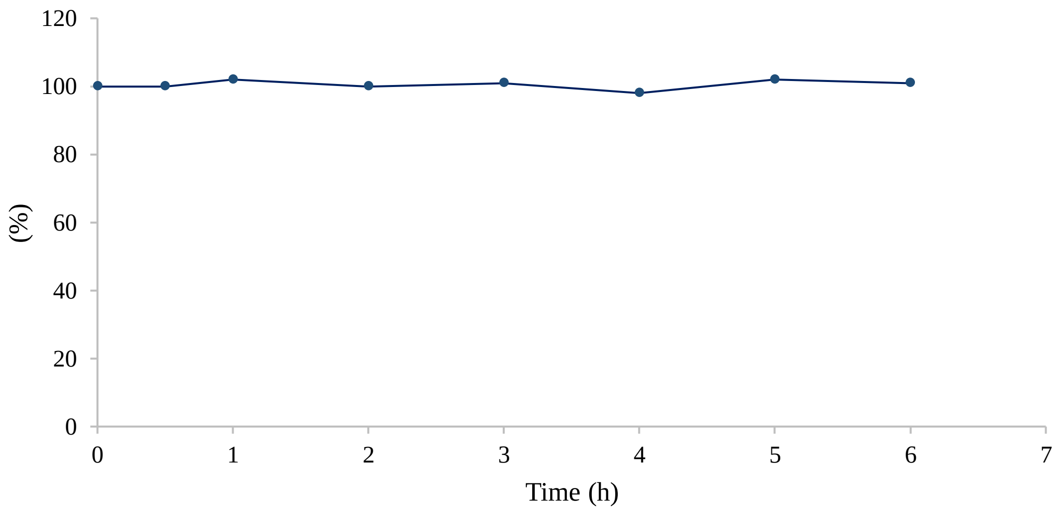

(E)-(2-Cyclohexylvinyl)benzene (37.3 mg, 200 µmol, 1.0 equiv.), tris(2,2'-bipyridine)ruthenium hexafluorophosphate (1.7 mg, 2.00 µmol, 1 mol%), and N,N-dimethylaniline (2.5 µL, 20.0 µmol, 10 mol%) in DMSO-d_6 (2 mL, 0.2. M). MTBE (8.0 µL, 66.6 µmol, 1 equiv.) was added. An aliquot was added to a J.

Youngs NMR tube and was analysed by ^{1}H NMR spectroscopy. The NMR tube was irradiated under blue LEDs for XX minutes and ^{1}H NMR was recorded. This process was repeated for 6 hours.

3. General procedures

3.1 Synthesis of NHPI Esters

NHPI esters were synthesized according to the literature using either DIC (General procedure 1A)[1] or EDCl (General procedure 1B).[2]

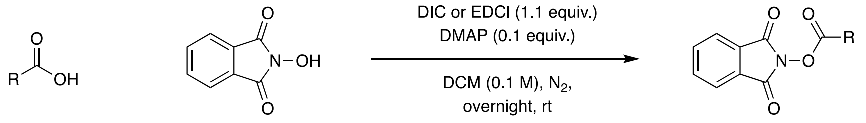

2, 4, 61, 64
N1 to N46

Scheme 1: General synthesis of NPHI Esters.

General Procedure 1A (DIC)

Prepared according to the literature.[1] A flame-dried three-necked round bottom flask equipped with a Teflon-coated stir bar was charged with *N*-hydroxyphthalimide (1.0 equiv.), 4-dimethylaminopyridine (10 mol%), and the desired carboxylic acid (1.0 equiv., if solid). The flask was purged using an N_2-vacuum cycle (3 times) and backfilled with N_2. DCM (0.1 M, total volume) was added, followed by the desired carboxylic acid (1.0 equiv., if liquid). *N,N'*-Diisopropyl-carbodiimide (1.1 equiv.) was dissolved in DCM and added dropwise to the reaction mixture. The reaction mixture was left to stir overnight at room temperature. Once complete, the reaction mixture was concentrated *in vacuo*. The crude residue was purified by flash chromatography (silica gel) using hexane/ethyl acetate or hexane/diethyl ether.

General Procedure 1B (EDCl)

Prepared according to the literature.[2] A flame-dried three-necked round bottom flask equipped with a Teflon-coated stir bar was charged with the desired carboxylic acid (1.0 equiv., if solid), EDC hydrochloride (1.2 equiv.), and 4-dimethylaminopyridine (10 mol%). The flask was purged using an N_2-vacuum cycle (3 times) and backfilled with N_2. DCM (0.1 M, total volume) was added followed by the desired carboxylic acid (1.0 equiv., if liquid). *N*-Hydroxyphthalimide (1.0 equiv.) was added portionwise to the reaction mixture. The reaction mixture was left to stir overnight at room temperature. Once complete, the reaction mixture was washed with 1 M HCl. The organic layer was washed with brine, dried over sodium sulfate, filtered, and concentrated *in vacuo*. The crude residue was purified by flash chromatography (silica gel) using hexane/ethyl acetate or hexane/diethyl ether.

The following NHPI esters were synthesized:

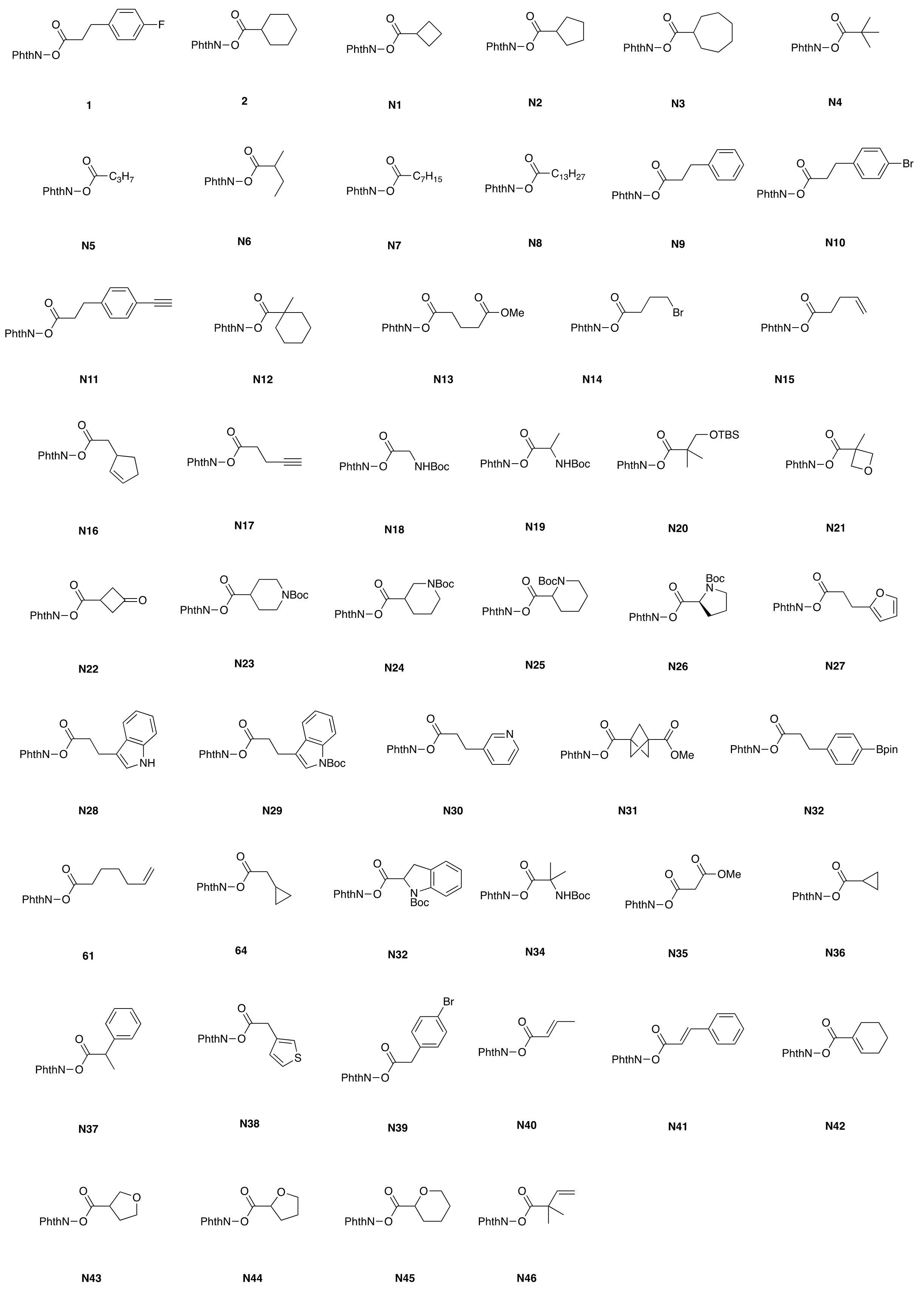

Figure 1: NHPI esters synthesized.

3.2 Synthesis of styrenyl boronic acids

The styrenyl boronic acids were synthesized according to Scheme 2. In the case of a commercially available alkynes, the synthesis started with a Cu-mediated hydroboration.[3–6] Otherwise, the synthesis started with a Sonogashira cross-coupling reaction with the (hetero)aryl iodide (or bromide).

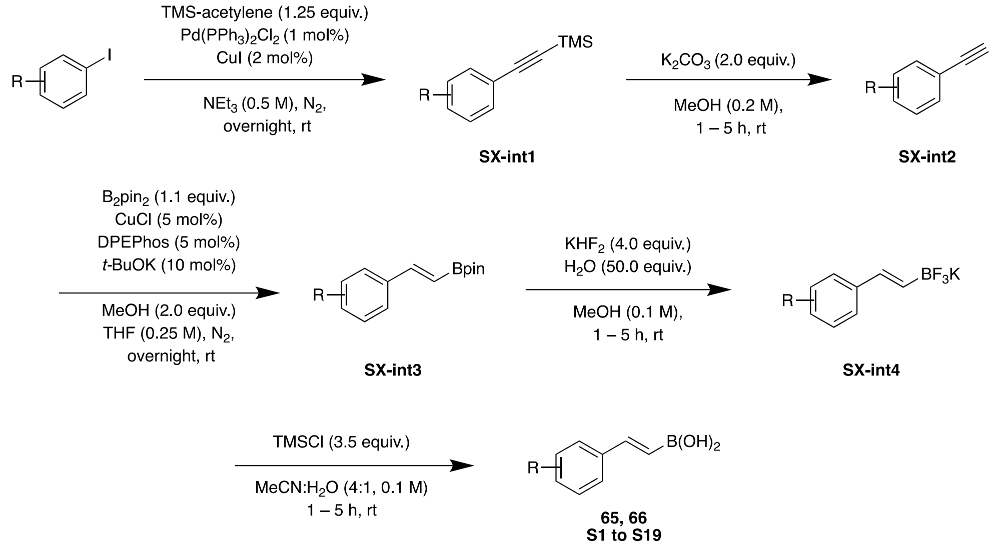

Scheme 2: General synthesis of styrenyl boronic acids.

General Procedure 2: Sonogashira cross-coupling reaction

An oven-dried microwave vial equipped with a Teflon-coated stir bar was charged with dichlorobis(triphenylphosphine)palladium (1 mol%), copper iodide (2 mol%), and the desired (hetero)aryl iodide (or bromide) (1.0 equiv., if solid). The vial was then sealed, purged using an N_2-vacuum cycle (3 times), and backfilled with N_2. Triethylamine (0.5 M) was added followed by the desired (hetero)aryl iodide (or bromide) (1.0 equiv., if liquid), and trimethylsilylacetylene (1.25 equiv.). The reaction mixture was left to stir overnight at room temperature. Once complete, the crude mixture was filtered through celite, rinsed with DCM, and the combined organics were concentrated *in vacuo*. The crude residue was purified by flash chromatography (silica gel) using hexane/ethyl acetate or hexane/diethyl ether affording the desired product **SX-int1**.

General Procedure 3: TMS deprotection

SX-int1 (1.0 equiv.) was dissolved in MeOH (0.2 M) and potassium carbonate (2.0 equiv.) was added. The reaction mixture was stirred one to five hours at room temperature. Once complete, the volatiles were removed under vacuum. The crude mixture was partitioned between water and DCM and the organics were extracted with DCM. The combined organics were washed with water, dried over sodium sulfate, filtered, and concentrated *in vacuo*. The crude residue was purified by flash chromatography (silica gel) using hexane/ethyl acetate or hexane/diethyl ether affording the desired product **SX-int2**.

General Procedure 4: Cu-mediated hydroboration

Prepared according to the literature.[3–6] An oven-dried microwave vial equipped with a Teflon-coated stir bar was charged with copper(I) chloride (5 mol%), potassium *tert*-butoxide (10 mol%), and bis(2-diphenylphosphinophenyl) ether (DPEPhos) (5 mol%). The vial was sealed and purged using an N_2-Vacuum cycles (3 times) and backfilled with N_2. THF (0.25 M, total volume) was added, and the mixture

was stirred for 45 minutes at room temperature. Bis(pinacolato)diboron (1.1 equiv.) was dissolved in THF and added yia syringe. The reaction mixture was then left to stir at room temperature for 30 minutes. The alkyne **SX-int2** (1.0 equiv.) was dissolved in THF if solid or added directly to the reaction mixture if liquid, followed by MeOH (2.0 equiv.). The reaction mixture was stirred overnight at room temperature. Once complete, the reaction mixture was filtered through a pad of celite, rinsing with DCM, and the combined organics were concentrated *in vacuo*. The crude residue was purified by flash chromatography (silica gel) using hexane/ethyl acetate or hexane/diethyl ether affording the desired product **SX-int3**.

General Procedure 5: BF_3K Synthesis

SX-int3 (1.0 equiv.) was dissolved in MeOH (0.1 M) and potassium hydrogen fluoride (4.0 equiv.) was added. Water (50.0 equiv.) was added dropwise, and the reaction was left to stir for one to five hours at room temperature. The reaction was monitored by ^{11}B NMR and once complete, the mixture was concentrated *in vacuo* and the crude residue was dissolved in hot acetone, filtered, and concentrated *in vacuo.* The white precipitate was then dissolved in acetone and diethyl ether was added to precipitate the salt. The desired product, **SX-int4** was obtained after filtration.

General Procedure 6: Hydrolysis of BF_3K substrate to styrenyl boronic acid

SX-int4 (1.0 equiv.) was dissolved in a mixture of MeCN and H_2O (4:1, 0.1 M). Freshly distilled chlorotrimethylsilane (3.5 equiv.) was added and the reaction mixture and was left to stir for one to five hours at room temperature, monitoring by ^{11}B NMR spectroscopy. Once complete, the reaction mixture was partitioned between water and ethyl acetate and the organics were extracted with ethyl acetate (3 times). The organic layers were combined, dried over sodium sulfate, filtered, and concentrated *in vacuo* affording the desired product **SX**. **SX** was usually obtained as a mixture of the desired product and boroxine, a drop of water was added in order to hydrolyse the boroxine into the boronic acid.

The following styrenyl boronic acids were synthesized:

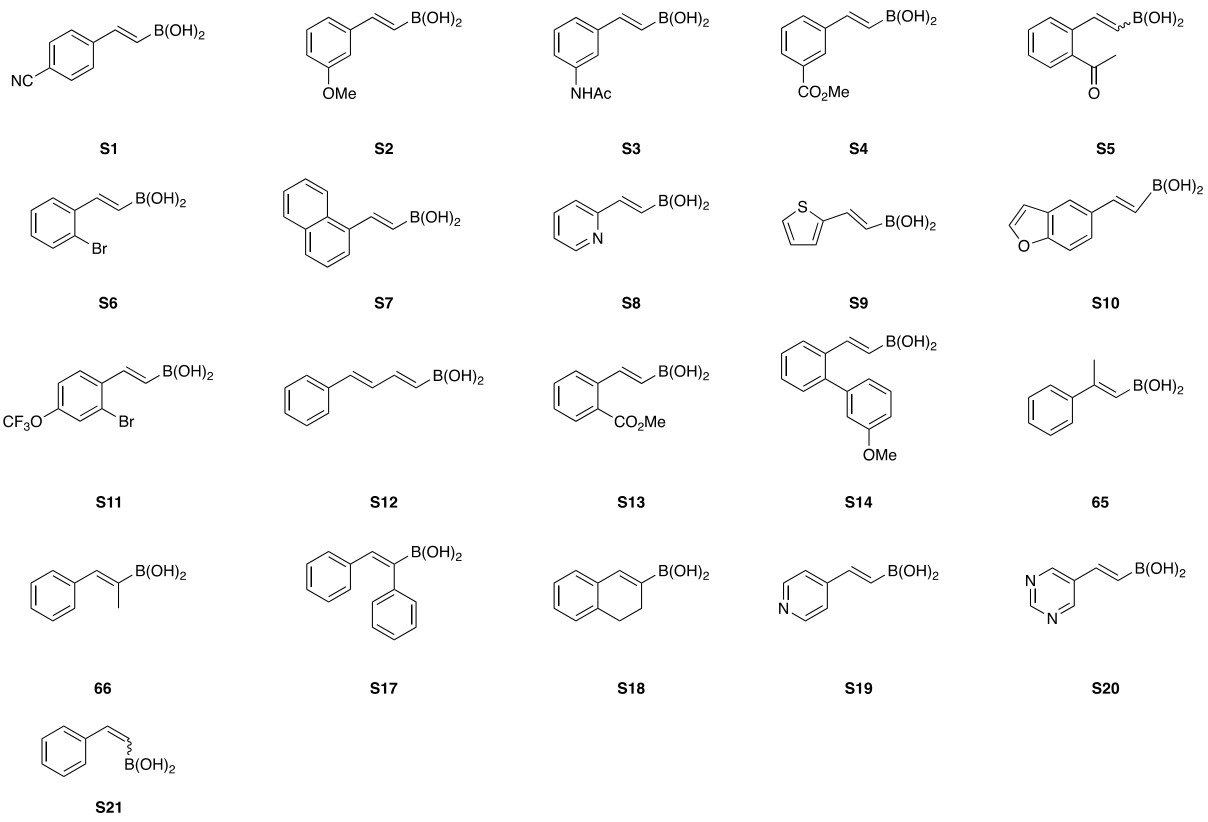

Figure 2: Styrenyl boronic acids synthesized.

3.3 Procedure for the developed reaction

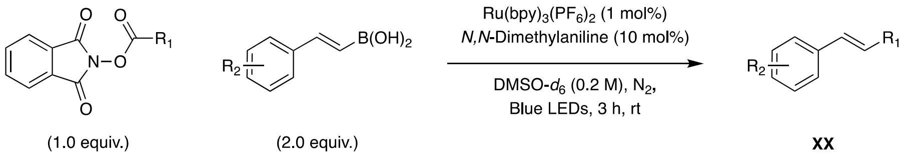

Scheme 3: Developed reaction between NHPI esters and styrenyl boronic acids.

General Procedure 7

An oven-dried photoreactor vial, equipped with a Teflon-coated stir bar, was charged with the NHPI ester **NX** (200 μmol, 1.0 equiv.), the styrenyl boronic acid **SX** (400 μmol, 2.0 equiv.), and tris(2,2'-bipyridine)ruthenium hexafluorophosphate (1.7 mg, 2.00 μmol, 1 mol%). The vial was then sealed, purged with N_2-vacuum cycles (3 times), and backfilled with N_2. Degassed dry DMSO-d_6 (1 mL, 0.2 M) was then added followed by *N,N*-dimethylaniline (2.5 μL, 20.0 μmol, 10 mol%). The cap was wrapped with parafilm and the reaction mixture was stirred under blue LEDs under N_2 for three hours. After three hours, the reaction mixture was partitioned between diethyl ether (5 mL) and brine (5 mL). Organics were extracted with diethyl ether (2 × 10 mL). The organics were combined, washed with brine (15 mL), dried over sodium sulfate, filtered, and concentrated *in vacuo*. The crude residue was purified by flash chromatography (silica gel) using hexane/ethyl acetate or hexane/diethyl ether affording the desired product.

Note: Product *E:Z* ratio was determined by ^{1}H NMR Unless otherwise noted, only the *E* product was observed.

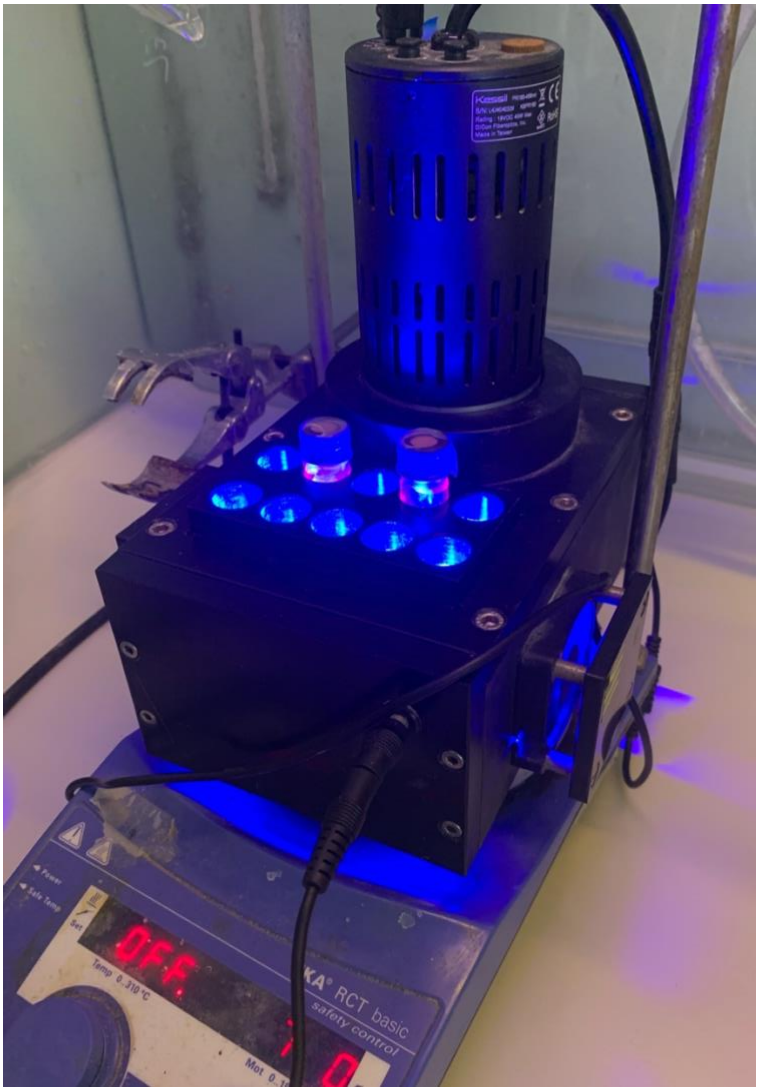

Picture 2: Photoreactor and set up.

4. **Control reactions and mechanistic investigations**

4.1 General comments

The following reactions were run in DMSO-d_6 using MTBE (methyl *tert*-butyl ether) as an internal standard (added after the reaction) for ^{1}H NMR analysis. In some cases, accurate integration of the crude reaction could not be performed. Thus, the reaction mixture was worked-up: the mixture was partitioned between diethyl ether (5 mL) and brine (5 mL). Organics were extracted with diethyl ether (2 × 10 mL). Organics were combined, washed with brine (15 mL), dried over sodium sulfate, filtered, and concentrated *in vacuo* affording the crude mixture. The crude mixture was dissolved in $CDCl_3$, the internal standard was added prior to ^{1}H NMR analysis.

4.2 Reaction with (*Z*)-styrenyl boronic acid

Table 16: Reactions with (*Z*)-styrenyl organoborons.

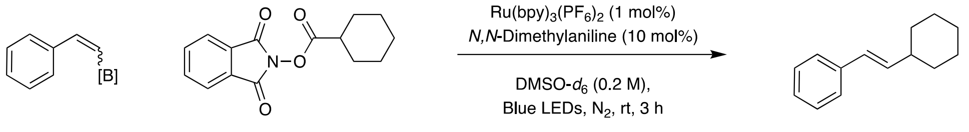

Entry	[B]	Ratio *Z*:*E*	**5** (%)[a]	**4** (%)[a]
1	Bpin, **S19-int3cis**	1:0.35	13	40
2	$B(OH)_2$, **S19**	1:1.1	34	64
3[b]	$B(OH)_2$, **S19**	1:1.1	33	49

Reaction run on 0.2 mmol scale in dry and degassed DMSO-d_6. [a] Determined by ^{1}H NMR using MTBE as internal standard (added to the reaction mixture after reaction). [b] Using 1.0 equiv. of **S19**.

4.3 Reaction with *α*- and *β*-substituted styrene boronic acids.

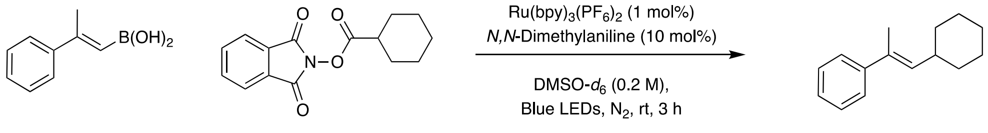

Scheme 4: Standard reaction with an α-substituted styrene boronic acid, **65**.

Prepared according to General Procedure 7 using 1,3-dioxoisoindolin-2-yl cyclohexanecarboxylate, **4** (27.3 mg, 100 µmol, 1.0 equiv.), (*E*)-(2-phenylprop-1-en-1-yl)boronic acid, **65** (32.4 mg, 200 µmol, 2.0 equiv.), tris(2,2'-bipyridine)ruthenium hexafluorophosphate (0.9 mg, 1.00 µmol, 1 mol%), and *N*,*N*-dimethylaniline (1.3 µL, 10.0 µmol, 10 mol%) in DMSO-d_6 (1 mL, 0.1 M). The crude residue was purified by flash chromatography (silica gel) using hexane affording 15.8 mg of **P1** as a colourless oil (67%, *E*:*Z* > 20:1, purity 85%).

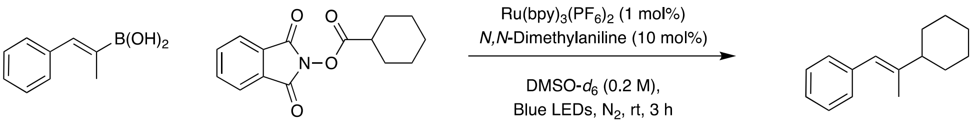

65 (2.0 equiv.) **4** (1.0 equiv.) **P2**, 0%

Scheme 5: Standard reaction with an β-substituted styrene boronic acid, **65**.

Prepared according to General Procedure 7 using 1,3-dioxoisoindolin-2-yl cyclohexanecarboxylate, **4** (54.7 mg, 200 µmol, 1.0 equiv.), (*Z*)-(1-phenylprop-1-en-2-yl)boronic acid, **65** (64.8 mg, 400 µmol, 2.0 equiv.), tris(2,2'-bipyridine)ruthenium hexafluorophosphate (1.7 mg, 2.00 µmol, 1 mol%), and *N*,*N*-dimethylaniline (2.5 µL, 20.0 µmol, 10 mol%) in DMSO-d_6 (1 mL, 0.2 M). No traces of product (**P2**) were observed.

4.4 Competition reactions

Table 17: Competition reactions with *p*-fluorostyrene.

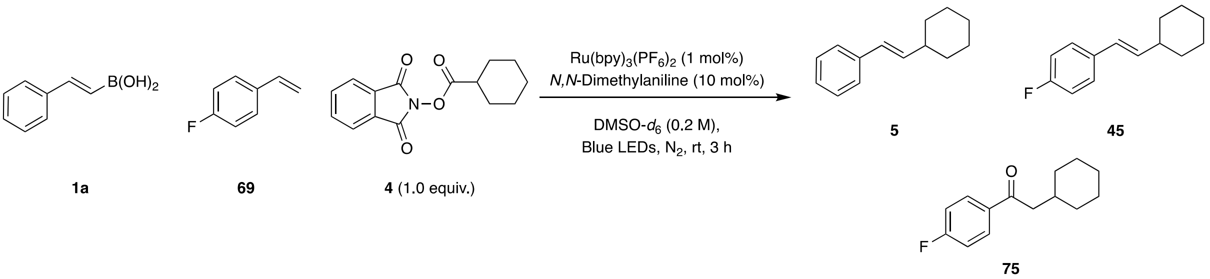

Entry	**1a** (equiv.)	**69** (equiv.)	**5** (%)[a]	**75** (%)[a]	**4** (%)[b]	**69** (%)[a]
1	1	1	35[b]	19	31	50
2	0	2	-	Traces	70	70
3[c]	1	1	42[b]	23	0	38
4[c]	0	2	-	5	0	80
5[d]	1	1	15[b]	10	60	40
6[d]	0	2	-	6	78	100
7[e]	0	2	-	17	55	71
8[f]	0	2	-	20	45	70

Reaction run on 0.2 mmol scale in dry and degassed DMSO-d_6. [a] Determined by ^{1}H NMR using MTBE as internal standard (added after reaction). [b] Determined by ^{1}H NMR using MTBE as internal standard (added after work up). [c] Using 50 mol% of *N*,*N*-dimethylaniline. [d] Using 20 mol% of Ru(bpy)$_3$(PF$_6$)$_2$. [e] 1.0 equiv. of PhB(OH)$_2$ added. [f] 1.0 equiv. of B(OH)$_3$ added.

Note that traces (< 3%) of (*E*)-1-(2-cyclohexylvinyl)-4-fluorobenzene, **45** were observed, as well as minor by-products derived from styrene.

Table 18: Competition reactions with *p*-fluorostyrene with an unsuccessful styrenyl boronic acid, **66**.

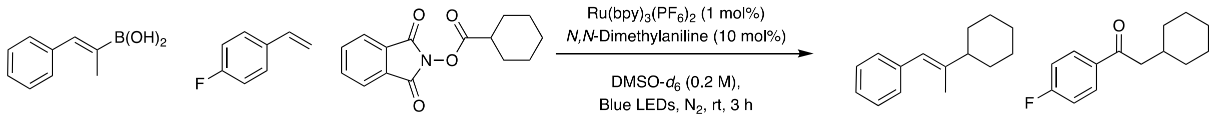

66 **69** **4** (1.0 equiv.) **P2** **75**

Entry	**66** (equiv.)	**69** (equiv.)	**P2** (%)[a]	**75** (%)[a]	**4** (%)[a]	**69** (%)[a]
1	1	1	0	23[b]	32 (34)[b]	40

Reaction run on 0.2 mmol scale in dry and degassed DMSO-d_6. [a] Determined by ^{1}H NMR using MTBE as internal standard (added after reaction). [b] Determined by ^{1}H NMR using MTBE as internal standard (added after work up).

Table 19: Competition reactions with **A1**.

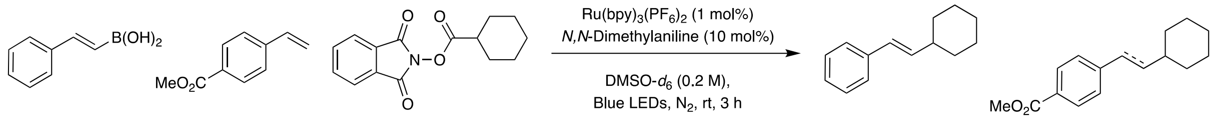

1a **A1** **4** (1.0 equiv.) **5** **P6a/b**

Entry	**1a** (equiv.)	**A1** (equiv.)	**5** (%)[a]	**P6a/b** (%)[a]	**4** (%)[a]
1	1	1	0	0/0	94
2	0	2	-	0/0	70
3[b]	0	2	-	0/0	73

Reaction run on 0.2 mmol scale in dry and degassed DMSO-d_6. [a] Determined by ^{1}H NMR using MTBE as internal standard (added after reaction). [b] 1.0 equiv. of $B(OH)_3$ added.

Table 20: Competition reactions with **A2**.

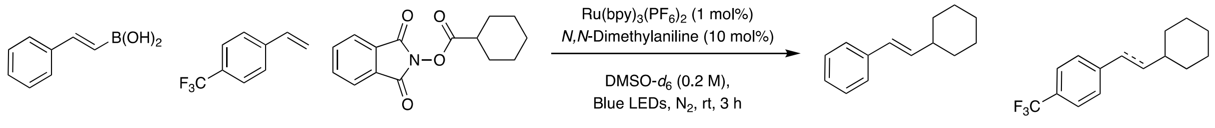

1a **A2** **4** (1.0 equiv.) **5** **P7a/b**

Entry	**1a** (equiv.)	**A1** (equiv.)	**5** (%)[a]	**P6a/b** (%)[a]	**4** (%)[a]
1	1	1	5	0/0	56
2	0	2	-	0/0	57
3[b]	0	2	-	0/0	58

Reaction run on 0.2 mmol scale in dry and degassed DMSO-d_6. [a] Determined by ^{1}H NMR using MTBE as internal standard (added after reaction). [b] 1.0 equiv. of $B(OH)_3$ added.

Table 21: Competition reactions with ethyl acrylate, **70**.

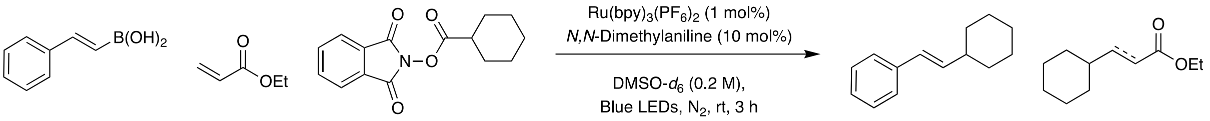

1a **70** **4** (1.0 equiv.) **5** **73a/b**

Entry	**1a** (equiv.)	**70** (equiv.)	**5** (%)[a]	**73a/b** (%)[a]	**4** (%)[a]	**70** (%)[a]
1	1	1	8	0/0	0	0
2	0	2	-	0/0	51	0
3[b]	0	2	-	0/0	70	0
4[c]	0	2	-	0/0	75	0

Reaction run on 0.2 mmol scale in dry and degassed DMSO-d_6. [a] Determined by ^{1}H NMR using MTBE as internal standard (added after reaction). [b] 1.0 equiv. of $PhB(OH)_2$ added. [c] 1.0 equiv. of $B(OH)_3$ added.

Table 22: Competition reactions with ethyl cinnamate, **67**.

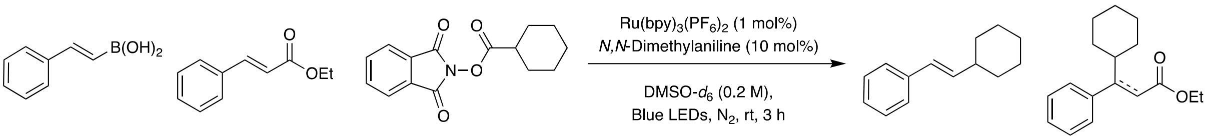

1a **71** **4** (1.0 equiv.) **5** **74a/b**

Entry	**1a** (equiv.)	**71** (equiv.)	**5** (%)[a]	**74a/b** (%)[a]	**4** (%)[a]	**71** (%)[a]
1	1	1	19	0/0	52	70
2	0	2	-	0/0	72	100
3[b]	1	1	14	0/0	53	62
4[b,c]	1	0	-	0/0	60	60

Reaction run on 0.2 mmol scale in dry and degassed DMSO-d_6. [a] Determined by ^{1}H NMR using MTBE as internal standard (added after reaction). [b] Reaction time = 6 hours. [c] 1.0 equiv. of $B(OH)_3$ added.

Note that in the case of ethyl acrylate, **70** and ethyl cinnamate, **71** no product from the Kornblum-type oxidation was observed.

Table 23: Competition reactions with alkenyl boronic acid, **67**.

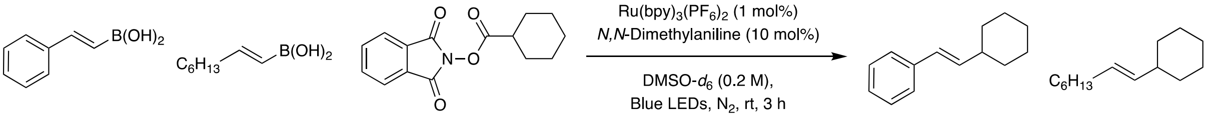

1a **67** **4** (1.0 equiv.) **5** **P5**

Entry	**1a** (equiv.)	**67** (equiv.)	**5** (%)[a]	**P5** (%)[a]	**4** (%)[a]	**67** (%)[a]

1	1	1	58[b]	0	19[b]	Not soluble[b]
2	0	2	-	0	74 (71)[b]	100

Reaction run on 0.2 mmol scale in dry and degassed DMSO-d_6. [a] Determined by ^{1}H NMR using MTBE as internal standard (added after reaction). [b] Determined by ^{1}H NMR using MTBE as internal standard (added after work up).

A photocatalyst screening was carried out with boronic acid **67**.

Table 24 : Photocatalyst screening.

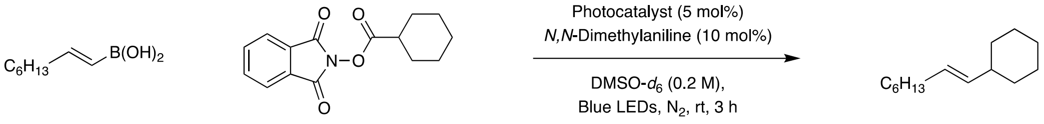

67 (2.0 equiv.) **4** (1.0 equiv.) **P5**

Entry	Photocatalyst	**P5** (%)[a]	**4** (%)[a]	**67** (%)[a]
1	$Ru(bpy)_3(PF_6)_2$	0	71	100
2	$Ir(ppy)_3$	0	38	99
3	Eosin Y	0	85	104
4	Methylene blue	0	100	104
5	10-Phenylphenothiazine	0	104	101
6	4-CzIPN	0	0	100
7	Xanthone	0	103	98
8	Rhodamine 6G	0	100	99

Reaction run on 0.1 mmol scale in dry and degassed DMSO-d_6. [a] Determined by ^{1}H NMR using MTBE as internal standard (added after reaction).

Table 25: Competition reactions with alkyl boronic acid, **68**.

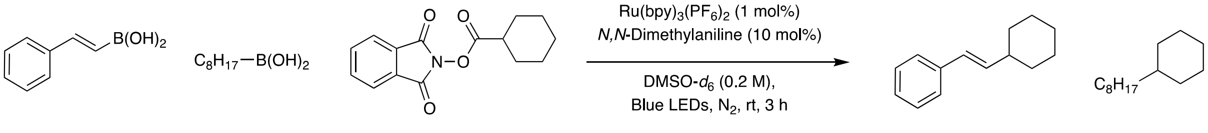

1a **68** **4** (1.0 equiv.) **5** **P6**

Entry	**1a** (equiv.)	**68** (equiv.)	**5** (%)[a]	**P6** (%)[a]	**4** (%)[a]	**68** (%)[a]
1	1	1	60 (62)[b]	0	7 (10)[b]	100
2	0	2	-	0	67 (66)[b]	100

Reaction run on 0.2 mmol scale in dry and degassed DMSO-d_6. [a] Determined by ^{1}H NMR using MTBE as internal standard (added after reaction). [b] Determined by ^{1}H NMR using MTBE as internal standard (added after work up).

4.5 Radical clock experiments

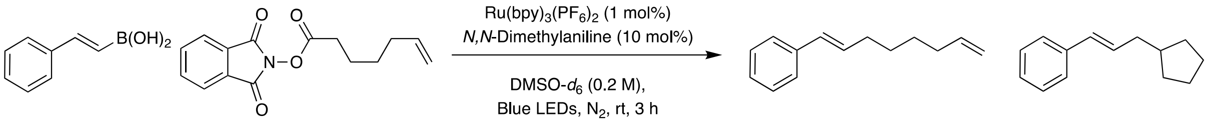

1a (2.0 equiv.) **61** (1.0 equiv.) **62**, 19% **63**, 56%

Scheme 6: Radical clock experiments: ring closure.

Prepared according to General Procedure 7 using 1,3-dioxoisoindolin-2-yl hept-6-enoate, **61** (54.7 mg, 200 µmol, 1.0 equiv.), *(E)*-2-phenylvinylboronic acid, **1a** (59.2 mg, 400 µmol, 2.0 equiv.), tris(2,2'-bipyridine)ruthenium hexafluorophosphate (1.7 mg, 2.00 µmol, 1 mol%), and *N,N*-dimethylaniline (2.5 µL, 20.0 µmol, 10 mol%) in DMSO-d_6 (1 mL, 0.2 M). The crude residue was purified by flash chromatography (silica gel) using pure hexane affording 7.2 mg of **62** as a colourless oil (19%, *E:Z* > 20:1) and 21.1 mg of **63** as a colourless oil (56%, *E:Z* > 20:1).

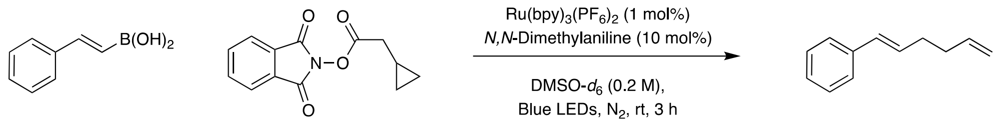

1a (2.0 equiv.) **64** (1.0 equiv.) **20**, 60%

Scheme 7: Radical clock experiments: ring opening.

Prepared according to General Procedure 7 using 1,3-dioxoisoindolin-2-yl 2-cyclopropylacetate, **64** (49.0 mg, 200 µmol, 1.0 equiv.), (*E*)-2-phenylvinylboronic acid, **1a** (59.2 mg, 400 µmol, 2.0 equiv.), tris(2,2'-bipyridine)ruthenium hexafluorophosphate (1.7 mg, 2.00 µmol, 1 mol%), and *N,N*-dimethylaniline (2.5 µL, 20.0 µmol, 10 mol%) in DMSO-d_6 (1 mL, 0.2 M). The crude residue was purified by flash chromatography (silica gel) using pure hexane affording 18.9 mg of **20** as a colourless oil (60%, *E:Z* > 20:1).

4.6 TEMPO experiment

Table 26: TEMPO experiment.

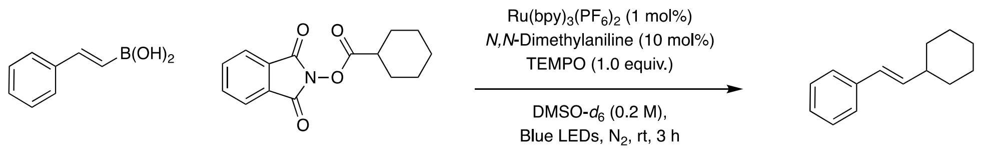

1a (2.0 equiv.) **4** (1.0 equiv.) **5**, 0%

Prepared according to General Procedure 7 using 1,3-dioxoisoindolin-2-yl cyclohexanecarboxylate, **4** (54.7 mg, 200 µmol, 1.0 equiv.), *(E)*-2-phenylvinylboronic acid, **1a** (59.2 mg, 400 µmol, 2.0 equiv.), tris(2,2'-bipyridine)ruthenium hexafluorophosphate (1.7 mg, 2.00 µmol, 1 mol%), TEMPO (31.2 mg, 200 µmol, 1.0 equiv.) and *N,N*-dimethylaniline (2.5 µL, 20.0 µmol, 10 mol%) in DMSO-d_6 (1 mL, 0.2 M). No traces of product (**5**) were observed and **4** could be fully recovered (96%).

4.7 On/off experiments

The following on/off experiment was performed in a J. Youngs NMR tube at 50 mM, using MTBE as an internal standard. Note: No mechanical stirring was used during this time study.

Scheme 8: On/Off experiment.

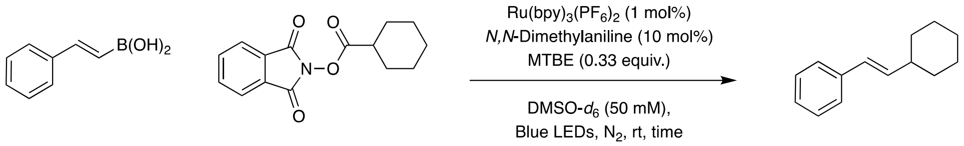

1a (2.0 equiv.) **4** (1.0 equiv.) **5**

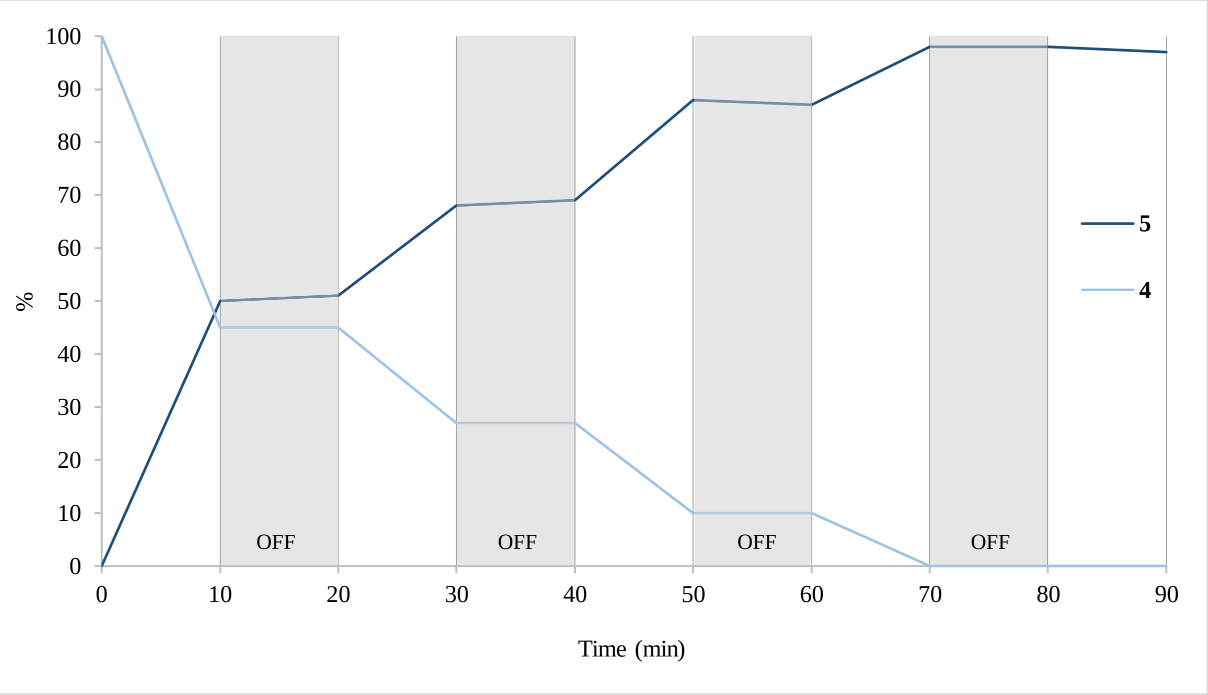

4.8 Reaction scale-up

Scheme 9: Reaction scale-up.

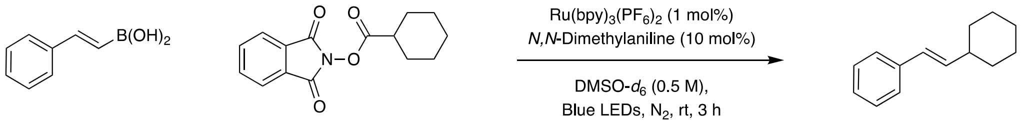

1a (2.0 equiv.) **4**, (1.0 equiv.) 8.00 mmol **5**, 85%

Prepared according to General Procedure 7 using 1,3-dioxoisoindolin-2-yl cyclohexanecarboxylate, **8** (2.19 g, 8.00 mmol, 1.0 equiv.), (*E*)-2-phenylvinylboronic acid, **1a** (2.37 g, 16.00 mmol, 2.0 equiv.), tris(2,2'-bipyridine)ruthenium hexafluorophosphate (68.8 mg, 80.00 µmol, 1 mol%), and *N*,*N*-dimethylaniline (101 µL, 800.0 µmol, 10 mol%) in DMSO-d_6 (16 mL, 0.5 M). The crude residue was purified by flash chromatography (silica gel) with pure hexane affording 1.27 g of a colourless oil as the desired product (85%, *E*:*Z* > 20:1).

4.9 Emission quenching and Stern-Volmer linearization

A stock solution of $Ru(bpy)_3(PF_6)_2$ at 10^{-5} M in DMSO was prepared and used for all the quenching studies. Quencher solutions of *N*,*N*-dimethylaniline, (*E*)-2-phenylvinylboronic acid, **1a**, and cyclohexane NHPI, **4** were prepared at 10^{-1} M in DMSO and degassed with N_2 bubbling through for 25 minutes prior their use. The cuvette sealed with a septum was purged with vacuum-N_2 cycles and backfilled with N_2. The solution of photocatalyst was added (2.5 mL) into the cuvette and was degassed with N_2 bubbling through for 25 minutes. Aliquot of quencher solutions were added (10 µL) using a micro-syringe attached through the septum (see set up from the literature).[7]

Graph 3: Emission quenching using **1a**.

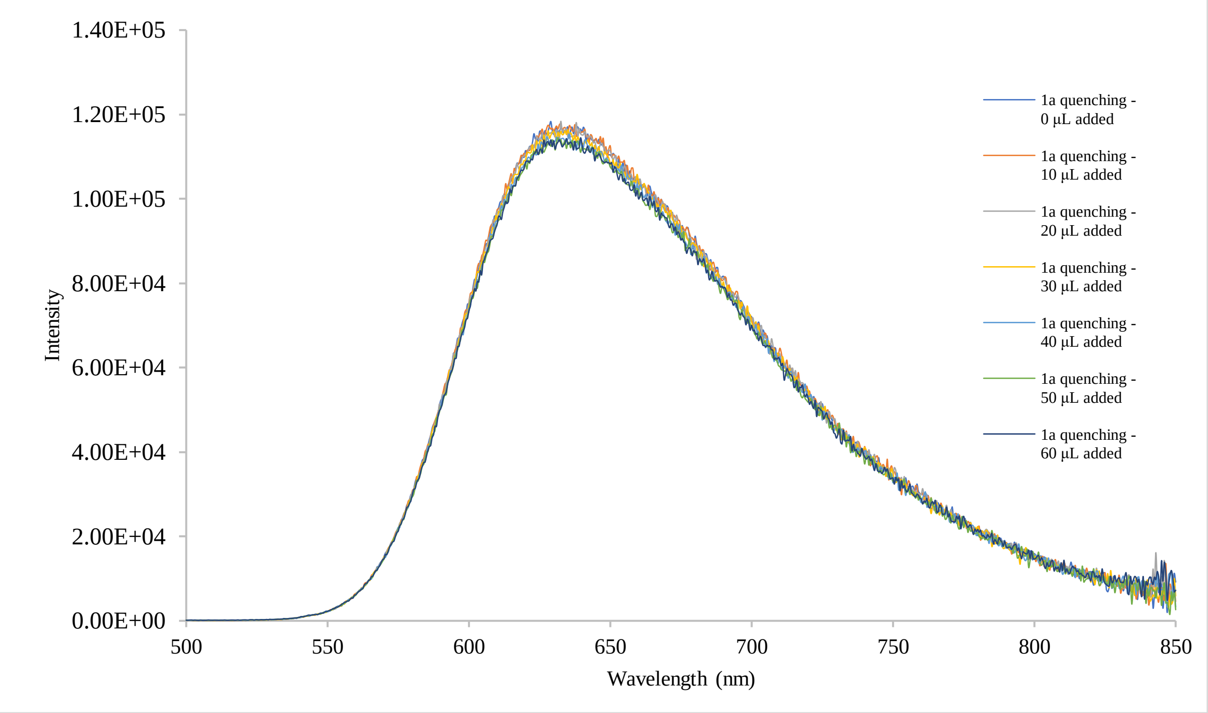

Graph 4: Emission quenching using **4**.

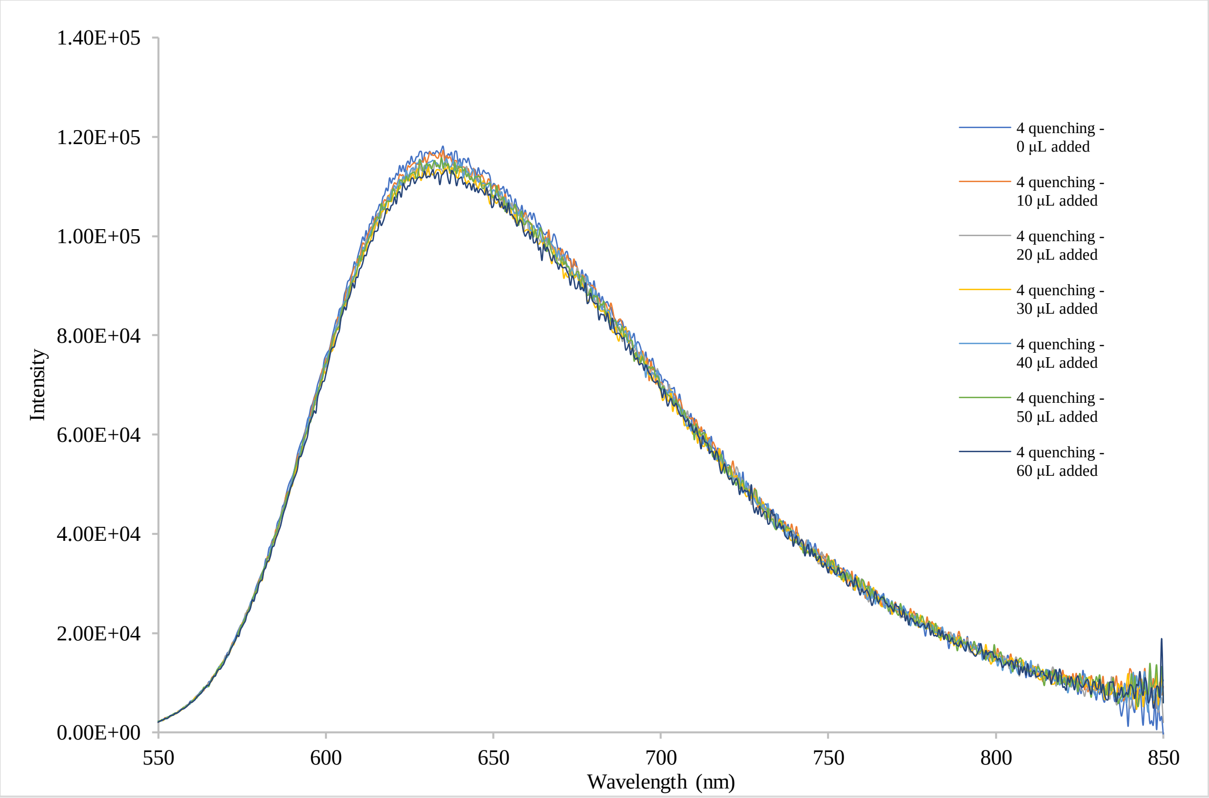

Graph 5: Emission quenching using *N*,*N*-dimethylaniline.

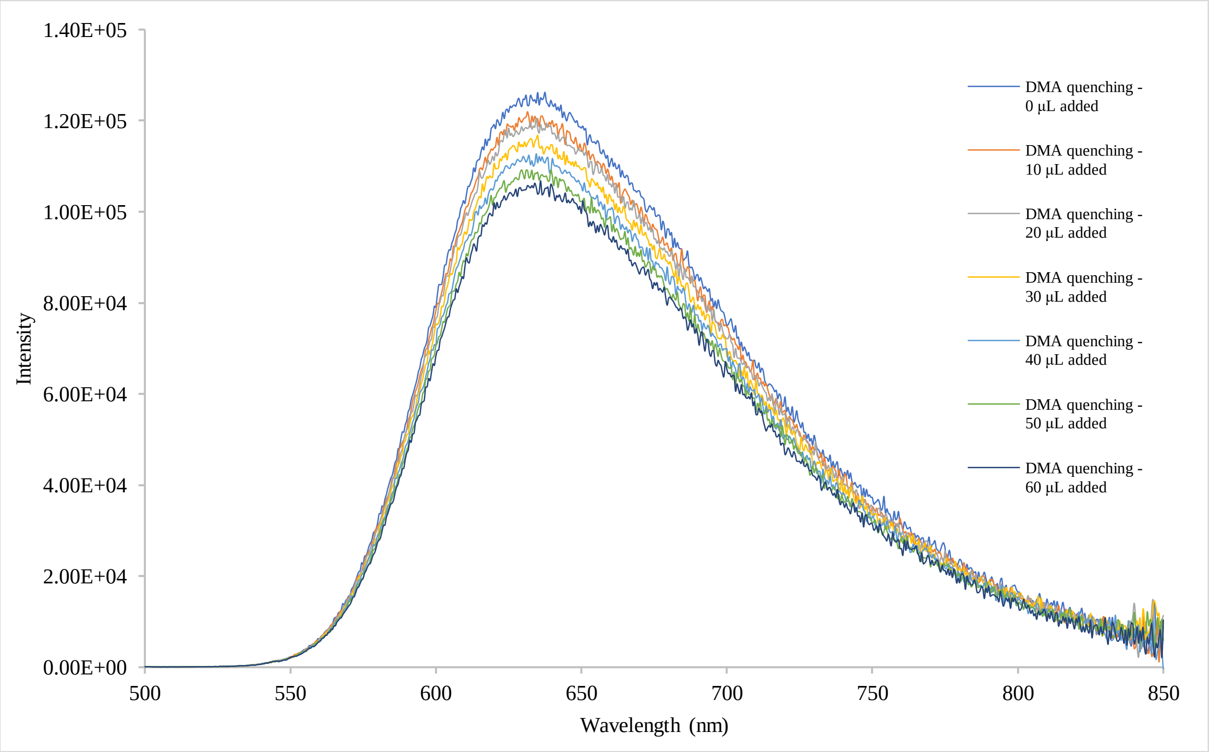

Graph 6 : Stern-Volmer linearization.

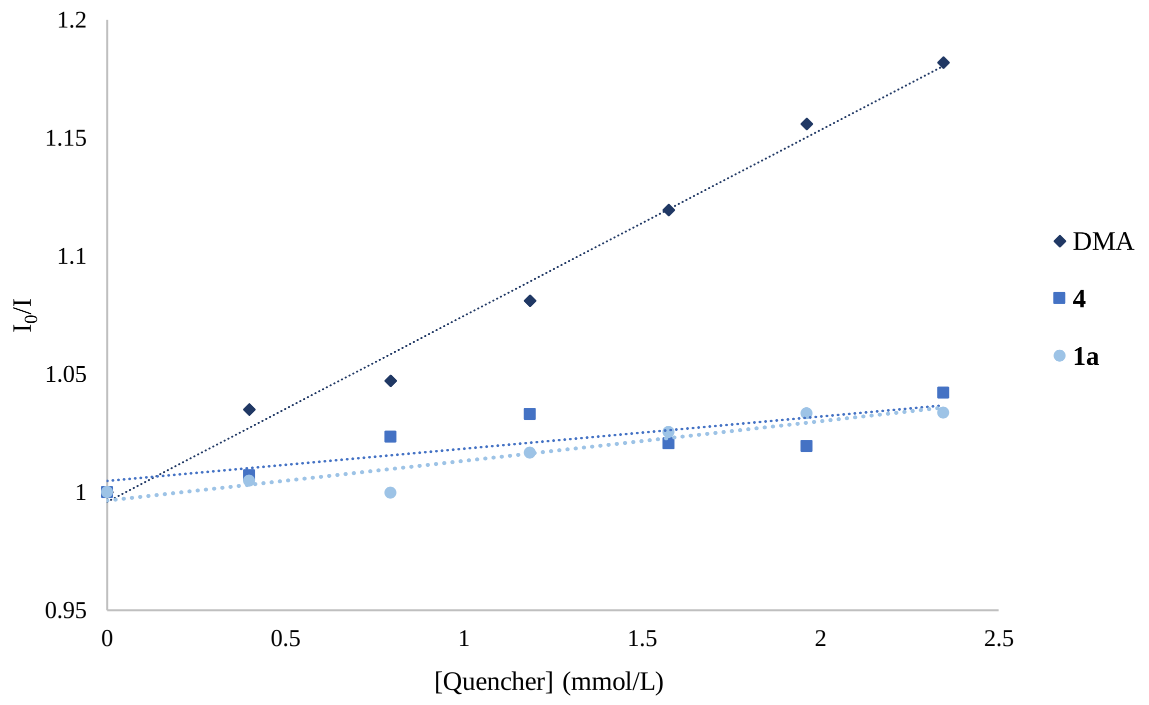

4.10 -Vis absorption

Solution of were prepared according to the following concentration: (*E*)-2-phenylvinylboronic acid (**1a**) at 1×10^{-1} M, cyclohexane NHPI (**4**) at 0.5×10^{-1} M and *N*,*N*-dimethylaniline at 0.1×10^{-1} M.

Graph 7: UV-Vis absorption spectra.

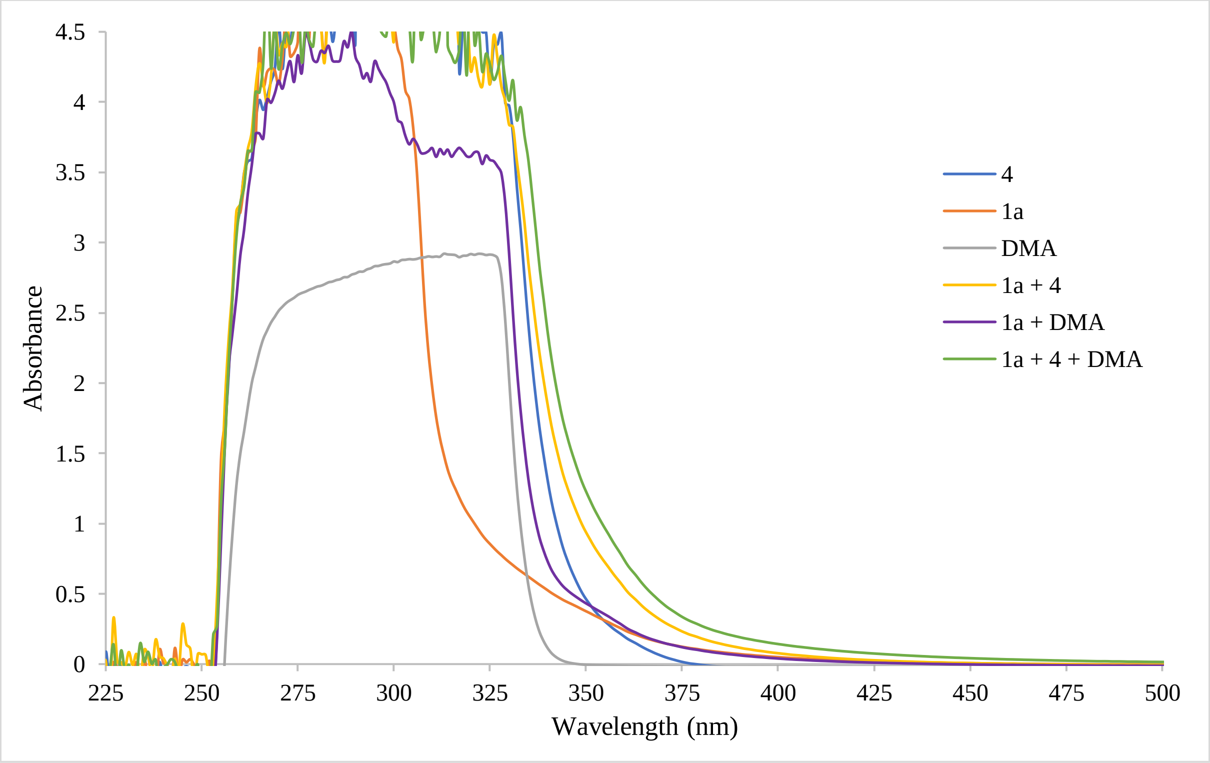

4.11 ^{1}H and ^{11}B NMR: formation of boronate *in situ*.

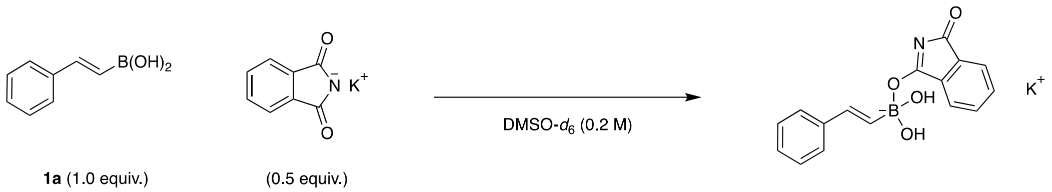

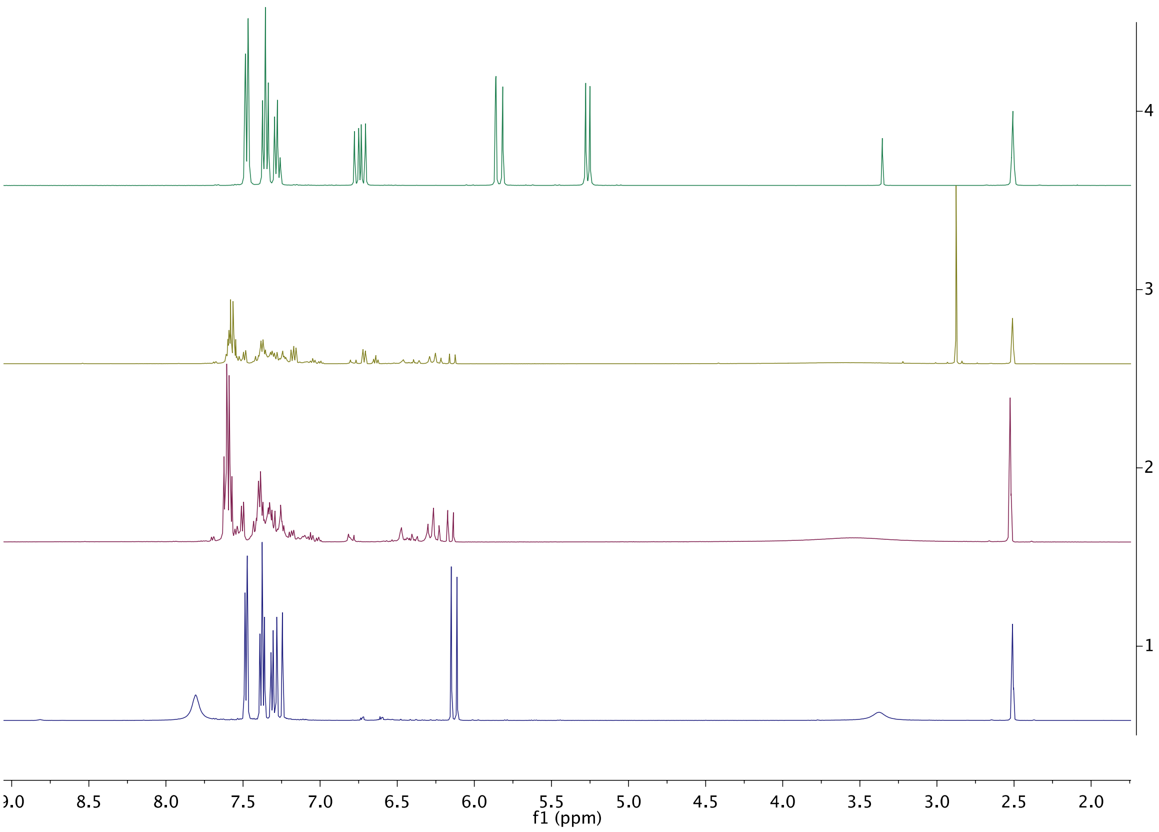

Picture 3: ^{1}H NMR spectra. In blue, **1a**; in red, **1a** + NPhthK (0.5 equiv.); in yellow, **1a** + NPhthaK (0.5 equiv.) + DMA (5 mol%); in green, styrene.

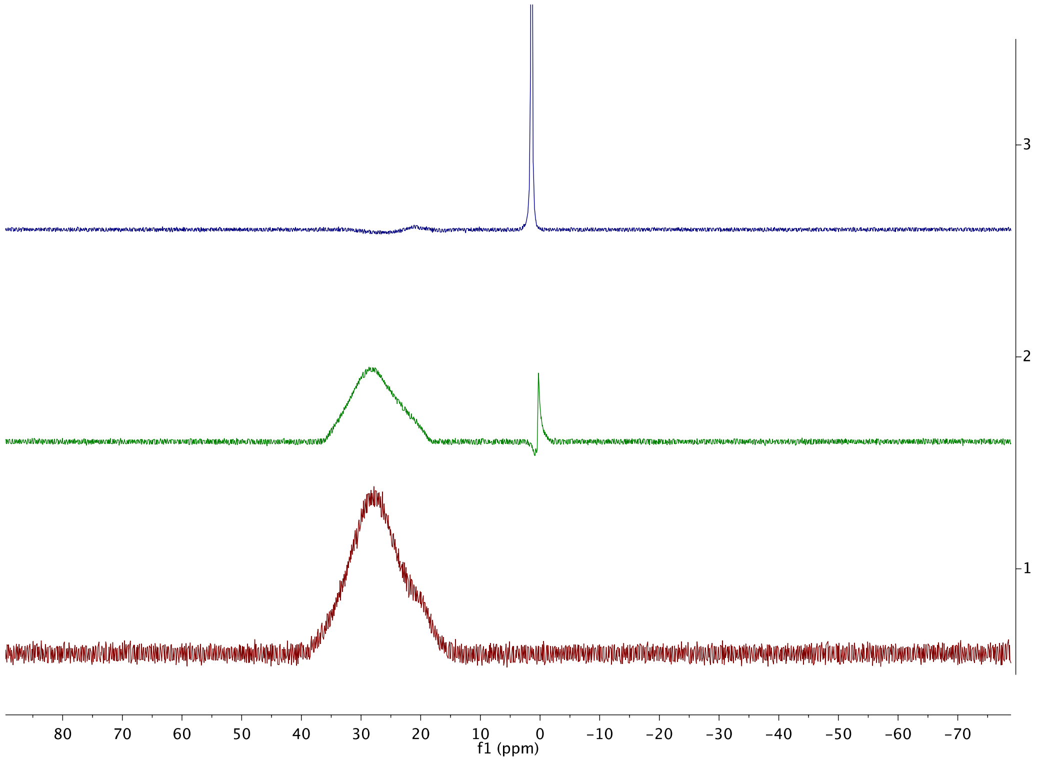

Picture 4: ^{11}B NMR spectra. In red, **1a** (27.73 ppm); in green, **1a** and NPhthK (0.5 equiv.) (27.85 and 0.24 ppm); in blue, $B(OH)_3$ + NPhthK (1 equiv.) (1.42 ppm).

5. Unsuccessful substrates

5.1 Low yielding substrate

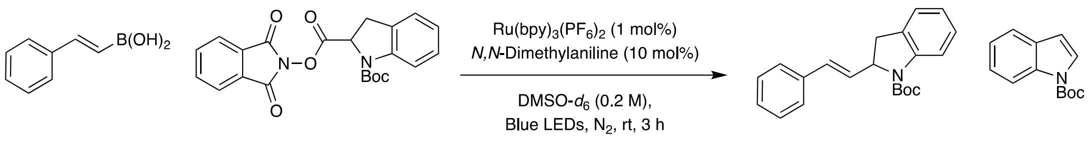

Scheme 10: Developed reaction using **N33**.

Prepared according to General Procedure 7 using 1-(*tert*-butyl) 2-(1,3-dioxoisoindolin-2-yl)-indoline-1,2-dicarboxylate, **N33** (81.7 mg, 200 µmol, 1.0 equiv.), (*E*)-2-phenylvinylboronic acid, **1a** (59.2 mg, 400 µmol, 2.0 equiv.), tris(2,2'-bipyridine)ruthenium hexafluorophosphate (1.7 mg, 2.00 µmol, 1 mol%), and *N*,*N*-dimethylaniline (2.5 µL, 20.0 µmol, 10 mol%) in DMSO-d_6 (1 mL, 0.2 M). The crude residue was purified by flash chromatography (silica gel) from pure hexane to a mixture of 2% diethyl ether in hexane affording 5.9 mg of **P3** as a colourless oil (9%, *E*:*Z* > 20:1) and 22.3 mg of **P4** as a colourless oil (51%).

5.2 Unsuccessful starting materials

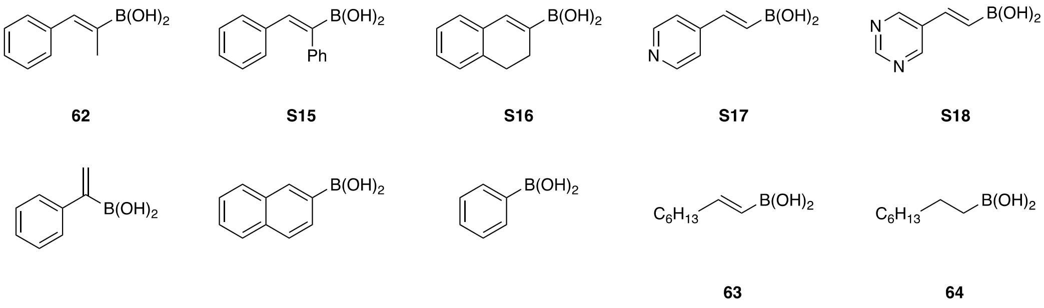

Figure 3: Unsuccessful styrenyl boronic acids.

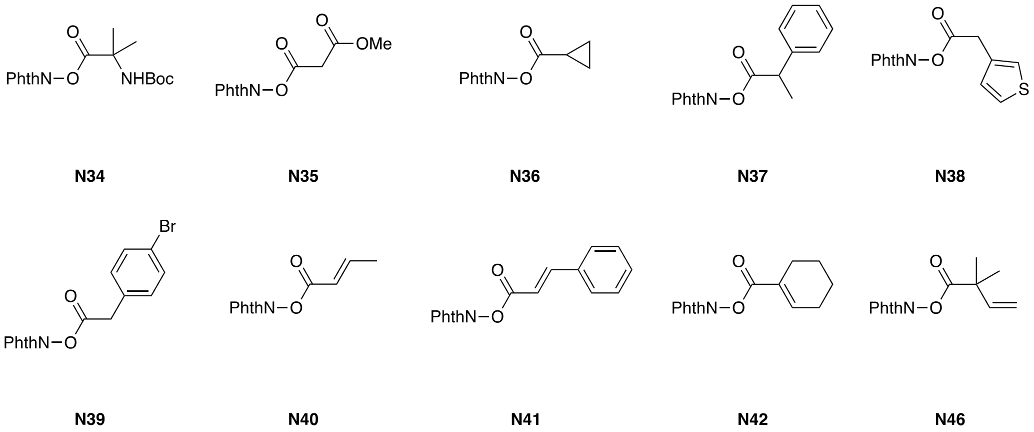

Figure 4: Unsuccessful NHPI esters.

6. Characterization

6.1 NHPI Esters

1,3-Dioxoisoindolin-2-yl 3-(4-fluorophenyl)propanoate, **2**

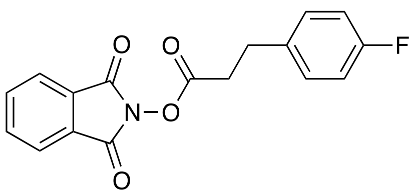

Prepared according to General Procedure 1A using 3-(4-fluorophenyl)propionic acid (1.68 g, 10.00 mmol, 1.0 equiv.), *N,N'*-diisopropyl-carbodiimide (1.70 mL, 11 mmol, 1.1 equiv.), 4-dimethylaminopyridine (122 mg, 1 mmol, 10 mol%), and *N*-hydroxyphthalimide (1.80 g, 11 mmol, 1.1 equiv.) in DCM (100 mL, 0.1 M). The crude residue was purified by flash chromatography (silica gel) from pure hexane to a mixture of 12% ethyl acetate in hexane affording 2.18 g of a white solid consistent with the desired product (70%).

^{1}H NMR (500 MHz, Chloroform-*d*) δ 7.97 – 7.84 (m, 2H), 7.84 – 7.74 (m, 2H), 7.39 – 7.14 (m, 2H), 7.12 – 6.88 (m, 2H), 3.07 (t, *J* = 7.71 Hz, 2H), 3.00 – 2.92 (m, 2H).

^{13}C NMR (126 MHz, Chloroform-*d*) δ 168.9, 162.0, 161.9 (d, $^{1}J_{CF}$ = 244.5 Hz), 135.0, 134.9, 130.0 (d, $^{3}J_{CF}$ = 7.9 Hz), 129.0, 124.2, 115.7 (d, $^{2}J_{CF}$ = 21.3 Hz), 33.0, 29.9.

^{19}F {^{1}H} NMR (377 MHz, Chloroform-*d*) δ –116.27.

Data are consistent with the literature.[8]

1,3-Dioxoisoindolin-2-yl cyclohexanecarboxylate, **4**

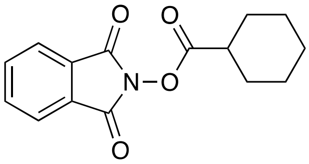

Prepared according to General Procedure 1A using cyclohexanecarboxylic acid (620 µL, 5.00 mmol, 1.0 equiv.), *N,N'*-diisopropyl-carbodiimide (852 µL, 5.50 mmol, 1.1 equiv.), 4-dimethylaminopyridine (61 mg, 500 µmol, 10 mol%), and *N*-hydroxyphthalimide (816 mg, 5.00 mmol, 1.0 equiv.) in DCM (50 mL, 0.1 M). The crude residue was purified by flash chromatography (silica gel) from pure hexane to a mixture of 8% ethyl acetate in hexane affording 938 mg of a white solid consistent with the desired product (69%).

^{1}H NMR (400 MHz, Chloroform-*d*) δ 7.91 – 7.85 (m, 2H), 7.82 – 7.75 (m, 2H), 2.73 (tt, *J* = 10.89, 3.79 Hz, 1H), 2.15 – 2.03 (m, 2H), 1.90 – 1.72 (m, 2H), 1.73 – 1.62 (m, 3H), 1.46 – 1.22 (m, 3H).

^{13}C NMR (126 MHz, Chloroform-*d*) δ 172.0, 162.2, 134.8, 129.1, 124.0, 40.6, 28.9, 25.6, 25.1.

Data are consistent with the literature.[9]

1,3-Dioxoisoindolin-2-yl cyclobutanecarboxylate, **N1**

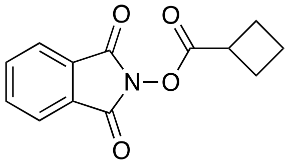

Prepared according to General Procedure 1A using cyclobutanecarboxylic acid (382 µL, 4.00 mmol, 1.0 equiv.), *N,N'*-diisopropyl-carbodiimide (681 µL, 4.40 mmol, 1.1 equiv.), 4-dimethylaminopyridine (49 mg, 400 µmol, 10 mol%), and *N*-hydroxyphthalimide (653 mg, 4.00 mmol, 1.0 equiv.) in DCM (40 mL, 0.1 M). The crude residue was purified by flash chromatography (silica gel) from pure hexane to a mixture of 8% of ethyl acetate in hexane affording 855 mg of a white solid consistent with the desired product (87%).

^{1}H NMR (500 MHz, Chloroform-*d*) δ 7.91 – 7.86 (m, 2H), 7.82 – 7.74 (m, 2H), 3.56 – 3.46 (m, 1H), 2.56 – 2.46 (m, 2H), 2.46 – 2.34 (m, 2H), 2.18 – 1.99 (m, 2H).

^{13}C NMR (126 MHz, Chloroform-*d*) δ 171.6, 162.2, 134.9, 129.1, 124.1, 35.1, 25.5, 18.9.

Data are consistent with the literature.[10]

1,3-Dioxoisoindolin-2-yl cyclopentanecarboxylate, **N2**

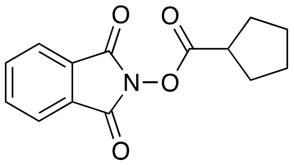

Prepared according to General Procedure 1A using cyclopentanecarboxylic acid (571 mg, 5.00 mmol, 1.0 equiv.), *N,N'*-diisopropyl-carbodiimide (852 µL, 5.50 mmol, 1.1 equiv.), 4-dimethylaminopyridine (61 mg, 500 µmol, 10 mol%), and *N*-hydroxyphthalimide (816 mg, 5.00 mmol, 1.0 equiv.) in DCM (50 mL, 0.1 M). The crude residue was purified by flash chromatography (silica gel) from pure hexane to a mixture of 8% of ethyl acetate in hexane affording 871 mg of a white solid consistent with the desired product (67%).

^{1}H NMR (500 MHz, Chloroform-*d*) δ 7.91 – 7.85 (m, 2H), 7.82 – 7.73 (m, 2H), 3.11 (tt, *J* = 8.71, 7.07 Hz, 1H), 2.15 – 1.97 (m, 4H), 1.87 – 1.71 (m, 2H), 1.73 – 1.62 (m, 2H).

^{13}C NMR (126 MHz, Chloroform-*d*) δ 173.0, 162.3, 134.8, 129.1, 124.0, 40.8, 30.4, 26.1.

Data are consistent with the literature.[11]

1,3-Dioxoisoindolin-2-yl cycloheptanecarboxylate, **N3**

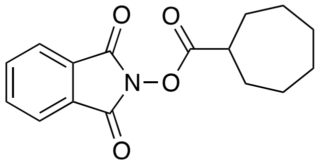

Prepared according to General Procedure 1A using cycloheptanecarboxylic acid (550 µL, 4.00 mmol, 1.0 equiv.), *N,N'*-diisopropyl-carbodiimide (681 µL, 4.40 mmol, 1.1 equiv.), 4-dimethylaminopyridine (49 mg, 400 µmol, 10 mol%), and *N*-hydroxyphthalimide (653 mg, 4.00 mmol, 1.0 equiv.) in DCM (40 mL, 0.1 M). The crude residue was purified by flash chromatography (silica gel) from pure hexane to a mixture of 4% of ethyl acetate in hexane affording 742 mg of a white solid consistent with the desired product (65%).

^{1}H NMR (500 MHz, Chloroform-*d*) δ 7.91 – 7.84 (m, 2H), 7.81 – 7.74 (m, 2H), 2.89 (tt, *J* = 9.29, 4.41 Hz, 1H), 2.18 – 2.09 (m, 2H), 1.93 – 1.82 (m, 2H), 1.85 – 1.77 (m, 2H), 1.66 – 1.51 (m, 6H).

^{13}C NMR (126 MHz, Chloroform-*d*) δ 172.9, 162.3, 134.8, 129.2, 124.0, 42.3, 30.9, 28.4, 26.4.

Data are consistent with the literature.[12]

1,3-Dioxoisoindolin-2-yl pivalate, **N4**

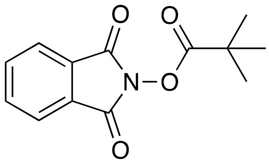

Prepared according to General Procedure 1A using pivalic acid (511 mg, 5.00 mmol, 1.0 equiv.), *N*,*N'*-diisopropyl-carbodiimide (852 μL, 5.50 mmol, 1.1 equiv.), 4-dimethylaminopyridine (61 mg, 500 μmol, 10 mol%), and *N*-hydroxyphthalimide (816 mg, 5.00 mmol, 1.0 equiv.) in DCM (50 mL, 0.1 M). The crude residue was purified by flash chromatography (silica gel) from pure hexane to a mixture of 8% of ethyl acetate in hexane affording 922 mg of a white solid consistent with the desired product (75%).

^{1}H NMR (500 MHz, Chloroform-*d*) δ 7.93 – 7.84 (m, 2H), 7.82 – 7.70 (m, 2H), 1.43 (s, 9H).

^{13}C NMR (126 MHz, Chloroform-*d*) δ 174.5, 162.2, 134.8, 129.2, 124.0, 38.5, 27.2.

Data are consistent with the literature.[13]

1,3-Dioxoisoindolin-2-yl butyrate, **N5**

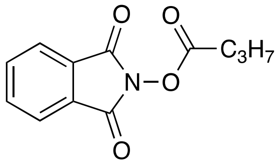

Prepared according to General Procedure 1A using butyric acid (341 μL, 4.00 mmol, 1.0 equiv.), *N*,*N'*-diisopropyl-carbodiimide (681 μL, 4.40 mmol, 1.1 equiv.), 4-dimethylaminopyridine (49 mg, 400 μmol, 10 mol%), and *N*-hydroxyphthalimide (653 mg, 4.00 mmol, 1.0 equiv.) in DCM (40 mL, 0.1 M). The crude residue was purified by flash chromatography (silica gel) from pure hexane to a mixture of 7% of ethyl acetate in hexane affording 561 mg of a colourless oil consistent with the desired product (56%).

^{1}H NMR (500 MHz, Chloroform-*d*) δ 7.91 – 7.86 (m, 2H), 7.81 – 7.76 (m, 2H), 2.65 (t, *J* = 7.31 Hz, 2H), 1.82 (q, *J* = 7.38 Hz, 2H), 1.07 (t, *J* = 7.41 Hz, 3H).

^{13}C NMR (126 MHz, Chloroform-*d*) δ 169.6, 162.1, 134.9, 129.1, 124.1, 32.9, 18.4, 13.5.

Data are consistent with the literature.[13]

1,3-Dioxoisoindolin-2-yl 2-methylbutanoate, **N6**

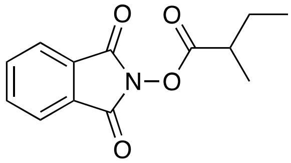

Prepared according to General Procedure 1A using (+/-)-2-methylbutyric acid (546 µL, 5.00 mmol, 1.0 equiv.), *N,N'*-diisopropyl-carbodiimide (852 µL, 5.50 mmol, 1.1 equiv.), 4-dimethylaminopyridine (61 mg, 500 µmol, 10 mol%), and *N*-hydroxyphthalimide (816 mg, 5.00 mmol, 1.0 equiv.) in DCM (50 mL, 0.1 M). The crude residue was purified by flash chromatography (silica gel) from pure hexane to a mixture of 6% of ethyl acetate in hexane affording 813 mg of a colourless oil consistent with the desired product (66%).

^{1}H NMR (500 MHz, Chloroform-*d*) δ 7.91 – 7.85 (m, 2H), 7.81 – 7.76 (m, 2H), 2.78 (h, *J* = 7.00 Hz, 1H), 1.92 – 1.80 (m, 1H), 1.75 – 1.64 (m, 1H), 1.35 (d, *J* = 6.94 Hz, 3H), 1.07 (t, *J* = 7.46 Hz, 3H).

^{13}C NMR (126 MHz, Chloroform-*d*) δ 172.8, 162.2, 134.8, 129.1, 124.0, 38.7, 27.0, 16.6, 11.4.

Data are consistent with the literature.[14]

1,3-Dioxoisoindolin-2-yl octanoate, **N7**

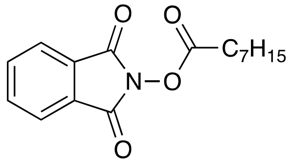

Prepared according to General Procedure 1A using octanoic acid (792 µL, 5.00 mmol, 1.0 equiv.), *N,N'*-diisopropyl-carbodiimide (852 µL, 5.50 mmol, 1.1 equiv.), 4-dimethylaminopyridine (61 mg, 500 µmol, 10 mol%), and *N*-hydroxyphthalimide (816 mg, 5.00 mmol, 1.0 equiv.) in DCM (50 mL, 0.1 M). The crude residue was purified by flash chromatography (silica gel) from pure hexane to a mixture of 6% of ethyl acetate in hexane affording 1.11 g of a white solid consistent with the desired product (71%).

^{1}H NMR (500 MHz, Chloroform-*d*) δ 7.91 – 7.85 (m, 2H), 7.81 – 7.76 (m, 2H), 2.66 (t, *J* = 7.49 Hz, 2H), 1.78 (p, *J* = 7.53 Hz, 2H), 1.49 – 1.39 (m, 2H), 1.38 – 1.24 (m, 6H), 0.89 (d, *J* = 6.66 Hz, 3H).

^{13}C NMR (126 MHz, Chloroform-*d*) δ 169.8, 162.2, 134.9, 129.1, 124.1, 31.7, 31.1, 28.9, 28.9, 24.8, 22.7, 14.2.

Data are consistent with the literature.[13]

1,3-Dioxoisoindolin-2-yl tetradecanoate, **N8**

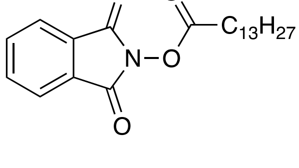

Prepared according to General Procedure 1A using tetradecanoic acid (114 mg, 500 µmol, 1.0 equiv.), *N,N'*-diisopropyl-carbodiimide (85 µL, 550 µmol, 1.1 equiv.), 4-dimethylaminopyridine (6 mg, 50.0 µmol, 10 mol%), and *N*-hydroxyphthalimide (89.7 mg, 550 µmol, 1.1 equiv.) in DCM (5 mL, 0.1 M). The crude

residue was purified by flash chromatography (silica gel) from pure hexane to a mixture of 3% of ethyl acetate in hexane affording 160 mg of a white solid consistent with the desired product (86%).

^{1}H NMR (500 MHz, Chloroform-*d*) δ 7.94 – 7.84 (m, 2H), 7.83 – 7.61 (m, 2H), 2.65 (t, *J* = 7.49 Hz, 2H), 1.77 (d, *J* = 7.54 Hz, 2H), 1.47 – 1.39 (m, 2H), 1.36 – 1.19 (m, 18H), 0.87 (t, *J* = 6.86 Hz, 3H).

^{13}C NMR (126 MHz, Chloroform-*d*) δ 169.8, 162.1, 134.8, 129.0, 124.0, 32.0, 31.1, 29.8, 29.8, 29.7, 29.7, 29.5, 29.5, 29.2, 28.9, 24., 22.8, 14.2.

Data are consistent with the literature.[9]

1,3-Dioxoisoindolin-2-yl 3-phenylpropanoate, **N9**

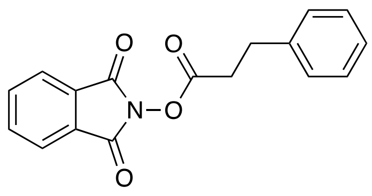

Prepared according to General Procedure 1A using phenylpropanoic acid (751 mg, 5.00 mmol, 1.0 equiv.), *N,N'*-diisopropyl-carbodiimide (852 µL, 5.50 mmol, 1.1 equiv.), 4-dimethylaminopyridine (61 mg, 500 µmol, 10 mol%), and *N*-hydroxyphthalimide (816 mg, 5.00 mmol, 1.0 equiv.) in DCM (50 mL, 0.1 M). The crude residue was purified by flash chromatography (silica gel) from pure hexane to a mixture of 10% of ethyl acetate in hexane affording 1.10 g of a white solid consistent with the desired product (75%).

^{1}H NMR (400 MHz, Chloroform-*d*) δ 7.93 – 7.86 (m, 2H), 7.83 – 7.76 (m, 2H), 7.37 – 7.31 (m, 2H), 7.29 – 7.21 (m, 3H), 3.14 – 3.07 (m, 2H), 3.02 – 2.96 (m, 2H).

^{13}C NMR (101 MHz, Chloroform-*d*) δ 169.0, 162.0, 139.3, 134.9, 129.0, 128.8, 128.4, 126.8, 124.1, 32.8, 30.7.

Data are consistent with the literature.[15]

1,3-Dioxoisoindolin-2-yl 3-(4-bromophenyl)propanoate, **N10**

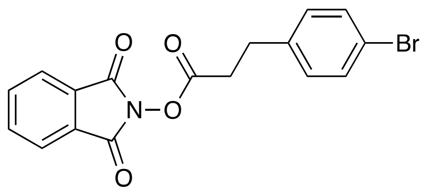

Prepared according to General Procedure 1A using 3-(4-bromophenyl)propionic acid (916 mg, 4 mmol, 1.0 equiv.), *N,N'*-diisopropyl-carbodiimide (681 µL, 4.40 mmol, 1.1 equiv.), 4-dimethylaminopyridine (49 mg, 400 µmol, 10 mol%), and *N*-hydroxyphthalimide (718 mg, 4.40 mmol, 1.1 equiv.) in DCM (40 mL, 0.1 M). The crude residue was purified by flash chromatography (silica gel) from pure hexane to a mixture of 18% of ethyl acetate in acetate affording 217 mg of a white solid consistent with the desired product (14%).

^{1}H NMR (500 MHz, Chloroform-*d*) δ 7.95 – 7.88 (m, 2H), 7.85 – 7.78 (m, 2H), 7.52 – 7.43 (m, 2H), 7.24 – 7.13 (m, 2H), 3.11 – 3.05 (m, 2H), 3.02 – 2.96 (m, 2H).

^{13}C NMR (126 MHz, Chloroform-*d*) δ 168.8, 162.0, 138.2, 135.0, 132.0, 130.2, 129.0, 124.2, 120.8, 32.6, 30.1.

IR (solid): 1788, 1735, 1610, 1487, 1463, 1450, 1369, 1282, 1186, 1153, 1068, 962 cm^{-1}.

HRMS (ESI): *m/z* calculated for $[M + H]^+$ $(C_{17}H_{13}BrNO_4)^+$: 374.0022; found = 374.0015.

Data are consistent with the literature.[16]

3-(4-Ethynylphenyl)propanoic acid, **N11-int1**

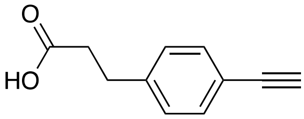

An oven-dried microwave vial was charged with dichlorobis(triphenylphosphine)palladium (14 mg, 20.0 µmol, 1 mol%), copper iodide (8 mg, 40.0 µmol, 2 mol%), and 3-(4-iodophenyl)propanoic acid (251 µL, 2.00 mmol, 1 equiv.). The vial was then sealed and purged with vacuum-N_2 cycles (3 times) and backfilled with N_2. Triethylamine (4 mL, 0.5 M) was added followed by trimethylsilylacetylene (264 µL, 2.50 mmol, 1.25 equiv.). The reaction mixture was stirred overnight at room temperature. The crude mixture was partitioned between DCM (20 mL) and an aqueous solution of 1 M NaOH (20 mL). Organic layer was extracted with an aqueous solution of 1 M NaOH (2 × 10 mL). Aqueous layers were combined and acidified with an aqueous solution of 2 M HCl (until pH ~ 2). Organics were extracted with DCM (3 × 15 mL). Organic layers were combined, washed with brine (20 mL), dried over sodium sulfate, filtered, and concentrated *in vacuo* affording 334 mg of a white solid consistent with the desired product (96%).

^{1}H NMR (400 MHz, Chloroform-*d*) δ 7.46 – 7.40 (m, 2H), 7.19 – 7.14 (m, 2H), 3.05 (s, 1H), 2.96 (t, *J* = 7.68 Hz, 2H), 2.71 – 2.65 (m, 2H). The carboxylic acid proton was not observed.

^{13}C NMR (101 MHz, Chloroform-*d*) δ 178.7, 141.2, 132.5, 128.5, 120.3, 83.6, 77.1, 35.3, 30.5.

Data are consistent with the literature.[17]

1,3-Dioxoisoindolin-2-yl 3-(4-ethynylphenyl)propanoate, **N11**

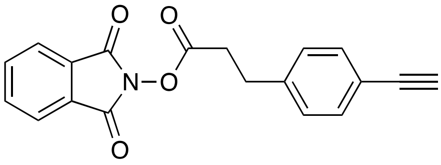

Prepared according to General Procedure 1B using 3-(4-ethynylphenyl)propanoic acid, **N11-int1** (315 mg, 1.81 mmol, 1.0 equiv.), EDC hydrochloride (417 mg, 2.17 mmol, 1.2 equiv.), 4-dimethylaminopyridine (22 mg, 181 µmol, 10 mol%), and *N*-hydroxyphthalimide (295 mg, 1.81 mmol, 1.0 equiv.) in DCM (18 mL, 0.1M). The crude residue was purified by flash chromatography (silica gel) from pure hexane to a mixture of 30% of diethyl ether in hexane affording 268 mg of a white solid consistent with the desired product (46%).

^{1}H NMR (500 MHz, Chloroform-*d*) δ 7.93 – 7.85 (m, 2H), 7.83 – 7.76 (m, 2H), 7.49 – 7.43 (m, 2H), 7.25 – 7.19 (m, 2H), 3.10 (t, *J* = 7.72 Hz, 2H), 3.06 (s, 1H), 3.01 – 2.96 (m, 2H).

^{13}C NMR (126 MHz, Chloroform-*d*) δ 168.8, 162.0, 140.1, 135.0, 132.6, 129.0, 128.5, 124.2, 120.7, 83.6, 77.2, 32.5, 30.5.

IR (solid): 3267, 1845, 1816, 1789, 1739, 1465, 1400, 1371, 1282, 1186, 1138, 1070, 1033, 964 cm^{-1}.

HRMS (ESI): *m/z* calculated for $[M + H]^+$ $(C_{19}H_{14}NO_4)^+$: 320.0917; found = 320.0914.

1,3-Dioxoisoindolin-2-yl 1-methylcyclohexane-1-carboxylate, **N12**

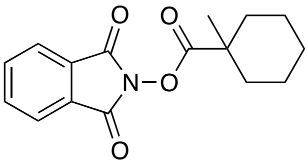

Prepared according to General Procedure 1B using 1-methyl-1-cyclohexanecarboxylic acid (284 mg, 2.00 mmol, 1.0 equiv.), EDC hydrochloride (460 mg, 2.40 mmol, 1.2 equiv.), 4-dimethylaminopyridine (24 mg, 200 µmol, 10 mol%), and *N*-hydroxyphthalimide (326 mg, 2.00 mmol, 1.0 equiv.) in DCM (20 mL, 0.1 M). The crude residue was purified by flash chromatography (silica gel) from pure hexane to a mixture of 4% of ethyl acetate in hexane affording 350 mg of a colourless oil consistent with the desired product (61%).

^{1}H NMR (500 MHz, Chloroform-*d*) δ 7.90 – 7.85 (m, 2H), 7.80 – 7.76 (m, 2H), 2.27 – 2.20 (m, 2H), 1.71 – 1.61 (m, 3H), 1.61 – 1.52 (m, 2H), 1.43 (s, 3H), 1.41 – 1.34 (m, 2H), 1.33 – 1.25 (m, 1H).

^{13}C NMR (126 MHz, Chloroform-*d*) δ 173.8, 162.4, 134.8, 129.2, 124.0, 43.3, 35.8, 26.9, 25.6, 23.2.

Data are consistent with the literature.[18]

1,3-Dioxoisoindolin-2-yl methyl glutarate, **N13**

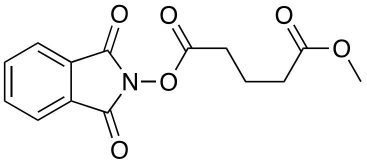

Prepared according to General Procedure 1B using mono-methyl glutarate (250 µL, 2.00 mmol, 1.0 equiv.), EDC hydrochloride (460 mg, 2.40 mmol, 1.2 equiv.), 4-dimethylaminopyridine (24 mg, 200 µmol, 10 mol%), and *N*-hydroxyphthalimide (326 mg, 2.00 mmol, 1.0 equiv.) in DCM (20 mL, 0.1 M). The crude residue was purified by flash chromatography (silica gel) from pure hexane to a mixture of 40% of diethyl ether in hexane affording 467 mg of a white solid consistent with the desired product (80%).

^{1}H NMR (500 MHz, Chloroform-*d*) δ 7.91 – 7.85 (m, 2H), 7.83 – 7.76 (m, 2H), 3.69 (s, 3H), 2.76 (t, *J* = 7.29 Hz, 2H), 2.50 (t, *J* = 7.28 Hz, 2H), 2.10 (p, *J* = 7.31 Hz, 2H).

^{13}C NMR (126 MHz, Chloroform-*d*) δ 173.1, 169.1, 162.0, 134.9, 129.0, 124.1, 51.9, 32.6, 30.2, 19.9.

Data are consistent with the literature.[19]

1,3-Dioxoisoindolin-2-yl 4-bromobutanoate, **N14**

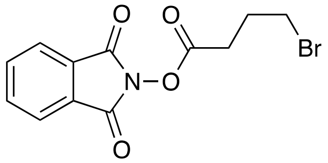

Prepared according to General Procedure 1A using 4-bromobutyric acid (835 mg, 5.00 mmol, 1.0 equiv.), *N,N'*-diisopropyl-carbodiimide (852 µL, 5.50 mmol, 1.1 equiv.), 4-dimethylaminopyridine (61 mg, 500 µmol, 10 mol%), and *N*-hydroxyphthalimide (816 mg, 5.00 mmol, 1.0 equiv.) in DCM (50 mL, 0.1 M). The crude residue was purified by flash chromatography (silica gel) from pure hexane to a mixture of 9% of ethyl acetate in hexane affording 865 mg of a white solid consistent with the desired product (55%).

^{1}H NMR (500 MHz, Chloroform-*d*) δ 7.89 – 7.82 (m, 2H), 7.80 – 7.73 (m, 2H), 3.52 (t, *J* = 6.37 Hz, 2H), 2.86 (t, *J* = 7.22 Hz, 2H), 2.30 (p, *J* = 6.79 Hz, 2H).

^{13}C NMR (126 MHz, Chloroform-*d*) δ 168.8, 161.9, 134.9, 128.8, 124.1, 31.8, 29.5, 27.5.

Data are consistent with the literature.[12]

1,3-Dioxoisoindolin-2-yl pent-4-enoate, **N15**

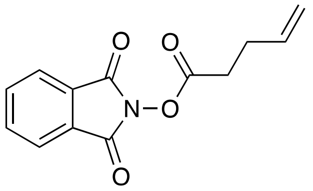

Prepared according to General Procedure 1A using 4-pentenoic acid (409 µL, 4.00 mmol, 1.0 equiv.), *N,N'*-diisopropyl-carbodiimide (681 µL, 4.40 mmol, 1.1 equiv.), 4-dimethylaminopyridine (49 mg, 400 µmol, 10 mol%), and *N*-hydroxyphthalimide (653 mg, 4.00 mmol, 1.0 equiv.) in DCM (40 mL, 0.1 M). The crude residue was purified by flash chromatography (silica gel) from pure hexane to a mixture of 5% of ethyl acetate in hexane affording 610 mg of a colourless oil consistent with the desired product (62%).

^{1}H NMR (500 MHz, Chloroform-*d*) δ 7.91 – 7.86 (m, 2H), 7.81 – 7.75 (m, 2H), 5.88 (ddt, *J* = 16.80, 10.18, 6.44 Hz, 1H), 5.16 (dd, *J* = 17.12, 1.62 Hz, 1H), 5.10 (dd, *J* = 10.25, 1.52 Hz, 1H), 2.78 (t, *J* = 7.45 Hz, 2H), 2.53 (q, *J* = 7.15 Hz, 2H).

^{13}C NMR (126 MHz, Chloroform-*d*) δ 169.1, 162.1, 135.3, 134.9, 129.0, 124.1, 116.8, 30.5, 28.6.

Data are consistent with the literature.[20]

1,3-Dioxoisoindolin-2-yl 2-(cyclopent-2-en-1-yl)acetate, **N16**

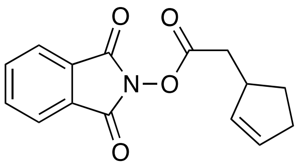

Prepared according to General Procedure 1A using 2-cyclopentene-1-acetic acid (631 mg, 5.00 mmol, 1.0 equiv.), *N,N'*-diisopropyl-carbodiimide (852 µL, 5.50 mmol, 1.1 equiv.), 4-dimethylaminopyridine (61 mg, 500 µmol, 10 mol%), and *N*-hydroxyphthalimide (816 mg, 5.00 mmol, 1.0 equiv.) in DCM (50 mL, 0.1 M). The crude residue was purified by flash chromatography (silica gel) from pure hexane to a mixture of 7% of ethyl acetate in hexane affording 550 mg of a beige solid consistent with the desired product (41%).

^{1}H NMR (500 MHz, Chloroform-*d*) δ 7.92 – 7.85 (m, 2H), 7.83 – 7.76 (m, 2H), 5.88 – 5.82 (m, 1H), 5.80 – 5.74 (m, 1H), 3.28 – 3.18 (m, 1H), 2.71 (d, *J* = 7.02 Hz, 1H), 2.66 (d, *J* = 7.95 Hz, 1H), 2.50 – 2.38 (m, 1H), 2.41 – 2.29 (m, 1H), 2.29 – 2.19 (m, 1H), 1.65 – 1.54 (m, 1H).

^{13}C NMR (126 MHz, Chloroform-*d*) δ 168.9, 162.1, 134.9, 132.7, 132.7, 129.0, 124.1, 42.1, 37.1, 32.0, 29.6.

IR (film): 1813, 1786, 1738, 1468, 1361, 1186, 1082, 972, 878, 694 cm^{-1}.

HRMS (ESI): *m/z* calculated for $[M + H]^+$ $(C_{15}H_{14}NO_4)^+$: 272.0917; found 272.0915.

1,3-Dioxoisoindolin-2-yl pent-4-ynoate, **N17**

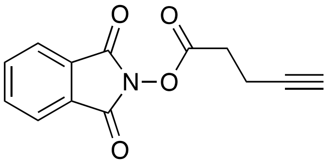

Prepared according to General Procedure 1A using 4-pentynoic acid (491 mg, 5.00 mmol, 1.0 equiv.), *N,N'*-diisopropyl-carbodiimide (852 µL, 5.50 mmol, 1.1 equiv.), 4-dimethylaminopyridine (61 mg, 500 µmol, 10 mol%), and *N*-hydroxyphthalimide (816 mg, 5.00 mmol, 1.0 equiv.) in DCM (50 mL, 0.1 M). The crude residue was purified by flash chromatography (silica gel) from pure hexane to a mixture of 12% of ethyl acetate in hexane affording 571 mg of a white solid consistent with the desired product (47%).

^{1}H NMR (400 MHz, Chloroform-*d*) δ 7.93 – 7.84 (m, 2H), 7.84 – 7.75 (m, 2H), 2.94 (t, *J* = 7.84, 2H), 2.65 (td, *J* = 7.57, 2.68 Hz, 2H), 2.07 (t, *J* = 2.67 Hz, 1H).

^{13}C NMR (176 MHz, Chloroform-*d*) δ 168.1, 161.9, 135.0, 129.0, 124.2, 81.0, 70.2, 30.5, 14.3.

Data are consistent with the literature.[21]

(*tert*-Butoxycarbonyl)glycine, **N18-int1**

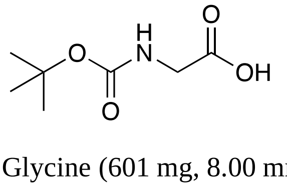

Glycine (601 mg, 8.00 mmol, 1.0 equiv.) and di-*tert*-butyl dicarbonate (2.10 g, 9.60 mmol, 1.2 equiv.) were dissolved in THF (40 mL, 0.2 M), and cooled down to 0 °C. Sodium hydroxide (320 mg, 8.00 mmol, 1.0 equiv.) was dissolved in water (10 mL) and then added dropwise to the reaction mixture at 0 °C. The reaction mixture was slowly warmed up to room temperature and stirred overnight. The volatiles were evaporated under vacuum and the resulting suspension was dissolved in water (40 mL). Organics were extracted with diethyl ether (30 mL). The aqueous layer was acidified with a solution of 1 M of HCl (pH ~ 2). Organics were extracted with diethyl ether (3 × 30 mL), dried over sodium sulfate, filtered, and

concentrated *in vacuo* affording 1.40 g of a white solid consistent with the desired product as a mixture of rotamers (100%, 0.6:0.4).

Major rotamer

^{1}H NMR (500 MHz, Chloroform-*d*) δ 9.88 (s, 1H), 5.13 (s, 1H), 3.96 (d, *J* = 5.64 Hz, 2H), 1.45 (s, 9H).

^{13}C NMR (126 MHz, Chloroform-*d*) δ 175.0, 156.1, 80.6, 42.4, 28.4.

Minor rotamer

^{1}H NMR (500 MHz, Chloroform-*d*) δ 9.88 (s, 1H), 6.83 (s, 1H), 3.89 (d, *J* = 4.91 Hz, 2H), 1.45 (s, 9H).

^{13}C NMR (126 MHz, Chloroform-*d*) δ 174.1, 157.4, 81.9, 43.5, 28.4.

Data are consistent with the literature.[22]

1,3-Dioxoisoindolin-2-yl (*tert*-butoxycarbonyl)glycinate, **N18**

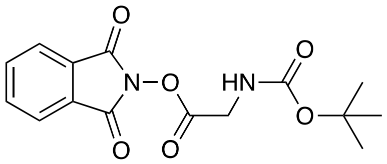

Prepared according to General Procedure 1B using (*tert*-butoxycarbonyl)glycine, **N18-int1** (350 mg, 2.00 mmol, 1.0 equiv.), EDC hydrochloride (460 mg, 2.40 mmol, 1.2 equiv.), 4-dimethylaminopyridine (24 mg, 200 μmol, 10 mol%), and *N*-hydroxyphthalimide (326 mg, 2.00 mmol, 1.0 equiv.) in DCM (20 mL, 0.1 M). The crude residue was dissolved with the minimum amount of DCM and hexane was added to precipitate the salt, 245 mg of a white solid was recovered after filtration consistent with the desired product as a mixture of rotamers (38%, 0.76:0.24).

Major rotamer

^{1}H NMR (500 MHz, Chloroform-*d*) δ 7.92 – 7.87 (m, 2H), 7.82 – 7.78 (m, 2H), 5.08 (t, *J* = 6.08 Hz, 1H), 4.25 – 4.19 (m, 2H), 1.46 (s, 9H).

^{13}C NMR (126 MHz, Chloroform-*d*) δ 167.3, 161.6, 155.4, 135.0, 128.9, 124.2, 80.8, 40.5, 28.4.

Minor rotamer

^{1}H NMR (500 MHz, Chloroform-*d*) δ 7.92 – 7.87 (m, 2H), 7.82 – 7.78 (m, 2H), 5.20 (s, 1H), 4.36 (d, *J* = 5.94 Hz, 2H), 1.50 (s, 9H).

^{13}C NMR (126 MHz, Chloroform-*d*) δ 167.1, 161.6, 155.2, 134.3, 129.3, 123.5, 82.0, 41.9, 28.1.

Data are consistent with the literature.[9]

(*tert*-Butoxycarbonyl)alanine, **N19-int1**

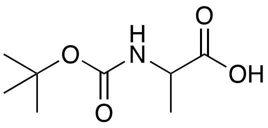

DL-Alanine (713 mg, 8.00 mmol, 1.0 equiv.) and di-*tert*-butyl dicarbonate (2.10 g, 9.60 mmol, 1.2 equiv.) were dissolved in THF (40 mL, 0.2 M), and cooled down to 0 °C. Sodium hydroxide (320 mg, 8.00 mmol, 1.0 equiv.) was dissolved in water (10 mL) and then added dropwise to the reaction mixture at 0 °C. The reaction mixture was slowly warmed up to room temperature and stirred overnight. Volatiles were evaporated under vacuum and the resulting suspension was dissolved in water (40 mL). Organics were extracted with diethyl ether (30 mL). The aqueous layer was acidified with a solution of 1 M of HCl (pH ~ 2). Organics were extracted with diethyl ether (3 × 30 mL), dried over sodium sulfate, filtered, and concentrated *in vacuo* affording 1.50 g of a white solid consistent with the desired product as a mixture of rotamers (99%, 0.62:0.38).

Major rotamer

^{1}H NMR (500 MHz, Chloroform-*d*) δ 10.03 (s, 1H), 5.13 (broad s, 1H), 4.41 – 4.27 (m, 1H), 1.44 (s, 9H), 1.42 (d, *J* = 7.22 Hz, 3H).

^{13}C NMR (126 MHz, Chloroform-*d*) δ 178.1, 155.6, 80.4, 49.2, 28.4, 18.5.

Minor rotamer

^{1}H NMR (500 MHz, Chloroform-*d*) δ 10.03 (s, 1H), 6.67 (broad s, 1H), 4.21 – 4.10 (m, 1H), 1.44 (s, 9H), 1.42 (d, *J* = 7.22 Hz, 3H).

^{13}C NMR (126 MHz, Chloroform-*d*) δ 177.5, 156.9, 81.7, 50.3, 28.4, 18.5.

Data are consistent with the literature.[23]

1,3-Dioxoisoindolin-2-yl (*tert*-butoxycarbonyl)alaninate, **N19**

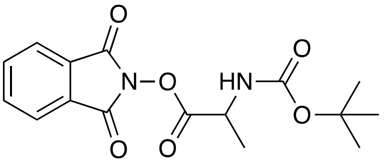

Prepared according to General Procedure 1B using (*tert*-butoxycarbonyl)alanine, **N19-int1** (378 mg, 2.00 mmol, 1.0 equiv.), EDC hydrochloride (460 mg, 2.40 mmol, 1.2 equiv.), 4-dimethylaminopyridine (24 mg, 200 μmol, 10 mol%), and *N*-hydroxyphthalimide (326 mg, 2.00 mmol, 1.0 equiv.) in DCM (20 mL, 0.1 M). The crude residue was dissolved with the minimum amount of DCM and hexane was added to precipitate the salt, 188 mg of a white solid was recovered after filtration consistent with the desired product as a mixture of rotamers (28%, 0.75:0.25).

Major rotamer

^{1}H NMR (500 MHz, Chloroform-*d*) δ 7.91 – 7.86 (m, 2H), 7.82 – 7.77 (m, 2H), 5.07 (d, *J* = 6.45 Hz, 1H), 4.76 (p, *J* = 7.64 Hz, 1H), 1.62 (d, *J* = 7.24 Hz, 3H), 1.46 (2s, 9H).

^{13}C NMR (126 MHz, Chloroform-*d*) δ 170.1, 161.7, 154.8, 135.0, 129.0, 124.2, 80.7, 47.8, 28.4, 19.0.

Minor rotamer

^{1}H NMR (500 MHz, Chloroform-*d*) δ 7.91 – 7.86 (m, 2H), 7.82 – 7.77 (m, 2H), 4.82 (broad s, 1H), 4.51 (broad, 1H), 1.62 (d, J = 7.24 Hz, 3H), 1.46 (2s, 9H).

^{13}C NMR (126 MHz, Chloroform-*d*) δ 170.1, 161.7, 154.8, 135.0, 129.0, 124.2, 80.7, 49.2, 28.2, 18.2.

Data are consistent with the literature.[24]

Methyl 3-((*tert*-butyldimethylsilyl)oxy)-2,2-dimethylpropanoate, **N20-int1**

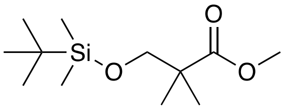

Imidazole (374 mg, 5.50 mmol, 1.1 equiv.) and *tert*-butyldimethylchlorosilane (829 mg, 5.50 mmol, 1.1 equiv.) were dissolved in DMF (20 mL, 0.25 M). Methyl 2,2-dimethyl-3-hydroxypropionate (638 µL, 5.00 mmol, 1.0 equiv.) was then added and the reaction mixture was left to stir overnight. Once completion was reached, the reaction mixture was portioned between brine (50 mL) and ethyl acetate (50 mL). The aqueous layer was extracted with ethyl acetate (2 × 30 mL). Organic layers were combined and washed with brine (50 mL), dried over sodium sulfate, filtered, and concentrated *in vacuo*. The crude residue was purified by flash chromatography (silica gel) from pure hexane to a mixture of 2% of diethyl ether in hexane affording 740 mg of a colourless oil consistent with the desired product (60%).

^{1}H NMR (500 MHz, Chloroform-*d*) δ 3.65 (s, 3H), 3.56 (s, 2H), 1.14 (s, 6H), 0.86 (s, 9H), 0.01 (s, 6H).

^{13}C NMR (126 MHz, Chloroform-*d*) δ 177.4, 70.2, 51.8, 45.0, 25.9, 22.0, 18.3, –5.5.

Data are consistent with the literature.[25]

3-((*tert*-Butyldimethylsilyl)oxy)-2,2-dimethylpropanoic acid, **N20-int2**

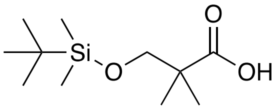

Methyl 3-((*tert*-butyldimethylsilyl)oxy)-2,2-dimethylpropanoate, **N20-int1** (739 mg, 3.00 mmol, 1.0 equiv.) was dissolved in a mixture of THF:H_2O:MeOH (2:2:1, 20 mL, 0.15 M) and cooled down to 0 °C. Lithium hydroxide (216 mg, 9.00 mmol, 3.0 equiv.) was added and the reaction mixture was slowly warmed up to room temperature, and left to stir for 48 hours. The reaction mixture was partitioned between ethyl acetate (30 mL) and water (30 mL). Aqueous layer was acidified with a 1M solution of HCl (pH ~ 2). Organics were extracted with ethyl acetate (3 × 30 mL), combined, dried over sodium sulfate, filtered, and concentrated *in vacuo* affording 535 mg of a colourless oil consistent with the desired product (77%).

^{1}H NMR (500 MHz, Chloroform-*d*) δ 3.59 (s, 2H), 1.18 (s, 6H), 0.89 (s, 9H), 0.07 (s, 6H). The carboxylic acid proton is not observed.

^{13}C NMR (126 MHz, Chloroform-*d*) δ 181.4, 69.8, 44.3, 25.9, 22.0, 18.3, –5.5.

Data are consistent with the literature.[25]

1,3-Dioxoisoindolin-2-yl 3-((*tert*-butyldimethylsilyl)oxy)-2,2-dimethylpropanoate, **N20**

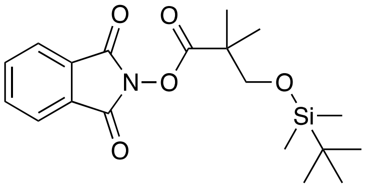

Prepared according to General Procedure 1B using 3-((*tert*-butyldimethylsilyl)oxy)-2,2-dimethylpropanoic acid, **N20-int2** (697 mg, 3.00 mmol, 1.0 equiv.), EDC hydrochloride (690 mg, 3.60 mmol, 1.2 equiv.), 4-dimethylaminopyridine (37 mg, 300 μmol, 10 mol%), and *N*-hydroxyphthalimide (489 mg, 3.00 mmol, 1.0 equiv.) in DCM (30 mL, 0.1 M). The crude residue was purified by flash chromatography (silica gel) from pure hexane to a mixture of 5% of diethyl ether in hexane affording 262 mg of a colourless oil consistent with the desired product (23%).

^{1}H NMR (500 MHz, Chloroform-*d*) δ 7.91 – 7.86 (m, 2H), 7.80 – 7.75 (m, 2H), 3.75 (s, 2H), 1.39 (s, 6H), 0.92 (s, 9H), 0.09 (s, 6H).

^{13}C NMR (126 MHz, Chloroform-*d*) δ 172.9, 162.1, 134.7, 129.2, 124.0, 69.3, 45.0, 25.9, 22.0, 18.4, –5.5.

Data are consistent with the literature.[26]

1,3-Dioxoisoindolin-2-yl 3-methyloxetane-3-carboxylate, **N21**

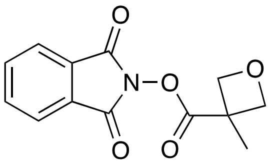

Prepared according to General Procedure 1A using 3-methyloxetane-3-carboxylic acid (232 mg, 2.00 mmol, 1.0 equiv.), *N*,*N'*-diisopropyl-carbodiimide (341 μL, 2.20 mmol, 1.1 equiv.), 4-dimethylaminopyridine (24 mg, 200 μmol, 10 mol%), and *N*-hydroxyphthalimide (326 mg, 2.00 mmol, 1.0 equiv.) in DCM (20 mL, 0.1 M). The crude residue was purified by flash chromatography (silica gel) from pure hexane to a mixture of 25% of ethyl acetate in hexane affording 215 mg of a white solid consistent with the desired product (41%).

^{1}H NMR (500 MHz, Chloroform-*d*) δ 7.91 – 7.86 (m, 2H), 7.82 – 7.77 (m, 2H), 5.16 (d, *J* = 6.25 Hz, 2H), 4.55 (d, *J* = 6.26 Hz, 2H), 1.83 (s, 3H).

^{13}C NMR (126 MHz, Chloroform-*d*) δ 170.6, 161.9, 135.0, 129.0, 124.2, 79.1, 43.4, 21.5.

Data are consistent with the literature.[26]

1,3-Dioxoisoindolin-2-yl 3-oxocyclobutane-1-carboxylate, **N22**

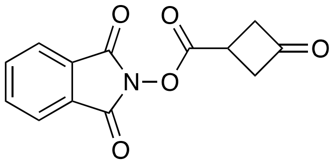

Prepared according to General Procedure 1A using 3-oxocyclobutanecarboxylic acid (456 mg, 4.00 mmol, 1.0 equiv.), *N,N'*-diisopropyl-carbodiimide (681 µL, 4.40 mmol, 1.1 equiv.), 4-dimethylaminopyridine (49 mg, 400 µmol, 10 mol%), and *N*-hydroxyphthalimide (653 mg, 4.00 mmol, 1.0 equiv.) in DCM (40 mL, 0.1 M). The crude residue was purified by flash chromatography (silica gel) from pure hexane to a mixture of 18% of ethyl acetate in hexane affording 221 mg of a pale yellow solid consistent with the desired product (21%). Some impurities were noted; however, the compound was used without further purification.

^{1}H NMR (500 MHz, Chloroform-*d*) δ 7.95 – 7.86 (m, 2H), 7.85 – 7.78 (m, 2H), 3.72 – 3.46 (m, 5H).

^{13}C NMR (126 MHz, Chloroform-*d*) δ 201.4, 170.8, 161.9, 135.1, 129.0, 124.3, 52.4, 25.2.

Data are consistent with the literature.[27]

1-(*tert*-Butoxycarbonyl)piperidine-4-carboxylic acid, **N23-int1**

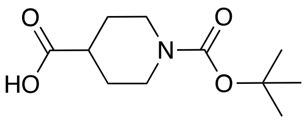

Isonipecotic acid (1.03 g, 8.00 mmol, 1.0 equiv.) was dissolved in 1,4-dioxane (30 mL) and cooled down to 0 °C. 8 mL of a solution of 1 M of sodium hydroxide (1 equiv.) were then slowly added. Di-*tert*-butyl dicarbonate (2.10 g, 9.60 mmol, 1.2 equiv.) was dissolved in 1,4-dioxane (10 mL) and was added dropwise at 0 °C. The reaction mixture was slowly warmed up to room temperature and stirred overnight. Volatiles were evaporated under vacuum and the resulting suspension was dissolved in water (40 mL). Organics were extracted with ethyl acetate (2 × 20 mL). The aqueous layer was acidified with a solution of 1 M of HCl (pH ~ 2). The precipitated white solid was filtered, washed with cold water (2 × 20 mL), and dried under vacuum overnight affording 1.51 g of a white solid consistent with the desired product (82%).

^{1}H NMR (500 MHz, Chloroform-*d*) δ 4.02 (broad s, 2H), 2.85 (bt, *J* = 11.95 Hz, 2H), 2.48 (tt, *J* = 10.96, 3.90 Hz, 1H), 1.90 (bd, *J* = 13.15 Hz, 2H), 1.74 – 1.51 (m, 2H), 1.45 (s, 9H). The carboxylic acid proton is not observed.

^{13}C NMR (126 MHz, Chloroform-*d*) δ 180.4, 154.9, 79.9, 43.1, 40.9, 28.6, 27.9.

Data are consistent with the literature.[28]

1-(*tert*-Butyl) 4-(1,3-dioxoisoindolin-2-yl) piperidine-1,4-dicarboxylate, **N23**

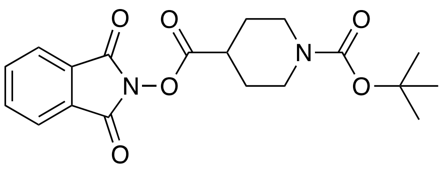

Prepared according to General Procedure 1A using 1-(*tert*-butoxycarbonyl)piperidine-4-carboxylic acid, **N23-int1** (917 mg, 4.00 mmol, 1.0 equiv.), *N,N'*-diisopropyl-carbodiimide (681 µL, 4.40 mmol, 1.1 equiv.), 4-dimethylaminopyridine (49 mg, 400 µmol, 10 mol%), and *N*-hydroxyphthalimide (653 mg, 4.00 mmol, 1.0 equiv.) in DCM (40 mL, 0.1 M). The crude residue was purified by flash chromatography (silica gel) from pure hexane to a mixture of 25% of ethyl acetate in hexane affording 1.14 g of a beige solid consistent with the desired product (76%).

^{1}H NMR (500 MHz, Chloroform-*d*) δ 7.87 – 7.90 (m, 2H), 7.82 – 7.76 (m, 2H), 4.10 – 3.95 (m, 2H), 3.05 – 2.96 (m, 2H), 2.91 (tt, *J* = 10.40, 3.97 Hz, 1H), 2.10 – 2.02 (m, 2H), 1.89 – 1.79 (m, 2H), 1.46 (s, 9H).

^{13}C NMR (126 MHz, Chloroform-*d*) δ 170.8, 162.1, 154.7, 134.9, 129.0, 124.1, 80.0, 42.7 (broad), 38.7, 28.5, 27.9.

Data are consistent with the literature.[10]

1-(*tert*-Butoxycarbonyl)piperidine-3-carboxylic acid, **N24-int1**

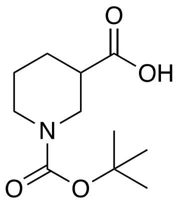

3-Piperidinecarboxylic acid (1.03 g, 8.00 mmol, 1.0 equiv.) and di-*tert*-butyl dicarbonate (2.10 g, 9.60 mmol, 1.2 equiv.) were dissolved in THF (40 mL, 0.2 M) and cooled down to 0 °C. Sodium hydroxide (320 mg, 8.00 mmol, 1.0 equiv.) was dissolved in water (10 mL) and then added dropwise to the reaction mixture at 0 °C. The reaction mixture was slowly warmed up to room temperature and stirred overnight. Volatiles were evaporated under vacuum and the resulting suspension was dissolved in water (40 mL). Organics were extracted with ethyl acetate (2 × 30 mL). The aqueous layer was acidified with a solution of 1 M of HCl (pH ~ 2). Organics were extracted with ethyl acetate (3 × 30 mL), dried over sodium sulfate, filtered, and concentrated *in vacuo* affording 1.84 g of a white solid consistent with the desired product (100%).

^{1}H NMR (500 MHz, Chloroform-*d*) δ 11.13 (broad s, 1H), 4.31 – 3.94 (m, 1H), 3.94 – 3.81 (broad s, 1H), 3.21 – 2.92 (broad s, 1H), 2.89 – 2.78 (m, 1H), 2.54 – 2.40 (m, 1H), 2.12 – 2.00 (m, 1H), 1.75 – 1.67 (m, 1H), 1.66 – 1.57 (m, 1H), 1.52 – 1.46 (m, 1H), 1.45 (s, 9H).

^{13}C NMR (126 MHz, Chloroform-*d*) δ 179.3, 154.9, 80.1, 45.7, 43.7, 41.2, 28.5, 27.3, 24.2.

Data are consistent with the literature.[29]

1-(*tert*-Butyl) 3-(1,3-dioxoisoindolin-2-yl) piperidine-1,3-dicarboxylate, **N24**

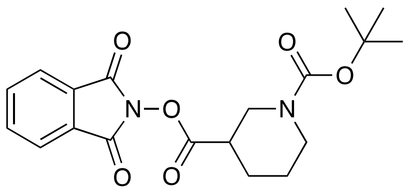

Prepared according to General Procedure 1A using 1-(*tert*-butoxycarbonyl)piperidine-3-carboxylic acid, **N24-int1** (459 mg, 2.00 mmol, 1.0 equiv.), *N,N'*-diisopropyl-carbodiimide (341 µL, 2.20 mmol, 1.1 equiv.), 4-dimethylaminopyridine (24 mg, 200 µmol, 10 mol%), and *N*-hydroxyphthalimide (326 mg, 2.00 mmol, 1.0 equiv.) in DCM (20 mL, 0.1 M). The crude residue was purified by flash chromatography (silica gel) from pure hexane to a mixture of 20% of ethyl acetate in hexane affording 515 mg of a white solid consistent with the desired product (69%).

^{1}H NMR (500 MHz, Chloroform-*d*) δ 7.92 – 7.83 (m, 2H), 7.83 – 7.66 (m, 2H), 4.30 (bd, *J* = 78.63 Hz, 1H), 3.95 (d, *J* = 13.27 Hz, 1H), 3.17 (broad s, 1H), 2.96 – 2.79 (m, 2H), 2.36 – 2.21 (m, 1H), 1.87 – 1.74 (m, 2H), 1.60 – 1.50 (m, 1H), 1.46 (s, 9H).

^{13}C NMR (126 MHz, Chloroform-*d*) δ 169.6, 161.9, 154.6, 134.9, 129.0, 124.1, 80.2, 45.4, 43.4, 39.2, 28.5, 27.6, 24.1.

Data are consistent with the literature.[19]

1-(*tert*-Butoxycarbonyl)piperidine-2-carboxylic acid, **N25-int1**

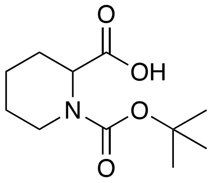

Piperidine-2-carboxylic acid (1.03 g, 8.00 mmol, 1.0 equiv.) and di-*tert*-butyl dicarbonate (2.10 g, 9.60 mmol, 1.2 equiv.) were dissolved in THF (40 mL, 0.2 M), and cooled down to 0 °C. Sodium hydroxide (320 mg, 8.00 mmol, 1.0 equiv.) was dissolved in water (10 mL) and then added dropwise to the reaction mixture at 0 °C. The reaction mixture was slowly warmed up to room temperature and stirred overnight. Volatiles were evaporated under vacuum and the resulting suspension was dissolved in water (40 mL). Organics were extracted with ethyl acetate (2 × 20 mL). The aqueous layer was acidified with a solution of 1 M of HCl (pH ~ 2). Organics were extracted with ethyl acetate (3 × 30 mL), dried over sodium sulfate, filtered, and concentrated *in vacuo* affording 1.54 g of a white solid as the desired product as a mixture of rotamers (84%, 0.55:0.45).

Major rotamer

^{1}H NMR (500 MHz, Chloroform-*d*) δ 11.28 (broad s, 1H), 5.01 – 4.88 (m, 1H), 3.97 – 3.85 (m, 1H), 3.05 – 2.93 (m, 1 H), 2.29 – 2.15 (m, 1H), 1.74 – 1.64 (m, 2H), 1.63 – 1.57 (m, 1H), 1.46 (s, 9H), 1.42 – 1.37 (m, 1H), 1.35 – 1.24 (m, 1H).

^{13}C NMR (126 MHz, Chloroform-*d*) δ 178.2, 156.3, 80.4, 53.7, 42.2, 28.5, 26.7, 24.9, 20.9.

Minor rotamer

^{1}H NMR (500 MHz, Chloroform-*d*) δ 11.28 (broad s, 1H), 4.81 – 4.69 (m, 1H), 4.07 – 3.98 (m, 1H), 2.93 – 2.82 (m, 1H), 2.29 – 2.15 (m, 1H), 1.74 – 1.64 (m, 2H), 1.63 – 1.57 (m, 1H), 1.43 (s, 9H), 1.42 – 1.37 (m, 1H), 1.35 – 1.24 (m, 1H).

^{13}C NMR (126 MHz, Chloroform-*d*) δ 178.4, 155.6, 80.4, 54.8, 41.2, 28.4, 26.7, 24.6, 20.8.

Data are consistent with the literature.[30]

1-(*tert*-Butyl) 2-(1,3-dioxoisoindolin-2-yl) piperidine-1,2-dicarboxylate, **N25**

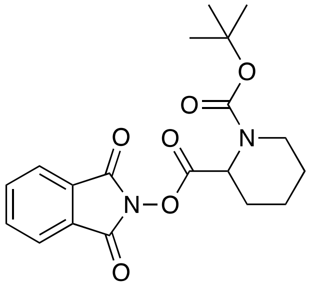

Prepared according to General Procedure 1A using 1-(*tert*-butoxycarbonyl)piperidine-2-carboxylic acid, **N25-int1** (459 mg, 2.00 mmol, 1.0 equiv.), *N,N'*-diisopropyl-carbodiimide (341 µL, 2.20 mmol, 1.1 equiv.), 4-dimethylaminopyridine (24 mg, 200 µmol, 10 mol%), and *N*-hydroxyphthalimide (326 mg, 2.00 mmol, 1.0 equiv.) in DCM (20 mL, 0.1 M). The crude residue was purified by flash chromatography (silica gel) from pure hexane to a mixture of 11% of ethyl acetate in hexane affording 639 mg of a white solid consistent with the desired product as a mixture of rotamers (85%, 0.68:0.32).

Major rotamer

^{1}H NMR (500 MHz, Chloroform-*d*) δ 7.91 – 7.85 (m, 2H), 7.83 – 7.76 (m, 2H), 5.13 (app. d, *J* = 6.13 Hz, 1H), 4.10 – 4.03 (m, 1H), 3.15 – 2.96 (m, 1H), 2.46 – 2.29 (m, 1H), 1.92 – 1.72 (m, 3H), 1.56 – 1.40 (m, 11H).

^{13}C NMR (126 MHz, Chloroform-*d*) δ 168.9, 161.9, 155.3, 134.9, 129.1, 124.1, 81.2, 53.7, 41.3, 28.2, 27.3, 24.5, 20.4.

Minor rotamer

^{1}H NMR (500 MHz, Chloroform-*d*) δ 7.91 – 7.85 (m, 2H), 7.83 – 7.76 (m, 2H), 5.38 (broad s, 1H), 4.03 – 3.90 (m, 1H), 3.15 – 2.96 (m, 1H), 2.46 – 2.29 (m, 1H), 1.92 – 1.72 (m, 3H), 1.56 – 1.40 (m, 11H).

^{13}C NMR (126 MHz, Chloroform-*d*) δ 168.7, 161.9, 155.5, 134.9, 129.1, 124.1, 80.7, 52.7, 42.3, 28.4, 27.3, 24.9, 20.7.

Data are consistent with the literature.[31]

1-(*tert*-Butyl) 2-(1,3-dioxoisoindolin-2-yl) (*S*)-pyrrolidine-1,2-dicarboxylate, **N26**

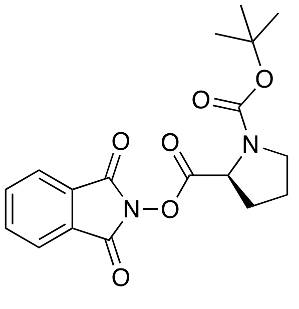

Prepared according to General Procedure 1A using *N-(tert*-butoxycarbonyl)-L-proline (1.08 g, 5.00 mmol, 1.0 equiv.), *N,N'*-diisopropyl-carbodiimide (852 µL, 5.50 mmol, 1.1 equiv.), 4-dimethylaminopyridine (61 mg, 500 µmol, 10 mol%), and *N*-hydroxyphthalimide (816 mg, 5.00 mmol, 1.0 equiv.) in DCM (50 mL, 0.1 M). The crude residue was purified by flash chromatography (silica gel) from pure hexane to a mixture of 20% of ethyl acetate in hexane affording 1.32 g of a white solid consistent with the desired product as a mixture of rotamers (73%, 0.82:018).

Major rotamer

^{1}H NMR (500 MHz, Chloroform-*d*) δ 7.91 – 7.84 (m, 2H), 7.82 – 7.73 (m, 2H), 4.60 (dd, *J* = 8.83, 3.70 Hz, 1H), 3.62 (ddd, *J* = 10.42, 7.86, 4.46 Hz, 1H), 3.48 (dt, *J* = 10.47, 7.48 Hz, 1H), 2.48 – 2.31 (m, 2H), 2.13 – 1.92 (m, 2H), 1.51 (s, 9H).

^{13}C NMR (126 MHz, Chloroform-*d*) δ 169.8, 161.8, 153.6, 134.9, 129.0, 124.1, 81.2, 57.3, 46.4, 31.5, 28.2, 23.7.

Minor rotamer

^{1}H NMR (500 MHz, Chloroform-*d*) δ 7.91 – 7.84 (m, 2H), 7.82 – 7.73 (m, 2H), 4.70 (dd, *J* = 7.49, 4.72 Hz, 1H), 3.55 (ddd, *J* = 11.64, 7.89, 4.25 Hz, 1H), 3.42 (dt, *J* = 10.30, 7.55 Hz, 1H), 2.48 – 2.31 (m, 2H), 2.13 – 1.92 (m, 2H), 1.47 (s, 9H).

^{13}C NMR (126 MHz, Chloroform-*d*) δ 169.5, 161.7, 154.2, 134.8, 129.1, 124.1, 80.5, 57.2, 46.6, 30.4, 28.5, 24.5.

Data consistent with the literature.[9]

1,3-Dioxoisoindolin-2-yl 3-(furan-2-yl)propanoate, **N27**

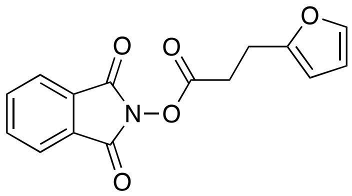

Prepared according to General Procedure 1B using 3-(2-furyl)propionic acid (280 mg, 2.00 mmol, 1.0 equiv.), EDC hydrochloride (460 mg, 2.40 mmol, 1.2 equiv.), 4-dimethylaminopyridine (24 mg, 200 µmol, 10 mol%), and *N*-hydroxyphthalimide (653 mg, 4.00 mmol, 1.0 equiv.) in DCM (40 mL, 0.1 M). The crude residue was dissolved with the minimum amount of DCM and hexane was added to precipitate the salt, 410 mg of a white solid was recovered after filtration consistent with the desired product (72%).

^{1}H NMR (500 MHz, Chloroform-*d*) δ 7.92 – 7.83 (m, 2H), 7.83 – 7.74 (m, 2H), 7.34 (dd, *J* = 1.91, 0.87 Hz, 1H), 6.31 (dd, *J* = 3.20, 1.89 Hz, 1H), 6.13 (dd, *J* = 3.16, 0.97 Hz, 1H), 3.13 – 3.09 (m, 2H), 3.05 – 2.98 (m, 2H).

^{13}C NMR (126 MHz, Chloroform-*d*) δ 168.7, 162.0, 152.7, 141.7, 134.9, 129.0, 124.1, 110.5, 106.2, 29.8, 23.2.

Data are consistent with the literature.[32]

1,3-Dioxoisoindolin-2-yl 3-(1*H*-indol-3-yl)propanoate, **N28**

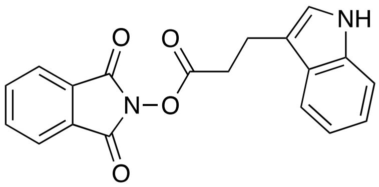

Prepared according to General Procedure 1B using 3-indolepropionic acid (378 mg, 2.00 mmol, 1.0 equiv.), EDC hydrochloride (460 mg, 2.40 mmol, 1.2 equiv.), 4-dimethylaminopyridine (24 mg, 200 μmol, 10 mol%), and *N*-hydroxyphthalimide (653 mg, 4.00 mmol, 1.0 equiv.) in DCM (40 mL, 0.1 M). The crude residue was dissolved with the minimum amount of DCM and hexane was added to precipitate the salt, 538 mg of a yellow solid was recovered after filtration consistent with the desired product (77%).

^{1}H NMR (500 MHz, Chloroform-*d*) δ 8.06 (s, 1H), 7.92 – 7.83 (m, 2H), 7.82 – 7.76 (m, 2H), 7.62 (d, *J* = 7.86 Hz, 1H), 7.38 (d, *J* = 8.06 Hz, 1H), 7.24 – 7.19 (m, 1H), 7.18 – 7.11 (m, 2H), 3.26 (t, *J* = 7.55 Hz, 2H), 3.07 (t, *J* = 7.55 Hz, 2H).

^{13}C NMR (126 MHz, Chloroform-*d*) δ 169.4, 162.1, 136.4, 134.9, 129.0, 127.1, 124.1, 122.3, 122.1, 119.6, 118.6, 113.8, 111.4, 32.0, 20.5.

Data are consistent with the literature.[33]

tert-Butyl 3-(3-((1,3-dioxoisoindolin-2-yl)oxy)-3-oxopropyl)-1*H*-indole-1-carboxylate, **N29**

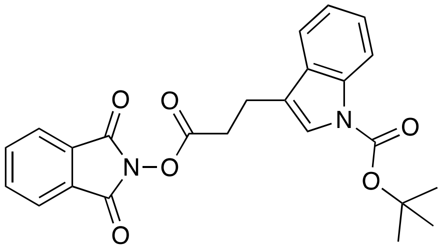

In a round bottom flask, 1,3-dioxoisoindolin-2-yl 3-(1*H*-indol-3-yl)propanoate, **N28** (267 mg, 800 μmol, 1.0 equiv.), di-*tert*-butyl dicarbonate (175 mg, 800 μmol, 1.0 equiv.), and 4-dimethylaminopyridine (5 mg, 40.0 μmol, 5 mol%) were dissolved in THF (8 mL, 0.1M). The reaction mixture was stirred overnight at room temperature. Once completion was reached, the mixture was partitioned between a solution of 1 M of HCl (10 mL) and diethyl ether (20 mL). Aqueous layer was extracted with diethyl ether (2 × 20 mL). Organic layers were combined, dried over sodium sulfate, filtered, and concentrated *in vacuo*. The crude residue was dissolved with the minimum amount of DCM and hexane was added to precipitate the salt, 256 mg of a yellow solid was recovered after filtration consistent with the desired product (74%).

^{1}H NMR (500 MHz, Chloroform-*d*) δ 8.23 – 8.10 (m, 1H), 7.92 – 7.86 (m, 2H), 7.83 – 7.76 (m, 2H), 7.55 (ap. d, *J* = 7.74 Hz, 1H), 7.50 (broad s, 1H), 7.37 – 7.32 (m, 1H), 7.30 – 7.24 (m, 1H), 3.22 – 3.17 (m, 2H), 3.11 – 3.06 (m, 2H), 1.68 (s, 9H).

^{13}C NMR (126 MHz, Chloroform-*d*) δ 169.1, 162.0, 149.8, 135.7, 134.9, 130.0, 129.0, 124.7, 124.1, 123.2, 122.7, 118.7, 118.2, 115.5, 83.7, 31.1, 28.3, 20.2.

Data are consistent with the literature.[34]

1,3-Dioxoisoindolin-2-yl 3-(pyridin-3-yl)propanoate, **N30**

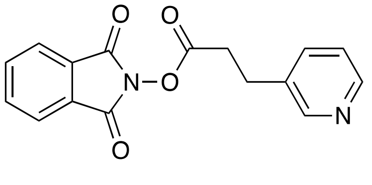

Prepared according to General Procedure 1B using 3-pyridinepropionic acid (302 mg, 2.00 mmol, 1.0 equiv.), EDC hydrochloride (460 mg, 2.40 mmol, 1.2 equiv.), 4-dimethylaminopyridine (24 mg, 200 μmol, 10 mol%), and *N*-hydroxyphthalimide (326 mg, 2.00 mmol, 1.0 equiv.) in DCM (20 mL, 0.1 M). The crude residue was purified by flash chromatography (silica gel) from pure hexane to a mixture of 50% of ethyl acetate in hexane affording 115 mg of white solid consistent with the desired product (19 %).

^{1}H NMR (500 MHz, Chloroform-*d*) δ 8.54 (s, 1H), 8.50 (d, *J* = 4.74 Hz, 1H), 7.89 – 7.84 (m, 2H), 7.80 – 7.74 (m, 2H), 7.60 (dt, *J* = 7.79, 1.97 Hz, 1H), 7.29 – 7.23 (m, 1H), 3.10 (t, *J* = 7.56 Hz, 2H), 2.99 (t, *J* = 7.41 Hz, 2H).

^{13}C NMR (126 MHz, Chloroform-*d*) δ 168.6, 161.9, 149.8, 148.2, 136.1, 134.9, 134.7, 128.9, 124.1, 123.7, 32.4, 27.8.

Data are consistent with the literature.[35]

1-(1,3-Dioxoisoindolin-2-yl) 3-methyl bicyclo[1.1.1]pentane-1,3-dicarboxylate, **N31**

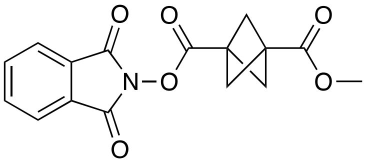

Prepared according to General Procedure 1B using 3-(methoxycarbonyl)bicyclo[1.1.1]pentane-1-carboxylic acid (510 mg, 3.00 mmol, 1.0 equiv.), EDC hydrochloride (690 mg, 3.60 mmol, 1.2 equiv.) 4-dimethylaminopyridine (37 mg, 300 μmol, 10 mol%), and *N*-hydroxyphthalimide (489 mg, 3.00 mmol, 1.0 equiv.) in DCM (30 mL, 0.1 M). The crude residue was dissolved with the minimum amount of DCM and hexane was added to precipitate the salt, 658 mg of a white solid was recovered after filtration consistent with the desired product (70%).

^{1}H NMR (500 MHz, Chloroform-*d*) δ 7.91 – 7.86 (m, 2H), 7.82 – 7.77 (m, 2H), 3.72 (s, 3H), 2.55 (s, 6H).

^{13}C NMR (126 MHz, Chloroform-*d*) δ 169.0, 164.8, 161.8, 135.0, 129.0, 124.2, 53.7, 52.2, 38.7, 35.5.

Data are consistent with the literature.[36]

3-(4-(4,4,5,5-Tetramethyl-1,3,2-dioxaborolan-2-yl)phenyl)propanoic acid, **N32-int1**

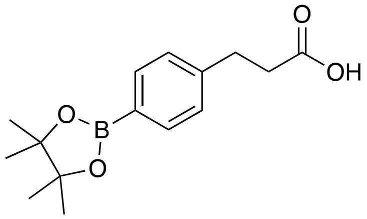

In an oven-dried round bottom flask, charged with a Teflon-coated stir bar, 3-(4-bromophenyl)propionic acid (916 mg, 4.00 mmol, 1.0 equiv.), bis(pinacolato)diboron (1.52 g, 6.00 mmol, 1.5 equiv.), potassium acetate (1.18 g, 12.0 mmol, 3.0 equiv.), and 1,1'-bis(diphenylphosphino)ferrocenepalladium (II) chloride (146 mg, 200 μmol, 5 mol%) were weighed out. The flask was sealed and purged with vacuum-N_2 and backfilled with N_2. Dry and degassed 1,4-dioxane (25 mL, 0.16 M) was added and the reaction mixture was stirred at 90 °C overnight. Once completion was reached, the reaction mixture was concentrated *in vacuo.* The crude residue

was partitioned between an aqueous solution of NaOH (2 M, 15 mL) and ethyl acetate (30 mL). Organics were extracted with an aqueous solution of NaOH (2 M, 15 mL). Aqueous layers were combined, acidified (pH ~ 4) with an aqueous solution of HCl (2 M). Organics were extracted with ethyl acetate (3 × 20 mL). Organic layers were combined, washed with brine (30 mL), dried over sodium sulfate, filtered, and concentrated *in vacuo* affording 1.10 g of a brown solid consistent with the desired product (95%). It contained B_2Pin_2 or related adducts.

^{1}H NMR (500 MHz, Chloroform-*d*) δ 7.77 – 7.73 (m, 2H), 7.24 – 7.19 (m, 2H), 2.97 (t, *J* = 7.85 Hz, 2H), 2.67 (t, *J* = 7.86 Hz, 2H), 1.33 (s, 12H). Carboxylic acid proton is not observed.

^{11}B NMR (96 MHz, Chloroform-*d*) δ 30.79.

^{13}C NMR (126 MHz, Chloroform-*d*) δ 178.5, 143.8, 135.2, 127.9, 83.9, 35.7, 31.0, 25.0. The boron-bearing carbon is not observed due to quadrupolar relaxation.

Data are consistent with the literature.[37]

1,3-Dioxoisoindolin-2-yl 3-(4-(4,4,5,5-tetramethyl-1,3,2-dioxaborolan-2-yl)phenyl)propanoate, **N32**

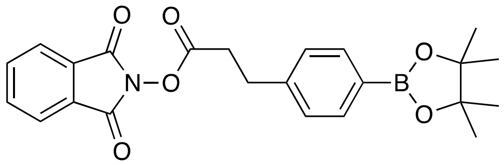

Prepared according to General Procedure 1B using 3-(4-(4,4,5,5-Tetramethyl-1,3,2-dioxaborolan-2-yl)phenyl)propanoic acid, **N32-int1** (500 mg, 1.81 mmol, 1.0 equiv.), EDC hydrochloride (417 mg, 2.17 mmol, 1.2 equiv.), 4-dimethylaminopyridine (22 mg, 181 μmol, 10 mol%), and *N*-hydroxyphthalimide (295 mg, 1.81 mmol, 1.0 equiv.) in DCM (18 mL, 0.1 M). The crude residue was purified by flash chromatography (silica gel) from pure hexane to a mixture of 40% of diethyl ether in hexane affording 178 mg of white solid consistent with the desired product (23%).

^{1}H NMR (500 MHz, Chloroform-*d*) δ 7.90 – 7.86 (m, 2H), 7.82 – 7.76 (m, 4H), 7.30 – 7.26 (m, 2H), 3.14 – 3.09 (m, 2H), 3.01 – 2.96 (m, 2H), 1.34 (s, 12H).

^{11}B NMR (96 MHz, Chloroform-*d*) δ 31.45.

^{13}C NMR (126 MHz, Chloroform-*d*) δ 168.9, 162.0, 142.5, 135.4, 134.9, 129.0, 127.8, 124.1, 83.9, 32.6, 30.8, 25.0. The boron-bearing carbon is not observed due to quadrupolar relaxation.

IR (solid): 1822, 1786, 1737, 1610, 1471, 1446, 1371, 1354, 1317, 1267, 1190, 1139, 1080, 964 cm^{-1}.

HRMS (ESI): *m/z* calculated for $[M + H]^+$ $(C_{23}H_{25}BNO_6)^+$: 422.1769; found = 422.1777.

1,3-Dioxoisoindolin-2-yl hept-6-enoate, **61**

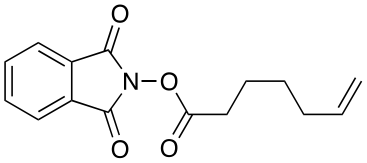

Prepared according to General Procedure 1A using 6-heptenoic acid (271 μL, 2.00 mmol, 1.0 equiv.), *N*,*N'*-diisopropyl-carbodiimide (341 μL, 2.20 mmol, 1.1 equiv.), 4-dimethylaminopyridine (24 mg, 200 μmol,

10 mol%), and *N*-hydroxyphthalimide (326 mg, 2.00 mmol, 1.0 equiv.) in DCM (20 mL, 0.1 M). The crude residue was purified by flash chromatography (silica gel) from pure hexane to a mixture of 4% of ethyl acetate in hexane affording 406 mg of a colourless oils consistent with the desired product (99%).

^{1}H NMR (500 MHz, Chloroform-*d*) δ 7.90 – 7.84 (m, 2H), 7.81 – 7.73 (m, 2H), 5.80 (ddt, *J* = 16.89, 10.11, 6.64 Hz, 1H), 5.03 (dq, *J* = 17.15, 1.81 Hz, 1H), 4.97 (dq, *J* = 10.22, 1.69 Hz, 1H), 2.66 (t, *J* = 7.41 Hz, 2H), 2.11 (q, *J* = 7.13 Hz, 2H), 1.79 (p, *J* = 7.48 Hz, 2H), 1.54 (p, *J* = 7.50 Hz, 2H).

^{13}C NMR (126 MHz, Chloroform-*d*) δ 169.6, 162.1, 138.1, 134.9, 129.0, 124.0, 115.1, 33.2, 30.9, 28.0, 24.2.

Data are consistent with the literature.[12]

1,3-Dioxoisoindolin-2-yl 2-cyclopropylacetate, **64**

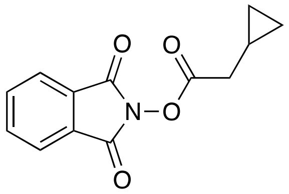

Prepared according to General Procedure 1B using cyclopropylacetic acid (372 µL, 4.00 mmol, 1.0 equiv.), EDC hydrochloride (920 mg, 4.80 mmol, 1.2 equiv.), 4-dimethylaminopyridine (49 mg, 400 µmol, 10 mol%), and *N*-hydroxyphthalimide (653 mg, 4.00 mmol, 1.0 equiv.) in DCM (40 mL, 0.1 M). The crude residue was dissolved with the minimum amount of DCM and hexane was added to precipitate the salt, 675 mg of a white solid was recovered after filtration consistent with the desired product (69%).

^{1}H NMR (500 MHz, Chloroform-*d*) δ 7.90 – 7.85 (m, 2H), 7.80 – 7.76 (m, 2H), 2.58 (d, *J* = 7.11 Hz, 2H), 1.22 – 1.12 (m, 1H), 0.69 – 0.62 (m, 2H), 0.35 – 0.27 (m, 2H).

^{13}C NMR (126 MHz, Chloroform-*d*) δ 169.1, 162.1, 134.9, 129.0, 124.0, 36.1, 6.6, 4.7.

Data are consistent with the literature.[38]

1-(*tert*-Butoxycarbonyl)indoline-2-carboxylic acid, **N33-int1**

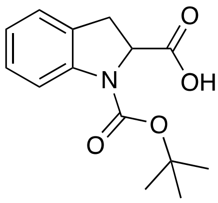

Indoline-2-carboxylic acid (1.31 g, 8 mmol, 1.0 equiv.) and di-*tert*-butyl dicarbonate (2.10 g, 9.60 mmol, 1.2 equiv.) were dissolved in THF (40 mL, 0.2 M), and cooled down to 0 °C. Sodium hydroxide (320 mg, 8.00 mmol, 1.0 equiv.) was dissolved in water (10 mL) and then added dropwise to the reaction mixture at 0 °C. The reaction mixture was slowly warmed up to room temperature and stirred overnight. Volatiles were evaporated under vacuum and the resulting suspension was dissolved in water (40 mL). Organics were extracted with diethyl ether (30 mL). The aqueous layer was acidified with a solution of 1 M of HCl (pH ~ 2). Organics were extracted with diethyl ether (3 × 30 mL), dried over sodium sulfate, filtered, and concentrated in *vacuo* affording 1.52 g of a pale brown solid as the desired product as a mixture of rotamers (72%, 0.62:0.38).

Major rotamer:

^{1}H NMR (500 MHz, Chloroform-*d*) δ 11.23 (s, 1H), 7.93 (d, *J* = 8.13 Hz, 1H), 7.23 (t, *J* = 7.86 Hz, 1H), 7.14 (d, *J* = 7.14 Hz, 1H), 6.99 (t, *J* = 7.44 Hz, 1H), 4.89 (dd, *J* = 11.56, 4.66 Hz, 1H), 3.55 (dd, *J* = 16.81, 11.40 Hz, 1H), 3.20 (dd, *J* = 16.71, 5.09 Hz, 1H), 1.54 (s, 9H).

^{13}C NMR (126 MHz, Chloroform-*d*) δ 178.2, 151.6, 142.4, 128.0, 127.7, 124.4, 122.8, 114.6, 81.7, 60.1, 32.6, 28.2.

Minor rotamer:

^{1}H NMR (500 MHz, Chloroform-*d*) δ 11.23 (s, 1H), 7.52 (d, *J* = 8.05 Hz, 1H), 7.23 (t, *J* = 7.86 Hz, 1H), 7.14 (d, *J* = 7.14 Hz, 1H), 6.99 (t, *J* = 7.44 Hz, 1H), 5.00 (d, *J* = 11.56 Hz, 1H), 3.55 (dd, *J* = 16.81, 11.40 Hz, 1H), 3.20 (dd, *J* = 16.71, 5.09 Hz, 1H), 1.64 (s, 9H).

^{13}C NMR (126 MHz, Chloroform-*d*) δ 177.5, 153.1, 141.3, 128.8, 127.9, 124.9, 122.8, 114.7, 82.9, 59.9, 31.7, 28.4.

IR (film): 2980, 1703, 1603, 1485, 1465, 1370, 1319, 1252, 1149, 1045, 1020, 907, 727 cm^{-1}.

HRMS: *m/z* calculated for $[M+Na]^+$ $(C_{14}H_{17}NO_4Na)^+$: 286.1050; found 286.1046.

1-(*tert*-Butyl) 2-(1,3-dioxoisoindolin-2-yl) indoline-1,2-dicarboxylate, **N33**

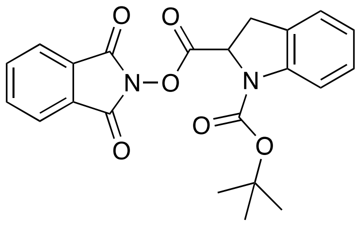

Prepared according to General Procedure 1B using 1-(*tert*-butoxycarbonyl)indoline-2-carboxylic acid, **N33-int1** (527 mg, 2.00 mmol, 1.0 equiv.), EDC hydrochloride (460 mg, 2.40 mmol, 1.2 equiv.), 4-dimethylaminopyridine (24 mg, 200 μmol, 10 mol%), and *N*-hydroxyphthalimide (326 mg, 2.00 mmol, 1.0 equiv.) in DCM (20 mL, 0.1 M). The crude residue was purified by flash chromatography (silica gel) from pure hexane to a mixture of 11% of diethyl ether in hexane affording 278 mg of white solid consistent with the desired product (34%).

^{1}H NMR (500 MHz, Chloroform-*d*) δ 7.95 – 7.90 (m, 1H), 7.90 – 7.85 (m, 2H), 7.82 – 7.76 (m, 2H), 7.22 (app. t, *J* = 7.92 Hz, 1H), 7.19 (app. d, *J* = 7.53 Hz, 1H), 6.99 (app. t, *J* = 7.45 Hz, 1H), 5.22 (dd, *J* = 11.75, 4.61 Hz, 1H), 3.73 (dd, *J* = 16.83, 11.73 Hz, 1H), 3.53 (dd, *J* = 16.78, 4.72 Hz, 1H), 1.60 (s, 9H).

^{13}C NMR (126 MHz, Chloroform-*d*) δ 168.5, 161.7, 151.3, 142.3, 135.0, 129.0, 128.3, 127.3, 124.7, 124.2, 123.1, 114.8, 82.8, 58.5, 33.2, 28.2.

IR (film): 2978, 2360, 1820, 1790, 1714, 1605, 1529, 1485, 1466, 1383, 1369, 1147, 1086, 982 cm^{-1}.

HRMS: *m/z* calculated for $[M]^+$ $(C_{22}H_{20}N_2O_6)^+$: 408.1316; found 408.1316.

2-((*tert*-Butoxycarbonyl)amino)-2-methylpropanoic acid, **N34-int1**

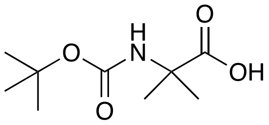

2-Aminoisobutyric acid (825 mg, 8.00 mmol, 1.0 equiv.) and di-*tert*-butyl dicarbonate (2.10 g, 9.60 mmol, 1.2 equiv.) were dissolved in THF (40 mL, 0.2 M), and cooled down to 0 °C. Sodium hydroxide (320 mg, 8.00 mmol, 1.0 equiv.) was dissolved in water (10 mL) and then added dropwise to the reaction mixture at 0 °C. The reaction mixture was slowly warmed up to room temperature and stirred overnight. Volatiles were evaporated under vacuum and the resulting suspension was dissolved in water (40 mL). Organics were extracted with ethyl acetate (2 × 30 mL). The aqueous layer was acidified with a solution of 1 M of HCl (pH ~ 2). Organics were extracted with ethyl acetate (3 × 30 mL), dried over sodium sulfate, filtered, and concentrated *in vacuo* affording 1.57 g of a white solid as the desired product (97%).

^{1}H NMR (500 MHz, Chloroform-*d*) δ 10.48 (broad s, 1H), 6.39 – 5.12 (2 × broad s, 1H), 1.52 (s, 6H), 1.43 (s, 9H).

^{13}C NMR (126 MHz, Chloroform-*d*) δ 180.1, 155.2, 80.2, 56.2, 28.4, 25.4.

Data are consistent with the literature.[39]

1,3-Dioxoisoindolin-2-yl 2-((*tert*-butoxycarbonyl)amino)-2-methylpropanoate, **N34**

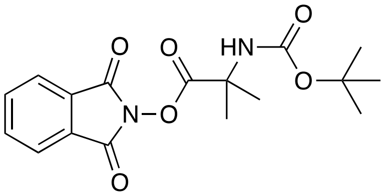

Prepared according to General Procedure 1B using 2-((*tert*-butoxycarbonyl)amino)-2-methylpropanoic acid, **N34-int1** (406 mg, 2.00 mmol, 1.0 equiv.), EDC hydrochloride (460 mg, 2.40 mmol, 1.2 equiv.), 4-dimethylaminopyridine (24 mg, 200 μmol, 10 mol%) *N*-hydroxyphthalimide (653 mg, 4.00 mmol, 1.0 equiv.) in DCM (40 mL, 0.1 M). The crude residue was dissolved with the minimum amount of DCM and hexane was added to precipitate the salt, 497 mg of a white solid was recovered after filtration consistent with the desired product (71%).

^{1}H NMR (500 MHz, Chloroform-*d*) δ 7.90 – 7.85 (m, 2H), 7.81 – 7.74 (m, 2H), 5.02 (broad s, 1H), 1.70 (s, 6H), 1.51 (s, 9H).

^{13}C NMR (126 MHz, Chloroform-*d*) δ 171.2, 161.8, 154.4, 134.8, 129.1, 124.0, 80.6, 55.9, 28.3, 25.7.

Data are consistent with the literature.[40]

1,3-Dioxoisoindolin-2-yl methyl malonate, **N35**

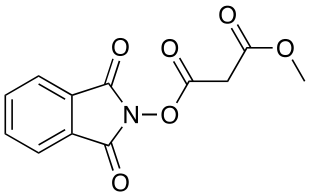

Prepared according to General Procedure 1A using 3-methoxy-3-oxopropanoic acid (209 µL, 2.00 mmol, 1.0 equiv.), *N,N'*-diisopropyl-carbodiimide (341 µL, 2.20 mmol, 1.1 equiv.), 4-dimethylaminopyridine (24 mg, 200 µmol, 10 mol%), and *N*-hydroxyphthalimide (326 mg, 2.00 mmol, 1.0 equiv.) in DCM (20 mL, 0.1 M). The crude residue was purified by flash chromatography (silica gel) from pure hexane to a mixture of 30% of ethyl acetate in hexane affording 290 mg of a white solid consistent with the desired product (55%).

^{1}H NMR (500 MHz, Chloroform-*d*) δ 7.92 – 7.85 (m, 2H), 7.84 – 7.77 (m, 2H), 3.83 (s, 3H), 3.74 (s, 2H).

^{13}C NMR (126 MHz, Chloroform-*d*) δ 164.9, 162.8, 161.6, 135.1, 128.9, 124.2, 53.3, 38.2.

IR (film): 1821, 1790, 1735, 1611, 1468, 1439, 1358, 1189, 1136, 1080, 975, 955, 914, 875 cm^{-1}.

HRMS: *m/z* calculated for $[M+Na]^+$ $(C_{12}H_9NO_6Na)^+$: 286.0322; found 286.0318.

1,3-Dioxoisoindolin-2-yl cyclopropanecarboxylate, **N36**

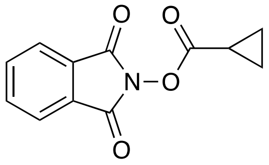

Prepared according to General Procedure 1A using cyclopropanecarboxylic acid (417 µL, 5.00 mmol, 1.0 equiv.), *N,N'*-diisopropyl-carbodiimide (852 µL, 5.50 mmol, 1.1 equiv.), 4-dimethylaminopyridine (61 mg, 500 µmol, 10 mol%), and *N*-hydroxyphthalimide (816 mg, 5.00 mmol, 1.0 equiv.) in DCM (50 mL, 0.1 M). The crude residue was purified by flash chromatography (silica gel) from pure hexane to a mixture of 10% of ethyl acetate in hexane affording 761 mg of a white solid consistent with the desired product (66%).

^{1}H NMR (500 MHz, Chloroform-*d*) δ 7.91 – 7.85 (m, 2H), 7.81 – 7.73 (m, 2H), 1.99 – 1.92 (m, 1H), 1.29 – 1.24 (m, 2H), 1.20 – 1.14 (m, 2H).

^{13}C NMR (126 MHz, Chloroform-*d*) δ 171.3, 162.2, 134.9, 129.0, 124.1, 10.8, 10.4.

IR (solid): 1800, 1777, 1746, 1611, 1464, 1430, 1387, 1373, 1287, 1182, 1134, 1113, 1015, 1003, 975, 964, 876, 789, 692 cm^{-1}.

HRMS (ESI): *m/z* calculated for $[M + H]^+$ $(C_{12}H_{10}NO_4)^+$: 232.0604; found 232.0607.

Data consistent with the literature.[14]

1,3-Dioxoisoindolin-2-yl 2-phenylpropanoate, **N37**

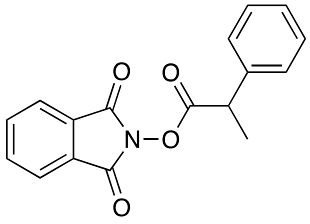

Prepared according to General Procedure 1A using 2-phenylpropionic acid (683 µL, 5.00 mmol, 1.0 equiv.), *N,N'*-diisopropyl-carbodiimide (852 µL, 5.50 mmol, 1.1 equiv.), 4-dimethylaminopyridine (61 mg, 500 µmol,

10 mol%), and *N*-hydroxyphthalimide (816 mg, 5.00 mmol, 1.0 equiv.) in DCM (50 mL, 0.1 M). The crude residue was purified by flash chromatography (silica gel) from pure hexane to a mixture of 8% of ethyl acetate in hexane affording 1.08 g of a colourless oil consistent with the desired product (73%).

^{1}H NMR (500 MHz, Chloroform-*d*) δ 7.89 – 7.83 (m, 2H), 7.81 – 7.74 (m, 2H), 7.44 – 7.37 (m, 4H), 7.36 – 7.30 (m, 1H), 4.12 (q, *J* = 7.18 Hz, 1H), 1.68 (d, *J* = 7.22 Hz, 3H).

^{13}C NMR (126 MHz, Chloroform-*d*) δ 170.9, 162.0, 138.5, 134.9, 129.1, 128.0, 127.7 124.1, 43.1, 19.1.

Data are consistent with the literature.[41]

1,3-Dioxoisoindolin-2-yl 2-(thiophen-3-yl)acetate, **N38**

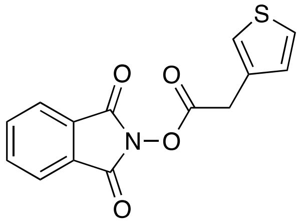

Prepared according to General Procedure 1B using 3-thiopheneacetic acid (284 mg, 2.00 mmol, 1.0 equiv.), EDC hydrochloride (460 mg, 2.40 mmol, 1.2 equiv.), 4-dimethylaminopyridine (24 mg, 200 µmol, 10 mol%), and *N*-hydroxyphthalimide (653 mg, 4.00 mmol, 1.0 equiv.) in DCM (40 mL, 0.1 M). The crude residue was dissolved with the minimum amount of DCM and hexane was added to precipitate the salt, 313 mg of a white solid was recovered after filtration consistent with the desired product (54%).

^{1}H NMR (500 MHz, Chloroform-*d*) δ 7.92 – 7.86 (m, 2H), 7.82 – 7.77 (m, 2H), 7.35 (dd, *J* = 4.93, 2.98 Hz, 1H), 7.33 – 7.31 (m, 1H), 7.13 (dd, *J* = 4.99, 1.37 Hz, 1H), 4.03 (s, 2H).

^{13}C NMR (126 MHz, Chloroform-*d*) δ 167.4, 162.0, 135.0, 130.9, 129.0, 128.3, 126.5, 124.2, 124.0, 32.6.

Data are consistent with the literature.[42]

2-(2-(4-Bromophenyl)acetyl)isoindoline-1,3-dione, **N39**

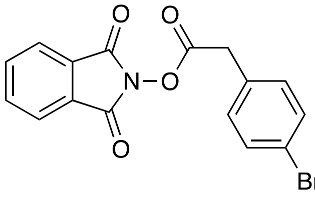

Prepared according to General Procedure 1A using 4-bromophenylacetic acid (1.08 g, 5.00 mmol, 1.0 equiv.), *N,N'*-diisopropyl-carbodiimide (852 µL, 5.50 mmol, 1.1 equiv.), 4-dimethylaminopyridine (61 mg, 500 µmol, 10 mol%), and *N*-hydroxyphthalimide (816 mg, 5.00 mmol, 1.0 equiv.) in DCM (50 mL, 0.1 M). The crude was purified by flash chromatography from pure hexane to a mixture of 12% of ethyl acetate in hexane affording 1.42 g of a white solid consistent with the desired product (79%).

^{1}H NMR (500 MHz, Chloroform-*d*) δ 7.98 – 7.85 (m, 2H), 7.86 – 7.64 (m, 2H), 7.58 – 7.45 (m, 2H), 7.38 – 7.13 (m, 2H), 3.95 (s, 2H).

^{13}C NMR (126 MHz, Chloroform-*d*) δ 167.4, 161.9, 135.0, 132.1, 131.1, 130.6, 128.9, 124.2, 122.1, 37.3.

Data consistent with the literature.[42]

1,3-Dioxoisoindolin-2-yl (*E*)-but-2-enoate, **N40**

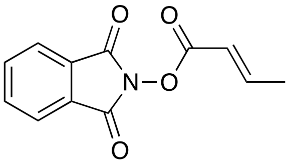

Prepared according to General Procedure 1A using 3-butenoic acid (411 μL, 5.00 mmol, 1.0 equiv.), *N*,*N'*-diisopropyl-carbodiimide (852 μL, 5.50 mmol, 1.1 equiv.), 4-dimethylaminopyridine (61 mg, 500 μmol, 10 mol%), and *N*-hydroxyphthalimide (816 mg, 5.00 mmol, 1.0 equiv.) in DCM (50 mL, 0.1 M). The crude residue was purified by flash chromatography (silica gel) from pure hexane to a mixture of 6% of ethyl acetate in hexane affording 468 mg of a white solid consistent with the desired product (40%).

^{1}H NMR (500 MHz, Chloroform-*d*) δ 7.93 – 7.85 (m, 2H), 7.82 – 7.75 (m, 2H), 7.33 (dq, *J* = 15.69, 6.93 Hz, 1H), 6.11 (dq, *J* = 15.63, 1.72 Hz, 1H), 2.02 (dd, *J* = 6.95, 1.73 Hz, 3H).

^{13}C NMR (126 MHz, Chloroform-*d*) δ 162.3, 162.3, 151.4, 134.9, 129.1, 124.1, 117.2, 18.9.

IR (film): 1798, 1774, 1728, 1651, 1470, 1367, 1358, 1313, 1292, 1184, 1172, 1132, 1088, 1084, 972, 878, 786, 704, 519 cm^{-1}.

HRMS (ESI): *m/z* calculated for $[M + H]^+$ $(C_{12}H_{10}NO_4)^+$: 232.0604; found 232.0596.

1,3-Dioxoisoindolin-2-yl cinnamate, **N41**

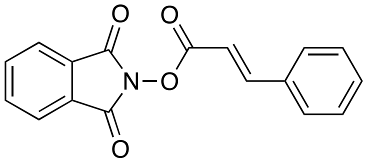

Prepared according to General Procedure 1A using (*E*)-cinnamic acid (741 mg, 5.00 mmol, 1.0 equiv.), *N*,*N'*-diisopropyl-carbodiimide (852 μL, 5.50 mmol, 1.1 equiv.), 4-dimethylaminopyridine (61 mg, 500 μmol, 10 mol%), and *N*-hydroxyphthalimide (816 mg, 5.00 mmol, 1.0 equiv.) in DCM (50 mL, 0.1 M). The crude residue was purified by flash chromatography (silica gel) from pure hexane to a mixture of 10% of ethyl acetate in hexane affording 685 mg of a white solid consistent with the desired product (47%).

^{1}H NMR (500 MHz, Chloroform-*d*) δ 7.97 (d, *J* = 16.05 Hz, 1H), 7.93 – 7.89 (m, 2H), 7.85 – 7.77 (m, 2H), 7.63 – 7.54 (m, 2H), 7.50 – 7.41 (m, 3H), 6.66 (d, *J* = 16.04 Hz, 1H).

^{13}C NMR (126 MHz, Chloroform-*d*) δ 163.2, 162.2, 150.1, 134.9, 133.7, 131.7, 129.3, 129.1, 128.8, 124.1, 111.8.

Data are consistent with the literature.[13]

1,3-Dioxoisoindolin-2-yl cyclohex-1-ene-1-carboxylate, **N42**

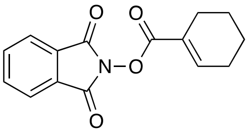

Prepared according to General Procedure 1A using 1-cyclohexene-1-carboxylic acid (810 µL, 5.00 mmol, 1.0 equiv.), *N,N'*-diisopropyl-carbodiimide (852 µL, 5.50 mmol, 1.1 equiv.), 4-dimethylaminopyridine (61 mg, 500 µmol, 10 mol%), and *N*-hydroxyphthalimide (816 mg, 5.00 mmol, 1.0 equiv.) in DCM (50 mL, 0.1 M). The crude residue was purified by flash chromatography (silica gel) from pure hexane to a mixture of 7% of ethyl acetate in hexane affording 954 mg of a white solid consistent with the desired product (70%).

^{1}H NMR (400 MHz, Chloroform-*d*) δ 7.93 – 7.84 (m, 2H), 7.82 – 7.73 (m, 2H), 7.39 (tt, *J* = 3.88, 1.73 Hz, 1H), 2.42 – 2.35 (m, 2H), 2.34 – 2.26 (m, 2H), 1.77 – 1.63 (m, 4H).

^{13}C NMR (126 MHz, Chloroform-*d*) δ 163.1, 162.5, 146.0, 134.8, 129.2, 126.3, 124.0, 26.4, 24.1, 21.8, 21.1.

IR (film): 1764, 1744, 1636, 1464, 1356, 1182, 1151, 1134, 1120, 989, 970, 877, 810, 696, 518 cm^{-1}.

HRMS (ESI): *m/z* calculated for $[M+Na]^+$ $(C_{15}H_{13}NO_4Na)^+$: 294.0742; found = 294.0727.

1,3-Dioxoisoindolin-2-yl tetrahydrofuran-3-carboxylate, **N43**

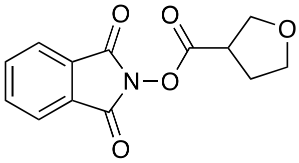

Prepared according to General Procedure 1B using tetrahydro-3-furoic acid (383 µL, 4.00 mmol, 1 equiv.), EDC hydrochloride (920 mg, 4.80 mmol, 1.2 equiv.), 4-dimethylaminopyridine (49 mg, 400 µmol, 10 mol%), and *N*-hydroxyphthalimide (653 mg, 4.00 mmol, 1 equiv.) in DCM (40 mL, 0.1 M). The crude residue was purified by flash chromatography (silica gel) from pure hexane to a mixture of 25% of ethyl acetate in hexane affording 612 mg of a white solid consistent with the desired product (59%).

^{1}H NMR (500 MHz, Chloroform-*d*) δ 7.91 – 7.87 (m, 2H), 7.82 – 7.77 (m, 2H), 4.17 – 4.10 (m, 2H), 3.99 – 3.86 (m, 2H), 3.45 (ddt, *J* = 9.38, 7.89, 5.97 Hz, 1H), 2.43 – 2.29 (m, 2H).

^{13}C NMR (126 MHz, Chloroform-*d*) δ 170.5, 161.9, 135.0, 129.0, 124.1, 70.1, 68.4, 41.0, 30.0.

IR (solid): 1805, 1782, 1735, 1649, 1463, 1288, 1186, 1172, 1138, 1062, 970 cm^{-1}.

HRMS (ESI): *m/z* calculated for $[M + H^+]$ $(C_{13}H_{12}NO_5)^+$: 262.0710, found 262.0715.

1,3-Dioxoisoindolin-2-yl tetrahydrofuran-2-carboxylate, **N44**

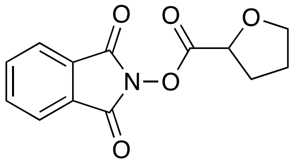

Prepared according to General Procedure 1B using tetrahydro-2-furoic acid (384 µL, 4.00 mmol, 1 equiv.), EDC hydrochloride (920 mg, 4.80 mmol, 1.2 equiv.), 4-dimethylaminopyridine (49 mg, 400 µmol, 10 mol%), and *N*-hydroxyphthalimide (653 mg, 4.00 mmol, 1 equiv.) in DCM (40 mL, 0.1 M). The crude residue was

purified by flash chromatography (silica gel) from pure hexane to a mixture of 20% of ethyl acetate in hexane affording 563 mg of a white solid consistent with the desired product (69%).

^{1}H NMR (500 MHz, Chloroform-*d*) δ 7.92 – 7.84 (m, 2H), 7.83 – 7.75 (m, 2H), 4.86 (dd, *J* = 8.55, 4.94 Hz, 1H), 4.09 (ddd, *J* = 8.29, 7.33, 6.38 Hz, 1H), 4.00 (ddd, *J* = 8.26, 7.22, 6.13 Hz, 1H), 2.50 – 2.33 (m, 2H), 2.15 – 1.97 (m, 2H).

^{13}C NMR (126 MHz, Chloroform-*d*) δ 169.9, 161.8, 134.9, 129.0, 124.1, 75.1, 70.0, 31.0, 25.2.

Data are consistent with the literature.[9]

1,3-Dioxoisoindolin-2-yl tetrahydro-2*H*-pyran-2-carboxylate, **N45**

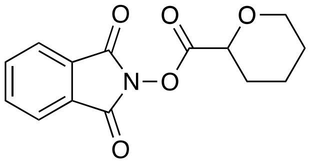

Prepared according to General Procedure 1B using tetrahydro-2H-pyran-2-carboxylic acid (429 μL, 4.00 mmol, 1 equiv.), EDC hydrochloride (920 mg, 4.80 mmol, 1.2 equiv.), 4-dimethylaminopyridine (49 mg, 400 μmol, 10 mol%), and *N*-hydroxyphthalimide (653 mg, 4.00 mmol, 1 equiv.) in DCM (40 mL, 0.1 M). The crude residue was purified by flash chromatography (silica gel) from pure hexane to a mixture of 20% of ethyl acetate in hexane affording 456 mg of a white solid consistent with the desired product (41%).

^{1}H NMR (400 MHz, Chloroform-*d*) δ 7.91 – 7.85 (m, 2H), 7.81 – 7.76 (m, 2H), 4.48 (dd, *J* = 9.49, 3.22 Hz, 1H), 4.13 (dt, *J* = 11.73, 3.75 Hz, 1H), 3.65 – 3.56 (m, 1H), 2.18 – 2.08 (m, 1H), 1.99 – 1.88 (m, 2H), 1.76 – 1.63 (m, 2H), 1.63 – 1.58 (m, 1H).

^{13}C NMR (101 MHz, Chloroform-*d*) δ 168.1, 161.7, 134.9, 129.0, 124.1, 74.3, 68.1, 29.0, 25.2, 22.4.

IR (solid): 1816, 1786, 1734, 1463, 1355, 1213, 1138, 1120, 1093, 1082, 1006, 968 cm^{-1}.

HRMS (ESI): *m/z* calculated for [M + H^{+}] ($C_{14}H_{14}NO_5$)$^{+}$: 276.0866, found 276.0867.

1,3-Dioxoisoindolin-2-yl 2,2-dimethylbut-3-enoate, **N46**

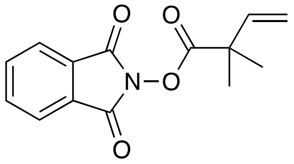

Prepared according to General Procedure 1B using 2,2-dimethylbut-3-enoic acid (429 μL, 4.00 mmol, 1 equiv.), EDC hydrochloride (920 mg, 4.80 mmol, 1.2 equiv.), 4-dimethylaminopyridine (49 mg, 400 μmol, 10 mol%), and *N*-hydroxyphthalimide (653 mg, 4.00 mmol, 1 equiv.) in DCM (40 mL, 0.1 M). The crude residue was purified by flash chromatography (silica gel) from pure hexane to a mixture of 6% of diethyl ether in hexane affording 826 mg of a white solid consistent with the desired product (80%).

^{1}H NMR (500 MHz, Chloroform-*d*) δ 7.90 – 7.85 (m, 2H), 7.80 – 7.76 (m, 2H), 6.13 (dd, *J* = 17.36, 10.63 Hz, 1H), 5.34 (d, *J* = 17.40 Hz, 1H), 5.25 (d, *J* = 10.65 Hz, 1H), 1.52 (s, 6H).

^{13}C NMR (126 MHz, Chloroform-*d*) δ 172.6, 162.1, 140.3, 134.8, 129.1, 124.0, 115.0, 44.6, 24.9.

IR (solid): 1809, 1780, 1639, 1608, 1589, 1465, 1363, 1184, 1170, 1136, 1053, 1037, 1016, 995 cm^{-1}.

HRMS (ESI): *m/z* calculated for [M + Na$^+$] ($C_{14}H_{13}NO_4Na$)$^+$: 282.0737, found 282.0732.

6.2 Boronic acid starting materials

4-((Trimethylsilyl)ethynyl)benzonitrile, **S1-int1**

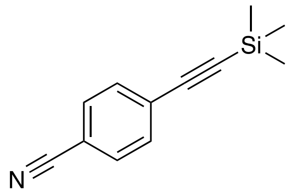

Prepared according to General Procedure 2 using 4-iodobenzonitrile (1.15 g, 5.00 mmol, 1.0 equiv.), dichlorobis(triphenylphosphine)palladium (35 mg, 50.0 µmol, 1 mol%), copper iodide (19 mg, 100 µmol, 2 mol%), and trimethylsilylacetylene (660 µL, 6.25 mmol, 1.25 equiv.) in triethylamine (10 mL, 0.5 M). The crude residue was purified by flash chromatography (silica gel) from pure hexane to a mixture of 3% of ethyl acetate/ in hexane affording 932 mg of a white solid as the desired product (94%).

^{1}H NMR (500 MHz, Chloroform-*d*) δ 7.61 – 7.57 (m, 2H), 7.56 – 7.51 (m, 2H), 0.26 (s, 9H).

^{13}C NMR (126 MHz, Chloroform-*d*) δ 132.6, 132.1, 128.1, 118.6, 111.9, 103.1, 99.7, –0.1.

Data are consistent with the literature.[43]

4-Ethynylbenzonitrile, **S1-int2**

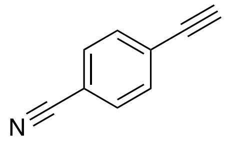

Prepared according to General Procedure 3 using 4-(trimethylsilyl)ethynyl)benzonitrile, **S1-int1** (932 mg, 4.68 mmol, 1.0 equiv.) and potassium carbonate (1.29 g, 9.35 mmol, 2.0 equiv.) in MeOH (23 mL, 0.2 M). The crude residue was purified by flash chromatography (silica gel) from pure hexane to a mixture of 10% of diethyl ether in hexane affording 478 mg of a white solid consistent with the desired product (80%).

^{1}H NMR (500 MHz, Chloroform-*d*) δ 7.63 – 7.60 (m, 2H), 7.59 – 7.55 (m, 2H), 3.30 (s, 1H).

^{13}C NMR (126 MHz, Chloroform-*d*) δ 132.8, 132.2, 127.1, 118.4, 112.4, 82.0, 81.7.

Data are consistent with the literature.[44]

(*E*)-4-(2-(4,4,5,5-Tetramethyl-1,3,2-dioxaborolan-2-yl)vinyl)benzonitrile, **S1-int3**

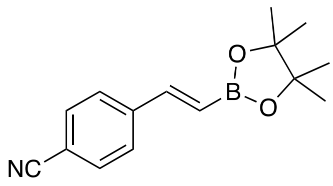

Prepared according to General Procedure 4 using 4-ethynylbenzonitrile, **S1-int2** (191 mg, 1.50 mmol, 1.0 equiv.), copper(I) chloride (7 mg, 75 μmol, 5 mol%), potassium *tert*-butoxide (17 mg, 150 μmol, 10 mol%), bis(2-diphenylphosphinophenyl)ether (DPEPhos) (40 mg, 75 μmol, 5 mol%), bis(pinacolato)diboron (420 mg, 1.65 mmol, 1.1 equiv.), and MeOH (162 μL, 4.00 mmol, 2.0 equiv.) in THF (6 mL, 0.25 M). The crude residue was purified by flash chromatography (silica gel) from pure hexane to a mixture of 10% of diethyl ether in hexane affording 157 mg of a white solid consistent with the desired product (41%).

^{1}H NMR (500 MHz, Chloroform-*d*) δ 7.65 – 7.61 (m, 2H), 7.56 – 7.53 (m, 2H), 7.36 (d, J = 18.45 Hz, 1H), 6.28 (d, J = 18.45 Hz, 1H), 1.32 (s, 12H).

^{11}B NMR (96 MHz, Chloroform-*d*) δ 30.42.

^{13}C NMR (126 MHz, Chloroform-*d*) δ 147.3, 141.8, 132.6, 127.6, 121.1 (broad), 119.0, 112.1, 83.9, 24.9.

Data are consistent with literature.[45]

(*E*)-4-(2-(Trifluoro-λ^4-boraneyl)vinyl)benzonitrile, potassium salt, **S1-int4**

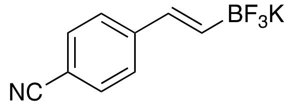

Prepared according to General Procedure 5 using (*E*)-4-(2-(4,4,5,5-tetramethyl-1,3,2-dioxaborolan-2-yl)vinyl)benzonitrile, **S1-int3** (250 mg, 1.09 mmol, 1.0 equiv.), potassium hydrogen fluoride (192 mg, 2.46 mmol, 4.0 equiv.), and water (554 μL, 30.8 mmol, 50.0 equiv.) in MeOH (6 mL, 0.1 M). 74 mg of a white solid was obtained after filtration, consistent with the desired product (51%).

^{1}H NMR (500 MHz, Acetone-d_6) δ 7.64 – 7.59 (m, 2H), 7.53 – 7.49 (m, 2H), 6.68 (d, J = 18.27 Hz, 1H), 6.57 (dq, J = 18.17, 3.36 Hz, 1H).

^{11}B NMR (96 MHz, Acetone-d_6) δ 2.74.

^{13}C NMR (126 MHz, Acetone-d_6) δ 146.6, 132.9, 132.8 (q, $^{3}J_{CF}$ = 4.1 Hz), 127.1, 119.9, 109.4. The boron-bearing carbon is not observed due to quadrupolar relaxation.

^{19}F {^{1}H} NMR (376 MHz, Acetone-d_6) δ –142.75.

Data are consistent with the literature.[46]

(*E*)-(4-Cyanostyryl)boronic acid, **S1**

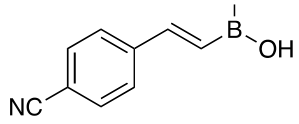

Prepared according to General Procedure 6 using (*E*)-4-(2-(trifluoro-λ^4-boraneyl)vinyl)benzonitrile, potassium salt, **S1-int4** (432 mg, 1.84 mmol, 1.0 equiv.) and chlorotrimethylsilane (816 µL, 6.43 mmol, 3.5 equiv.) in MeCN:H_2O (14 mL:3 mL, 0.1 M). 233 mg of a white solid was obtained as the desired product (73%).

^{1}H NMR (500 MHz, DMSO-*d*$_6$) δ 7.96 (s, 2H), 7.84 – 7.79 (m, 2H), 7.68 – 7.63 (m, 2H), 7.29 (d, *J* = 18.39 Hz, 1H), 6.30 (d, *J* = 18.39 Hz, 1H).

^{11}B NMR (128 MHz, DMSO-*d*$_6$) δ 30.67.

^{13}C NMR (126 MHz, DMSO-*d*$_6$) δ 143.9, 142.1, 132.7, 127.8 (broad), 127.4, 118.9, 110.5.

IR (solid): 1604, 1340, 1323, 1290, 1269, 1230, 1095, 1074, 1039, 997, 987, 958, 931 cm^{-1}.

HRMS (ESI): *m/z* calculated for [M (methyl boronic ester) + Na]$^+$ ($C_{11}H_{12}BNNaO_2$)$^+$: 224.0853; found = 224.0857.

(*E*)-2-(3-Methoxystyryl)-4,4,5,5-tetramethyl-1,3,2-dioxaborolane, **S2-int3**

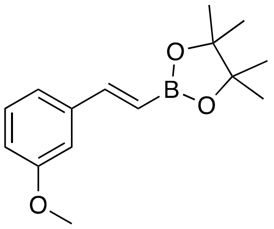

Prepared according to General Procedure 4 using 3-methoxyphenylacetylene (635 µL, 5.00 mmol, 1.0 equiv.), copper(I) chloride (25 mg, 250 µmol, 5 mol%), potassium *tert*-butoxide (56 mg, 500 µmol, 10 mol%), bis(2-diphenylphosphinophenyl) ether (DPEPhos) (135 mg, 250 µmol, 5 mol%), bis(pinacolato)diboron (1.40 g, 5.50 mmol, 1.1 equiv.), and MeOH (405 µL, 10.0 mmol, 2.0 equiv.) in THF (20 mL, 0.25 M). The crude residue was purified by flash chromatography (silica gel) from pure hexane to a mixture of 5% of diethyl ether in hexane affording 1.28 g of a colourless oil consistent with the desired product (98%).

^{1}H NMR (500 MHz, Chloroform-*d*) δ 7.37 (d, *J* = 18.39 Hz, 1H), 7.25 (t, *J* = 7.89 Hz, 1H), 7.08 (dt, *J* = 7.62, 1.26 Hz, 1H), 7.03 (dd, *J* = 2.57, 1.57 Hz, 1H), 6.86 – 6.83 (m, 1H), 6.16 (d, *J* = 18.40 Hz, 1H), 3.80 (s, 3H), 1.31 (s, 12H).

^{13}C NMR (126 MHz, Chloroform-*d*) δ 159.9, 149.5, 139.0, 129.7, 119.9, 116.9 (broad), 114.9, 112.0, 83.5, 55.3, 24.9.

^{11}B NMR (96 MHz, Chloroform-*d*) δ 30.49.

Data consistent with the literature.[47]

(*E*)-Trifluoro(3-methoxystyryl)-λ^4-borane, potassium salt, **S2-int4**

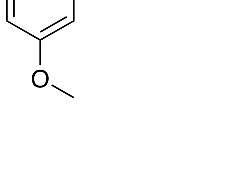

Prepared according to General Procedure 5 using (*E*)-2-(3-methoxystyryl)-4,4,5,5-tetramethyl-1,3,2-dioxaborolane, **S2-int3** (1.25 g, 4.81 mmol, 1.0 equiv.), potassium hydrogen fluoride (1.50 g, 19.2 mmol, 4.0 equiv.), and water (4.33 mL, 240 mmol, 50.0 equiv.) in MeOH (40 mL, 0.1 M). 827 mg of a white solid was obtained after filtration, consistent with the desired product (72%).

^{1}H NMR (500 MHz, DMSO-d_6) δ 7.15 (t, J = 7.85 Hz, 1H), 6.89 (dt, J = 7.58, 1.22 Hz, 1H), 6.86 (dd, J = 2.65, 1.53 Hz, 1H), 6.68 (ddd, J = 8.17, 2.59, 0.96 Hz, 1H), 6.44 (d, J = 18.27, 1H), 6.18 (dq, J = 18.19, 3.54 Hz, 1H), 3.74 (s, 3H).

^{11}B NMR (96 MHz, DMSO-d_6) δ 2.90.

^{13}C NMR (126 MHz, DMSO-d_6) δ 159.4, 141.9, 139.5 (broad), 133.1 (q, $^{3}J_{CF}$ = 4.4 Hz), 129.2, 118.1, 111.6, 110.4, 54.8.

^{19}F {^{1}H} NMR (377 MHz, DMSO-d_6) δ –137.84.

IR (solid): 1625, 1600, 1577, 1487, 1463, 1429, 1288, 1265, 1149, 1138, 1232, 1093, 1037, 989 cm^{-1}.

HRMS (ESI): *m/z* calculated for $[M – K]^{-}$ $(C_9H_9BF_3O)^{-}$: 201.0705; found = 201.0691.

(*E*)-(3-Methoxystyryl)boronic acid, **S2**

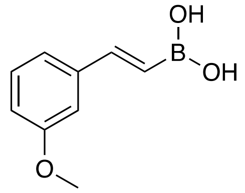

Prepared according to General Procedure 6 using (*E*)-trifluoro(3-methoxystyryl)-λ^{4}-borane, potassium salt, **S2-int4** (770 mg, 3.21 mmol, 1.0 equiv.) and chlorotrimethylsilane (1.42 mL, 11.2 mmol, 3.5 equiv.) in MeCN:H_2O (26 mL:6 mL, 0.1 M). 489 mg of a white solid was obtained as a mixture of the desired product and boroxine (86%).

^{1}H NMR (500 MHz, DMSO-d_6) δ 7.81 (s, 2H), 7.28 (t, J = 7.89 Hz, 1H), 7.23 (d, J = 18.35 Hz, 1H), 7.05 (d, J = 7.61 Hz, 1H), 7.01 (t, J = 1.98 Hz, 1H), 6.87 (dd, J = 8.18, 2.56 Hz, 1H), 6.13 (d, J = 18.35 Hz, 1H), 3.77 (s, 3H).

^{11}B NMR (96 MHz, DMSO-d_6) δ 29.49.

^{13}C NMR (126 MHz, DMSO-d_6) δ 159.6, 145.8, 139.2, 129.8, 123.6 (broad), 119.2, 114.3, 111.6, 55.1.

IR (solid): 1625, 1593, 1583, 1490, 1350, 1313, 1286, 1249, 1234, 1213, 1153, 989 cm^{-1}.

HRMS (ESI): *m/z* calculated for $[M + H]^{+}$ $(C_9H_{12}BO_3)^{+}$: 179.0874; found = 179.0871.

N-(3-iodophenyl)acetamide, **S3-int0**

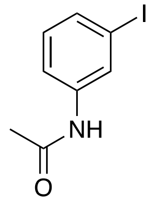

3-Iodoaniline (602 μL, 5.00 mmol, 1.0 equiv.) was dissolved in DCM (25 mL, 0.2 M). Triethylamine (1.39 mL, 10.0 mmol, 2.0 equiv.) was added and the reaction mixture was cooled down to 0 °C. Acetic anhydride (1.42 mL, 15.0 mmol, 3.0 equiv.) was added dropwise at 0 °C and the reaction mixture was left to

stir for six hours. Once completion was reached, the reaction mixture was partitioned between DCM (10 mL) and a saturated solution of sodium bicarbonate (20 mL). Organics were extracted with DCM (2 × 15 mL). Organic layers were combined washed with brine (20 mL), dried over sodium sulfate, filtered, and concentrated *in vacuo*. The crude residue was purified by flash chromatography (silica gel) from pure hexane to a mixture of 35% of ethyl acetate in hexane affording 1.27 g of a white solid as the desired product (97%).

^{1}H NMR (500 MHz, Chloroform-*d*) δ 7.91 (t, *J* = 1.88 Hz, 1H), 7.80 (s, 1H), 7.49 – 7.43 (m, 1H), 7.42 (dt, *J* = 7.90, 1.28 Hz, 1H), 7.01 (t, *J* = 8.01 Hz, 1H), 2.16 (s, 3H).

^{13}C NMR (126 MHz, Chloroform-*d*) δ 168.9, 139.2, 133.4, 130.6, 128.8, 119.3, 94.2, 24.7.

Data are consistent with the literature.[48]

N-(3-((Trimethylsilyl)ethynyl)phenyl)acetamide, **S3-int1**

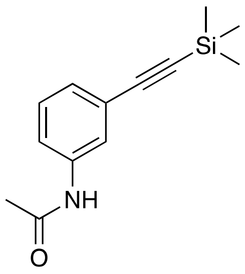

Prepared according to General Procedure 2 using *N*-(3-iodophenyl)acetamide, **S3-int0** (1.47 g, 5.63 mmol, 1.0 equiv.), dichlorobis(triphenylphosphine)palladium (39 mg, 56.3 µmol, 1 mol%), copper iodide (21 mg, 113 µmol, 2 mol%), and trimethylsilylacetylene (743 µL, 7.04 mmol, 1.25 equiv.) in triethylamine (12 mL, 0.5 M). The crude residue was purified by flash chromatography (silica gel) from pure hexane to a mixture of 45% of ethyl acetate in hexane affording 1.21 g of a pale-brown solid as the desired product (93%).

^{1}H NMR (500 MHz, Chloroform-*d*) δ 7.61 (s, 1H), 7.51 – 7.45 (m, 2H), 7.25 – 7.17 (m, 2H), 2.16 (s, 3H), 0.23 (s, 9H).

^{13}C NMR (126 MHz, Chloroform-*d*) δ 168.6, 137.9, 129.0, 128.0, 123.9, 123.2, 120.2, 104.6, 94.7, 24.7, 0.0.

IR (film): 2156, 1666, 1606, 1383, 1550, 1483, 1421, 1404, 1371, 1247, 839, 758 cm^{-1}.

HRMS (ESI): *m/z* calculated for $[M + H]^+$ $(C_{13}H_{18}NOSi)^+$: 232.1152; found = 232.1150.

N-(3-Ethynylphenyl)acetamide, **S3-int2**

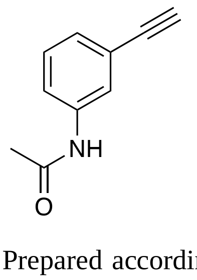

Prepared according to General Procedure 3 using *N*-(3-((trimethylsilyl)ethynyl)phenyl)acetamide, **S3-int1** (1.21 g, 5.23 mmol, 1.0 equiv.) and potassium carbonate (1.45 g, 10.5 mmol, 2.0 equiv.) in MeOH (26 mL, 0.2 M). The crude residue was purified by flash chromatography (silica gel) from pure hexane to a mixture

of 35% of ethyl acetate in hexane affording 696 mg of a white solid consistent with the desired product (84%).

^{1}H NMR (500 MHz, Chloroform-*d*) δ 7.68 – 7.59 (m, 2H), 7.55 – 7.51 (m, 1H), 7.27 – 7.24 (m, 1H), 7.24 – 7.20 (m, 1H), 3.06 (s, 1H), 2.17 (s, 3H).

^{13}C NMR (126 MHz, Chloroform-*d*) δ 168.8, 138.1, 129.1, 128.1, 123.4, 122.9, 120.6, 83.3, 77.6, 24.7.

Data are consistent with the literature.[49]

(*E*)-*N*-(3-(2-(4,4,5,5-Tetramethyl-1,3,2-dioxaborolan-2-yl)vinyl)phenyl)acetamide, **S3-int3**

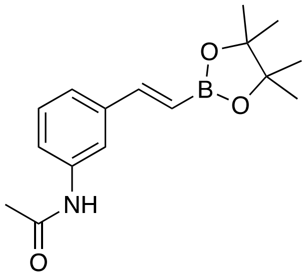

Prepared according to General Procedure 4 using *N*-(3-ethynylphenyl)acetamide, **S3-int2** (637 mg, 4.00 mmol, 1.0 equiv.), copper(I) chloride (20 mg, 200 μmol, 5 mol%), potassium *tert*-butoxide (45 mg, 400 μmol, 10 mol%), bis(2-diphenylphosphinophenyl) ether (DPEPhos) (108 mg, 200 umol, 5 mol%), bis(pinacolato)diboron (1.12 g, 4.40 mmol, 1.1 equiv.), and MeOH (324 μL, 8.00 mmol, 2.0 equiv.) in THF (16 mL, 0.25M). The crude residue was purified by flash chromatography (silica gel) from pure hexane to a mixture of 35% of ethyl acetate in hexane affording 819 mg of white solid consistent with the desired product (71%).

^{1}H NMR (500 MHz, Chloroform-*d*) δ 8.20 (s, 1H), 7.58 (t, J = 1.92 Hz, 1H), 7.50 – 7.46 (m, 1H), 7.31 (d, J = 18.41 Hz, 1H), 7.25 – 7.18 (m, 2H), 6.10 (d, J = 18.41 Hz, 1H), 2.11 (s, 3H), 1.28 (s, 12H).

^{11}B NMR (96 MHz, Chloroform-*d*) δ 31.95.

^{13}C NMR (126 MHz, Chloroform-*d*) δ 169.1, 149.2, 138.4, 138.2, 129.2, 122.9, 120.7, 118.7, 117.0 (broad), 83.4, 24.8, 24.5.

IR (film): 2994, 1666, 1626, 1587, 1483, 1431, 1379, 1372, 1346, 1321, 1248, 1139, 968, 729 cm^{-1}.

HRMS (ESI): *m/z* calculated for $[M + H]^+$ $(C_{16}H_{23}BNO_3)^+$: 288.1765; found = 288.1760.

(*E*)-*N*-(3-(2-(Trifluoro-λ^4-boraneyl)vinyl)phenyl)acetamide, potassium salt, **S3-int4**

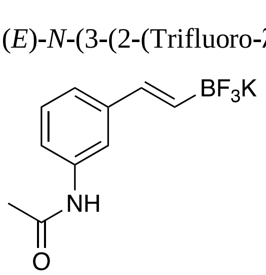

Prepared according to General Procedure 5 using (*E*)-*N*-(3-(2-(4,4,5,5-tetramethyl-1,3,2-dioxaborolan-2-yl)vinyl)phenyl)acetamide, **S3-int3** (800 mg, 2.79 mmol, 1.0 equiv.), potassium hydrogen fluoride (870 mg,

11.1 mmol, 4.0 equiv.), and water (2.51 mL, 139 mmol, 50.0 equiv.) in MeOH (26 mL, 0.1 M). 502 mg of a white solid was obtained after filtration, consistent with the desired product (67%).

^{1}H NMR (500 MHz, DMSO-d_6) δ 9.82 (s, 1H), 7.49 (t, J = 1.90 Hz, 1H), 7.41 – 7.38 (m, 1H), 7.15 (t, J = 7.82 Hz, 1H), 6.97 (dt, J = 7.79, 1.38 Hz, 1H), 6.41 (d, J = 18.14 Hz, 1H), 6.14 (dq, J = 18.19, 3.55 Hz, 1H), 2.03 (s, 3H).

^{11}B NMR (128 MHz, DMSO-d_6) δ 2.62.

^{13}C NMR (126 MHz, DMSO-d_6) δ 168.2, 140.8, 139.4, 138.9 (broad), 133.1 (q, $^{3}J_{CF}$ = 4.4 Hz), 128.5, 120.5, 116.8, 116.0, 24.1.

^{19}F {^{1}H} NMR (377 MHz, DMSO-d_6) δ –137.87.

IR (solid): 1676, 1660, 1583, 1541, 1494, 1423, 1406, 1369, 1307, 1296, 1103, 1074, 1010, 993 cm^{-1}.

HRMS (ESI): *m/z* calculated for $[M - K]^-$ $(C_{10}H_{10}BNOF_3)^-$: 228.0813; found = 228.0822.

(*E*)-(3-Acetamidostyryl)boronic acid, **S3**

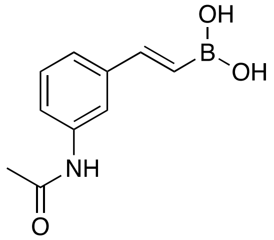

Prepared according to General Procedure 6 using (*E*)-*N*-(3-(2-(trifluoro-λ^4-boraneyl)vinyl) phenyl)acetamide, potassium salt, **S3-int4** (430 mg, 1.61 mmol, 1.0 equiv.) and chlorotrimethylsilane (715 μL, 5.63 mmol, 3.5 equiv.) in MeCN:H_2O (13 mL:3 mL, 0.1 M). 250 mg of a pale-brown solid was obtained (65%) as a mixture of the desired product and protodeboronated product (ratio 2:1).

^{1}H NMR (400 MHz, Acetone-d_6) δ 7.90 (t, J = 1.93 Hz, 1H), 7.54 – 7.51 (m, 1H), 7.33 (d, J = 18.36 Hz, 1H), 7.28 – 7.24 (m, 1H), 7.20 – 7.16 (m, 1H), 6.19 (d, J = 18.34 Hz, 1H), 2.08 (s, 3H). Amide and boronic acid protons are not observed.

^{11}B NMR (128 MHz, Acetone-d_6) δ 28.75.

^{13}C NMR (126 MHz, Acetone-d_6) δ 168.9, 147.5, 140.8, 139.5, 129.8, 122.7, 119.9, 117.9, 24.3. The boron-bearing carbon is not observed due to quadrupolar relaxation.

IR (film): 2972, 1672, 1556, 1487, 1425, 1371, 1323, 1071, 1045, 879 cm^{-1}.

HRMS (MALDI): *m/z* calculated for $[M + H]^+$ $(C_{10}H_{13}BNO_3)^+$: 206.0983; found = 206.0984.

Methyl 3-((trimethylsilyl)ethynyl)benzoate, **S4-int1**

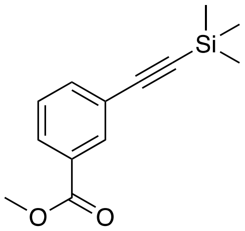

Prepared according to General Procedure 2 using methyl 3-iodobenzoate (1.31 g, 5.00 mmol, 1.0 equiv.), dichlorobis(triphenylphosphine)palladium (35 mg, 50.0 µmol, 1 mol%), copper iodide (19 mg, 100 µmol, 2 mol%), and trimethylsilylacetylene (660 µL, 6.25 mmol, 1.25 equiv.) in triethylamine (10 mL, 0.5 M). The crude residue was purified by flash chromatography (silica gel) from pure hexane to a mixture of 2% of diethyl ether in hexane affording 1.15 g of a pale-yellow solid as the desired product (99%).

^{1}H NMR (500 MHz, Chloroform-*d*) δ 8.15 – 8.12 (m, 1H), 7.97 (dt, *J* = 7.80, 1.52 Hz, 1H), 7.63 (dt, *J* = 7.77, 1.55 Hz, 1H), 7.38 (t, *J* = 7.78 Hz, 1H), 3.92 (s, 3H), 0.26 (s, 9H).

^{13}C NMR (126 MHz, Chloroform-*d*) δ 166.5, 136.2, 133.3, 130.4, 129.6, 128.5, 123.7, 104.0, 95.5, 52.4, 0.0.

Data are consistent with the literature.[50]

Methyl 3-ethynylbenzoate, **S4-int2**

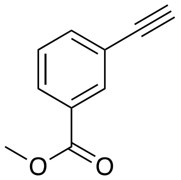

Prepared according to General Procedure 3 using methyl 3-((trimethylsilyl)ethynyl)benzoate, **S4-int1** (1.10 g, 4.73 mmol, 1.0 equiv.) and potassium carbonate (1.31 g, 9.47 mmol, 2.0 equiv.) in MeOH (23 mL, 0.2 M). After work-up 354 mg of a white solid was afforded consistent with the desired product (47%).

^{1}H NMR (500 MHz, Chloroform-*d*) δ 8.16 (t, *J* = 1.76 Hz, 1H), 8.01 (dt, *J* = 7.89, 1.49 Hz, 1H), 7.66 (dt, *J* = 7.69, 1.48 Hz, 1H), 7.40 (t, *J* = 7.77 Hz, 1H), 3.92 (s, 3H), 3.12 (s, 1H).

^{13}C NMR (126 MHz, Chloroform-*d*) δ 166.4, 136.4, 133.4, 130.6, 129.9, 128.6, 122.7, 82.7, 78.3, 52.4.

Data are consistent with the literature.[51]

Methyl (*E*)-3-(2-(4,4,5,5-tetramethyl-1,3,2-dioxaborolan-2-yl)vinyl)benzoate, **S4-int3**

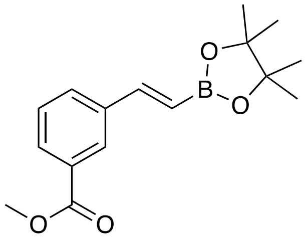

Prepared according to General Procedure 4 using methyl 3-ethynylbenzoate, **S4-int3** (641 mg, 4.00 mmol, 1.0 equiv.), copper(I) chloride (20 mg, 200 µmol, 5 mol%), potassium *tert*-butoxide (45 mg, 400 µmol, 10 mol%), bis(2-diphenylphosphinophenyl)ether (DPEPhos) (108 mg, 200 µmol, 5 mol%), bis(pinacolato)diboron (1.12 g, 4.40 mmol, 1.1 equiv.), and MeOH (324 µL, 8.00 mmol, 2.0 equiv.) in THF (16 mL). The crude residue was purified by flash chromatography (silica gel) from pure hexane to a mixture of 10% of diethyl ether in hexane affording 670 mg of a white solid consistent with the desired product (58%).

^{1}H NMR (500 MHz, Chloroform-*d*) δ 8.14 (t, *J* = 1.78 Hz, 1H), 7.93 (dt, *J* = 7.72, 1.45 Hz, 1H), 7.63 (dt, *J* = 7.76, 1.52 Hz, 1H), 7.43 – 7.35 (m, 2H), 6.22 (d, *J* = 18.48 Hz, 1H), 3.88 (s, 3H), 1.29 (s, 12H).

^{11}B NMR (96 MHz, Chloroform-*d*) δ 30.30.

^{13}C NMR (126 MHz, Chloroform-*d*) δ 166.9, 148.3, 137.9, 131.2, 130.6, 129.8, 128.7, 128.3, 118.0 (broad), 83.5, 52.2, 24.9.

IR (film): 2551, 1722, 1626, 1379, 1345, 1325, 1288, 1265, 1203, 1141, 970, 848, 748 cm^{-1}.

HRMS (ESI): *m/z* calculated for $[M + H]^+$ $(C_{16}H_{22}BO_4)^+$: 289.1605; found = 289.1602.

Methyl (*E*)-3-(2-(trifluoro-λ^4-boraneyl)vinyl)benzoate, potassium salt, **S4-int4**

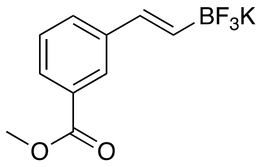

Prepared according to General Procedure 5 using methyl (*E*)-3-(2-(4,4,5,5-tetramethyl-1,3,2-dioxaborolan-2-yl)vinyl)benzoate, **S4-int3** (670 mg, 2.33 mmol, 1.0 equiv.), potassium hydrogen fluoride (726 mg, 9.30 mmol, 4.0 equiv.), and water (2.09 mL, 116 mmol, 50.0 equiv.) in MeOH (22 mL, 0.1 M). 582 mg of a white solid was obtained after filtration, consistent with the desired product (93%).

^{1}H NMR (500 MHz, DMSO-d_6) δ 7.88 (t, *J* = 1.85 Hz, 1H), 7.71 (dt, *J* = 7.71, 1.50 Hz, 1H), 7.60 (dt, *J* = 7.83, 1.45 Hz, 1H), 7.41 (t, *J* = 7.69 Hz, 1H), 6.53 (d, *J* = 18.19 Hz, 1H), 6.28 (dq, *J* = 18.22, 3.51 Hz, 1H), 3.85 (s, 3H).

^{11}B NMR (96 MHz, DMSO-d_6) δ 2.86.

^{13}C NMR (126 MHz, DMSO-d_6) δ 166.5, 140.8, 132.1 (q, $^{3}J_{CF}$ = 4.5 Hz), 130.1, 129.8, 128.8, 126.6, 125.9, 52.1. The boron-bearing carbon is not observed due to quadrupolar relaxation.

^{19}F {^{1}H} NMR (376 MHz, DMSO-d_6) δ –138.11.

Data are consistent with the literature.[52]

(*E*)-(3-(Methoxycarbonyl)styryl)boronic acid, **S4**

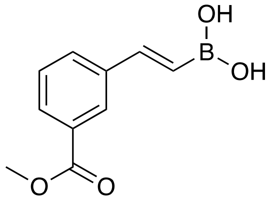

Prepared according to General Procedure 6 using methyl (*E*)-3-(2-(trifluoro-λ^4-boraneyl)vinyl)benzoate, potassium salt, **S4-int4** (500 mg, 1.87 mmol, 1.0 equiv.) and chlorotrimethylsilane (829 µL, 6.53 mmol, 3.5 equiv.) in MeCN:H_2O (15 mL:4 mL, 0.1 M). 251 mg of a white solid was afforded as the desired product (65%) as a mixture of the desired product and boroxine.

^{1}H NMR (500 MHz, DMSO-d_6) δ 8.02 (t, J = 1.82 Hz, 1H), 7.90 (s, 2H), 7.87 (dt, J = 7.80, 1.36 Hz, 1H), 7.74 (dt, J = 7.71, 1.50 Hz, 1H), 7.51 (t, J = 7.72 Hz, 1H), 7.31 (d, J = 18.37 Hz, 1H), 6.22 (d, J = 18.38 Hz, 1H), 3.86 (s, 3H).

^{11}B NMR (128 MHz, DMSO-d_6) δ 26.62.

^{13}C NMR (126 MHz, DMSO-d_6) δ 166.2, 144.6, 138.2, 131.4, 130.2, 129.4, 129.0, 126.9, 125.0 (broad), 52.3.

IR (solid): 1720, 1705, 1624, 1442, 1355, 1348, 1288, 1226, 1170, 1099, 1078, 989, 844, 744 cm^{-1}.

HRMS (ESI): *m/z* calculated for [M (methyl boronic ester) + H]$^+$ ($C_{12}H_{16}BO_4$)$^+$: 235.1136; found = 235.1136.

1-(2-((Trimethylsilyl)ethynyl)phenyl)ethan-1-one, **S5-int1**

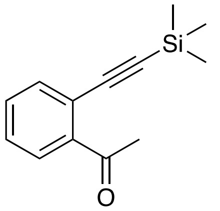

Prepared according to General Procedure 2 using iodoacetophenone (718 µL, 5.00 mmol, 1.0 equiv.), dichlorobis(triphenylphosphine)palladium (35 mg, 50.0 µmol, 1 mol%), copper iodide (19 mg, 100 µmol, 2 mol%), and trimethylsilylacetylene (660 µL, 6.25 mmol, 1.25 equiv.) in triethylamine (10 mL, 0.5 M). The crude residue was purified by flash chromatography (silica gel) from pure hexane to a mixture of 4% of diethyl ether in hexane affording 1.08 g of a yellow oil as the desired product (100%).

^{1}H NMR (500 MHz, Chloroform-d) δ 7.68 (ddd, J = 7.68, 1.54, 0.55 Hz, 1H), 7.56 (ddd, J = 7.61, 1.49, 0.56 Hz, 1H), 7.42 (td, J = 7.52, 1.55 Hz, 1H), 7.38 (td, J = 7.56, 1.49 Hz, 1H), 2.75 (s, 3H), 0.26 (s, 9H).

^{13}C NMR (126 MHz, Chloroform-d) δ 201.0, 141.7, 134.4, 131.3, 128.7, 128.6, 121.5, 104.0, 101.3, 30.3,

–0.2.

Data are consistent with the literature.[53]

1-(2-Ethynylphenyl)ethan-1-one, **S5-int2**

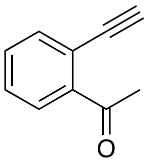

Prepared according to General Procedure 3 using 1-(2-((trimethylsilyl)ethynyl)phenyl)ethan-1-one, **S5-int1** (1.08 g, 5.0 mmol, 1.0 equiv.) and potassium carbonate (1.38 g, 10.0 mmol, 2.0 equiv.) in MeOH (25 mL, 0.2 M). The crude residue was purified by flash chromatography (silica gel) from pure hexane to a mixture of 4% of diethyl ether in hexane affording 276 mg of a pale-yellow oil consistent with the desired product (38%).

^{1}H NMR (500 MHz, Chloroform-d) δ 7.70 (dd, J = 7.63, 1.51 Hz, 1H), 7.60 (dd, J = 7.51, 1.42 Hz, 1H), 7.45 (td, J = 7.69, 1.91 Hz, 1H), 7.41 (td, J = 6.02, 1.46 Hz, 1H), 3.39 (s, 1H), 2.71 (s, 3H).

^{13}C NMR (126 MHz, Chloroform-*d*) δ 200.3, 141.6, 134.9, 131.4, 128.9, 128.6, 120.5, 83.0, 82.6, 30.0.

Data are consistent with the literature.[53]

(*E*) and (*Z*)-1-(2-(2-(4,4,5,5-Tetramethyl-1,3,2-dioxaborolan-2-yl)vinyl)phenyl)ethan-1-one, **S5-int3**

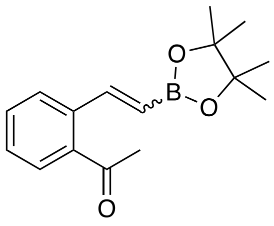

Prepared according to General Procedure 4 using 1-(2-ethynylphenyl)ethan-1-one, **S5-int2** (276 mg, 1.91 mmol, 1.0 equiv.), copper(I) chloride (9 mg, 95.7 µmol, 5 mol%), potassium *tert*-butoxide (21 mg, 191 µmol, 10 mol%), bis(2-diphenylphosphinophenyl)ether (DPEPhos) (52 mg, 95.7 µmol, 5 mol%), bis(pinacolato)diboron (535 mg, 2.11 mmol, 1.1 equiv.), and MeOH (155 µL, 3.83 mmol, 2.0 equiv.) in THF (7 mL, 0.25 M). The crude residue was purified by flash chromatography (silica gel) from pure hexane to a mixture of 10% of diethyl ether in hexane affording 246 mg of a colourless oil consistent with a mixture of *E* and *Z* of the desired product (47%, 0.65:0.35 *Z*:*E*).

(*E*)-1-(2-(2-(4,4,5,5-Tetramethyl-1,3,2-dioxaborolan-2-yl)vinyl)phenyl)ethan-1-one

^{1}H NMR (400 MHz, Chloroform-*d*) δ 7.77 (d, *J* = 18.21 Hz, 1H), 7.67 – 7.59 (m, 2H), 7.49 – 7.43 (m, 1H), 7.39 – 7.34 (m, 1H), 6.06 (d, *J* = 18.21 Hz, 1H), 2.59 (s, 3H), 1.30 (s, 12H).

^{11}B NMR (96 MHz, Chloroform-*d*) δ 30.91.

^{13}C NMR (126 MHz, Chloroform-*d*) δ 202.2, 148.1, 138.4, 137.8, 131.7, 128.5, 128.3, 127.9, 83.5, 30.0, 25.0. The boron-bearing carbon is not observed due to quadrupolar relaxation.

(*Z*)-1-(2-(2-(4,4,5,5-Tetramethyl-1,3,2-dioxaborolan-2-yl)vinyl)phenyl)ethan-1-one

^{1}H NMR (400 MHz, Chloroform-*d*) δ 7.76 – 7.71 (app. d, 1H), 7.67 – 7.59 (m, 1H), 7.43 – 7.40 (m, 2H), 7.39 – 7.34 (m, 1H), 5.68 (d, *J* = 14.51 Hz, 1H), 2.58 (s, 3H), 1.17 (s, 12H).

^{11}B NMR (96 MHz, Chloroform-*d*) δ 30.91.

^{13}C NMR (126 MHz, Chloroform-*d*) δ, 201.0, 149.4, 139.5, 137.0, 131.2, 131.1, 129.1, 127.8, 83.3, 29.6, 24.8. The boron-bearing carbon is not observed due to quadrupolar relaxation.

IR (film): 2978, 1616, 1564, 1477, 1379, 1371, 1348, 1325, 1249, 1141, 968, 758 cm^{-1}.

HRMS (ESI): *m/z* calculated for $[M + H]^+$ $(C_{16}H_{22}BO_3)^+$: 273.1656; found = 273.1665.

(*E*) and (*Z*)-1-(2-(2-(Trifluoro-λ^4-boraneyl)vinyl)phenyl)ethan-1-one, potassium salt, **S5-int4**

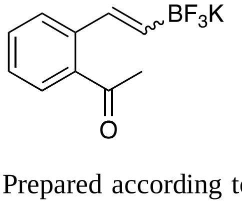

Prepared according to General Procedure 5 using the mixture of (*E*) and (*Z*)-1-(2-(2-(4,4,5,5-tetramethyl-1,3,2-dioxaborolan-2-yl)vinyl)phenyl)ethan-1-one, **S5-int3** (275 mg, 1.01 mmol, 1.0 equiv.), potassium

hydrogen fluoride (316 mg, 4.04 mmol, 4.0 equiv.), and water (910 µL, 50.5 mmol, 50.0 equiv.) in MeOH (10 mL, 0.1 M). 150 mg of a white solid was obtained after filtration, consistent with a mixture of *E* and *Z* of the desired product (59%, 0.68:0.32 *Z:E*).

(*E*)-1-(2-(2-(Trifluoro-λ^4-boraneyl)vinyl)phenyl)ethan-1-one, potassium salt

^{1}H NMR (400 MHz, DMSO-d_6) δ, 7.53 (app. d, *J* = 7.87 Hz, 1H), 7.51 – 7.43 (m, 1H), 7.39 (app. t, *J* = 7.55 Hz, 1H), 7.24 – 7.15 (m, 1H), 6.74 (d, *J* = 18.07 Hz, 1H), 6.10 (dq, *J* = 18.09, 3.53 Hz, 1H), 2.47 (s, 3H).

^{11}B NMR (96 MHz, DMSO-d_6) δ 2.47.

^{13}C NMR (101 MHz, DMSO-d_6) δ 203.7, 138.9, 138.3, 132.9 (q, $^3J_{CF}$ = 4.9 Hz), 130.5, 127.4, 126.0, 125.6, 30.7. The boron-bearing carbon is not observed due to quadrupolar relaxation.

^{19}F {^{1}H} NMR (377 MHz, Chloroform-*d*) δ –133.30.

(*Z*)-1-(2-(2-(Trifluoro-λ^4-boraneyl)vinyl)phenyl)ethan-1-one, potassium salt

^{1}H NMR (400 MHz, DMSO-d_6) δ 7.85 (app. d, *J* = 7.83 Hz, 1H), 7.51 – 7.43 (m, 1H), 7.35 – 7.29 (m, 1H), 7.24 – 7.15 (m, 1H), 6.67 (d, *J* = 14.99 Hz, 1H), 5.61 (dq, *J* = 15.03, 5.90 Hz, 1H), 2.47 (s, 3H).

^{11}B NMR (96 MHz, DMSO-d_6) δ 2.47.

^{13}C NMR (101 MHz, DMSO-d_6) δ 203.1, 139.8, 138.1, 130.6 (q, $^3J_{CF}$ = 4.0 Hz), 130.5, 129.8, 126.9, 125.3, 30.7. The boron-bearing carbon is not observed due to quadrupolar relaxation.

^{19}F {^{1}H} NMR (377 MHz, Chloroform-*d*) δ –127.47.

IR (solid): 1611, 1230, 1159, 1053, 1004, 954, 931 cm^{-1}.

HRMS (ESI): *m/z* calculated for $[M – K]^-$ $(C_{10}H_9BOF_3)^-$: 213.0704; found = 213.0710.

(*E*) and (*Z*)-(2-Acetylstyryl)boronic acid, **S5**

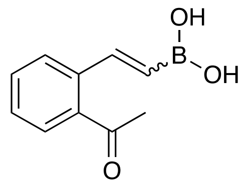

Prepared according to General Procedure 6 using a mixture of (*E*) and (*Z*)-1-(2-(2-(Trifluoro-λ^4-boraneyl)vinyl)phenyl)ethan-1-one, potassium salt, **S5-int4** (463 mg, 1.84 mmol, 1.0 equiv.) and chlorotrimethylsilane (816 µL, 6.43 mmol, 3.5 equiv.) in MeCN:H_2O (15 mL:4 mL, 0.1 M). 86 mg of a pale-yellow solid was obtained as a mixture of *E* and *Z* of the desired product and boroxine (25%, 0.5:0.5 *Z:E*).

(*E*)-(2-acetylstyryl)boronic acid

^{1}H NMR (500 MHz, Acetonitrile-d_3) δ 7.69 (dd, *J* = 7.79, 1.40 Hz, 1H), 7.67 (d, *J* = 7.89 Hz, 1H), 7.62 (d, *J* = 18.17 Hz, 1H), 7.52 – 7.48 (m, 1H), 7.42 – 7.40 (m, 1H), 6.02 (d, *J* = 18.27 Hz, 1H), 5.94 (s, 2H), 2.54 (s, 3H).

^{11}B NMR (128 MHz, Acetonitrile-d_3) δ 29.07.

^{13}C NMR (126 MHz, Acetonitrile-d_3) δ 203.3, 146.7, 139.4, 138.3 (or 138.2), 132.4, 129.5, 129.1, 128.3, 125.7 (broad), 30.3 (or 29.9).

(*Z*)-(2-acetylstyryl)boronic acid

^{1}H NMR (500 MHz, Acetonitrile-d_3) δ 7.79 (dd, *J* = 7.69, 1.41 Hz, 1H), 7.47 (dd, *J* = 7.58, 1.40 Hz, 1H), 7.42 – 7.40 (m, 1H), 7.40 – 7.36 (m, 2H), 5.74 (s, 2H), 5.66 (d, *J* = 14.71 Hz, 1H), 2.54 (s, 3H).

^{11}B NMR (128 MHz, Acetonitrile-d_3) δ 29.07.

^{13}C NMR (126 MHz, Acetonitrile-d_3) δ 202.4, 145.8, 140.0, 138.3 (or 138.2), 132.4, 130.8, 130.1, 128.7, 125.7 (broad), 30.3 (or 29.9).

IR (film): 3414, 1678, 1616, 1595, 1562, 1475, 1355, 1290, 1253, 1089, 1049, 995 cm^{-1}.

HRMS (ESI): *m/z* calculated for $[M + H]^+$ $(C_{10}H_{12}BO_3)^+$: 191.0874; found = 191.0873.

((2-Bromophenyl)ethynyl)trimethylsilane, **S6-int1**

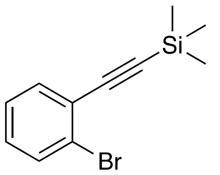

Prepared according to General Procedure 2 using 2-bromoiodobenzene (718 µL, 5.00 mmol, 1.0 equiv.), dichlorobis(triphenylphosphine)palladium (35 mg, 50.0 µmol, 1 mol%), copper iodide (19 mg, 100 µmol, 2 mol%), and trimethylsilylacetylene (660 µL, 6.25 mmol, 1.25 equiv.) in triethylamine (10 mL, 0.5 M). The crude residue was purified by flash chromatography (silica gel) with pure hexane affording 1.20 g of a pale-yellow oil as the desired product (95%).

^{1}H NMR (500 MHz, Chloroform-*d*) δ 7.57 (dd, *J* = 8.09, 1.22 Hz, 1H), 7.49 (dd, *J* = 7.70, 1.72 Hz, 1H), 7.24 (td, *J* = 7.59, 1.27 Hz, 1H), 7.15 (td, *J* = 7.74, 1.73 Hz, 1H), 0.28 (s, 9H).

^{13}C NMR (126 MHz, Chloroform-*d*) δ 133.7, 132.5, 129.7, 127.0, 125.9, 125.4, 103.2, 99.8, 0.0.

Data are consistent with the literature.[54]

1-Bromo-2-ethynylbenzene, **S6-int2**

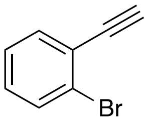

Prepared according to General Procedure 3 using (2-bromophenylethynyl)trimethylsilane, **S6-int1** (1.20 g, 4.74 mmol, 1.0 equiv.) and potassium carbonate (1.31 g, 9.48 mmol, 2.0 equiv.) in MeOH (25 mL, 0.2 M). The crude residue was purified by flash chromatography (silica gel) with pure hexane affording 785 mg of a pale-yellow oil consistent with the desired product (92%).

^{1}H NMR (500 MHz, Chloroform-*d*) δ 7.59 (dd, *J* = 8.03, 1.27 Hz, 1H), 7.53 (dd, *J* = 7.65, 1.75 Hz, 1H), 7.27 (td, *J* = 7.59, 1.29 Hz, 1H), 7.21 (td, *J* = 7.74, 1.76 Hz, 1H), 3.38 (s, 1H).

^{13}C NMR (126 MHz, Chloroform-*d*) δ 134.2, 132.6, 130.1, 127.2, 125.7, 124.4, 82.0, 81.9.

Data are consistent with the literature.[54]

(E)-2-(2-Bromostyryl)-4,4,5,5-tetramethyl-1,3,2-dioxaborolane, **S6-int3**

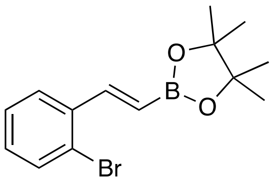

Prepared according to General Procedure 4 using 1-bromo-2-ethynylbenzene, **S6-int2** (780 mg, 4.31 mmol, 1.0 equiv.), copper(I) chloride (21 mg, 215 μmol, 5 mol%), potassium *tert*-butoxide (48 mg, 431 μmol, 10 mol%), bis(2-diphenylphosphinophenyl)ether (DPEPhos) (116 mg, 215 μmol, 5 mol%), bis(pinacolato)diboron (1.20 g, 4.74 mmol, 1.1 equiv.), and MeOH (349 μL, 8.62 mmol, 2.0 equiv.) in THF (18 mL, 0.25 M). The crude residue was purified by flash chromatography (silica gel) from pure hexane to a mixture of 3% of diethyl ether in hexane affording 927 mg of a colourless oil consistent with the desired product (70%).

^{1}H NMR (500 MHz, Chloroform-*d*) δ 7.71 (d, J = 18.23 Hz, 1H), 7.61 (dd, J = 7.83, 1.69 Hz, 1H), 7.55 (dd, J = 8.02, 1.24 Hz, 1H), 7.31 – 7.27 (m, 1H), 7.14 (td, J = 7.75, 1.65 Hz, 1H), 6.13 (d, J = 18.22 Hz, 1H), 1.32 (s, 12H).

^{11}B NMR (96 MHz, Chloroform-*d*) δ 30.07.

^{13}C NMR (126 MHz, Chloroform-*d*) δ 147.7, 137.5, 133.2, 130.0, 127.6, 127.4, 124.4, 120.3 (broad), 83.6, 25.0.

Data are consistent with the literature.[55]

(E)-(2-Bromostyryl)trifluoro-λ^{4}-borane, potassium salt, **S6-int4**

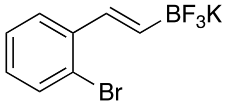

Prepared according to General Procedure 5 using (E)-2-(2-bromostyryl)-4,4,5,5-tetramethyl-1,3,2-dioxaborolane, **S6-int3** (900 mg, 2.91 mmol, 1.0 equiv.), potassium hydrogen fluoride (910 mg, 11.7 mmol, 4.0 equiv.), and water (2.62 mL, 146 mmol, 50.0 equiv.) in MeOH (25 mL, 0.1 M). 579 mg of a white solid was obtained after filtration, consistent with the desired product (69%).

^{1}H NMR (500 MHz, DMSO-d_6) δ 7.56 (dd, J = 7.87, 1.71 Hz, 1H), 7.51 (dd, J = 7.96, 1.28 Hz, 1H), 7.28 (td, J = 6.85, 0.96 Hz, 1H), 7.06 (td, J = 7.66, 1.69 Hz, 1H), 6.76 (d, J = 17.99 Hz, 1H), 6.21 (dq, J = 18.01, 3.53 Hz, 1H).

^{11}B NMR (96 MHz, DMSO-d_6) δ 2.66.

^{13}C NMR (126 MHz, DMSO-d_6) δ 139.3, 132.5, 131.1 (q, $^{3}J_{CF}$ = 4.4 Hz), 127.7, 127.7, 126.4, 122.4. The boron-bearing carbon is not observed due to quadrupolar relaxation.

^{19}F {^{1}H} NMR (377 MHz, DMSO-d_6) δ –138.07.

IR (solid): 1579, 1465, 1433, 1290, 1269, 1236, 1159, 1151, 1136, 1095, 1053, 989, 952 cm^{-1}.

HRMS (ESI): *m/z* calculated for $[M - K]^-$ $(C_8H_6BBrF_3)^-$: 248.9703; found = 248.9714.

(*E*)-(2-Bromostyryl)boronic acid, **S6**

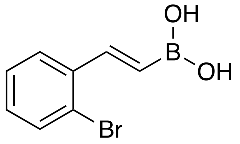

Prepared according to General Procedure 6 using (*E*)-(2-bromostyryl)trifluoro-λ^4-borane, potassium salt, **S6-int4** (500 mg, 1.73 mmol, 1.0 equiv.) and chlorotrimethylsilane (769 µL, 6.06 mmol, 3.5 equiv.) in MeCN:H_2O (14 mL:3 mL, 0.1 M). 360 mg of a white solid was obtained as a mixture of the desired product and boroxine (92%).

^{1}H NMR (500 MHz, DMSO-d_6) δ 7.95 (s, 2H), 7.66 (dd, *J* = 7.88, 1.69 Hz, 1H), 7.62 (dd, *J* = 8.02, 1.22 Hz, 1H), 7.50 (d, *J* = 18.19 Hz, 1H), 7.42 – 7.37 (m, 1H), 7.24 (td, *J* = 7.66, 1.67 Hz, 1H), 6.12 (d, *J* = 18.16 Hz, 1H).

^{11}B NMR (96 MHz, DMSO-d_6) δ 26.85.

^{13}C NMR (126 MHz, DMSO-d_6) δ 143.6, 137.2, 133.0, 130.2, 128.2, 127.3, 123.3. The boron-bearing carbon is not observed due to quadrupolar relaxation.

IR (solid): 1606, 1460, 1436, 1363, 1340, 1321, 1290, 1276, 1267, 1195, 1097, 1047, 966 cm^{-1}.

HRMS (ESI): *m/z* calculated for $[M + Na]^+$ $(C_8H_8BBrO_2Na)^+$: 248.9692; found = 248.9693.

Trimethyl(naphthalen-1-ylethynyl)silane, **S7-int1**

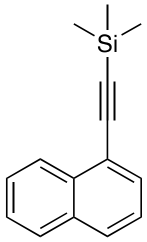

Prepared according to General Procedure 2 using 1-iodonaphthalene (721 µL, 5.00 mmol, 1.0 equiv.), dichlorobis(triphenylphosphine)palladium (35 mg, 50.0 µmol, 1 mol%), copper iodide (19 mg, 100 µmol, 2 mol%), and trimethylsilylacetylene (660 µL, 6.25 mmol, 1.25 equiv.) in triethylamine (10 mL, 0.5 M). The crude residue was purified by flash chromatography (silica gel) with pure hexane affording 1.12 g of a colourless oil as the desired product (100%).

^{1}H NMR (500 MHz, Chloroform-*d*) δ 8.36 (app. d, *J* = 8.32 Hz, 1H), 7.84 (app. t, *J* = 8.70 Hz, 2H), 7.62 – 7.58 (m, 1H), 7.62 – 7.58 (m, 1H), 7.55 – 7.51 (m, 1H), 7.44 – 7.40 (m, 1H), 0.36 (s, 9H).

^{13}C NMR (126 MHz, Chloroform-*d*) δ 133.5, 133.2, 130.9, 129.1, 128.4, 127.0, 126.5, 126.3, 125.3, 120.9, 103.2, 99.6, 0.3.

Data are consistent with the literature.[56]

1-Ethynylnaphthalene, **S7-int2**

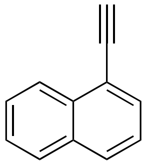

Prepared according to General Procedure 3 using trimethyl(naphthalen-1-ylethynyl)silane, **S7-int1** (1.12 g, 4.99 mmol, 1.0 equiv.) and potassium carbonate (1.38 g, 9.98 mmol, 2.0 equiv.) in MeOH (25 mL, 0.2 M). The crude residue was purified by flash chromatograpghy (silica gel) with pure hexane affording 707 mg of a pale-orange oil consistent with the desired product (64%).

^{1}H NMR (500 MHz, Chloroform-*d*) δ 8.40 (dd, *J* = 8.13, 1.21 Hz, 1H), 7.90 – 7.86 (m, 2H), 7.77 (dd, *J* = 7.08, 1.17 Hz, 1H), 7.61 (ddd, *J* = 8.30, 6.74, 1.35 Hz, 1H), 7.55 (ddd, *J* = 8.21, 6.82, 1.28 Hz, 1H), 7.45 (dd, *J* = 8.27, 7.14 Hz, 1H), 3.50 (s, 1H).

^{13}C NMR (126 MHz, Chloroform-*d*) δ 133.6, 133.2, 131.4, 129.4, 128.4, 127.1, 126.6, 126.2, 125.2, 119.9, 82.1, 81.9.

Data are consistent with the literature.[57]

(*E*)-4,4,5,5-Tetramethyl-2-(2-(naphthalen-1-yl)vinyl)-1,3,2-dioxaborolane, **S7-int3**

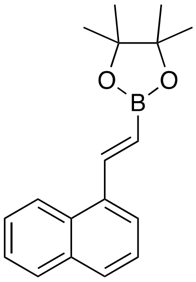

Prepared according to General Procedure 4 using 1-ethynylnaphthalene, **S7-int2** (700 mg, 4.60 mmol, 1.0 equiv.), copper(I) chloride (23 mg, 230 μmol, 5 mol%), potassium *tert*-butoxide (57 mg, 460 μmol, 10 mol%), bis(2-diphenylphosphinophenyl) ether (DPEPhos) (124 mg, 230 μmol, 5 mol%), bis(pinacolato)diboron (1.28 g, 5.06 mmol, 1.1 equiv.), and MeOH (372 μL, 9.20 mmol, 2.0 equiv.) in THF (18 mL, 0.25 M).The crude residue was purified by flash chromatography (silica gel) from pure hexane to a mixture of 4% of diethyl ether in hexane affording 920 mg of a pale-yellow oil consistent with the desired product (71%).

^{1}H NMR (500 MHz, Chloroform-*d*) δ 8.31 – 8.28 (m, 1H), 8.25 (d, *J* = 18.13 Hz, 1H), 7.88 – 7.85 (m, 1H), 7.83 (dd, *J* = 8.19, 1.08 Hz, 1H), 7.77 (dt, *J* = 7.22, 0.93 Hz, 1H), 7.54 (ddd, *J* = 8.44, 6.81, 1.62 Hz, 1H), 7.52 – 7.50 (m, 1H), 7.49 – 7.46 (m, 1H), 6.31 (d, *J* = 18.10 Hz, 1H), 1.37 (s, 12H).

^{11}B NMR (128 MHz, Chloroform-*d*) δ 29.56.

^{13}C NMR (126 MHz, Chloroform-*d*) δ 146.6, 135.4, 133.7, 131.2, 129.1, 128.6, 126.3, 125.9, 125.7, 124.2, 123.9, 120.3 (broad), 83.5, 25.0.

Data are consistent with the literature.[58]

(*E*)-Trifluoro(2-(naphthalen-1-yl)vinyl)-λ^{4}-borane, potassium salt, **S7-int4**

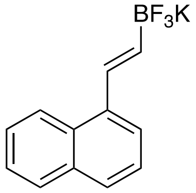

Prepared according to General Procedure 5 using (*E*)-4,4,5,5-tetramethyl-2-(2-(naphthalen-1-yl)vinyl)-1,3,2-dioxaborolane, **S7-int3** (920 mg, 3.28 mmol, 1.0 equiv.), potassium hydrogen fluoride (870 mg, 11.1 mmol, 4.0 equiv.), and water (2.51 mL, 139 mmol, 50.0 equiv.) in MeOH (26 mL, 0.1 M). 710 mg of a white solid was obtained after filtration, consistent with the desired product (98%).

^{1}H NMR (500 MHz, DMSO-d_6) δ 8.18 – 8.14 (m, 1H), 7.89 – 7.85 (m, 1H), 7.72 (d, J = 8.09 Hz, 1H), 7.57 (dt, J = 7.16, 0.93 Hz, 1H), 7.53 – 7.46 (m, 2H), 7.46 – 7.42 (m, 1H), 7.25 (d, J = 17.86 Hz, 1H), 6.25 (dq, J = 17.95, 3.54 Hz, 1H).

^{11}B NMR (128 MHz, DMSO-d_6) δ 2.52.

^{13}C NMR (126 MHz, DMSO-d_6) δ 143.4 (broad), 138.0, 133.3, 130.5, 129.3 (q, $^{3}J_{CF}$ = 4.5 Hz), 128.3, 126.0, 125.9, 125.6, 125.4, 123.6, 122.2.

^{19}F {^{1}H} NMR (377 MHz, DMSO-d_6) δ –137.86.

Data are consistent with the literature.[59]

(*E*)-(2-(Naphthalen-1-yl)vinyl)boronic acid, **S7**

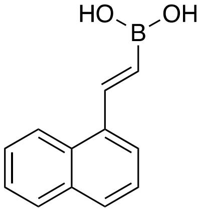

Prepared according to General Procedure 6 using (*E*)-trifluoro(2-(naphthalen-1-yl)vinyl)-λ^4-borane, potassium salt, **S7-int4** (620 mg, 2.38 mmol, 1.0 equiv.) and chlorotrimethylsilane (1.06 mL, 8.34 mmol, 3.5 equiv.) in MeCN:H_2O (20 mL:5 mL, 0.1 M). 326 mg of a white solid was afforded as the desired product (69%) as a mixture of the desired product and boroxine.

^{1}H NMR (500 MHz, DMSO-d_6) δ 8.30 (dd, J = 8.55, 1.29 Hz, 1H), 8.15 (d, J = 18.19 Hz, 1H), 7.96 (s, 2H), 7.94 (dd, J = 7.98, 1.49 Hz, 1H), 7.89 (d, J = 8.14 Hz, 1H), 7.74 (dd, J = 7.28, 1.19 Hz, 1H), 7.61 – 7.56 (m, 1H), 7.56 – 7.50 (m, 2H), 6.24 (d, J = 18.16 Hz, 1H).

^{11}B NMR (96 MHz, DMSO-d_6) δ 27.88.

^{13}C NMR (126 MHz, DMSO-d_6) δ 142.5, 135.1, 133.4, 130.7, 128.5, 126.8 (broad), 126.4, 126.0, 125.8, 123.4, 123.3.

IR (solid): 1604, 1342, 1317, 1280, 1224, 1192, 1101, 985, 786, 694 cm^{-1}.

HRMS (ESI): m/z calculated for $[M + H]^+$ $(C_{12}H_{12}BNO_2)^+$: 199.0924; found = 199.0923.

(*E*)-2-(2-(4,4,5,5-Tetramethyl-1,3,2-dioxaborolan-2-yl)vinyl)pyridine, **S8-int3**

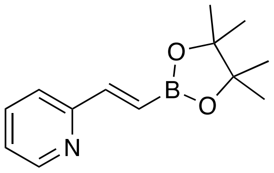

Prepared according to General Procedure 4 using 2-ethynylpyridine (303 µL, 3.00 mmol, 1.0 equiv.), copper(I) chloride (15 mg, 150 µmol, 5 mol%), potassium *tert*-butoxide (34 mg, 300 µmol, 10 mol%), bis(2-diphenylphosphinophenyl)ether (DPEPhos) (81 mg, 150 µmol, 5 mol%), bis(pinacolato)diboron (838 mg, 3.30 mmol, 1.1 equiv.), and MeOH (243 µL, 6.00 mmol, 2.0 equiv.) in THF (12 mL, 0.1 M). The crude residue was purified by flash chromatography (silica gel) from pure hexane to a mixture of 10% ethyl acetate in hexane affording 545 mg of a colourless oil consistent with the desired product (79%).

^{1}H NMR (500 MHz, Chloroform-*d*) δ 8.58 (ddd, *J* = 4.81, 1.88, 0.91 Hz, 1H), 7.63 (td, *J* = 7.65, 1.81 Hz, 1H), 7.44 (d, *J* = 18.28 Hz, 1H), 7.38 (dt, *J* = 7.95, 1.07 Hz, 1H), 7.15 (ddd, *J* = 7.53, 4.76, 1.14 Hz, 1H), 6.61 (d, *J* = 18.28 Hz, 1H), 1.29 (s, 12H).

^{11}B NMR (96 MHz, Chloroform-*d*) δ 30.24.

^{13}C NMR (126 MHz, Chloroform-*d*) δ 155.5, 149.8, 148.9, 136.6, 123.2, 122.3, 121.3 (broad), 83.6, 24.9.

Data are consistent with the literature.[60]

(*E*)-2-(2-(Trifluoro-λ^4-boraneyl)vinyl)pyridine, potassium salt, **S8-int4**

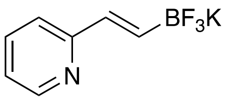

Prepared according to General Procedure 5 using (*E*)-2-(2-(4,4,5,5-tetramethyl-1,3,2-dioxaborolan-2-yl)vinyl)pyridine, **S8-int3** (855 mg, 3.70 mmol, 1.0 equiv.), potassium hydrogen fluoride (1.16 g, 14.8 mmol, 4.0 equiv.), and water (3.33 mL, 185 mmol, 50.0 equiv.) in MeOH (32 mL, 0.1 M). 472 mg of a white solid was obtained after filtration, consistent with the desired product (60%).

^{1}H NMR (500 MHz, DMSO-d_6) δ 8.42 (ddd, *J* = 4.83, 1.88, 0.92 Hz, 1H), 7.63 (td, *J* = 7.65, 1.87 Hz, 1H), 7.39 – 7.35 (m, 1H), 7.08 (ddd, *J* = 7.42, 4.81, 1.14 Hz, 1H), 6.62 – 6.56 (m, 2H).

^{11}B NMR (96 MHz, DMSO-d_6) δ 2.70.

^{13}C NMR (126 MHz, DMSO-d_6) δ 158.2, 148.9, 143.9 (broad), 136.1, 134.2 (q, $^{3}J_{CF}$ = 4.3 Hz), 120.9, 119.6.

^{19}F {^{1}H} NMR (376 MHz, DMSO-d_6) δ –138.17.

IR (solid): 1585, 1267, 1255, 1244, 1093, 1074, 1053, 1039, 987, 956 cm^{-1}.

HRMS (ESI): *m/z* calculated for [M – K]$^-$ ($C_7H_6NBF_3$)$^-$: 172.0551; found = 172.0570.

(*E*)-(2-(pyridin-2-yl)vinyl)boronic acid, **S8**

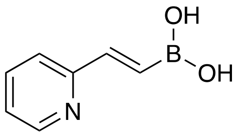

Prepared according to General Procedure 6 using (*E*)-2-(2-(trifluoro-λ^4-boraneyl)vinyl)pyridine, potassium salt, **S8-int4** (400 mg, 1.90 mmol, 1.0 equiv.) and chlorotrimethylsilane (842 µL, 6.63 mmol, 3.5 equiv.) in

MeCN:H_2O (15 mL:4 mL, 0.1 M). The aqueous layer was concentrated *in vacuo* affording 280 mg of a white solid as the desired product (99%).

^{1}H NMR (500 MHz, DMSO-d_6) δ 8.77 (d, J = 5.92, 1H), 8.52 (td, J = 7.91, 1.56 Hz, 1H), 8.24 (d, J = 8.15 Hz, 1H), 7.94 – 7.89 (m, 1H), 7.47 (d, J = 18.47 Hz, 1H), 6.95 (d, J = 18.40 Hz, 1H).

^{11}B NMR (128 MHz, DMSO-d_6) δ 27.43.

^{13}C NMR (126 MHz, DMSO-d_6) δ 149.6, 146.0, 141.8, 137.6 (broad), 135.6, 126.0, 124.2.

IR (solid): 1606, 1452, 1406, 1357, 1296, 1257, 1219, 1103, 1012, 991, 929 cm^{-1}.

HRMS (ESI): *m/z* calculated for $[M + H]^+$ $(C_7H_9BNO_2)^+$: 150.0720; found = 150.0724.

(*E*)-4,4,5,5-Tetramethyl-2-(2-(thiophen-2-yl)vinyl)-1,3,2-dioxaborolane, **S9-int3**

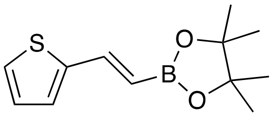

Prepared according to General Procedure 4 using 2-ethynylthiophene (501 µL, 5.00 mmol, 1.0 equiv.), copper(I) chloride (25 mg, 250 µmol, 5 mol%), potassium *tert*-butoxide (56 mg, 500 µmol, 10 mol%), bis(2-diphenylphosphinophenyl) ether (DPEPhos) (135 mg, 250 µmol, 5 mol%), bis(pinacolato)diboron (1.40 g, 5.50 mmol, 1.1 equiv.), and MeOH (405 µL, 10.0 mmol, 2.0 equiv.) in THF (20 mL, 0.25 M). The crude residue was purified by flash chromatography (silica gel) from pure hexane to a mixture of 2% of diethyl ether in hexane affording 1.05 g of a pale-yellow oil consistent with the desired product (89%).

^{1}H NMR (500 MHz, Chloroform-*d*) δ 7.47 (d, J = 18.08 Hz, 1H), 7.24 (dd, J = 5.04, 1.03 Hz, 1H), 7.09 – 7.06 (m, 1H), 6.98 (dd, J = 5.05, 3.58 Hz, 1H), 5.91 (d, J = 18.11 Hz, 1H), 1.30 (s, 12H).

^{11}B NMR (128 MHz, Chloroform-*d*) δ 29.83.

^{13}C NMR (126 MHz, Chloroform-*d*) δ 144.0, 141.9, 127.9, 127.8, 126.4, 115.9 (broad), 83.5, 24.9.

Data are consistent with the literature.[61]

(*E*)-Trifluoro(2-(thiophen-2-yl)vinyl)-λ^4-borane, potassium salt, **S9-int4**

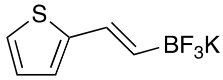

Prepared according to General Procedure 5 using (*E*)-4,4,5,5-tetramethyl-2-(2-(thiophen-2-yl)vinyl)-1,3,2-dioxaborolane, **S9-int3** (1.00 g, 4.23 mmol, 1.0 equiv.), potassium hydrogen fluoride (1.32 g, 16.9 mmol, 4.0 equiv.), and water (3.81 mL, 212 mmol, 50.0 equiv.) in MeOH (42 mL, 0.1 M). 702 mg of a white solid was obtained after filtration, consistent with the desired product (77%).

^{1}H NMR (500 MHz, DMSO-d_6) δ 7.18 (dd, J = 5.06, 1.04 Hz, 1H), 6.91 (dd, J = 5.07, 3.45 Hz, 1H), 6.80 (dd, J = 3.57, 1.13 Hz, 1H), 6.56 (d, J = 17.92 Hz, 1H), 5.89 (dq, J = 17.94, 3.64 Hz, 1H).

^{11}B NMR (128 MHz, DMSO-d_6) δ 2.30.

^{13}C NMR (126 MHz, DMSO-d_6) δ 147.3, 139.8 (broad), 127.3, 126.4 (q, $^{3}J_{CF}$ = 4.6 Hz), 122.8, 122.4.

^{19}F {^{1}H} NMR (377 MHz, DMSO-d_6) δ –138.23.

IR (solid): 1606, 1581, 1290, 1269, 1240, 1220, 1161, 1153, 1093, 1083, 1052, 989 cm^{-1}.

HRMS (ESI): *m/z* calculated for $[M - K]^-$ $(C_6H_5BF_3S)^-$: 177.0163; found = 177.0151.

(*E*)-(2-(Thiophen-2-yl)vinyl)boronic acid, **S9**

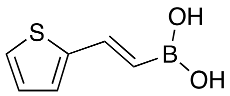

Prepared according to General Procedure 6 using (*E*)-trifluoro(2-(thiophen-2-yl)vinyl)-λ^4-borane, potassium salt, **S9-int4** (650 mg, 3.01 mmol, 1.0 equiv.) and chlorotrimethylsilane (1.34 mL, 10.5 mmol, 3.5 equiv.) in MeCN:H_2O (24 mL:6 mL, 0.1 M). 233 mg of a pale-brown solid was afforded as the desired product (50%) as a mixture of the desired product and boroxine.

^{1}H NMR (500 MHz, DMSO-*d*$_6$) δ 7.46 (d, *J* = 5.01 Hz, 1H), 7.37 (d, *J* = 18.07 Hz, 1H), 7.16 – 7.12 (m, 1H), 7.07 – 7.01 (m, 1H), 5.81 (d, *J* = 18.06 Hz, 1H).

^{11}B NMR (96 MHz, DMSO-*d*$_6$) δ 27.88.

^{13}C NMR (126 MHz, DMSO-*d*$_6$) δ 144.0, 138.7, 128.1, 127.6, 126.3, 122.7 (broad).

Data are consistent with the literature.[62]

(Benzofuran-5-ylethynyl)trimethylsilane, **S10-int1**

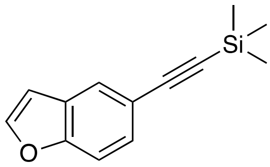

Prepared according to General Procedure 2 using 5-bromobenzofuran (626 μL, 5.00 mmol, 1 equiv.), dichlorobis(triphenylphosphine)palladium (35 mg, 50.0 μmol, 1 mol%), copper iodide (19 mg, 100 μmol, 2 mol%), trimethylsilylacetylene (5.28 mL, 50.0 mmol, 10 equiv.), and piperidine (1.48 mL, 15.0 mmol, 3 equiv.) in THF (10 mL, 0.5 M). The reaction mixture was stirred for 36 hours at 90 °C. The crude residue was purified by flash chromatography (silica gel) with pure hexane affording 827 mg of a pale-orange oil as the desired product (77%).

^{1}H NMR (500 MHz, Chloroform-*d*) δ 7.76 (t, *J* = 1.21 Hz, 1H), 7.63 (d, *J* = 2.19 Hz, 1H), 7.44 – 7.43 (m, 2H), 6.73 (d, *J* = 2.29 Hz, 1H), 0.29 (s, 9H).

^{13}C NMR (126 MHz, Chloroform-*d*) δ 154.8, 145.9, 128.5, 127.5, 125.4, 117.8, 111.5, 106.6, 105.7, 92.6, 0.2.

IR (film): 2958, 2160, 1539, 1462, 1408, 1328, 1257, 1249, 1201, 1126, 1029, 885, 812 cm^{-1}.

HRMS (MALDI): *m/z* calculated for $[M + H]^+$ $(C_{13}H_{15}OSi)^+$: 215.0887; found = 215.0886.

5-Ethynylbenzofuran, **S10-int2**

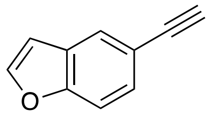

Prepared according to General Procedure 3 using (benzofuran-5-ylethynyl)trimethylsilane, **S10-int1** (820 mg, 3.83 mmol, 1 equiv.) and potassium carbonate (1.06 g, 7.65 mmol, 2 equiv.) in MeOH (20 mL, 0.2 M). The crude residue was purified by flash chromatograpghy (silica gel) with pure hexane affording 330 mg of a pale-orange oil consistent with the desired product (61%).

^{1}H NMR (400 MHz, Chloroform-*d*) δ 7.77 (t, *J* = 1.03 Hz, 1H), 7.65 (d, *J* = 2.22 Hz, 1H), 7.47 – 7.42 (m, 2H), 6.75 (dd, *J* = 2.23, 0.72 Hz, 1H), 3.04 (s, 1H).

^{13}C NMR (101 MHz, Chloroform-*d*) δ 154.9, 146.1, 128.6, 127.6, 125.6, 116.7, 111.7, 106.6, 84.1, 75.9.

Data are consistent with the literature.[63]

(*E*)-2-(2-(Benzofuran-5-yl)vinyl)-4,4,5,5-tetramethyl-1,3,2-dioxaborolane, **S10-int3**

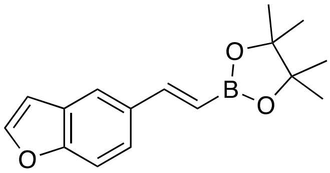

Prepared according to General Procedure 4 using 5-ethynylbenzofuran, **S10-int2** (330 mg, 2.32 mmol, 1 equiv.), copper(I) chloride (11 mg, 116 µmol 5 mol%), potassium *tert*-butoxide (26.0 mg, 232 µmol, 10 mol%), bis(2-diphenylphosphinophenyl) ether (DPEPhos) (62.5 mg, 116 µmol, 5 mol%), bis(pinacolato)diboron (648 mg, 2.55 mmol, 1.1 equiv.), and methanol (188 µL, 4.64 mmol, 2 equiv.) in THF (10 mL, 0.25 M). The crude residue was purified by flash chromatography (silica gel) from pure hexane to a mixture of 5% of diethyl ether in hexane affording 570 mg of a pale-yellow oil consistent with the desired product (91%).

^{1}H NMR (500 MHz, Chloroform-*d*) δ 7.70 (d, *J* = 1.67 Hz, 1H), 7.60 (d, *J* = 2.27 Hz, 1H), 7.51 (d, *J* = 18.21 Hz, 1H), 7.50 – 7.47 (m, 1H), 7.45 (d, *J* = 8.62 Hz, 1H), 6.76 (dd, *J* = 2.25, 0.86 Hz, 1H), 6.16 (d, *J* = 18.38 Hz, 1H), 1.32 (s, 12H).

^{11}B NMR (96 MHz, Chloroform-*d*) δ 30.41.

^{13}C NMR (126 MHz, Chloroform-*d*) δ 155.5, 149.9, 145.7, 132.8, 127.8, 123.6, 120.3, 115.1 (broad), 111.6, 106.9, 83.4, 24.9.

Data are consistent with the literature.[58]

(*E*)-(2-(Benzofuran-5-yl)vinyl)trifluoro-λ^{4}-borane, potassium salt, **S10-int4**

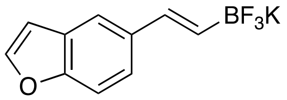

Prepared according to General Procedure 5 using (*E*)-2-(2-(benzofuran-5-yl)vinyl)-4,4,5,5-tetramethyl-1,3,2-dioxaborolane, **S10-int3** (590 mg, 2.18 mmol, 1 equiv.), potassium hydrogen fluoride (682 mg,

8.74 mmol, 4 equiv.), and water (1.97 mL, 109 mmol, 50 equiv.) in MeOH (21 mL, 0.1 M). 380 mg of a white solid was obtained after filtration, consistent with the desired product (70%).

^{1}H NMR (500 MHz, DMSO-d_6) δ 7.90 (d, J = 2.18 Hz, 1H), 7.52 (d, J = 1.70 Hz, 1H), 7.45 (d, J = 8.54 Hz, 1H), 7.31 (dd, J = 8.53, 1.74 Hz, 1H), 6.89 (d, J = 2.14 Hz, 1H), 6.55 (d, J = 18.10 Hz, 1H), 6.13 (dq, J = 18.17, 3.51 Hz, 1H).

^{11}B NMR (96 MHz, DMSO-d_6) δ 3.12.

^{13}C NMR (126 MHz, DMSO-d_6) δ 153.3, 145.9, 137.7 (broad), 135.7, 133.0 (q, $^{3}J_{CF}$ = 4.4 Hz), 127.4, 122.1, 117.7, 110.9, 106.8.

^{19}F {^{1}H} NMR (470 MHz, DMSO-d_6) δ –137.58.

IR (solid): 1627, 1537, 1406, 1330, 1309, 1236, 1124, 1093, 1026, 972, 921 cm^{-1}.

HRMS (ESI): m/z calculated for [M - K]$^-$ ($C_{10}H_7BF_3O$)$^-$: 211.0549; found = 211.0535.

(E)-(2-(Benzofuran-5-yl)vinyl)boronic acid, **S10**

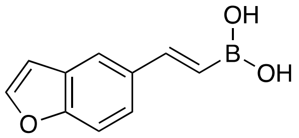

Prepared according to General Procedure 6 (E)-(2-(benzofuran-5-yl)vinyl)trifluoro-λ^4-borane, potassium salt, **S10-int4** (340 mg, 1.36 mmol, 1 equiv.) and chlorotrimethylsilane (604 µL, 4.76 mmol, 3.5 equiv.) in MeCN:H_2O (11 mL:3 mL, 0.1 M). 183 mg of a white solid was obtained as a mixture of the desired product and boroxine (72%).

^{1}H NMR (500 MHz, DMSO-d_6) δ 7.99 (d, J = 2.22 Hz, 1H), 7.78 (broad s, 2H), 7.74 (d, J = 1.75 Hz, 1H), 7.57 (d, J = 8.51 Hz, 1H), 7.47 (dd, J = 8.60, 1.81 Hz, 1H), 7.36 (d, J = 18.32 Hz, 1H), 6.97 (d, J = 2.19 Hz, 1H), 6.10 (d, J = 18.36 Hz, 1H).

^{11}B NMR (96 MHz, DMSO-d_6) δ 30.24.

^{13}C NMR (126 MHz, DMSO-d_6) δ 154.5, 146.7, 146.2, 133.0, 127.7, 123.1, 121.9 (broad), 119.7, 111.5, 107.0.

IR (solid): 1622, 1589, 1533, 1465, 1442, 1352, 1332, 1307, 1286, 1271, 1255, 1201, 1184, 1124, 1111, 1026, 898 cm^{-1}.

HRMS (ESI): m/z calculated for [M + H]$^+$ ($C_{1O}H_{10}BO_3$)$^+$: 189.0717; found = 189.0708.

((2-Bromo-4-(trifluoromethoxy)phenyl)ethynyl)trimethylsilane, **S11-int1**

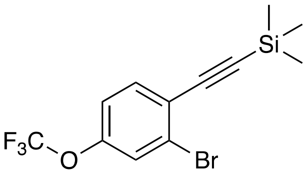

Prepared according to General Procedure 2 using 2-bromo-4-(trifluoromethoxy)iodobenzene (834 µL, 5.00 mmol, 1.0 equiv.), dichlorobis(triphenylphosphine)palladium (35 mg, 50.0 µmol, 1 mol%), copper

iodide (19 mg, 100 μmol, 2 mol%), and trimethylsilylacetylene (660 μL, 6.25 mmol, 1.25 equiv.) in triethylamine (10 mL, 0.5 M). The crude residue was purified by flash chromatography (silica gel) from pure hexane to a mixture of 45% of ethyl acetate in hexane affording 1.69 g of a yellow oil as the desired product (100%).

^{1}H NMR (500 MHz, Chloroform-*d*) δ 7.51 (d, *J* = 8.63 Hz, 1H), 7.45 (dd, *J* = 2.45, 1.02 Hz, 1H), 7.11 (ddd, *J* = 8.61, 2.40, 1.05 Hz, 1H), 0.28 (s, 9H).

^{13}C NMR (126 MHz, Chloroform-*d*) δ 148.8 (app. d, $^{3}J_{CF}$ = 2.4 Hz), 134.5, 126.5, 125.0, 124.3, 120.4 (q, $^{1}J_{CF}$ = 258.8 Hz), 119.6, 101.8, 101.0, –0.1.

^{19}F {^{1}H} NMR (377 MHz, Chloroform-*d*) δ –57.90.

IR (film): 2166, 1595, 1564, 1481, 1247, 1211, 1166, 1043, 941, 839, 759 cm^{-1}.

HRMS (ESI): *m/z* calculated for [M + H]$^{+}$ ($C_{12}H_{13}BrF_3OSi$)$^{+}$: 336.9866; found = 336.9865.

2-Bromo-1-ethynyl-4-(trifluoromethoxy)benzene, **S11-int2**

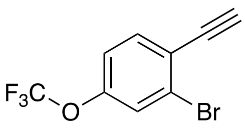

Prepared according to General Procedure 3 using ((2-bromo-4-(trifluoromethoxy)phenyl)ethynyl) trimethylsilane, **S11-int1** (1.69 g, 5.01 mmol, 1.0 equiv.) and potassium carbonate (1.39 g, 10.0 mmol, 2.0 equiv.) in MeOH (25 mL, 0.2 M). The crude residue was purified by flash chromatography (silica gel) with pure hexane affording 809 mg of an orange oil consistent with the desired product (67%).

^{1}H NMR (500 MHz, Chloroform-*d*) δ 7.55 (d, *J* = 8.56 Hz, 1H), 7.48 (dd, *J* = 2.44, 0.98 Hz, 1H), 7.18 – 7.12 (m, 1H), 3.40 (s, 1H).

^{13}C NMR (126 MHz, Chloroform-*d*) δ 149.2, 135.1, 126.4, 125.1, 123.3, 120.4 (q, $^{1}J_{CF}$ = 259.1 Hz), 119.7, 82.9, 80.8.

^{19}F {^{1}H} NMR (470 MHz, Chloroform-*d*) δ –57.87.

IR (film): 3310, 1569, 1566, 1481, 1249, 1211, 1166, 1043, 912, 825, 144 cm^{-1}.

HRMS (ESI): *m/z* calculated for [M + H]$^{+}$ ($C_9H_5BrF_3O$)$^{+}$: 264.9470; found = 264.9470.

(*E*)-2-(2-Bromo-4-(trifluoromethoxy)styryl)-4,4,5,5-tetramethyl-1,3,2-dioxaborolane, **S11-int3**

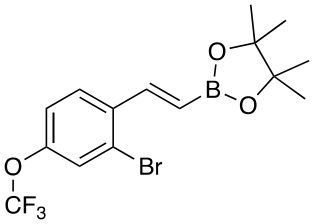

Prepared according to General Procedure 4 using 2-bromo-1-ethynyl-4-(trifluoromethoxy)benzene, **S11-int2** (890 mg, 3.36 mmol, 1.0 equiv.), copper(I) chloride (17 mg, 168 μmol, 5 mol%), potassium *tert*-butoxide (38 mg, 336 μmol, 10 mol%), bis(2-diphenylphosphinophenyl) ether (DPEPhos) (90 mg, 168 μmol, 5 mol%), bis(pinacolato)diboron (938 mg, 3.69 mmol, 1.1 equiv.), and MeOH (272 μL, 6.72 mmol, 2.0 equiv.) in THF

(14 mL, 0.25 M). The crude residue was purified by flash chromatography (silica gel) from pure hexane to a mixture of 5% of diethyl ether in hexane affording 889 mg of a pale-yellow oil consistent with the desired product (67%).

^{1}H NMR (500 MHz, Chloroform-*d*) δ 7.64 (d, *J* = 18.23 Hz, 1H), 7.62 (d, *J* = 8.67 Hz, 1H), 7.44 (dd, *J* = 2.44, 0.99 Hz, 1H), 7.18 – 7.15 (m, 1H), 6.10 (d, *J* = 18.26 Hz, 1H), 1.32 (s, 12H).

^{11}B NMR (96 MHz, Chloroform-*d*) δ 29.54.

^{13}C NMR (126 MHz, Chloroform-*d*) δ 149.2, 146.1, 136.4, 128.2, 125.4, 124.3, 121.2 (broad), 120.1, 120.1 (q, $^{1}J_{CF}$ = 260.3 Hz), 83.8, 25.0.

^{19}F {^{1}H} NMR (470 MHz, Chloroform-*d*) δ –57.87.

IR (film): 2980, 1622, 1595, 1568, 1479, 1388, 1379, 1371, 1348, 1249, 1213, 1163, 1139, 1111, 991 cm^{-1}.

HRMS (MALDI): *m/z* calculated for $[M + H]^+$ $(C_{15}H_{18}BBrF_3O_3)^+$: 393.0479; found = 393.0481.

(*E*)-(2-Bromo-4-(trifluoromethoxy)styryl)trifluoro-λ^4-borane, potassium salt, **S11-int4**

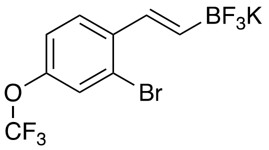

Prepared according to General Procedure 5 using (*E*)-2-(2-bromo-4-(trifluoromethoxy)styryl)-4,4,5,5-tetramethyl-1,3,2-dioxaborolane, **S11-int3** (890 mg, 2.26 mmol, 1.0 equiv.), potassium hydrogen fluoride (707 mg, 9.06 mmol, 4.0 equiv.), and water (2.04 mL, 113 mmol, 50.0 equiv.) in MeOH (21 mL, 0.1 M). 559 mg of a white solid was obtained after filtration, consistent with the desired product (66%).

^{1}H NMR (500 MHz, DMSO-d_6) δ 7.68 (d, *J* = 8.71 Hz, 1H), 7.60 – 7.55 (m, 1H), 7.37 – 7.29 (m, 1H), 6.75 (d, *J* = 17.92 Hz, 1H), 6.26 (dq, *J* = 18.03, 3.47 Hz, 1H).

^{11}B NMR (96 MHz, DMSO-d_6) δ 2.74.

^{13}C NMR (126 MHz, DMSO-d_6) δ 146.3, 144.9 (broad), 139.0, 129.8 (q, $^{3}J_{CF}$ = 4.4 Hz), 127.5, 125.0, 122.0, 120.5, 120.0 (q, $^{1}J_{CF}$ = 256.7 Hz).

^{19}F {^{1}H} NMR (376 MHz, DMSO-d_6) δ –57.02, –138.33.

IR (solid): 1629, 1600, 1483, 1309, 1288, 1209, 1159, 1099, 1037, 987, 937 cm^{-1}.

HRMS (ESI): *m/z* calculated for $[M - K]^-$ $(C_9H_5BBrF_6O)^-$: 332.9528; found = 332.9512.

(*E*)-(2-Bromo-4-(trifluoromethoxy)styryl)boronic acid, **S11**

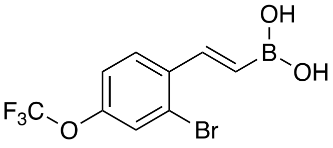

Prepared according to General Procedure 6 using (*E*)-(2-bromo-4-(trifluoromethoxy)styryl)trifluoro-λ^4-borane, potassium salt, **S11-int4** (507 mg, 1.36 mmol, 1.0 equiv.), chlorotrimethylsilane (604 µL, 4.76 mmol,

3.5 equiv.) in MeCN:H_2O (11 mL:3 mL, 0.1 M). 245 mg of a white solid was obtained as a mixture of the desired product and boroxine (58%).

^{1}H NMR (500 MHz, DMSO-d_6) δ 7.94 (s, 2H), 7.78 (d, J = 8.73 Hz, 1H), 7.69 (dd, J = 2.51, 0.93 Hz, 1H), 7.47 (d, J = 18.17 Hz, 1H), 7.43 – 7.40 (m, 1H), 6.15 (d, J = 18.11 Hz, 1H).

^{11}B NMR (96 MHz, DMSO-d_6) δ 30.41.

^{13}C NMR (126 MHz, DMSO-d_6) δ 148.0, 142.1, 136.8, 128.8 (broad), 128.6, 125.3, 123.3, 120.8, 120.0 (q, $^1J_{CF}$ = 257.4 Hz).

^{19}F {^{1}H} NMR (470 MHz, DMSO-d_6) δ –56.99.

IR (solid): 1616, 1477, 1363, 1268, 1249, 1209, 1155, 1037, 993, 817 cm^{-1}.

HRMS (ESI): *m/z* calculated for $[M + Na]^-$ ($C_9H_7BBrF_3O_3Na$)$^-$: 332.9529; found = 332.9518.

4,4,5,5-Tetramethyl-2-((1*E*,3*E*)-4-phenylbuta-1,3-dien-1-yl)-1,3,2-dioxaborolane, **S12-int3**

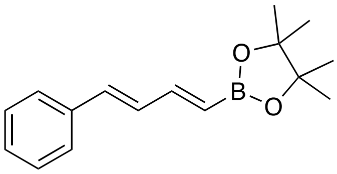

Prepared according to the literature.[64] To a flame-dried flask, purged with vacuum-N_2 cycles, and backfilled with N_2 was added 2,2,6,6-tetramethylpiperidine (658 µL, 3.90 mmol, 1.3 equiv.) in THF (10 mL). The flask was cooled to –78 °C and *n*-BuLi (2.5 M in hexanes, 1.95 mL, 3.90 mmol, 1.3. equiv.) was added dropwise. The mixture was allowed to warm to 0 °C and stirred at 0 °C for one hour. Then a solution of bis(4,4,5,5-tetramethyl-1,3,2-dioxaborolan-2-yl)methane (884 mg, 3.30 mmol, 1.1 equiv.) in THF (5 mL) was added. The reaction vial was allowed to stir for 20 minutes at 0 °C. Then the flask was cooled to –78°C and a solution of cinnamaldehyde (378 µL, 3.00 mmol, 1.0 equiv.) in THF (5 mL) was added. The reaction vial was slowly warmed up to room temperature and left to stir for six hours. The reaction mixture was partitioned between water (20 mL) and diethyl ether (20 mL). The organics were extracted with diethyl ether (3 × 20 mL). The combined organic layers were washed with brine (30 mL), dried over sodium sulfate, and concentrated *in vacuo*. The crude residue was purified by column chromatography (silica gel) from pure hexane to a mixture of 5% of diethyl ether in hexane affording 283 mg of a yellow oil as the desired product (37%).

^{1}H NMR (500 MHz, Chloroform-*d*) δ 7.45 – 7.42 (m, 2H), 7.36 – 7.29 (m, 2H), 7.28 – 7.24 (m, 1H), 7.18 (dd, J = 17.60, 10.45 Hz, 1H), 6.85 (ddd, J = 15.58, 10.42, 0.85 Hz, 1H), 6.70 (d, J = 15.58 Hz, 1H), 5.67 (d, J = 17.60 Hz, 1H), 1.30 (s, 12H).

^{11}B NMR (96 MHz, Chloroform-*d*) δ 30.17.

^{13}C NMR (126 MHz, Chloroform-*d*) δ 149.9, 136.9, 136.3, 130.7, 128.8, 128.3, 127.0, 121.3 (broad), 83.4, 24.9.

Data are consistent with the literature.[65]

Trifluoro((1*E*,3*E*)-4-phenylbuta-1,3-dien-1-yl)-λ^4-borane, potassium salt, **S12-int4**

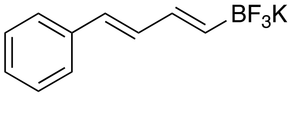

Prepared according to General Procedure 5 using 4,4,5,5-tetramethyl-2-((1*E*,3*E*)-4-phenylbuta-1,3-dien-1-yl)-1,3,2-dioxaborolane, **S12-int3** (286 mg, 1.12 mmol, 1.0 equiv.) potassium hydrogen fluoride (349 mg, 4.47 mmol, 4.0 equiv.), and water (1.01 mL, 55.8 mmol, 50.0 equiv.) in MeOH (11 mL, 0.1 M). 231 mg of a white solid was obtained after filtration, consistent with the desired product (89%).

^{1}H NMR (500 MHz, DMSO-d_6) δ 7.42 – 7.38 (m, 2H), 7.32 – 7.25 (m, 2H), 7.18 – 7.13 (m, 1H), 6.73 (dd, *J* = 15.67, 10.30 Hz, 1H), 6.32 (d, *J* = 15.98 Hz, 1H), 6.27 (dd, *J* = 10.73, 7.03 Hz, 1H), 5.75 (dq, *J* = 17.38, 3.89 Hz, 1H).

^{11}B NMR (96 MHz, DMSO-d_6) δ 3.55.

^{13}C NMR (126 MHz, DMSO-d_6) δ 146.9 (broad), 138.0, 134.1 (q, $^{3}J_{CF}$ = 4.5 Hz), 133.9, 128.6, 127.2, 126.6, 125.8.

^{19}F {^{1}H} NMR (470 MHz, DMSO-d_6) δ –137.90.

IR (solid): 1597, 1490, 1488, 1388, 1201, 1134, 1091, 954, 840 cm^{-1}.

HRMS (ESI): *m/z* calculated for $[M – K]^-$ $(C_{10}H_9BF_3)^-$: 197.0754; found = 197.0751.

Data are consistent with the literature.[66]

((1*E*,3*E*)-4-Phenylbuta-1,3-dien-1-yl)boronic acid, **S12**

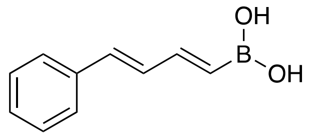

Prepared according to General Procedure 6 using trifluoro((1*E*,3*E*)-4-phenylbuta-1,3-dien-1-yl)-λ^4-borane, potassium salt, **S12-int4** (300 mg, 1.27 mmol, 1.0 equiv.) and chlorotrimethylsilane (564 µL, 4.45 mmol, 3.5 equiv.) in MeCN:H_2O (10 mL:2.5 mL, 0.1 M). 41 mg of a yellow solid was obtained as a mixture of the desired product and boroxine (19%).

^{1}H NMR (500 MHz, Acetone-d_6) δ 7.53 – 7.47 (m, 2H), 7.38 – 7.31 (m, 2H), 7.29 – 7.22 (m, 1H), 7.16 (dd, *J* = 17.47, 10.42 Hz, 1H), 6.95 (ddd, *J* = 15.68, 10.44, 0.88 Hz, 1H), 6.88 (s, 2H), 6.72 (d, *J* = 15.62 Hz, 1H), 5.73 (d, *J* = 17.46 Hz, 1H).

^{11}B NMR (96 MHz, Acetone-d_6) δ 28.35.

^{13}C NMR (126 MHz, Acetone-d_6) δ 148.0, 138.0, 135.5, 131.9, 129.5, 128.8, 127.5. The boron-bearing the carbon is not observed due to quadrupolar relaxation.

Data are consistent with the literature.[3]

Methyl 2-((trimethylsilyl)ethynyl)benzoate, **S13-int1**

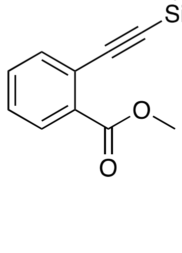

Prepared according to General Procedure 2 using methyl 2-iodobenzoate (1.47 mL, 10.0 mmol, 1.0 equiv.), dichlorobis(triphenylphosphine)palladium (70 mg, 100 µmol, 1 mol%), copper iodide (38 mg, 200 µmol, 2 mol%), and trimethylsilylacetylene (1.32 mL, 12.5 mmol, 1.25 equiv.) in triethylamine (20 mL, 0.5 M). The crude residue was purified by flash chromatography (silica gel) from pure hexane to a mixture of 2% of diethyl ether in hexane affording 2.32 g of a yellow oil as the desired product (100%).

^{1}H NMR (400 MHz, Chloroform-*d*) δ 7.90 (ddd, *J* = 7.78, 1.48, 0.56 Hz, 1H), 7.58 (ddd, *J* = 7.78, 1.44, 0.58 Hz, 1H), 7.44 (td, *J* = 7.58, 1.46 Hz, 1H), 7.36 (td, *J* = 7.70, 1.41 Hz, 1H), 3.92 (s, 3H), 0.27 (s, 9H).

^{13}C NMR (101 MHz, Chloroform-*d*) δ 167.0, 134.7, 132.7, 131.6, 130.4, 128.3, 123.3, 103.4, 99.8, 52.1, 0.0.

Data are consistent with the literature.[67]

Methyl 2-ethynylbenzoate, **S13-int2**

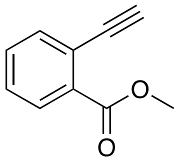

Prepared according to General Procedure 3 using methyl 2-((trimethylsilyl)ethynyl)benzoate, **S13-int1** (2.60 g, 11.2 mmol, 1.0 equiv.) and potassium carbonate (3.09 g, 22.4 mmol, 2.0 equiv.) in MeOH (55 mL, 0.2 M). Organics layers were concentrated *in vacuo* affording 1.32 g of a dark red oil consistent with the desired product (74%).

^{1}H NMR (500 MHz, Chloroform-*d*) δ 7.93 (ddd, *J* = 7.80, 1.46, 0.54 Hz, 1H), 7.62 (dd, *J* = 7.72, 1.14 Hz, 1H), 7.47 (td, *J* = 7.61, 1.46 Hz, 1H), 7.40 (td, *J* = 7.70, 1.37 Hz, 1H), 3.92 (s, 3H), 3.40 (s, 1H).

^{13}C NMR (126 MHz, Chloroform-*d*) δ 166.5, 135.1, 132.6, 131.8, 130.4, 128.6, 122.7, 82.4, 82.1, 52.3.

Data are consistent with the literature.[67]

Methyl (*E*)-2-(2-(4,4,5,5-tetramethyl-1,3,2-dioxaborolan-2-yl)vinyl)benzoate, **S13-int3**

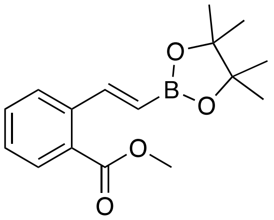

Prepared according to General Procedure 4 using methyl 2-ethynylbenzoate, **S13-int2** (1.30 g, 8.12 mmol, 1.0 equiv.), copper(I) chloride (40 mg, 406 µmol, 5 mol%), potassium *tert*-butoxide (91 mg, 812 µmol, 10 mol%), bis(2-diphenylphosphinophenyl) ether (DPEPhos) (219 mg, 406 µmol, 5 mol%), bis(pinacolato)diboron (2.27 g, 8.93 mmol, 1.1 equiv.), and MeOH (657 µL, 16.2 mmol, 2.0 equiv.) in THF (32 mL, 0.25 M). The crude residue was purified by flash chromatography (silica gel) from pure hexane to a mixture of 5% of diethyl ether in hexane affording 2.27 g of a pale-yellow oil consistent with the desired product (97%).

^{1}H NMR (400 MHz, Chloroform-*d*) δ 8.07 (d, *J* = 18.25 Hz, 1H), 7.85 (ddd, *J* = 7.82, 1.47, 0.52 Hz, 1H), 7.63 (ddd, *J* = 7.93, 1.30, 0.63 Hz, 1H), 7.48 (td, *J* = 7.88, 1.45, 1H), 7.33 (td, *J* = 7.59, 1.30 Hz, 1H), 6.07 (d, *J* = 18.23 Hz, 1H), 3.90 (s, 3H), 1.30 (s, 12H).

^{11}B NMR (96 MHz, Chloroform-*d*) δ 30.22.

^{13}C NMR (101 MHz, Chloroform-*d*) δ 167.8, 148.1, 139.7, 132.2, 130.3, 129.2, 128.2, 127.6, 120.1 (broad), 83.5, 52.3, 24.9.

IR (film): 1720, 1620, 1474, 1381, 1371, 1346, 1325, 1290, 1251, 1205, 1141, 1128, 1076, 955 cm^{-1}.

HRMS (ESI): *m/z* calculated for $[M + K]^+$ $(C_{16}H_{21}BO_4K)^+$: 327.1173; found = 327.1167.

Methyl (*E*)-2-(2-(trifluoro-λ^4-boraneyl)vinyl)benzoate, potassium salt, **S13-int4**

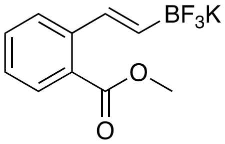

Prepared according to General Procedure 5 using (methyl (*E*)-2-(2-(4,4,5,5-tetramethyl-1,3,2-dioxaborolan-2-yl)vinyl)benzoate, **S13-int3** (2.20 g, 7.63 mmol, 1.0 equiv.), potassium hydrogen fluoride (2.39 g, 30.5 mmol, 4.0 equiv.), and water (6.88 mL, 382 mmol, 50.0 equiv.) in MeOH (65 mL, 0.1 M). 3.1 g of a white solid was obtained, consistent with the desired product and residual potassium hydrogen fluoride (147%). No further purification was performed and the crude was taken directly into the next step.

^{1}H NMR (400 MHz, DMSO-d_6) δ 7.61 (dd, *J* = 8.04, 1.25 Hz, 1H), 7.57 (dd, *J* = 7.78, 1.44 Hz, 1H), 7.47 – 7.41 (m, 1H), 7.21 (td, *J* = 7.45, 1.24 Hz, 1H), 6.99 (d, *J* = 18.07 Hz, 1H), 6.19 (dq, *J* = 18.09, 3.58 Hz, 1H), 3.80 (s, 3H).

^{11}B NMR (96 MHz, DMSO-d_6) δ 2.74.

^{13}C NMR (126 MHz, DMSO-d_6) δ 168.3, 142.8 (broad), 140.5, 131.3, 130.2 (q, $^3J_{CF}$ = 4.6 Hz), 129.0, 128.7, 125.7, 125.6, 51.9.

^{19}F {^{1}H} NMR (376 MHz, DMSO-d_6) δ –137.87.

IR (solid): 1707, 1479, 1438, 1305, 1267, 1232, 1205, 1132, 1078, 997, 928 cm^{-1}.

HRMS (MALDI): *m/z* calculated for $[M – K]^-$ $(C_{10}H_9BF_3O_2)^-$: 229.0639; found = 229.0655.

(*E*)-(2-(methoxycarbonyl)styryl)boronic acid, **S13**

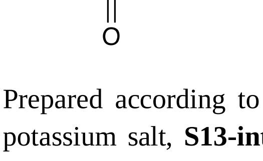

Prepared according to General Procedure 6 using methyl (*E*)-2-(2-(trifluoro-λ^4-boraneyl)vinyl)benzoate, potassium salt, **S13-int4** (190 mg, 709 µmol, 1.0 equiv.) and chlorotrimethylsilane (315 µL, 2.48 mmol, 3.5 equiv.) in MeCN:H_2O (6 mL:1.5 mL, 0.1 M). 120 mg of a white solid was obtained as a mixture of the desired product and boroxine (82%).

^{1}H NMR (500 MHz, DMSO-d_6) δ 7.86 (s, 2H), 7.75 – 7.72 (m, 1H), 7.74 (d, *J* = 17.92 Hz, 1H), 7.67 (d, *J* = 7.87 Hz, 1H), 7.57 (t, *J* = 7.58 Hz, 1H), 7.41 (t, *J* = 7.53 Hz, 1H), 6.04 (d, *J* = 18.20 Hz, 1H), 3.84 (s, 3H).

^{11}B NMR (96 MHz, DMSO-d_6) δ 26.65.

^{13}C NMR (126 MHz, DMSO-d_6) δ 167.6, 143.7, 138.6, 132.1, 129.6, 129.4, 128.1, 126.8, 52.3. The boron-bearing carbon is not observed due to quadrupolar relaxation.

IR (solid): 1722, 1618, 1597, 1568, 1479, 1448, 1435, 1371, 1292, 1249, 1163, 1130, 1074, 987 cm^{-1}.

HRMS (ESI): *m/z* calculated for $[M + H]^+$ $(C_{10}H_{12}BO_4)^+$: 207.0823; found = 207.0826.

((3'-Methoxy-[1,1'-biphenyl]-2-yl)ethynyl)trimethylsilane, **S14-int1**

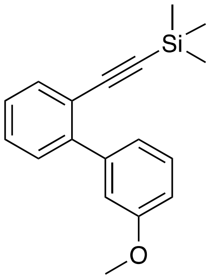

An oven-dried microwave vial was charged with 1,1'-bis(diphenylphosphino)ferrocenepalladium chloride (173 mg, 237 µmol, 5 mol%), 3-methoxybenzeneboronic acid (1.44 g, 9.48 mmol, 2.0 equiv.), and potassium phosphate (3.02 g, 14.2 mmol, 3.0 equiv.). The vial was then sealed and purged with vacuum-N_2 cycles (3 times) and backfilled with N_2. Toluene (19 mL, 0.25 M) was added followed by ((2-bromophenyl)ethynyl)trimethylsilane, **S6-int1** (844 µL, 4.74 mmol, 1.0 equiv.) and water (4.27 mL, 237 mmol, 50.0 equiv.). The reaction mixture was stirred for 24 hours at 90 °C. The crude mixture was cooled down, partitioned between ethyl acetate (20 mL) and brine (20 mL). Organics were extracted with ethyl acetate (2 × 20 mL). Organics were combined, washed with brine (20 mL–, dried over sodium sulfate, filtered, and concentrated *in vacuo*. The crude residue was purified by flash chromatography (silica gel) from pure hexane to a mixture of 1% of diethyl ether in hexane affording 1.29 g of a yellow oil as the desired product (97%).

^{1}H NMR (500 MHz, Chloroform-*d*) δ 7.61 (dd, *J* = 7.69, 0.71 Hz, 1H), 7.43 – 7.41 (m, 1H), 7.39 (td, *J* = 7.39, 1.39 Hz, 1H), 7.35 (t, *J* = 7.99 Hz, 1H), 7.30 (td, *J* = 7.59, 1.76 Hz, 1H), 7.23 – 7.20 (m, 2H), 6.95 (ddd, *J* = 8.23, 2.55, 1.03 Hz, 1H), 3.87 (s, 3H), 0.18 (s, 9H).

^{13}C NMR (126 MHz, Chloroform-*d*) δ 159.2, 144.2, 141.8, 133.5, 129.4, 128.9, 128.8, 127.1, 121.9, 121.5, 115.1, 113.2, 104.8, 97.7, 55.3, –0.1.

IR (neat liquid): 2156, 1602, 1579, 1469, 1438, 1419, 1296, 1247, 1207, 1178, 1053, 1020, 866 cm^{-1}.

HRMS (ESI): *m/z* calculated for $[M + H]^+$ $(C_{28}H_{21}OSi)^+$: 281.1356; found = 281.1356.

2-Ethynyl-3'-methoxy-1,1'-biphenyl, **S14-int2**

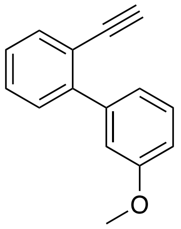

Prepared according to General Procedure 3 using ((3'-methoxy-[1,1'-biphenyl]-2-yl)ethynyl)trimethylsilane, **S14-int1** (1.29 g, 4.60 mmol, 1.0 equiv.) and potassium carbonate (1.27 g, 9.20 mmol, 2.0 equiv.) in MeOH (23 mL, 0.2 M). The crude residue was purified by flash chromatography (silica gel) from pure hexane to a mixture of 10% of diethyl ether in hexane affording 967 mg of a yellow oil consistent with the desired product (100%).

^{1}H NMR (500 MHz, Chloroform-*d*) δ 7.63 (dt, *J* = 7.76, 1.05 Hz, 1H), 7.43 – 7.39 (m, 2H), 7.35 (t, *J* = 8.11 Hz, 1H), 7.31 (ddd, *J* = 7.67, 6.43, 2.32 Hz, 1H), 7.21 – 7.14 (m, 2H), 6.94 (ddd, *J* = 8.26, 2.54, 1.06 Hz, 1H), 3.86 (s, 3H), 3.07 (s, 1H).

^{13}C NMR (126 MHz, Chloroform-*d*) δ 159.3, 144.4, 141.7, 134.0, 129.7, 129.2, 129.1, 127.2, 121.8, 120.5, 114.9, 113.5, 83.2, 80.5, 55.4.

IR (neat liquid): 3280, 1602, 1579, 1562, 1496, 1469, 1438, 1317, 1296, 1265, 1209, 1170, 1041, 877 cm^{-1}.

HRMS (ESI): *m/z* calculated for $[M + H]^+$ $(C_{15}H_{13}O)^+$: 209.0960; found = 209.0966.

(*E*)-2-(2-(3'-Methoxy-[1,1'-biphenyl]-2-yl)vinyl)-4,4,5,5-tetramethyl-1,3,2-dioxaborolane, **S14-int3**

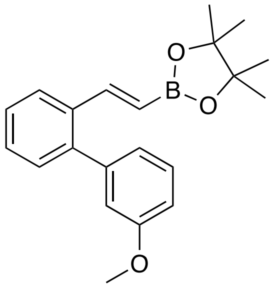

Prepared according to General Procedure 4 using 2-ethynyl-3'-methoxy-1,1'-biphenyl, **S14-int2** (900 mg, 4.32 mmol, 1.0 equiv.), copper(I) chloride (43 mg, 432 μmol, 5 mol%), potassium *tert*-butoxide (97 mg, 864 μmol, 10 mol%), bis(2-diphenylphosphinophenyl) ether (DPEPhos) (233 mg, 432 μmol, 5 mol%), bis(pinacolato)diboron 2.41 g, 9.51 mmol, 1.1 equiv.), and MeOH (699 μL, 17.3 mmol, 2.0 equiv.) in THF (17 mL, 0.25 M). The crude residue was purified by flash chromatography (silica gel) from pure hexane to a mixture of 5% of diethyl ether in hexane affording 1.30 g of a pale-yellow oil consistent with the desired product (90%).

^{1}H NMR (500 MHz, Chloroform-*d*) δ 7.74 – 7.67 (m, 1H), 7.49 (d, *J* = 18.30 Hz, 1H), 7.38 – 7.33 (m, 4H), 6.97 (dt, *J* = 7.60, 1.26 Hz, 1H), 6.93 (ddd, *J* = 8.14, 2.60, 0.97 Hz, 1H), 6.93 – 6.89 (m, 1H), 6.14 (d, *J* = 18.29 Hz, 1H), 3.84 (s, 3H), 1.27 (s, 12H).

^{11}B NMR (96 MHz, Chloroform-*d*) δ 31.72.

^{13}C NMR (126 MHz, Chloroform-*d*) δ 159.3, 148.9, 142.0, 141.3, 136.1, 130.2, 129.2, 128.6, 127.7, 126.6, 122.5, 118.1 (broad), 115.2, 113.6, 83.3, 55.4, 24.9.

IR (neat liquid): 1616, 1577, 1467, 1419, 1379, 1371, 1344, 1321, 1267, 1211, 1139, 1043, 968 cm^{-1}.

HRMS (ESI): *m/z* calculated for $[M + H]^+$ $(C_{21}H_{26}BO_3)^+$: 337.1969; found = 337.1967.

(*E*)-trifluoro(2-(3'-methoxy-[1,1'-biphenyl]-2-yl)vinyl)-λ^4-borane, potassium salt, **S14-int4**

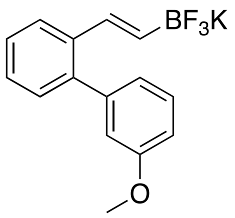

Prepared according to General Procedure 5 using (*E*)-2-(2-(3'-Methoxy-[1,1'-biphenyl]-2-yl)vinyl)-4,4,5,5-tetramethyl-1,3,2-dioxaborolane, **S14-int3** (1.00 g, 2.97 mmol, 1.0 equiv.), potassium hydrogen fluoride (929 mg, 11.9 mmol, 4.0 equiv.), and water (2.68 mL, 149 mmol, 50.0 equiv.) in MeOH (26 mL, 0.1 M). 408 mg of a white solid was obtained after filtration, consistent with the desired product (43%).

^{1}H NMR (500 MHz, DMSO-d_6) δ 7.59 (dd, *J* = 7.90, 1.31 Hz, 1H), 7.33 (t, *J* = 7.87 Hz, 1H), 7.28 (td, *J* = 8.06, 1.67 Hz, 1H), 7.18 (td, *J* = 7.29, 1.28 Hz, 1H), 7.15 (dd, *J* = 7.59, 1.70 Hz, 1H), 6.91 (ddd, *J* = 8.28, 2.61, 0.97 Hz, 1H), 6.85 (dt, *J* = 7.48, 1.27 Hz, 1H), 6.82 (dd, *J* = 2.66, 1.51 Hz, 1H), 6.50 (d, *J* = 18.04 Hz, 1H), 6.15 (dq, *J* = 18.10, 3.58 Hz, 1H), 3.77 (s, 3H).

^{11}B NMR (96 MHz, DMSO-d_6) δ 2.89.

^{13}C NMR (126 MHz, DMSO-d_6) δ 158.8, 142.6, 140.5 (broad), 139.2, 138.0, 131.1 (q, $^3J_{CF}$ = 4.7 Hz), 129.7, 129.1, 127.4, 125.8, 125.0, 121.9, 115.1, 112.4, 55.0.

^{19}F {^{1}H} NMR (470 MHz, DMSO-d_6) δ –137.53.

IR (solid): 1608, 1579, 1469, 1458, 1421, 1292, 1269, 1234, 1211, 1166, 1089, 1047, 927 cm^{-1}.

HRMS (ESI): *m/z* calculated for $[M - K]^-$ $(C_{15}H_{13}BF_3O)^-$: 277.1017; found = 277.1025.

(*E*)-(2-(3'-Methoxy-[1,1'-biphenyl]-2-yl)vinyl)boronic acid, **S14**

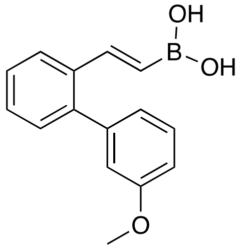

Prepared according to General Procedure 6 using a mixture of (*E*)-trifluoro(2-(3'-methoxy-[1,1'-biphenyl]-2-yl)vinyl)-λ^4-borane, potassium salt, **S14-int4** (300 mg, 1.05 mmol, 1.0 equiv.) and chlorotrimethylsilane (466 µL, 3.67 mmol, 3.5 equiv.) in MeCN:H_2O (8 mL:2 mL, 0.1 M). 136 mg of a white solid was obtained as the desired product (33%). It contained protodeboronated product, no further purification was caried out (35%).

^{1}H NMR (500 MHz, Acetone-d_6) δ 7.75 – 7.72 (m, 1H), 7.45 (d, *J* = 18.29 Hz, 1H), 7.43 – 7.28 (m, 4H), 6.98 – 6.88 (m, 3H), 6.87 (s, 2H), 6.20 (d, *J* = 18.25 Hz, 1H), 3.83 (s, 3H).

^{11}B NMR (96 MHz, Acetone-d_6) δ 28.75.

^{13}C NMR (126 MHz, Acetone-d_6) δ 160.4, 146.5, 143.0, 142.0, 137.1, 130.9, 130.0, 129.0, 128.5, 126.9, 122.9, 116.0, 113.7, 55.5. The boron-bearing atom is not observed due to quadrupolar relaxation.

IR (solid): 1612, 1597, 1577, 1467, 1442, 1419, 1346, 1290, 1267, 1211, 1178, 1045, 995 cm^{-1}.

HRMS (ESI): *m/z* calculated for $[M + H]^+$ $(C_{15}H_{16}BO_3)^+$: 255.1187; found = 255.1182.

(*E*)-4,4,5,5-Tetramethyl-2-(2-phenylprop-1-en-1-yl)-1,3,2-dioxaborolane, **65-int3**

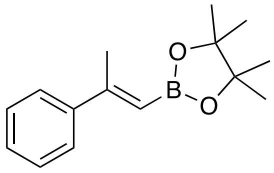

Prepared according to the literature.[5] In an oven-dried microwave vial loaded with a Teflon-coated stir bar, copper(I) chloride (10 mg, 100 µmol, 10 mol%), bis(pinacolato)diboron (279 mg, 1.10 mmol, 1.1 equiv.), 4,5-bis(diphenylphosphino)-8,9-dimethylxanthene (XantPhos) (58 mg, 100 µmol, 10 mol%), and potassium *tert*-butoxide (123 mg, 1.10 mmol, 1.1 equiv.) were weighed out. The vial was sealed, purged with N_2-vacuum cycles, and backfilled with N_2. THF (5.00 mL) was added and the mixture was stirred for 10 minutes at room temperature. Phenylacetylene (110 µL, 1.00 mmol, 1.0 equiv.) was added followed by iodomethane (249 µL, 4.00 mmol, 4.0 equiv.). The reaction mixture was stirred overnight at room temperature. The reaction mixture was filtered through a celite pad and the crude residue was purified by flash chromatography (silica gel) from pure hexane to a mixture of 2% of diethyl ether in hexane affording 137 mg of a colourless oil consistent with the desired product (56%).

^{1}H NMR (500 MHz, Chloroform-*d*) δ 7.53 – 7.49 (m, 2H), 7.36 – 7.31 (m, 2H), 7.31 – 7.27 (m, 1H), 5.77 (q, *J* = 0.98 Hz, 1H), 2.42 (d, *J* = 1.01 Hz, 3H), 1.32 (s, 12H).

^{11}B NMR (96 MHz, Chloroform-*d*) δ 30.00.

^{13}C NMR (126 MHz, Chloroform-*d*) δ 157.9, 143.9, 128.3, 128.0, 125.9, 115.6 (broad), 83.1, 25.0, 20.2.

Data are consistent with the literature.[68]

(*E*)-trifluoro(2-phenylprop-1-en-1-yl)-λ^4-borane, potassium salt, **65-int4**

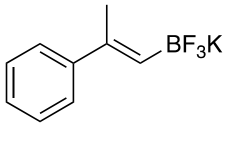

Prepared according to General Procedure 5 using (*E*)-4,4,5,5-tetramethyl-2-(2-phenylprop-1-en-1-yl)-1,3,2-dioxaborolane, **65-int3** (440 mg, 1.80 mmol, 1.0 equiv.), potassium hydrogen fluoride (563 mg, 7.21 mmol, 4.0 equiv.), and water (1.62 mL, 90.1 mmol, 50.0 equiv.) in MeOH (15 mL, 0.1 M). 311 mg of a white solid was obtained after filtration, consistent with the desired product (77%).

^{1}H NMR (500 MHz, DMSO-d_6) δ 7.36 – 7.33 (m, 2H), 7.26 – 7.21 (m, 2H), 7.13 – 7.08 (m, 1H), 5.72 (qd, *J* = 5.07, 1.07 Hz, 1H), 2.03 (s, 3H).

^{13}C NMR (126 MHz, DMSO-d_6) δ 146.0, 138.2 (q, $^{3}J_{CF}$ = 4.7 Hz), 127.8, 125.4, 124.8, 18.4. The boron-bearing carbon is not observed due to quadrupolar relaxation.

^{11}B NMR (96 MHz, DMSO-d_6) δ 2.75.

^{19}F {^{1}H} NMR (376 MHz, DMSO-d_6) δ –132.41.

Data are consistent with the literature.[68]

(*E*)-(2-Phenylprop-1-en-1-yl)boronic acid, **65**

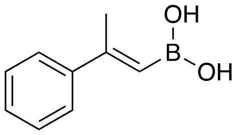

Prepared according to General Procedure 6 using (*E*)-trifluoro(2-phenylprop-1-en-1-yl)-λ^4-borane, potassium salt, **65-int4** (270 mg, 1.20 mmol, 1.0 equiv.) and chlorotrimethylsilane (535 µL, 4.22 mmol, 3.5 equiv.) in MeCN:H_2O (10 mL:3 mL, 0.1 M). 41 mg of a white solid was obtained as a mixture of the desired product and boroxine (21%). It contained 7% of (*E*)-styrylboronic acid (**1a**).

^{1}H NMR (400 MHz, Acetone-d_6) δ 7.56 – 7.48 (m, 2H), 7.39 – 7.32 (m, 2H), 7.31 – 7.23 (m, 1H), 6.98 (s, 2H), 5.80 (q, *J* = 1.07 Hz, 1H), 2.39 (d, *J* = 1.04 Hz, 3H).

^{11}B NMR (96 MHz, Acetone-d_6) δ 28.67.

^{13}C NMR (126 MHz, Acetone-d_6) δ 154.2, 145.5, 129.0, 128.3, 126.4, 19.8. The boron-bearing carbon is not observed due to quadrupolar relaxation.

Data are consistent with the literature.[69]

(*Z*)-4,4,5,5-Tetramethyl-2-(1-phenylprop-1-en-2-yl)-1,3,2-dioxaborolane, **66-int3**

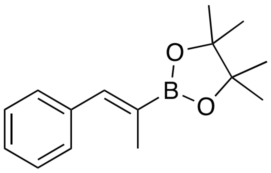

Prepared according to the literature.[6] In an oven-dried microwave vial, loaded with a Teflon-coated stir bar, copper(I) chloride (25 mg, 250 µmol, 5 mol%), potassium carbonate (138 mg, 1.00 mmol, 20 mol%), tris(*p*-methoxyphenyl)phosphine (88 mg, 250 µmol, 5 mol%), and bis(pinacolato)diboron (1.52 g, 6.00 mmol, 1.2 equiv.) were weighed out. The vial was sealed and purged with N_2-vacuum cycles, and backfilled with N_2. Et_2O (20 mL, 0.25 M) was added, followed by 1-phenyl-1-propyne (626 µL, 5.00 mmol, 1.0 equiv.) and isopropanol (765 µL, 10.0 mmol, 2.0 equiv.). The reaction mixture was stirred for 16 hours at room temperature. Once completion was reached, the reaction mixture was filtered through a pad of celite, the vial was rinsed with DCM (2 × 20 mL), and concentrated *in vacuo*. The crude residue was purified by flash chromatography (silica gel) from pure hexane to a mixture of 2% of diethyl ether in hexane affording 1.13 g of a colourless oil consistent with the desired product (93%).

^{1}H NMR (500 MHz, Chloroform-*d*) δ 7.41 – 7.38 (m, 2H), 7.37 – 7.32 (m, 2H), 7.27 – 7.22 (m, 2H), 2.00 (d, *J* = 1.79 Hz, 3H), 1.32 (s, 12H).

^{11}B NMR (96 MHz, Chloroform-*d*) δ 30.89.

^{13}C NMR (126 MHz, Chloroform-*d*) δ 142.5, 138.1, 129.5, 128.2, 127.2, 83.6, 25.0, 16.0. The boron-bearing carbon is not observed due to quadrupolar relaxation.

Data are consistent with the literature.[70]

(*Z*)-Trifluoro(1-phenylprop-1-en-2-yl)-λ^4-borane, potassium salt, **66-int4**

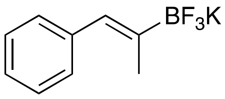

Prepared according to General Procedure 5 using (*Z*)-4,4,5,5-tetramethyl-2-(1-phenylprop-1-en-2-yl)-1,3,2-dioxaborolane, **66-int3** (1.13 g, 4.63 mmol, 1.0 equiv.), potassium hydrogen fluoride (1.45 g, 18.5 mmol, 4.0 equiv.), and water (4.17 mL, 231 mmol, 50.0 equiv.) in MeOH (46 mL, 0.1 M). 845 mg of a white solid was obtained after filtration, consistent with the desired product (81%).

^{1}H NMR (500 MHz, DMSO-d_6) δ 7.28 – 7.24 (m, 2H), 7.19 – 7.15 (m, 2H), 7.10 – 7.05 (m, 1H), 6.38 (s, 1H), 1.71 (d, J = 1.72 Hz, 3H).

^{11}B NMR (96 MHz, DMSO-d_6) δ 3.05.

^{13}C NMR (126 MHz, DMSO-d_6) δ 140.6, 128.5, 127.8, 126.1 (q, $^3J_{CF}$ = 3.1 Hz), 124.6, 16.6. The boron-bearing carbon is not observed due to quadrupolar relaxation.

^{19}F {^1H} NMR (376 MHz, DMSO-d_6) δ –143.51.

IR (solid): 1489, 1446, 1230, 1217, 1192, 1180, 1035, 1020, 958, 937, 920, 844 cm^{-1}.

HRMS (ESI): *m/z* calculated for $[M – K]^-$ $(C_9H_9BF_3)^-$: 185.0754; found = 185.0761.

(*Z*)-(1-Phenylprop-1-en-2-yl)boronic acid, **66**

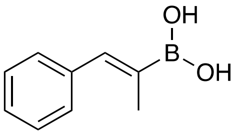

Prepared according to General Procedure 6 using (*Z*)-trifluoro(1-phenylprop-1-en-2-yl)-λ^4-borane, potassium salt, **66-int4** (800 mg, 3.57 mmol, 1.0 equiv.), and chlorotrimethylsilane (1.59 mL, 12.5 mmol, 3.5 equiv.) in MeCN:H_2O (28 mL:7 mL, 0.1 M). 436 mg of a white solid was obtained as a mixture of the desired product and boroxine (75%).

^{1}H NMR (500 MHz, DMSO-d_6) δ 7.67 (s, 2H), 7.38 – 7.34 (m, 2H), 7.33 – 7.30 (m, 2H), 7.25 – 7.21 (m, 1H), 7.12 (d, J = 1.17 Hz, 1H), 1.88 (d, J = 1.80 Hz, 3H).

^{11}B NMR (96 MHz, DMSO-d_6) δ 29.26.

^{13}C NMR (126 MHz, DMSO-d_6) δ 139.0, 138.2, 134.3 (broad), 129.1, 128.3, 126.8, 16.3.

IR (solid): 1610, 1573, 1489, 1446, 1386, 1359, 1328, 1303, 1263, 1203, 1180, 1099, 1076, 927, 792 cm^{-1}.

HRMS (EI): *m/z* calculated for $[M]^+$ $(C_9H_{11}BNO_2)^+$: 162.0846; found = 162.0852.

1,2-Diphenylethyne, **S15-int2**

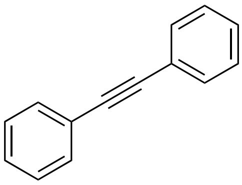

Prepared according to General Procedure 2 using iodobenzene (1.14 mL, 10.0 mmol, 1.0 equiv.), dichlorobis(triphenylphosphine)palladium (70 mg, 100 μmol, 1 mol%), copper iodide (38 mg, 200 μmol, 2 mol%), and phenylacetylene (1.37 mL, 12.5 mmol, 1.25 equiv.) in triethylamine (20 mL, 0.5 M). The crude residue was purified by flash chromatography (silica gel) with pure hexane affording 1.74 g of a yellow solid as the desired product (98%).

^{1}H NMR (400 MHz, Chloroform-*d*) δ 7.60 – 7.53 (m, 4H), 7.43 – 7.34 (m, 6H).

^{13}C NMR (101 MHz, Chloroform-*d*) δ 131.7, 128.5, 128.4, 123.4, 89.5.

Data are consistent with the literature.[71]

(*Z*)-2-(1,2-Diphenylvinyl)-4,4,5,5-tetramethyl-1,3,2-dioxaborolane, **S15-int3**

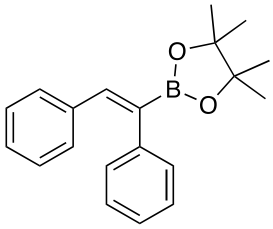

Prepared according to the literature.[6] In an oven-dried microwave vial, loaded with a Teflon-coated stir bar, copper(I) chloride (42 mg, 421 μmol, 5 mol%), potassium carbonate (233 mg, 1.68 mmol, 20 mol%), tris(*p*-methoxyphenyl)phosphine (148 mg, 421 μmol, 5 mol%), and bis(pinacolato)diboron (2.56 g, 10.1 mmol, 1.2 equiv.) were weighed out. The vial was sealed and purged with N_2-vacuum cycles, and backfilled with N_2. Et_2O (34 mL, 0.25 M) was added, followed by 1,2-diphenylethyne, **S15-int2** (1.50 g, 8.42 mmol, 1.0 equiv.), and isopropanol (765 μL, 16.8 mmol, 2.0 equiv.). The reaction mixture was stirred for 16 hours at room temperature. Once completion was reached, the reaction mixture was filtered through a pad of celite and the vial was rinsed with DCM (2 × 20 mL), and concentrated *in vacuo*. The crude residue was purified by flash chromatography (silica gel) from pure hexane to a mixture of 2% of diethyl ether in hexane affording 1.74 g of a white solid consistent with the desired product (68%).

^{1}H NMR (500 MHz, Chloroform-*d*) δ 7.38 (s, 1H), 7.29 – 7.25 (m, 2H), 7.24 – 7.20 (m, 1H), 7.19 – 7.16 (m, 2H), 7.14 – 7.11 (m, 3H), 7.09 – 7.05 (m, 2H), 1.32 (s, 12H).

^{11}B NMR (96 MHz, Chloroform-*d*) δ 30.93.

^{13}C NMR (126 MHz, Chloroform-*d*) δ 143.3, 140.6, 137.1, 130.1, 129.0, 128.4, 128.0, 127.7, 126.4, 83.9, 24.9. The boron-bearing carbon is not observed due to quadrupolar relaxation.

Data are consistent with the literature.[72]

(*Z*)-(1,2-Diphenylvinyl)trifluoro-λ^4-borane, potassium salt, **S15-int4**

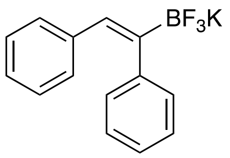

Prepared according to General Procedure 5 using (*Z*)-2-(1,2-diphenylvinyl)-4,4,5,5-tetramethyl-1,3,2-dioxaborolane, **S15-int3** (1.74 g, 5.68 mmol, 1.0 equiv.) potassium hydrogen fluoride (1.78 g, 22.7 mmol, 4.0 equiv.), and water (5.12 mL, 284 mmol, 50.0 equiv.) in MeOH (56 mL, 0.1 M). 1.21 g of a white solid was obtained after filtration, consistent with the desired product (74%).

^{1}H NMR (400 MHz, DMSO-d_6) δ 7.17 – 7.11 (m, 2H), 7.06 – 7.02 (m, 1H), 7.02 – 6.97 (m, 4H), 6.96 – 6.90 (m, 1H), 6.88 – 6.81 (m, 2H), 6.53 (s, 1H).

^{11}B NMR (96 MHz, DMSO-d_6) δ 3.02.

^{13}C NMR (101 MHz, DMSO-d_6) δ 146.3, 139.6, 128.7, 127.7, 127.5, 127.4 (broad), 127.4, 124.9, 124.0. The boron-bearing carbon is not observed due to quadrupolar relaxation.

^{19}F {^{1}H} NMR (377 MHz, DMSO-d_6) δ –140.72.

IR (solid): 1593, 1489, 1446, 1198, 1122, 1016, 1097, 1070, 977, 947 cm^{-1}.

HRMS (ESI): *m/z* calculated for $[M – K]^{–}$ $(C_{14}H_{11}BF_3)^{–}$: 247.0897; found = 247.0913.

Data are consistent with the literature.[73]

(*Z*)-(1,2-Diphenylvinyl)boronic acid, **S15**

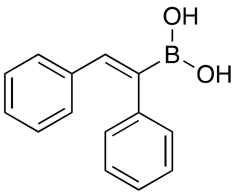

Prepared according to General Procedure 6 using (*Z*)-(1,2-diphenylvinyl)trifluoro-λ^4-borane, potassium salt, **S15-int4** (1.10 g, 3.84 mmol, 1.0 equiv.) and chlorotrimethylsilane (1.71 mL, 13.5 mmol, 3.5 equiv.) in MeCN:H_2O (31 mL:8 mL, 0.1 M). 769 mg of a pale-yellow solid was obtained as a mixture of the desired product and boroxine (89%).

^{1}H NMR (400 MHz, Acetone-d_6) δ 7.38 (s, 1H), 7.33 – 7.25 (m, 3H), 7.23 – 7.18 (m, 1H), 7.13 – 7.09 (m, 4H), 7.04 – 6.99 (m, 2H), 6.86 (s, 2H).

^{11}B NMR (96 MHz, Acetone-d_6) δ 28.48.

^{13}C NMR (101 MHz, Acetone-d_6) δ 143.0, 141.1, 138.3, 130.6, 129.4, 129.2, 128.7, 128.1, 126.8. The boron-bearing carbon is not observed due to quadrupolar relaxation.

IR (solid): 3192, 2260, 1678, 1597, 1444, 1406, 1282, 1192, 1074, 997 cm^{-1}.

HRMS: Desired mass not found due to fragmentation/instability.

3,4-Dihydronaphthalen-2-yl trifluoromethanesulfonate, **S16-int0**

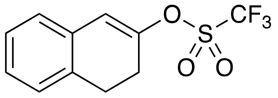

In a flame-dried two neck flask, loaded with a Teflon-coated stir bar, potassium *tert*-butoxide (842 mg, 7.50 mmol, 1.5 equiv.) was weighed out. The flask was purged with vacuum-N_2 and backfilled with N_2. THF (30 mL) was added and the reaction mixture was cooled down to 0 °C. β-Tetralone (731 mg, 5.00 mmol, 1.0 equiv.) was dissolved in THF (10 mL) and added dropwise at 0 °C. The reaction mixture was left to stir for one hour. Finally, *N*-phenylbis(trifluoromethanesulphonimide) (2.14 g, 6.00 mmol, 1.2 equiv.) was dissolved in THF (10 mL) and added dropwise at 0 °C. The reaction was left to stir for four hours. Once completion was reached, the reaction mixture was concentrated *in vacuo*. The crude residue was partitioned between brine (30 mL) and diethyl ether (30 mL). Organics were extracted with diethyl ether (2 × 15 mL). Organic layers were combined, washed with brine (30 mL), dried over sodium sulfate, filtered, and concentrated *in vacuo*. The crude residue was purified by flash chromatography (silica gel) from pure hexane to mixture of 2% of diethyl ether in hexane affording 1.38 g of a colourless oil consistent with the desired product (99%).

^{1}H NMR (500 MHz, Chloroform-*d*) δ 7.23 – 7.19 (m, 2H), 7.17 – 7.14 (m, 1H), 7.11 – 7.07 (m, 1H), 6.49 (t, J = 1.35 Hz, 1H), 3.07 (t, J = 8.38 Hz, 2H), 2.70 (td, J = 8.41, 1.30 Hz, 2H).

^{13}C NMR (126 MHz, Chloroform-*d*) δ 150.1, 133.1, 131.2, 128.6, 127.7, 127.5, 127.2, 118.7 (q, $^{1}J_{CF}$ = 320.7 Hz), 118.7, 28.7, 26.7.

^{19}F {^{1}H} NMR (470 MHz, Chloroform-*d*) δ –73.56.

Data are consistent with the literature.[74]

2-(3,4-Dihydronaphthalen-2-yl)-4,4,5,5-tetramethyl-1,3,2-dioxaborolane, **S16-int3**

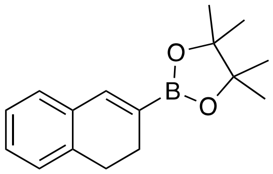

Prepared according to the literature.[64] In an oven-dried microwave vial, charged with a Teflon-coated stir bar, 3,4-dihydronaphthalen-2-yl trifluoromethanesulfonate, **S16-int0** (1.20 g, 4.31 mmol, 1.0 equiv.), triphenylphosphine (68 mg, 259 µmol, 6 mol%), bis(triphenylphosphine)palladium (II) chloride (91 mg, 129 µmol, 3 mol%), bis(pinacolato)diboron (1.20 g, 4.74 mmol, 1.1 equiv.), and potassium acetate (1.27 g, 12.9 mmol, 3.0 equiv.). The vial was sealed and purged with vacuum-N_2 and backfilled with N_2. Dry and degassed 1,4-dioxane (24 mL, 0.2 M) was added and the reaction mixture was stirred at 60 °C overnight. Once completion was reached, the reaction mixture was concentrated *in vacuo*. The crude residue was partitioned between brine (20 mL) and diethyl ether (20 mL). Organics were extracted with diethyl ether (2 × 20 mL). Organic layers were combined, washed with brine (20 mL), dried over sodium sulfate, filtered, and concentrated *in vacuo*. The crude residue was purified by flash chromatography (silica gel) from pure hexane to mixture of 4% of diethyl ether in hexane affording 944 mg of a colourless oil consistent with the desired product (85%).

^{1}H NMR (500 MHz, Chloroform-*d*) δ 7.22 (t, J = 1.83 Hz, 1H), 7.18 – 7.15 (m, 2H), 7.14 – 7.09 (m, 2H), 2.76 (t, J = 8.13 Hz, 2H), 2.41 (td, J = 8.17, 1.79 Hz, 2H), 1.32 (s, 12H).

^{11}B NMR (96 MHz, Chloroform-*d*) δ 30.85.

^{13}C NMR (126 MHz, Chloroform-*d*) δ 140.6, 137.3, 133.9, 128.1, 127.6, 127.1, 126.5, 83.5, 27.5, 25.0, 24.2. The boron-bearing carbon is not observed due quadrupolar relaxation.

Data are consistent with the literature.[75]

(3,4-Dihydronaphthalen-2-yl)trifluoro-λ⁴-borane, potassium salt, **S16-int4**

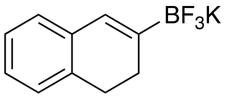

Prepared according to General Procedure 5 using 2-(3,4-dihydronaphthalen-2-yl)-4,4,5,5-tetramethyl-1,3,2-dioxaborolane, **S16-int3** (900 mg, 3.51 mmol, 1.0 equiv.), potassium hydrogen fluoride (1.10 g, 14.1 mmol, 4.0 equiv.), and water (3.16 mL, 176 mmol, 50.0 equiv.) in MeOH (35 mL, 0.1 M). 760 mg of a white solid was obtained after filtration, consistent with the desired product (92%).

^{1}H NMR (500 MHz, DMSO-d_6) δ 7.03 (td, *J* = 7.32, 1.59 Hz, 1H), 7.01 – 6.97 (m, 1H), 6.94 (td, *J* = 7.28, 1.38 Hz, 1H), 6.86 (dd, *J* = 7.40, 1.36 Hz, 1H), 6.31 (s, 1H), 2.53 (t, *J* = 8.11 Hz, 2H), 2.10 (td, *J* = 7.72, 1.46 Hz, 2H).

^{11}B NMR (96 MHz, DMSO-d_6) δ 2.47.

^{13}C NMR (126 MHz, DMSO-d_6) δ 151.3 (broad), 136.2, 135.2, 126.9, 126.0, 124.8 (q, $^{3}J_{CF}$ = 3.3 Hz), 124.8, 124.5, 27.7, 25.4.

^{19}F {^{1}H} NMR (377 MHz, DMSO-d_6) δ –142.59.

IR (solid): 1624, 1485, 1448, 1265, 1240, 1197, 1180, 1166, 1103, 999, 966 cm^{-1}.

HRMS (ESI): *m/z* calculated for $[M – K]^-$ $(C_{10}H_9BF_3)^-$: 197.0754; found = 197.0755.

(3,4-Dihydronaphthalen-2-yl)boronic acid, **S16**

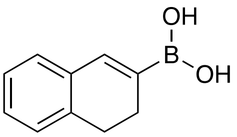

Prepared according to General Procedure 6 using (3,4-dihydronaphthalen-2-yl)trifluoro-λ⁴-borane, potassium salt, **S16-int4** (650 mg, 2.75 mmol, 1.0 equiv.) and chlorotrimethylsilane (1.22 mL, 9.64 mmol, 3.5 equiv.) in MeCN:H_2O (22 mL:6 mL, 0.1 M). 422 mg of a white solid as was obtained as a mixture of the desired product and boroxine (88%).

^{1}H NMR (400 MHz, DMSO-d_6) δ 7.69 (s, 2H), 7.19 – 7.12 (m, 3H), 7.11 (t, *J* = 1.45 Hz, 1H), 7.08 – 7.05 (m, 1H), 2.63 (t, *J* = 8.25 Hz, 2H), 2.27 (td, *J* = 8.10, 1.46 Hz, 2H).

^{11}B NMR (96 MHz, DMSO-d_6) δ 26.50.

^{13}C NMR (101 MHz, DMSO-d_6) δ 137.2, 136.4, 135.7 (broad), 134.0, 127.5, 127.3, 126.5, 126.3, 27.1, 24.3.

IR (solid): 2929, 1612, 1566, 1450, 1371, 1313, 1282, 1209, 1157, 1112, 992, 904 cm^{-1}.

HRMS (ESI): *m/z* calculated for $[M – H]^-$ $(C_{10}H_{10}BO_2)^-$: 173.0779; found = 173.0775.

Data are consistent with the literature.[76]

(E)-4-(2-(4,4,5,5-Tetramethyl-1,3,2-dioxaborolan-2-yl)vinyl)pyridine, **S17-int3**

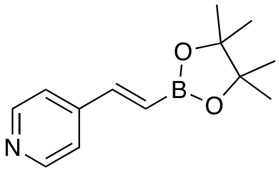

Prepared according to General Procedure 4 using 4-ethynylpyridine (516 mg, 5.00 mmol, 1.0 equiv.), copper(I) chloride (25 mg, 250 µmol, 5 mol%), potassium *tert*-butoxide (56 mg, 500 µmol, 10 mol%), bis(2-diphenylphosphinophenyl) ether (DPEPhos) (135 mg, 250 µmol, 5 mol%), bis(pinacolato)diboron (1.40 g, 5.50 mmol, 1.1 equiv.), and MeOH (405 µL, 10.0 mmol, 2.0 equiv.) in THF (20 mL, 0.25 M). 1.05 g of a brown solid was afforded consistent with the desired product (91%). It contained B_2Pin_2 or related adducts.

^{1}H NMR (500 MHz, Chloroform-*d*) δ 8.53 – 8.50 (m, 2H), 7.29 – 7.26 (m, 2H), 7.24 (d, J = 18.37 Hz, 1H), 6.32 (d, J = 18.41 Hz, 1H), 1.25 (s, 12H).

^{11}B NMR (128 MHz, Chloroform-*d*) δ 29.89.

^{13}C NMR (126 MHz, Chloroform-*d*) δ 150.0, 146.4, 144.8, 122.3 (broad), 121.3, 83.8, 24.8.

Data are consistent with the literature.[77]

(E)-4-(2-(Trifluoro-λ^4-boraneyl)vinyl)pyridine, potassium salt, **S17-int4**

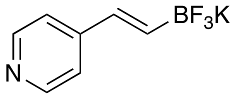

Prepared according to General Procedure 5 using (E)-4-(2-(4,4,5,5-tetramethyl-1,3,2-dioxaborolan-2-yl)vinyl)pyridine, **S17-int3** (800 mg, 3.46 mmol, 1.0 equiv.), potassium hydrogen fluoride (1.08 g, 13.8 mmol, 4.0 equiv.), and water (3.12 mL, 173 mmol, 50.0 equiv.) in MeOH (32 mL, 0.1 M). 220 mg of a pale-brown solid was obtained after filtration, consistent with the desired product (30%).

^{1}H NMR (500 MHz, DMSO-d_6) δ 8.42 – 8.37 (m, 2H), 7.29 – 7.25 (m, 2H), 6.52 (dq, J = 18.22, 3.31 Hz, 1H), 6.44 (d, J = 18.30 Hz, 1H).

^{11}B NMR (128 MHz, DMSO-d_6) δ 2.34.

^{13}C NMR (126 MHz, DMSO-d_6) δ 149.6, 147.2, 145.6 (broad), 131.1 (q, $^3J_{CF}$ = 4.5 Hz), 120.3.

^{19}F {^{1}H} NMR (377 MHz, DMSO-d_6) δ –138.51.

IR (solid): 1604, 1548, 1425, 1344, 1257, 1238, 1124, 1089, 995, 970 cm^{-1}.

HRMS (ESI): *m/z* calculated for $[M – K]^–$ $(C_7H_6BF_3N)^–$: 172.0551; found = 172.0550.

(E)-(2-(Pyridin-4-yl)vinyl)boronic acid, **S17**

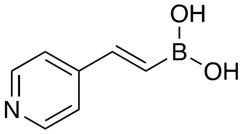

Prepared according to General Procedure 5 using (*E*)-4-(2-(trifluoro-λ^4-boraneyl)vinyl)pyridine, potassium salt, **S17-int4** (200 mg, 948 μmol, 1.0 equiv.) and chlorotrimethylsilane (421 μL, 3.32 mmol, 3.5 equiv.) in MeCN:H_2O (8 mL:2 mL, 0.1 M). Aqueous layer was concentrated *in vacuo* affording 183 mg of a brown solid as the desired product (99%).

^{1}H NMR (400 MHz, DMSO-d_6) δ 8.92 – 8.83 (m, 2H), 8.18 – 8.03 (m, 2H), 7.40 (d, *J* = 18.37 Hz, 1H), 6.79 (d, *J* = 18.35 Hz, 1H).

^{11}B NMR (96 MHz, DMSO-d_6) δ 26.10.

^{13}C NMR (101 MHz, DMSO-d_6) δ 153.4, 141.9, 140.5, 137.1 (broad), 124.0.

IR (solid): 1633, 1591, 1496, 1442, 1406, 1344, 1321, 1294, 1228, 1190, 1116, 936 cm^{-1}.

HRMS (ESI): *m/z* calculated for $[M + H]^+$ $(C_7H_9BNO_2)^+$: 150.0721; found = 150.0720.

5-((Trimethylsilyl)ethynyl)pyrimidine, **S18-int1**

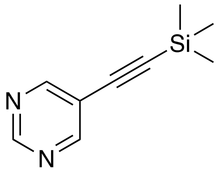

Prepared according to General Procedure 2 using 5-bromopyrimidine (954 mg, 6.00 mmol, 1.0 equiv.), dichlorobis(triphenylphosphine)palladium (42 mg, 60.0 μmol, 1 mol%), copper iodide (23 mg, 120 μmol, 2 mol%), and trimethylsilylacetylene (6.34 mL, 60.0 mmol, 10 equiv.) in triethylamine (12 mL, 0.5 M) at 90 °C. The crude residue was purified by flash chromatography (silica gel) from pure hexane to a mixture of 5% of diethyl ether in hexane affording 1.05 g of a pale-brown oil as the desired product (99%).

^{1}H NMR (500 MHz, Chloroform-*d*) δ 9.10 (s, 1H), 8.76 (s, 2H), 0.25 (s, 9H).

^{13}C NMR (126 MHz, Chloroform-*d*) δ 159.2, 156.8, 119.9, 102.9, 97.7, –0.2.

IR (film): 2164, 1541, 1408, 1246, 1184, 908, 860, 840 cm^{-1}.

HRMS (ESI): *m/z* calculated for $[M + H]^+$ $(C_9H_{13}N_2Si)^+$: 177.0843; found = 177.0842.

5-Ethynylpyrimidine, **S18-int2**

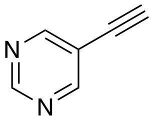

Prepared according to General Procedure 3 using 5-((trimethylsilyl)ethynyl)pyrimidine, **S18-int1** (1.00 g, 5.67 mmol, 1.0 equiv.) and potassium carbonate (1.57 g, 11.3 mmol, 2.0 equiv.) in MeOH (28 mL, 0.2 M). Organics layers were combined, washed with water (20 mL), dried over sodium sulfate, filtered, and concentrated *in vacuo* affording 515 mg of a beige solid consistent with the desired product (87%).

^{1}H NMR (500 MHz, Chloroform-*d*) δ 9.16 (s, 1H), 8.82 (s, 2H), 3.40 (s, 1H).

^{13}C NMR (126 MHz, Chloroform-*d*) δ 159.5, 157.4, 118.9, 84.6, 77.0.

Data are consistent with the literature.[78]

(*E*)-5-(2-(4,4,5,5-Tetramethyl-1,3,2-dioxaborolan-2-yl)vinyl)pyrimidine, **S18-int3**

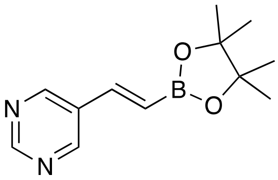

Prepared according to General Procedure 4 using 5-ethynylpyrimidine, **S18-int2** (800 mg, 7.68 mmol, 1.0 equiv.), copper(I) chloride (38 mg, 384 µmol, 5 mol%), potassium *tert*-butoxide (86 mg, 768 µmol, 10 mol%), bis(2-diphenylphosphinophenyl)ether (DPEPhos) (207 mg, 384 µmol, 5 mol%), bis(pinacolato)diboron (2.15 g, 8.45 mmol, 1.1 equiv.), and MeOH (622 µL, 15.4 mmol, 2.0 equiv.) in THF (30 mL, 0.25 M). The crude residue was purified by flash chromatography (silica gel) from pure hexane to a mixture of 25% of ethyl acetate in hexane affording 1.39 g of a white solid consistent with the desired product (78%). It contained B_2Pin_2 or related adducts.

^{1}H NMR (500 MHz, Chloroform-*d*) δ 9.02 (s, 1H), 8.73 (s, 2H), 7.20 (d, *J* = 18.60 Hz, 1H), 6.25 (d, *J* = 18.60 Hz, 1H), 1.22 (s, 12H).

^{11}B NMR (96 MHz, Chloroform-*d*) δ 29.67.

^{13}C NMR (126 MHz, Chloroform-*d*) δ 158.1, 154.8, 141.8, 130.8, 122.0 (broad), 83.8, 24.7.

Data are consistent with the literature.[58]

(*E*)-5-(2-(trifluoro-λ^4-boraneyl)vinyl)pyrimidine, potassium salt, **S18-int4**

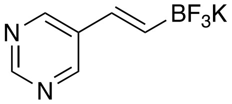

Prepared according to General Procedure 5 using (*E*)-5-(2-(4,4,5,5-tetramethyl-1,3,2-dioxaborolan-2-yl)vinyl)pyrimidine, **S18-int3** (1.35 g, 5.82 mmol, 1.0 equiv.), potassium hydrogen fluoride (1.82 g, 23.3 mmol, 4.0 equiv.), and water (5.24 mL, 291 mmol, 50.0 equiv.) in MeOH (54 mL, 0.1 M). 232 mg of a white solid was obtained after filtration, consistent with the desired product (19%).

^{1}H NMR (500 MHz, Acetone-d_6) δ 8.85 (s, 1H), 8.68 (s, 2H), 6.61 – 6.55 (m, 2H).

^{11}B NMR (96 MHz, Acetone-d_6) δ 2.61.

^{13}C NMR (126 MHz, Acetone-d_6) δ 156.7, 154.3, 134.7, 127.0 (q, $^3J_{CF}$ = 4.5 Hz). The boron-bearing carbon is not observed due to quadrupolar relaxation.

^{19}F {^1H} NMR (377 MHz, Acetone-d_6) δ –142.74.

IR (solid): 1564, 1450, 1406, 1261, 1232, 1165, 1124, 1076, 991, 952 cm^{-1}.

HRMS (ESI): *m/z* calculated for $[M - K]^-$ $(C_6H_5BF_3N_2)^-$: 173.0503; found = 173.0501.

(*E*)-(2-(Pyrimidin-5-yl)vinyl)boronic acid, **S18**

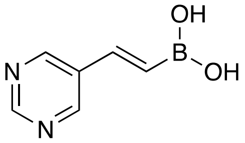

Prepared according to General Procedure 6 using (*E*)-5-(2-(trifluoro-λ^4-boraneyl)vinyl)pyrimidine, potassium salt, **S18-int4** (128 mg, 604 µmol, 1.0 equiv.) and chlorotrimethylsilane (268 µL, 2.11 mmol, 3.5 equiv.) in MeCN:H_2O (5 mL:1 mL, 0.1 M). 82 mg of a white solid was obtained as the desired product (91%).

^1H NMR (500 MHz, DMSO-d_6) δ 9.11 (s, 1H), 8.94 (s, 2H), 7.23 (d, *J* = 18.58 Hz, 1H), 6.40 (d, *J* = 18.59 Hz, 1H), 5.88 (broad s, 2H).

^{11}B NMR (96 MHz, DMSO-d_6) δ 28.54.

^{13}C NMR (126 MHz, DMSO-d_6) δ 157.3, 154.7, 138.8, 131.1, 128.9 (broad).

IR (solid): 3209, 1629, 1581, 1521, 1431, 1382, 1361, 1340, 1265, 1251, 1155, 1134, 1097, 997 cm^{-1}.

HRMS (ESI): *m/z* calculated for $[M + H]^+$ $(C_6H_8BN_2O_2)^+$: 151.0673; found = 151.0675.

(*E*)-4,4,5,5-Tetramethyl-2-styryl-1,3,2-dioxaborolane, **1b**

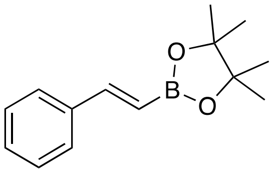

Prepared according to General Procedure 4 using phenylacetylene (982 µL, 10.0 mmol, 1.0 equiv.), copper(I) chloride (49 mg, 500 µmol, 5 mol%), potassium *tert*-butoxide (112 mg, 1.00 mmol, 10 mol%), bis(2-diphenylphosphinophenyl)ether (DPEPhos) (269 mg, 500 µmol, 5 mol%), bis(pinacolato)diboron (2.79 g, 11.0 mmol, 1.1 equiv.), and MeOH (809 µL, 20.0 mmol, 2.0 equiv.) in THF (40 mL, 0.25 M). The crude residue was purified by flash chromatography (silica gel) from pure hexane to a mixture of 2% of diethyl ether in hexane affording 2.10 g of a pale-yellow oil consistent with the desired product (91%).

^1H NMR (500 MHz, Chloroform-*d*) δ 7.51 – 7.48 (m, 2H), 7.41 (d, *J* = 18.45 Hz, 1H), 7.36 – 7.32 (m, 2H), 7.31 – 7.27 (m, 1H), 6.18 (d, *J* = 18.42 Hz, 1H), 1.32 (s, 12H).

^{11}B NMR (96 MHz, Chloroform-*d*) δ 30.21.

^{13}C NMR (126 MHz, Chloroform-*d*) δ 149.6, 137.6, 129.0, 128.7, 127.2, 116.5 (broad), 83.5, 24.9.

Data are consistent with the literature.[79]

(*Z*)-4,4,5,5-Tetramethyl-2-styryl-1,3,2-dioxaborolane, **S19-int3cis**

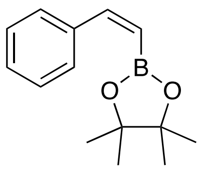

Prepared according the literature.[69] In an oven-dried microwave vial, loaded with a Teflon-coated stir bar, (*E*)-4,4,5,5-tetramethyl-2-styryl-1,3,2-dioxaborolane, **1b** (1.00 g, 4.35 mmol, 1.0 equiv.) and tris(2-phenylpyridine)iridium (28.5 mg, 43.5 μmol, 1 mol%) were weighed out. The vial was sealed, purged with vacuum-N_2 cycles (3 times), and backfilled with N_2. Degassed dry MeCN (21 mL, 0.2 M) was then added. The reaction mixture was stirred overnight under blue LEDs under N_2. The reaction mixture was worked-up: it was partitioned between diethyl ether (10 mL) and brine (10 mL). Organics were extracted with diethyl ether (2 × 15 mL). Organics were combined, washed with brine (20 mL), dried over sodium sulfate, filtered, and concentrated *in vacuo*. The crude residue was purified by flash chromatography (silica gel) from hexane to a mixture of 4% of diethyl ether in hexane affording 920 mg of a pale-yellow oil consistent with a mixture of *E* and *Z* of the desired product (92%, 0.74:0.26 *Z:E*).

^{1}H NMR (500 MHz, Chloroform-*d*) δ 7.56 – 7.52 (m, 2H), 7.36 – 7.25 (m, 3H), 7.22 (d, *J* = 15.01 Hz, 1H), 5.60 (d, *J* = 14.85 Hz, 1H), 1.30 (s, 12H).

^{11}B NMR (96 MHz, Chloroform-*d*) δ 30.19.

^{13}C NMR (126 MHz, Chloroform-*d*) δ 148.3, 138.6, 128.7, 128.1, 128.1, 119.5 (broad), 83.6, 24.9.

Data are consistent with the literature.[80]

(*E*) and (*Z*)-Trifluoro(styryl)-λ^4-borane, potassium salt, **S19-int4**

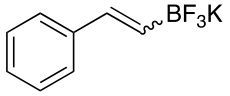

Prepared according to General Procedure 5 using the mixture of (*E*) and (*Z*)-4,4,5,5-tetramethyl-2-styryl-1,3,2-dioxaborolane, **S19-int3cis** (770 mg, 3.35 mmol, 1.0 equiv.), potassium hydrogen fluoride (1.05 g, 13.4 mmol, 4.0 equiv.), and water (3.01 mL, 167 mmol, 50.0 equiv.) in MeOH (30 mL, 0.1 M). 262 mg of a white solid was obtained after filtration, consistent with a mixture of *E* and *Z* of the desired product (37%, 1:0.95 *Z:E*).

(*Z*)-Trifluoro(styryl)-λ^4-borane, potassium salt

^{1}H NMR (500 MHz, DMSO-d_6) δ 7.59 – 7.54 (m, 2H), 7.18 (app. t, *J* = 7.68 Hz, 2H), 7.08 – 7.05 (m, 1H), 6.45 (d, *J* = 15.42 Hz, 1H), 5.58 (dq, *J* = 15.17, 6.45 Hz, 1H).

^{11}B NMR (96 MHz, DMSO-d_6) δ 2.47.

^{13}C NMR (126 MHz, DMSO-d_6) δ 143.2 (broad), 140.7, 135.0 (q, $^3J_{CF}$ = 5.4 Hz), 128.4 (q, $^5J_{CF}$ = 2.8 Hz), 127.3, 125.4.

^{19}F {^{1}H} NMR (376 MHz, DMSO-d_6) δ –137.79.

Data are consistent with the literature.[81,82]

(*E*)-Trifluoro(styryl)-λ^4-borane, potassium salt, **(1d)**

^{1}H NMR (500 MHz, DMSO-d_6) δ 7.33 – 7.29 (m, 2H), 7.25 (app. t, J = 7.65 Hz, 2H), 7.13 – 7.09 (m, 1H), 6.47 (d, J = 18.17 Hz, 1H), 6.19 (dq, J = 18.23, 3.56 Hz, 1H).

^{11}B NMR (96 MHz, DMSO-d_6) δ 2.47.

^{13}C NMR (126 MHz, DMSO-d_6) δ 140.3, 138.9 (broad), 133.1 (q, $^{3}J_{CF}$ = 4.5 Hz), 128.3, 125.9, 125.4.

^{19}F {^{1}H} NMR (376 MHz, DMSO-d_6) δ –132.84.

Data are consistent with the literature.[83]

(*E*) and (*Z*)-Styrylboronic acid, **S19**

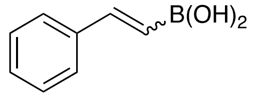

Prepared according the literature.[81] The mixture of (*E*) and (*Z*)-trifluoro(styryl)-λ^4-borane, potassium salt, **S19-int4** (200 mg, 952 µmol, 1.0 equiv.) was dissolved in water (10 mL, 0.1 M). Silica gel (400 mg) was added and the reaction was left to stir for three hours at room temperature. The organics were extracted with diethyl ether (3 × 10 mL). Organic layers were combined, washed with brine (15 mL), dried over sodium sulfate, filtered, and concentrated *in vacuo* affording 126 mg of a white solid consistent with a mixture of *E* and *Z* of the desired product (89%, 1:1.1 *Z:E*).

(*Z*)-Styrylboronic acid, **S19**

^{1}H NMR (500 MHz, Acetone-d_6) δ 7.49 – 7.46 (m, 2H), 7.33 – 7.30 (m, 2H), 7.26 – 7.19 (m, 1H), 6.98 (s, 2H), 6.94 (d, J = 14.78 Hz, 1H), 5.69 (d, J = 14.95 Hz, 1H).

^{11}B NMR (96 MHz, Acetone-d_6) δ 29.44.

^{13}C NMR (126 MHz, Acetone-d_6) δ 142.6, 140.1, 129.3, 128.6, 128.2. The boron-bearing carbon is not observed due to quadrupolar relaxation.

Data are consistent with the literature.[84]

(*E*)-Styrylboronic acid, **1a**

^{1}H NMR (500 MHz, Acetone-d_6) δ 7.52 – 7.49 (m, 2H), 7.39 (d, J = 18.42 Hz, 1H), 7.37 – 7.33 (m, 2H), 7.30 – 7.27 (m, 1H), 6.90 (s, 2H), 6.23 (d, J = 18.36 Hz, 1H).

^{11}B NMR (96 MHz, Acetone-d_6) δ 29.44.

^{13}C NMR (126 MHz, Acetone-d_6) δ 147.5, 138.9, 129.5, 129.0, 127.6. The boron-bearing carbon is not observed due to quadrupolar relaxation.

Data are consistent with the literature.[85]

6.3 Products from the developed coupling reaction

(*E*)-1-Fluoro-4-(4-phenylbut-3-en-1-yl)benzene, **3**

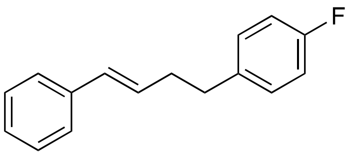

Prepared according to General Procedure 7 using 1,3-dioxoisoindolin-2-yl 3-(4-fluorophenyl)propanoate, **2** (62.7 mg, 200 μmol, 1.0 equiv.), (*E*)-2-phenylvinylboronic acid (59.2 mg, 400 μmol, 2.0 equiv.), tris(2,2'-bipyridine)ruthenium hexafluorophosphate (1.7 mg, 2.00 μmol, 1 mol%), and *N,N*-dimethylaniline (2.5 μL, 20.0 μmol, 10 mol%) in DMSO-d_6 (1 mL, 0.2 M). The crude residue was purified by flash chromatography (silica gel) with pure hexane affording 35.0 mg of a white solid as the desired product (77%, *E:Z* > 20:1).

^{1}H NMR (400 MHz, Chloroform-*d*) δ 7.36 – 7.27 (m, 4H), 7.24 – 7.19 (m, 1H), 7.19 – 7.12 (m, 2H), 7.05 – 6.92 (m, 2H), 6.45 – 6.37 (m, 1H), 6.24 (dd, *J* = 15.84, 6.83 Hz, 1H), 2.95 – 2.68 (m, 2H), 2.65 – 2.41 (m, 2H).

^{13}C NMR (126 MHz, Chloroform-*d*) δ 161.8 (d, $^1J_{CF}$ = 243.4 Hz), 138.1, 137.8 (d, $^4J_{CF}$ = 3.3 Hz), 131.1, 130.3 (d, $^3J_{CF}$ = 7.7 Hz), 130.1, 129.1, 127.6, 126.5, 115.6 (d, $^2J_{CF}$ = 21.0 Hz), 35.6, 35.5.

^{19}F {^{1}H} NMR (376 MHz, Chloroform-*d*) δ –117.66.

IR (film): 3026, 2926, 2360, 1600, 1508, 1448, 1220, 908, 732 cm^{-1}.

HRMS (EI): *m/z* calculated for $[M]^+$ ($C_{16}H_{15}F$): 226.1152; found 226.1147.

(*E*)-(2-Cyclohexylvinyl)benzene, **5**

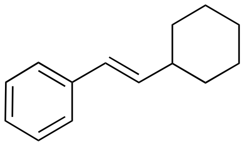

Prepared according to General Procedure 7 using 1,3-dioxoisoindolin-2-yl cyclohexanecarboxylate, **4** (54.7 mg, 200 μmol, 1.0 equiv.), (*E*)-2-phenylvinylboronic acid, **1a** (59.2 mg, 400 μmol, 2.0 equiv.), tris(2,2'-bipyridine)ruthenium hexafluorophosphate (1.7 mg, 2.00 μmol, 1 mol%), and *N,N*-dimethylaniline (2.5 μL, 20.0 μmol, 10 mol%) in DMSO-d_6 (1 mL, 0.2 M). The crude residue was purified by flash chromatography (silica gel) with pure hexane affording 30.1 mg of a colourless oil as the desired product (81%, *E:Z* > 20:1).

^{1}H NMR (500 MHz, Chloroform-*d*) δ 7.37 – 7.33 (m, 2H), 7.31 – 7.27 (m, 2H), 7.21 – 7.16 (m, 1H), 6.35 (d, *J* = 16.01 Hz, 1H), 6.18 (dd, *J* = 15.98, 6.96 Hz, 1H), 2.18 – 2.08 (m, 1H), 1.86 – 1.74 (m, 4H), 1.72 – 1.65 (m, 1H), 1.39 – 1.25 (m, 2H), 1.25 – 1.14 (m, 3H).

^{13}C NMR (126 MHz, Chloroform-*d*) δ 138.2, 137.0, 128.6, 127.3, 126.9, 126.1, 41.3, 33.1, 26.3, 26.2.

Data are consistent with the literature.[86]

(*E*)-(2-Cyclobutylvinyl)benzene, **6**

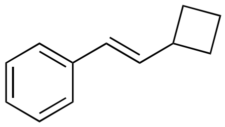

Prepared according to General Procedure 7 using 1,3-dioxoisoindolin-2-yl cyclobutanecarboxylate, **N1** (49 mg, 200 μmol, 1.0 equiv.), (*E*)-2-phenylvinylboronic acid, **1a** (59.2 mg, 400 μmol, 2.0 equiv.), tris(2,2'-bipyridine)ruthenium hexafluorophosphate (1.7 mg, 2.00 μmol, 1 mol%), and *N,N*-dimethylaniline (2.5 μL, 20.0 μmol, 10 mol%) in DMSO-d_6 (1 mL, 0.2 M). The crude residue was purified by flash chromatography (silica gel) with pure hexane affording 21.4 mg of a colourless oil as the desired product (68%, *E:Z* > 20:1).

^{1}H NMR (500 MHz, Chloroform-*d*) δ 7.39 – 7.35 (m, 2H), 7.34 – 7.28 (m, 2H), 7.22 – 7.18 (m, 1H), 6.39 – 6.27 (m, 2H), 3.17 – 3.07 (m, 1H), 2.25 – 2.14 (m, 2H), 2.03 – 1.91 (m, 3H), 1.91 – 1.79 (m, 1H).

^{13}C NMR (176 MHz, Chloroform-*d*) δ 137.9, 135.4, 128.6, 127.7, 126.9, 126.1, 38.9, 28.9, 18.7.

Data are consistent with the literature.[87]

(*E*)-(2-Cyclopentylvinyl)benzene, **7**

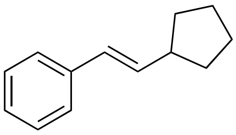

Prepared according to General Procedure 7 using 1,3-dioxoisoindolin-2-yl cyclopentanecarboxylate, **N2** (51.9 mg, 200 μmol, 1.0 equiv.), (*E*)-2-phenylvinylboronic acid, **1a** (59.2 mg, 400 μmol, 2.0 equiv.), tris(2,2'-bipyridine)ruthenium hexafluorophosphate (1.7 mg, 2.00 μmol, 1 mol%), and *N,N*-dimethylaniline (2.5 μL, 20.0 μmol, 10 mol%) in DMSO-d_6 (1 mL, 0.2 M). The crude residue was purified by flash chromatography (silica gel) with pure hexane affording 23.9 mg of a colourless oil as the desired product (70%, *E:Z* = 20:1).

^{1}H NMR (500 MHz, Chloroform-*d*) δ 7.40 – 7.32 (m, 2H), 7.33 – 7.26 (m, 2H), 7.25 – 7.15 (m, 1H), 6.38 (dd, *J* = 15.83, 0.98 Hz, 1H), 6.22 (dd, *J* = 15.79, 7.81 Hz, 1H), 2.67 – 2.56 (m, 1H), 1.95 – 1.81 (m, 2H), 1.77 – 1.68 (m, 2H), 1.68 – 1.56 (m, 2H), 1.47 – 1.32 (m, 2H).

^{13}C NMR (126 MHz, Chloroform-*d*) δ 138.1, 135.8, 128.6, 128.0, 126.8, 126.0, 44.0, 33.4, 25.4.

Data are consistent with the literature.[87]

(*E*)-Styrylcycloheptane, **8**

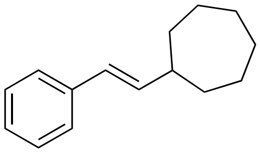

Prepared according to General Procedure 7 using 1,3-Dioxoisoindolin-2-yl cycloheptanecarboxylate, **N3** (57.5 mg, 200 μmol, 1.0 equiv.), (*E*)-2-phenylvinylboronic acid (59.2 mg, 400 μmol, 2.0 equiv.), tris(2,2'-bipyridine)ruthenium hexafluorophosphate (1.7 mg, 2.00 μmol, 1 mol%), and *N,N*-dimethylaniline (2.5 μL, 20.0 μmol, 10 mol%) in DMSO-d_6 (1 mL, 0.2 M). The crude residue was purified by flash

chromatography (silica gel) with pure hexane affording 28.2 mg of a colourless oil as the desired product (71%, *E:Z* > 20:1).

^{1}H NMR (500 MHz, Chloroform-*d*) δ 7.39 – 7.33 (m, 2H), 7.33 – 7.26 (m, 2H), 7.23 – 7.16 (m, 1H), 6.34 (d, *J* = 15.93 Hz, 1H), 6.24 (dd, *J* = 15.87, 7.53 Hz, 1H), 2.39 – 2.30 (m, 1H), 1.88 – 1.81 (m, 2H), 1.76 – 1.69 (m, 2H), 1.67 – 1.60 (m, 2H), 1.59 – 1.40 (m, 6H).

^{13}C NMR (126 MHz, Chloroform-*d*) δ 138.2, 137.8, 128.6, 126.8 (2C), 126.1, 43.4, 34.9, 28.5, 26.4.

Data are consistent with the literature.[88]

(*E*)-(3,3-Dimethylbut-1-en-1-yl)benzene, **9**

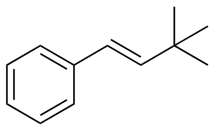

Prepared according to General Procedure 7 using 1,3-dioxoisoindolin-2-yl pivalate, **N4** (49.4 mg, 200 µmol, 1.0 equiv.), (*E*)-2-phenylvinylboronic acid, **1a** (59.2 mg, 400 µmol, 2.0 equiv.), tris(2,2'-bipyridine)ruthenium hexafluorophosphate (1.7 mg, 2.00 µmol, 1 mol%), and *N,N*-dimethylaniline (2.5 µL, 20.0 µmol, 10 mol%) in DMSO-d_6 (1 mL, 0.2 M). The crude residue was purified by flash chromatography (silica gel) with pure hexane affording 14.8 mg of a colourless oil as the desired product (47%, *E:Z* > 20:1).

^{1}H NMR (500 MHz, Chloroform-*d*) δ 7.42 – 7.37 (m, 2H), 7.32 (s, 2H), 7.24 – 7.19 (m, 1H), 6.41 – 6.27 (m, 2H), 1.15 (s, 9H).

^{13}C NMR (126 MHz, Chloroform-*d*) δ 142.0, 138.2, 128.6, 126.9, 126.1, 124.7, 33.5, 29.7.

Data are consistent with the literature.[87]

(*E*)-Pent-1-en-1-ylbenzene, **10**

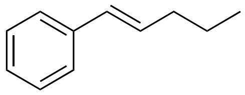

Prepared according to General Procedure 7 using 1,3-dioxoisoindolin-2-yl butyrate, **N5** (46.6 mg, 200 µmol, 1.0 equiv.), (*E*)-2-phenylvinylboronic acid, **1a** (59.2 mg, 400 µmol, 2.0 equiv.), tris(2,2'-bipyridine)ruthenium hexafluorophosphate (1.7 mg, 2.00 µmol, 1 mol%), and *N,N*-dimethylaniline (2.5 µL, 20.0 µmol, 10 mol%) in DMSO-d_6 (1 mL, 0.2 M). The crude residue was purified by flash chromatography (silica gel) with pure hexane affording 16.9 mg of a colourless oil as the desired product (58%, *E:Z* > 20:1).

^{1}H NMR (500 MHz, Chloroform-*d*) δ 7.39 – 7.33 (m, 2H), 7.34 – 7.27 (m, 2H), 7.24 – 7.17 (m, 1H), 6.40 (dt, *J* = 15.86, 1.52 Hz, 1H), 6.24 (dt, *J* = 15.79, 6.89 Hz, 1H), 2.21 (qd, *J* = 7.13, 1.46 Hz, 2H), 1.51 (h, *J* = 7.26 Hz, 2H), 0.97 (t, *J* = 7.38 Hz, 3H).

^{13}C NMR (126 MHz, Chloroform-*d*) δ 138.1, 131.1, 130.0, 128.6, 126.9, 126.0, 35.3, 22.7, 13.9.

Data are consistent with the literature.[89]

(E)-(3-Methylpent-1-en-1-yl)benzene, **11**

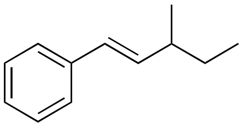

Prepared according to General Procedure 7 using 1,3-dioxoisoindolin-2-yl 2-methylbutanoate, **N6** (49.4 mg, 200 µmol, 1.0 equiv.), (E)-2-phenylvinylboronic acid, **1a** (59.2 mg, 400 µmol, 2.0 equiv.), tris(2,2'-bipyridine)ruthenium hexafluorophosphate (1.7 mg, 2.00 µmol, 1 mol%), and *N,N*-dimethylaniline (2.5 µL, 20.0 µmol, 10 mol%) in DMSO-d_6 (1 mL, 0.2 M). The crude residue was purified by flash chromatography (silica gel) with pure hexane affording 24.0 mg of a colourless oil as the desired product (75%, E:Z > 20:1).

^{1}H NMR (400 MHz, Chloroform-*d*) δ 7.40 – 7.33 (m, 2H), 7.34 – 7.25 (m, 2H), 7.24 – 7.15 (m, 1H), 6.35 (broad d, J = 15.89 Hz, 1H), 6.11 (dd, J = 15.87, 7.89 Hz, 1H), 2.27 – 2.15 (m, 1H), 1.46 – 1.38 (m, 2H), 1.09 (d, J = 6.76 Hz, 3H), 0.92 (t, J = 7.43 Hz, 3H).

^{13}C NMR (126 MHz, Chloroform-*d*) δ 138.1, 136.9, 128.6, 128.2, 126.9, 126.1, 39.1, 29.9, 20.4, 12.0.

Data are consistent with the literature.[90]

(E)-Non-1-en-1-ylbenzene, **12**

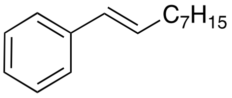

Prepared according to General Procedure 7 using 1,3-dioxoisoindolin-2-yl octanoate, **N7** (57.9 mg, 200 µmol, 1.0 equiv.), (E)-2-phenylvinylboronic acid, **1a** (59.2 mg, 400 µmol, 2.0 equiv.), tris(2,2'-bipyridine)ruthenium hexafluorophosphate (1.7 mg, 2.00 µmol, 1 mol%), and *N,N*-dimethylaniline (2.5 µL, 20.0 µmol, 10 mol%) in DMSO-d_6 (1 mL, 0.2 M). The crude residue was purified by flash chromatography (silica gel) with pure hexane affording 34.6 mg of a colourless oil as the desired product (86%, E:Z > 20:1).

^{1}H NMR (400 MHz, Chloroform-*d*) δ 7.37 – 7.33 (m, 2H), 7.32 – 7.27 (m, 2H), 7.22 – 7.16 (m, 1H), 6.41 – 6.32 (d, J = 15.95 Hz, 1H), 6.23 (dt, J = 15.79, 6.82 Hz, 1H), 2.21 (qd, J = 7.17, 1.33 Hz, 2H), 1.52 – 1.41 (m, 2H), 1.41 – 1.23 (m, 8H), 0.89 (t, J = 7.09 Hz, 3H).

^{13}C NMR (126 MHz, Chloroform-*d*) δ 138.1, 131.4, 129.8, 128.6, 126.9, 126.0, 33.2, 32.0, 29.5, 29.4 (2C), 22.8, 14.3.

Data are consistent with the literature.[89]

(E)-Pentadec-1-en-1-ylbenzene, **13**

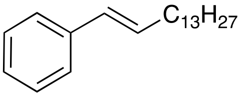

Prepared according to General Procedure 7 using 1,3-dioxoisoindolin-2-yl tetradecanoate, **N8** (74.7 mg, 200 µmol, 1.0 equiv.), (E)-2-phenylvinylboronic acid, **1a** (59.2 mg, 400 µmol, 2.0 equiv.), tris(2,2'-bipyridine)ruthenium hexafluorophosphate (1.7 mg, 2.00 µmol, 1 mol%), and *N,N*-dimethylaniline

(2.5 µL, 20.0 µmol, 10 mol%) in DMSO-d_6 (1 mL, 0.2 M). The crude residue was purified by flash chromatography (silica gel) with pure hexane affording 48.1 mg of a colourless oil as the desired product (84%, *E:Z* > 20:1).

^{1}H NMR (400 MHz, Chloroform-*d*) δ 7.37 – 7.33 (m, 2H), 7.32 – 7.27 (m, 2H), 7.22 – 7.18 (m, 1H), 6.38 (d, *J* = 15.86 Hz, 1H), 6.23 (dt, *J* = 15.80, 6.82 Hz, 1H), 2.24 – 2.17 (m, 2H), 1.51 – 1.42 (m, 2H), 1.27 (m, 20H), 0.88 (d, *J* = 6.74 Hz, 3H).

^{13}C NMR (126 MHz, Chloroform-*d*) δ 138.1, 131.4, 129.8, 128.6, 126.9, 126.0, 33.2, 32.1, 29.8, 29.8, 29.8, 29.7, 29.5, 29.5, 29.4, 22.9, 14.3. 2 carbon peaks are missing (overlapping between each other).

IR (film): 3024, 2922, 2852, 2358, 2341, 1494, 1465, 1377 cm^{-1}.

HRMS (EI): *m/z* calculated for $[M]^+$ $(C_{21}H_{34})^+$: 286.2655; found 286.2650.

(*E*)-But-1-ene-1,4-diyldibenzene, **14**

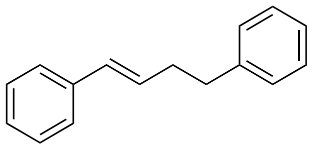

Prepared according to General Procedure 7 using 1,3-dioxoisoindolin-2-yl 3-phenylpropanoate, **N9** (59.1 mg, 200 µmol, 1.0 equiv.), (*E*)-2-phenylvinylboronic acid, **1a** (59.2 mg, 400 µmol, 2.0 equiv.), tris(2,2'-bipyridine)ruthenium hexafluorophosphate (1.7 mg, 2.00 µmol, 1 mol%), and *N,N*-dimethylaniline (2.5 µL, 20.0 µmol, 10 mol%) in DMSO-d_6 (1 mL, 0.2 M). The crude residue was purified by flash chromatography (silica gel) from pure hexane to a mixture of 1% of diethyl ether in hexane affording 32.8 mg of a white solid as the desired product (79%, *E:Z* > 20:1).

^{1}H NMR (500 MHz, Chloroform-d) δ 7.36 – 7.28 (m, 6H), 7.25 – 7.17 (m, 4H), 6.42 (d, J = 15.84 Hz, 1H), 6.27 (td, *J* = 15.80, 6.90 Hz, 1H), 2.83 – 2.75 (m, 2H), 2.57 – 2.51 (m, 2H).

^{13}C NMR (126 MHz, Chloroform-d) δ 141.9, 137.8, 130.5, 130.1, 128.6 (2 C), 128.5, 127.1, 126.1, 126.0, 36.0, 35.0.

Data consistent with the literature.[1]

(*E*)-1-Bromo-4-(4-phenylbut-3-en-1-yl)benzene, **15**

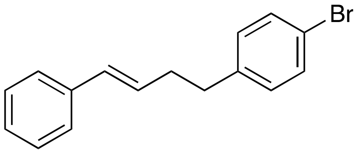

Prepared according to General Procedure 7 using 1,3-dioxoisoindolin-2-yl 3-(4-bromophenyl)propanoate, **N10** (56.1 mg, 150 µmol, 1.0 equiv.), (*E*)-2-phenylvinylboronic acid, **1a** (44.4 mg, 300 µmol, 2.0 equiv.), tris(2,2'-bipyridine)ruthenium hexafluorophosphate (1.3 mg, 1.50 µmol, 1 mol%), and *N,N*-dimethylaniline (1.9 µL, 15.0 µmol, 10 mol%) in DMSO-d_6 (1 mL, 0.15 M). The crude residue was purified by flash chromatography (silica gel) with pure hexane affording 34.5 mg of a white solid as the desired product (79%, *E:Z* > 20:1).

^{1}H NMR (700 MHz, Chloroform-*d*) δ 7.44 – 7.40 (m, 2H), 7.36 – 7.32 (m, 2H), 7.33 – 7.29 (m, 2H), 7.25 – 7.19 (m, 1H), 7.12 – 7.08 (m, 2H), 6.41 (d, *J* = 15.82 Hz, 1H), 6.23 (dt, *J* = 15.76, 6.87 Hz, 1H), 2.76 (t, *J* = 7.80 Hz, 2H), 2.55 – 2.46 (m, 2H).

^{13}C NMR (176 MHz, Chloroform-*d*) δ 140.8, 137.7, 131.5, 130.9, 130.4, 129.5, 128.6, 127.2, 126.1, 119.8, 35.4, 34.8.

Data are consistent with the literature.[91]

(*E*)-1-Ethynyl-4-(4-phenylbut-3-en-1-yl)benzene, **16**

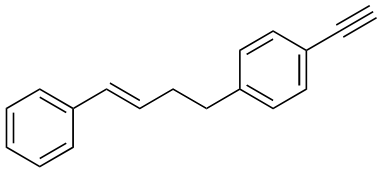

Prepared according to General Procedure 7 using 1,3-dioxoisoindolin-2-yl 3-(4-ethynylphenyl)propanoate, **N11** (31.9 mg, 100 μmol, 1.0 equiv.), (*E*)-2-phenylvinylboronic acid, **1a** (29.6 mg, 200 μmol, 2.0 equiv.), tris(2,2'-bipyridine)ruthenium hexafluorophosphate (0.9 mg, 1.00 μmol, 1 mol%), and *N,N*-dimethylaniline (1.3 μL, 10.0 μmol, 10 mol%) in DMSO-d_6 (1 mL, 0.2 M). The crude residue was purified by flash chromatography (silica gel) from pure hexane to a mixture of 4% of diethyl ether in hexane affording 16.2 mg of a white solid as the desired product (71%, *E:Z* > 20:1).

^{1}H NMR (500 MHz, Chloroform-*d*) δ 7.47 – 7.40 (m, 2H), 7.36 – 7.26 (m, 4H), 7.24 – 7.18 (m, 1H), 7.21 – 7.15 (m, 2H), 6.41 (dt, *J* = 15.85, 1.49 Hz, 1H), 6.23 (dt, *J* = 15.80, 6.85 Hz, 1H), 3.05 (s, 1H), 2.80 (t, *J* = 8.76 Hz, 2H), 2.57 – 2.48 (m, 2H).

^{13}C NMR (126 MHz, Chloroform-*d*) δ 142.9, 137.7, 132.3, 130.8, 129.6, 128.6 (2 C), 127.2, 126.1, 119.7, 83.9, 76.8, 35.9, 34.7.

IR (solid): 3277, 1508, 1492, 1448, 1242, 1213, 1176, 1105, 1068, 1020, 991, 979 cm^{-1}.

HRMS (EI): *m/z* calculated for $[M]^+$ ($C_{18}H_{16}$): 232.1246; found 232.1247.

(*E*)-(2-(1-Methylcyclohexyl)vinyl)benzene, **17**

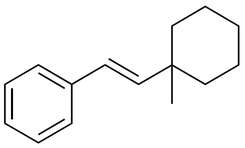

Prepared according to General Procedure 7 using 1,3-dioxoisoindolin-2-yl 1-methylcyclohexane-1-carboxylate, **N12** (57.5 mg, 200 μmol, 1.0 equiv.), (*E*)-2-phenylvinylboronic acid, **1a** (59.2 mg, 400 μmol, 2.0 equiv.), tris(2,2'-bipyridine)ruthenium hexafluorophosphate (1.7 mg, 2.00 μmol, 1 mol%), and *N,N*-dimethylaniline (2.5 μL, 20.0 μmol, 10 mol%) in DMSO-d_6 (1 mL, 0.2 M). The crude residue was purified by flash chromatography (silica gel) with pure hexane affording 23.6 mg of a colourless oil as the desired product (59%, *E:Z* > 20:1).

^{1}H NMR (500 MHz, Chloroform-*d*) δ 7.40 – 7.37 (m, 2H), 7.33 – 7.29 (m, 2H), 7.22 – 7.17 (m, 1H), 6.34 (d, *J* = 16.38 Hz, 1H), 6.23 (d, *J* = 16.35 Hz, 1H), 1.66 – 1.59 (m, 2H), 1.57 – 1.49 (m, 4H), 1.46 – 1.36 (m, 4H), 1.08 (s, 3H).

^{13}C NMR (126 MHz, Chloroform-*d*) δ 141.2, 138.4, 128.6, 126.8, 126.1, 126.1, 38.1, 36.3, 27.7, 26.5, 22.6. Data are consistent with the literature.[87]

Methyl (*E*)-6-phenylhex-5-enoate, **18**

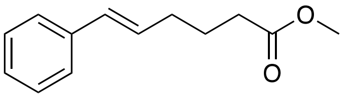

Prepared according to General Procedure 7 using 1,3-dioxoisoindolin-2-yl methyl glutarate, **N13** (58.3 mg, 200 µmol, 1.0 equiv.), (*E*)-2-phenylvinylboronic acid, **1a** (59.2 mg, 400 µmol, 2.0 equiv.), tris(2,2'-bipyridine)ruthenium hexafluorophosphate (1.7 mg, 2.00 µmol, 1 mol%), and *N,N*-dimethylaniline (2.5 µL, 20.0 µmol, 10 mol%) in DMSO-d_6 (1 mL, 0.2 M). The crude residue was purified by flash chromatography (silica gel) from pure hexane to a mixture of 1% of diethyl ether in hexane affording 33.1 mg of a colourless oil as the desired product (81%, *E:Z* > 20:1).

^{1}H NMR (500 MHz, Chloroform-*d*) δ 7.37 – 7.31 (m, 2H), 7.34 – 7.26 (m, 2H), 7.24 – 7.17 (m, 1H), 6.40 (dt, *J* = 15.81, 1.49 Hz, 1H), 6.19 (dt, *J* = 15.80, 6.97 Hz, 1H), 3.67 (s, 3H), 2.38 (t, *J* = 7.51 Hz, 2H), 2.26 (qd, *J* = 7.21, 1.47 Hz, 2H), 1.83 (p, *J* = 7.41 Hz, 2H).

^{13}C NMR (126 MHz, Chloroform-*d*) δ 174.2, 137.7, 130.9, 129.6, 128.6, 127.1, 126.1, 51.6, 33.5, 32.5, 24.6.

IR (film): 1734, 1597, 1435, 1363, 1269, 1246, 1196, 1172, 1149, 1022, 962, 742 cm^{-1}.

HRMS (ESI): *m/z* calculated for [M + Na^+] $(C_{13}H_{16}O_2Na)^+$: 227.1042, found 227.1040.

(*E*)-(5-Bromopent-1-en-1-yl)benzene, **19**

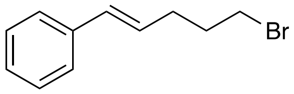

Prepared according to General Procedure 7 using 1,3-dioxoisoindolin-2-yl 4-bromobutanoate, **N14** (62.4 mg, 200 µmol, 1.0 equiv.), (*E*)-2-phenylvinylboronic acid, **1a** (59.2 mg, 400 µmol, 2.0 equiv.), tris(2,2'-bipyridine)ruthenium hexafluorophosphate (1.7 mg, 2.00 µmol, 1 mol%), and *N,N*-dimethylaniline (2.5 µL, 20.0 µmol, 10 mol%) in DMSO-d_6 (1 mL, 0.2 M). The crude residue was purified by flash chromatography (silica gel) with pure hexane affording 37.9 mg of a colourless oil as the desired product (66%, *E:Z* > 20:1).

^{1}H NMR (500 MHz, Chloroform-*d*) δ 7.38 – 7.34 (m, 2H), 7.34 – 7.29 (m, 2H), 7.26 – 7.19 (m, 1H), 6.46 (d, *J* = 15.82 Hz, 1H), 6.18 (dt, *J* = 15.84, 7.04 Hz, 1H), 3.47 (t, *J* = 6.64 Hz, 2H), 2.39 (m, 2H), 2.05 (p, *J* = 6.74 Hz, 2H).

^{13}C NMR (126 MHz, Chloroform-*d*) δ 137.5, 131.4, 128.7, 128.6, 127.3, 126.1, 33.4, 32.3, 31.4.

Data are consistent with the literature.[92]

(*E*)-Hexa-1,5-dien-1-ylbenzene, **20**

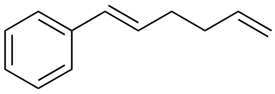

Prepared according to General Procedure 7 using 1,3-dioxoisoindolin-2-yl pent-4-enoate, **N15** (49.0 mg, 200 μmol, 1.0 equiv.), (*E*)-2-phenylvinylboronic acid, **1a** (59.2 mg, 400 μmol, 2.0 equiv.), tris(2,2'-bipyridine)ruthenium hexafluorophosphate (1.7 mg, 2.00 μmol, 1 mol%), and *N,N*-dimethylaniline (2.5 μL, 20.0 μmol, 10 mol%) in DMSO-d_6 (1 mL, 0.2 M). The crude residue was purified by flash chromatography (silica gel) with pure hexane affording 25.1 mg of a colourless oil as the desired product (79%, *E:Z* > 20:1).

^{1}H NMR (500 MHz, Chloroform-*d*) δ 7.38 – 7.34 (m, 2H), 7.32 – 7.28 (m, 2H), 7.24 – 7.18 (m, 1H), 6.42 (d, *J* = 15.83 Hz, 1H), 6.24 (dt, *J* = 15.86, 6.72 Hz, 1H), 5.88 (ddt, *J* = 16.78, 10.20, 6.50 Hz, 1H), 5.08 (dq, *J* = 17.14, 1.70 Hz, 1H), 5.01 (dq, *J* = 10.20, 1.39 Hz, 1H), 2.38 – 2.30 (m, 2H), 2.28 – 2.20 (m, 2H).

^{13}C NMR (126 MHz, Chloroform-*d*) δ 138.3, 137.9, 130.3, 130.3, 128.6, 127.0, 126.1, 115.1, 33.7, 32.6.

Data are consistent with the literature.[87]

(*E*)-(3-(Cyclopent-2-en-1-yl)prop-1-en-1-yl)benzene, **21**

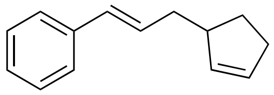

Prepared according to General Procedure 7 using 1,3-dioxoisoindolin-2-yl 2-(cyclopent-2-en-1-yl)acetate, **N16** (54.3 mg, 200 μmol, 1.0 equiv.), (*E*)-2-phenylvinylboronic acid, **1a** (59.2 mg, 400 μmol, 2.0 equiv.), tris(2,2'-bipyridine)ruthenium hexafluorophosphate (1.7 mg, 2.00 μmol, 1 mol%), and *N,N*-dimethylaniline (2.5 μL, 20.0 μmol, 10 mol%) in DMSO-d_6 (1 mL, 0.2 M). The crude residue was purified by flash chromatography (silica gel) with pure hexane affording 21.0 mg of a colourless oil as the desired product (57%, *E:Z* > 20:1).

^{1}H NMR (500 MHz, Chloroform-*d*) δ 7.39 – 7.33 (m, 2H), 7.33 – 7.26 (m, 2H), 7.23 – 7.16 (m, 1H), 6.40 (d, *J* = 15.80 Hz, 1H), 6.23 (dt, *J* = 15.66, 7.17 Hz, 1H), 5.80 – 5.64 (m, 2H), 2.88 – 2.77 (m, 1H), 2.42 – 2.14 (m, 4H), 2.10 – 2.00 (m, 1H), 1.55 – 1.44 (m, 1H).

^{13}C NMR (126 MHz, Chloroform-*d*) δ 138.0, 134.7, 131.0, 130.8, 129.6, 128.6, 127.0, 126.1, 45.6, 39.5, 32.2, 29.4.

IR (film): 3055, 3024, 2924, 2851, 2364, 2357, 1495, 1448, 962, 912, 741, 723, 692 cm^{-1}.

HRMS (EI): *m/z* calculated for $[M]^+$ $(C_{14}H_{16})^+$: 184.1246; found 184.1243.

(*E*)-Hex-1-en-5-yn-1-ylbenzene, **22**

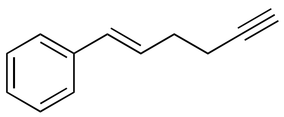

Prepared according to General Procedure 7 using 1,3-dioxoisoindolin-2-yl pent-4-ynoate, **N17** (48.6 mg, 200 μmol, 1.0 equiv.), (*E*)-2-phenylvinylboronic acid, **1a** (59.2 mg, 400 μmol, 2.0 equiv.),

tris(2,2'-bipyridine)ruthenium hexafluorophosphate (1.7 mg, 2.00 µmol, 1 mol%), and *N,N*-dimethylaniline (2.5 µL, 20.0 µmol, 10 mol%) in DMSO-d_6 (1 mL, 0.2 M). The crude residue was purified by flash chromatography (silica gel) from pure hexane to a mixture of 1% of diethyl ether in hexane affording 25.8 mg of a colourless oil as the desired product (84%, *E:Z* > 20:1).

^{1}H NMR (500 MHz, Chloroform-*d*) δ 7.39 – 7.36 (m, 2H), 7.34 – 7.28 (m, 2H), 7.24 – 7.20 (m, 1H), 6.47 (d, *J* = 15.76 Hz, 1H), 6.28 (dt, *J* = 15.78, 6.75 Hz, 1H), 2.49 – 2.43 (m, 2H), 2.37 (td, *J* = 7.00, 2.02 Hz, 2H), 2.01 (t, *J* = 2.61 Hz, 1H).

^{13}C NMR (126 MHz, Chloroform-*d*) δ 137.5, 131.2, 128.6, 128.5, 127.3, 126.2, 83.9, 69.0, 32.1, 18.9.

IR (film): 3298, 3026, 2912, 1495, 1447, 1433, 1068, 962, 741, 692, 635 cm^{-1}.

HRMS (EI): *m/z* calculated for $[M]^+$ $(C_{12}H_{12})^+$: 156.0933; found 156.0929.

tert-Butyl cinnamylcarbamate, **23**

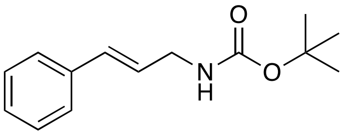

Prepared according to General Procedure 7 using 1,3-dioxoisoindolin-2-yl (*tert*-butoxycarbonyl)glycinate, **N18** (61.4 mg, 200 µmol, 1.0 equiv.), (*E*)-2-phenylvinylboronic acid, **1a** (59.2 mg, 400 µmol, 2.0 equiv.), tris(2,2'-bipyridine)ruthenium hexafluorophosphate (1.7 mg, 2.00 µmol, 1 mol%), and *N,N*-dimethylaniline (2.5 µL, 20.0 µmol, 10 mol%) in DMSO-d_6 (1 mL, 0.2 M). The crude residue was purified by flash chromatography (silica gel) from pure hexane to a mixture of 10% of diethyl ether in hexane affording 27.0 mg of a white solid as the desired product (58%, *E:Z* > 20:1).

^{1}H NMR (500 MHz, Chloroform-*d*) δ 7.37 – 7.34 (m, 2H), 7.33 – 7.29 (m, 2H), 7.25 – 7.21 (m, 1H), 6.50 (d, *J* = 15.79, 1H), 6.19 (dt, *J* = 15.69, 6.12 Hz, 1H), 4.70 (broad s, 1 NH), 3.91 (t, *J* = 6.20 Hz, 2H), 1.47 (s, 9H).

^{13}C NMR (126 MHz, Chloroform-*d*) δ 155.9, 136.8, 131.6, 128.7, 127.7, 126.5 (2 C), 79.6, 42.8, 28.5.

Data are consistent with the literature.[93]

tert-Butyl (*E*)-methyl(4-phenylbut-3-en-2-yl)carbamate, **24**

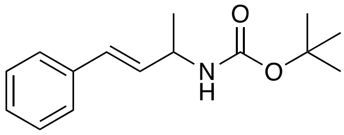

Prepared according to General Procedure 7 using 1,3-dioxoisoindolin-2-yl (*tert*-butoxycarbonyl)alaninate, **N19** (66.9 mg, 200 µmol, 1.0 equiv.), (*E*)-2-phenylvinylboronic acid, **1a** (59.2 mg, 400 µmol, 2.0 equiv.), tris(2,2'-bipyridine)ruthenium hexafluorophosphate (1.7 mg, 2.00 µmol, 1 mol%), and *N,N*-dimethylaniline (2.5 µL, 20.0 µmol, 10 mol%) in DMSO-d_6 (1 mL, 0.2 M). The crude residue was purified by flash chromatography (silica gel) from pure hexane to a mixture of 5% of diethyl ether in hexane affording 12.6 mg of a colourless oil as the desired product (25%, *E:Z* > 20:1).

^{1}H NMR (500 MHz, Chloroform-*d*) δ 7.40 – 7.36 (m, 2H), 7.35 – 7.30 (m, 2H), 7.27 – 7.23 (m, 1H), 6.52 (d, *J* = 16.10 Hz, 1H), 6.18 (dd, *J* = 15.94, 5.71 Hz, 1H), 4.57 (broad s, 1 NH), 4.43 (broad s, 1H), 1.49 (s, 9H), 1.34 (d, *J* = 6.80 Hz, 3H).

^{13}C NMR (126 MHz, Chloroform-*d*) δ 155.3, 137.0, 131.8, 129.3, 128.7, 127.6, 126.5, 79.5, 48.0 (broad), 28.6, 21.3.

Data are consistent with the literature.[94]

(*E*)-*tert*-Butyl((2,2-dimethyl-4-phenylbut-3-en-1-yl)oxy)dimethylsilane, **25**

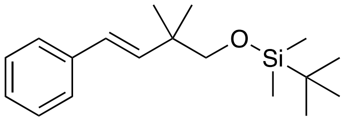

Prepared according to General Procedure 7 using 1,3-dioxoisoindolin-2-yl 3-((*tert*-butyldimethylsilyl)oxy)-2,2-dimethylpropanoate, **N20** (75.5 mg, 200 µmol, 1.0 equiv.), (*E*)-2-phenylvinylboronic acid, **1a** (59.2 mg, 400 µmol, 2.0 equiv.), tris(2,2'-bipyridine)ruthenium hexafluorophosphate (1.7 mg, 2.00 µmol, 1 mol%), and *N,N*-dimethylaniline (2.5 µL, 20.0 µmol, 10 mol%) in DMSO-d_6 (1 mL, 0.2 M). The crude residue was purified by flash chromatography (silica gel) with pure hexane affording 41.3 mg of a colourless oil as the desired product (71%, *E:Z* > 20:1).

^{1}H NMR (500 MHz, Chloroform-*d*) δ 7.39 – 7.35 (m, 2H), 7.33 – 7.27 (m, 2H), 7.24 – 7.18 (m, 1H), 6.38 (d, *J* = 16.32 Hz, 1H), 6.27 (d, *J* = 16.30 Hz, 1H), 3.40 (s, 2H), 1.11 (s, 6H), 0.92 (s, 9H), 0.05 (s, 6H).

^{13}C NMR (126 MHz, Chloroform-*d*) δ 138.3, 138.2, 128.6, 126.9, 126.9, 126.2, 72.0, 38.8, 26.1, 24.2, 18.5, –5.3.

IR (film): 2954, 2854, 1494, 1471, 1388, 1359, 1249, 1093, 968, 912, 850 cm^{-1}.

HRMS (ESI): *m/z* calculated for $[M + H^{+}]$ $(C_{18}H_{31}OSi)^{+}$: 291.21387, found 291.2125.

(*E*)-3-Methyl-3-styryloxetane, **26**

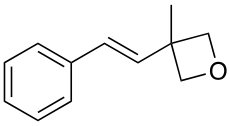

Prepared according to General Procedure 7 using 1,3-dioxoisoindolin-2-yl 3-methyloxetane-3-carboxylate, **N21** (52.2 mg, 200 µmol, 1.0 equiv.), (*E*)-2-phenylvinylboronic acid, **1a** (59.2 mg, 400 µmol, 2.0 equiv.), tris(2,2'-bipyridine)ruthenium hexafluorophosphate (1.7 mg, 2.00 µmol, 1 mol%), and *N,N*-dimethylaniline (2.5 µL, 20.0 µmol, 10 mol%) in DMSO-d_6 (1 mL, 0.2 M). The crude residue was purified by flash chromatography (silica gel) with pure hexane to 4% of diethyl ether in hexane affording 18.6 mg of a colourless oil as the desired product (63%, *E:Z* > 20:1).

^{1}H NMR (500 MHz, Chloroform-*d*) δ 7.42 – 7.38 (m, 2H), 7.35 – 7.29 (m, 2H), 7.27 – 7.21 (m, 1H), 6.57 (d, *J* = 16.21 Hz, 1H), 6.43 (d, *J* = 16.17 Hz, 1H), 4.73 (d, *J* = 5.59 Hz, 2H), 4.49 (d, *J* = 5.67 Hz, 2H), 1.59 (s, 3H).

^{13}C NMR (126 MHz, Chloroform-*d*) δ 137.1, 134.4, 128.8, 128.3, 127.6, 126.3, 83.0, 41.4, 23.6.

IR (film): 2961, 2932, 2866, 1491, 1449, 1379, 978, 912, 827, 746, 731, 692 cm^{-1}.

HRMS (ESI): *m/z* calculated for $[M + Na]^+$ $(C_{12}H_{14}ONa)^+$: 197.0937; found 197.0935.

(*E*)-3-Styrylcyclobutan-1-one, **27**

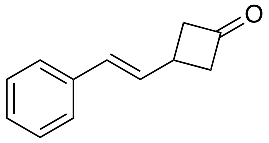

Prepared according to General Procedure 7 using 1,3-dioxoisoindolin-2-yl 3-oxocyclobutane-1-carboxylate, **N22** (51.8 mg, 200 μmol, 1.0 equiv.), (*E*)-2-phenylvinylboronic acid, **1a** (59.2 mg, 400 μmol, 2.0 equiv.), tris(2,2'-bipyridine)ruthenium hexafluorophosphate (1.7 mg, 2.00 μmol, 1 mol%), and *N,N*-dimethylaniline (2.5 μL, 20.0 μmol, 10 mol%) in DMSO-d_6 (1 mL, 0.2 M). The crude residue was purified by flash chromatography (silica gel) with pure hexane affording 21.0 mg of a white solid as the desired product (61%, *E:Z* > 20:1).

^{1}H NMR (500 MHz, Chloroform-*d*) δ 7.41 – 7.35 (m, 2H), 7.35 – 7.28 (m, 2H), 7.28 – 7.21 (m, 1H), 6.50 (d, *J* = 15.75 Hz, 1H), 6.38 (dd, *J* = 15.74, 7.08 Hz, 1H), 3.37 – 3.29 (m, 2H), 3.29 – 3.20 (m, 1H), 3.10 – 3.02 (m, 2H).

^{13}C NMR (126 MHz, Chloroform-*d*) δ 207.0, 136.8, 132.2, 130.3, 128.8, 127.6, 126.2, 53.5, 26.8.

IR (film): 1769, 1732, 1674, 1599, 1493, 1450, 1377, 1178, 1105, 988, 978, 752, 694 cm^{-1}.

HRMS (ESI): *m/z* calculated for $[M + H]^+$ $(C_{12}H_{13}O)^+$: 173.0960; found 173.0968.

tert-Butyl (*E*)-4-styrylpiperidine-1-carboxylate, **28**

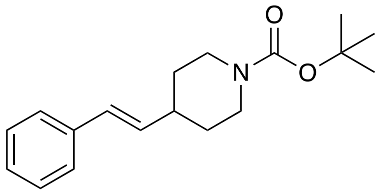

Prepared according to General Procedure 7 using 1-(*tert*-butyl) 4-(1,3-dioxoisoindolin-2-yl) piperidine-1,4-dicarboxylate, **N23** (74.9 mg, 200 μmol, 1.0 equiv.), (*E*)-2-phenylvinylboronic acid, **1a** (59.2 mg, 400 μmol, 2.0 equiv.), tris(2,2'-bipyridine)ruthenium hexafluorophosphate (1.7 mg, 2.00 μmol, 1 mol%), and *N,N*-dimethylaniline (2.5 μL, 20.0 μmol, 10 mol%) in DMSO-d_6 (1 mL, 0.2 M). The crude residue was purified by flash chromatography (silica gel) from pure hexane to a mixture of 6% of diethyl ether in hexane affording 38.9 mg of a white solid as the desired product (68%, *E:Z* > 20:1).

^{1}H NMR (500 MHz, Chloroform-*d*) δ 7.37 – 7.33 (m, 2H), 7.32 – 7.27 (m, 2H), 7.23 – 7.17 (m, 1H), 6.39 (broad d, *J* = 15.74 Hz, 1H), 6.15 (dd, *J* = 15.97, 6.89 Hz, 1H), 4.13 (broad s, 2H), 2.77 (broad s, 2H), 2.35 – 2.22 (m, 1H), 1.80 – 1.72 (m, 2H), 1.47 (s, 9H), 1.44 – 1.32 (m, 2H).

^{13}C NMR (126 MHz, Chloroform-*d*) δ 155.0, 137.6, 134.5, 128.7, 128.6, 127.2, 126.2, 79.5, 44.0 (broad), 39.5, 31.9, 28.6.

Data are consistent with the literature.[95]

tert-Butyl (*E*)-3-styrylpiperidine-1-carboxylate, **29**

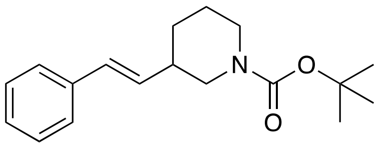

Prepared according to General Procedure 7 using 1-(*tert*-butyl) 3-(1,3-dioxoisoindolin-2-yl) piperidine-1,3-dicarboxylate, **N24** (74.9 mg, 200 μmol, 1.0 equiv.), (*E*)-2-phenylvinylboronic acid, **1a** (59.2 mg, 400 μmol, 2.0 equiv.), tris(2,2'-bipyridine)ruthenium hexafluorophosphate (1.7 mg, 2.00 μmol, 1 mol%), and *N*,*N*-dimethylaniline (2.5 μL, 20.0 μmol, 10 mol%) in DMSO-d_6 (1 mL, 0.2 M). The crude residue was purified by flash chromatography (silica gel) with pure hexane to a mixture of 5% of diethyl ether in hexane affording 34.7 mg of a colourless oil as the desired product (61%%, *E:Z* > 20:1).

^{1}H NMR (500 MHz, Chloroform-*d*) δ 7.38 – 7.32 (m, 2H), 7.32 – 7.27 (m, 2H), 7.24 – 7.18 (m, 1H), 6.45 (dd, *J* = 16.09, 1.28 Hz, 1H), 6.09 (dd, *J* = 16.04, 7.05 Hz, 1H), 4.14 (broad s, 1H), 4.02 – 3.95 (broad d, *J* = 13.36 Hz, 1H), 2.82 – 2.73 (m, 1H), 2.73 – 2.55 (m, 1H), 2.39 – 2.25 (m, 1H), 1.98 – 1.87 (m, 1H), 1.74 – 1.67 (m, 1H), 1.55 – 1.48 (m, 1H), 1.47 (s, 9H), 1.42 – 1.33 (m, 1H).

^{13}C NMR (126 MHz, Chloroform-*d*) δ 155.0, 137.5, 131.9, 129.9, 128.7, 127.3, 126.2, 79.5, 49.3 (broad), 44.3 (broad), 39.5, 31.0, 28.6, 24.9.

Data are consistent with the literature.[87]

tert-Butyl (*E*)-2-styrylpyrrolidine-1-carboxylate, **30**

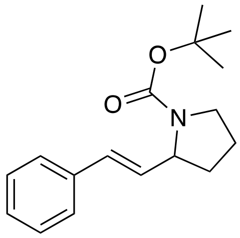

Prepared according to General Procedure 7 using 1-(*tert*-butyl) 2-(1,3-dioxoisoindolin-2-yl) (*S*)-pyrrolidine-1,2-dicarboxylate, **N26** (72.1 mg, 200 μmol, 1.0 equiv.), (*E*)-2-phenylvinylboronic acid, **1a** (59.2 mg, 400 μmol, 2.0 equiv.), tris(2,2'-bipyridine)ruthenium hexafluorophosphate (1.7 mg, 2.00 μmol, 1 mol%), and *N*,*N*-dimethylaniline (2.5 μL, 20.0 μmol, 10 mol%) in DMSO-d_6 (1 mL, 0.2 M). The crude residue was purified by flash chromatography (silica gel) with pure hexane affording 26.0 mg of a white solid as the desired product (84%, *E:Z* > 20:1).

^{1}H NMR (400 MHz, Chloroform-*d*) δ 7.41 – 7.28 (m, 4H), 7.28 – 7.20 (m, 1H), 6.42 (bd, *J* = 16.02 Hz, 1H), 6.25 – 6.00 (broad s, 1H), 4.60 – 4.34 (broad s, 1H), 3.61 – 3.26 (broad s, 2H), 2.23 – 2.05 (broad s, 1H), 2.00 – 1.85 (m, 2H), 1.85 – 1.78 (m, 1H), 1.47 (broad s, 9H).

^{13}C NMR (176 MHz, Chloroform-*d*) δ 154.9, 137.2, 130.9, 129.6, 128.7, 127.4, 126.4, 79.3, 59.1, 46.4, 32.7, 28.6, 23.2.

Data are consistent with the literature.[95]

tert-Butyl (*E*)-2-styrylpiperidine-1-carboxylate, **31**

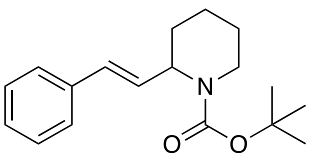

Prepared according to General Procedure 7 using 1-(*tert*-butyl) 2-(1,3-dioxoisoindolin-2-yl) piperidine-1,2-dicarboxylate, **N25** (74.9 mg, 200 µmol, 1.0 equiv.), (*E*)-2-phenylvinylboronic acid, **1a** (59.2 mg, 400 µmol, 2.0 equiv.), tris(2,2'-bipyridine)ruthenium hexafluorophosphate (1.7 mg, 2.00 µmol, 1 mol%), and *N,N*-dimethylaniline (2.5 µL, 20.0 µmol, 10 mol%) in DMSO-d_6 (1 mL, 0.2 M). The crude residue was purified by flash chromatography (silica gel) from pure hexane to a mixture of 5% of diethyl ether in hexane affording 29.7 mg of a colourless oil as the desired product (52%, *E:Z* > 20:1).

^{1}H NMR (500 MHz, Chloroform-*d*) δ 7.42 – 7.36 (m, 2H), 7.37 – 7.30 (m, 2H), 7.29 – 7.22 (m, 1H), 6.41 (dd, *J* = 16.14, 1.94 Hz, 1H), 6.21 (dd, *J* = 16.12, 4.77 Hz, 1H), 4.99 (broad s, 1H), 4.07 – 3.96 (m, 1H), 2.99 – 2.89 (m, 1H), 1.90 – 1.83 (m, 1H), 1.83 – 1.74 (m, 1H), 1.69 – 1.66 (m, 1H), 1.66 – 1.62 (m, 1H), 1.62 – 1.55 (m, 1H), 1.50 (s, 9H), 1.48 – 1.44 (m, 1H).

^{13}C NMR (126 MHz, Chloroform-*d*) δ 155.5, 137.2, 130.8, 128.9, 128.7, 127.5, 126.4, 79.6, 52.3, 40.0, 29.6, 28.6, 25.7, 19.8.

Data are consistent with the literature.[96]

(*E*)-3-Styryltetrahydrofuran, **32**

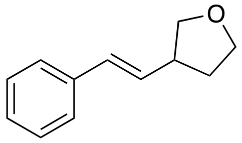

Prepared according to General Procedure 7 using 1,3-Dioxoisoindolin-2-yl tetrahydrofuran-3-carboxylate, **N43** (52.2 mg, 200 µmol, 1.0 equiv.), (*E*)-2-phenylvinylboronic acid, **1a** (59.2 mg, 400 µmol, 2.0 equiv.), tris(2,2'-bipyridine)ruthenium hexafluorophosphate (1.7 mg, 2.00 µmol, 1 mol%), and *N,N*-dimethylaniline (2.5 µL, 20.0 µmol, 10 mol%) in DMSO-d_6 (1 mL, 0.2 M). The crude residue was purified by flash chromatography (silica gel) from pure hexane to a mixture of 10% of diethyl ether in hexane affording 23.1 mg of a colourless oil as the desired product (66%, *E:Z* > 20:1).

^{1}H NMR (400 MHz, Chloroform-*d*) δ 7.39 – 7.32 (m, 2H), 7.35 – 7.27 (m, 2H), 7.26 – 7.18 (m, 1H), 6.46 (d, *J* = 15.73 Hz, 1H), 6.15 (dd, *J* = 15.77, 8.42 Hz, 1H), 4.03 – 3.91 (m, 2H), 3.85 (td, *J* = 8.13, 7.18 Hz, 1H), 3.54 (dd, *J* = 8.41, 7.53 Hz, 1H), 3.03 (h, *J* = 7.88 Hz, 1H), 2.18 (dtd, *J* = 12.22, 7.23, 4.62 Hz, 1H), 1.82 (dq, *J* = 12.28, 7.99 Hz, 1H).

^{13}C NMR (101 MHz, Chloroform-*d*) δ 137.3, 130.8, 130.6, 128.7, 127.4, 126.2, 73.1, 68.4, 43.3, 33.4.

Data are consistent with the literature.[97]

(*E*)-2-Styryltetrahydrofuran, **33**

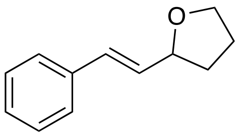

Prepared according to General Procedure 7 using 1,3-dioxoisoindolin-2-yl tetrahydrofuran-2-carboxylate, **N44** (52.2 mg, 200 μmol, 1.0 equiv.), (*E*)-2-phenylvinylboronic acid, **1a** (59.2 mg, 400 μmol, 2.0 equiv.), tris(2,2'-bipyridine)ruthenium hexafluorophosphate (1.7 mg, 2.00 μmol, 1 mol%), and *N,N*-dimethylaniline (2.5 μL, 20.0 μmol, 10 mol%) in DMSO-d_6 (1 mL, 0.2 M). The crude residue was purified by flash chromatography (silica gel) from pure hexane to a mixture of 10% of diethyl ether in hexane affording 22.3 mg of a colorless oil as the desired product (64%, *E:Z* > 20:1).

^{1}H NMR (400 MHz, Chloroform-*d*) δ 7.42 – 7.37 (m, 2H), 7.36 – 7.28 (m, 2H), 7.25 – 7.20 (m, 1H), 6.59 (d, *J* = 15.88 Hz, 1H), 6.22 (dd, *J* = 15.84, 6.61 Hz, 1H), 4.48 (td, *J* = 7.82, 7.23, 6.04 Hz, 1H), 3.98 (ddd, *J* = 8.31, 7.25, 6.22 Hz, 1H), 3.85 (td, *J* = 7.90, 6.16 Hz, 1H), 2.19 – 2.09 (m, 1H), 2.03 – 1.91 (m, 2H), 1.79 – 1.66 (m, 1H).

^{13}C NMR (101 MHz, Chloroform-*d*) δ 137.0, 130.6, 130.6, 128.6, 127.6, 126.6, 79.8, 68.3, 32.5, 26.0.

Data are consistent with the literature.[98]

(*E*)-2-Styryltetrahydro-2*H*-pyran, **34**

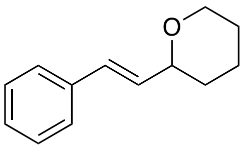

Prepared according to General Procedure 7 using 1,3-dioxoisoindolin-2-yl tetrahydro-2*H*-pyran-2-carboxylate, **N45** (55.1 mg, 200 μmol, 1.0 equiv.), (*E*)-2-phenylvinylboronic acid, **1a** (59.2 mg, 400 μmol, 2.0 equiv.), tris(2,2'-bipyridine)ruthenium hexafluorophosphate (1.7 mg, 2.00 μmol, 1 mol%), and *N,N*-dimethylaniline (2.5 μL, 20.0 μmol, 10 mol%) in DMSO-d_6 (1 mL, 0.2 M). The crude residue was purified by flash chromatography (silica gel) from pure hexane to a mixture of 8% of diethyl ether in hexane affording 24.4 mg of a colorless oil as the desired product (65%, *E:Z* > 20:1).

^{1}H NMR (400 MHz, Chloroform-*d*) δ 7.42 – 7.35 (m, 2H), 7.34 – 7.26 (m, 2H), 7.26 – 7.18 (m, 1H), 6.59 (dd, *J* = 16.10, 1.37 Hz, 1H), 6.22 (dd, *J* = 16.03, 5.80 Hz, 1H), 4.12 – 4.03 (m, 1H), 3.98 (ddt, *J* = 10.63, 5.80, 1.86 Hz, 1H), 3.55 (td, *J* = 11.58, 2.68 Hz, 1H), 1.95 – 1.84 (m, 1H), 1.80 – 1.70 (m, 1H), 1.68 – 1.54 (m, 3H), 1.53 – 1.45 (m, 1H).

^{13}C NMR (101 MHz, Chloroform-*d*) δ 137.1, 131.0, 129.9, 128.6, 127.6, 126.5, 78.2, 68.6, 32.4, 26.0, 23.6.

Data are consistent with the literature.[99]

(*E*)-2-(4-Phenylbut-3-en-1-yl)furan, **35**

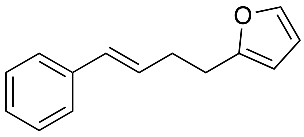

Prepared according to General Procedure 7 using 1,3-dioxoisoindolin-2-yl 3-(furan-2-yl)propanoate, **N27** (57.1 mg, 200 μmol, 1.0 equiv.), (*E*)-2-phenylvinylboronic acid, **1a** (59.2 mg, 400 μmol, 2.0 equiv.), tris(2,2'-bipyridine)ruthenium hexafluorophosphate (1.7 mg, 2.00 μmol, 1 mol%), and *N,N*-dimethylaniline (2.5 μL, 20.0 μmol, 10 mol%) in DMSO-d_6 (1 mL, 0.2 M). The crude residue was purified by flash

chromatography (silica gel) with pure hexane affording 30.1 mg of a colourless oil as the desired product (76%, *E:Z* > 20:1).

^{1}H NMR (500 MHz, Chloroform-*d*) δ 7.37 – 7.33 (m, 3H), 7.33 – 7.28 (m, 2H), 7.24 – 7.20 (m, 1H), 6.45 (d, *J* = 15.83 Hz, 1H), 6.31 (dd, *J* = 3.14, 1.88 Hz, 1H), 6.26 (dt, *J* = 15.81, 6.84 Hz, 1H), 6.05 (dd, *J* = 3.11, 1.05 Hz, 1H), 2.83 (t, *J* = 7.59 Hz, 2H), 2.62 – 2.54 (m, 2H).

^{13}C NMR (126 MHz, Chloroform-*d*) δ 155.6, 141.0, 137.7, 130.8, 129.5, 128.6, 127.1, 126.1, 110.3, 105.2, 31.6, 28.1.

IR (film): 2918, 2849, 2361, 2339, 1797, 1711, 1597, 1506, 1493, 1447, 1209, 1149, 1006, 962, 921 cm^{-1}.

HRMS (ESI): *m/z* calculated for $[M + Na]^+$ $(C_{14}H_{14}ONa)^+$: 221.0937; found 221.0934.

(*E*)-3-(4-Phenylbut-3-en-1-yl)-1*H*-indole, **36**

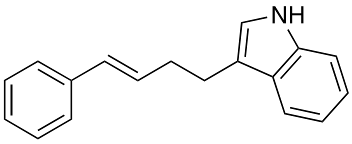

Prepared according to General Procedure 7 using 1,3-dioxoisoindolin-2-yl 3-(1*H*-indol-3-yl)propanoate, **N28** (66.9 mg, 200 µmol, 1.0 equiv.), (*E*)-2-phenylvinylboronic acid, **1a** (59.2 mg, 400 µmol, 2.0 equiv.), tris(2,2'-bipyridine)ruthenium hexafluorophosphate (1.7 mg, 2.00 µmol, 1 mol%), and *N,N*-dimethylaniline (2.5 µL, 20.0 µmol, 10 mol%) in DMSO-d_6 (1 mL, 0.2 M). The crude residue was purified by flash chromatography (silica gel) from pure hexane to a mixture of 5% of diethyl ether in hexane affording 19.6 mg of a white solid as the desired product (40%, *E:Z* > 20:1).

^{1}H NMR (500 MHz, Chloroform-*d*) δ 7.91 (s, 1H), 7.70 – 7.63 (m, 1H), 7.40 – 7.33 (m, 3H), 7.34 – 7.26 (m, 2H), 7.26 – 7.18 (m, 2H), 7.18 – 7.11 (m, 1H), 7.03 (d, *J* = 2.26 Hz, 1H), 6.47 (d, *J* = 15.80 Hz, 1H), 6.36 (dt, *J* = 15.81, 6.73 Hz, 1H), 2.99 – 2.93 (m, 2H), 2.69 – 2.61 (m, 2H).

^{13}C NMR (126 MHz, Chloroform-*d*) δ 137.9, 136.4, 130.9, 130.3, 128.6, 127.6, 127.0, 126.1, 122.1, 121.4, 119.3, 119.1, 116.3, 111.2, 33.7, 25.3.

IR (film): 2918, 2847, 1597, 1491, 1456, 1420, 1339, 1223, 1091, 1028, 1010, 964, 738, 692 cm^{-1}.

HRMS (ESI): *m/z* calculated for $[M + Na]^+$ $(C_{18}H_{17}NNa)^+$: 270.1253; found 270.1250.

tert-Butyl (*E*)-3-(4-phenylbut-3-en-1-yl)-1*H*-indole-1-carboxylate, **37**

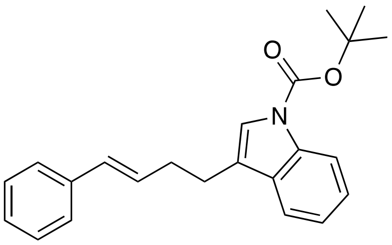

Prepared according to General Procedure 7 using *tert*-butyl 3-(3-((1,3-dioxoisoindolin-2-yl)oxy)-3-oxopropyl)-1*H*-indole-1-carboxylate **N29** (84.1 mg, 200 µmol, 1.0 equiv.), (*E*)-2-phenylvinylboronic acid, **1a** (59.2 mg, 400 µmol, 2.0 equiv.), tris(2,2'-bipyridine)ruthenium hexafluorophosphate (1.7 mg, 2.00 µmol,

1 mol%), and *N,N*-dimethylaniline (2.5 µL, 20.0 µmol, 10 mol%) in DMSO-d_6 (1 mL, 0.2 M). The crude residue was purified by flash chromatography (silica gel) from pure hexane to a mixture of 1% of diethyl ether in hexane affording 38.8 mg of a white solid as the desired product (55%, *E:Z* > 20:1).

^{1}H NMR (500 MHz, Chloroform-*d*) δ 8.16 (broad s, 1H, H^{16}), 7.60 – 7.56 (m, 1H), 7.43 (broad s, 1H), 7.38 – 7.35 (m, 2H), 7.35 – 7.30 (m, 3H), 7.29 – 7.25 (m, 1H), 7.25 – 7.20 (m, 1H), 6.49 (d, *J* = 15.83 Hz, 1H), 6.34 (dt, *J* = 15.72, 6.77 Hz, 1H), 2.89 (t, *J* = 7.65 Hz, 2H), 2.72 – 2.59 (m, 2H), 1.68 (s, 9H).

^{13}C NMR (126 MHz, Chloroform-*d*) δ 150.0, 137.7, 135.7, 130.8, 130.6, 130.1, 128.6, 127.1, 126.1, 124.4, 122.6, 122.4, 120.6, 119.1, 115.4, 83.5, 32.9, 28.4, 25.1.

IR (film): 2978, 2930, 1726, 1450, 1367, 1308, 1251, 1223, 11531084, 963, 857, 765, 740 cm^{-1}.

HRMS (EI): *m/z* calculated for [M]$^+$ ($C_{23}H_{25}NO_2$)$^+$: 347.1880; found 347.1885.

(*E*)-3-(4-Phenylbut-3-en-1-yl)pyridine, **38**

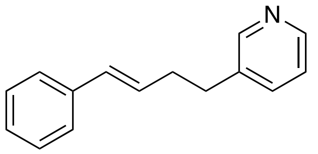

Prepared according to General Procedure 7 using 1,3-dioxoisoindolin-2-yl 3-(pyridin-3-yl)propanoate, **N30** (59.3 mg, 200 µmol, 1.0 equiv.), (*E*)-2-phenylvinylboronic acid, **1a** (59.2 mg, 400 µmol, 2.0 equiv.), tris(2,2'-bipyridine)ruthenium hexafluorophosphate (1.7 mg, 2.00 µmol, 1 mol%), and *N,N*-dimethylaniline (2.5 µL, 20.0 µmol, 10 mol%) in DMSO-d_6 (1 mL, 0.2 M). The crude residue was purified by flash chromatography (silica gel) from pure hexane to a mixture of 20% of ethyl acetate in hexane affording 28.1 mg of a colourless oil as the desired product (67%, *E:Z* > 20:1).

^{1}H NMR (500 MHz, Chloroform-*d*) δ 8.50 (broad s, 2H), 7.54 (dt, *J* = 7.69, 1.74 Hz, 1H), 7.34 – 7.27 (m, 4H), 7.26 – 7.22 (m, 1H), 7.22 – 7.18 (m, 1H), 6.40 (dt, *J* = 15.81, 1.51 Hz, 1H), 6.22 (dt, *J* = 15.81, 6.90 Hz, 1H), 2.80 (t, *J* = 8.07 Hz, 2H), 2.57 – 2.51 (m, 2H).

^{13}C NMR (126 MHz, Chloroform-*d*) δ 149.9, 147.4, 137.5, 137.2, 136.2, 131.2, 129.0, 128.7, 127.3, 126.1, 123.6, 34.6, 33.1.

IR (film): 1575, 1490, 1477, 1421, 964, 912, 740, 713 cm^{-1}.

HRMS (ESI) *m/z* calculated for [M+H$^+$] ($C_{15}H_{16}N$)$^+$: 210.1277, found 210.1277.

Methyl (*E*)-3-styrylbicyclo[1.1.1]pentane-1-carboxylate, **39**

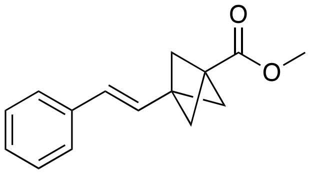

Prepared according to General Procedure 7 using 1-(1,3-dioxoisoindolin-2-yl) 3-methyl bicyclo[1.1.1]pentane-1,3-dicarboxylate, **N31** (63.1 mg, 200 µmol, 1.0 equiv.), (*E*)-2-phenylvinylboronic acid, **1a** (59.2 mg, 400 µmol, 2.0 equiv.), tris(2,2'-bipyridine)ruthenium hexafluorophosphate (1.7 mg, 2.00 µmol, 1 mol%), and *N,N*-dimethylaniline (2.5 µL, 20.0 µmol, 10 mol%) in DMSO-d_6 (1 mL, 0.2 M).

The crude residue was purified by flash chromatography (silica gel) from pure hexane to a mixture of 4% of diethyl ether in hexane affording 30.3 mg of a colourless oil as the desired product (65%, *E:Z* > 20:1).

^{1}H NMR (500 MHz, Chloroform-*d*) δ 7.37 – 7.34 (m, 2H), 7.33 – 7.28 (m, 2H), 7.25 – 7.20 (m, 1H), 6.37 (d, *J* = 15.93 Hz, 1H), 6.28 (d, *J* = 15.96 Hz, 1H), 3.70 (s, 3H), 2.18 (s, 6H).

^{13}C NMR (126 MHz, Chloroform-*d*) δ 170.8, 136.8, 131.2, 128.7, 127.8, 127.7, 126.3, 53.3, 51.8, 40.9, 37.9.

Data are consistent with the literature.[87]

(*E*)-4,4,5,5-Tetramethyl-2-(4-(4-phenylbut-3-en-1-yl)phenyl)-1,3,2-dioxaborolane, **40**

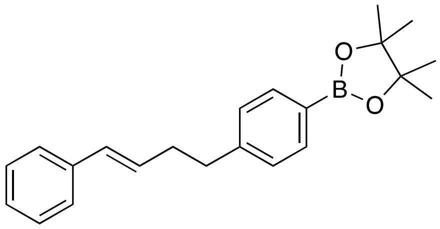

Prepared according to General Procedure 7 using 1,3-dioxoisoindolin-2-yl 3-(4-(4,4,5,5-tetramethyl-1,3,2-dioxaborolan-2-yl)phenyl)propanoate, **N32** (84.3 mg, 200 μmol, 1.0 equiv.), (*E*)-2-phenylvinylboronic acid, **1a** (59.2 mg, 400 μmol, 2.0 equiv.), tris(2,2'-bipyridine)ruthenium hexafluorophosphate (1.7 mg, 2.00 μmol, 1 mol%), and *N,N*-dimethylaniline (2.5 μL, 20.0 μmol, 10 mol%) in DMSO-d_6 (1 mL, 0.2 M). The crude residue was purified by flash chromatography (silica gel) from pure hexane to a mixture of 5% of diethyl ether in hexane affording 44.8 mg of a white solid as the desired product (67%, *E:Z* > 20:1).

^{1}H NMR (400 MHz, Chloroform-*d*) δ 7.82 – 7.77 (m, 2H), 7.39 – 7.30 (m, 4H), 7.30 – 7.26 (m, 2H), 7.26 – 7.21 (m, 1H), 6.45 (dt, *J* = 15.87, 1.46 Hz, 1H), 6.28 (dt, *J* = 15.86, 6.83 Hz, 1H), 2.84 (t, *J* = 7.75 Hz, 2H), 2.62 – 2.52 (m, 2H), 1.37 (d, *J* = 9.54 Hz, 12H).

^{11}B NMR (96 MHz, Chloroform-*d*) δ 31.45.

^{13}C NMR (101 MHz, Chloroform-*d*) δ 145.3, 137.8, 135.0, 130.5, 129.9, 128.6, 128.1, 127.1, 126.1, 83.8, 36.2, 34.9, 25.0. The boron-bearing carbon is not observed due quadrupolar relaxation.

IR (film): 2978, 1448, 1610, 1398, 1388, 1357, 1319, 1271, 1165, 1141, 1087, 962 cm^{-1}.

HRMS (ESI): *m/z* calculated for $[M + H]^+$ $(C_{22}H_{28}BO_2)^+$: 335.2176; found = 335.2171.

(*E*)-1-(2-cyclohexylvinyl)-4-methylbenzene, **42**

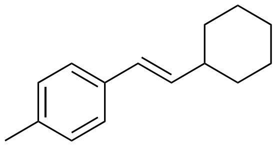

Prepared according to General Procedure 7 using 1,3-dioxoisoindolin-2-yl cyclohexanecarboxylate, **4** (54.7 mg, 200 μmol, 1.0 equiv.), (*E*)-2-(4-methylphenyl)vinylboronic acid (64.8 mg, 400 μmol, 2.0 equiv.), tris(2,2'-bipyridine)ruthenium hexafluorophosphate (1.7 mg, 2.00 μmol, 1 mol%), and *N,N*-dimethylaniline (2.5 μL, 20.0 μmol, 10 mol%) in DMSO-d_6 (1 mL, 0.2 M). The crude residue was purified by flash chromatography (silica gel) with pure hexane affording 29.9 mg of a colourless oil as the desired product (75%, *E:Z* > 20:1).

^{1}H NMR (500 MHz, Chloroform-*d*) δ 7.29 – 7.21 (m, 2H), 7.14 – 7.08 (m, 2H), 6.33 (dd, *J* = 16.06, 1.27 Hz, 1H), 6.14 (dd, *J* = 15.95, 6.96 Hz, 1H), 2.34 (s, 3H), 2.17 – 2.08 (m, 1H), 1.85 – 1.74 (m, 4H), 1.73 – 1.66 (m, 1H), 1.39 – 1.26 (m, 2H), 1.26 – 1.08 (m, 3H).

^{13}C NMR (126 MHz, Chloroform-*d*) δ 136.5, 136.0, 135.4, 129.3, 127.1, 125.9, 41.3, 33.1, 26.3, 26.2, 21.3.

Data are consistent with the literature.[100]

(*E*)-1-chloro-4-(2-cyclohexylvinyl)benzene, **43**

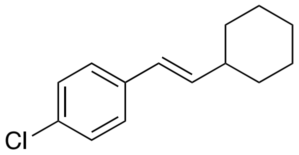

Prepared according to General Procedure 7 using 1,3-dioxoisoindolin-2-yl cyclohexanecarboxylate, **4** (54.7 mg, 200 µmol, 1.0 equiv.), (*E*)-2-(4-chlorophenyl)vinylboronic acid (73.0 mg, 400 µmol, 2.0 equiv.), tris(2,2'-bipyridine)ruthenium hexafluorophosphate (1.7 mg, 2.00 µmol, 1 mol%), and *N,N*-dimethylaniline (2.5 µL, 20.0 µmol, 10 mol%) in DMSO-d_6 (1 mL, 0.2 M). The crude residue was purified by flash chromatography (silica gel) with pure hexane affording 32.0 mg of a colourless oil as the desired product (72%, *E:Z* > 20:1).

^{1}H NMR (500 MHz, Chloroform-*d*) δ 7.36 – 7.21 (m, 4H), 6.32 (dd, *J* = 15.94, 1.19 Hz, 1H), 6.18 (dd, *J* = 15.97, 6.91 Hz, 1H), 2.20 – 2.10 (m, 1H), 1.88 – 1.77 (m, 4H), 1.77 – 1.66 (m, 1H), 1.41 – 1.30 (m, 2H), 1.28 – 1.12 (m, 3H).

^{13}C NMR (126 MHz, Chloroform-*d*) δ 137.7, 136.7, 132.3, 128.7, 127.3, 126.2, 41.3, 33.0, 26.3, 26.1.

Data are consistent with the literature.[100]

(*E*)-1-(2-cyclohexylvinyl)-4-methoxybenzene, **44**

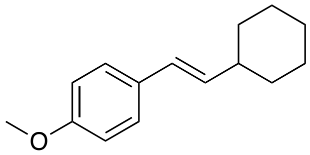

Prepared according to General Procedure 7 using 1,3-dioxoisoindolin-2-yl cyclohexanecarboxylate, **4** (54.7 mg, 200 µmol, 1.0 equiv.), (*E*)-2-(4-methoxyphenyl)vinylboronic acid (71.2 mg, 400 µmol, 2.0 equiv.), tris(2,2'-bipyridine)ruthenium hexafluorophosphate (1.7 mg, 2.00 µmol, 1 mol%), and *N,N*-dimethylaniline (2.5 µL, 20.0 µmol, 10 mol%) in DMSO-d_6 (1 mL, 0.2 M). The crude residue was purified by flash chromatography (silica gel) from pure hexane to 1% of diethyl ether in hexane affording 15.8 mg of a white solid as the desired product (35%, *E:Z* > 20:1).

^{1}H NMR (700 MHz, Chloroform-*d*) δ 7.30 – 7.22 (m, 2H), 6.85 – 6.77 (m, 2H,), 6.29 (dd *J* = 15.99, 1.30 Hz, 1H), 6.04 (dd, *J* = 15.96, 6.99 Hz, 1H), 3.80 (s, 3H), 2.14 – 2.05 (m, 1H), 1.83 – 1.72 (m, 4H), 1.72 – 1.62 (m, 1H), 1.36 – 1.26 (m, 2H), 1.24 – 1.11 (m, 3H).

^{13}C NMR (176 MHz, Chloroform-*d*) δ 158.7, 135.0, 131.0, 127.1, 126.7, 114.0, 55.4, 41.3, 33.2, 26.3, 26.2.

Data are consistent with the literature.[100]

(*E*)-1-(2-cyclohexylvinyl)-4-fluorobenzene, **45**

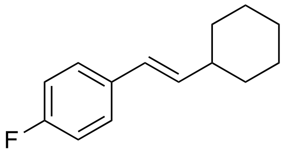

Prepared according to General Procedure 7 using 1,3-dioxoisoindolin-2-yl cyclohexanecarboxylate, **4** (54.7 mg, 200 µmol, 1.0 equiv.), (*E*)-2-(4-fluorophenyl)vinylboronic acid (66.4 mg, 400 µmol, 2.0 equiv.), tris(2,2'-bipyridine)ruthenium hexafluorophosphate (1.7 mg, 2.00 µmol, 1 mol%), *N,N*-dimethylaniline (2.5 µL, 20.0 µmol, 10 mol%) in DMSO-d_6 (1 mL, 0.2 M). The crude residue was purified by flash chromatography (silica gel) with pure hexane affording 29.9 mg of a colourless oil as the desired product (73%, *E:Z* > 20:1).

^{1}H NMR (500 MHz, Chloroform-*d*) δ 7.34 – 7.27 (m, 2H), 7.01 – 6.94 (m, 2H), 6.30 (bd, *J* = 15.97, 1H), 6.09 (dd, *J* = 15.96, 6.99 Hz, 1H), 2.17 – 2.07 (m, 1H), 1.85 – 1.73 (m, 4H), 1.73 – 1.63 (m, 1H), 1.40 – 1.25 (m, 2H), 1.25 – 1.09 (m, 3H).

^{13}C NMR (126 MHz, Chloroform-*d*) δ 162.0 (d, $^1J_{CF}$ = 245.4 Hz), 136.7 (d, $^5J_{CF}$ = 2.2 Hz), 134.3 (d, $^4J_{CF}$ = 3.2 Hz), 127.4 (d, $^3J_{CF}$ = 7.8 Hz), 126.2, 115.4 (d, $^2J_{CF}$ = 21.4 Hz), 41.3, 33.1, 26.3, 26.2.

^{19}F {^{1}H} NMR (376 MHz, Chloroform-*d*) δ –116.01.

Data are consistent with the literature.[100]

(*E*)-4-(2-Cyclohexylvinyl)-1,1'-biphenyl, **46**

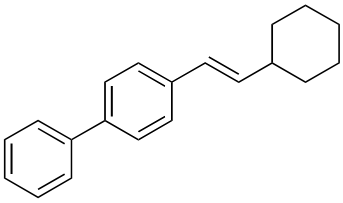

Prepared according to General Procedure 7 using 1,3-dioxoisoindolin-2-yl cyclohexanecarboxylate, **4** (54.7 mg, 200 µmol, 1.0 equiv.), (*E*)-2-(4-biphenyl)vinylboronic acid (89.6 mg, 400 µmol, 2.0 equiv.), tris(2,2'-bipyridine)ruthenium hexafluorophosphate (1.7 mg, 2.00 µmol, 1 mol%), and *N,N*-dimethylaniline (2.5 µL, 20.0 µmol, 10 mol%) in DMSO-d_6 (1 mL, 0.2 M). The crude residue was purified by flash chromatography (silica gel) with pure hexane affording 42.6 mg of a white solid as the desired product (81%, *E:Z* > 20:1).

^{1}H NMR (500 MHz, Chloroform-*d*) δ 7.65 – 7.61 (m, 2H), 7.58 – 7.55 (m, 2H), 7.48 – 7.43 (m, 4H), 7.38 – 7.34 (m, 1H), 6.42 (d, *J* = 16.00, 1H), 6.26 (dd, *J* = 15.97, 6.95 Hz, 1H), 2.23 – 2.14 (m, 1H), 1.89 – 1.77 (m, 4H), 1.76 – 1.70 (m, 1H), 1.43 – 1.32 (m, 2H), 1.31 – 1.15 (m, 3H).

^{13}C NMR (126 MHz, Chloroform-*d*) δ 141.0, 139.6, 137.3, 137.2, 128.9, 127.3, 127.2, 127.0, 126.9, 126.5, 41.4, 33.1, 26.3, 26.2.

Data are consistent with the literature.[101]

(*E*)-4-(2-Cyclohexylvinyl)benzonitrile, **47**

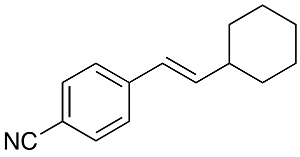

Prepared according to General Procedure 7 using 1,3-dioxoisoindolin-2-yl cyclohexanecarboxylate, **4** (54.7 mg, 200 μmol, 1.0 equiv.), (*E*)-(4-cyanostyryl)boronic acid, **S1** (69.2 mg, 400 μmol, 2.0 equiv.), tris(2,2'-bipyridine)ruthenium hexafluorophosphate (1.7 mg, 2.00 μmol, 1 mol%), and *N,N*-dimethylaniline (2.5 μL, 20.0 μmol, 10 mol%) in DMSO-d_6 (1 mL, 0.2 M). The crude residue was purified by flash chromatography (silica gel) from pure hexane to a mixture of 2% of diethyl ether in hexane affording 36.9 mg of a colourless oil as the desired product (88%, *E:Z* > 20:1).

^{1}H NMR (400 MHz, Chloroform-*d*) δ 7.59 – 7.53 (m, 2H), 7.44 – 7.38 (m, 2H), 6.38 – 6.27 (m, 2H), 2.22 – 2.11 (m, 1H), 1.86 – 1.73 (m, 4H), 1.75 – 1.64 (m, 1H), 1.38 – 1.27 (m, 2H), 1.27 – 1.13 (m, 3H).

^{13}C NMR (101 MHz, Chloroform-*d*) δ 142.8, 141.1, 132.4, 126.5, 126.1, 119.3, 110.0, 41.4, 32.8, 26.2, 26.0.

Data are consistent with the literature.[86]

(*E*)-1-(2-Cyclohexylvinyl)-3-methoxybenzene, **48**

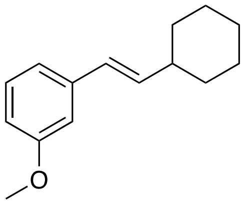

Prepared according to General Procedure 7 using 1,3-dioxoisoindolin-2-yl cyclohexanecarboxylate, **4** (54.7 mg, 200 μmol, 1.0 equiv.), (*E*)-(3-methoxystyryl)boronic acid, **S2** (71.2 mg, 400 μmol, 2.0 equiv.), tris(2,2'-bipyridine)ruthenium hexafluorophosphate (1.7 mg, 2.00 μmol, 1 mol%), and *N,N*-dimethylaniline (2.5 μL, 20.0 μmol, 10 mol%) in DMSO-d_6 (1 mL, 0.2 M). The crude residue was purified by flash chromatography (silica gel) from pure hexane to a mixture of 4% of diethyl ether in hexane affording 37.9 mg of a colourless oil as the desired product (88%, *E:Z* = 20:1.6).

^{1}H NMR (500 MHz, Chloroform-*d*) δ 7.22 (t, *J* = 7.90 Hz, 1H), 6.99 – 6.94 (m, 1H), 6.91 (dd, *J* = 2.61, 1.58 Hz, 1H), 6.76 (ddd, *J* = 8.21, 2.60, 0.94 Hz, 1H), 6.33 (d, *J* = 15.96 Hz, 1H), 6.19 (dd, *J* = 15.94, 6.93 Hz, 1H), 3.82 (s, 3H), 2.20 – 2.09 (m, 1H), 1.87 – 1.74 (m, 4H), 1.73 – 1.66 (m, 1H), 1.41 – 1.28 (m, 2H), 1.25 – 1.16 (m, 3H).

^{13}C NMR (126 MHz, Chloroform-*d*) δ 159.9, 139.7, 137.3, 129.5, 127.2, 118.8, 112.5, 111.3, 55.3, 41.3, 33.0, 26.3, 26.2.

Data are consistent with the literature.[102]

(*E*)-*N*-(3-(2-Cyclohexylvinyl)phenyl)acetamide, **49**

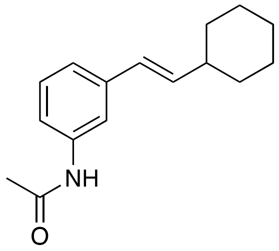

Prepared according to General Procedure 7 using 1,3-dioxoisoindolin-2-yl cyclohexanecarboxylate, **4** (27.3 mg, 100 µmol, 1.0 equiv.), (*E*)-(3-acetamidostyryl)boronic acid, **S3** (41.0 mg, 200 µmol, 2.0 equiv.), tris(2,2'-bipyridine)ruthenium hexafluorophosphate (0.9 mg, 1.00 µmol, 1 mol%), and *N,N*-dimethylaniline (1.3 µL, 10.0 µmol, 10 mol%) in DMSO-d_6 (1 mL, 0.1 M). The crude residue was purified by flash chromatography (silica gel) from pure hexane to a mixture of 20% of ethyl acetate in hexane affording 10.8 mg of a colourless oil as the desired product (44%, *E:Z* = 20:1.2).

^{1}H NMR (500 MHz, Chloroform-*d*) δ 7.51 (t, *J* = 1.94 Hz, 1H), 7.30 (dt, *J* = 8.05, 1.62 Hz, 1H), 7.22 (t, *J* = 7.80 Hz, 1H), 7.09 (d, *J* = 7.67 Hz, 1H), 6.30 (d, *J* = 15.98 Hz, 1H), 6.17 (dd, *J* = 15.95, 6.89 Hz, 1H), 2.17 (s, 3H), 2.14 – 2.07 (m, 1H), 1.83 – 1.73 (m, 4H), 1.71 – 1.64 (m, 1H), 1.35 – 1.24 (m, 2H), 1.23 – 1.12 (m, 3H). Amide proton is not observed.

^{13}C NMR (126 MHz, Chloroform-*d*) δ 168.5, 139.2, 138.2, 137.7, 129.2, 126.9, 122.2, 118.3, 117.4, 41.3, 33.0, 26.3, 26.2, 24.8.

IR (film): 2922, 2848, 1664, 1608, 1585, 1552, 1485, 1444, 1431, 1371, 1319, 1301, 962 cm^{-1}.

HRMS (MALDI): *m/z* calculated for $[M + H]^+$ $(C_{16}H_{22}NO)^+$: 244.1696; found = 244.1696.

Methyl (*E*)-3-(2-cyclohexylvinyl)benzoate, **50**

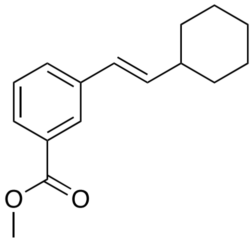

Prepared according to General Procedure 7 using 1,3-dioxoisoindolin-2-yl cyclohexanecarboxylate, **4** (54.7 mg, 200 µmol, 1.0 equiv.), (*E*)-(3-(methoxycarbonyl)styryl)boronic acid, **S4** (82.4 mg, 400 µmol, 2.0 equiv.), tris(2,2'-bipyridine)ruthenium hexafluorophosphate (1.7 mg, 2.00 µmol, 1 mol%), and *N,N*-dimethylaniline (2.5 µL, 20.0 µmol, 10 mol%) in DMSO-d_6 (1 mL, 0.2 M).The crude residue was purified by flash chromatography (silica gel) from pure hexane to a mixture of 2% of diethyl ether in hexane affording 36.7 mg of a colourless oil as the desired product (76%, *E:Z* = 20:1).

^{1}H NMR (500 MHz, Chloroform-*d*) δ 8.03 (t, *J* = 1.82 Hz, 1H), 7.85 (dt, *J* = 7.70, 1.40 Hz, 1H), 7.51 (dt, *J* = 7.68, 1.54 Hz, 1H), 7.35 (t, *J* = 7.72 Hz, 1H), 6.37 (dd, *J* = 16.03, 1.13 Hz, 1H), 6.26 (dd, *J* = 15.98, 6.86 Hz, 1H), 3.9 (s, 3H) 2.20 – 2.09 (m, 1H), 1.85 – 1.74 (m, 4H), 1.72 – 1.65 (m, 1H), 1.38 – 1.26 (m, 2H), 1.26 – 1.14 (m, 3H).

^{13}C NMR (126 MHz, Chloroform-*d*) δ 167.3, 138.5, 138.3, 130.5, 130.4, 128.6, 127.8, 127.1, 126.4, 52.2, 41.3, 33.0, 26.2, 26.1.

IR (film): 2922, 2850, 1720, 1440, 1286, 1265, 1253, 1199, 1105, 964, 748 cm^{-1}.

HRMS (MALDI): *m/z* calculated for $[M + Na]^+$ $(C_{16}H_{20}NaO_2)^+$: 267.1356; found = 267.1355.

(*E*)-1-(2-(2-Cyclohexylvinyl)phenyl)ethan-1-one, **51**

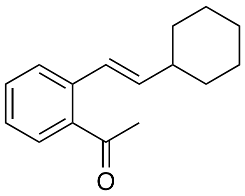

Prepared according to General Procedure 7 using 1,3-dioxoisoindolin-2-yl cyclohexanecarboxylate, **4** (54.7 mg, 200 µmol, 1.0 equiv.), mixture of (*E*) and (*Z*)-(2-acetylstyryl)boronic acid, **S5** (76.0 mg, 400 µmol, 2.0 equiv.), tris(2,2'-bipyridine)ruthenium hexafluorophosphate (1.7 mg, 2.00 µmol, 1 mol%), and *N,N*-dimethylaniline (2.5 µL, 20.0 µmol, 10 mol%) in DMSO-d_6 (1 mL, 0.2 M). The crude residue was purified by flash chromatography (silica gel) from pure hexane to a mixture of 2% of diethyl ether in hexane affording 15.9 mg of a colourless oil as the desired product (35%, *E:Z* > 20:1).

^{1}H NMR (500 MHz, Chloroform-*d*) δ 7.56 (dd, *J* = 7.75, 1.20 Hz, 1H), 7.53 – 7.48 (m, 1H), 7.40 (td, *J* = 7.80, 1.39, 1H), 7.26 (td, *J* = 7.53, 1.28 Hz, 1H), 6.81 (d, *J* = 15.93, 1H), 6.05 (dd, *J* = 15.85, 6.89 Hz, 1H), 2.56 (s, 3H), 2.22 – 2.11 (m, 1H), 1.86 – 1.79 (m, 2H), 1.80 – 1.72 (m, 2H), 1.72 – 1.64 (m, 1H), 1.37 – 1.25 (m, 2H), 1.25 – 1.13 (m, 3H).

^{13}C NMR (126 MHz, Chloroform-*d*) δ 203.0, 140.2, 137.9, 137.6, 131.4, 128.6, 127.6, 126.6, 126.1, 41.4, 32.9, 30.3, 26.3, 26.1.

Data are consistent with the literature.[103]

(*E*)-1-Bromo-2-(2-cyclohexylvinyl)benzene, **52**

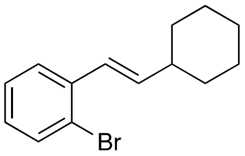

Prepared according to General Procedure 7 using 1,3-dioxoisoindolin-2-yl cyclohexanecarboxylate, **4** (54.7 mg, 200 µmol, 1.0 equiv.), (*E*)-(2-bromostyryl)boronic acid, **S16** (90.7 mg, 400 µmol, 2.0 equiv.), tris(2,2'-bipyridine)ruthenium hexafluorophosphate (1.7 mg, 2.00 µmol, 1 mol%), and *N,N*-dimethylaniline (2.5 µL, 20.0 µmol, 10 mol%) in DMSO-d_6 (1 mL, 0.2 M). The crude residue was purified by flash chromatography (silica gel) with pure hexane affording 45.3 mg of a colourless oil as the desired product (86%, *E:Z* = 20:1.4).

^{1}H NMR (400 MHz, Chloroform-*d*) δ 7.55 – 7.47 (m, 2H), 7.27 – 7.21 (m, 1H), 7.05 (td, *J* = 7.84, 1.70 Hz, 1H), 6.68 (d, *J* = 15.82 Hz, 1H), 6.12 (dd, *J* = 15.85, 6.95 Hz, 1H), 2.26 – 2.14 (m, 1H), 1.89 – 1.74 (m, 4H), 1.74 – 1.65 (m, 1H), 1.41 – 1.28 (m, 2H), 1.28 – 1.13 (m, 3H).

^{13}C NMR (101 MHz, Chloroform-*d*) δ 140.0, 137.9, 132.9, 128.2, 127.5, 126.9, 126.4, 123.5, 41.4, 33.0, 26.3, 26.1.

Data are consistent with the literature.[101]

(*E*)-1-(2-Cyclohexylvinyl)naphthalene, **53**

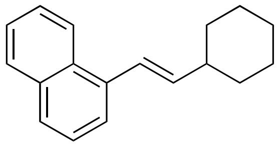

Prepared according to General Procedure 7 using 1,3-dioxoisoindolin-2-yl cyclohexanecarboxylate, **4** (54.7 mg, 200 µmol, 1.0 equiv.), (*E*)-(2-(naphthalen-1-yl)vinyl)boronic acid, **S7** (79.2 mg, 400 µmol, 2.0 equiv.), tris(2,2'-bipyridine)ruthenium hexafluorophosphate (1.7 mg, 2.00 µmol, 1 mol%), and *N,N*-dimethylaniline (2.5 µL, 20.0 µmol, 10 mol%) in DMSO-d_6 (1 mL, 0.2 M). The crude residue was purified by flash chromatography (silica gel) with pure hexane affording 32.8 mg of a colourless oil as the desired product (69%, *E:Z* > 20:1).

^{1}H NMR (500 MHz, Chloroform-*d*) δ 8.19 – 8.11 (m, 1H), 7.84 (dd, *J* = 8.08, 1.57 Hz, 1H), 7.77 – 7.71 (m, 1H), 7.57 (dt, *J* = 7.17, 1.00 Hz, 1H), 7.54 – 7.45 (m, 2H), 7.43 (t, *J* = 7.71 Hz, 1H), 7.09 (d, *J* = 15.65 Hz, 1H), 6.21 (dd, *J* = 15.69, 6.89 Hz, 1H), 2.33 – 2.21 (m, 1H), 1.96 – 1.88 (m, 2H), 1.87 – 1.78 (m, 2H), 1.77 – 1.68 (m, 1H), 1.44 – 1.33 (m, 2H), 1.33 – 1.22 (m, 3H).

^{13}C NMR (126 MHz, Chloroform-*d*) δ 140.4, 136.0, 133.7, 131.3, 128.6, 127.3, 125.9, 125.8, 125.7, 124.5, 124.1, 123.6, 41.7, 33.2, 26.4, 26.2.

Data are consistent with the literature.[104]

(*E*)-2-(2-Cyclohexylvinyl)pyridine, **54**

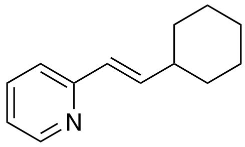

Prepared according to General Procedure 7 using 1,3-dioxoisoindolin-2-yl cyclohexanecarboxylate, **4** (54.7 mg, 200 µmol, 1.0 equiv.), (*E*)-(2-(pyridin-2-yl)vinyl)boronic acid, **S8** (59.6 mg, 400 µmol, 2.0 equiv.), tris(2,2'-bipyridine)ruthenium hexafluorophosphate (1.7 mg, 2.00 µmol, 1 mol%), and *N,N*-dimethylaniline (2.5 µL, 20.0 µmol, 10 mol%) in DMSO-d_6 (1 mL, 0.2 M). The crude residue was purified by flash chromatography (silica gel) from pure hexane to a mixture of 10% of diethyl ether in hexane affording 18.6 mg of a colourless oil as the desired product (51%, *E:Z* > 20:1).

^{1}H NMR (500 MHz, Chloroform-*d*) δ 8.54 – 8.48 (m, 1H), 7.58 (td, *J* = 7.69, 1.88 Hz, 1H), 7.23 (dd, *J* = 7.88, 1.35 Hz, 1H), 7.07 (ddd, *J* = 7.55, 4.88, 1.17 Hz, 1H), 6.69 (dd, *J* = 15.84, 6.95 Hz, 1H), 6.43 (dd, *J* = 15.82, 1.32 Hz, 1H), 2.22 – 2.14 (m, 1H), 1.88 – 1.80 (m, 2H), 1.80 – 1.74 (m, 1H), 1.71 – 1.63 (m, 2H), 1.37 – 1.27 (m, 2H), 1.27 – 1.15 (m, 3H).

^{13}C NMR (126 MHz, Chloroform-*d*) δ 156.4, 149.5, 141.6, 136.5, 127.5, 121.6, 121.2, 41.1, 32.7, 26.2, 26.1.

Data are consistent with the literature.[105]

(*E*)-2-(2-Cyclohexylvinyl)thiophene, **55**

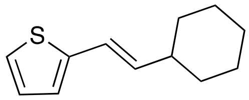

Prepared according to General Procedure 7 using 1,3-dioxoisoindolin-2-yl cyclohexanecarboxylate, **4** (54.7 mg, 200 µmol, 1.0 equiv.), (*E*)-(2-(thiophen-2-yl)vinyl)boronic acid, **S9** (61.6 mg, 400 µmol, 2.0 equiv.), tris(2,2'-bipyridine)ruthenium hexafluorophosphate (1.7 mg, 2.00 µmol, 1 mol%), and *N,N*-dimethylaniline (2.5 µL, 20.0 µmol, 10 mol%) in DMSO-d_6 (1 mL, 0.2 M). The crude residue was purified by flash chromatography (silica gel) from pure hexane to a mixture of 4% of diethyl ether in hexane affording 30.5 mg of a colourless oil as the desired product (79%, *E:Z* = 20:3).

(*E*)-2-(2-Cyclohexylvinyl)thiophene

^{1}H NMR (500 MHz, Chloroform-*d*) δ 7.08 (d, *J* = 5.06 Hz, 1H), 6.93 (dd, *J* = 5.12, 3.47 Hz, 1H), 6.87 (d, *J* = 3.49 Hz, 1H), 6.47 (d, *J* = 15.82 Hz, 1H), 6.04 (dd, *J* = 15.81, 6.91 Hz, 1H), 2.15 – 2.06 (m, 1H), 1.85 – 1.73 (m, 4H), 1.72 – 1.65 (m, 1H), 1.40 – 1.26 (m, 2H), 1.24 – 1.11 (m, 3H).

^{13}C NMR (126 MHz, Chloroform-*d*) δ 143.6, 136.9, 127.3, 124.3, 123.1, 120.7, 41.1, 32.9, 26.3, 26.1.

Data are consistent with the literature.[102]

(*Z*)-2-(2-Cyclohexylvinyl)thiophene

^{1}H NMR (500 MHz, Chloroform-*d*) not reported since mostly overlapped with the *E* product.

^{13}C NMR (126 MHz, Chloroform-*d*) δ 140.6, 137.1, 127.2, 126.8, 124.9, 119.9, 38.1, 32.9, 26.1, 26.0.

(*E*)-5-(2-Cyclohexylvinyl)benzofuran, **56**

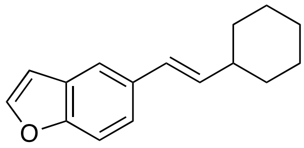

Prepared according to General Procedure 7 using 1,3-dioxoisoindolin-2-yl cyclohexanecarboxylate, **4** (54.7 mg, 200 µmol, 1.0 equiv.), (*E*)-(2-(benzofuran-5-yl)vinyl)boronic acid, **S10** (75.2 mg, 400 µmol, 2.0 equiv.), tris(2,2'-bipyridine)ruthenium hexafluorophosphate (1.7 mg, 2.00 µmol, 1 mol%), and *N,N*-dimethylaniline (2.5 µL, 20.0 µmol, 10 mol%) in DMSO-d_6 (1 mL, 0.2 M). The crude residue was purified by flash chromatography (silica gel) from pure hexane to a mixture of 1% of diethyl ether in hexane affording 39.4 mg of a pale-yellow oil as the desired product (87%, *E:Z* > 20:1).

^{1}H NMR (400 MHz, Chloroform-*d*) δ 7.59 (d, *J* = 2.16 Hz, 1H), 7.56 (d, *J* = 1.77 Hz, 1H), 7.43 (dt, *J* = 8.56, 0.83 Hz, 1H), 7.34 (dd, *J* = 8.59, 1.81 Hz, 1H), 6.73 (dd, *J* = 2.21, 0.96 Hz, 1H), 6.45 (dd, *J* = 15.96, 1.27 Hz, 1H), 6.16 (dd, *J* = 15.91, 6.99 Hz, 1H), 2.21 – 2.11 (m, 1H), 1.90 – 1.75 (m, 4H), 1.75 – 1.67 (m, 1H), 1.42 – 1.30 (m, 2H), 1.29 – 1.15 (m, 3H).

^{13}C NMR (101 MHz, Chloroform-*d*) δ 154.4, 145.4, 135.9, 133.3, 127.8, 127.4, 122.7, 118.5, 111.3, 106.7, 41.3, 33.2, 26.3, 26.2.

IR (film): 2918, 2845, 1465, 1448, 1440, 1259, 1192, 1122, 1105, 1028, 962, 857 cm^{-1}.

HRMS (EI): *m/z* calculated for $[M]^+$ $(C_{16}H_{18}O)^+$: 226.1352; found = 226.1350.

(*E*)-2-Bromo-1-(2-cyclohexylvinyl)-4-(trifluoromethoxy)benzene, **57**

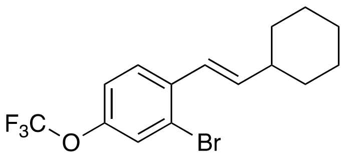

Prepared according to General Procedure 7 using 1,3-dioxoisoindolin-2-yl cyclohexanecarboxylate, **4** (54.7 mg, 200 μmol, 1.0 equiv.), (*E*)-(2-bromo-4-(trifluoromethoxy)styryl)boronic acid, **S11** (124 mg, 400 μmol, 2.0 equiv.), tris(2,2'-bipyridine)ruthenium hexafluorophosphate (1.7 mg, 2.00 μmol, 1 mol%), and *N*,*N*-dimethylaniline (2.5 μL, 20.0 μmol, 10 mol%) in DMSO-d_6 (1 mL, 0.2 M). The crude residue was purified by flash chromatography (silica gel) with pure hexane affording 68.3 mg of a colourless oil as the desired product (98%, *E:Z* > 20:1).

^{1}H NMR (400 MHz, Chloroform-*d*) δ 7.50 (d, *J* = 8.66 Hz, 1H), 7.42 (dd, *J* = 2.43, 1.09 Hz, 1H), 7.12 (ddt, *J* = 8.73, 2.60, 0.93 Hz, 1H), 6.63 (dd, *J* = 15.84, 1.37 Hz, 1H), 6.11 (dd, *J* = 15.85, 6.94 Hz, 1H), 2.27 – 2.12 (m, 1H), 1.89 – 1.75 (m, 4H), 1.75 – 1.64 (m, 1H), 1.41 – 1.28 (m, 2H), 1.27 – 1.14 (m, 3H).

^{13}C NMR (176 MHz, Chloroform-*d*) δ 147.9, 141.0, 137.0, 127.5, 125.4, 125.3, 123.2, 120.5 (q, $^1J_{CF}$ = 257.8 Hz), 120.2, 41.4, 32.9, 26.2, 26.1.

^{19}F {1H} NMR (377 MHz, Chloroform-*d*) δ –58.03.

IR (film): 2924, 2852, 1597, 1448, 1481, 1247, 1213, 1161, 964 cm^{-1}.

HRMS (MALDI): *m/z* calculated for $[M + H]^+$ $(C_{15}H_{17}BrF_3O)^+$: 349.0409; found = 349.0411.

((1*E*,3*E*) and (1*Z*,3*E*)-4-Cyclohexylbuta-1,3-dien-1-yl)benzene, **58**

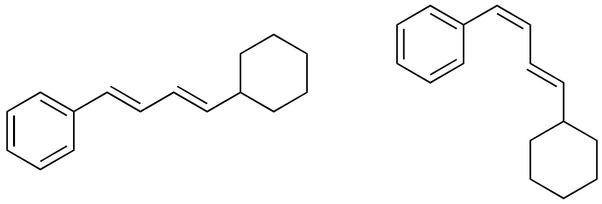

Prepared according to General Procedure 7 using 1,3-dioxoisoindolin-2-yl cyclohexanecarboxylate, **4** (27.3 mg, 100 μmol, 1.0 equiv.), ((1*E*,3*E*)-4-phenylbuta-1,3-dien-1-yl)boronic acid, **S12** (34.8 mg, 200 μmol, 2.0 equiv.), tris(2,2'-bipyridine)ruthenium hexafluorophosphate (0.9 mg, 1.00 μmol, 1 mol%), and *N*,*N*-dimethylaniline (1.3 μL, 10.0 μmol, 10 mol%) in DMSO-d_6 (1 mL, 0.1 M). The reaction was not complete and 46% of the NHPI was remaining. The crude residue was purified by flash chromatography (silica gel) with hexane affording 6.1 mg of a colourless oil as a mixture of desired product (27%, ratio EE/ZE/EZ/ZZ: 1:1:0.1:0.1).

Data for (**1*E*,3*E***) and (1*Z*,3*E*) as a 1:1 mixture:

^{1}H NMR (500 MHz, Chloroform-*d*) δ 7.39 – 7.35 (m, 2H), 7.35 – 7.32 (m, 4H), 7.31 – 7.27 (m, 2H), 7.25 – 7.17 (m, **1H** + 1H), 6.75 (dd, *J* = 15.65, 10.40 Hz, **1H**), 6.58 (ddt, *J* = 15.22, 11.07, 1.13 Hz, 1H), 6.45 (d, *J* = 15.59 Hz, **1H**), 6.31 (d, *J* = 11.55 Hz, 1H), 6.20 (t, *J* = 11.37 Hz, 1H), 6.21 – 6.14 (m, **1H**), 5.82 (dd, *J* = 15.21, 7.06 Hz, 1H), 5.79 (dd, *J* = 15.31, 6.98 Hz, **1H**), 2.11 – 2.01 (m, **1H** + 1H), 1.80 – 1.69 (m, **4H** + 4H), 1.69 – 1.63 (m, **1H** + 1H), 1.37 – 1.22 (m, **2H** + 2H), 1.22 – 1.08 (m, **3H** + 3H).

^{13}C NMR (126 MHz, Chloroform-*d*) δ 144.0 (C), 141.9. (**C**), 138.0, 137.9, 131.0 (C), 130.2 (**C**), 129.9 (**C**), 129.0, 128.7, 128.3, 128.1 (**C**), 127.8 (C), 127.2, 126.7, 126.2, 124.1 (C), 41.2, 41.1, 33.0, 32.9, 26.3, 26.2, 26.1, 26.1.

Data (for 1*E*,3*E*) are consistent with the literature.[106]

Methyl (*E*)-2-(2-cyclohexylvinyl)benzoate, **59**

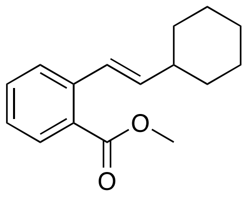

Prepared according to General Procedure 7 using 1,3-dioxoisoindolin-2-yl cyclohexanecarboxylate, **4** (27.3 mg, 100 μmol, 1.0 equiv.), (*E*)-(2-(methoxycarbonyl)styryl)boronic acid, **S13** (41.2 mg, 200 μmol, 2.0 equiv.), tris(2,2'-bipyridine)ruthenium hexafluorophosphate (0.9 mg, 1.00 μmol, 1 mol%), and *N*,*N*-dimethylaniline (1.3 μL, 10.0 μmol, 10 mol%) in DMSO-d_6 (1 mL, 0.1 M). The crude residue was purified by flash chromatography (silica gel) with hexane affording 14.8 mg of a colourless oil as the desired product (61%, *E*:*Z* > 20:1).

^{1}H NMR (500 MHz, Chloroform-*d*) δ 7.84 (dd, *J* = 7.83, 1.48 Hz, 1H), 7.54 (d, *J* = 7.85 Hz, 1H), 7.43 (td, *J* = 7.83, 1.44 Hz, 1H), 7.24 (td, *J* = 6.48, 1.09 Hz, 1H), 7.11 (d, *J* = 15.82 Hz, 1H), 6.09 (dd, *J* = 15.88, 6.85 Hz, 1H), 3.90 (s, 3H), 2.24 – 2.12 (m, 1H), 1.91 – 1.80 (m, 2H), 1.81 – 1.73 (m, 2H), 1.71 – 1.64 (m, 1H), 1.38 – 1.26 (m, 2H), 1.28 – 1.14 (m, 3H).

^{13}C NMR (126 MHz, Chloroform-*d*) δ 168.3, 140.0, 139.8, 132.0, 130.4, 128.3, 127.2, 126.5, 126.2, 52.1, 41.4, 33.0, 26.3, 26.2.

Data are consistent with the literature.[107]

(*E*)-2-(2-Cyclohexylvinyl)-3'-methoxy-1,1'-biphenyl, **60**

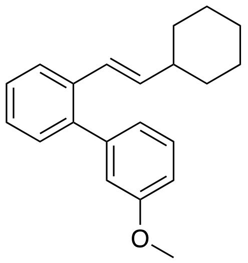

Prepared according to General Procedure 7 using 1,3-dioxoisoindolin-2-yl cyclohexanecarboxylate, **4** (27.3 mg, 100 μmol, 1.0 equiv.), (*E*)-(2-(3'-Methoxy-[1,1'-biphenyl]-2-yl)vinyl)boronic acid, **S14** (50.8 mg, 200 μmol, 2.0 equiv.), tris(2,2'-bipyridine)ruthenium hexafluorophosphate (0.9 mg, 1.00 μmol, 1 mol%), and *N*,*N*-dimethylaniline (1.3 μL, 10.0 μmol, 10 mol%) in DMSO-d_6 (1 mL, 0.1 M). The crude residue was purified by flash chromatography (silica gel) from pure hexane to a mixture of 3% of diethyl ether in hexane affording 19.2 mg of a white solid as the desired product (66%, *E*:*Z* > 20:1).

^{1}H NMR (400 MHz, Chloroform-*d*) δ 7.57 (app. d, *J* = 7.52 Hz, 1H), 7.37 – 7.24 (m, 4H), 6.99 – 6.88 (m, 3H), 6.36 (d, *J* = 15.96 Hz, 1H), 6.09 (dd, *J* = 15.92, 7.02 Hz, 1H), 3.84 (s, 3H), 2.12 – 1.99 (m, 1H), 1.78 – 1.68 (m, 4H), 1.69 – 1.58 (m, 1H), 1.34 – 1.20 (m, 2H), 1.19 – 1.07 (m, 3H).

^{13}C NMR (101 MHz, Chloroform-*d*) δ 159.3, 142.8, 140.3, 137.9, 136.2, 130.1, 129.0, 127.6, 126.8, 126.4, 125.9, 122.5, 115.3, 112.9, 55.4, 41.4, 33.1, 26.3, 26.1.

IR (neat liquid): 2922, 1597,1577, 1446, 1423, 1317, 1290, 1276, 1219, 1213, 1176, 1045, 1022, 966 cm^{-1}.

HRMS (ESI): *m/z* calculated for $[M + H]^+$ $(C_{21}H_{25}O)^+$: = 293.1899; found = 293.1898.

(*E*)-Octa-1,7-dien-1-ylbenzene, **62** and (*E*)-(3-cyclopentylprop-1-en-1-yl)benzene, **63**

Prepared according to General Procedure 7 using 1,3-dioxoisoindolin-2-yl hept-6-enoate, **61** (54.7 mg, 200 µmol, 1.0 equiv.), (*E*)-2-phenylvinylboronic acid, **1a** (59.2 mg, 400 µmol, 2.0 equiv.), tris(2,2'-bipyridine)ruthenium hexafluorophosphate (1.7 mg, 2.00 µmol, 1 mol%), and *N,N*-dimethylaniline (2.5 µL, 20.0 µmol, 10 mol%) in DMSO-d_6 (1 mL, 0.2 M). The crude residue was purified by flash chromatography (silica gel) with pure hexane affording 7.2 mg of **62** as a colourless oil (19%, *E:Z* > 20:1) and 21.1 mg of **63** as a colourless oil (56%, *E:Z* > 20:1).

(*E*)-Octa-1,7-dien-1-ylbenzene, **62**

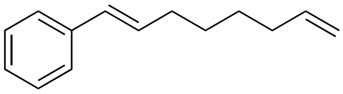

^{1}H NMR (500 MHz, Chloroform-*d*) δ 7.36 – 7.32 (m, 2H), 7.31 – 7.26 (m, 2H), 7.22 – 7.16 (m, 1H), 6.38 (d, *J* = 15.71 Hz, 1H), 6.22 (dt, *J* = 15.79, 6.90 Hz, 1H), 5.82 (ddt, *J* = 16.90, 10.16, 6.64 Hz, 1H), 5.01 (app. dq, *J* = 17.10, 1.74 Hz, 1H), 4.95 (ddt, *J* = 10.21, 2.31, 1.25 Hz, 1H), 2.22 (app. qd, *J* = 6.96, 1.45 Hz, 2H), 2.08 (td, *J* = 7.47, 5.87 Hz, 2H), 1.54 – 1.39 (m, 4H).

^{13}C NMR (126 MHz, Chloroform-*d*) δ 139.1, 138.0, 131.1, 130.0, 128.6, 126.9, 126.0, 114.5, 33.8, 33.0, 29.0, 28.6.

IR (film): 2924, 2852, 1641, 1493, 1460, 991, 692, 455 cm^{-1}.

HRMS (EI): *m/z* calculated for $[M]^+$ $(C_{14}H_{18})^+$: 186.1403; found 186.1408.

(*E*)-(3-Cyclopentylprop-1-en-1-yl)benzene, **63**

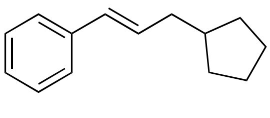

^{1}H NMR (500 MHz, Chloroform-*d*) δ 7.38 – 7.31 (m, 2H), 7.33 – 7.26 (m, 2H), 7.24 – 7.15 (m, 1H), 6.38 (d, *J* = 15.78 Hz, 1H), 6.24 (dt, *J* = 15.69, 7.07 Hz, 1H), 2.22 (dd, *J* = 7.15, 7.24 Hz, 2H), 1.95 (hept, *J* = 7.56 Hz, 1H), 1.83 – 1.72 (m, 2H), 1.71 – 1.59 (m, 2H), 1.59 – 1.46 (m, 2H), 1.25 – 1.14 (m, 2H).

^{13}C NMR (126 MHz, Chloroform-*d*) δ 138.1, 130.7, 130.2, 128.6, 126.9, 126.1, 40.1, 39.6, 32.5, 25.3.

Data are consistent with the literature.[108]

2-cyclohexyl-1-(4-fluorophenyl)ethan-1-one, **75**

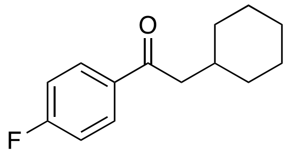

Prepared according to General Procedure 7 using 1,3-dioxoisoindolin-2-yl cyclohexanecarboxylate, **4** (54.7 mg, 200 μmol, 1.0 equiv.), (*E*)-2-phenylvinylboronic acid, **1a** (29.6 mg, 200 μmol, 1.0 equiv.), tris(2,2'-bipyridine)ruthenium hexafluorophosphate (1.7 mg, 2.00 μmol, 1 mol%), *p*-fluorostyrene (23.4 μL, 200 μmol, 1.0 equiv.), and *N,N*-dimethylaniline (2.5 μL, 20.0 μmol, 10 mol%) in DMSO-d_6 (1 mL, 0.2 M). The crude residue was purified by flash chromatography (silica gel) with pure hexane affording 6.5 mg of a colourless oil as the desired product (16%).

^{1}H NMR (500 MHz, Chloroform-*d*) δ 8.01 – 7.94 (m, 2H), 7.16 – 7.09 (m, 2H), 2.79 (d, *J* = 6.79 Hz, 2H), 2.02 – 1.90 (m, 1H), 1.80 – 1.66 (m, 4H), 1.66 – 1.62 (m, 1H), 1.33 – 1.27 (m, 2H), 1.21 – 1.12 (m, 1H), 1.01 (qd, *J* = 12.31, 3.09 Hz, 2H).

^{13}C NMR (126 MHz, Chloroform-*d*) δ 198.8, 165.8 (d, $^{1}J_{CF}$ = 254.3 Hz), 134.0 (d, $^{4}J_{CF}$ = 2.9 Hz,), 130.9 (d, $^{3}J_{CF}$ = 9.3 Hz), 115.7 (d, $^{2}J_{CF}$ = 21.8 Hz), 46.3, 34.7, 33.6, 26.4, 26.3.

^{19}F {1H} NMR (470 MHz, Chloroform-*d*) δ –105.81.

IR (film): 2922, 2850, 1680, 1597, 1506, 1448, 1409, 1354, 1286, 1224, 1193, 1155, 958, 829 cm^{-1}.

HRMS (ESI): *m/z* calculated for [M – H]$^{-}$ ($C_{14}H_{16}FO$)$^{-}$: 219.1190; found = 219.1198.

(*E*)-(1-Cyclohexylprop-1-en-2-yl)benzene, **P1**

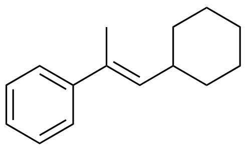

Prepared according to General Procedure 7 using 1,3-dioxoisoindolin-2-yl cyclohexanecarboxylate, **4** (27.3 mg, 100 μmol, 1.0 equiv.), (*E*)-(2-phenylprop-1-en-1-yl)boronic acid, **61** (32.4 mg, 200 μmol, 2.0 equiv.), tris(2,2'-bipyridine)ruthenium hexafluorophosphate (0.9 mg, 1.00 μmol, 1 mol%), and *N,N*-dimethylaniline (1.3 μL, 10.0 μmol, 10 mol%) in DMSO-d_6 (1 mL, 0.1 M). The crude residue was purified by flash chromatography (silica gel) with hexane affording 15.8 mg of a colourless oil as the desired product (67%, *E:Z* > 20:1). It contained 15% of (*E*)-(2-cyclohexylvinyl)benzene, **5** from the reaction between (*E*)-styrylboronic acid, **1a** contained in the starting material. They could not be separated by flash chromatography.

^{1}H NMR (500 MHz, Chloroform-*d*) δ 7.43 – 7.36 (m, 2H), 7.33 – 7.27 (m, 2H), 7.24 – 7.18 (m, 1H), 5.63 (dd, *J* = 9.01, 1.43 Hz, 1H), 2.41 – 2.30 (m, 1H), 2.05 (d, *J* = 1.37 Hz, 3H), 1.81 – 1.64 (m, 5H), 1.40 – 1.28 (m, 2H), 1.24 – 1.09 (m, 3H).

^{13}C NMR (126 MHz, Chloroform-*d*) δ 144.2, 134.7, 132.9, 128.2, 126.6, 125.8, 37.9, 33.2, 26.3, 26.2, 16.0.

Data are consistent with the literature.[109]

tert-Butyl (*E*)-2-styrylindoline-1-carboxylate, **P3** and *tert*-butyl 1*H*-indole-1-carboxylate, **P4**

Prepared according to General Procedure 7 using 1-(*tert*-butyl) 2-(1,3-dioxoisoindolin-2-yl) indoline-1,2-dicarboxylate, **N33** (81.7 mg, 200 μmol, 1.0 equiv.), (*E*)-2-phenylvinylboronic acid, **1a** (59.2 mg, 400 umol, 2.0 equiv.), tris(2,2'-bipyridine)ruthenium hexafluorophosphate (1.7 mg, 2.00 μmol, 1 mol%), and *N*,*N*-dimethylaniline (2.5 μL, 20.0 μmol, 10 mol%) in DMSO-d_6 (1 mL, 0.2 M). The crude residue was purified by flash chromatography (silica gel) from pure hexane to a mixture of 2% of diethyl ether in hexane affording 5.9 mg of **P3** as a colourless oil (9%, *E*:*Z* > 20:1) and 22.3 mg of **P4** as a colourless oil (51%).

tert-Butyl (*E*)-2-styrylindoline-1-carboxylate, **P3**

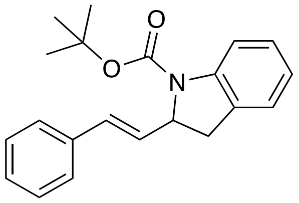

^{1}H NMR (400 MHz, Chloroform-*d*) δ 7.78 (broad s, 1H), 7.35 – 7.32 (m, 2H), 7.31 – 7.26 (m, 2H), 7.24 – 7.17 (m, 2H), 7.17 – 7.12 (m, 1H), 6.96 (td, *J* = 7.45, 1.10 Hz, 1H), 6.52 (d, *J* = 15.74 Hz, 1H), 6.18 (dd, *J* = 15.76, 7.70 Hz, 1H), 5.08 – 4.96 (m, 1H), 3.49 (dd, *J* = 16.20, 10.00 Hz, 1H), 2.88 (dd, *J* = 16.17, 2.79 Hz, 1H), 1.52 (s, 9H).

^{13}C NMR (126 MHz, Chloroform-*d*) δ 152.5, 142.2, 136.8, 130.6, 129.3, 128.7, 127.7, 127.7, 126.6, 125.0, 122.6, 115.3, 81.1, 61.3, 35.1, 28.6. One quaternary carbon could not be observed (signal too weak).

Data are consistent with the literature.[110]

tert-Butyl 1*H*-indole-1-carboxylate, **P4**

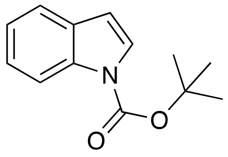

^{1}H NMR (500 MHz, Chloroform-*d*) δ 8.20 – 8.12 (m, 1H), 7.61 (d, *J* = 3.70 Hz, 1H), 7.57 (dt, *J* = 7.66, 1.08 Hz, 1H), 7.34 – 7.30 (m, 1H), 7.26 – 7.21 (m, 1H), 6.58 (d, *J* = 3.74, 1H), 1.68 (s, 9H).

^{13}C NMR (126 MHz, Chloroform-*d*) δ 149.9, 135.3, 130.7, 126.0, 124.3, 122.7, 121.1, 115.3, 107.4, 83.8, 28.3.

Data are consistent with the literature.[111]

7. References

1. J. Wang, M. Shang, H. Lundberg, K. S. Feu, S. J. Hecker, T. Qin; D. G. Blackmond, P. S. Baran, *ACS Catal*. **2018**, *8*, 9537–9542.
2. J. Brauer, E. Quraishi, L. M. Kammer, T. Opatz, *Chem. Eur. J*. **2021**, *27*, 18168–18174.
3. G. Pisella, A. Gagnebin, J. Waser, *Org. Lett*. **2020**, *22*, 3884–3889.
4. J. E. Lee, J. Kwon, J. Yun, *Chem. Commun*. **2008**, *6*, 733–734.
5. R. Alfaro, A. Parra, J. Alemán, J. L. García Ruano, M. Tortosa, *J. Am. Chem. Soc*. **2012**, *134*, 15165–15168.
6. W. Yuan, S. Ma. *Org. Biomol. Chem*, **2012**, *10*, 7266–7268.
7. A. Juneau, T. O. Hope, J. Malenfant, M. Mesko, J. McNeill, M. Frenette, *ACS Catal*. **2022**, *12*, 2348–2356.
8. O. Jin, S. Haug, G. Nguyen, V. Flores-Hansen, C. Arman, H. Larionov, *ACS Catal*. **2019**, *9*, 9764–9774.
9. J. Schwarz, B. König, *Green Chem*. **2016**, *18*, 4743–4749.
10. J. Cornella, J. T. Edwards, T. Qin, S. Kawamura, J. Wang, C. M. Pan, R. Gianatassio, M. Schmidt, M. D. Eastgate, P. S. Baran, *J. Am. Chem. Soc*. **2016**, *138*, 2174–2177.
11. K. M. M. Huihui, J. A. Caputo, Z. Melchor, A. M. Olivares, A. M. Spiewak, K. A. Johnson, T. A. DiBenedetto, S. Kim, L. K. G. Ackerman, D. J. Weix, *J. Am. Chem. Soc.* **2016**, *138*, 5016–5019.
12. W. Zhao, R. P. Wurz, J. C. Peters, G. C. Fu, *J. Am. Chem. Soc.* **2017**, *139*, 12153–12156.
13. X. Xu, J. Sun, Y. Lin, J. Cheng, P. Li, X. Jiang, R., Bai, Y. Xie, *Eur. J. Org. Chem*. **2017**, 7160–7166.
14. P. F. Dai, Y. P. Wang, J. P. Qu, Y. B. Kang, *Org. Lett.* **2021**, *23*, 9360–9364.
15. M. C. Sheikh, S. Takagi, T. Yoshimura, H. Morita, *Tetrahedron* **2010**, *66*, 7272–7278.
16. X. Lu, B. Xiao, L. Liu, Y. Fu, *Chem. Eur. J.* **2016**, *22*, 11161–11164.
17. E. Christiansen, C. Urban, M. Grundmann, M. E. Due-Hansen, Ellen Hagesaether, J. Schmidt, L. Pardo, S. Ullrich, E. Kostenis, M. Kassack, T. Ulven, *J. Med. Chem.* **2011**, *54*, 6691–6703.
18. G. Pratsch, G. L. Lackner, L. E. Overman, *J. Org. Chem*. **2015**, *80*, 6025–6036.
19. X. G. Liu, C. J. Zhou, E. Lin, X. L. Han, S. S. Zhang, Q. Li, H. Wang, *Angew. Chem. Int. Ed*. **2018**, *57*, 13096–13100.
20. S. A. Green, S. Vásquez-Céspedes, R. A. Shenvi, *J. Am. Chem. Soc*. **2018**, *140*, 11317–11324.
21. H. Li, C. P. Breen, H. Seo, T. F. Jamison, Y. Q. Fang, M. M. Bio, *Org. Lett*. **2018**, *20*, 1338–1341.
22. C. C. Chen, S. F. Wang, Y. Y. Su, Y. A. Lin, P. C. Lin, *Chem. Asian J*. **2017**, *12*, 1326–1337.
23. E. C. Garnier-Amblard, S. G. Mays, R. F. Arrendale, M. T. Baillie, A. S. Bushnev, D. G. Culver, T. J. Evers, J. J. Holt, R. B. Howard, L. S. Liebeskind, D. S. Menaldino, M. G. Natchus, J. A. Petros, H Ramaraju, G. P. Reddy, D. C. Liotta, *ACS Med. Chem. Lett.* **2011**, *2*, 438–443.
24. M. L. Shen, Y. Shen, P. S. Wang, *Org. Lett*. **2019**, *21*, 2993–2997.
25. K. J. Hale, Z. Xiong, L. Wang, S. Manaviazar, R. Mackle, *Org. Lett*. **2015**, *17*, 198–201.

26. Y. Jiang, J. Pan, T. Yang, Y. Zhao, M.J. Koh, *Chem*. **2021**, *7*, 993–1005.

27. A. Tlahuext-Aca, R. A. Garza-Sanchez, M. Schäfer, F. Glorius, *Org. Lett*. **2018**, 20, 1546–1549.

28. T. Mani, D. Liu, D. Zhou, L. Li, W. E. Knabe, F. Wang, K. Oh, S. Meroueh, *ChemMedChem* **2013**, *8*, 1963–1977.

29. A. Grenier, M. C., Ding, S., Vézina, D., Chapleau, J. P., Tolbert, W., Sherburn, R., Schön, A., Somisetti, S., Abrams, C., Pazgier, M., Finzi, A. and Smith, *ACS Med. Chem. Lett*. **2020**, *11*, 371–378.

30. A. K. C. Schmidt, C. B. W. Stark, *Org. Lett*. **2011**, *13*, 4164–4167.

31. L. Yu, M. L. Tang, C. M. Si, Z. Meng, Y. Liang, J. Han, X. Sun, *Org. Lett*. **2018**, *20*, 4579–4583.

32. E. Watanabe, Y. Chen, O. May, S. V. Ley, *Chem. Eur. J.* **2020**, *26*, 186–191.

33. H. Song, R. Cheng, Q. Q. Min, X. Zhang, *Org. Lett*. **2020**, *22*, 7747–7751.

34. N. Kvasovs, V. Gevorgyan, *Org. Lett.* **2022**, *24*, 4176−4181.

35. F. Sandfort, M. J. O'Neill, J. Cornella, L. Wimmer, P. S. Baran, *Angew. Chem. Int. Ed*. **2017**, *56*, 3319–3323.

36. F. Toriyama, J. Cornella, L. Wimmer, T. G. Chen, D. D. Dixon, G. Creech, P. S. Baran, *J. Am. Chem. Soc*. **2016**, *138*, 11132–11135.

37. M. J. Bu, C. Cai, F. Gallou, B. H. Lipshutz, *Green Chem*. **2018**, *20*, 1233–1237.

38. T. Qin, L. Malins, J. Edwards, R. Merchant, A. Novak, J. Zhong, R. Mills, M. Yan, C. Yuan, M. Eastgate, P. S. Baran, *Angew. Chem. Int. Ed*. **2017**, *56*, 260–265.

39. D. Balamurugan, K.M. Muraleedharan, *Chem. Eur. J.* **2012**, *18*, 9516–9520.

40. J. T. Correia, G. Piva da Silva, C. M. Kisukuri, E. André, B. Pires, P.S. Carneiro, M. W. Paixão, *J. Org. Chem*. **2020**, *85*, 9820–9834.

41. D. Wang, N. Zhu, P. Chen, Z. Lin, G. Liu, *J. Am. Chem. Soc*. **2017**, *139*, 15632–15635.

42. T. Yang, Y. Jiang, Y. Luo, J. Jun Han Lim, Y. Lan, M. J. Koh, *J. Am. Chem. Soc*. **2020**, *142*, 21410–21419.

43. D. L. Zhu, R. Xu, Q. Wu, H. Y. Li, J. P. Lang, and H. X. Li, *J. Org. Chem*. **2020**, *85*, 9201–9212.

44. M. L. N. Rao, S. S. Islam, *Tetrahedron Lett.* **2021**, *71*, 153051.

45. J. S. Jia, T. X. Wu, Y. J. Fu, Z. R. Hu, H. T. Tang, Y. M. Pan, F. P. Huang, *Adv. Synth. Catal.* **2022**, 364, 1873–1878.

46. Y. Yasu, T. Koike, M. Akita, *Chem. Commun*. **2013**, *49*, 2037–2039.

47. M. Zhong, Y. Gagné, T. O. Hope, X. Pannecoucke, M. Frenette, P. Jubault, T. Poisson, *Angew. Chem. Int. Ed.* **2021**, *60*, 14498–14503.

48. E. J. Linstad, A. L. Vāvere, B. Hu, J. J. Kempinger, S. E. Snyder, S. G. DiMagno, *Org. Biomol. Chem*. **2017**, *15*, 2246–2252.

49. Y. Ly, W. Pu, Q. Chen, Q. Wang, J. Ni, Q. Zhang, *J. Org. Chem*. **2017**, *82*, 8282–8289.

50. D. A. Offermann, J. E. McKendrick, J. J. P. Sejberg, B. Mo, M. D. Holdom, B. A. Helm, R. J. Leatherbarrow, A. J. Beavil, B. J. Sutton, A. C. Spivey, *J. Org. Chem*. **2012**, *77*, 3197–3214.

51. G. T. Crisp, P. D. Turner, *Tetrahedron* **2000**, *56*, 407–415.

52. S. Liao, A. Porta, X. Cheng, X. Ma, G. Zanoni, L. Zhang, *Angew. Chem. Int. Ed.* **2018**, *130*, 8382–8386.

53. H. Shen, J. Fu, J. Gong, Z. Yang, *Org. Lett*. **2014**, *16*, 5588–5591.

54. C. Körner, P. Starkov, T. D. Sheppard, *J. Am. Chem. Soc*. **2010**, *132*, 5968–5969.

55. C. Morrill, R. H. Grubbs, *J. Org. Chem*. **2003**, *68*, 6031–6034.

56. Z. Tang, C. Li, W. Li, T. Zhang, Z. Li, and T. Chen, *Org. Lett*. **2021**, *23*, 3304–3309.

57. M. X. He, Z. Y. Mo, Z. Q. Wang, S. Y. Cheng, R. R. Xie, H. T. Tang, Y. M. Pan, *Org. Lett*. **2020**, *22*, 724–728.

58. W. Lu, Z. Shen, *Org. Lett*. **2019**, *21*, 142–146.

59. P. Xiong, M. Hemming, S. I. Ivlev, E. Meggers, *J. Am. Chem. Soc.* **2022**, *144*, 6964–6971.

60. P. Dominguez-Molano, G. Bru, O. Salvado, R. J. Maza, Jorge J. Carbó, E. Fernández, *Chem. Commun*. **2021**, *57*, 13361–13364.

61. C. Feng, H. Wang, L. Xu, P. Li, *Org. Biomol. Chem*. **2015**, *13*, 7136–7139.

62. S. Liu, L.S. Liebeskind, *J. Am. Chem. Soc*. **2008**, *130*, 6918–6919.

63. P. A. Wender, A. B. Lesser, L. E. Sirois, *Angew. Chem. Int. Ed*. **2012**, *51*, 2736–2740.

64. Y. Liu, D. Ni, M. K. Brown, *J. Am. Chem. Soc*. **2022**, *144*, 18790–18796.

65. C. Zheng, D. Wang, S. S. Stahl, *J. Am. Chem. Soc*. **2012**, *134*, 16496–16499.

66. A.T. Parsons, T.D. Senecal, S.L. Buchwald, *Angew. Chem. Int. Ed*. **2012**, *51*, 2947–2950.

67. K. Norseeda, N. Chaisan, C. Thongsornkleeb, J. Tummatorn, S. Ruchirawat, *J. Org. Chem*. **2019**, *84*, 16222–16236.

68. T. Brégent, J. P. Bouillon, T. Poisson, *Chem. Eur. J*. **2021**, *27*, 13966–13970.

69. J. J. Molloy, J. B. Metternich, C. G. Daniliuc, A. J. B. Watson, R. Gilmour, *Angew. Chem. Int. Ed.* **2018**, *130*, 3222–3226.

70. Y. Zhao, C. F. Liu, L. Q. H. Lin, A. S. C. Chan, M. J. Koh, *Angew. Chem. Int. Ed*. **2022**, *61*, e2022026.

71. D. P. Ojha, K. R. Prabhu, *Org. Lett*. **2015**, *17*, 18–21.

72. M. Aelterman, M. Sayes, P. Jubault, T. Poisson, *Chem. Eur. J*. **2021**, *27,* 8277–8282.

73. A. Music, C. M. Nuber, Y. Lemke, P. Spieß, D. Didier, *Org. Lett*. **2021**, *23*, 4179–4184.

74. T. Ueda, H. Konishi, K. Manabe, *Org. Lett*. **2012**, *14*, 5370–5373.

75. Y. Hu, W. Sun, T. Zhang, N. Xu, J. Xu, Y. Lan, C. Liu, *Angew. Chem. Int. Ed.* **2019**, *131*, 15960–15965.

76. C. W. Grathwol, N. Wössner, S. Swyter, A. C. Smith, E. Tapavicza, R. K. Hofstetter, A. Bodtke, M. Jung, A. Link, *Beilstein J. Org. Chem*. **2019**, *15*, 2170–2183.

77. Z. Liu, W. Wei, L. Xiong, Q. Feng, Y. Shi, N. Wang, L. Yu, *New J. Chem*. **2017**, *41*, 3172–3176.

78. C. Richardson, C. A. Reed, J. *Org. Chem.* **2007**, 72, 4750–4755.

79. C. A. Malapit, J. R. Bour, S. R. Laursen, M. S. Sanford, *J. Am. Chem. Soc*. **2019**, *141*, 17322–17330.

80. J. Polášek, J. Paciorek, J. Stošek, H. Semrád, M. Munzarová, C. Mazal, *J. Org. Chem.* **2020**, *85*, 6992–7000.

81. S. Eising, F. Lelivelt, K.M. Bonger, *Angew. Chem. Int. Ed*. **2016**, *128*, 12431–12435.

82. A. Music, A.N. Baumann, P. Spieß, A. Plantefol, T. C. Jagau, D. Didier, *J. Am. Chem. Soc*. **2020**, *142*, 4341–4348.

83. C. Cazorla, E. Métay, M. Lemaire, *Tetrahedron* **2011**, *67*, 8615–8621.

84. H. Huang, C. Yu, X. Li, Y. Zhang, Y. Zhang, X. Chen, Prof. P. S. Mariano, H. Xie, W. Wang, *Angew. Chem. Int. Ed*. **2017**, *129*, 8313–8317.

85. D. Haddenham, C. L. Bailey, C. Vu, G. Nepomuceno, S. Eagon, L. Pasumansky, B. Singaram, a *Tetrahedron* **2011**, *67,* 576–583.

86. Y. B. Zhou, Y. Q. Wang, L. C. Ning, Z. C. Ding, W. L. Wang, C. K. Ding, R. H. Li, J. J. Chen, X. Lu, Y. J. Ding, Z. P. Zhan, *J. Am. Chem. Soc*. **2017**, *139*, 3966–3969.

87. H. Cao, H. Jiang, H. Feng, J. Mun Chung Kwan, X. Liu, J. Wu, *J. Am. Chem. Soc*. **2018**, *140*, 16360–16367.

88. G. A. Molander, O. A. Argintaru, *Org. Lett.* **2014**, *16*, 1904–1907.

89. S. F. Pizzolato, M. Giannerini, P. H. Bos, M. Fañanás-Mastral, B. L. Feringa, *Chem.Commun*. **2015**, *51*, 8142–8145.

90. N. Kambe, Y. Moriwaki, Y. Fujii, T. Iwasaki, J. Terao, *Org. Lett*. **2011**, *13*, 4656–4659.

91. Y. L. Zhang, L. Yang, J. Wu, C. Zhu, P. Wang, *Org. Lett*. **2020**, *22*, 7768–7772.

92. R. Matsubara, T. F. Jamison, *Chem. Asian J*. **2011**, *6*, 1860–1875.

93. B. C. Van Veen, S. M. Wales, J. Clayden, *J. Org. Chem*. **2021**, *86*, 8538–8543.

94. K. Das, R. Shibuya, Y. Nakahara, N. Germain, T. Ohshima, K. Mashima, *Angew. Chem. Int. Ed*. **2012**, *51*, 150–154.

95. M. Koy, F. Sandfort, A. Tlahuext-Aca, L. Quach, C. G. Daniliuc, F. Glorius, *Chem. Eur. J.* **2018**, *24*, 4552–4555.

96. A. Noble, D. W. C. MacMillan, J. *Am. Chem. Soc*. **2014**, *136*, 11602–11605.

97. D. N. Prada Gori, C. Permingeat Squizatto, P. G. Cornier, C. M.L. Delpiccolo, *J. Org. Chem.* **2018**, *83*, 12798–12805.

98. J. Zhang, Y. Li, R. Xu, Y. Chen, *Angew. Chem. Int. Ed.* **2017**, *129*, 12793–12797.

99. A. Guérinot, A. Serra-Muns, C. Gnamm, C. Bensoussan, S. Reymond, J. Cossy, *Org Lett.* **2010**, *12*, 1808–1811.

100. Z. H. Xia, C. L. Zhang, Z. H. Gao, S. Ye, *Org. Lett.* **2018**, *20*, 3496–3499.

101. J. Hu, B. Cheng, X. Yang, T. P. Loh, *Adv. Synth. Catal.* **2019**, *361*, 4902–4908.

102. D. Zhang, Z. L. Tang, X. H. Ouyang, R. J. Song, J. H. Li, *Chem. Commun*. **2020**, *56*, 14055–14058.

103. S. Yu, S. Liu, Y. Lan, B. Wan, X. Li, *J. Am. Chem. Soc*. **2015**, *137*, 1623–1631.

104. H. Y. Wang, L. J. Zhong, G. F. Lv, Y. Li, J. H. Li, *Org. Biomol. Chem.* **2020**, *18*, 5589–5593.

105. D. Mao, G. Hong, S. Wu, X. Liu, J. Yu, L. Wang, *Eur. J. Org. Chem.* **2014**, 3009–3019.

106. M. Zhang, L. Yang, C. Tian, M. Zhou, G. An, G. Li, *Org. Biomol. Chem.* **2019**, *17*, 2258–2264.

107. E. M. Woerly, S. M. Banik, E. N. Jacobsen, *J. Am. Chem. Soc*. **2016**, *138*, 13858–13861.

108. L. L. Anka-Lufford, M. R. Prinsell, D. J. Weix, *J. Org. Chem.* **2012**, *77*, 9989–10000.

109. A. Krasovskiy, C. Duplais, B. H. Lipshutz, *Org. Lett*. **2010**, *12*, 4742–4744.

110. Z. Wang, P. Li, H. Fu, Q. Dai, C. Hu, *Adv. Synth. Catal*. **2019**, *361*, 192–200.

111. S. Minakata, H. Miwa, K. Yamamoto, A. Hirayama, S. Okumura, *J. Am. Chem. Soc*. **2021**, *143*, 4112–4118.

8. NMR Spectra

1,3-Dioxoisoindolin-2-yl 3-(4-fluorophenyl)propanoate, **2**

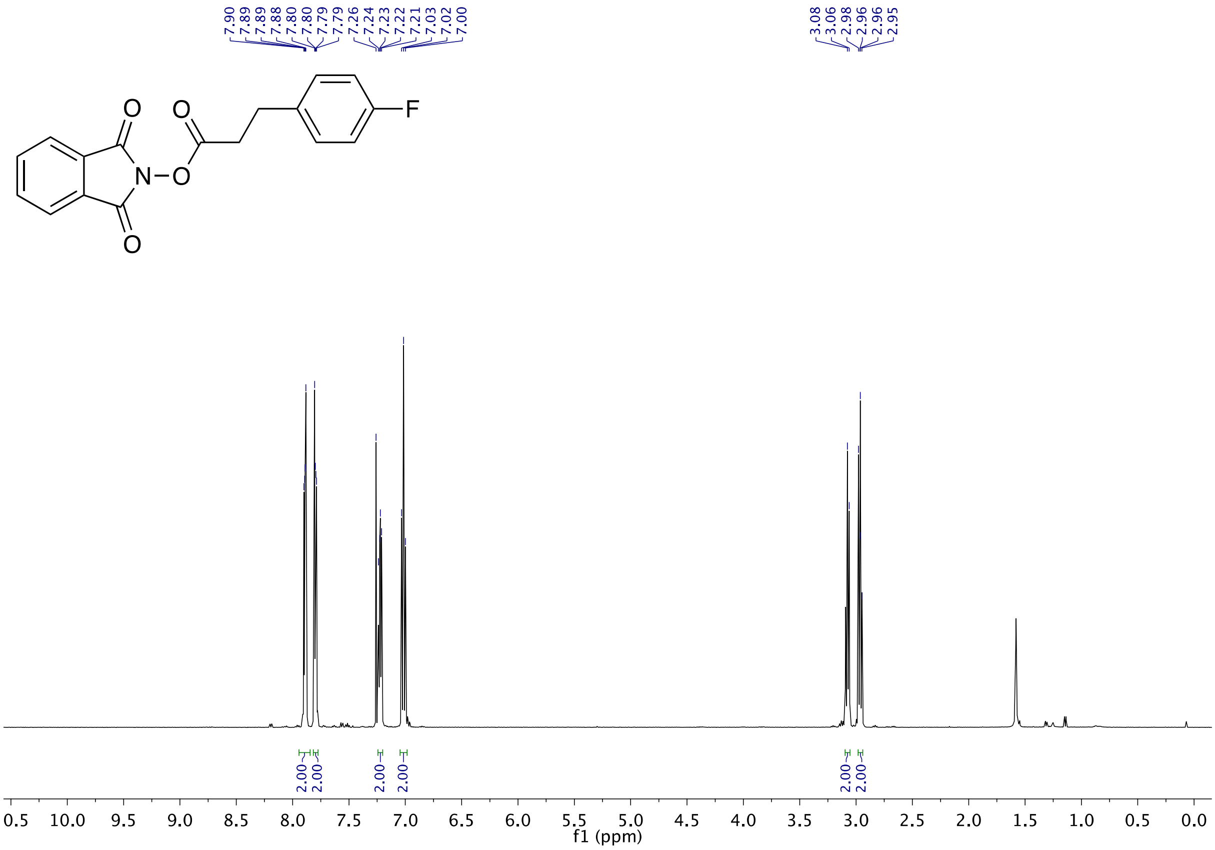

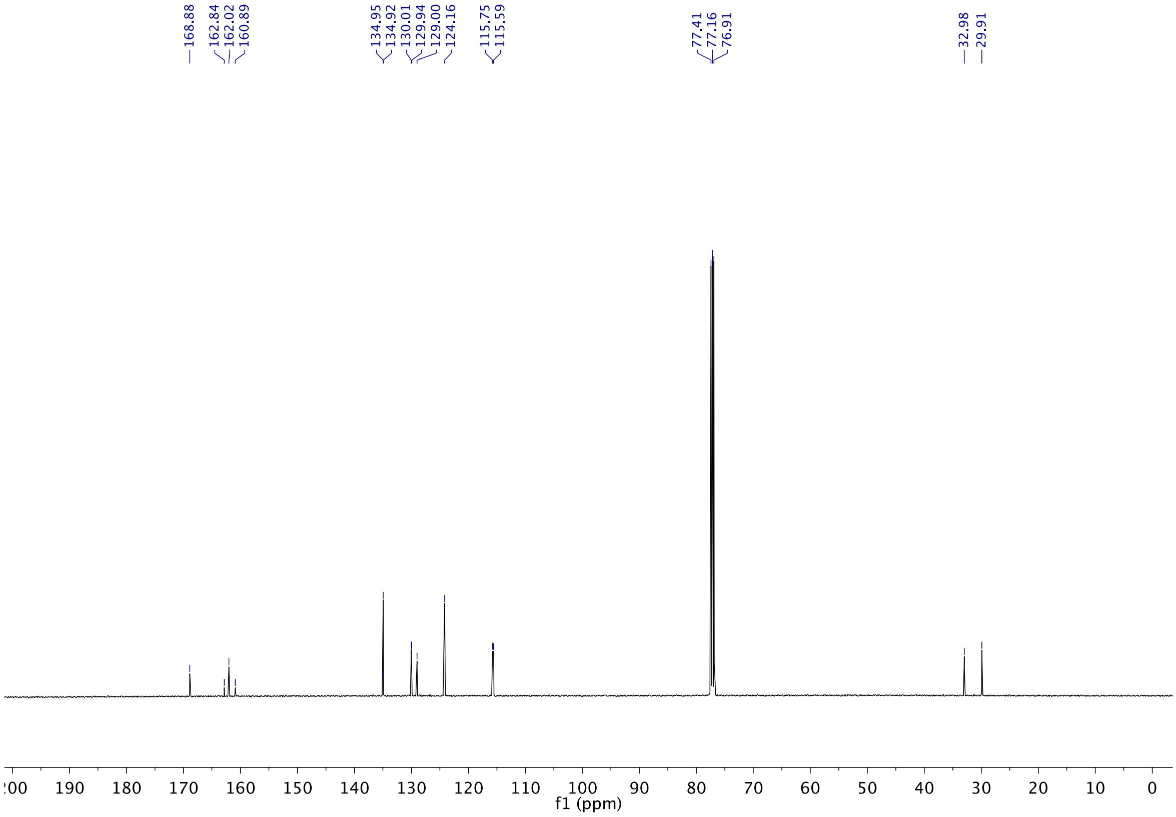

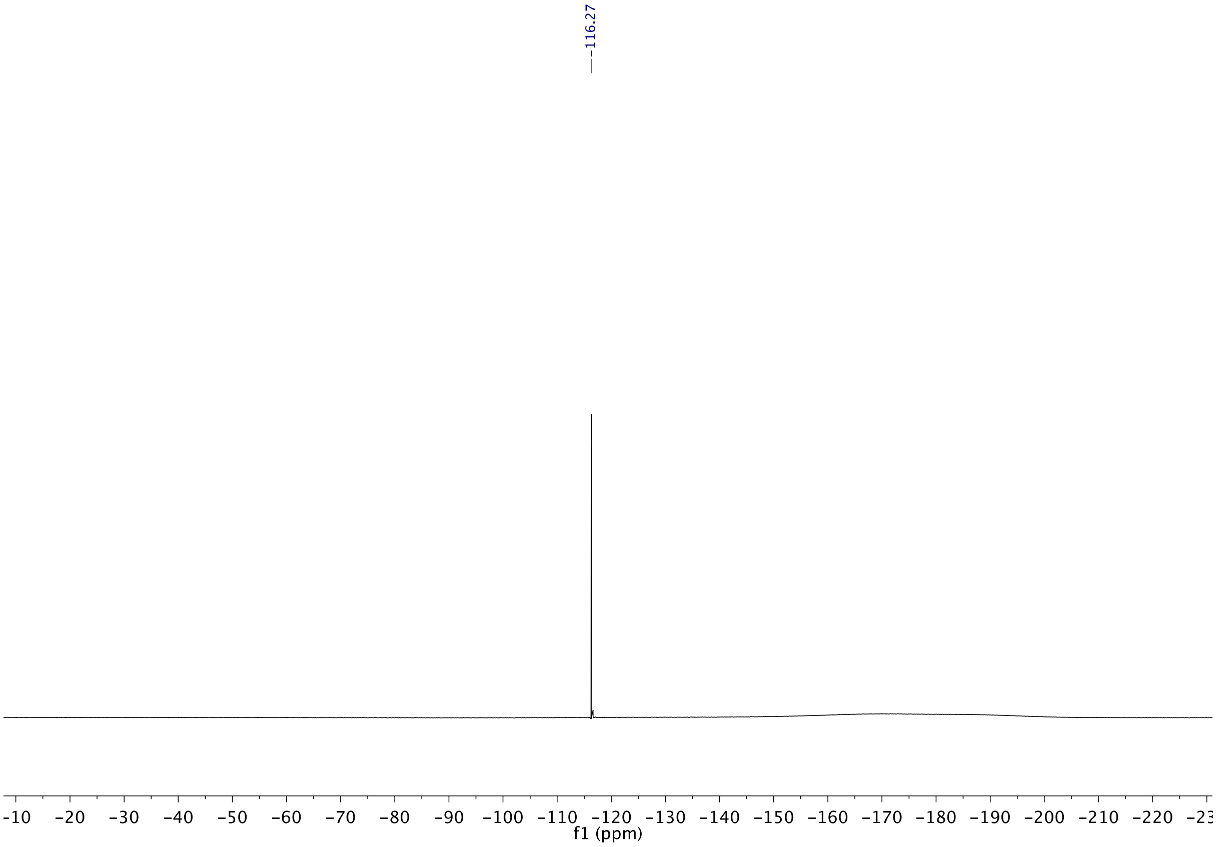

1,3-Dioxoisoindolin-2-yl cyclohexanecarboxylate, **4**

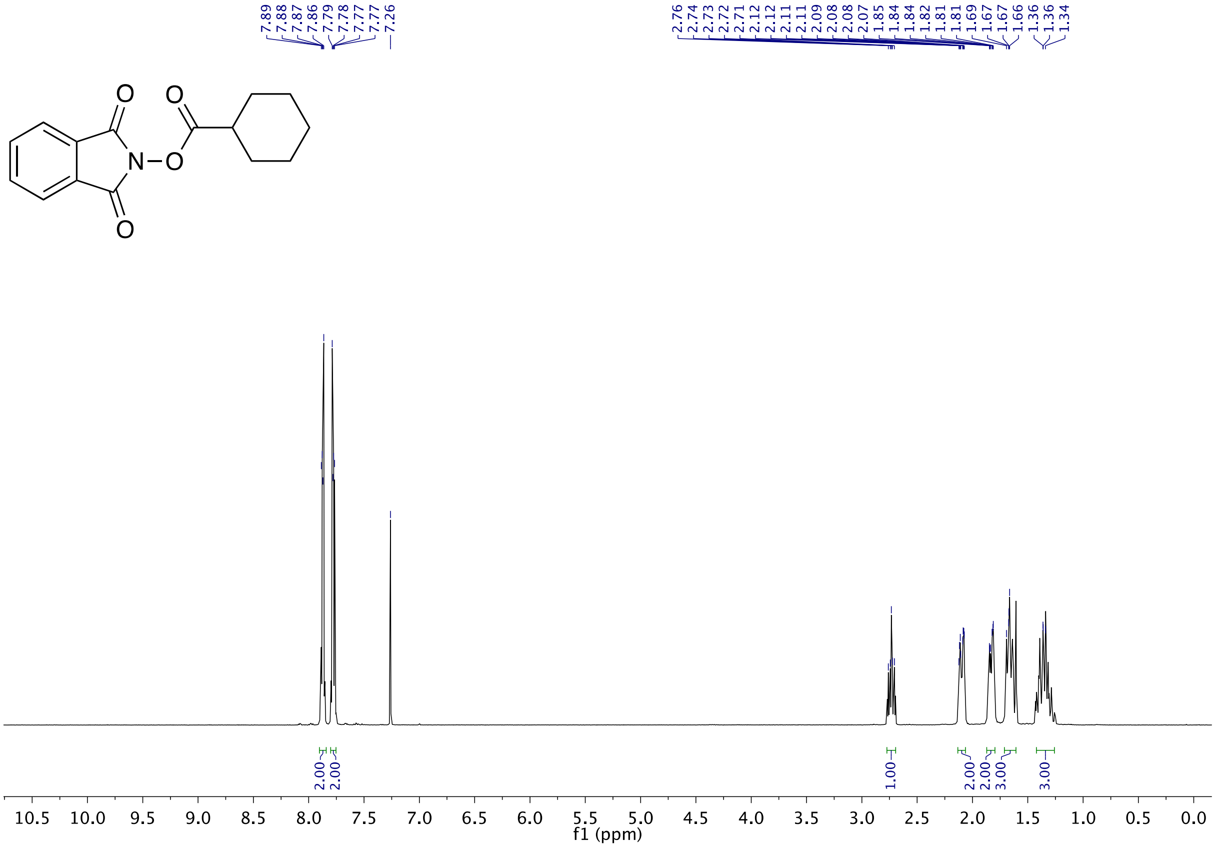

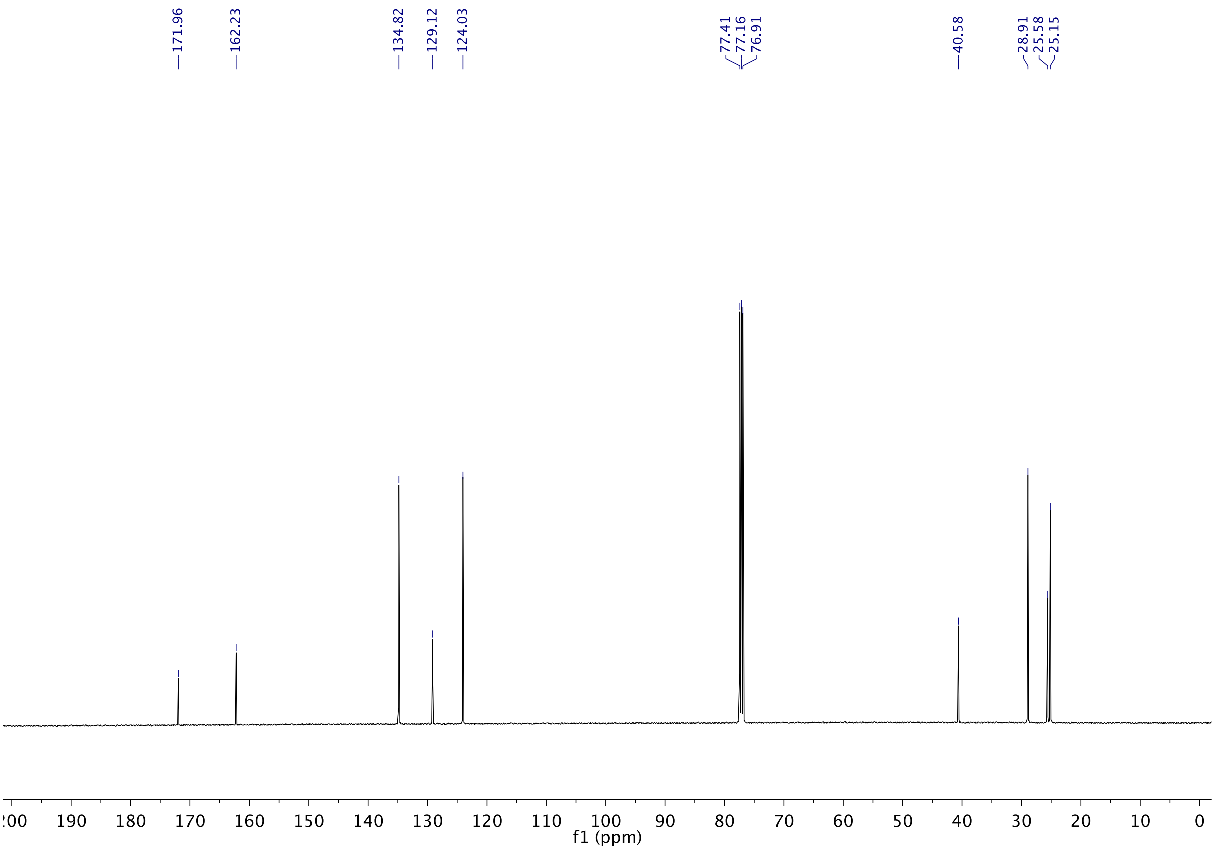

1,3-Dioxoisoindolin-2-yl cyclobutanecarboxylate, **N1**

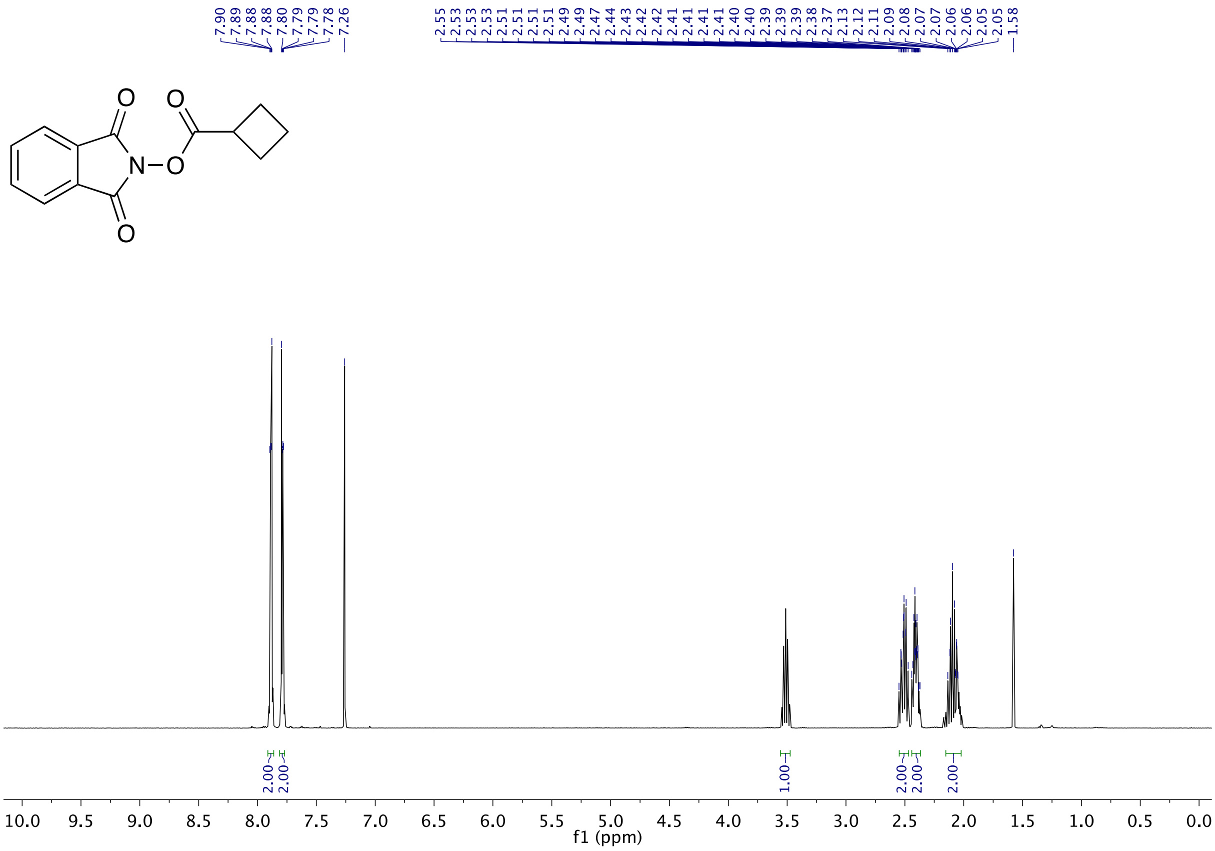

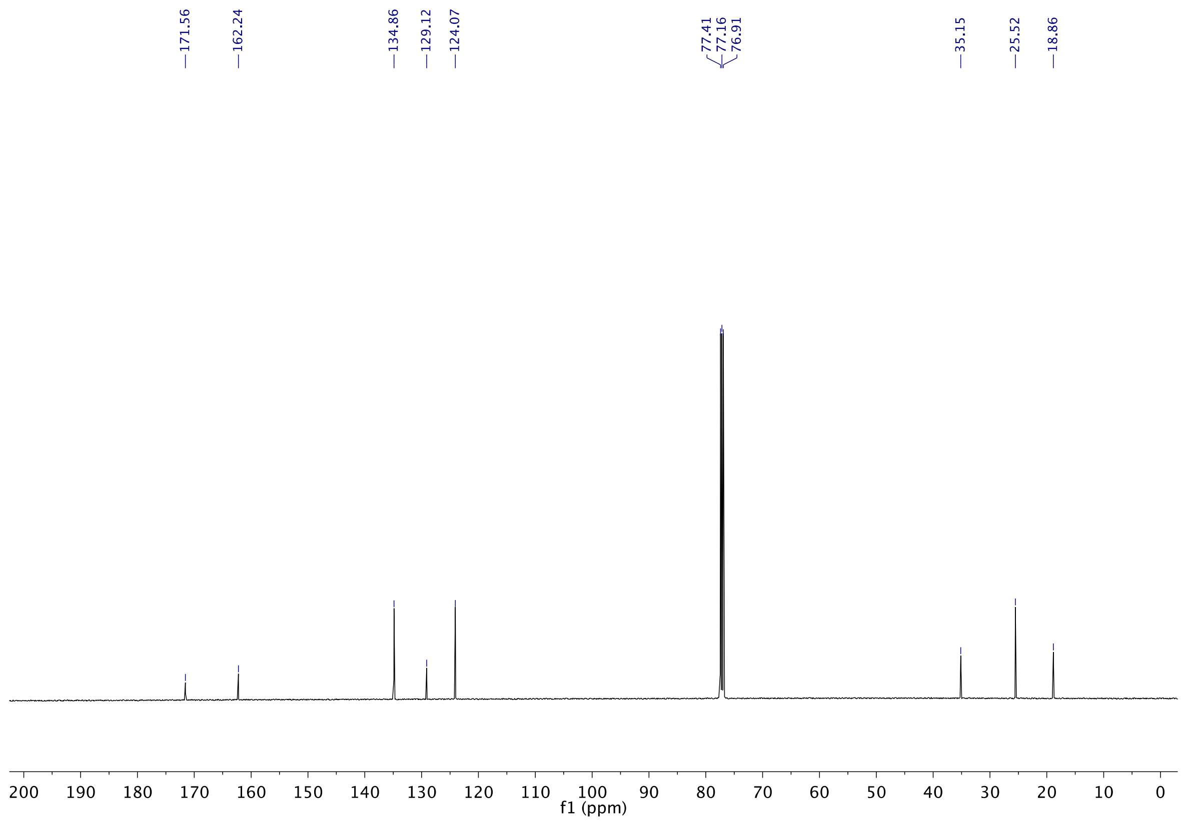

1,3-Dioxoisoindolin-2-yl cyclopentanecarboxylate, **N2**

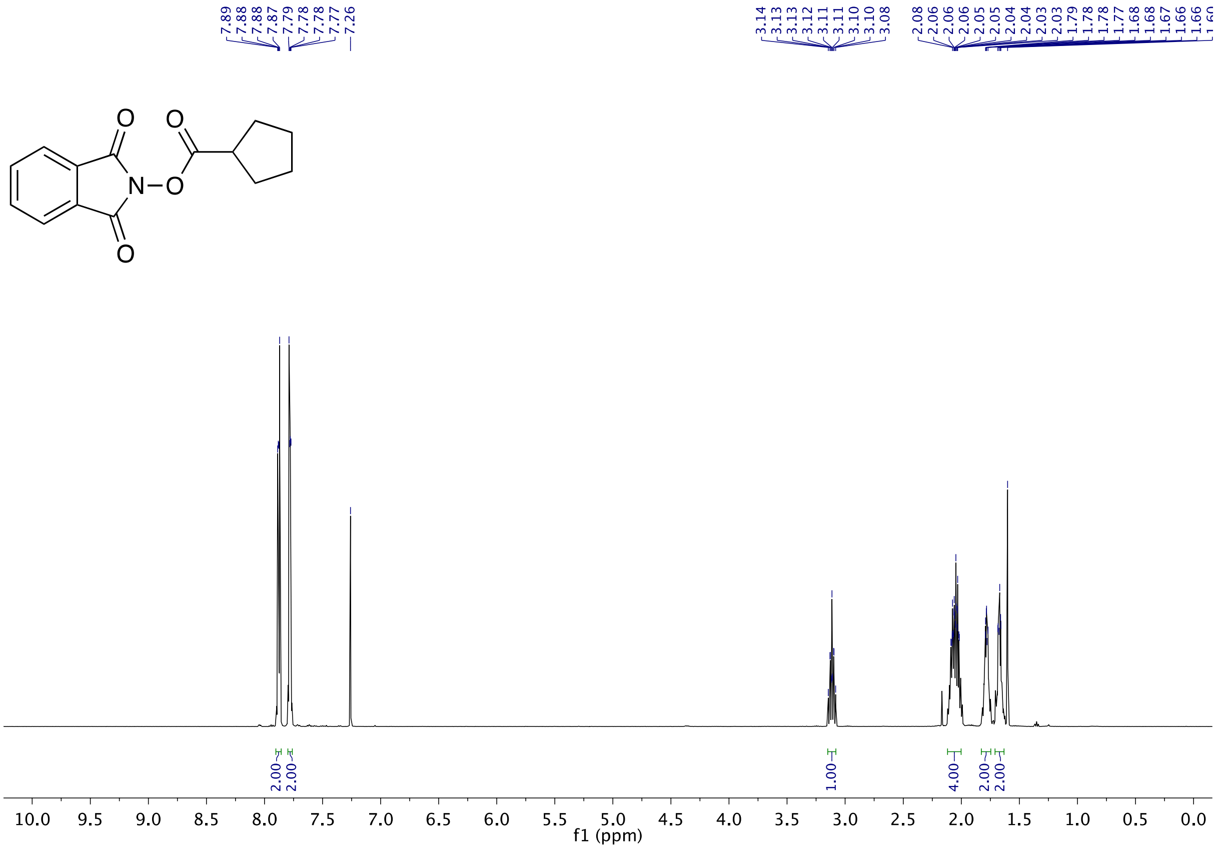

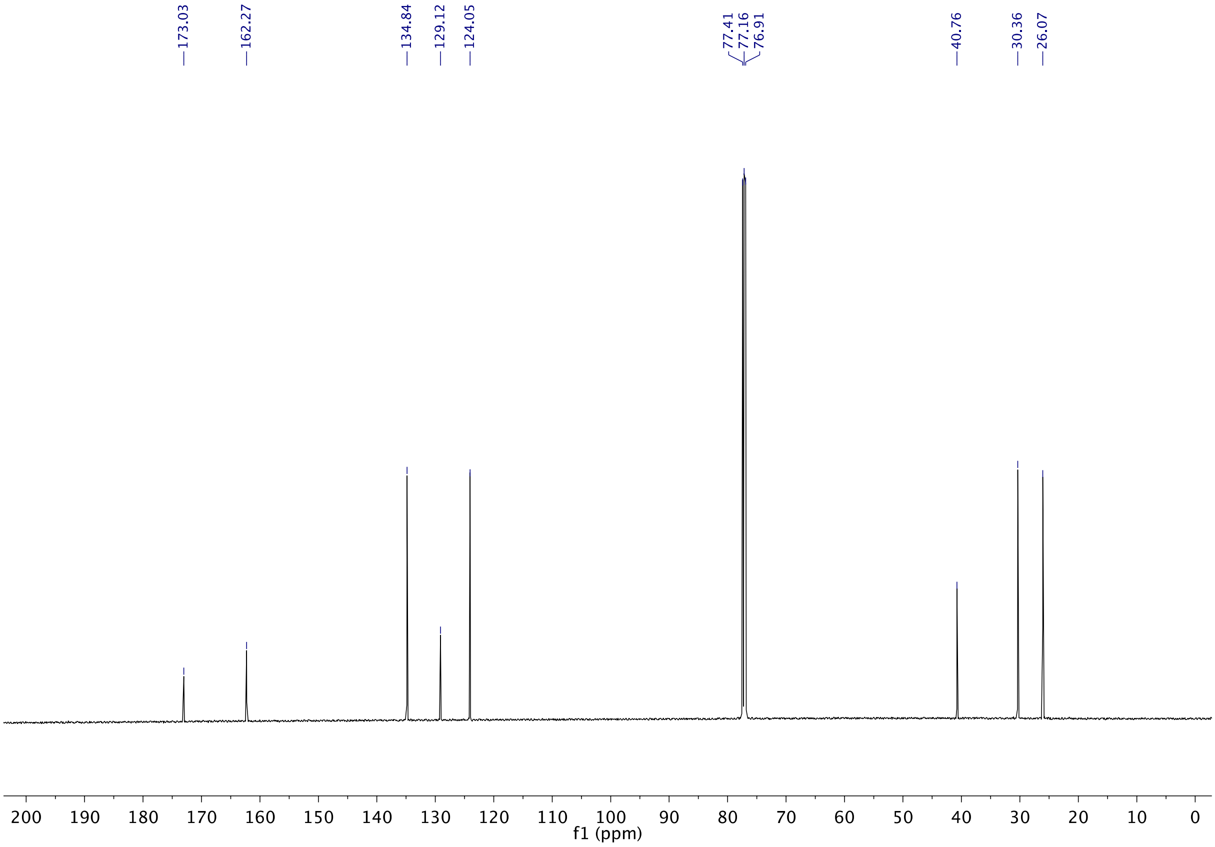

1,3-Dioxoisoindolin-2-yl cycloheptanecarboxylate, **N3**

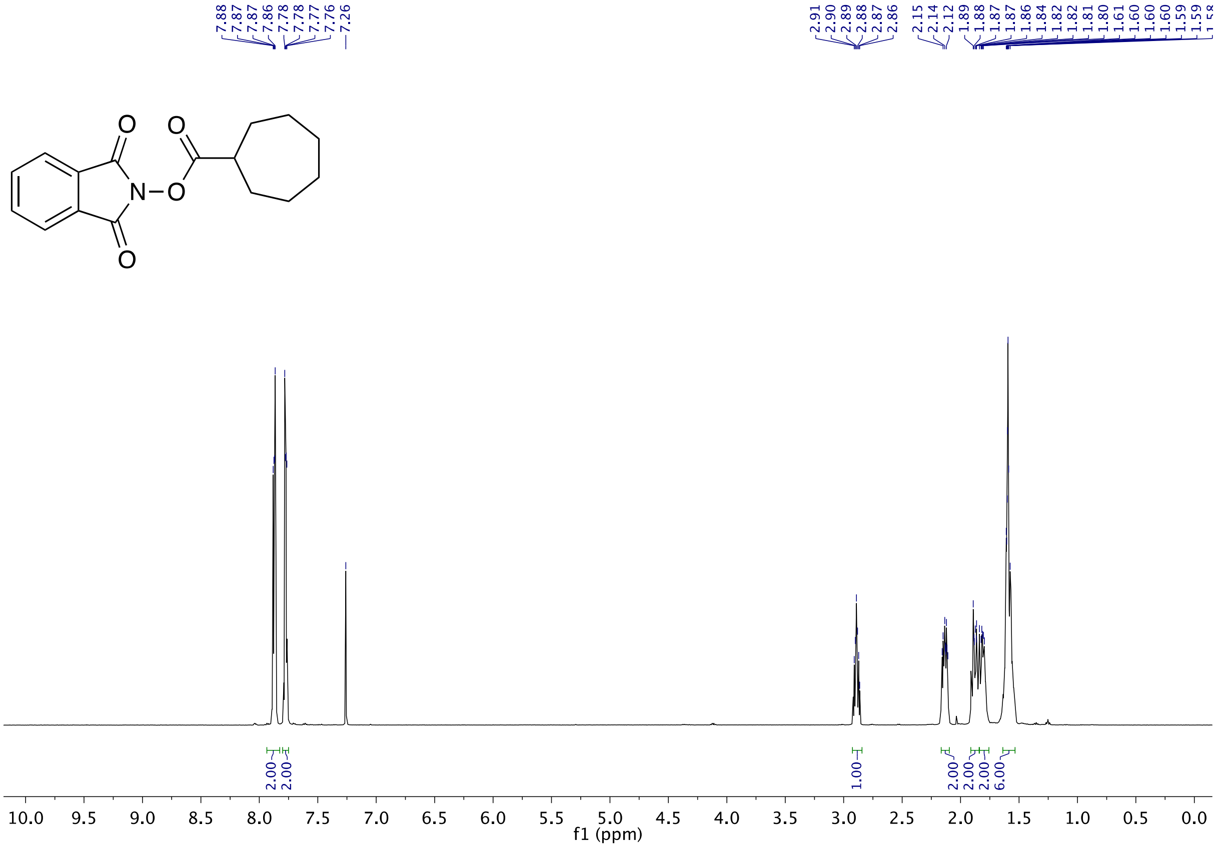

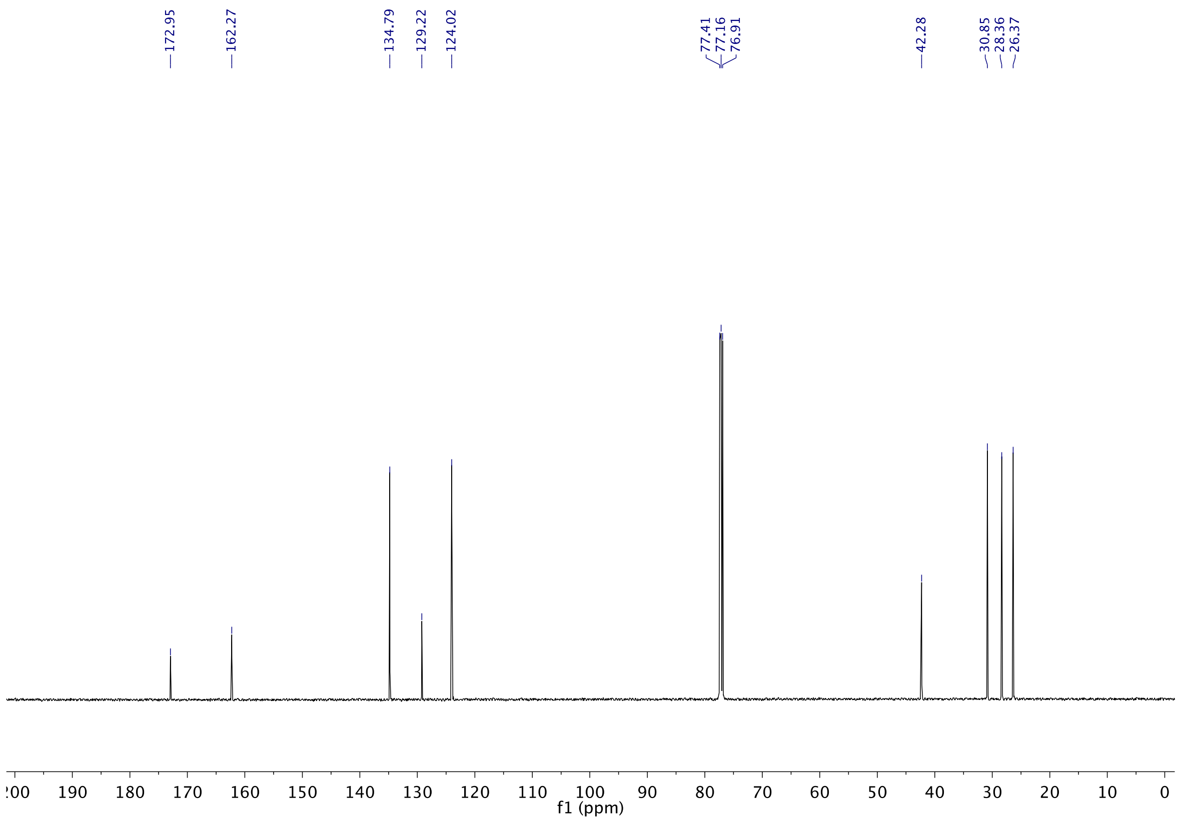

1,3-Dioxoisoindolin-2-yl pivalate, **N4**

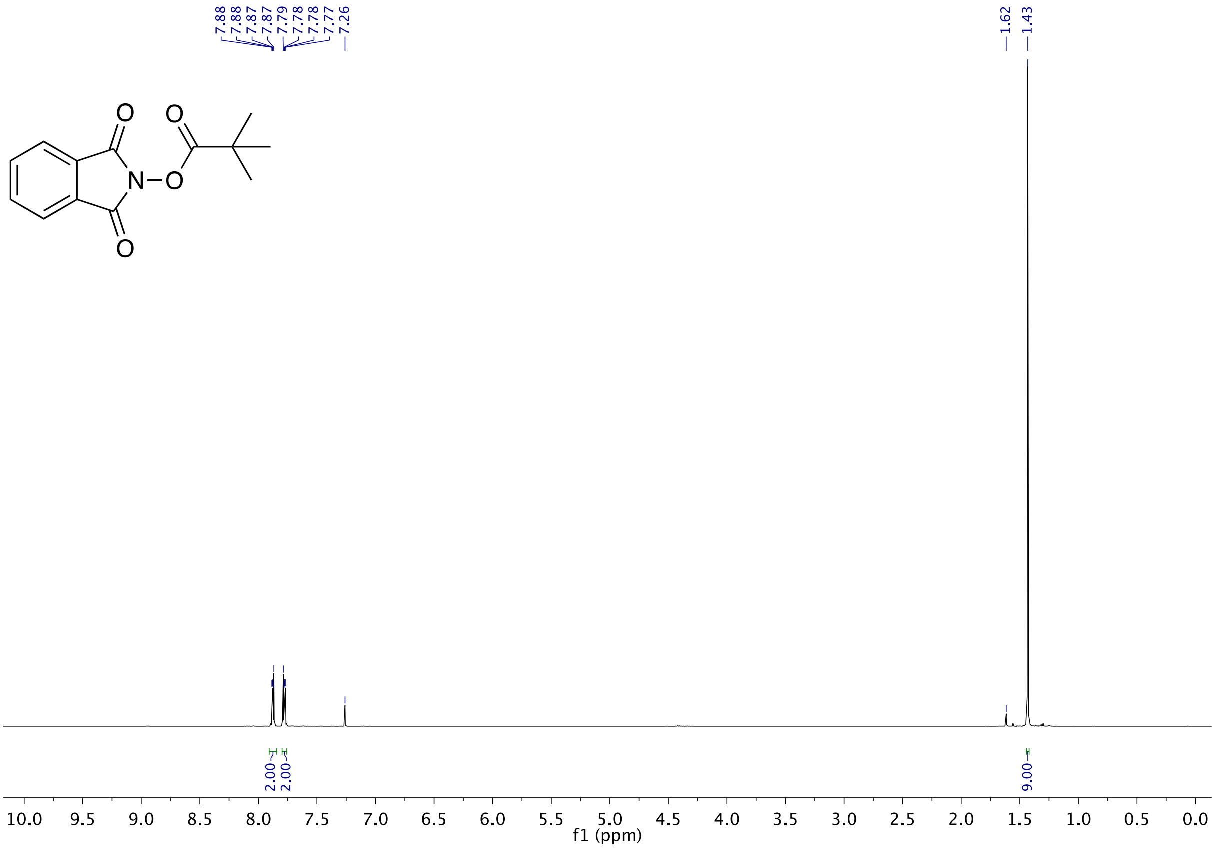

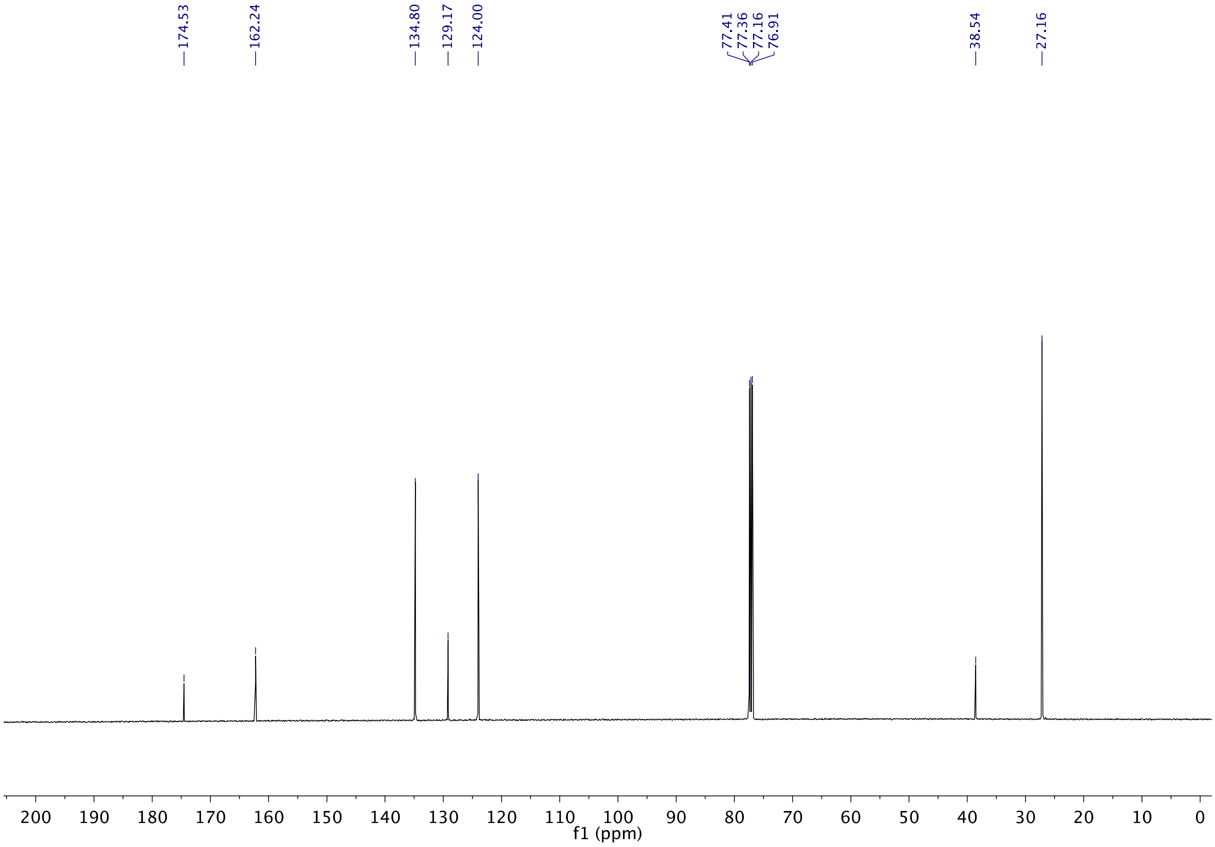

1,3-Dioxoisoindolin-2-yl butyrate, **N5**

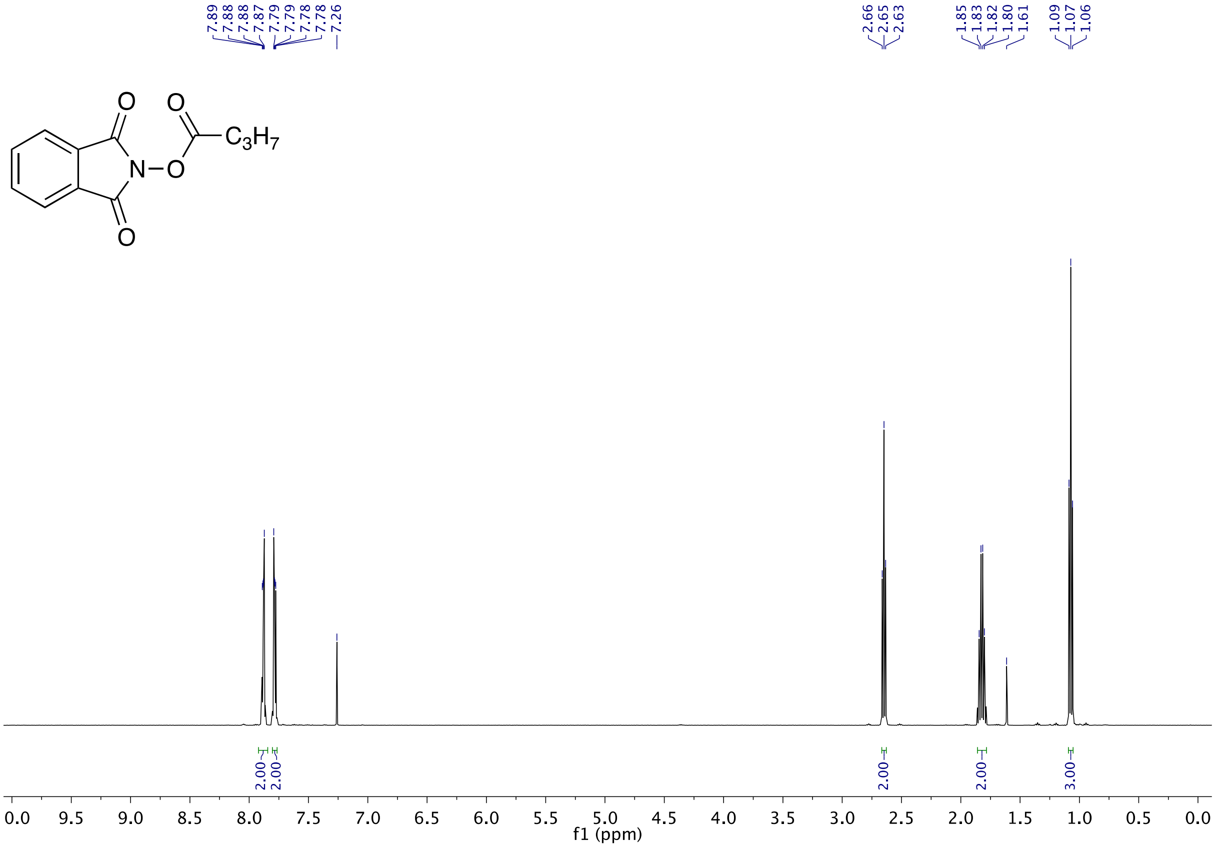

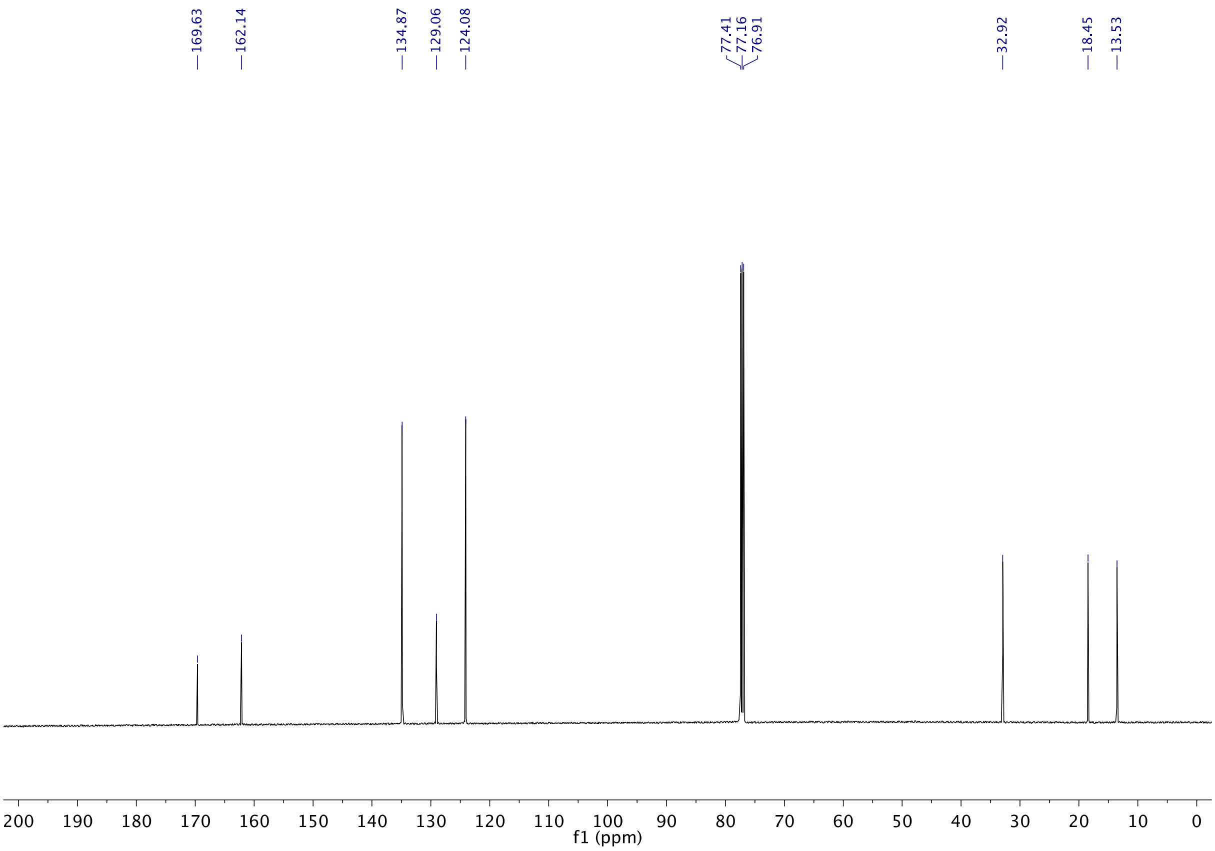

1,3-Dioxoisoindolin-2-yl 2-methylbutanoate, **N6**

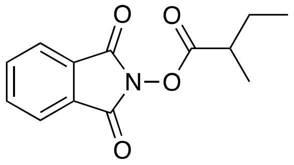

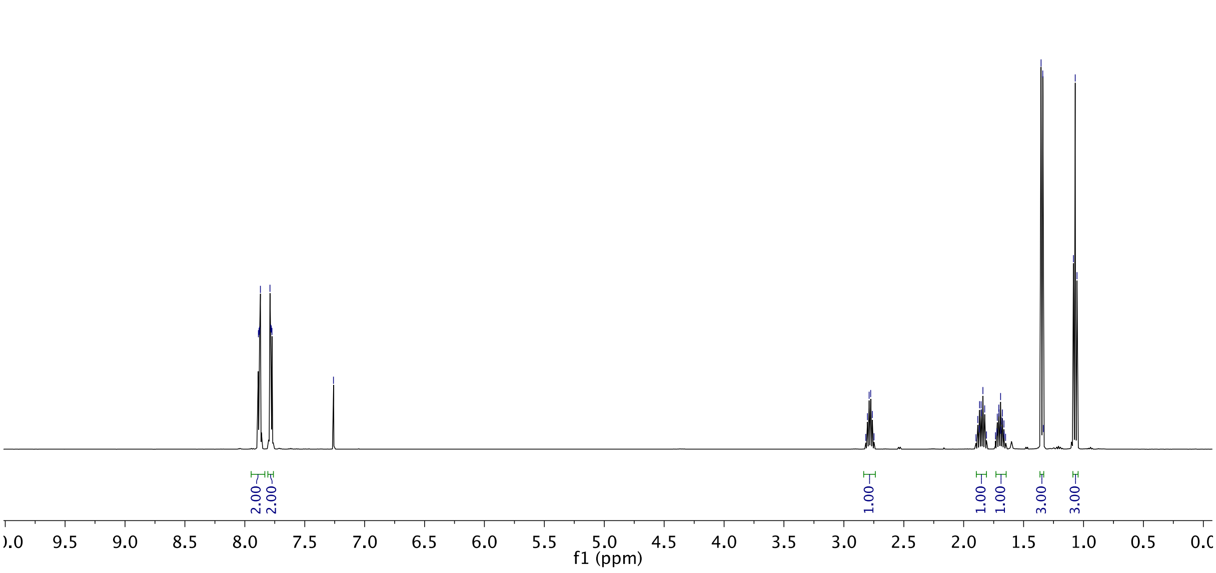

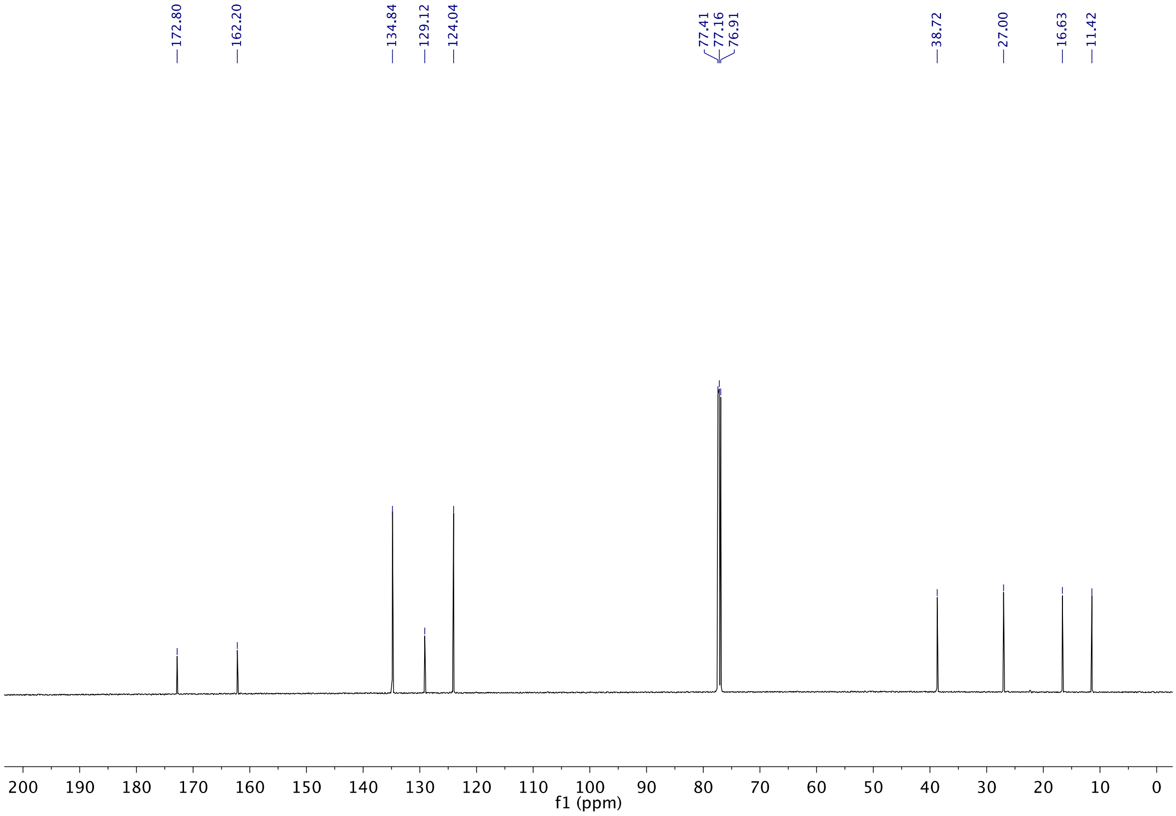

1,3-Dioxoisoindolin-2-yl octanoate, **N7**

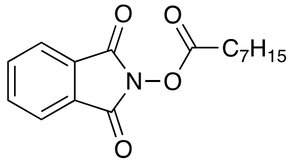

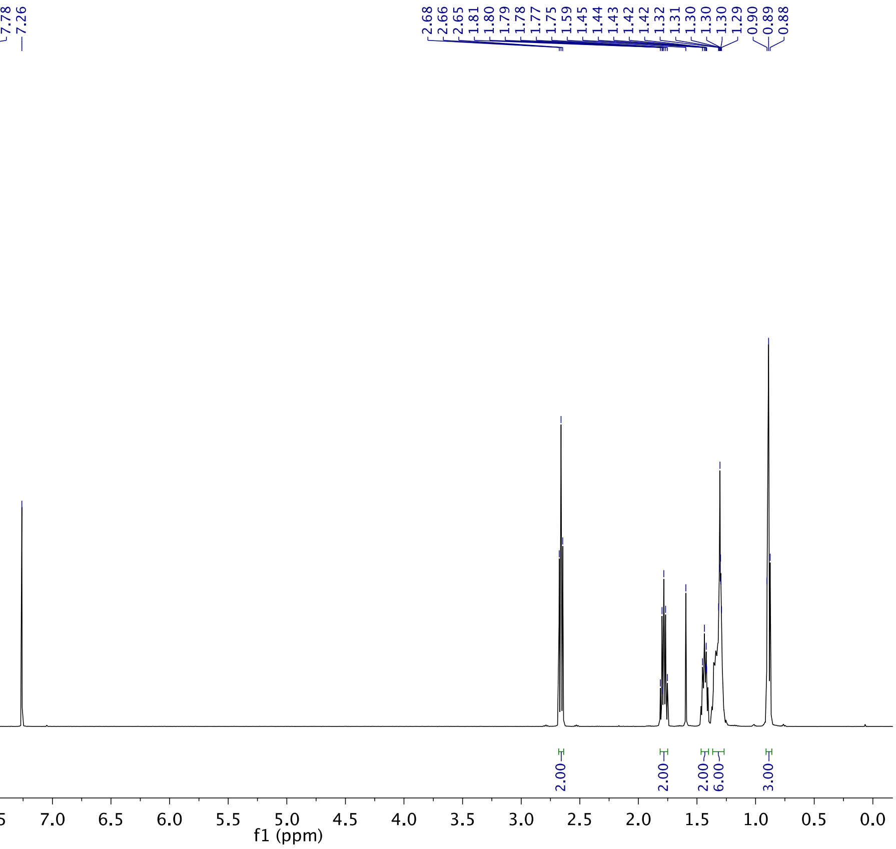

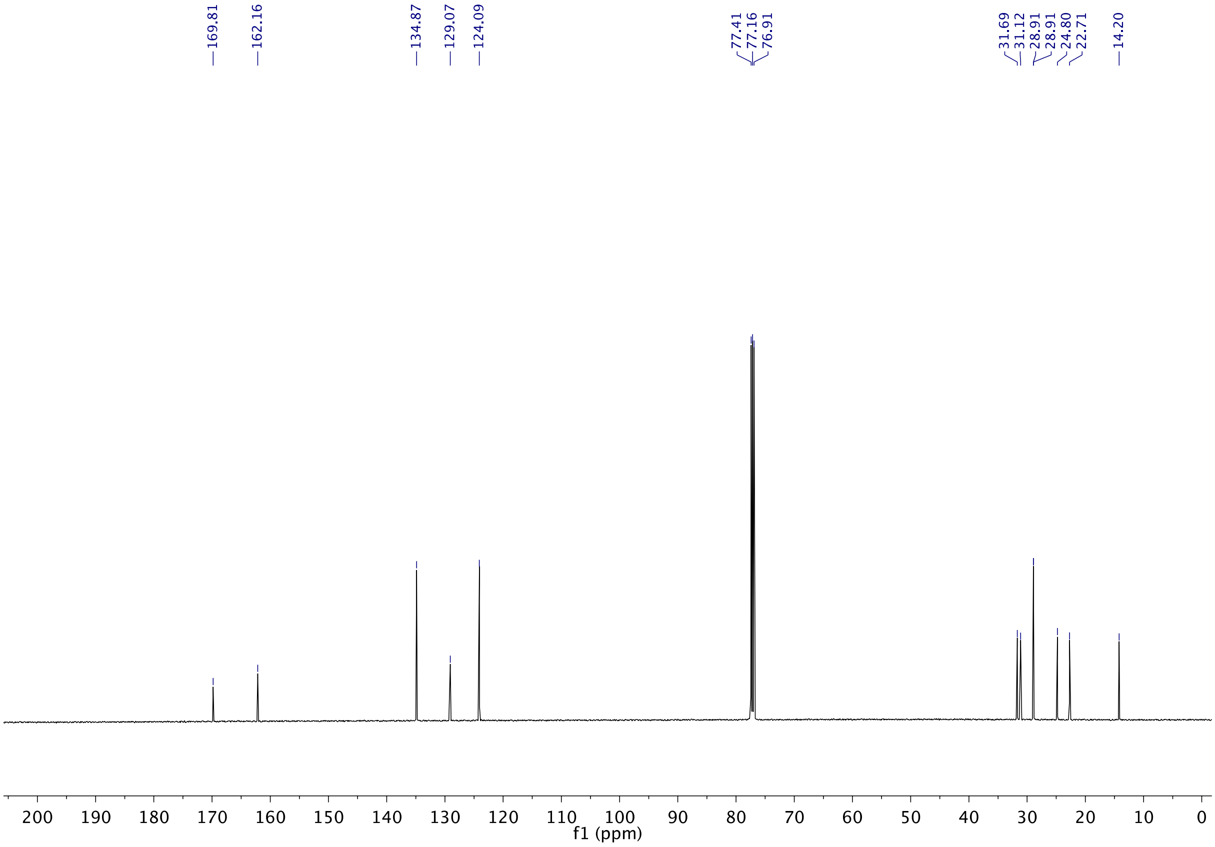

1,3-Dioxoisoindolin-2-yl tetradecanoate, **N8**

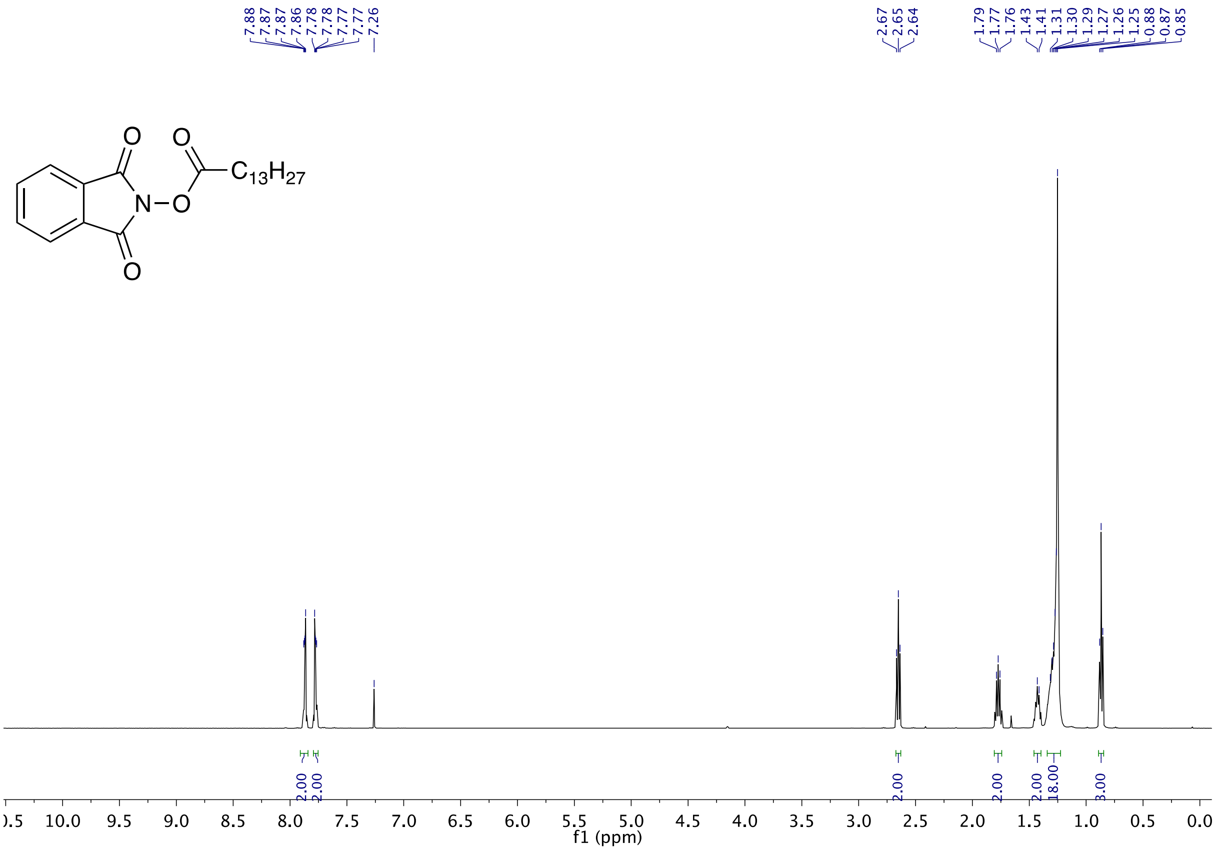

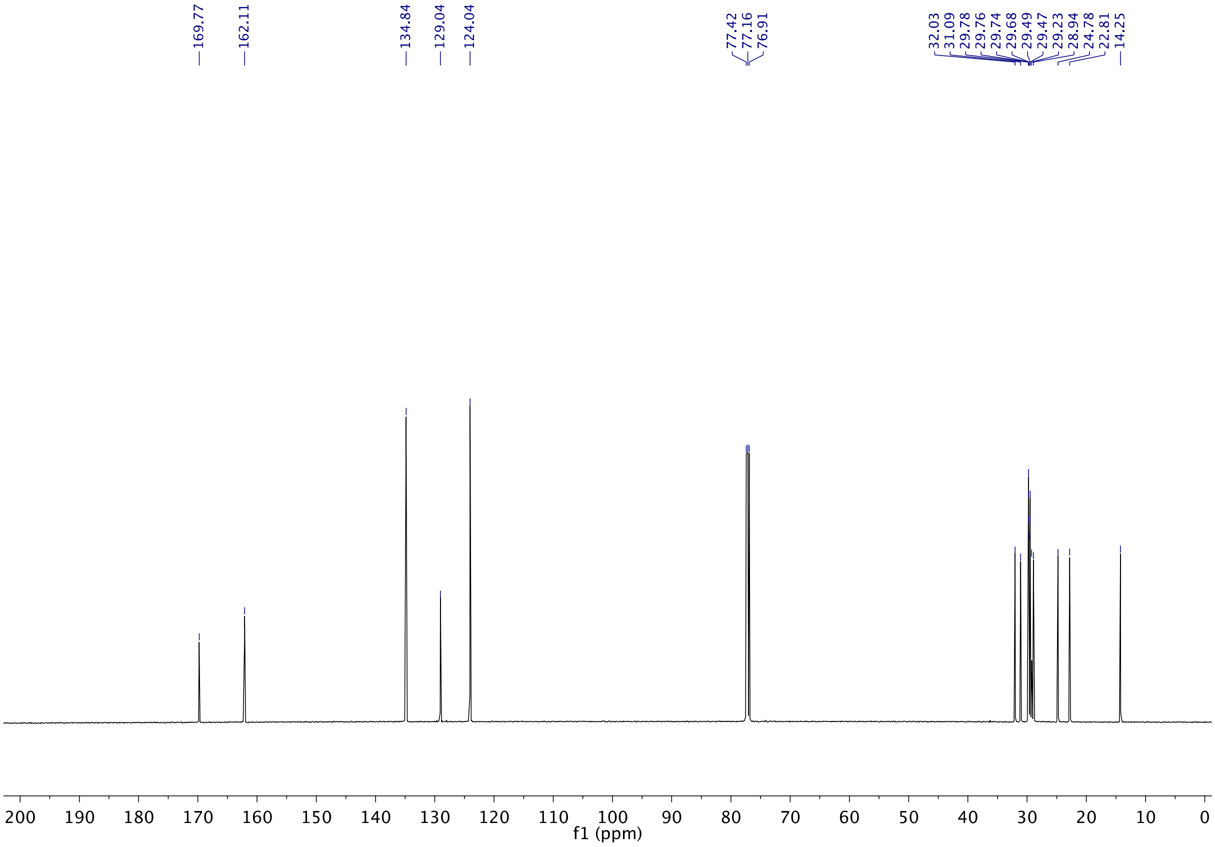

1,3-Dioxoisoindolin-2-yl 3-phenylpropanoate, **N9**

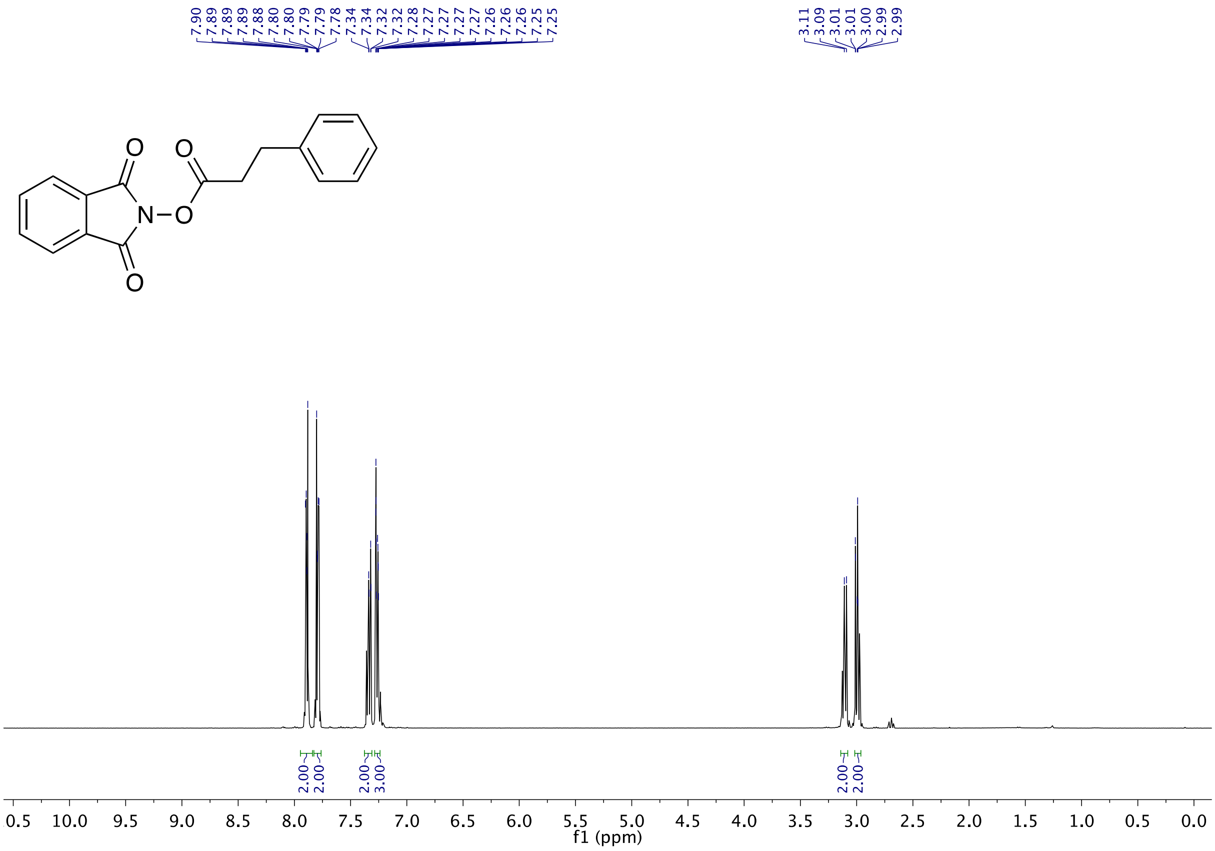

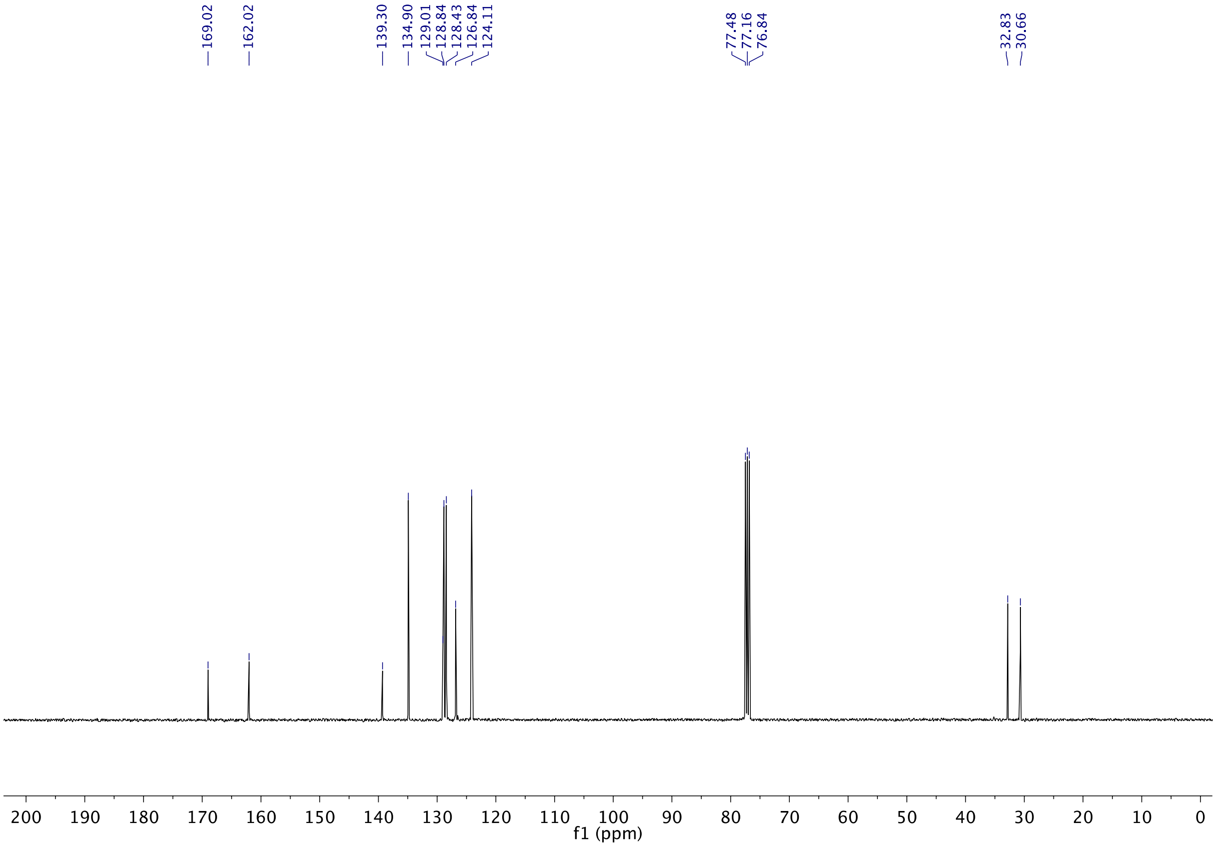

1,3-Dioxoisoindolin-2-yl 3-(4-bromophenyl)propanoate, **N10**

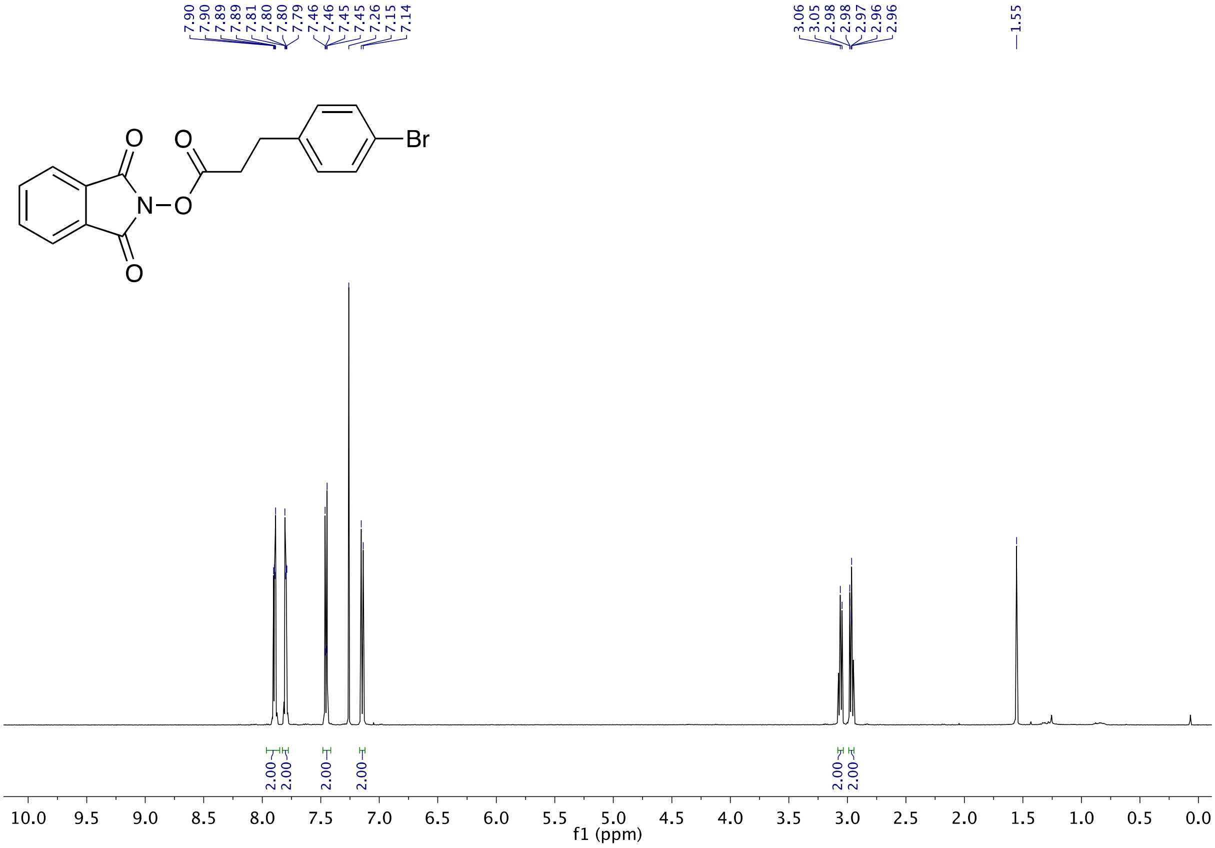

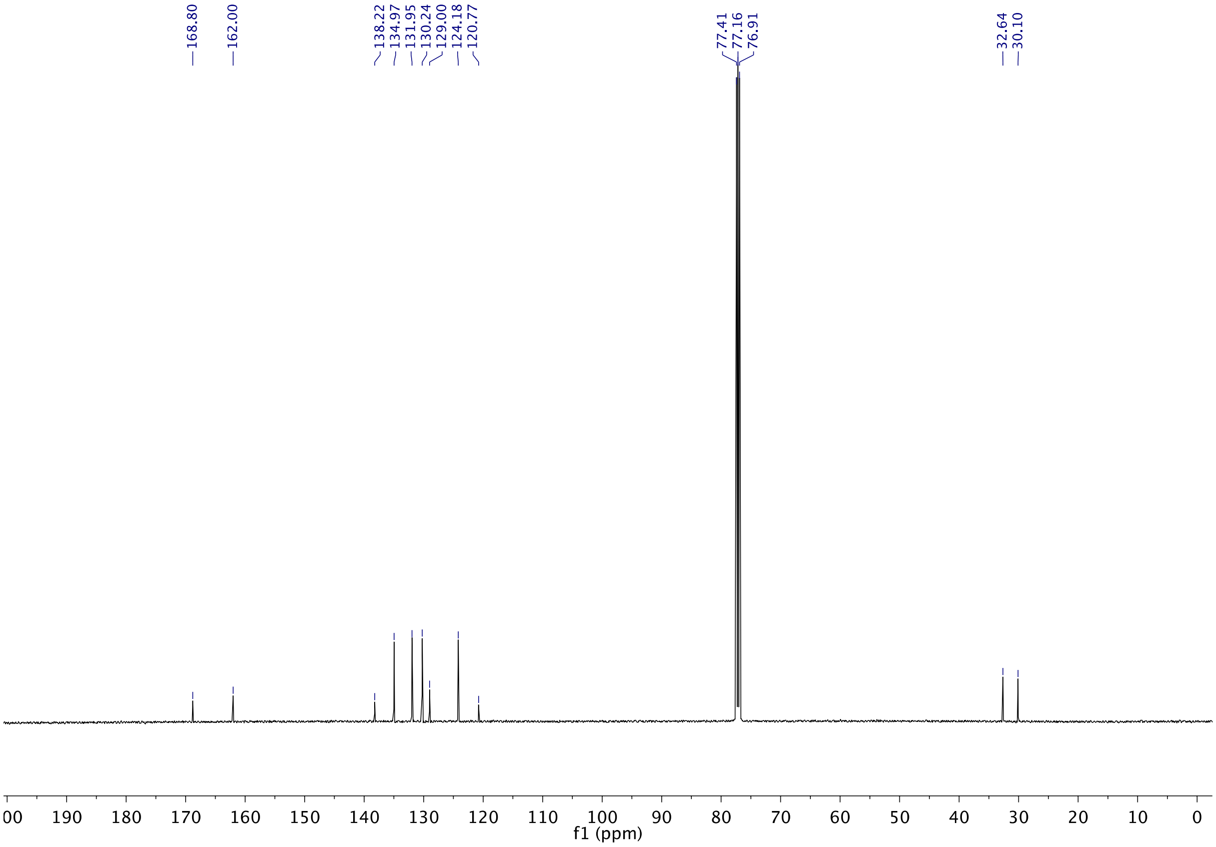

3-(4-Ethynylphenyl)propanoic acid, **N11-int1**

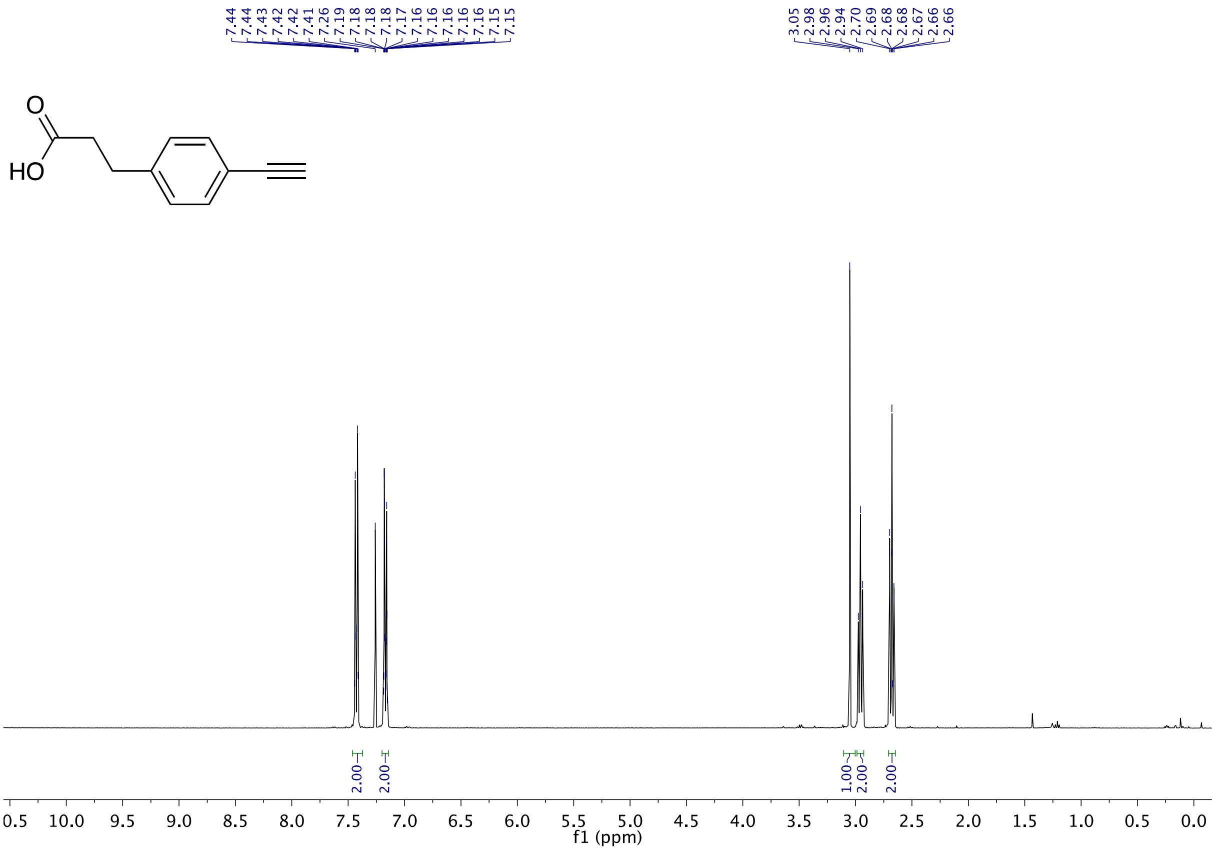

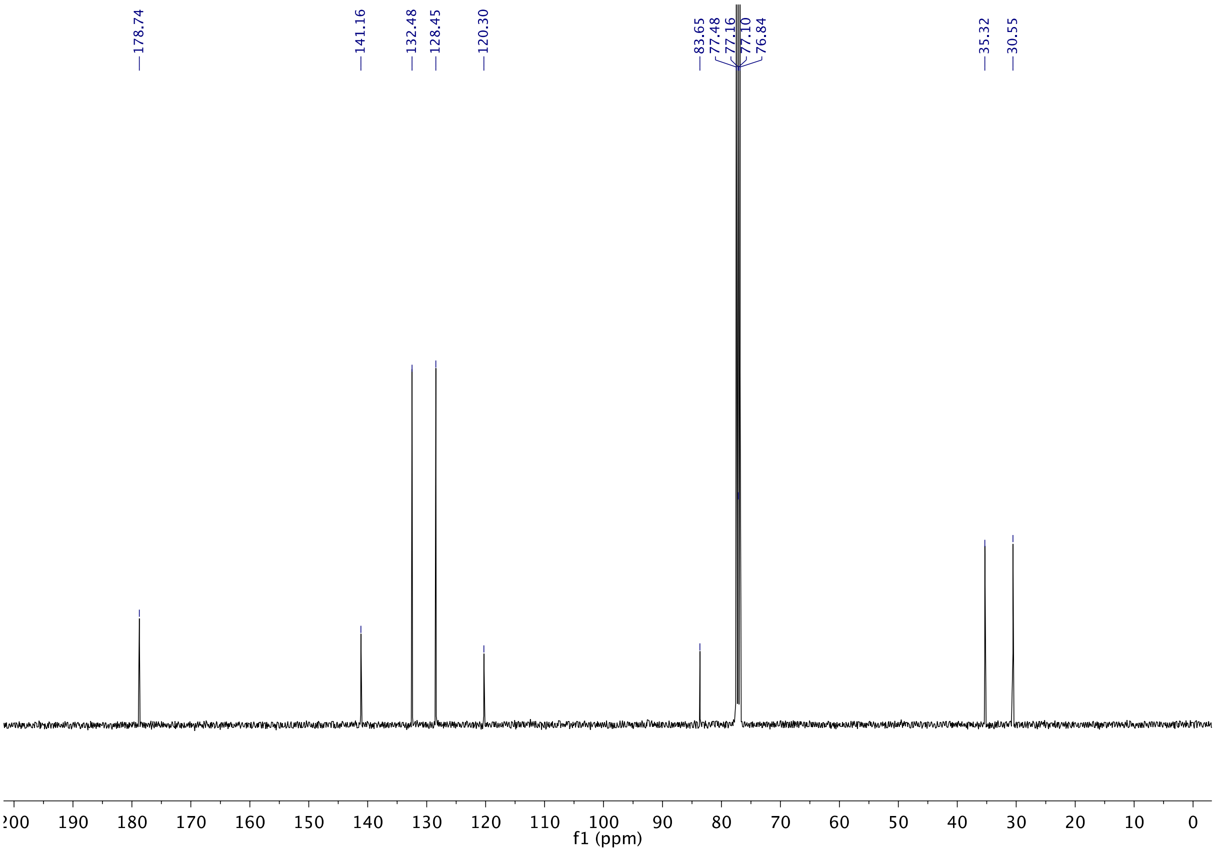

1,3-Dioxoisoindolin-2-yl 3-(4-ethynylphenyl)propanoate, **N11**

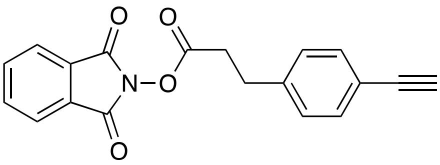

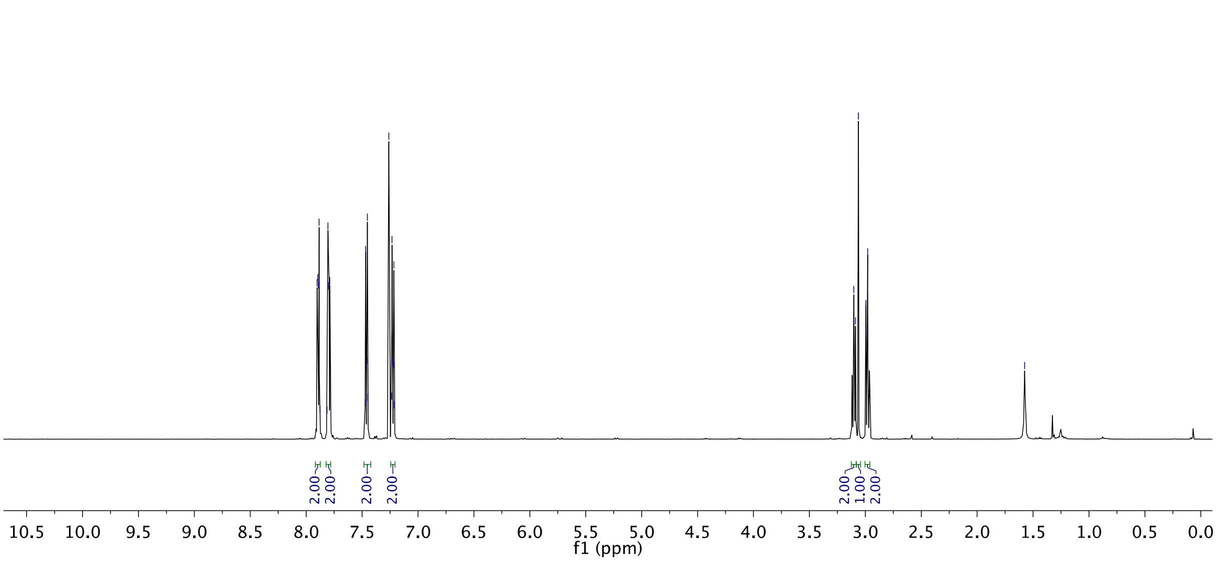

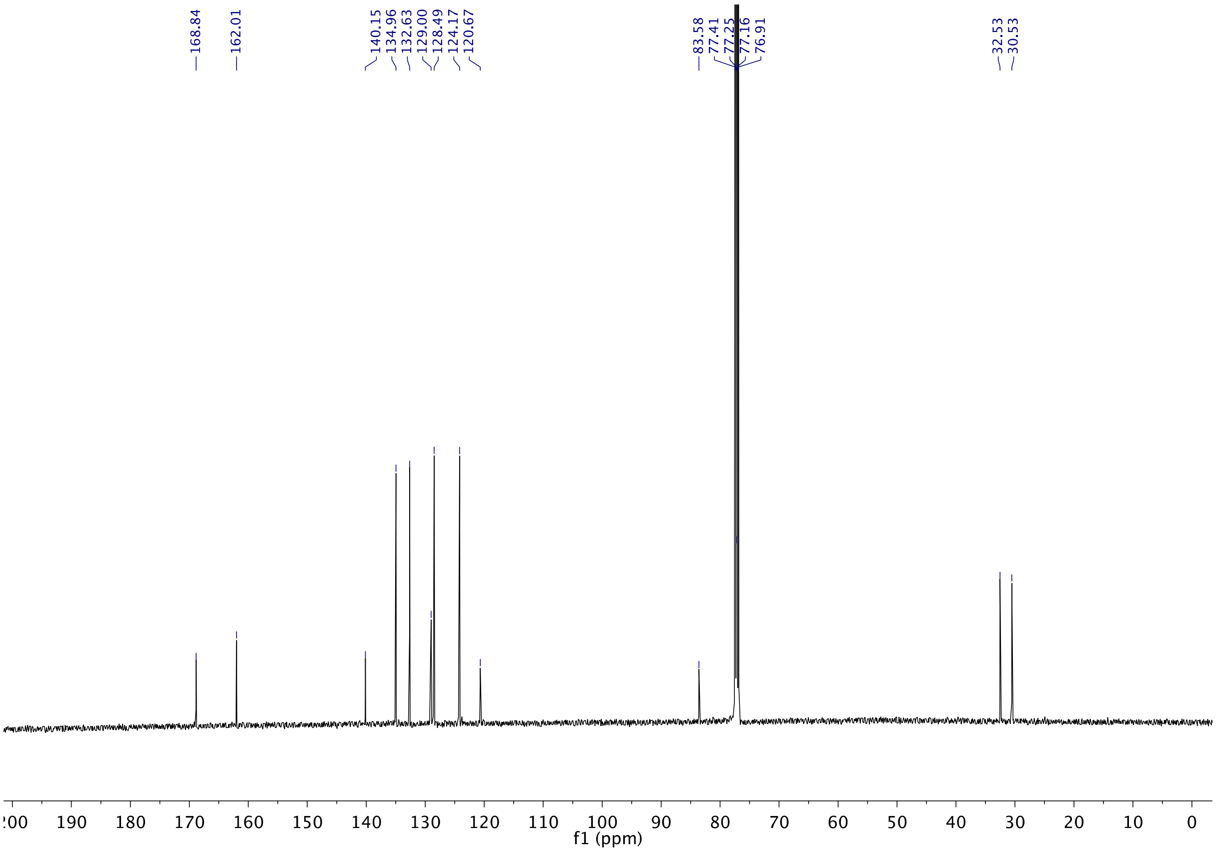

1,3-Dioxoisoindolin-2-yl 1-methylcyclohexane-1-carboxylate, **N12**

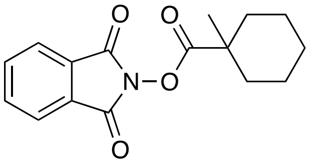

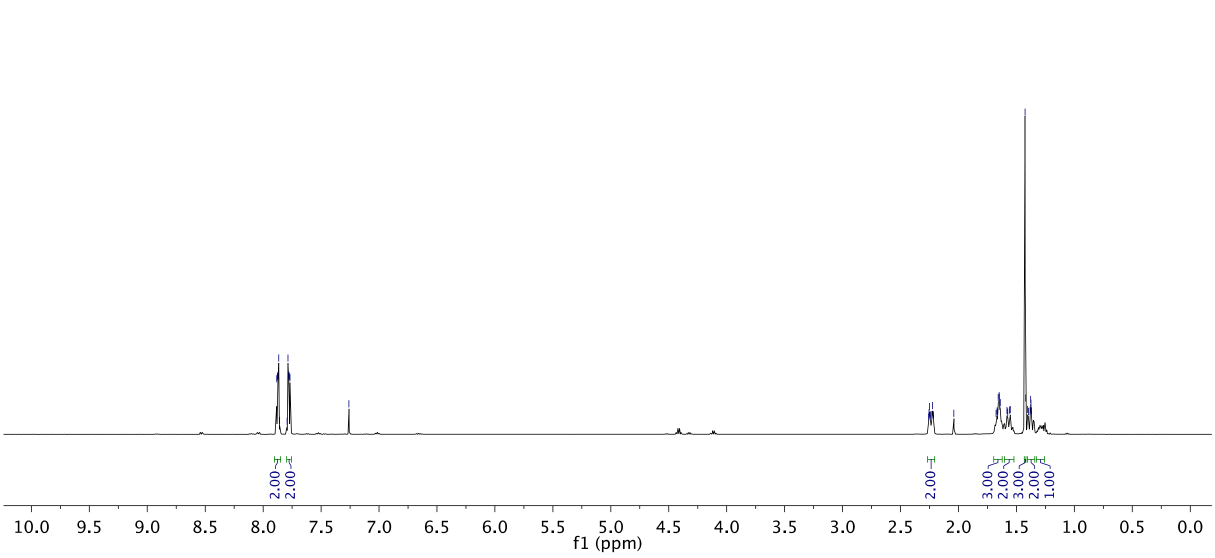

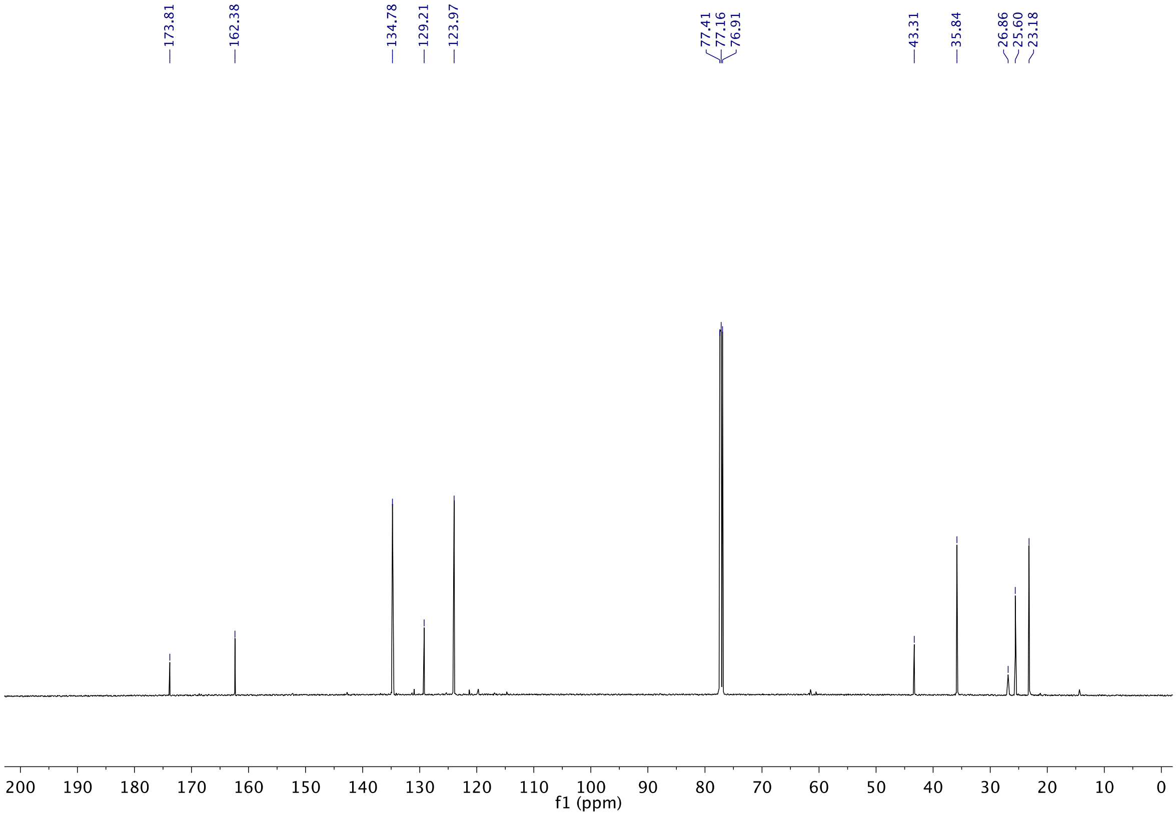

1,3-Dioxoisoindolin-2-yl methyl glutarate, **N13**

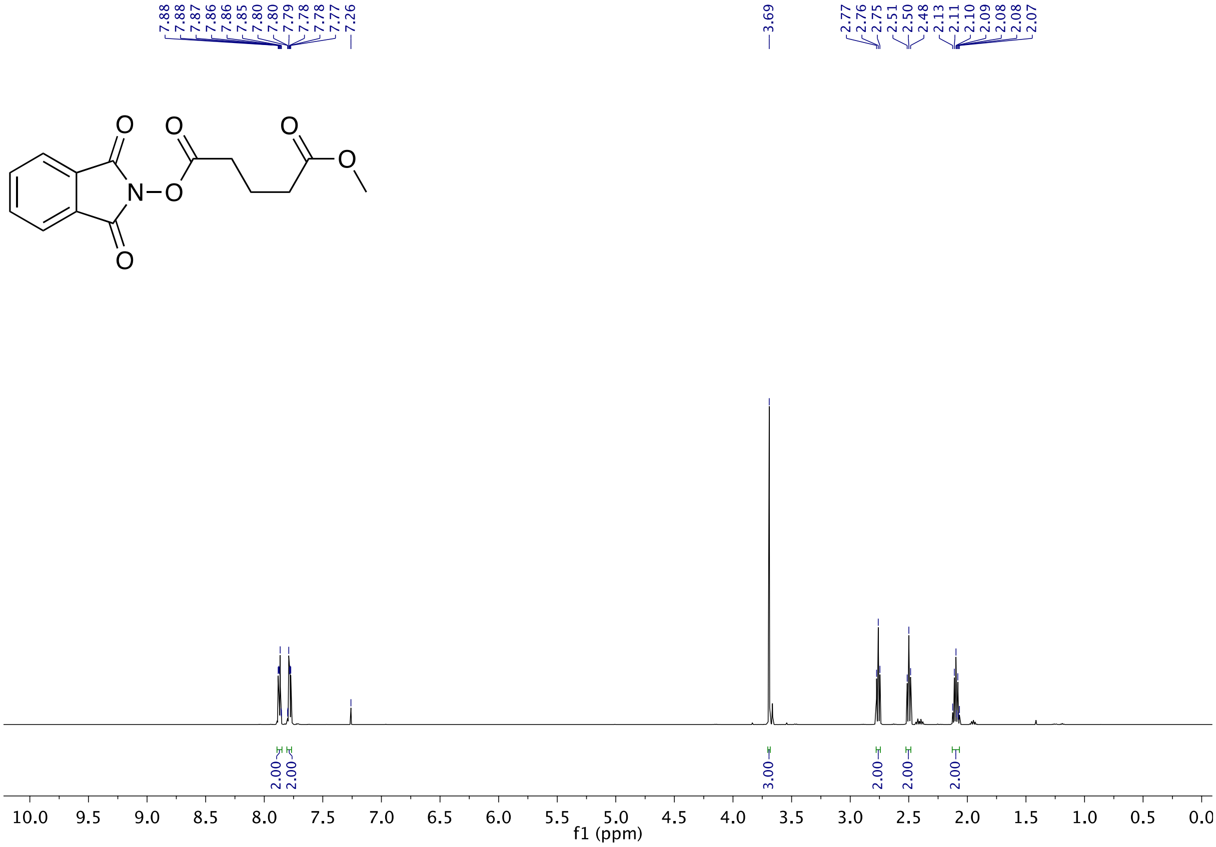

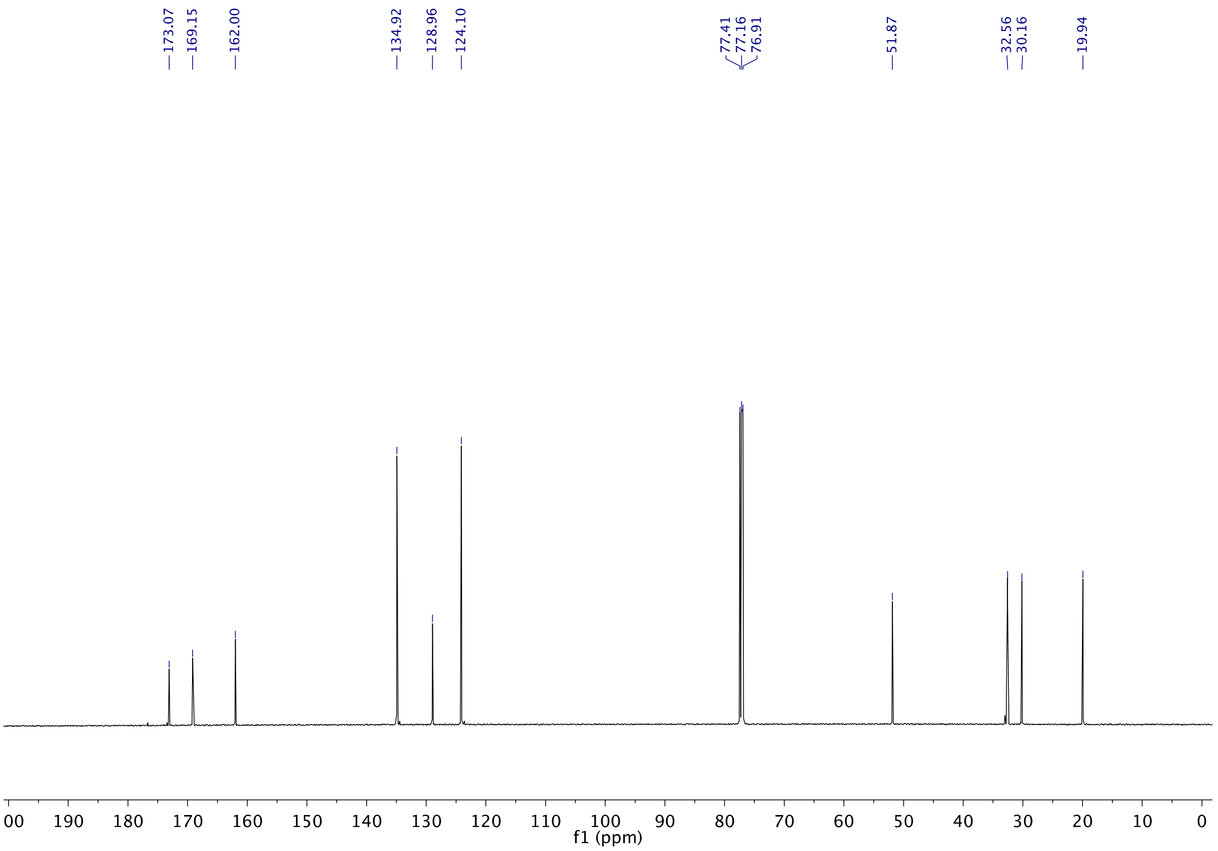

1,3-Dioxoisoindolin-2-yl 4-bromobutanoate, **N14**

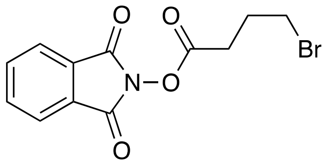

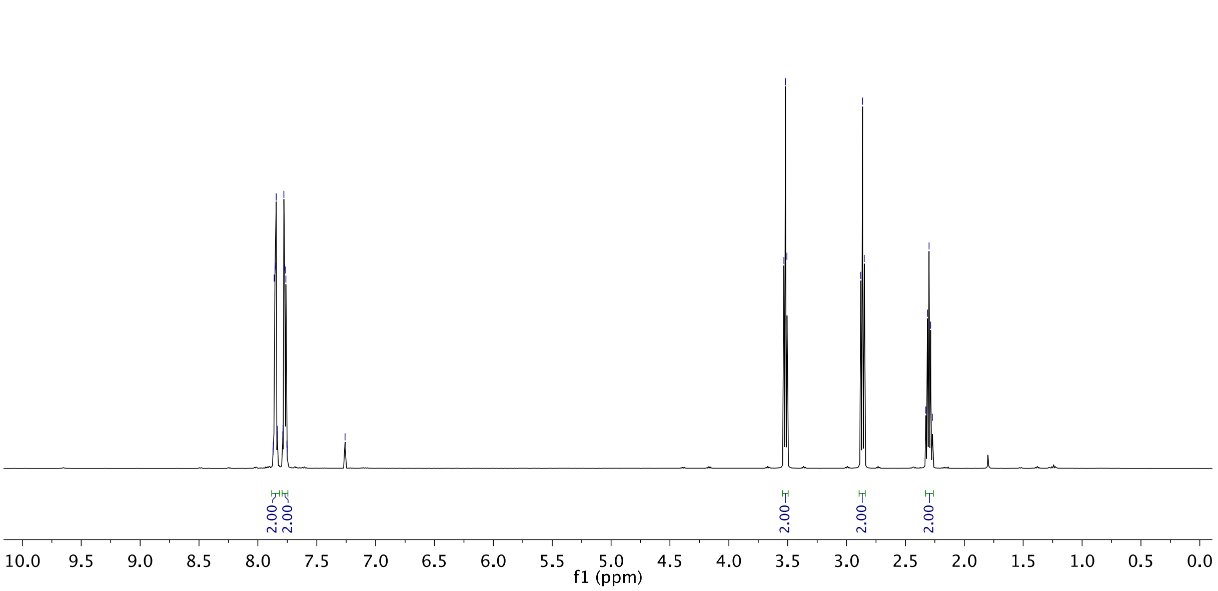

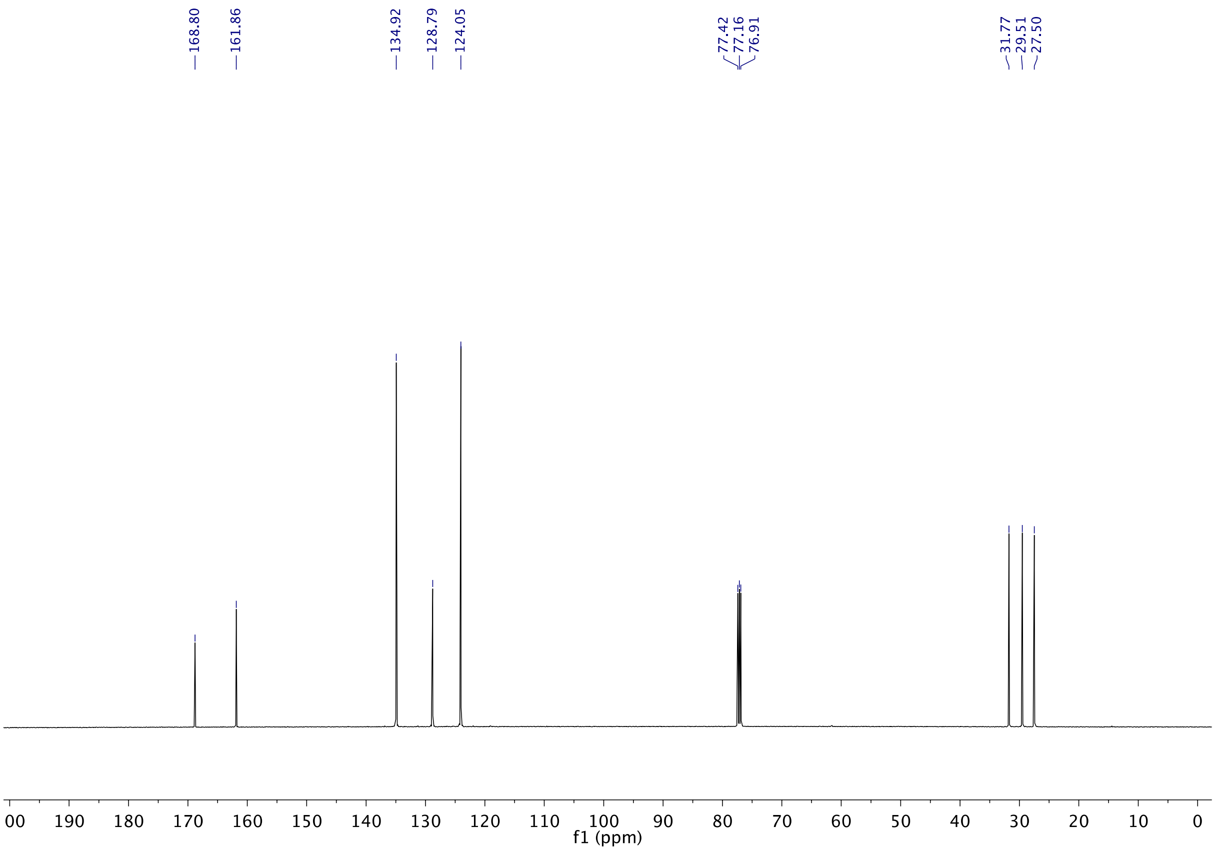

1,3-Dioxoisoindolin-2-yl pent-4-enoate, **N15**

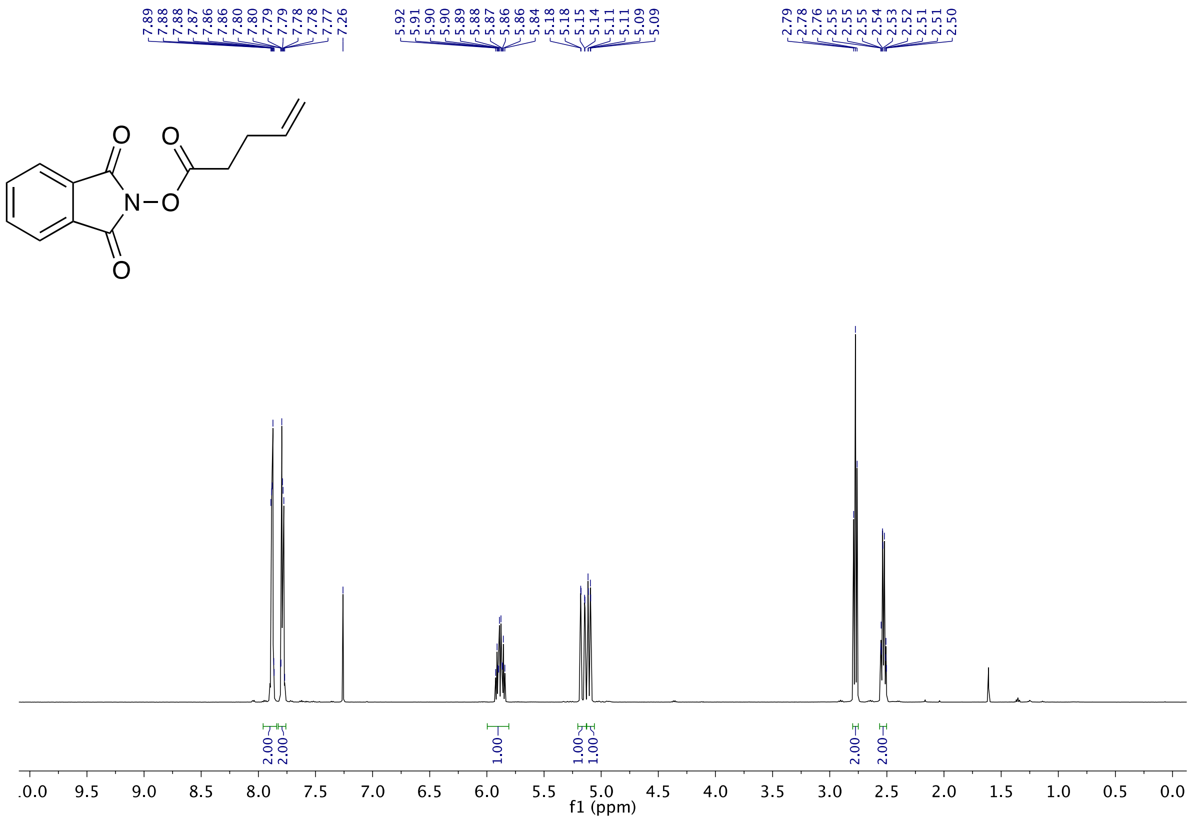

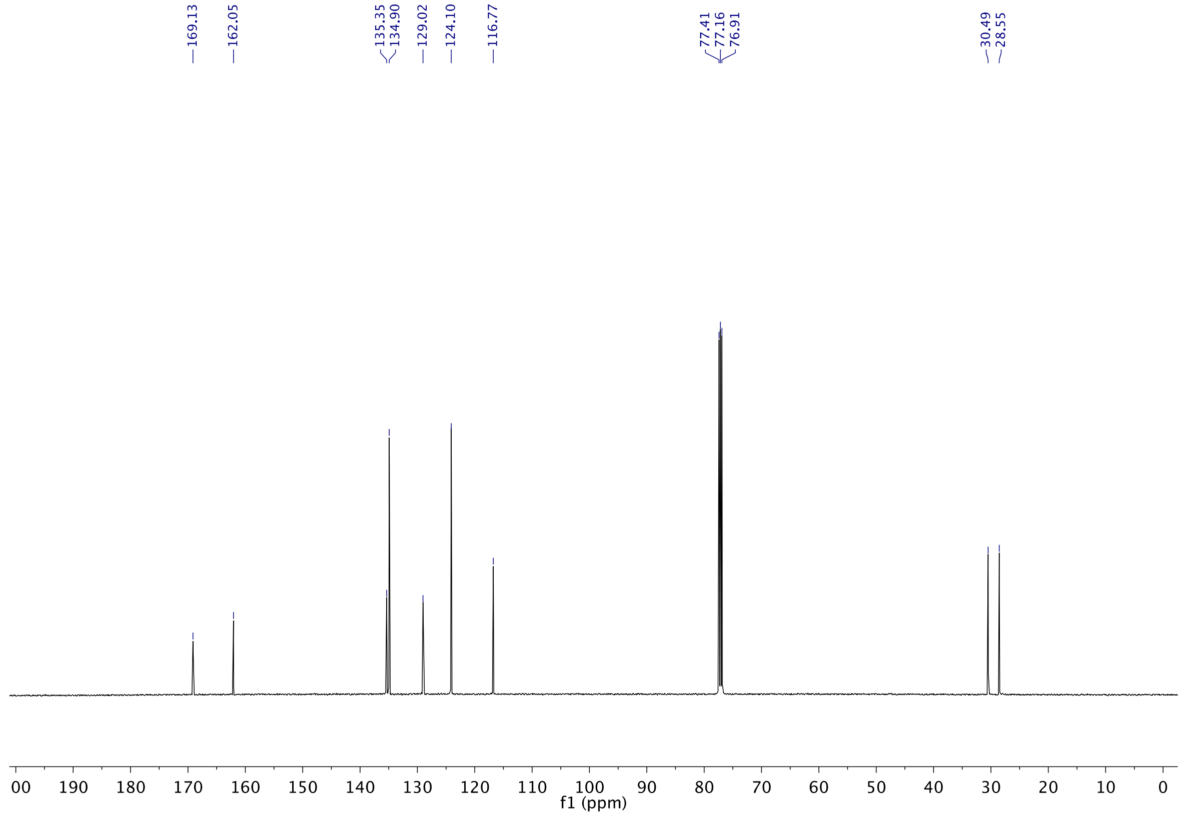

1,3-Dioxoisoindolin-2-yl 2-(cyclopent-2-en-1-yl)acetate, **N16**

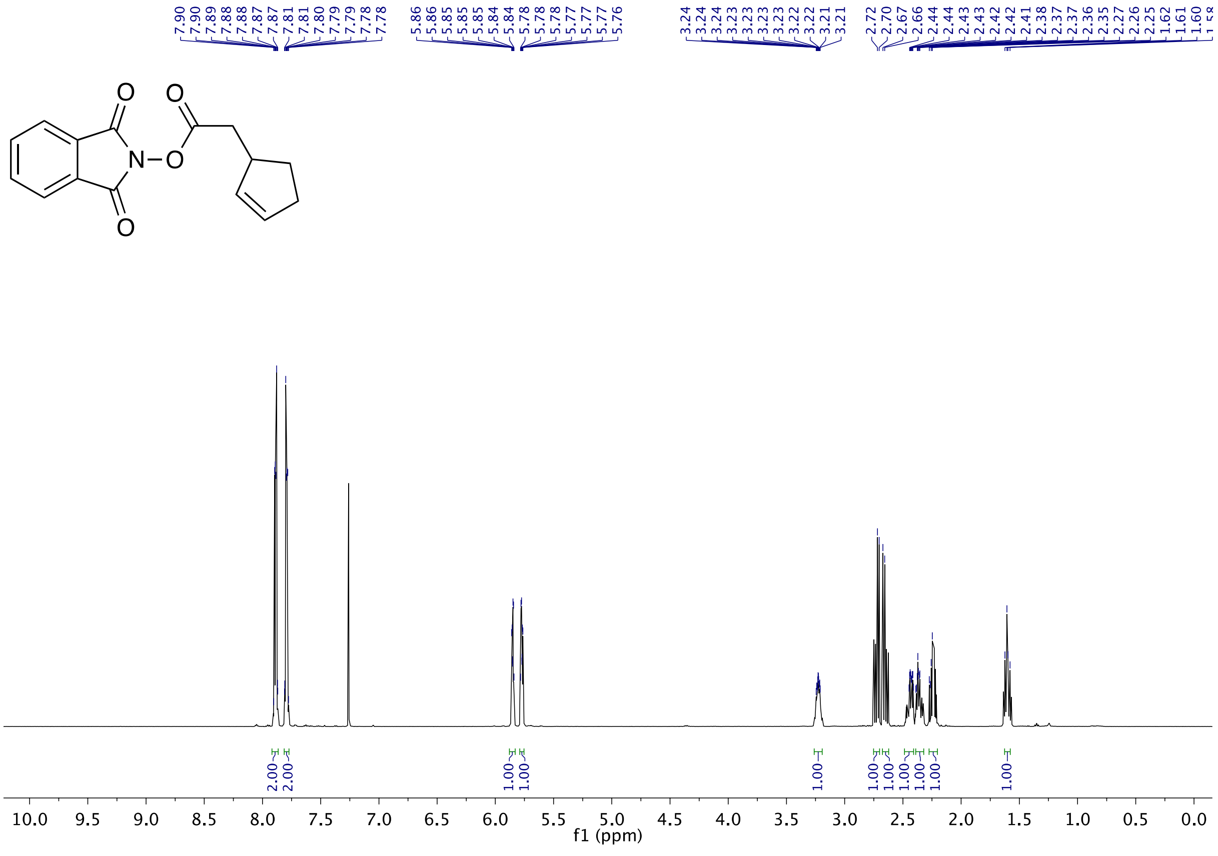

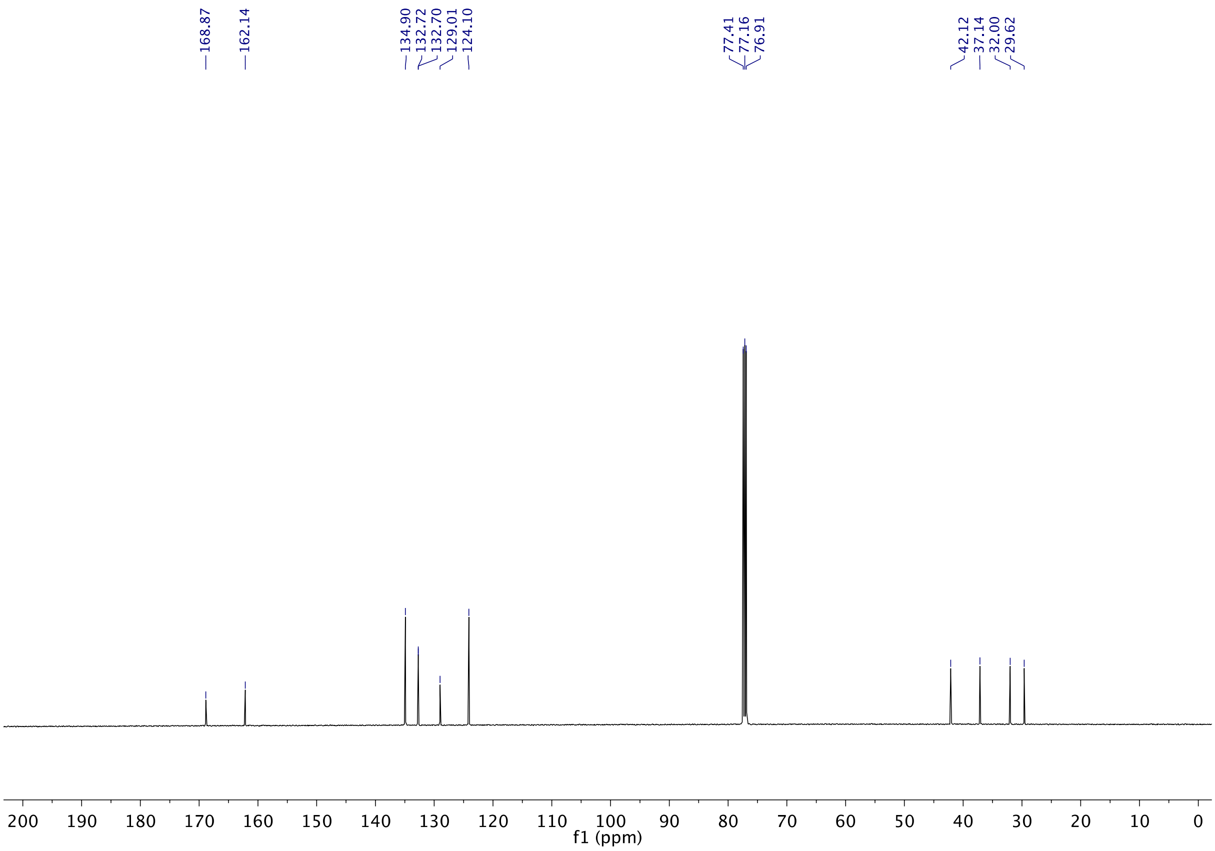

1,3-Dioxoisoindolin-2-yl pent-4-ynoate, **N17**

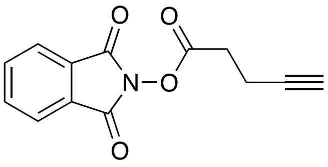

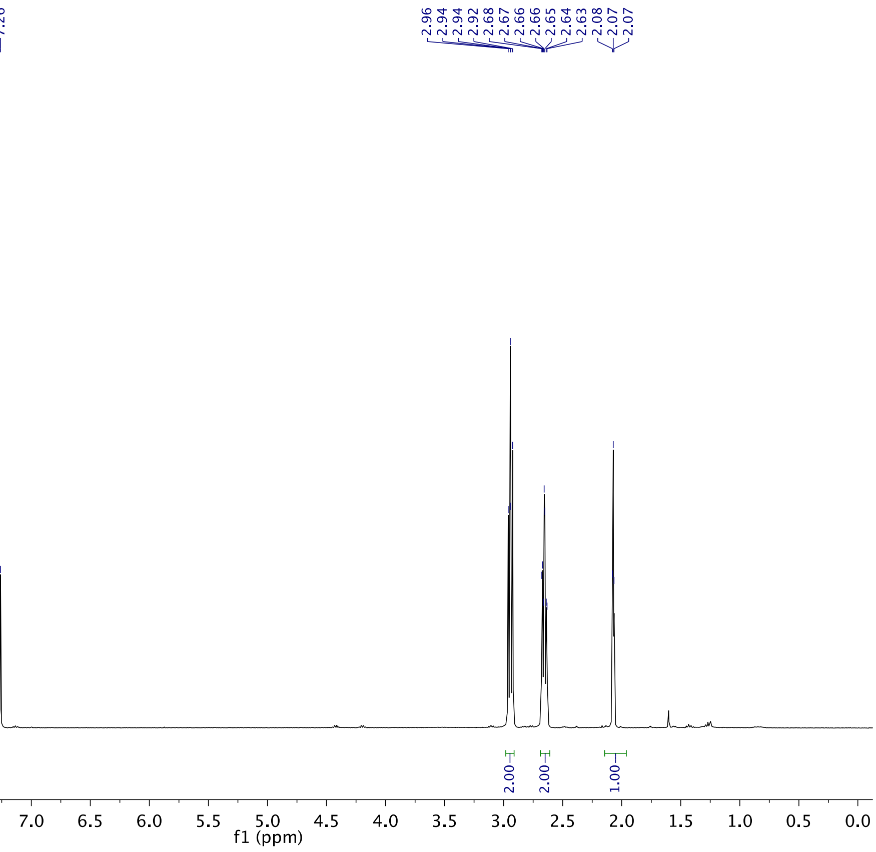

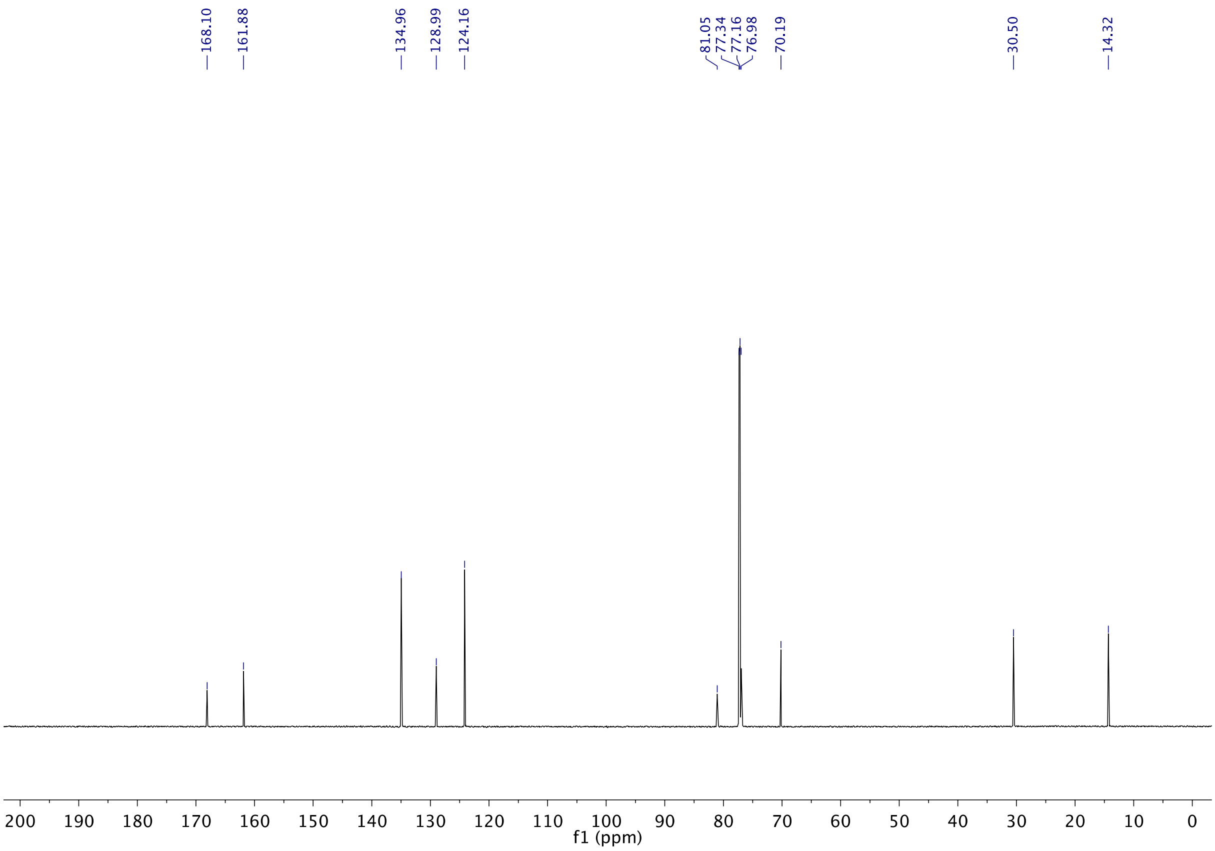

(*tert*-Butoxycarbonyl)glycine, **N18-int1**

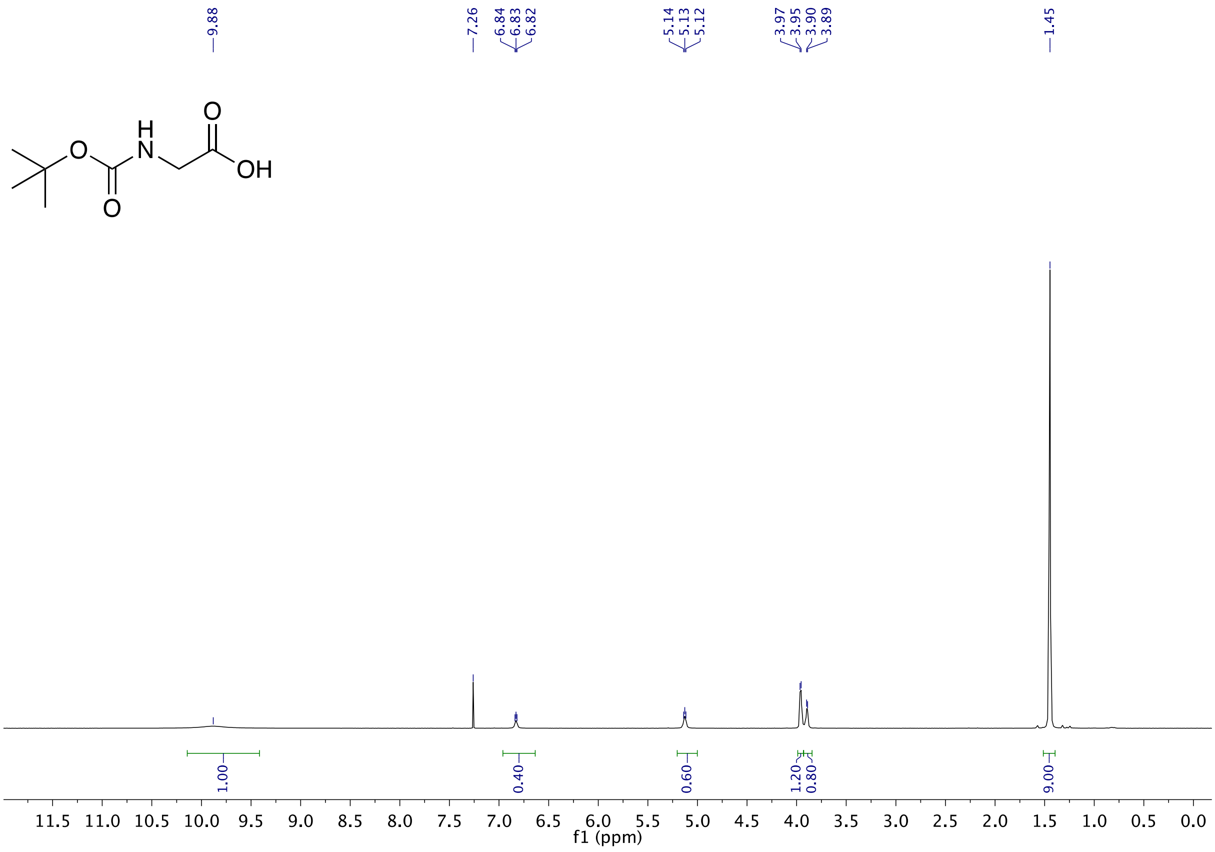

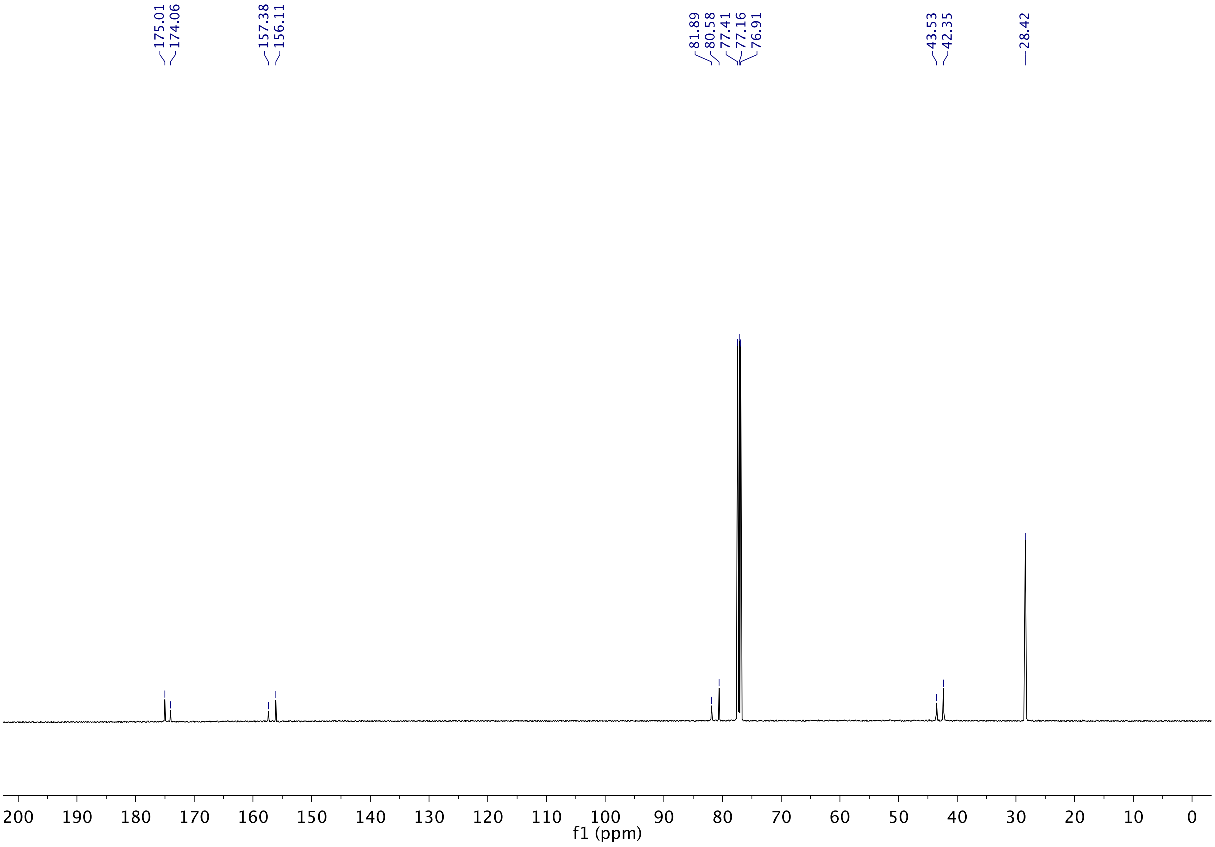

1,3-Dioxoisoindolin-2-yl (*tert*-butoxycarbonyl)glycinate, **N18**

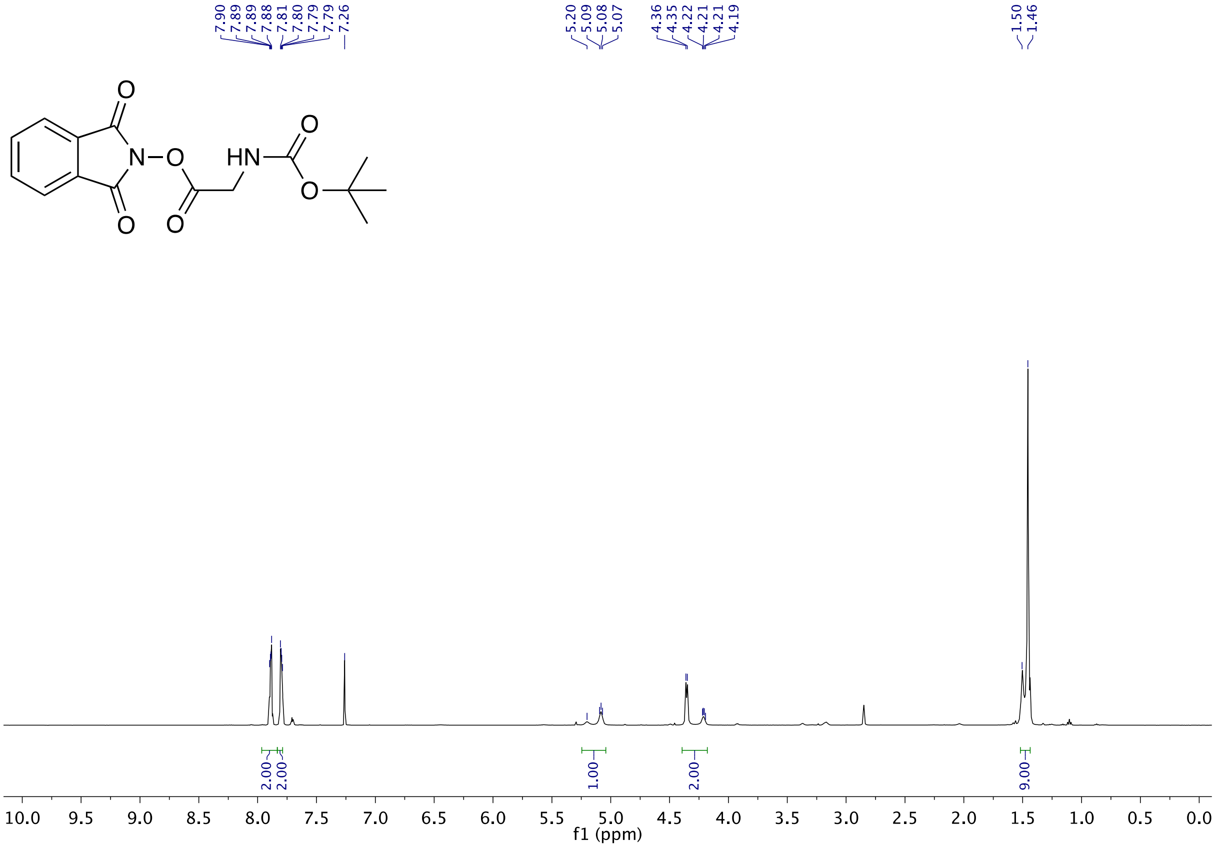

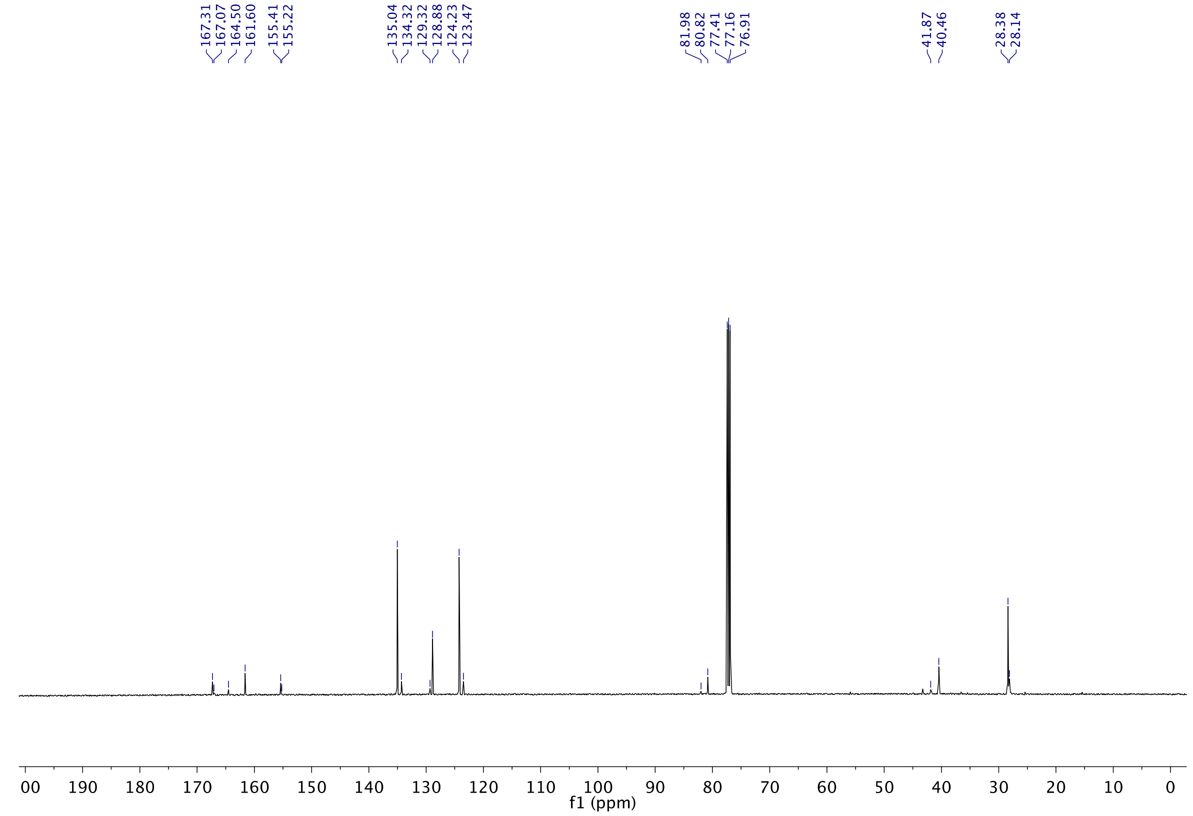

(*tert*-Butoxycarbonyl)alanine, **N19-int1**

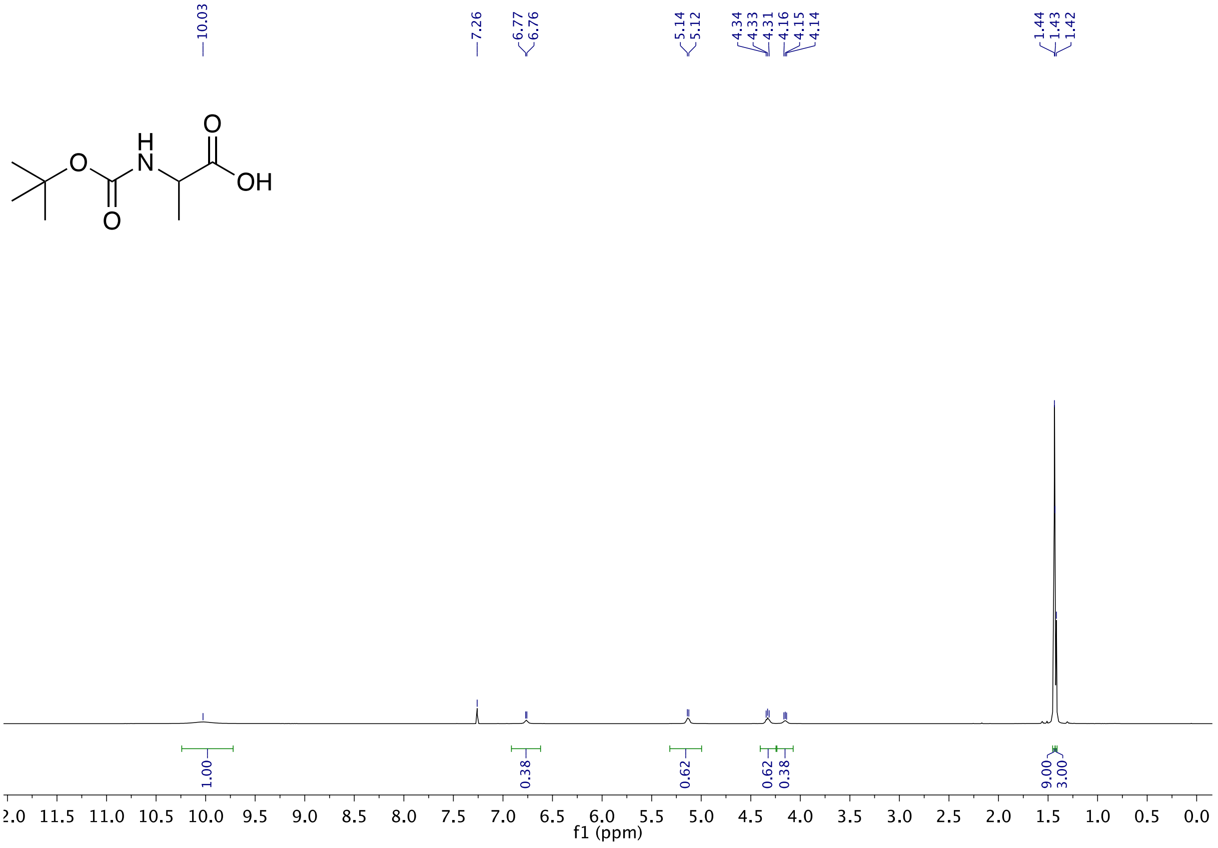

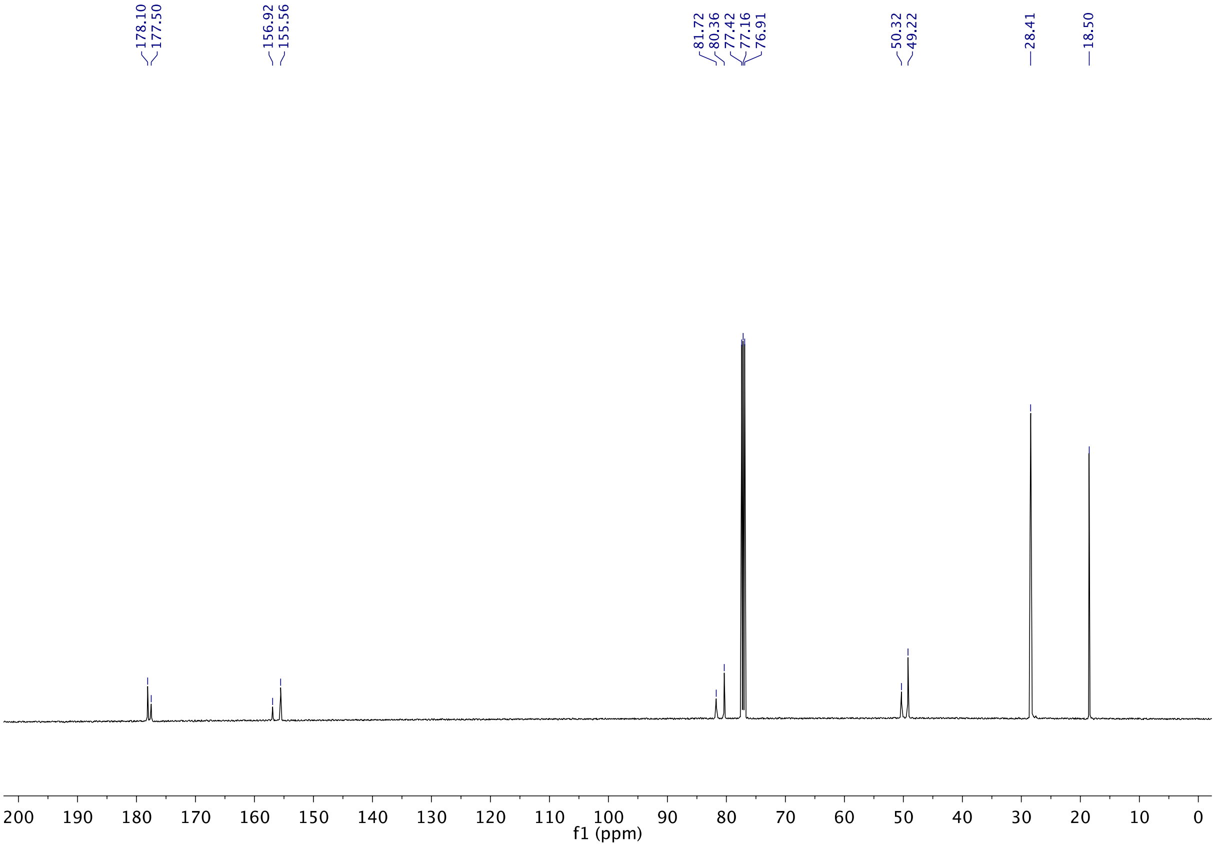

1,3-Dioxoisoindolin-2-yl (*tert*-butoxycarbonyl)alaninate, **N19**

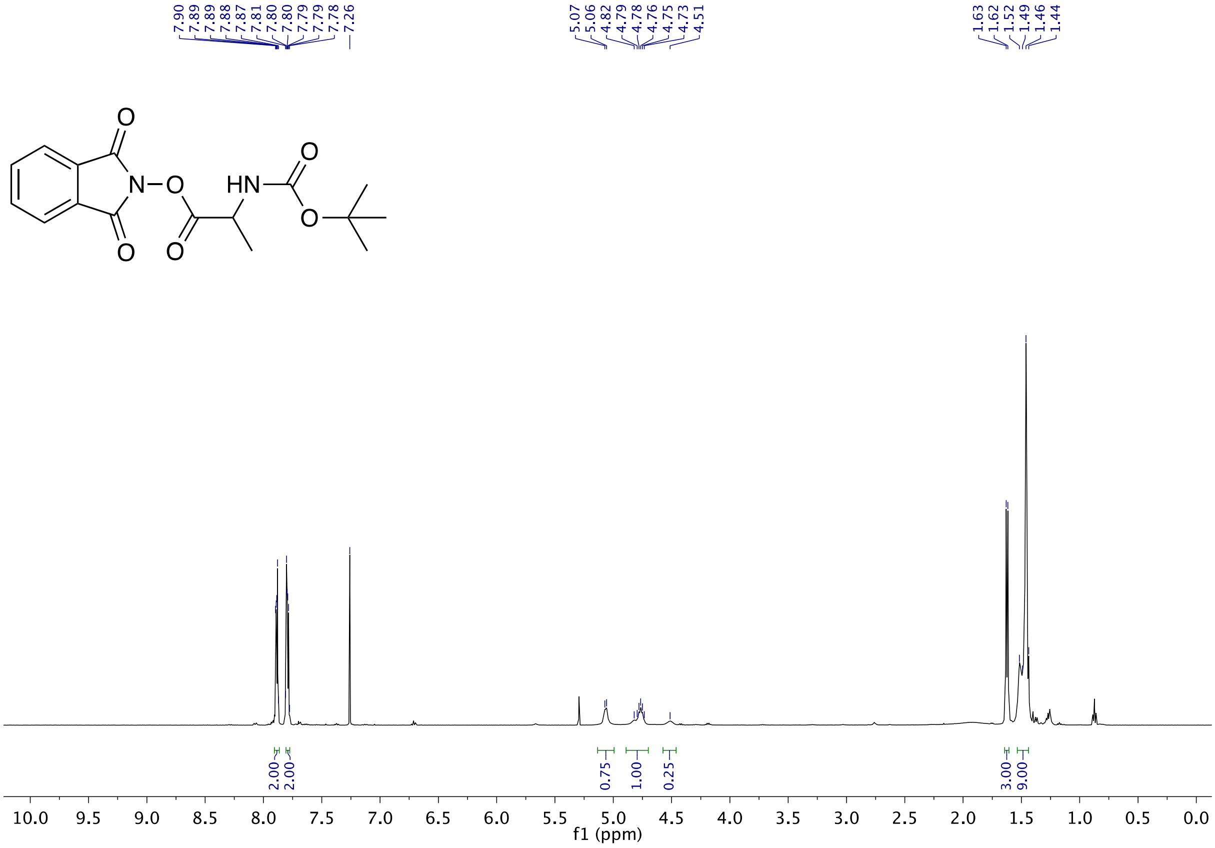

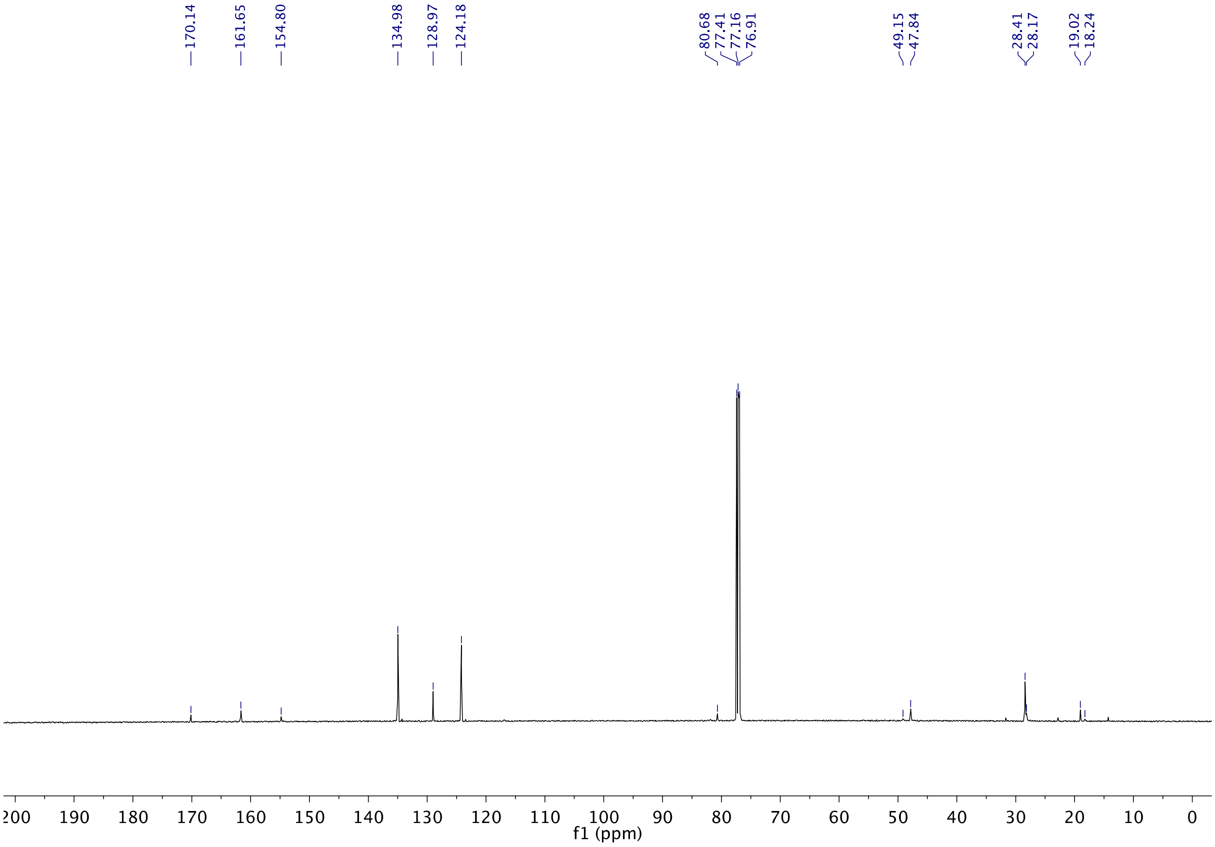

Methyl 3-((*tert*-butyldimethylsilyl)oxy)-2,2-dimethylpropanoate, **N20-int1**

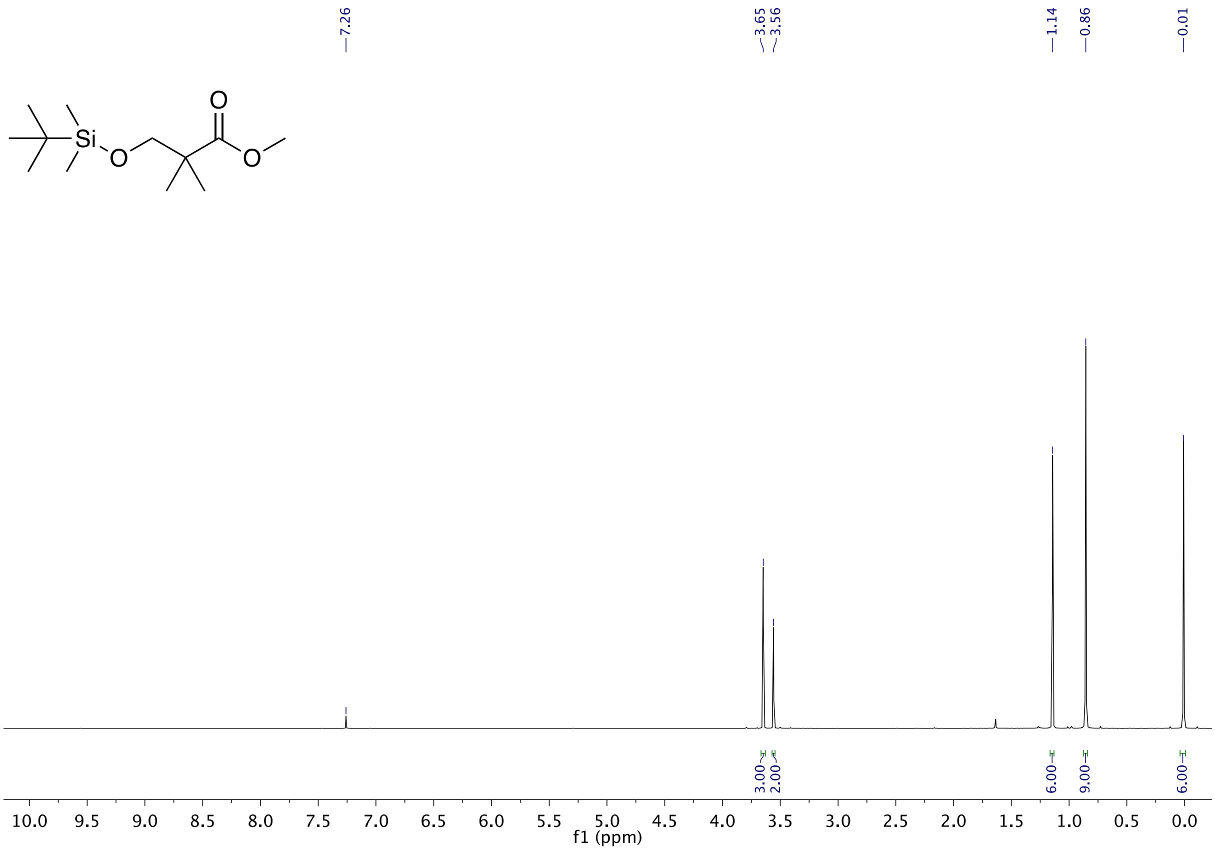

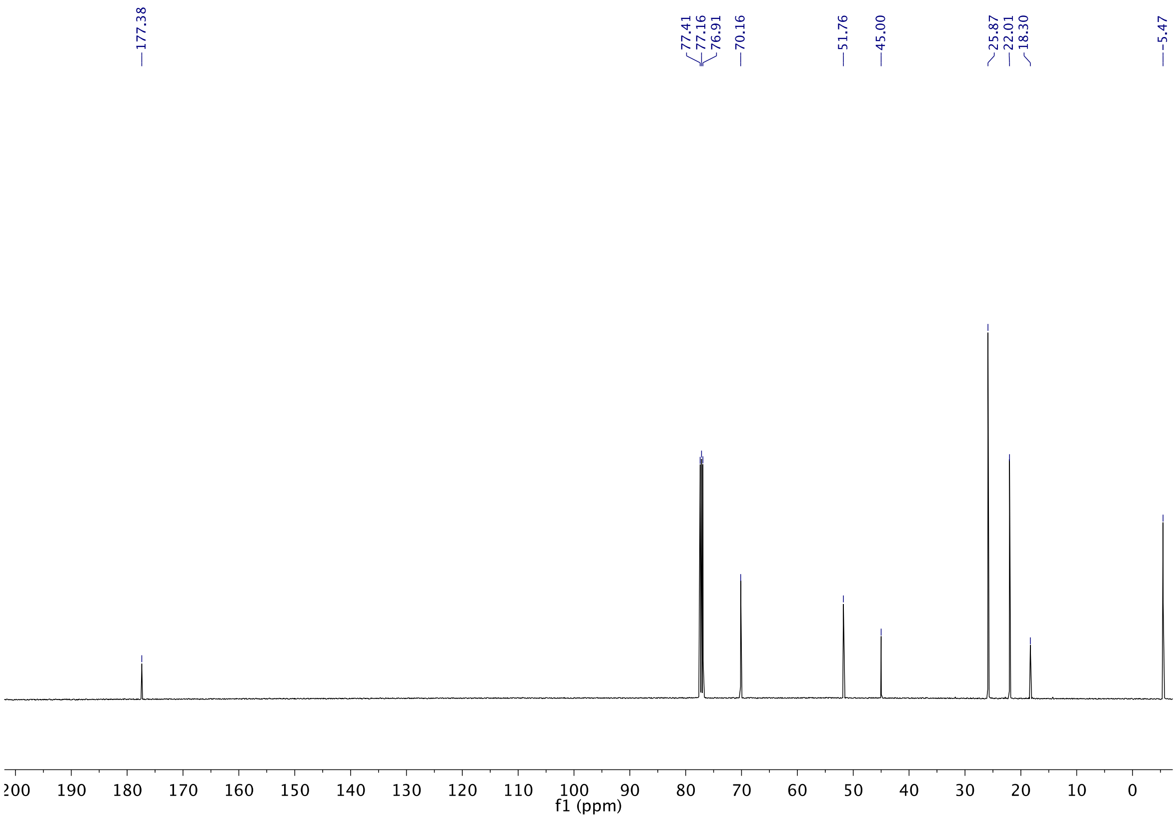

3-((*tert*-Butyldimethylsilyl)oxy)-2,2-dimethylpropanoic acid, **N20-int2**

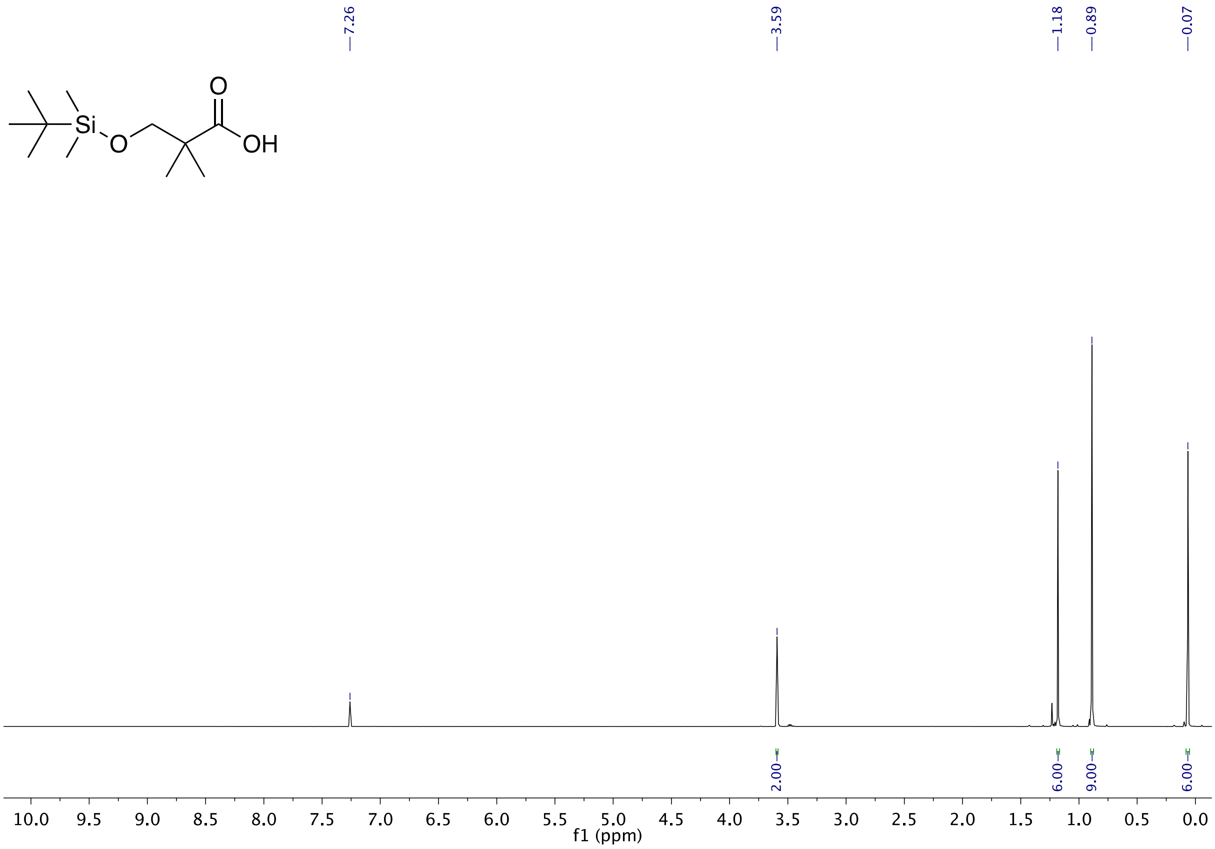

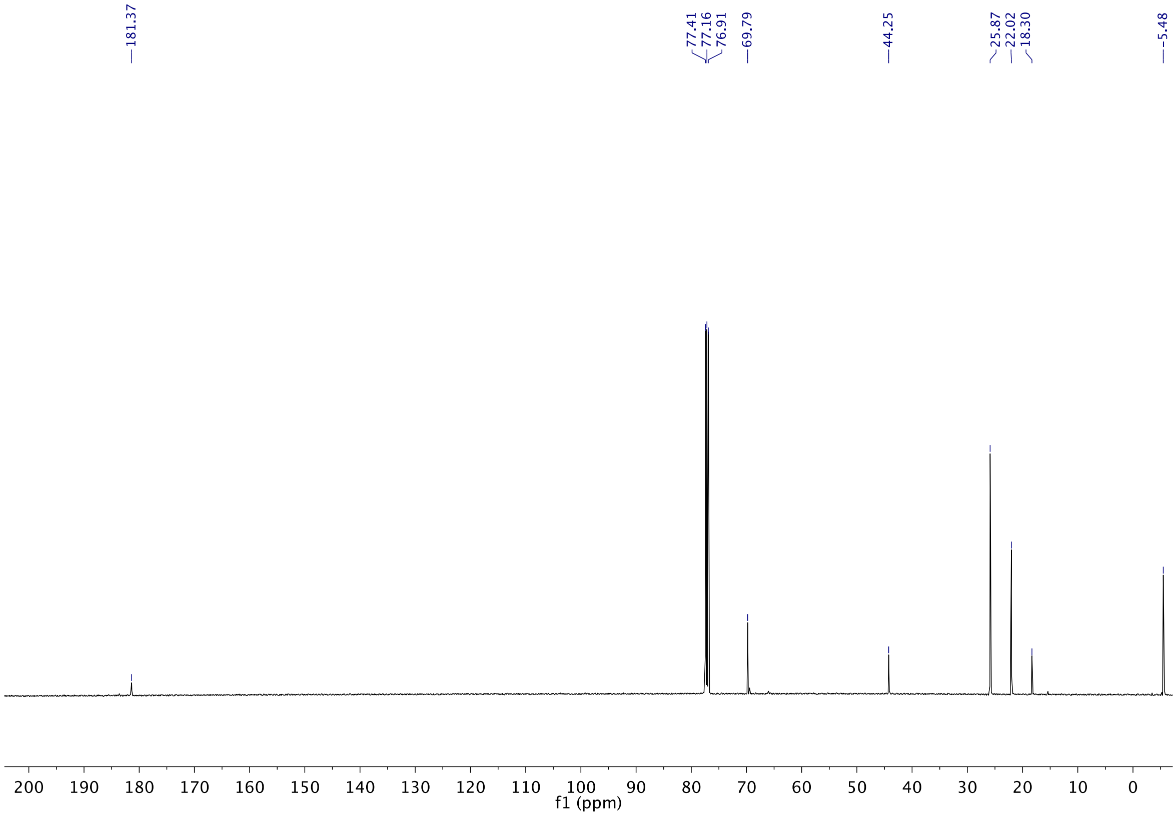

1,3-Dioxoisoindolin-2-yl 3-((*tert*-butyldimethylsilyl)oxy)-2,2-dimethylpropanoate, **N20**

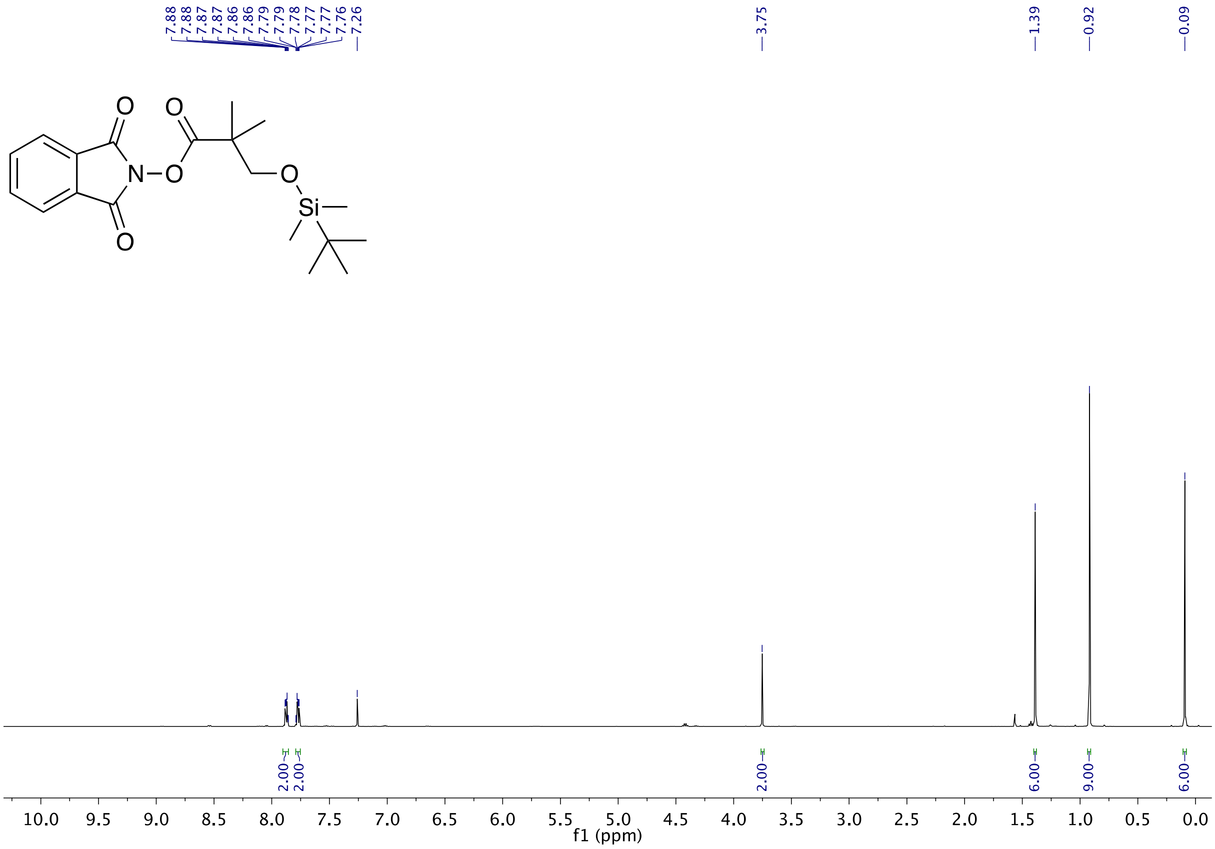

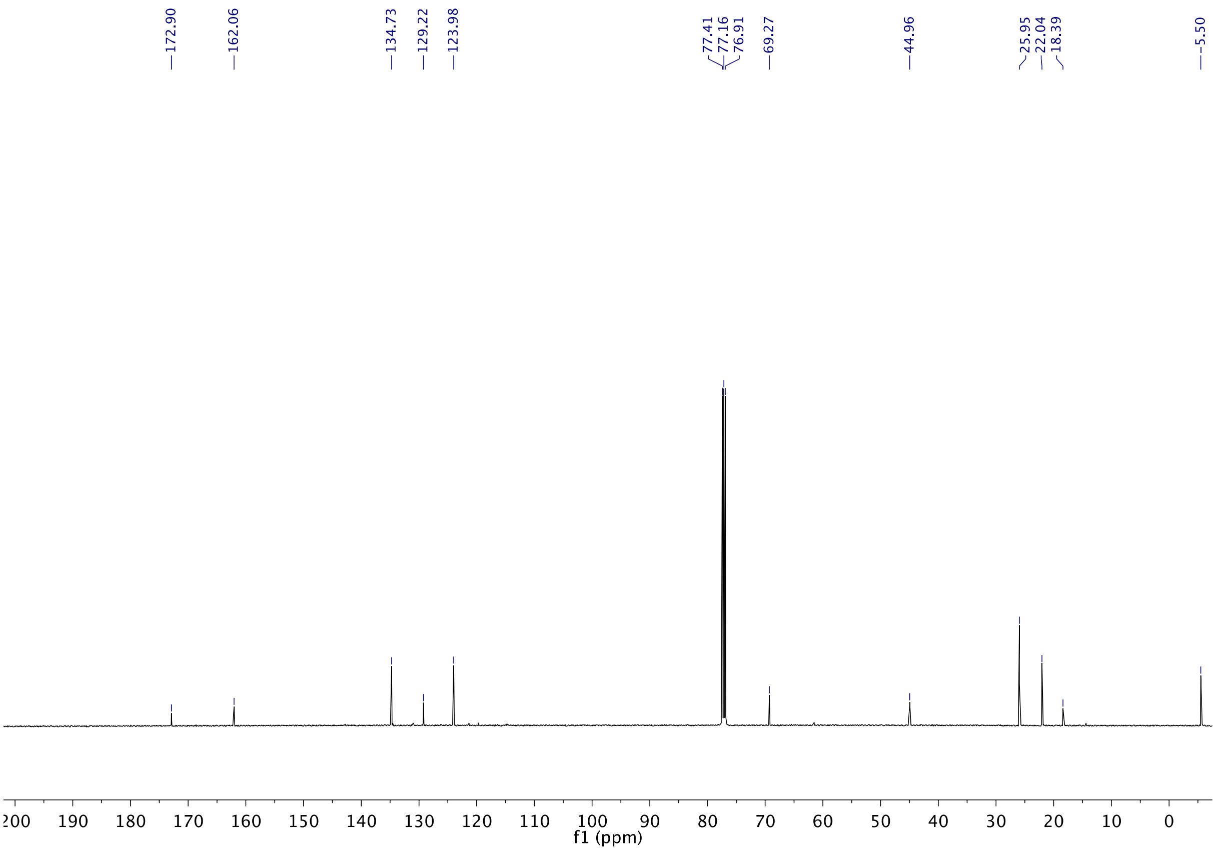

1,3-Dioxoisoindolin-2-yl 3-methyloxetane-3-carboxylate, **N21**

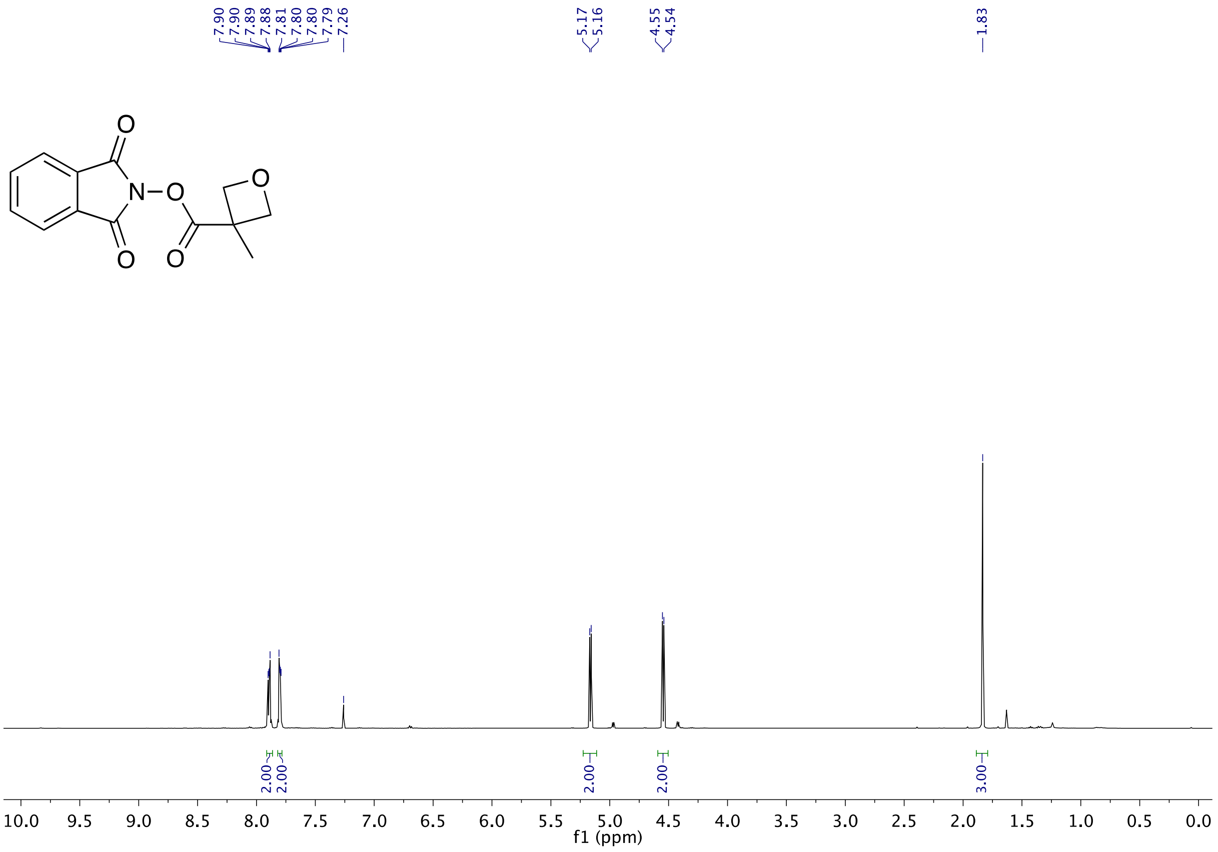

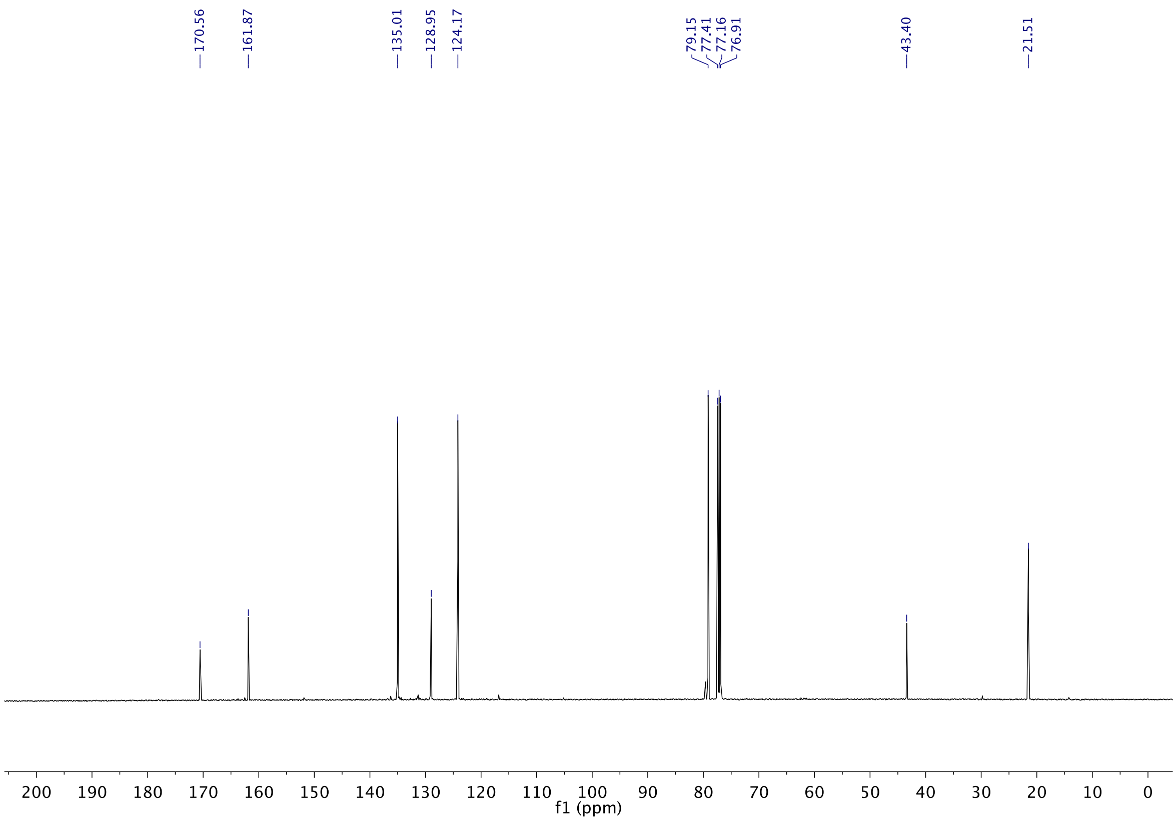

1,3-Dioxoisoindolin-2-yl 3-oxocyclobutane-1-carboxylate, **N22**

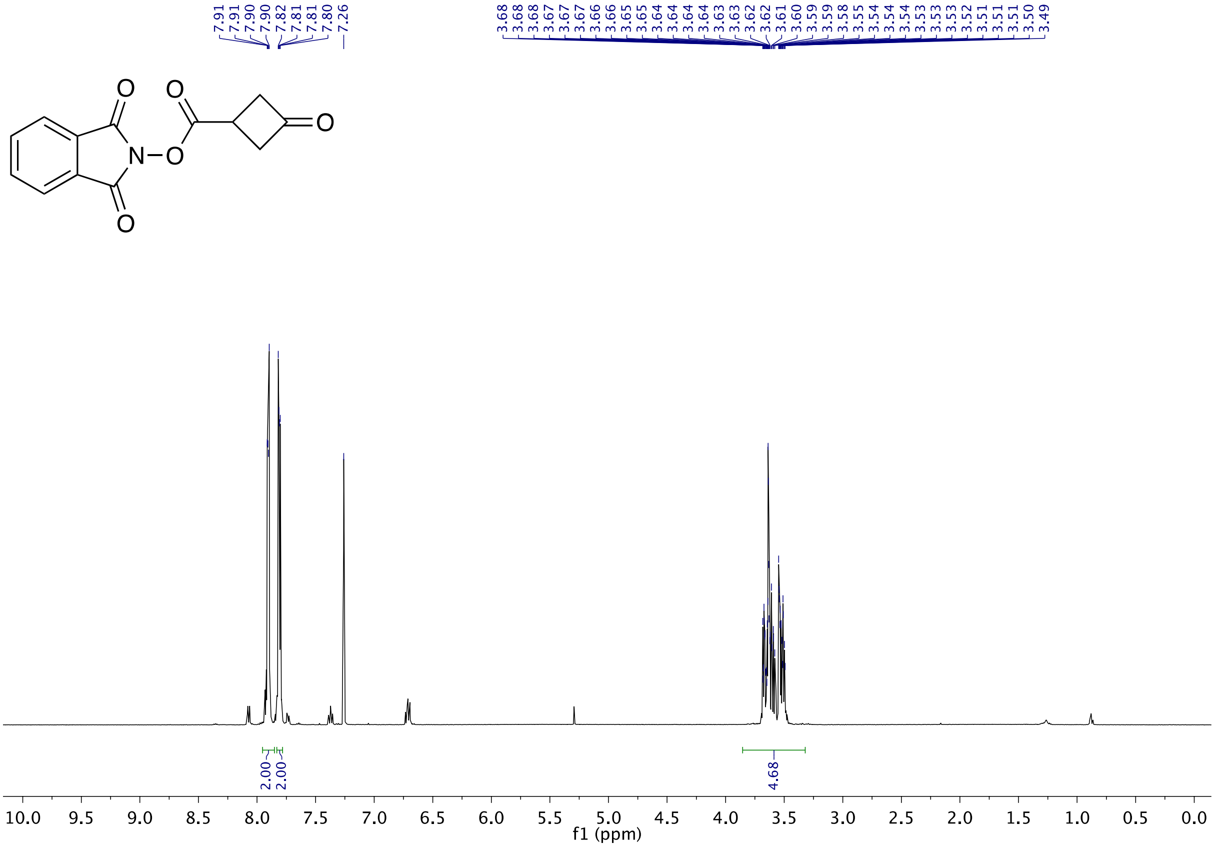

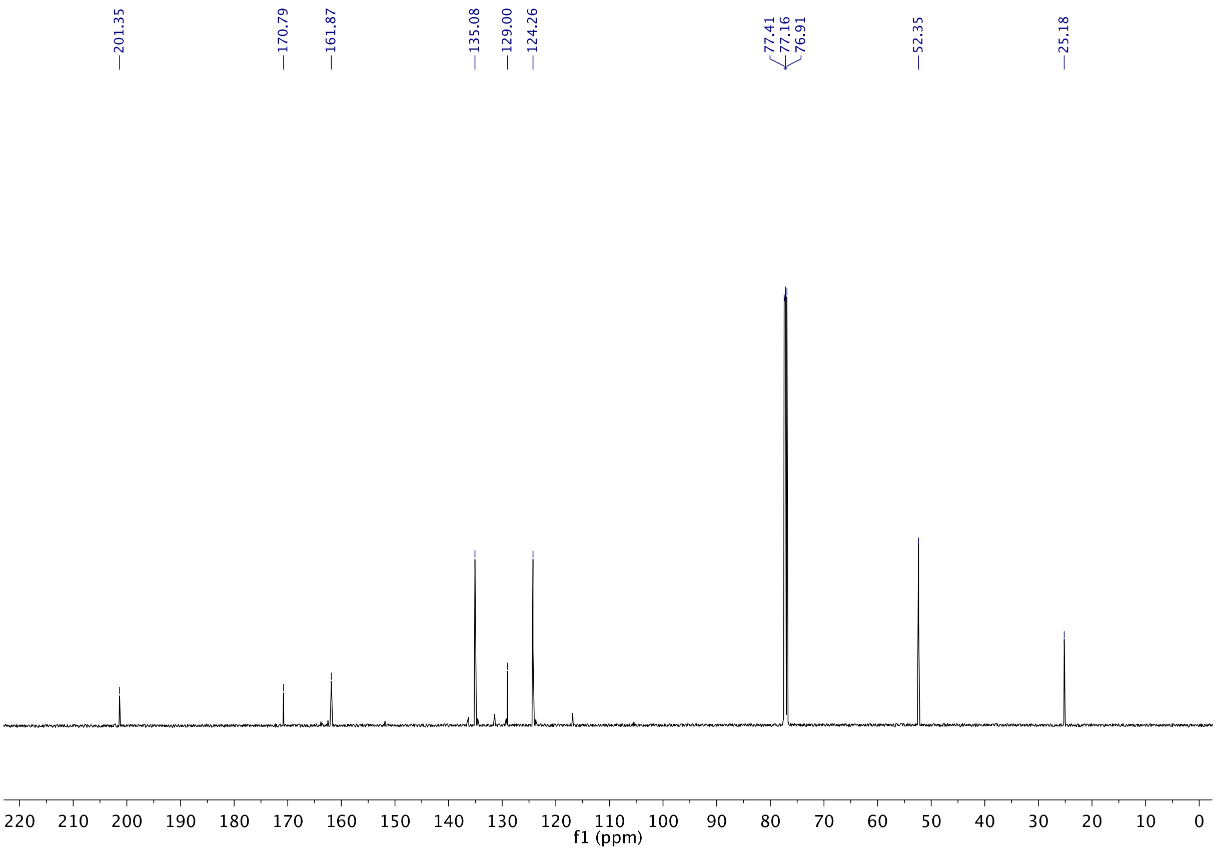

1-(*tert*-Butoxycarbonyl)piperidine-4-carboxylic acid, **N23-int1**

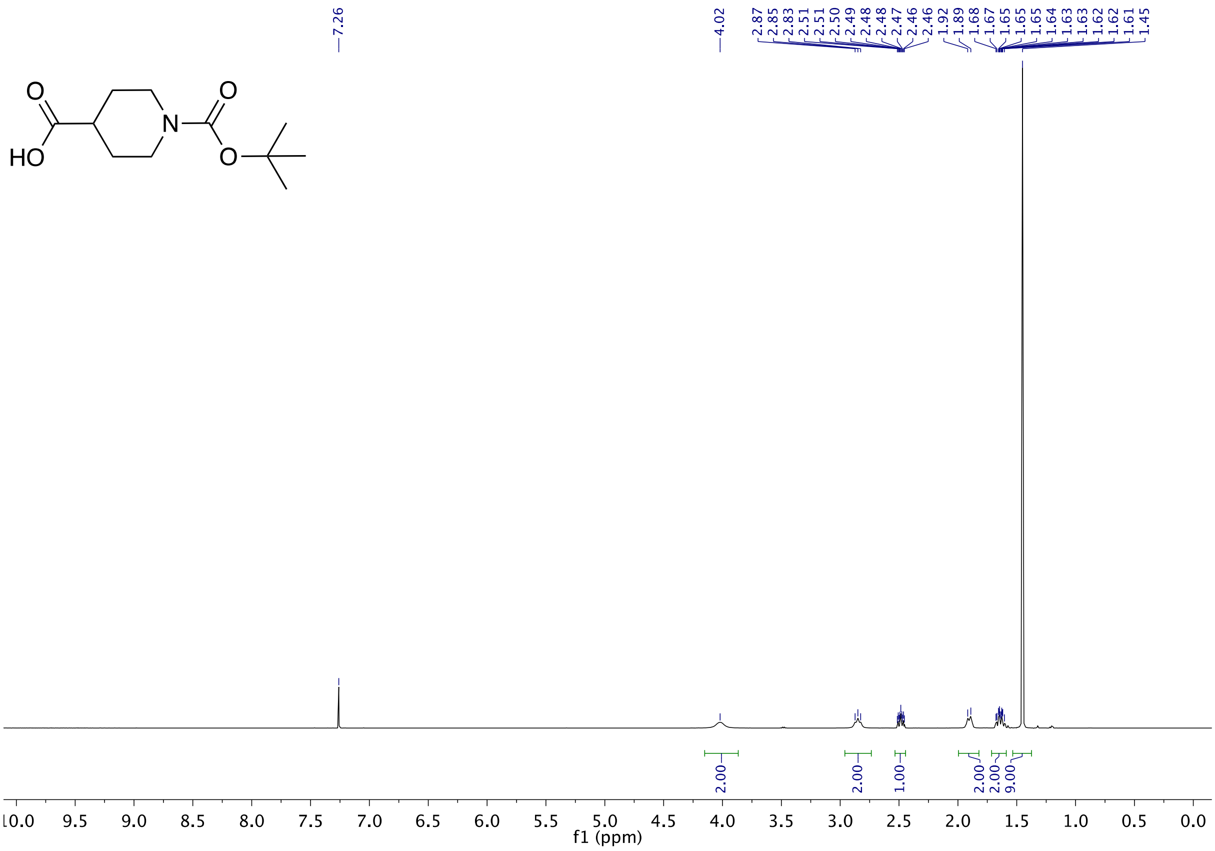

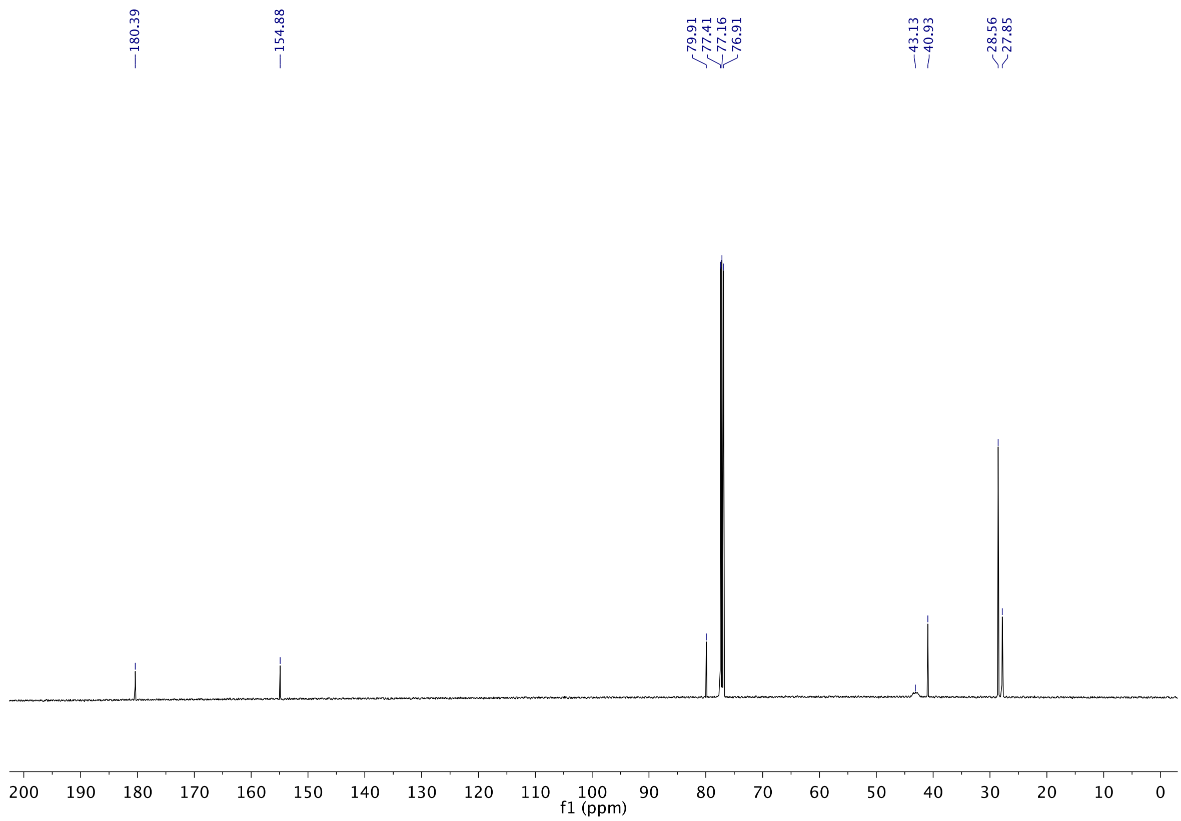

1-(*tert*-Butyl) 4-(1,3-dioxoisoindolin-2-yl) piperidine-1,4-dicarboxylate, **N23**

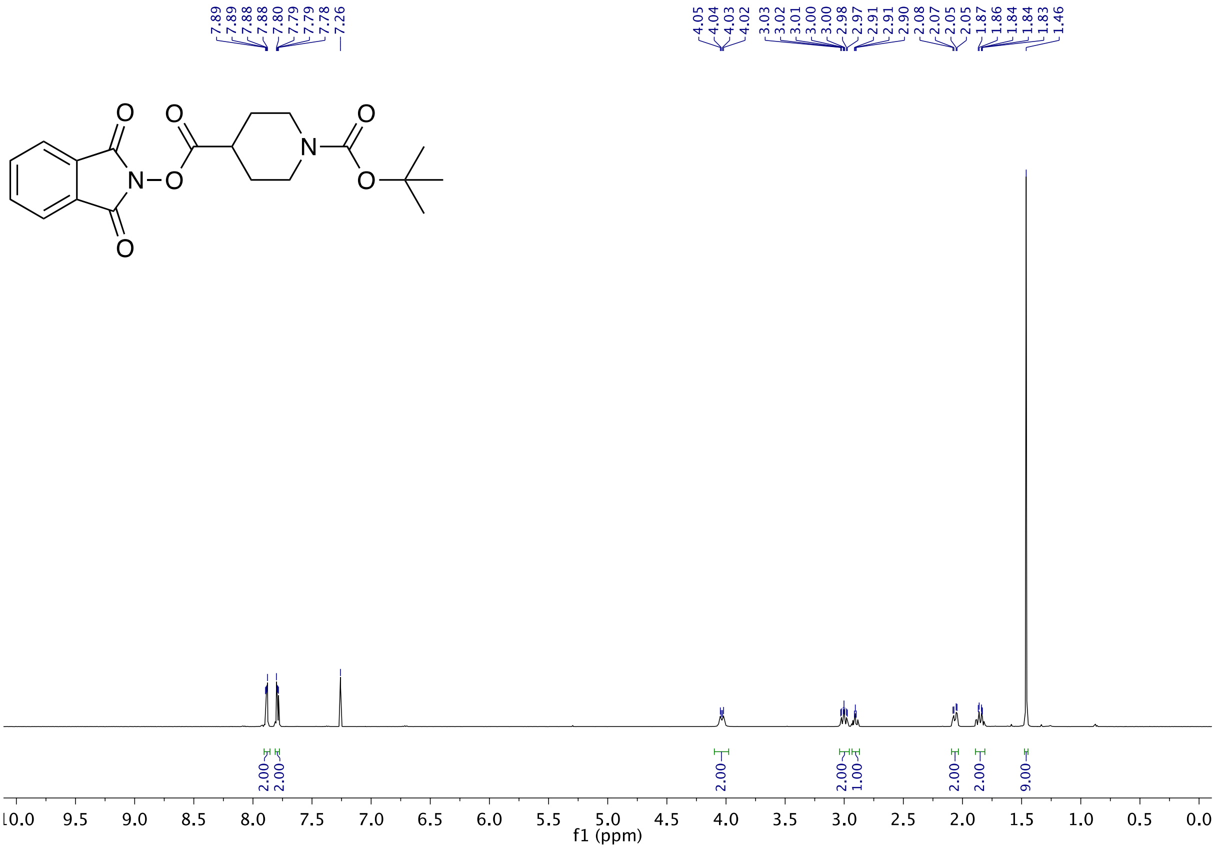

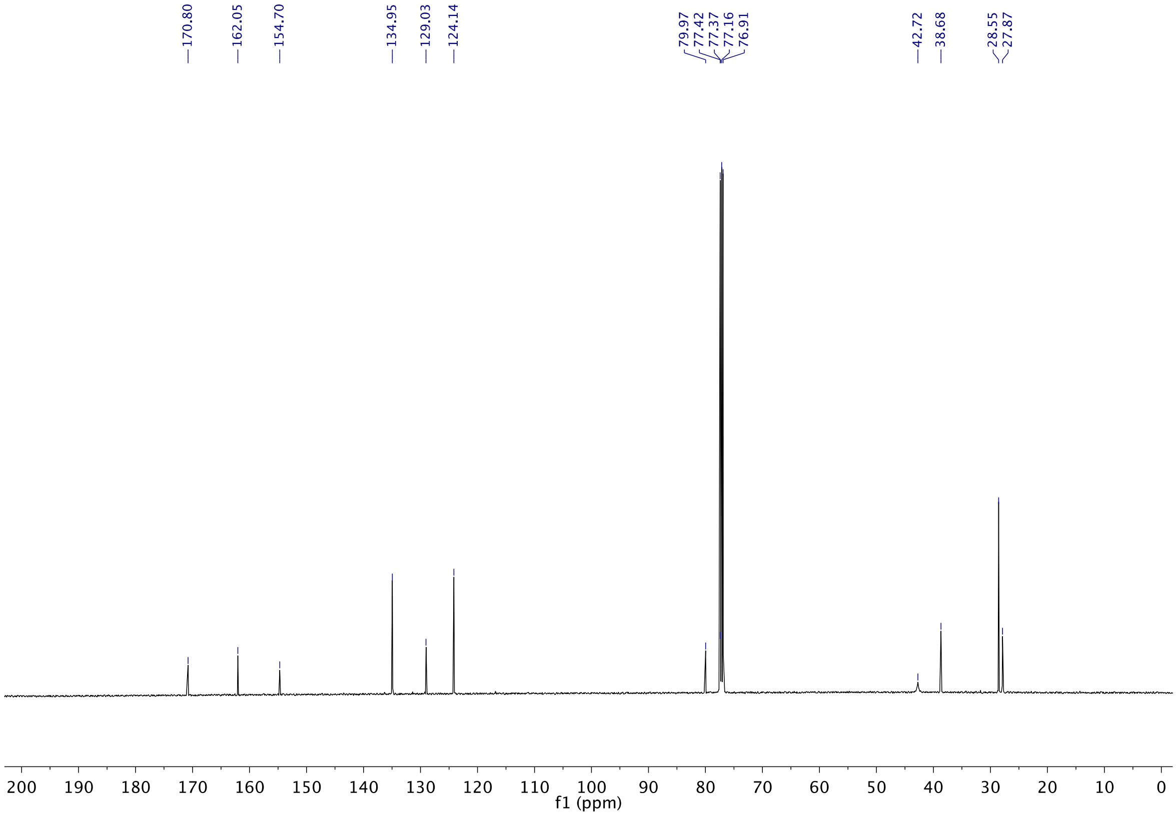

1-(*tert*-Butoxycarbonyl)piperidine-3-carboxylic acid, **N24-int1**

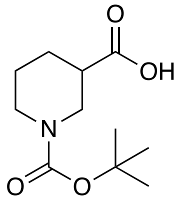

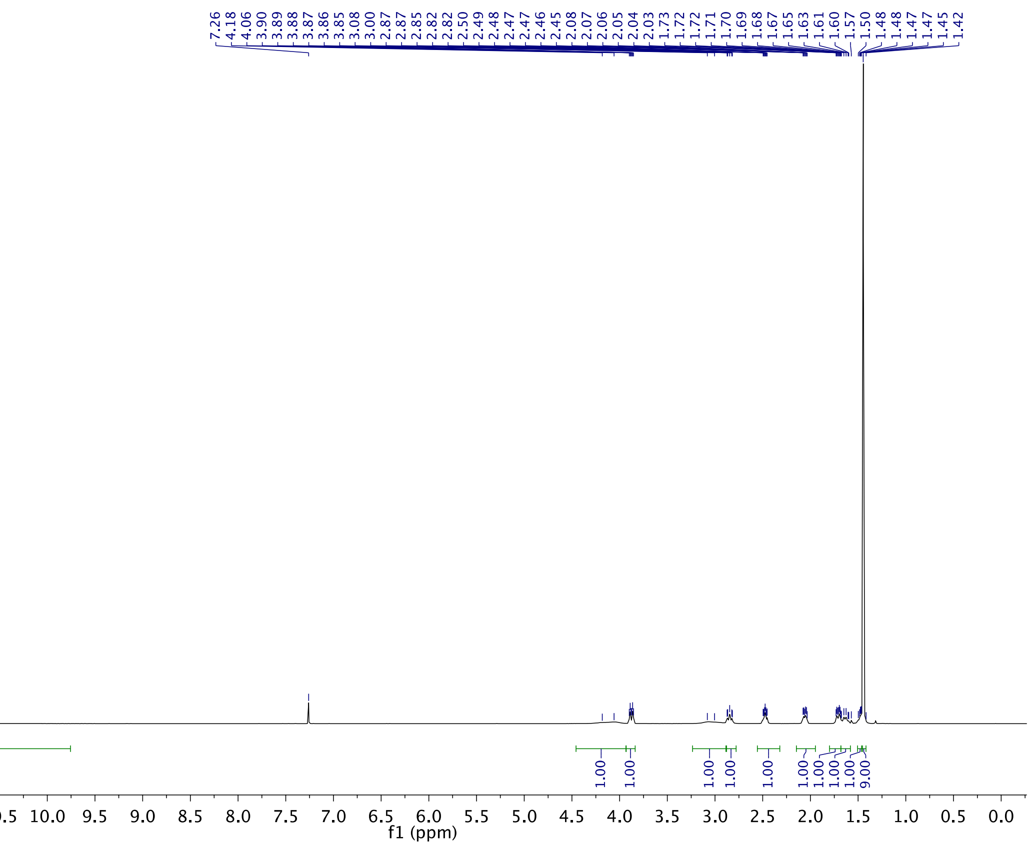

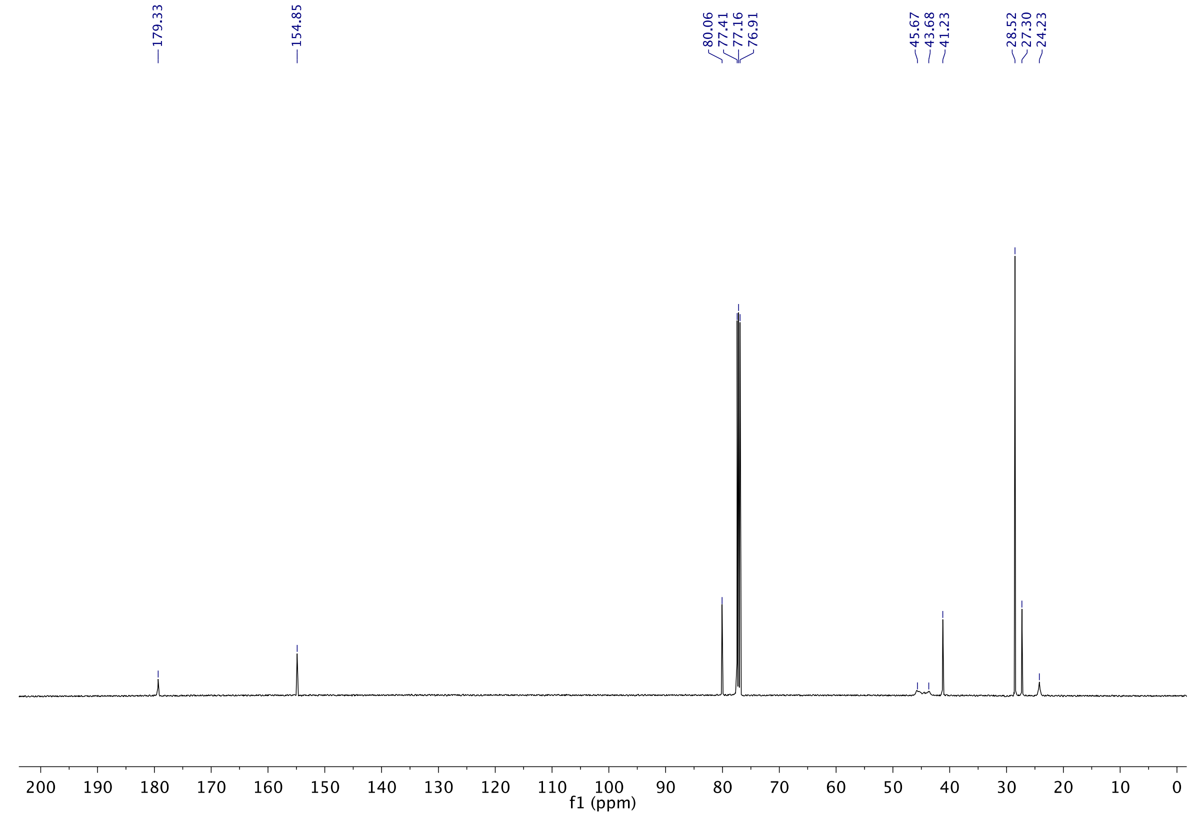

1-(*tert*-Butyl) 3-(1,3-dioxoisoindolin-2-yl) piperidine-1,3-dicarboxylate, **N24**

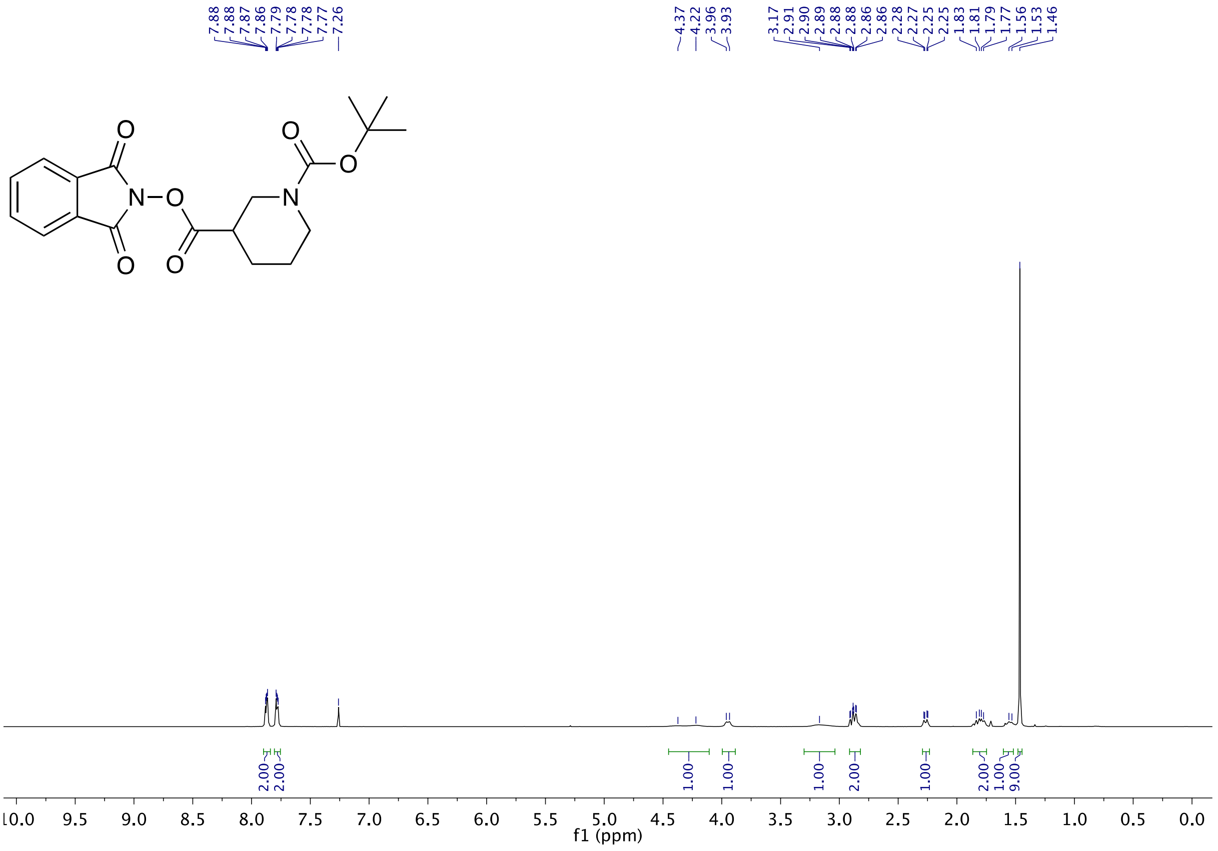

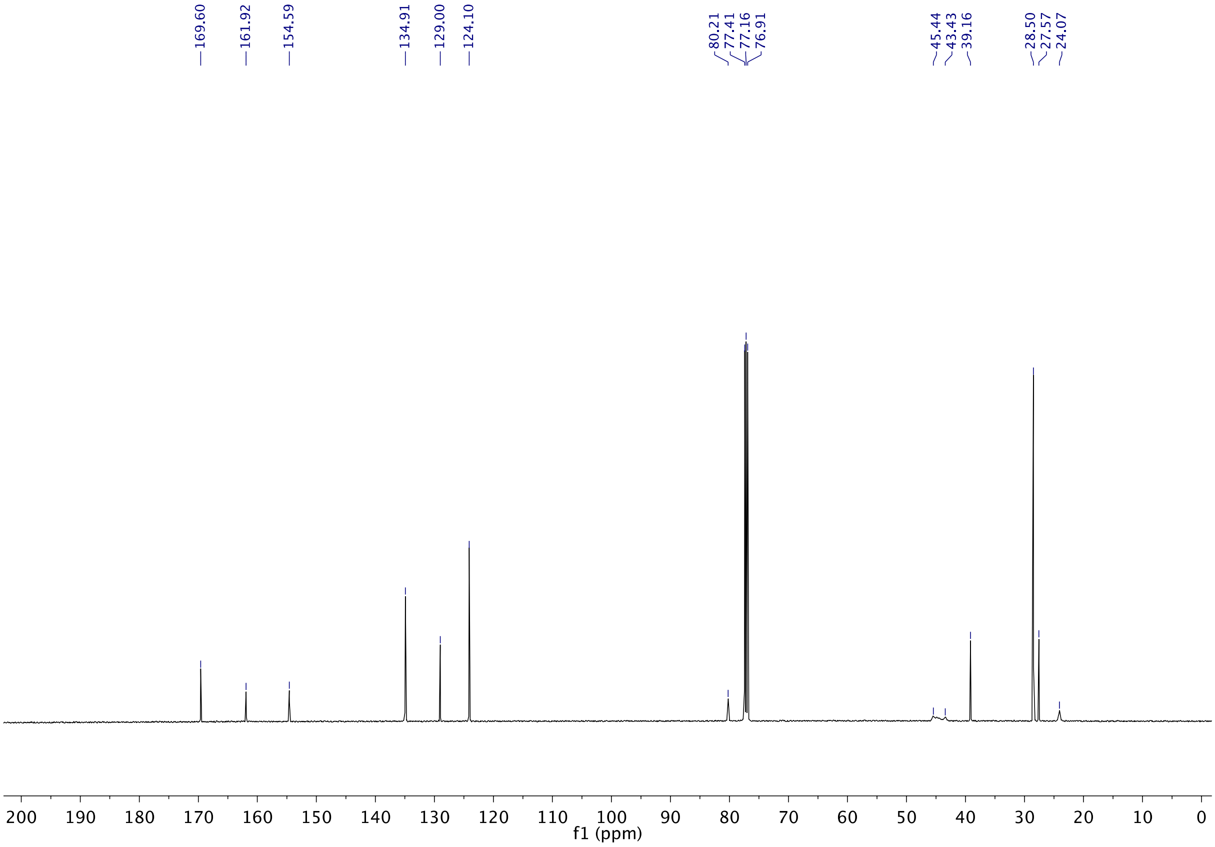

1-(*tert*-Butoxycarbonyl)piperidine-2-carboxylic acid, **N25-int1**

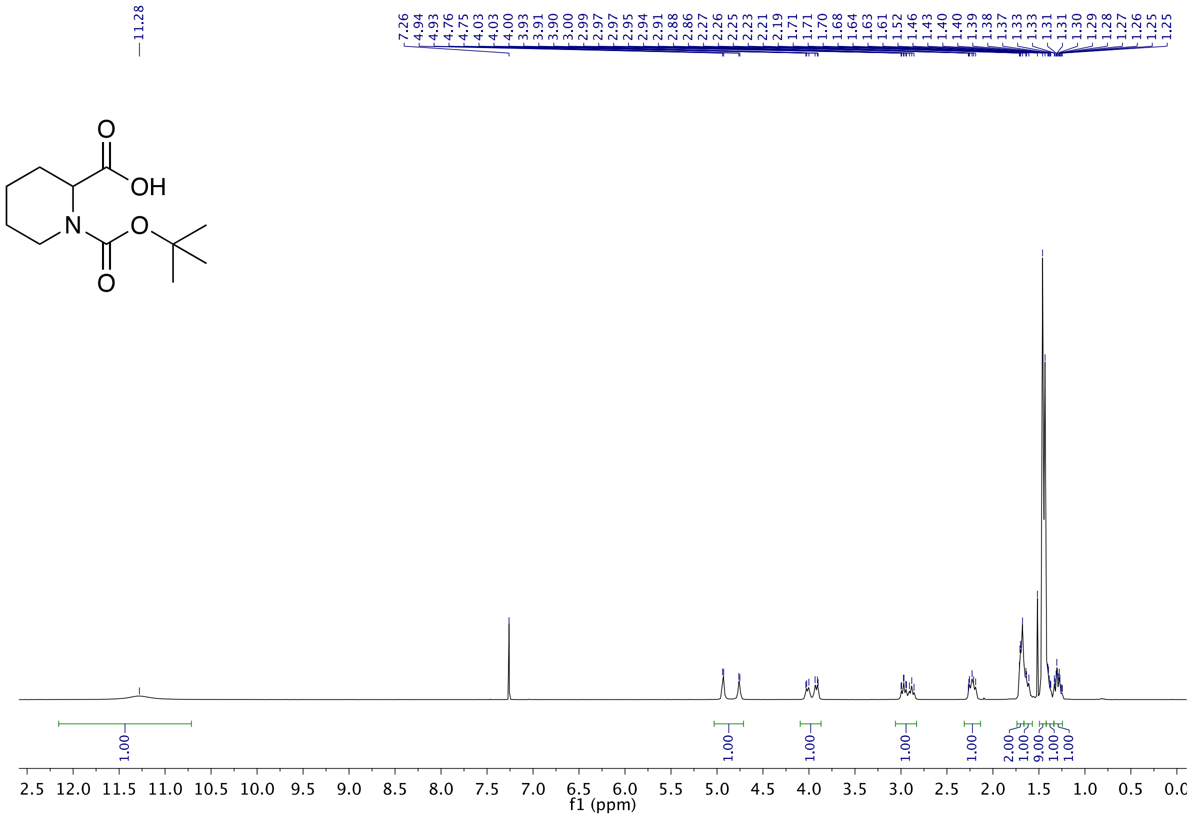

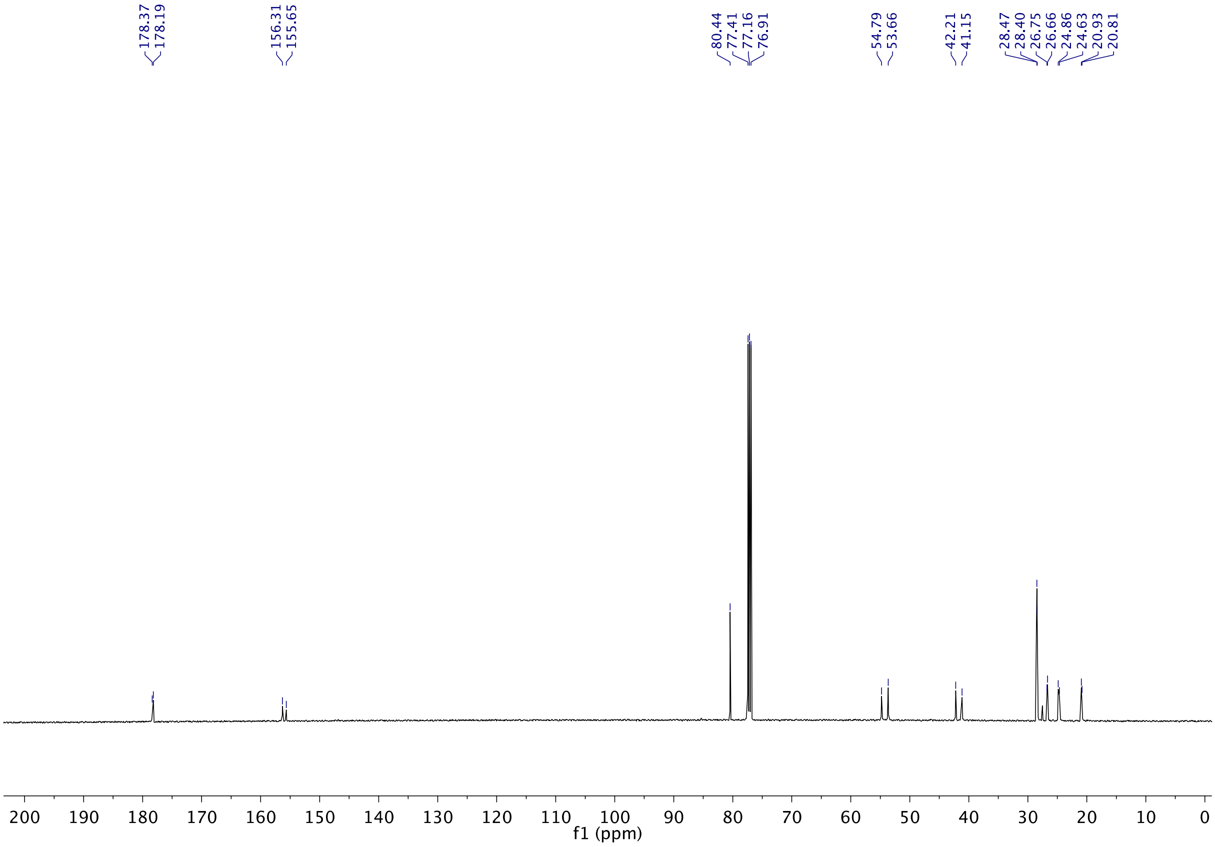

1-(*tert*-Butyl) 2-(1,3-dioxoisoindolin-2-yl) piperidine-1,2-dicarboxylate, **N25**

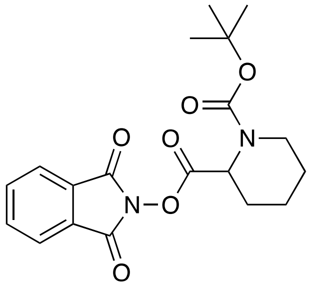

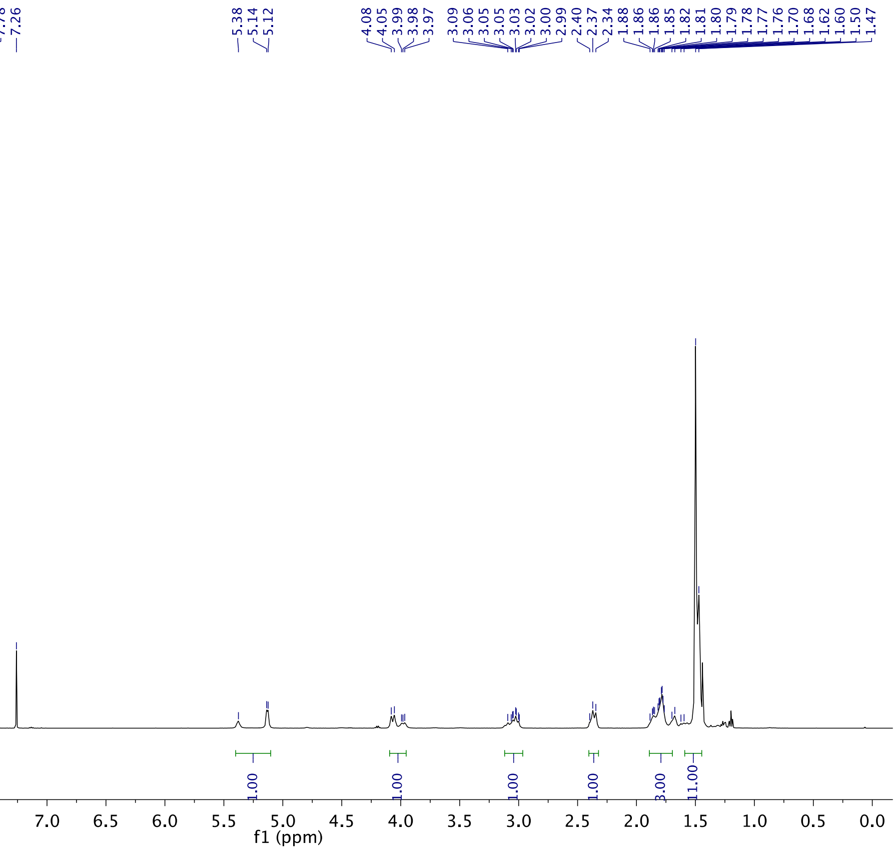

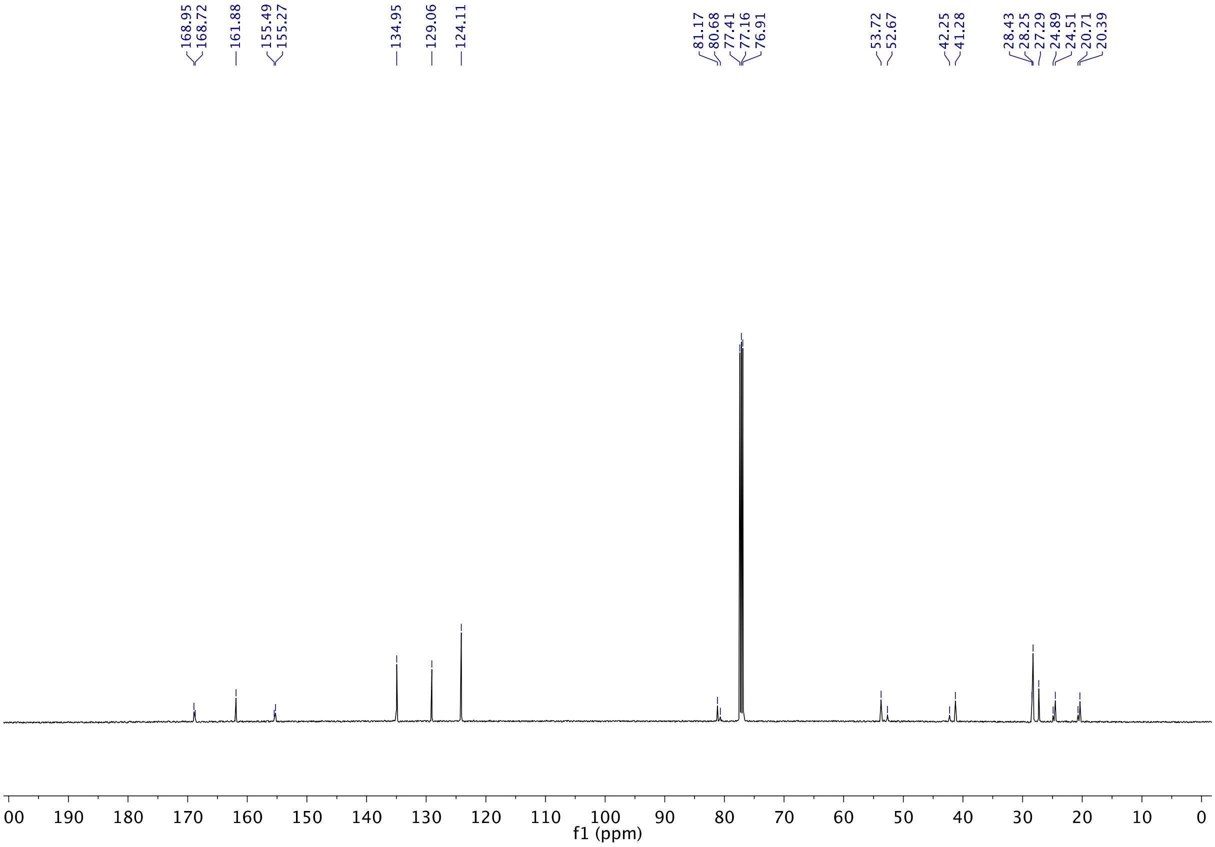

1-(*tert*-Butyl) 2-(1,3-dioxoisoindolin-2-yl) (*S*)-pyrrolidine-1,2-dicarboxylate, **N26**

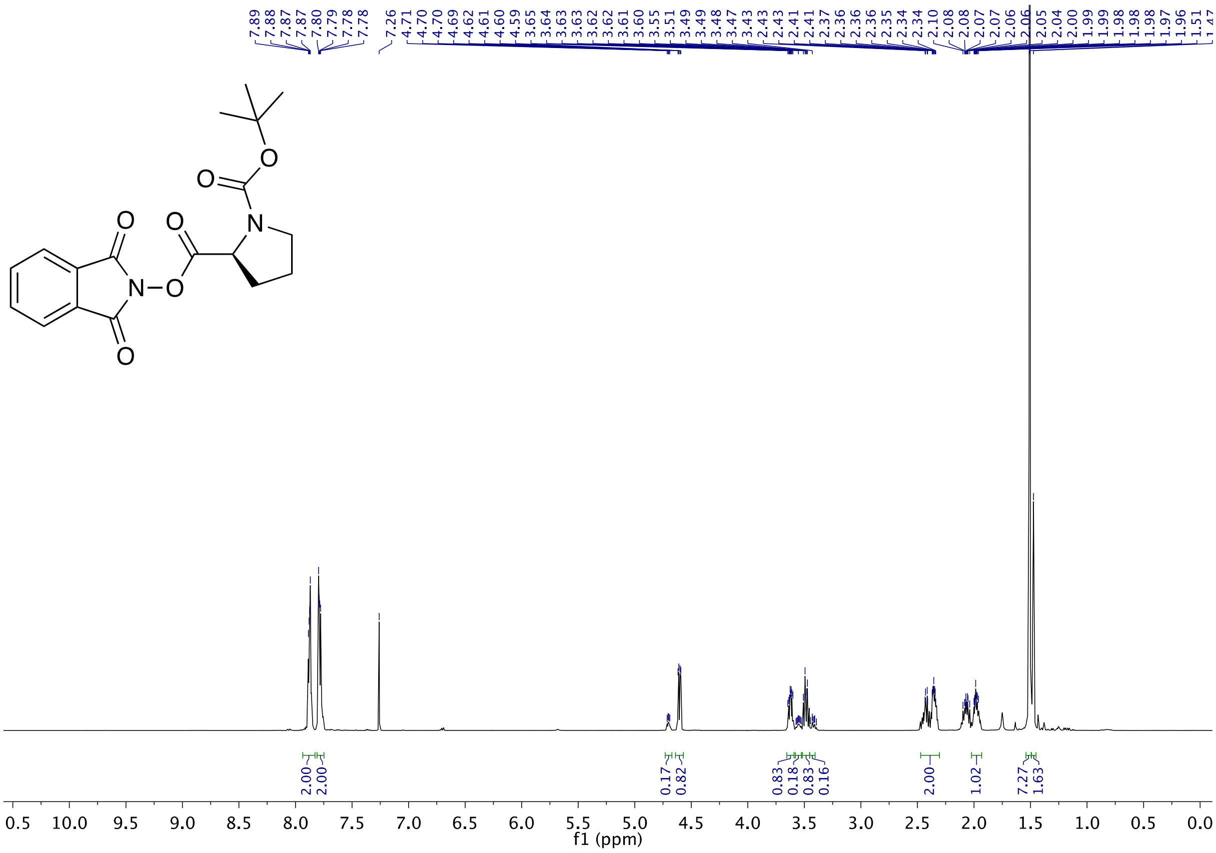

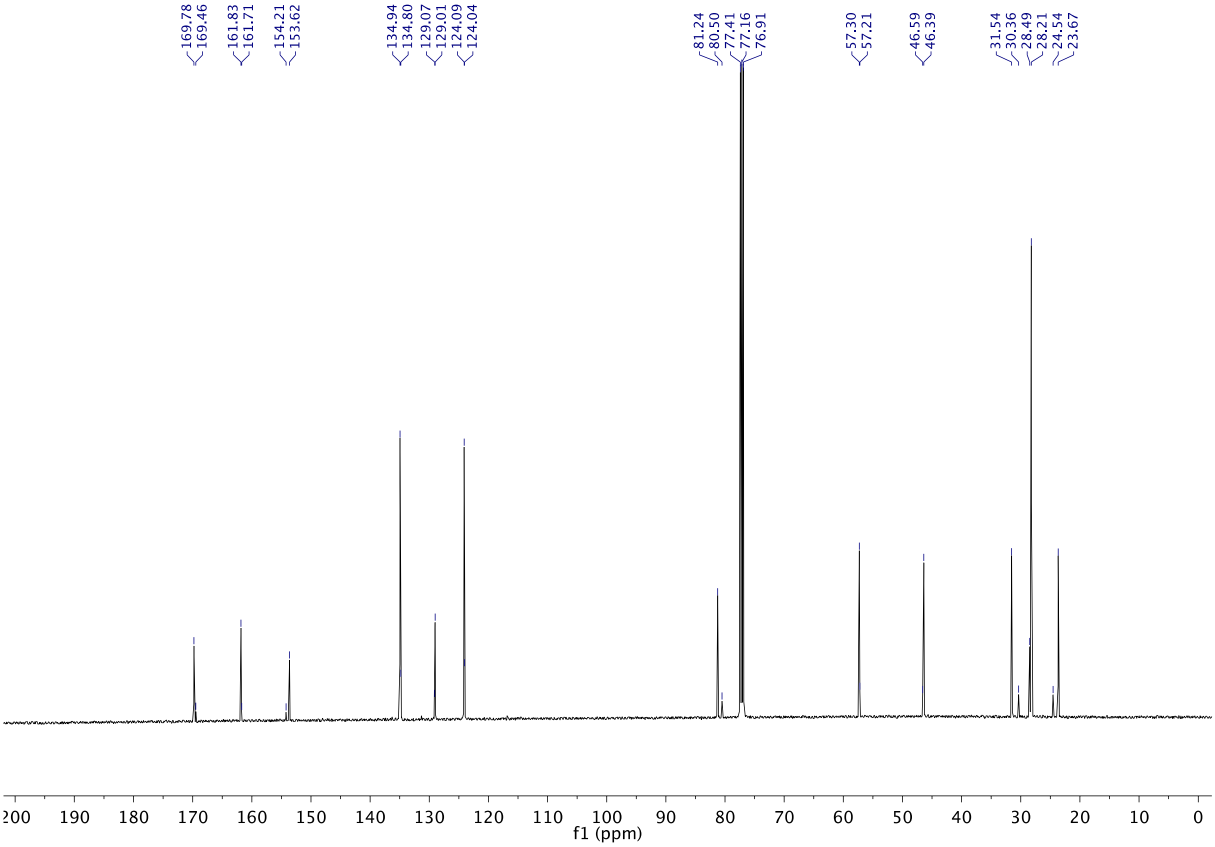

1,3-Dioxoisoindolin-2-yl 3-(furan-2-yl)propanoate, **N27**

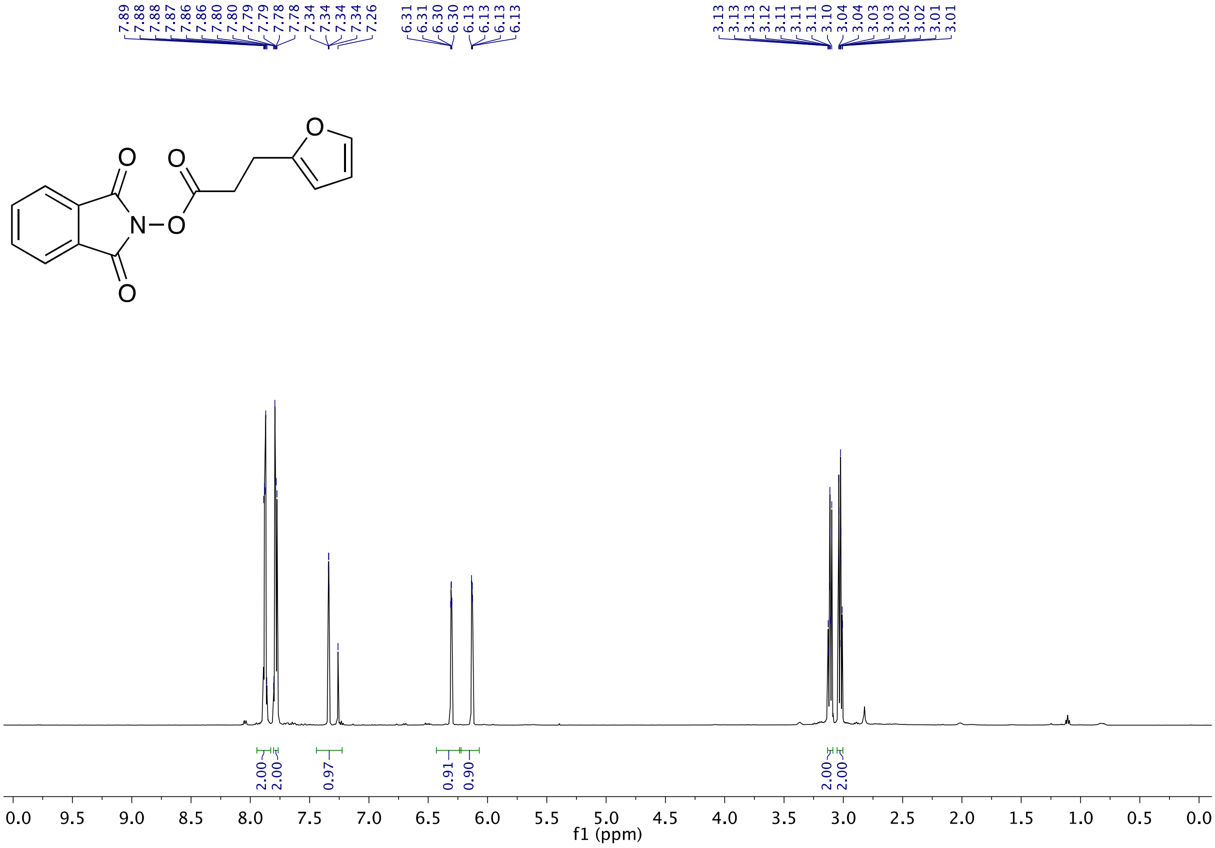

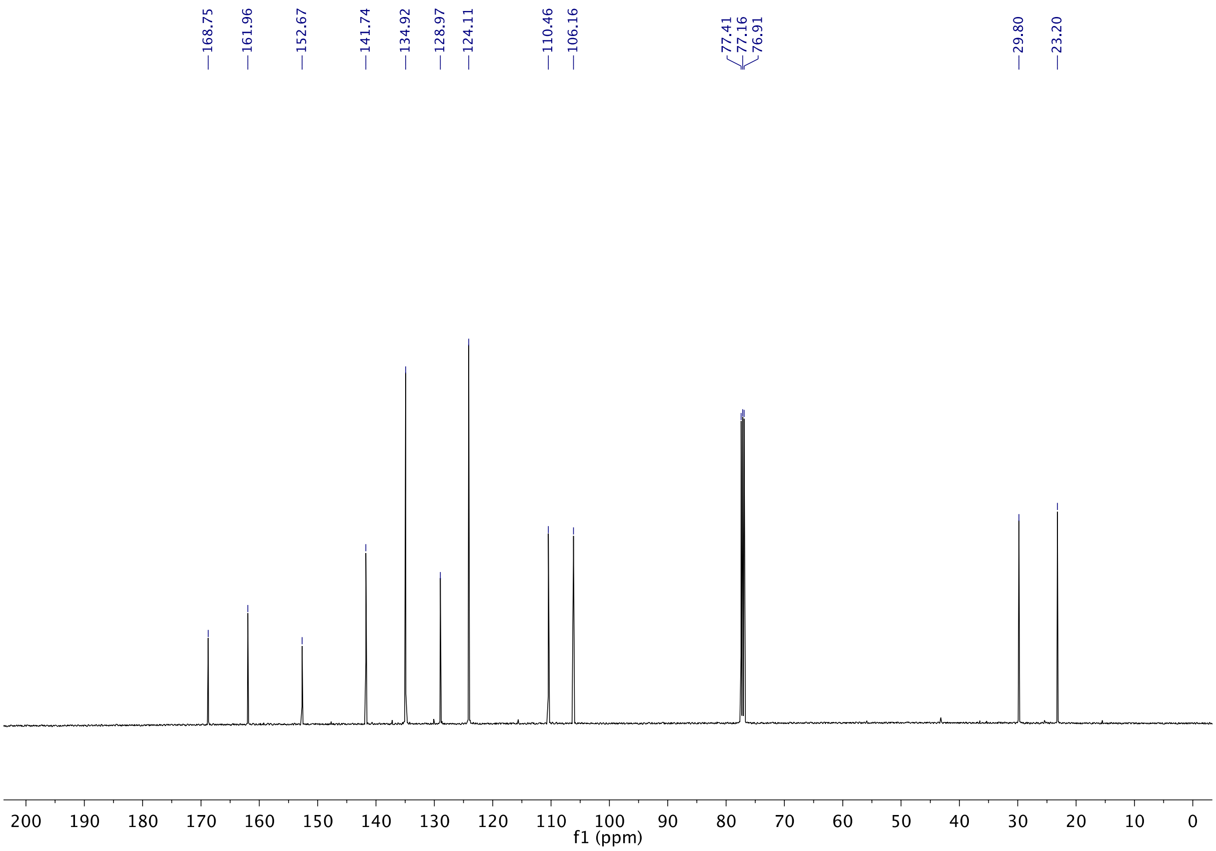

1,3-Dioxoisoindolin-2-yl 3-(1*H*-indol-3-yl)propanoate, **N28**

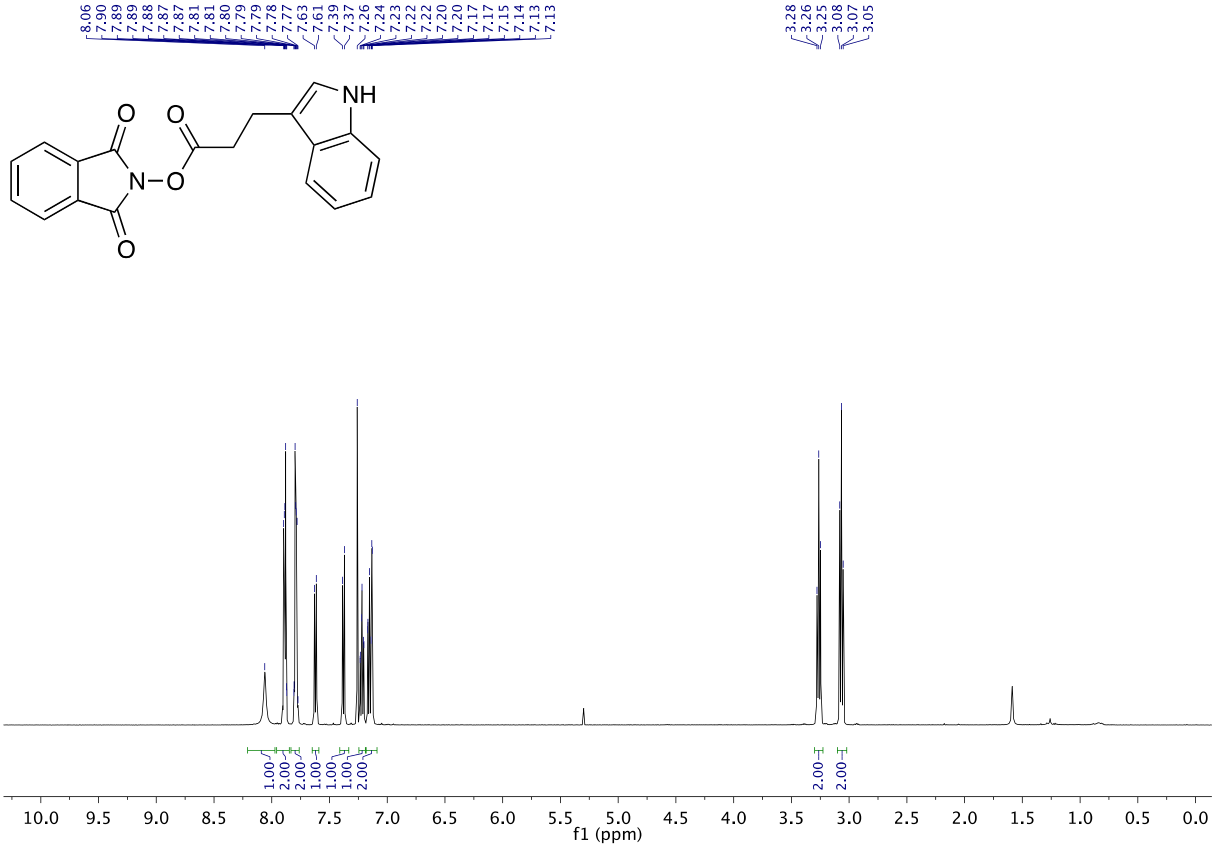

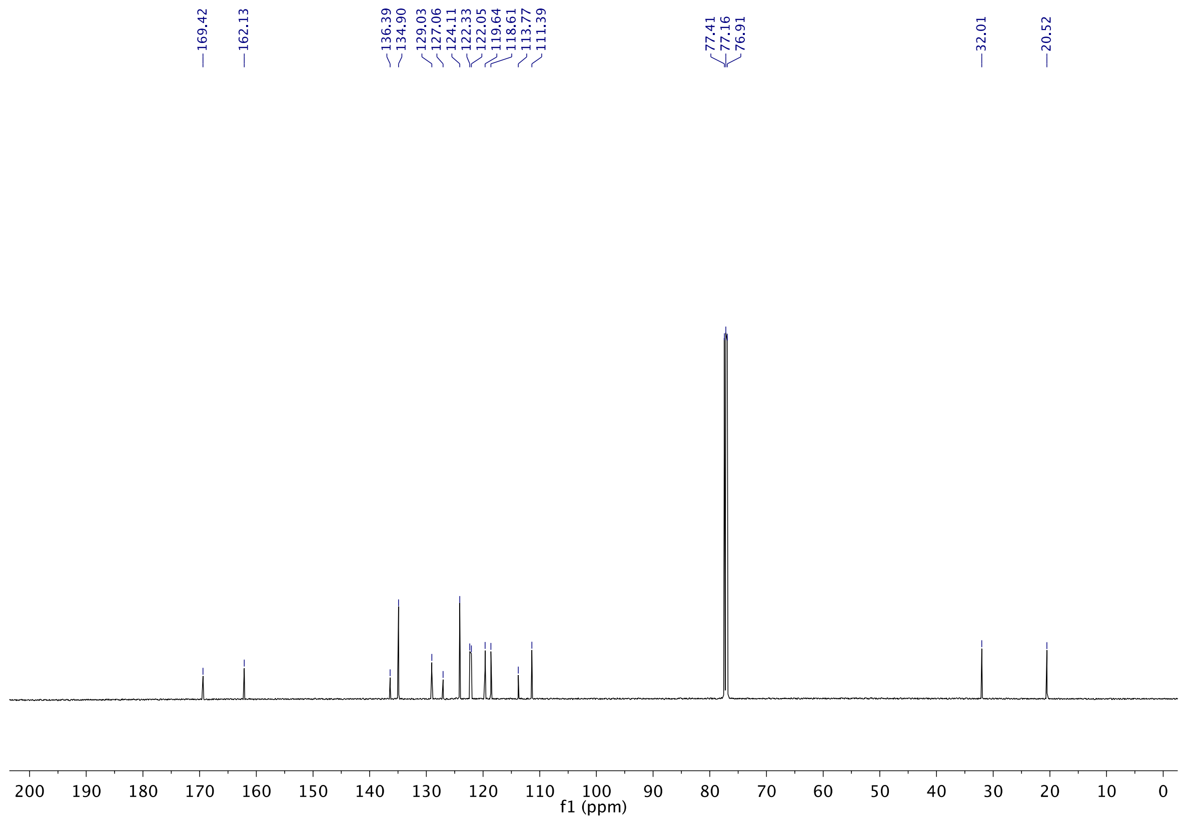

tert-Butyl 3-(3-((1,3-dioxoisoindolin-2-yl)oxy)-3-oxopropyl)-1*H*-indole-1-carboxylate, **N29**

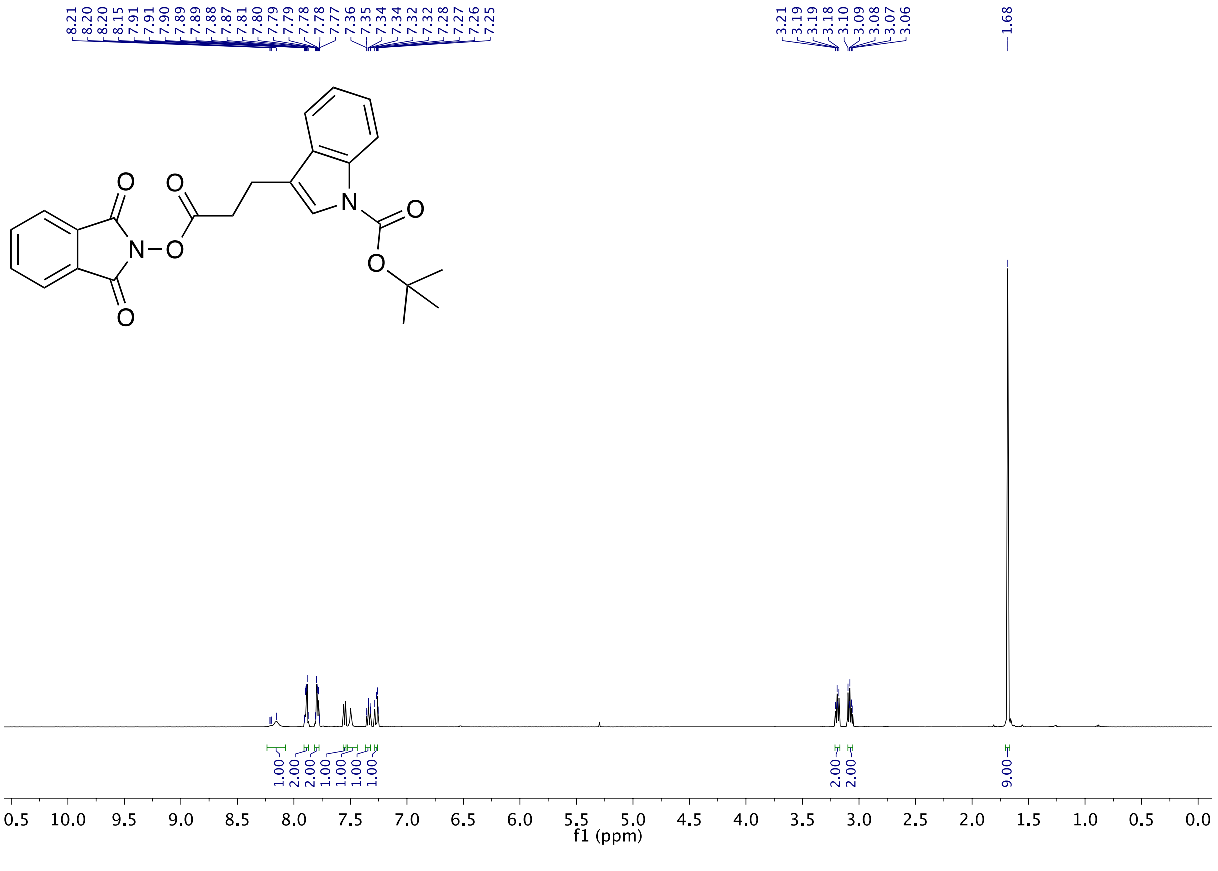

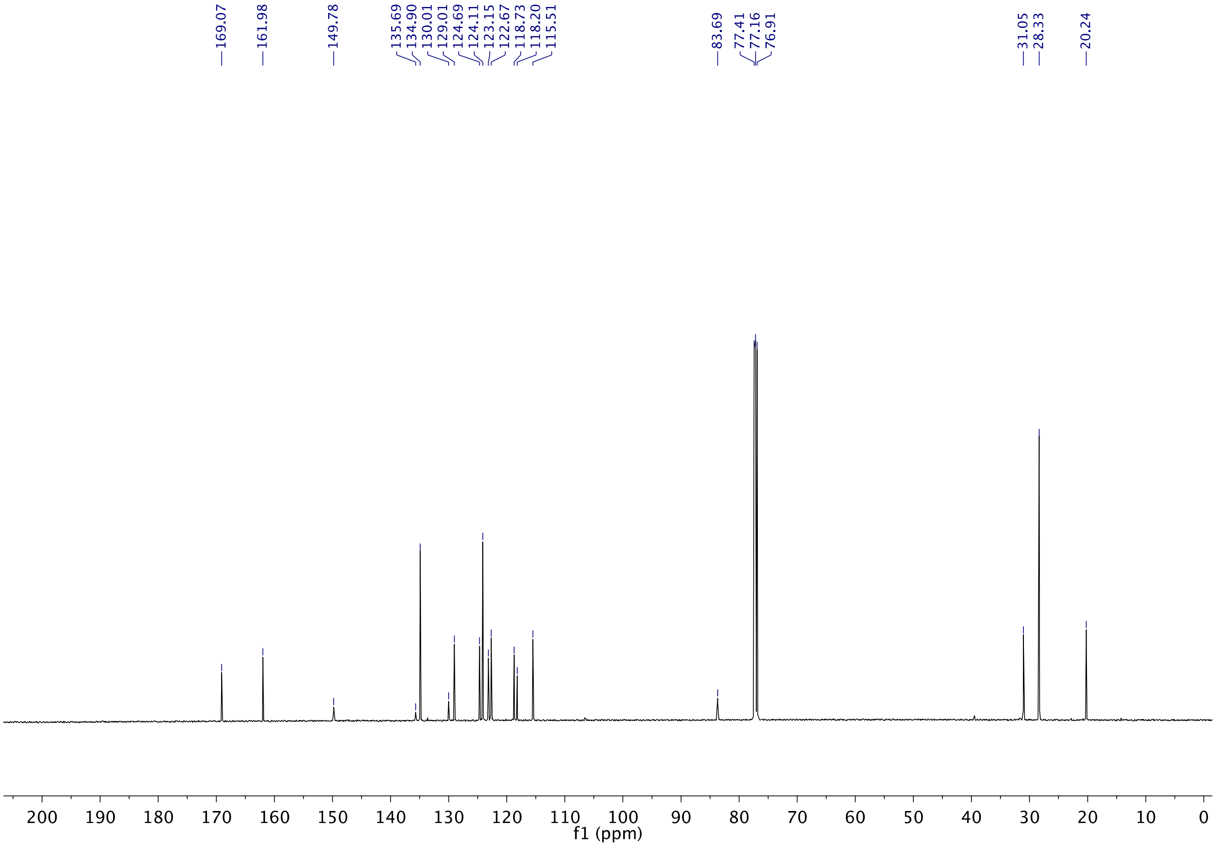

1,3-Dioxoisoindolin-2-yl 3-(pyridin-3-yl)propanoate, **N30**

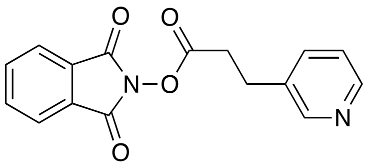

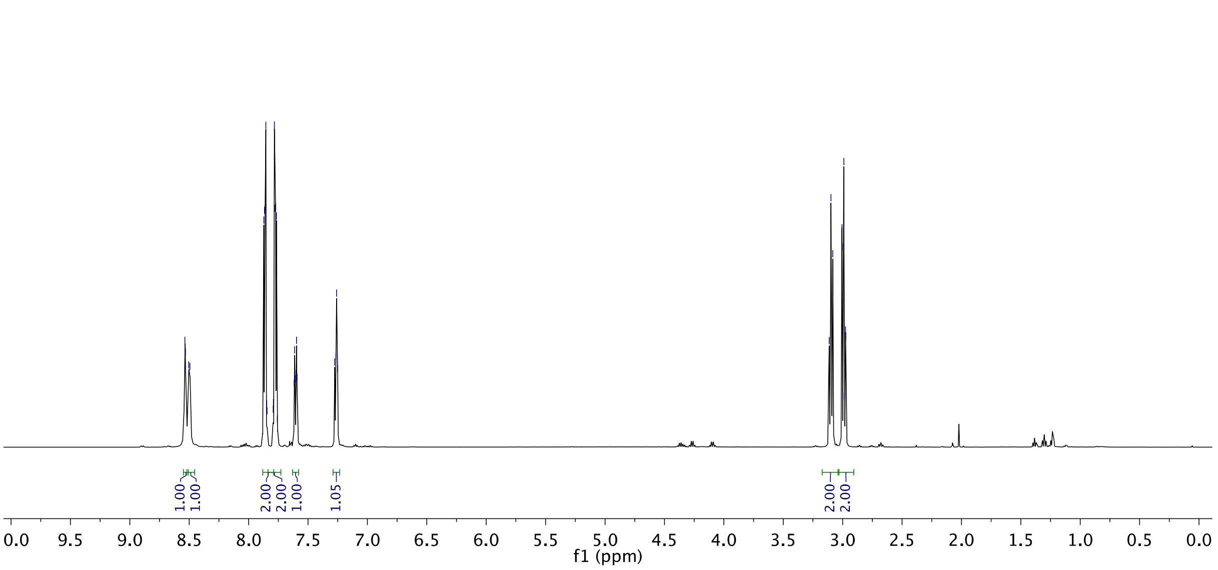

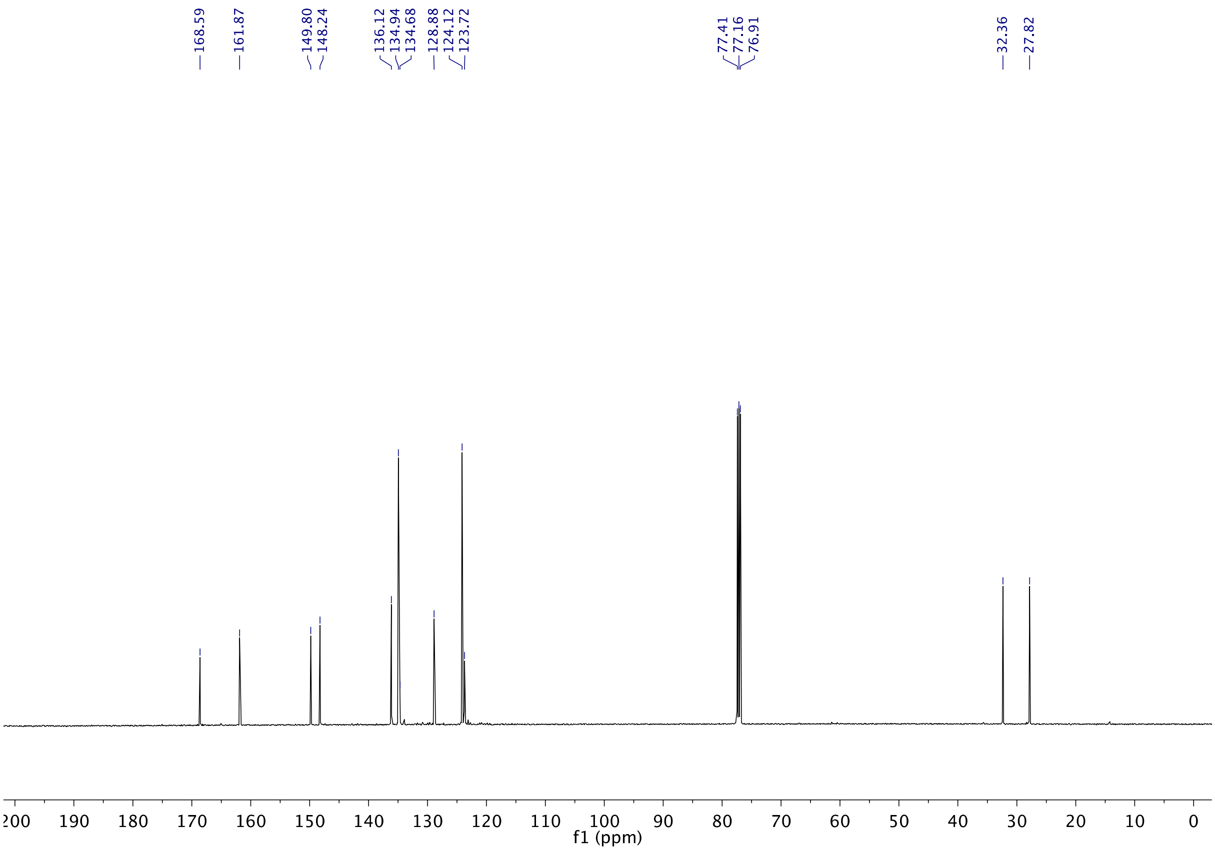

1-(1,3-Dioxoisoindolin-2-yl) 3-methyl bicyclo[1.1.1]pentane-1,3-dicarboxylate, **N31**

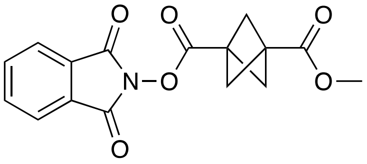

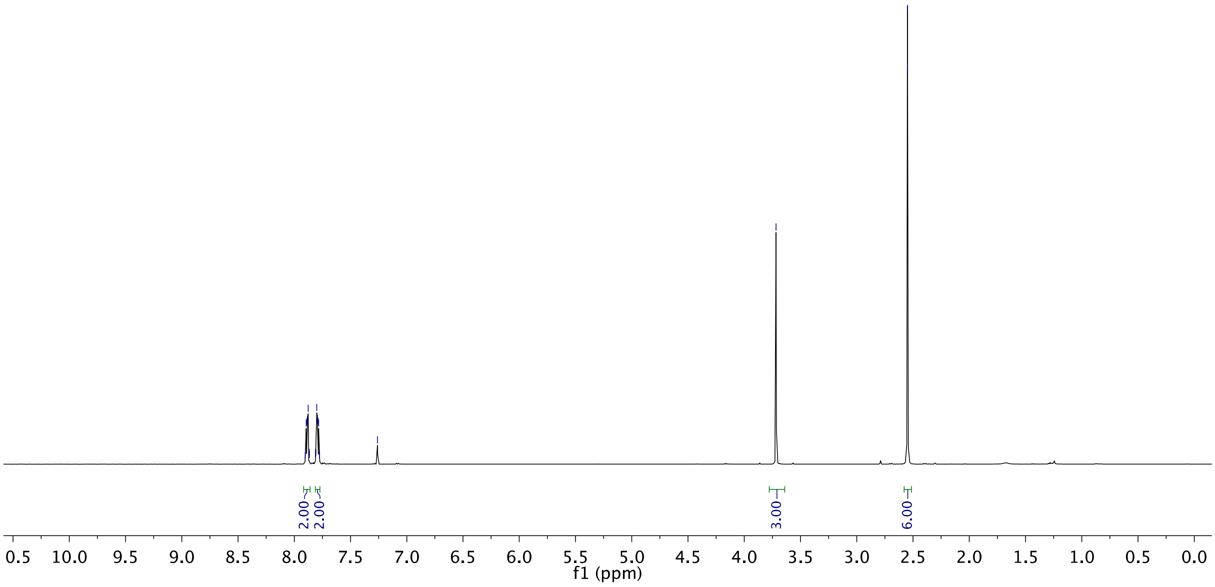

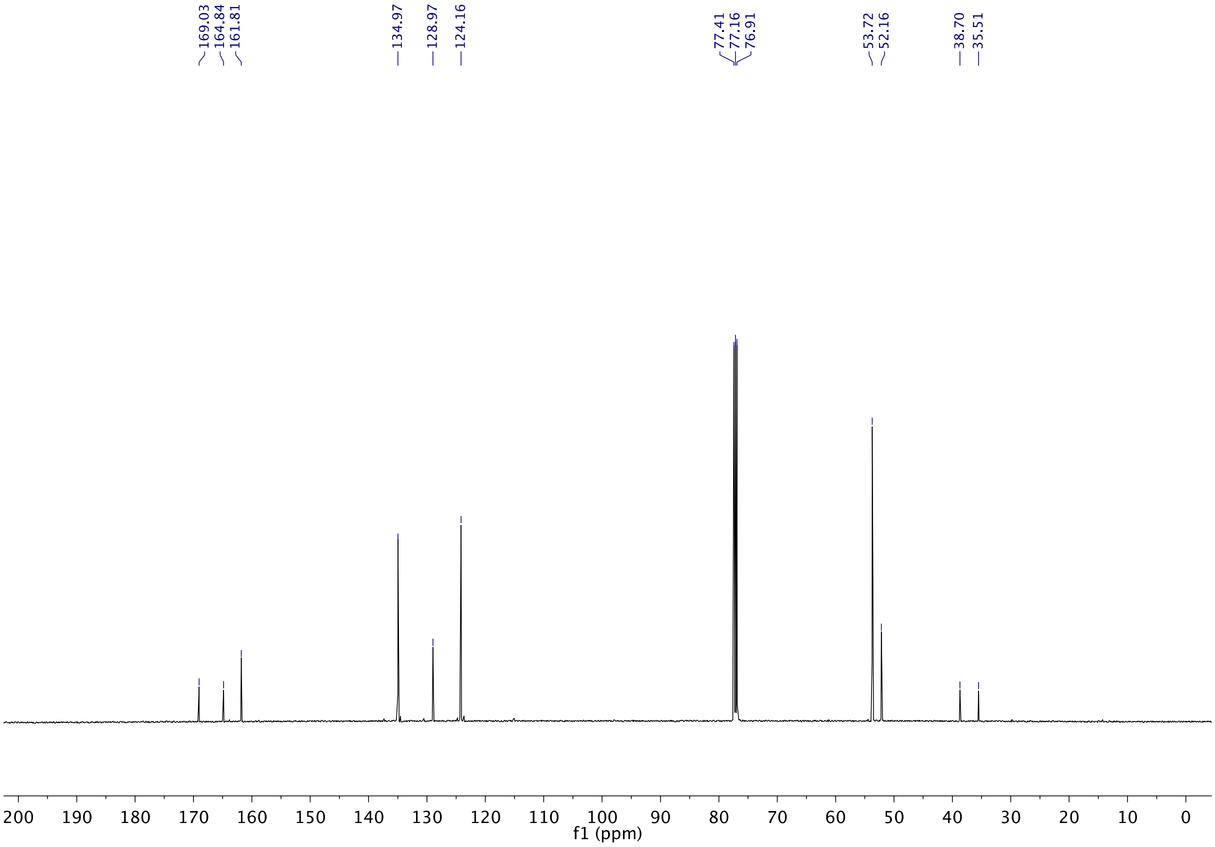

3-(4-(4,4,5,5-Tetramethyl-1,3,2-dioxaborolan-2-yl)phenyl)propanoic acid, **N32-int1**

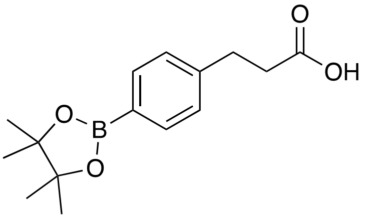

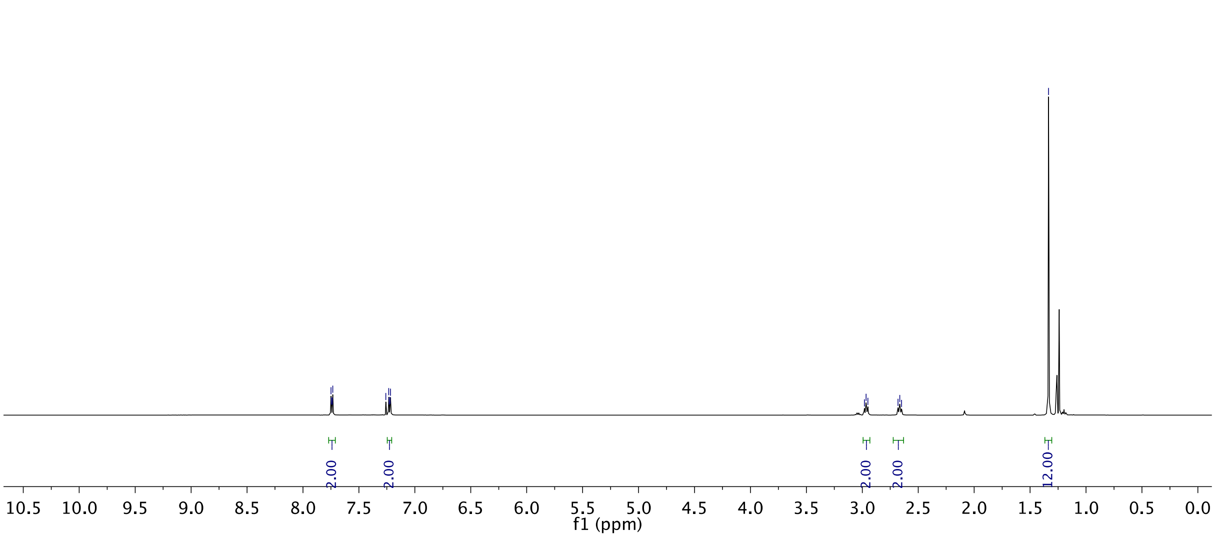

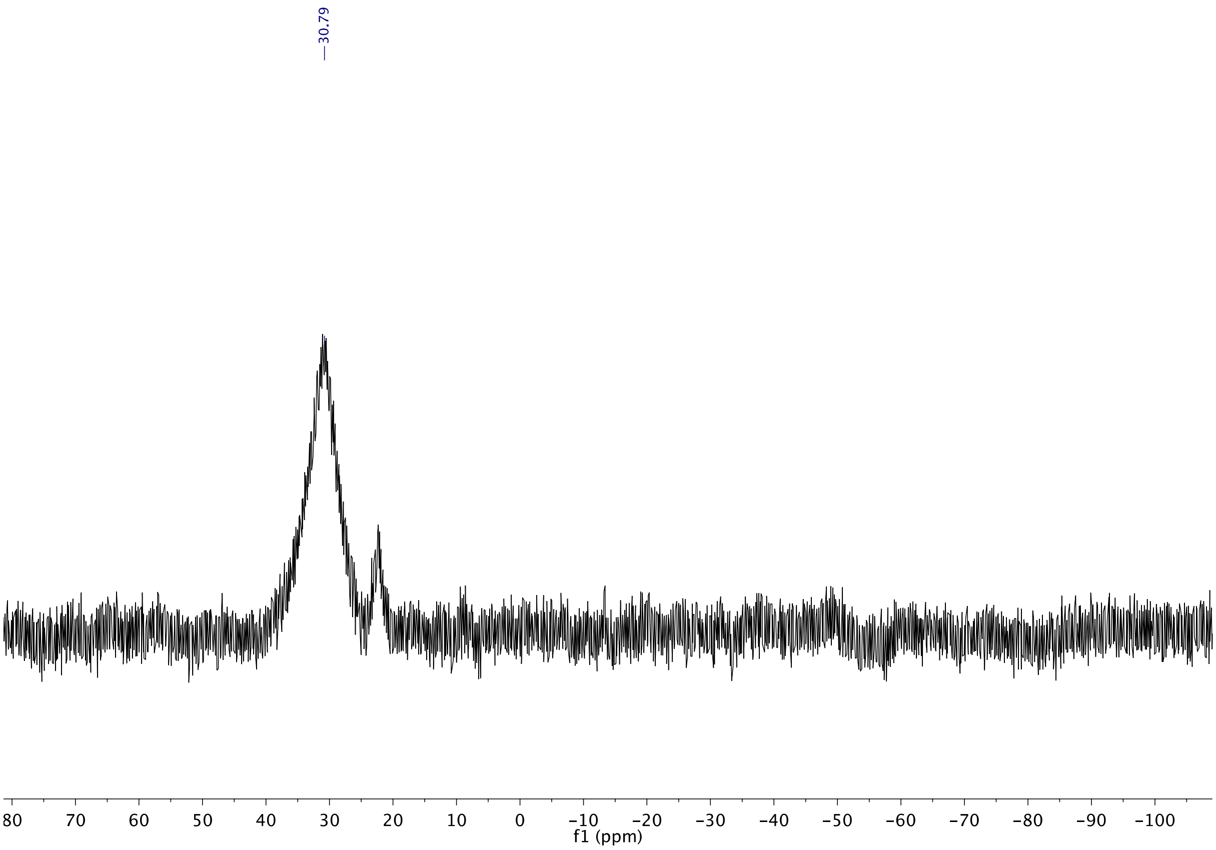
30.79
80 70 60 50 40 30 20 10 0 −10 −20 −30 −40 −50 −60 −70 −80 −90 −100
f1 (ppm)

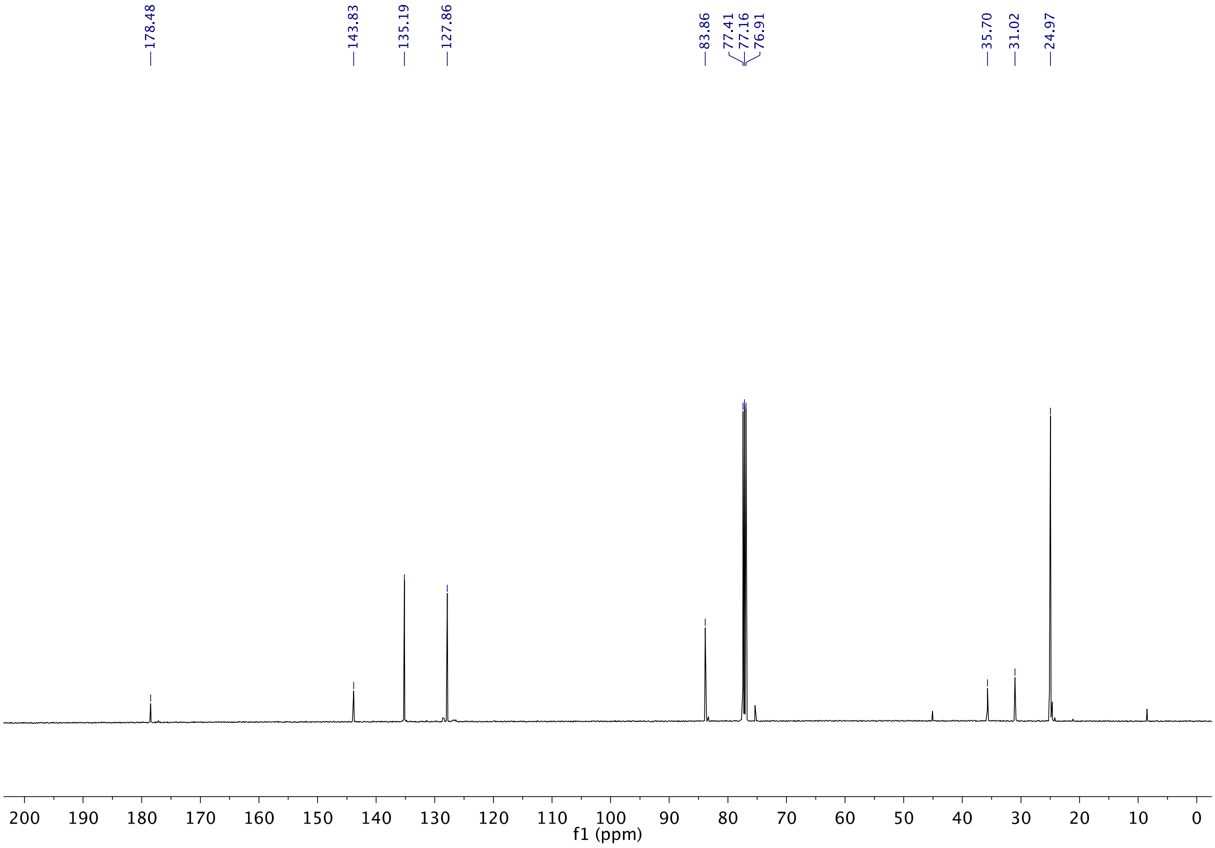
178.48
143.83
135.19
127.86
83.86
77.41
77.16
76.91
35.70
31.02
24.97
200 190 180 170 160 150 140 130 120 110 100 90 80 70 60 50 40 30 20 10 0
f1 (ppm)

1,3-Dioxoisoindolin-2-yl 3-(4-(4,4,5,5-tetramethyl-1,3,2-dioxaborolan-2-yl)phenyl)propanoate, **N32**

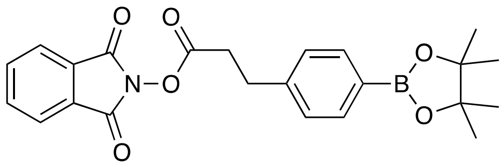

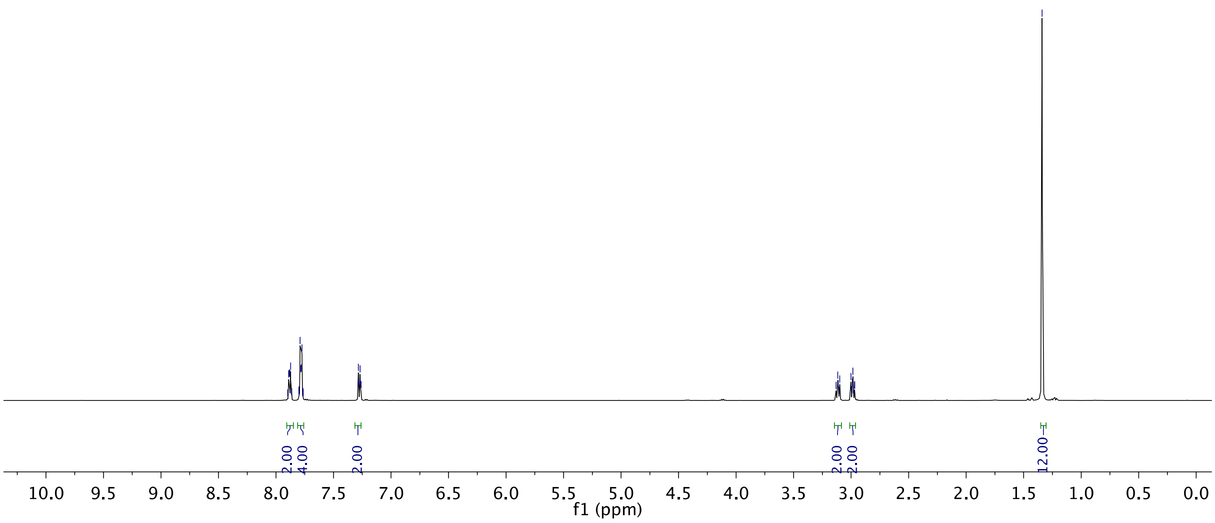

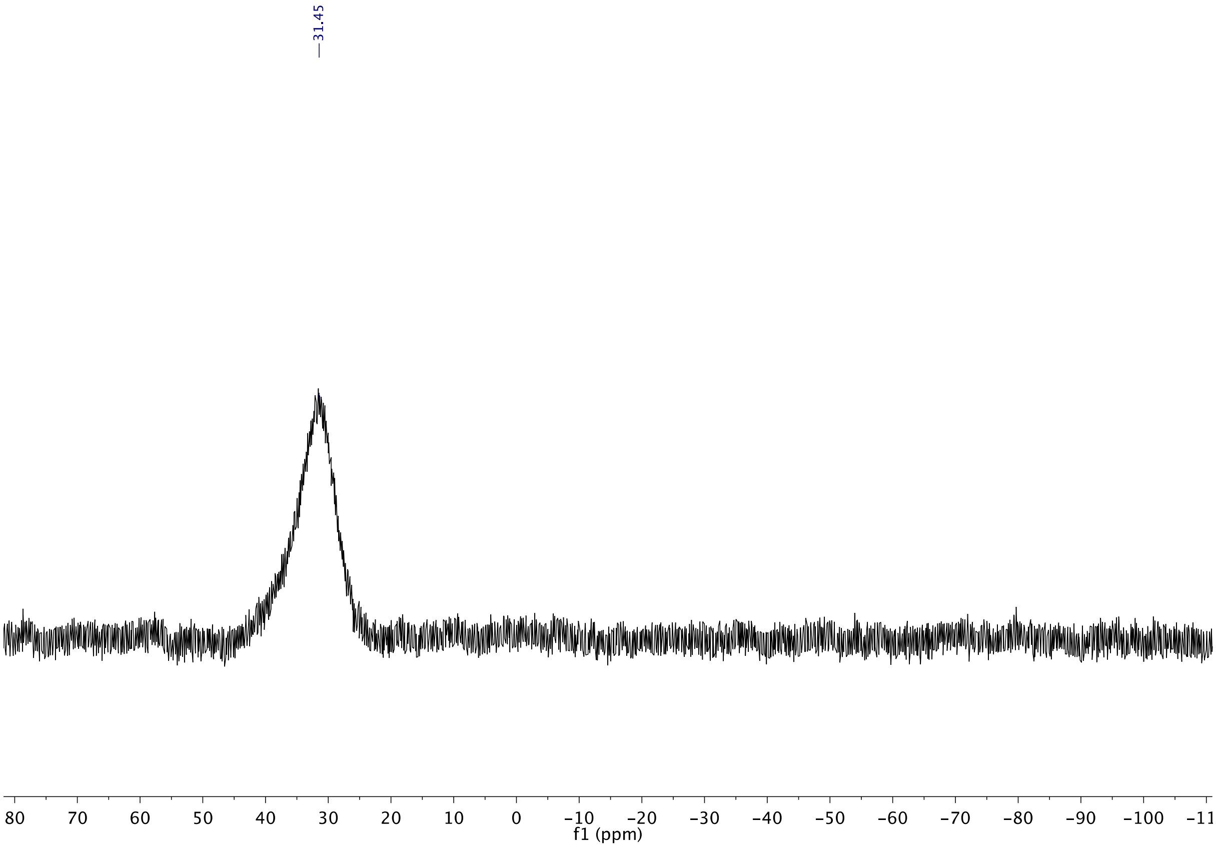

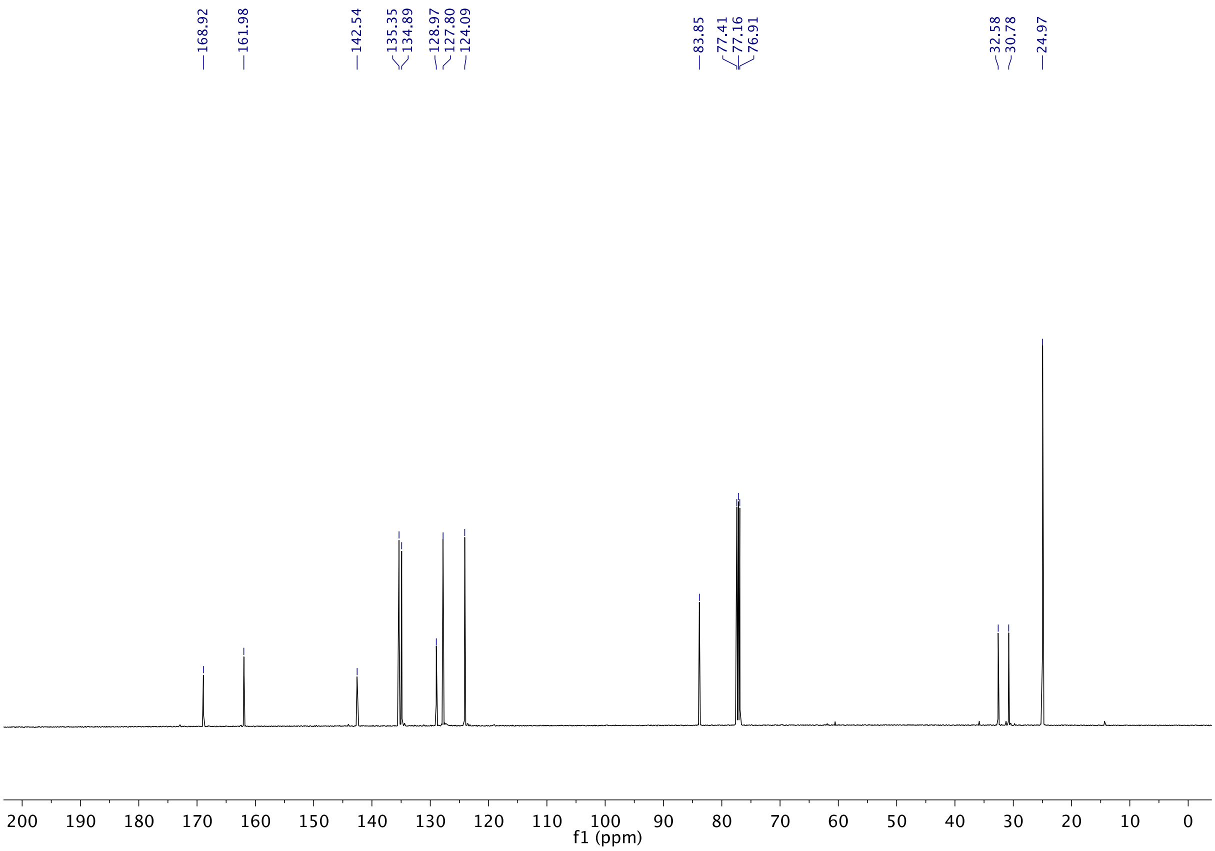

1,3-Dioxoisoindolin-2-yl hept-6-enoate, **61**

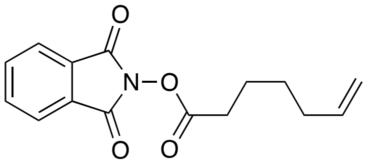

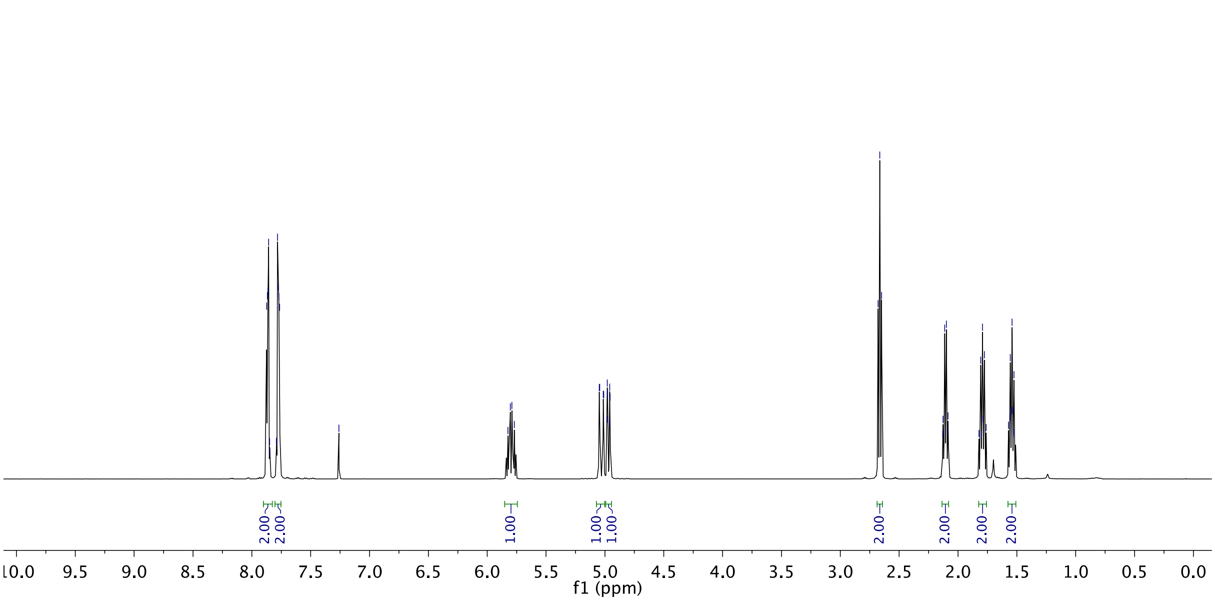

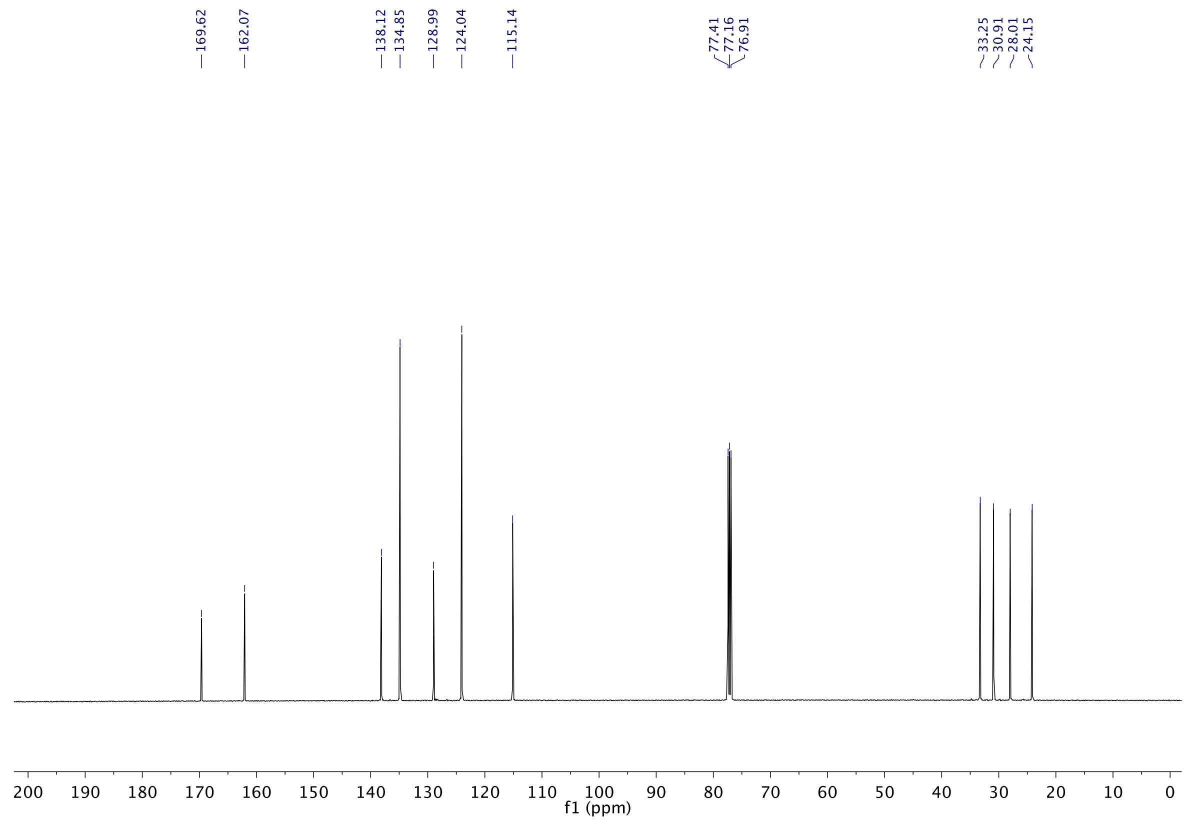

1,3-Dioxoisoindolin-2-yl 2-cyclopropylacetate, **64**

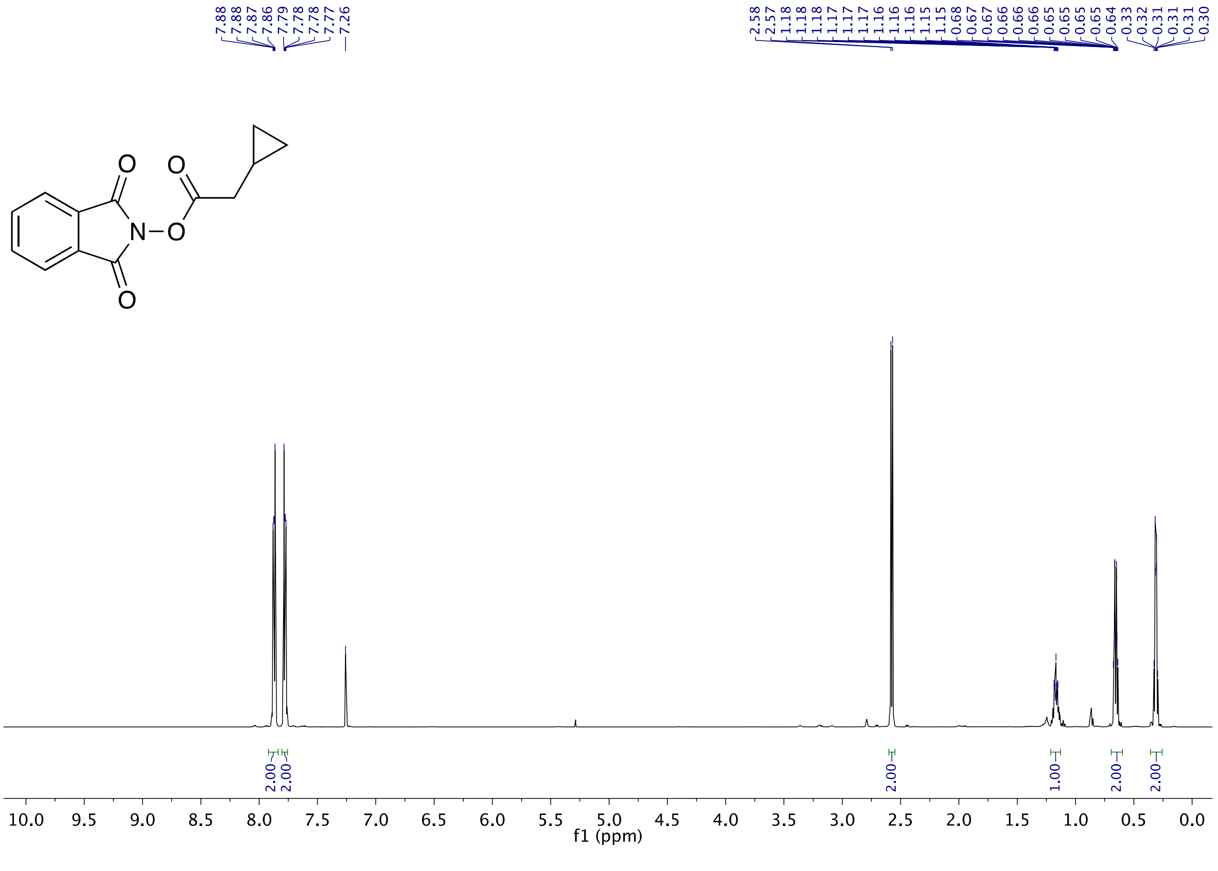

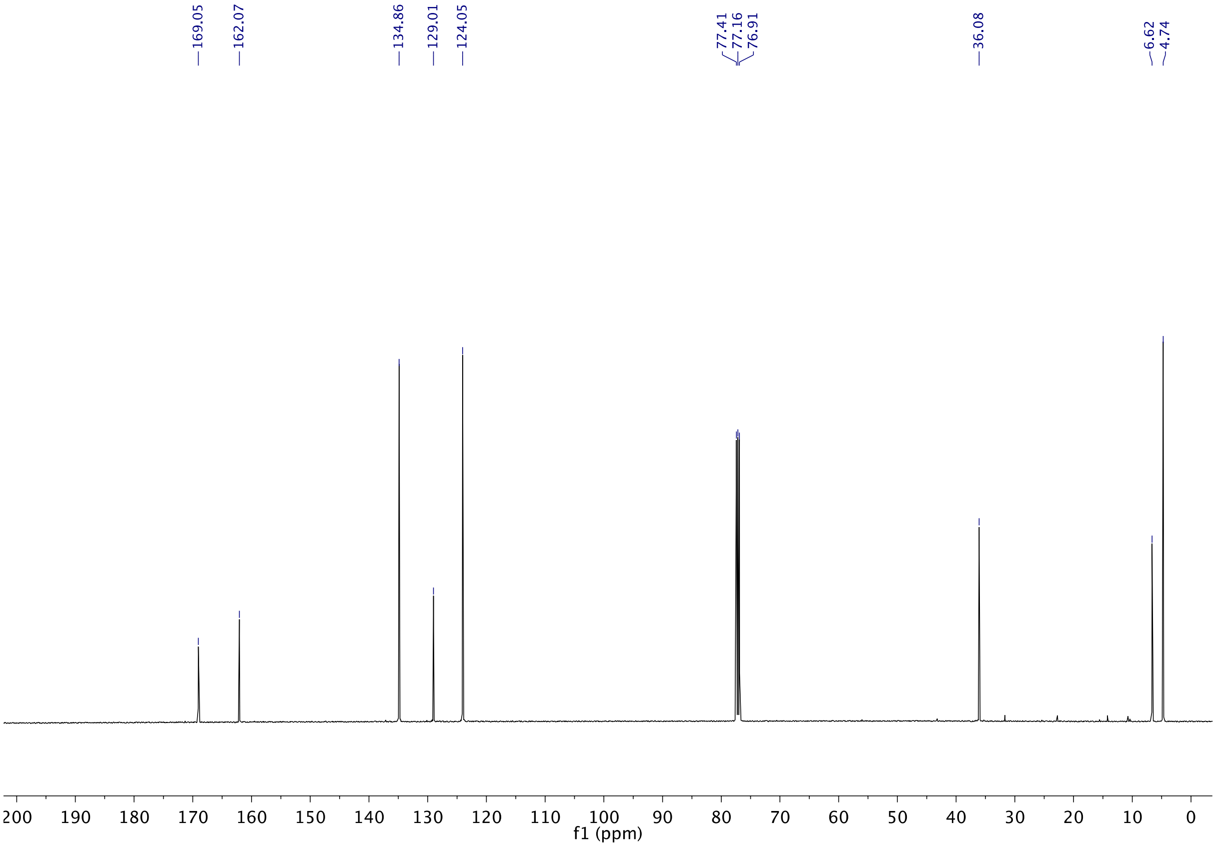

1-(*tert*-Butoxycarbonyl)indoline-2-carboxylic acid, **N33-int1**

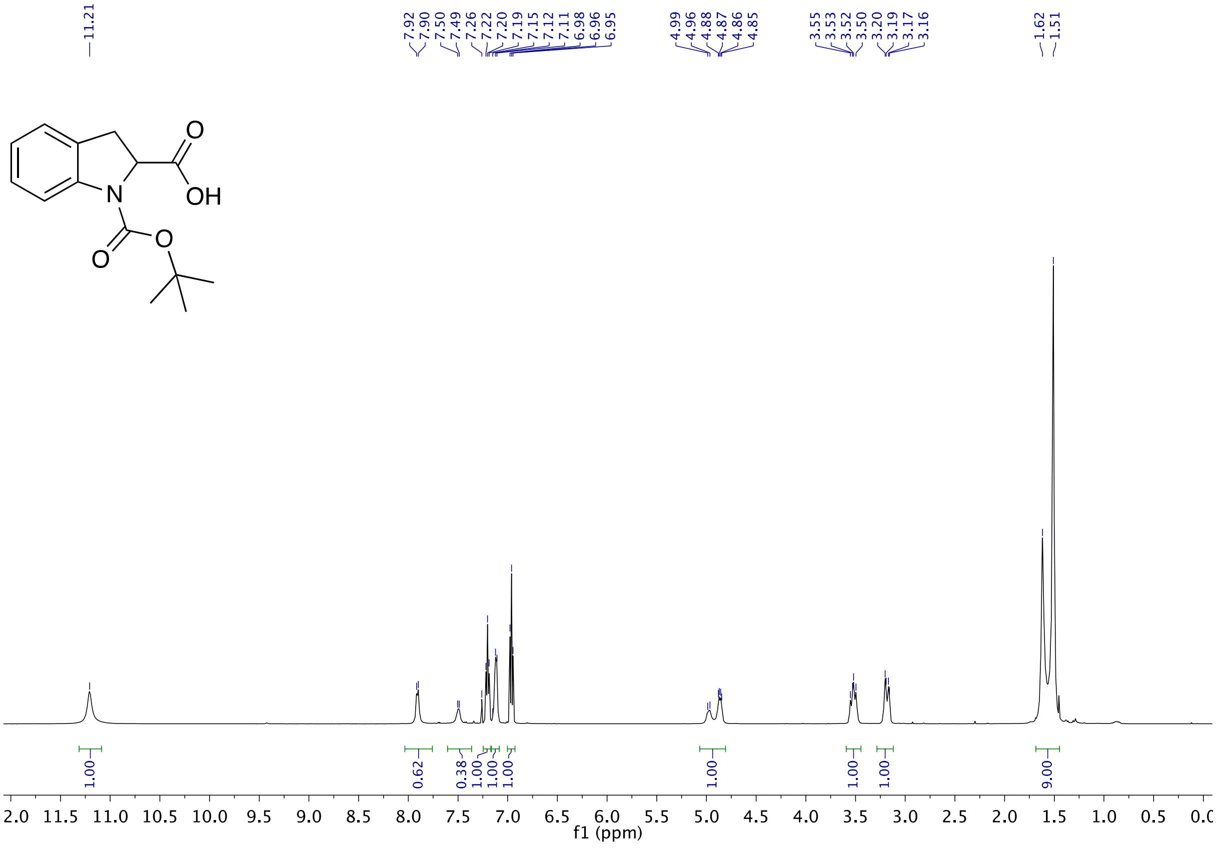

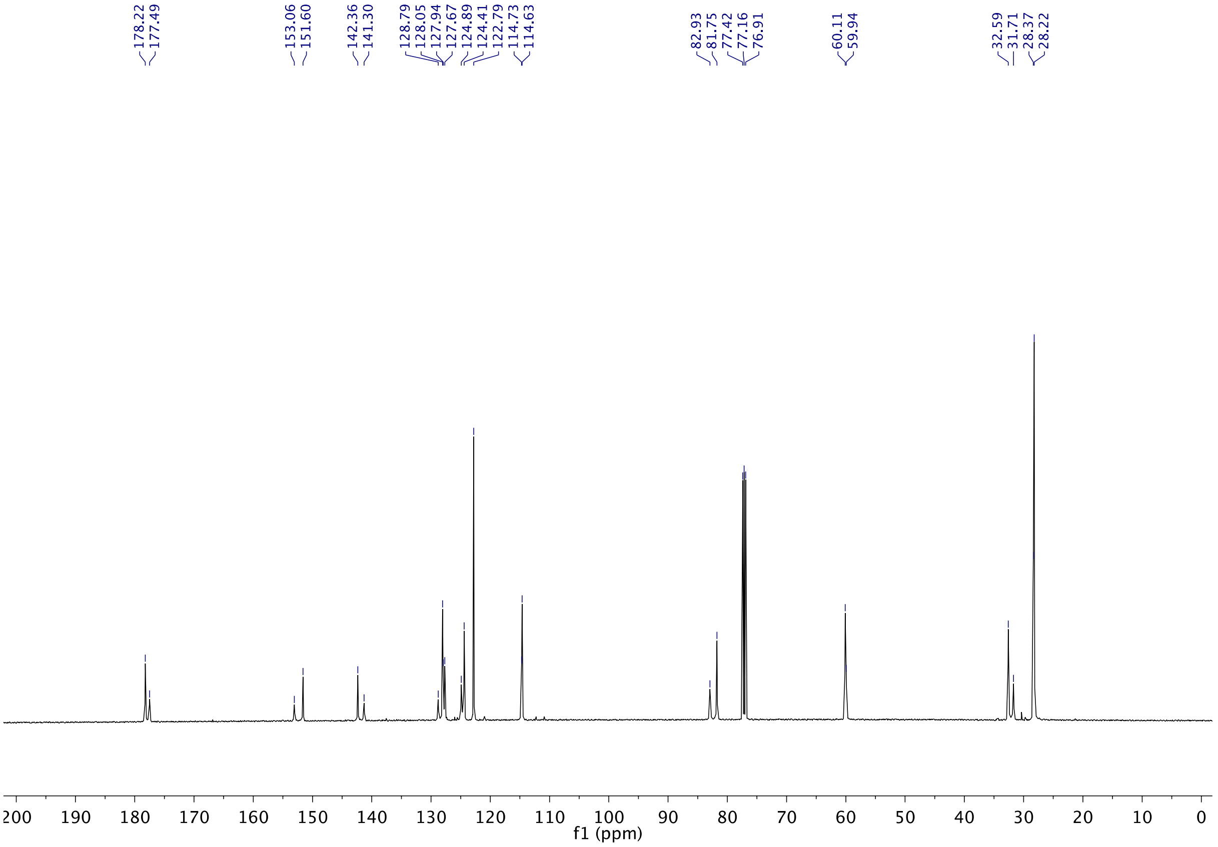

1-(*tert*-Butyl) 2-(1,3-dioxoisoindolin-2-yl) indoline-1,2-dicarboxylate, **N33**

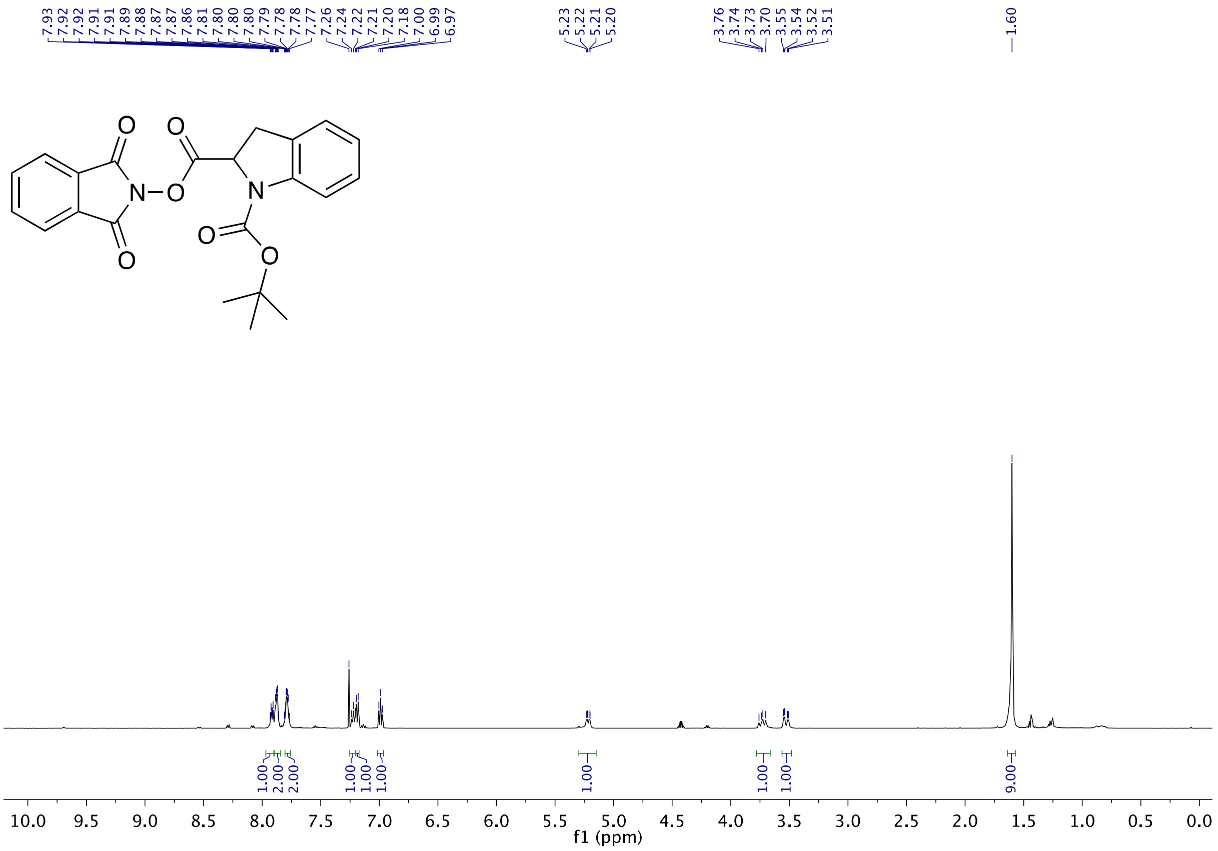

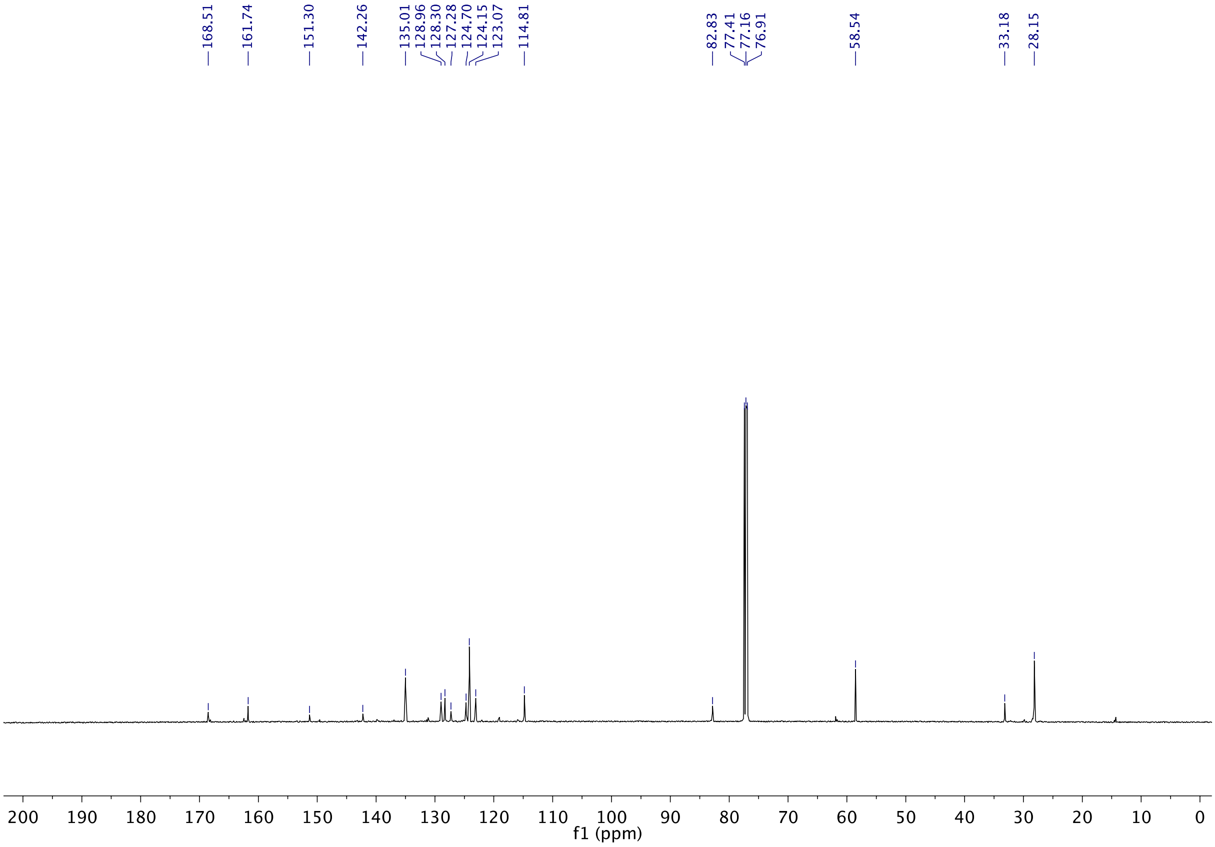

2-((*tert*-Butoxycarbonyl)amino)-2-methylpropanoic acid, **N34-int1**

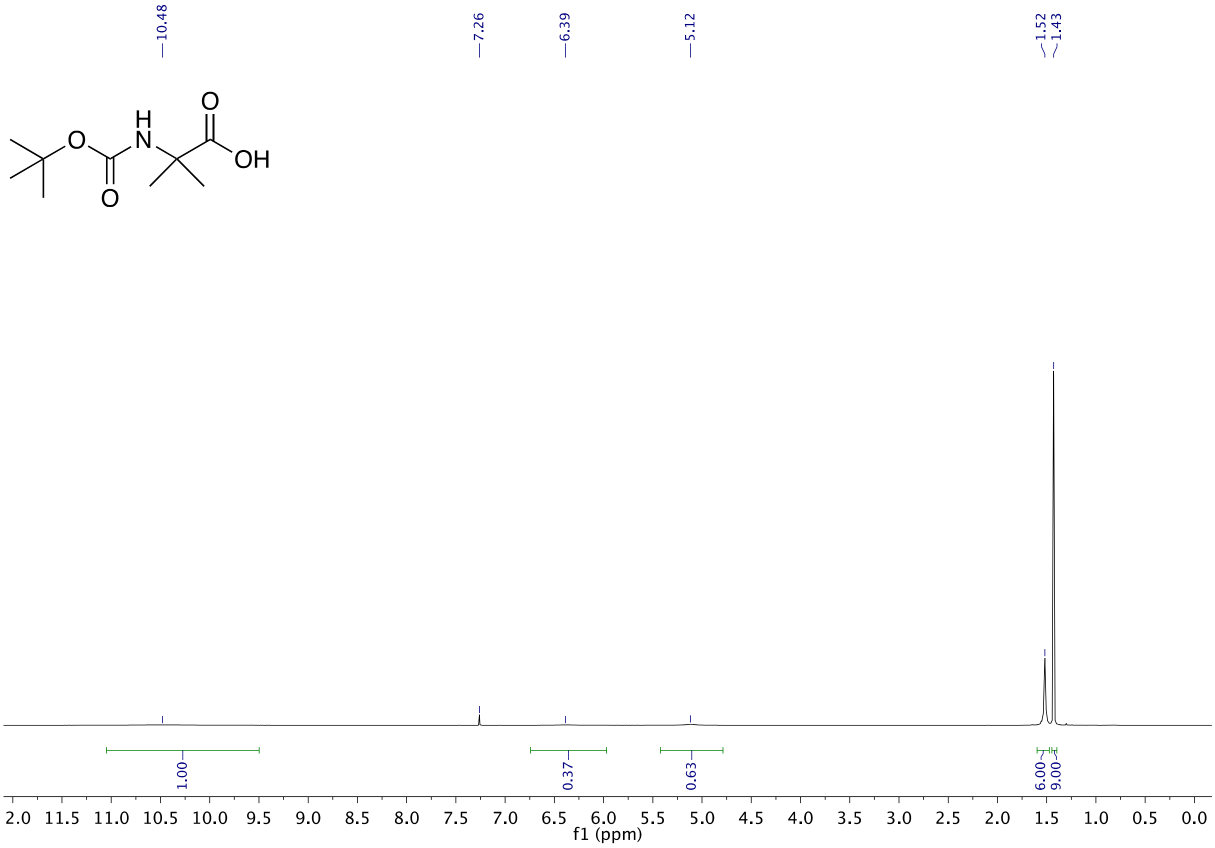

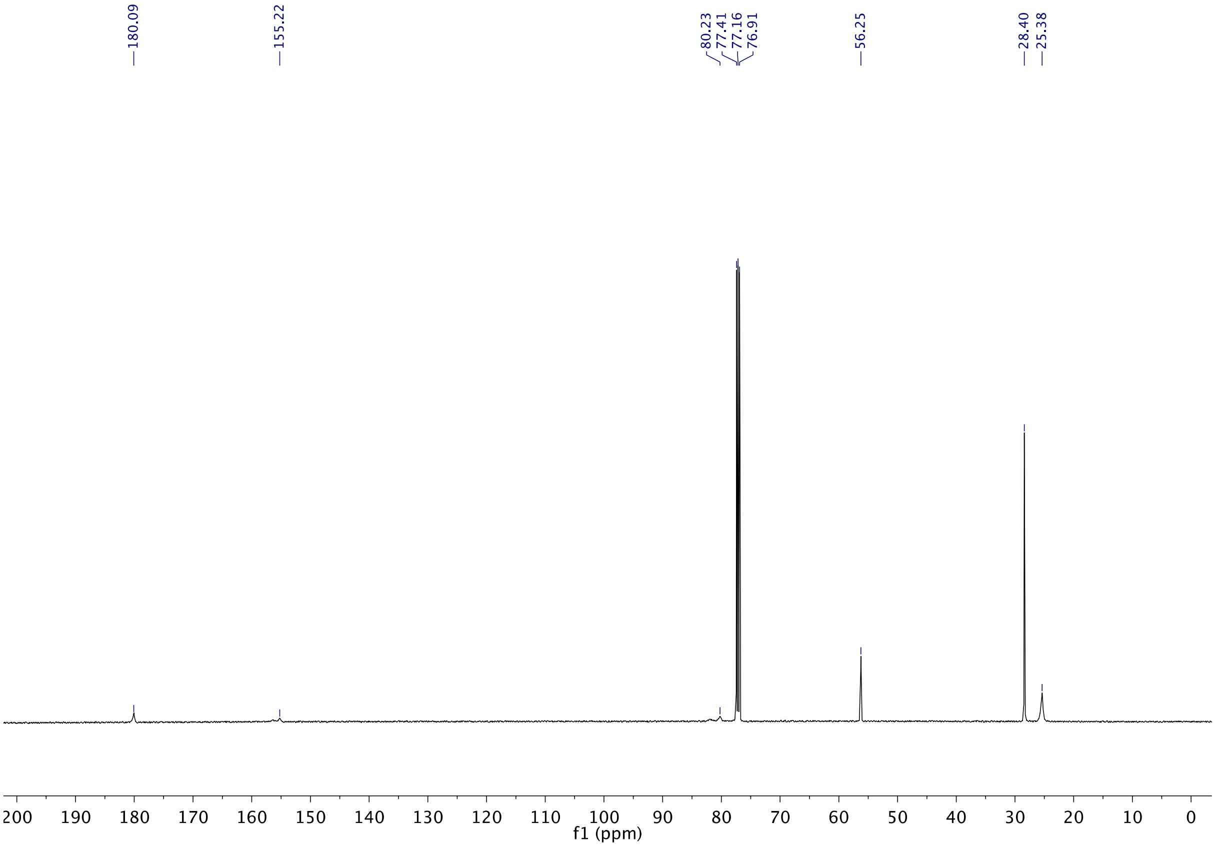

1,3-Dioxoisoindolin-2-yl 2-((*tert*-butoxycarbonyl)amino)-2-methylpropanoate, **N34**

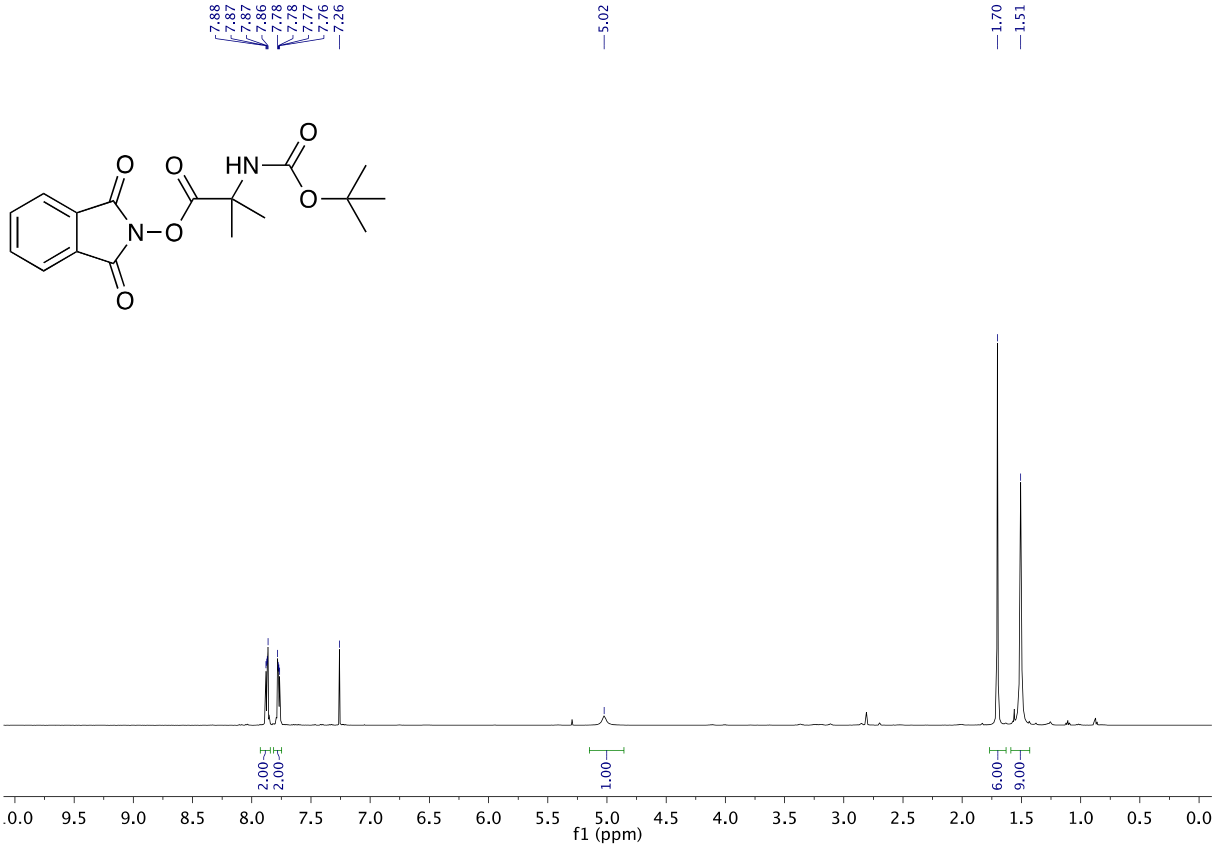

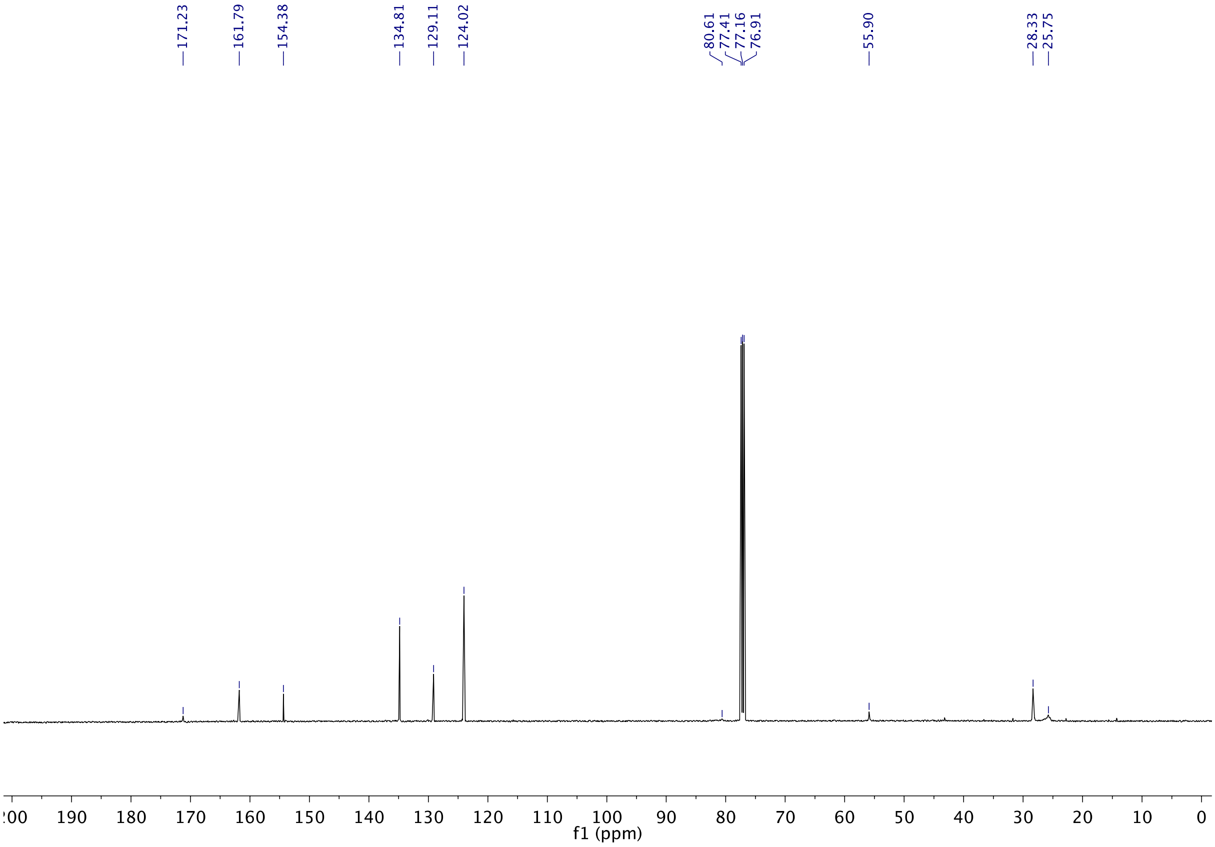

1,3-Dioxoisoindolin-2-yl methyl malonate, **N35**

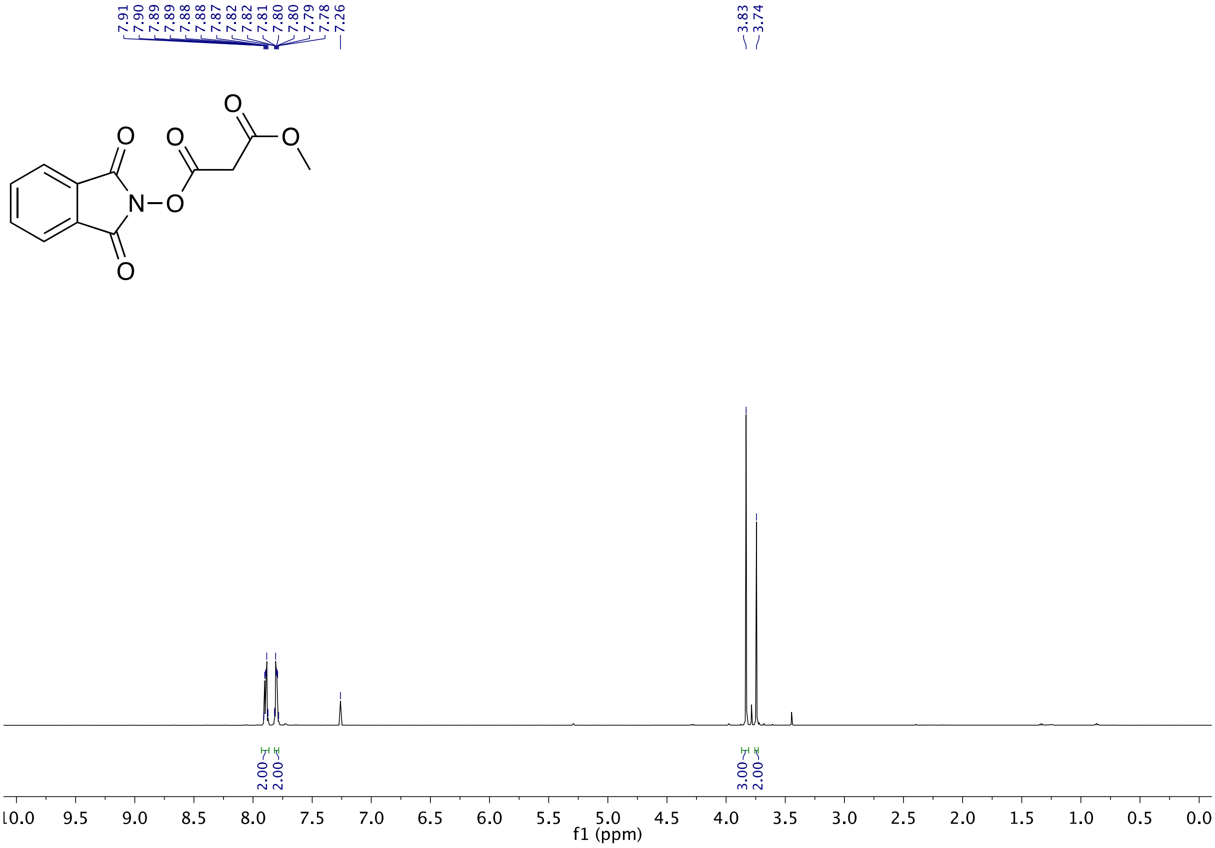

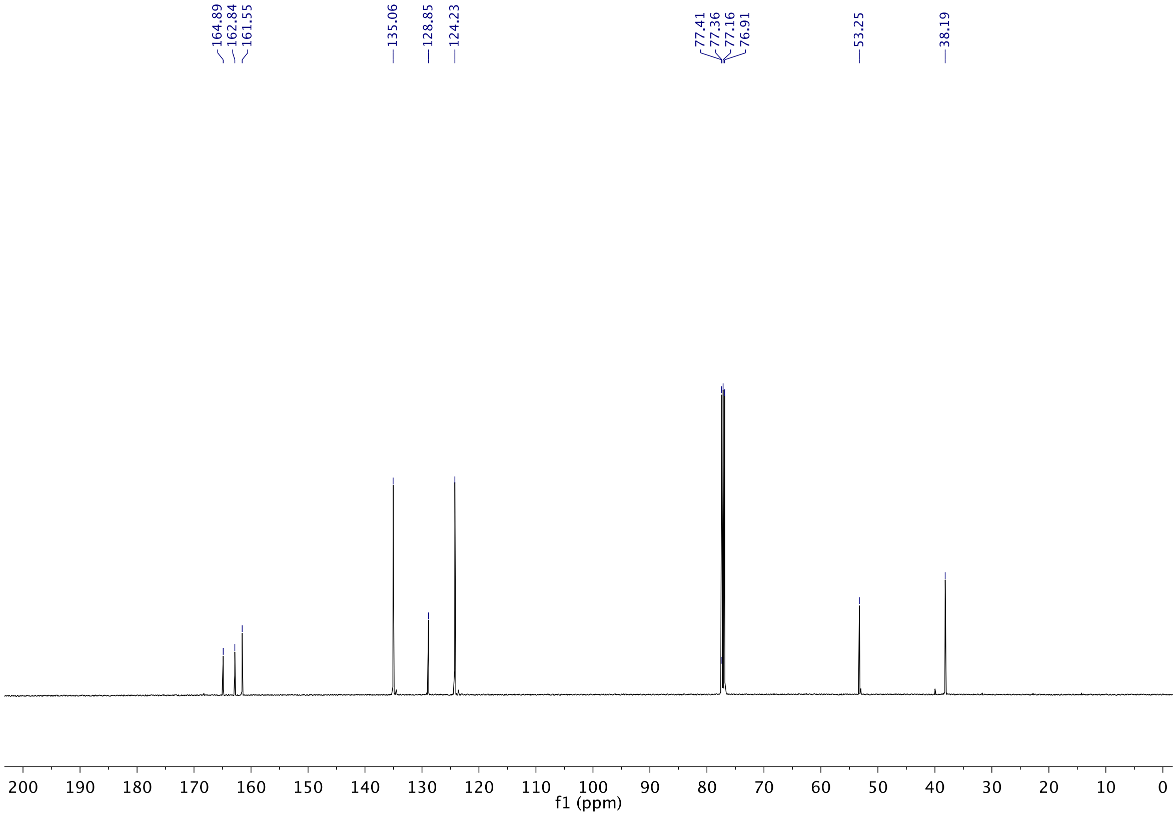

1,3-Dioxoisoindolin-2-yl cyclopropanecarboxylate, **N36**

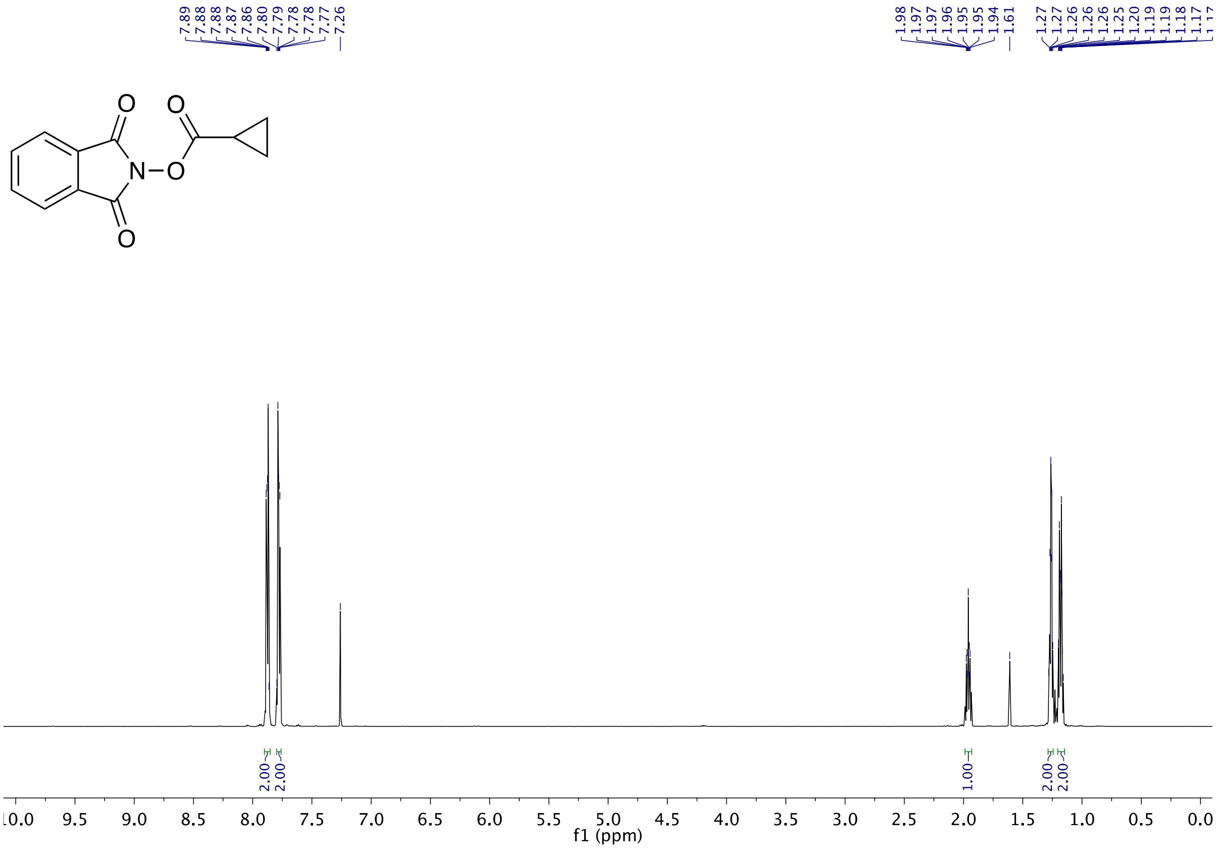

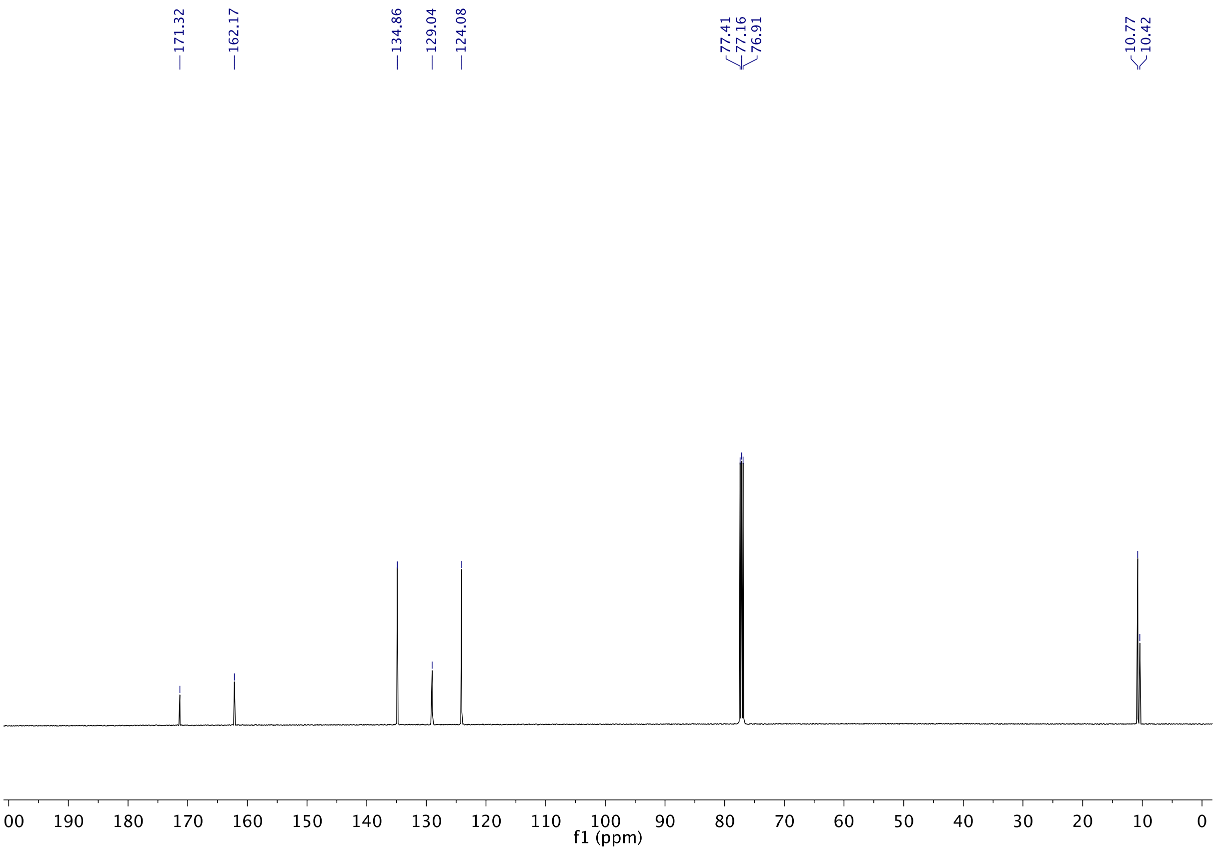

1,3-Dioxoisoindolin-2-yl 2-phenylpropanoate, **N37**

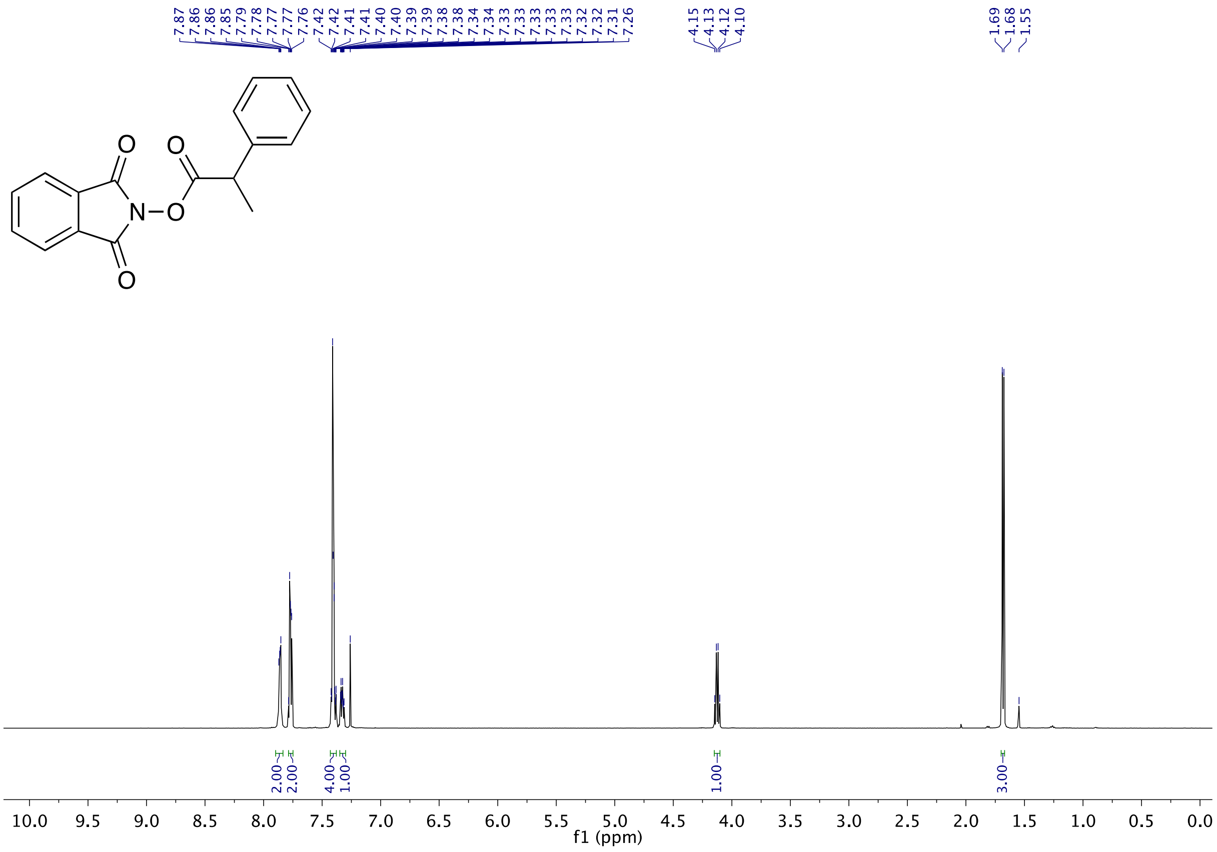

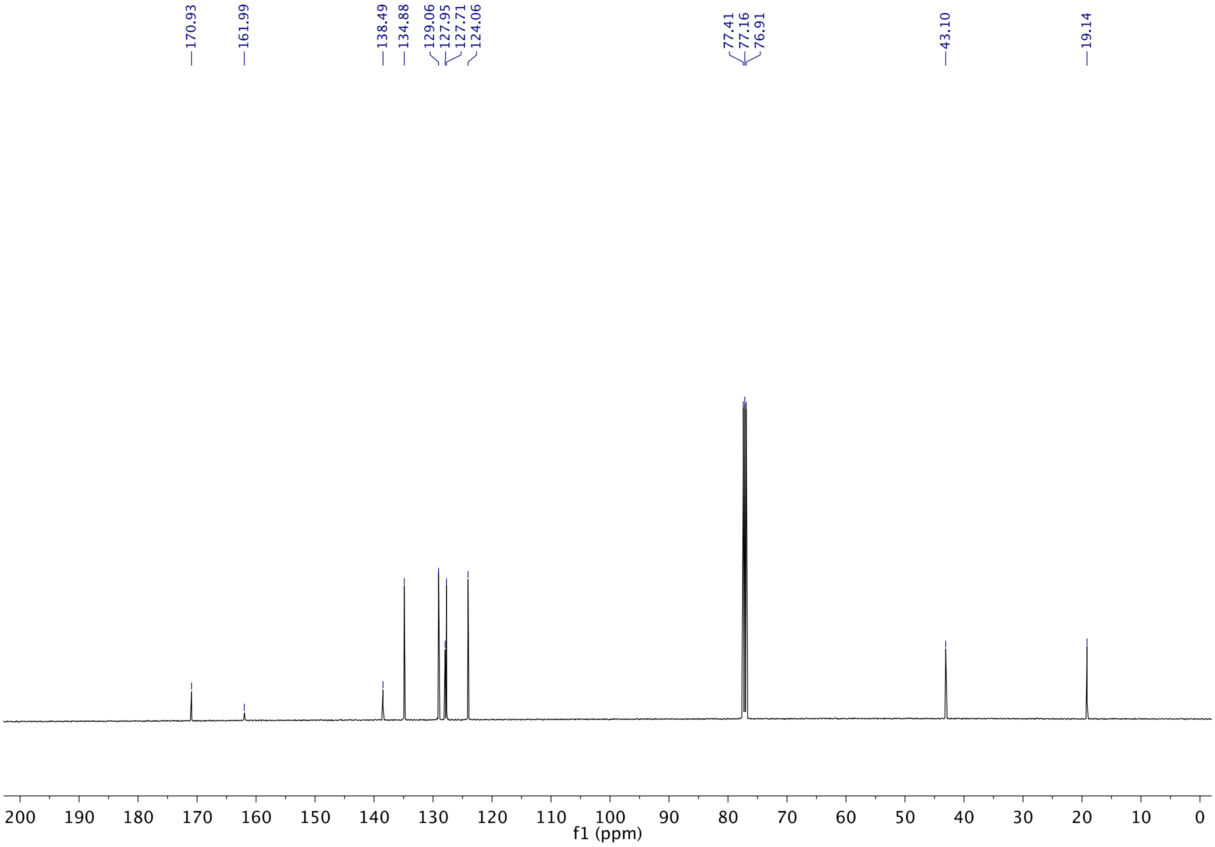

1,3-Dioxoisoindolin-2-yl 2-(thiophen-3-yl)acetate, **N38**

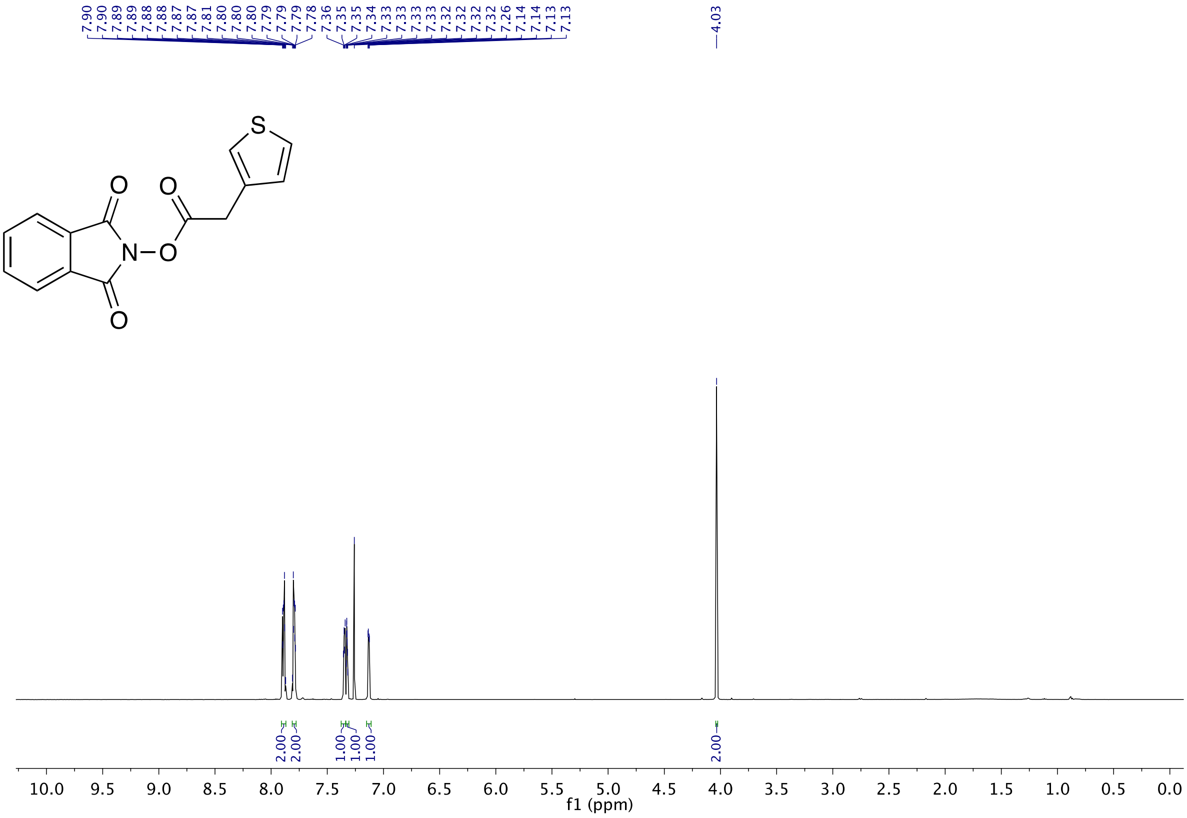

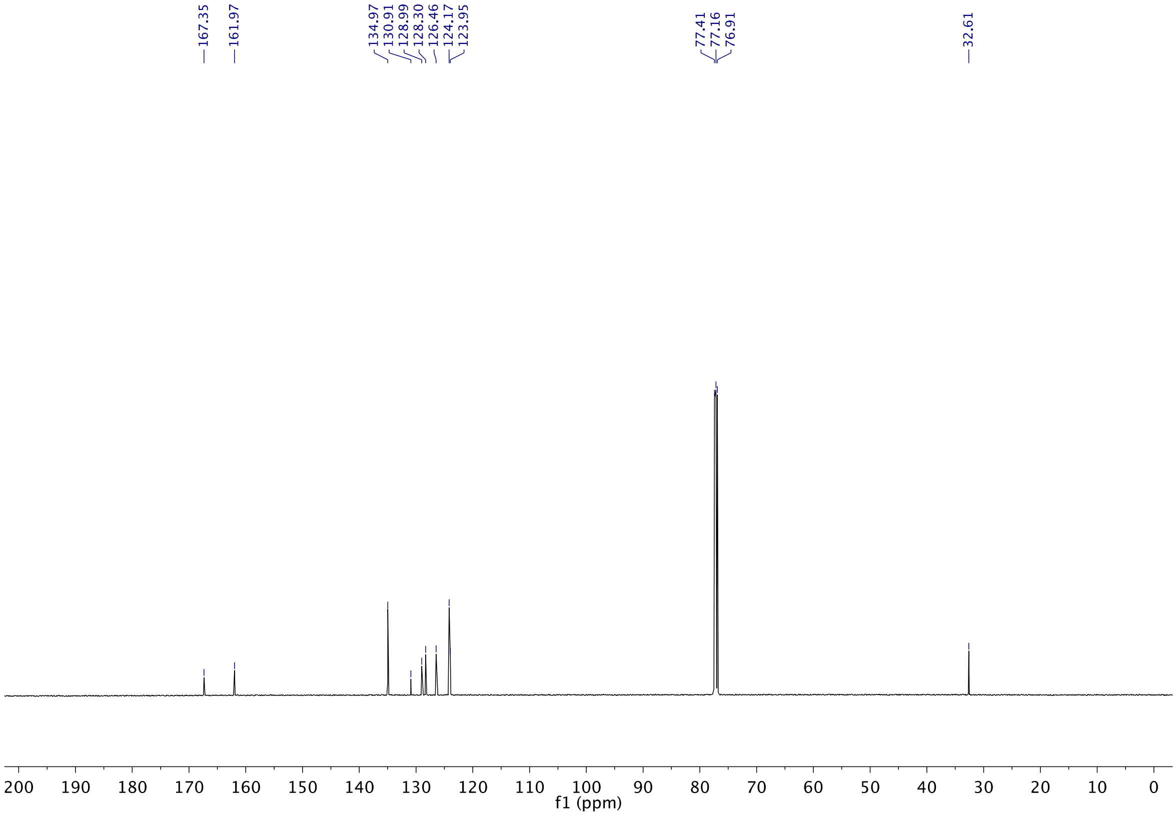

2-(2-(4-Bromophenyl)acetyl)isoindoline-1,3-dione, **N39**

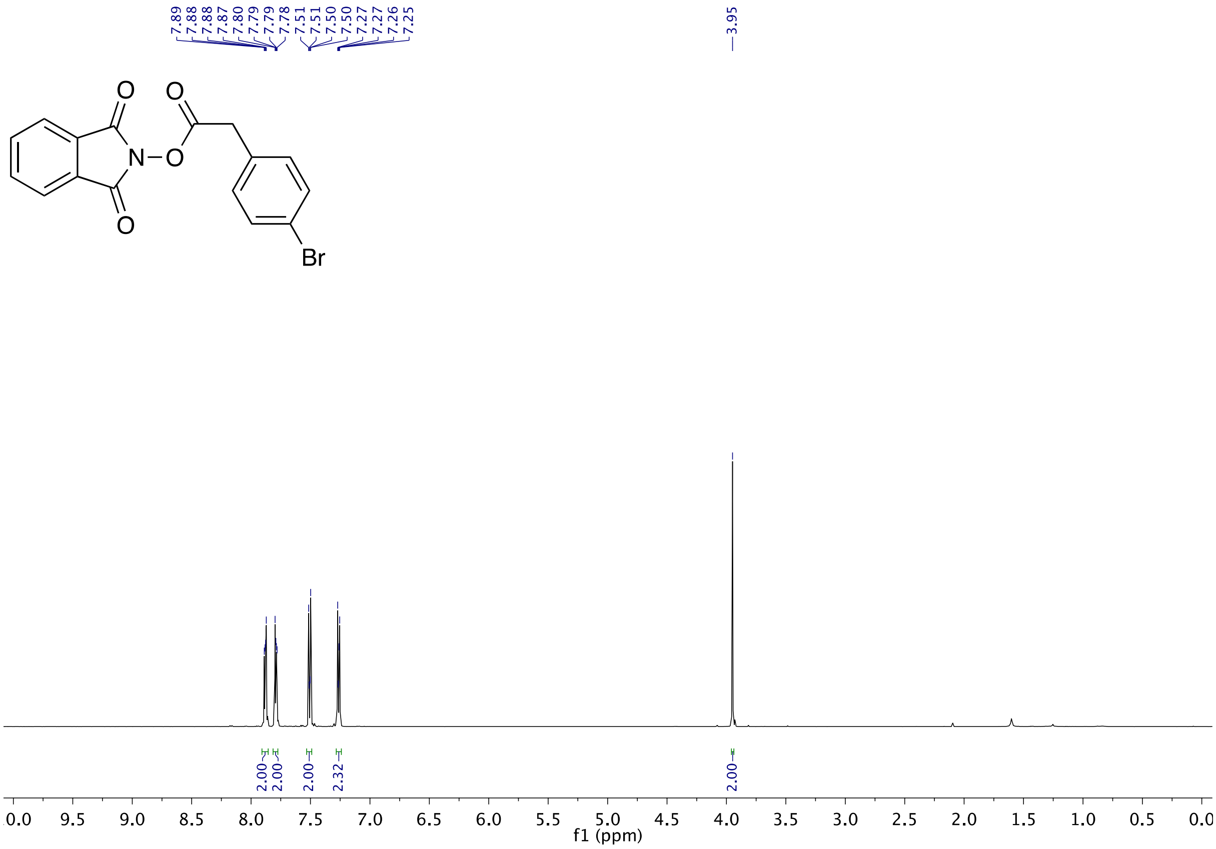

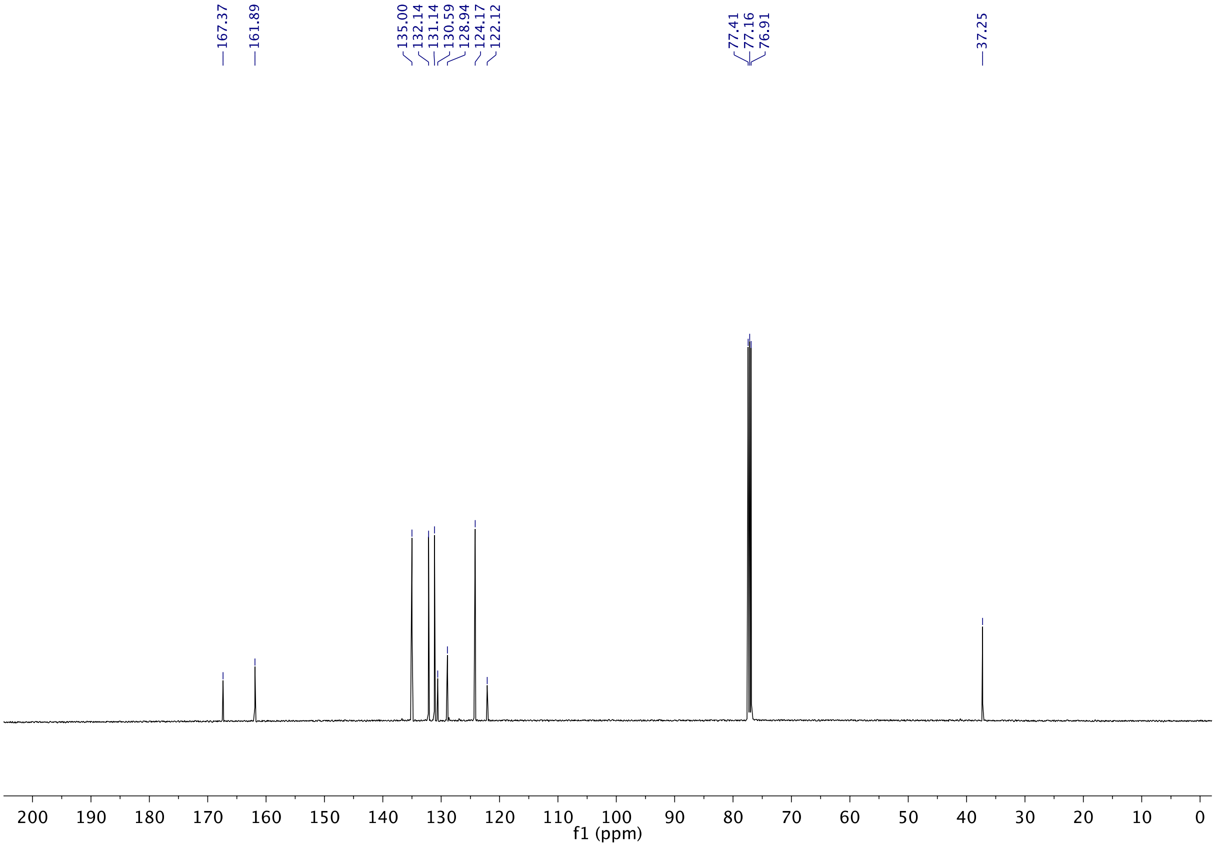

1,3-Dioxoisoindolin-2-yl (*E*)-but-2-enoate, **N40**

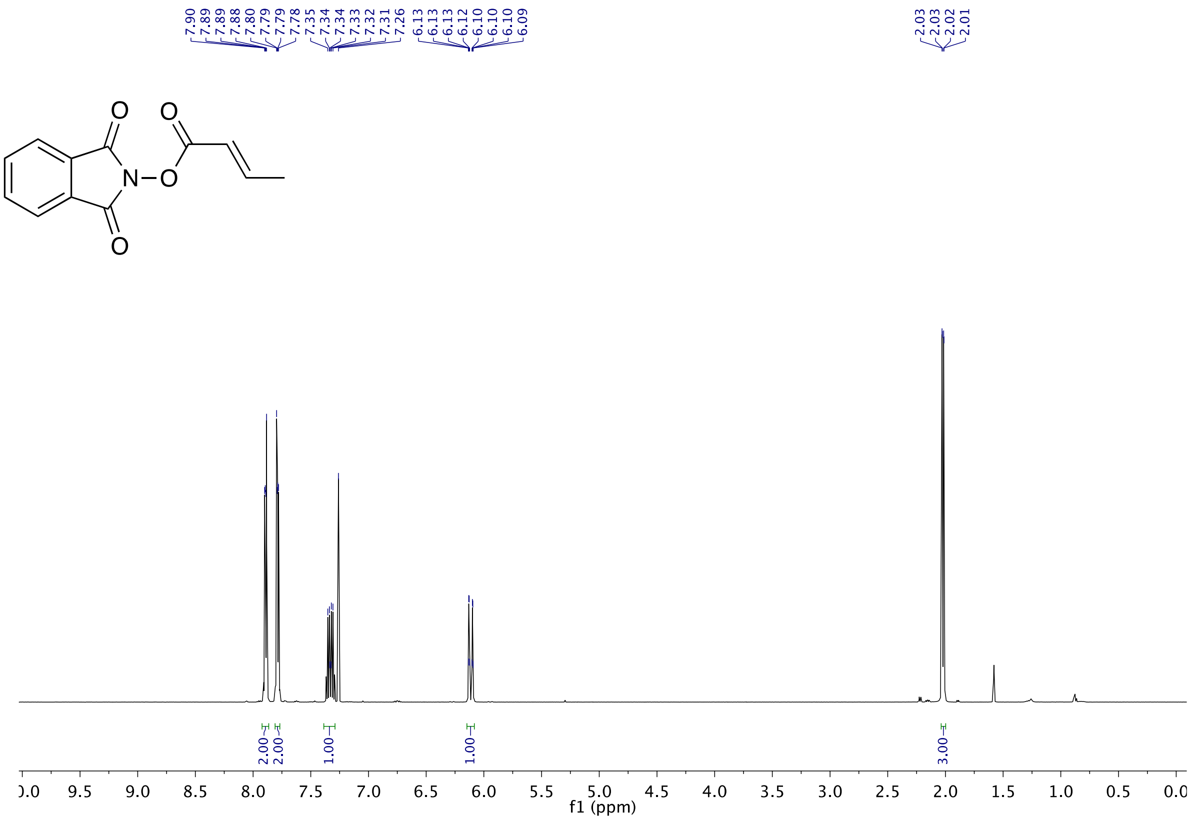

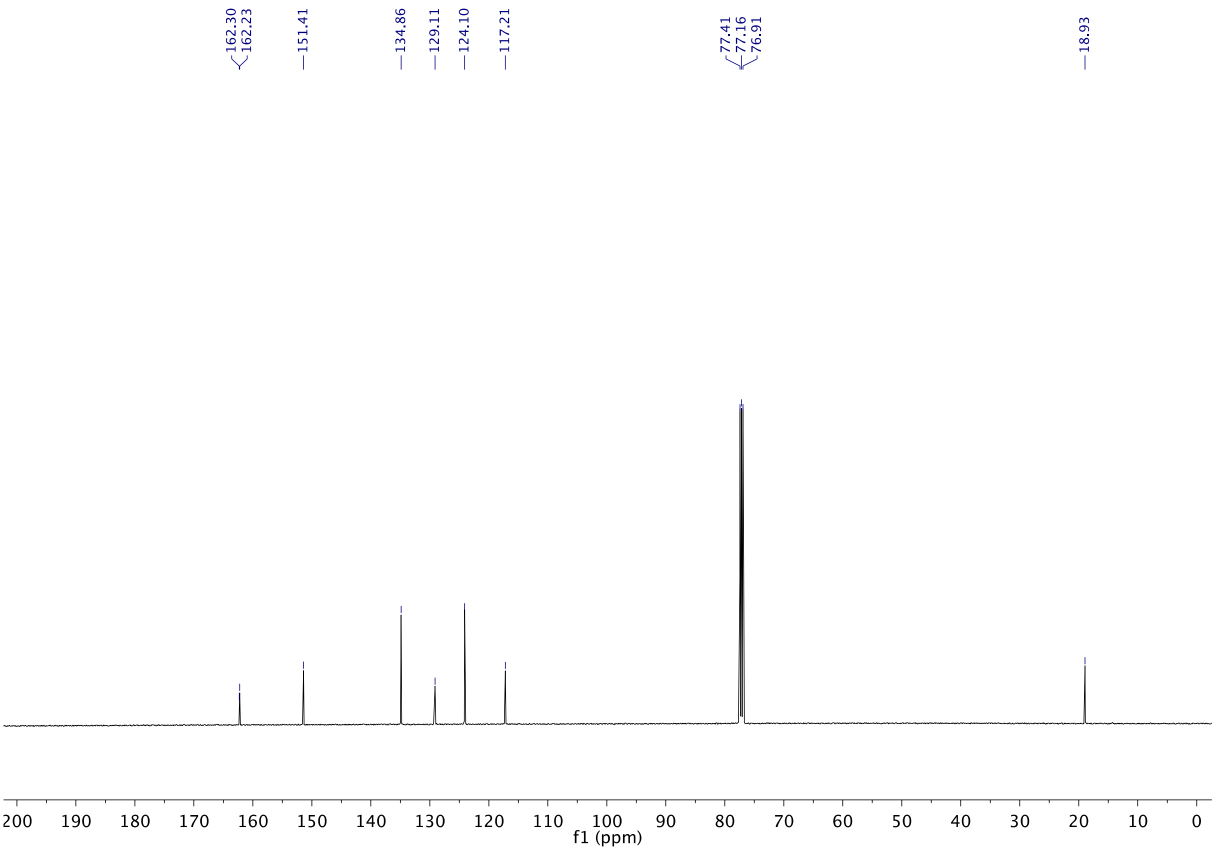

1,3-Dioxoisoindolin-2-yl cinnamate, **N41**

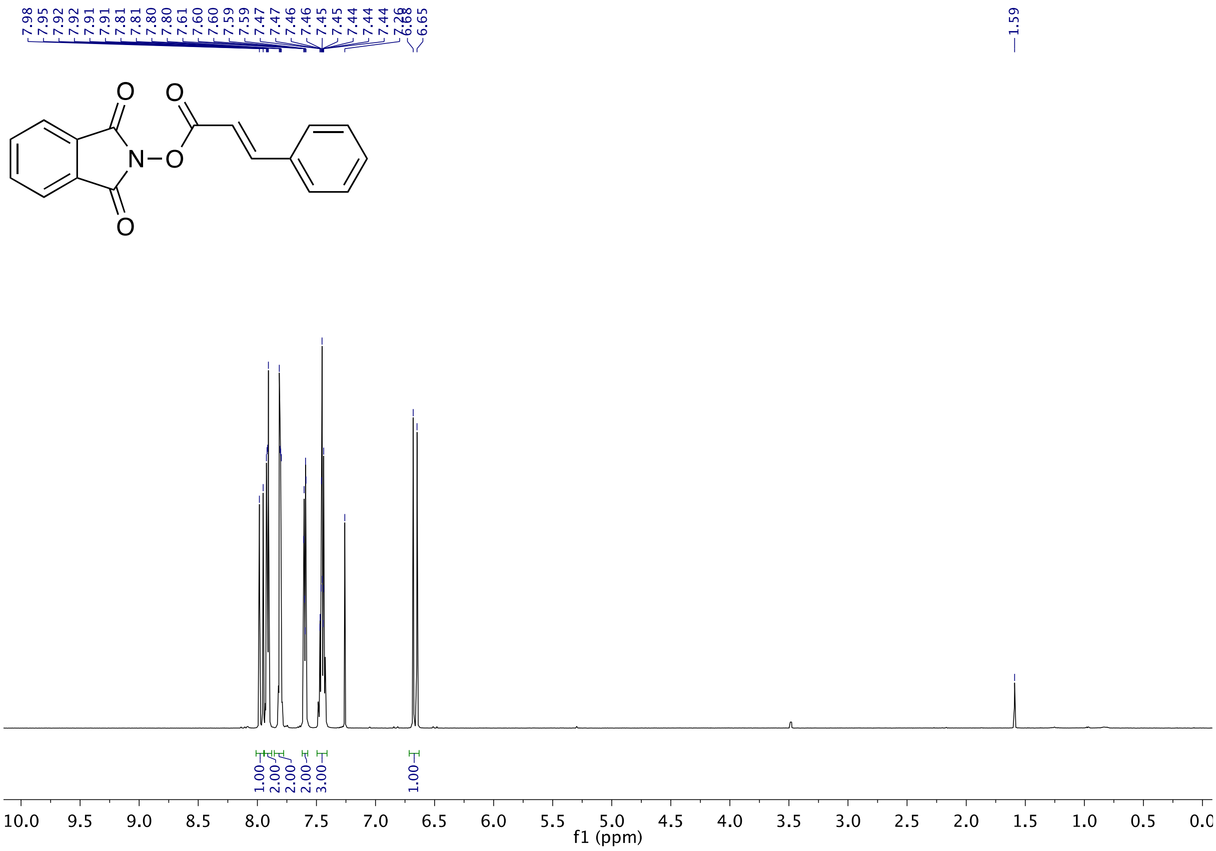

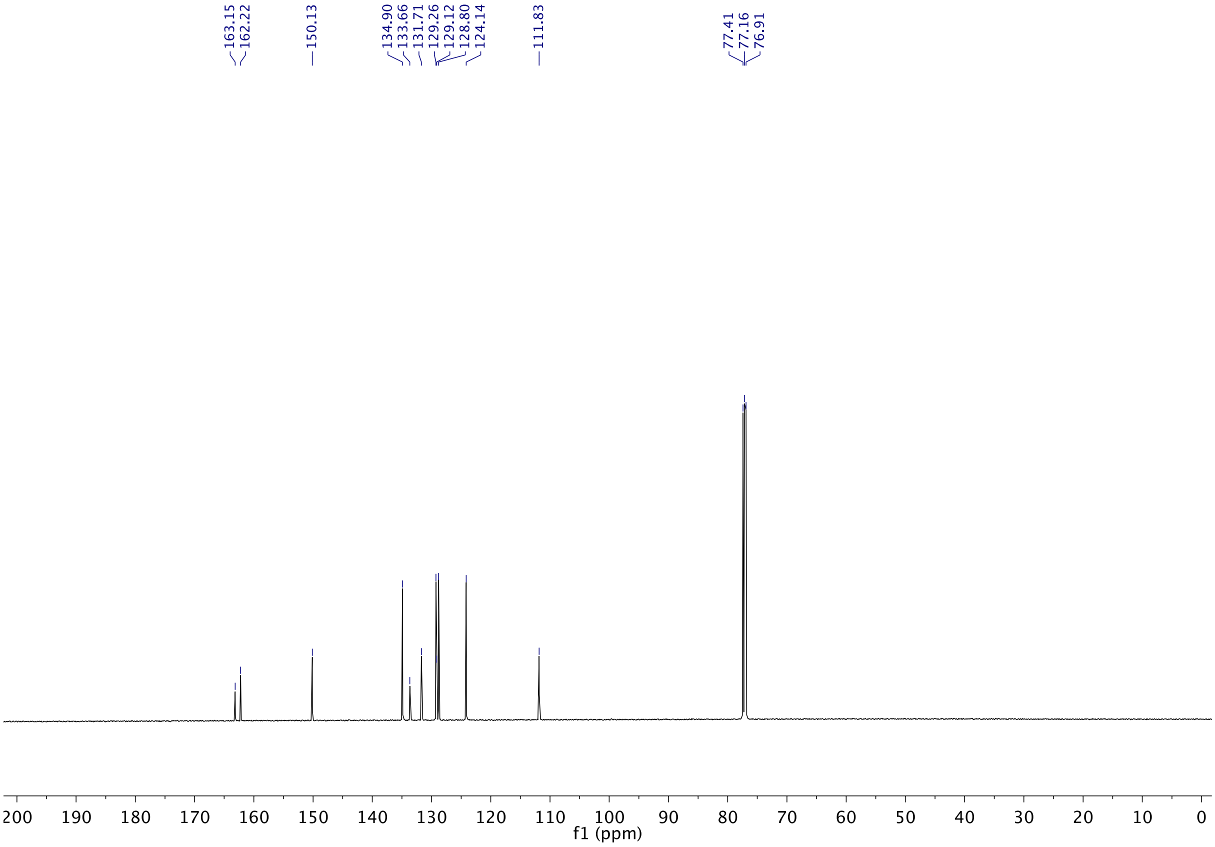

1,3-Dioxoisoindolin-2-yl cyclohex-1-ene-1-carboxylate, **N42**

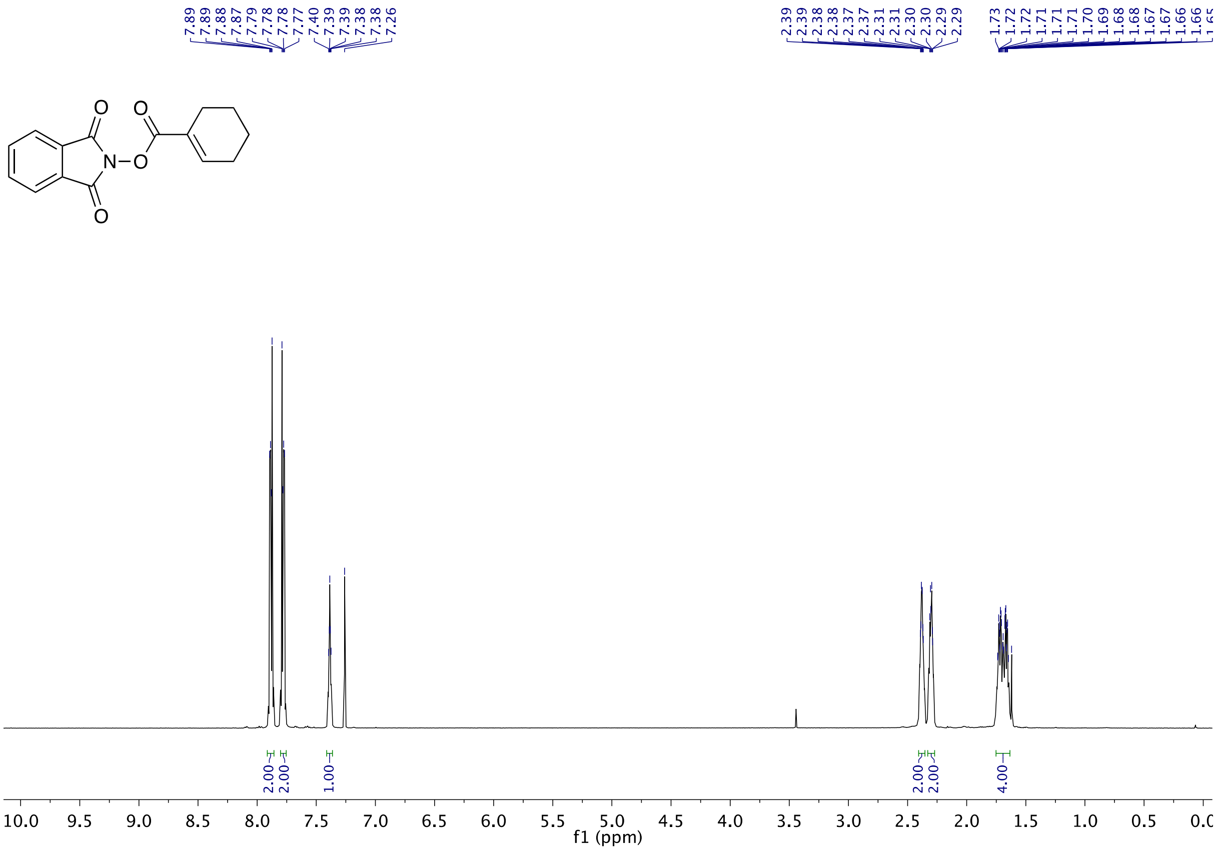

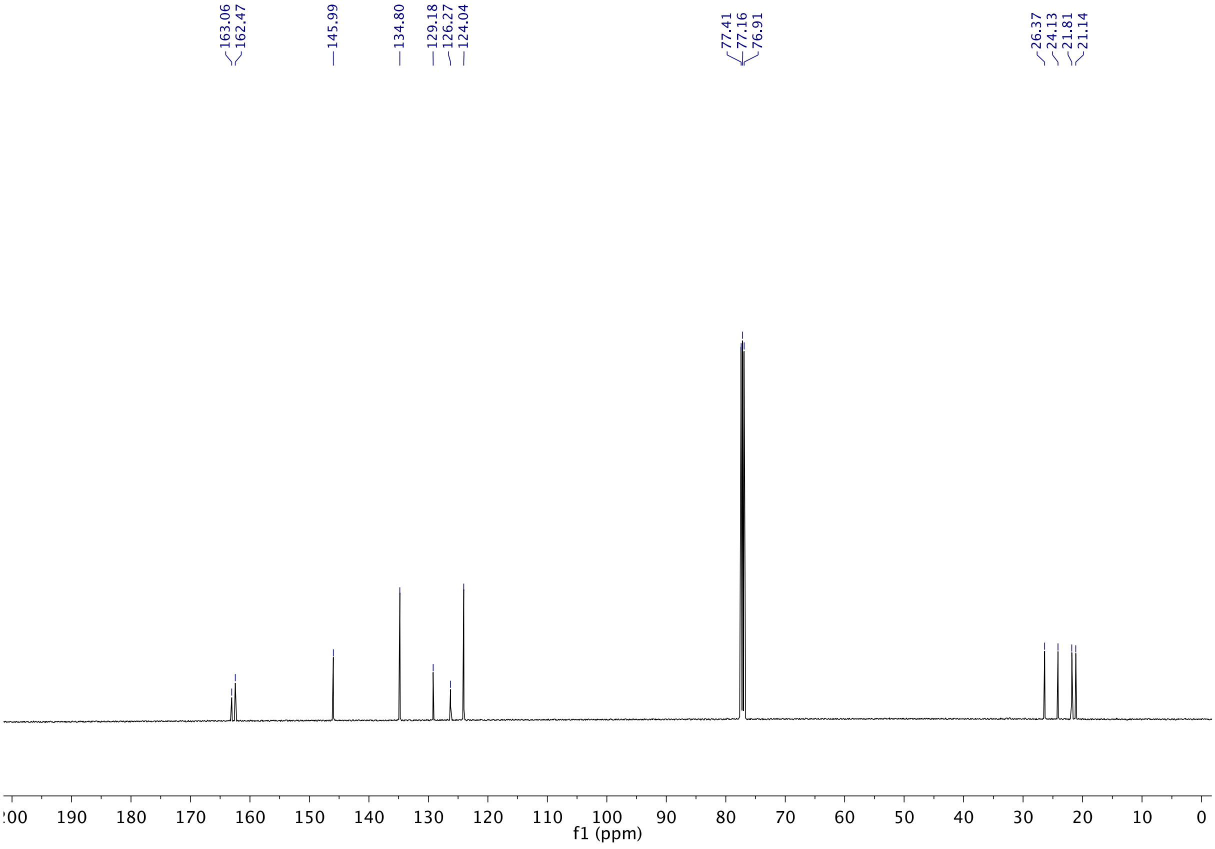

1,3-Dioxoisoindolin-2-yl tetrahydrofuran-3-carboxylate, **N43**

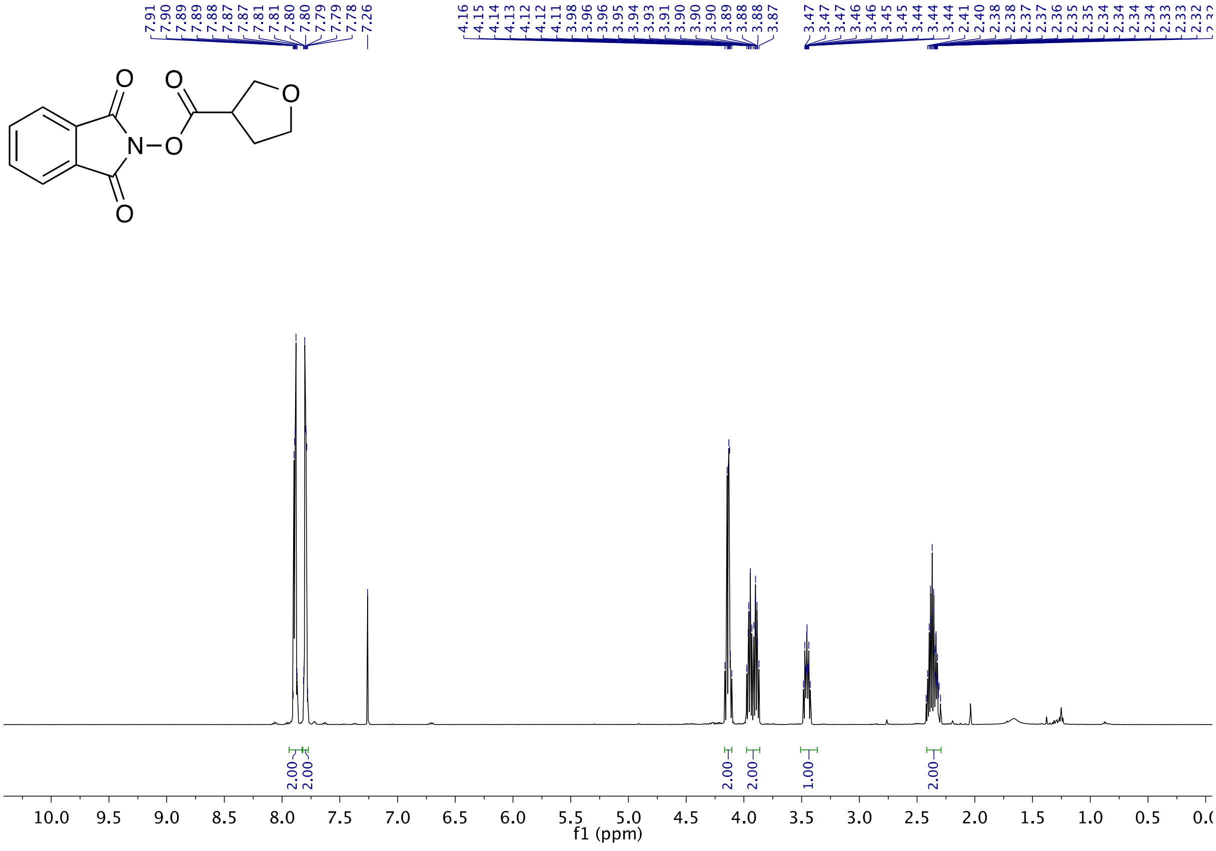

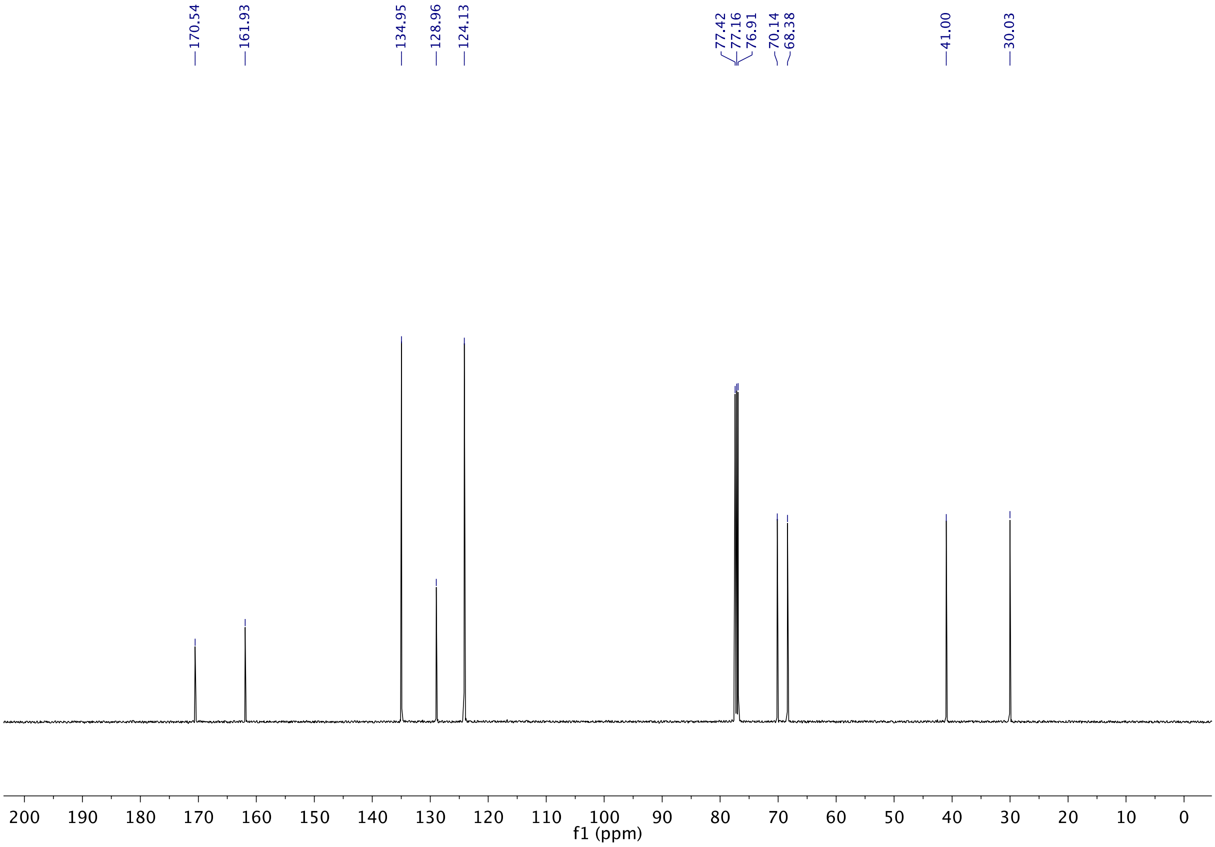

1,3-Dioxoisoindolin-2-yl tetrahydrofuran-2-carboxylate, **N44**

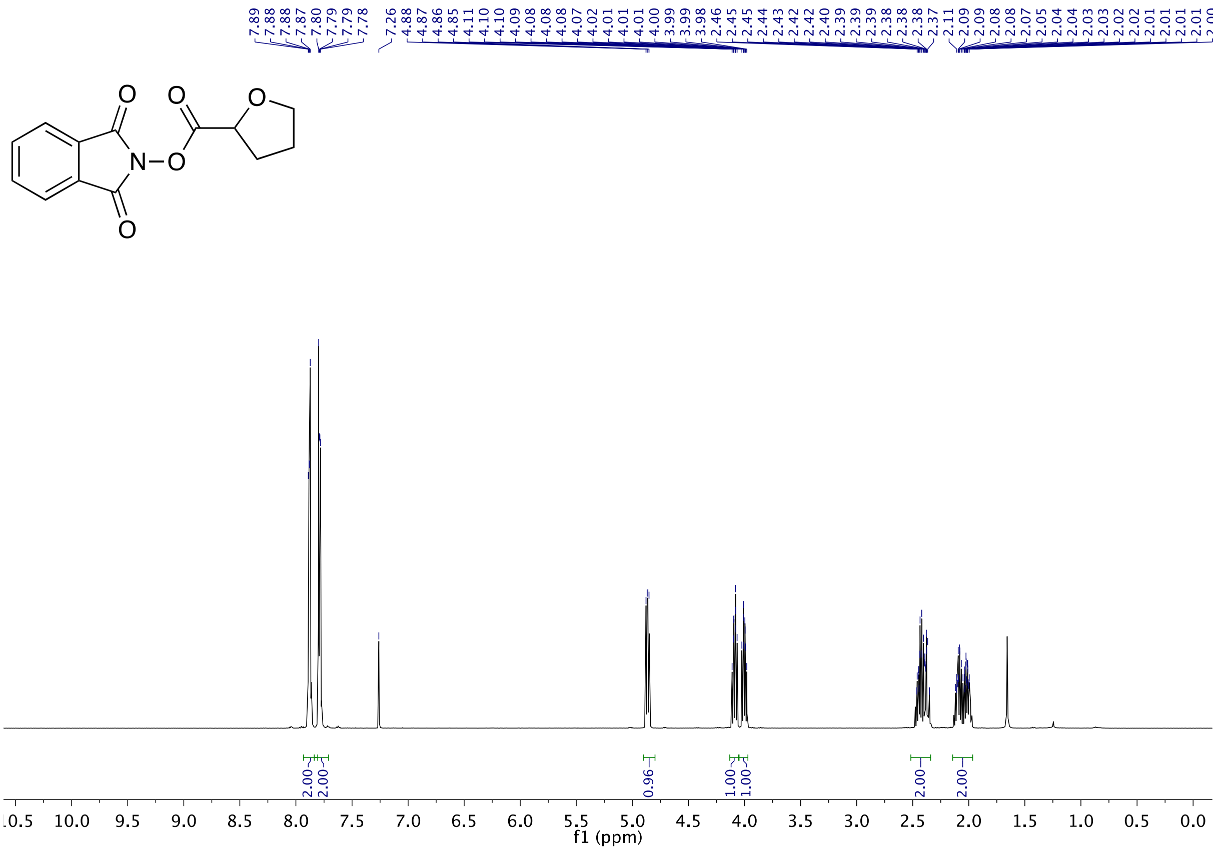

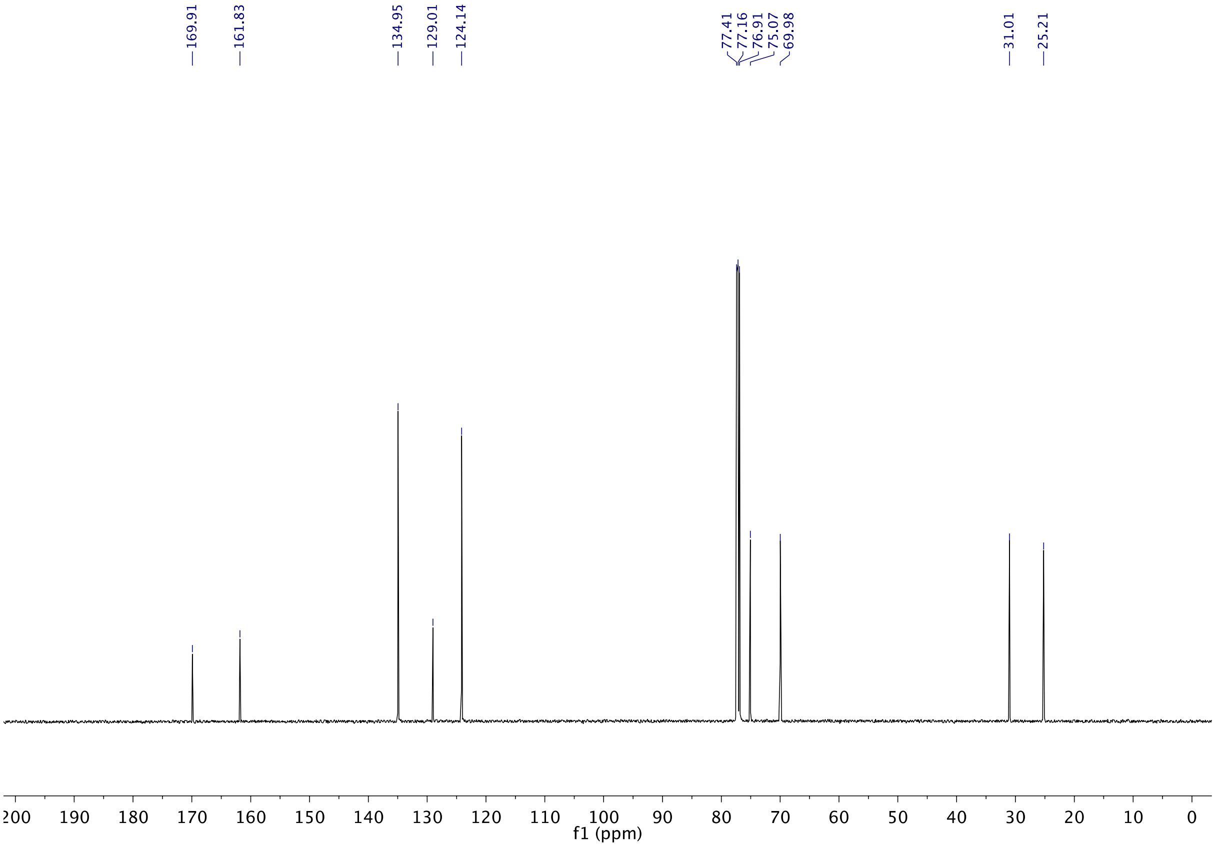

1,3-Dioxoisoindolin-2-yl tetrahydro-2*H*-pyran-2-carboxylate, **N45**

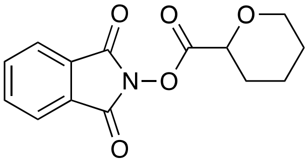

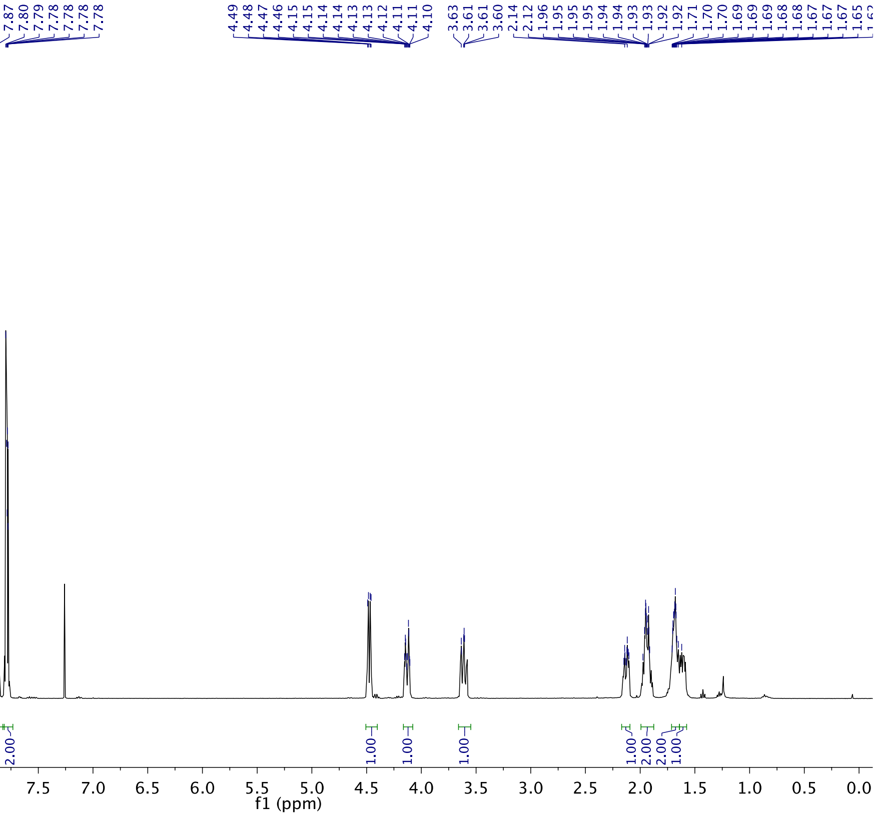

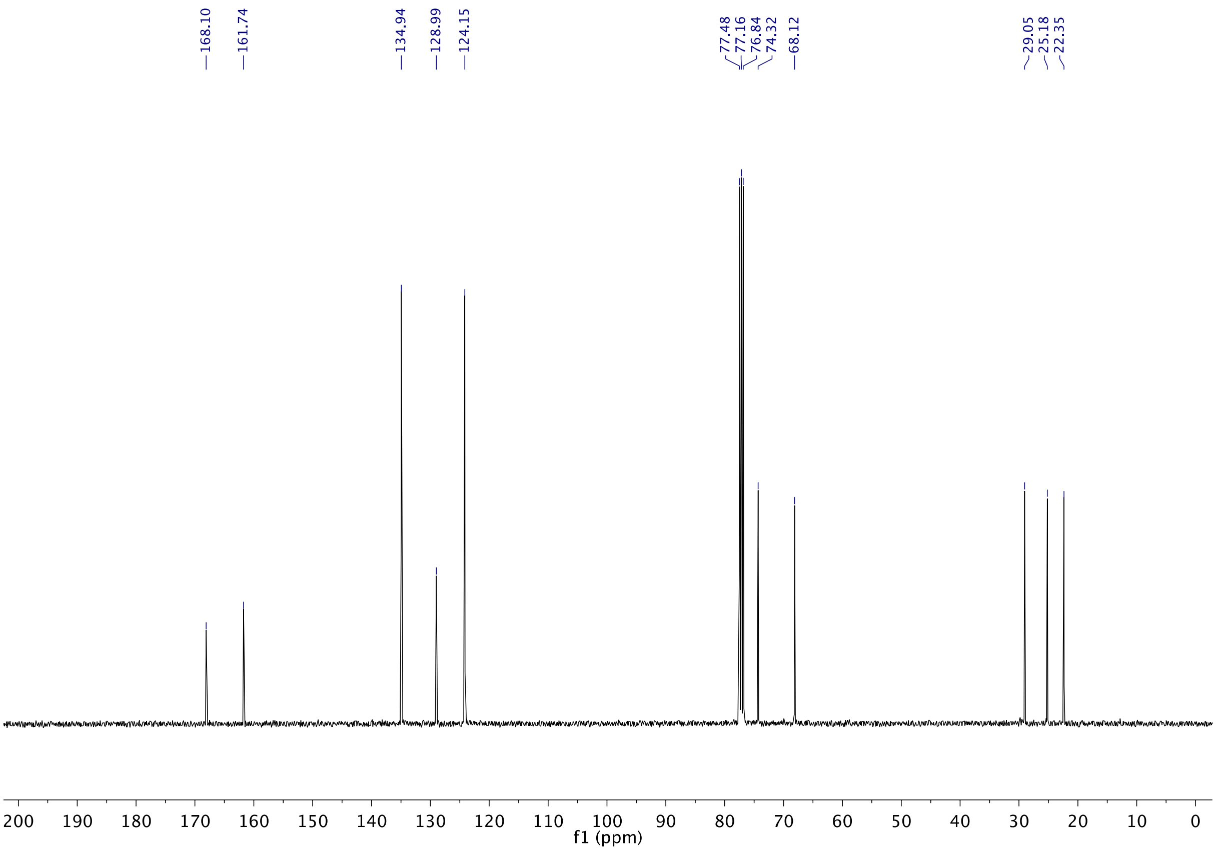

1,3-Dioxoisoindolin-2-yl 2,2-dimethylbut-3-enoate, **N46**

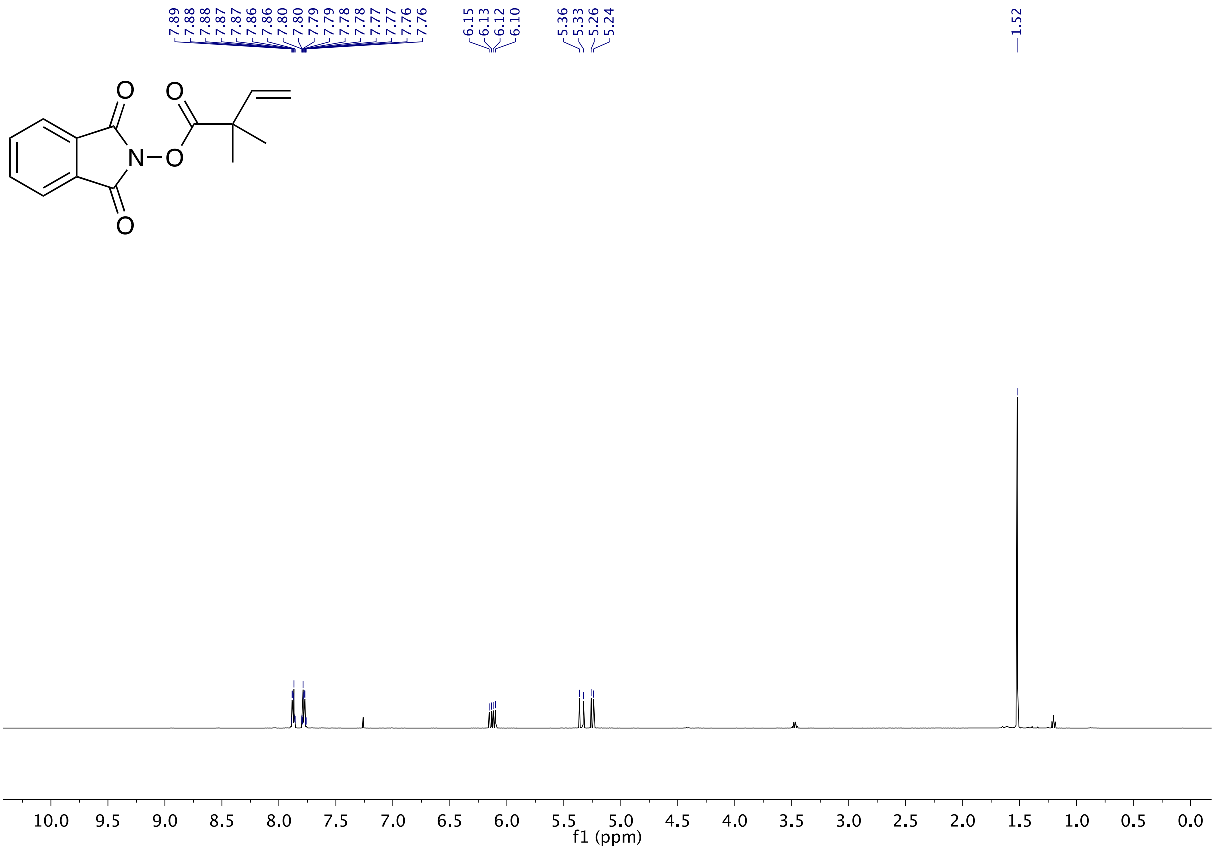

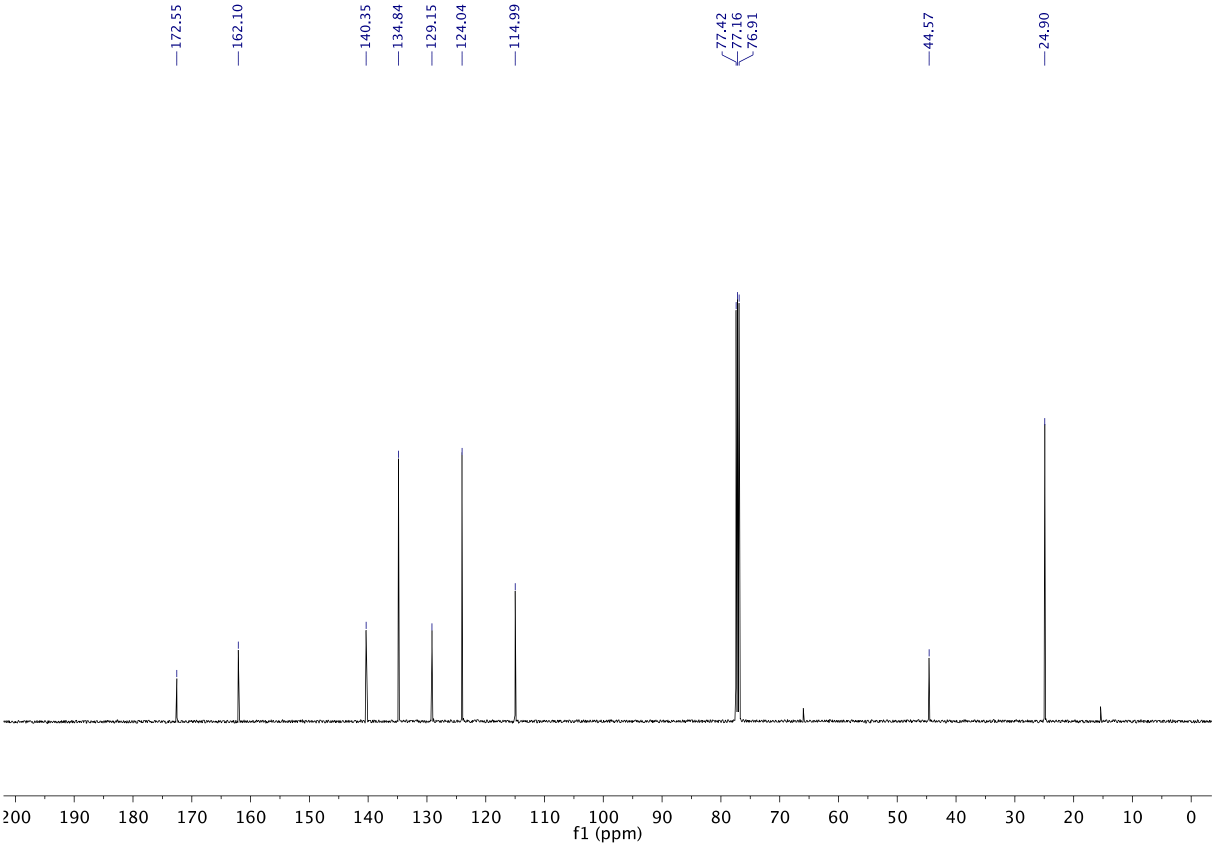

4-((Trimethylsilyl)ethynyl)benzonitrile, **S1-int1**

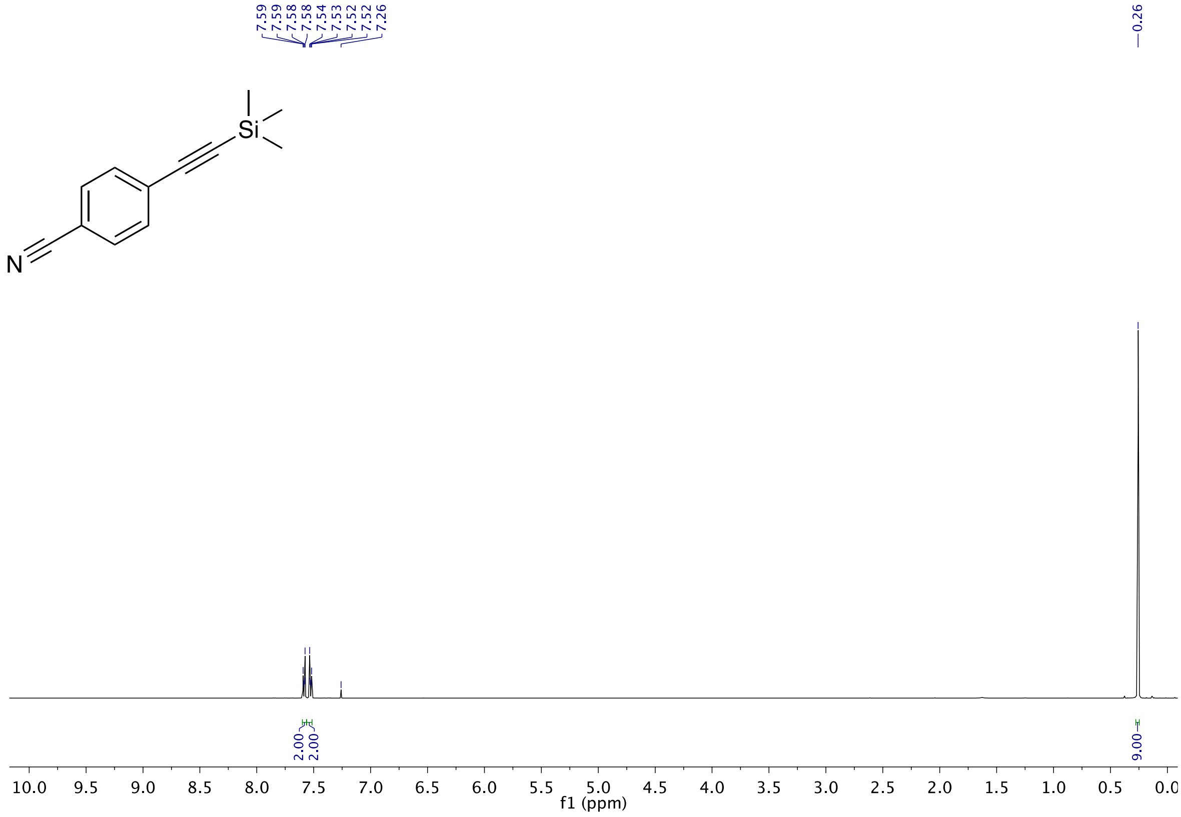

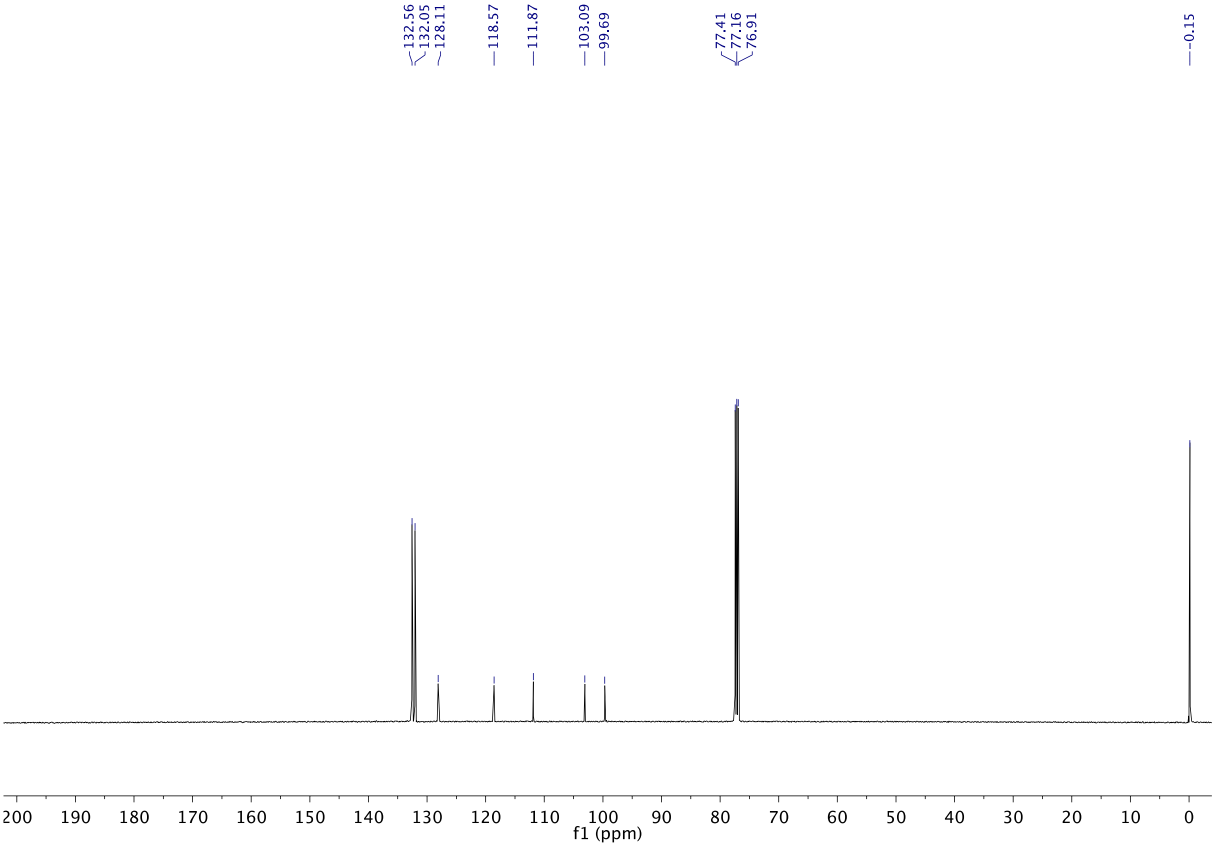

4-Ethynylbenzonitrile, **S1-int2**

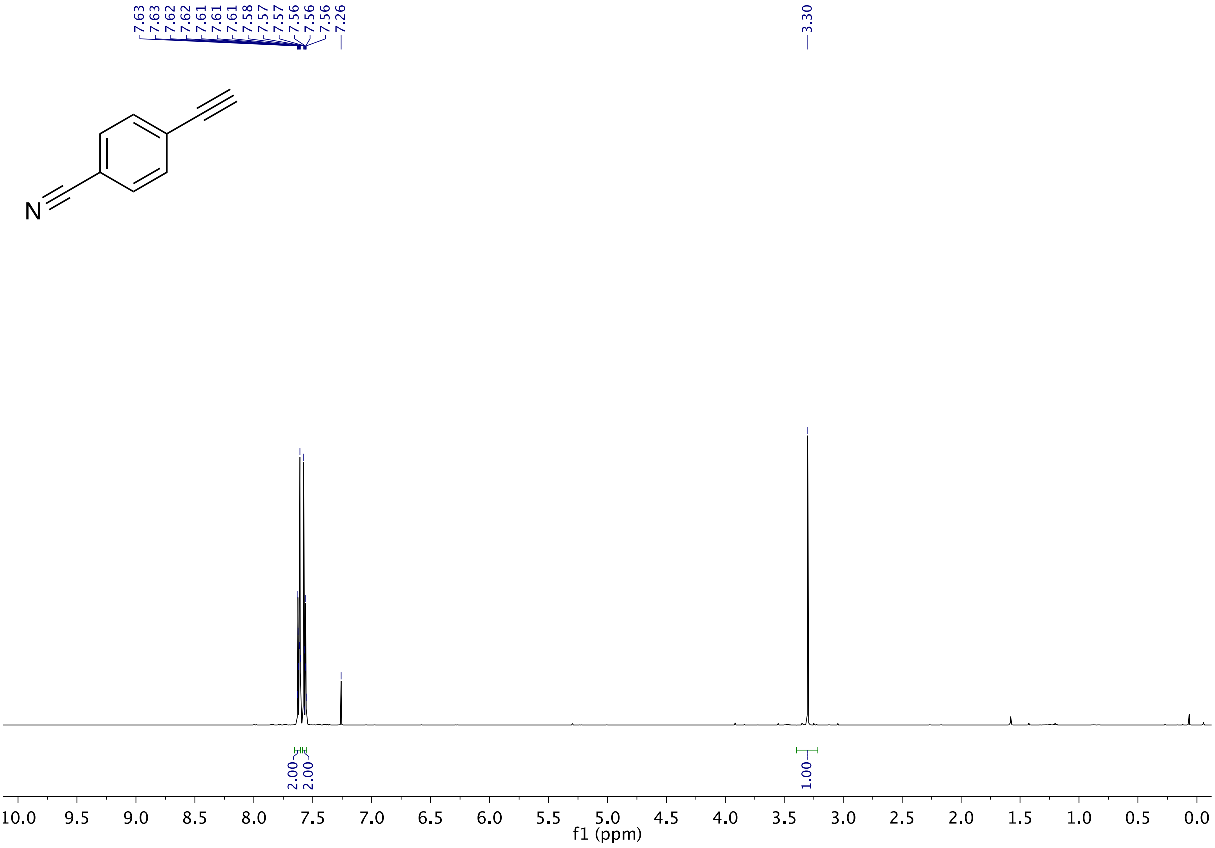

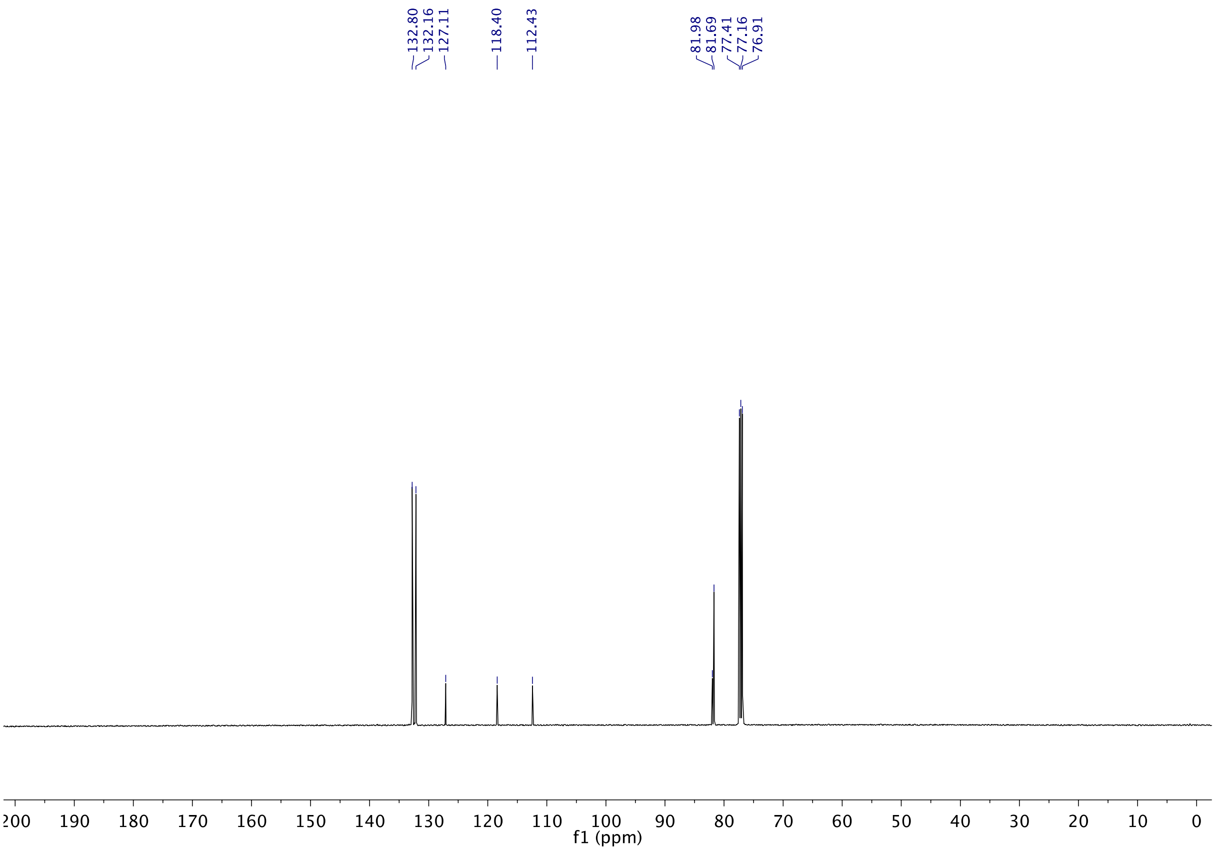

(*E*)-4-(2-(4,4,5,5-Tetramethyl-1,3,2-dioxaborolan-2-yl)vinyl)benzonitrile, **S1-int3**

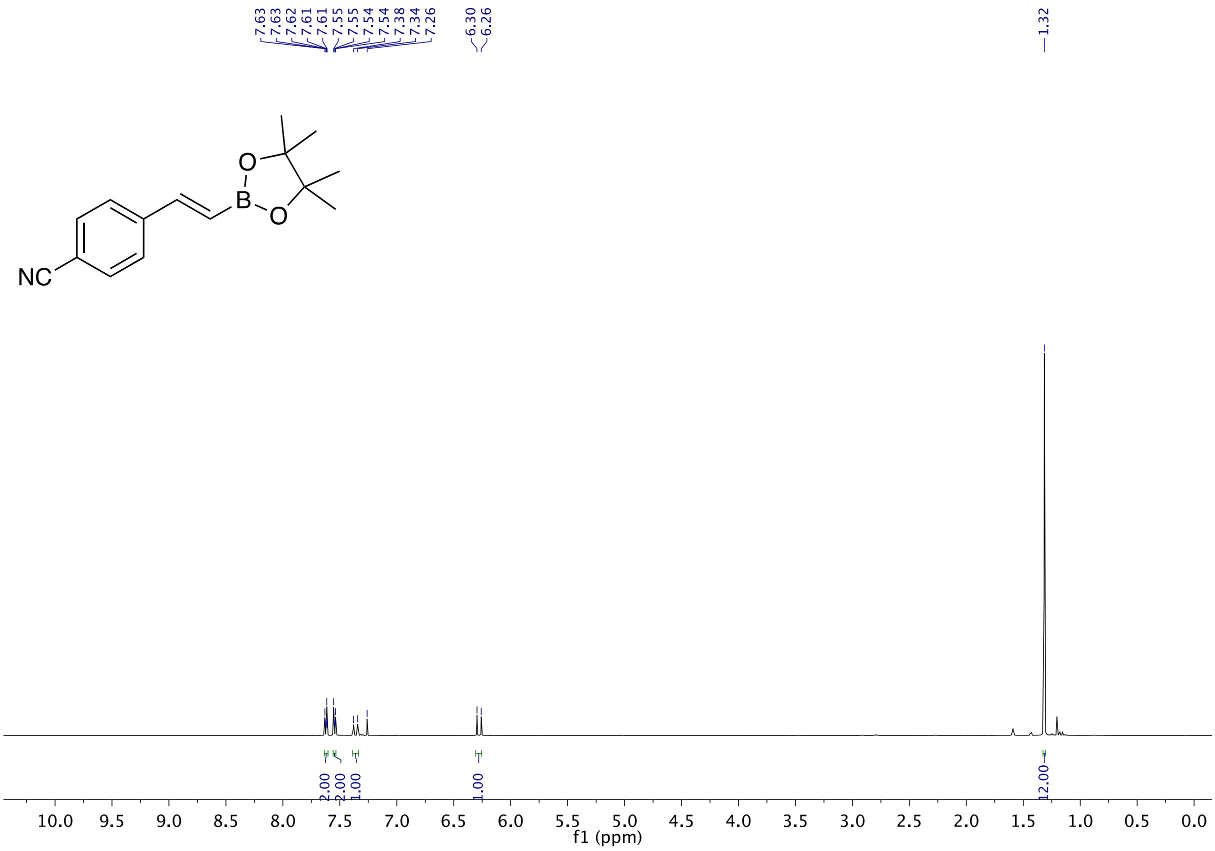

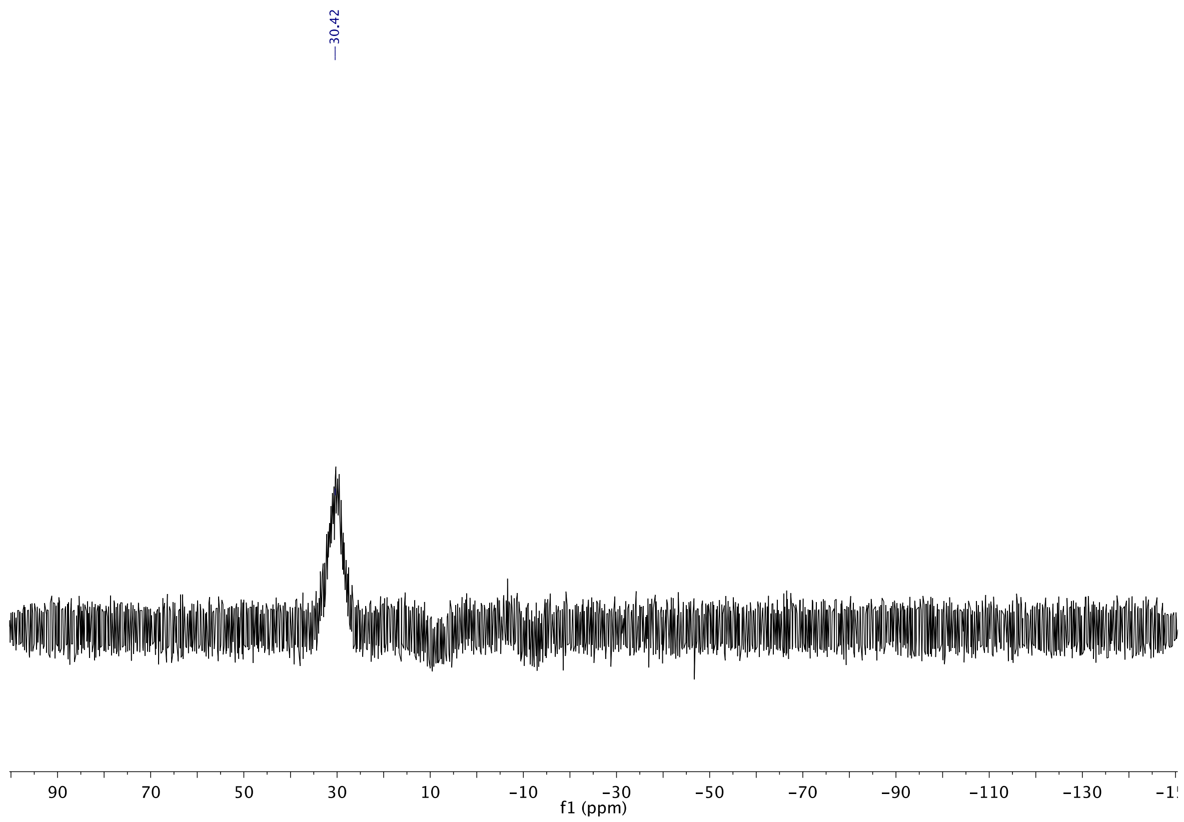
30.42
90 70 50 30 10 −10 −30 −50 −70 −90 −110 −130
f1 (ppm)

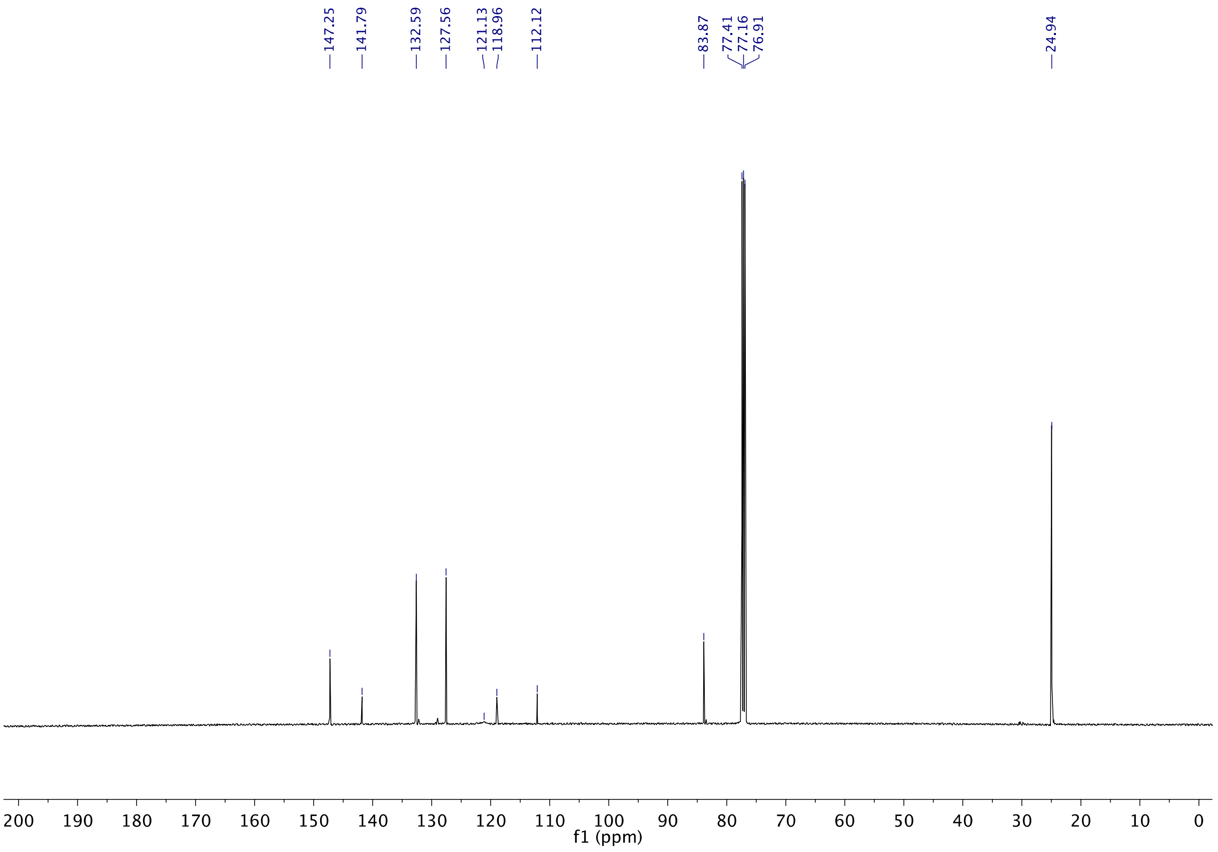
147.25
141.79
132.59
127.56
121.13
118.96
112.12
83.87
77.41
77.16
76.91
24.94
200 190 180 170 160 150 140 130 120 110 100 90 80 70 60 50 40 30 20 10 0
f1 (ppm)

(*E*)-4-(2-(Trifluoro-λ^4-boraneyl)vinyl)benzonitrile, potassium salt, **S1-int4**

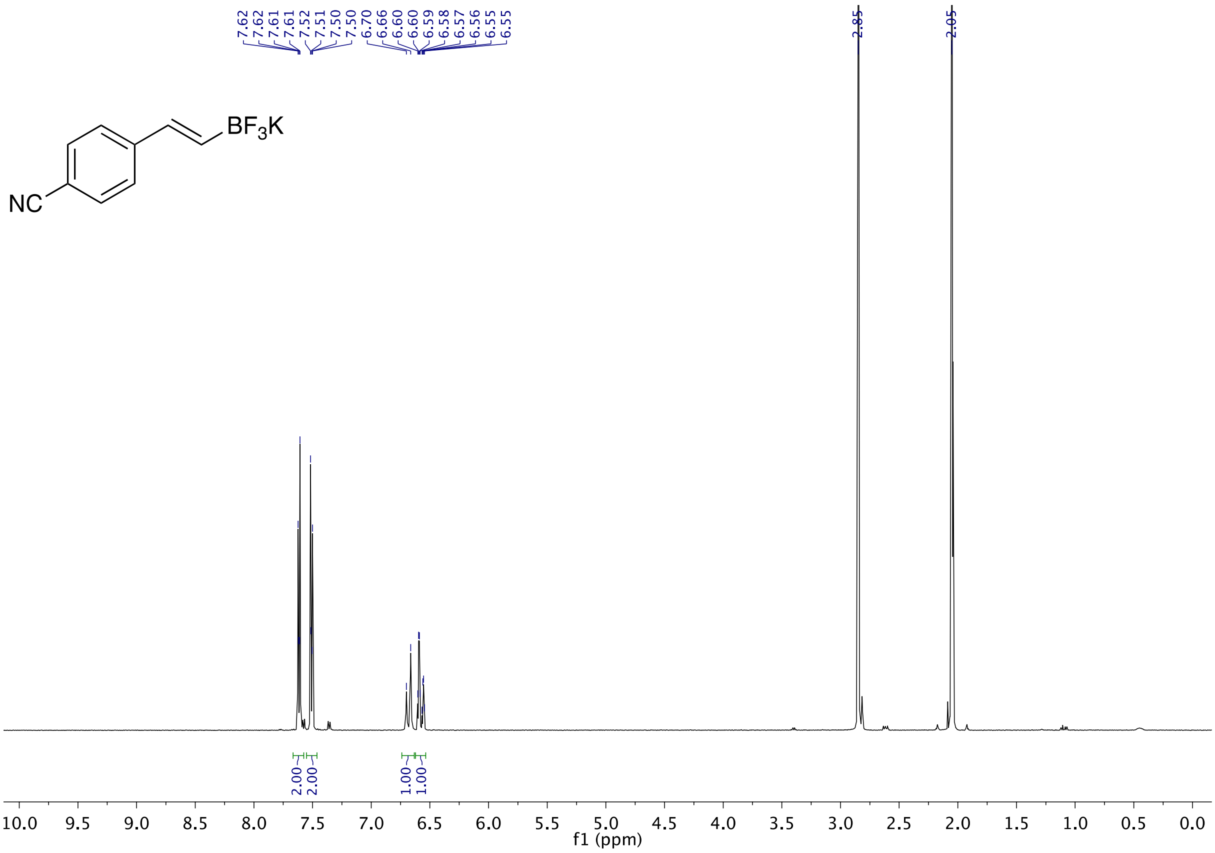

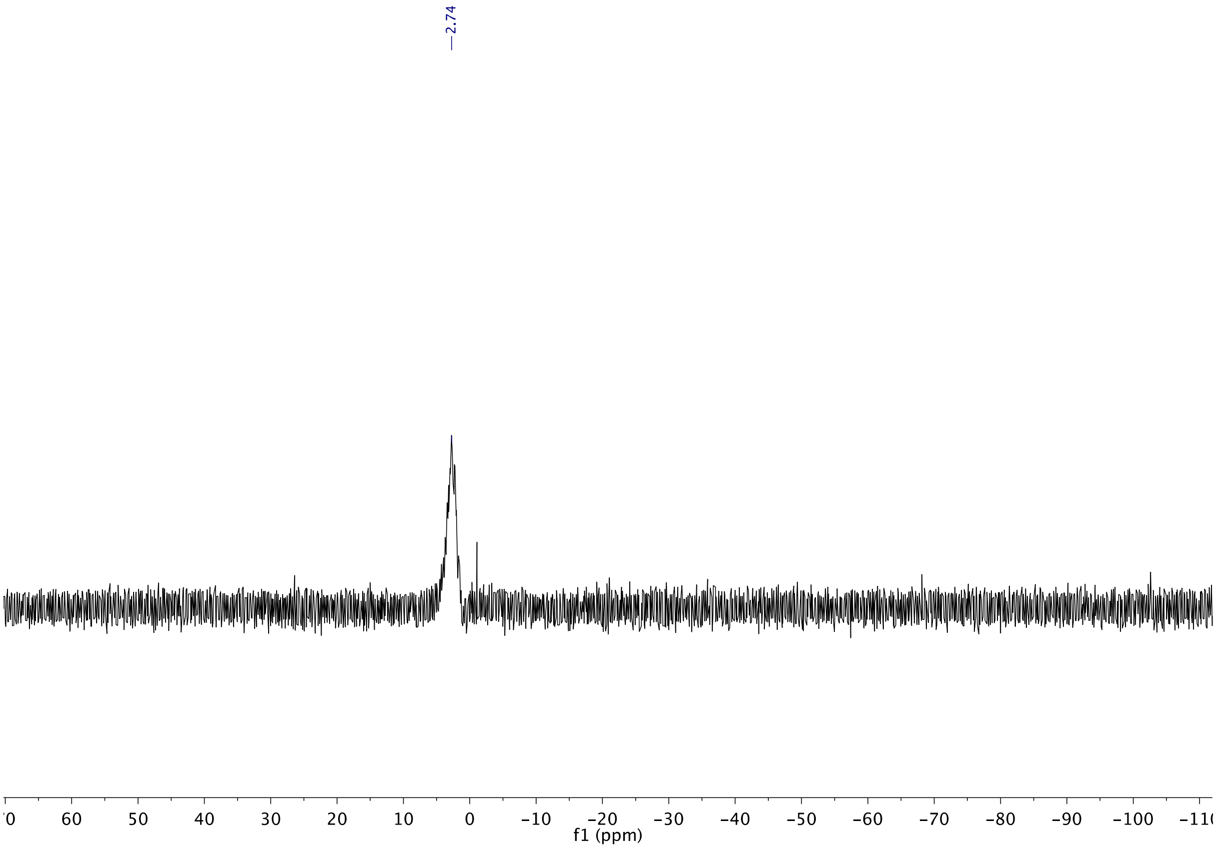

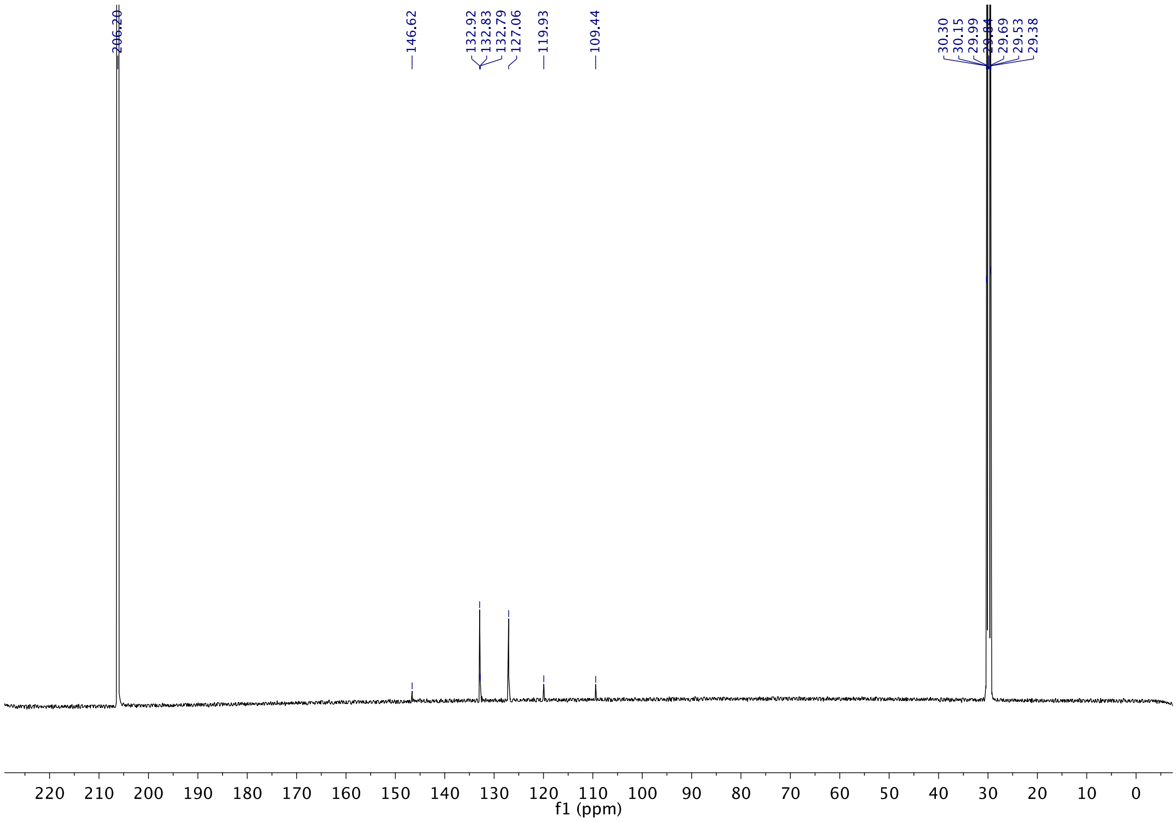
206.20
146.62
132.92
132.83
132.79
127.06
119.93
109.44
30.30
30.15
29.99
29.84
29.69
29.53
29.38
220 210 200 190 180 170 160 150 140 130 120 110 100 90 80 70 60 50 40 30 20 10 0
f1 (ppm)

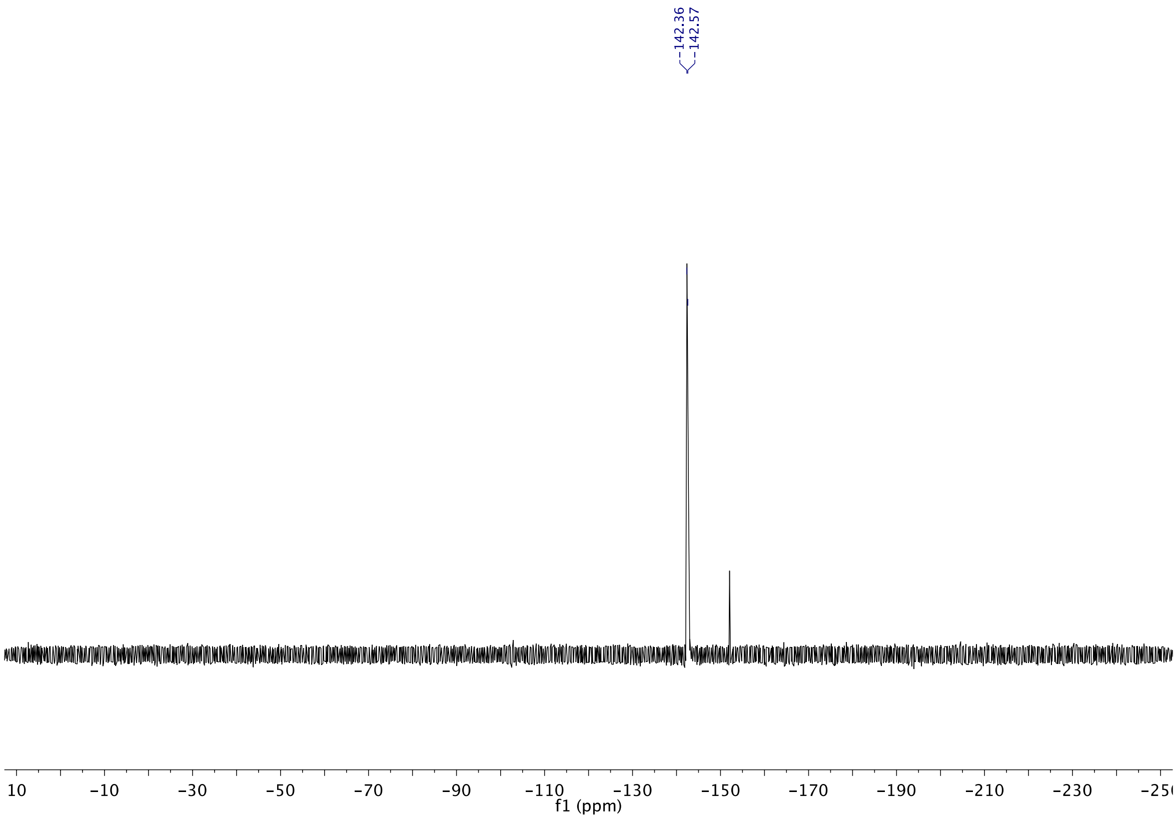
-142.36
-142.57
10 −10 −30 −50 −70 −90 −110 −130 −150 −170 −190 −210 −230
f1 (ppm)

(*E*)-(4-Cyanostyryl)boronic acid, **S1**

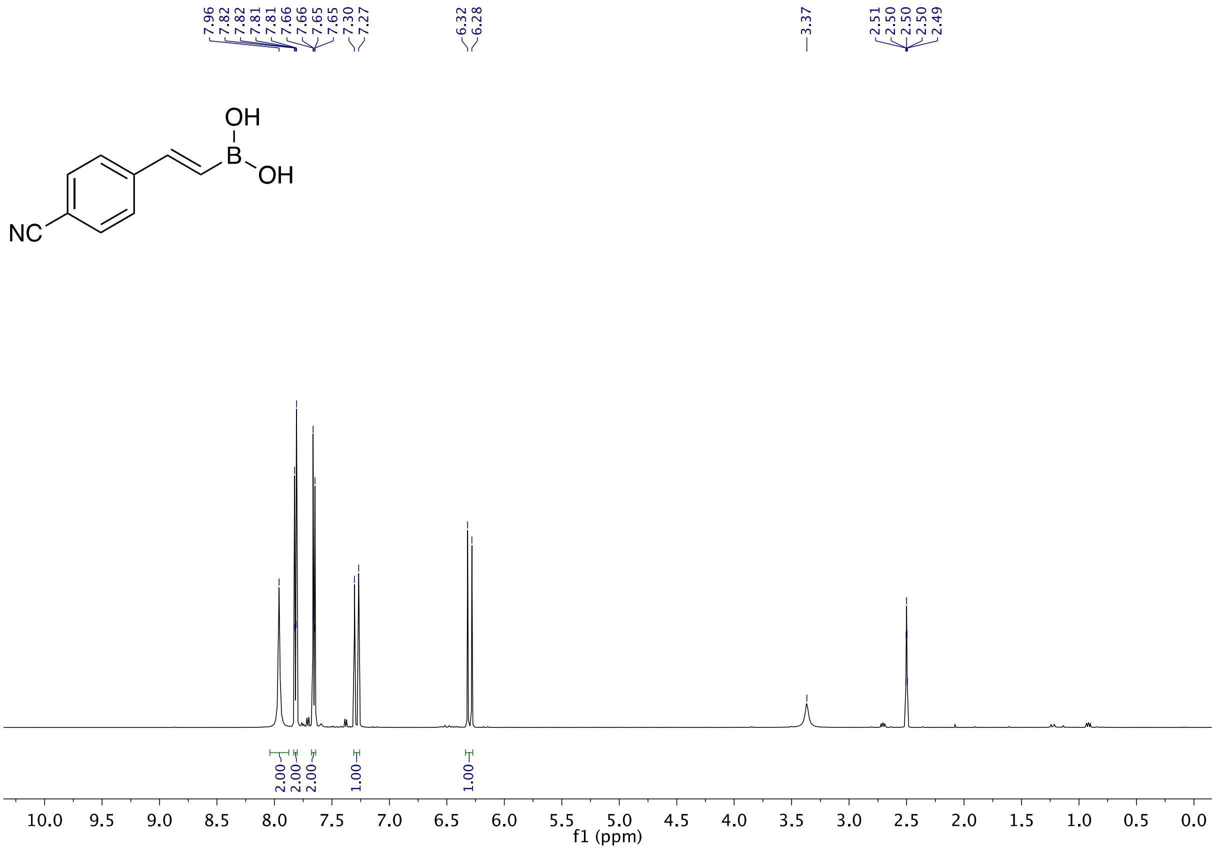

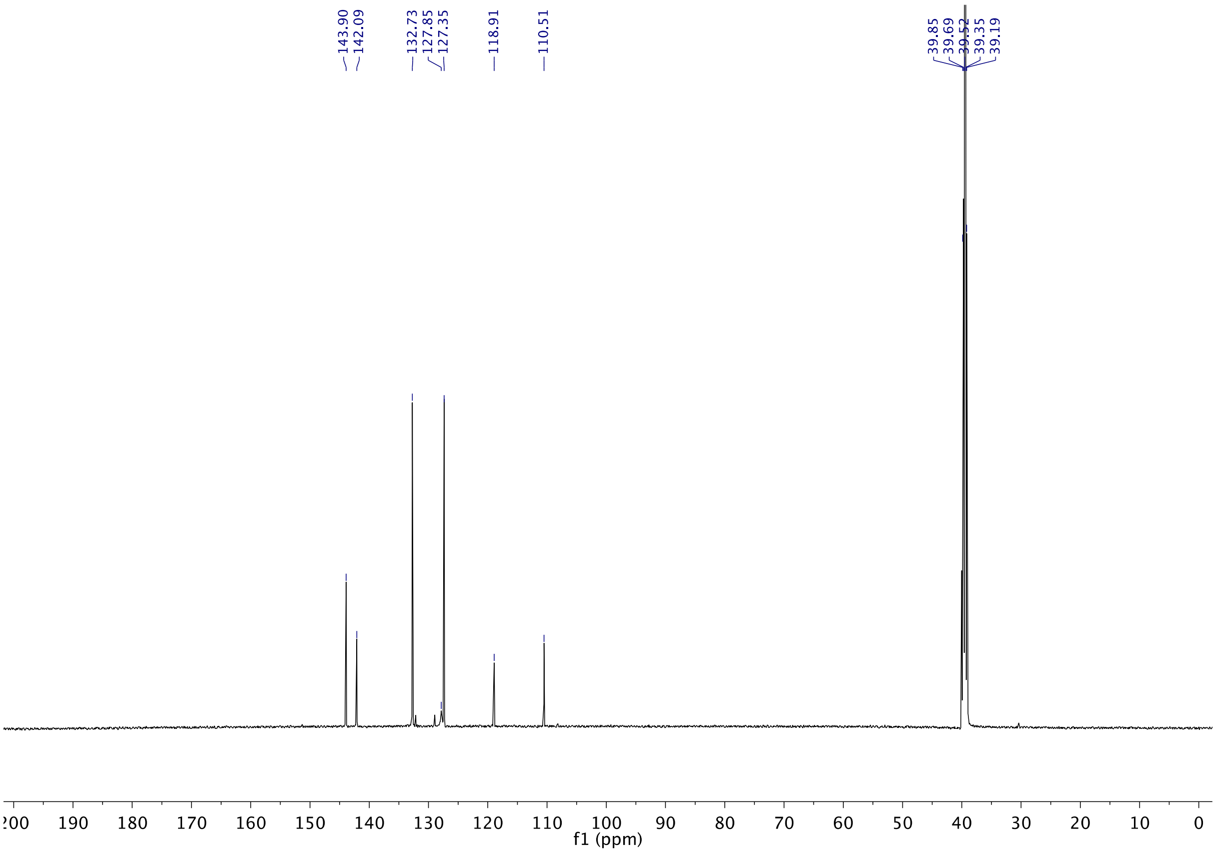

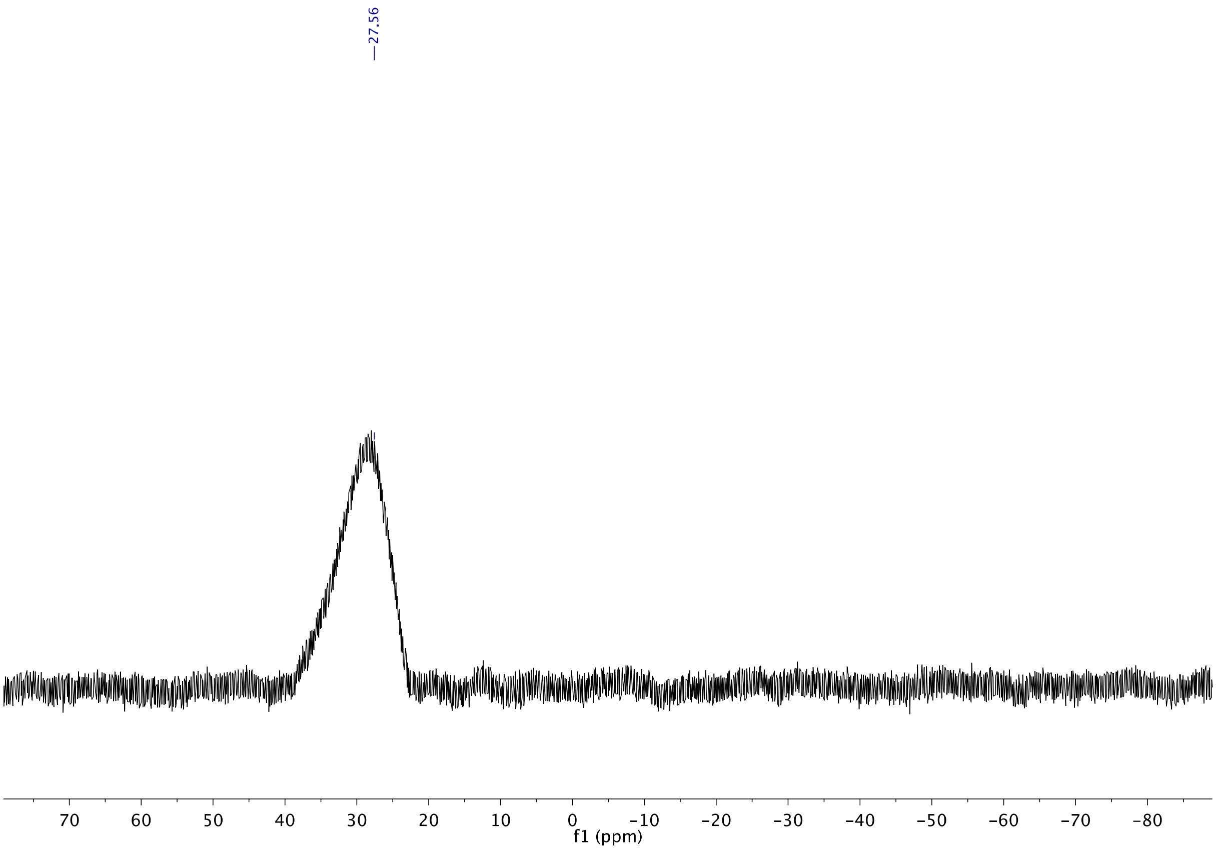

(*E*)-2-(3-Methoxystyryl)-4,4,5,5-tetramethyl-1,3,2-dioxaborolane, **S2-int3**

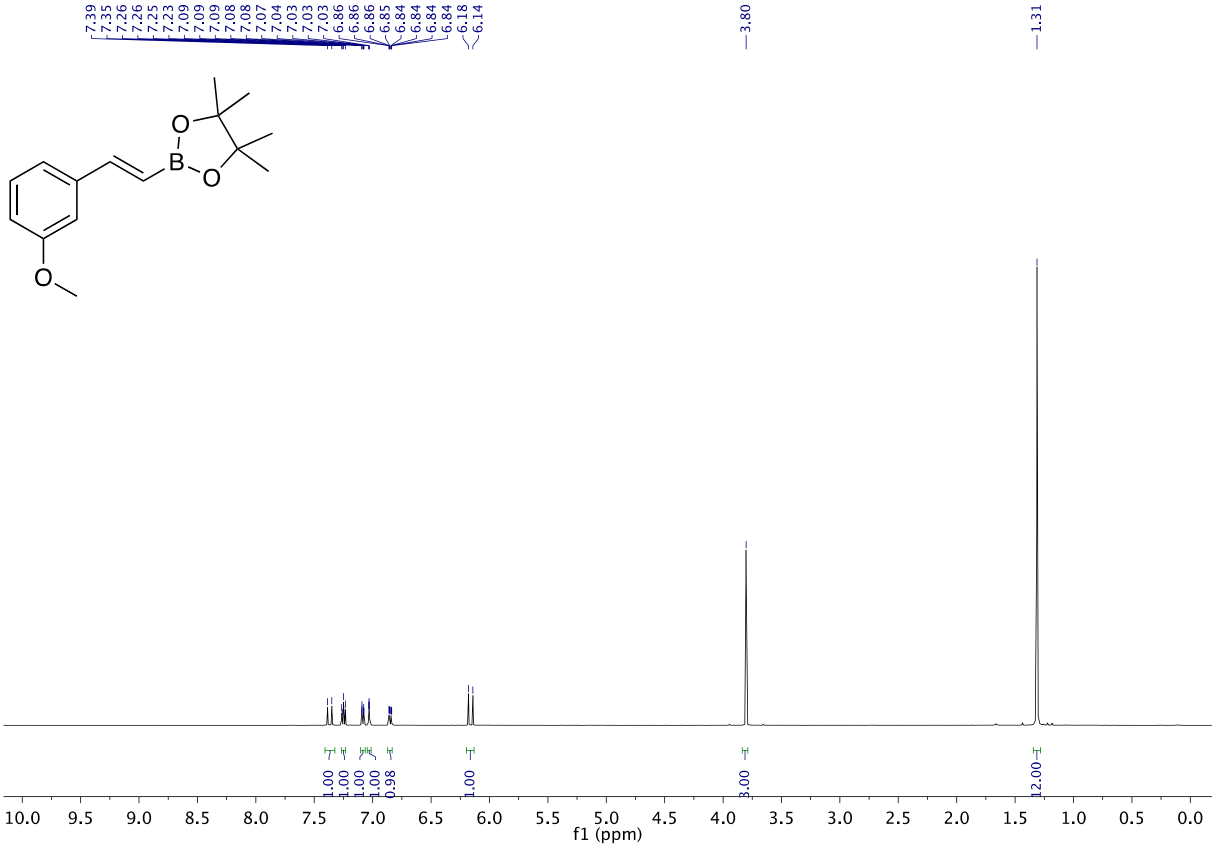

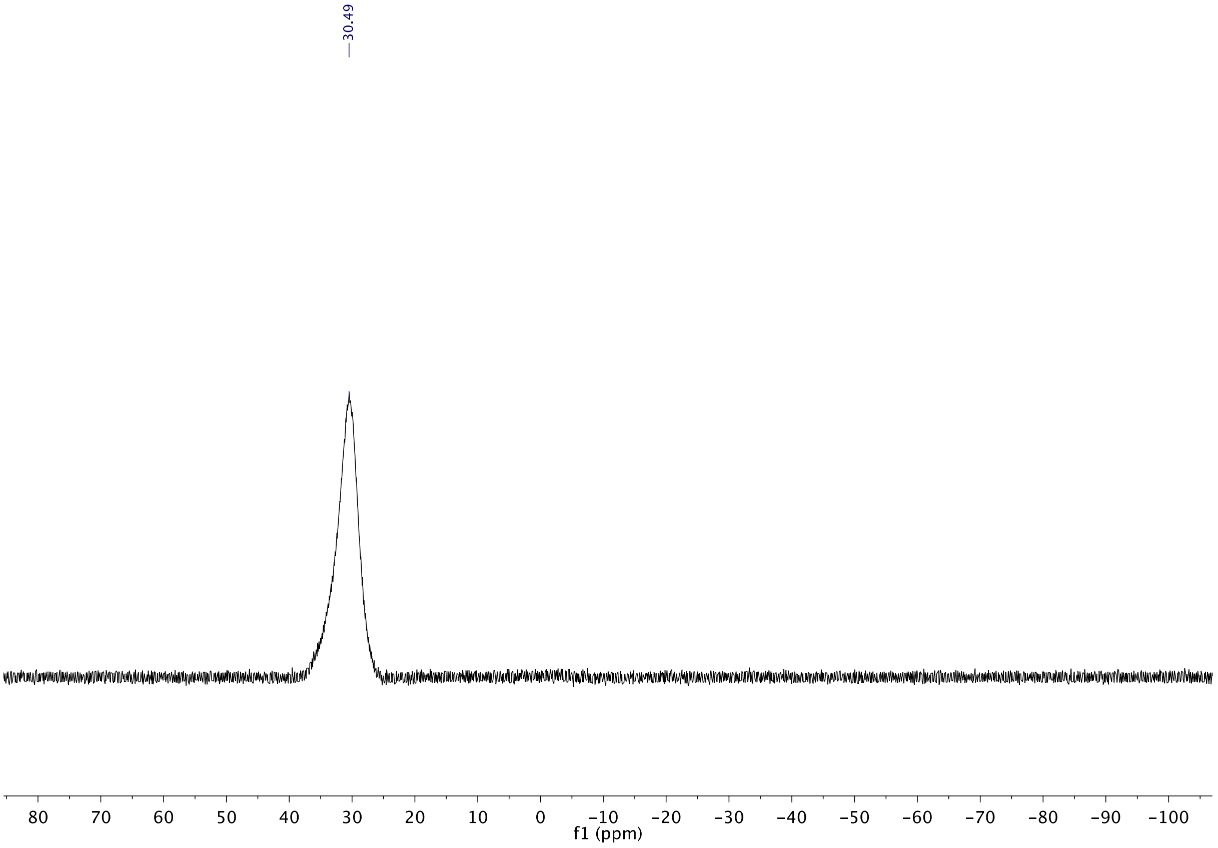
30.49
80 70 60 50 40 30 20 10 0 −10 −20 −30 −40 −50 −60 −70 −80 −90 −100
f1 (ppm)

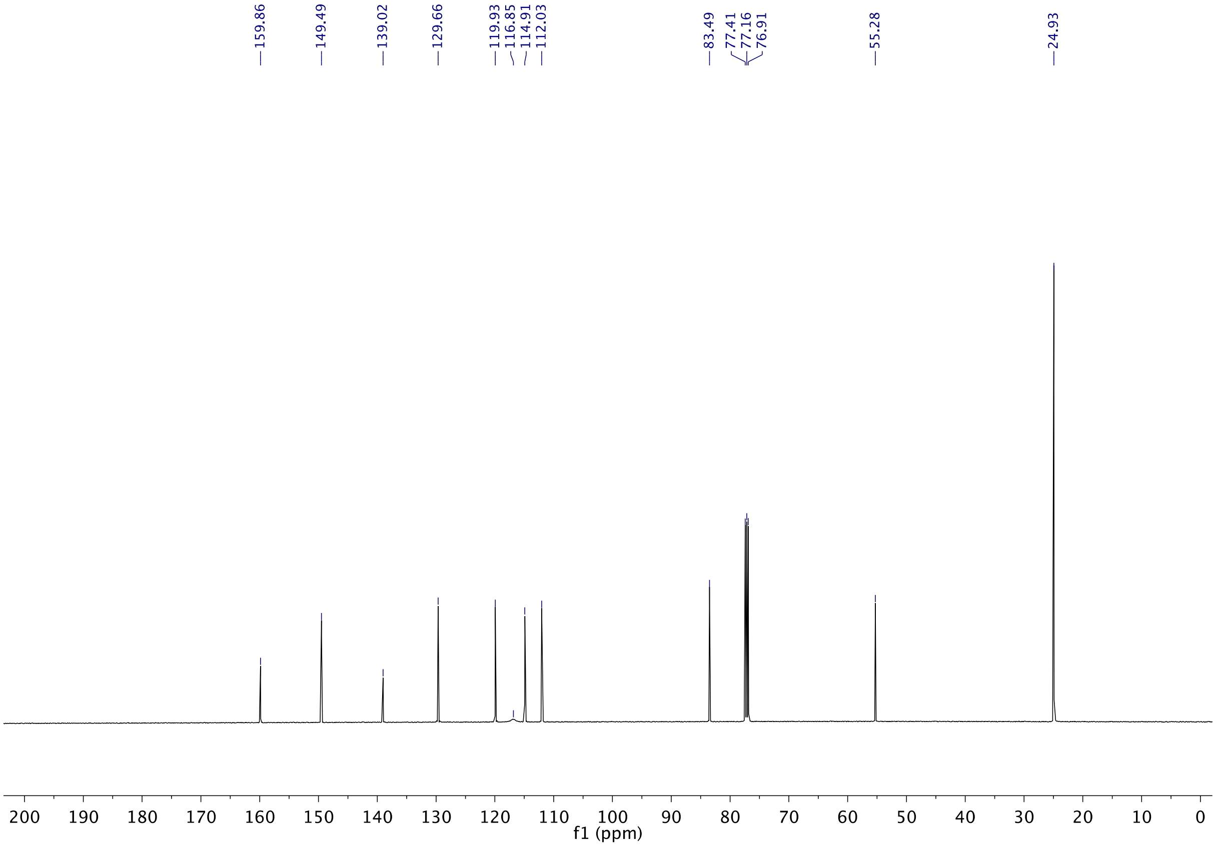
159.86
149.49
139.02
129.66
119.93
116.85
114.91
112.03
83.49
77.41
77.16
76.91
55.28
24.93
200 190 180 170 160 150 140 130 120 110 100 90 80 70 60 50 40 30 20 10 0
f1 (ppm)

(*E*)-Trifluoro(3-methoxystyryl)-λ^4-borane, potassium salt, **S2-int4**

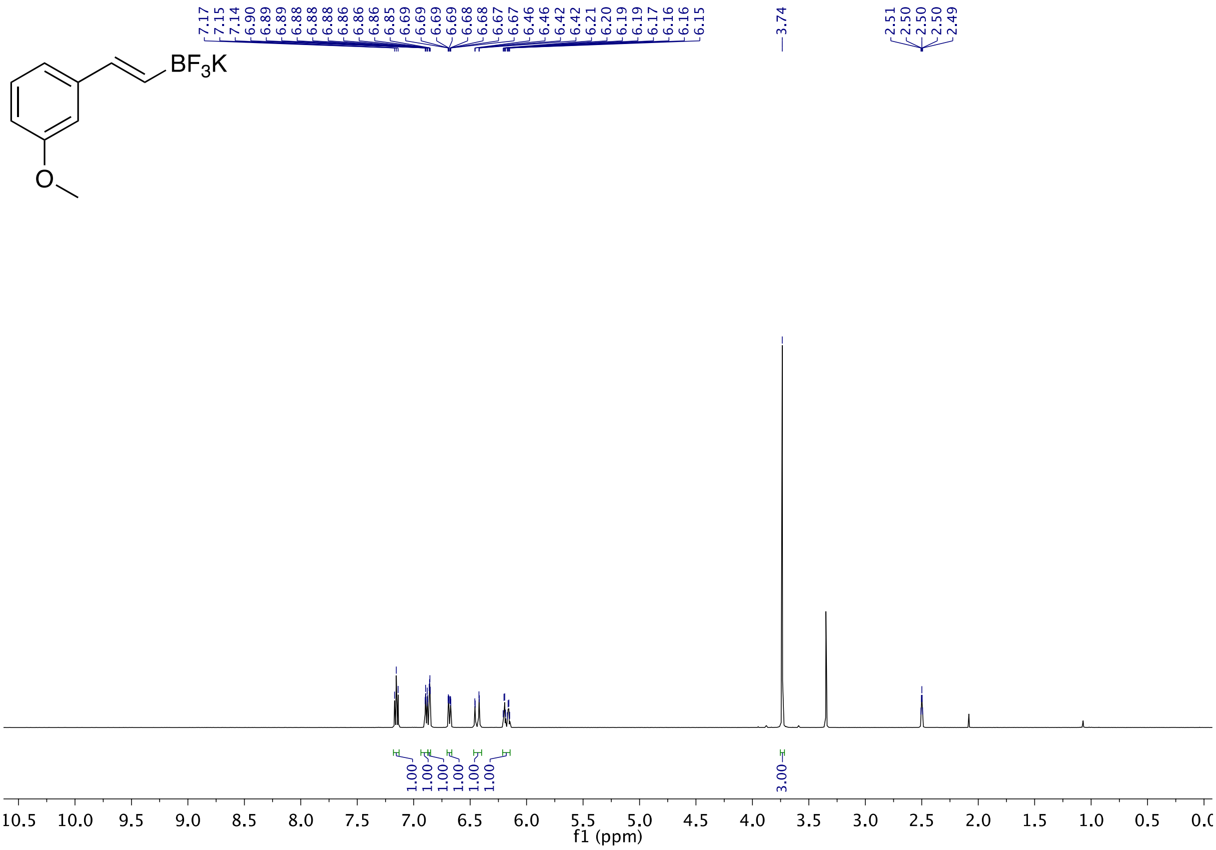

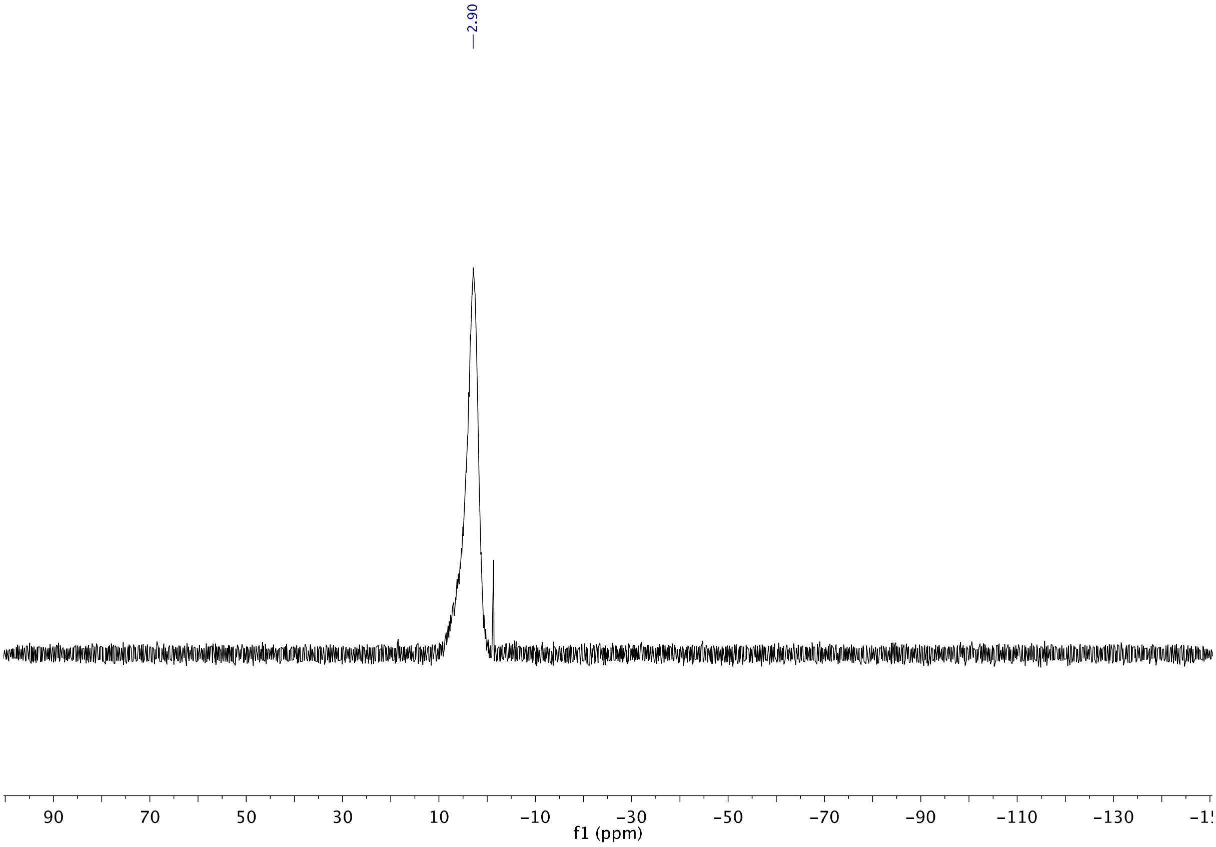

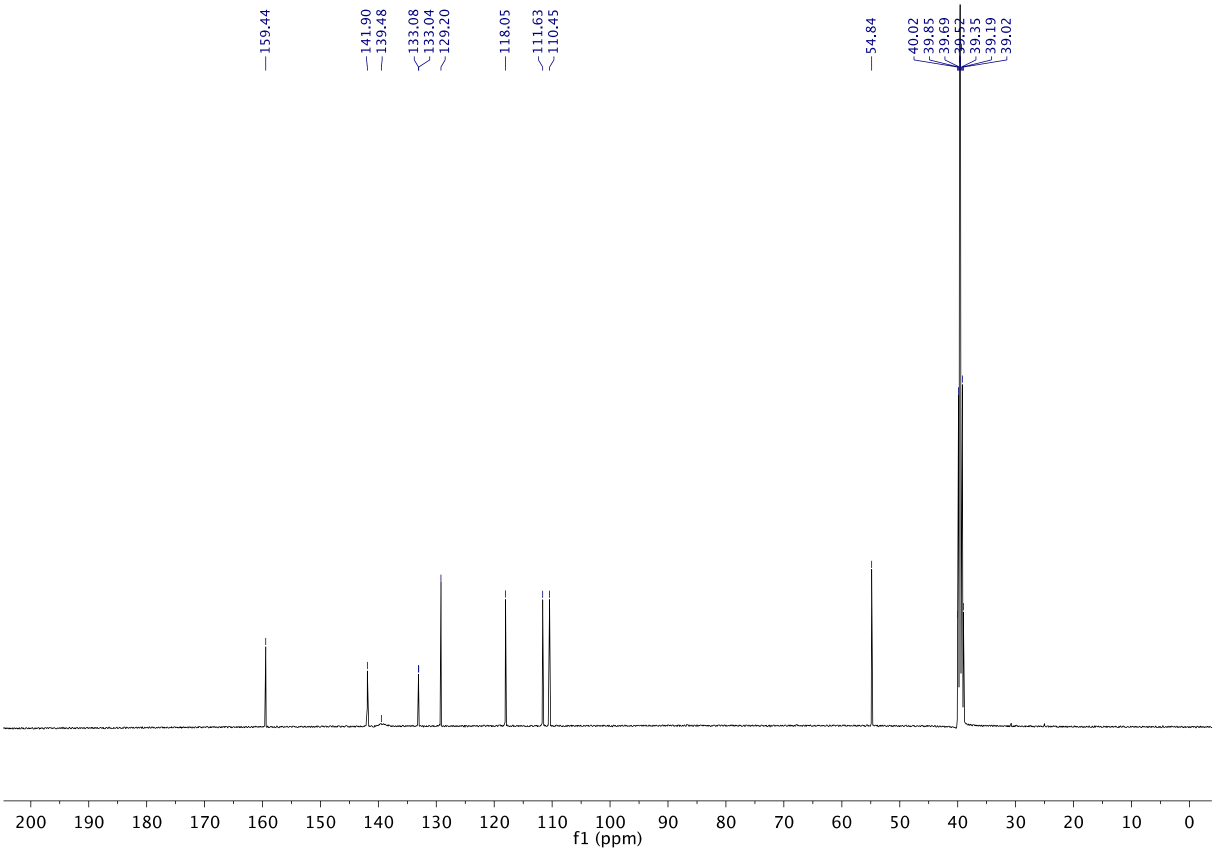
159.44
141.90
139.48
133.08
133.04
129.20
118.05
111.63
110.45
54.84
40.02
39.85
39.69
39.52
39.35
39.19
39.02
200 190 180 170 160 150 140 130 120 110 100 90 80 70 60 50 40 30 20 10 0
f1 (ppm)

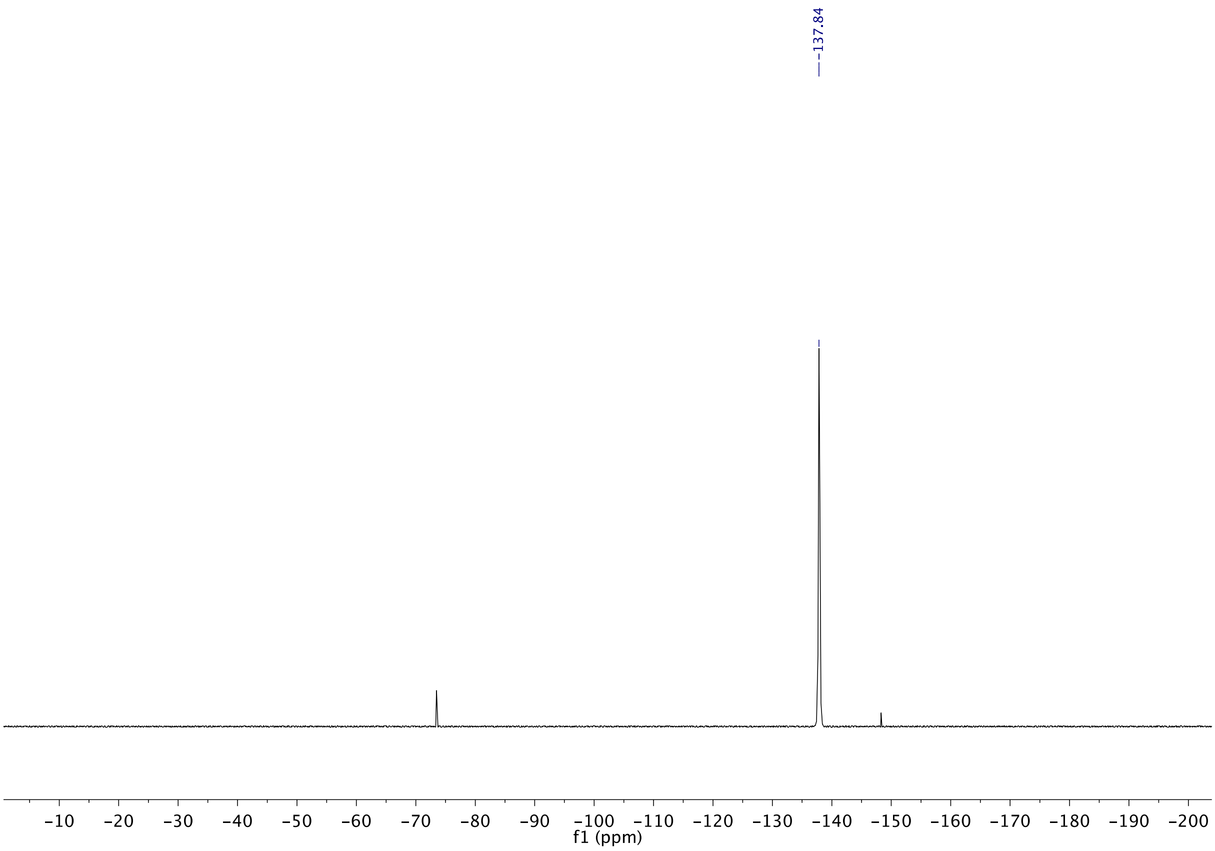
-137.84
-10 -20 -30 -40 -50 -60 -70 -80 -90 -100 -110 -120 -130 -140 -150 -160 -170 -180 -190 -200
f1 (ppm)

(*E*)-(3-Methoxystyryl)boronic acid, **S2**

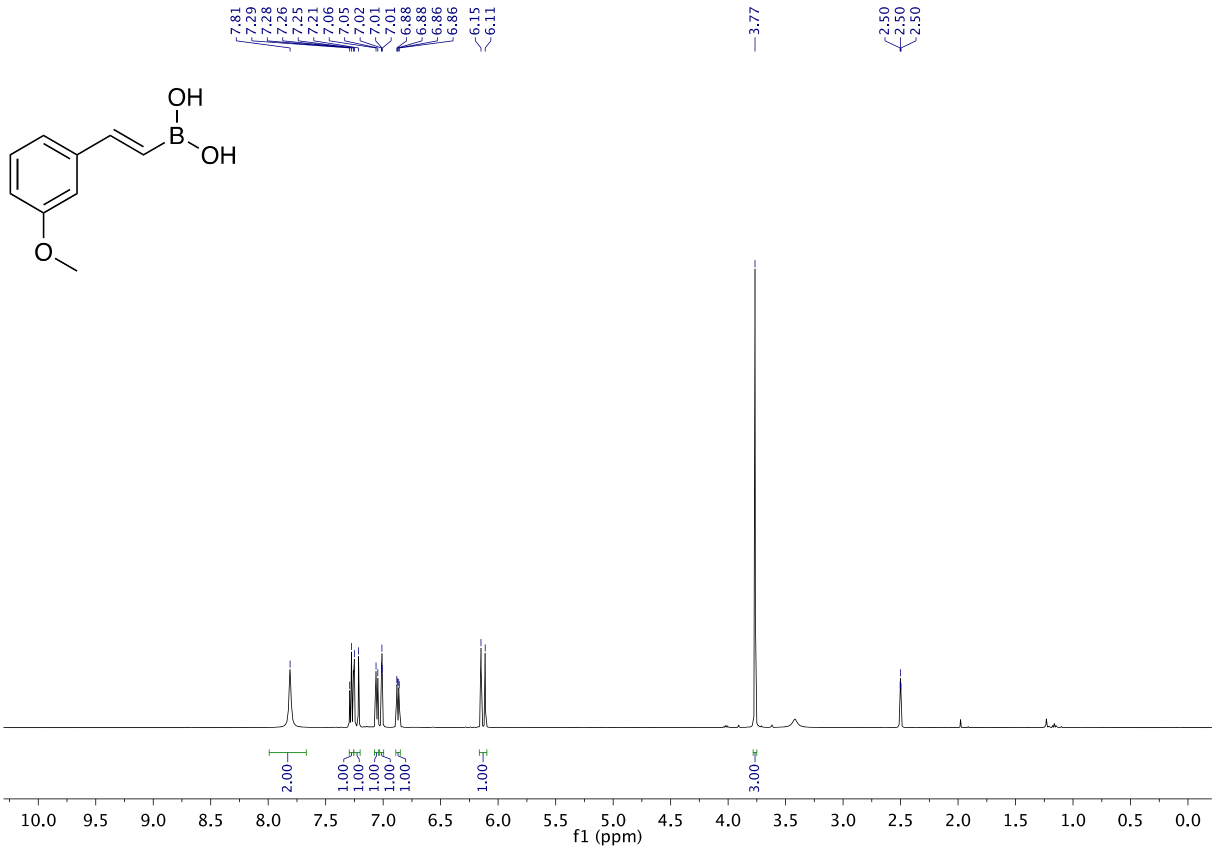

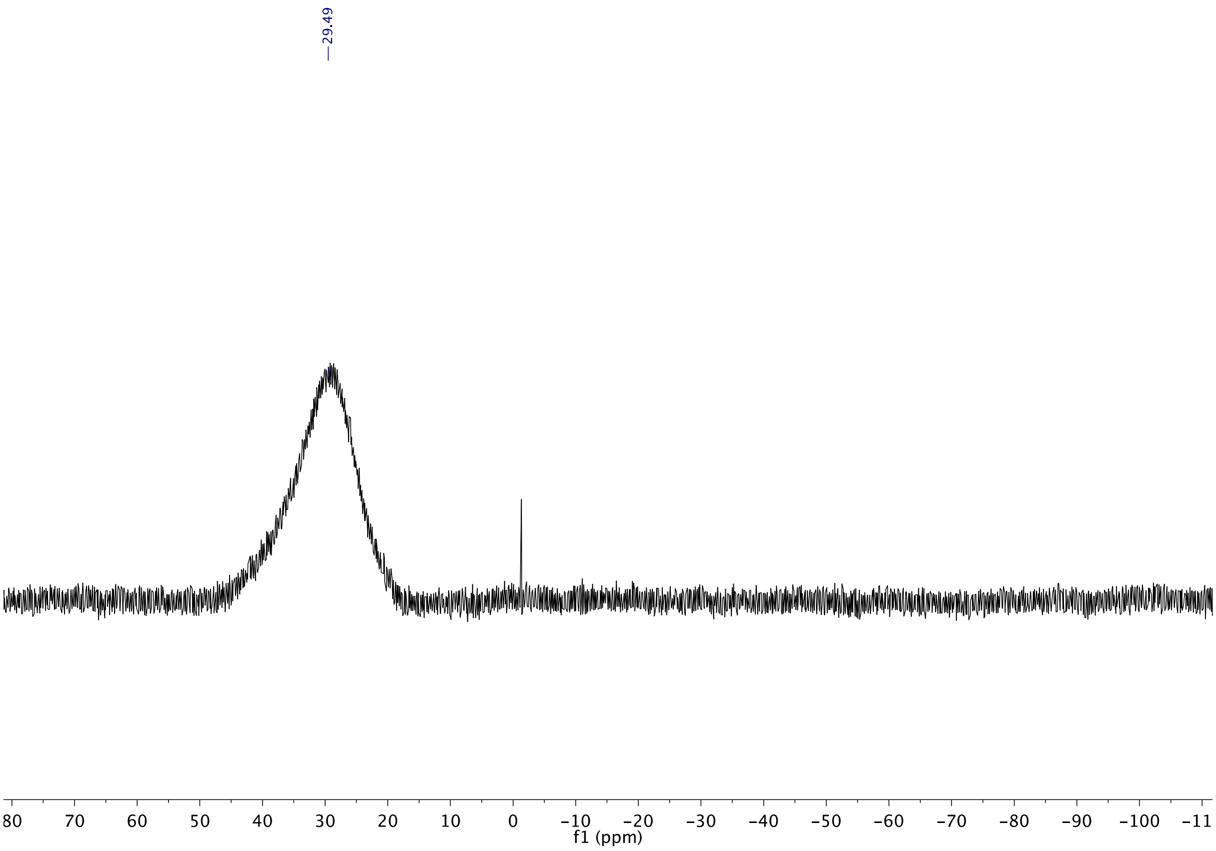

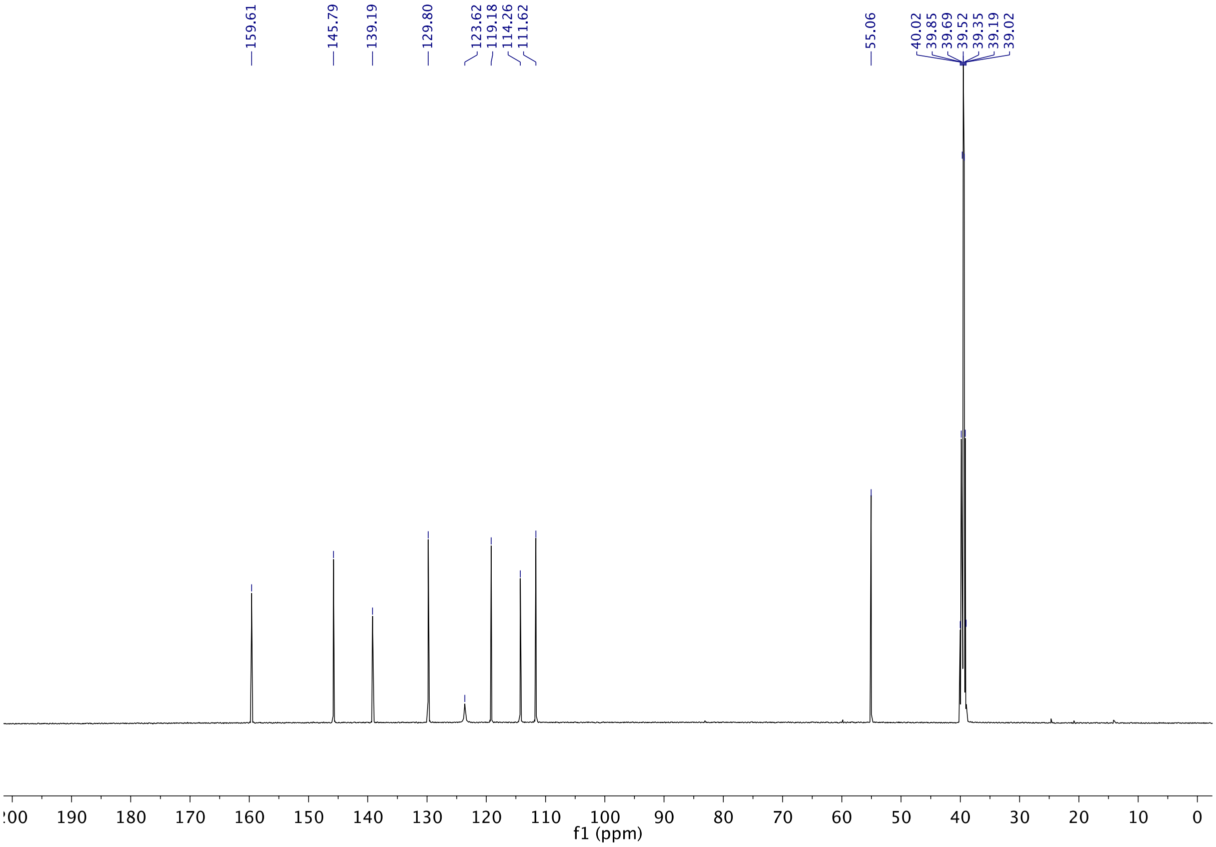

N-(3-iodophenyl)acetamide, **S3-int0**

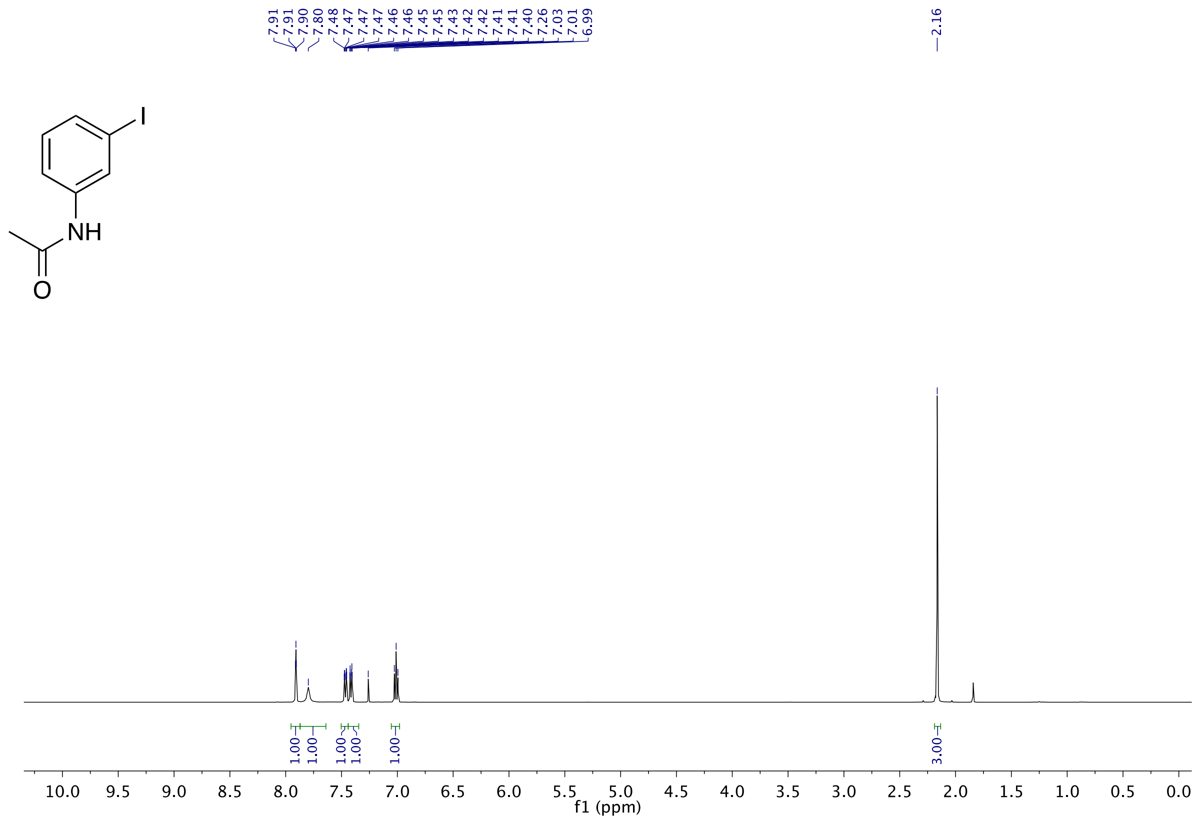

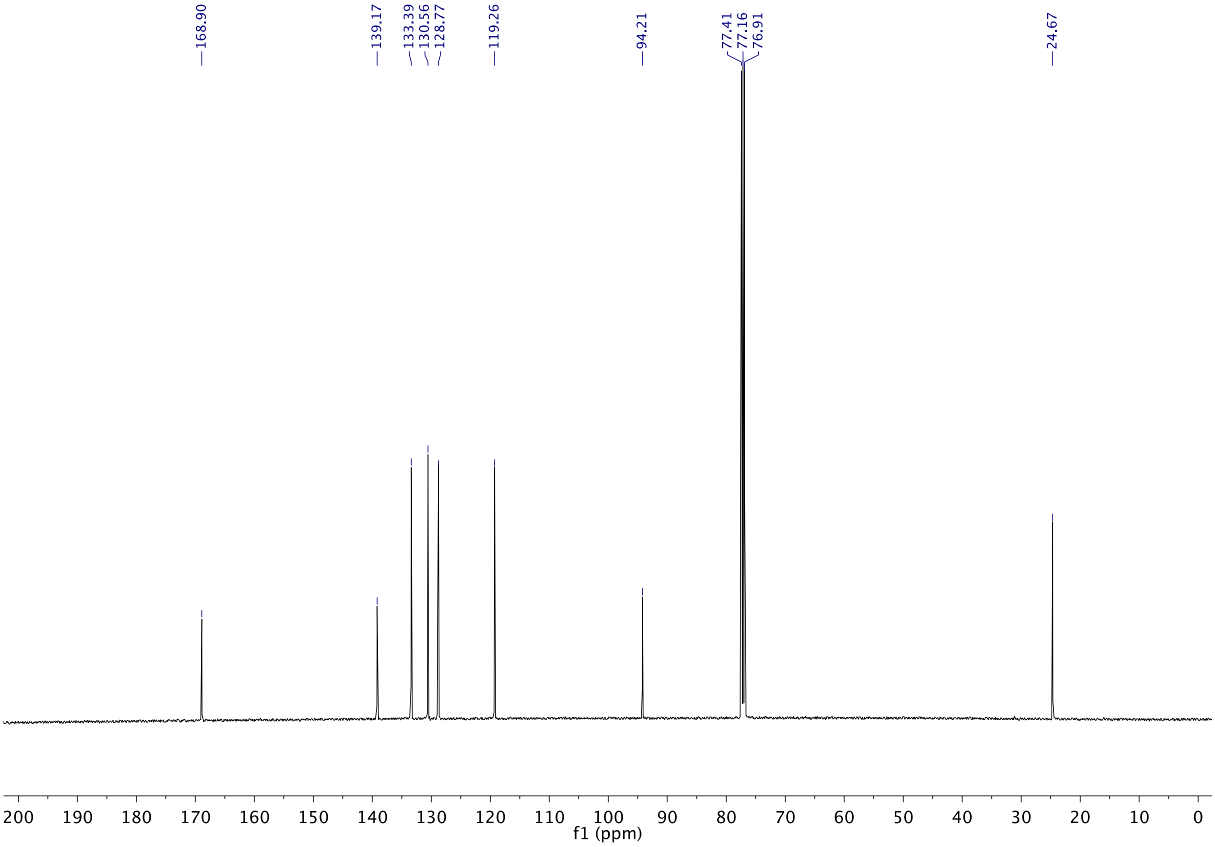

N-(3-((Trimethylsilyl)ethynyl)phenyl)acetamide, **S3-int1**

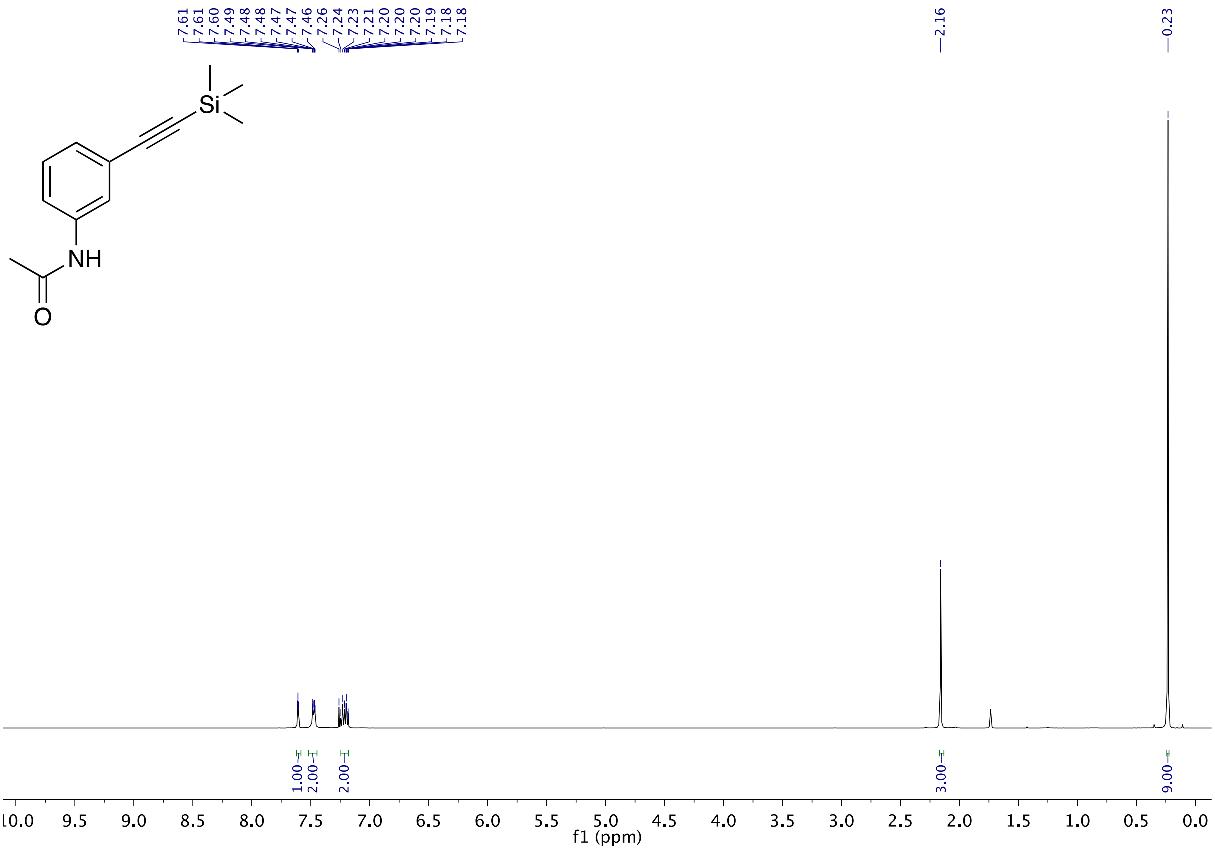

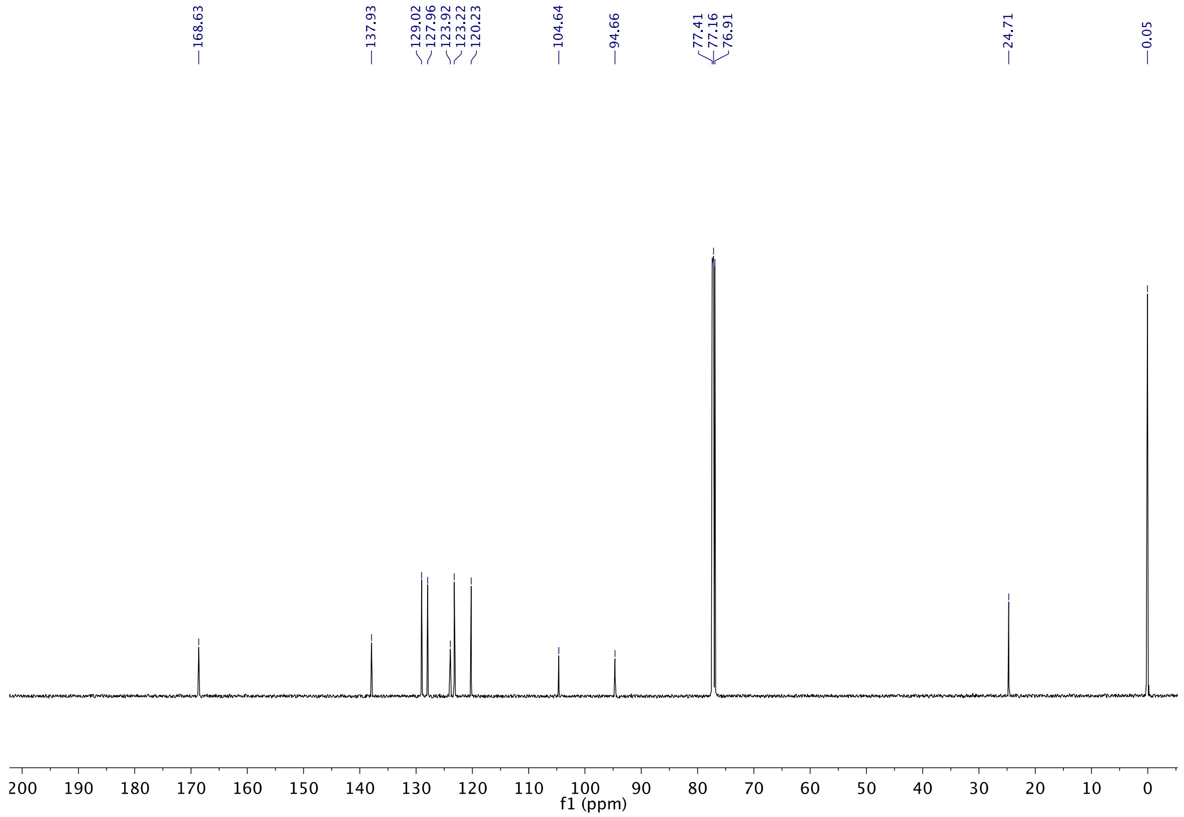

N-(3-Ethynylphenyl)acetamide, **S3-int2**

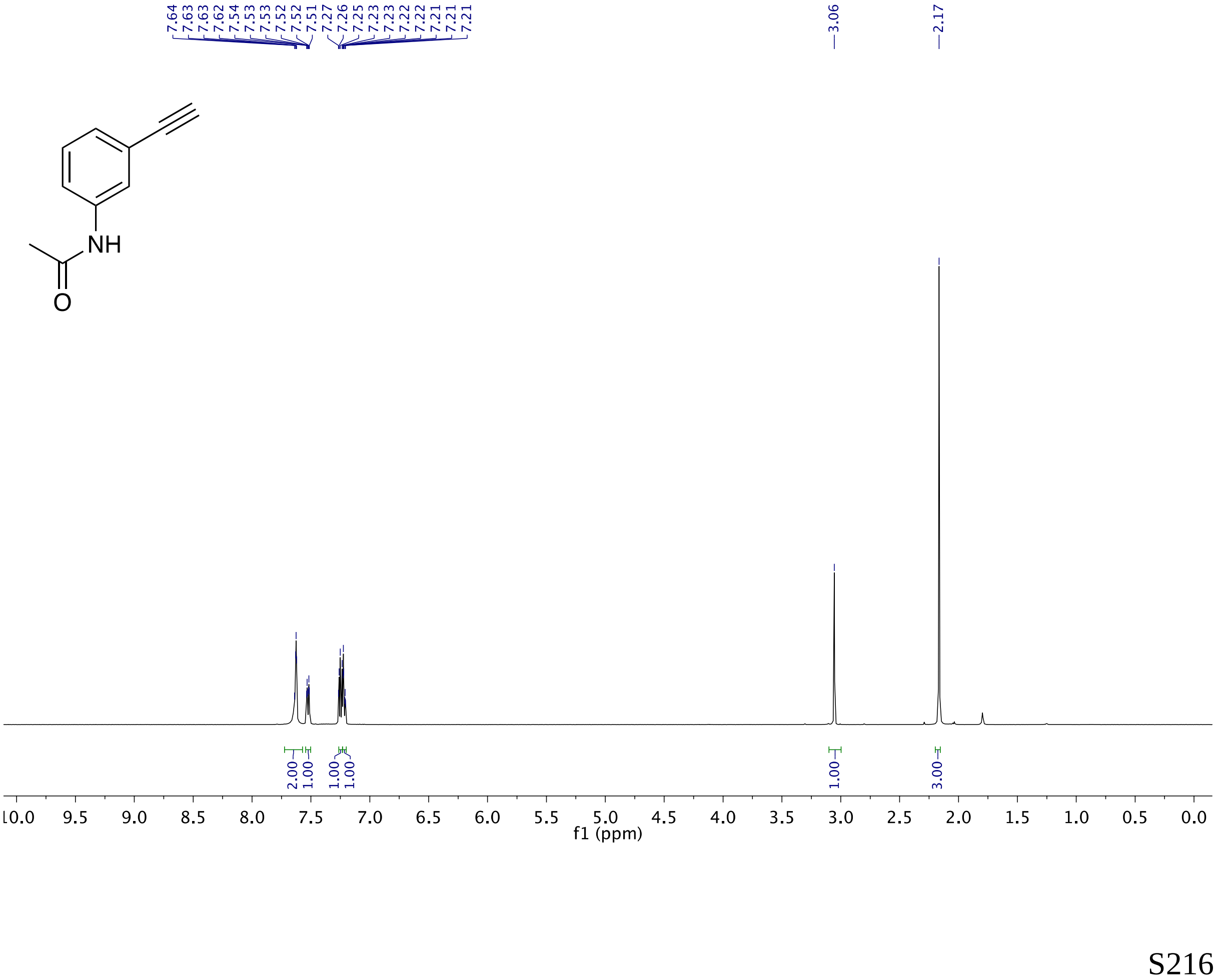

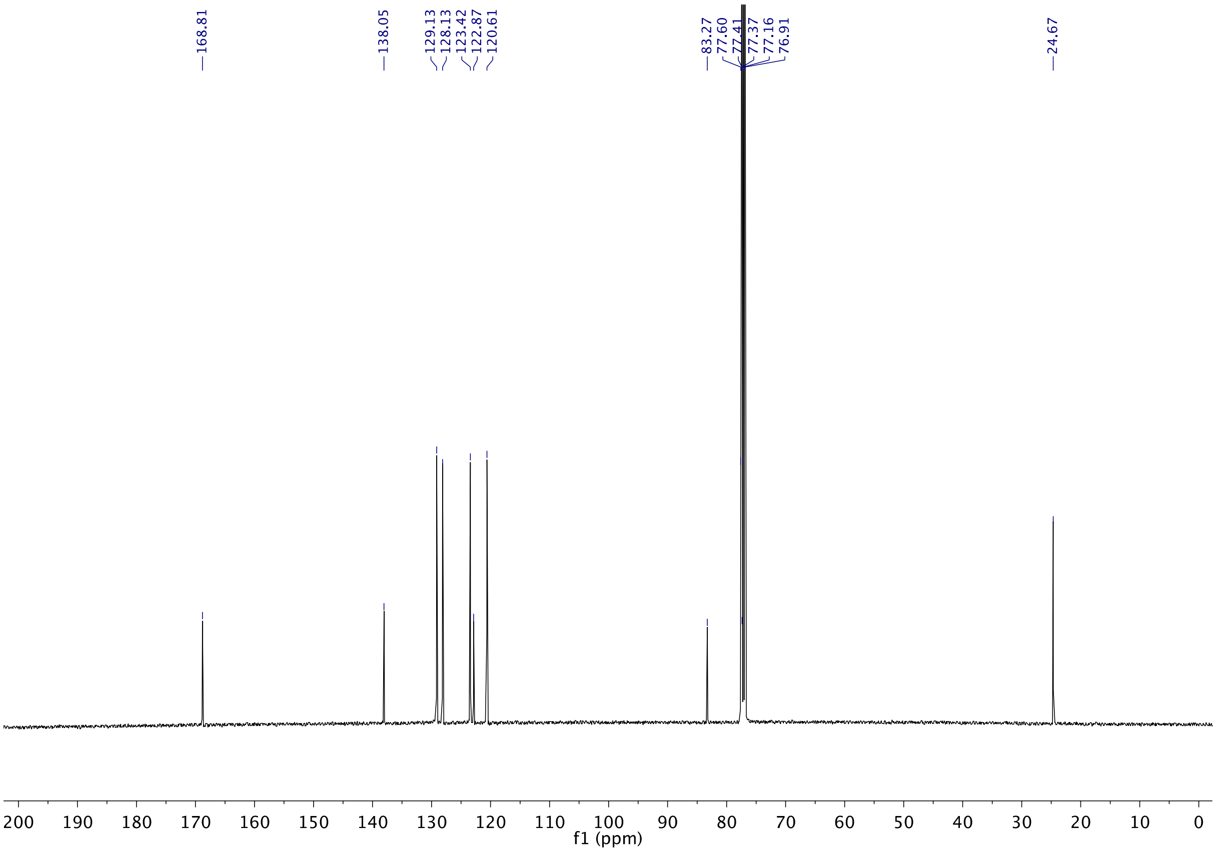

(*E*)-*N*-(3-(2-(4,4,5,5-Tetramethyl-1,3,2-dioxaborolan-2-yl)vinyl)phenyl)acetamide, **S3-int3**

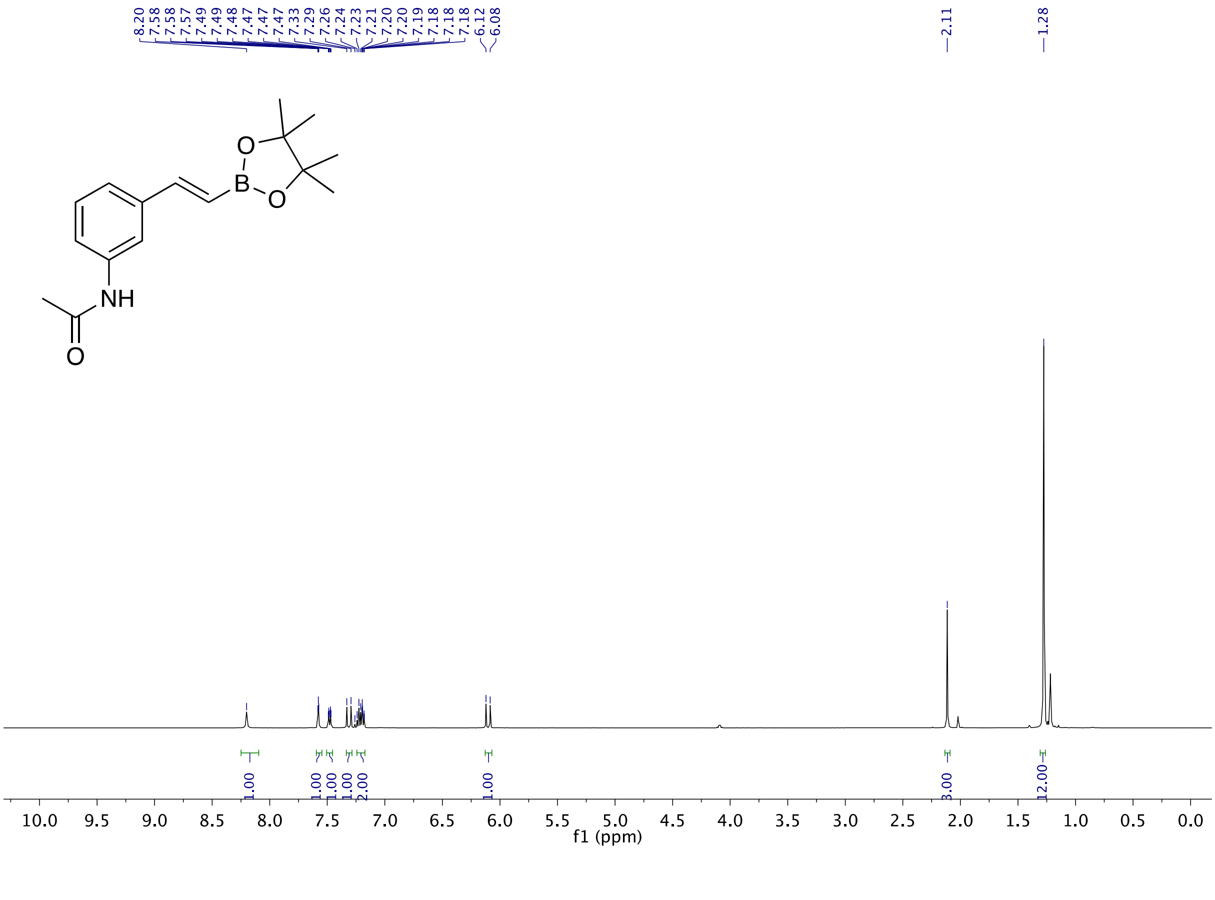

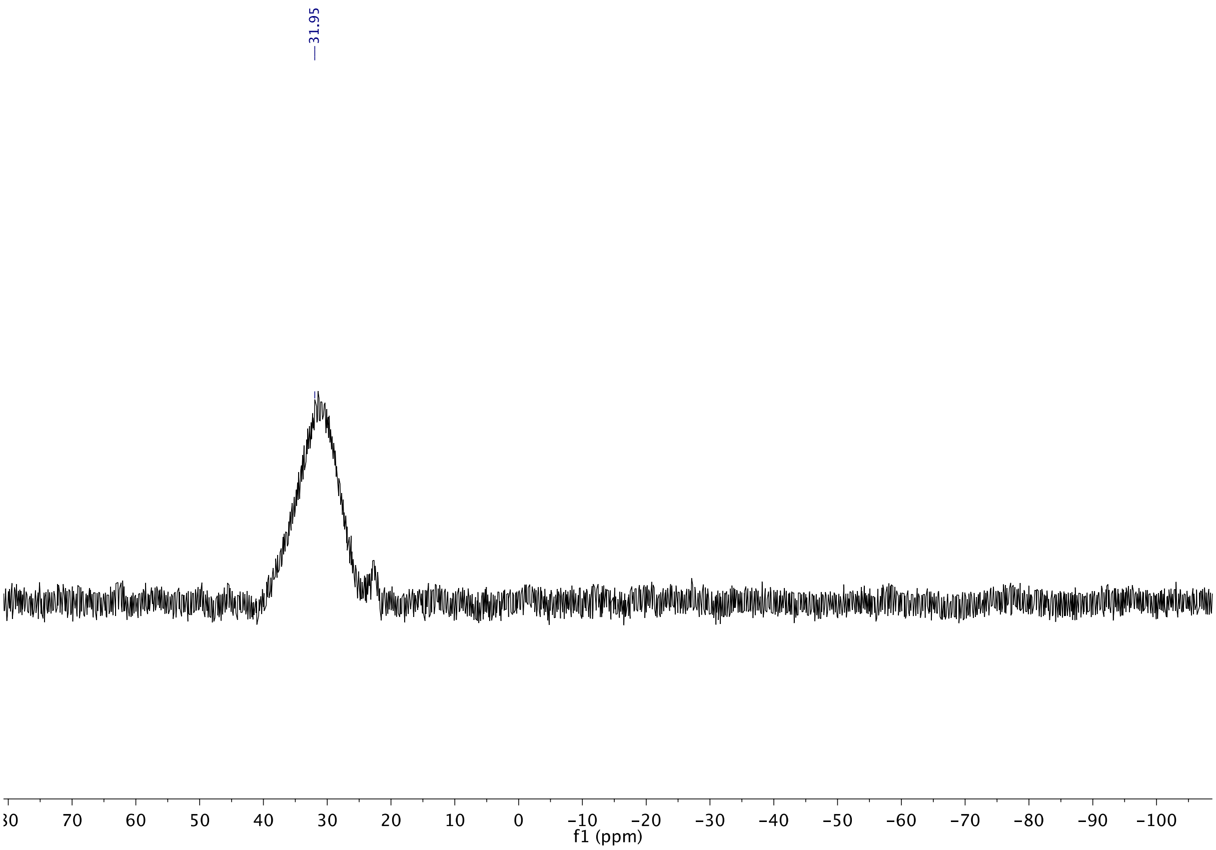
31.95
80
70
60
50
40
30
20
10
0
−10
−20
−30
−40
−50
−60
−70
−80
−90
−100
f1 (ppm)

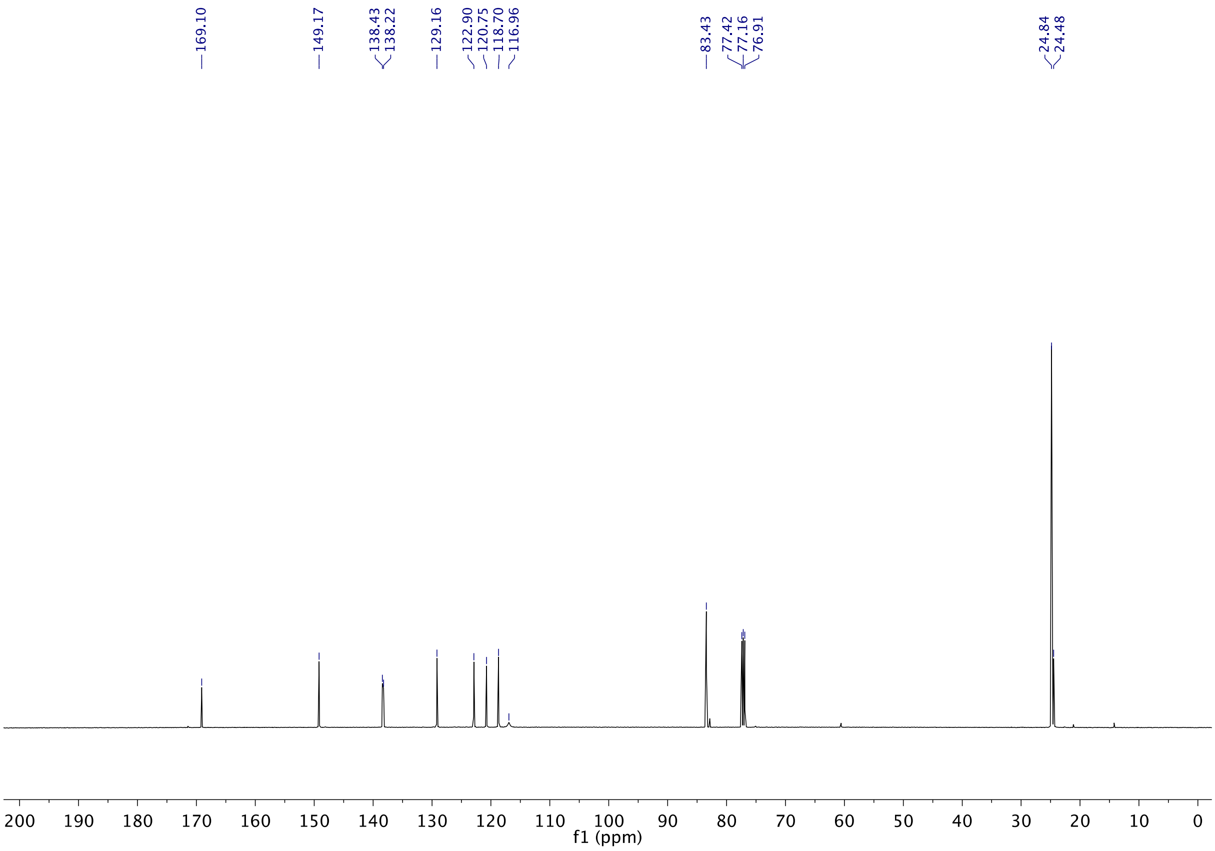
169.10
149.17
138.43
138.22
129.16
122.90
120.75
118.70
116.96
83.43
77.42
77.16
76.91
24.84
24.48
200
190
180
170
160
150
140
130
120
110
100
90
80
70
60
50
40
30
20
10
0
f1 (ppm)

(*E*)-*N*-(3-(2-(Trifluoro-λ[4]-boraneyl)vinyl)phenyl)acetamide, potassium salt, **S3-int4**

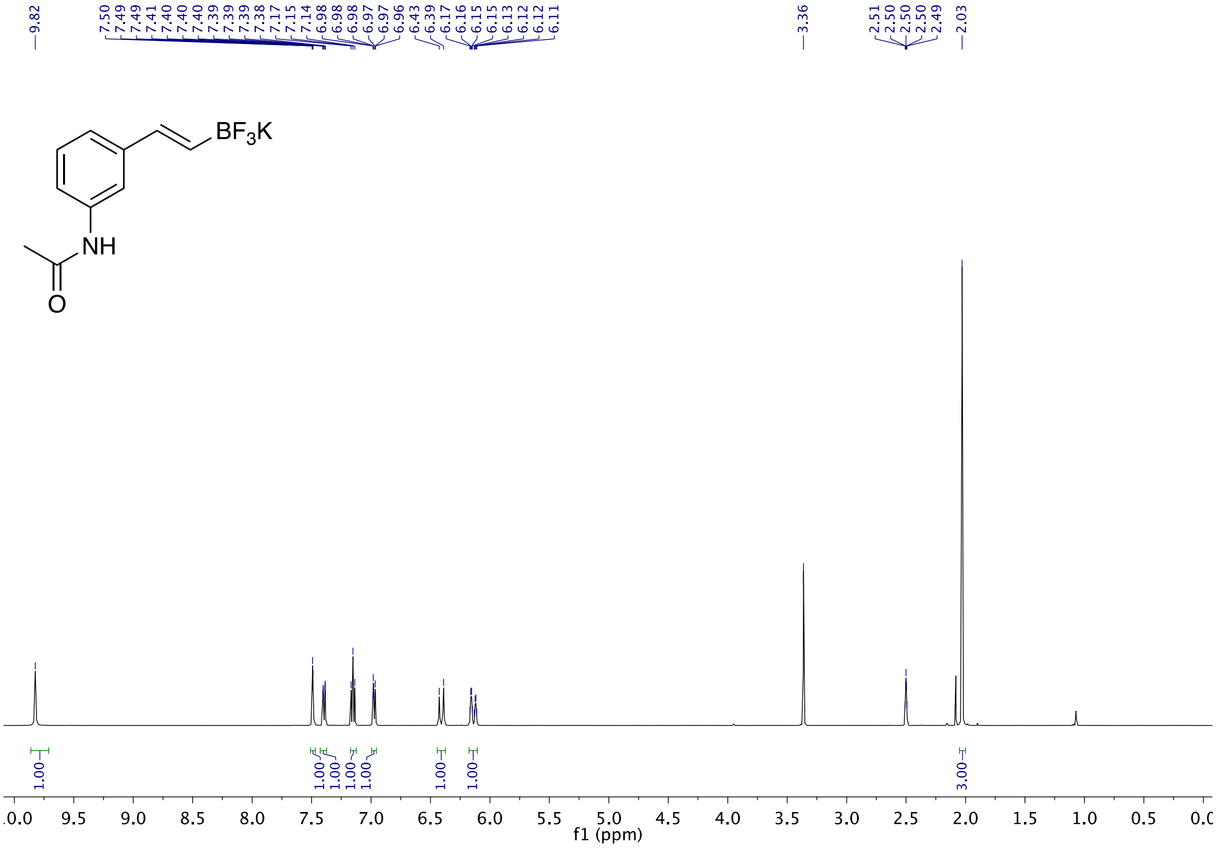

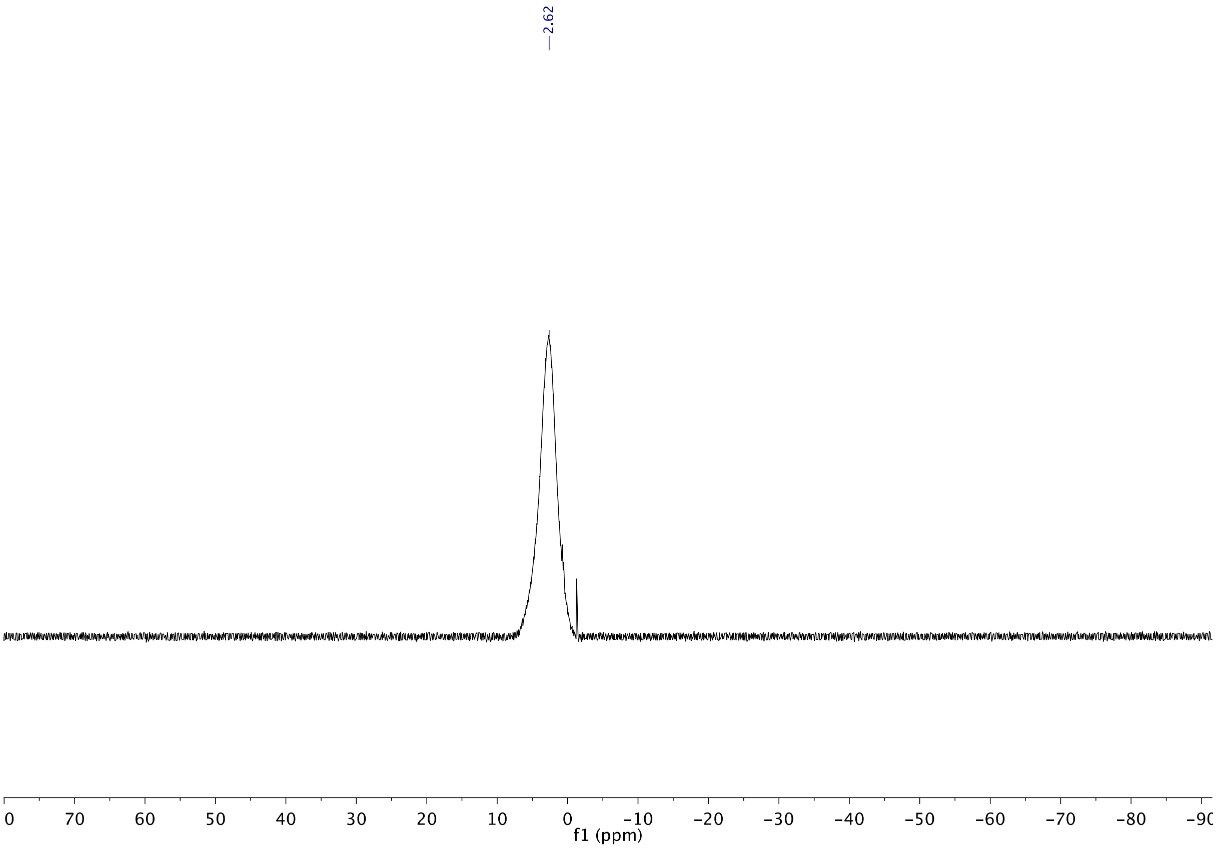

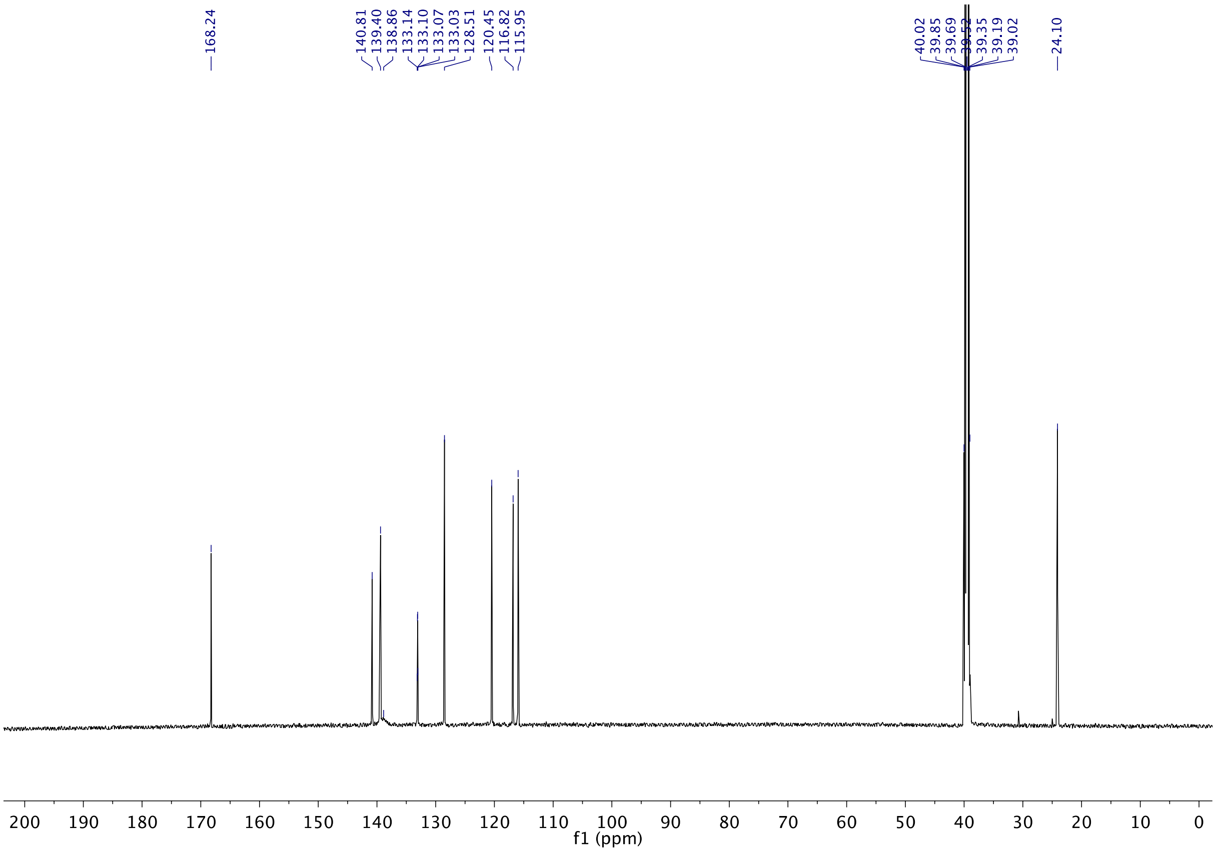

168.24
140.81
139.40
138.86
133.14
133.10
133.07
133.03
128.51
120.45
116.82
115.95
40.02
39.85
39.69
39.52
39.35
39.19
39.02
24.10
200 190 180 170 160 150 140 130 120 110 100 90 80 70 60 50 40 30 20 10 0
f1 (ppm)

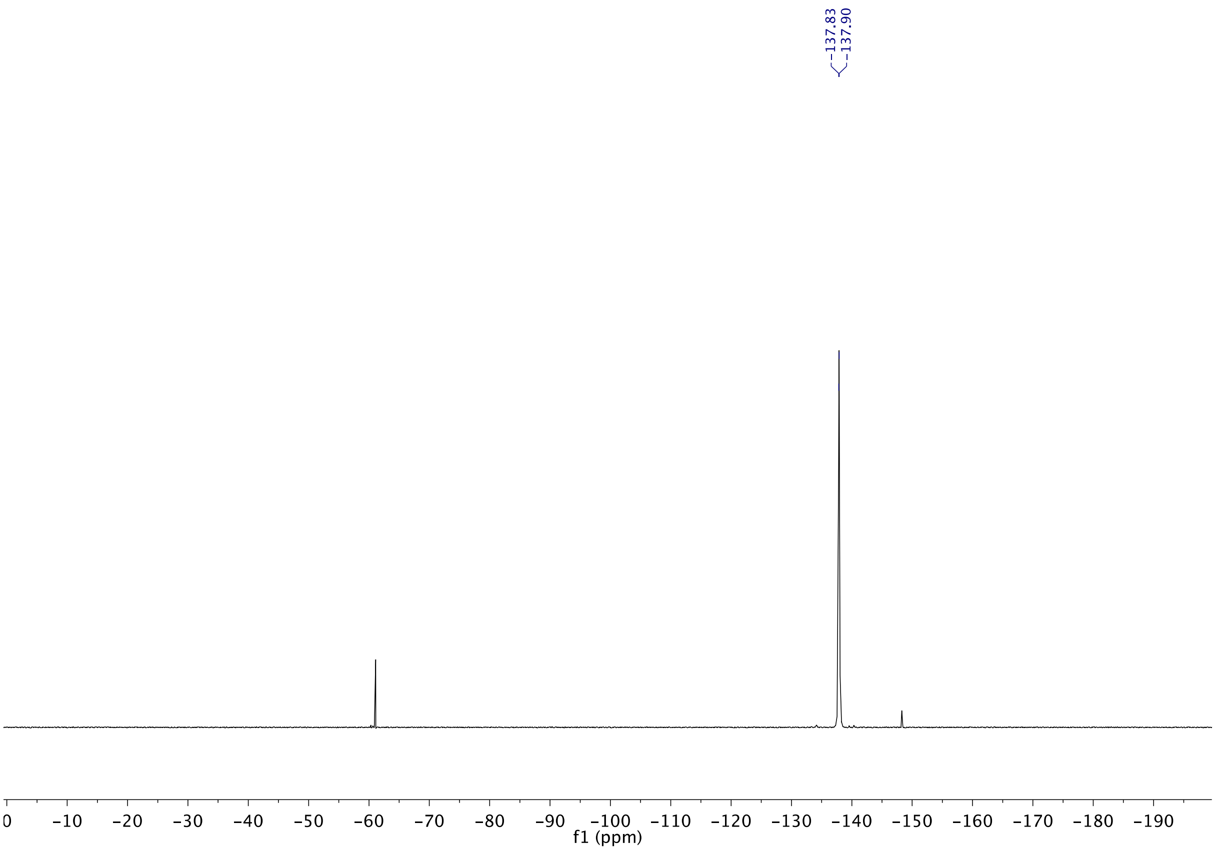

-137.83
-137.90
0 -10 -20 -30 -40 -50 -60 -70 -80 -90 -100 -110 -120 -130 -140 -150 -160 -170 -180 -190
f1 (ppm)

(*E*)-(3-Acetamidostyryl)boronic acid, **S3**

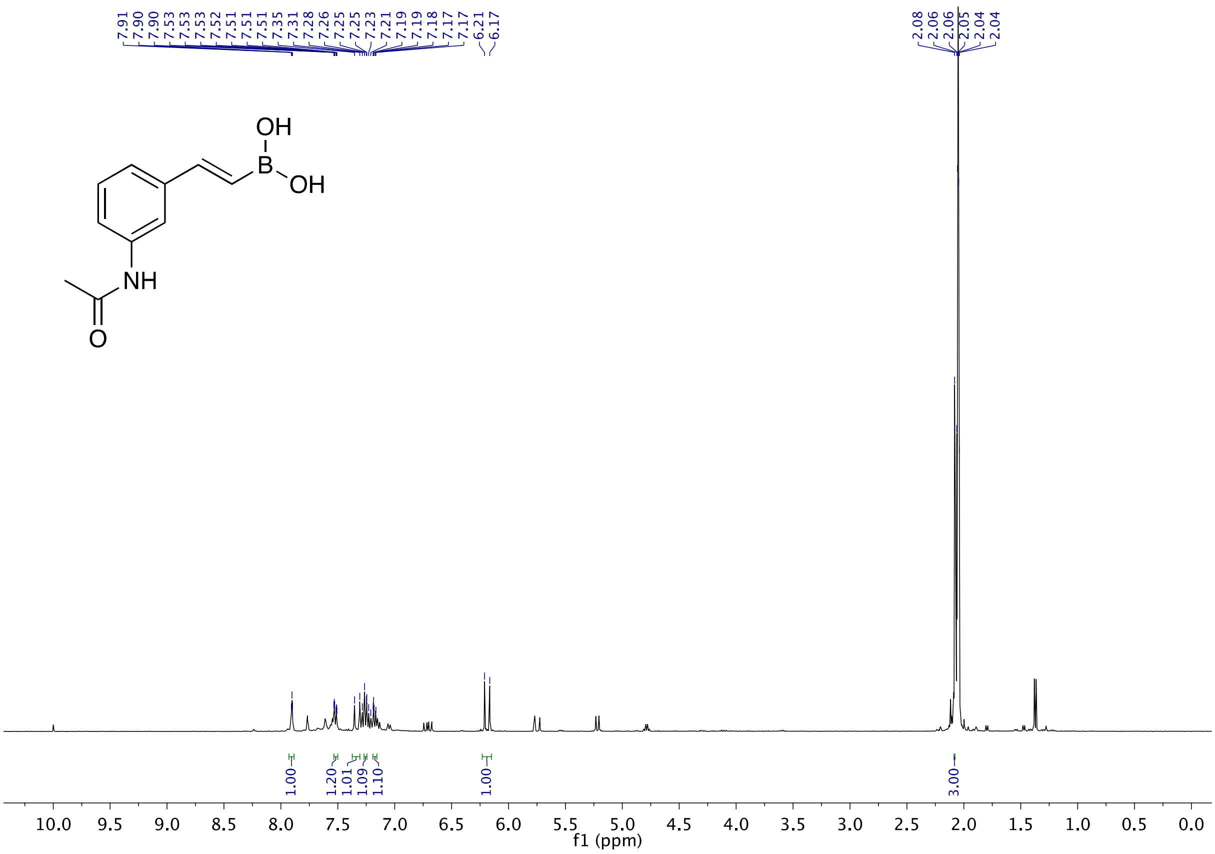

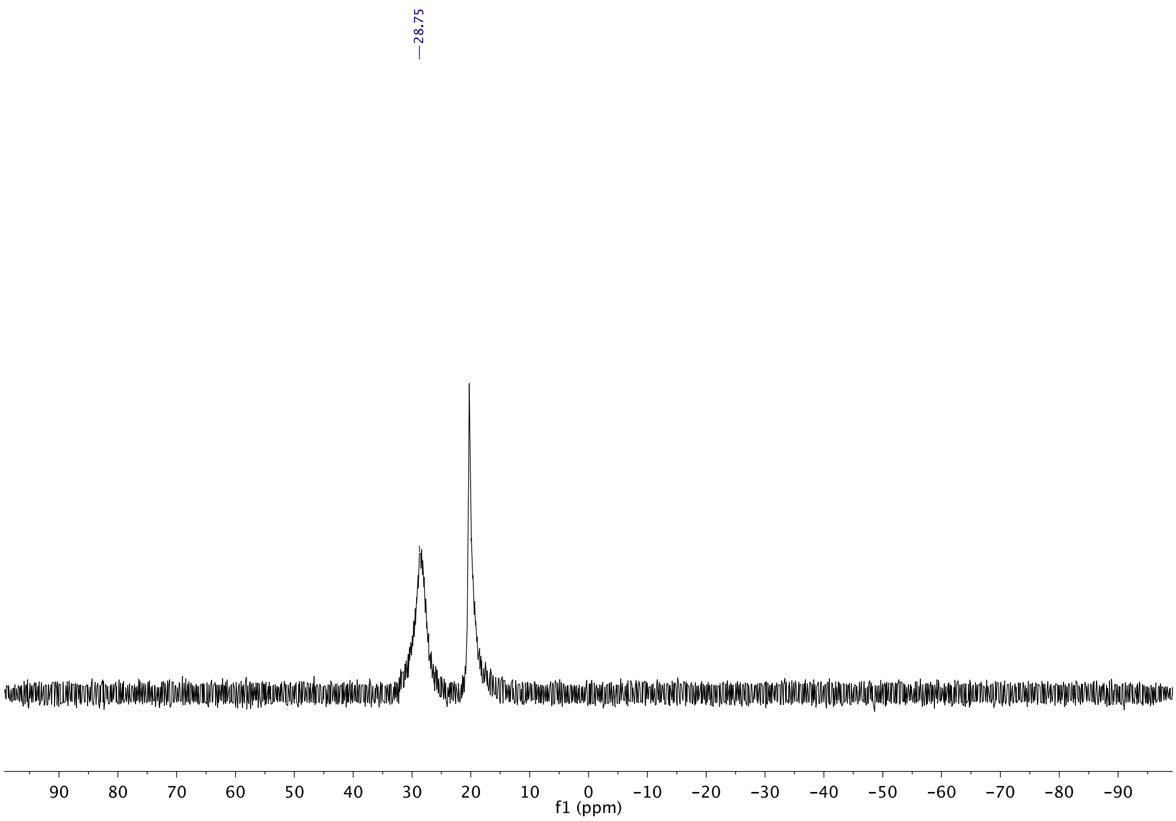

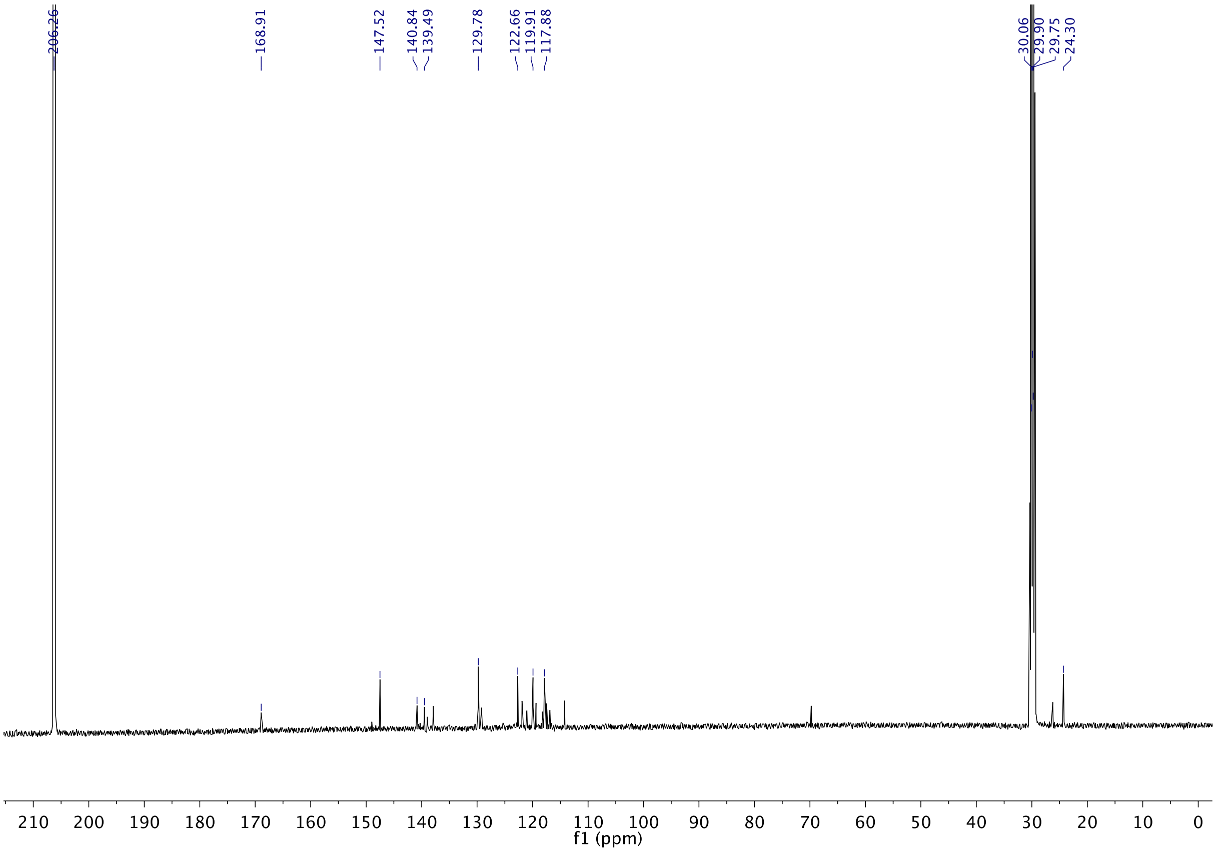

Methyl 3-((trimethylsilyl)ethynyl)benzoate, **S4-int1**

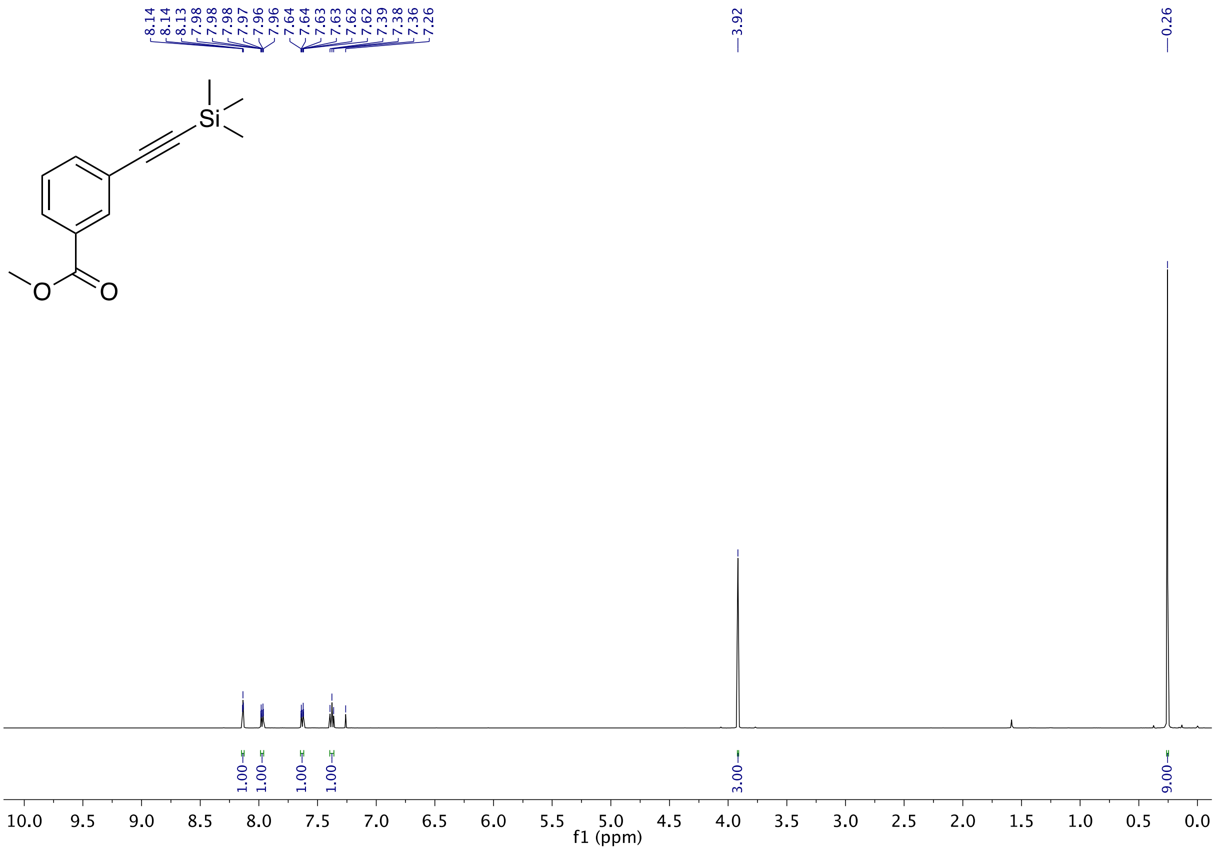

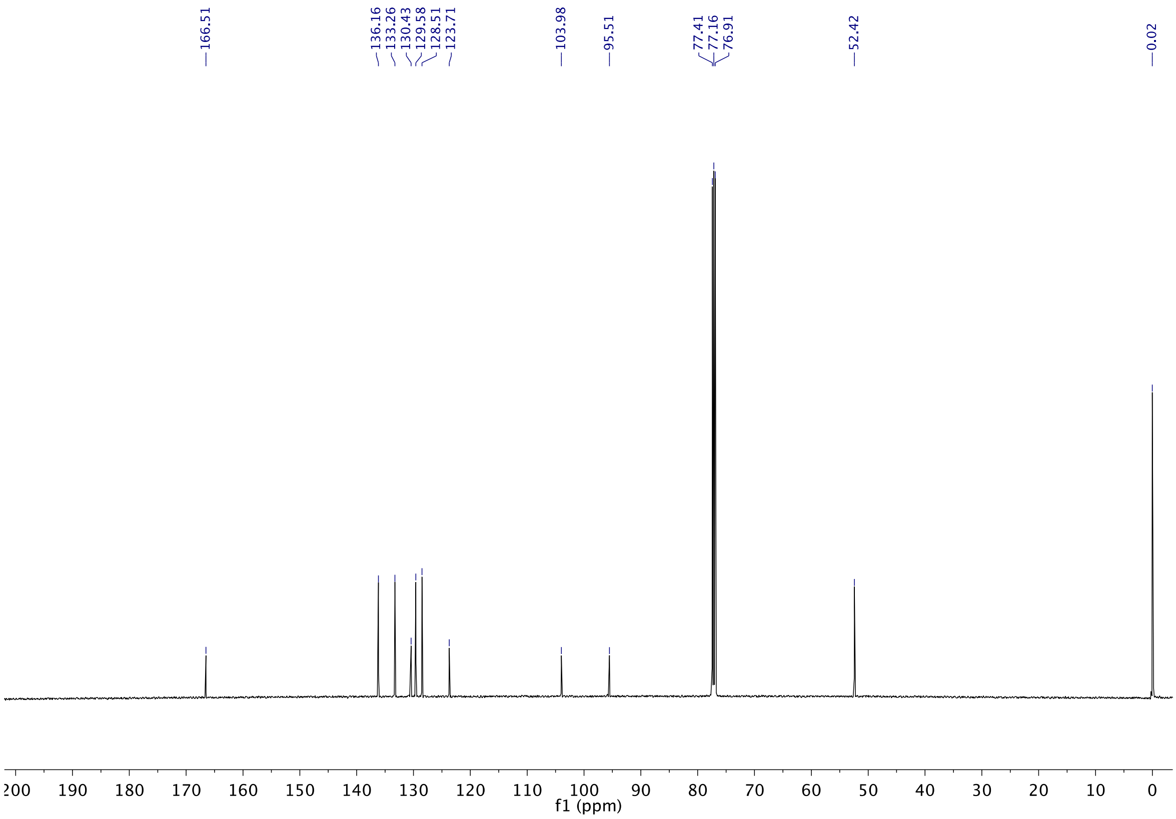

Methyl 3-ethynylbenzoate, **S4-int2**

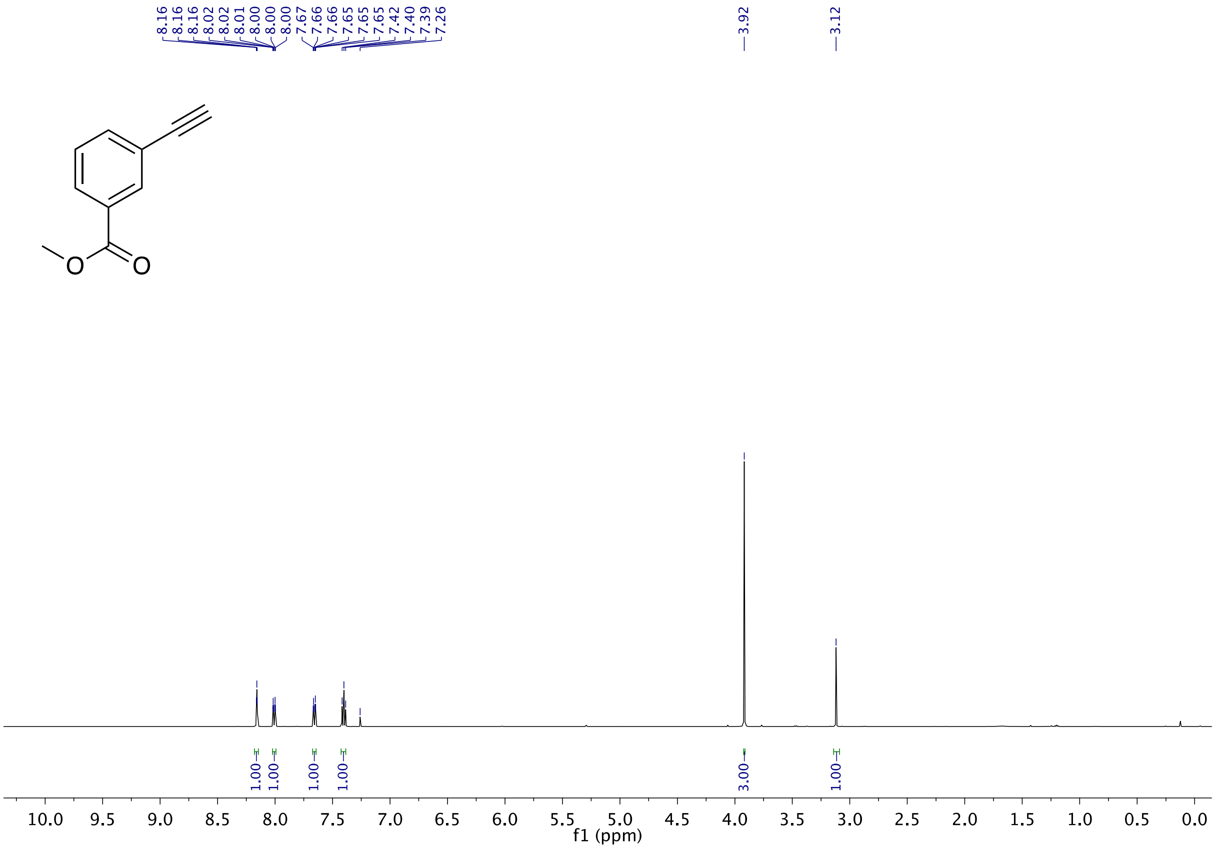

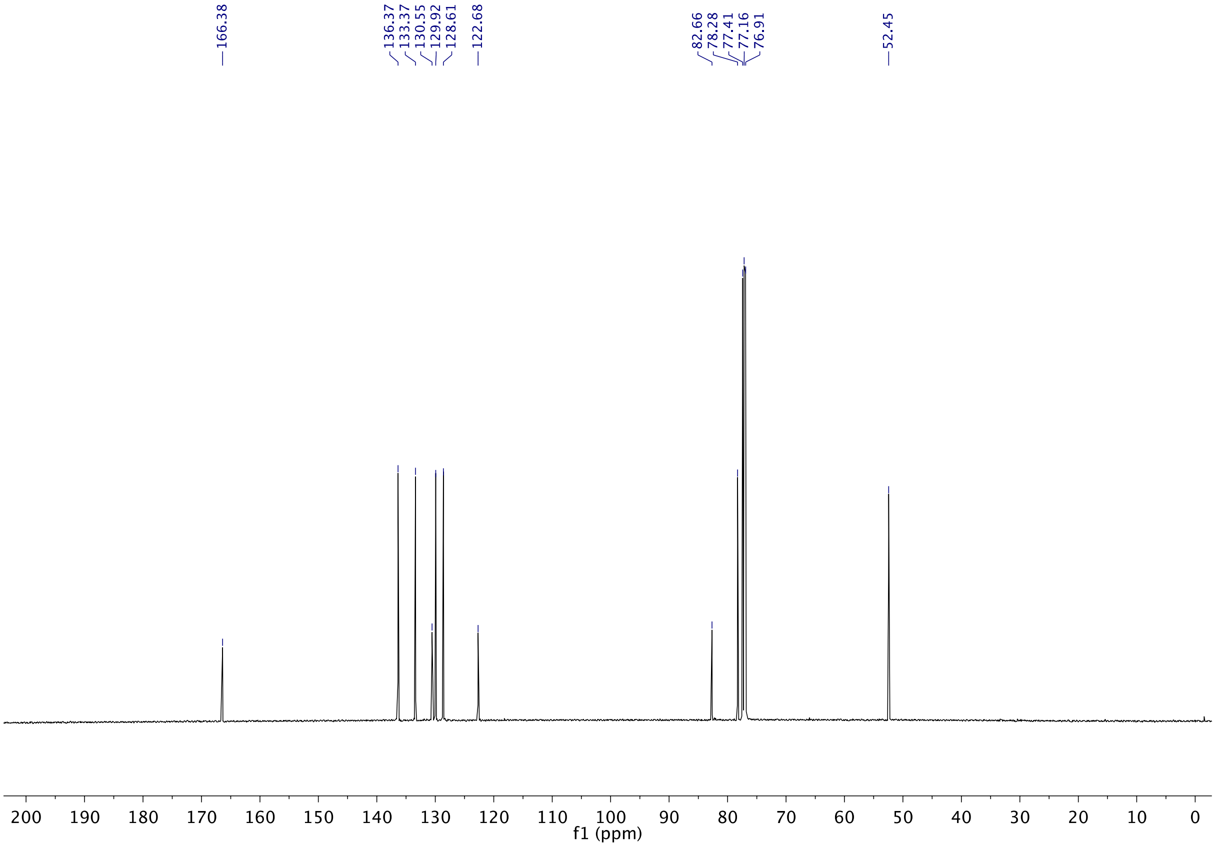

Methyl (*E*)-3-(2-(4,4,5,5-tetramethyl-1,3,2-dioxaborolan-2-yl)vinyl)benzoate, **S4-int3**

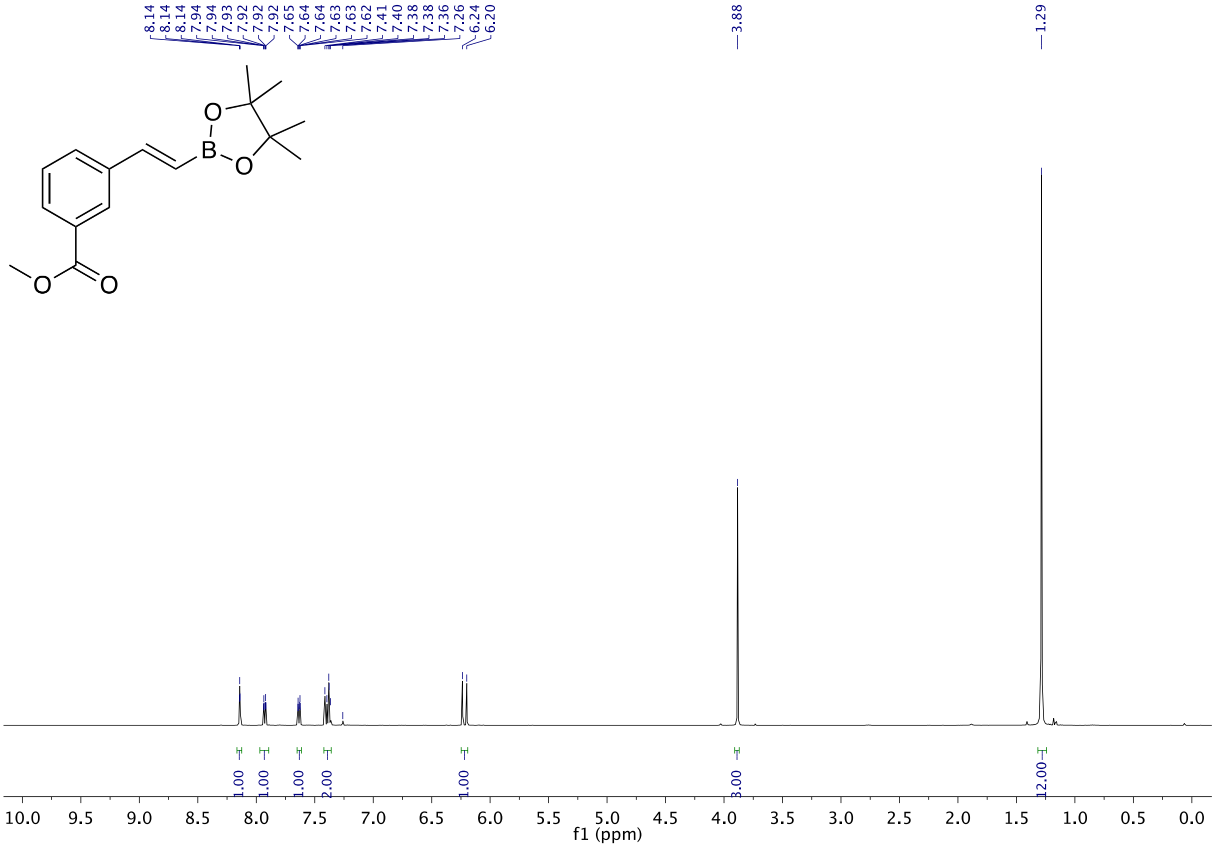

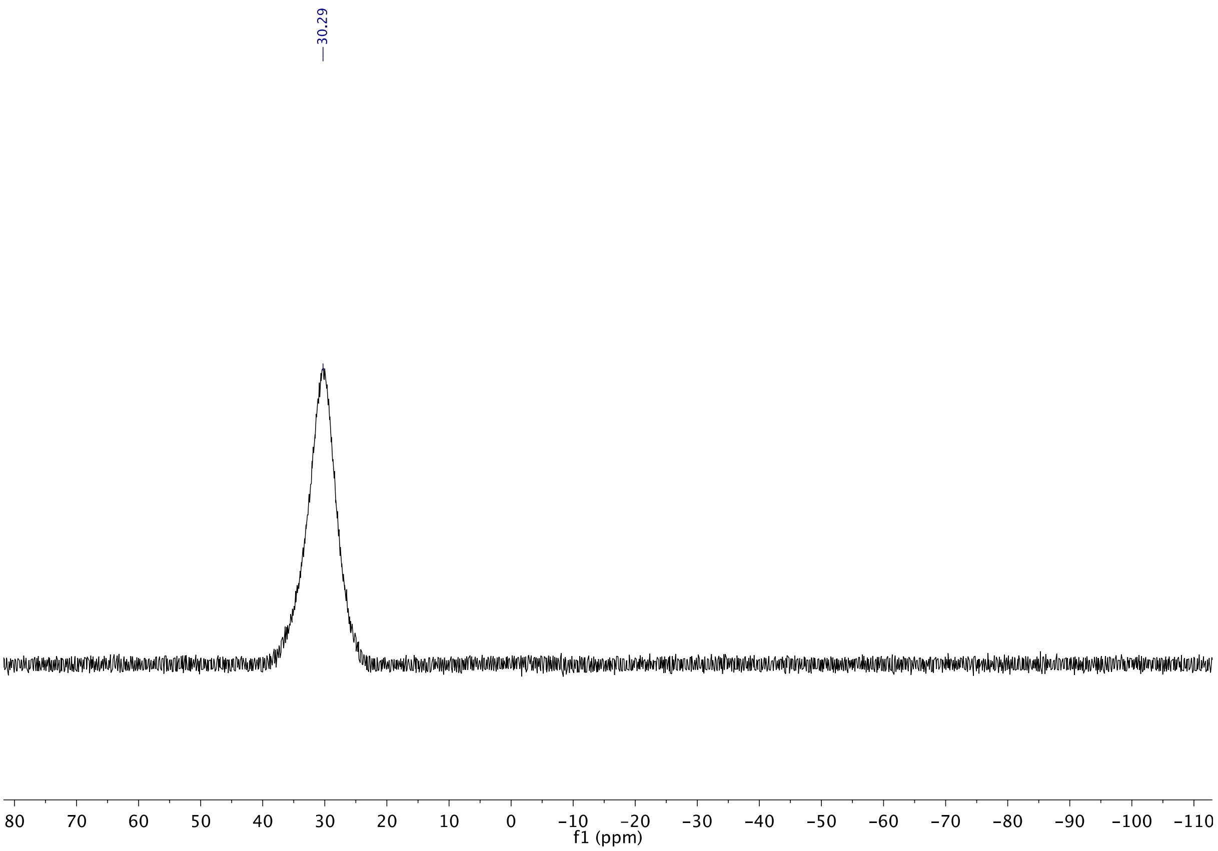
30.29
80 70 60 50 40 30 20 10 0 −10 −20 −30 −40 −50 −60 −70 −80 −90 −100 −110
f1 (ppm)

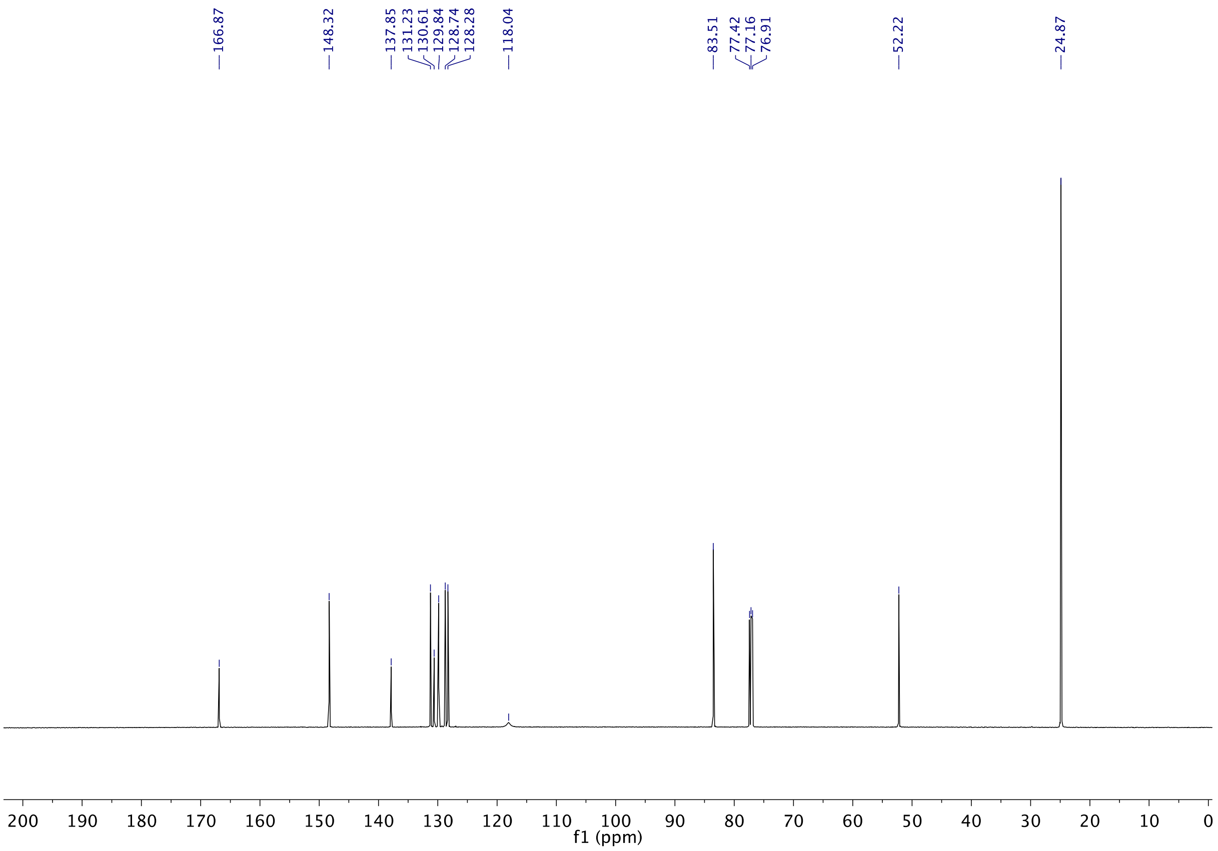
166.87
148.32
137.85
131.23
130.61
129.84
128.74
128.28
118.04
83.51
77.42
77.16
76.91
52.22
24.87
200 190 180 170 160 150 140 130 120 110 100 90 80 70 60 50 40 30 20 10 0
f1 (ppm)

Methyl (*E*)-3-(2-(trifluoro-λ[4]-boraneyl)vinyl)benzoate, potassium salt, **S4-int4**

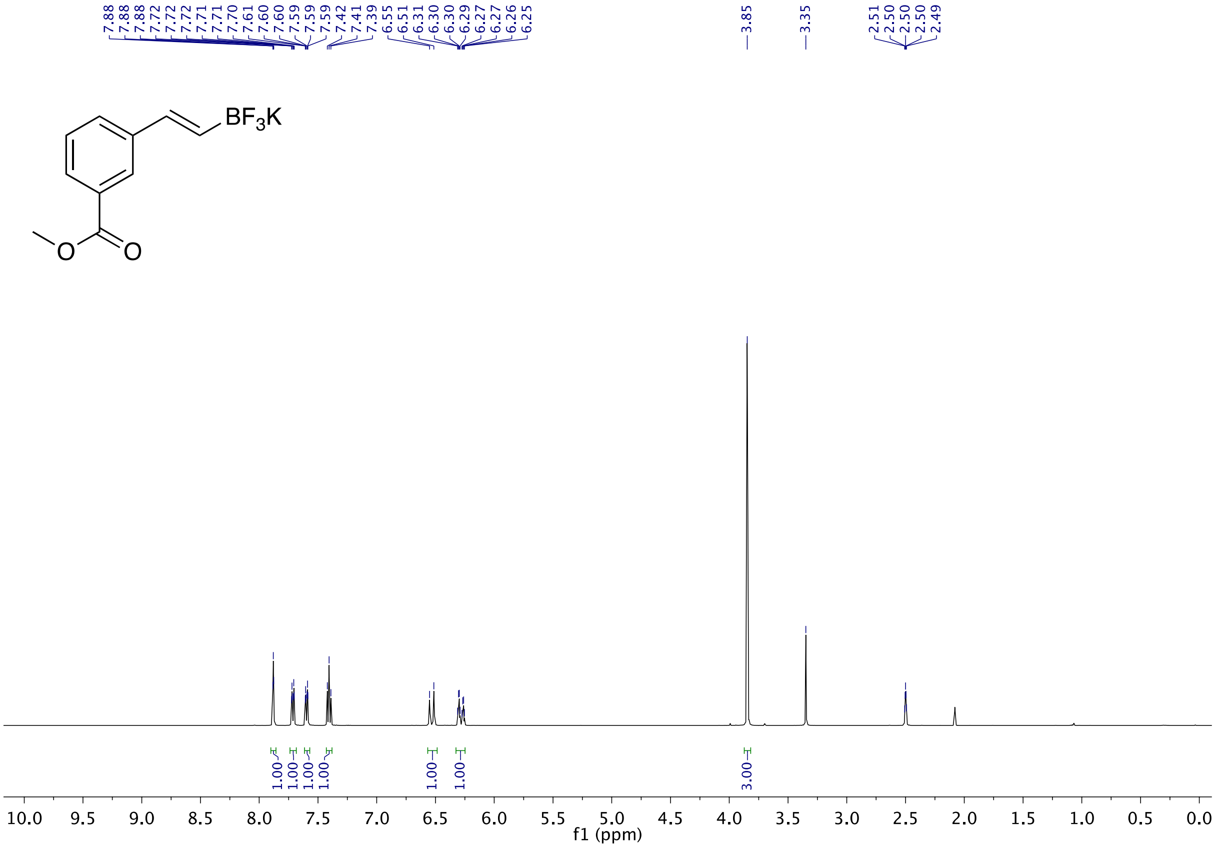

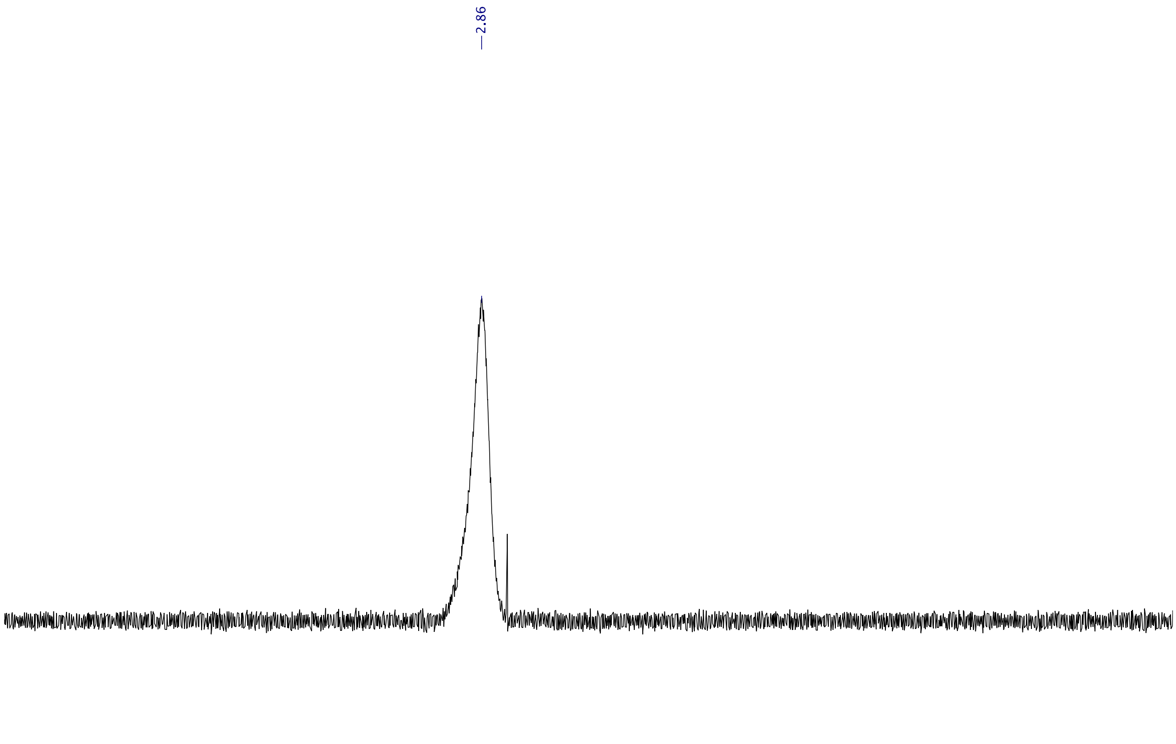

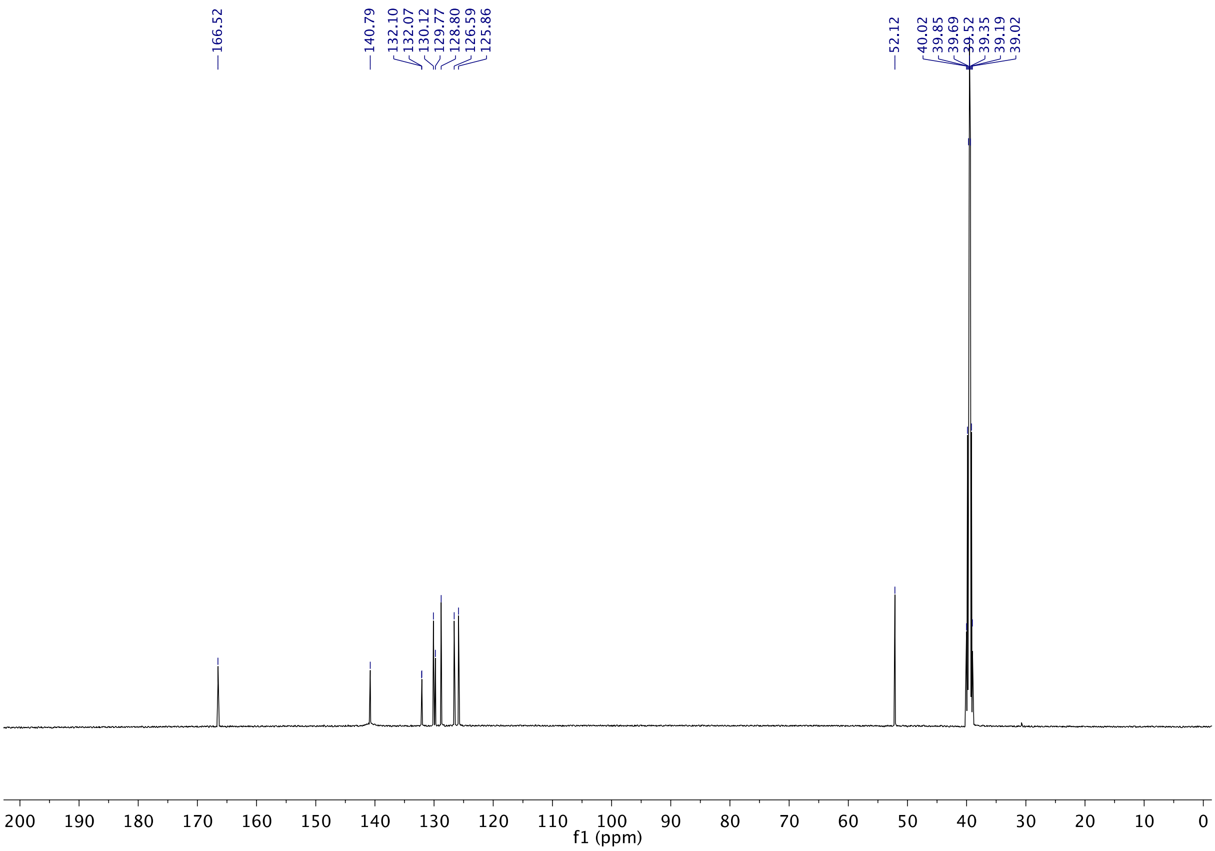

166.52
140.79
132.10
132.07
130.12
129.77
128.80
126.59
125.86
52.12
40.02
39.85
39.69
39.52
39.35
39.19
39.02
200 190 180 170 160 150 140 130 120 110 100 90 80 70 60 50 40 30 20 10 0
f1 (ppm)

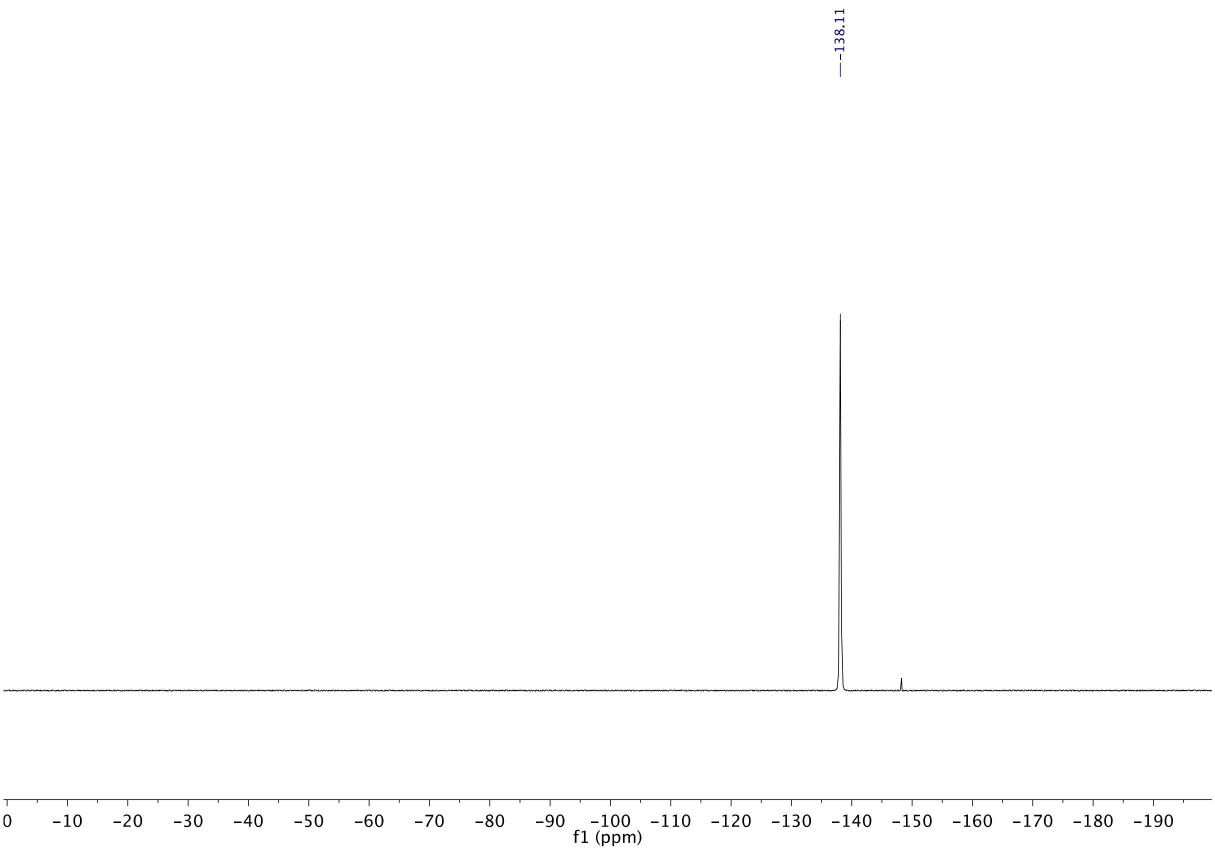

-138.11
0 -10 -20 -30 -40 -50 -60 -70 -80 -90 -100 -110 -120 -130 -140 -150 -160 -170 -180 -190
f1 (ppm)

(*E*)-(3-(Methoxycarbonyl)styryl)boronic acid, **S4**

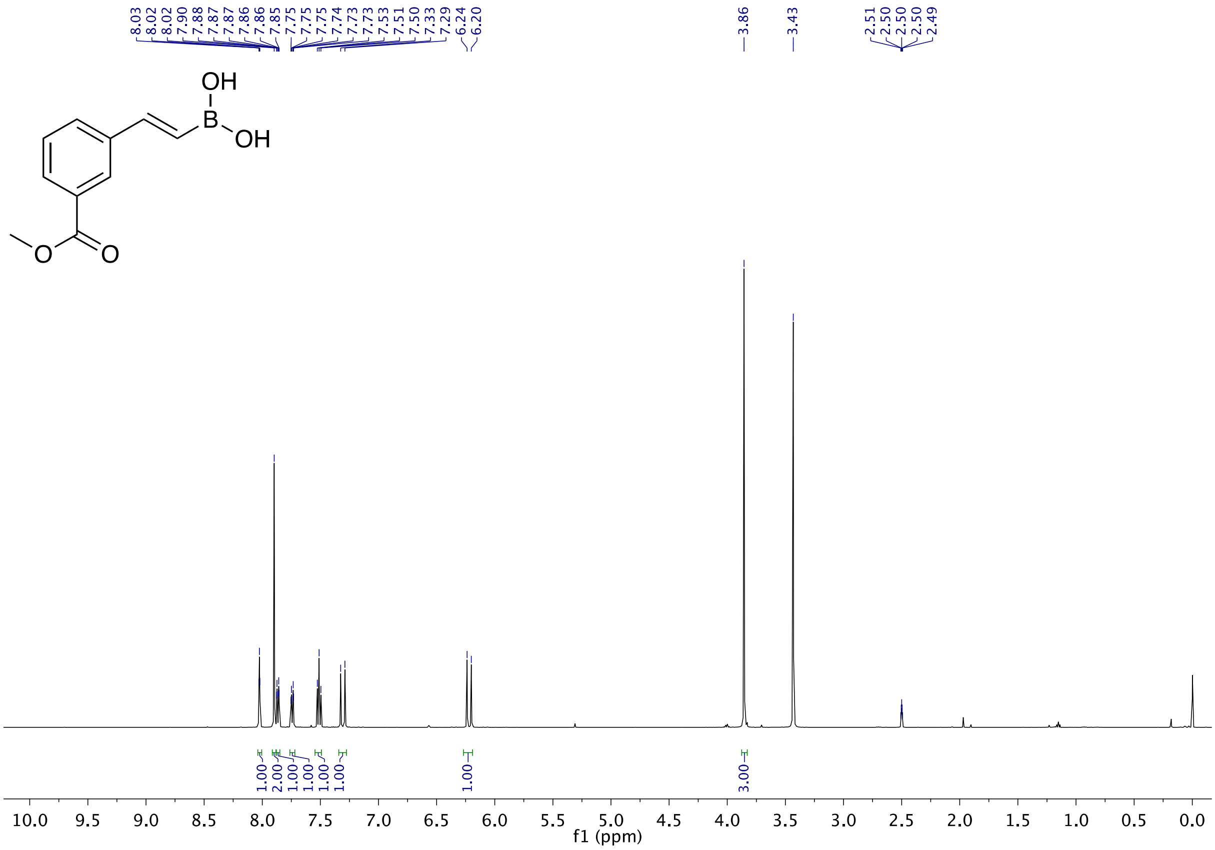

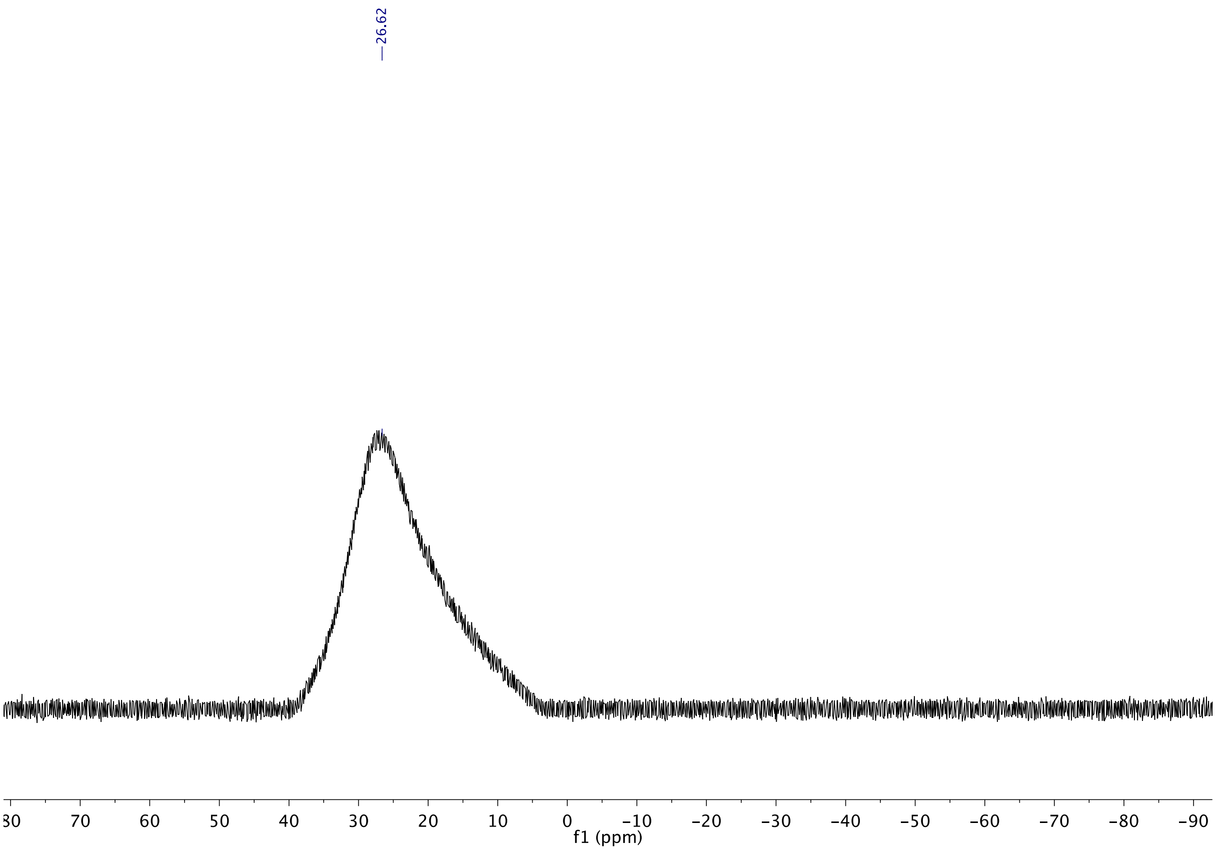

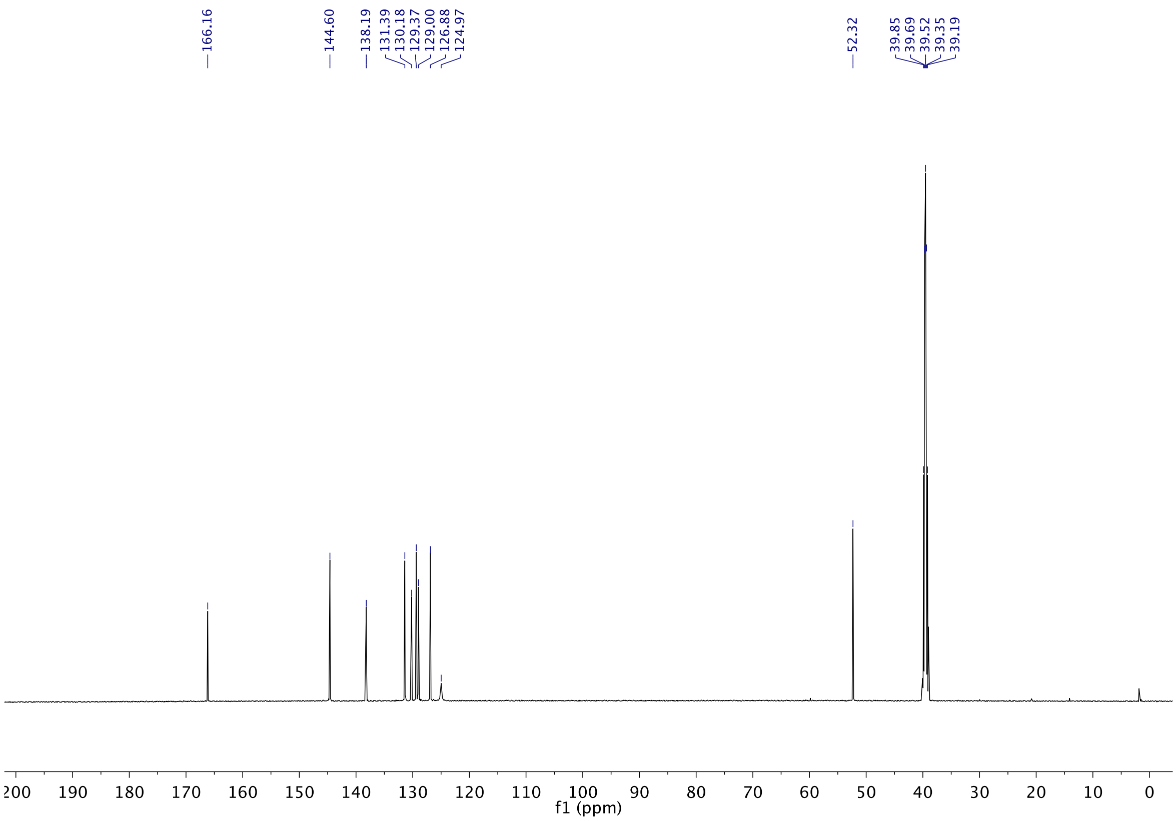

1-(2-((Trimethylsilyl)ethynyl)phenyl)ethan-1-one, **S5-int1**

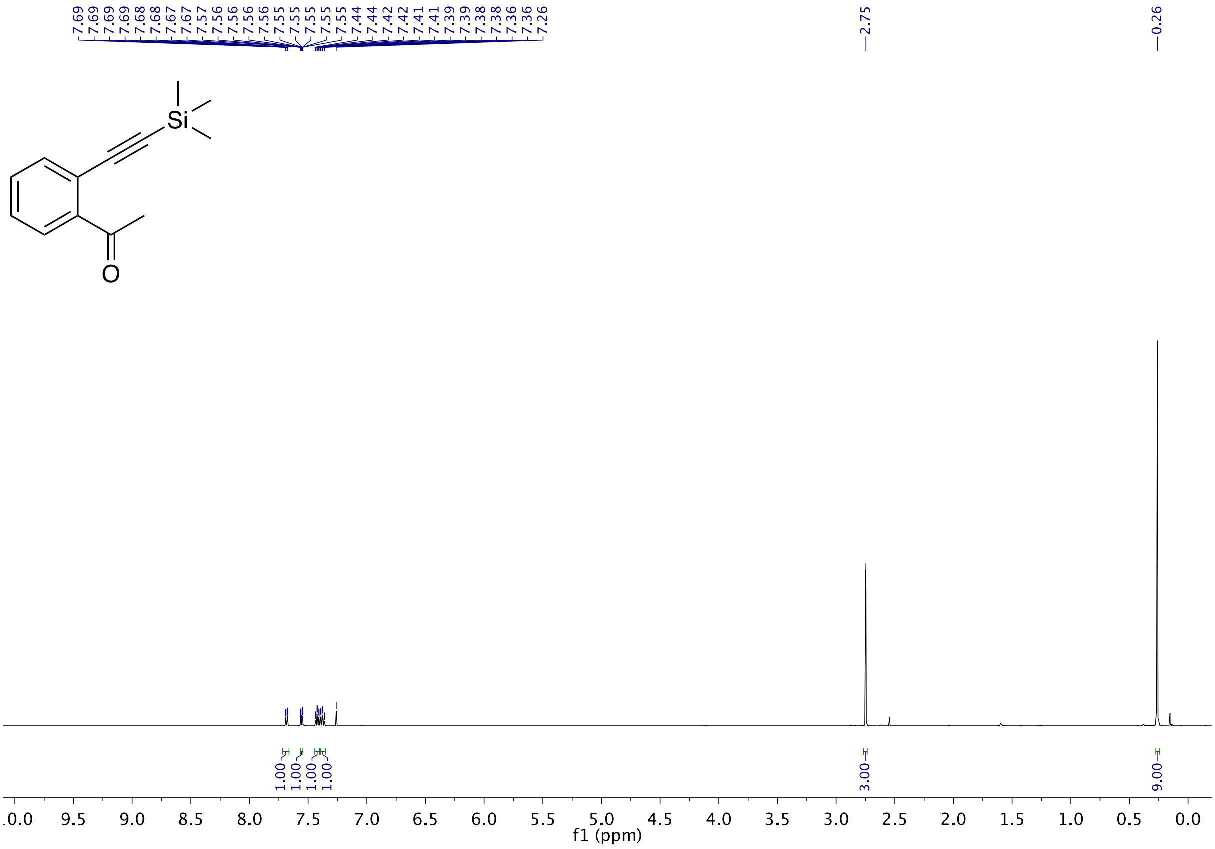

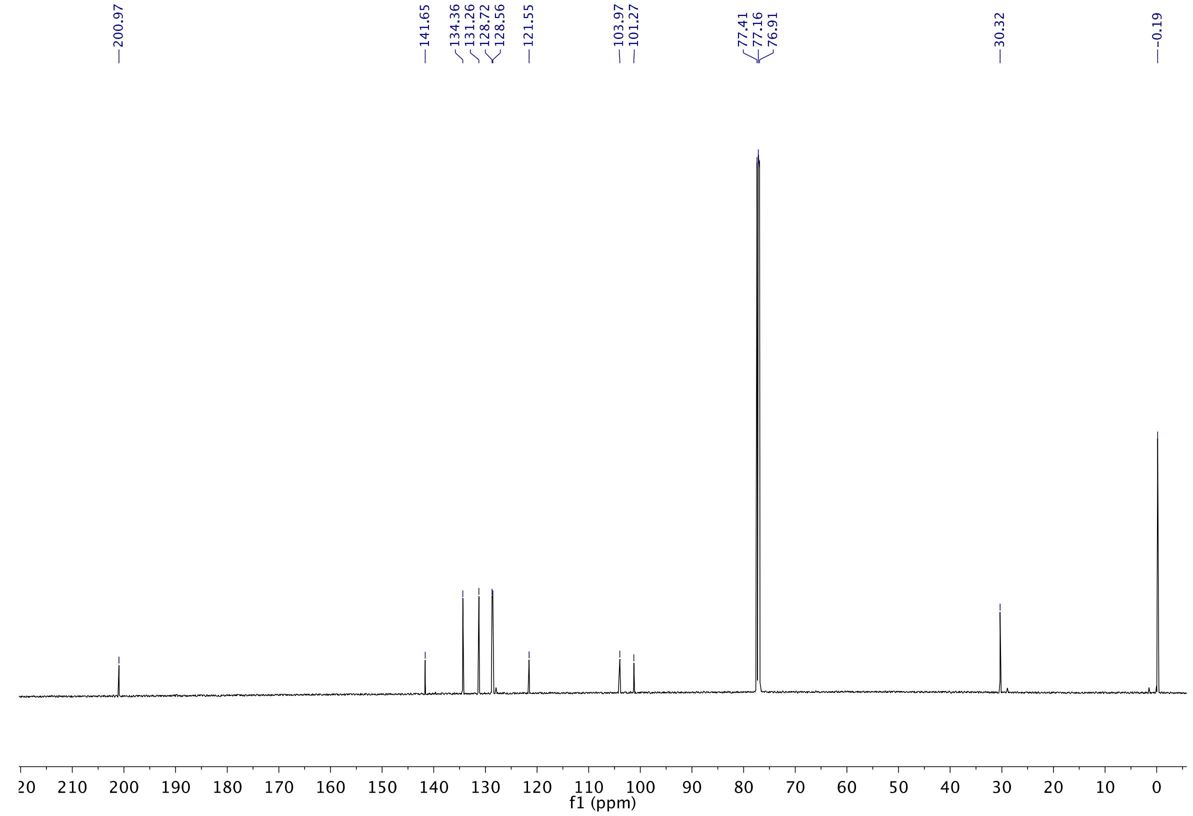

1-(2-Ethynylphenyl)ethan-1-one, **S5-int2**

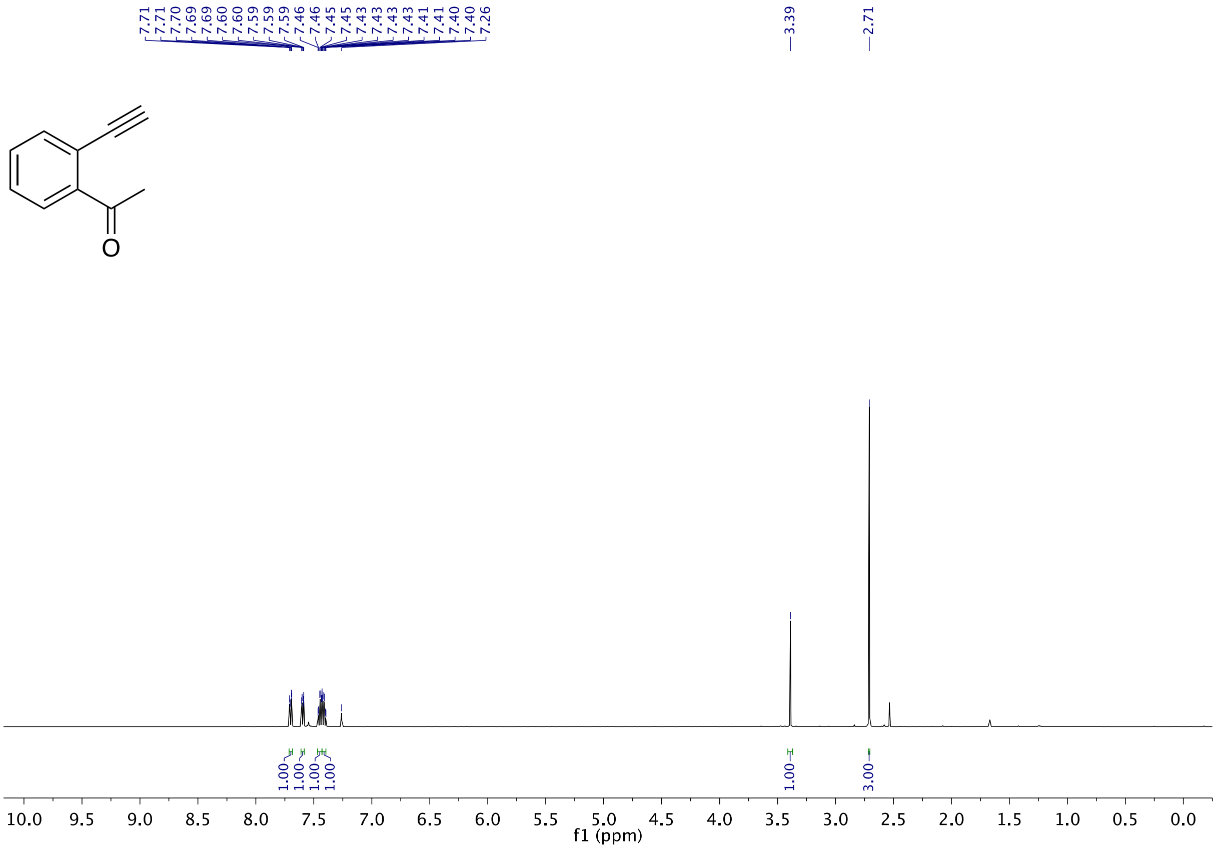

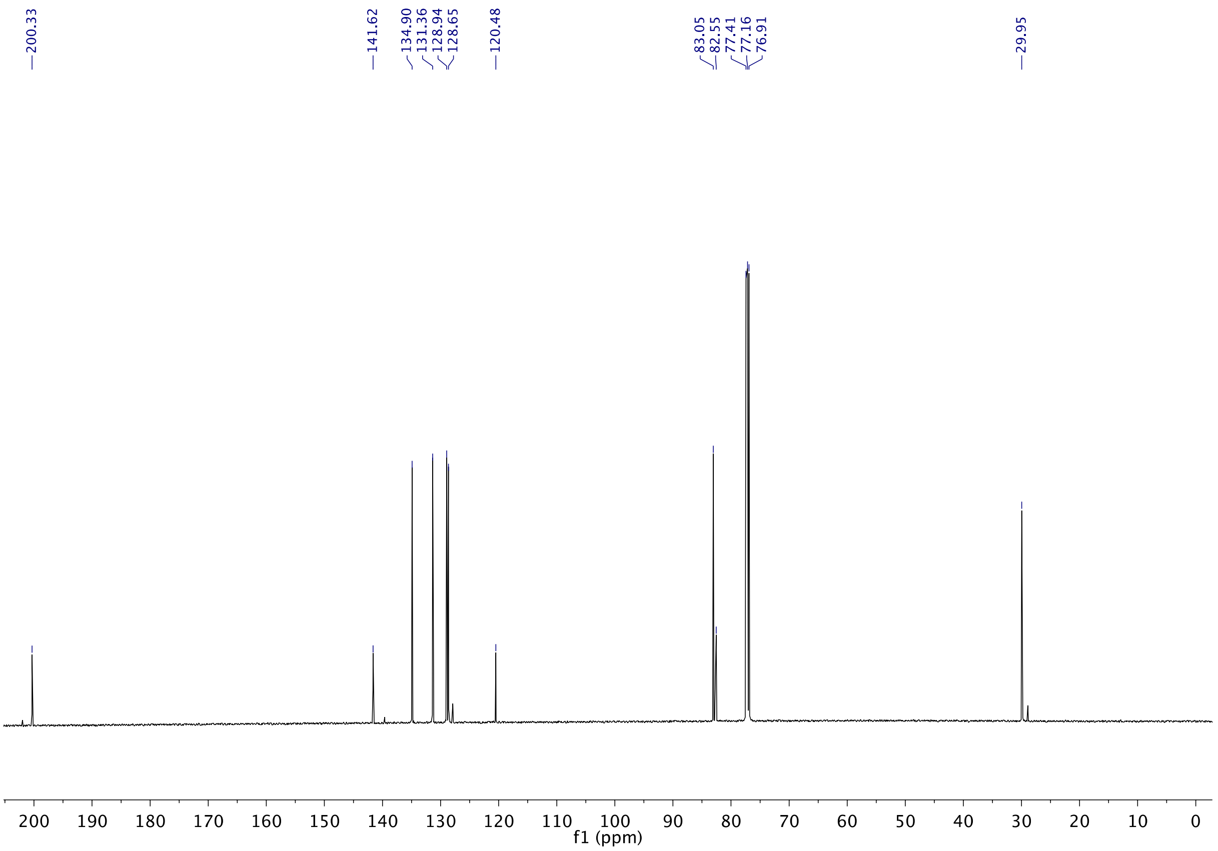

(*E*) and (*Z*)-1-(2-(2-(4,4,5,5-Tetramethyl-1,3,2-dioxaborolan-2-yl)vinyl)phenyl)ethan-1-one, **S5-int3**

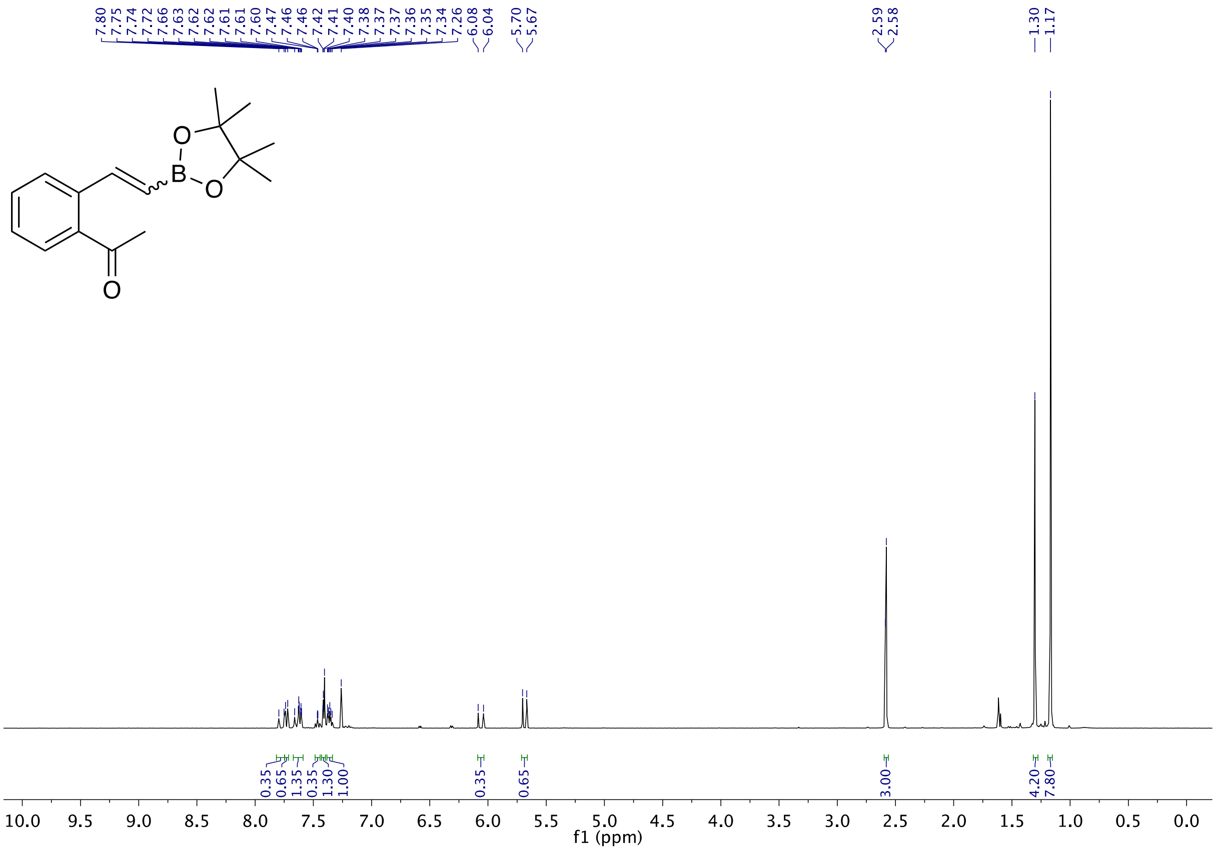

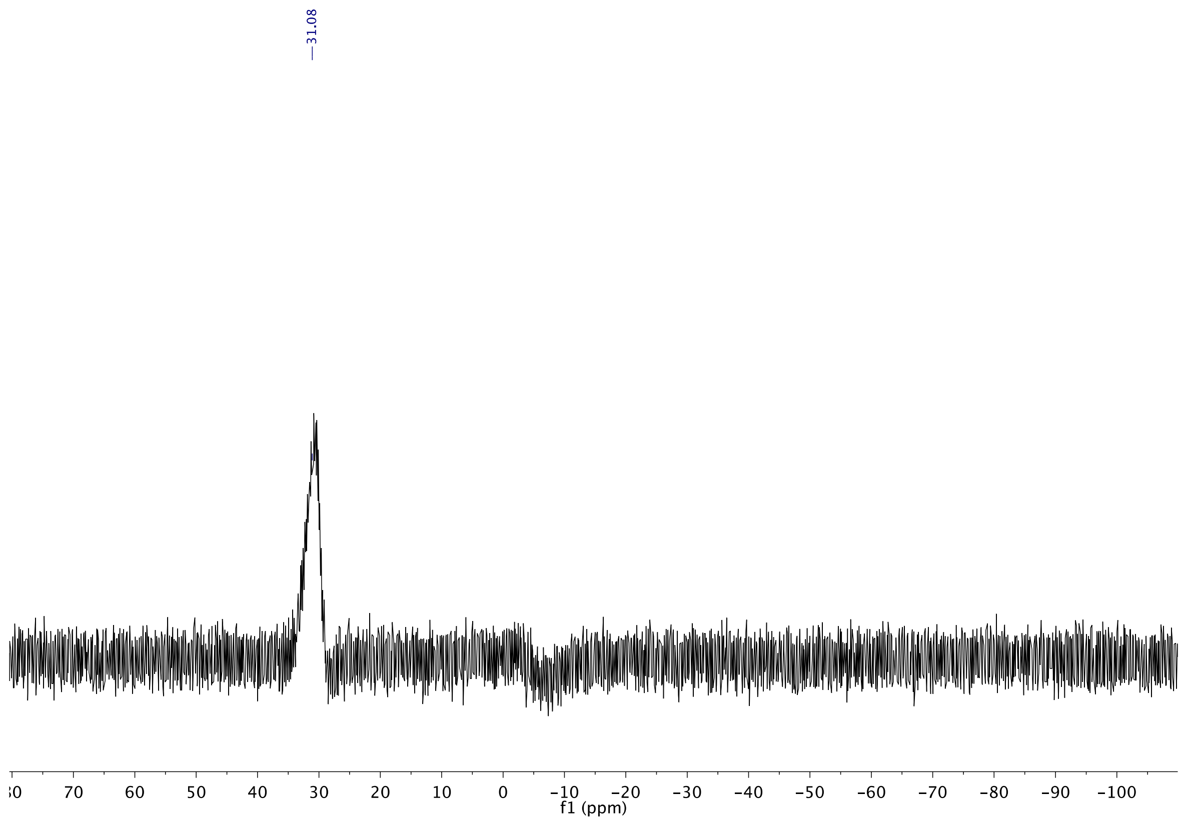

31.08
f1 (ppm)

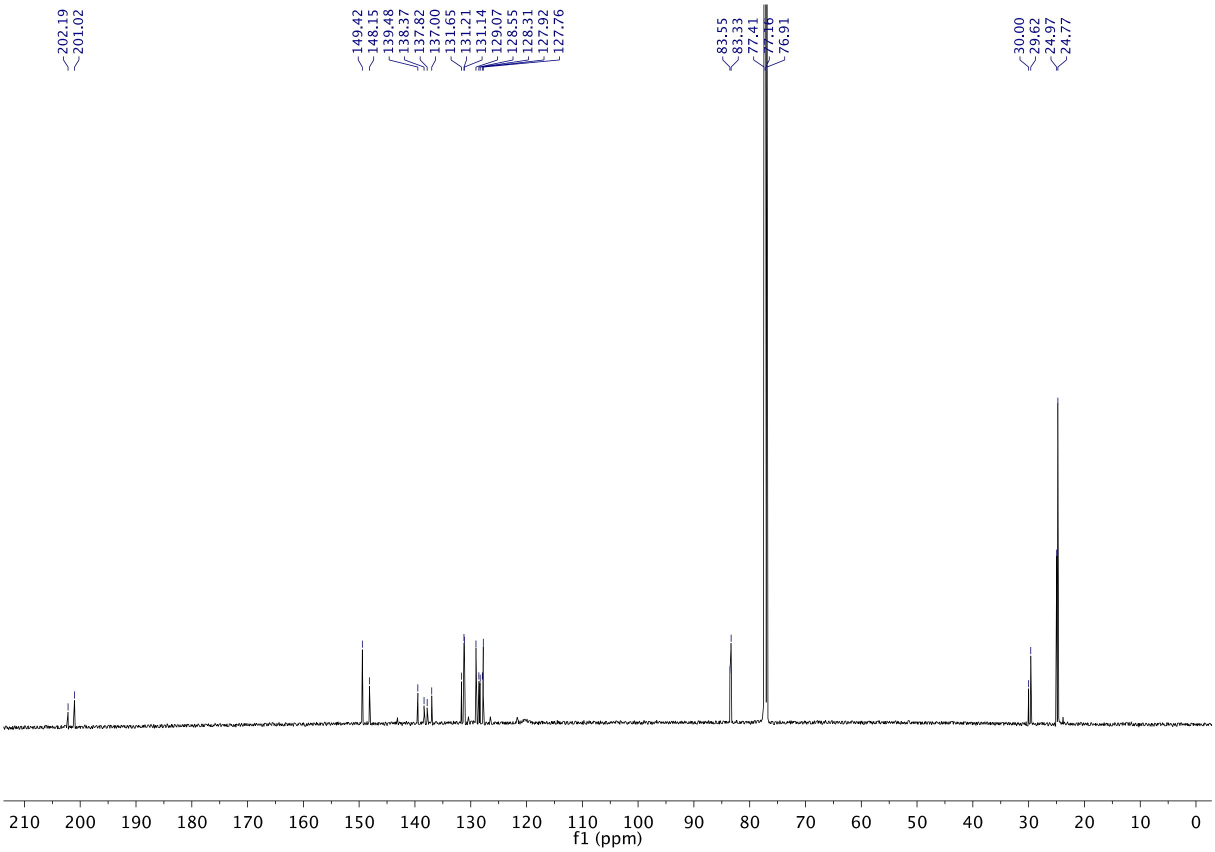

202.19
201.02
149.42
148.15
139.48
138.37
137.82
137.00
131.65
131.21
131.14
129.07
128.55
128.31
127.92
127.76
83.55
83.33
77.41
77.16
76.91
30.00
29.62
24.97
24.77
f1 (ppm)

(*E*) and (*Z*)-1-(2-(2-(Trifluoro-λ[4]-boraneyl)vinyl)phenyl)ethan-1-one, potassium salt, **S5-int4**

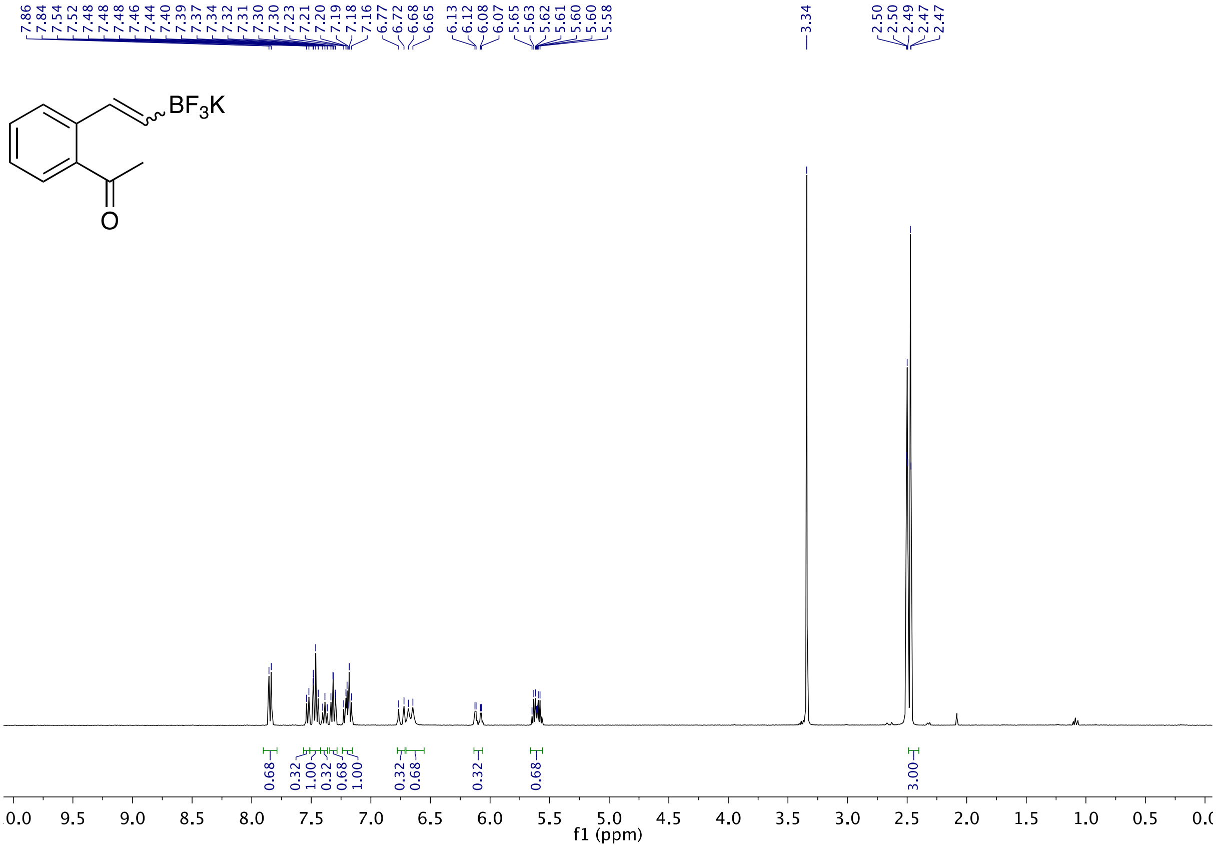

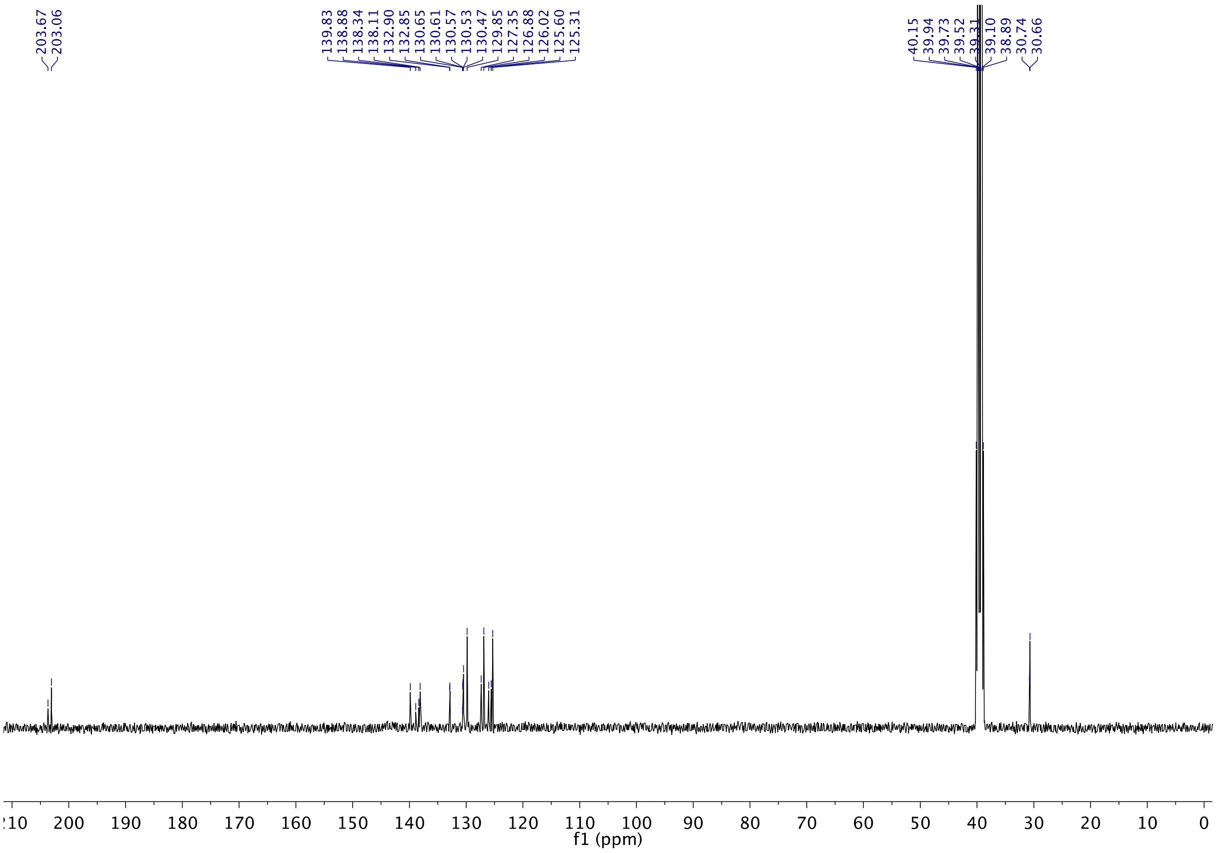

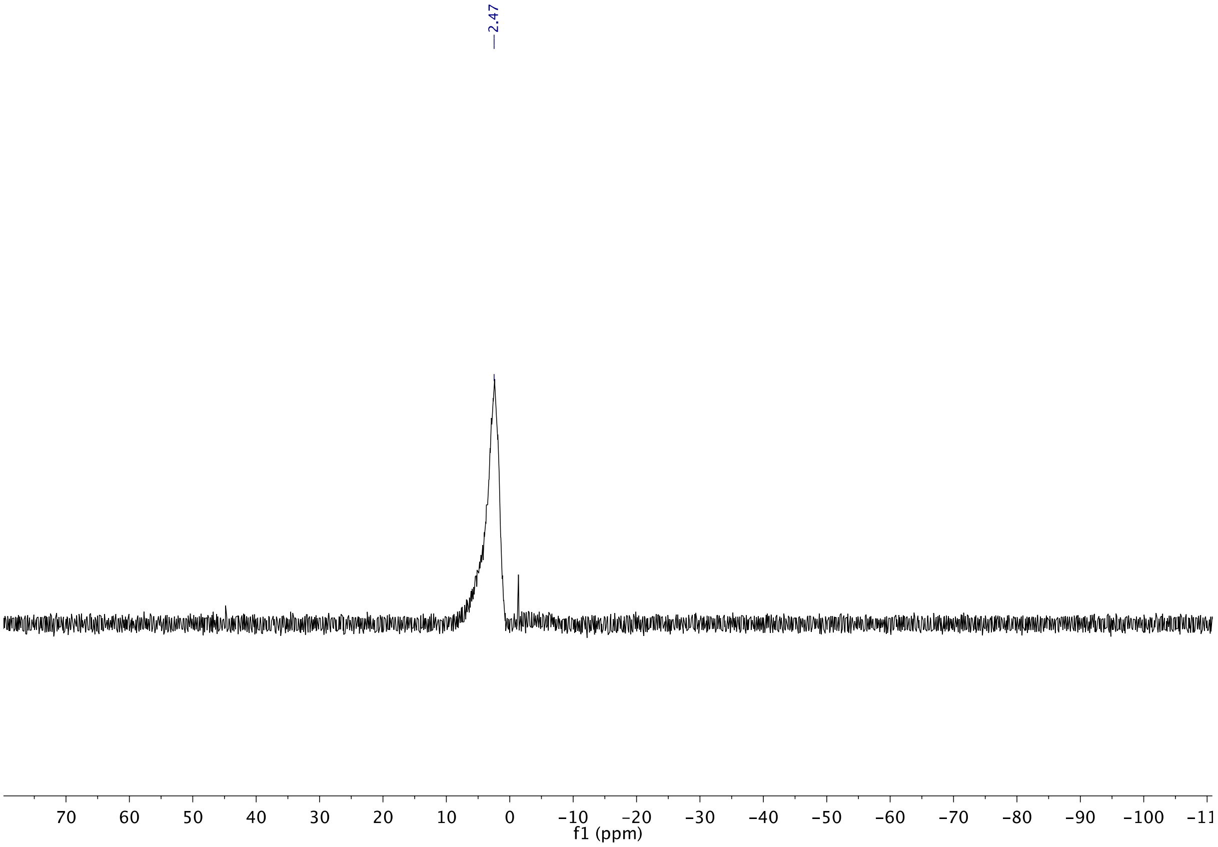

2.47
70 60 50 40 30 20 10 0 −10 −20 −30 −40 −50 −60 −70 −80 −90 −100
f1 (ppm)

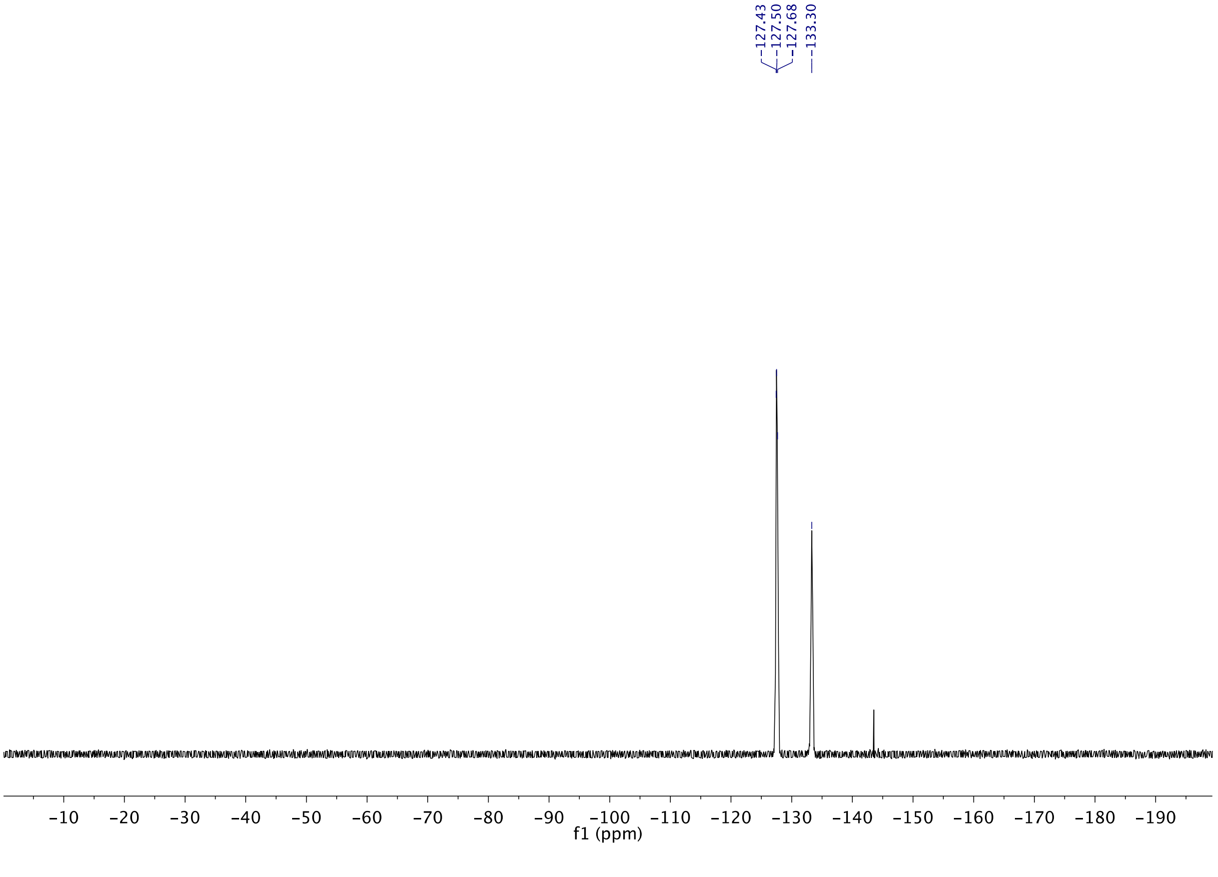

-127.43
-127.50
-127.68
-133.30
−10 −20 −30 −40 −50 −60 −70 −80 −90 −100 −110 −120 −130 −140 −150 −160 −170 −180 −190
f1 (ppm)

(*E*) and (*Z*)-(2-acetylstyryl)boronic acid, **S5**

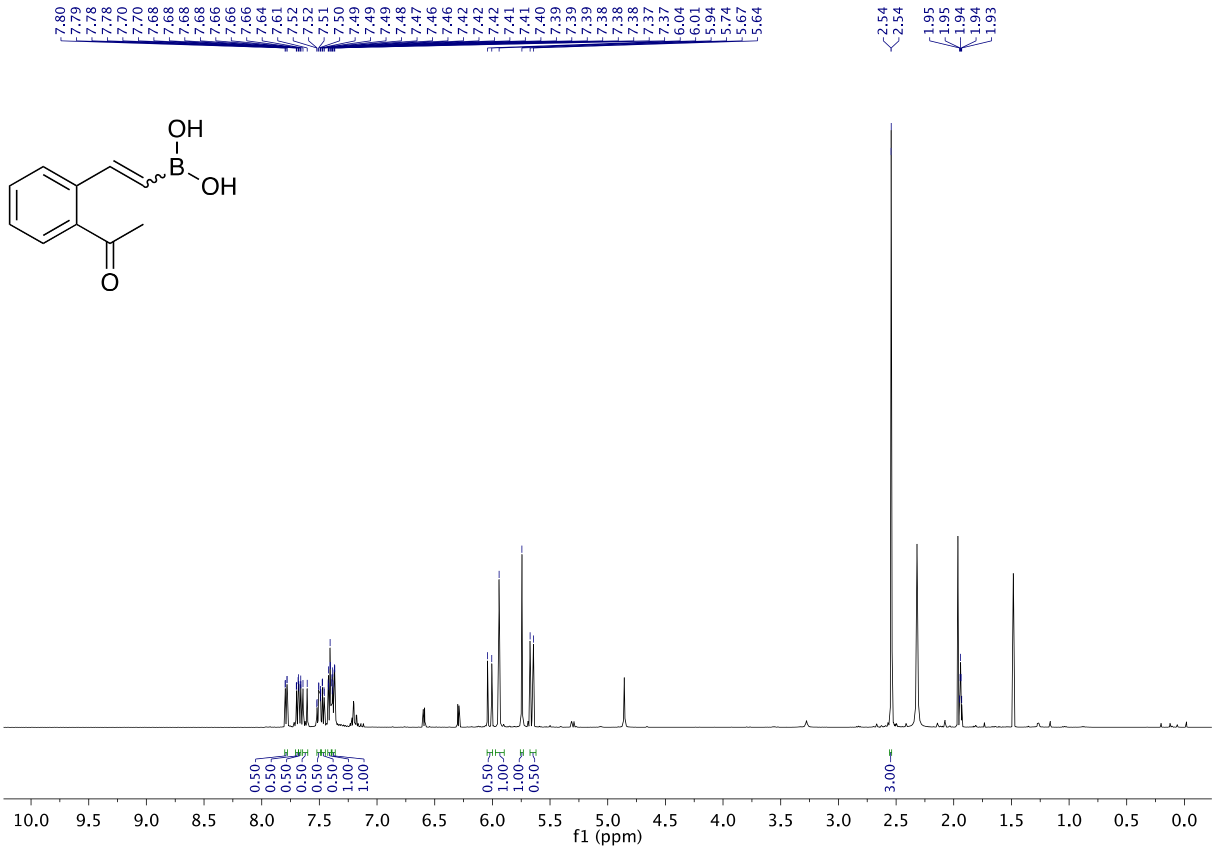

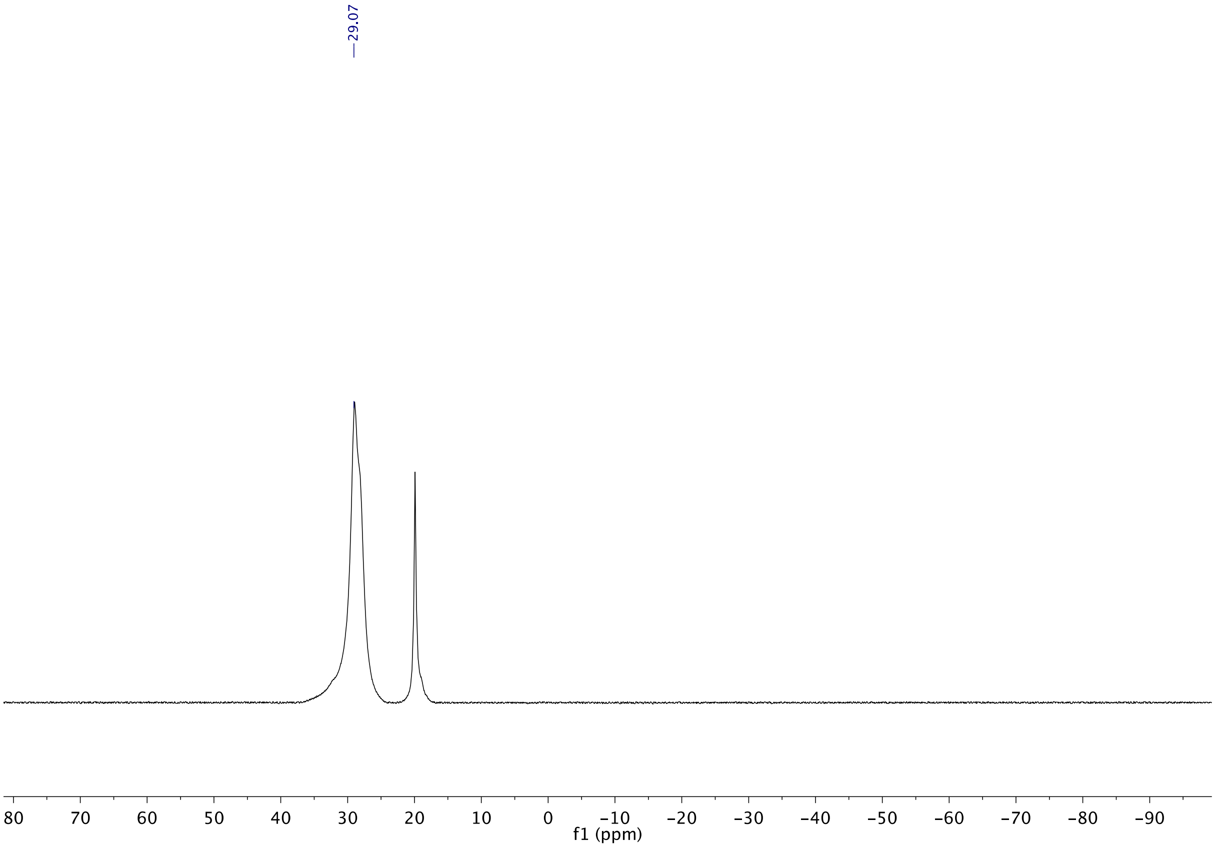

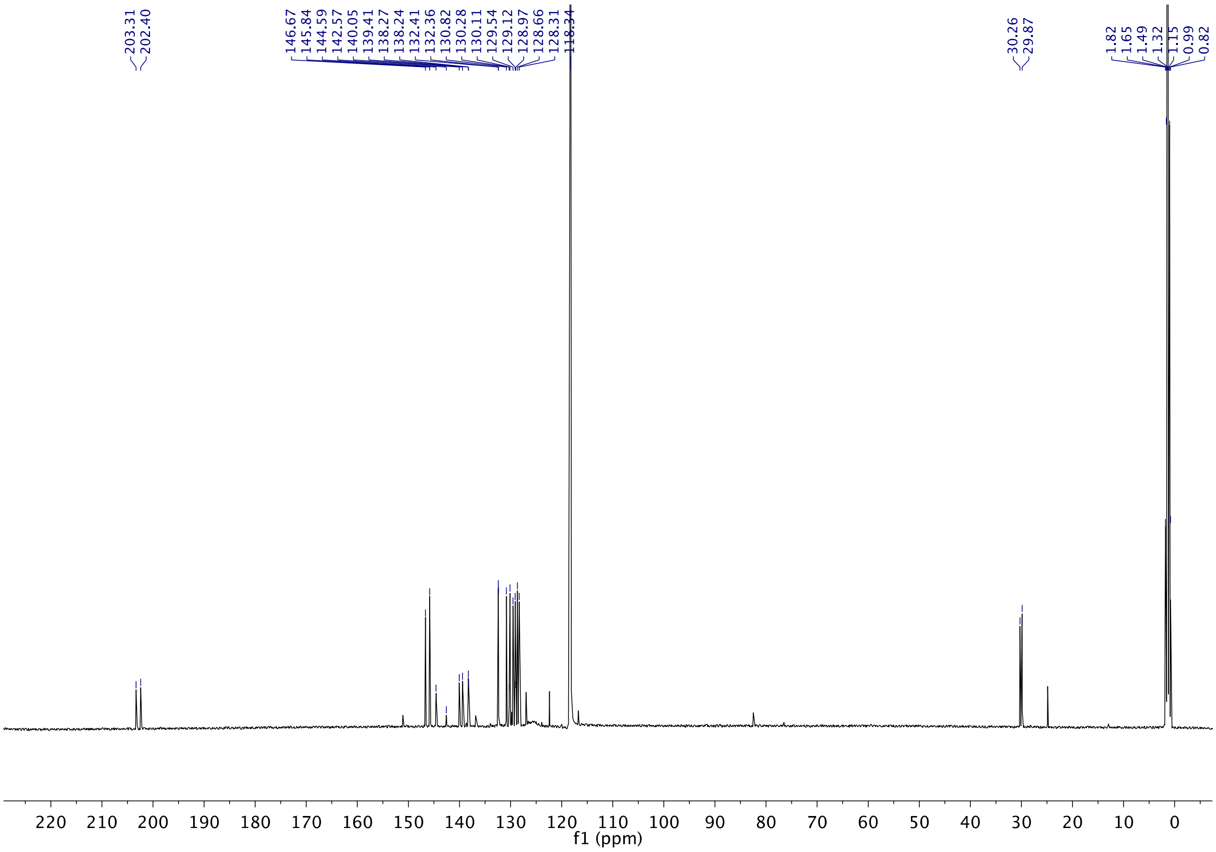

((2-Bromophenyl)ethynyl)trimethylsilane, **S6-int1**

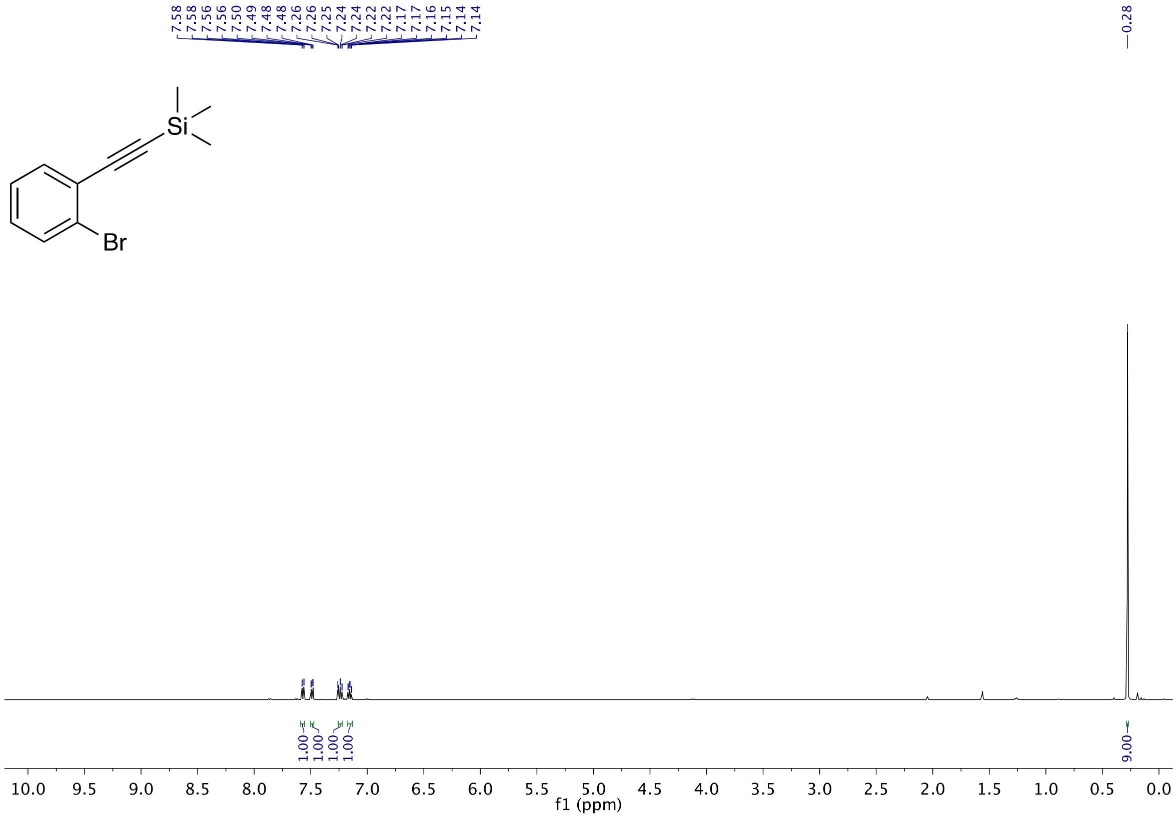

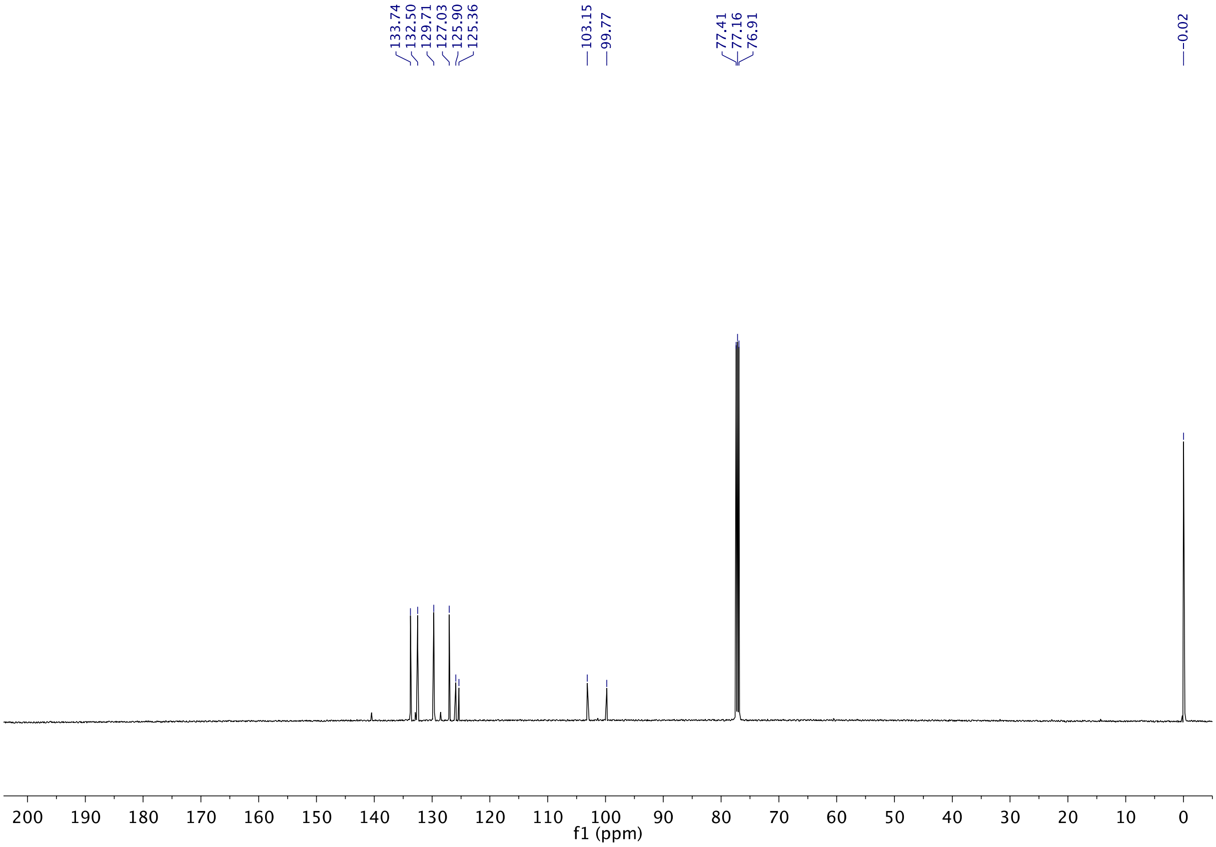

1-Bromo-2-ethynylbenzene, **S6-int2**

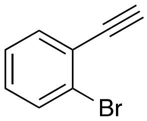

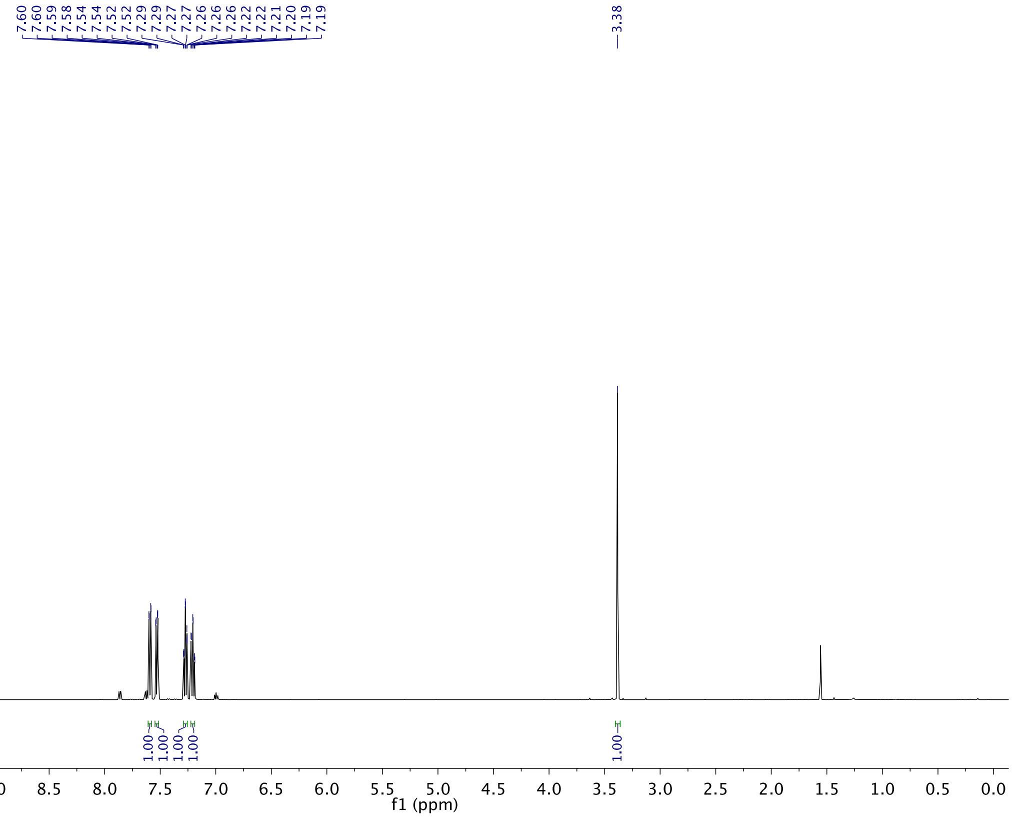

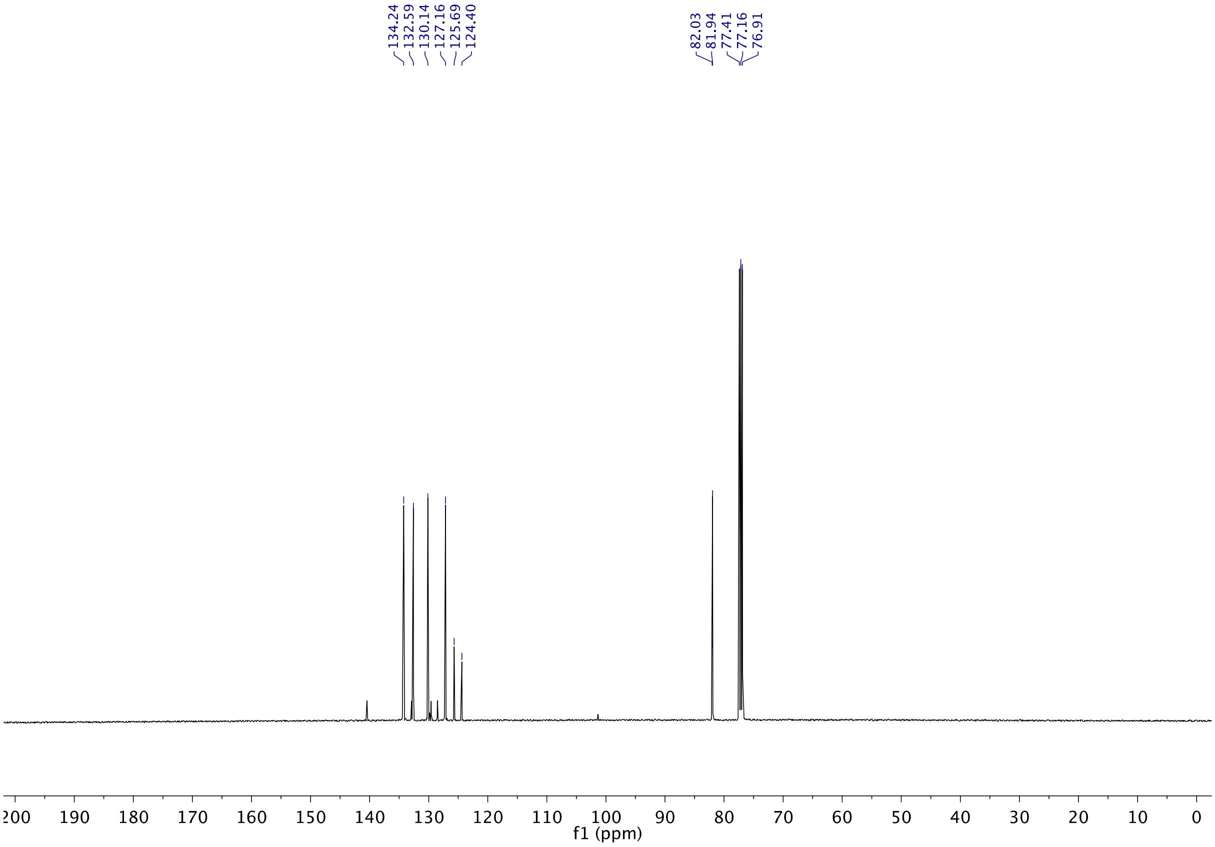

(*E*)-2-(2-Bromostyryl)-4,4,5,5-tetramethyl-1,3,2-dioxaborolane, **S6-int3**

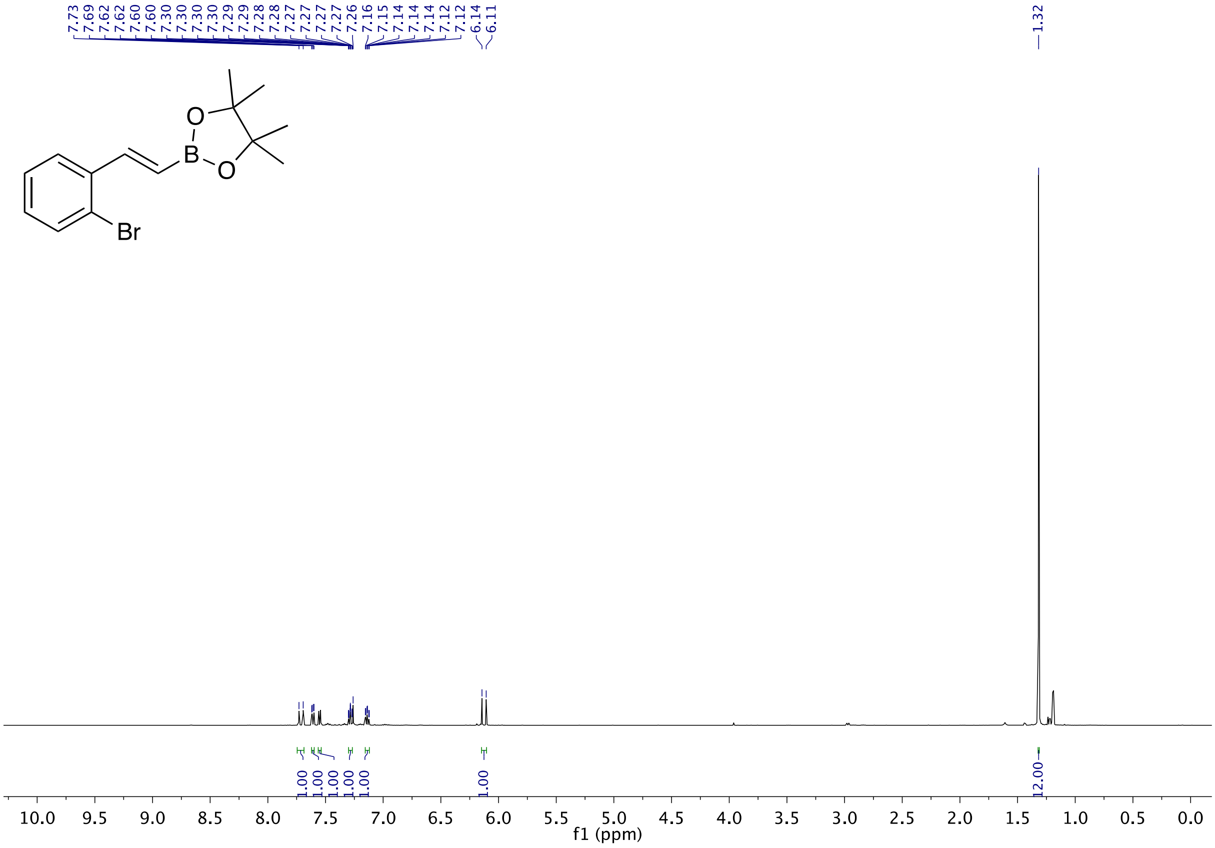

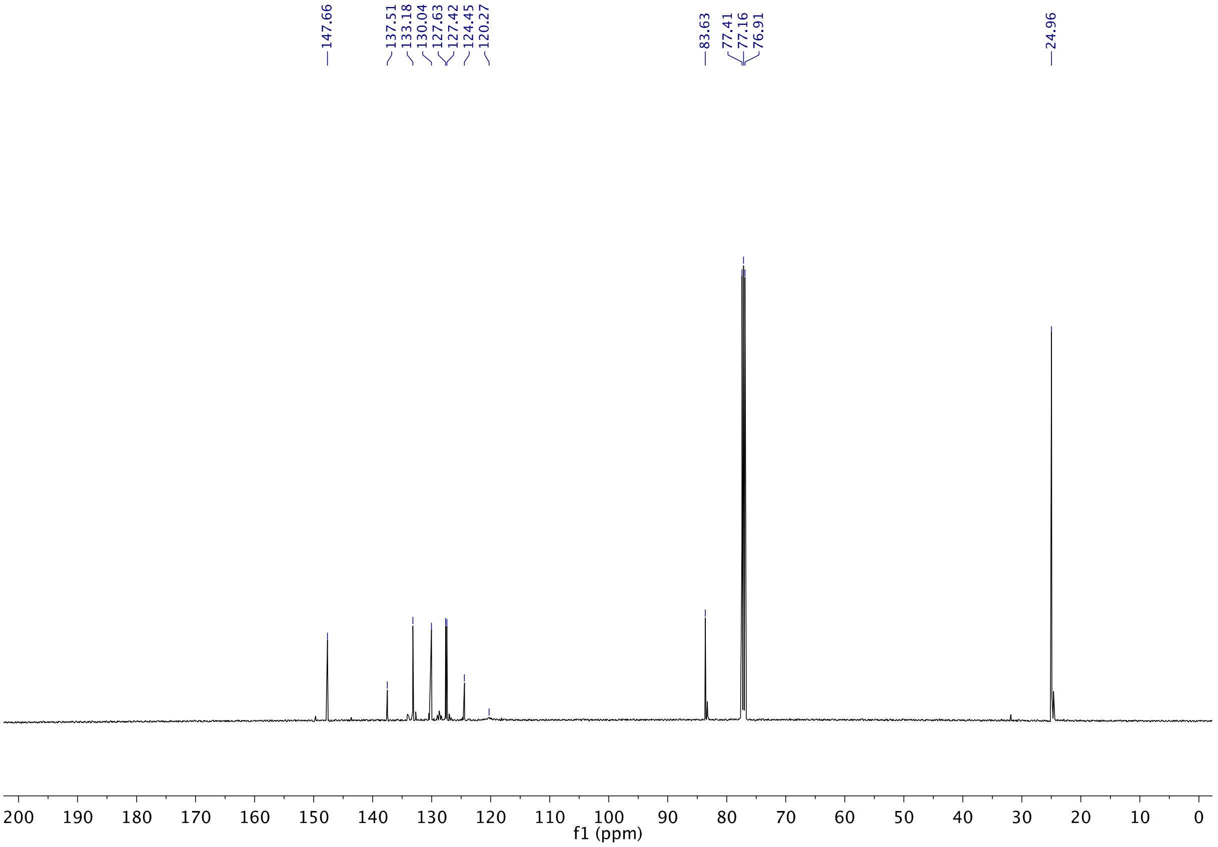
147.66
137.51
133.18
130.04
127.63
127.42
124.45
120.27
83.63
77.41
77.16
76.91
24.96
200 190 180 170 160 150 140 130 120 110 100 90 80 70 60 50 40 30 20 10 0
f1 (ppm)

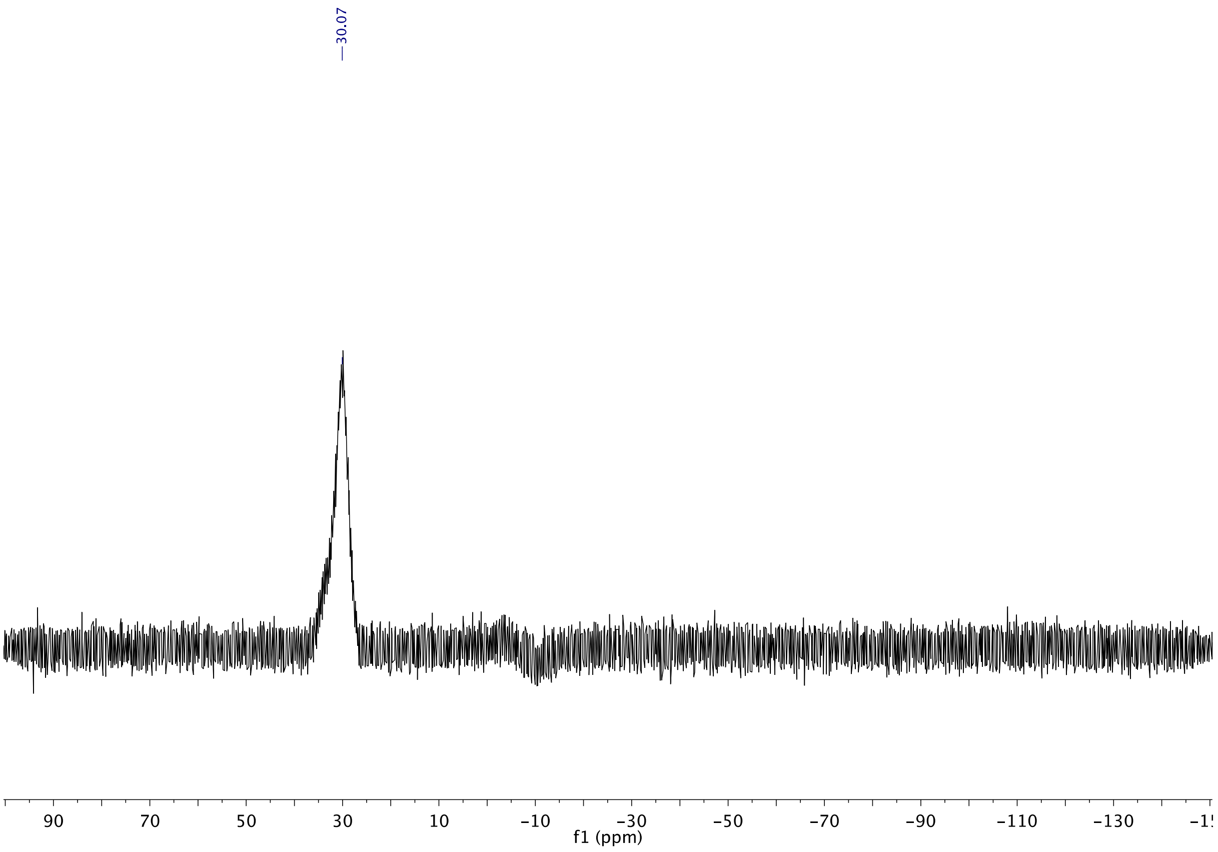
30.07
90 70 50 30 10 −10 −30 −50 −70 −90 −110 −130
f1 (ppm)

(*E*)-(2-Bromostyryl)trifluoro-λ[4]-borane, potassium salt, **S6-int4**

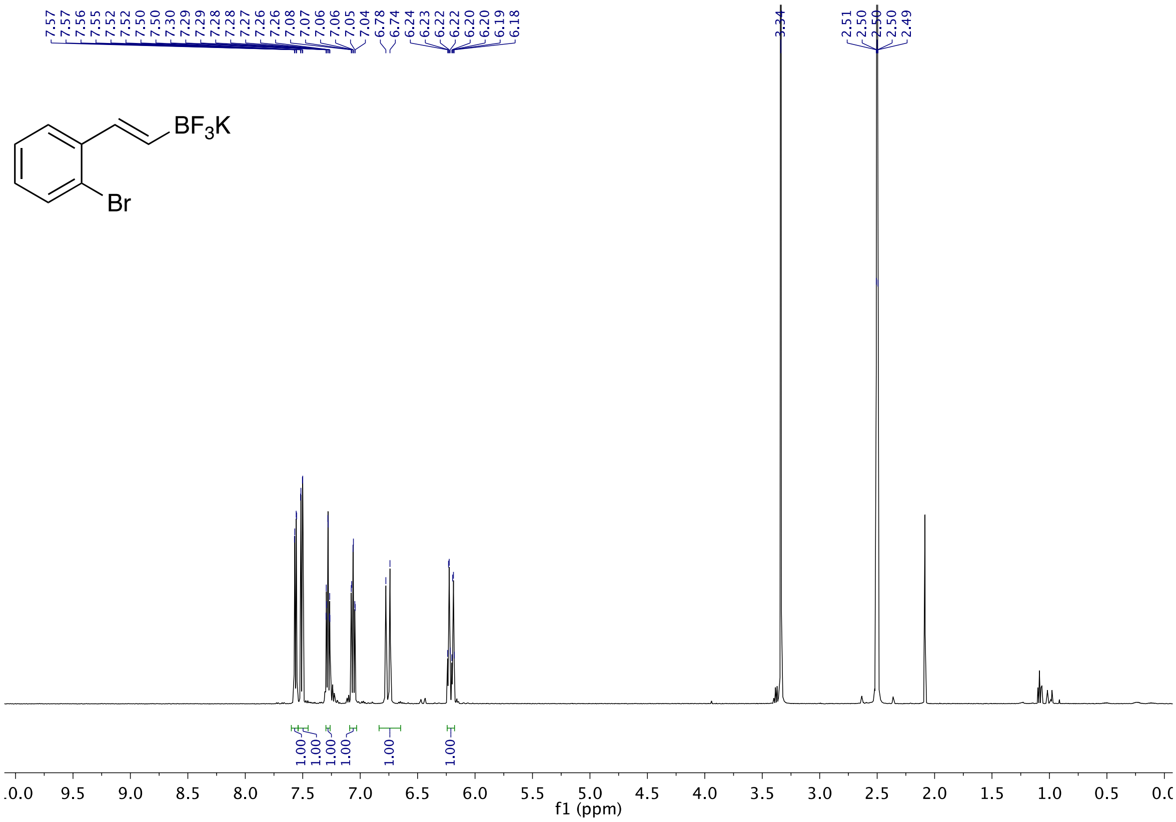

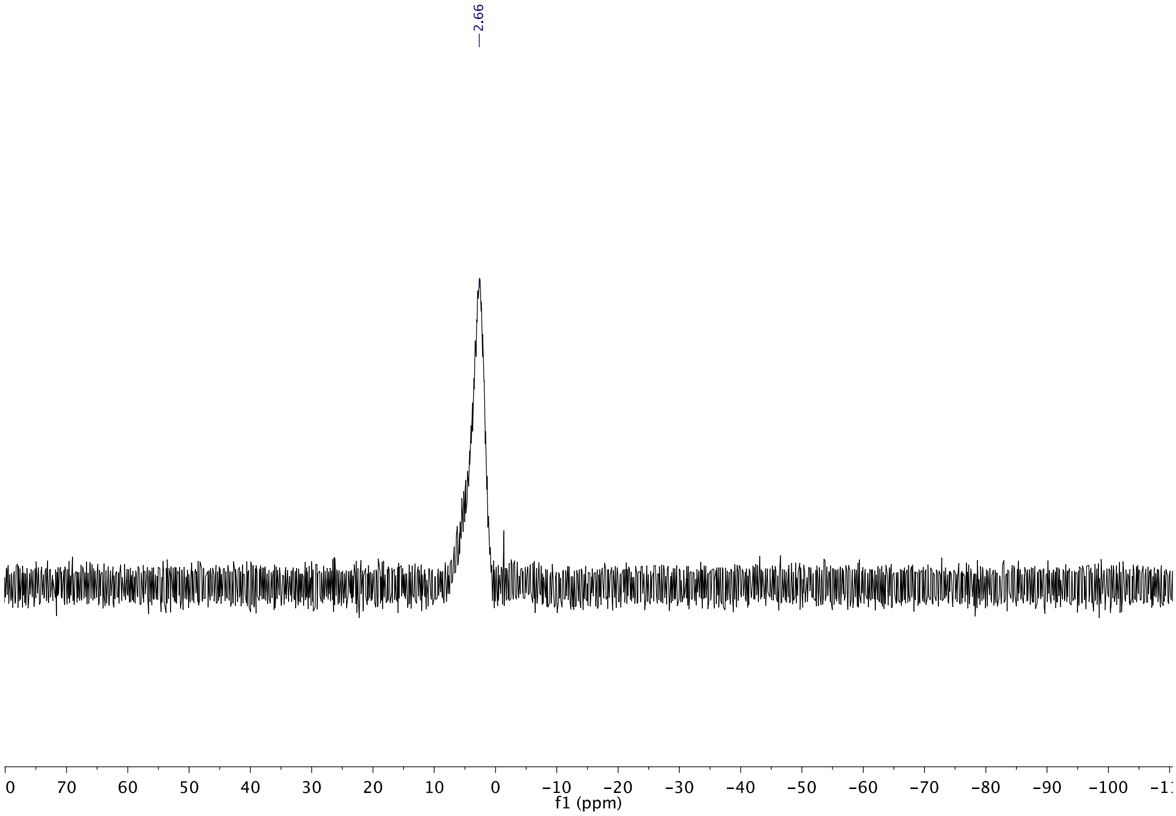

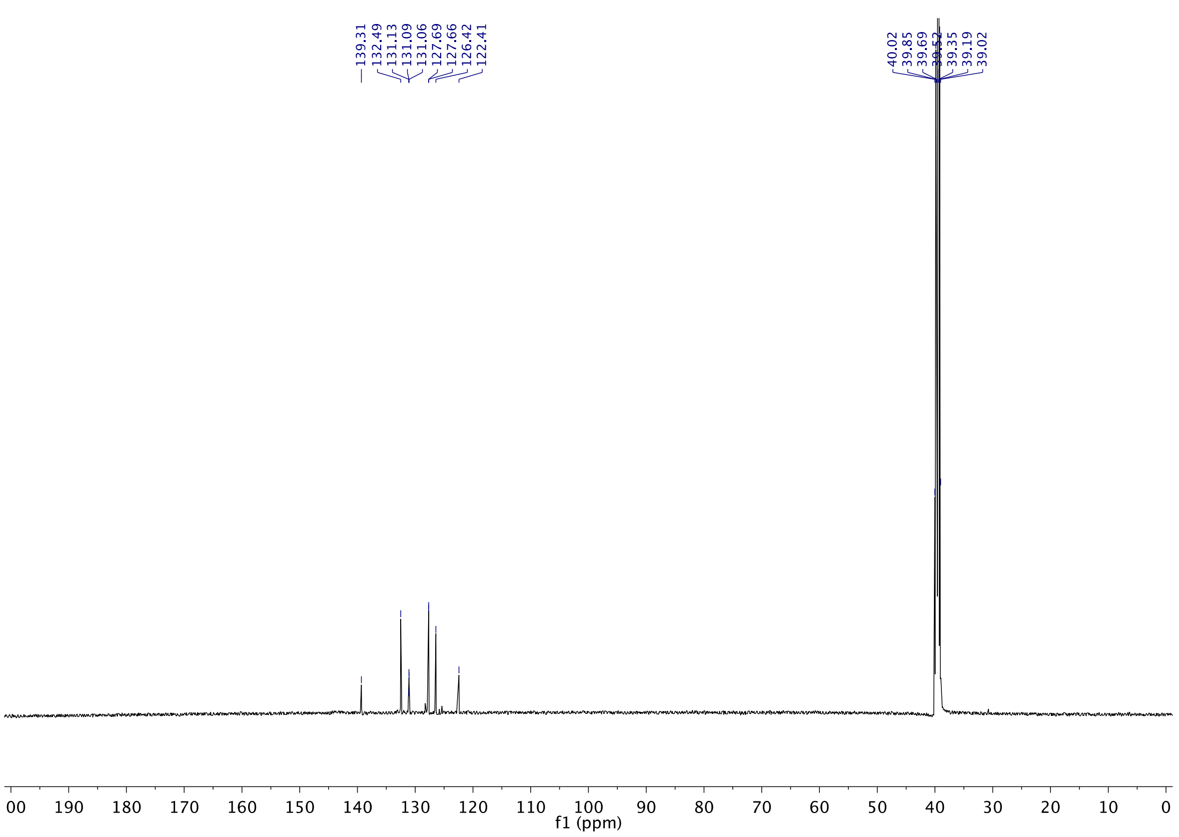

139.31
132.49
131.13
131.09
131.06
127.69
127.66
126.42
122.41
40.02
39.85
39.69
39.52
39.35
39.19
39.02
f1 (ppm)

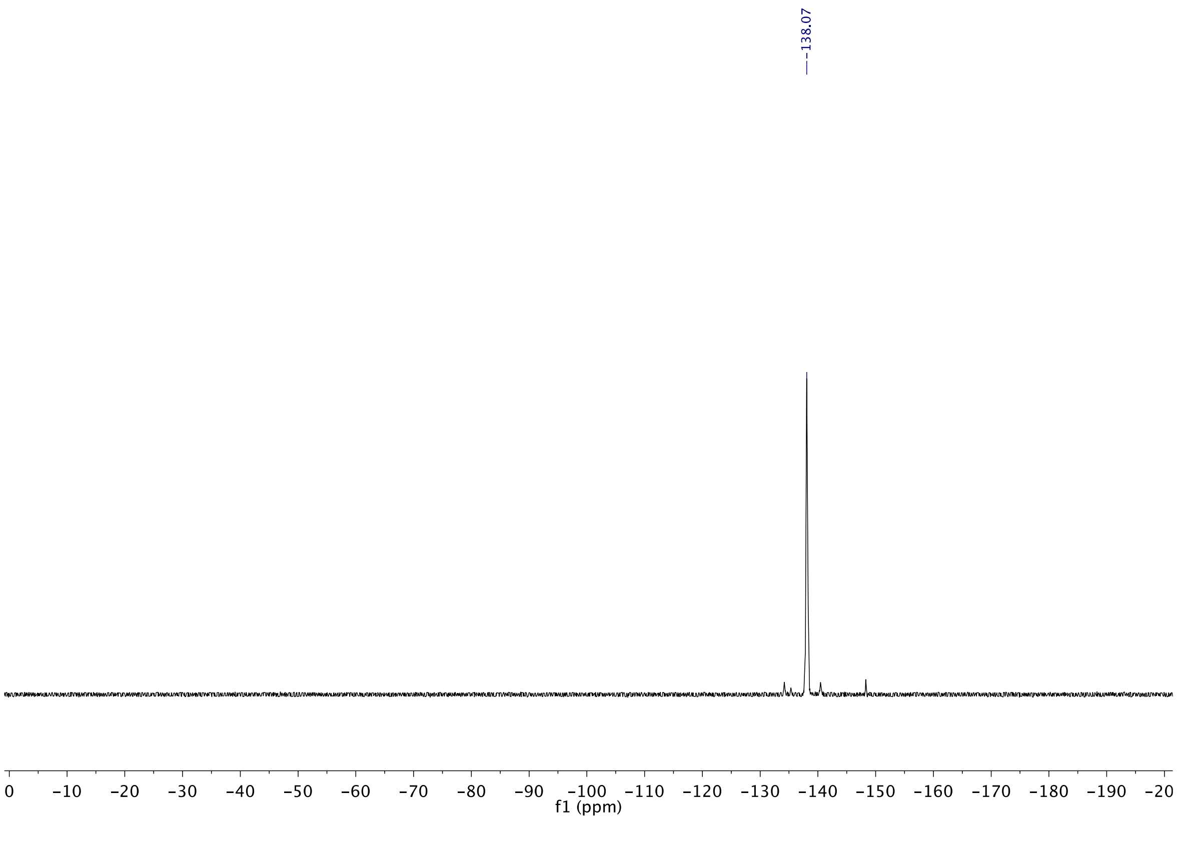

-138.07
f1 (ppm)

(*E*)-(2-Bromostyryl)boronic acid, **S6**

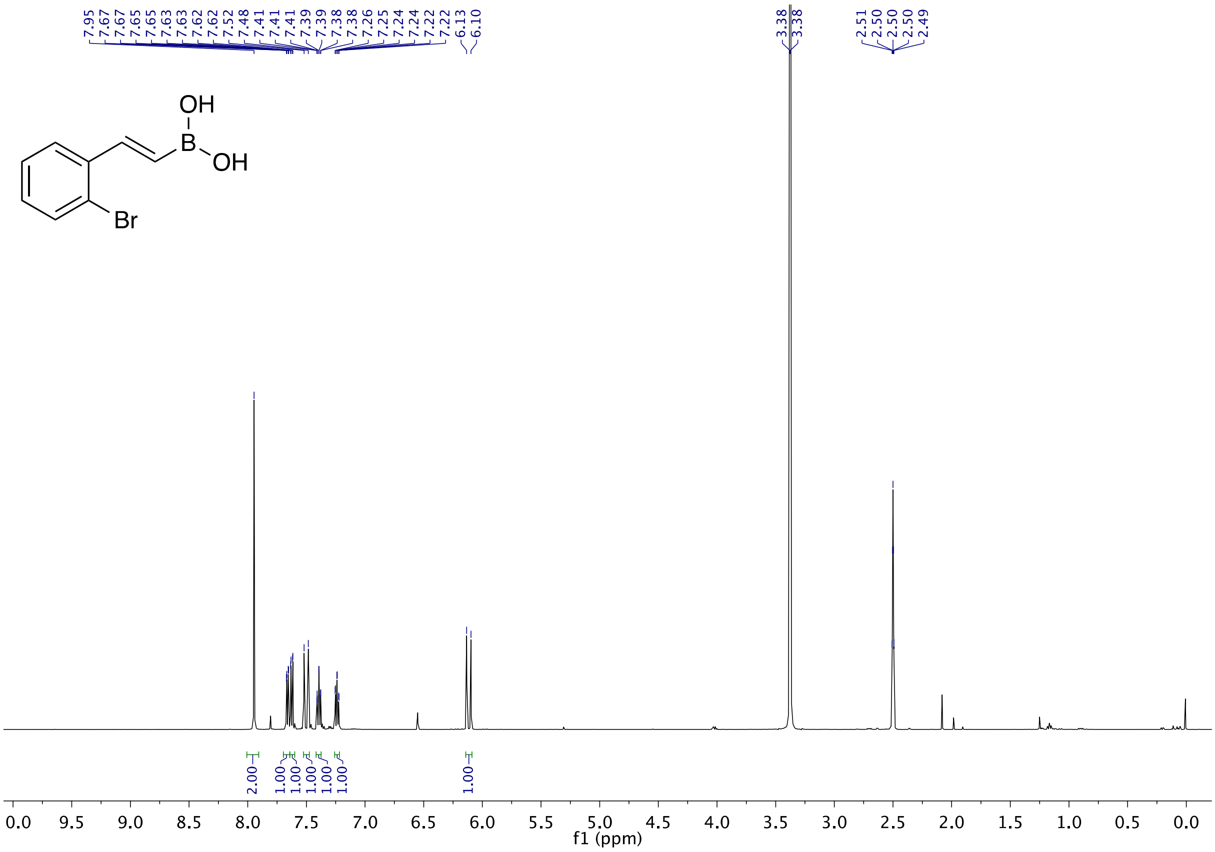

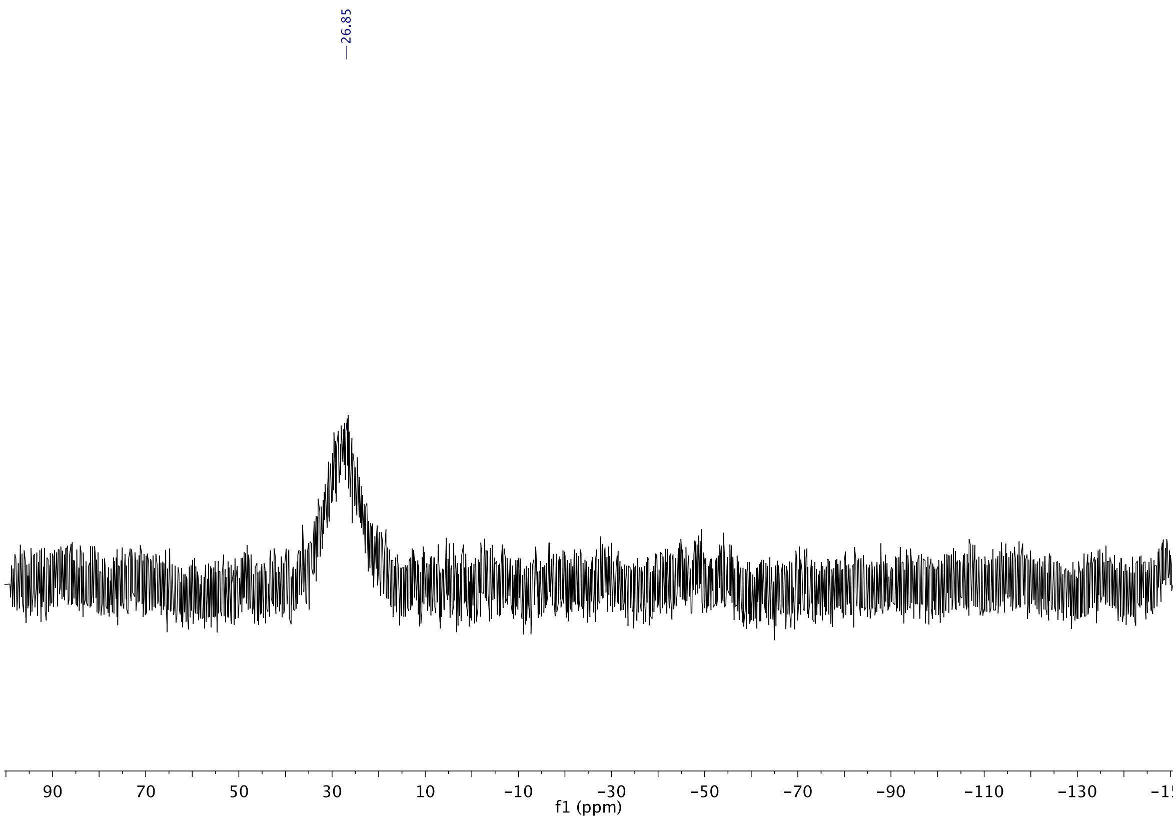

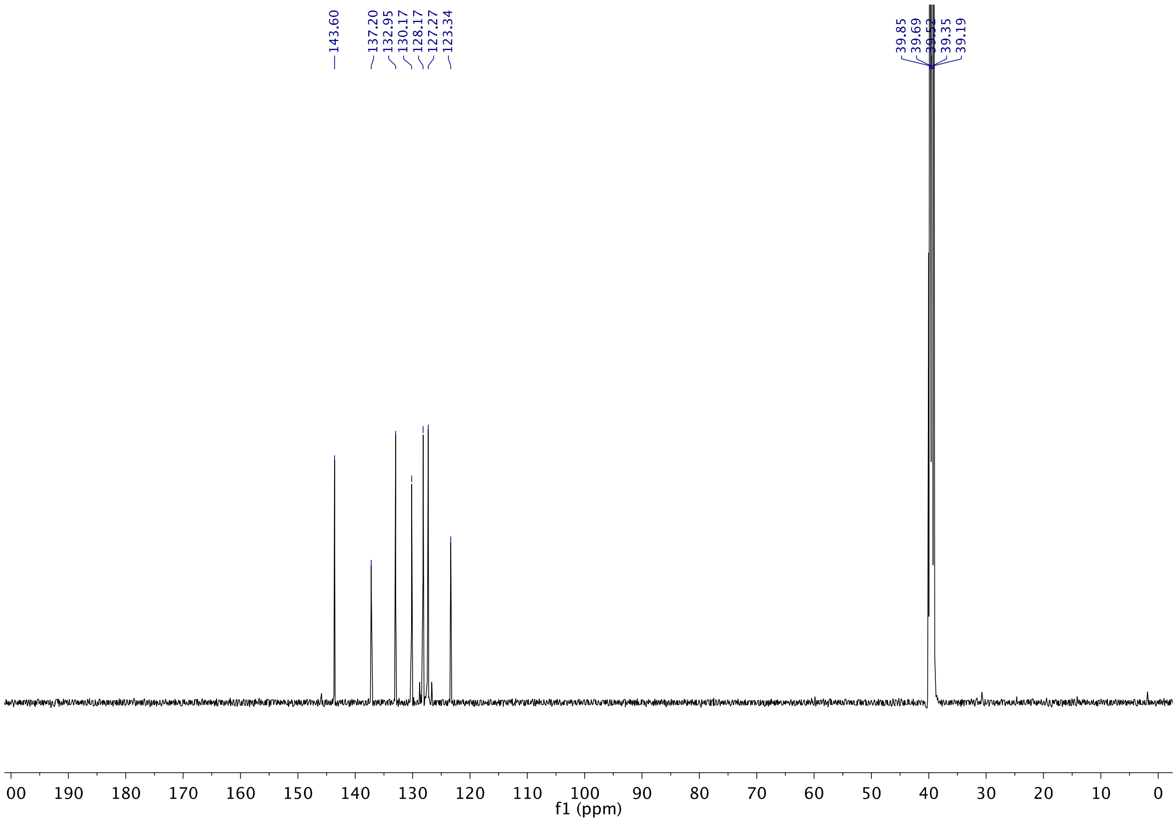

Trimethyl(naphthalen-1-ylethynyl)silane, **S7-int1**

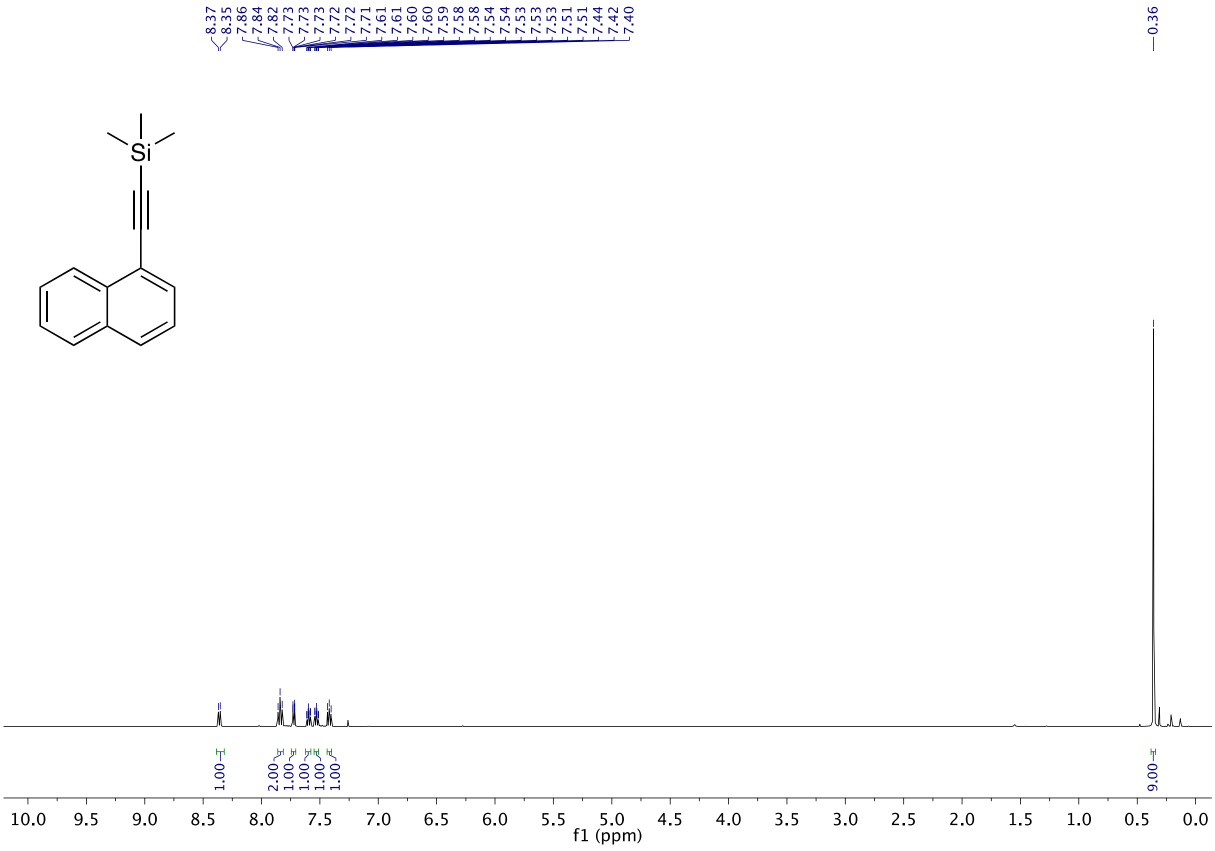

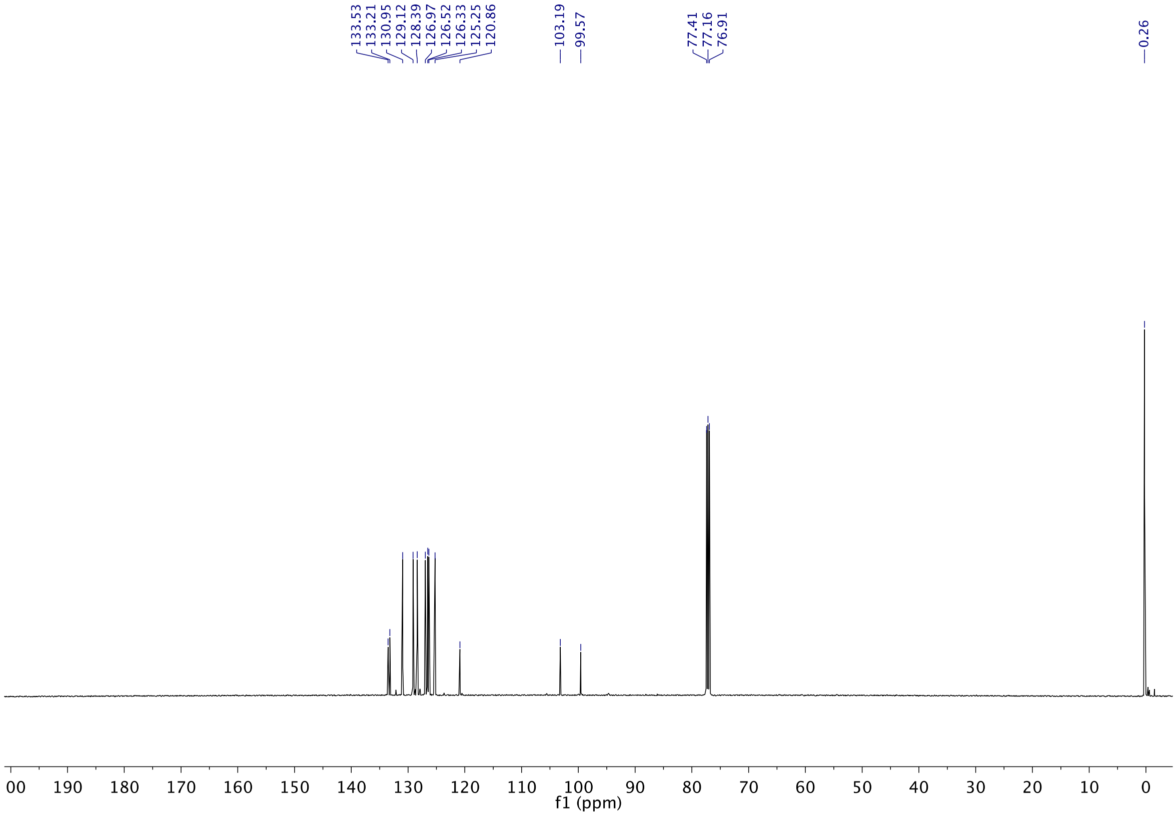

1-Ethynylnaphthalene, **S7-int2**

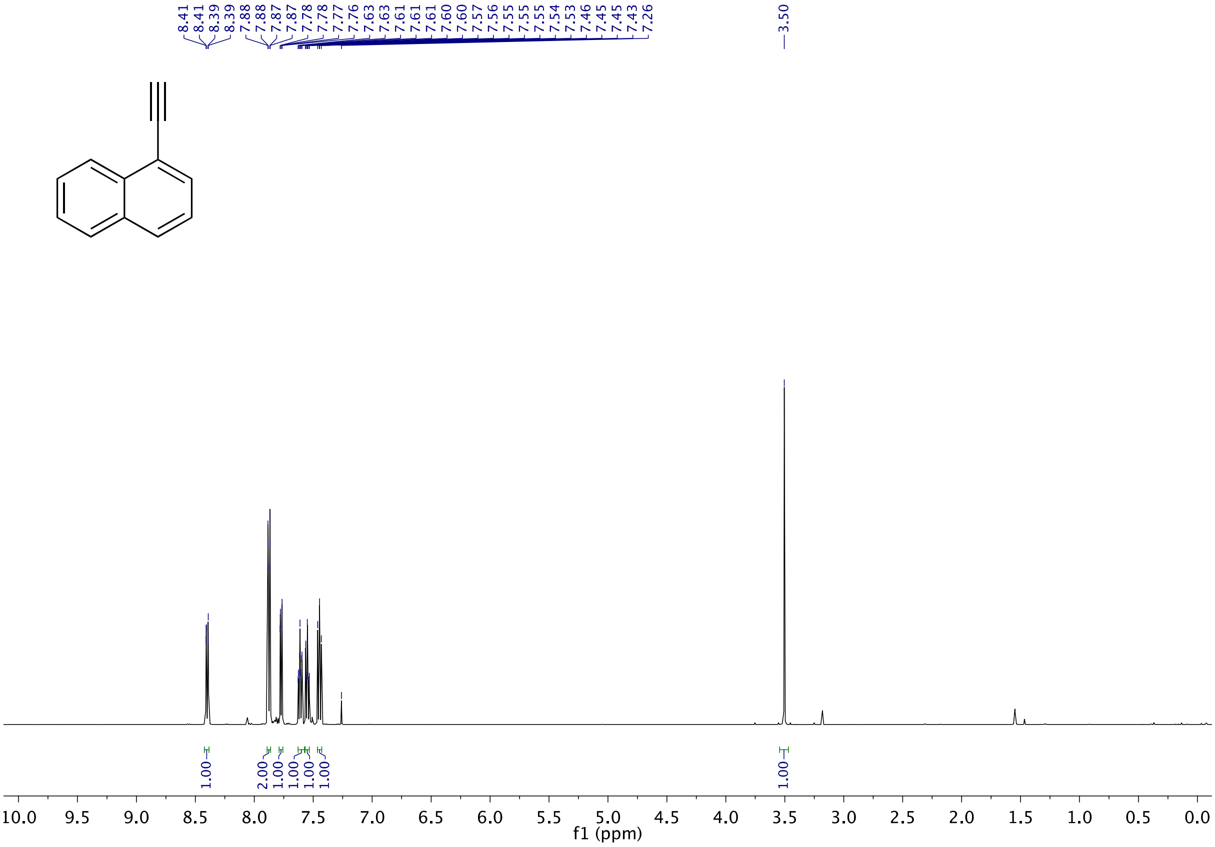

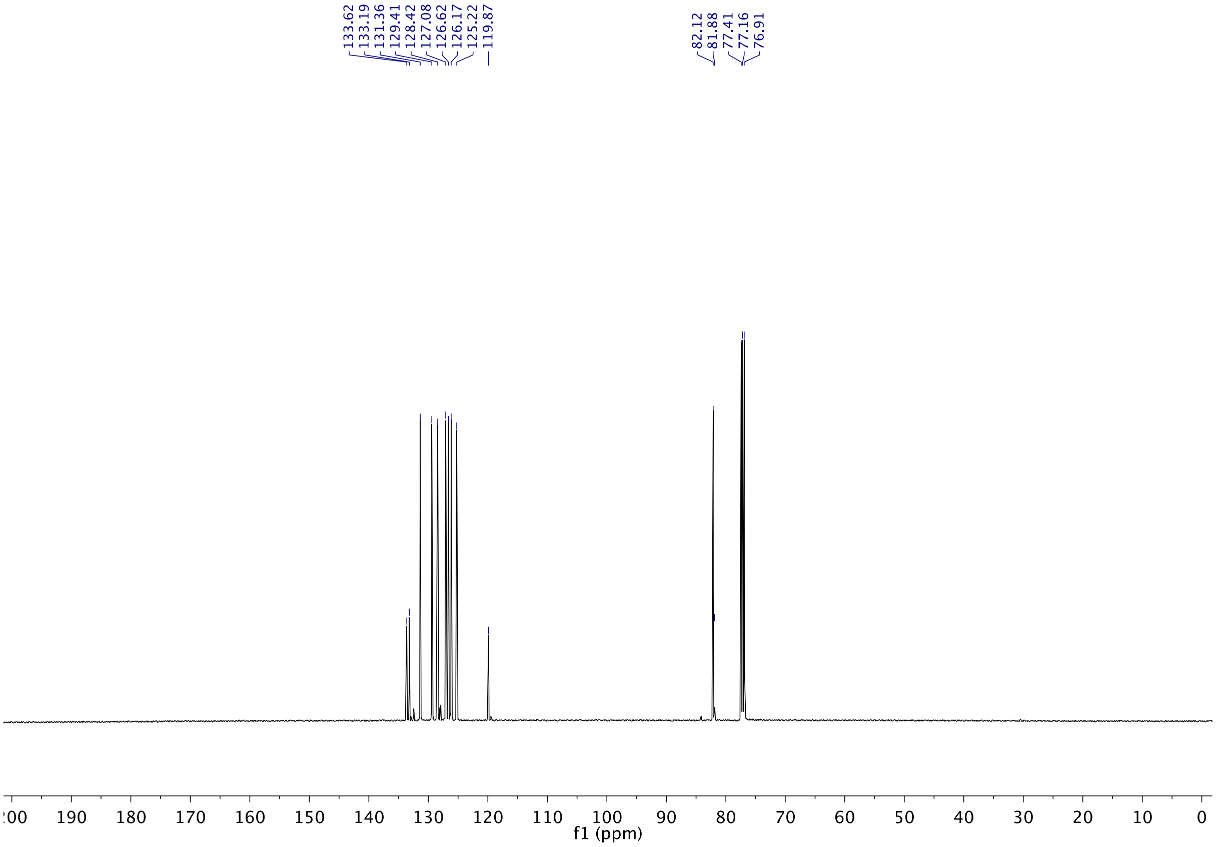

(*E*)-4,4,5,5-Tetramethyl-2-(2-(naphthalen-1-yl)vinyl)-1,3,2-dioxaborolane, **S7-int3**

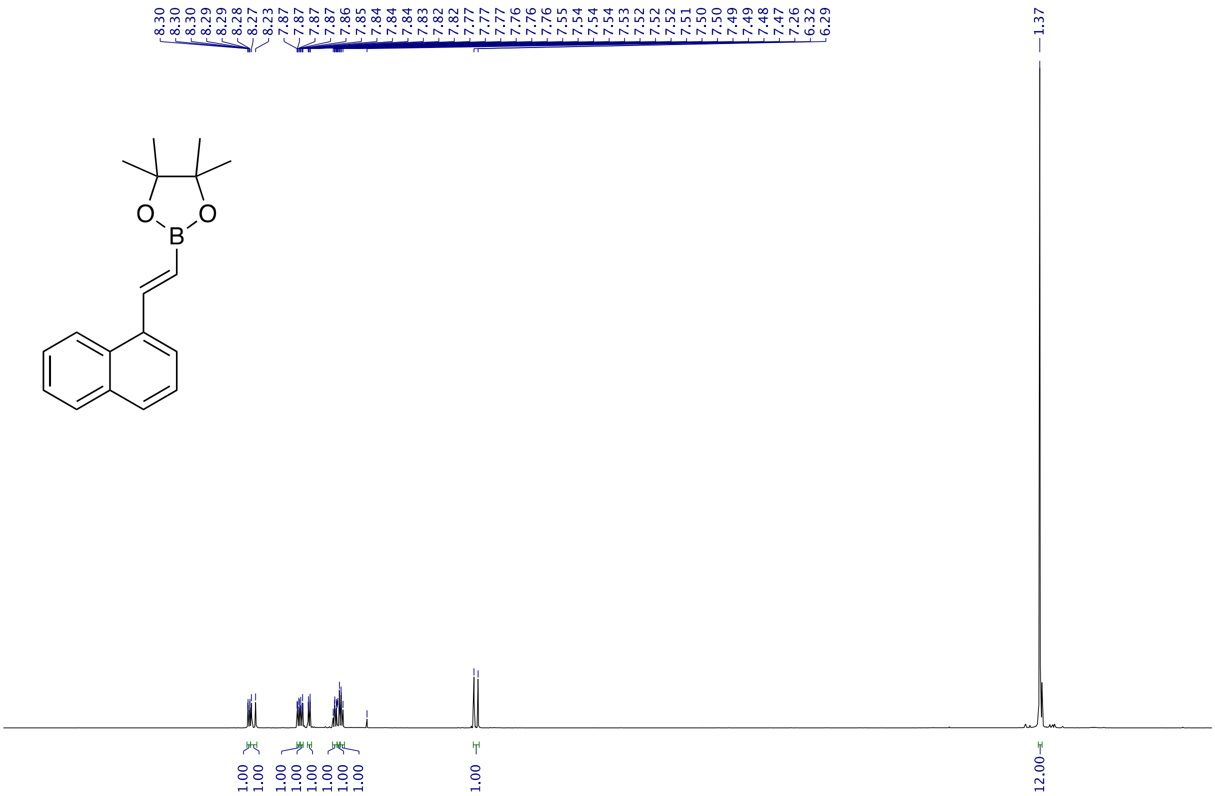

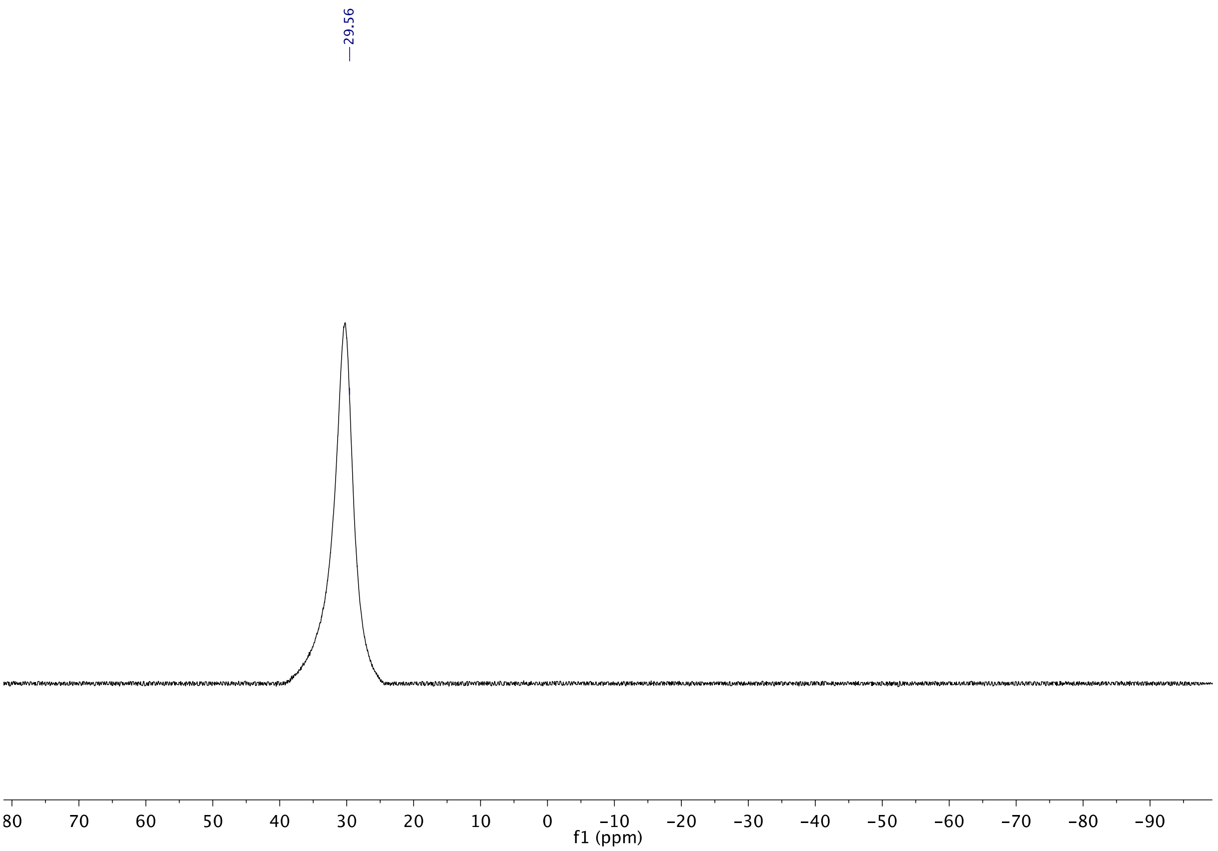
29.56
80 70 60 50 40 30 20 10 0 −10 −20 −30 −40 −50 −60 −70 −80 −90
f1 (ppm)

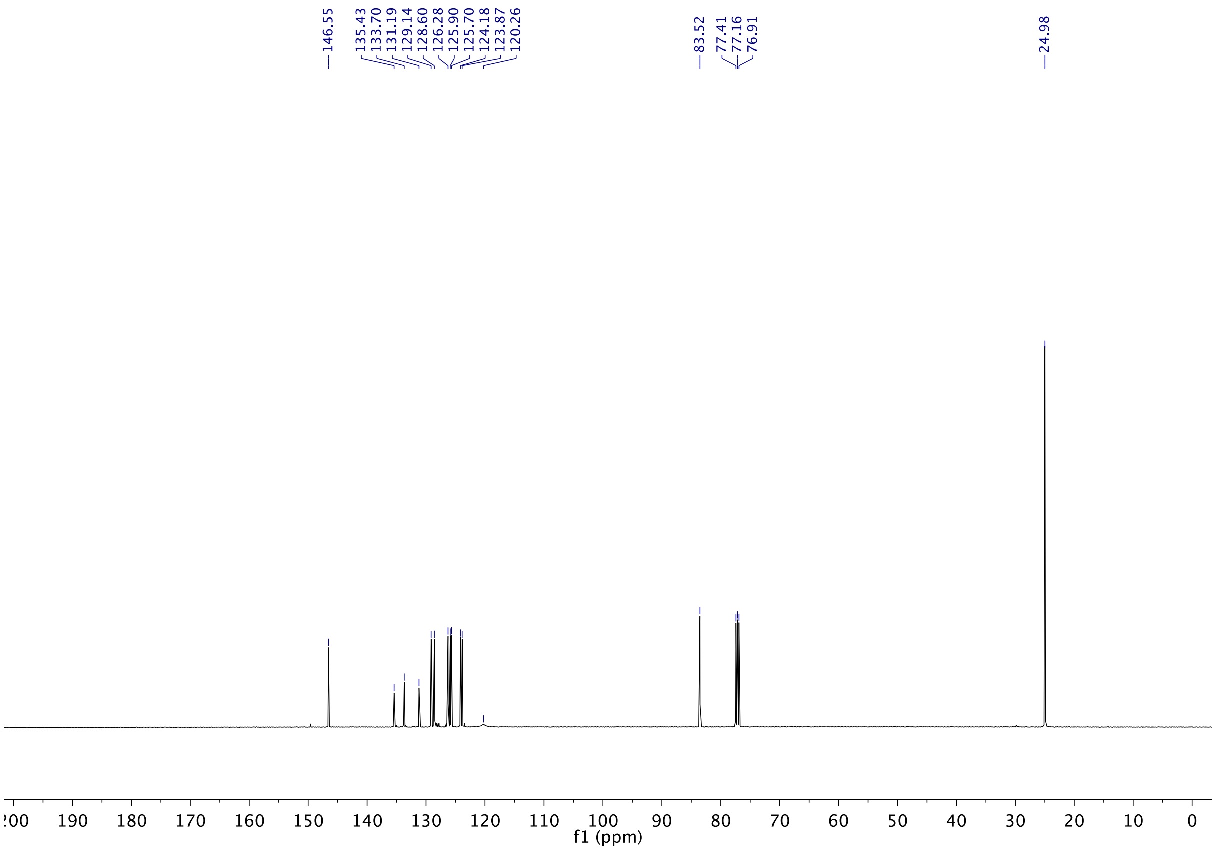
146.55
135.43
133.70
131.19
129.14
128.60
126.28
125.90
125.70
124.18
123.87
120.26
83.52
77.41
77.16
76.91
24.98
200 190 180 170 160 150 140 130 120 110 100 90 80 70 60 50 40 30 20 10 0
f1 (ppm)

(*E*)-Trifluoro(2-(naphthalen-1-yl)vinyl)-λ^4-borane, potassium salt, **S7-int4**

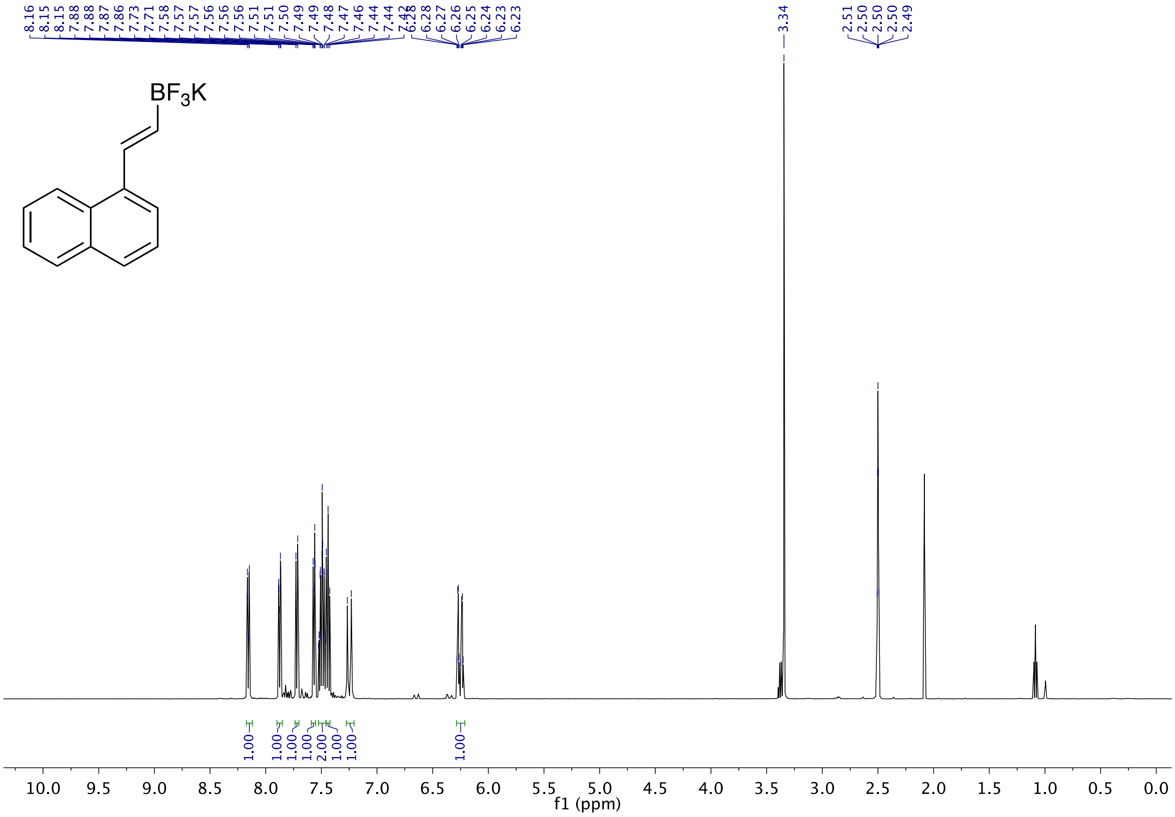

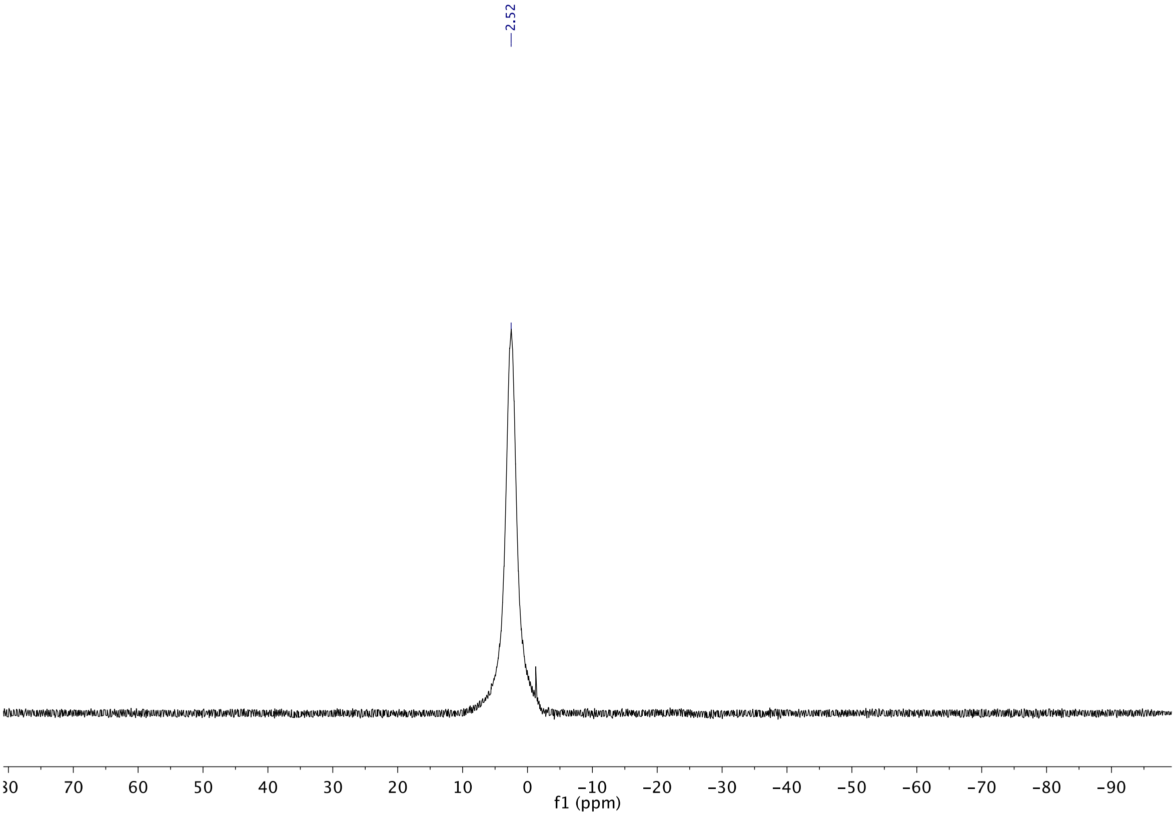

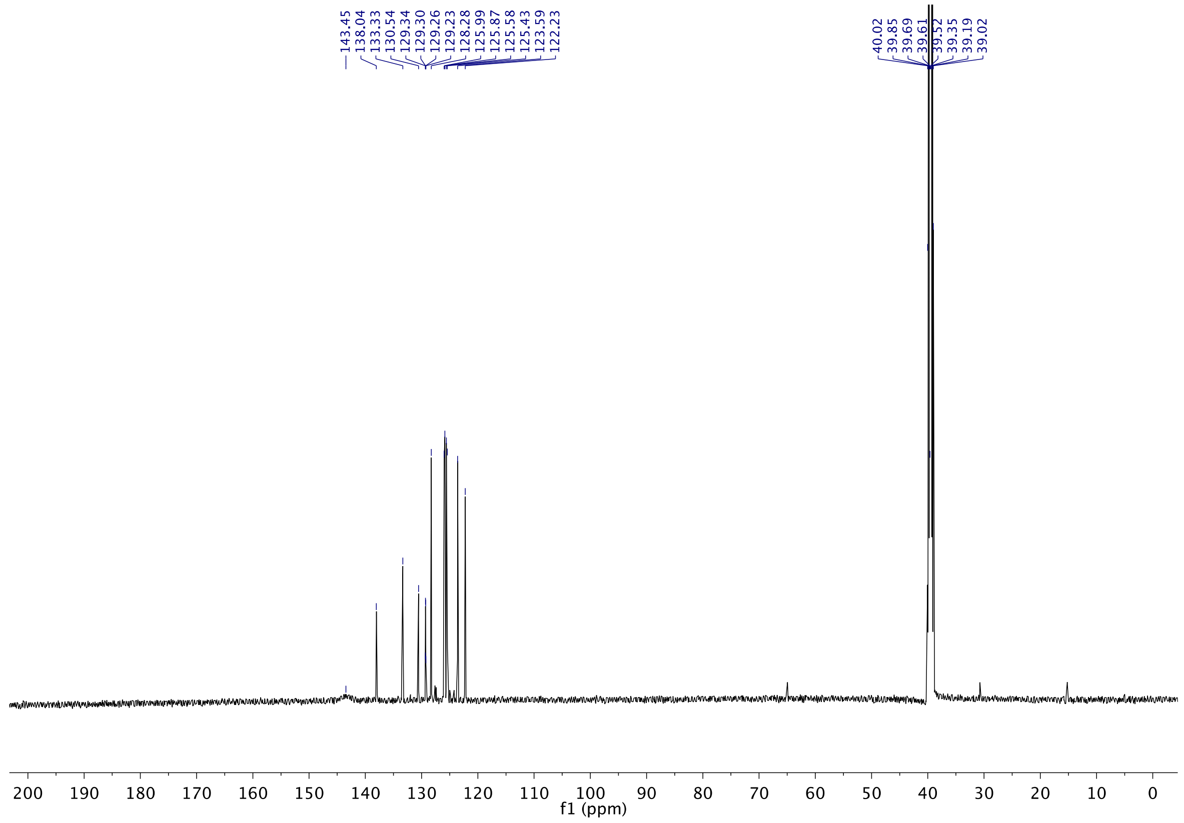
143.45
138.04
133.33
130.54
129.34
129.30
129.26
129.23
128.28
125.99
125.87
125.58
125.43
123.59
122.23
40.02
39.85
39.69
39.61
39.52
39.35
39.19
39.02
200 190 180 170 160 150 140 130 120 110 100 90 80 70 60 50 40 30 20 10 0
f1 (ppm)

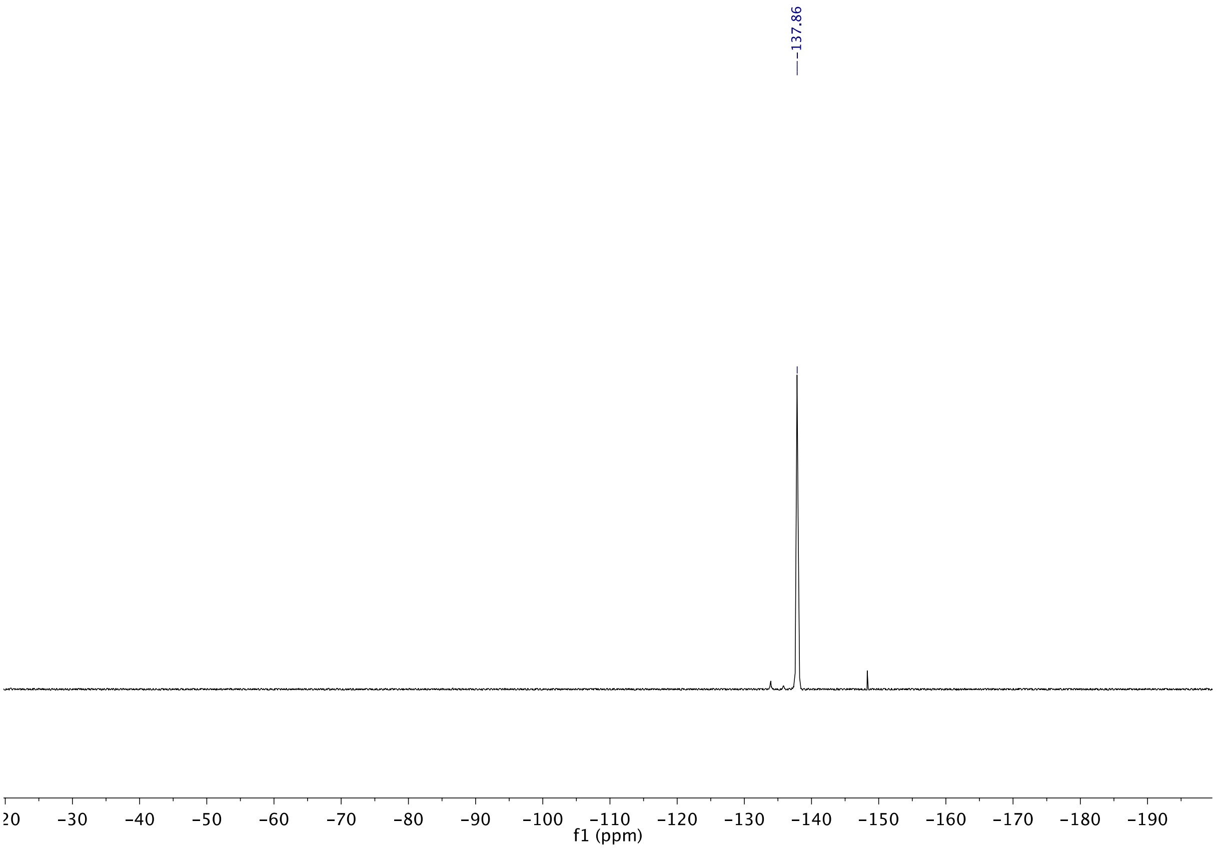
-137.86
-30 -40 -50 -60 -70 -80 -90 -100 -110 -120 -130 -140 -150 -160 -170 -180 -190
f1 (ppm)

(*E*)-(2-(Naphthalen-1-yl)vinyl)boronic acid, **S7**

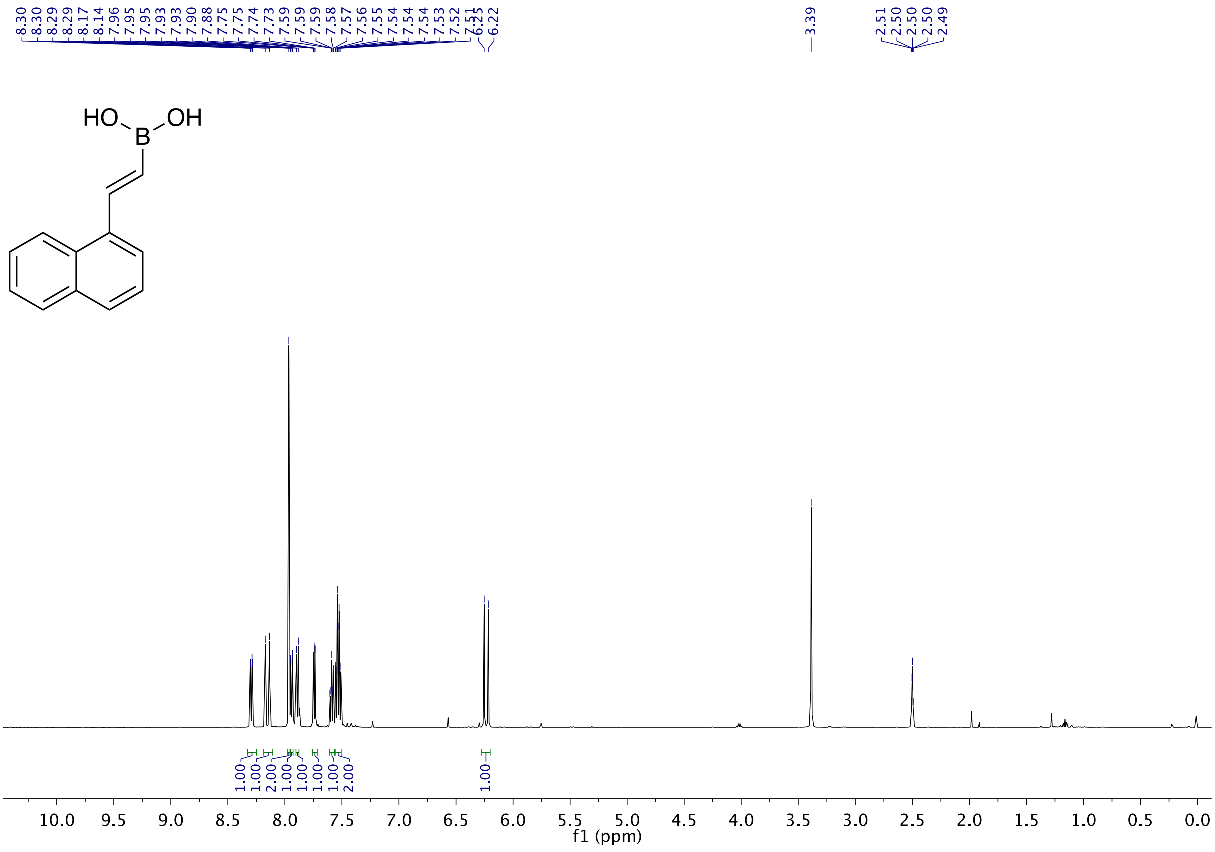

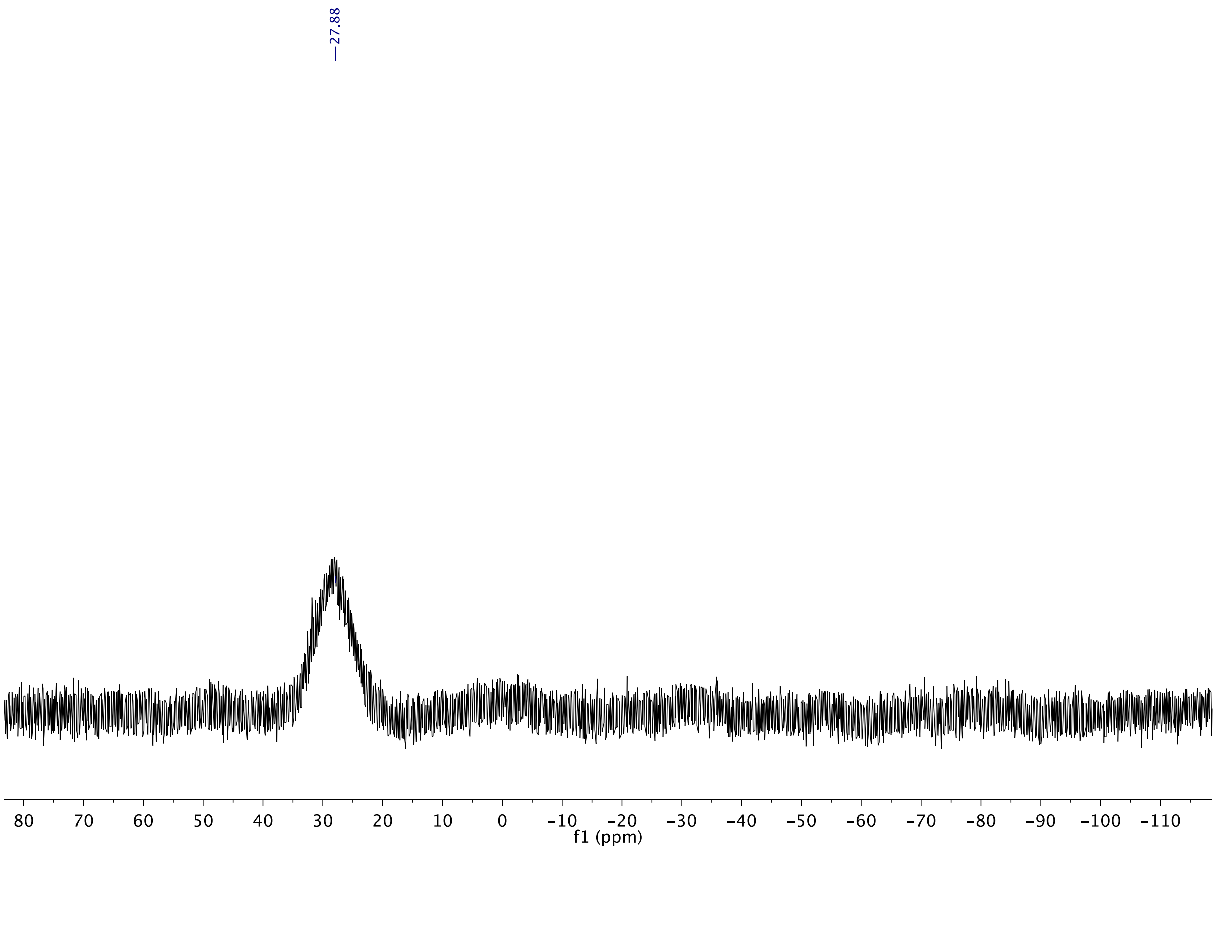

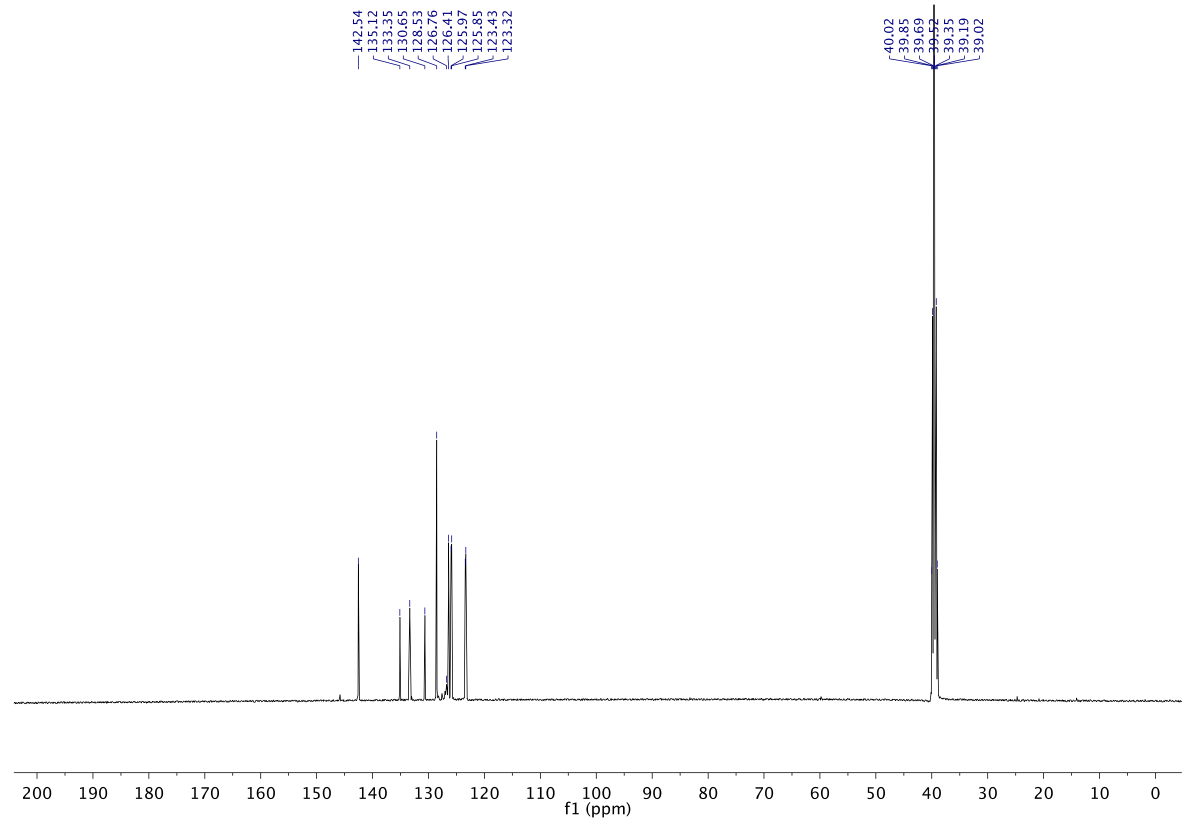

(*E*)-2-(2-(4,4,5,5-Tetramethyl-1,3,2-dioxaborolan-2-yl)vinyl)pyridine, **S8-int3**

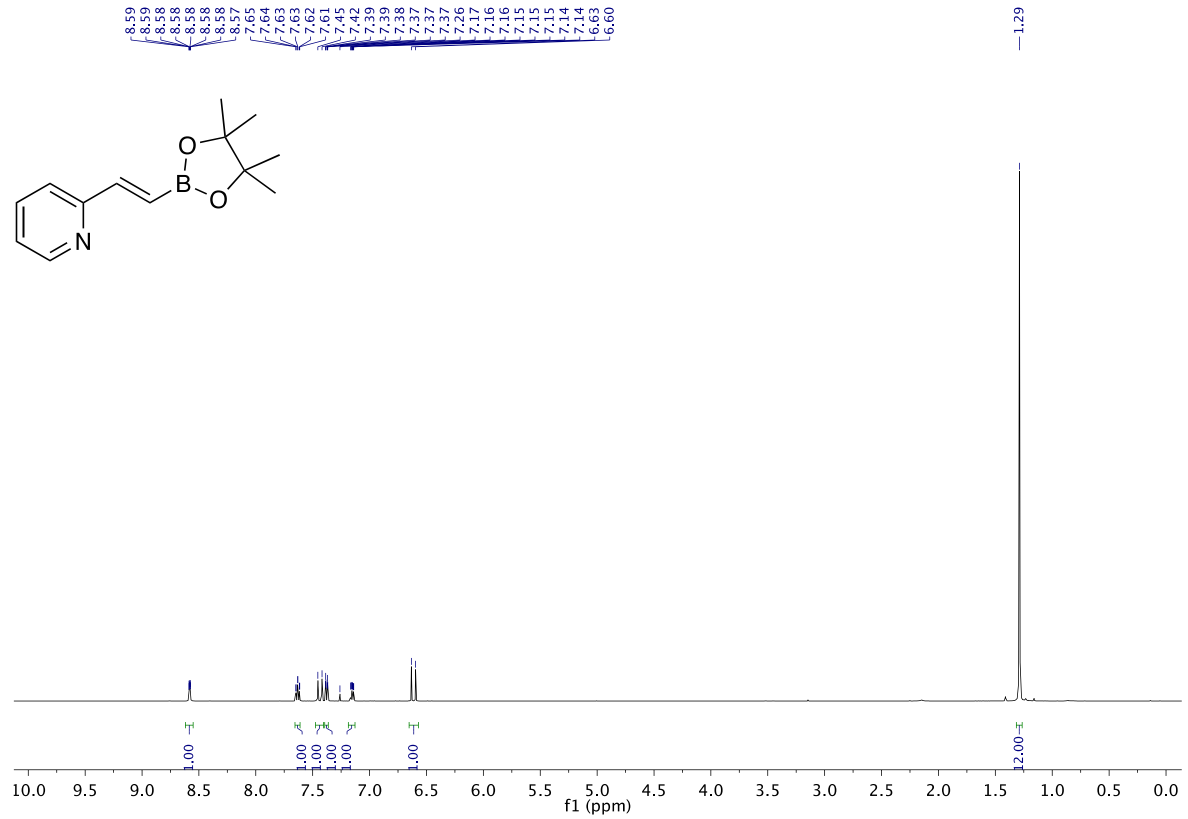

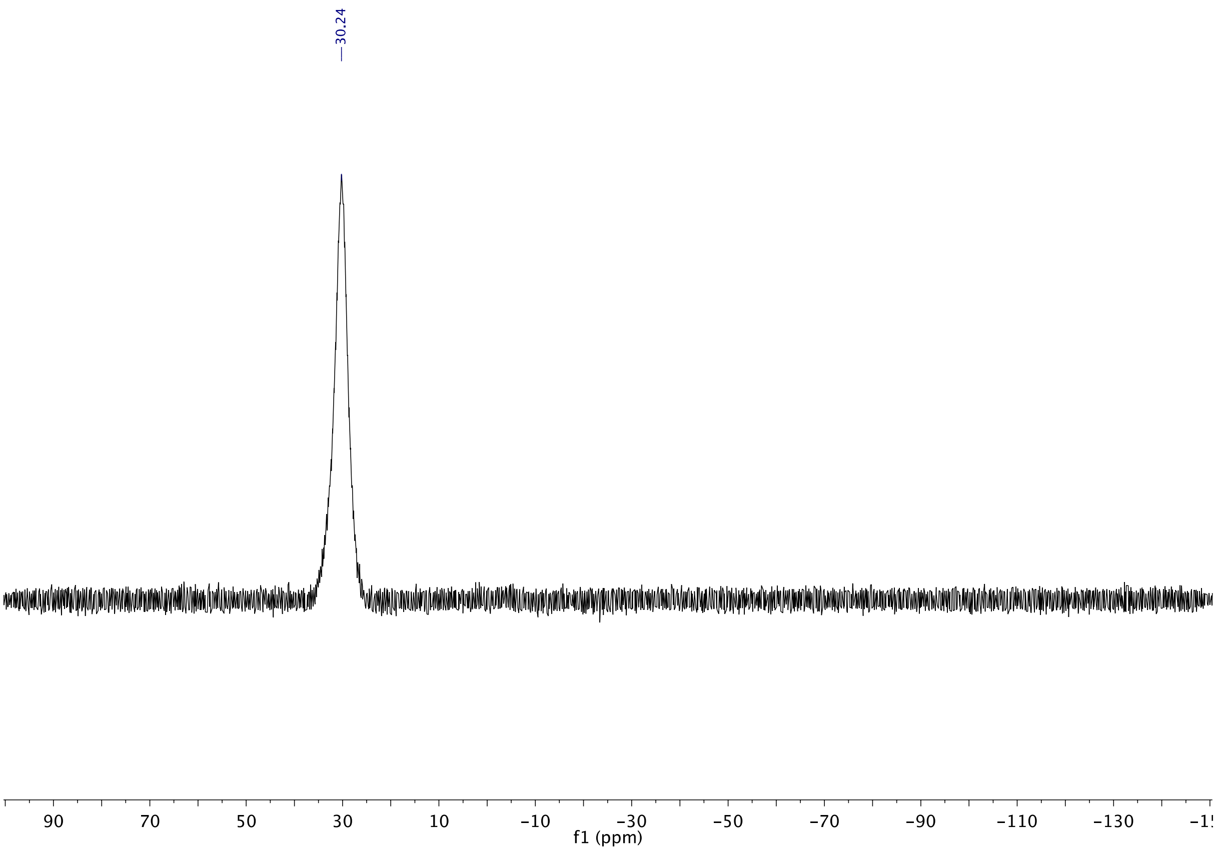
30.24
90 70 50 30 10 −10 −30 −50 −70 −90 −110 −130
f1 (ppm)

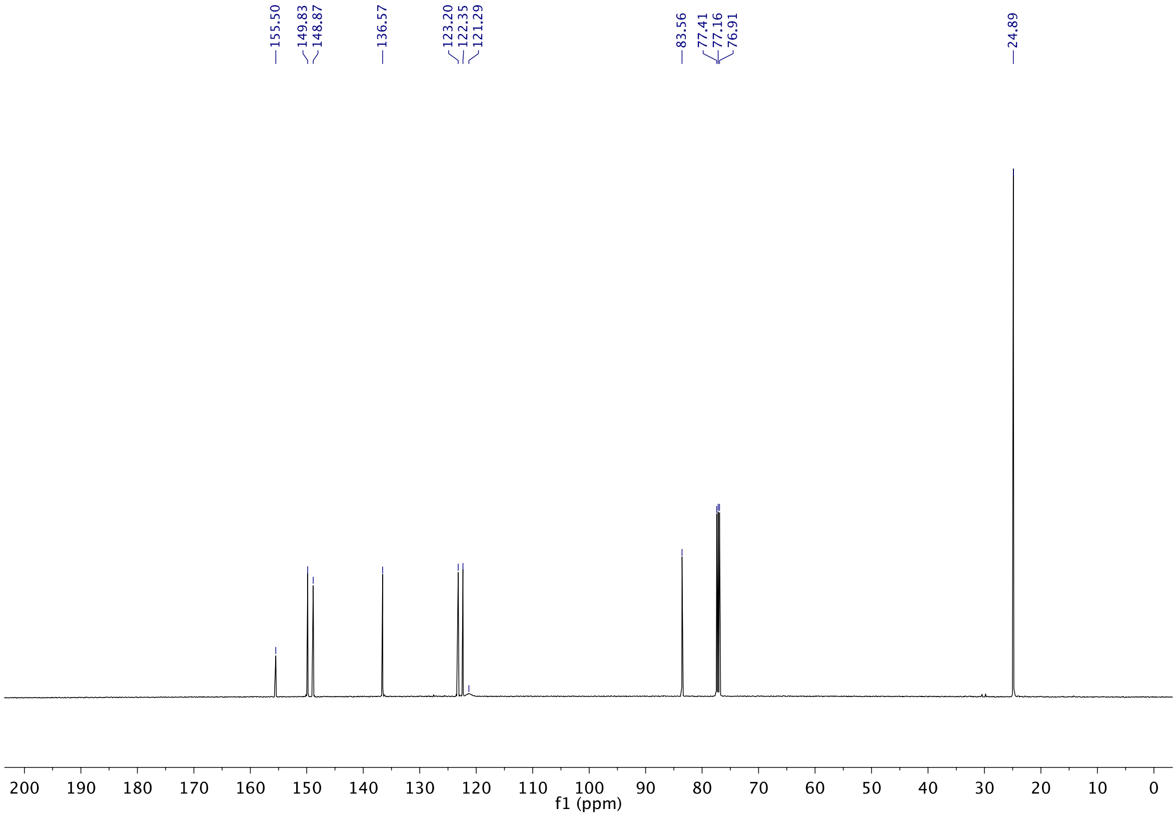
155.50
149.83
148.87
136.57
123.20
122.35
121.29
83.56
77.41
77.16
76.91
24.89
200 190 180 170 160 150 140 130 120 110 100 90 80 70 60 50 40 30 20 10 0
f1 (ppm)

(*E*)-2-(2-(Trifluoro-λ^4-boraneyl)vinyl)pyridine, potassium salt, **S8-int4**

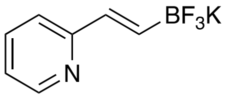

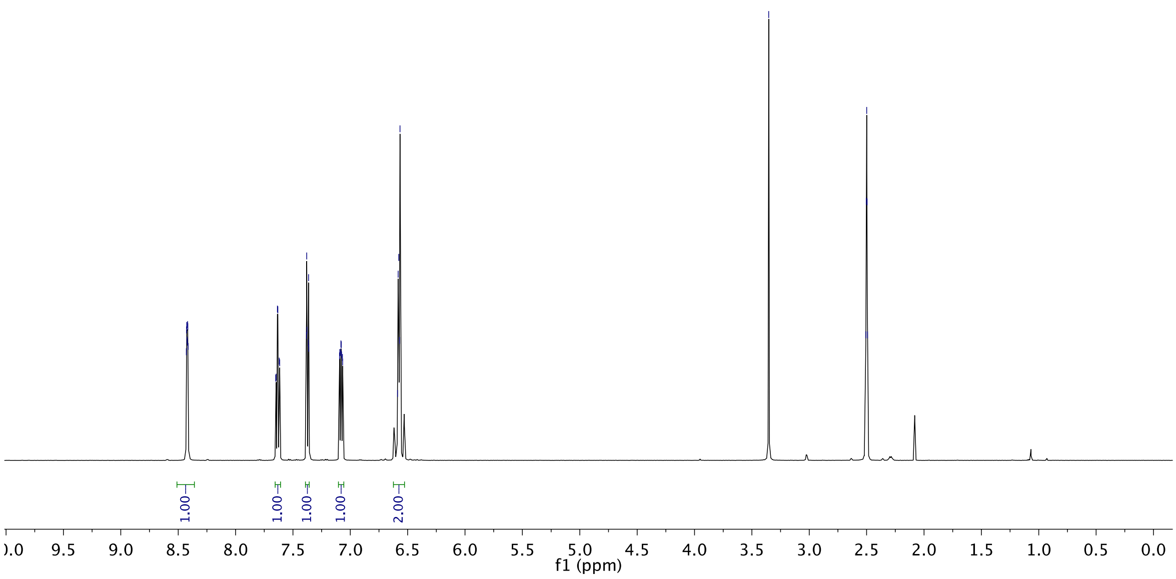

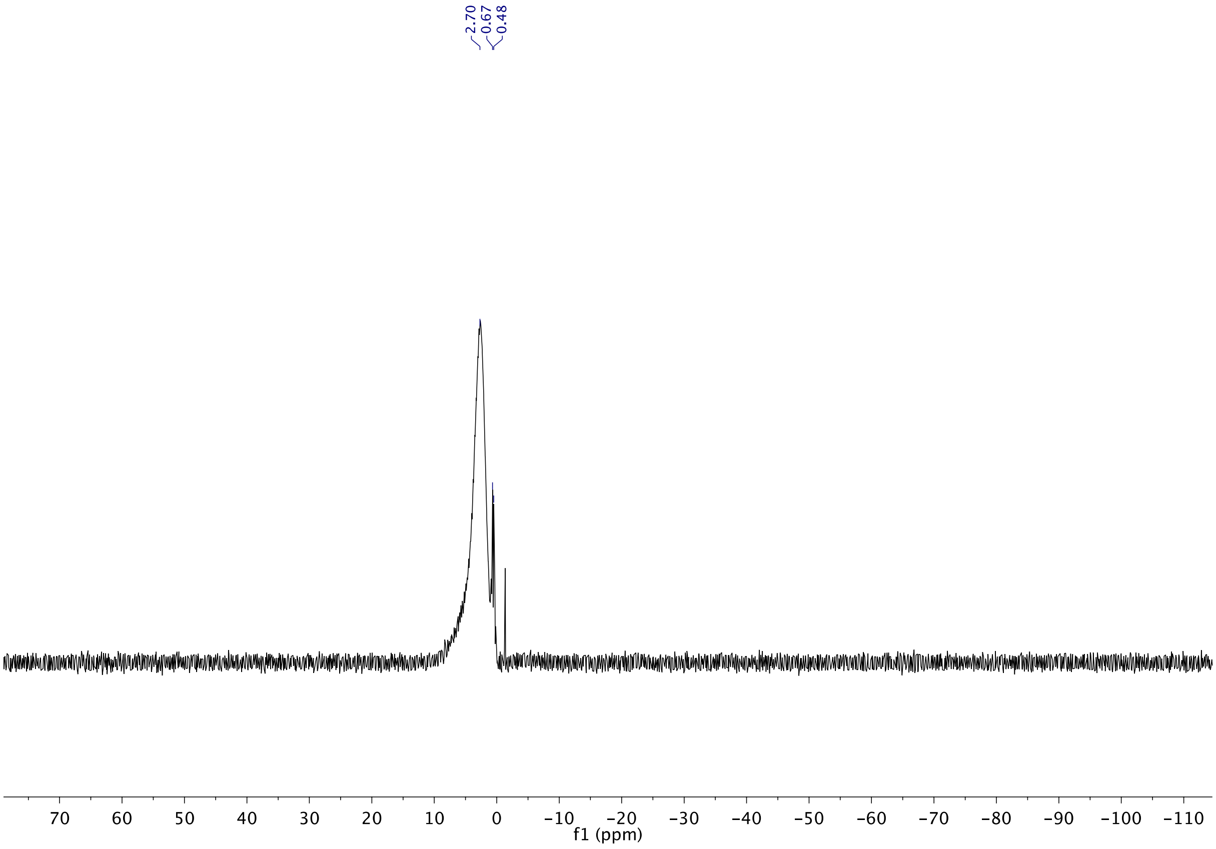

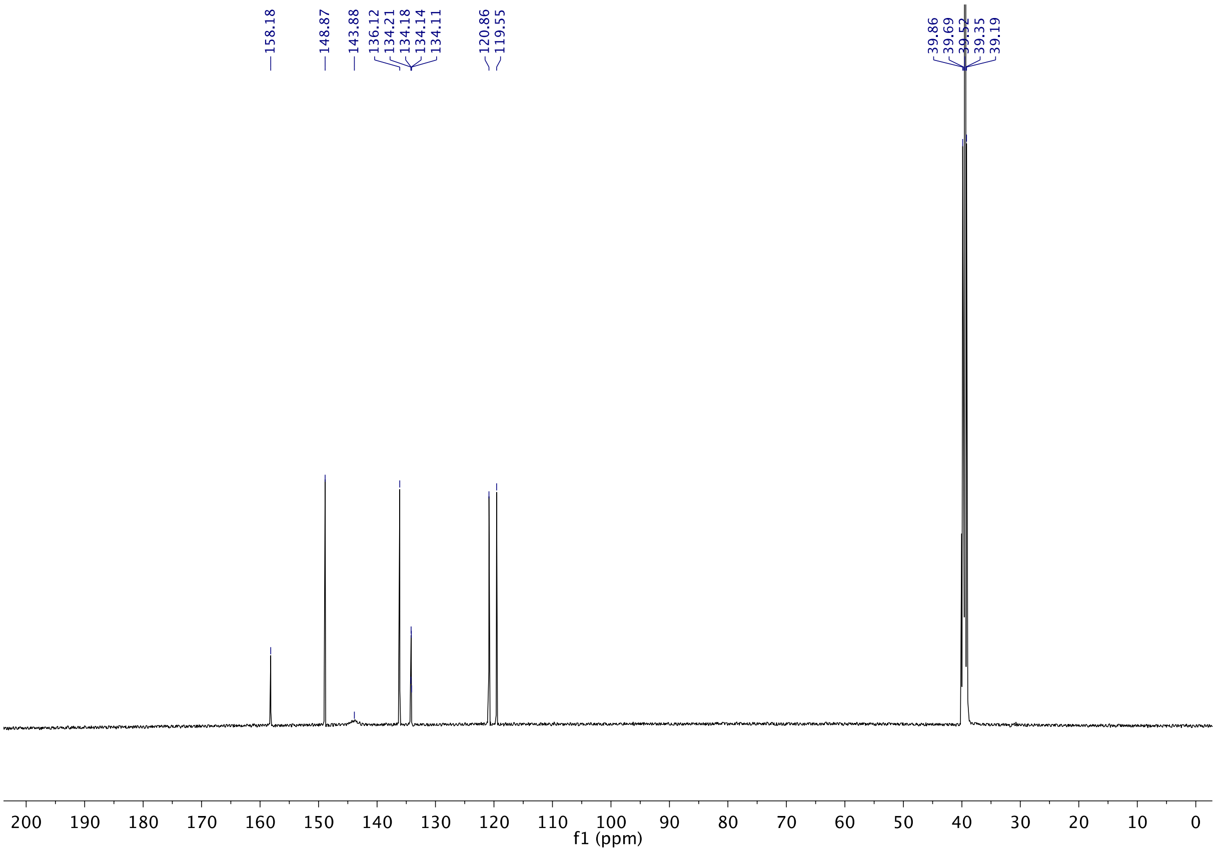
158.18
148.87
143.88
136.12
134.21
134.18
134.14
134.11
120.86
119.55
39.86
39.69
39.52
39.35
39.19
f1 (ppm)

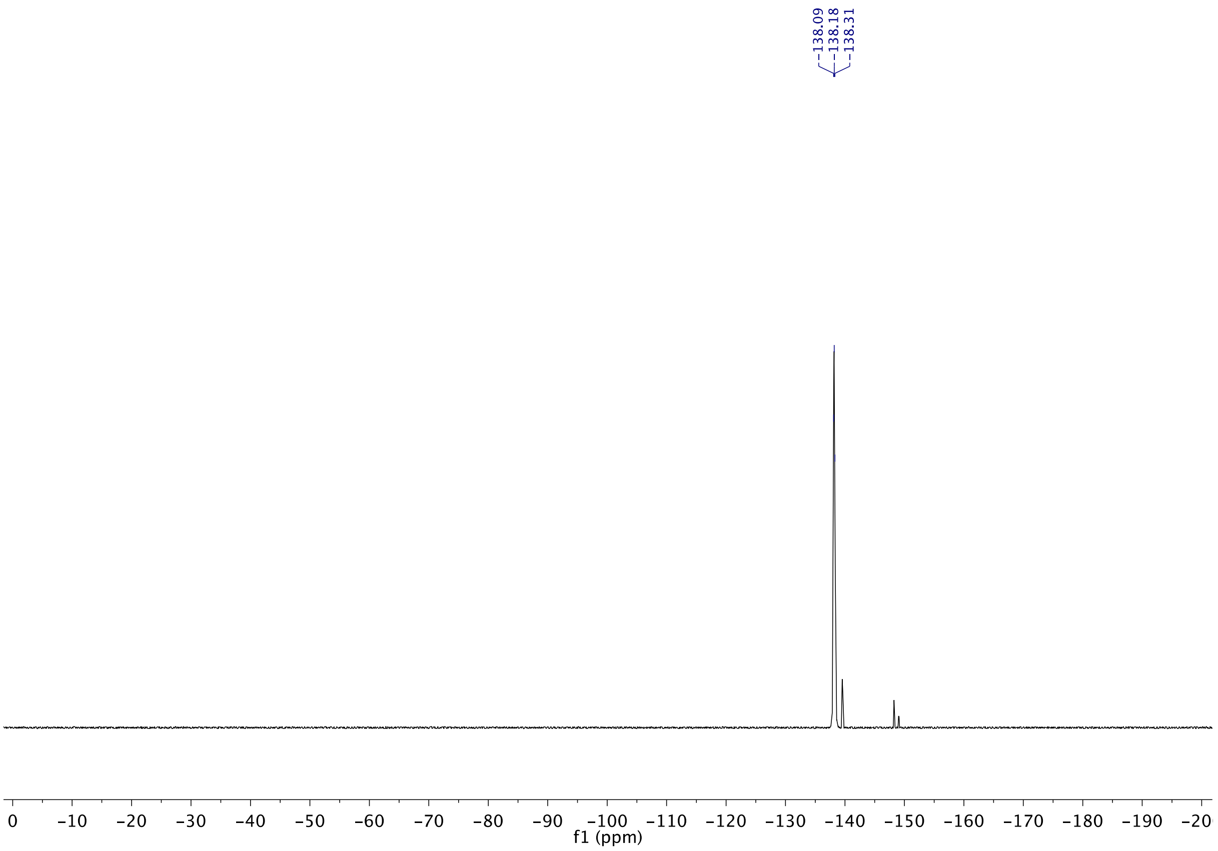
-138.09
-138.18
-138.31
f1 (ppm)

(*E*)-(2-(pyridin-2-yl)vinyl)boronic acid, **S8**

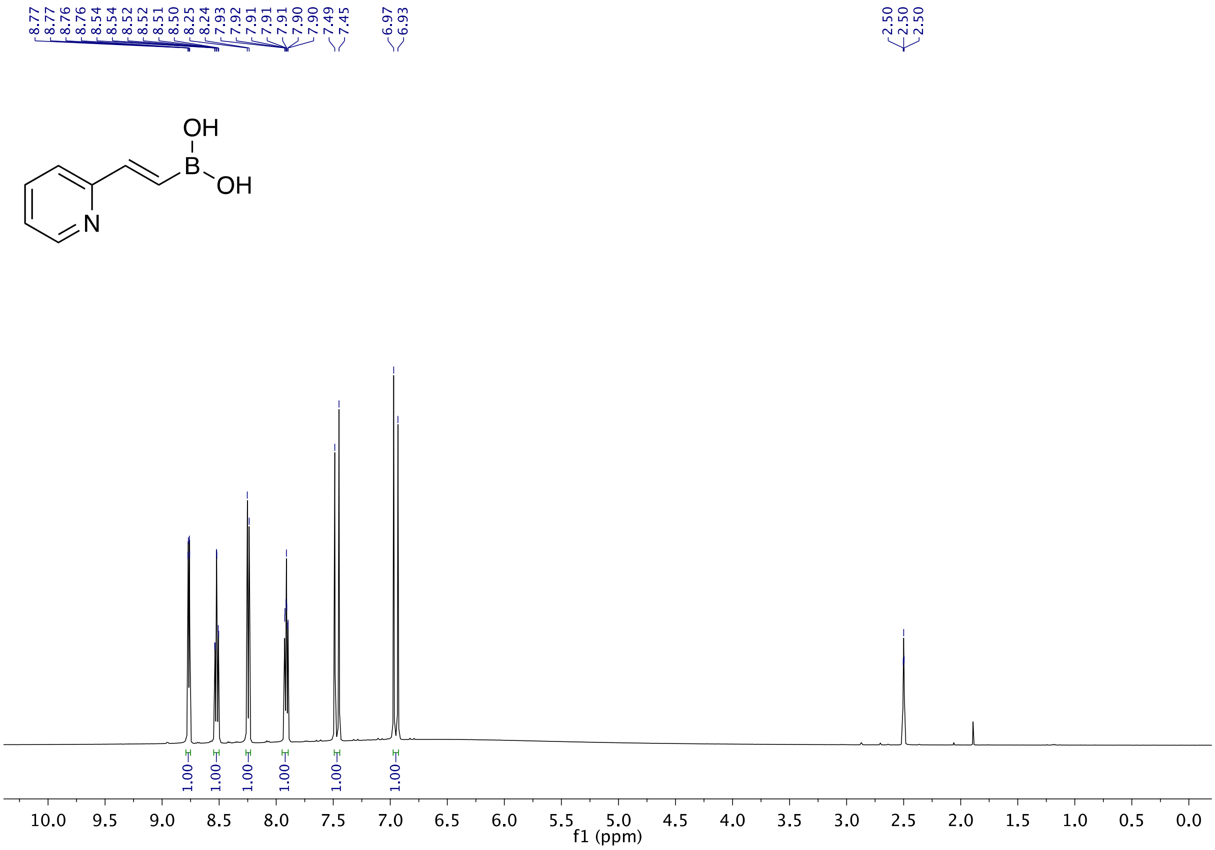

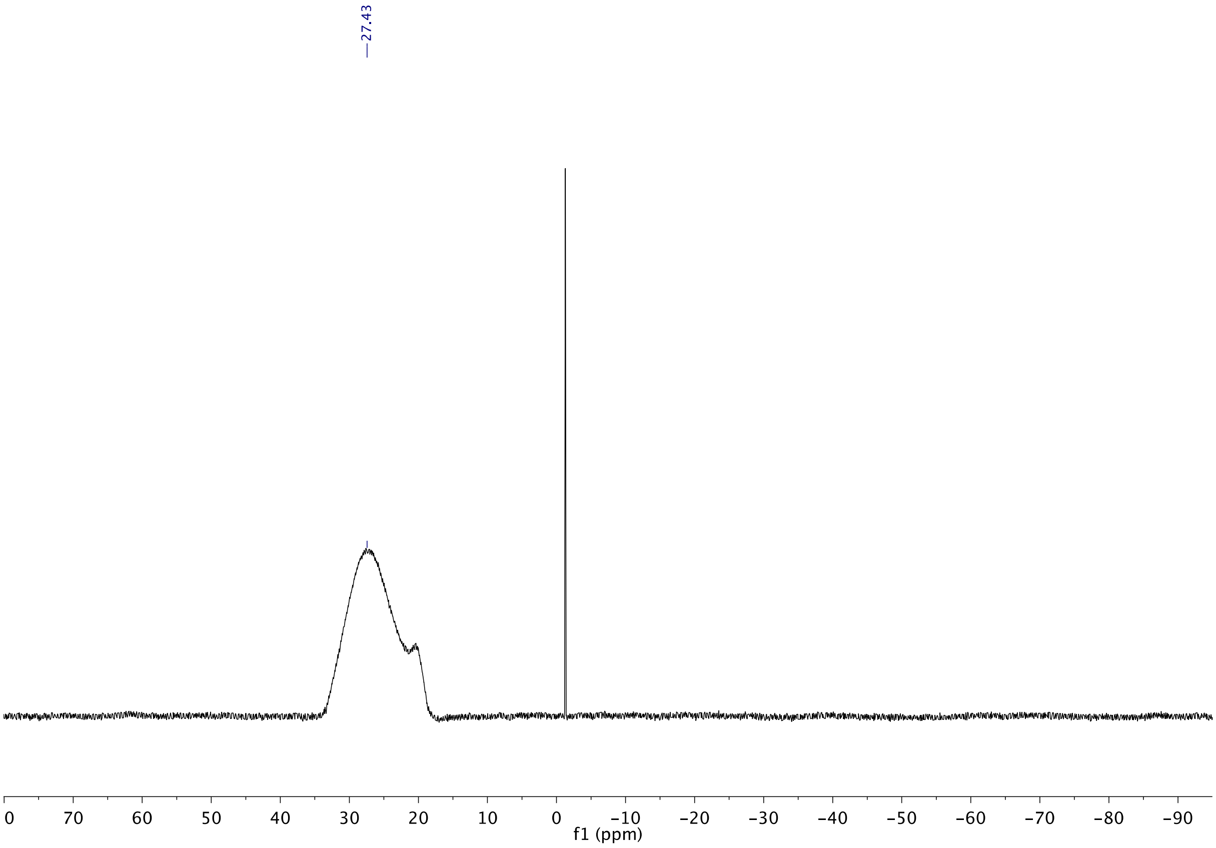

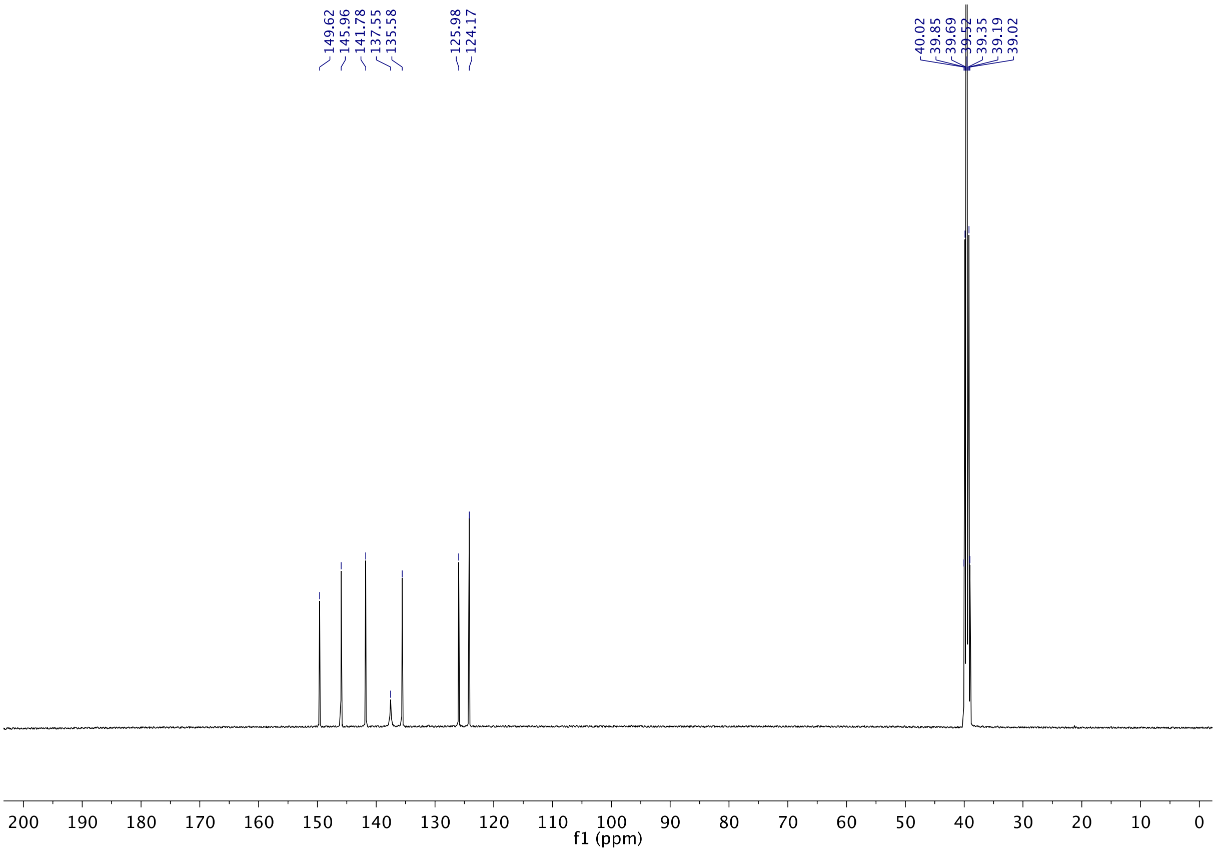

(*E*)-4,4,5,5-Tetramethyl-2-(2-(thiophen-2-yl)vinyl)-1,3,2-dioxaborolane, **S9-int3**

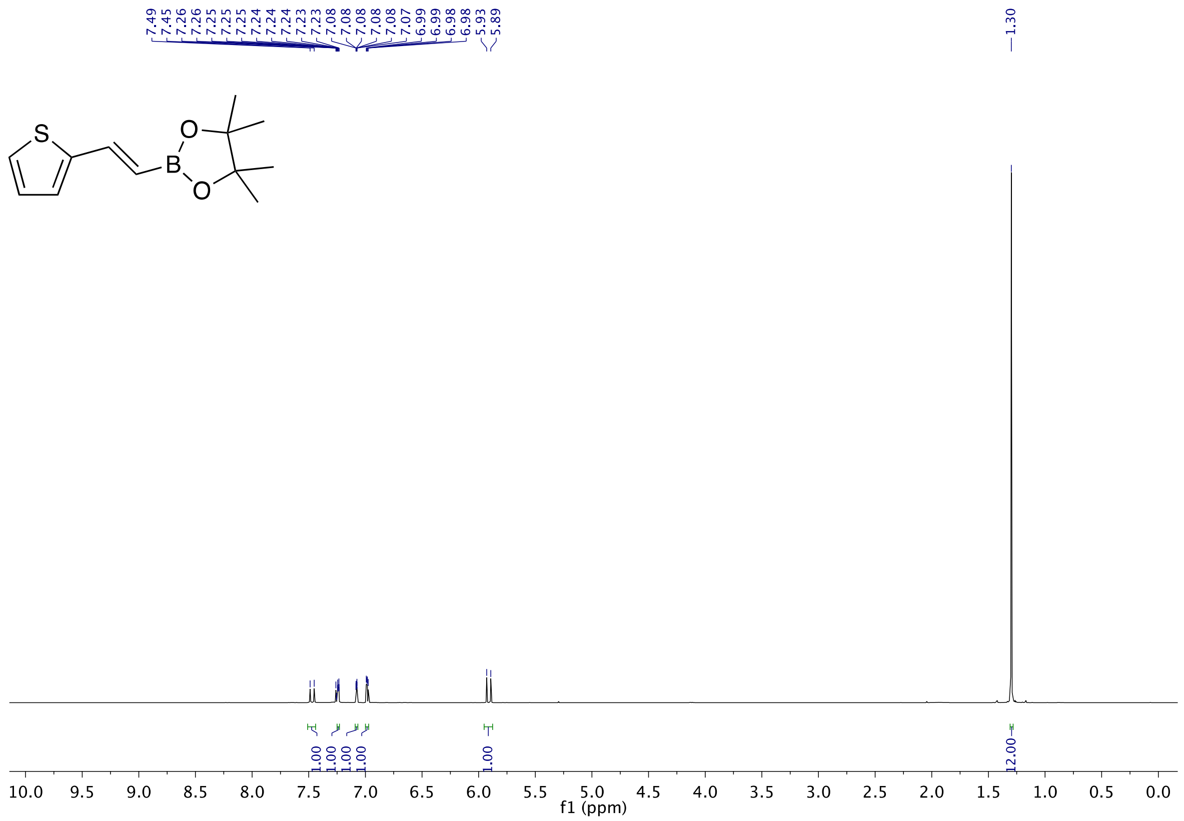

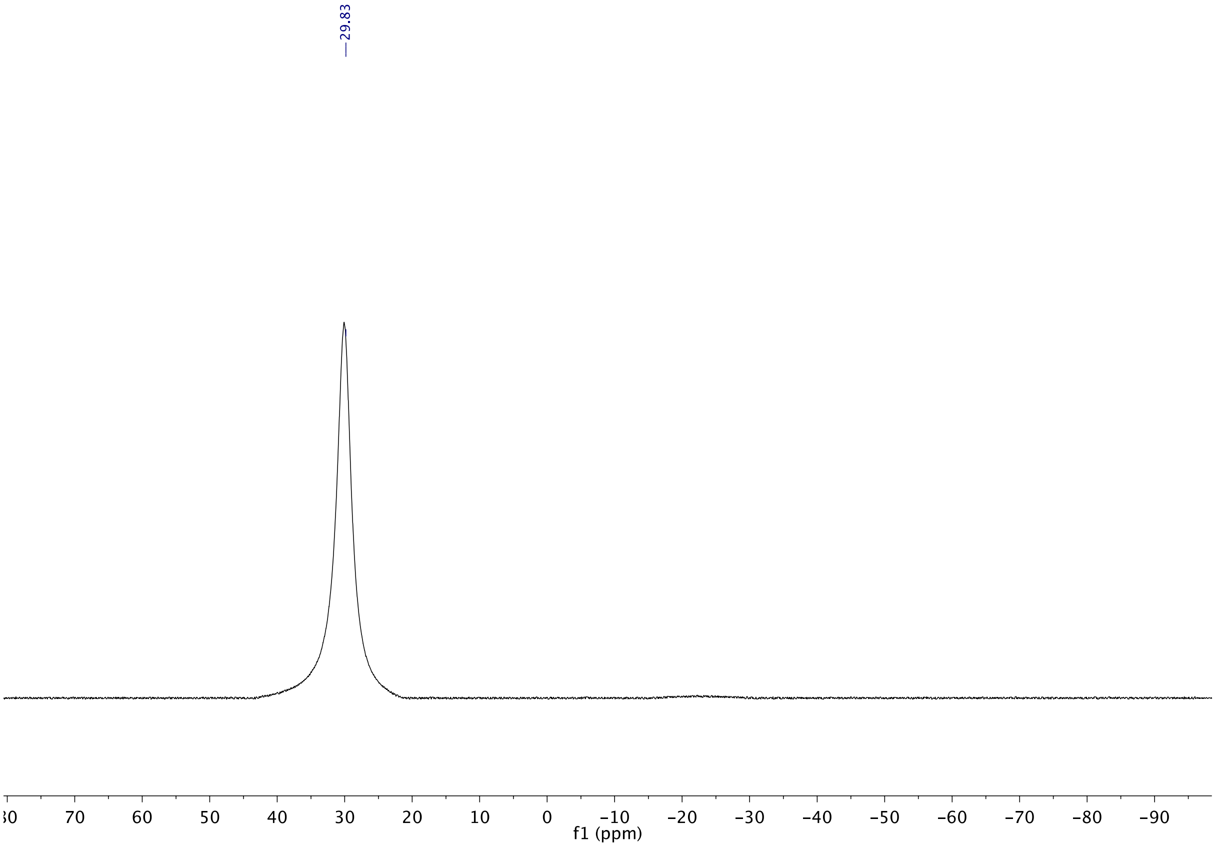
29.83
80
70
60
50
40
30
20
10
0
−10
−20
−30
−40
−50
−60
−70
−80
−90
f1 (ppm)

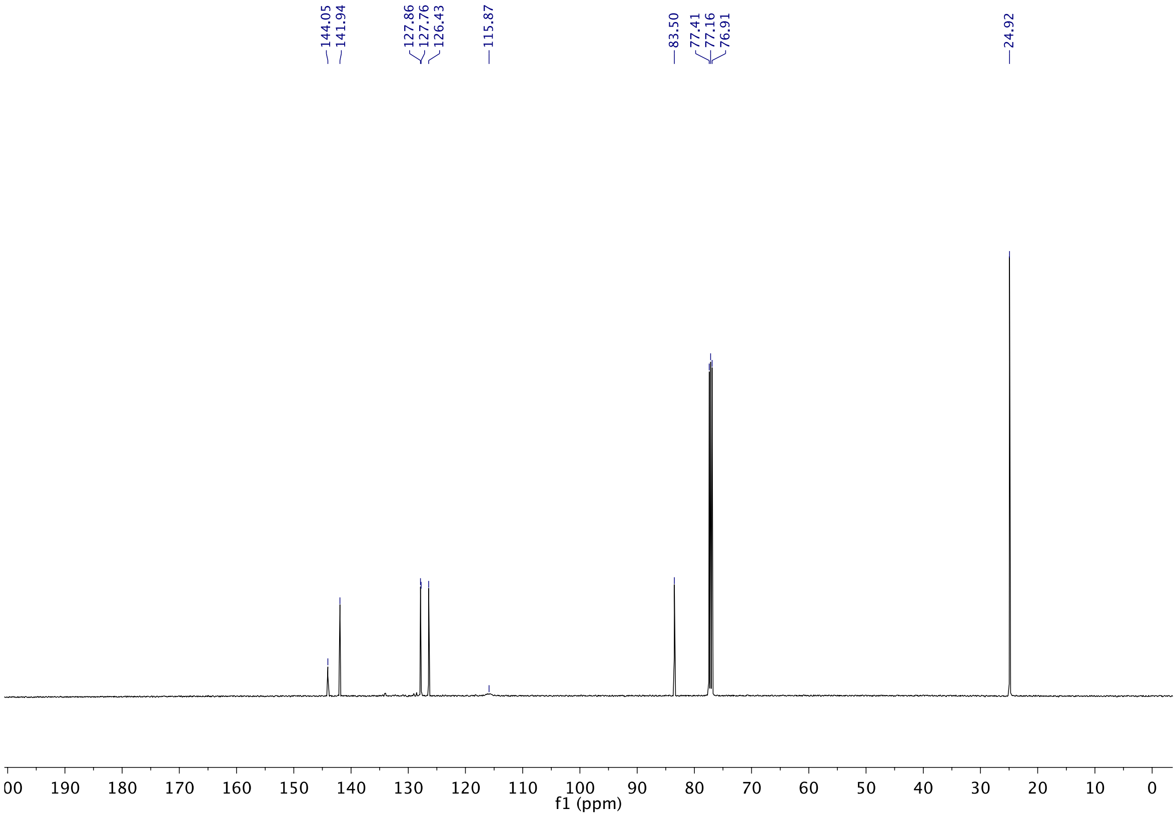
144.05
141.94
127.86
127.76
126.43
115.87
83.50
77.41
77.16
76.91
24.92
200
190
180
170
160
150
140
130
120
110
100
90
80
70
60
50
40
30
20
10
0
f1 (ppm)

(E)-Trifluoro(2-(thiophen-2-yl)vinyl)-λ[4]-borane, potassium salt, **S9-int4**

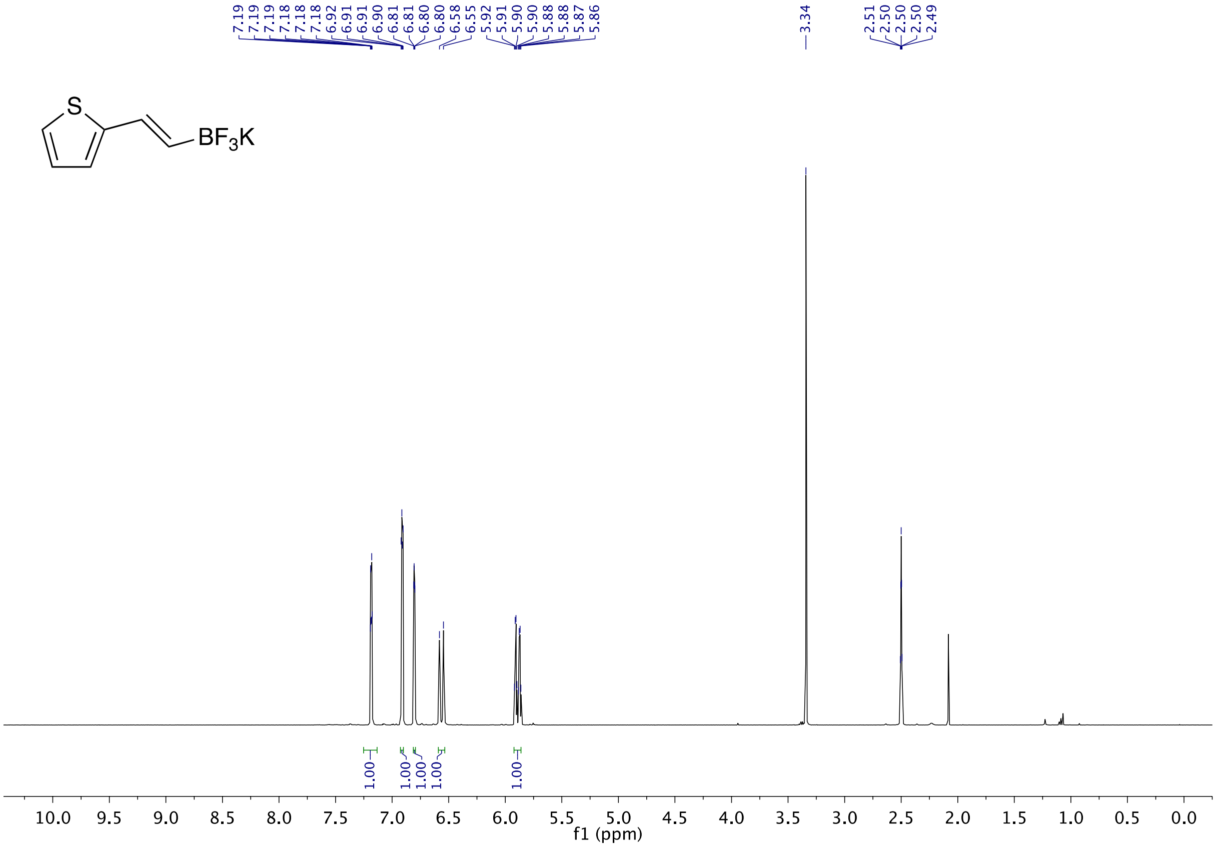

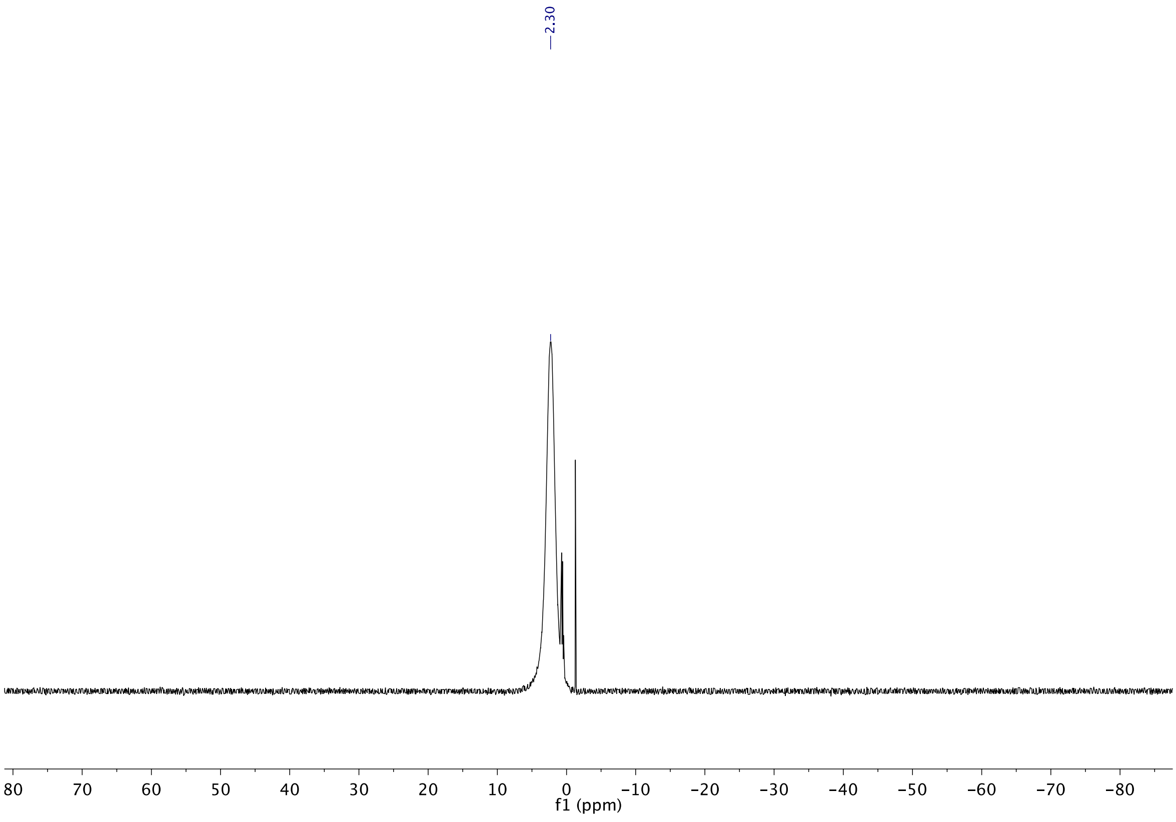

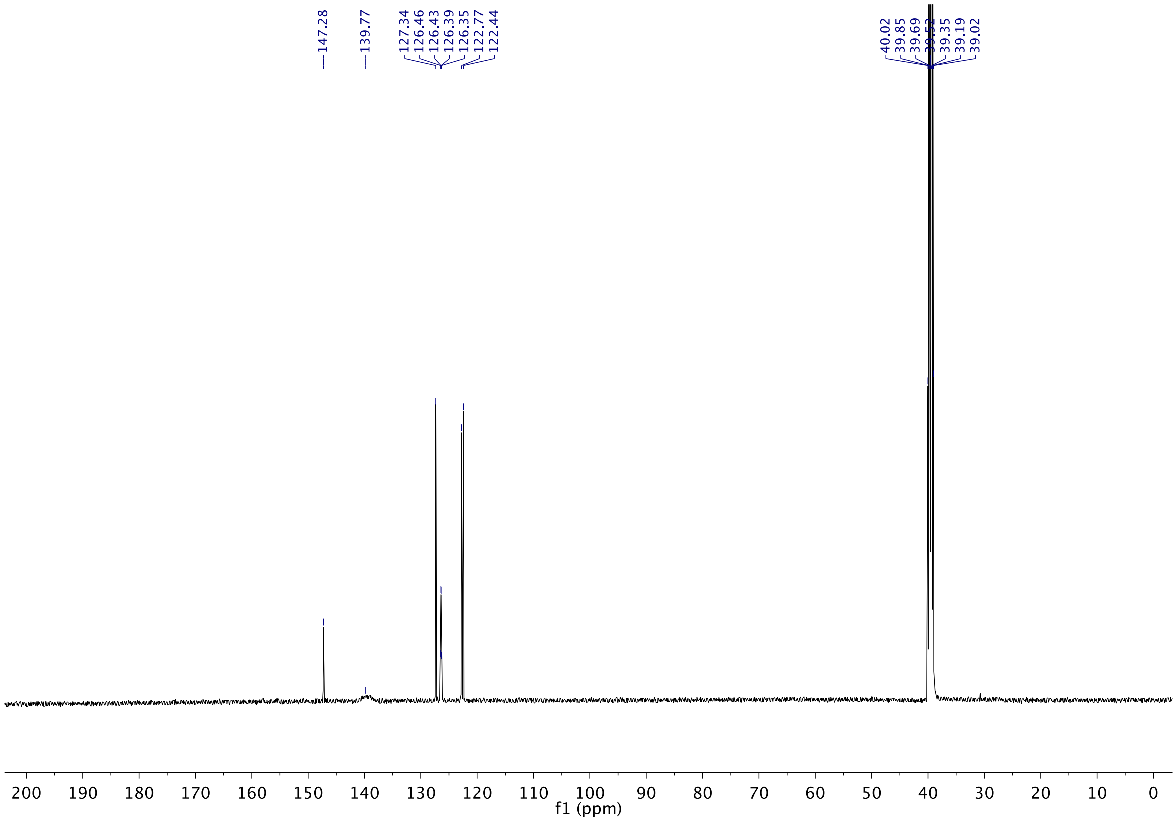
147.28
139.77
127.34
126.46
126.43
126.39
126.35
122.77
122.44
40.02
39.85
39.69
39.52
39.35
39.19
39.02
200 190 180 170 160 150 140 130 120 110 100 90 80 70 60 50 40 30 20 10 0
f1 (ppm)

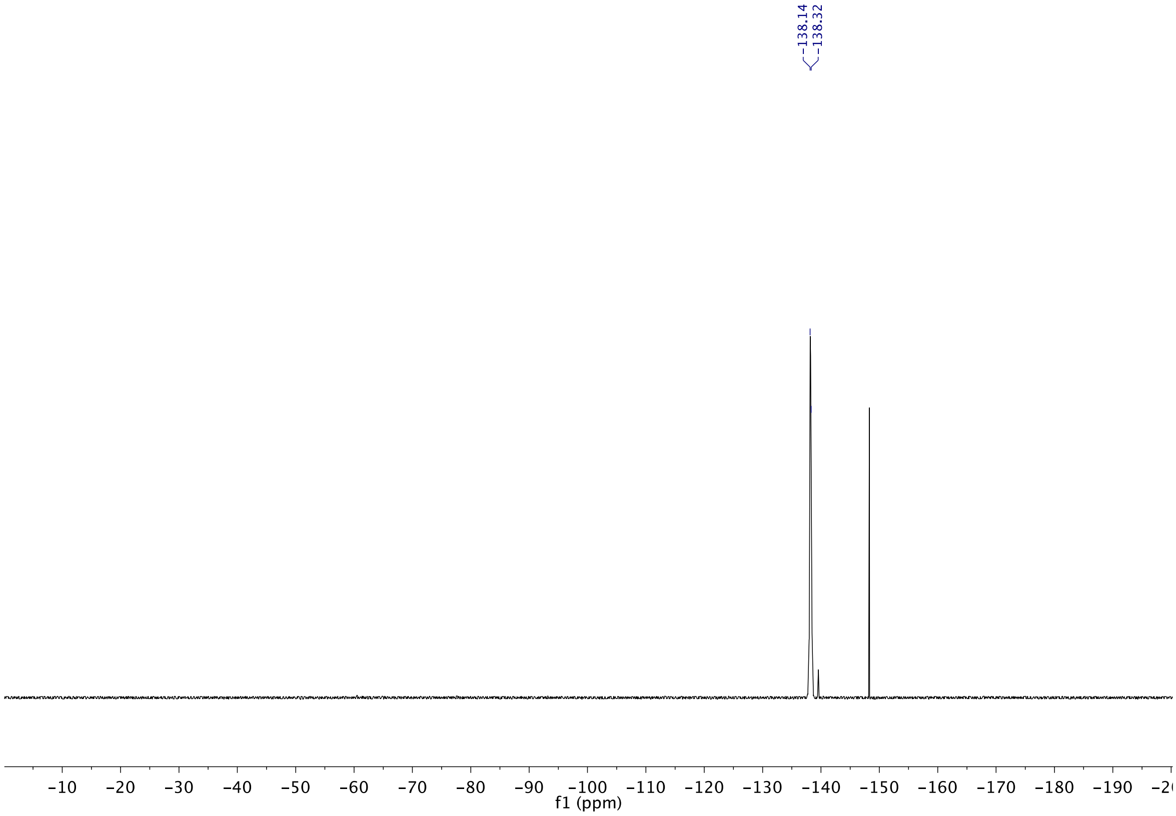
-138.14
-138.32
-10 -20 -30 -40 -50 -60 -70 -80 -90 -100 -110 -120 -130 -140 -150 -160 -170 -180 -190
f1 (ppm)

(*E*)-(2-(Thiophen-2-yl)vinyl)boronic acid, **S9**

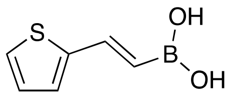

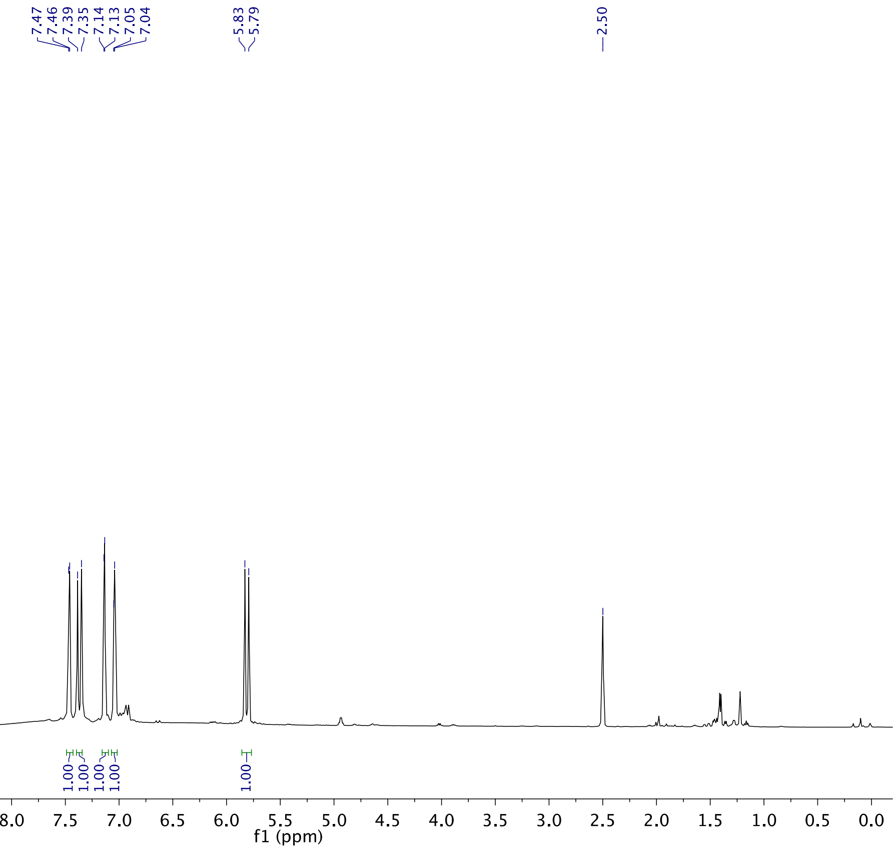

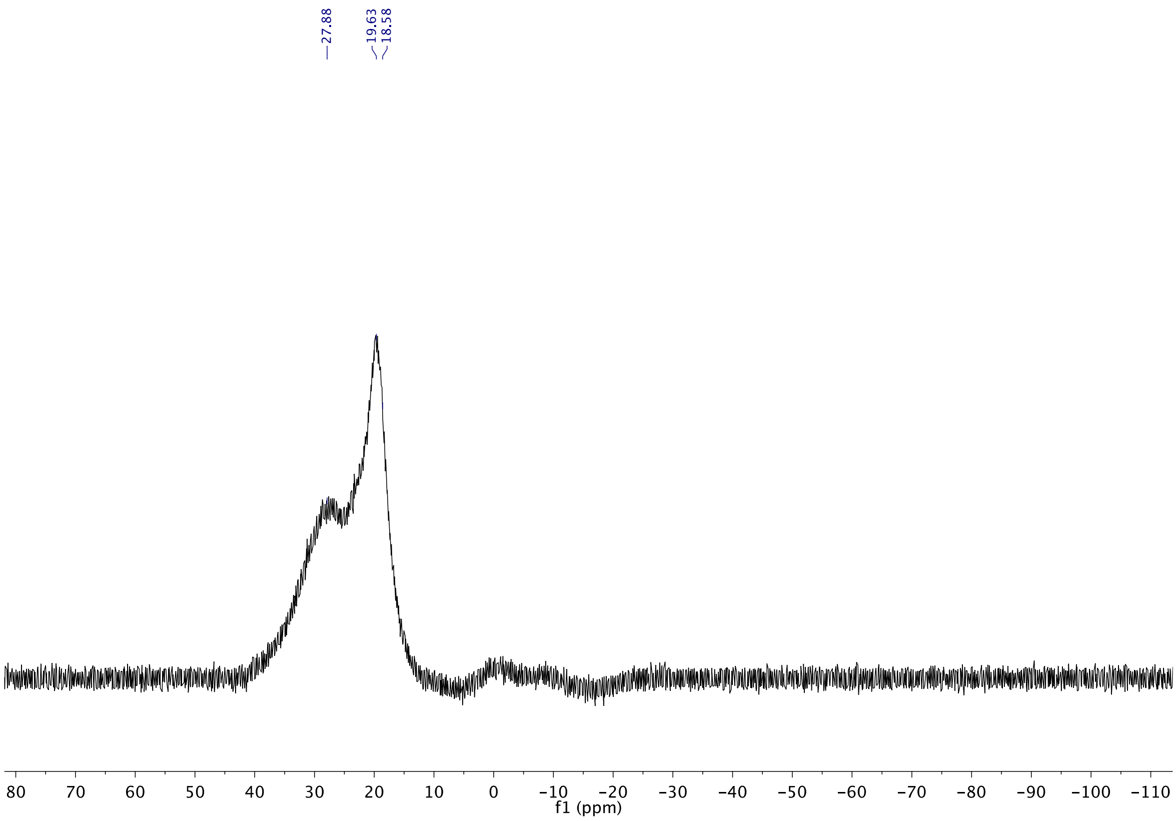

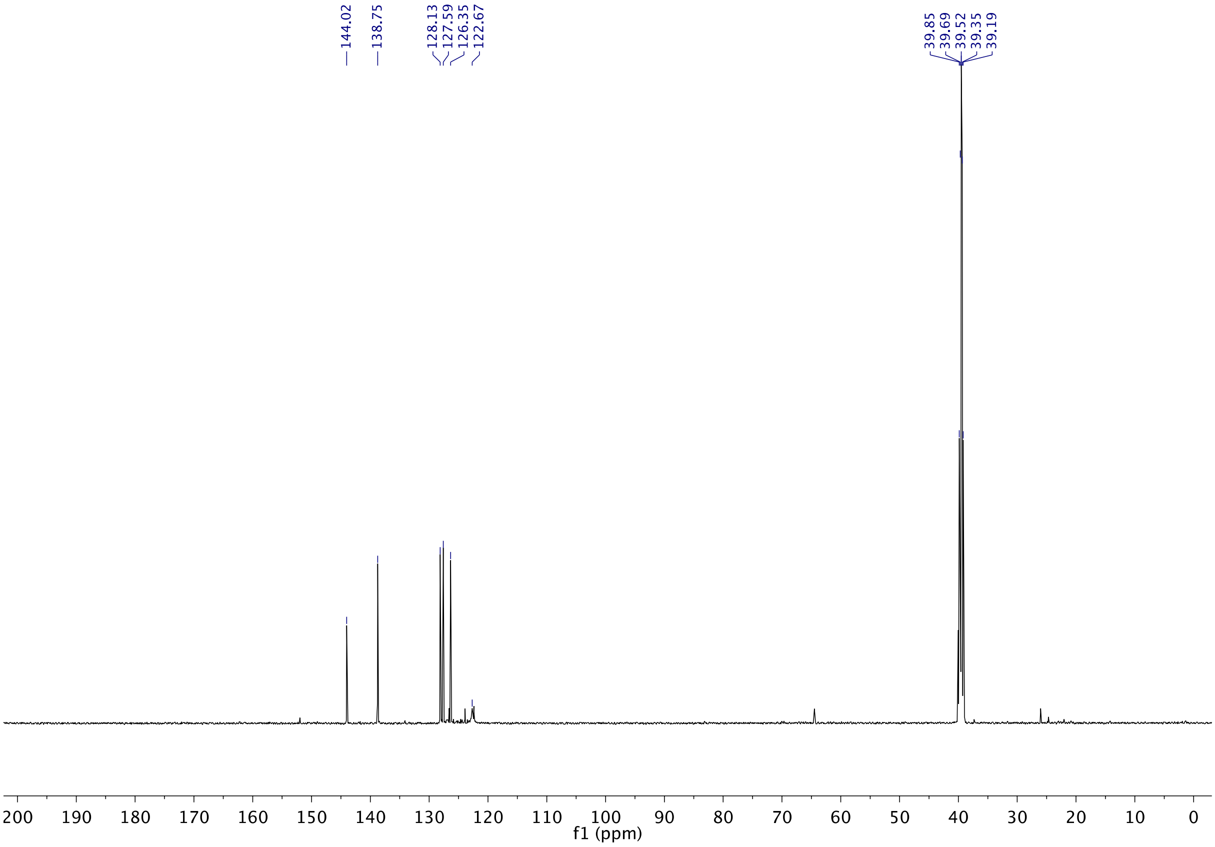

(Benzofuran-5-ylethynyl)trimethylsilane, **S10-int1**

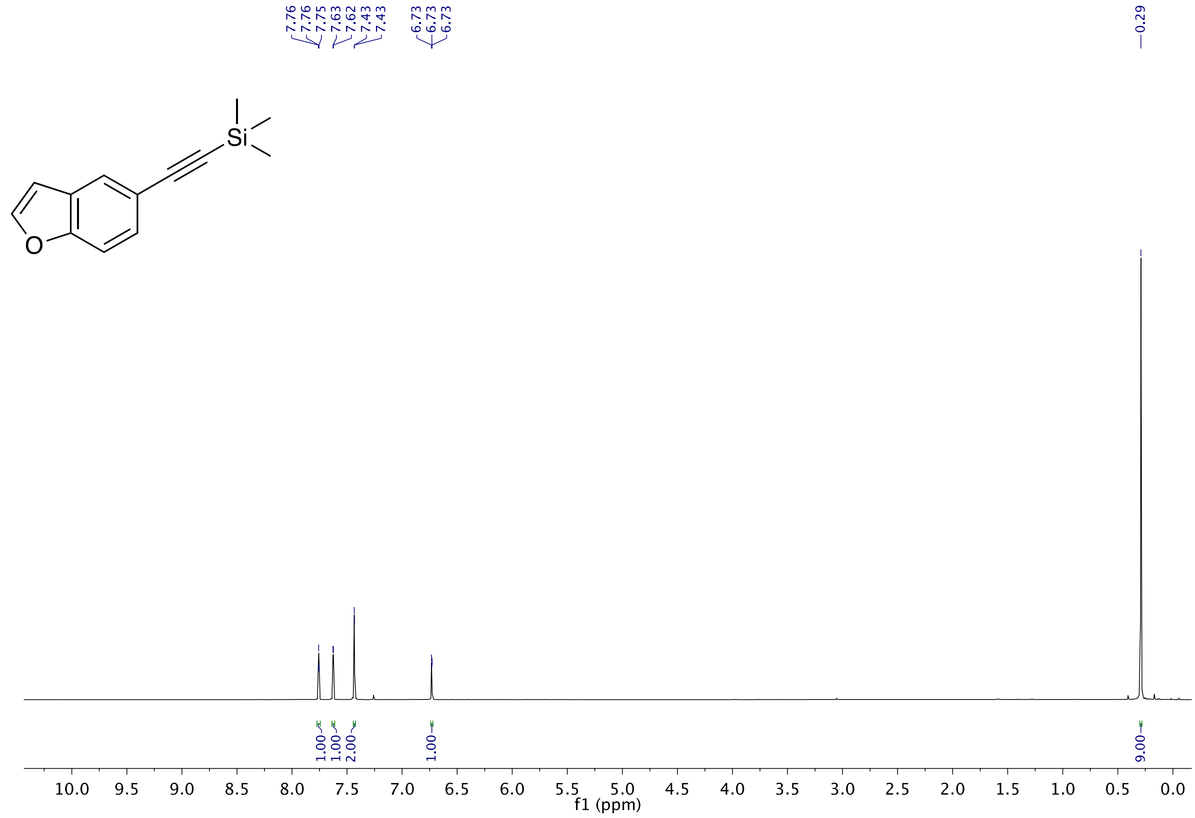

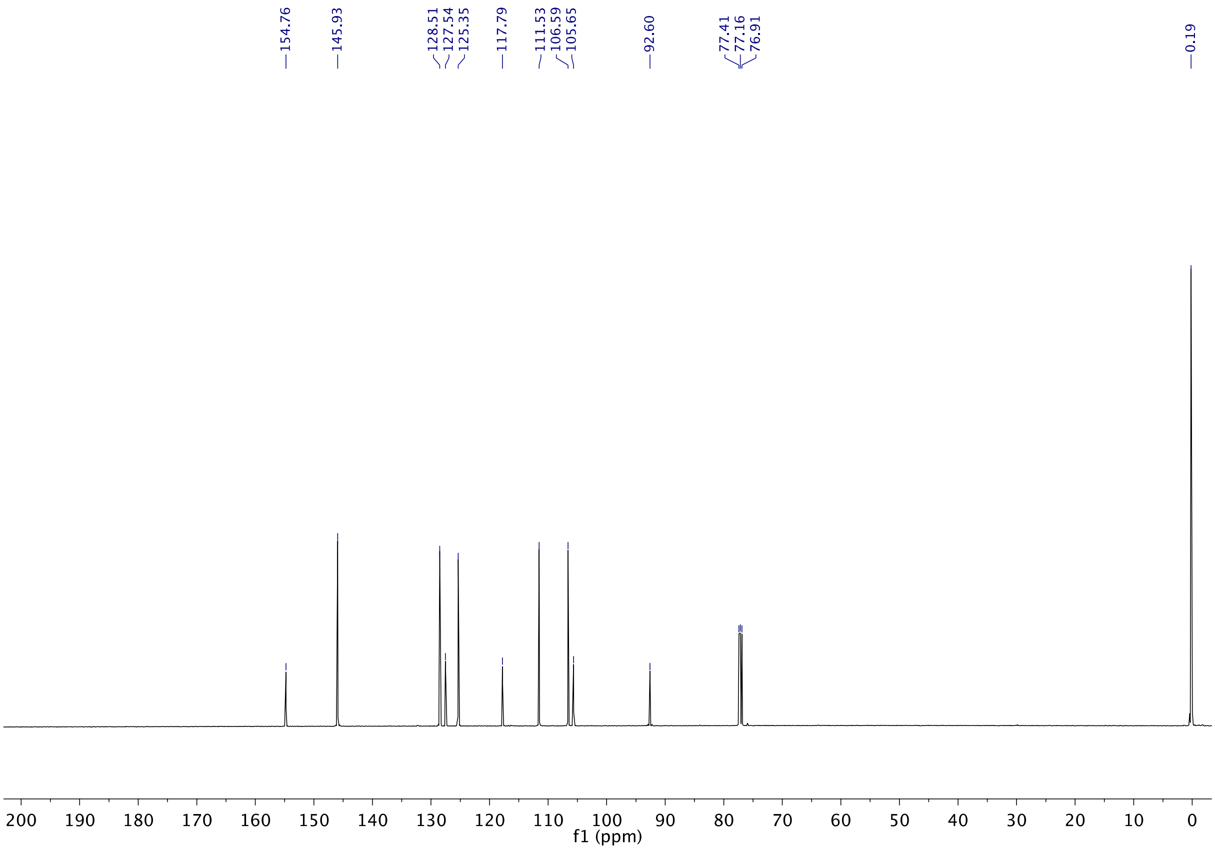

5-Ethynylbenzofuran, **S10-int2**

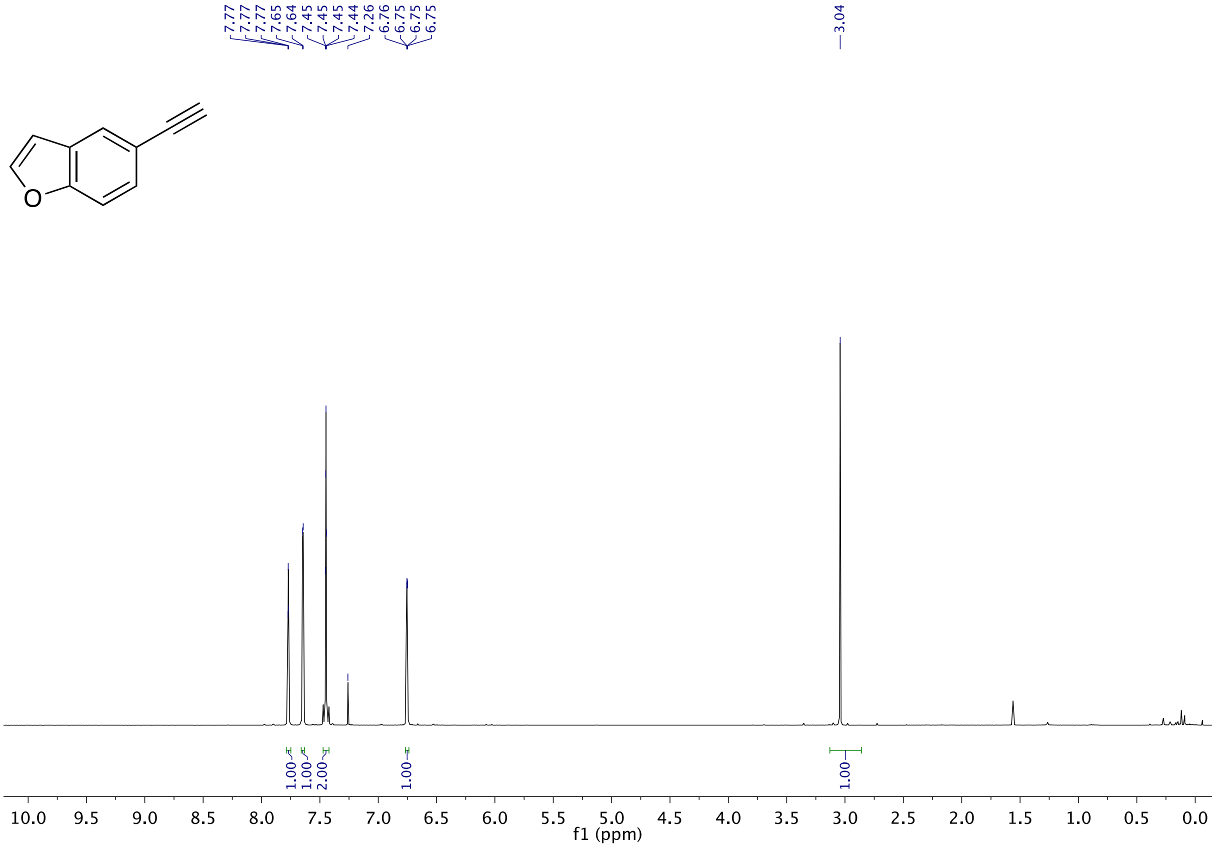

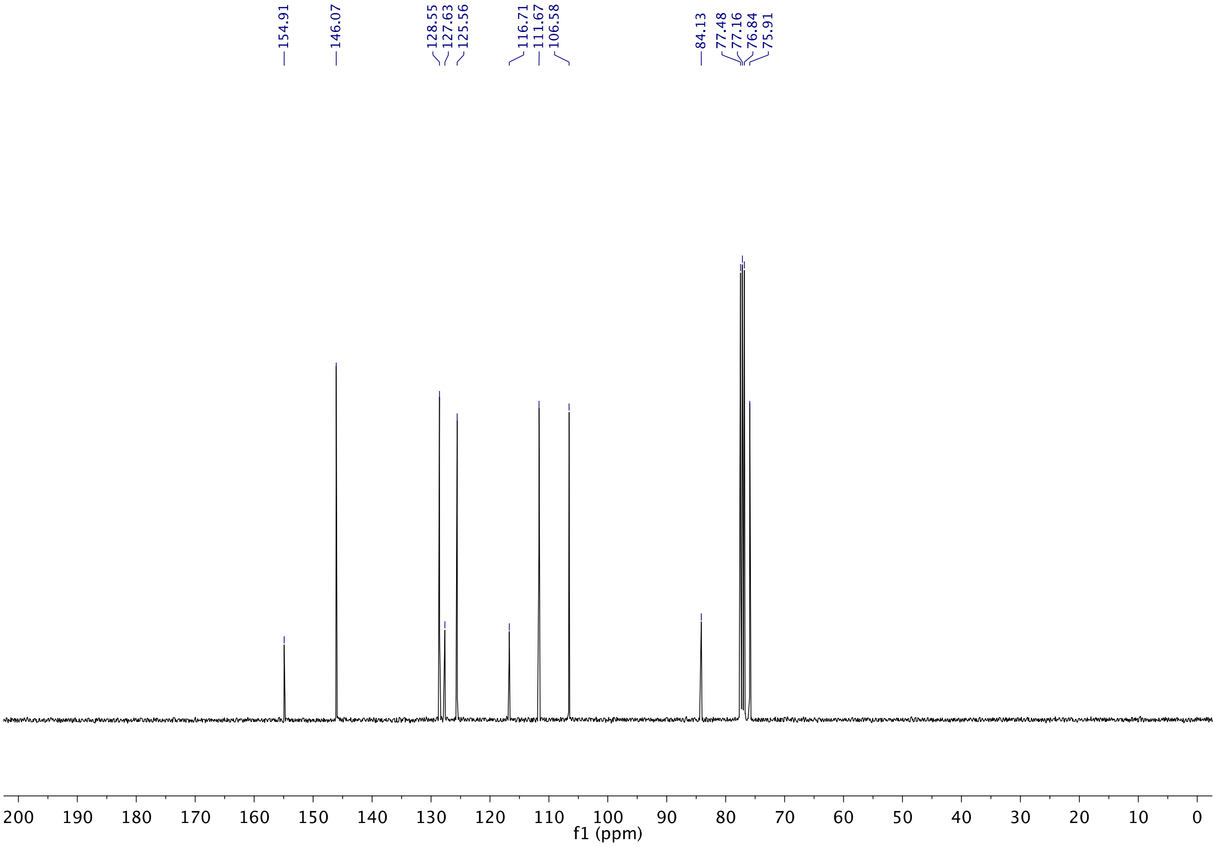

(E)-2-(2-(Benzofuran-5-yl)vinyl)-4,4,5,5-tetramethyl-1,3,2-dioxaborolane, **S10-int3**

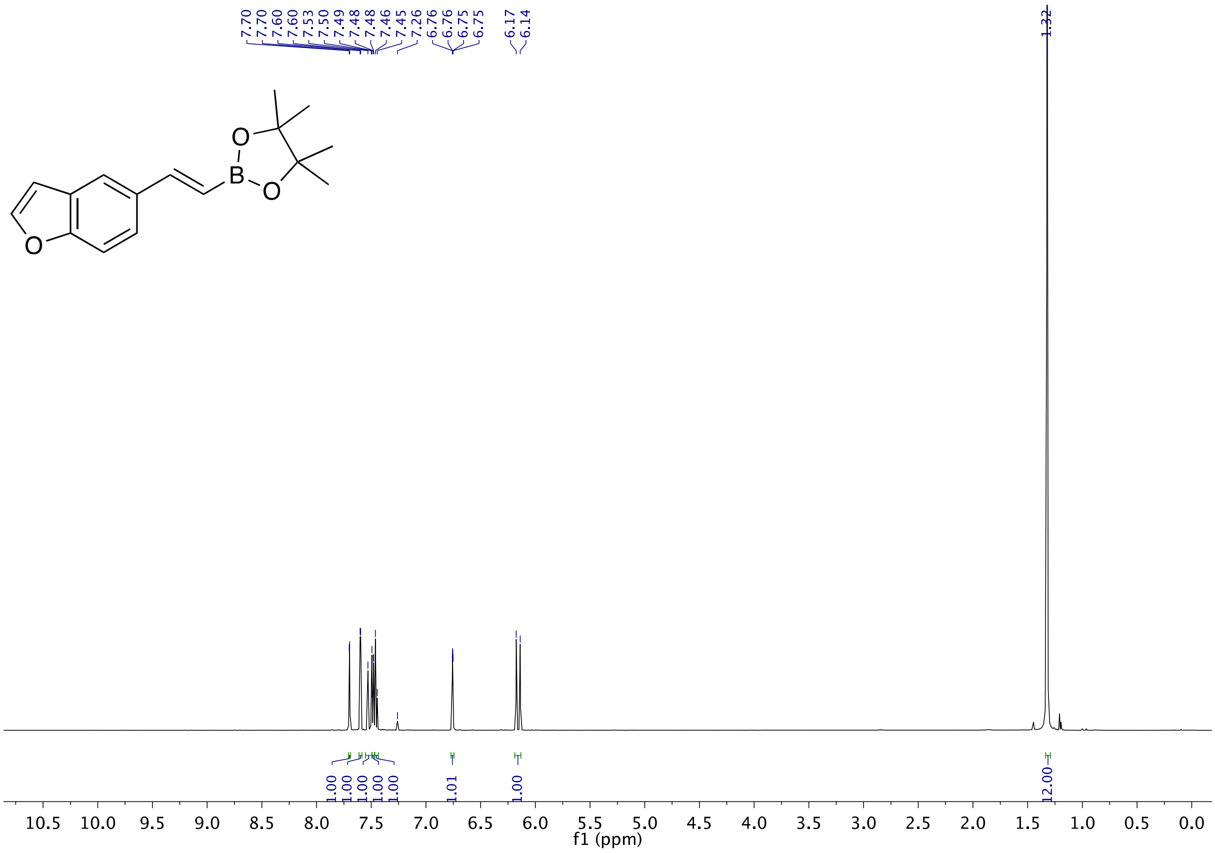

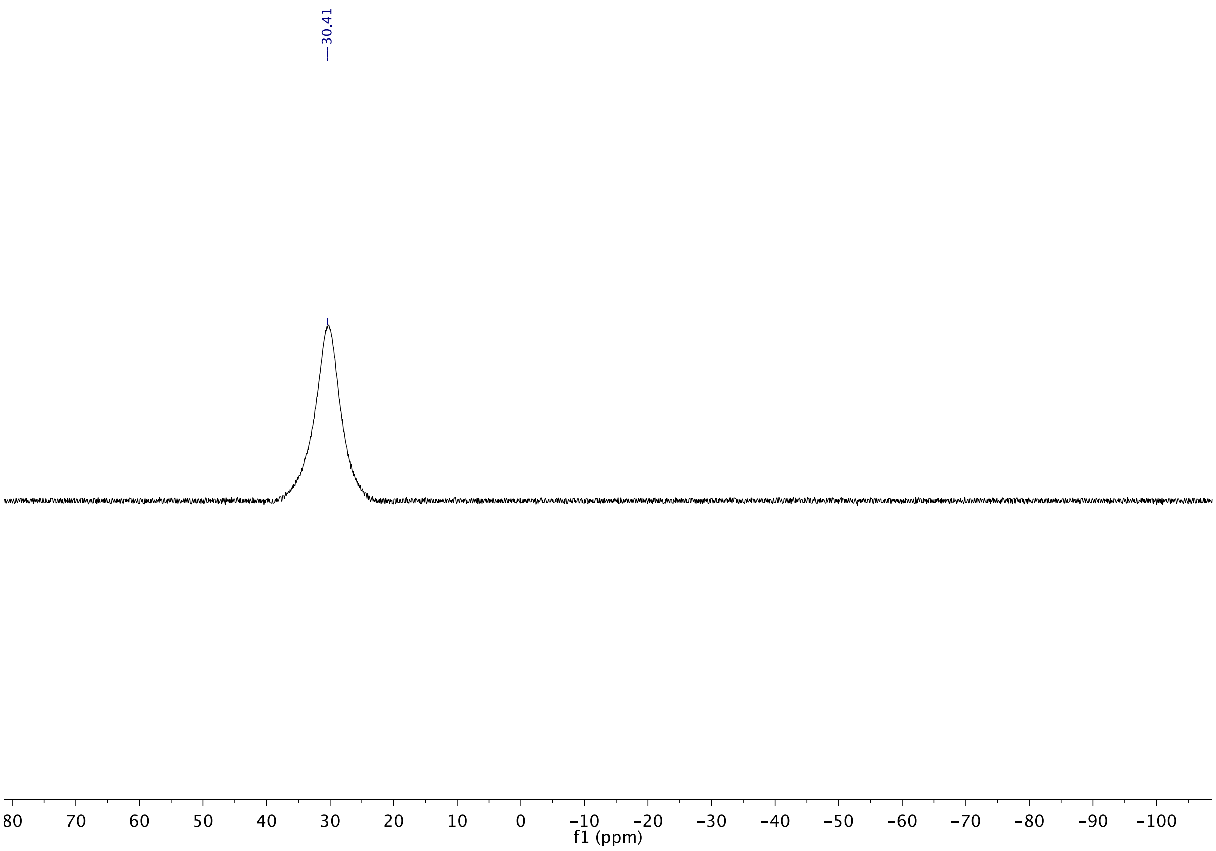
30.41
80 70 60 50 40 30 20 10 0 −10 −20 −30 −40 −50 −60 −70 −80 −90 −100
f1 (ppm)

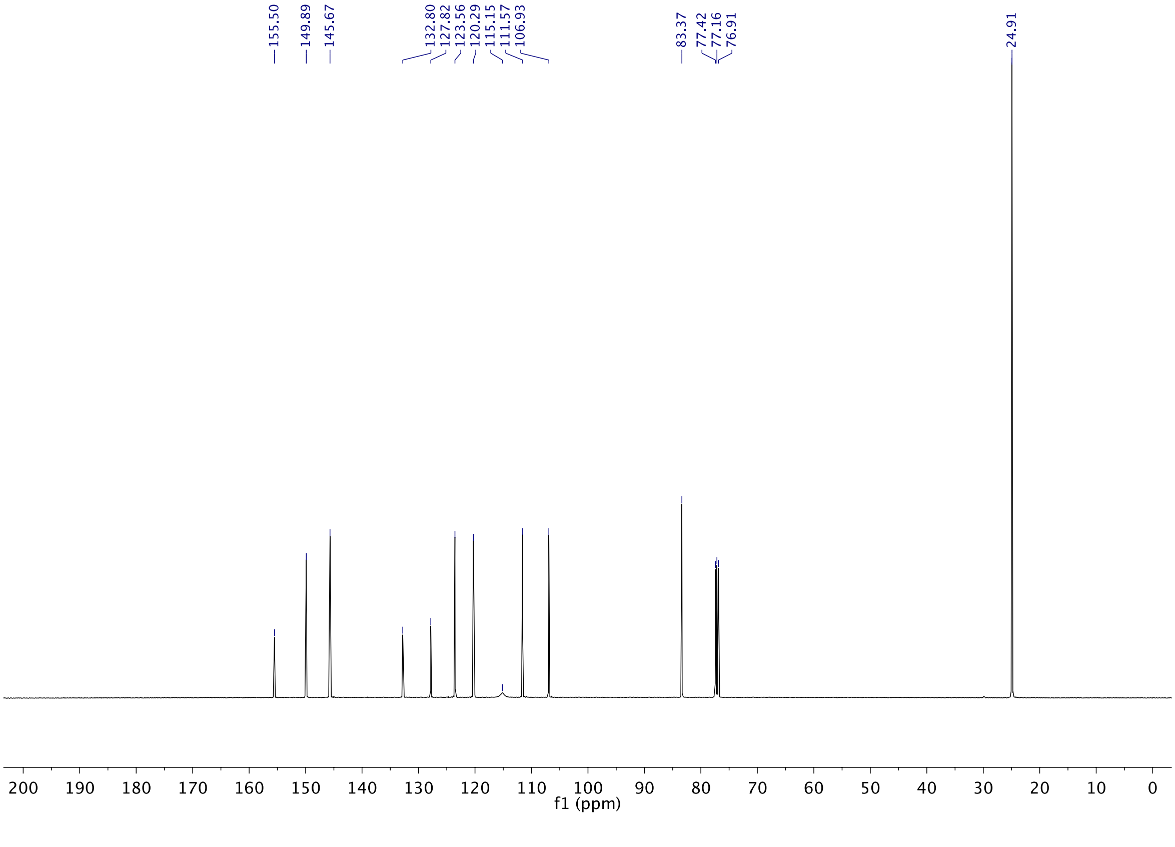
155.50
149.89
145.67
132.80
127.82
123.56
120.29
115.15
111.57
106.93
83.37
77.42
77.16
76.91
24.91
200 190 180 170 160 150 140 130 120 110 100 90 80 70 60 50 40 30 20 10 0
f1 (ppm)

(*E*)-(2-(Benzofuran-5-yl)vinyl)trifluoro-λ[4]-borane, potassium salt, **S10-int4**

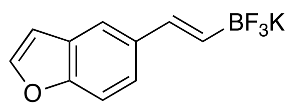

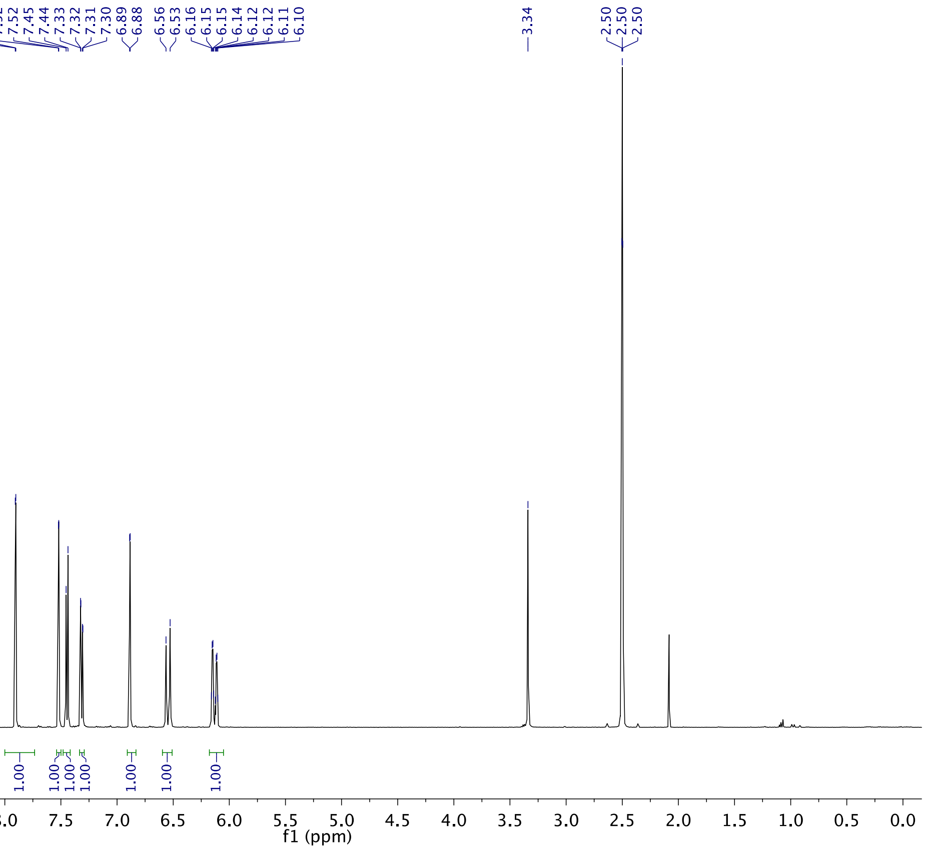

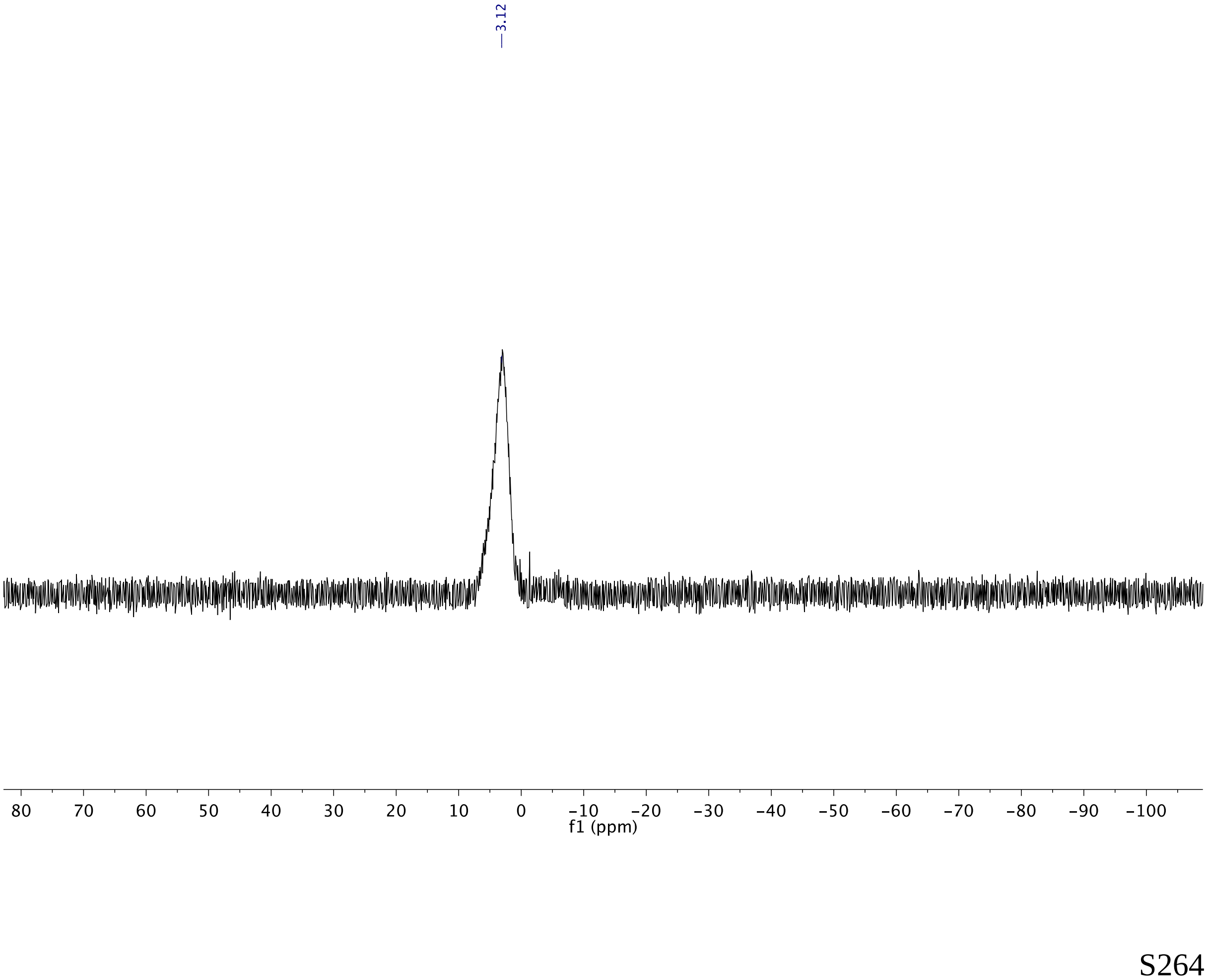

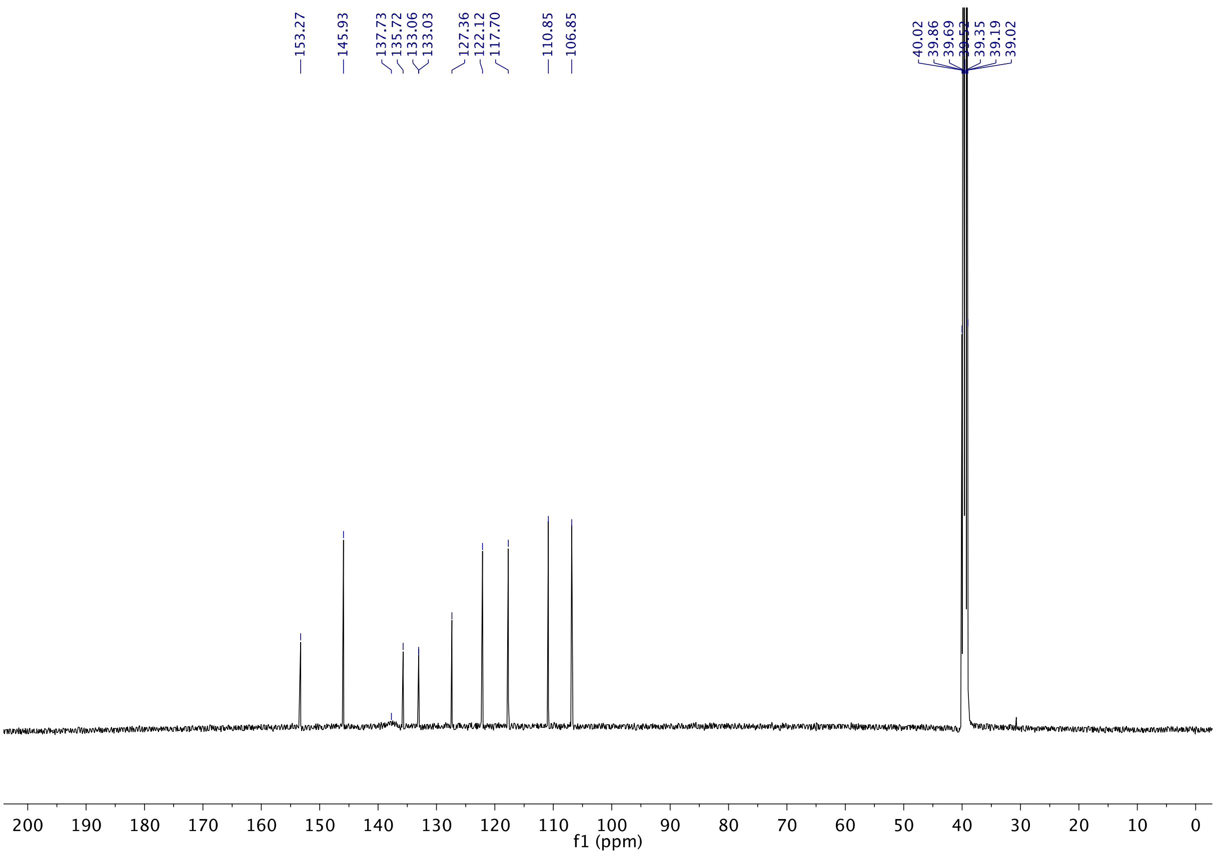
153.27
145.93
137.73
135.72
133.06
133.03
127.36
122.12
117.70
110.85
106.85
40.02
39.86
39.69
39.52
39.35
39.19
39.02
200 190 180 170 160 150 140 130 120 110 100 90 80 70 60 50 40 30 20 10 0
f1 (ppm)

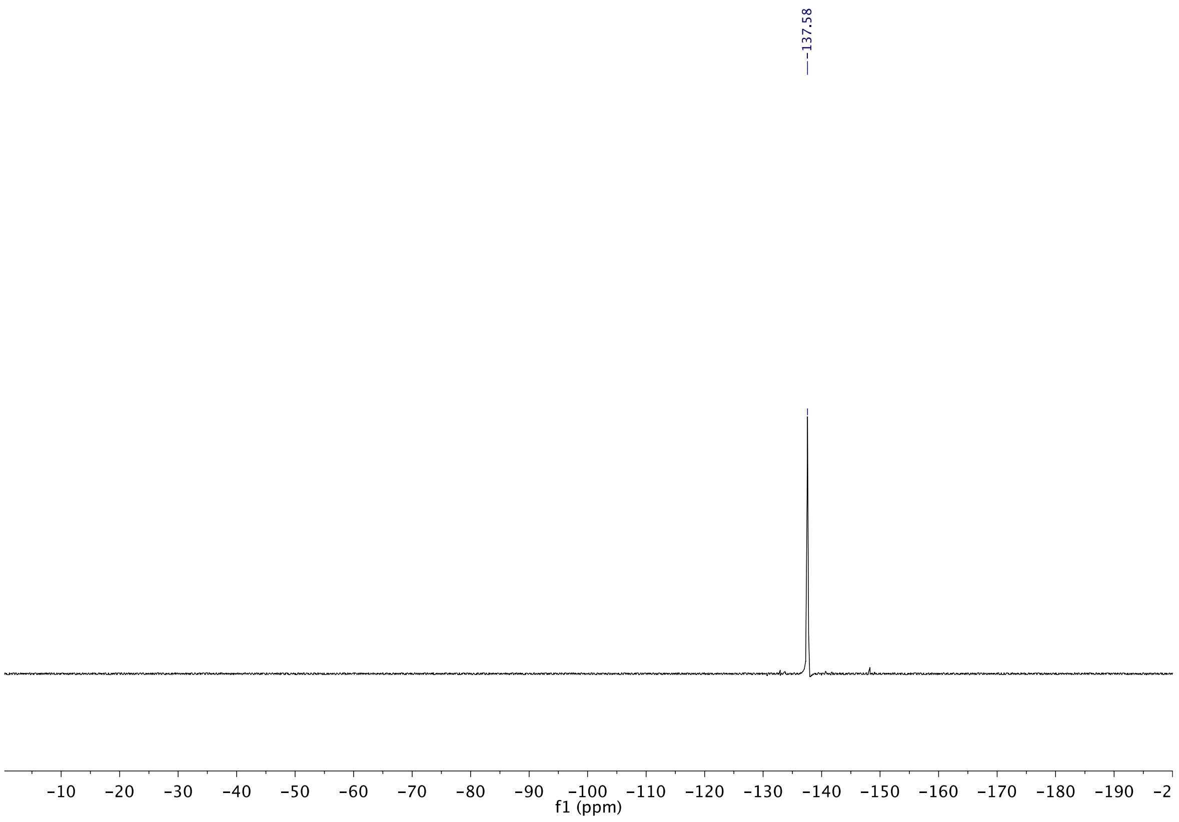
-137.58
-10 -20 -30 -40 -50 -60 -70 -80 -90 -100 -110 -120 -130 -140 -150 -160 -170 -180 -190 -2
f1 (ppm)

(*E*)-(2-(Benzofuran-5-yl)vinyl)boronic acid, **S10**

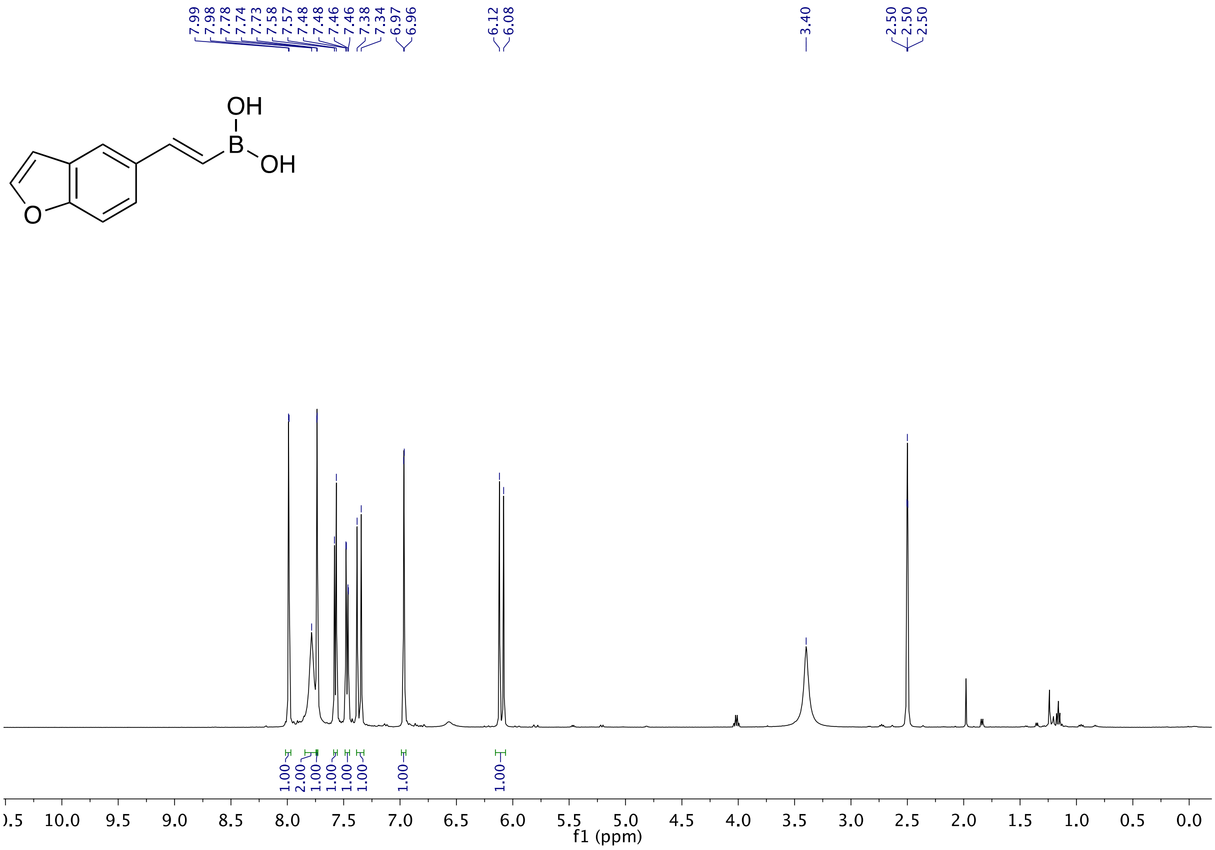

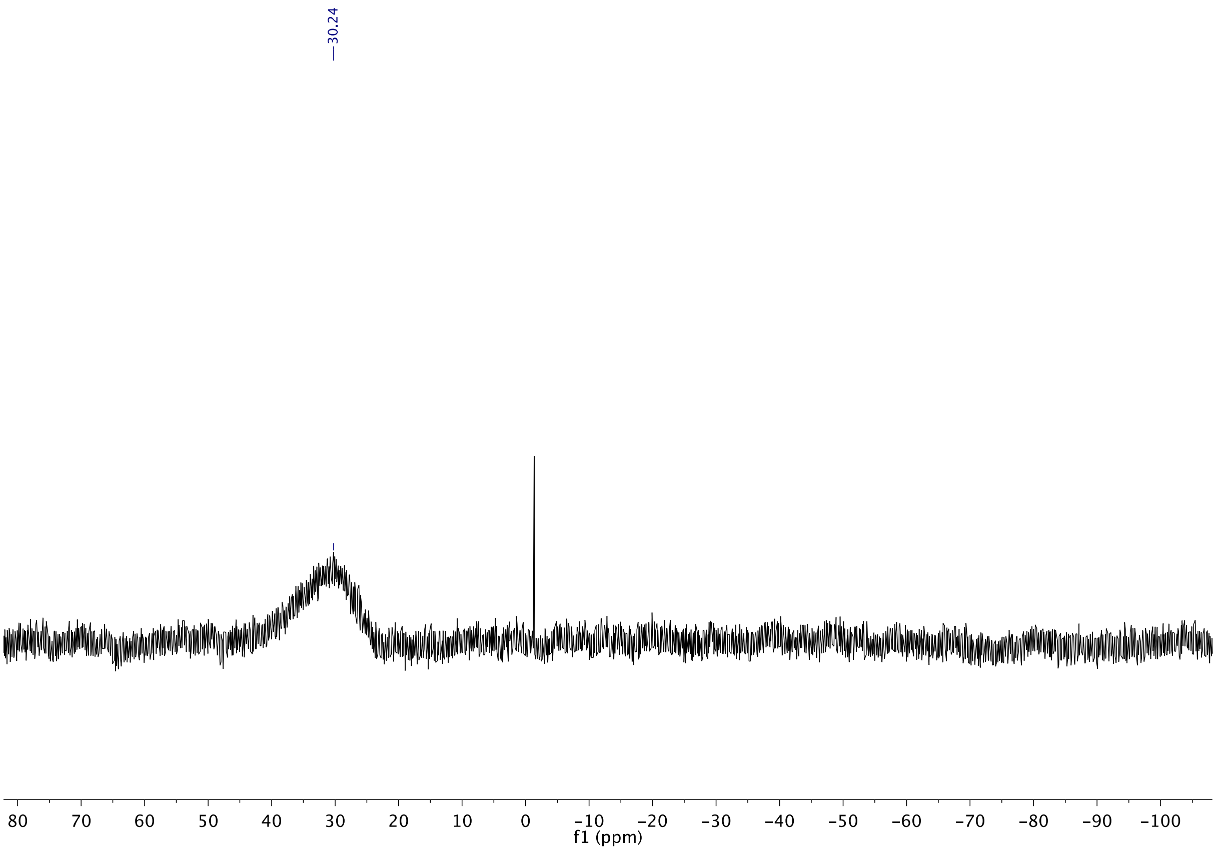

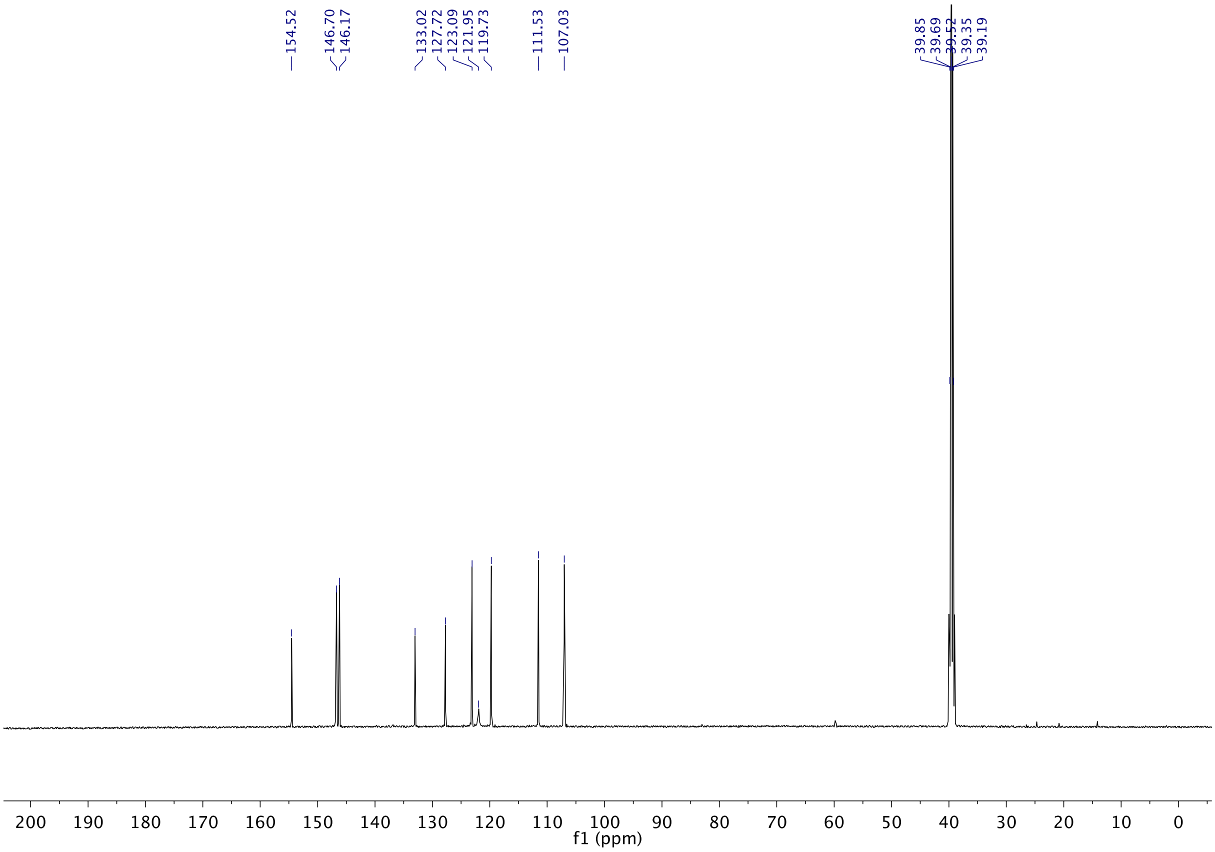

((2-Bromo-4-(trifluoromethoxy)phenyl)ethynyl)trimethylsilane, **S11-int1**

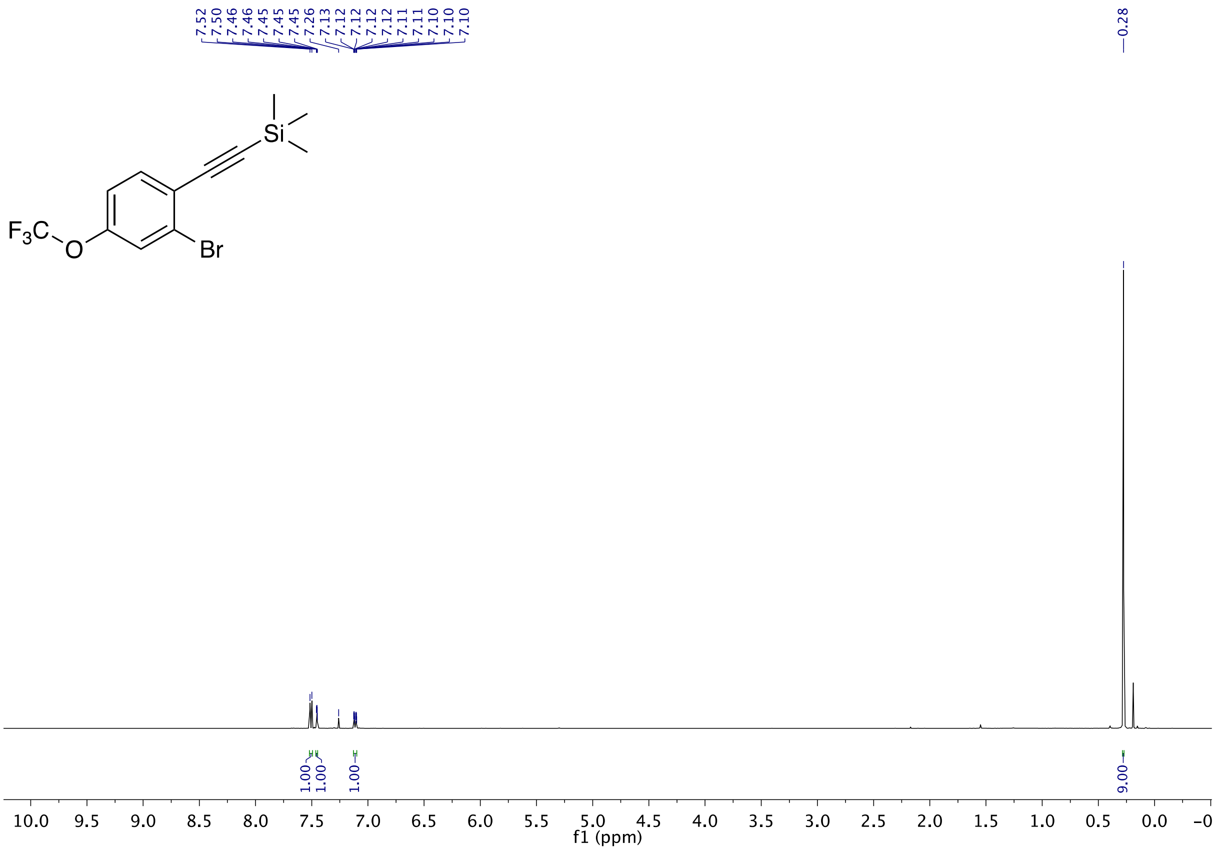

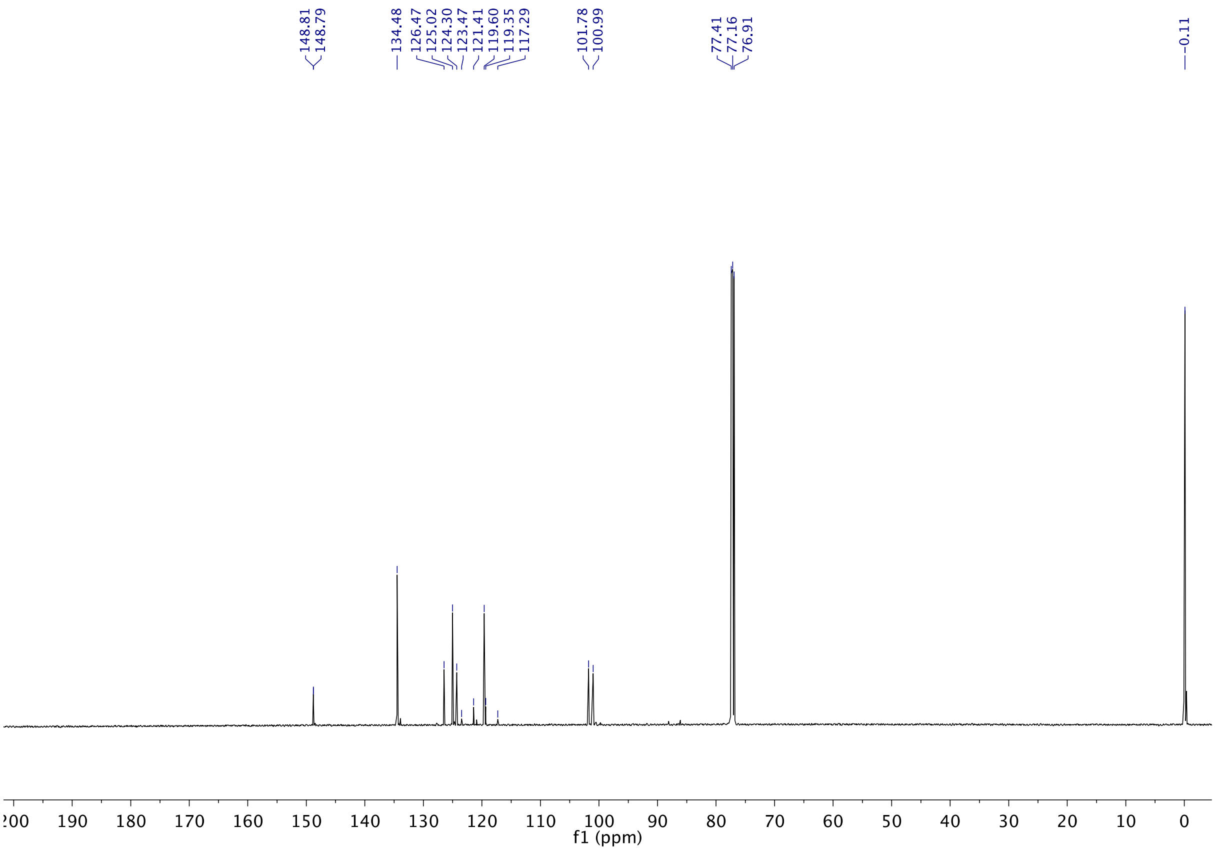
148.81
148.79
134.48
126.47
125.02
124.30
123.47
121.41
119.60
119.35
117.29
101.78
100.99
77.41
77.16
76.91
0.11
200 190 180 170 160 150 140 130 120 110 100 90 80 70 60 50 40 30 20 10 0
f1 (ppm)

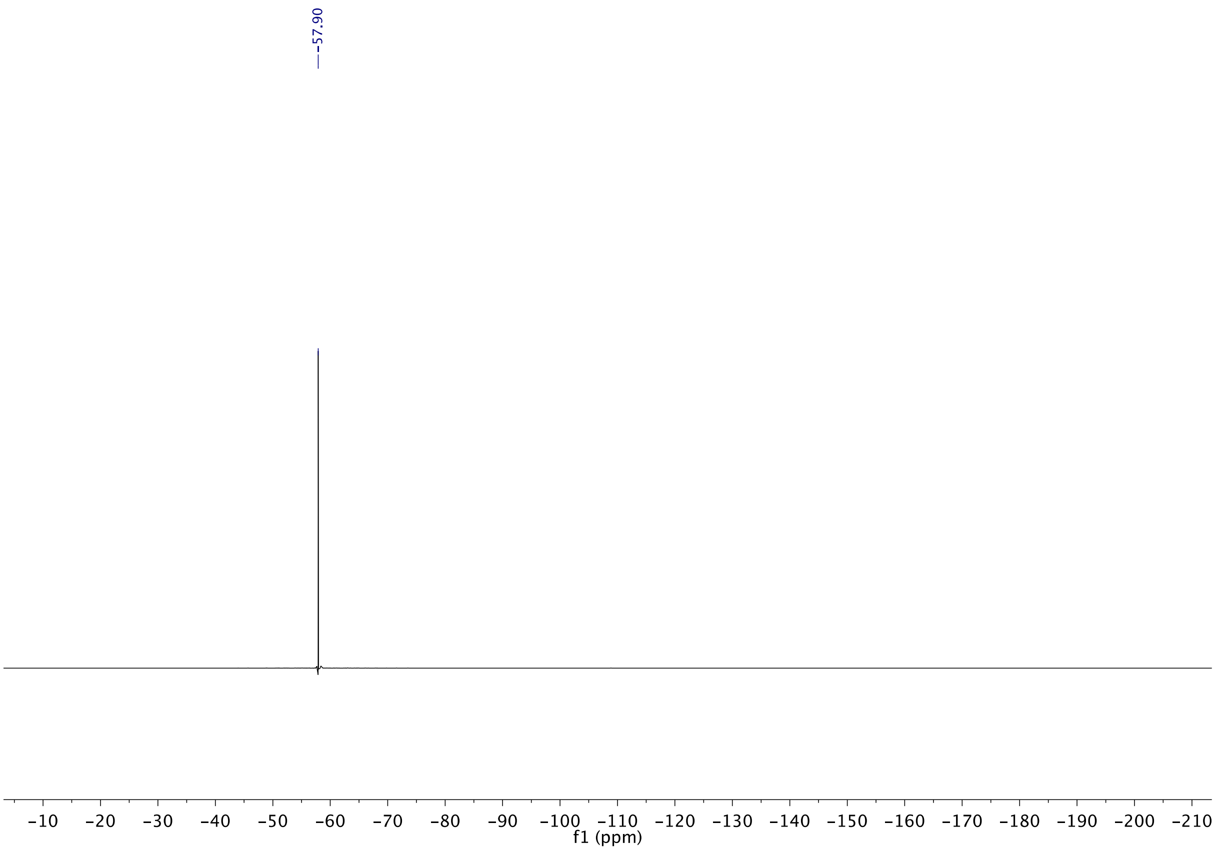
-57.90
-10 -20 -30 -40 -50 -60 -70 -80 -90 -100 -110 -120 -130 -140 -150 -160 -170 -180 -190 -200 -210
f1 (ppm)

2-Bromo-1-ethynyl-4-(trifluoromethoxy)benzene, **S11-int2**

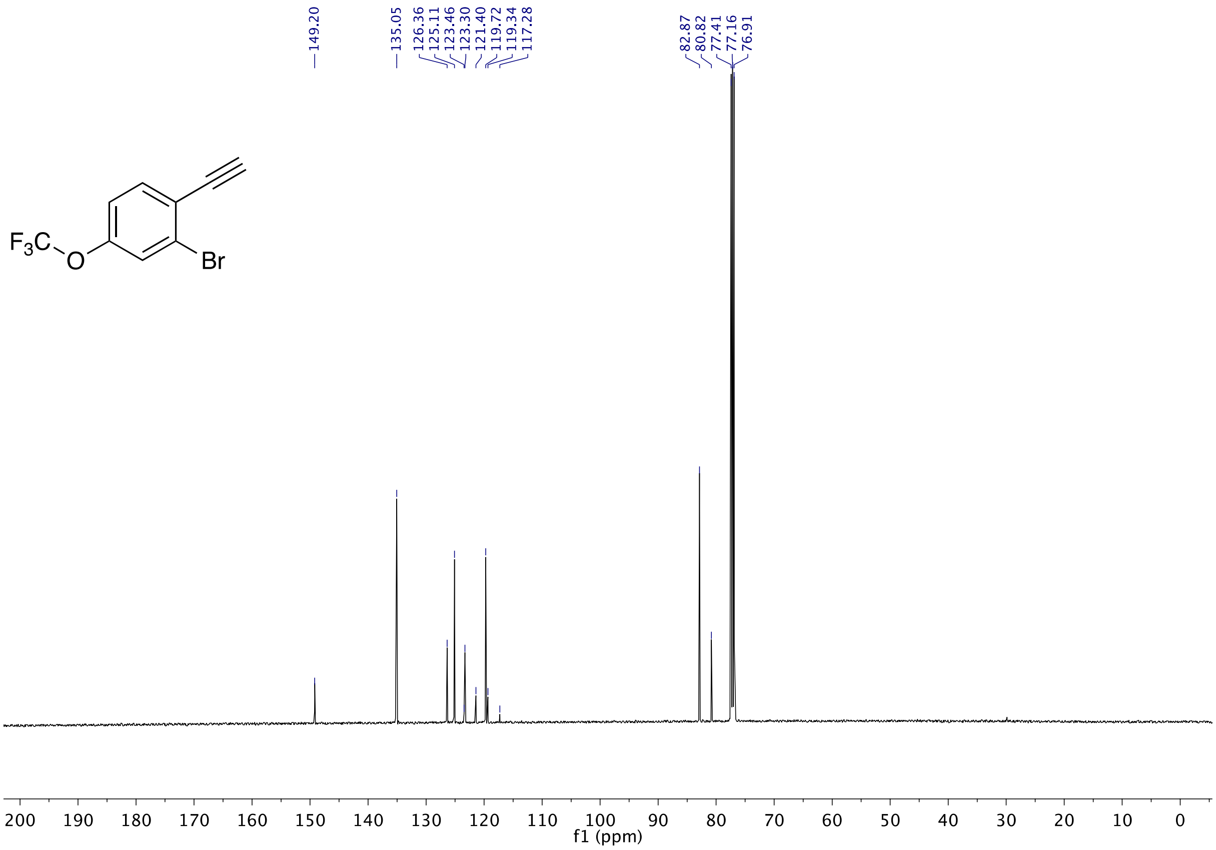

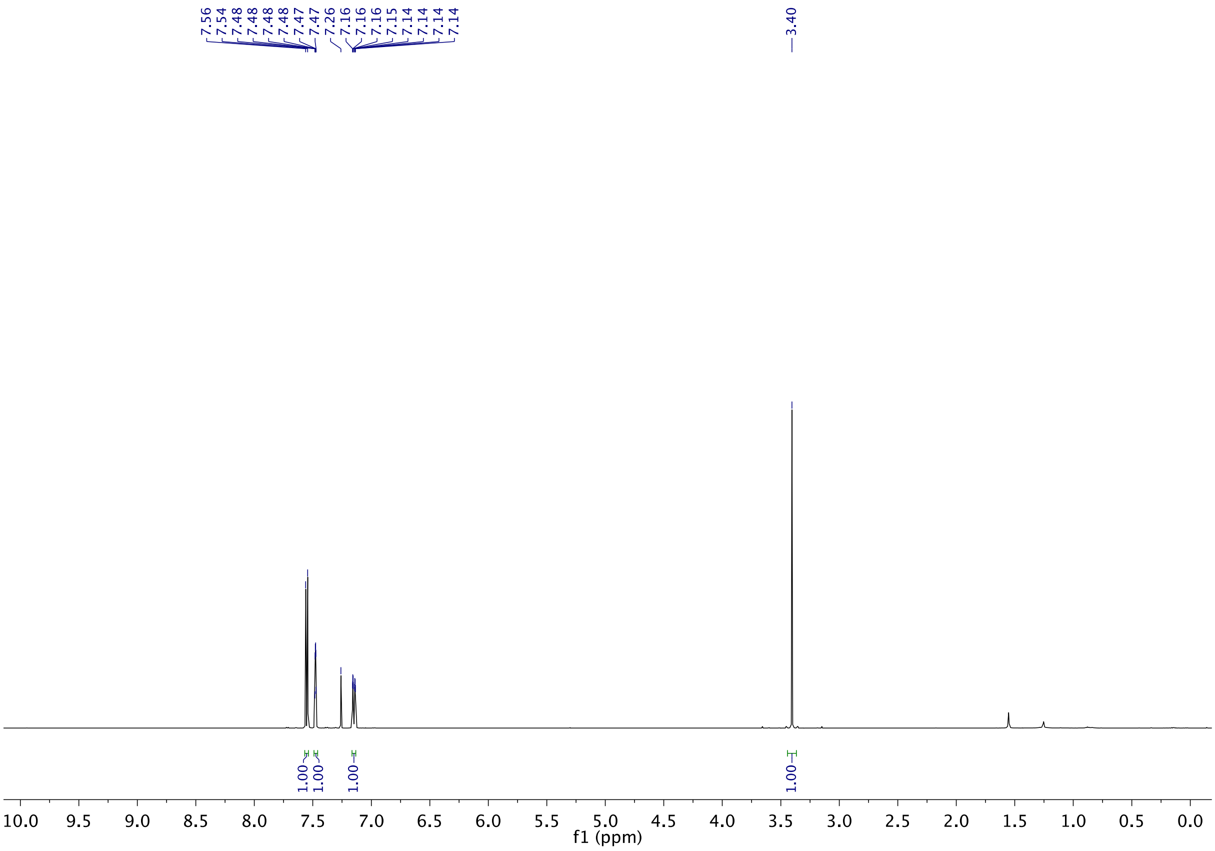

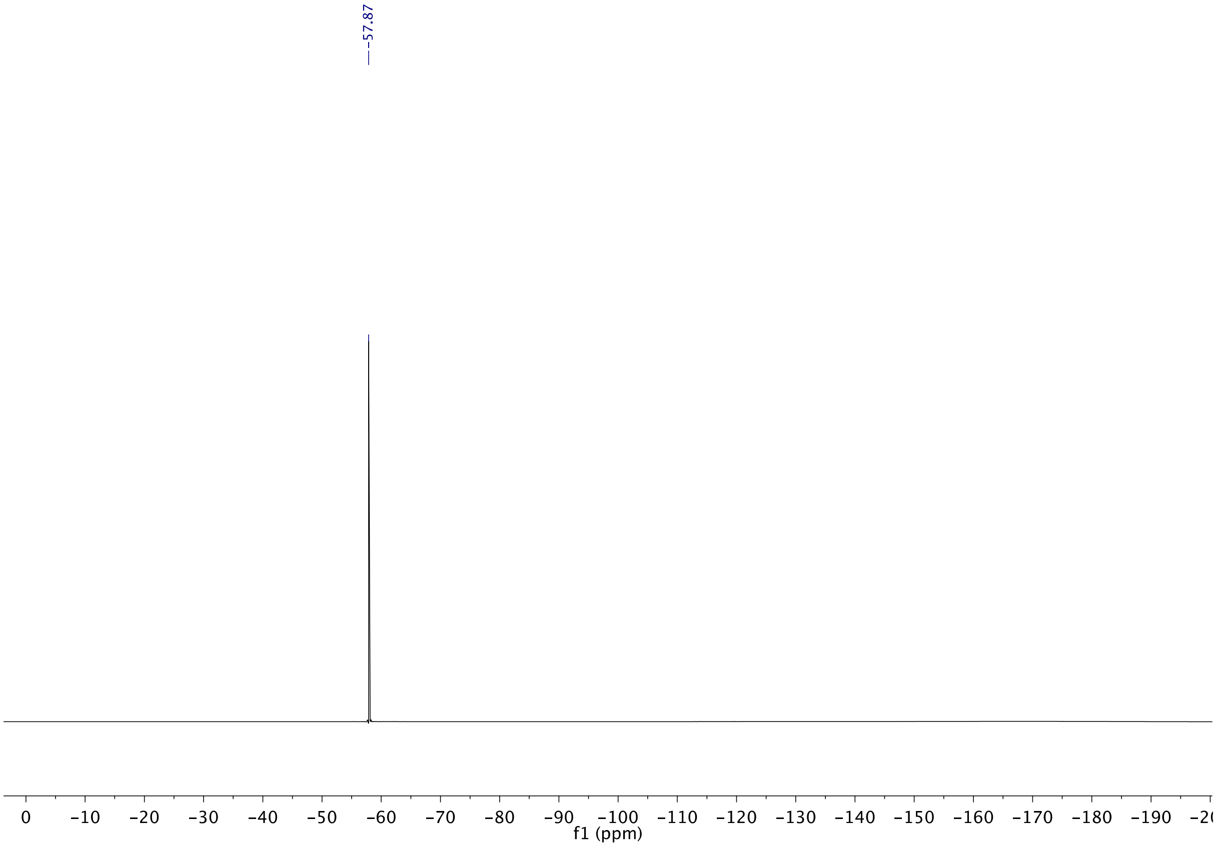

(*E*)-2-(2-Bromo-4-(trifluoromethoxy)styryl)-4,4,5,5-tetramethyl-1,3,2-dioxaborolane, **S11-int3**

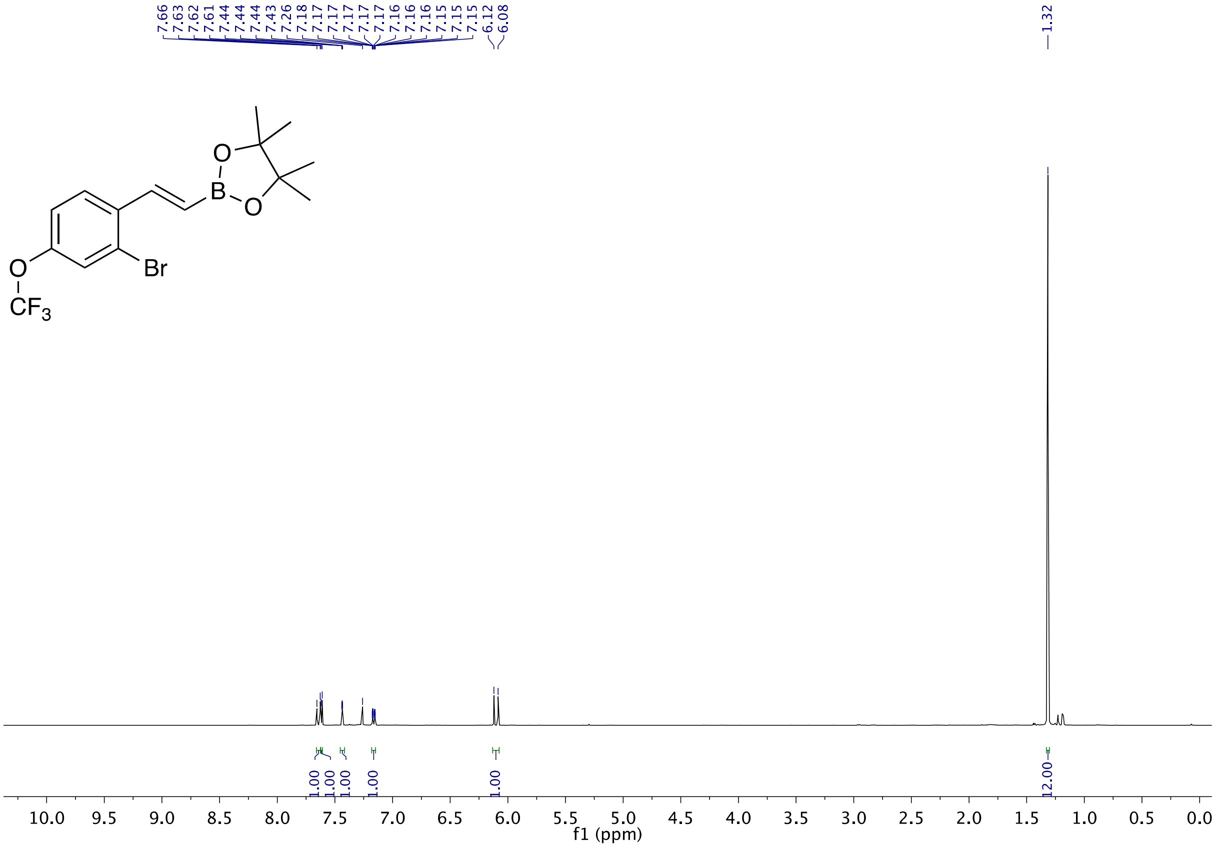

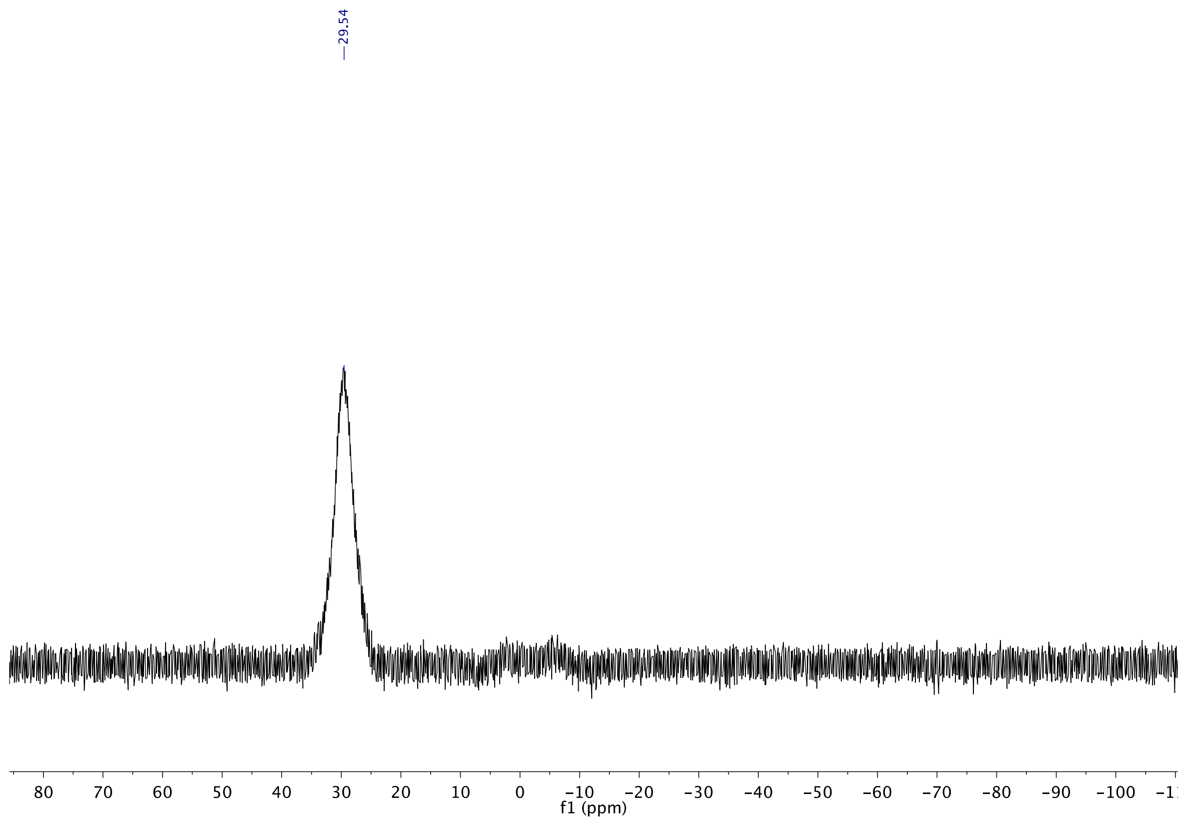

29.54
80 70 60 50 40 30 20 10 0 −10 −20 −30 −40 −50 −60 −70 −80 −90 −100
f1 (ppm)

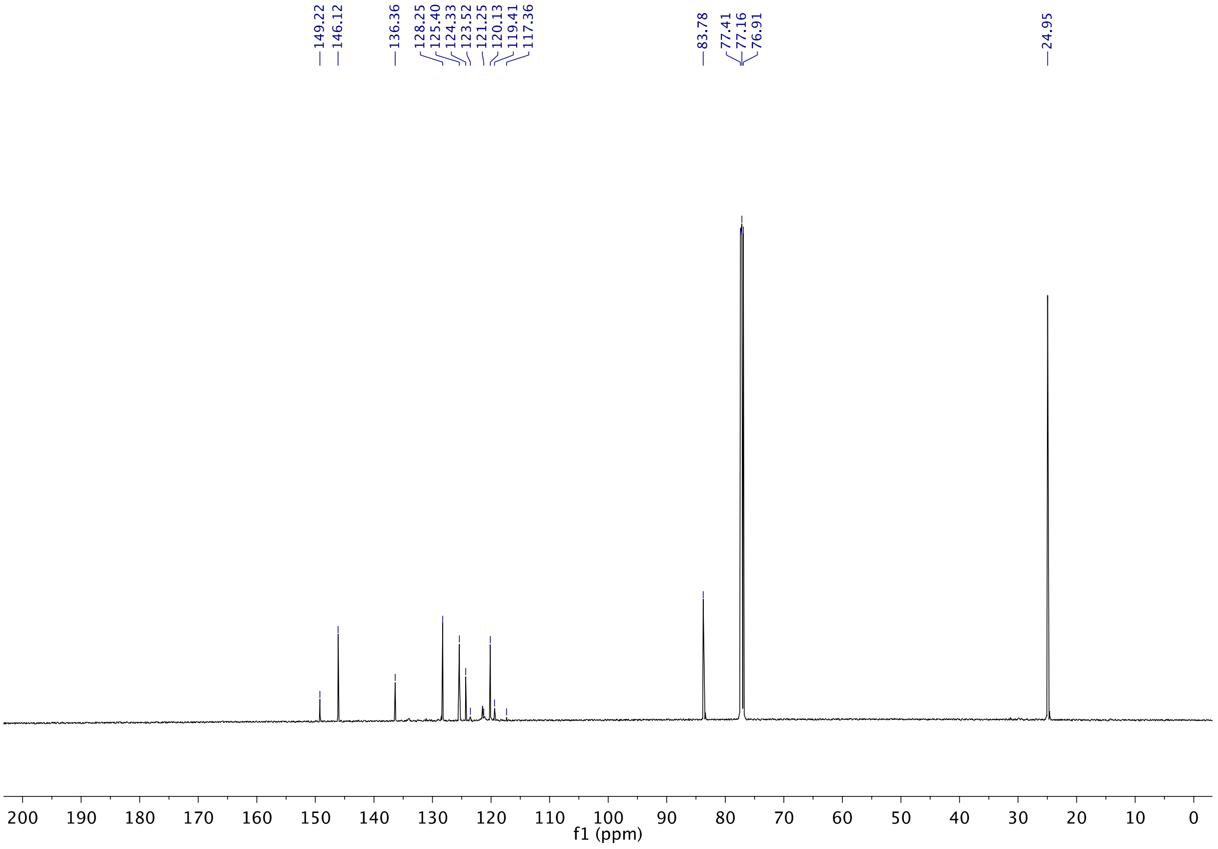

149.22
146.12
136.36
128.25
125.40
124.33
123.52
121.25
120.13
119.41
117.36
83.78
77.41
77.16
76.91
24.95
200 190 180 170 160 150 140 130 120 110 100 90 80 70 60 50 40 30 20 10 0
f1 (ppm)

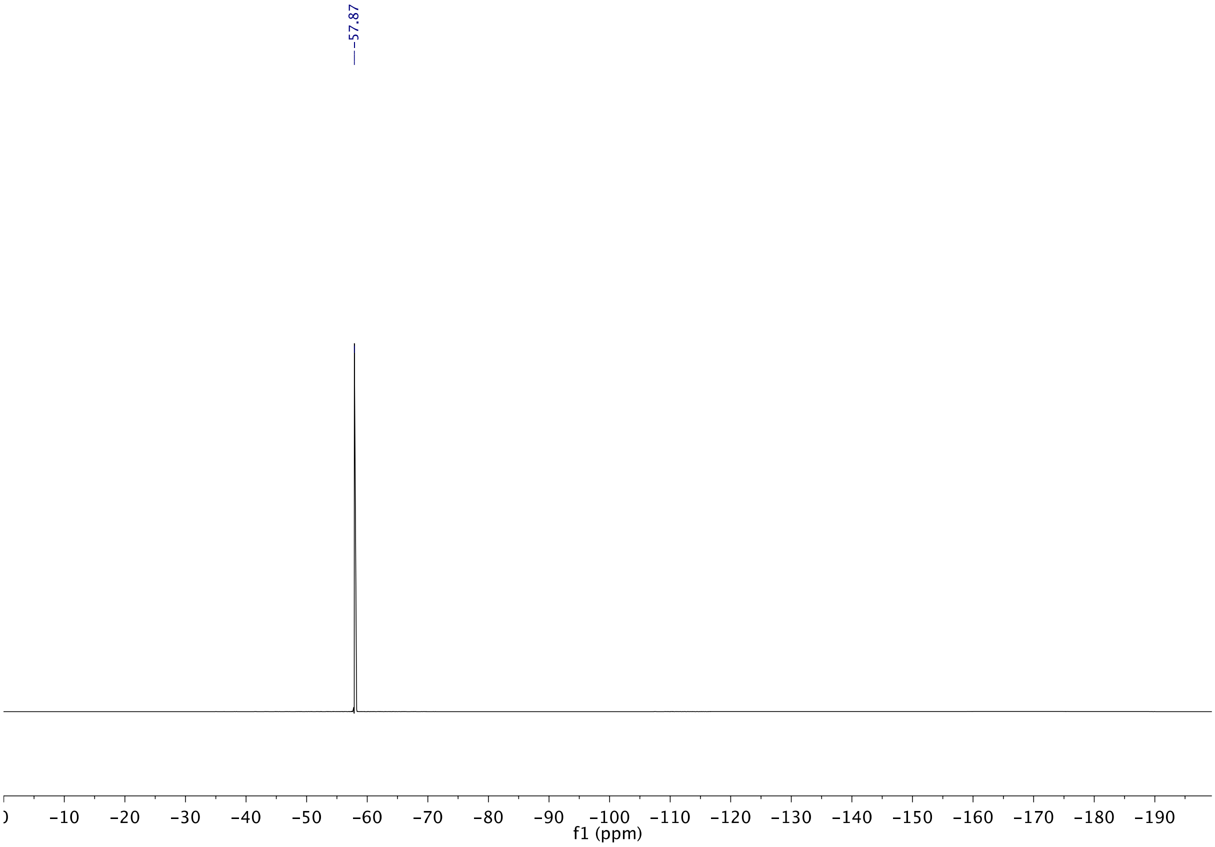

(*E*)-(2-Bromo-4-(trifluoromethoxy)styryl)trifluoro-λ4-borane, potassium salt, **S11-int4**

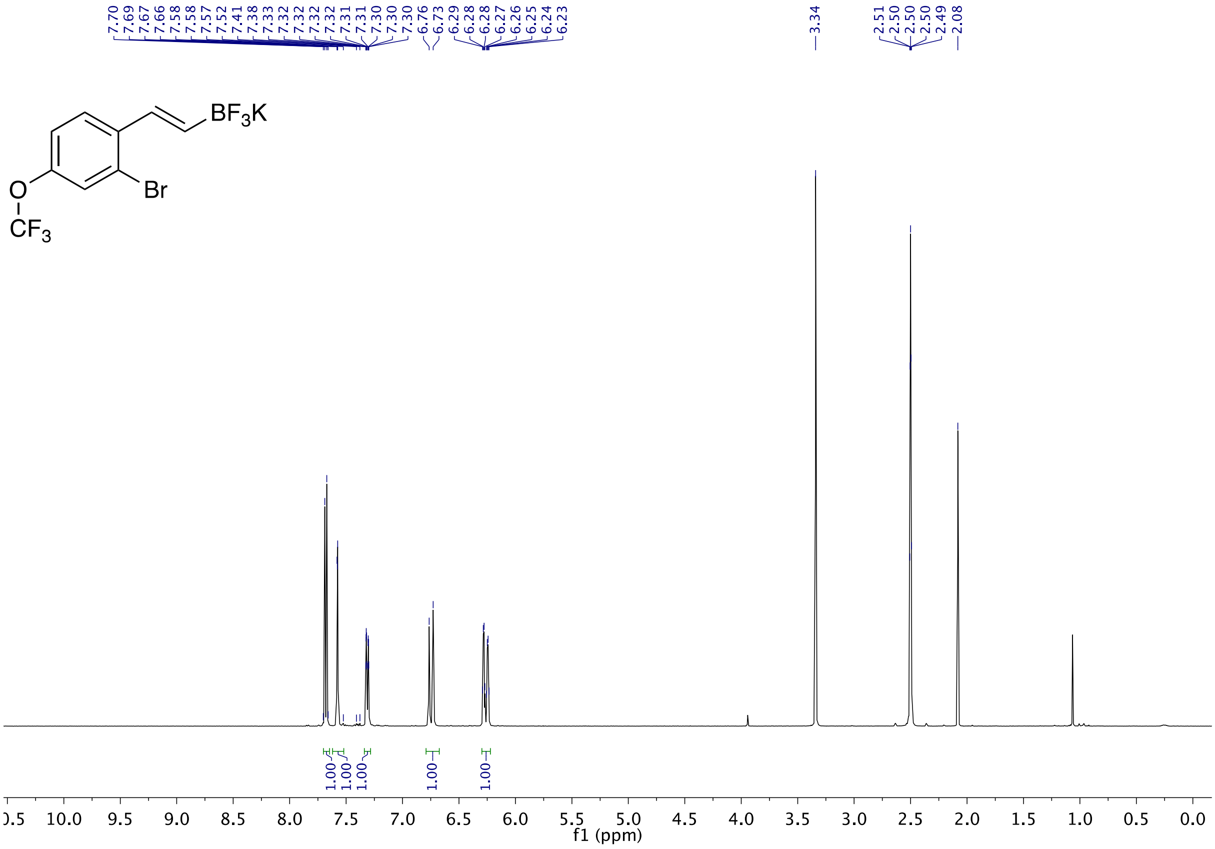

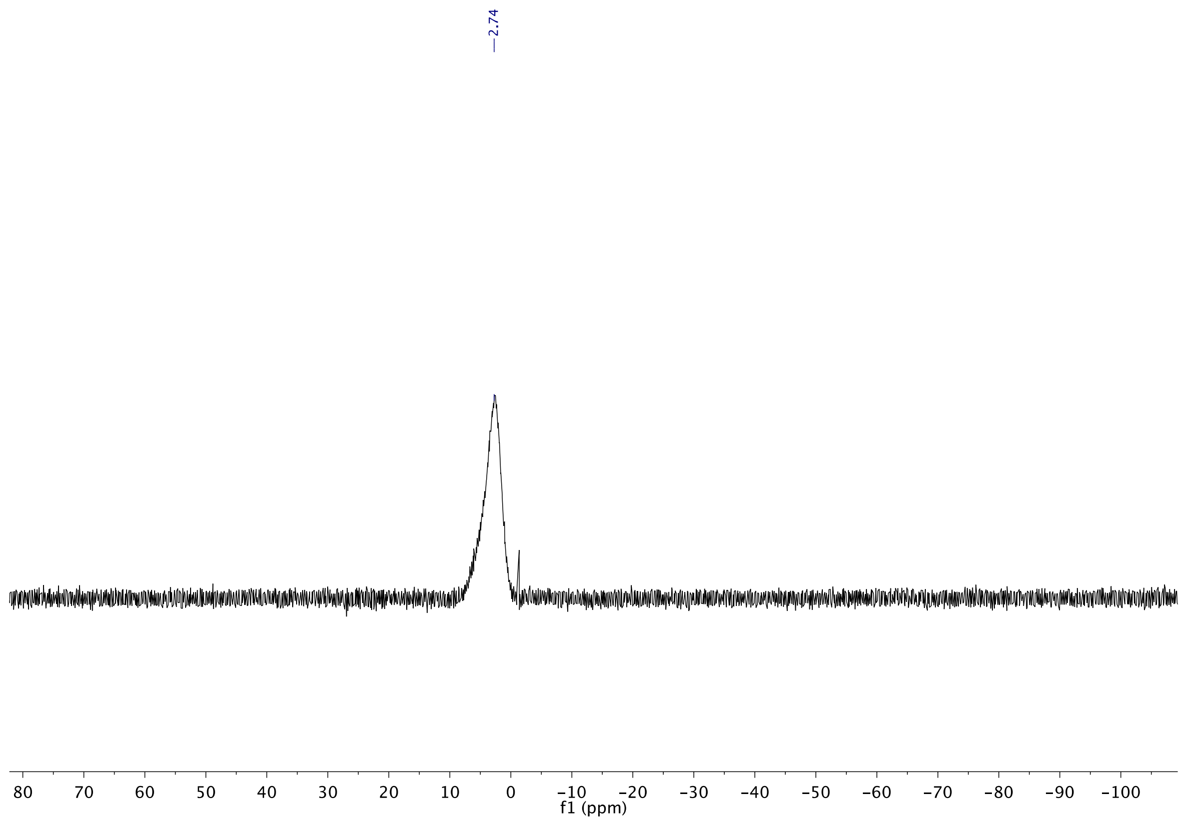

2.74
80 70 60 50 40 30 20 10 0 −10 −20 −30 −40 −50 −60 −70 −80 −90 −100
f1 (ppm)

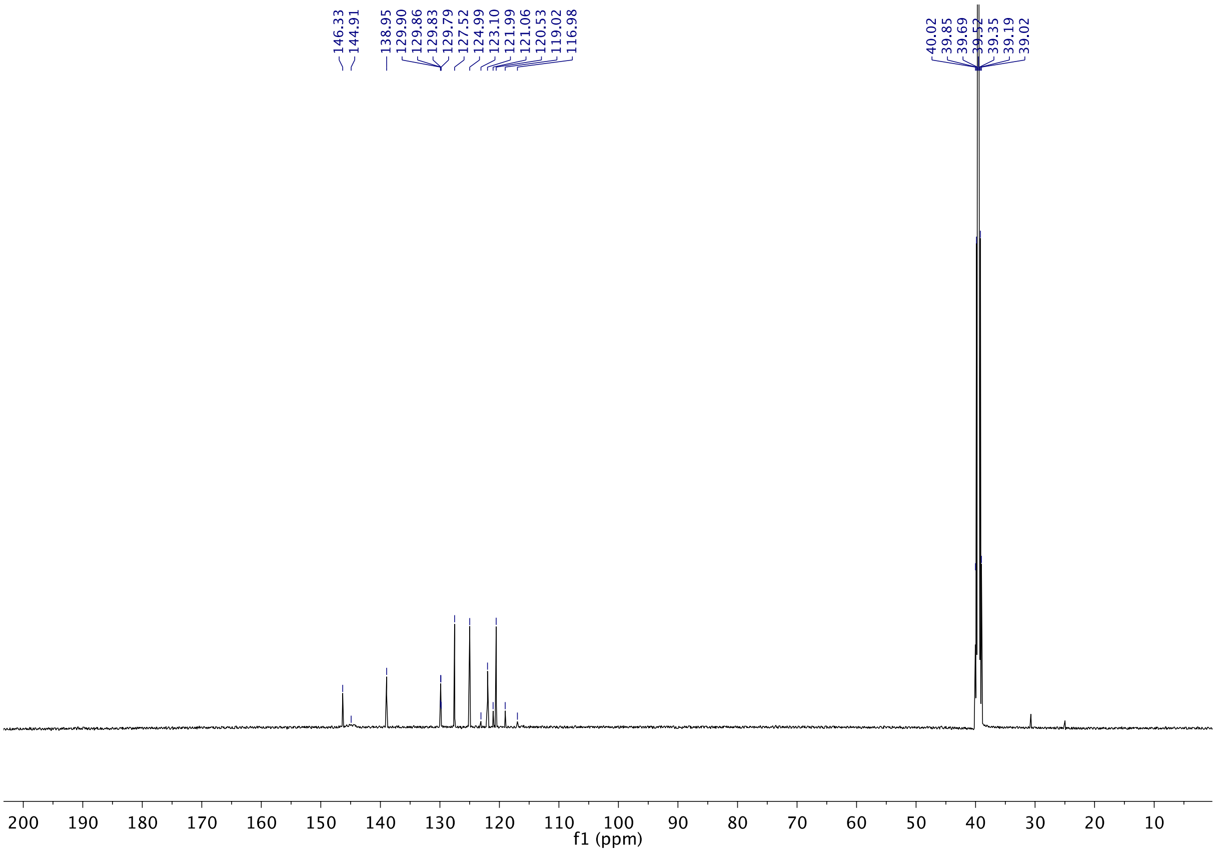

146.33
144.91
138.95
129.90
129.86
129.83
129.79
127.52
124.99
123.10
121.99
121.06
120.53
119.02
116.98
40.02
39.85
39.69
39.52
39.35
39.19
39.02
200 190 180 170 160 150 140 130 120 110 100 90 80 70 60 50 40 30 20 10
f1 (ppm)

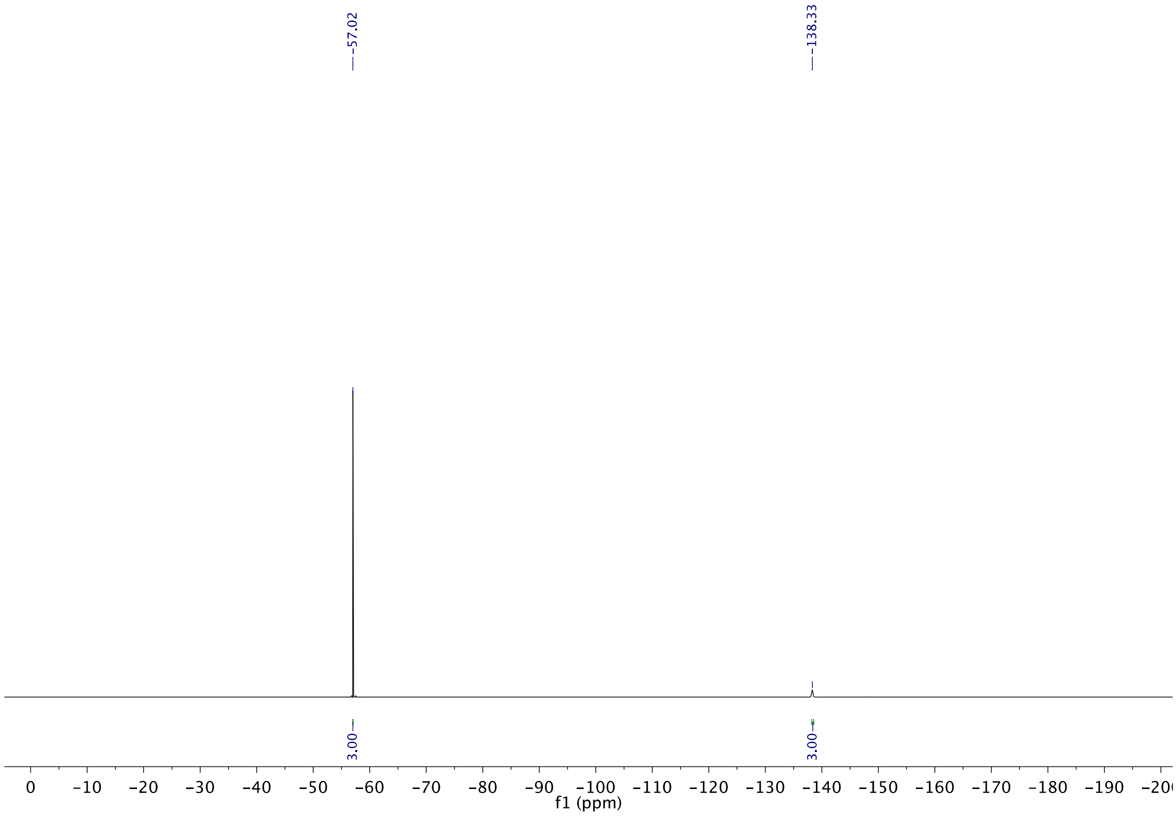

(*E*)-(2-Bromo-4-(trifluoromethoxy)styryl)boronic acid, **S11**

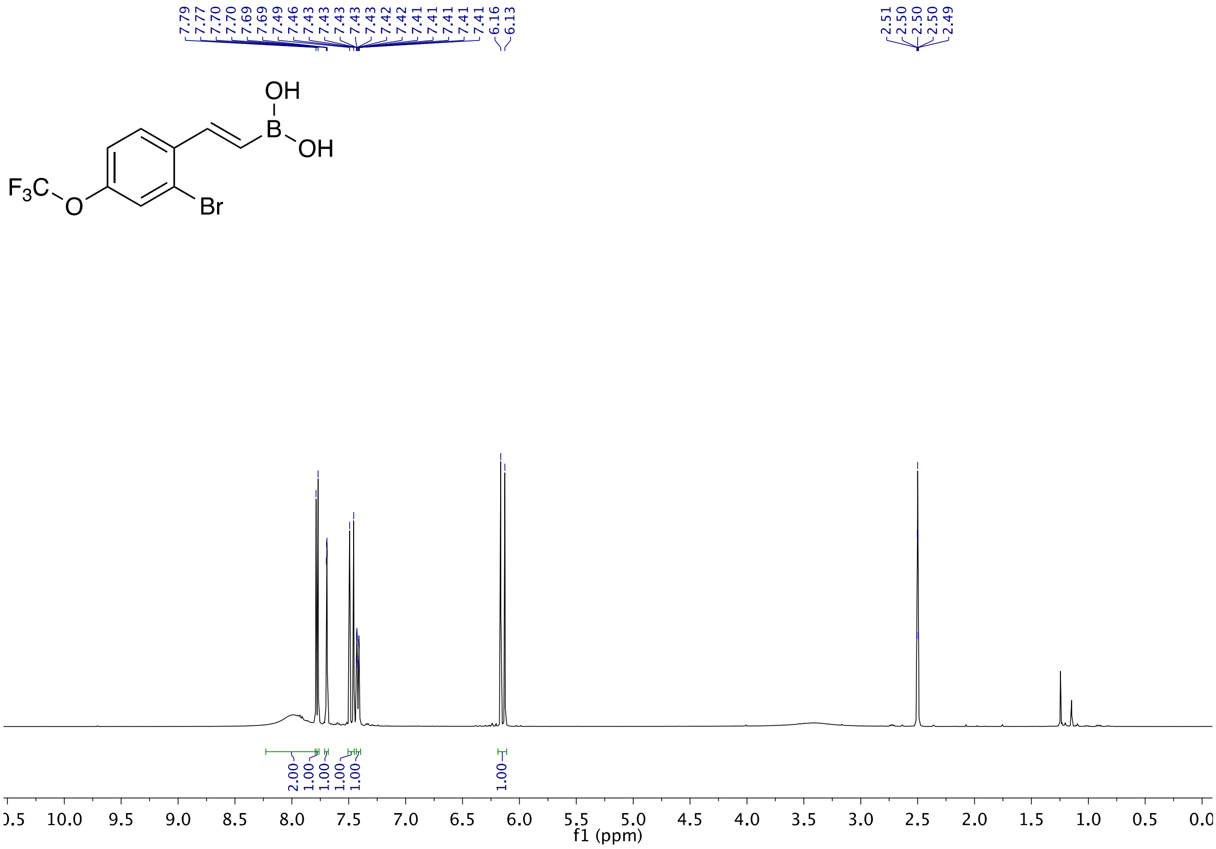

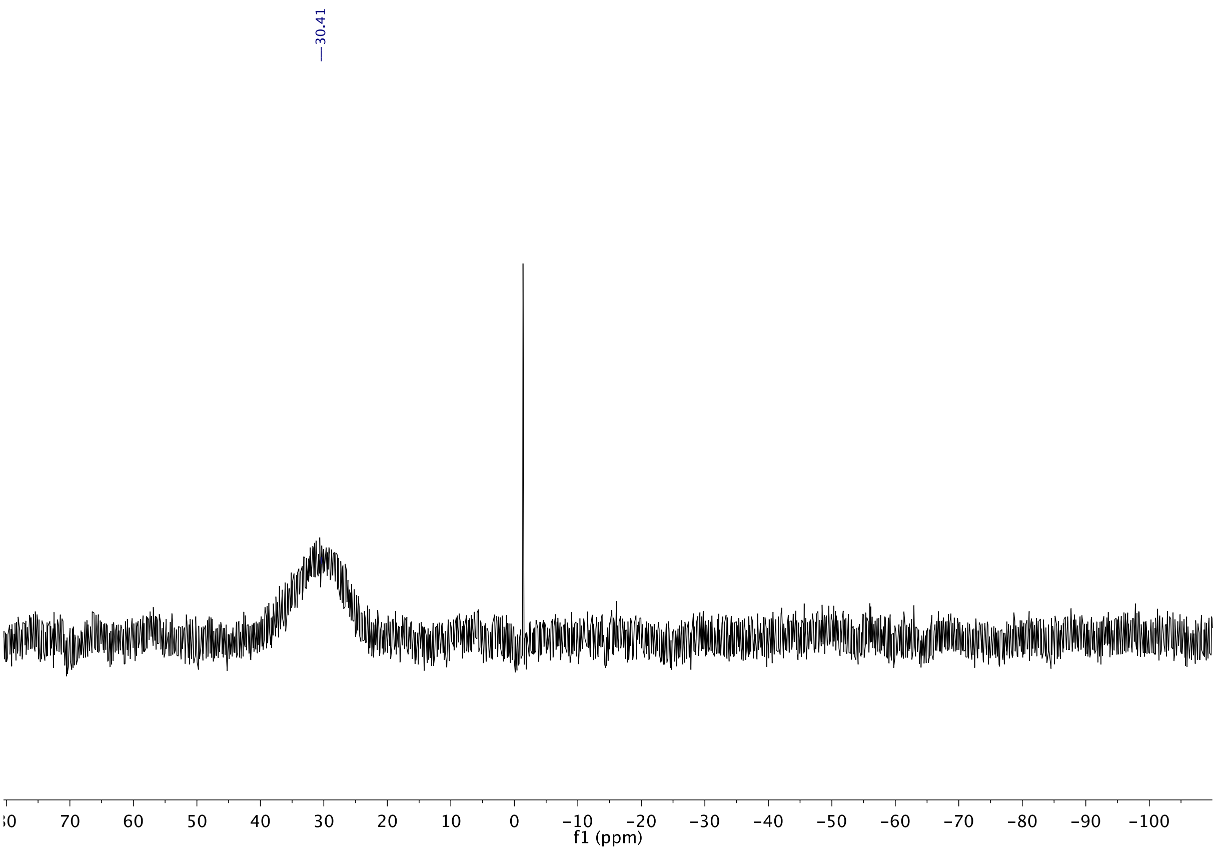
30.41
80
70
60
50
40
30
20
10
0
−10
−20
−30
−40
−50
−60
−70
−80
−90
−100
f1 (ppm)

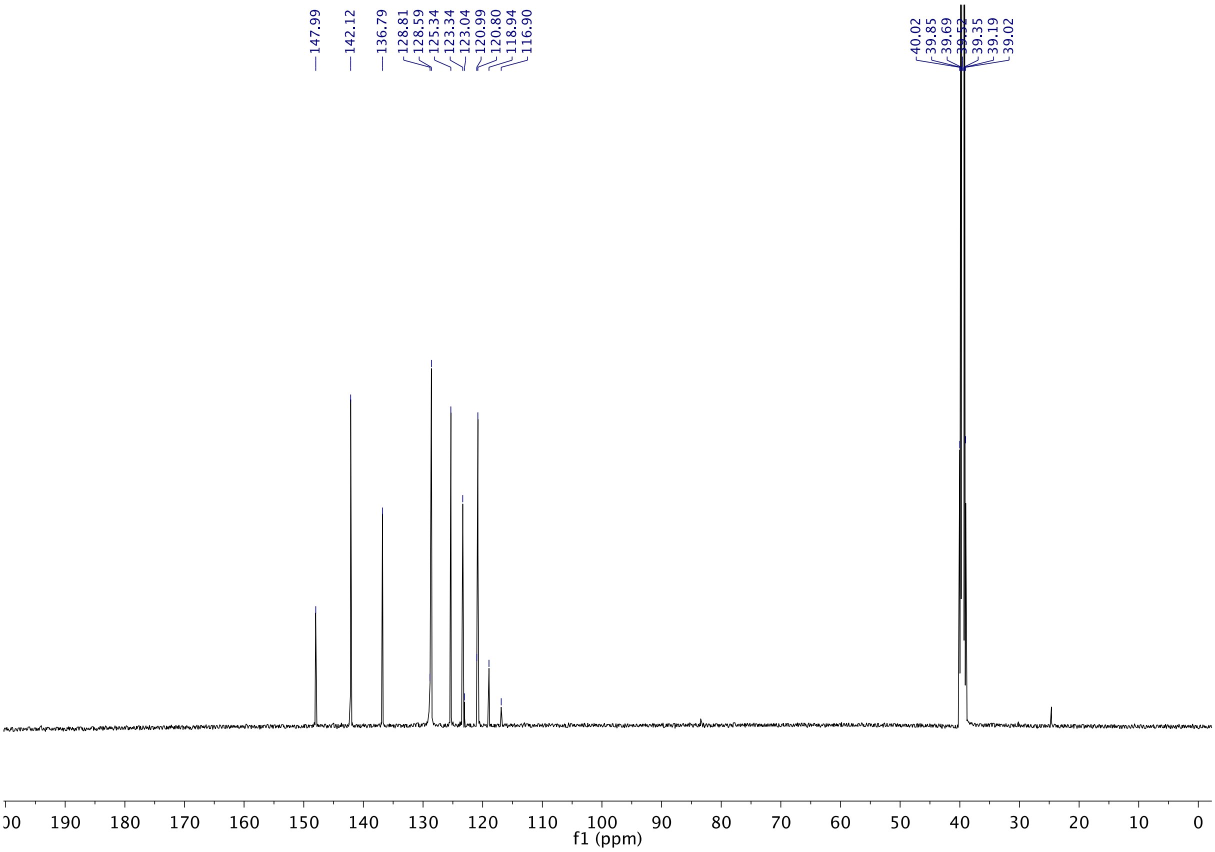
147.99
142.12
136.79
128.81
128.59
125.34
123.34
123.04
120.99
120.80
118.94
116.90
40.02
39.85
39.69
39.52
39.35
39.19
39.02
200
190
180
170
160
150
140
130
120
110
100
90
80
70
60
50
40
30
20
10
0
f1 (ppm)

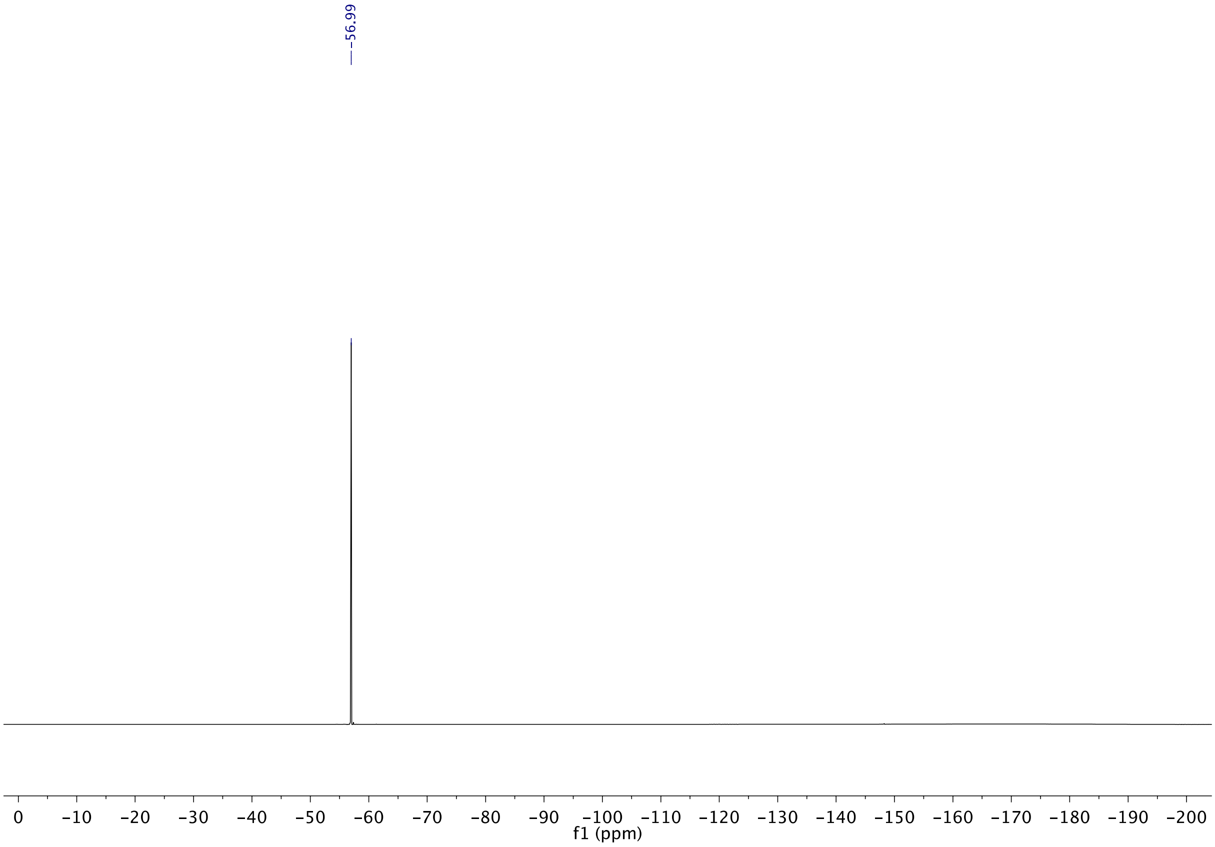

4,4,5,5-Tetramethyl-2-((1*E*,3*E*)-4-phenylbuta-1,3-dien-1-yl)-1,3,2-dioxaborolane, **S12-int3**

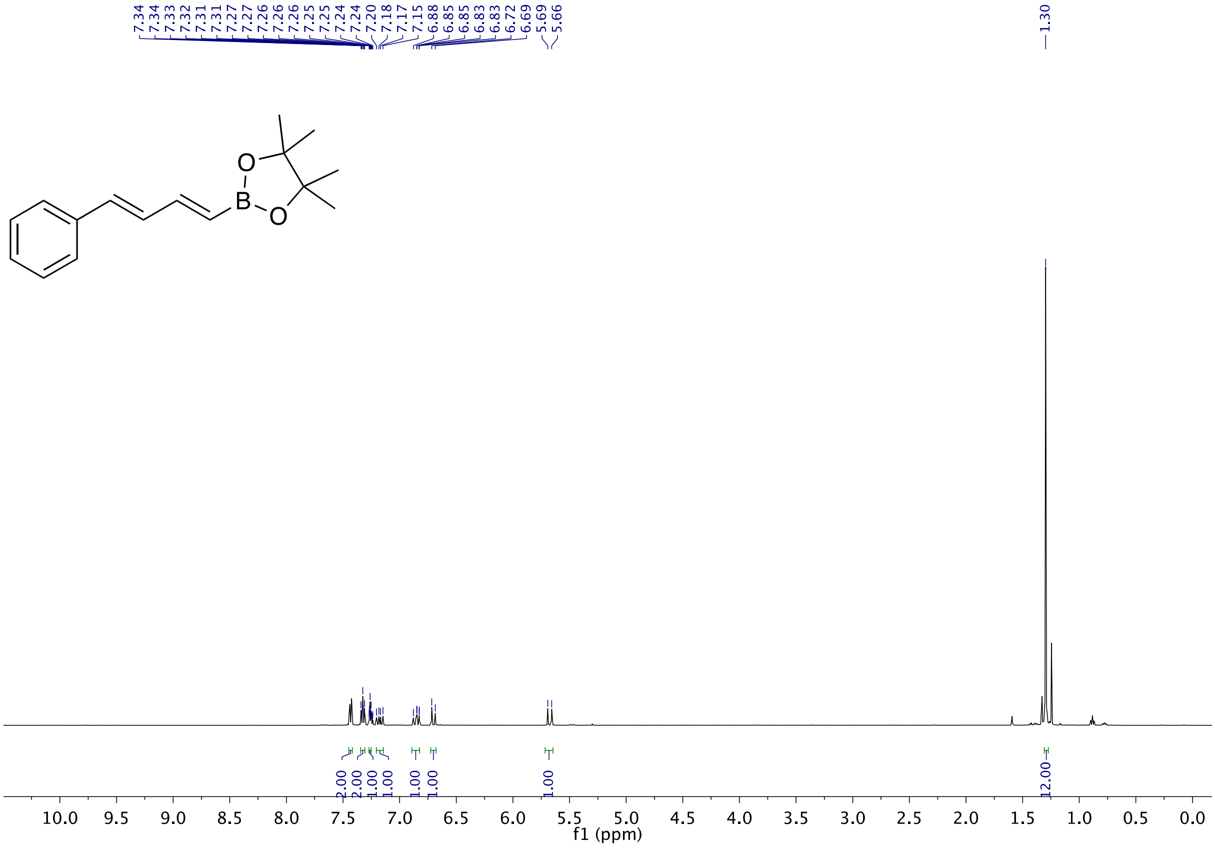

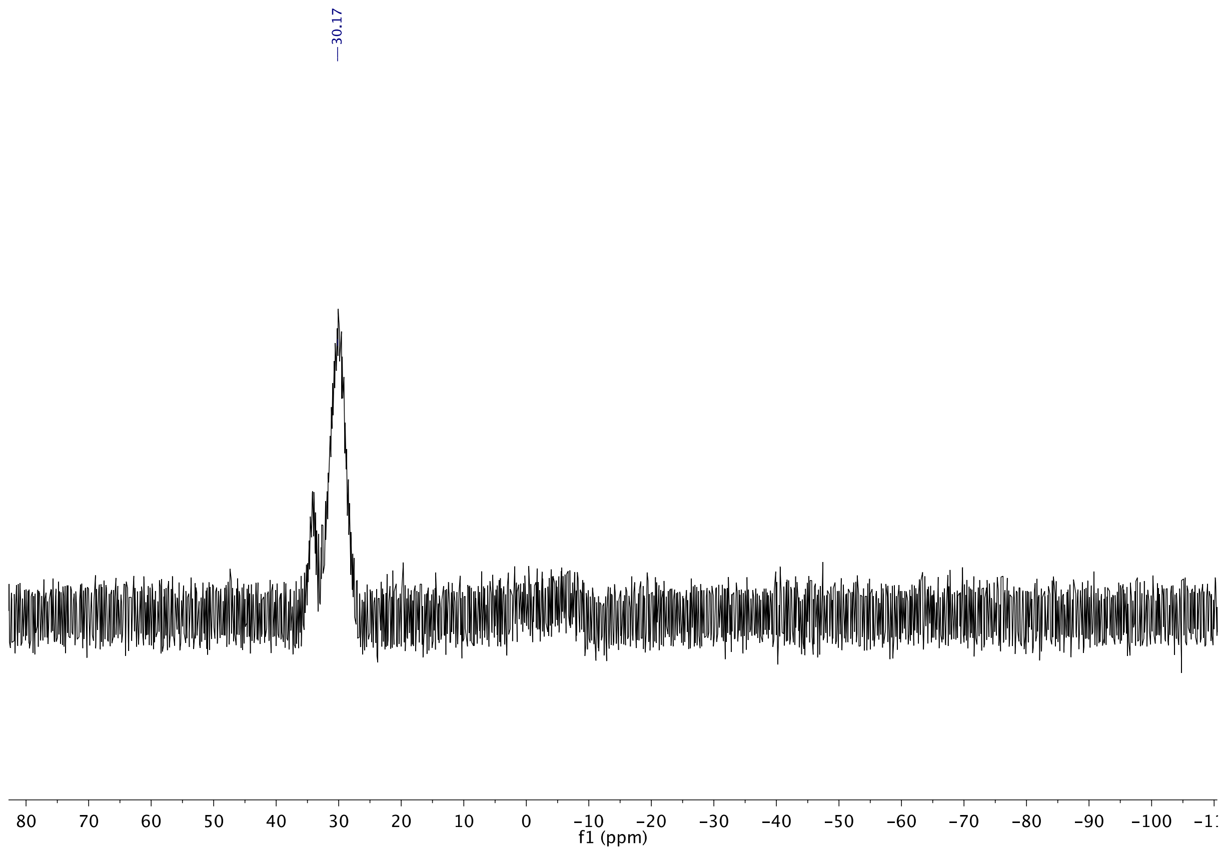
30.17
80 70 60 50 40 30 20 10 0 −10 −20 −30 −40 −50 −60 −70 −80 −90 −100
f1 (ppm)

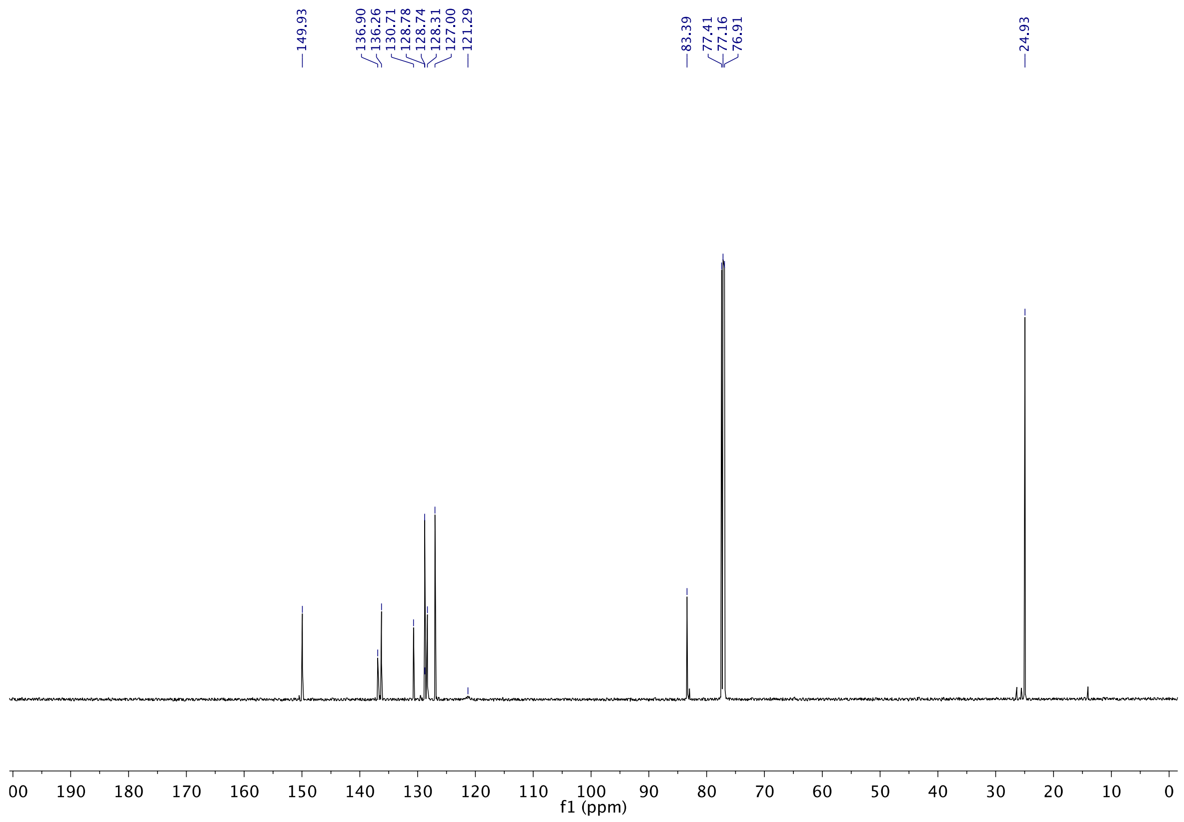
149.93
136.90
136.26
130.71
128.78
128.74
128.31
127.00
121.29
83.39
77.41
77.16
76.91
24.93
190 180 170 160 150 140 130 120 110 100 90 80 70 60 50 40 30 20 10 0
f1 (ppm)

Trifluoro((1*E*,3*E*)-4-phenylbuta-1,3-dien-1-yl)-λ[4]-borane, potassium salt, **S12-int4**

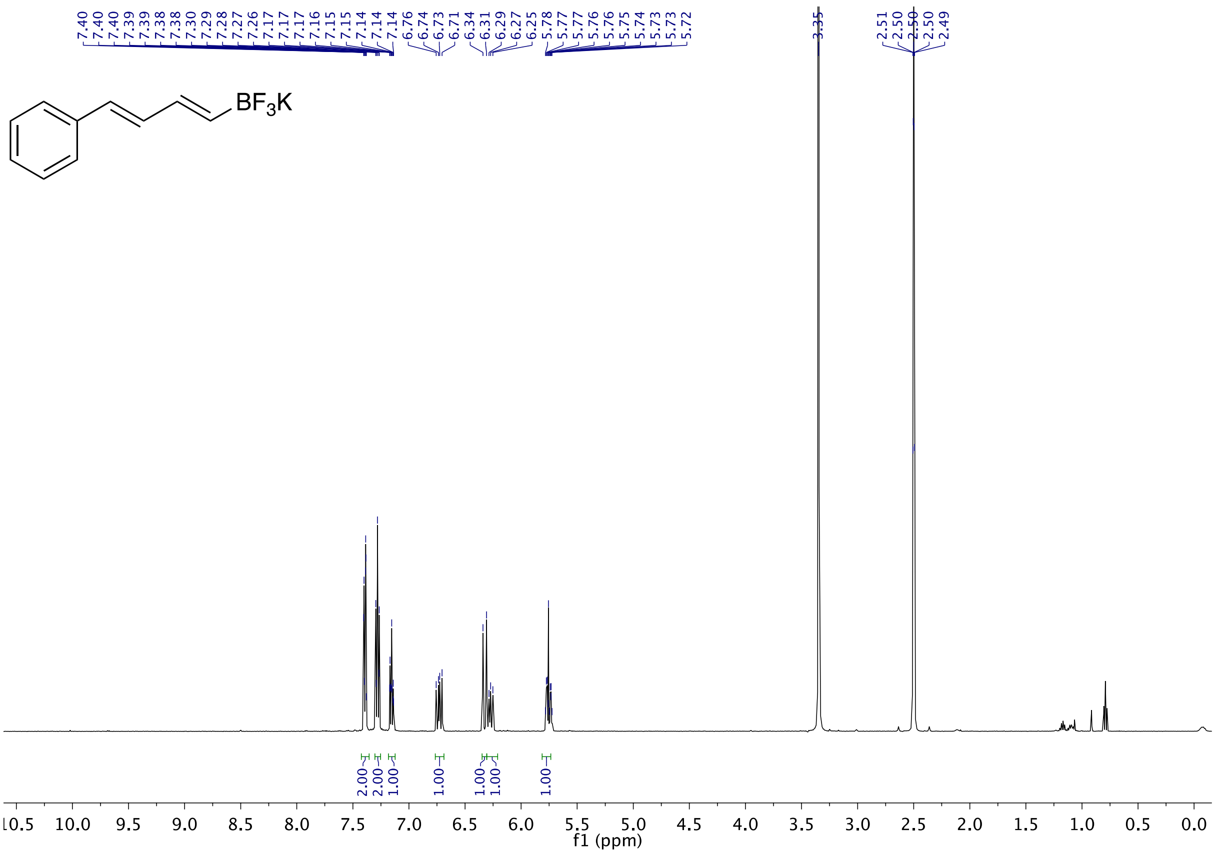

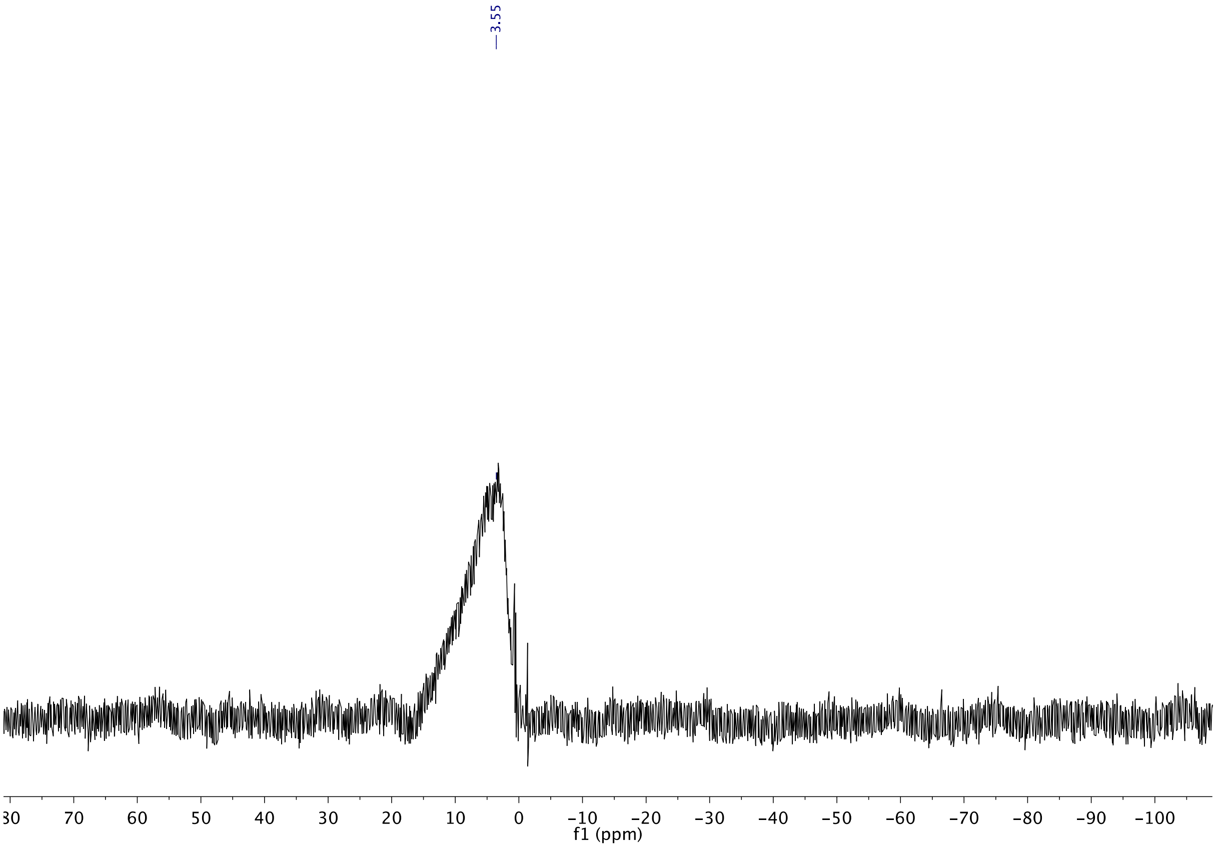

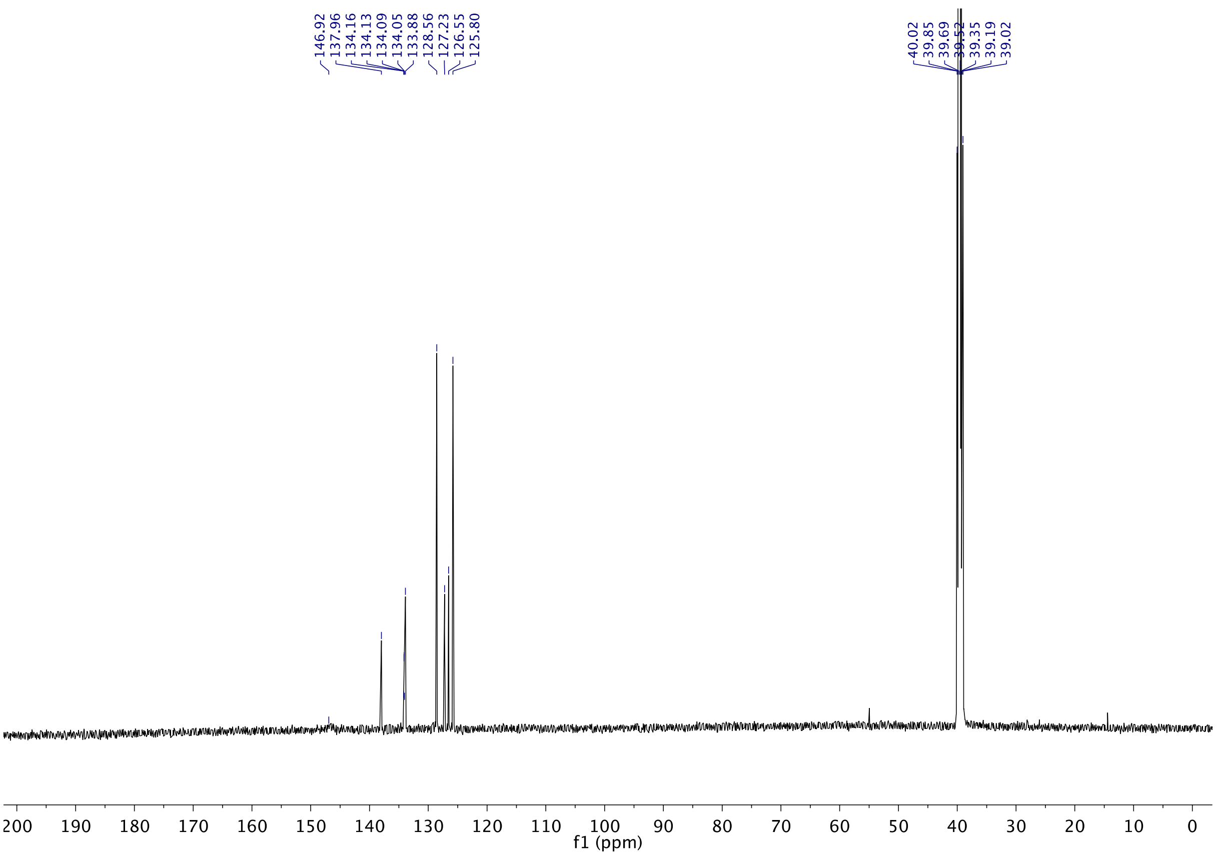
146.92
137.96
134.16
134.13
134.09
134.05
133.88
128.56
127.23
126.55
125.80
40.02
39.85
39.69
39.52
39.35
39.19
39.02
200 190 180 170 160 150 140 130 120 110 100 90 80 70 60 50 40 30 20 10 0
f1 (ppm)

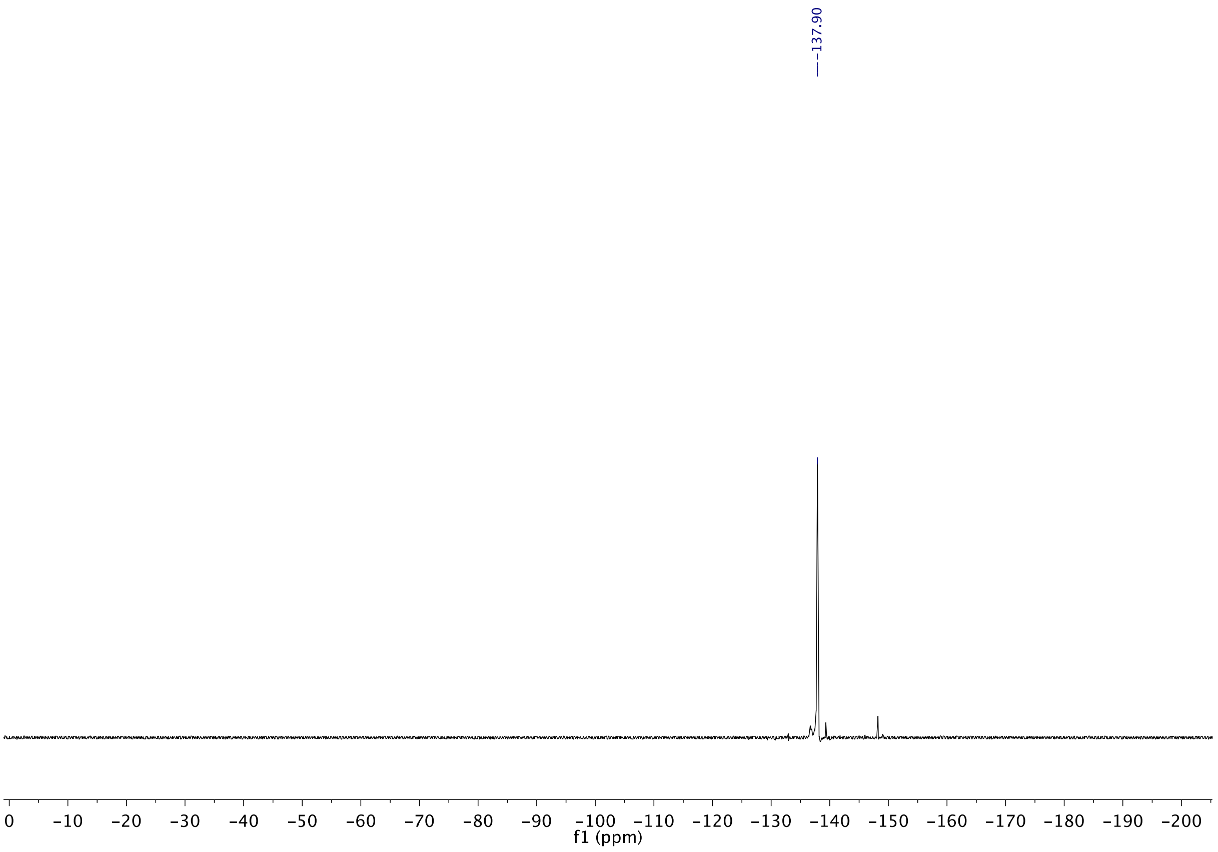
-137.90
0 −10 −20 −30 −40 −50 −60 −70 −80 −90 −100 −110 −120 −130 −140 −150 −160 −170 −180 −190 −200
f1 (ppm)

((1*E*,3*E*)-4-Phenylbuta-1,3-dien-1-yl)boronic acid, **S12**

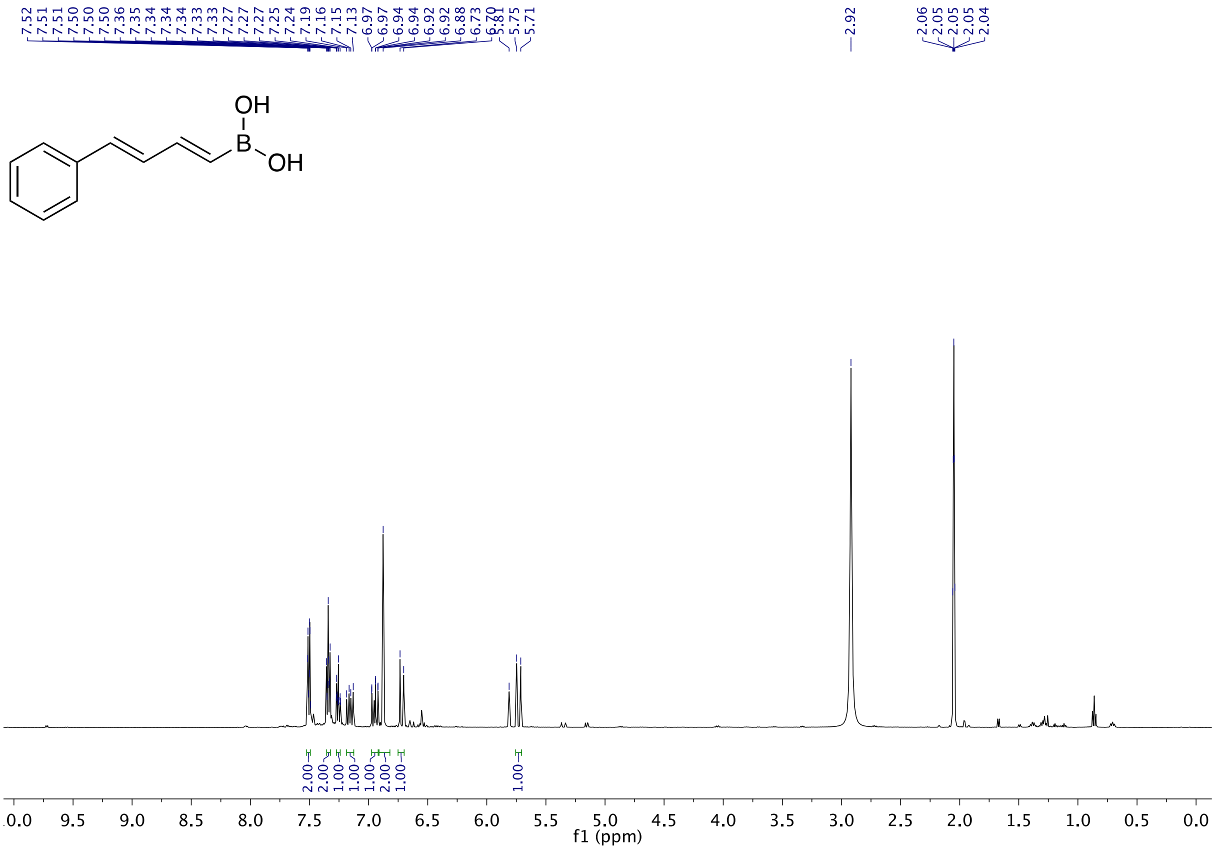

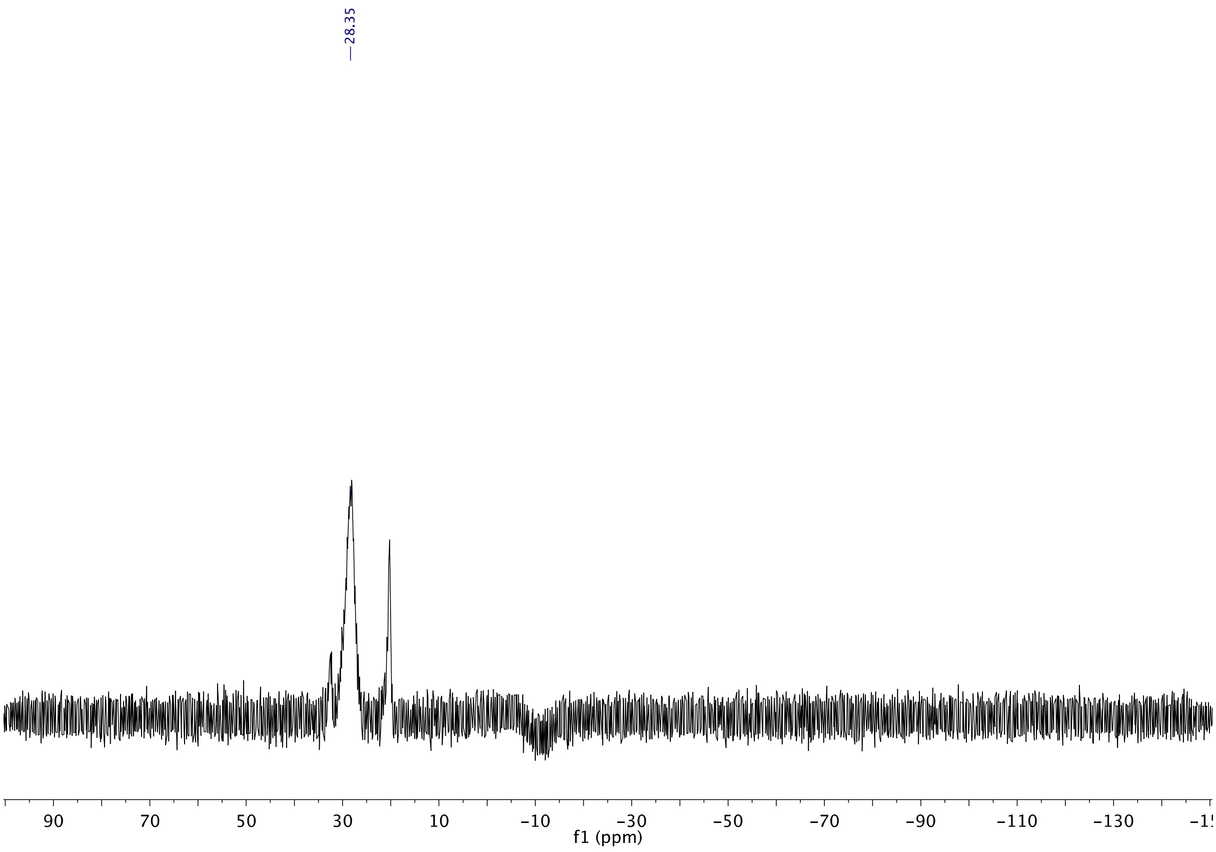

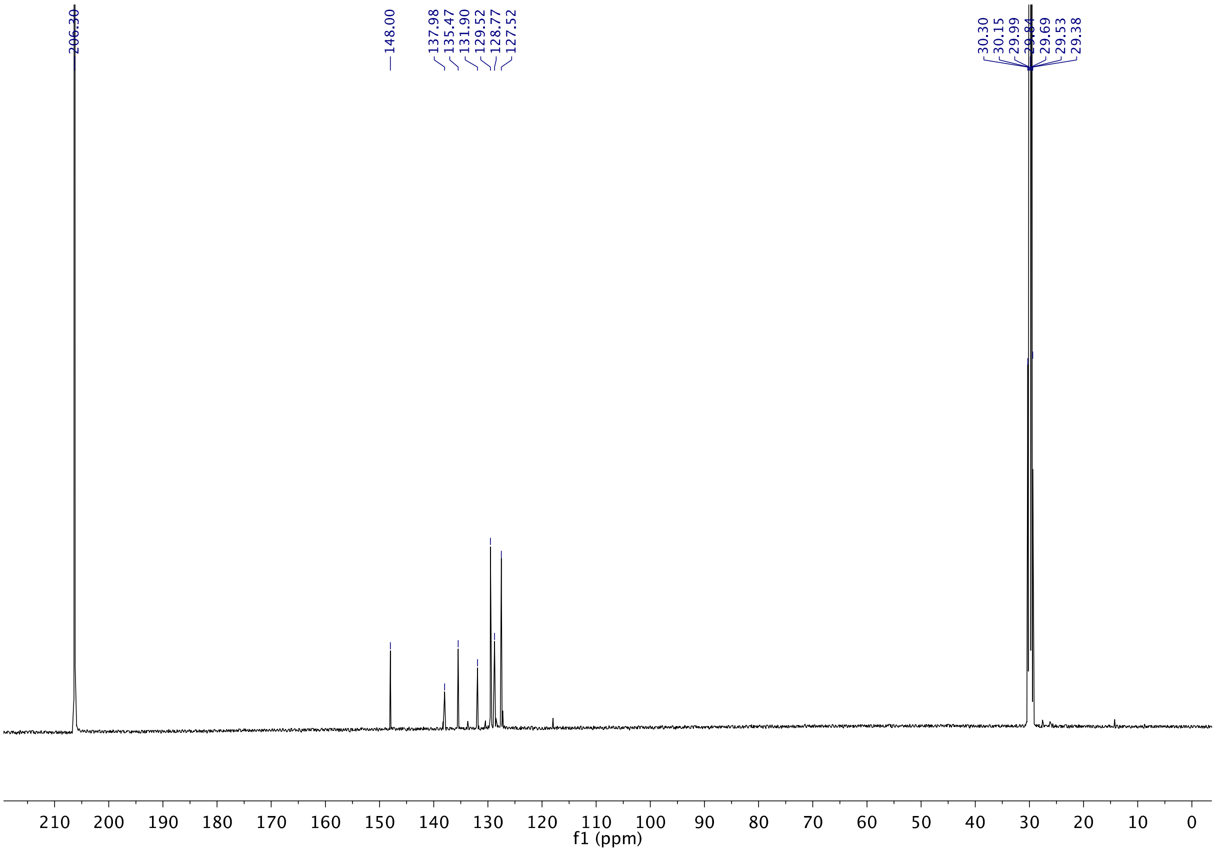

Methyl 2-((trimethylsilyl)ethynyl)benzoate, **S13-int1**

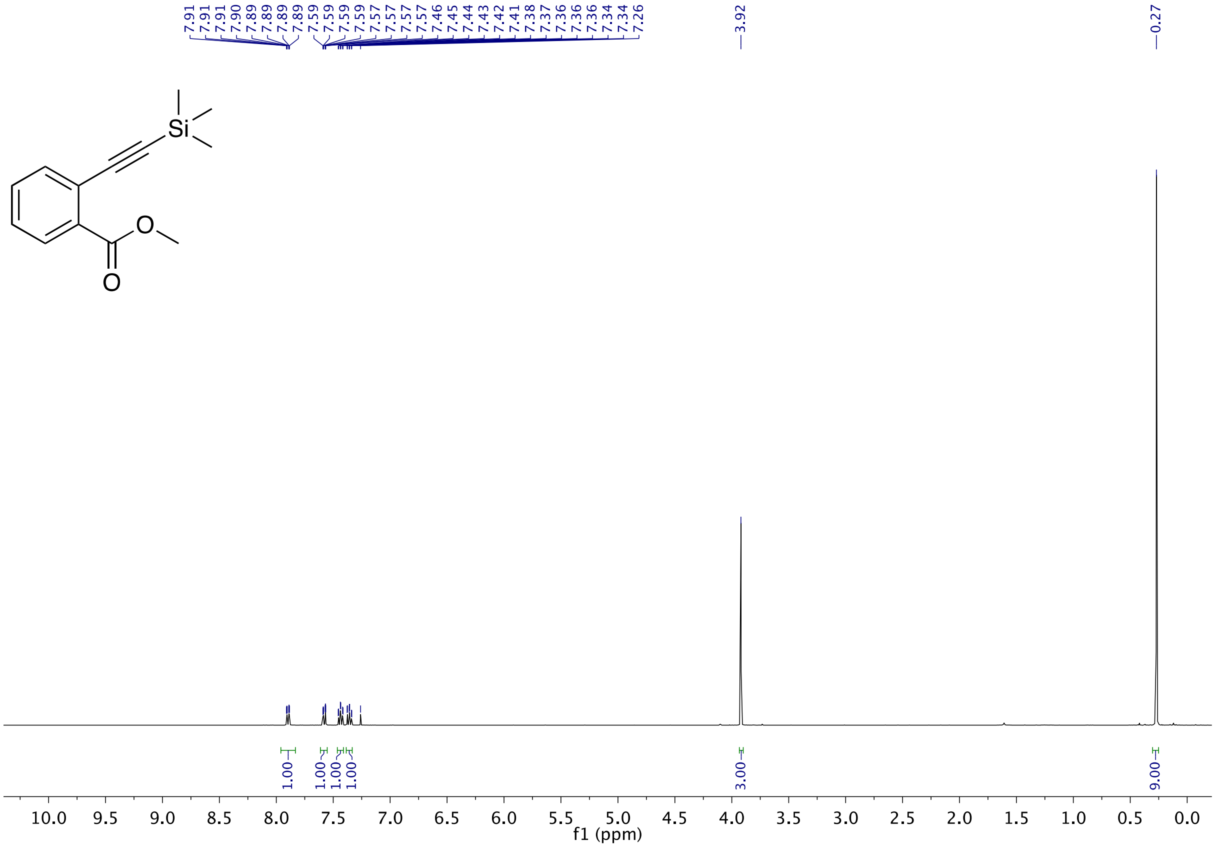

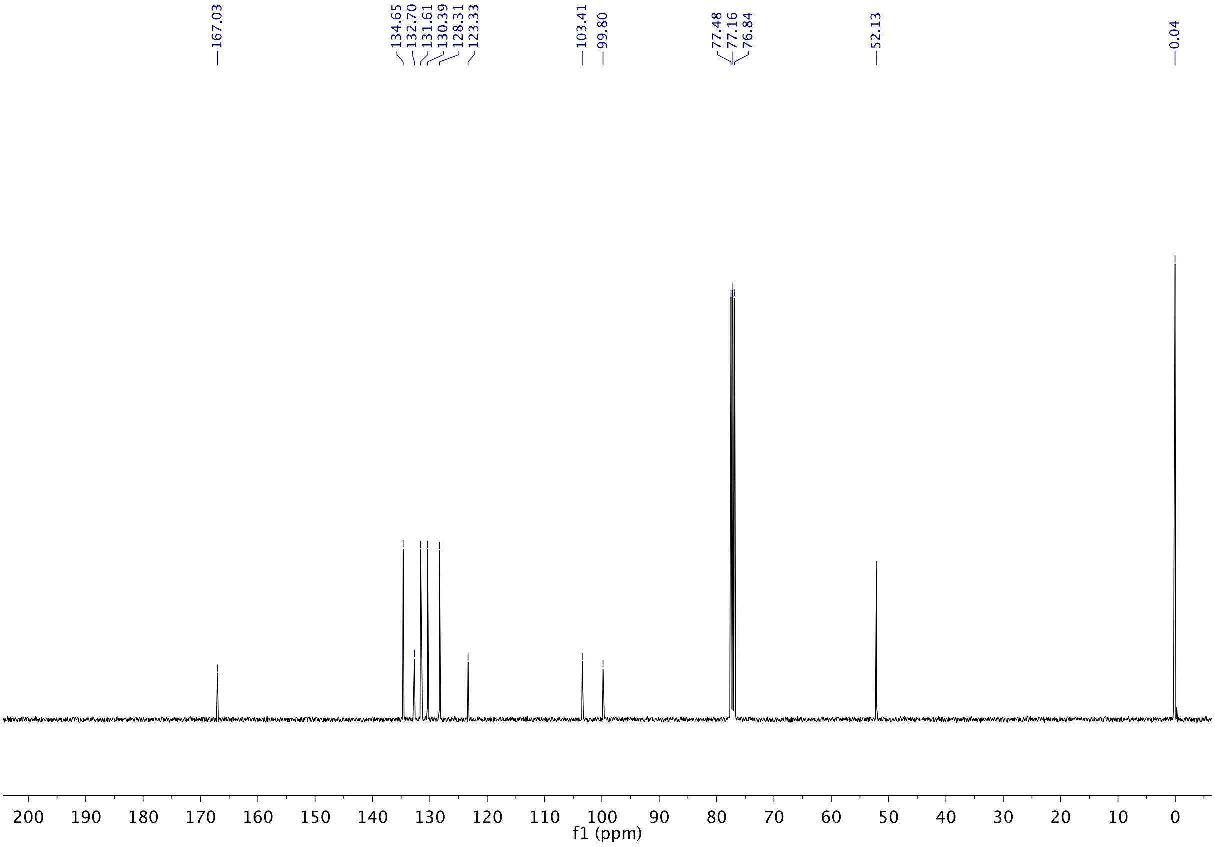

Methyl 2-ethynylbenzoate, **S13-int2**

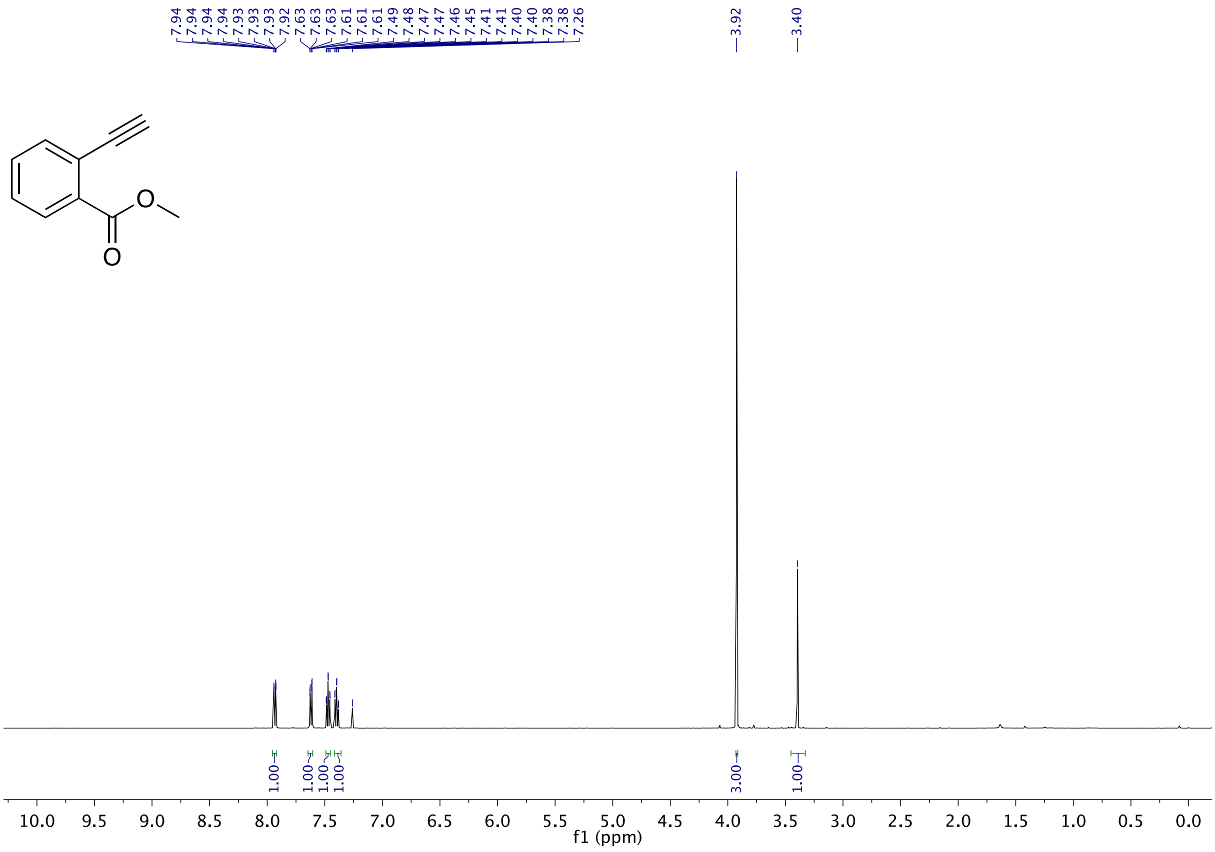

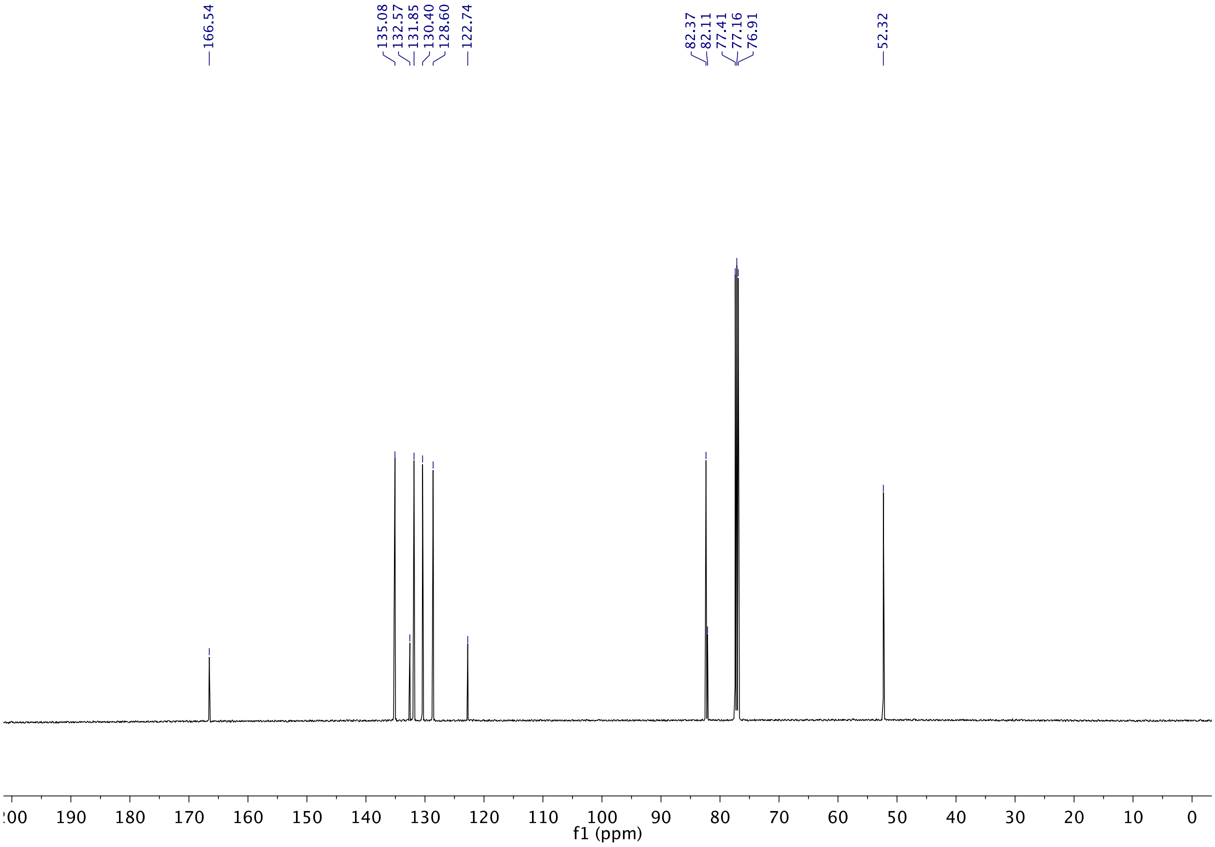

Methyl (*E*)-2-(2-(4,4,5,5-tetramethyl-1,3,2-dioxaborolan-2-yl)vinyl)benzoate, **S13-int3**

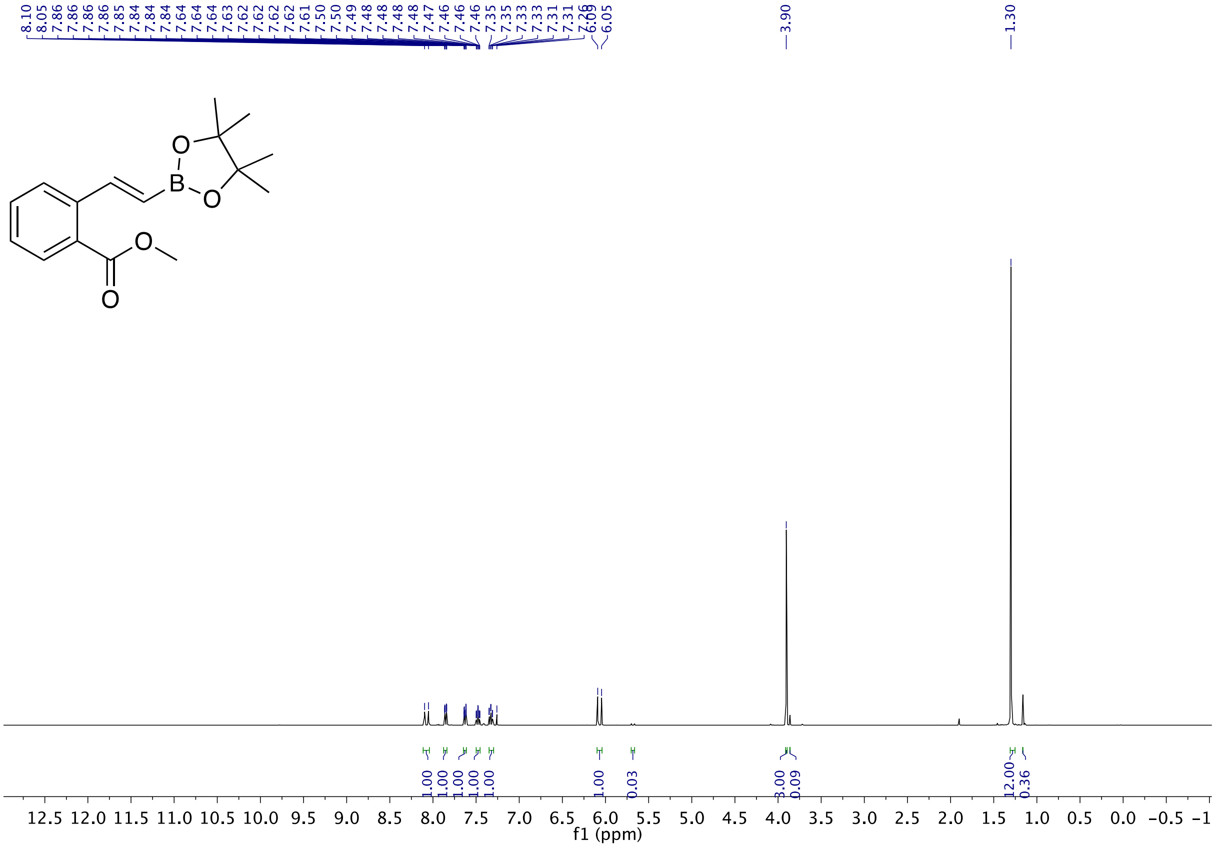

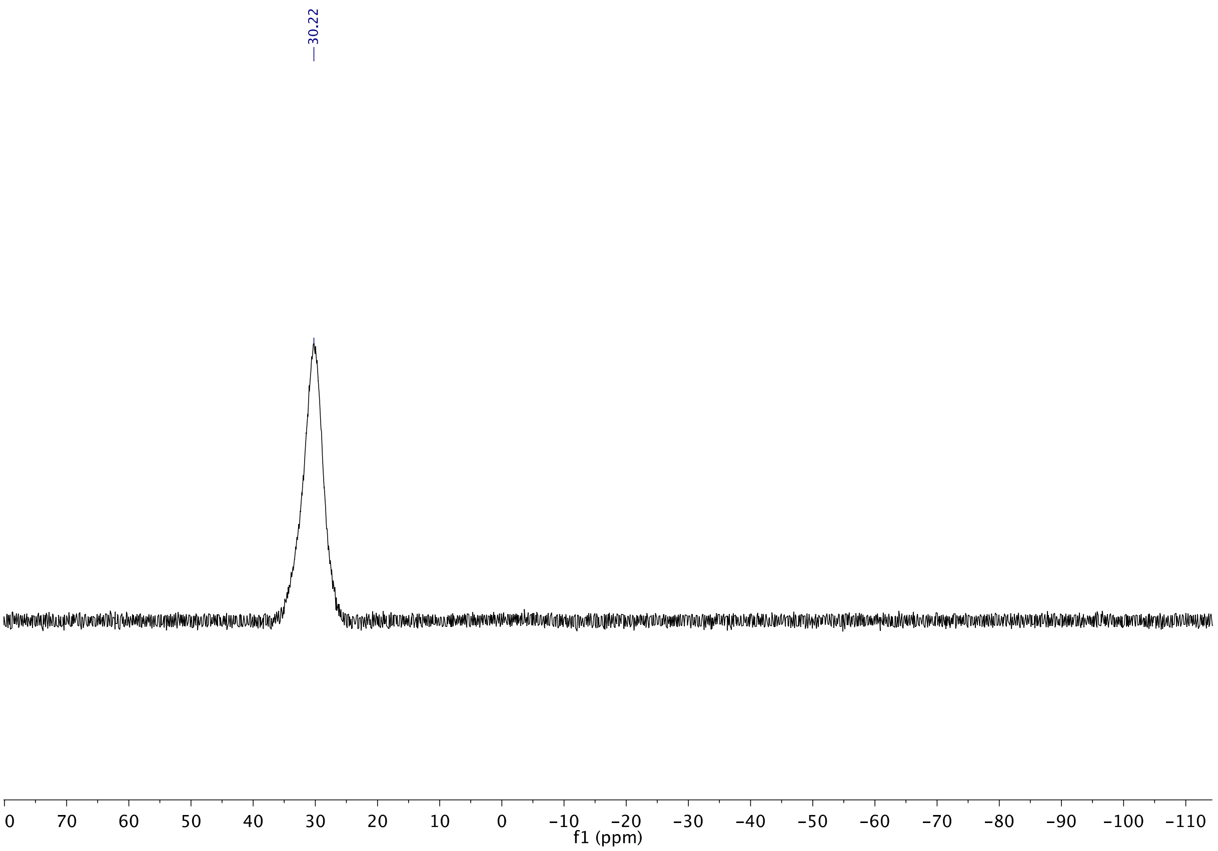
30.22
0
70
60
50
40
30
20
10
0
−10
−20
−30
−40
−50
−60
−70
−80
−90
−100
−110
f1 (ppm)

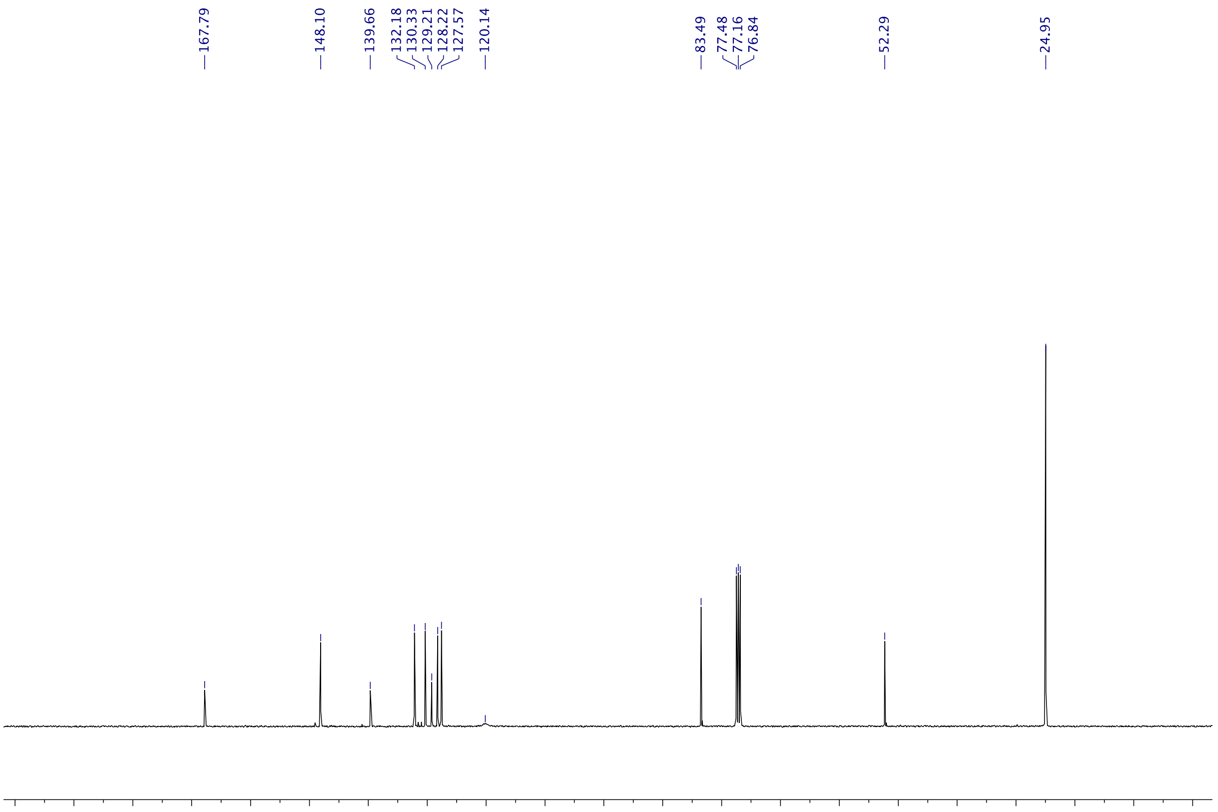
167.79
148.10
139.66
132.18
130.33
129.21
128.22
127.57
120.14
83.49
77.48
77.16
76.84
52.29
24.95
200
190
180
170
160
150
140
130
120
110
100
90
80
70
60
50
40
30
20
10
0
f1 (ppm)

Methyl (*E*)-2-(2-(trifluoro-λ[4]-boraneyl)vinyl)benzoate, potassium salt, **S13-int4**

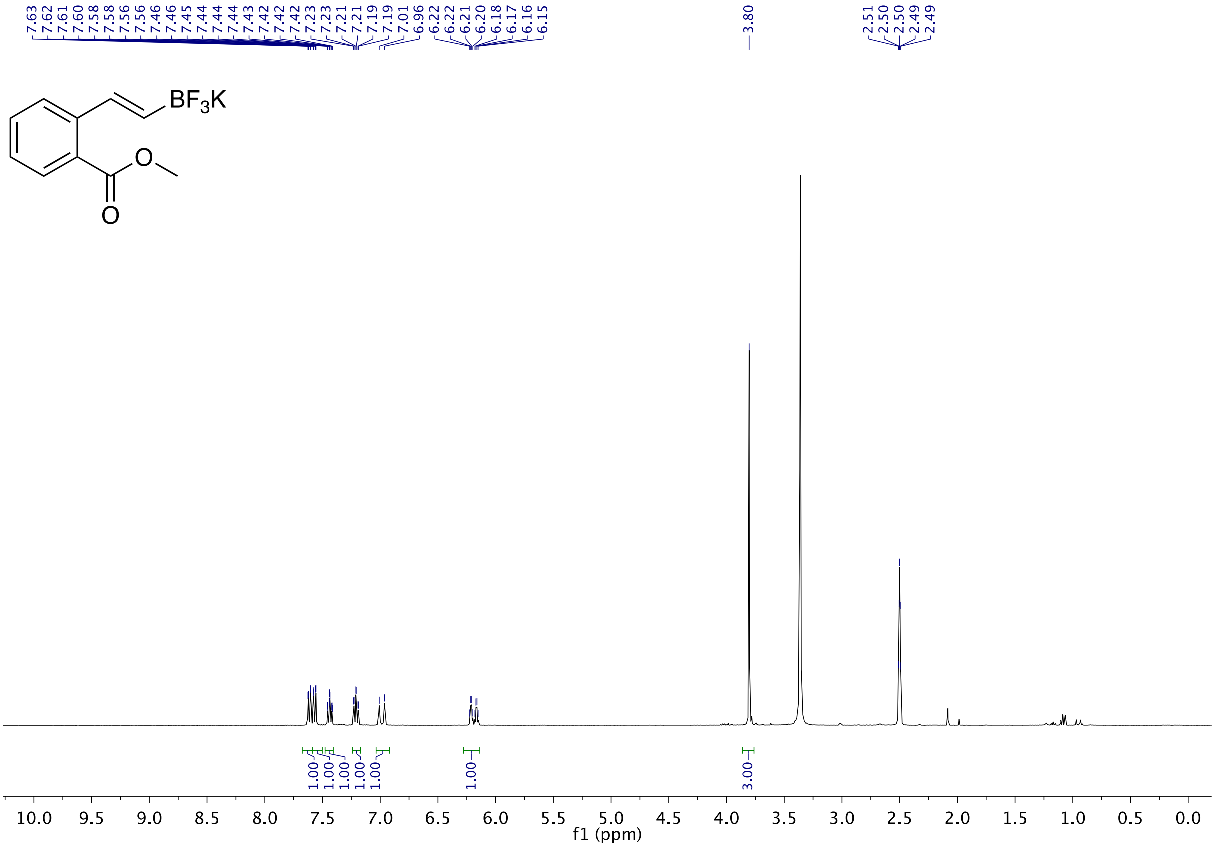

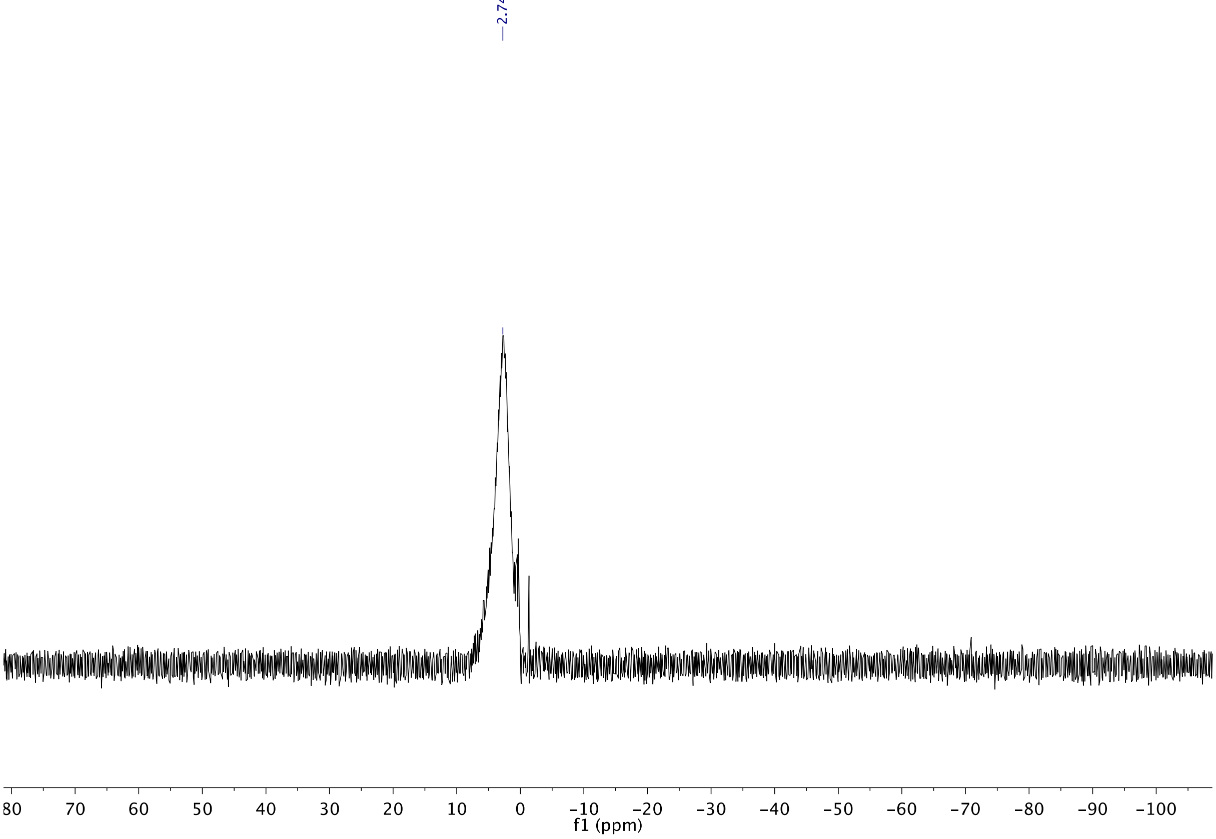

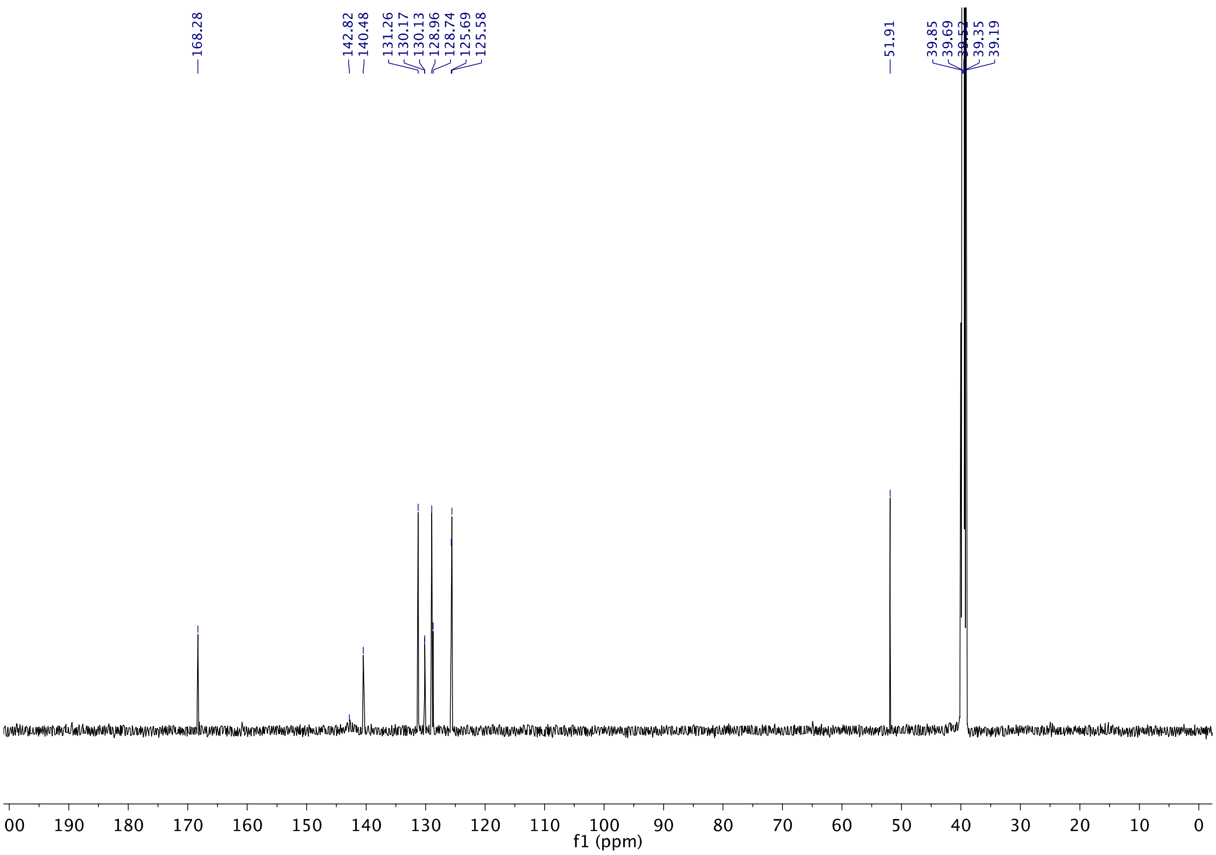
168.28
142.82
140.48
131.26
130.17
130.13
128.96
128.74
125.69
125.58
51.91
39.85
39.69
39.35
39.19
f1 (ppm)

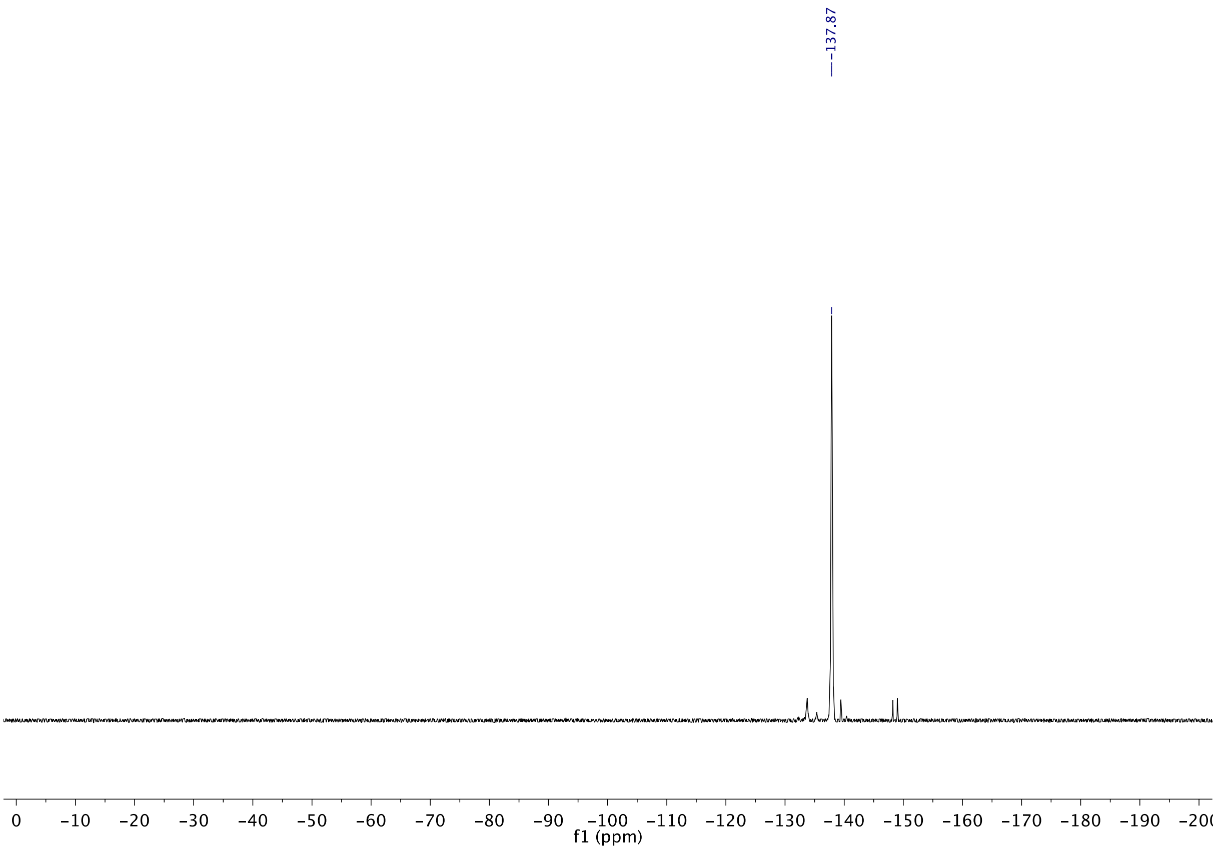
-137.87
f1 (ppm)

(*E*)-(2-(methoxycarbonyl)styryl)boronic acid, **S13**

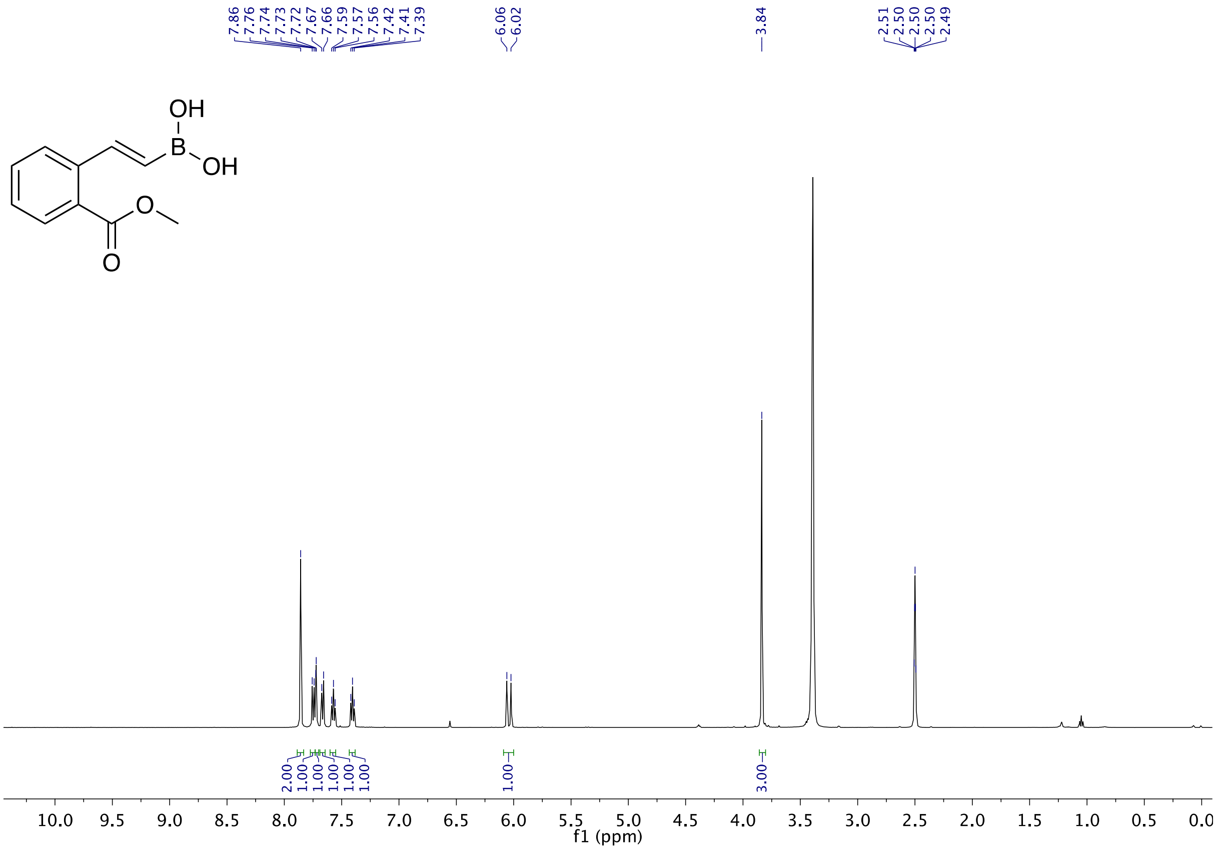

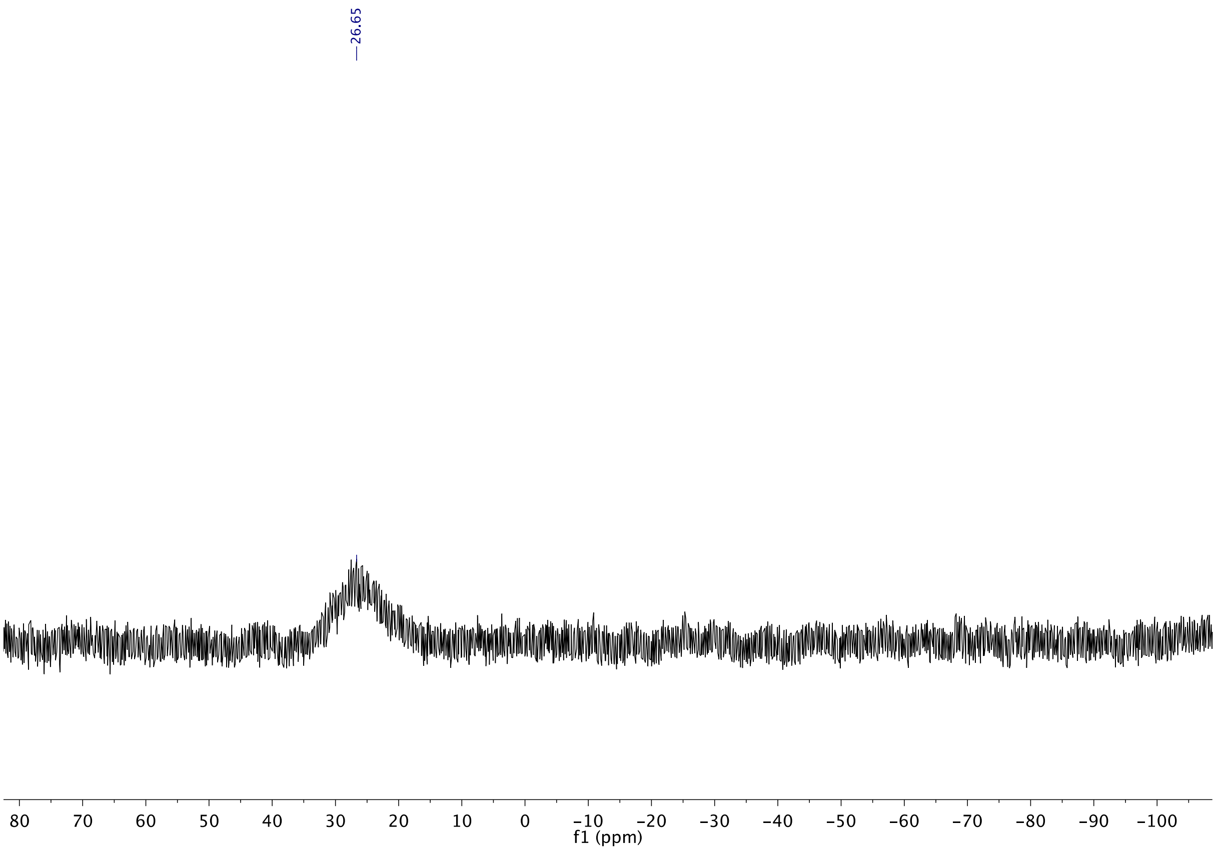

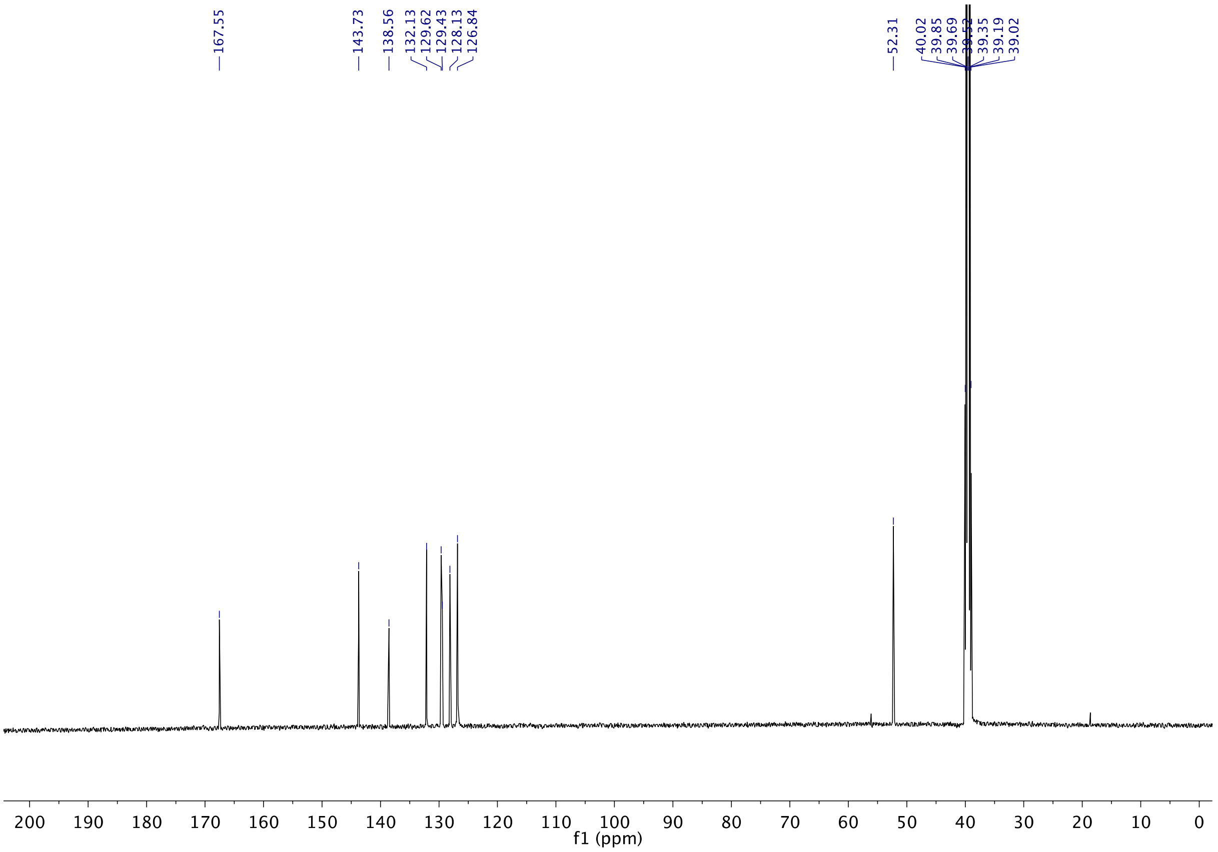

((3'-Methoxy-[1,1'-biphenyl]-2-yl)ethynyl)trimethylsilane, **S14-int1**

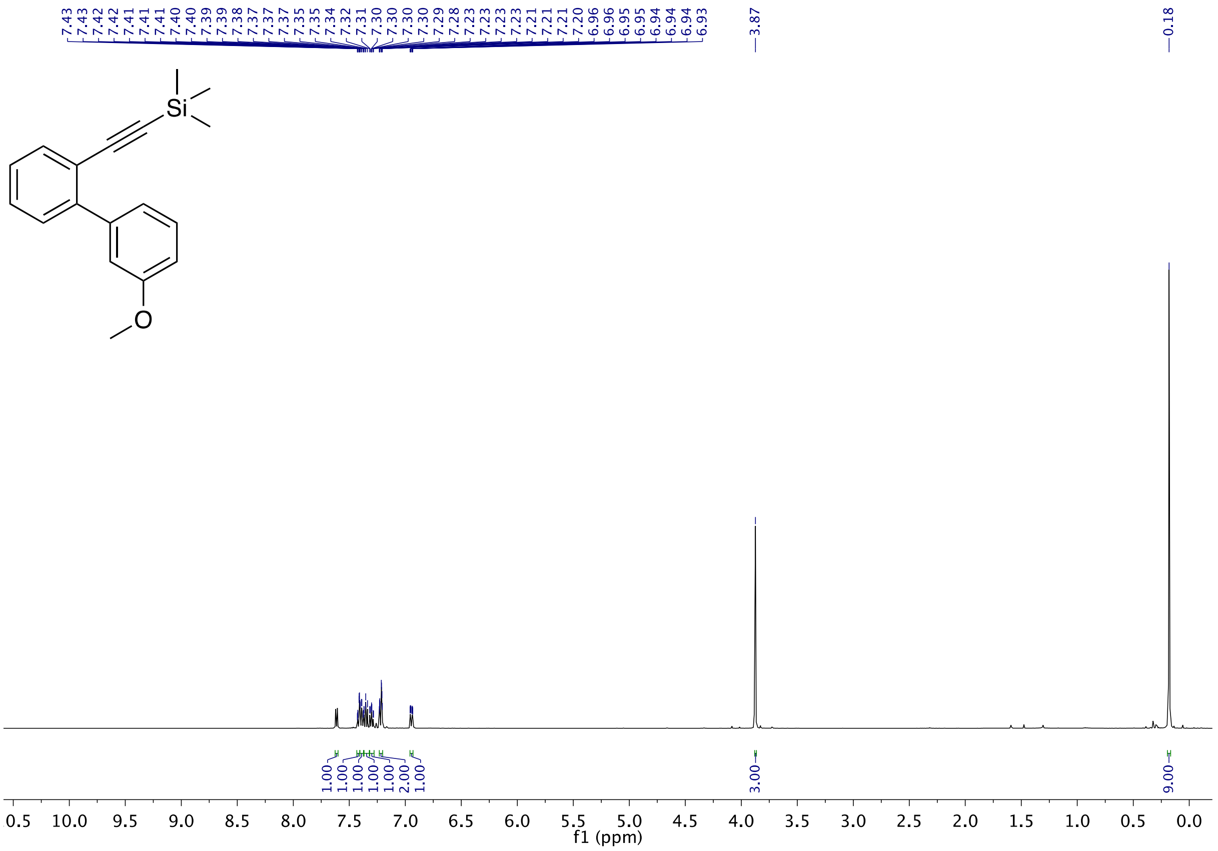

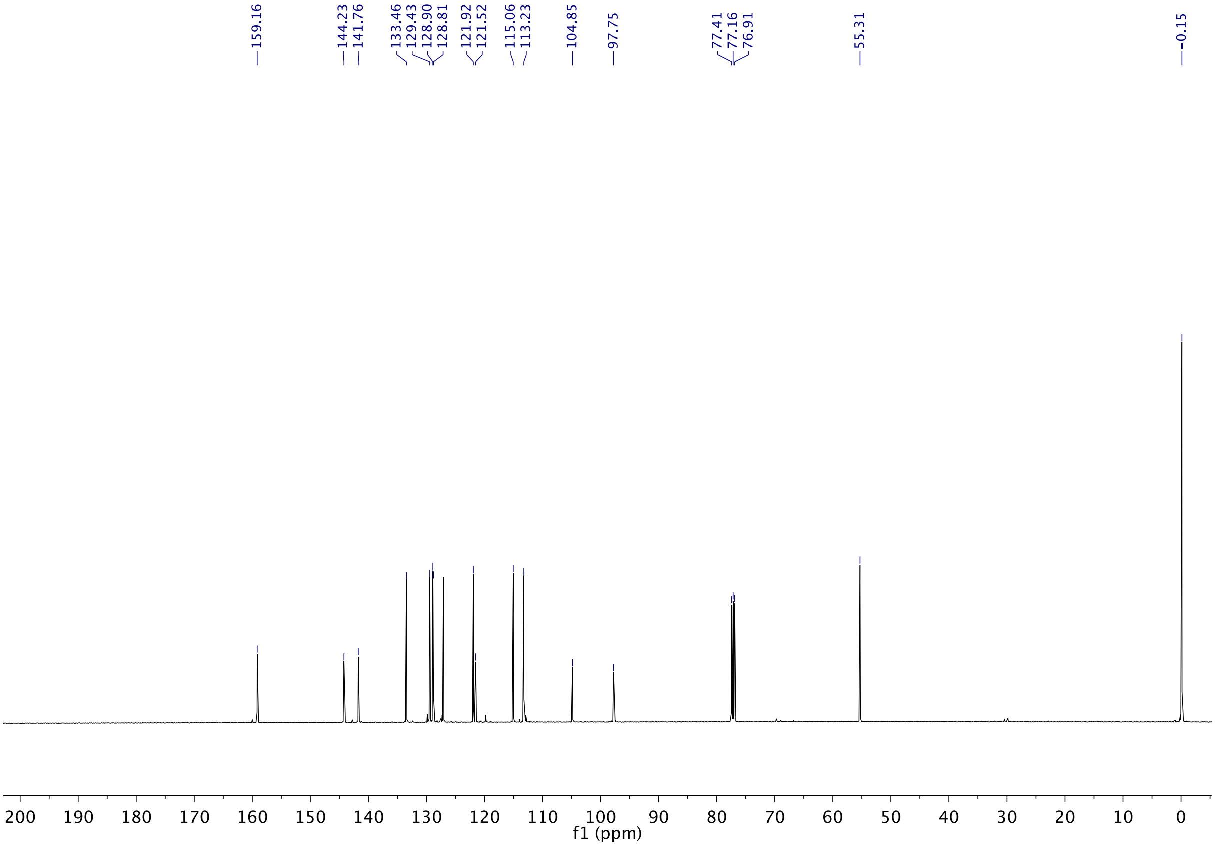

2-Ethynyl-3'-methoxy-1,1'-biphenyl, **S14-int2**

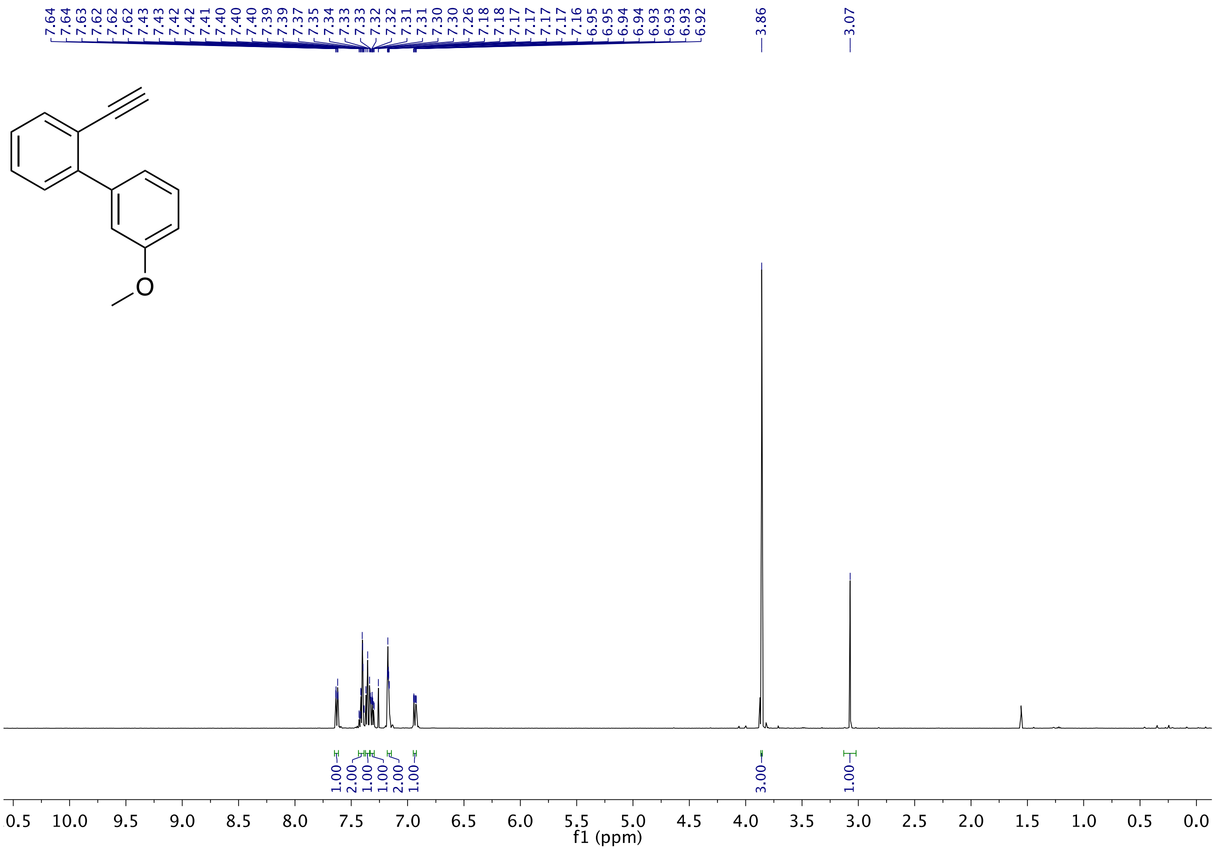

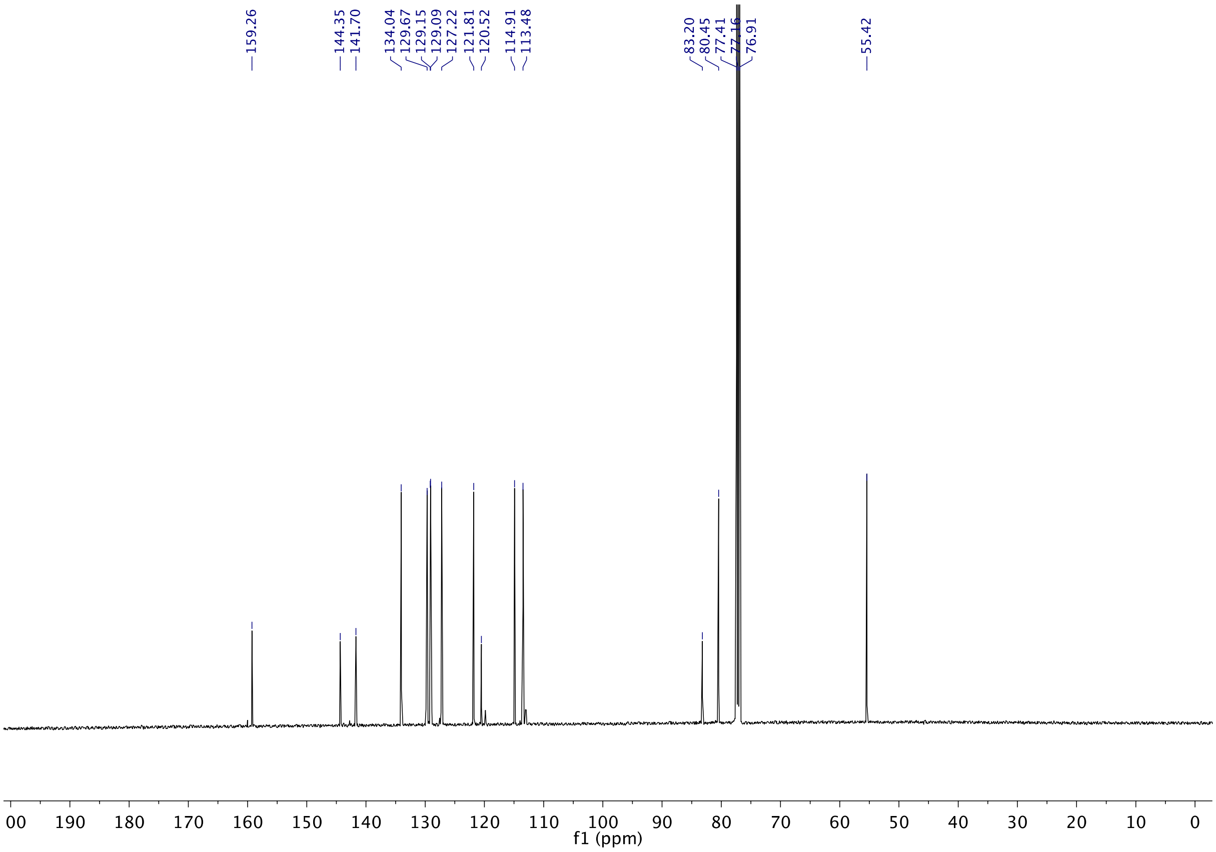

(*E*)-2-(2-(3'-Methoxy-[1,1'-biphenyl]-2-yl)vinyl)-4,4,5,5-tetramethyl-1,3,2-dioxaborolane, **S14-int3**

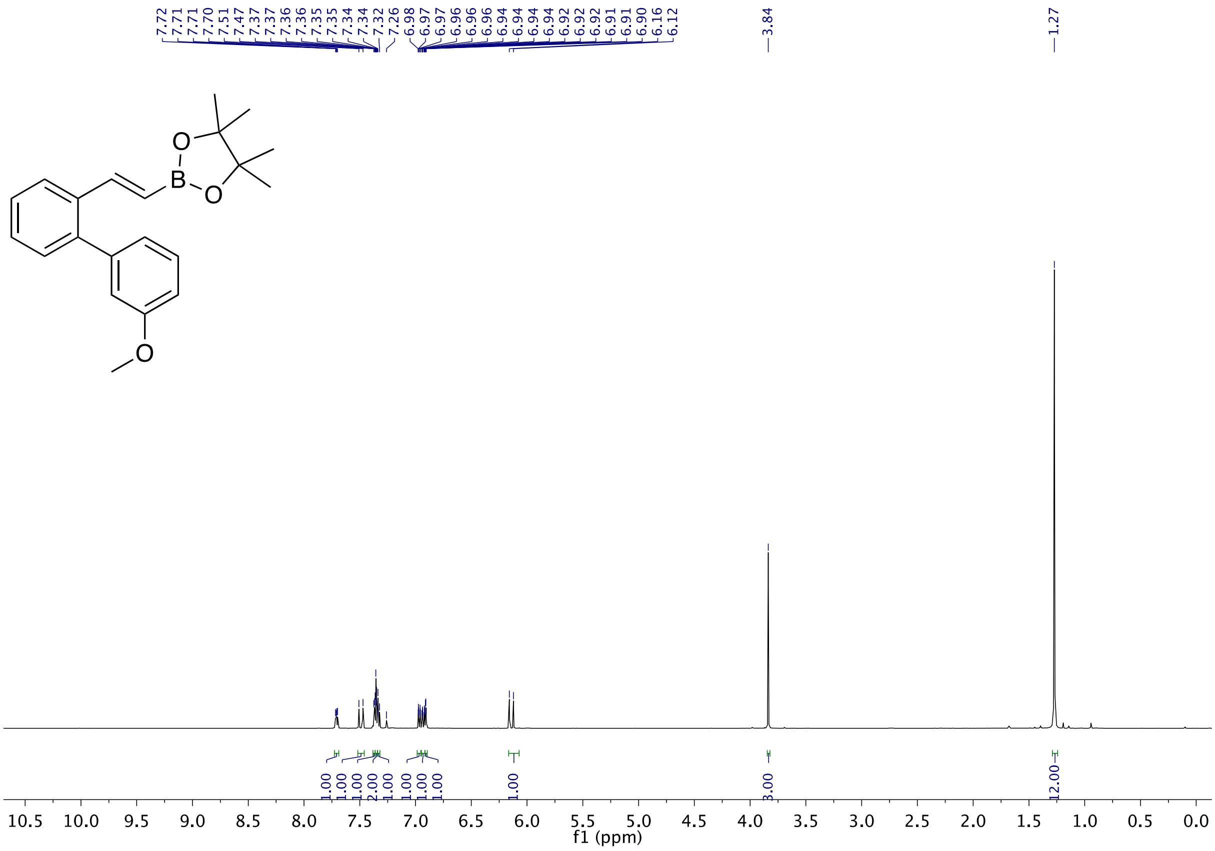

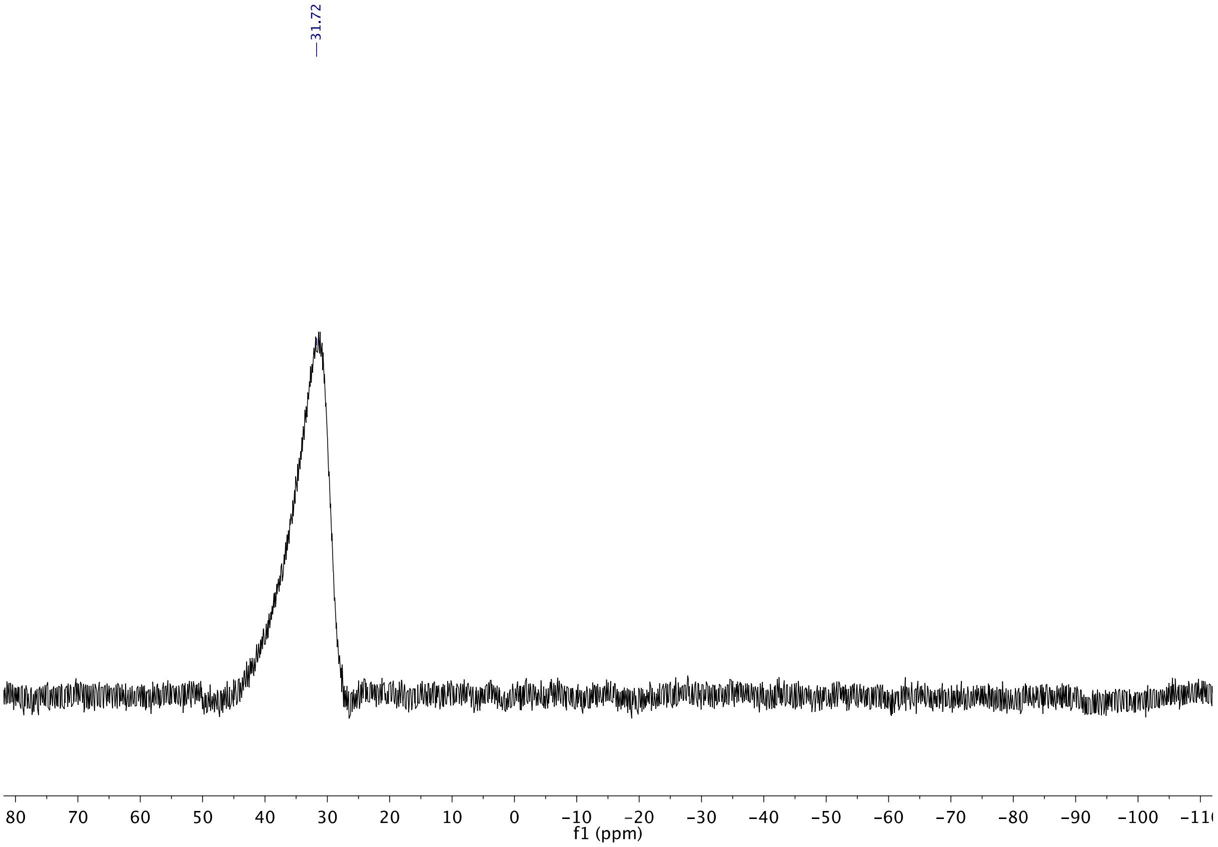

31.72
80
70
60
50
40
30
20
10
0
−10
−20
−30
−40
−50
−60
−70
−80
−90
−100
f1 (ppm)

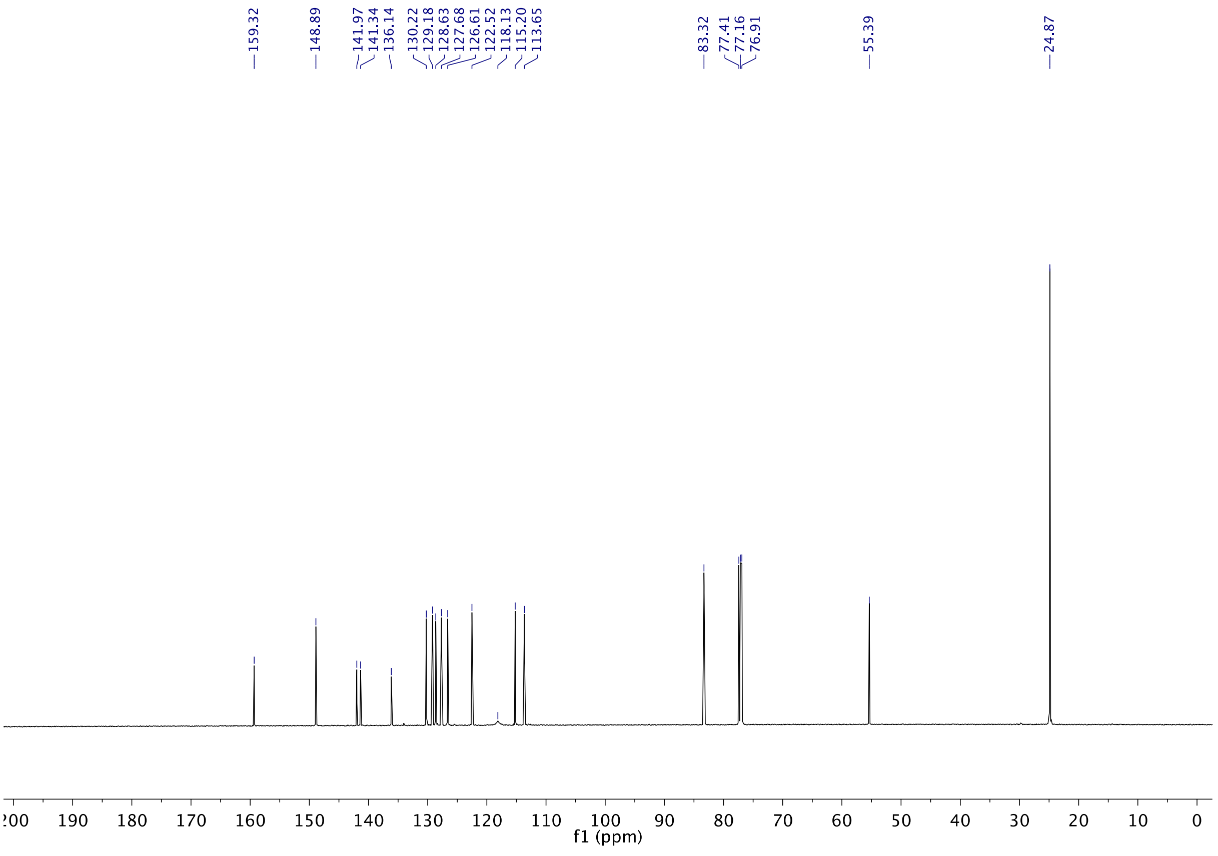

159.32
148.89
141.97
141.34
136.14
130.22
129.18
128.63
127.68
126.61
122.52
118.13
115.20
113.65
83.32
77.41
77.16
76.91
55.39
24.87
190
180
170
160
150
140
130
120
110
100
90
80
70
60
50
40
30
20
10
0
f1 (ppm)

(*E*)-trifluoro(2-(3'-methoxy-[1,1'-biphenyl]-2-yl)vinyl)-λ[4]-borane, potassium salt, **S14-int4**

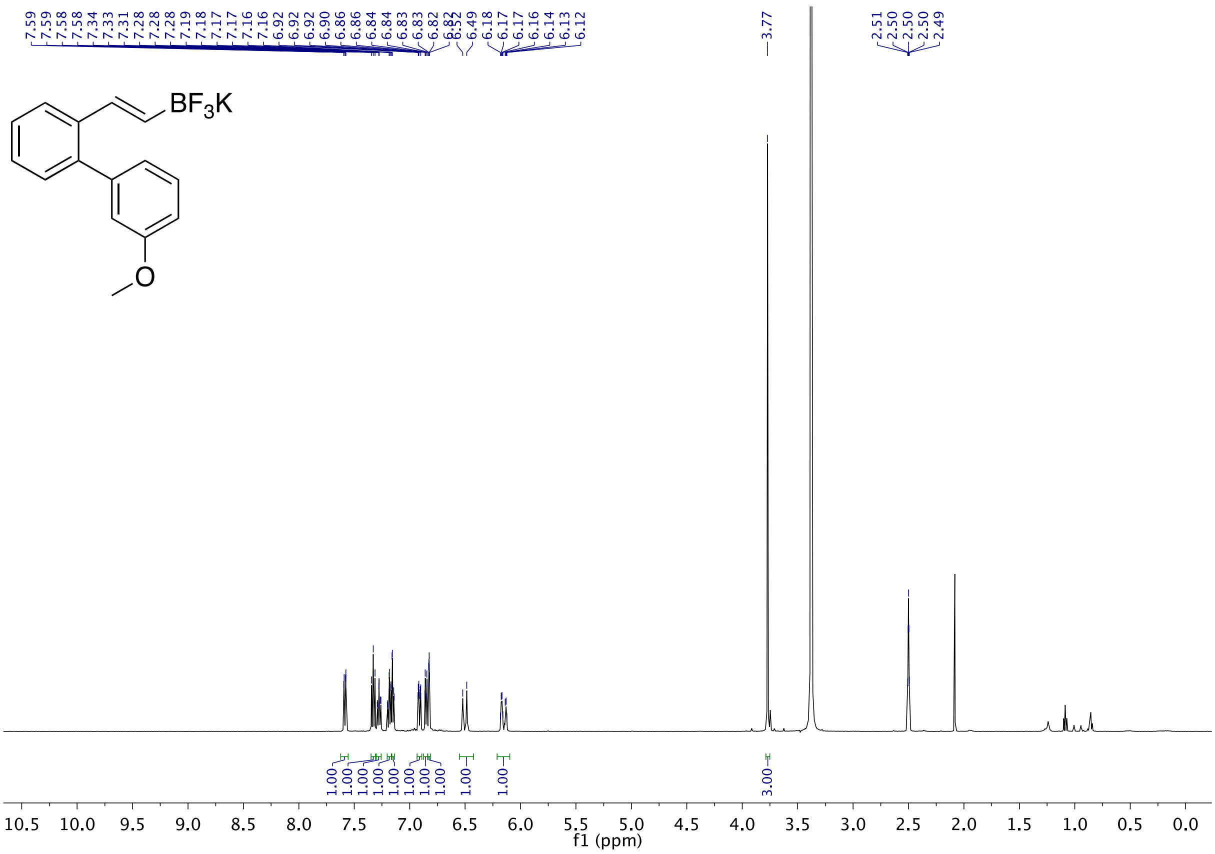

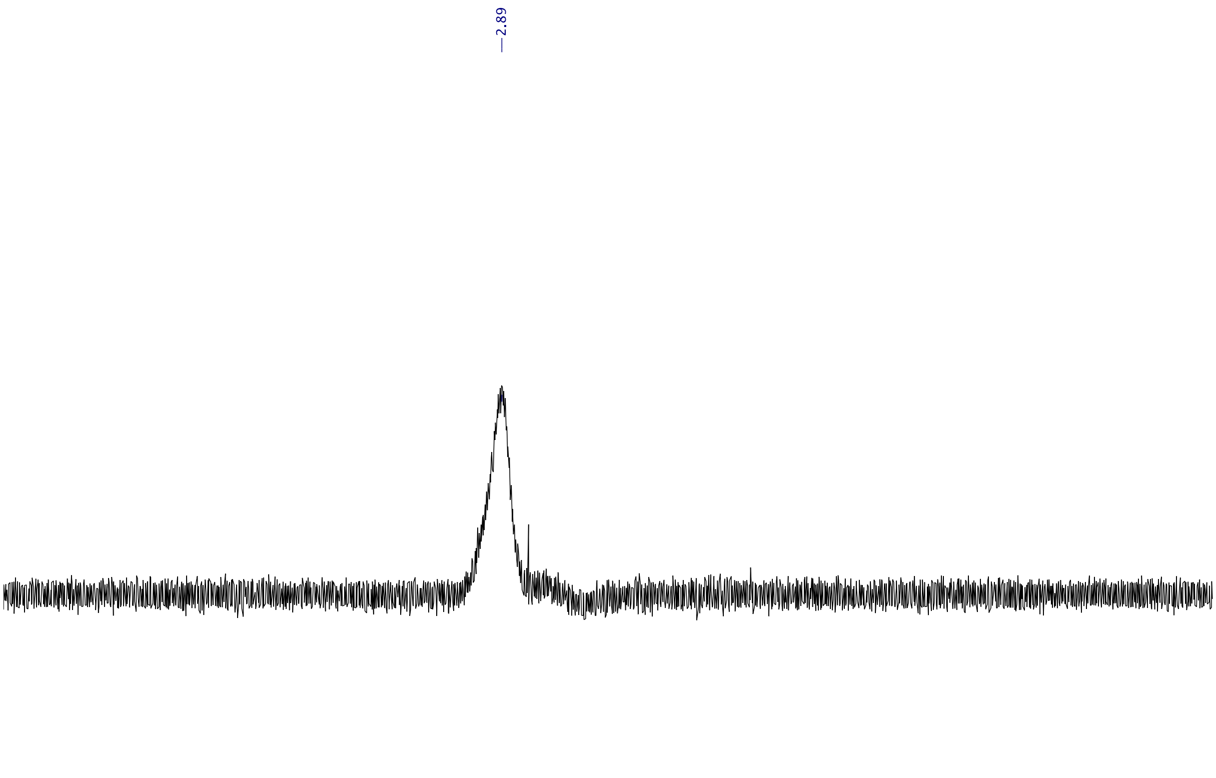

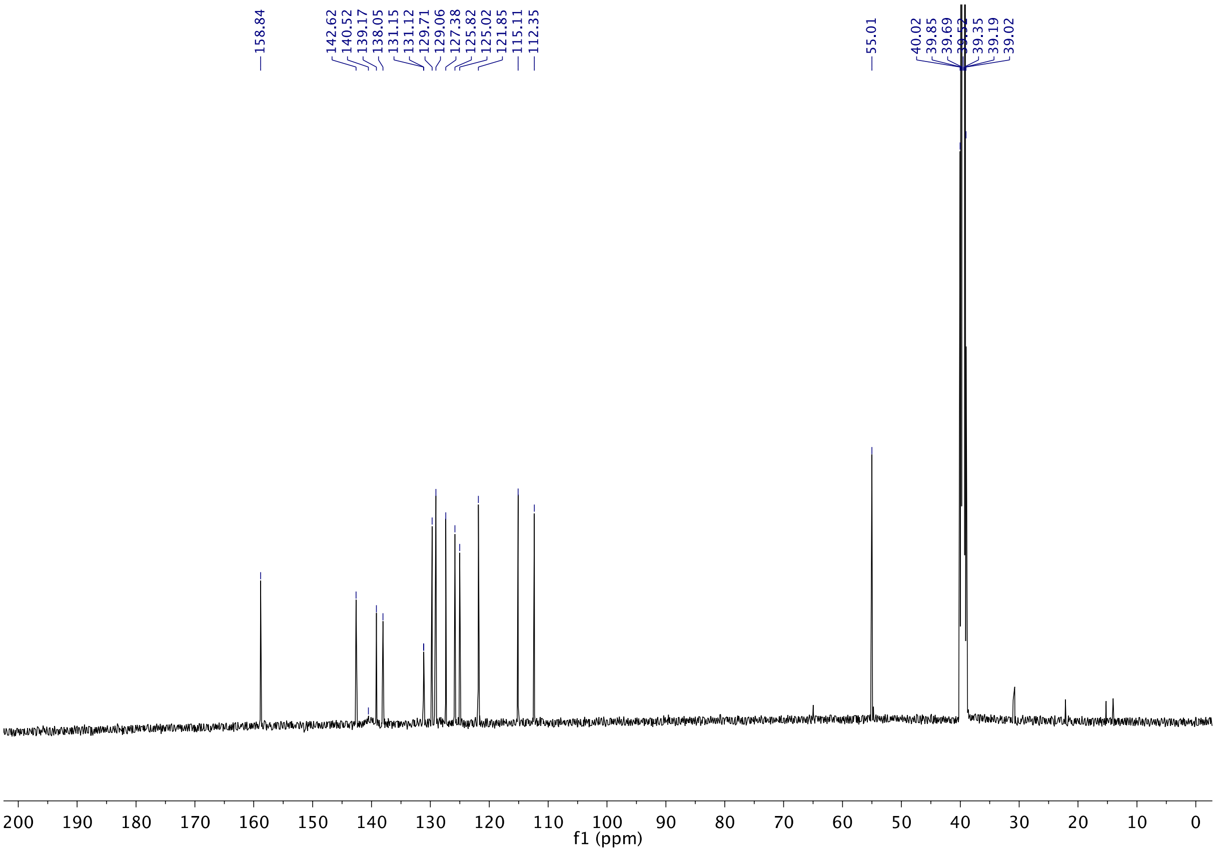
158.84
142.62
140.52
139.17
138.05
131.15
131.12
129.71
129.06
127.38
125.82
125.02
121.85
115.11
112.35
55.01
40.02
39.85
39.69
39.52
39.35
39.19
39.02
200 190 180 170 160 150 140 130 120 110 100 90 80 70 60 50 40 30 20 10 0
f1 (ppm)

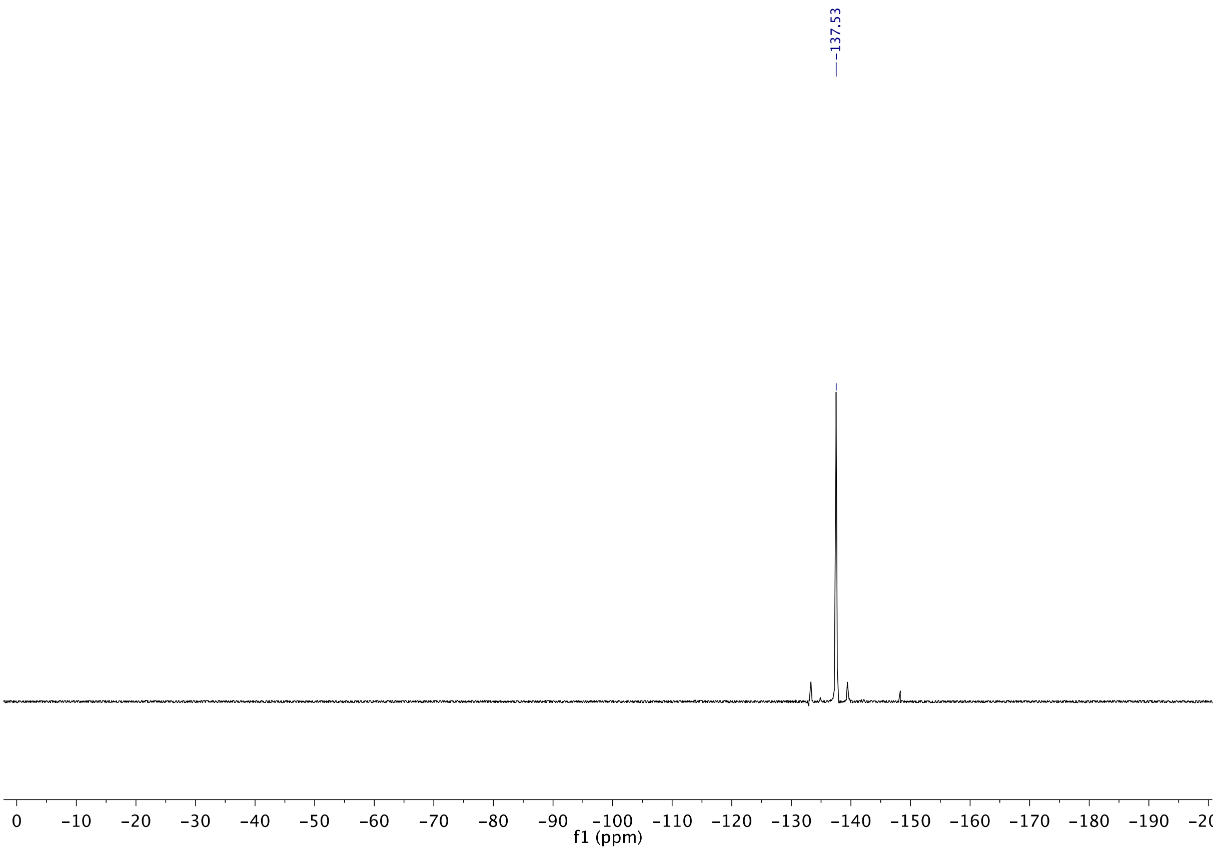
-137.53
0 −10 −20 −30 −40 −50 −60 −70 −80 −90 −100 −110 −120 −130 −140 −150 −160 −170 −180 −190
f1 (ppm)

(*E*)-(2-(3'-Methoxy-[1,1'-biphenyl]-2-yl)vinyl)boronic acid, **S14**

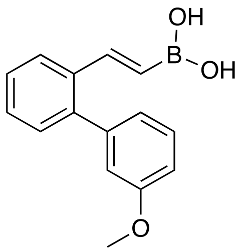

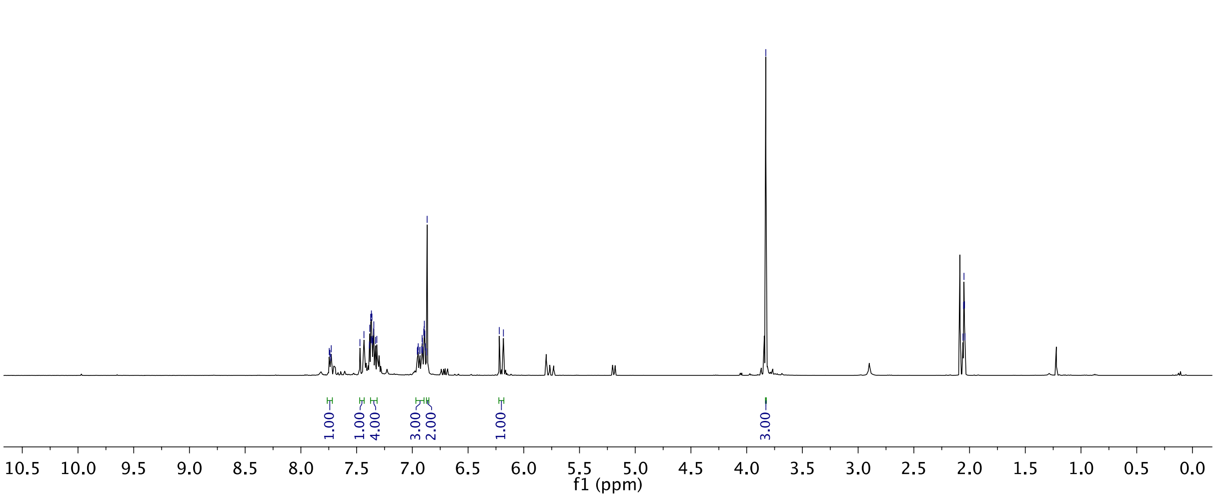

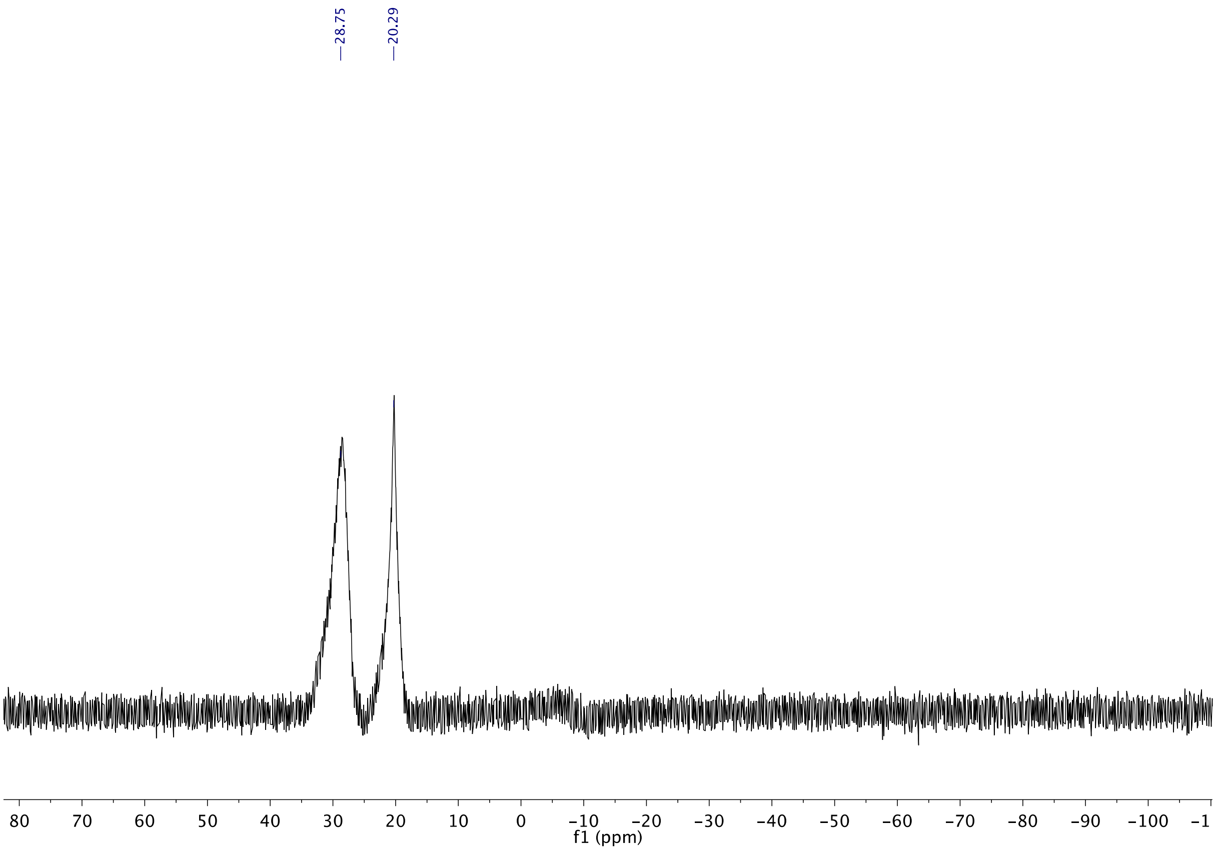

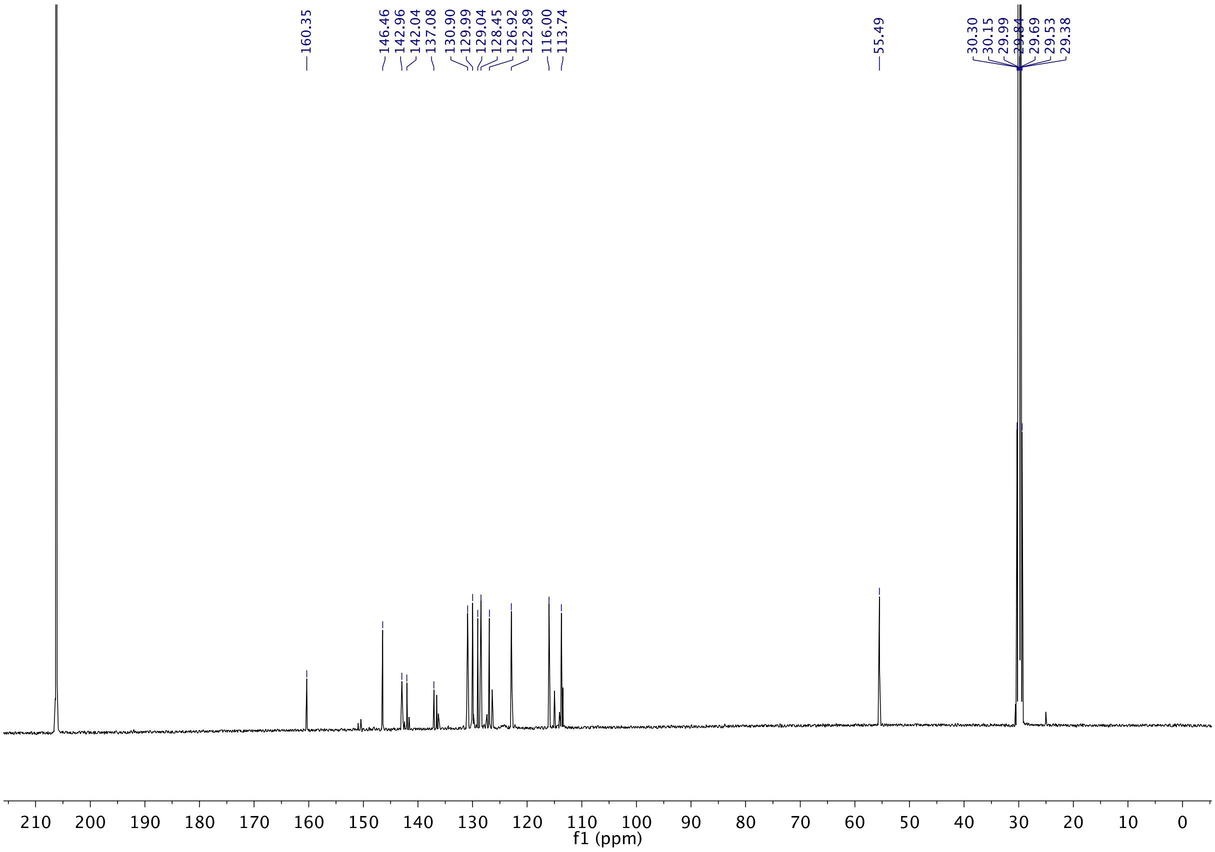

(*E*)-4,4,5,5-Tetramethyl-2-(2-phenylprop-1-en-1-yl)-1,3,2-dioxaborolane, **65-int3**

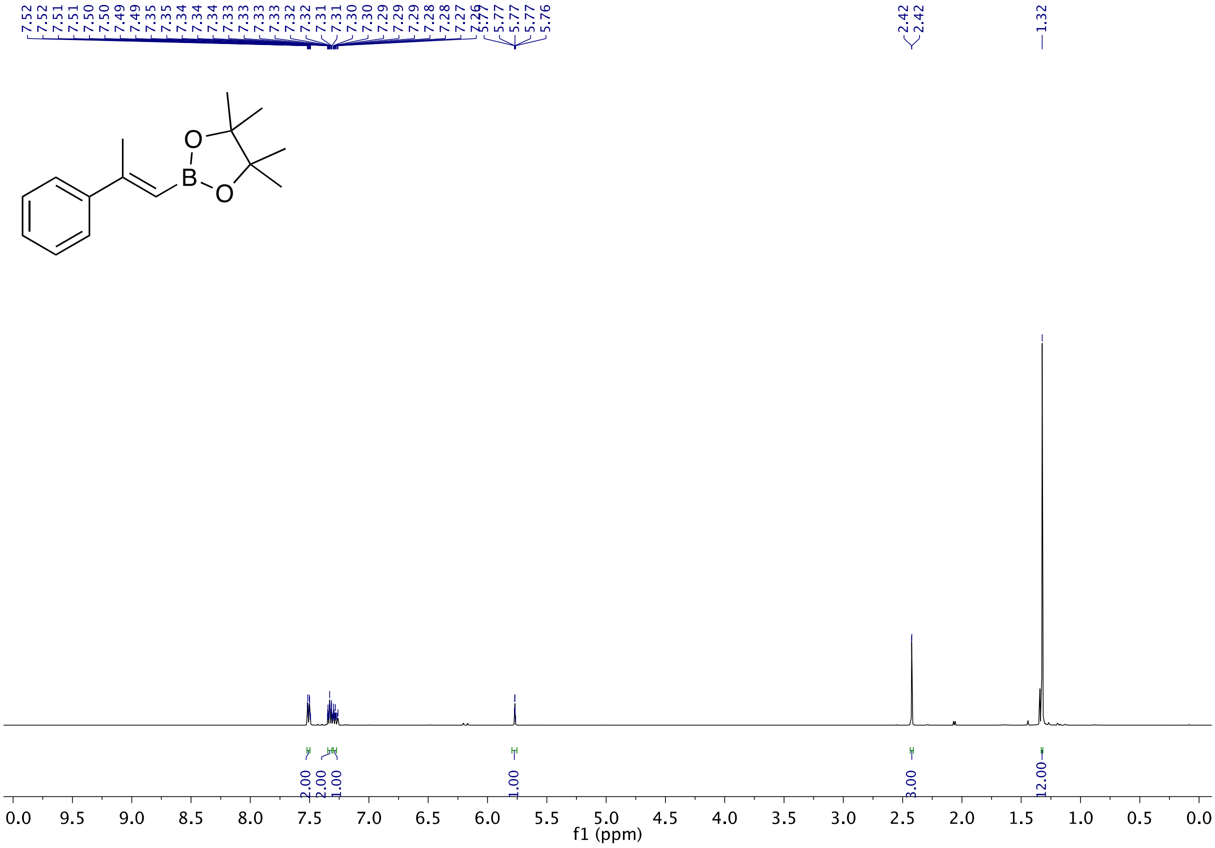

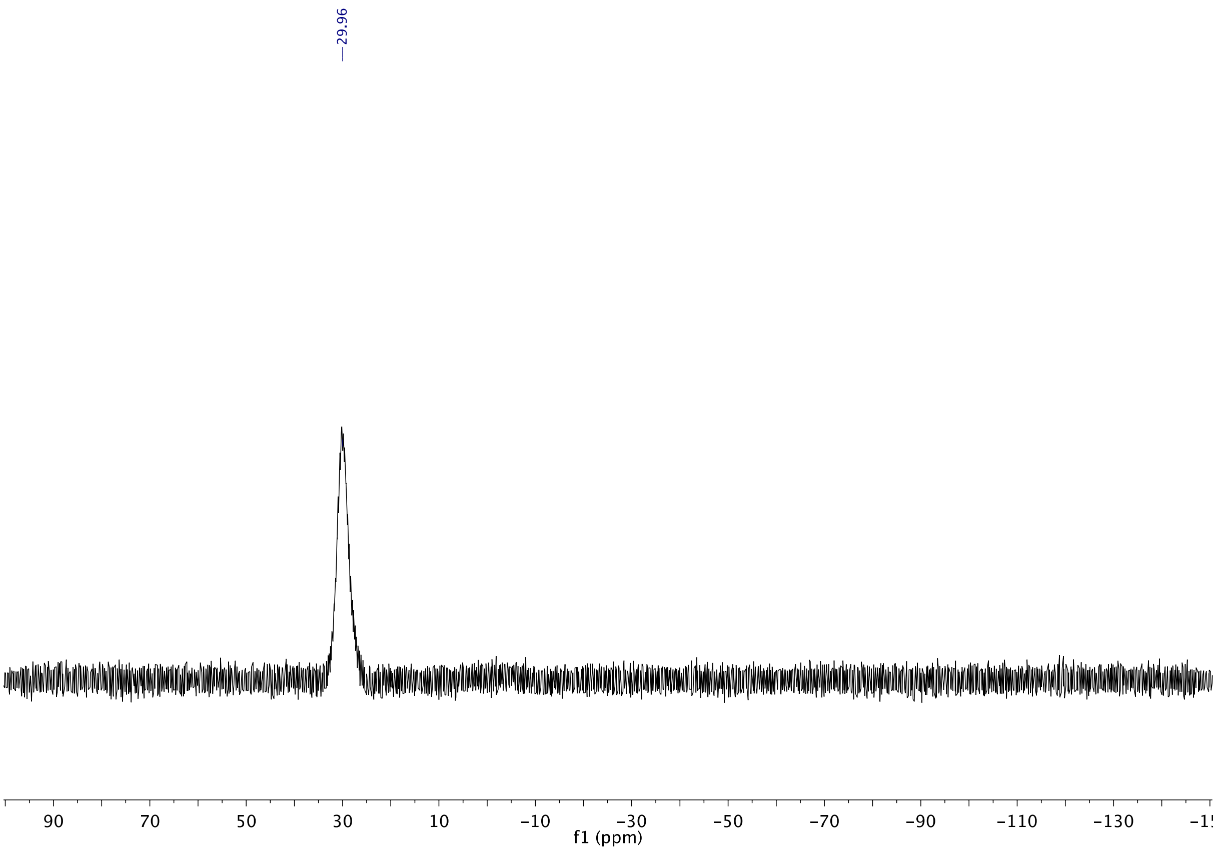
29.96
90 70 50 30 10 −10 −30 −50 −70 −90 −110 −130
f1 (ppm)

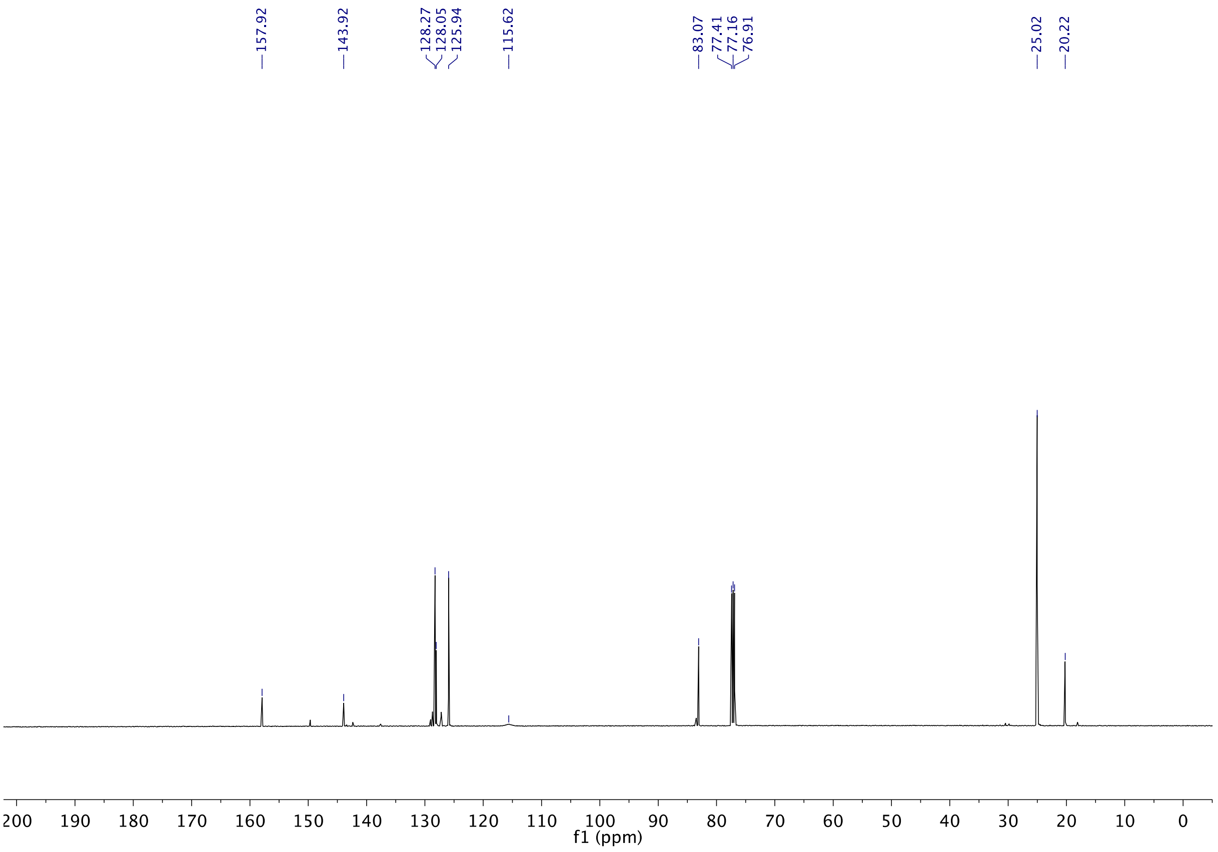
157.92
143.92
128.27
128.05
125.94
115.62
83.07
77.41
77.16
76.91
25.02
20.22
200 190 180 170 160 150 140 130 120 110 100 90 80 70 60 50 40 30 20 10 0
f1 (ppm)

(*E*)-trifluoro(2-phenylprop-1-en-1-yl)-λ^4-borane, potassium salt, **65-int4**

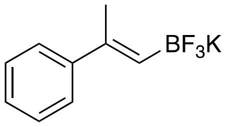

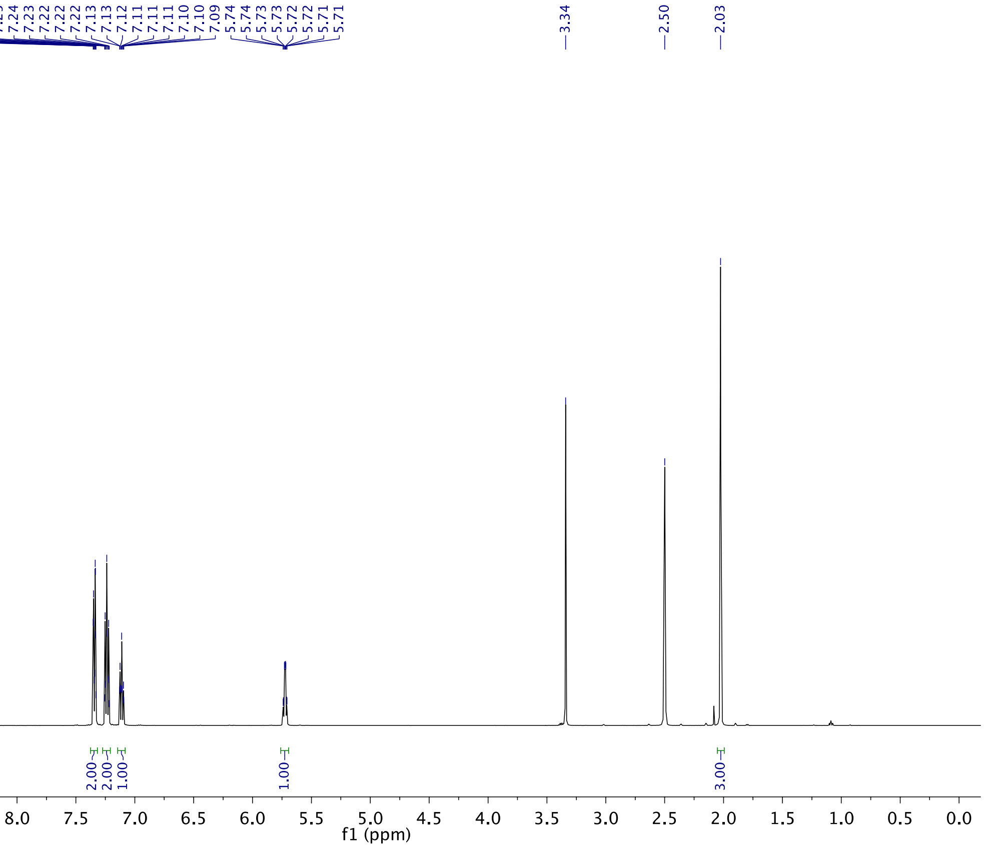

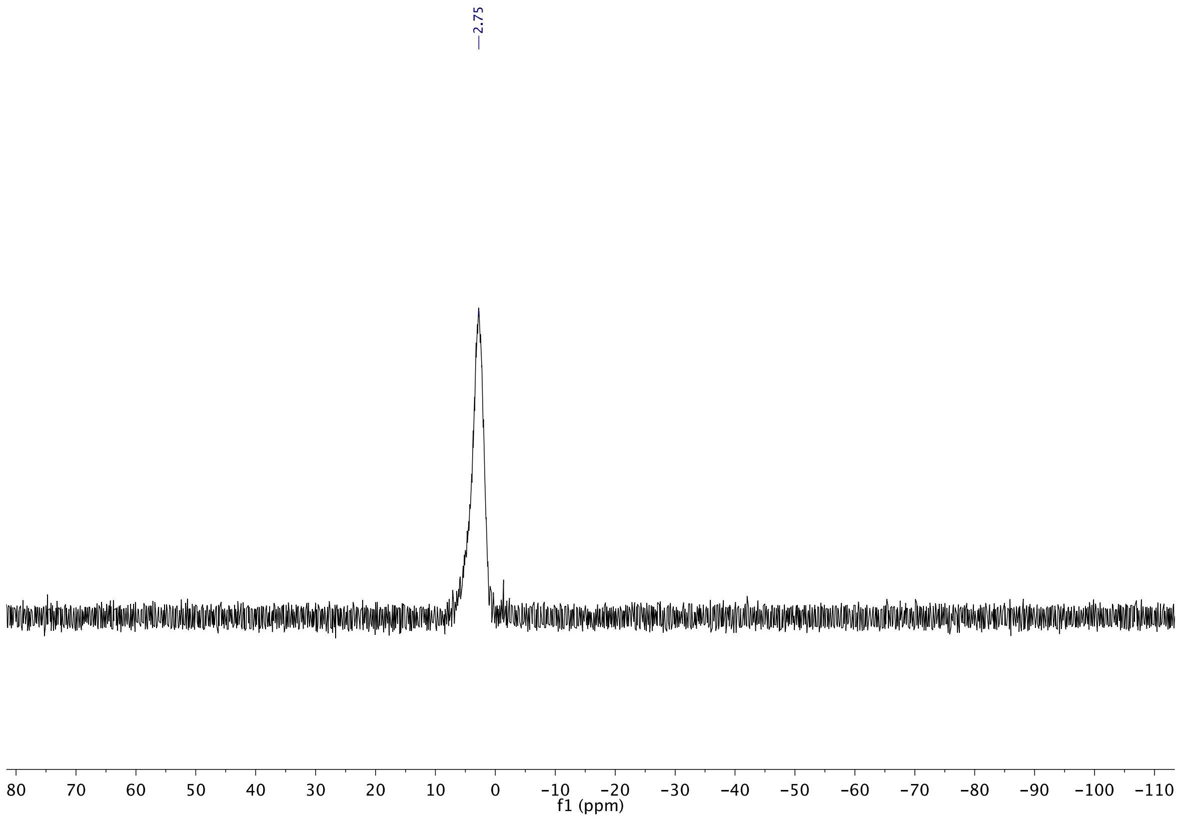

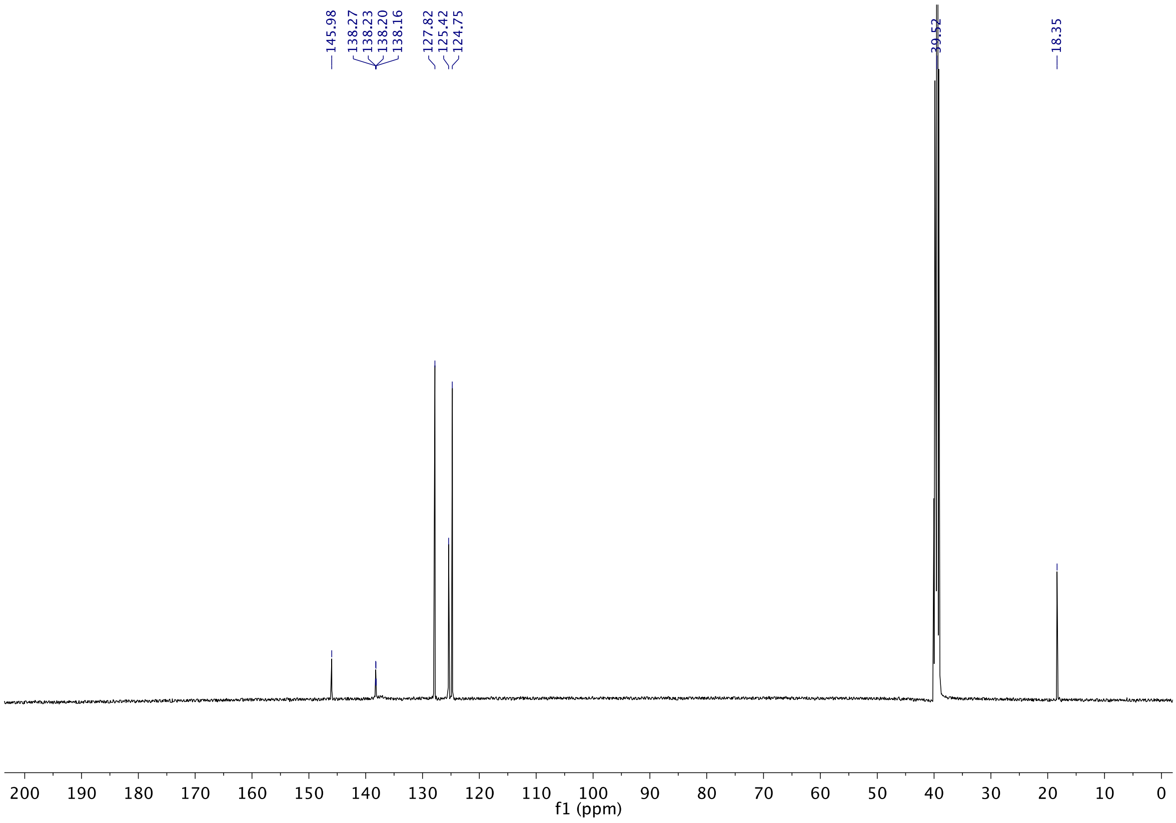
145.98
138.27
138.23
138.20
138.16
127.82
125.42
124.75
39.52
18.35
200 190 180 170 160 150 140 130 120 110 100 90 80 70 60 50 40 30 20 10 0
f1 (ppm)

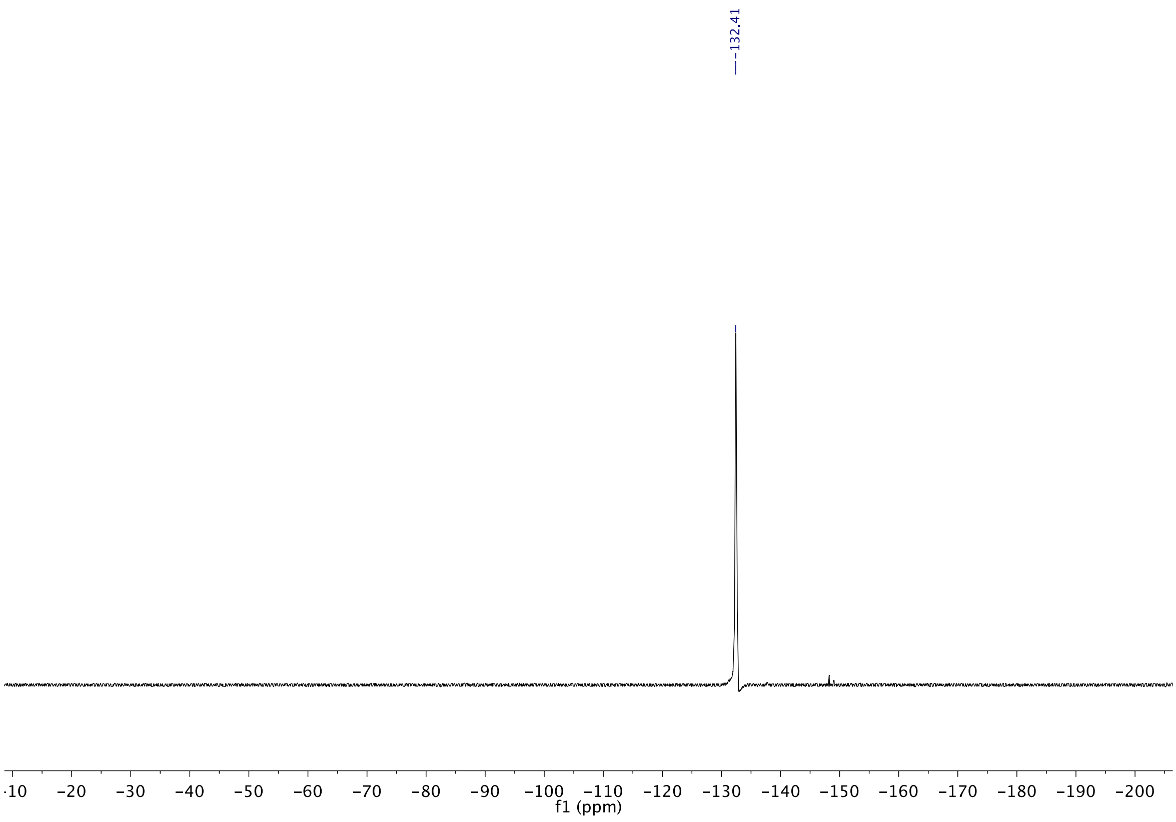
-132.41
-10 −20 −30 −40 −50 −60 −70 −80 −90 −100 −110 −120 −130 −140 −150 −160 −170 −180 −190 −200
f1 (ppm)

(*E*)-(2-Phenylprop-1-en-1-yl)boronic acid, **65**

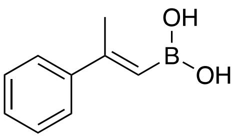

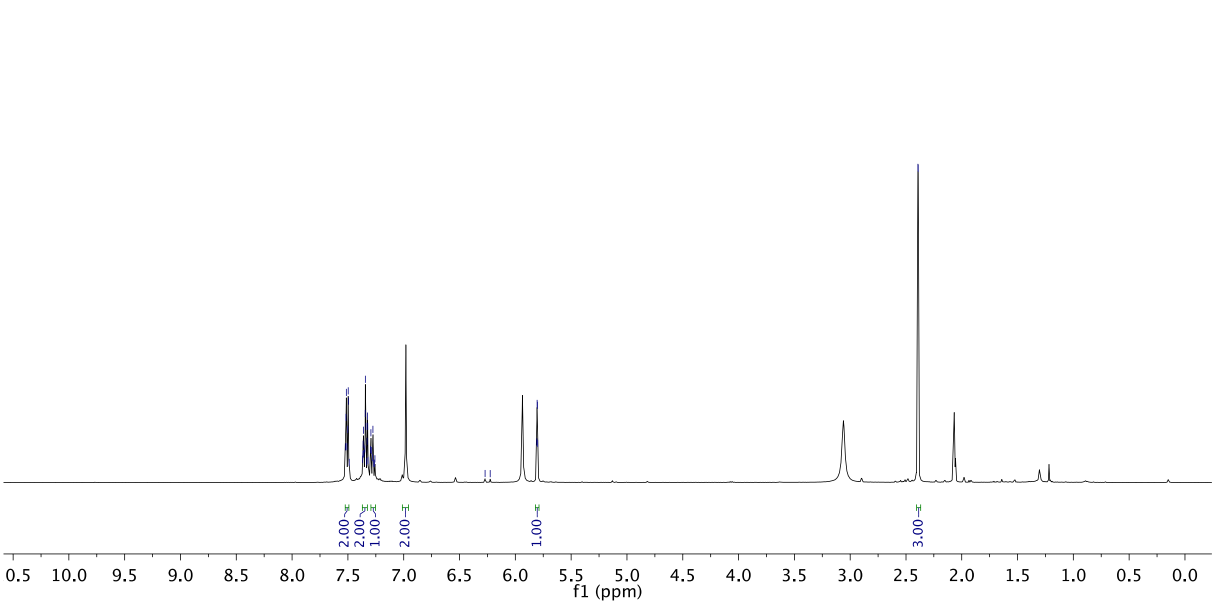

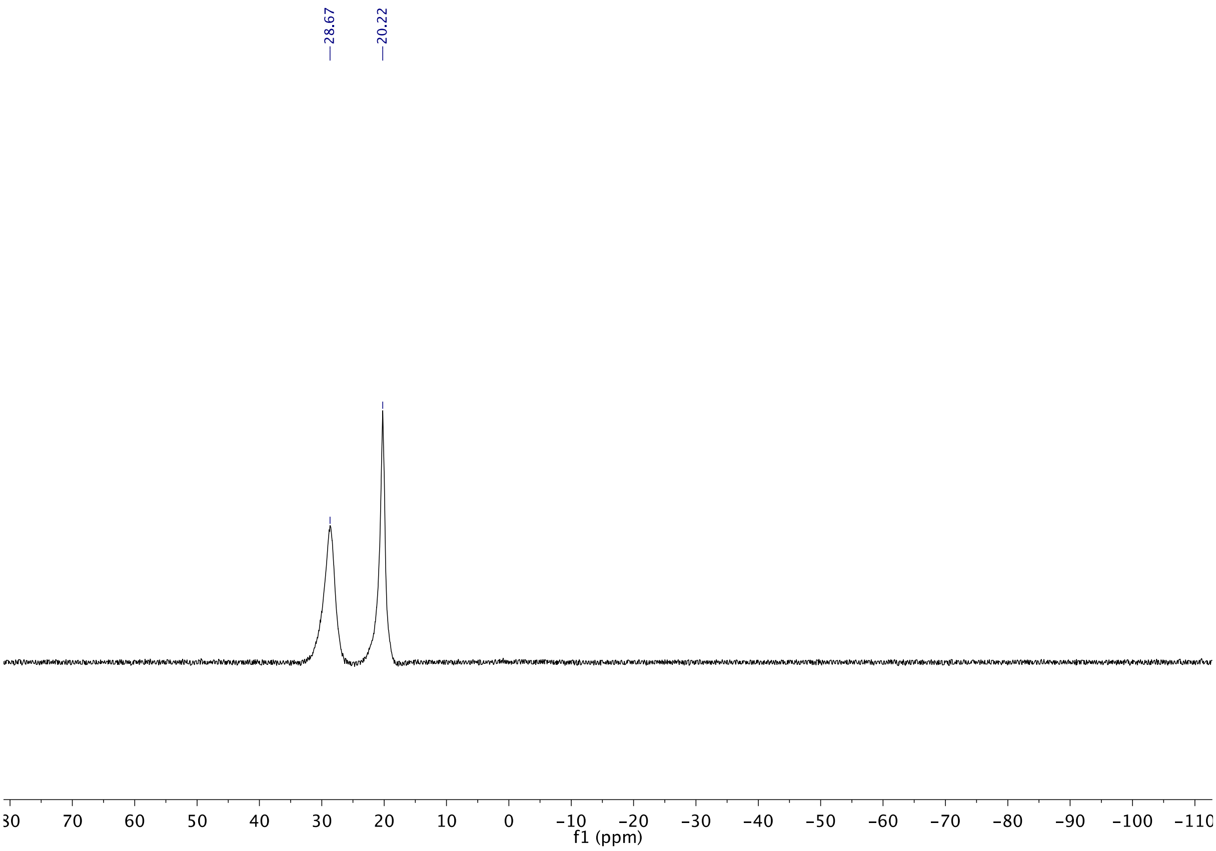

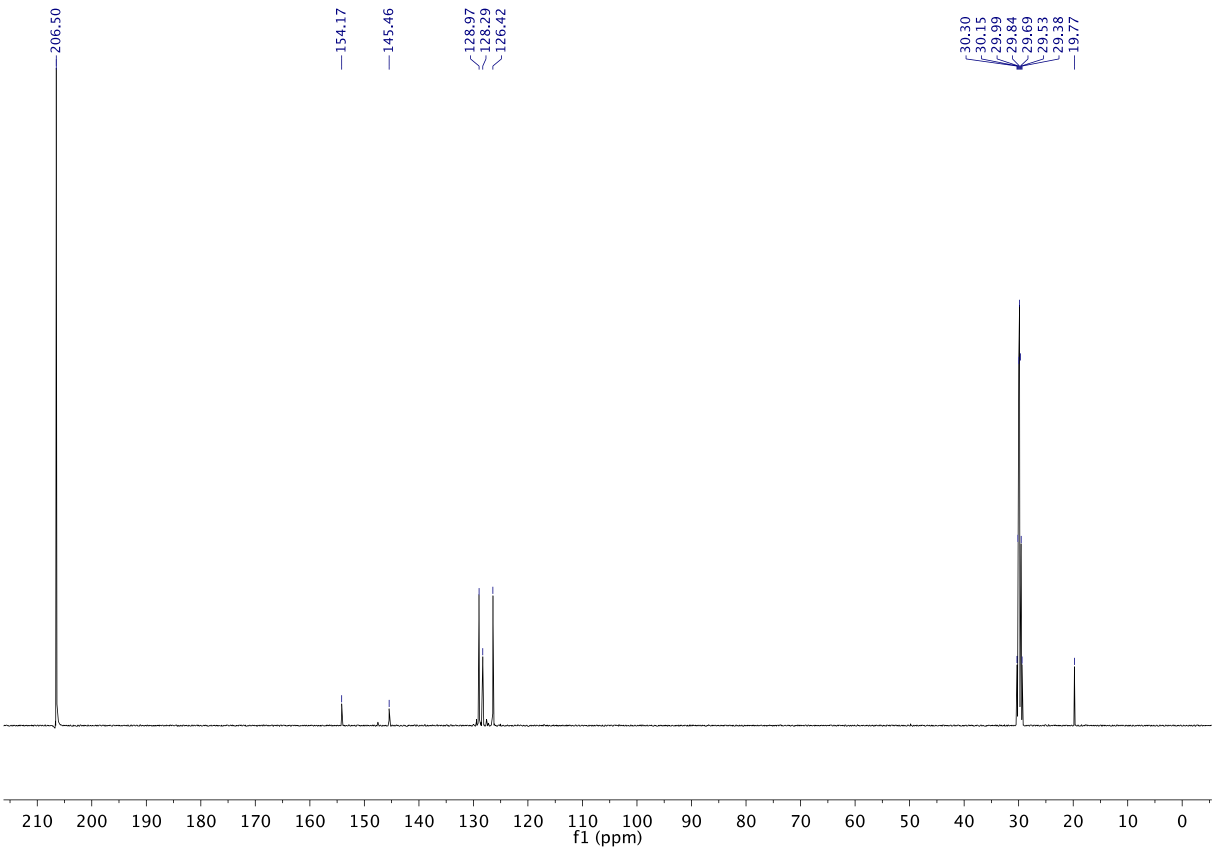

(*Z*)-4,4,5,5-Tetramethyl-2-(1-phenylprop-1-en-2-yl)-1,3,2-dioxaborolane, **66-int3**

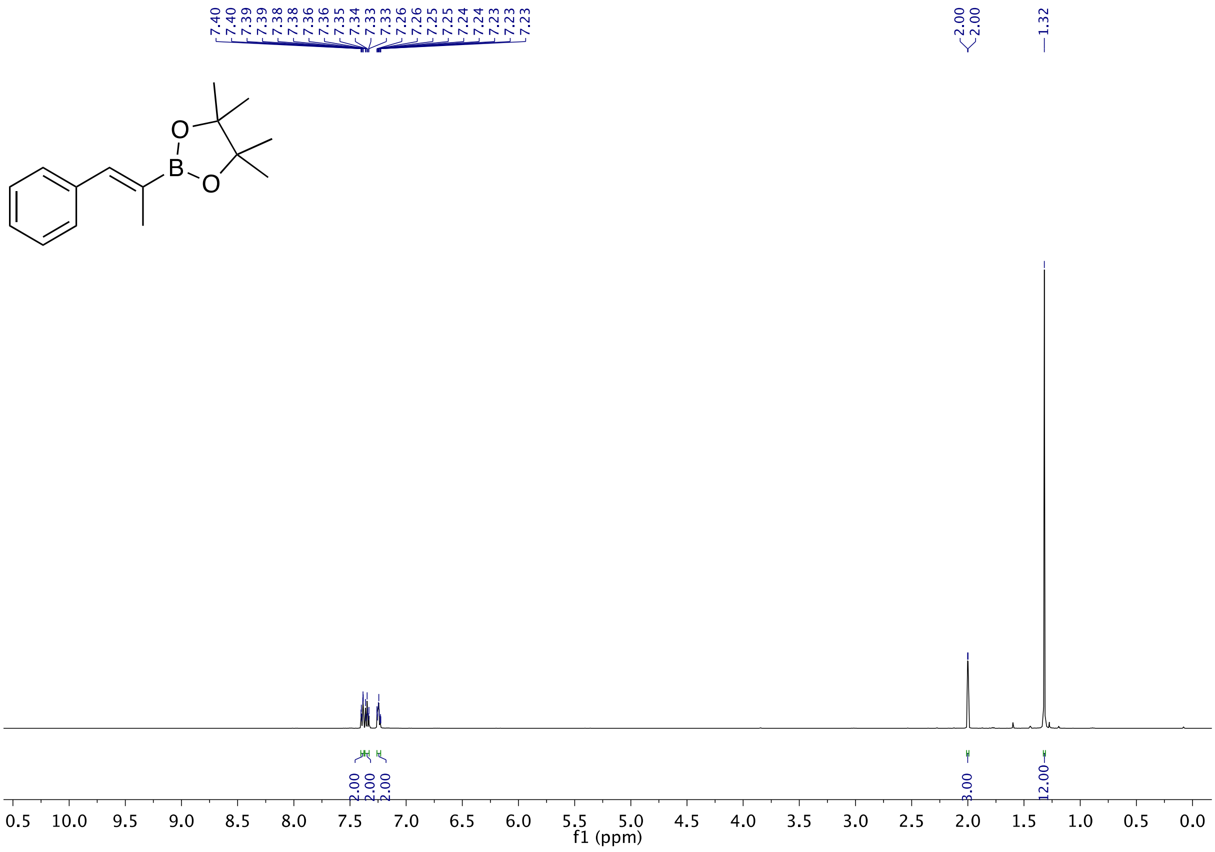

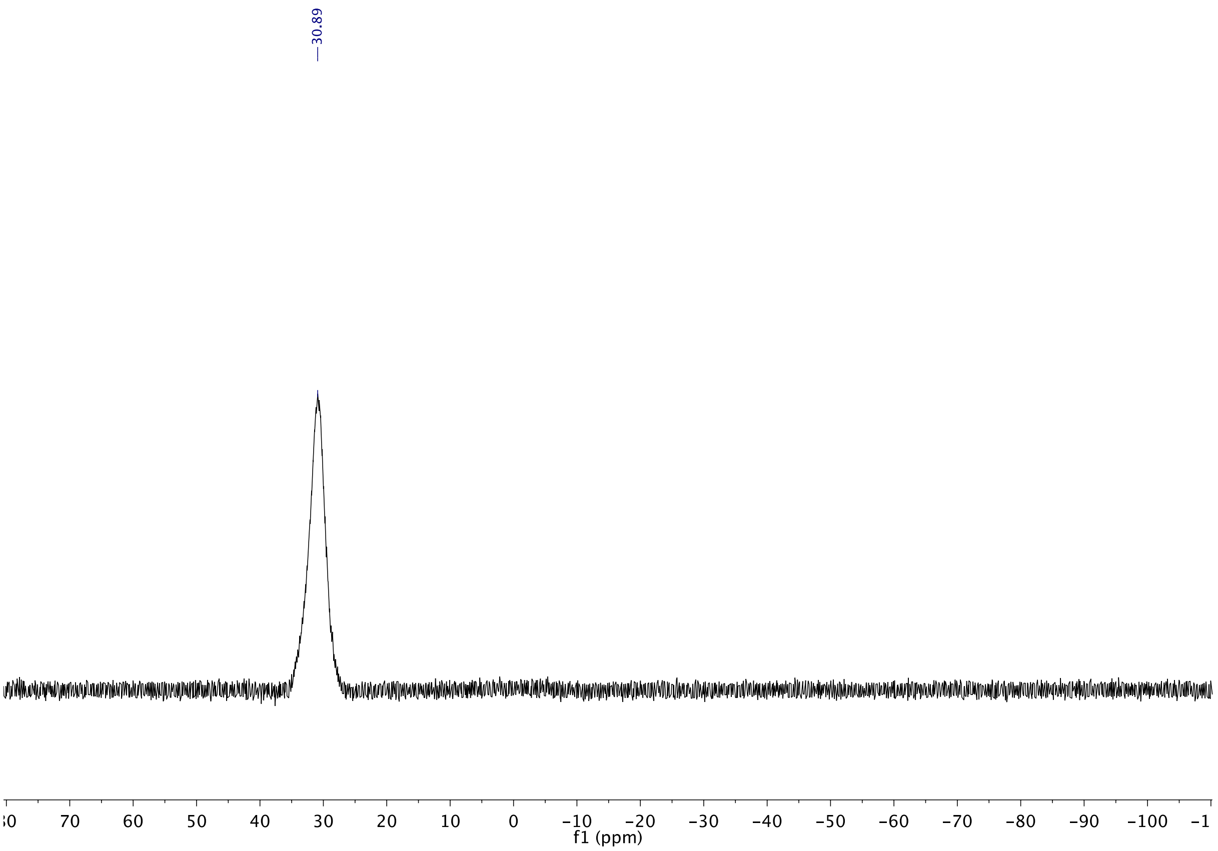
30.89
70
60
50
40
30
20
10
0
−10
−20
−30
−40
−50
−60
−70
−80
−90
−100
f1 (ppm)

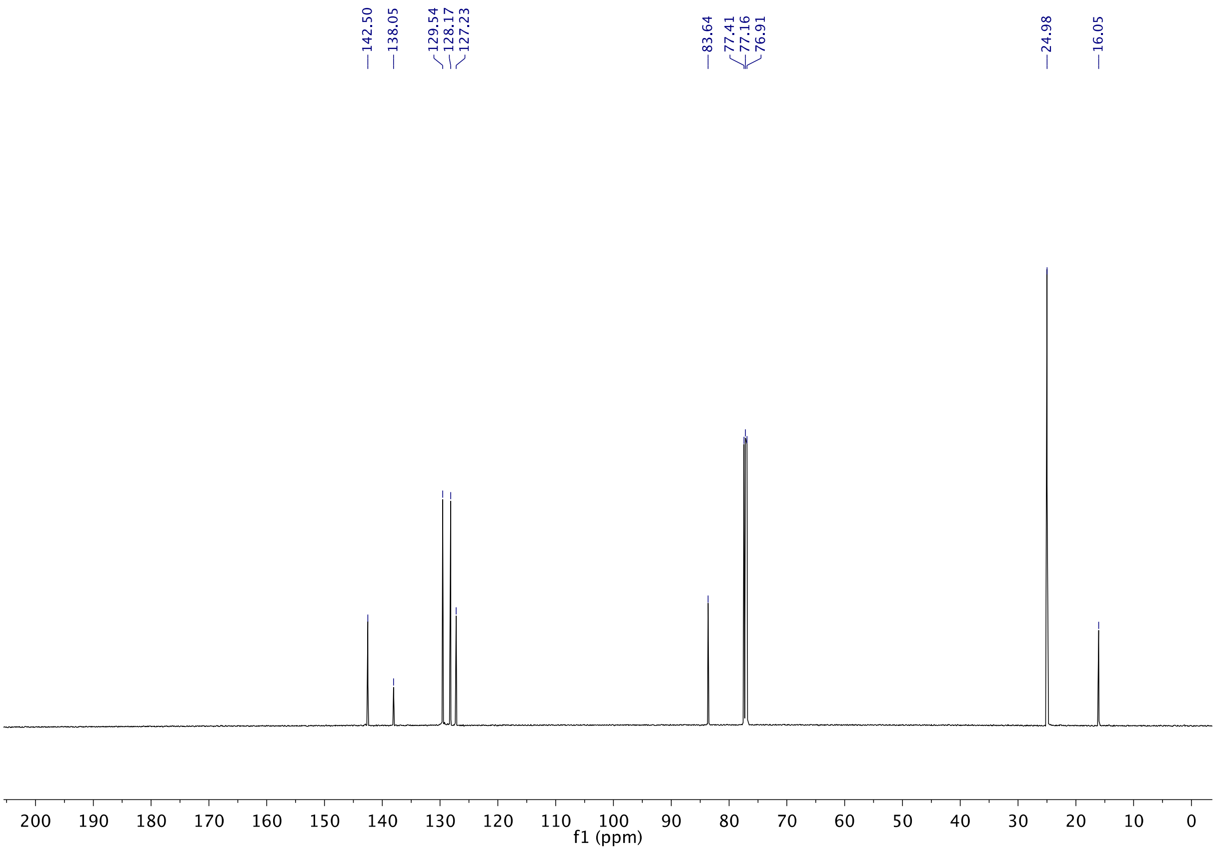
142.50
138.05
129.54
128.17
127.23
83.64
77.41
77.16
76.91
24.98
16.05
200
190
180
170
160
150
140
130
120
110
100
90
80
70
60
50
40
30
20
10
0
f1 (ppm)

(*Z*)-Trifluoro(1-phenylprop-1-en-2-yl)-λ4-borane, potassium salt, **66-int4**

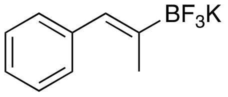

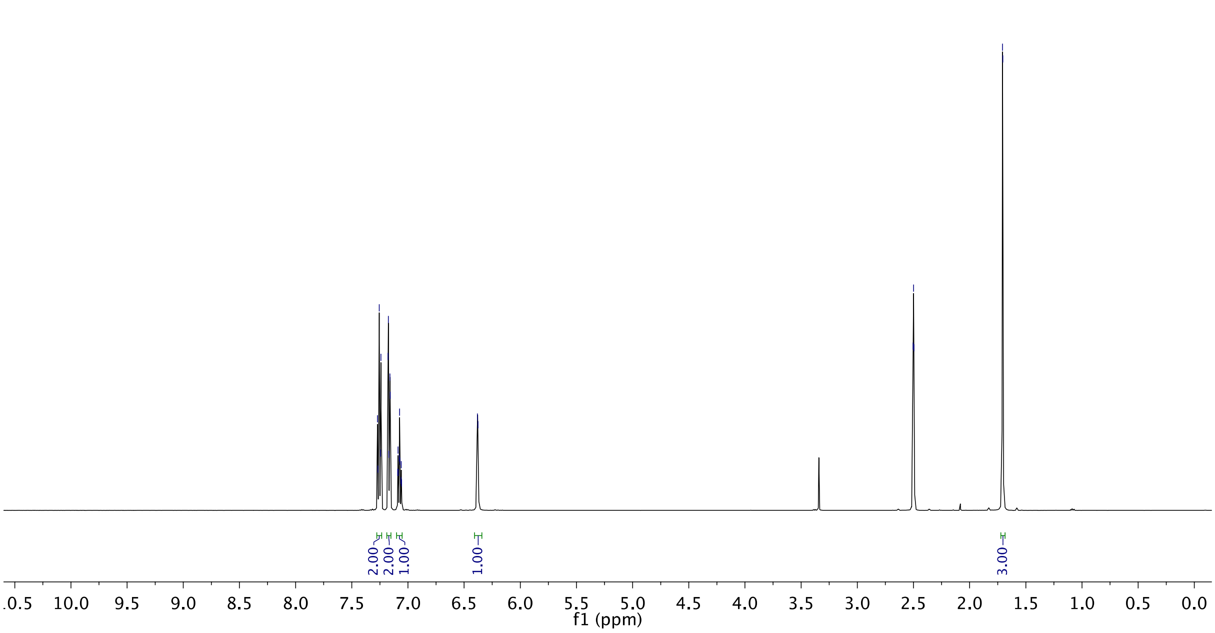

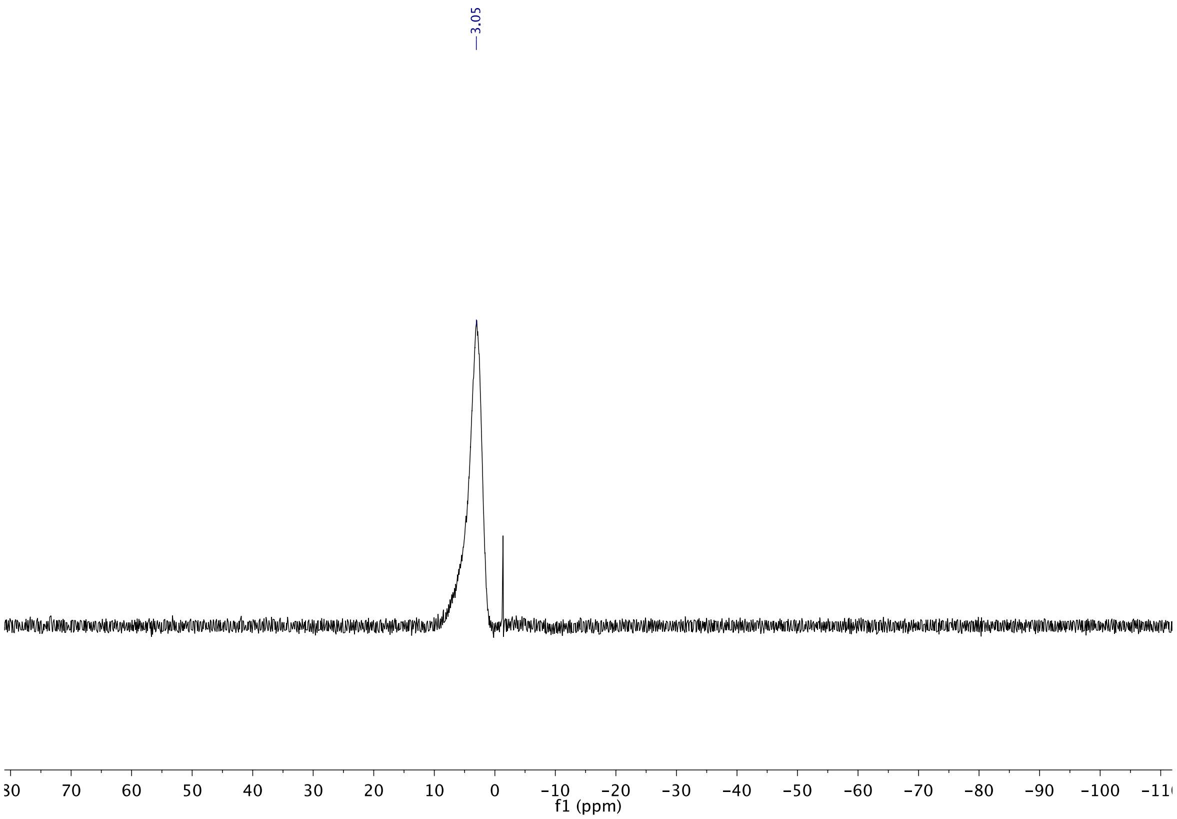

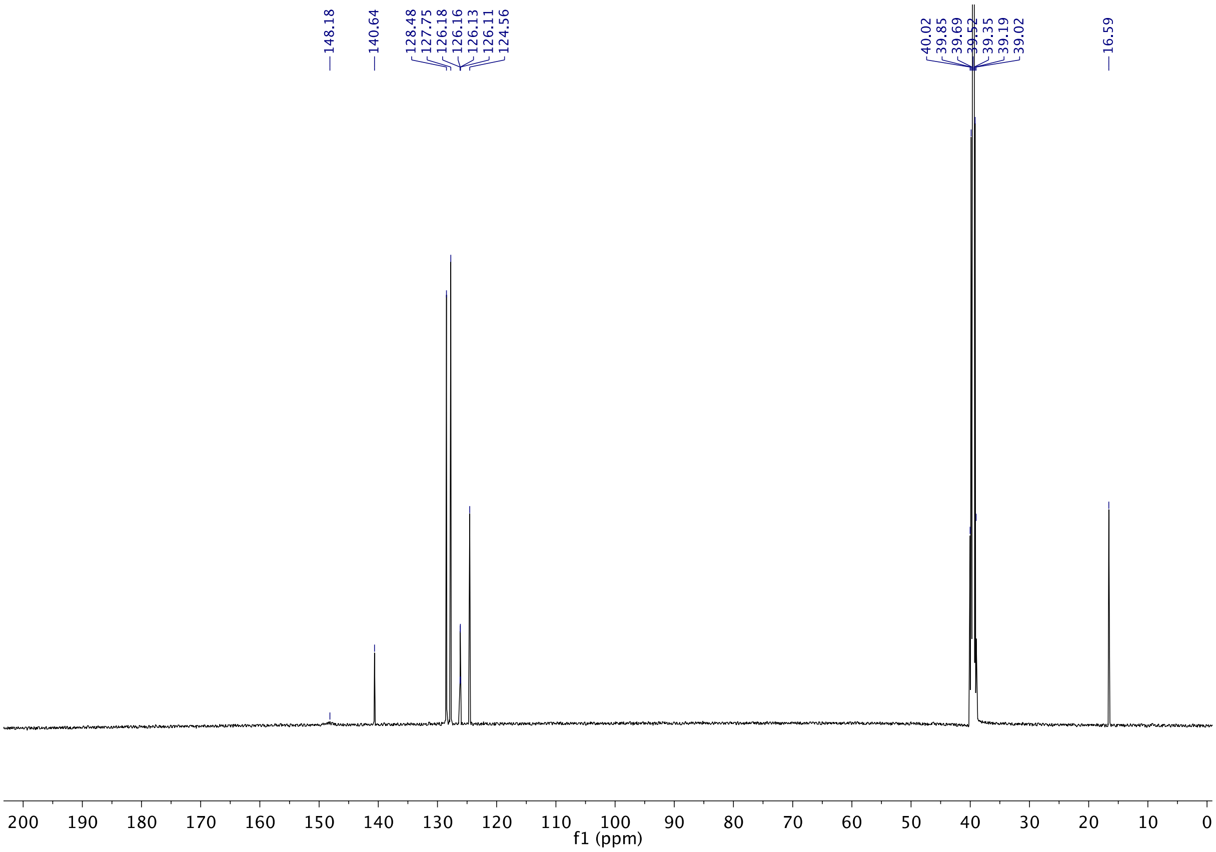
148.18
140.64
128.48
127.75
126.18
126.16
126.13
126.11
124.56
40.02
39.85
39.69
39.52
39.35
39.19
39.02
16.59
200 190 180 170 160 150 140 130 120 110 100 90 80 70 60 50 40 30 20 10 0
f1 (ppm)

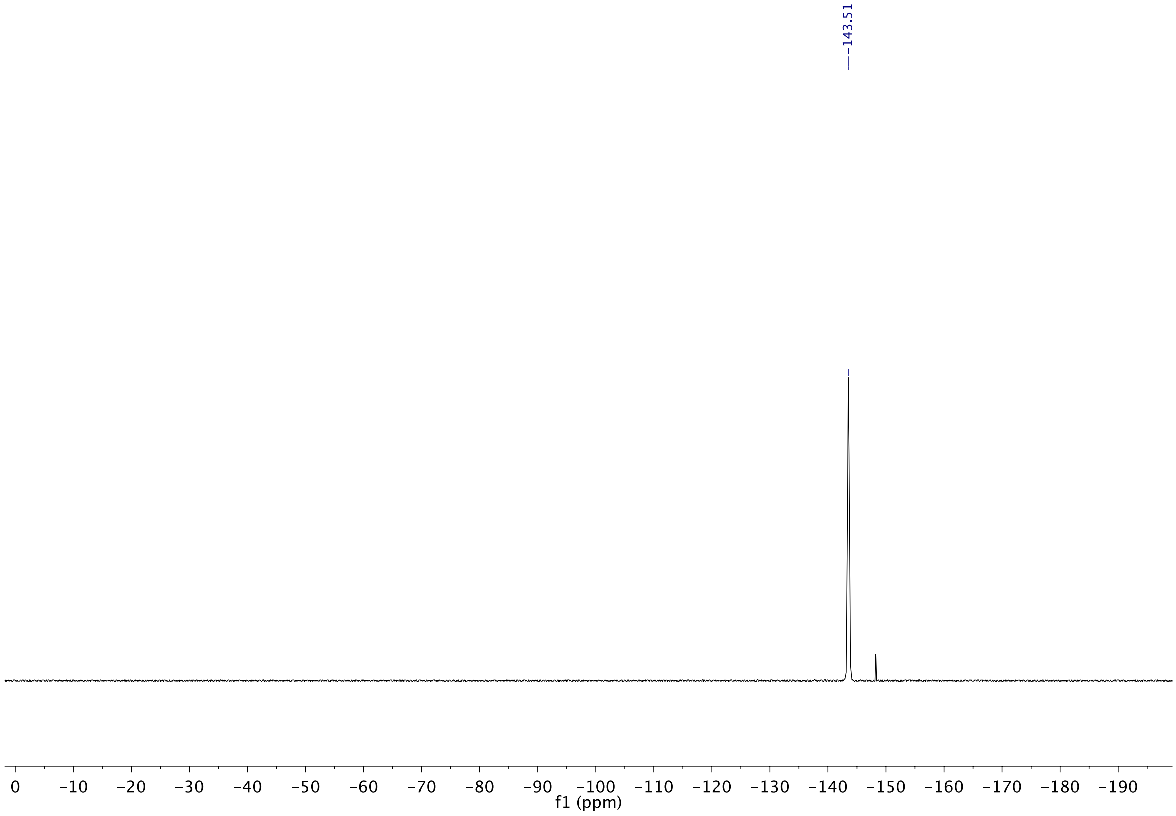
-143.51
0 −10 −20 −30 −40 −50 −60 −70 −80 −90 −100 −110 −120 −130 −140 −150 −160 −170 −180 −190
f1 (ppm)

(*Z*)-(1-Phenylprop-1-en-2-yl)boronic acid, **66**

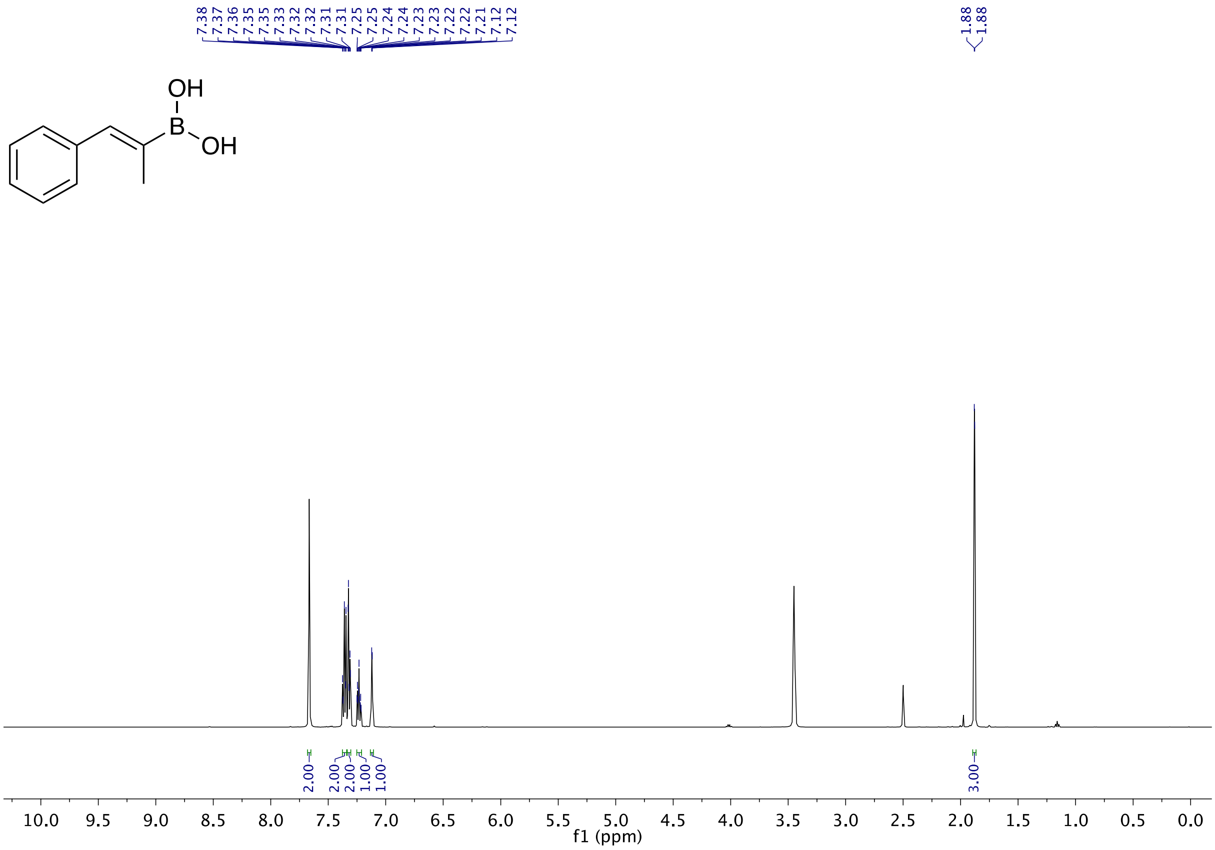

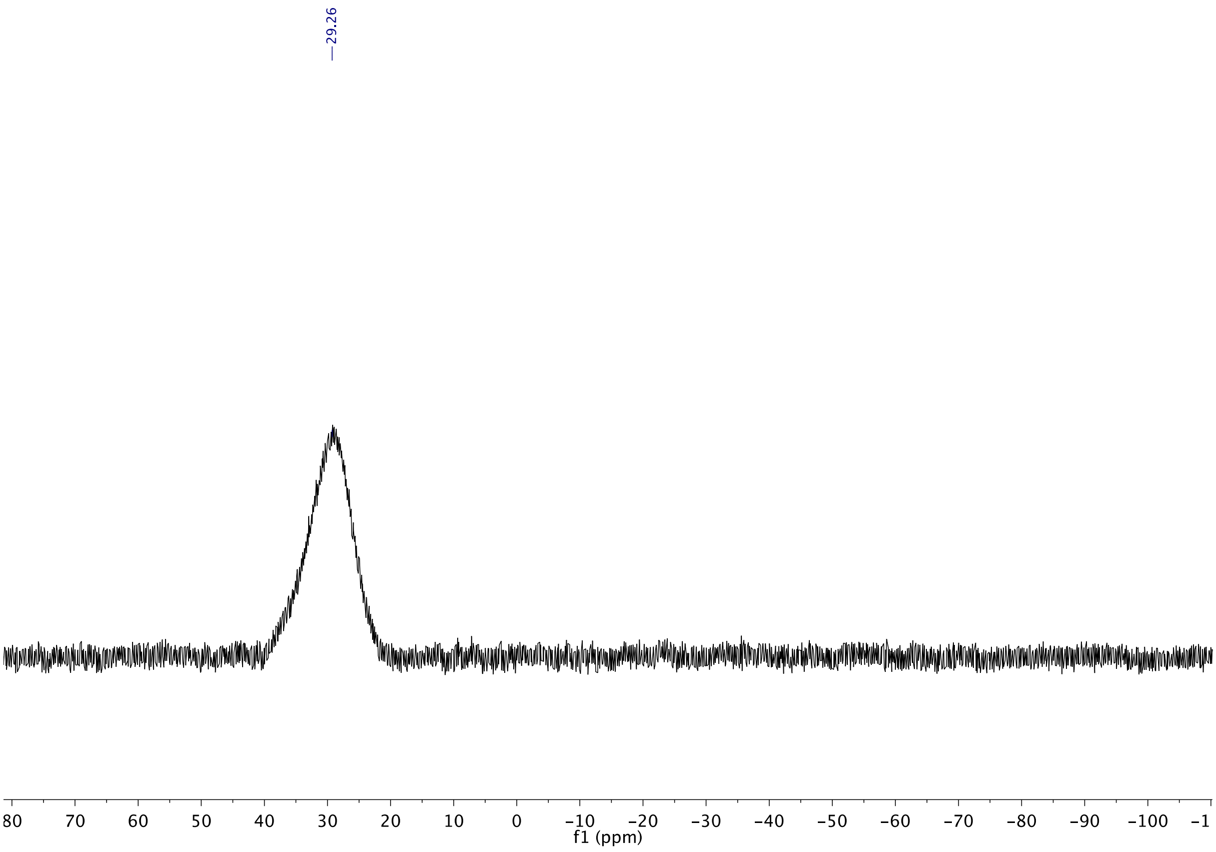

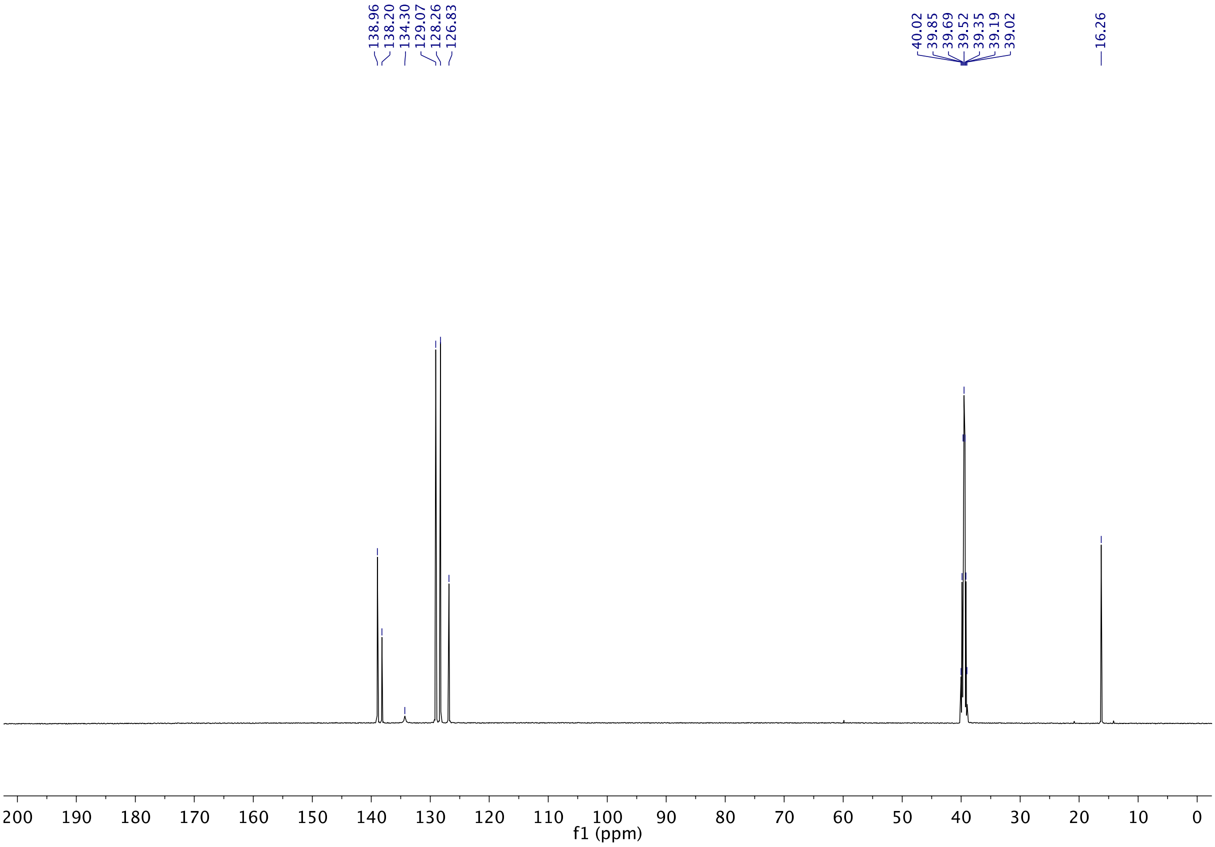

1,2-Diphenylethyne, **S15-int2**

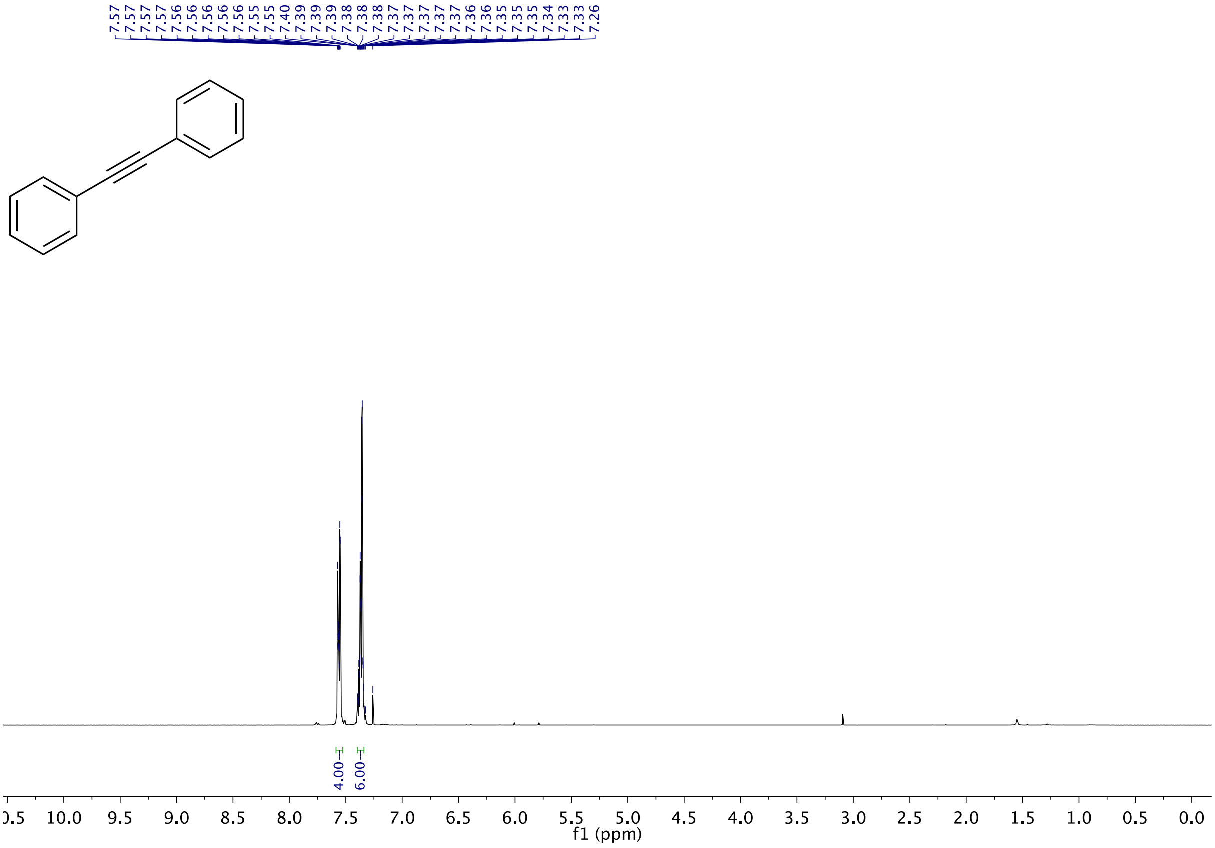

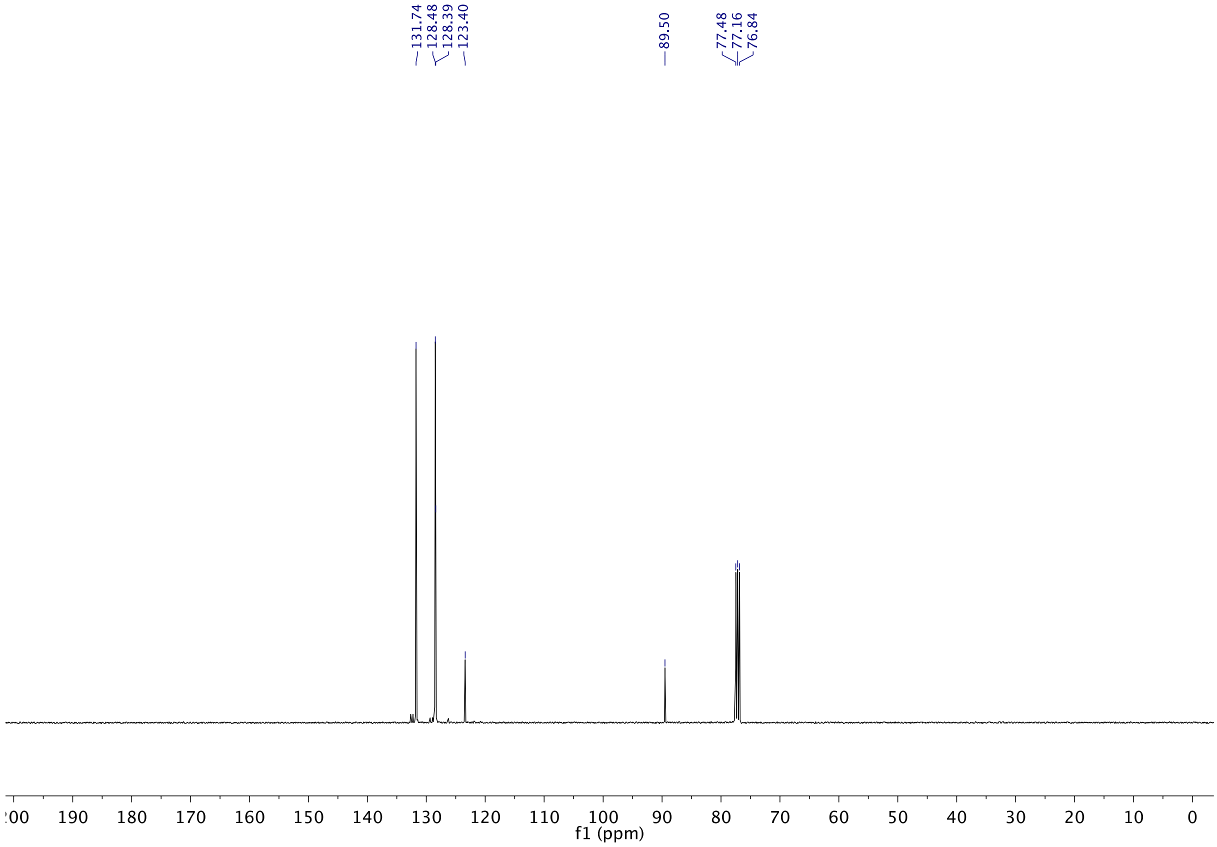

(*Z*)-2-(1,2-Diphenylvinyl)-4,4,5,5-tetramethyl-1,3,2-dioxaborolane, **S15-int3**

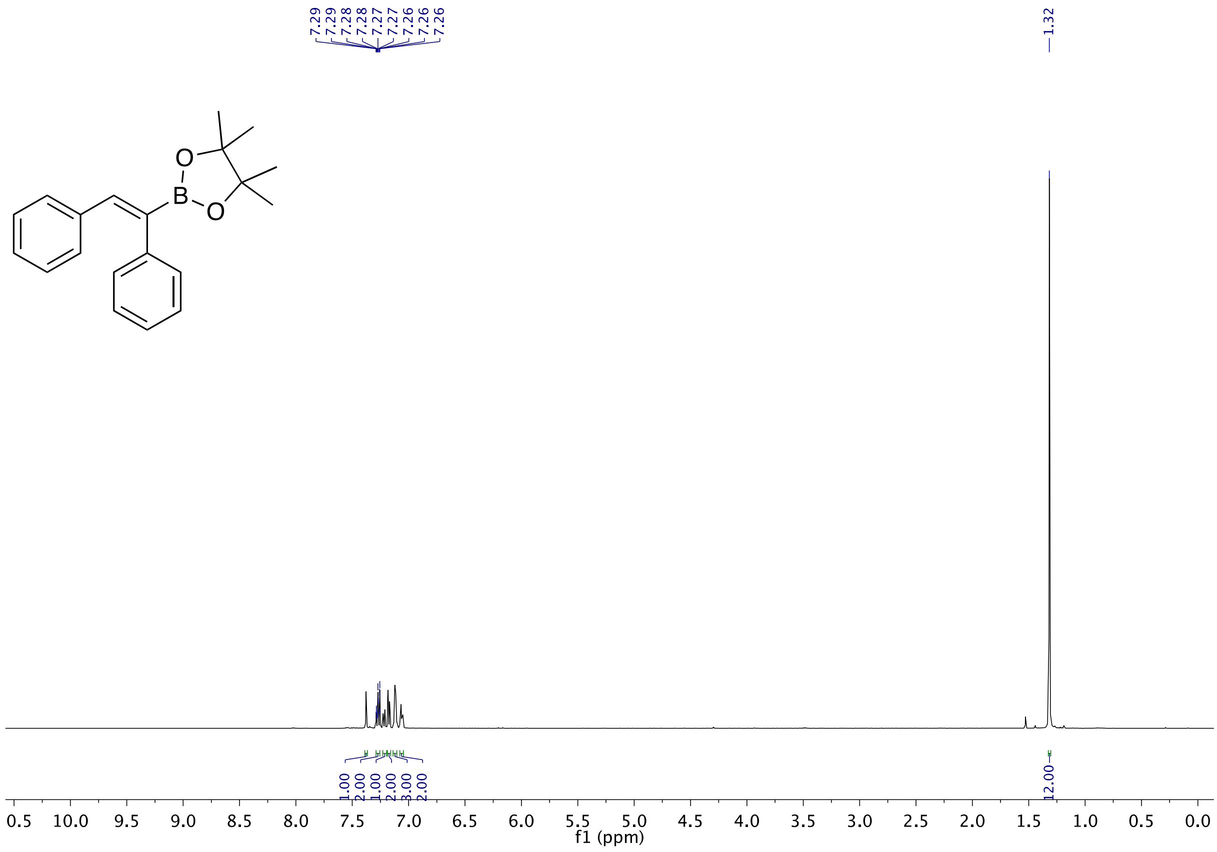

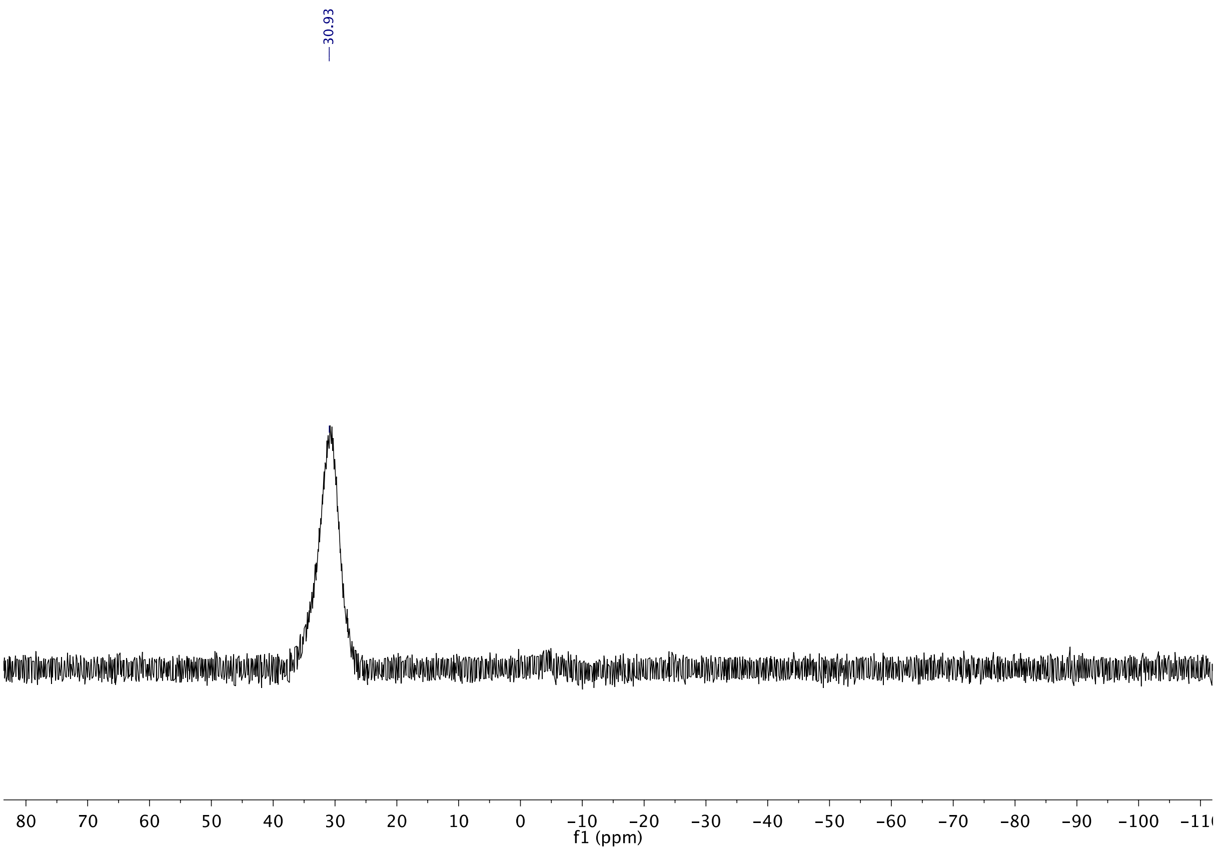

30.93
80
70
60
50
40
30
20
10
0
−10
−20
−30
−40
−50
−60
−70
−80
−90
−100
f1 (ppm)

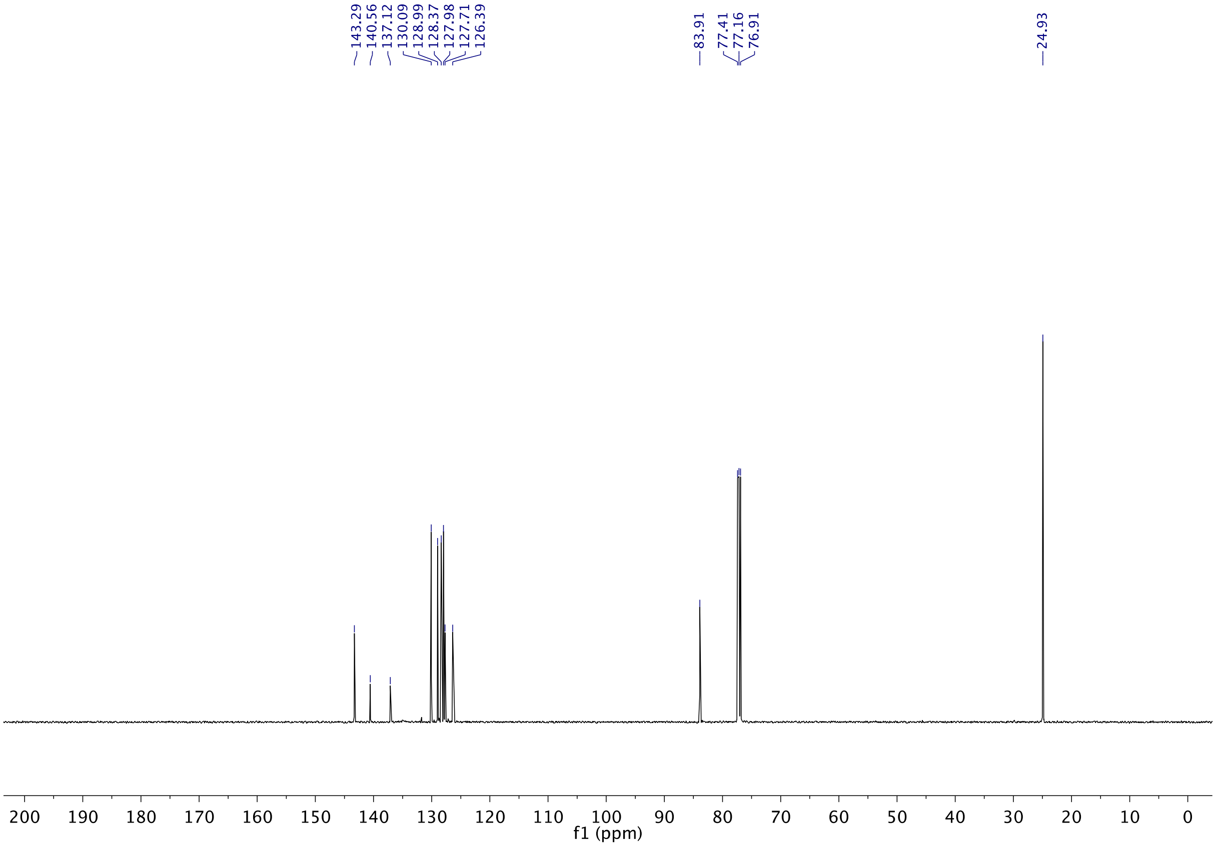

143.29
140.56
137.12
130.09
128.99
128.37
127.98
127.71
126.39
83.91
77.41
77.16
76.91
24.93
200
190
180
170
160
150
140
130
120
110
100
90
80
70
60
50
40
30
20
10
0
f1 (ppm)

(Z)-(1,2-Diphenylvinyl)trifluoro-λ^4-borane, potassium salt, **S15-int4**

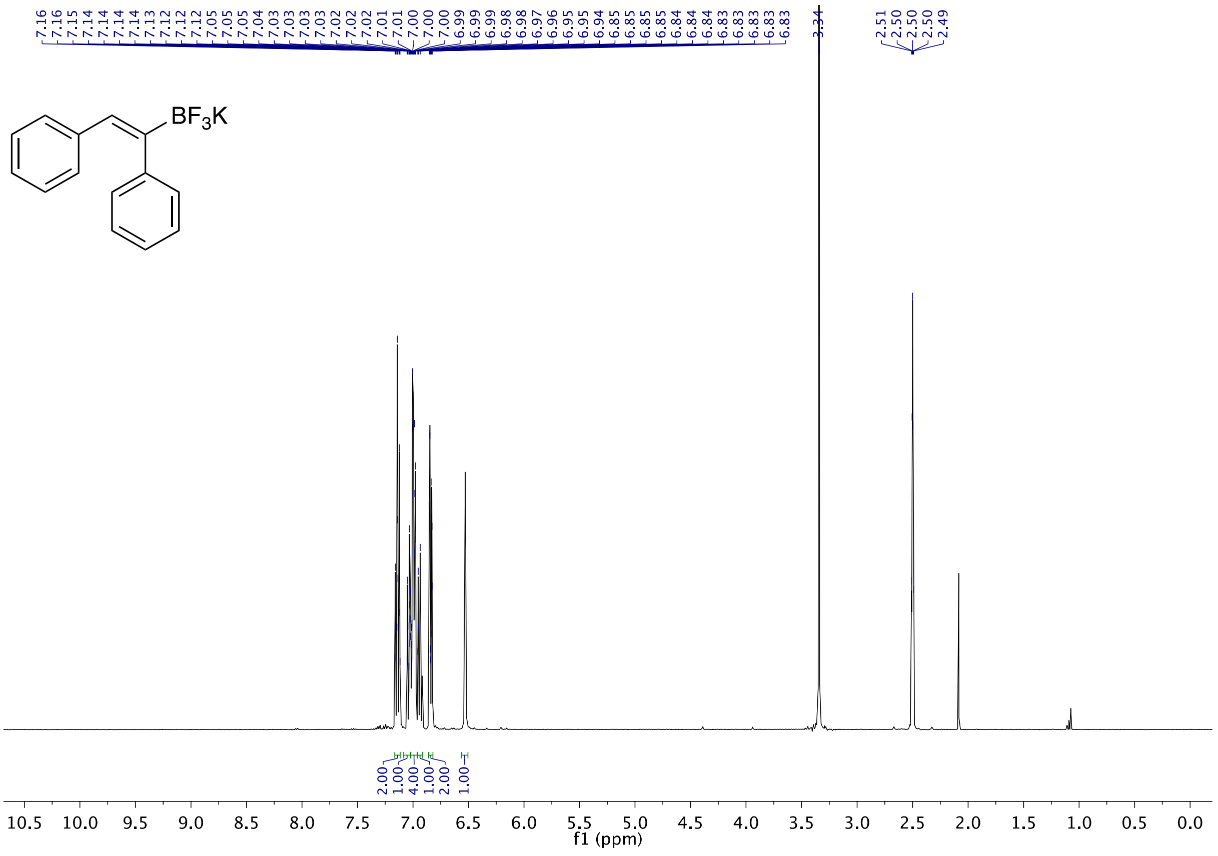

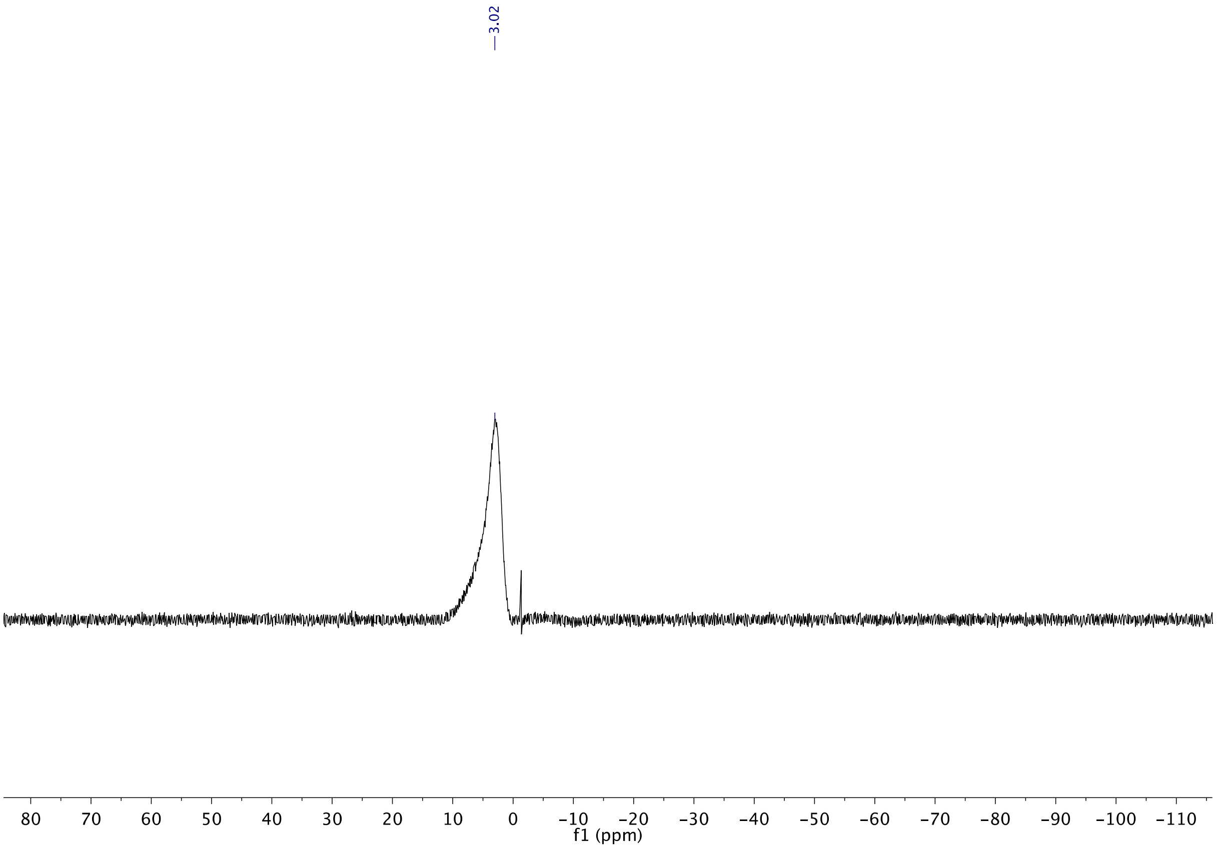

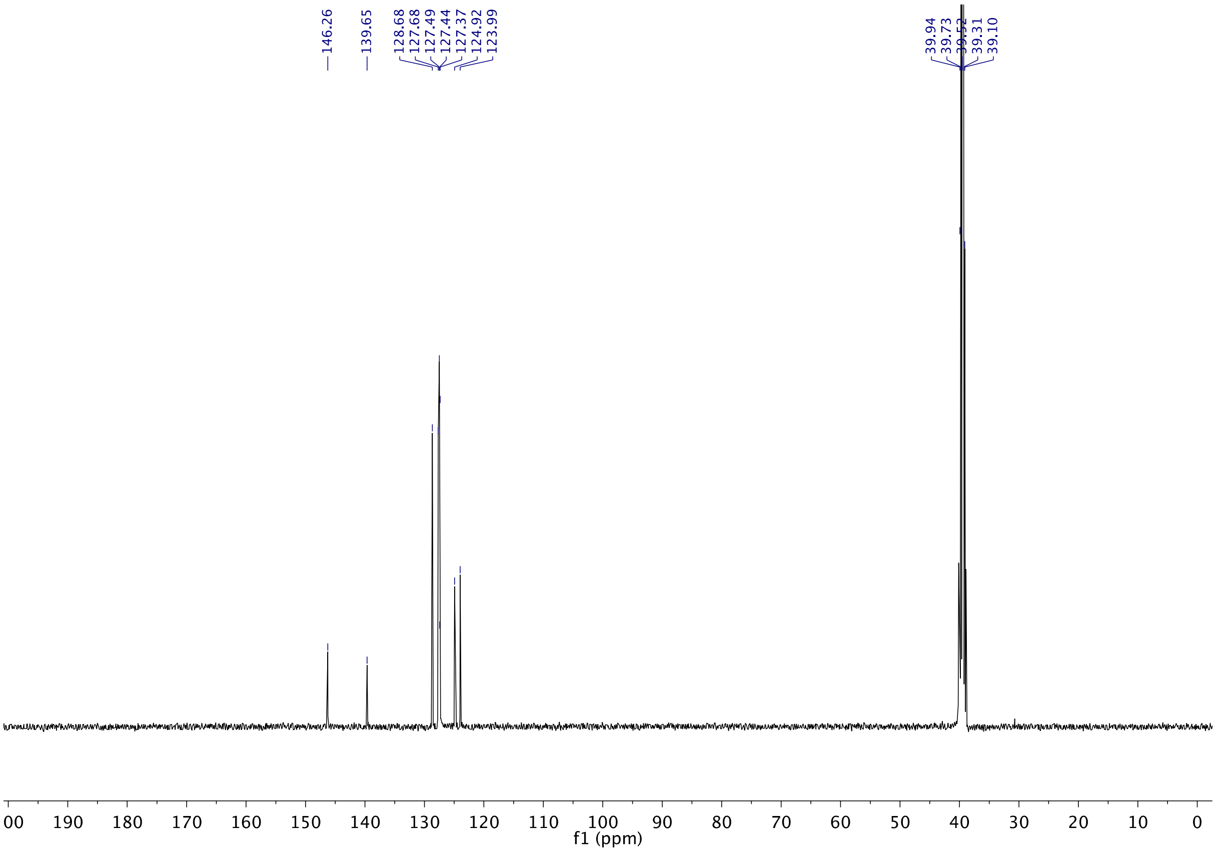
146.26
139.65
128.68
127.68
127.49
127.44
127.37
124.92
123.99
39.94
39.73
39.52
39.31
39.10
00 190 180 170 160 150 140 130 120 110 100 90 80 70 60 50 40 30 20 10 0
f1 (ppm)

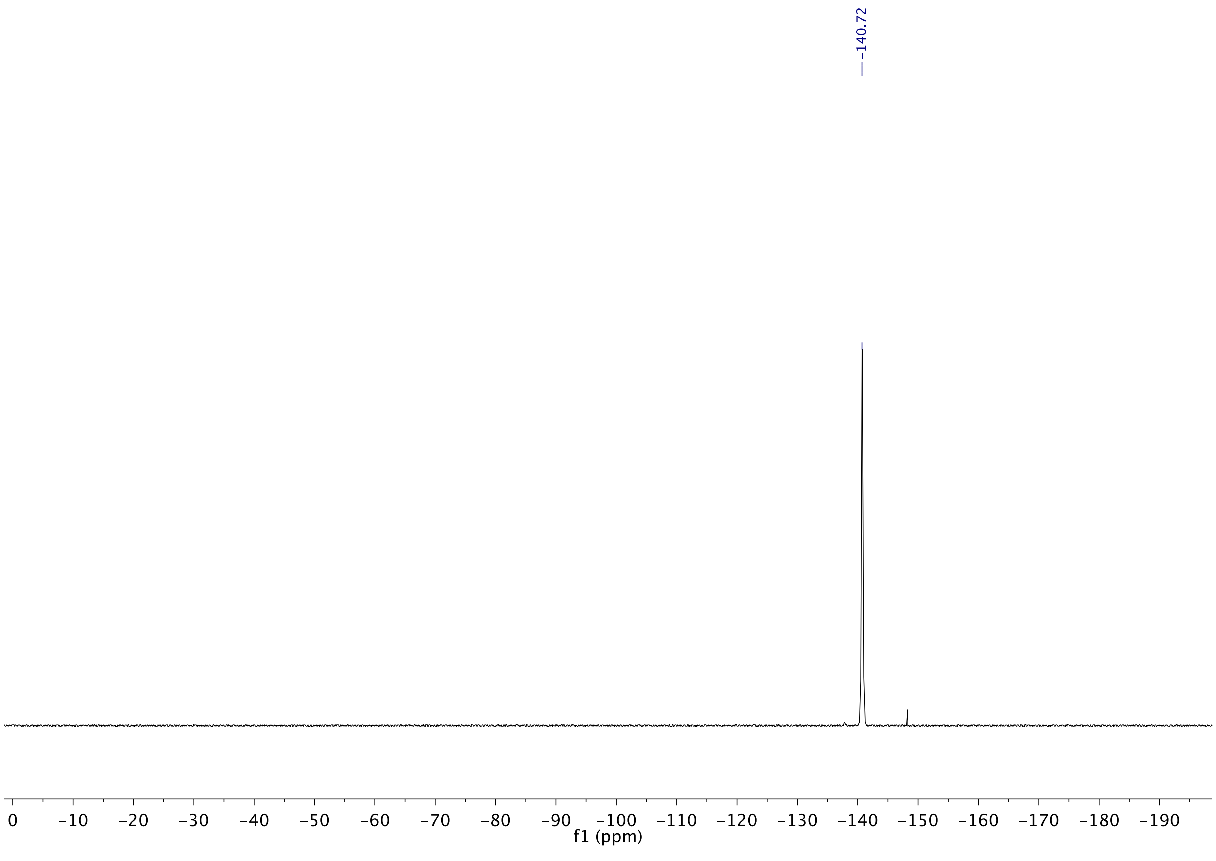
−140.72
0 −10 −20 −30 −40 −50 −60 −70 −80 −90 −100 −110 −120 −130 −140 −150 −160 −170 −180 −190
f1 (ppm)

(*Z*)-(1,2-Diphenylvinyl)boronic acid, **S15**

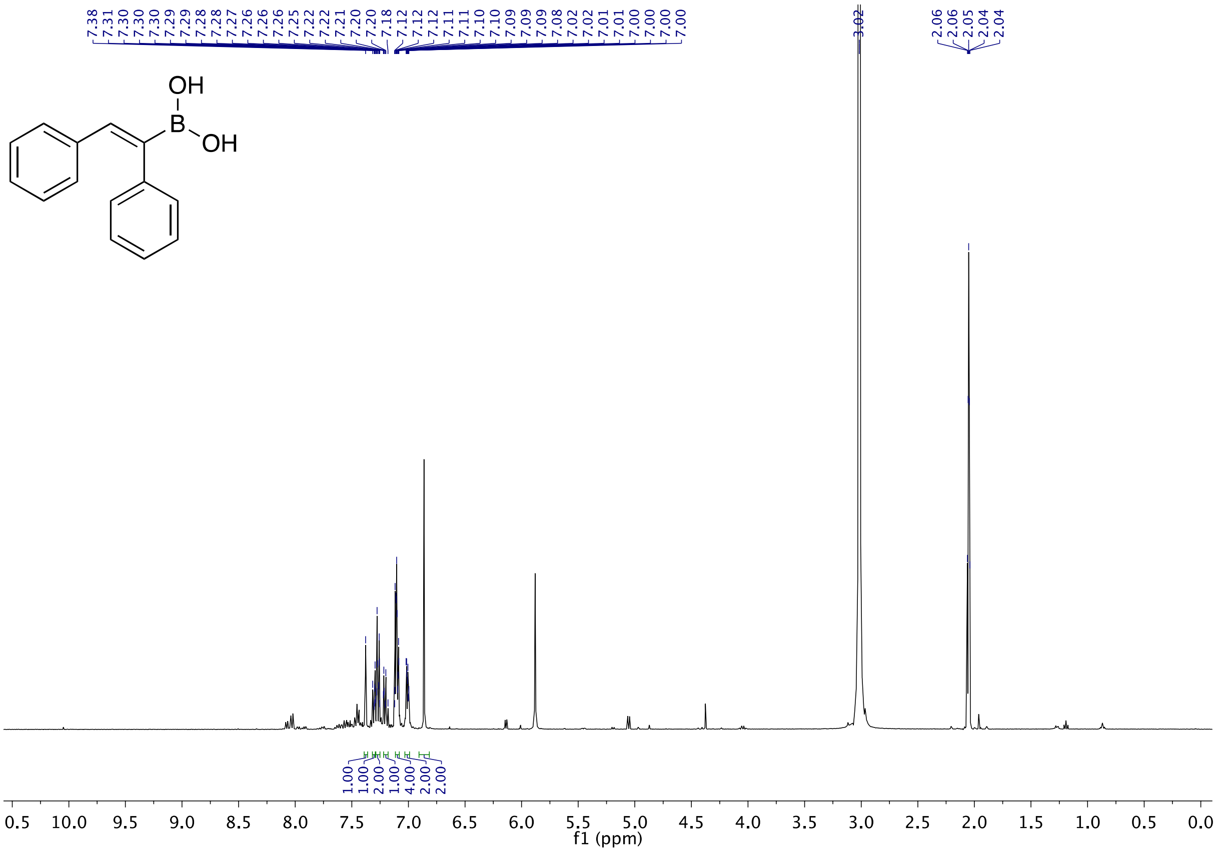

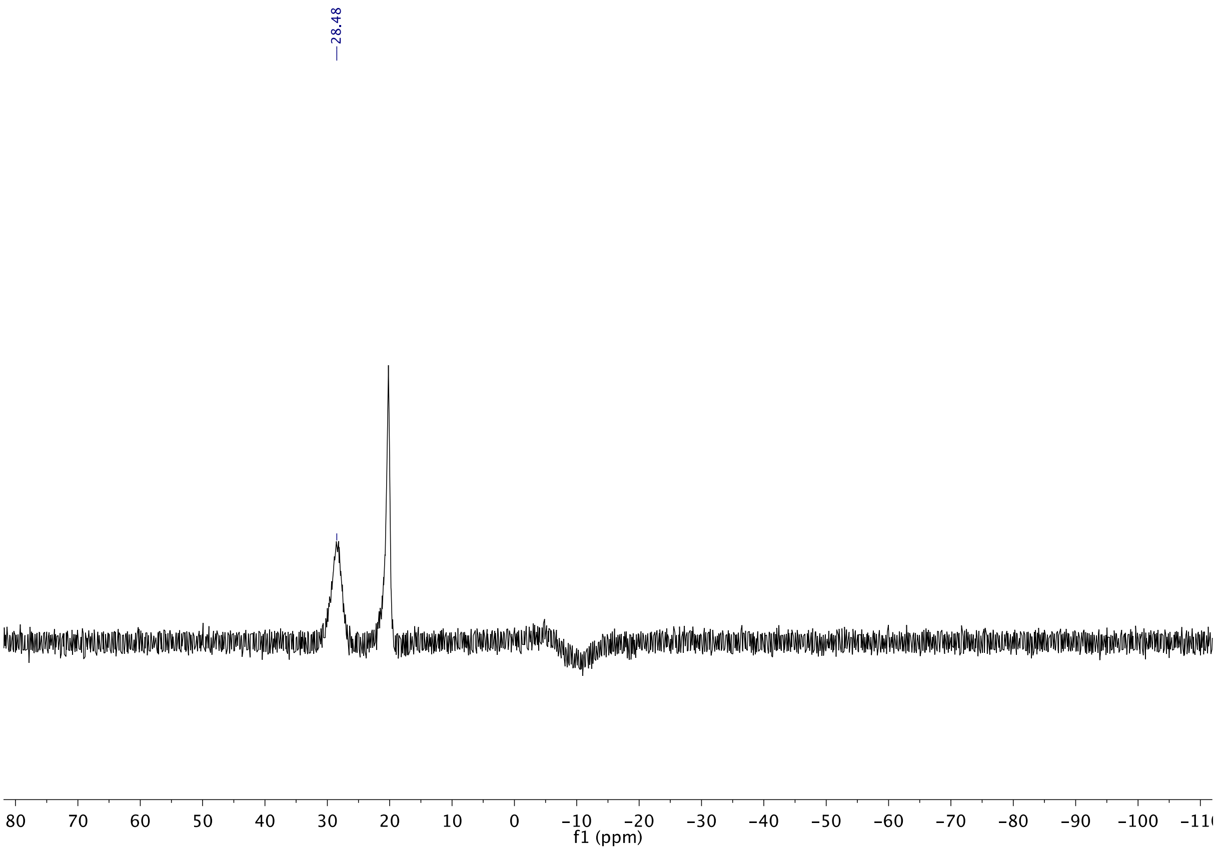

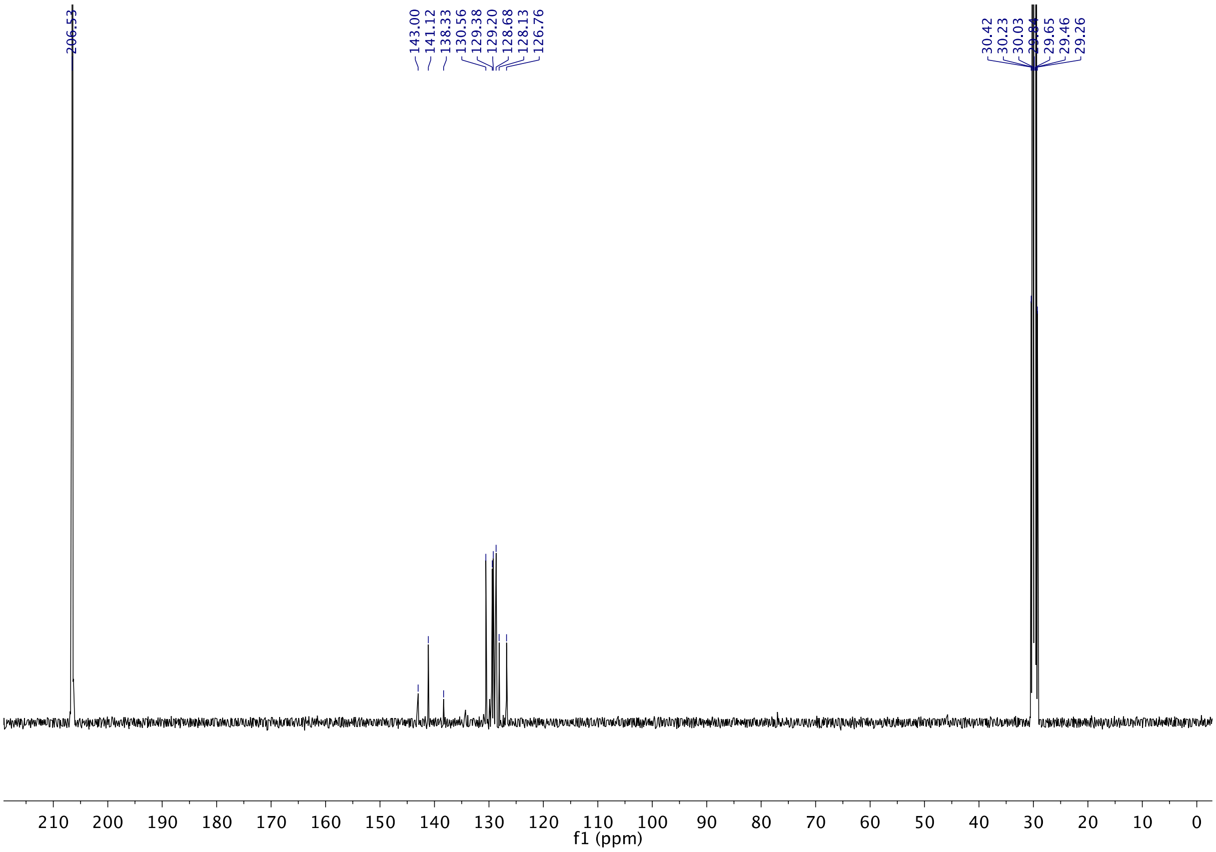

3,4-Dihydronaphthalen-2-yl trifluoromethanesulfonate, **S16-int0**

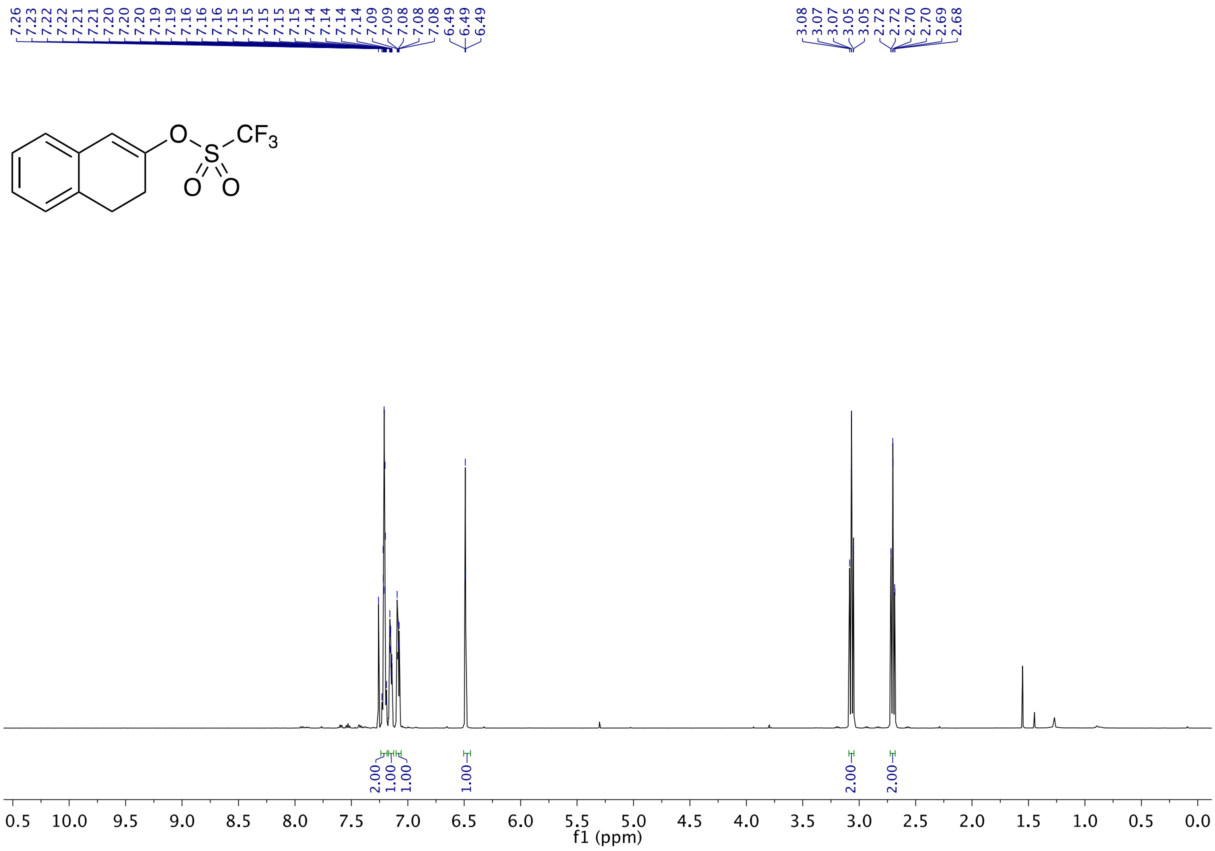

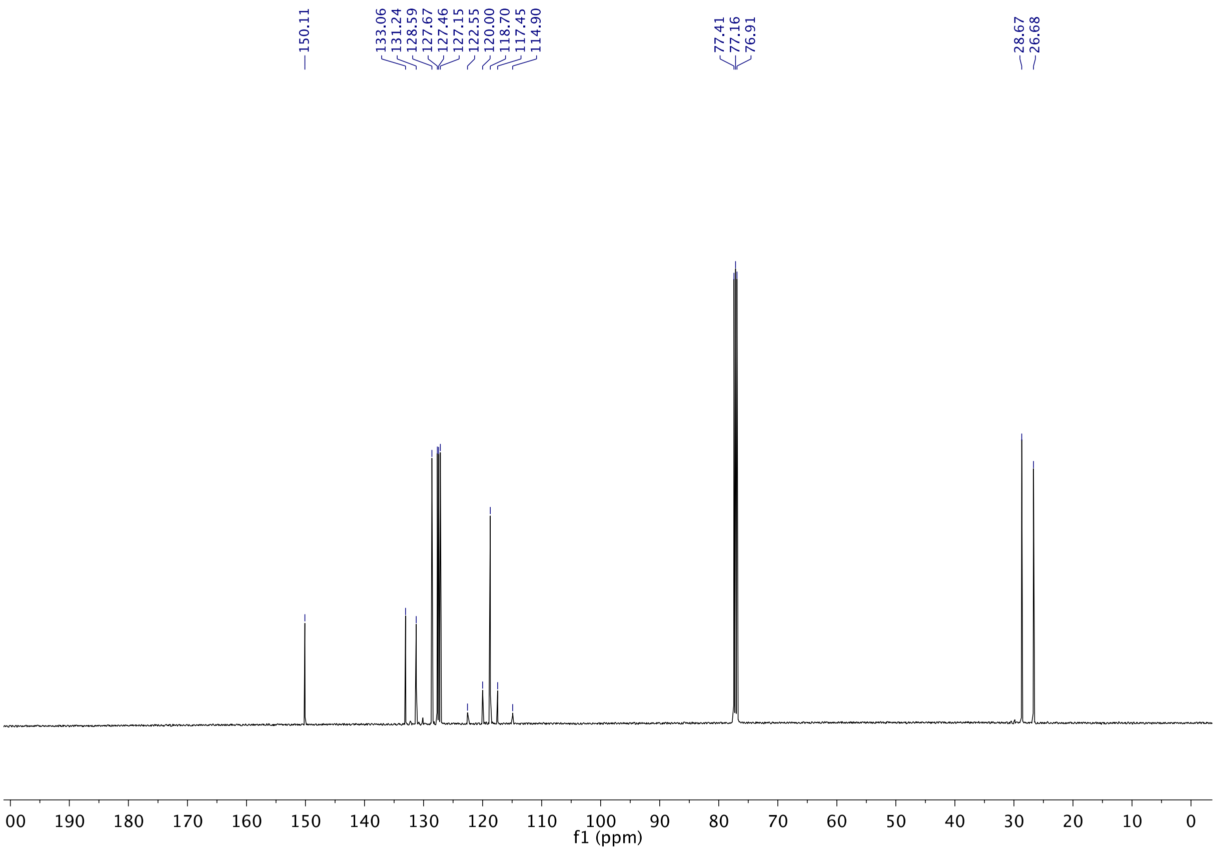
150.11
133.06
131.24
128.59
127.67
127.46
127.15
122.55
120.00
118.70
117.45
114.90
77.41
77.16
76.91
28.67
26.68
f1 (ppm)

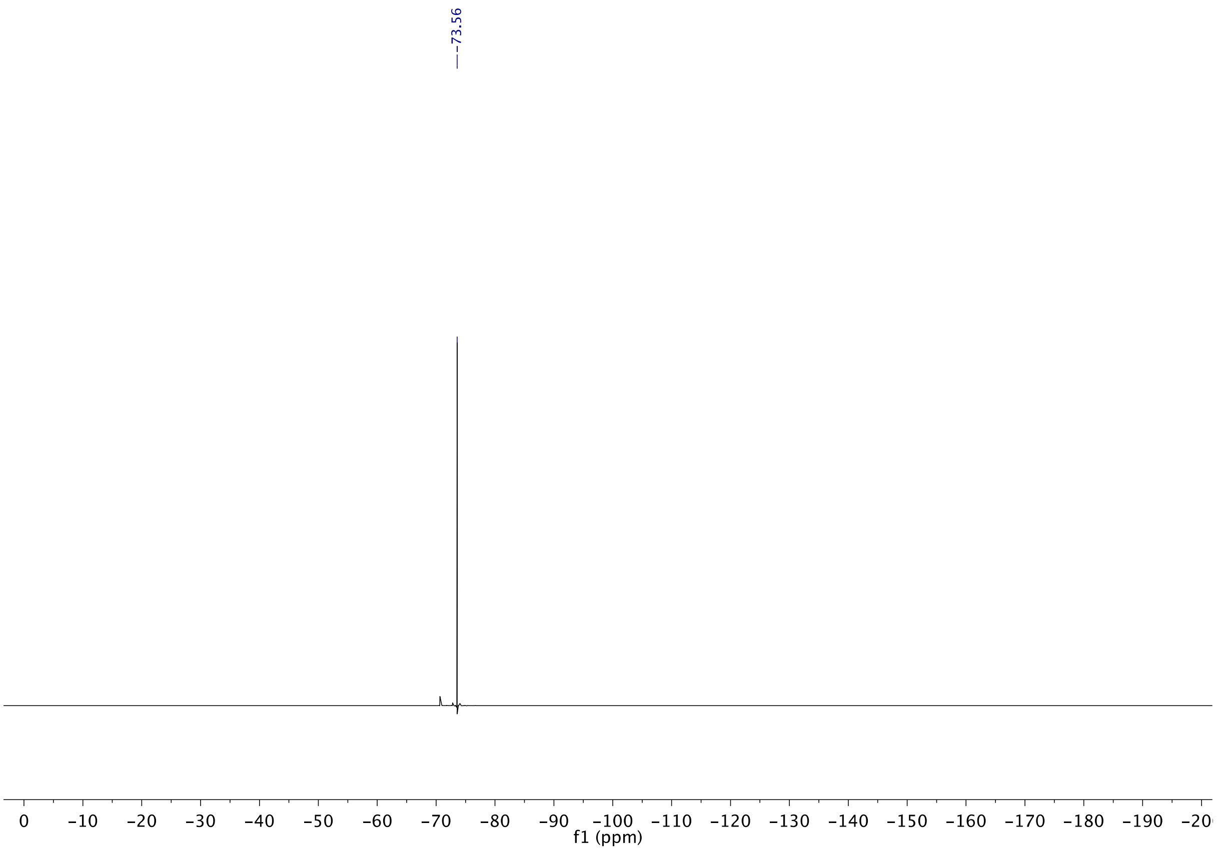
-73.56
f1 (ppm)

2-(3,4-Dihydronaphthalen-2-yl)-4,4,5,5-tetramethyl-1,3,2-dioxaborolane, **S16-int3**

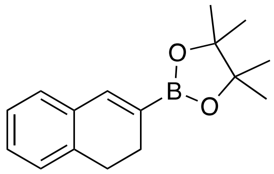

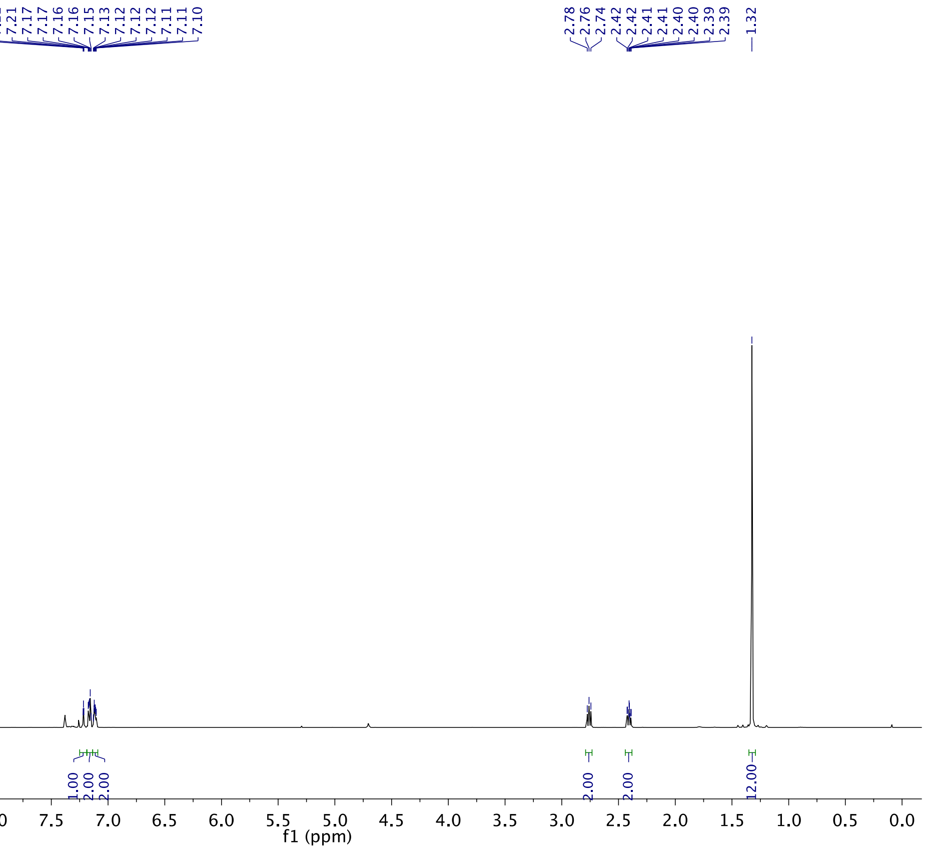

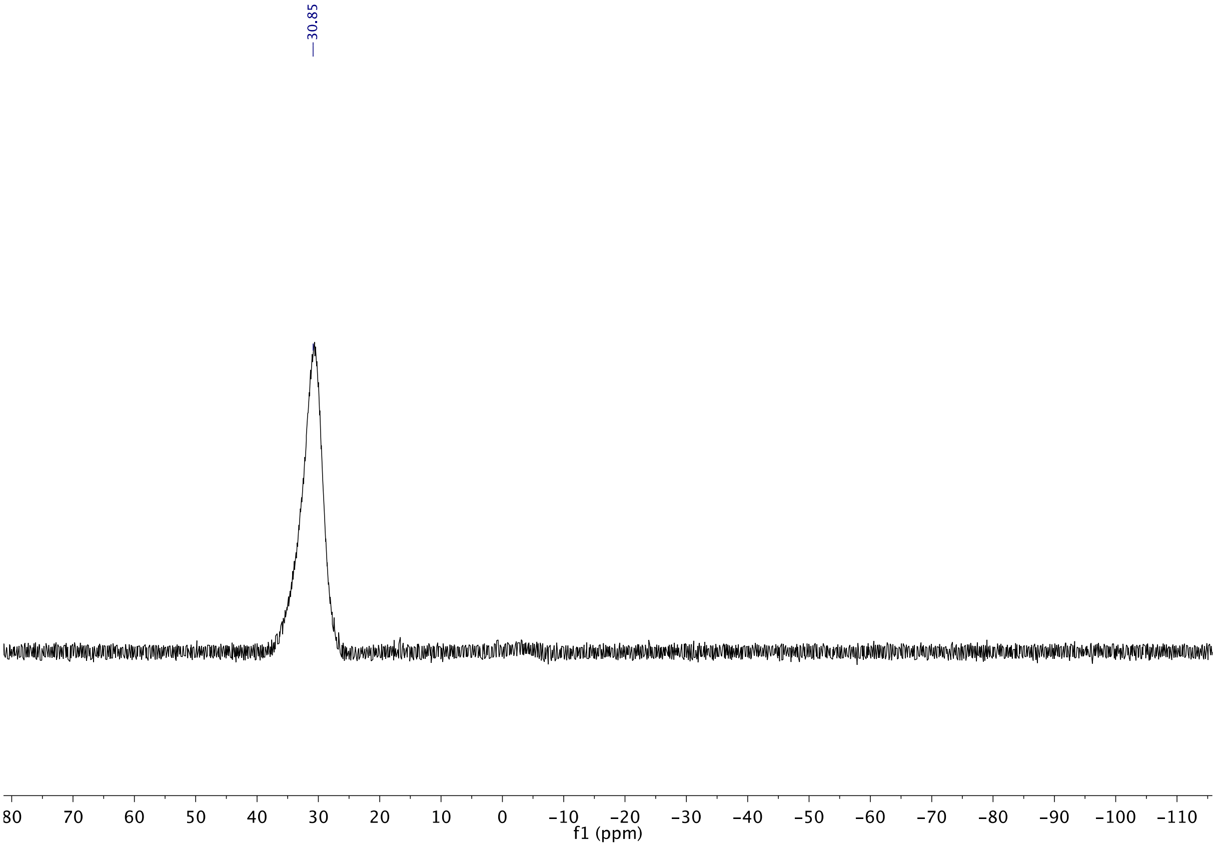

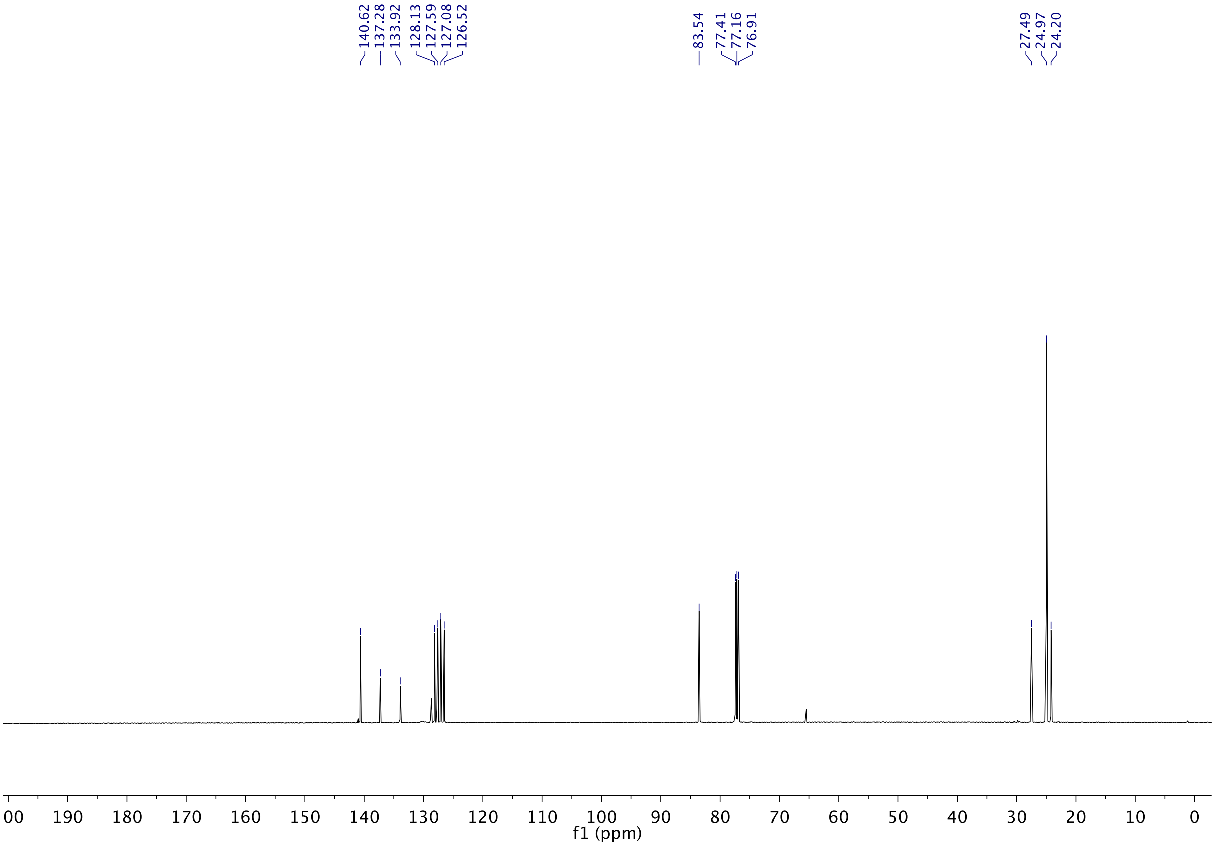

(3,4-Dihydronaphthalen-2-yl)trifluoro-λ^4-borane, potassium salt, **S16-int4**

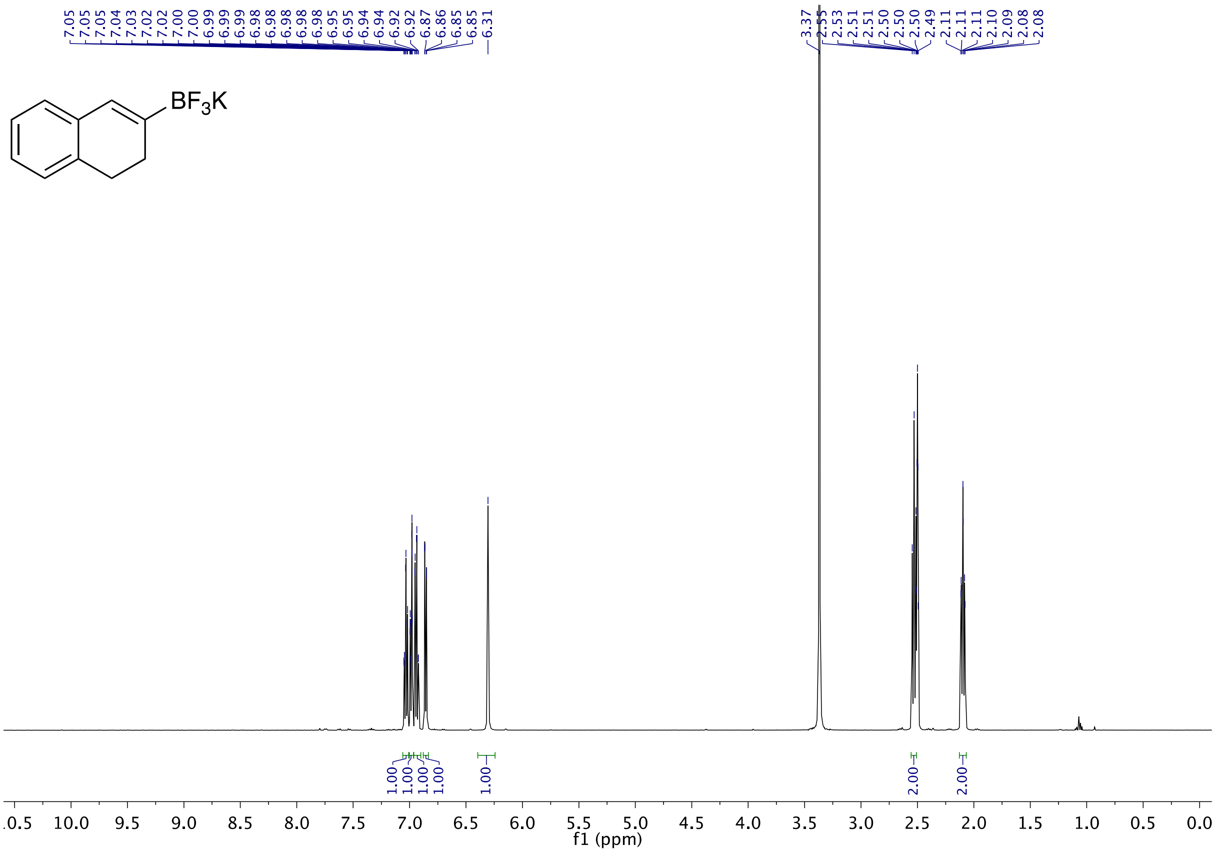

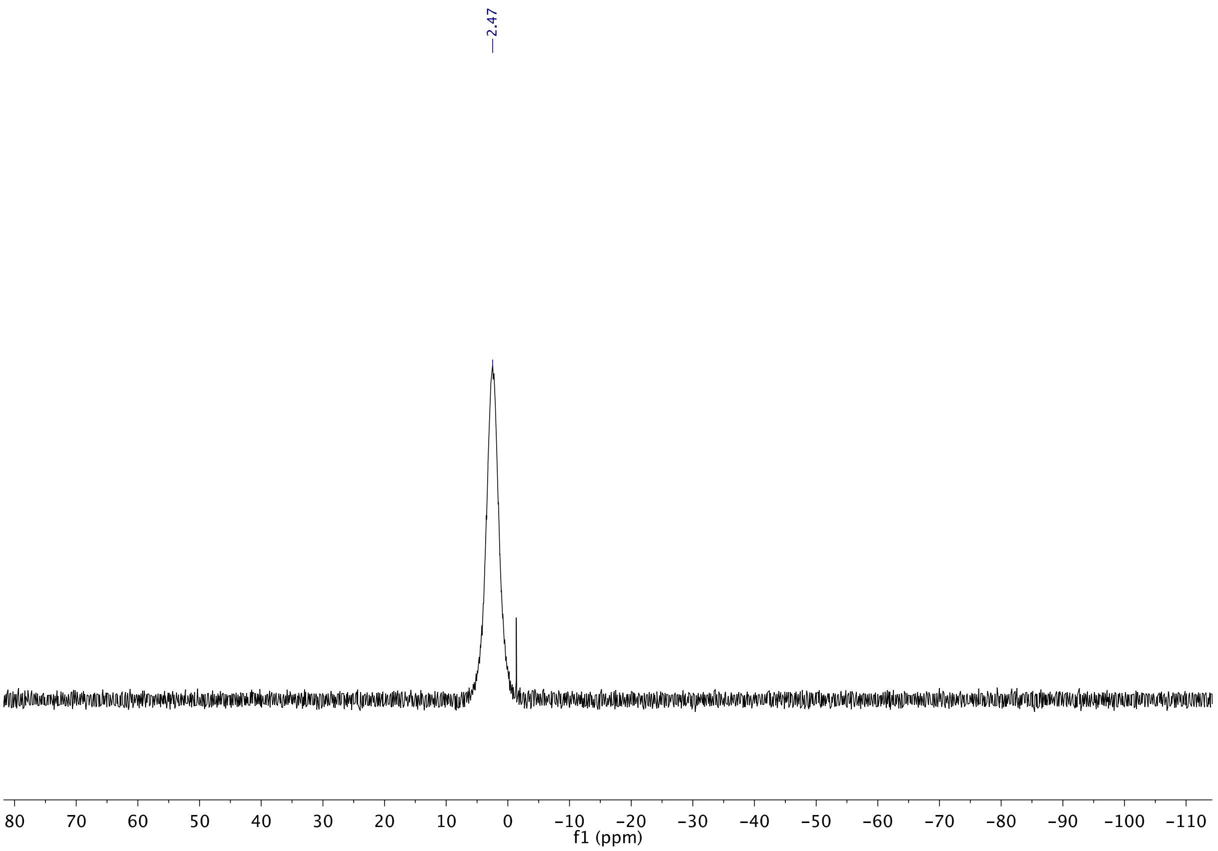
2.47
80 70 60 50 40 30 20 10 0 −10 −20 −30 −40 −50 −60 −70 −80 −90 −100 −110
f1 (ppm)

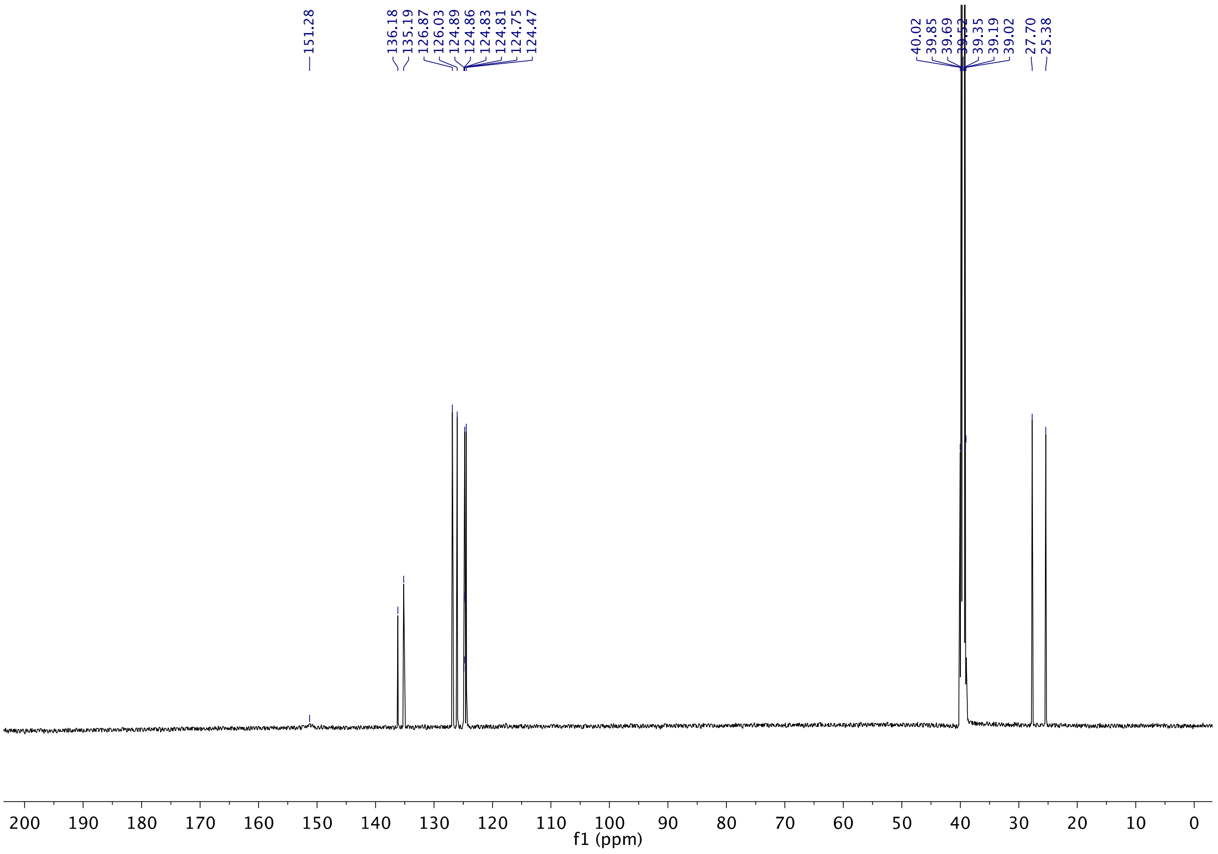
151.28
136.18
135.19
126.87
126.03
124.89
124.86
124.83
124.81
124.75
124.47
40.02
39.85
39.69
39.52
39.35
39.19
39.02
27.70
25.38
200 190 180 170 160 150 140 130 120 110 100 90 80 70 60 50 40 30 20 10 0
f1 (ppm)

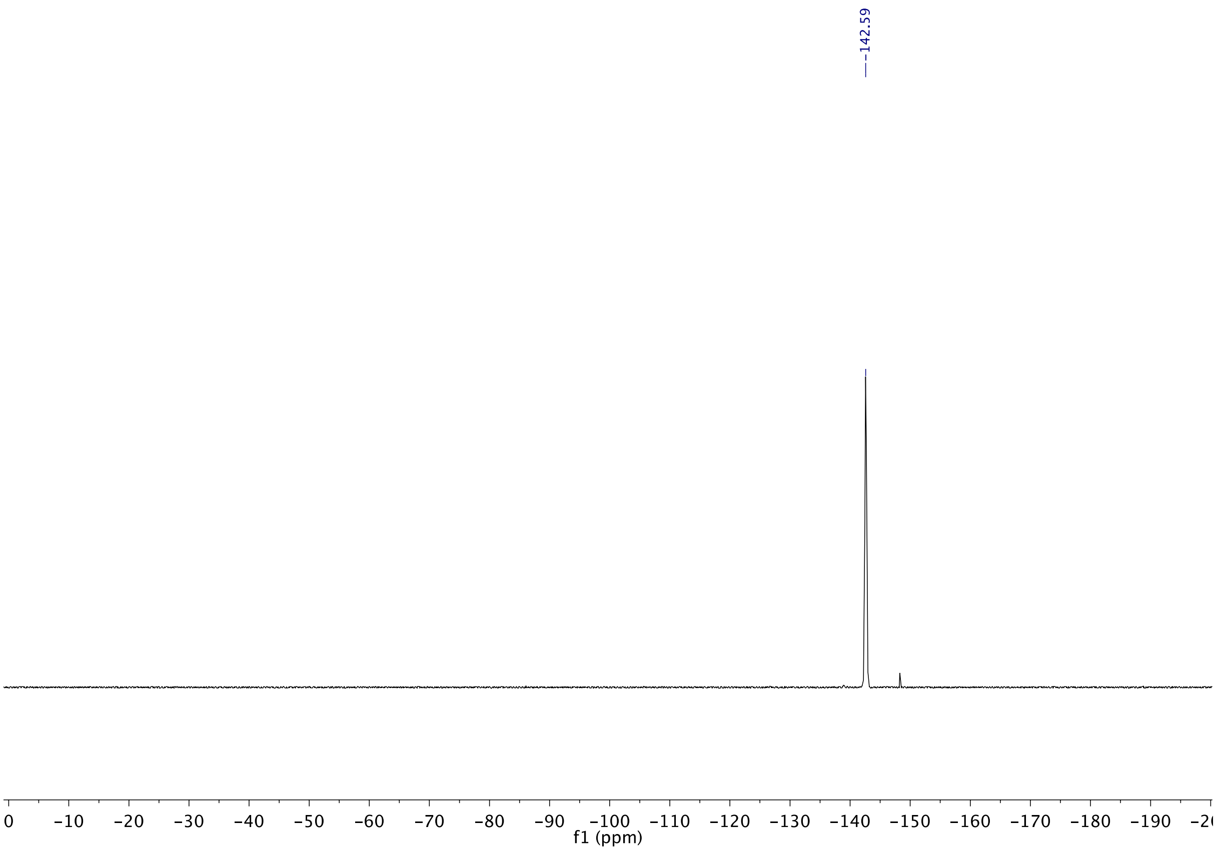

(3,4-Dihydronaphthalen-2-yl)boronic acid, **S16**

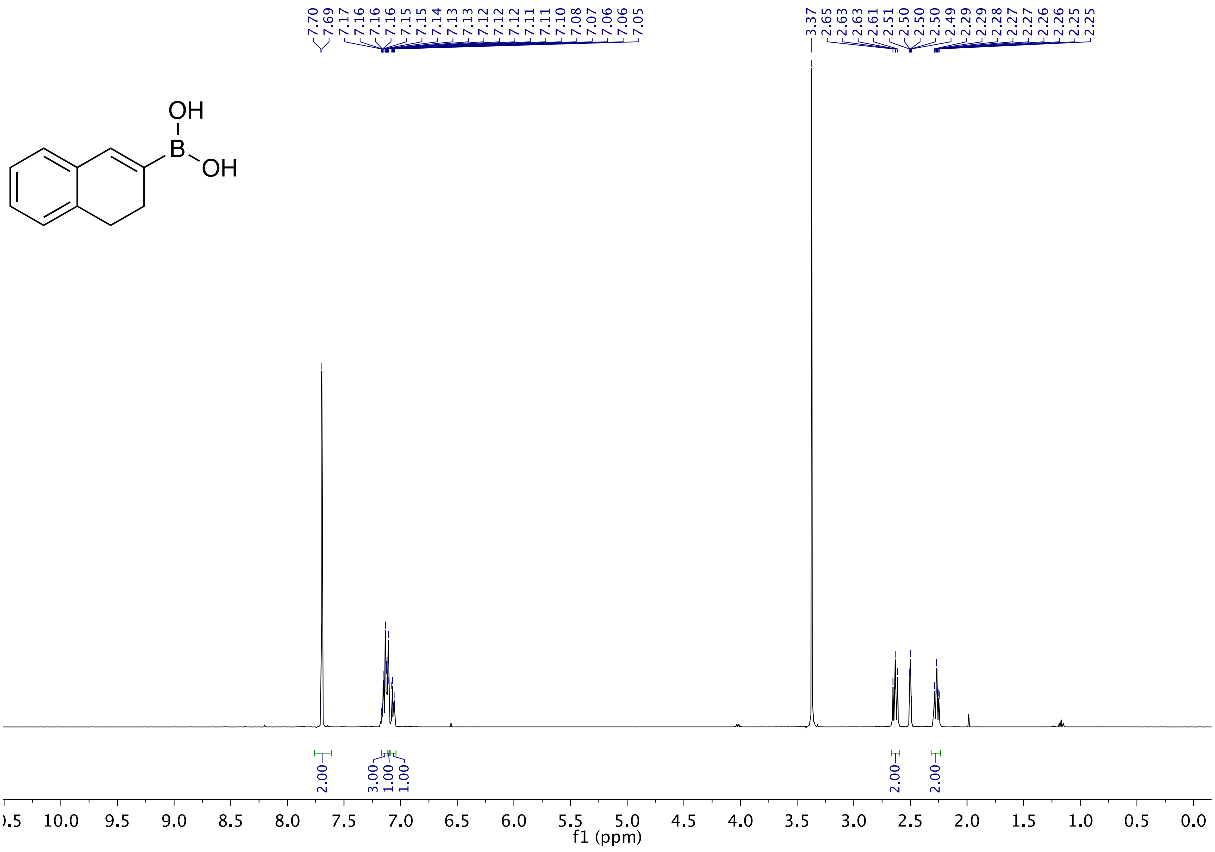

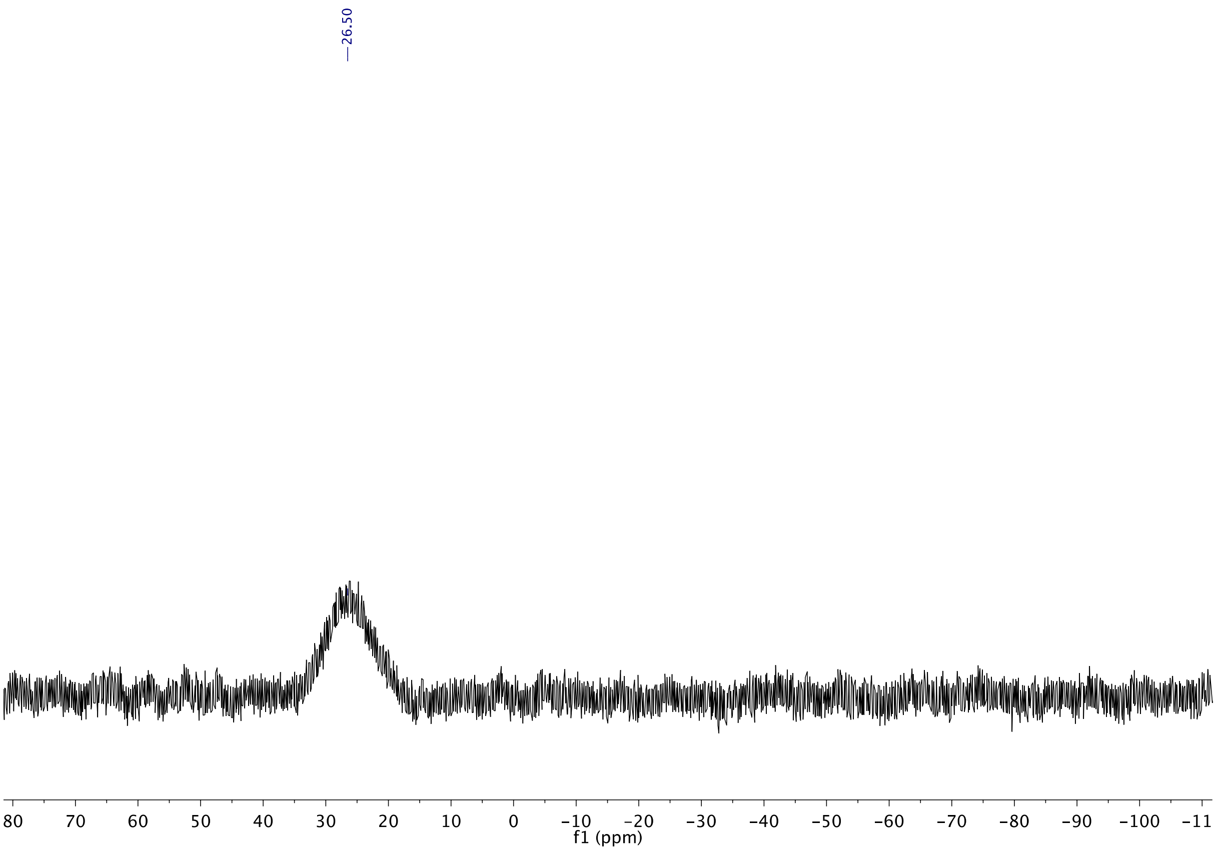
26.50
80 70 60 50 40 30 20 10 0 −10 −20 −30 −40 −50 −60 −70 −80 −90 −100 −11
f1 (ppm)

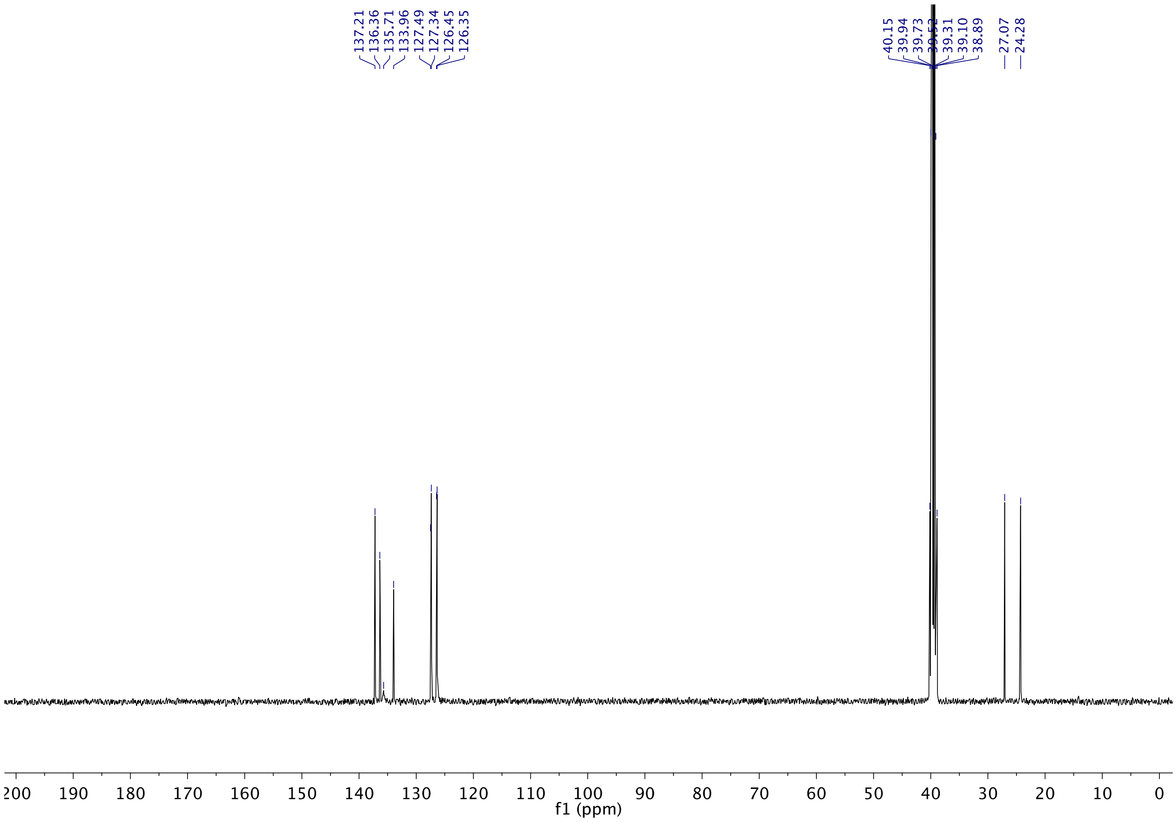
137.21
136.36
135.71
133.96
127.49
127.34
126.45
126.35
40.15
39.94
39.73
39.31
39.10
38.89
27.07
24.28
200 190 180 170 160 150 140 130 120 110 100 90 80 70 60 50 40 30 20 10 0
f1 (ppm)

(*E*)-4-(2-(4,4,5,5-Tetramethyl-1,3,2-dioxaborolan-2-yl)vinyl)pyridine, **S17-int3**

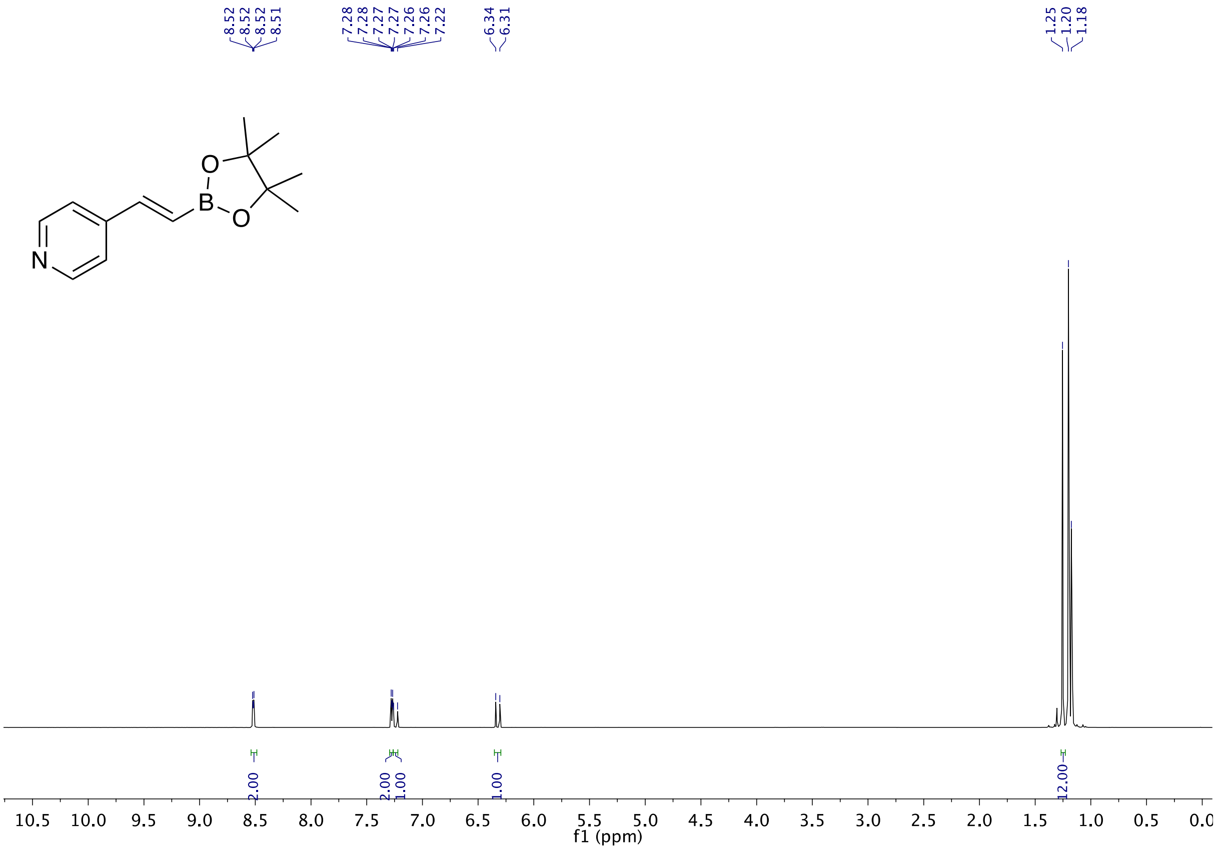

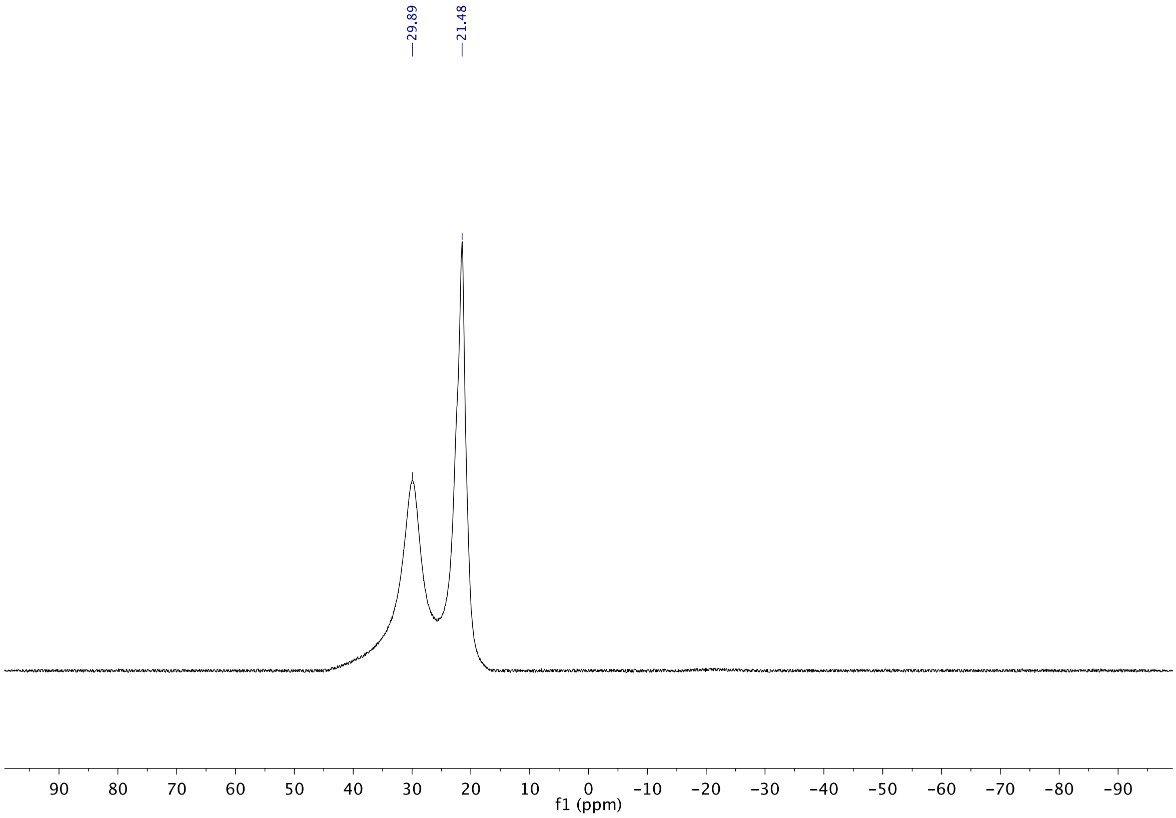

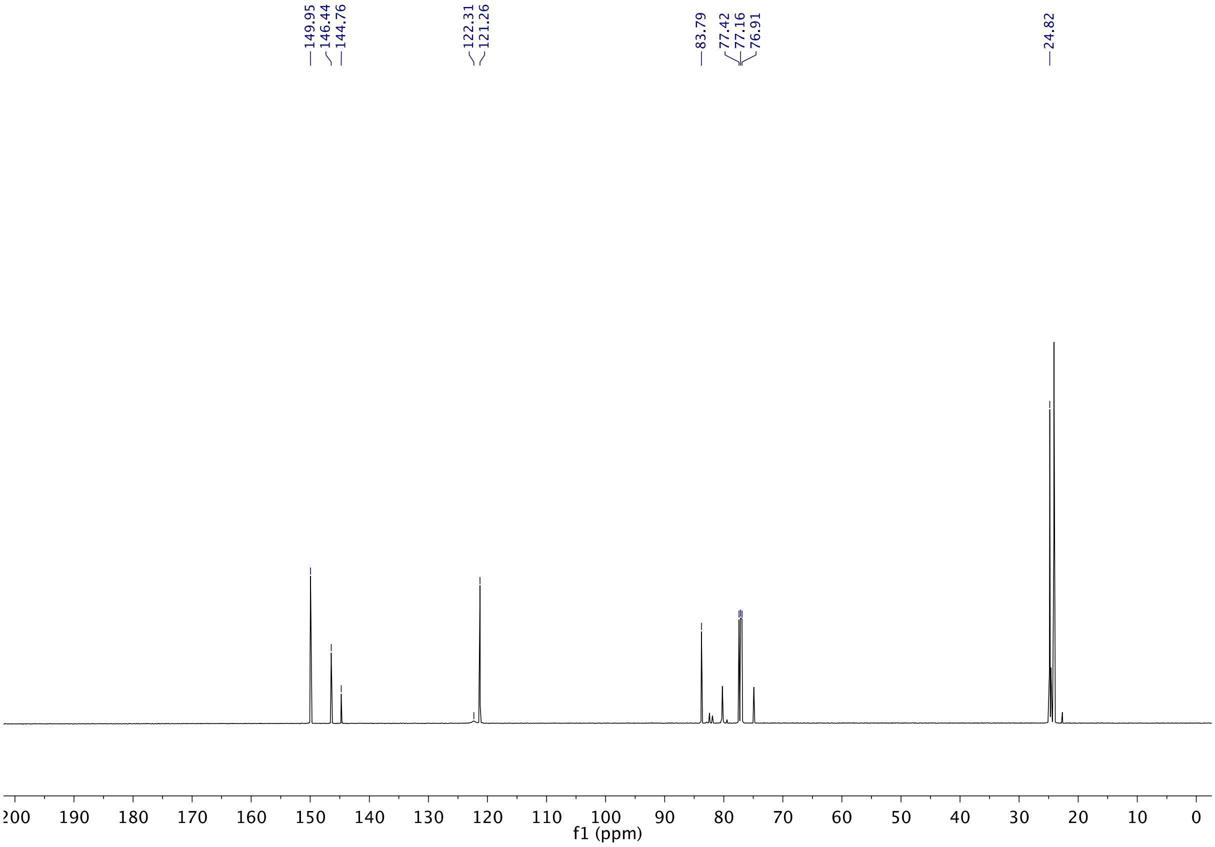

(E)-4-(2-(Trifluoro-λ^4-boraneyl)vinyl)pyridine, potassium salt, **S17-int4**

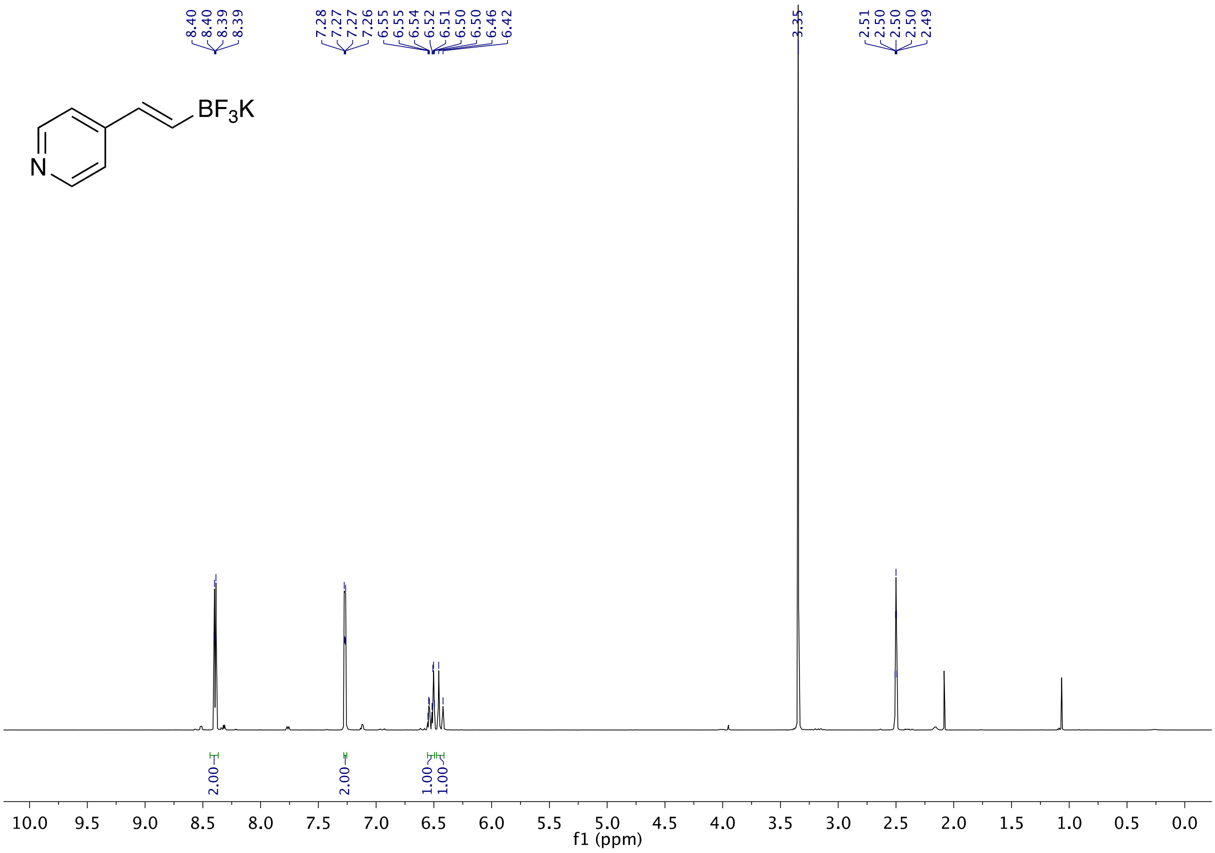

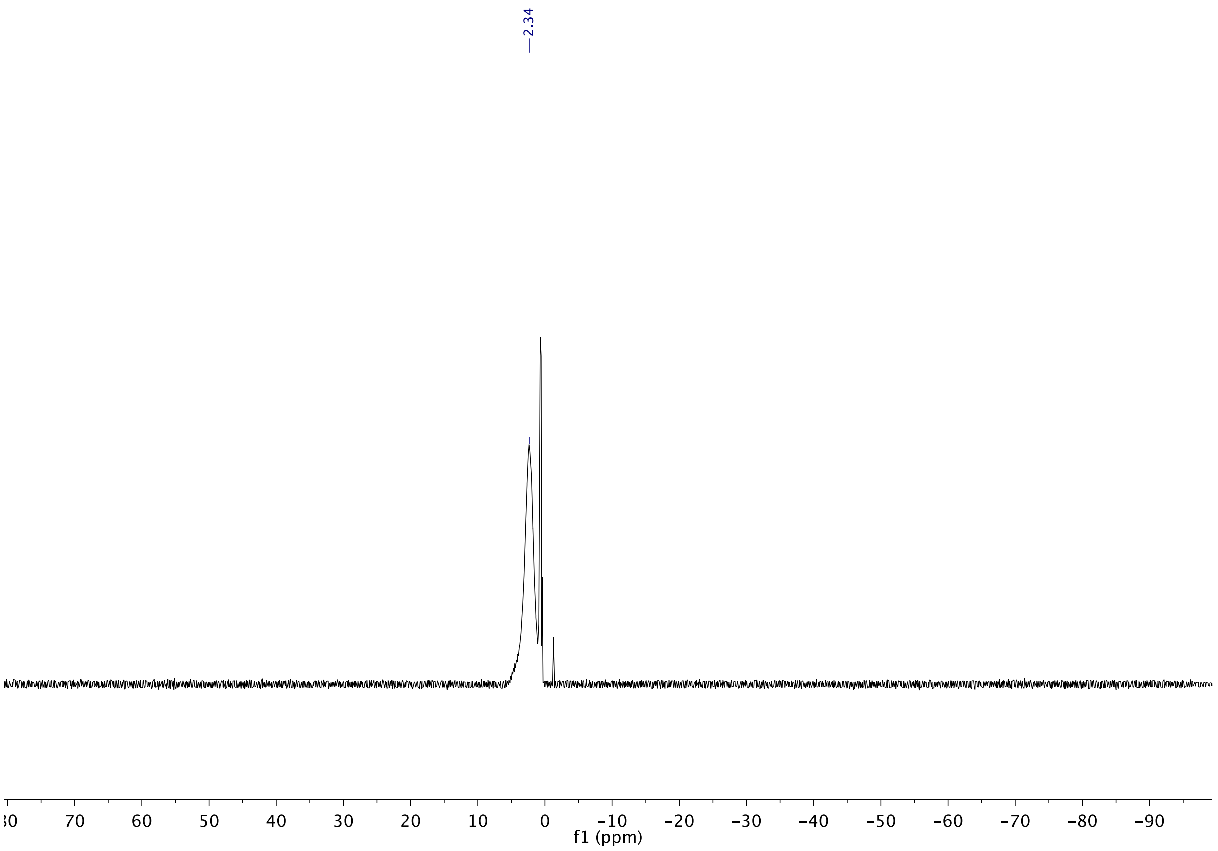
2.34
80
70
60
50
40
30
20
10
0
−10
−20
−30
−40
−50
−60
−70
−80
−90
f1 (ppm)

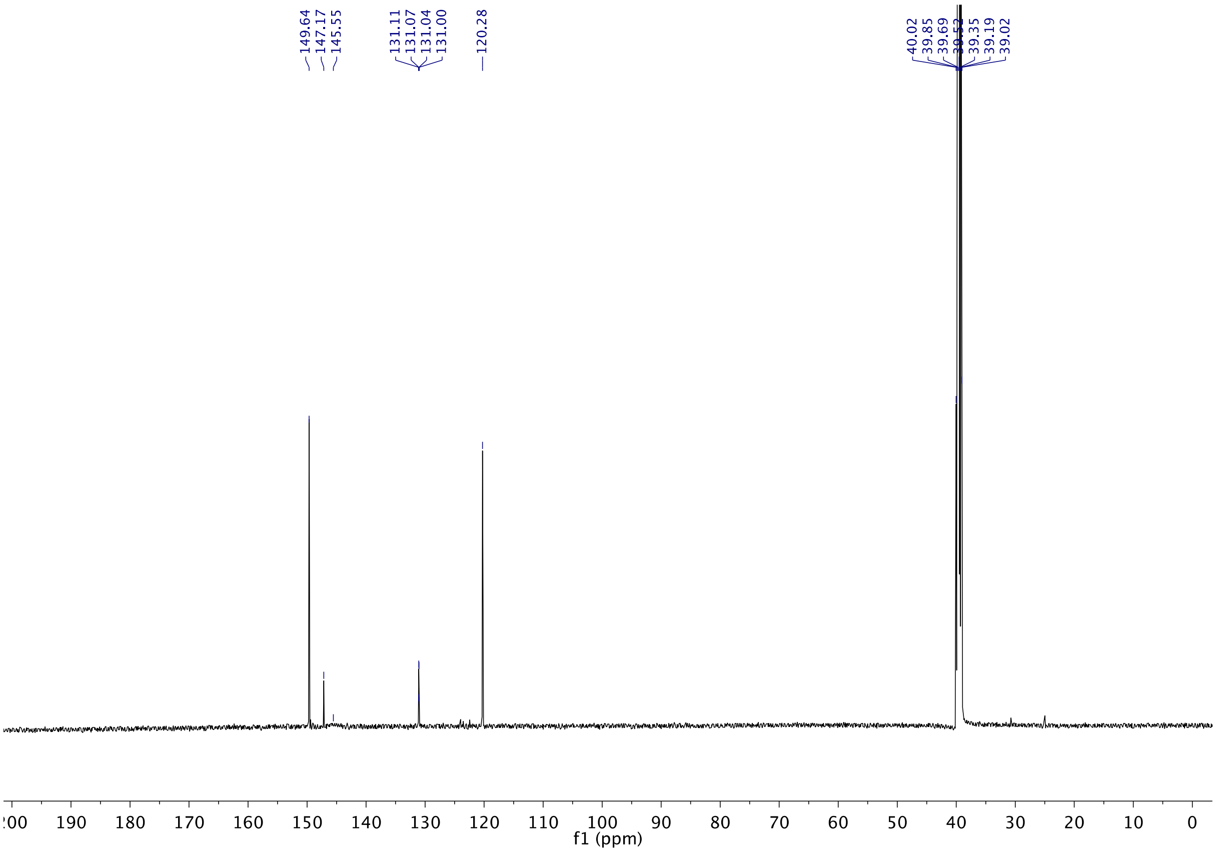
149.64
147.17
145.55
131.11
131.07
131.04
131.00
120.28
40.02
39.85
39.69
39.52
39.35
39.19
39.02
200
190
180
170
160
150
140
130
120
110
100
90
80
70
60
50
40
30
20
10
0
f1 (ppm)

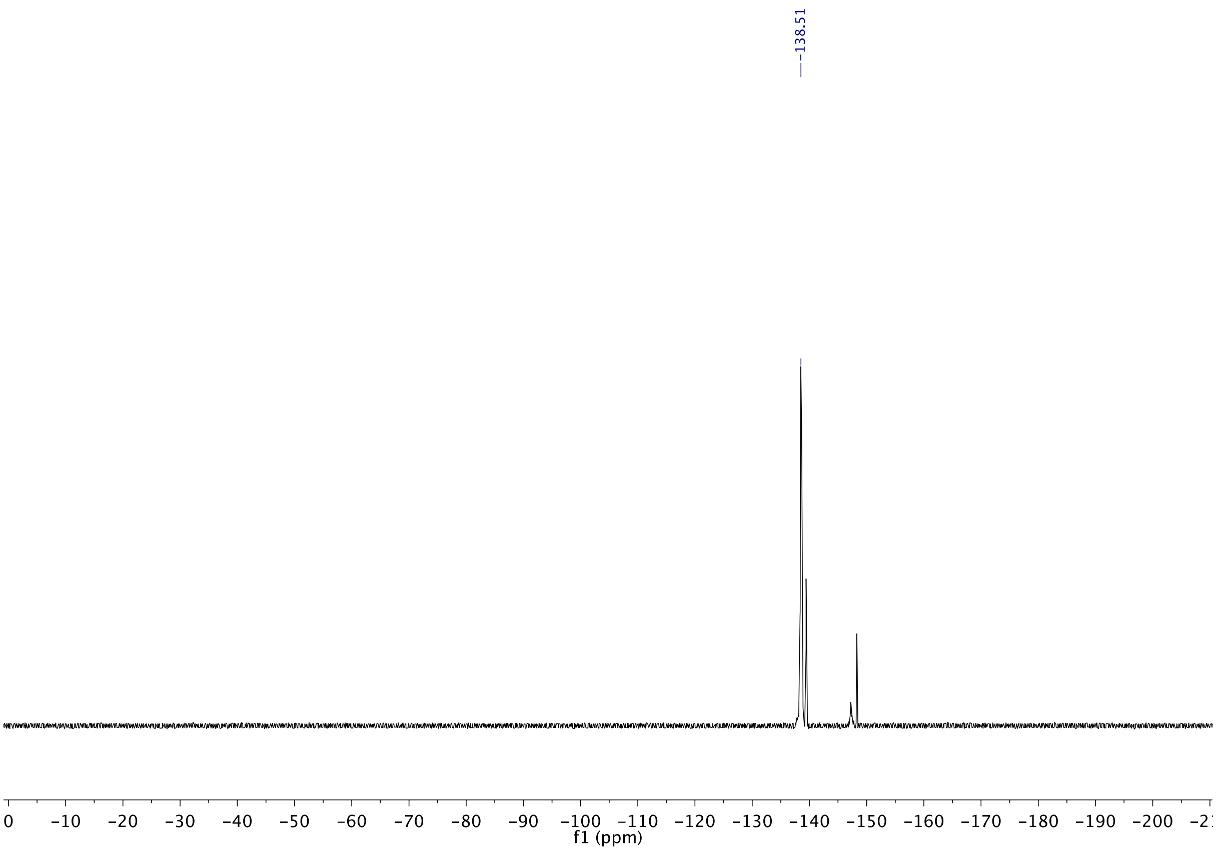

(*E*)-(2-(Pyridin-4-yl)vinyl)boronic acid, **S17**

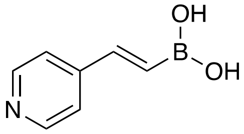

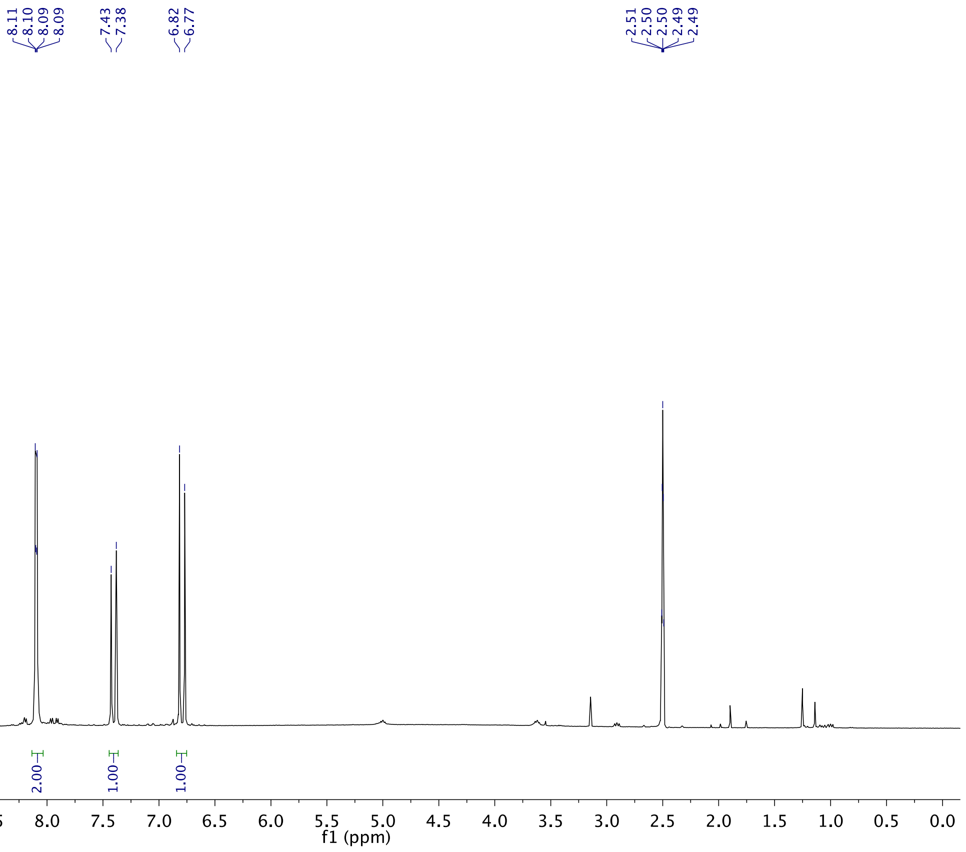

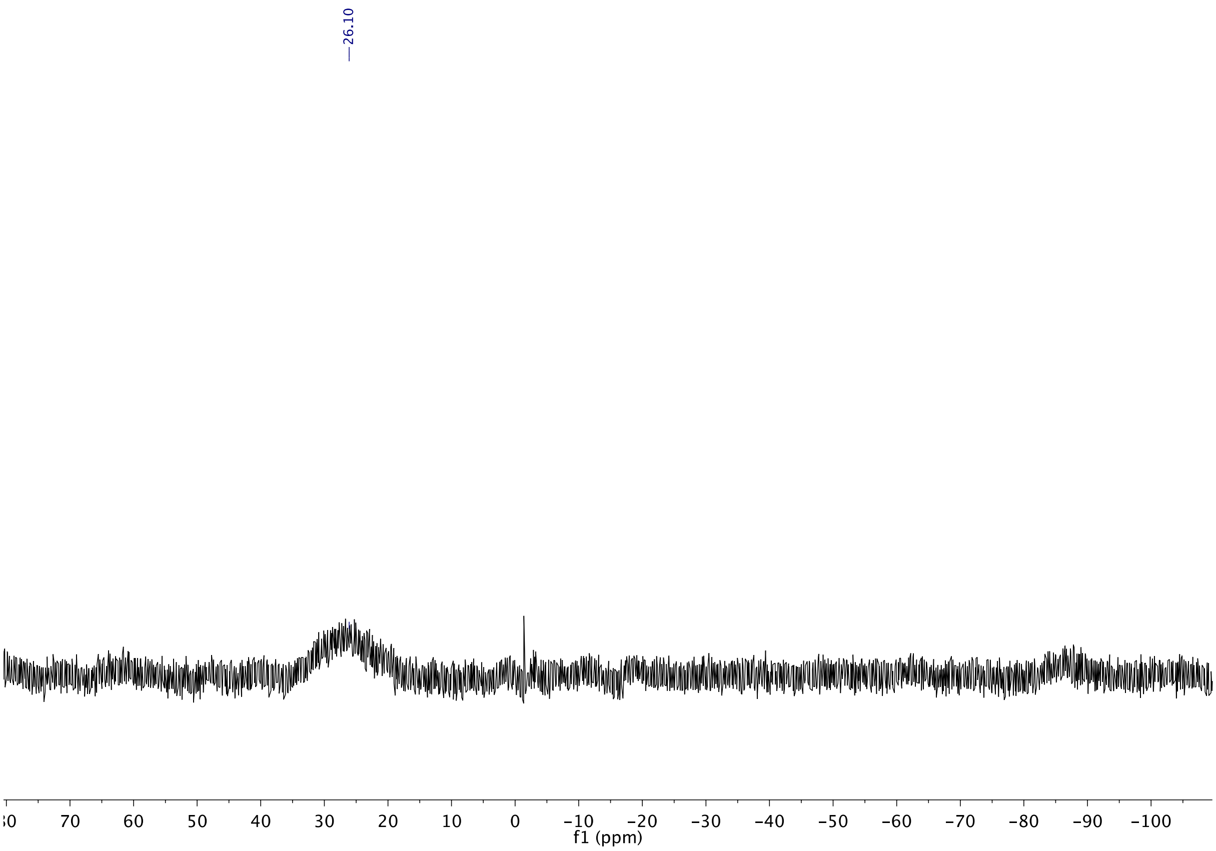
26.10
f1 (ppm)

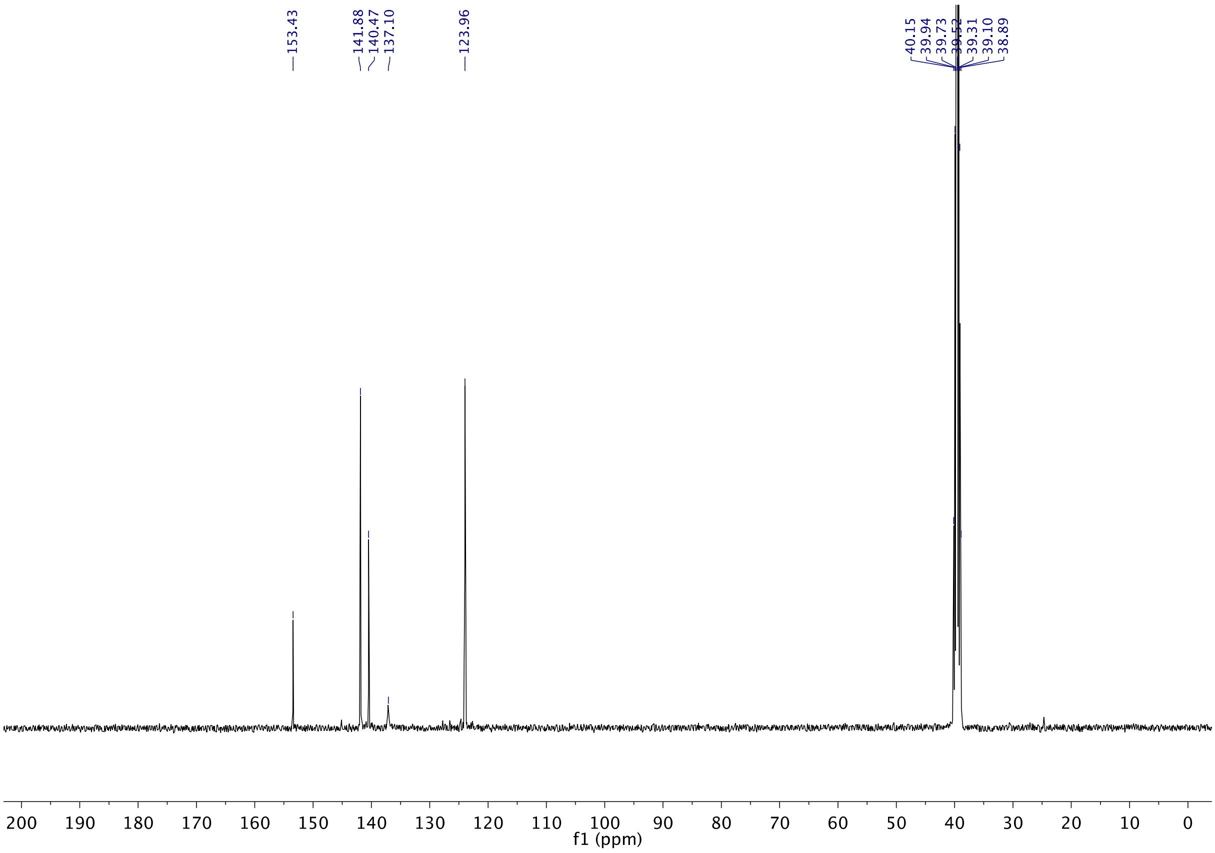
153.43
141.88
140.47
137.10
123.96
40.15
39.94
39.73
39.52
39.31
39.10
38.89
f1 (ppm)

5-((Trimethylsilyl)ethynyl)pyrimidine, **S18-int1**

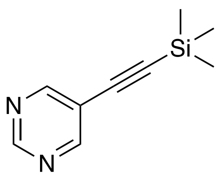

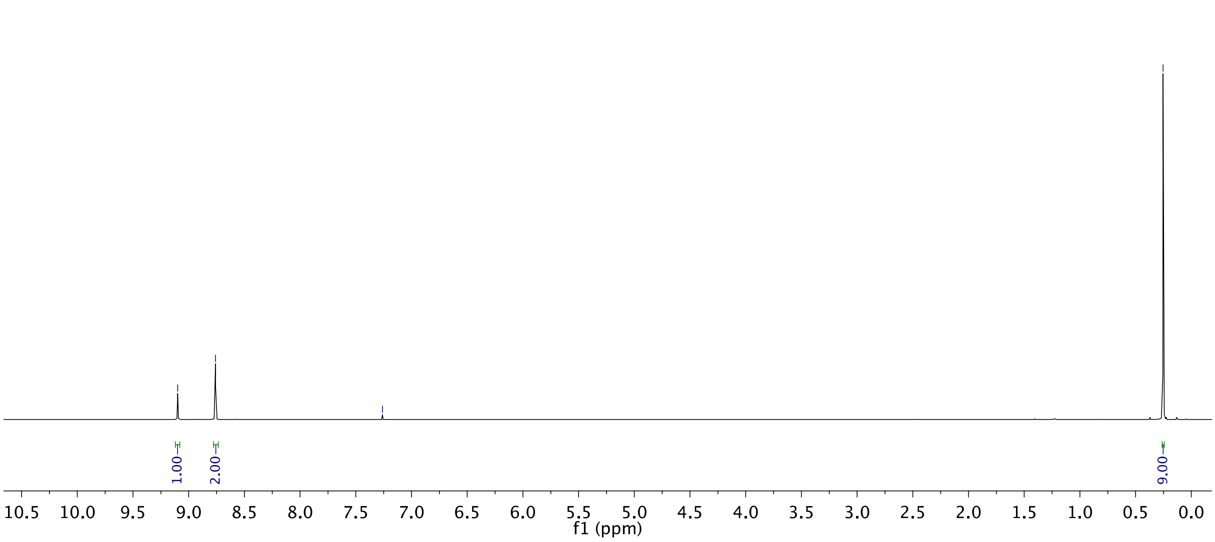

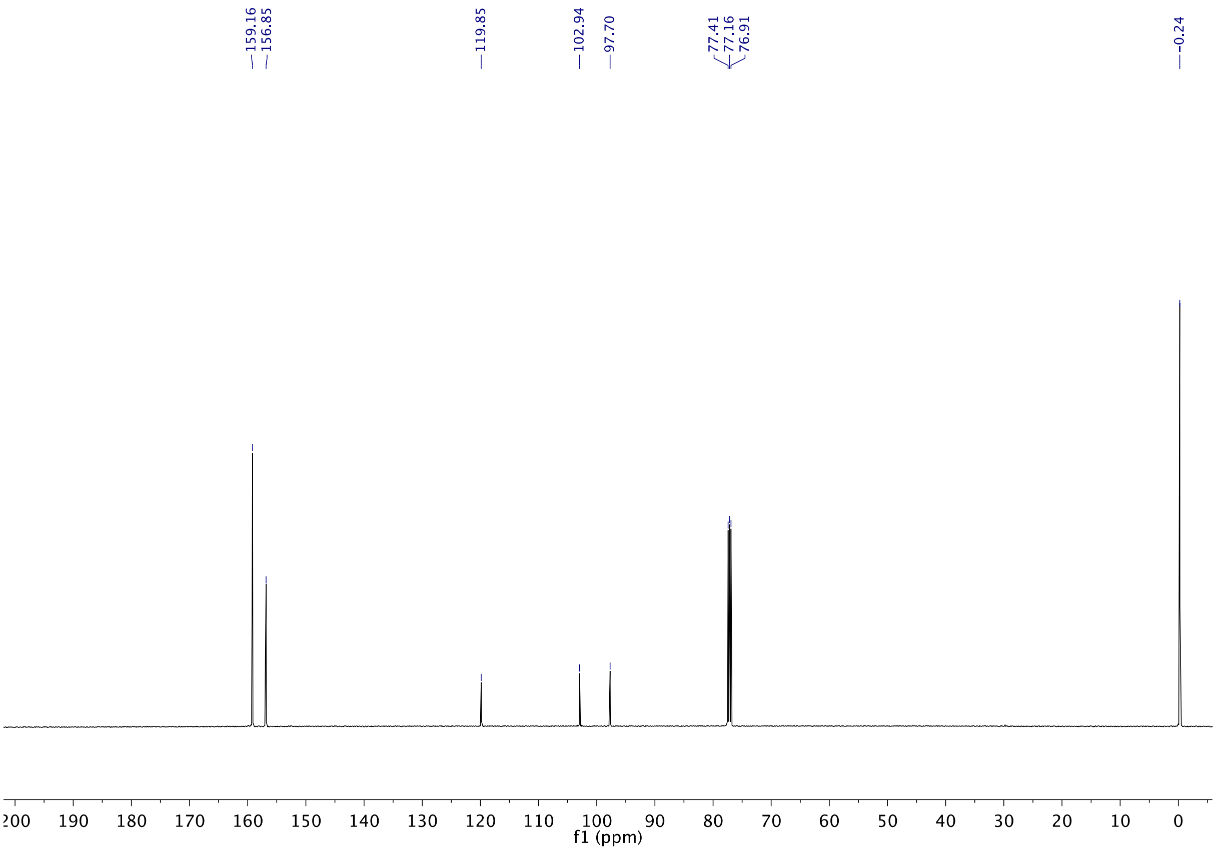

5-Ethynylpyrimidine, **S18-int2**

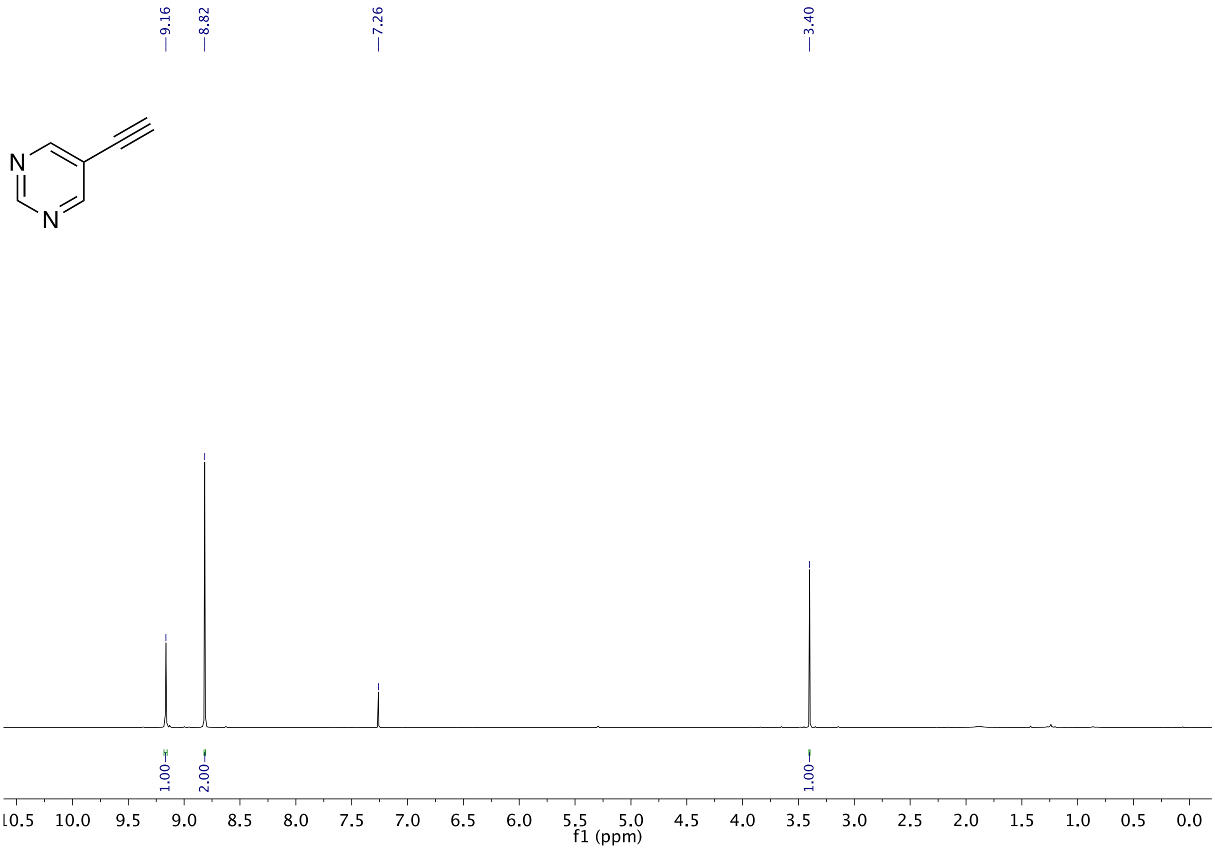

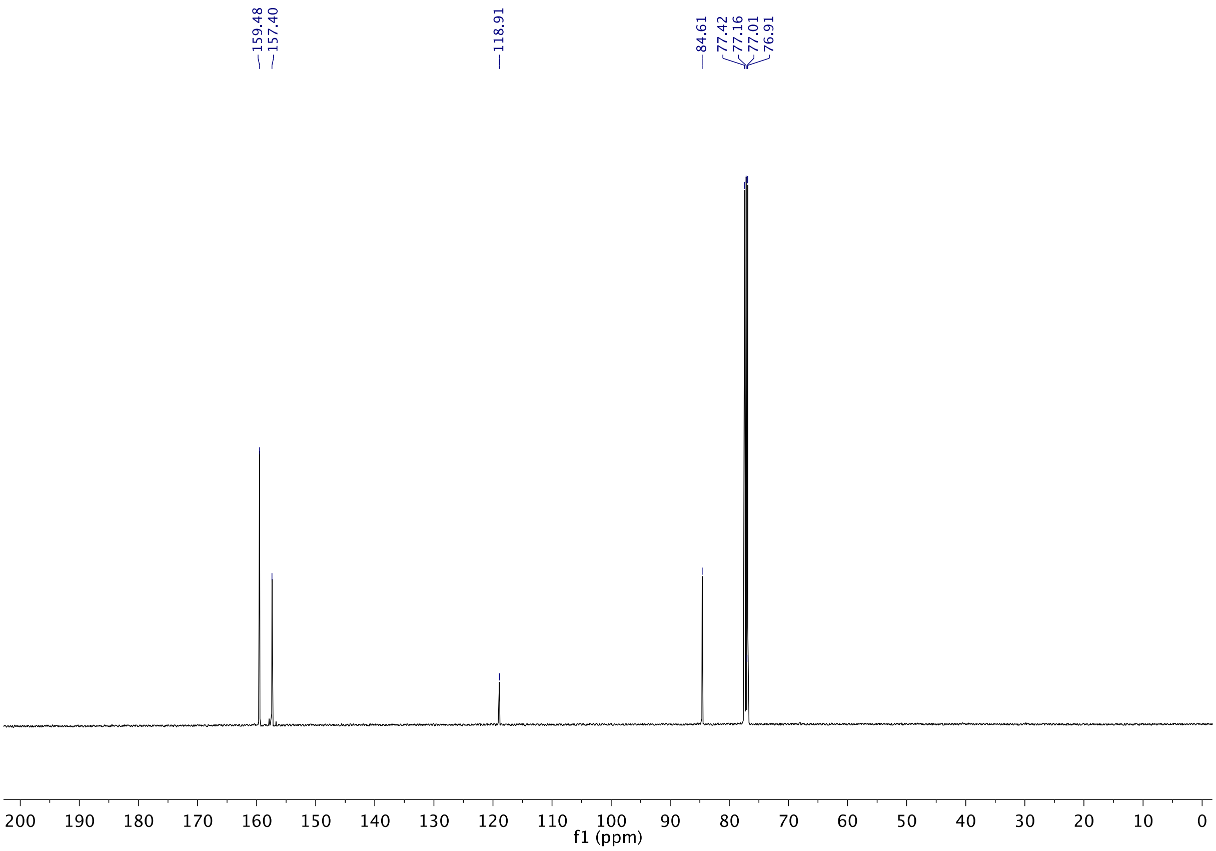

(*E*)-5-(2-(4,4,5,5-Tetramethyl-1,3,2-dioxaborolan-2-yl)vinyl)pyrimidine, **S18-int3**

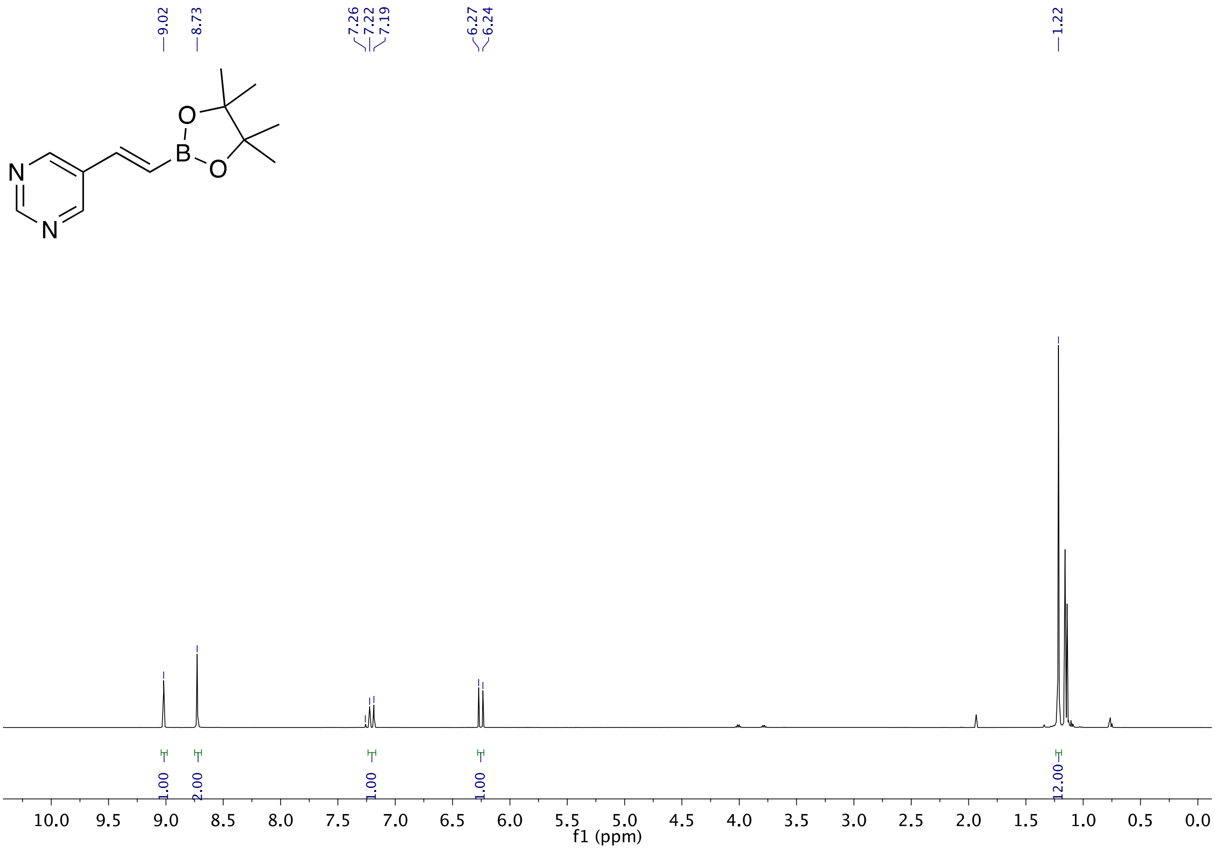

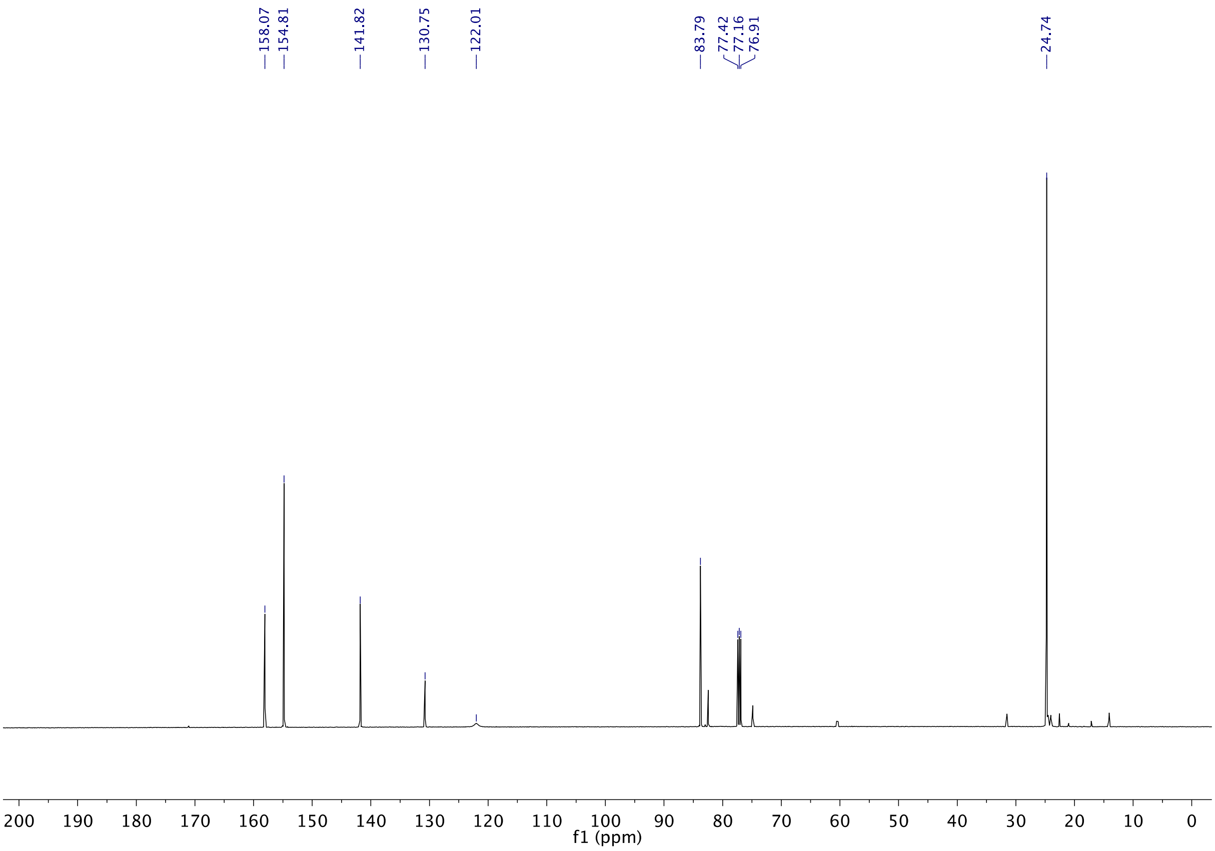

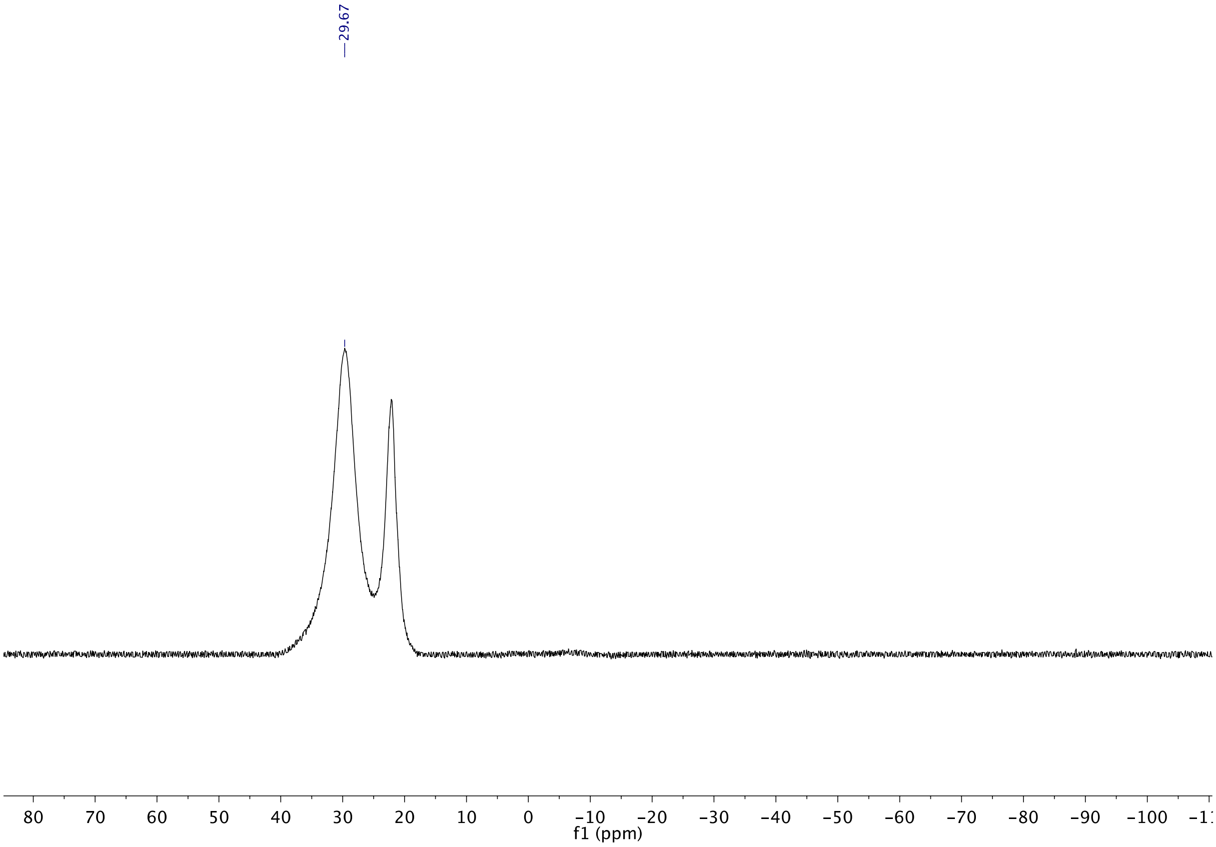

(*E*)-5-(2-(trifluoro-λ[4]-boraneyl)vinyl)pyrimidine, potassium salt, **S18-int4**

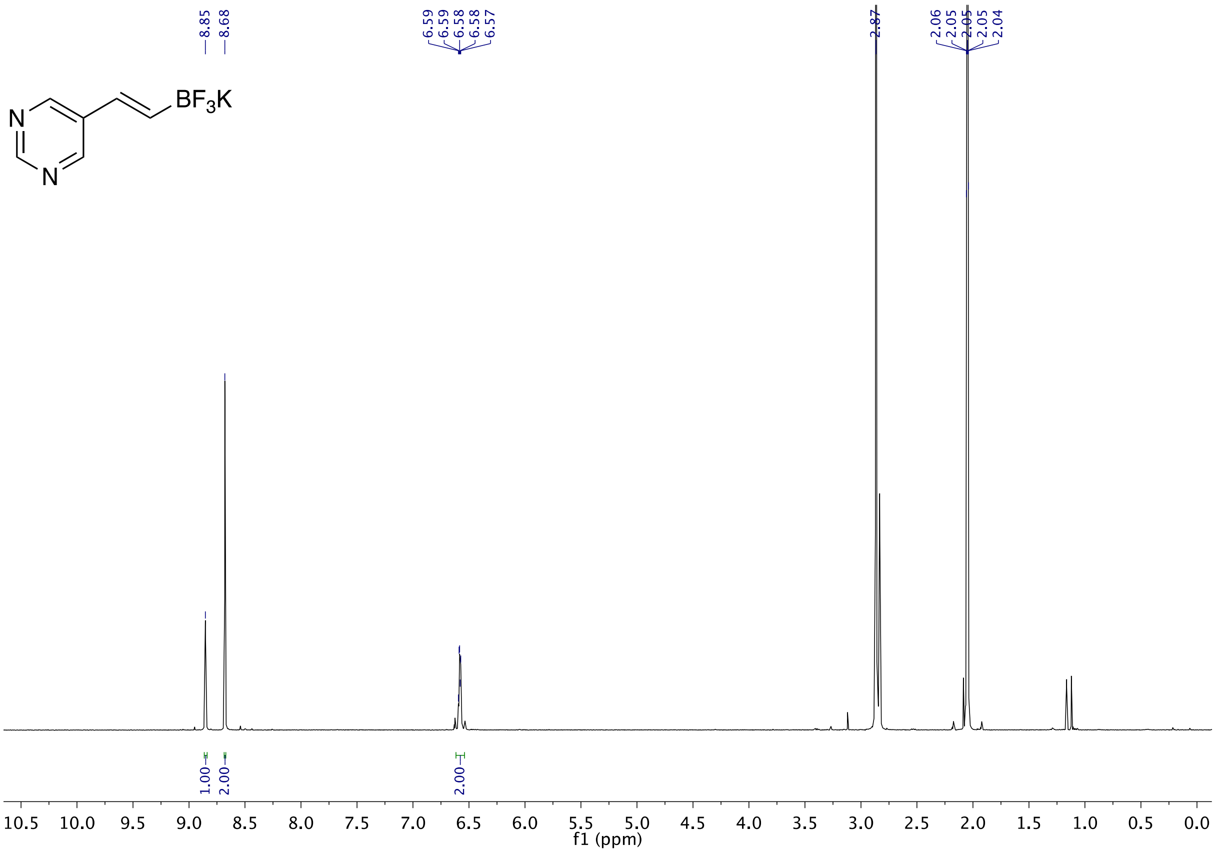

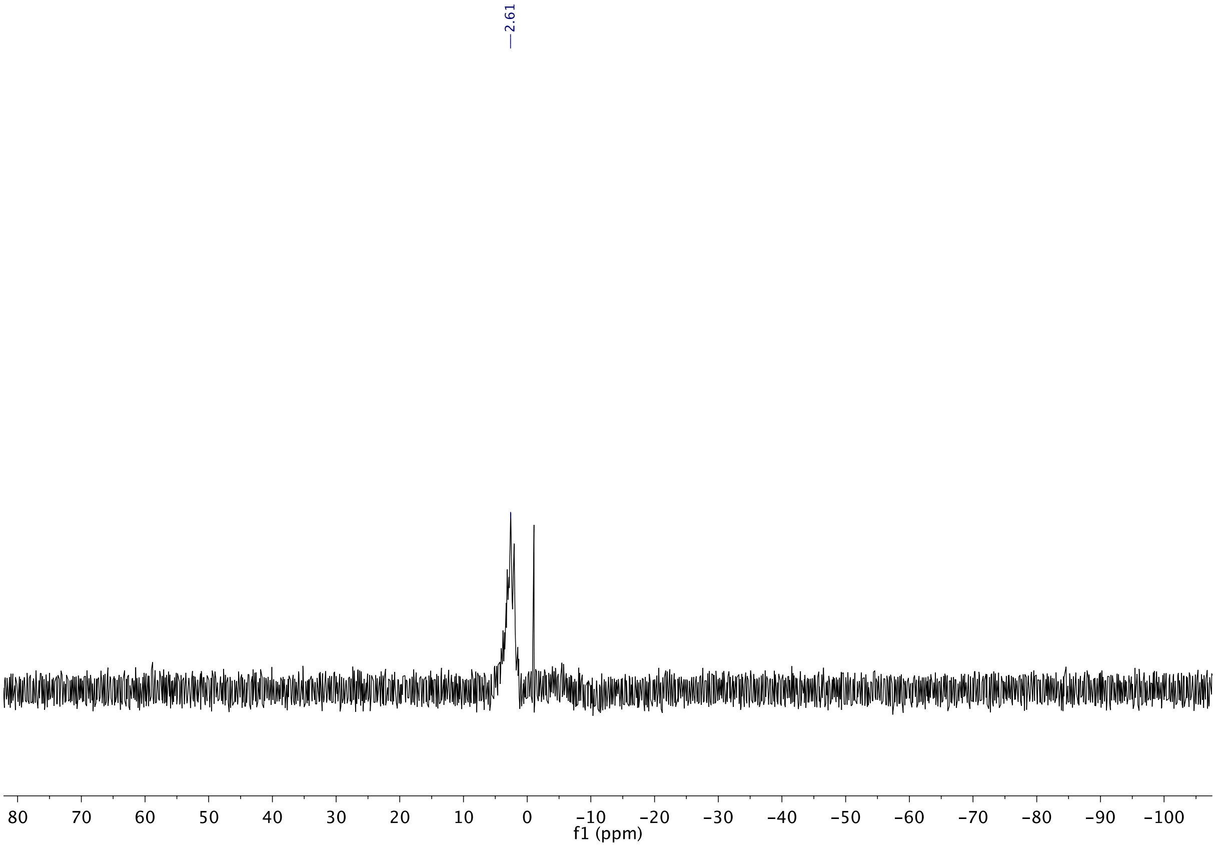
2.61
f1 (ppm)

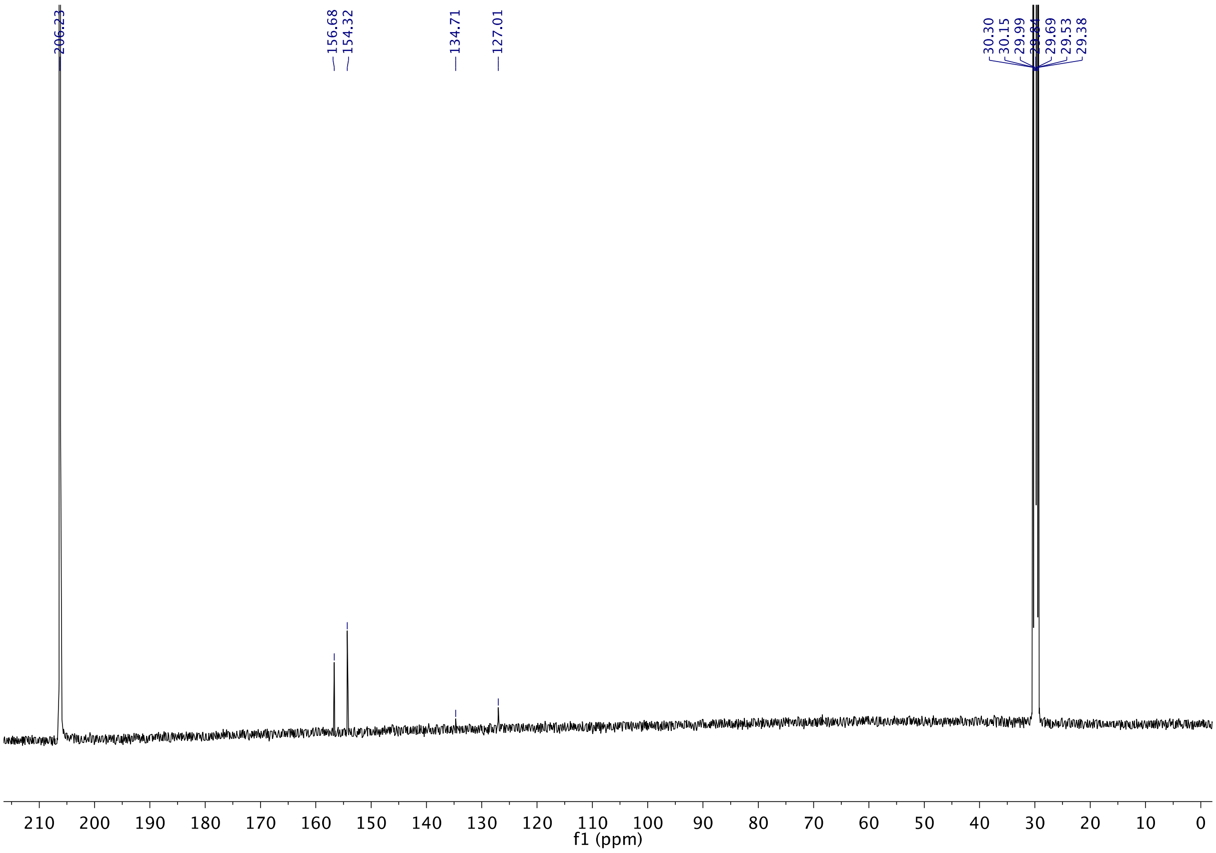
206.23
156.68
154.32
134.71
127.01
30.30
30.15
29.99
29.84
29.69
29.53
29.38
f1 (ppm)

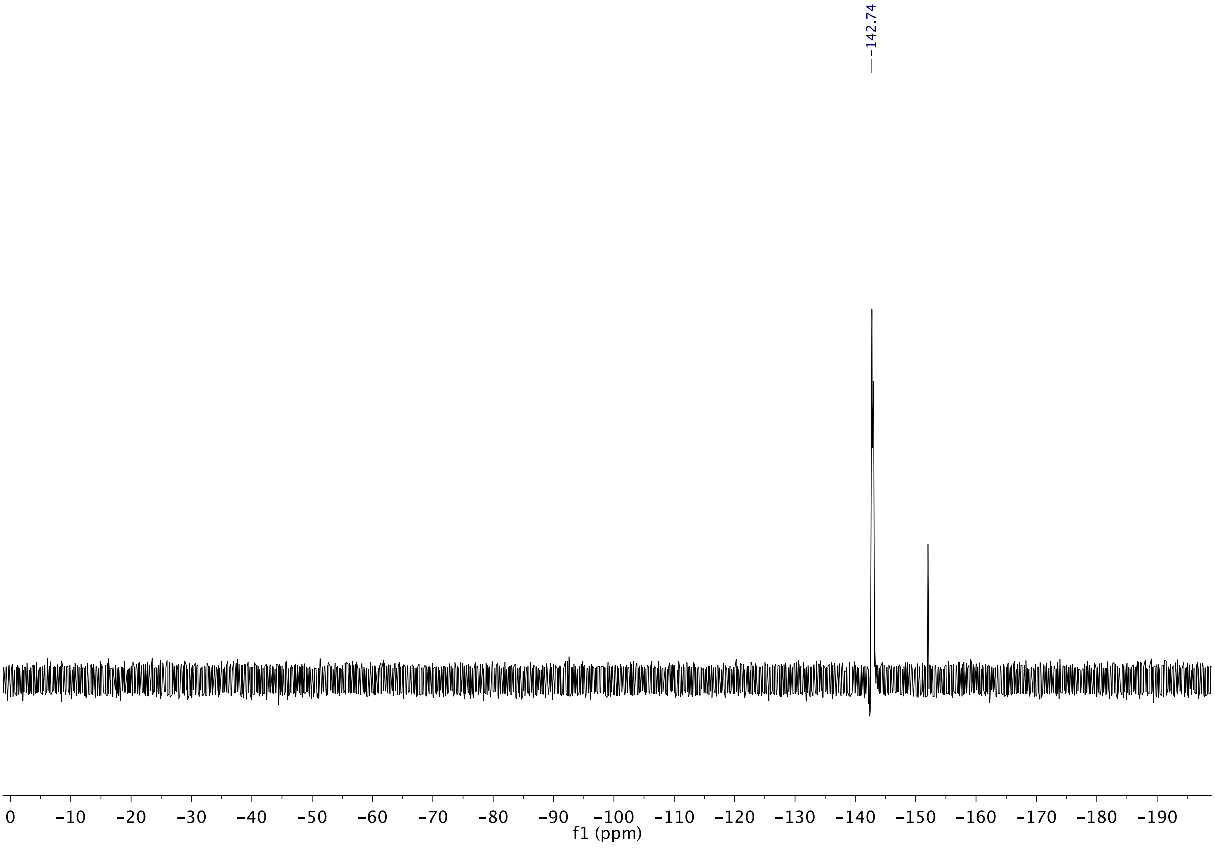

(*E*)-(2-(Pyrimidin-5-yl)vinyl)boronic acid, **S18**

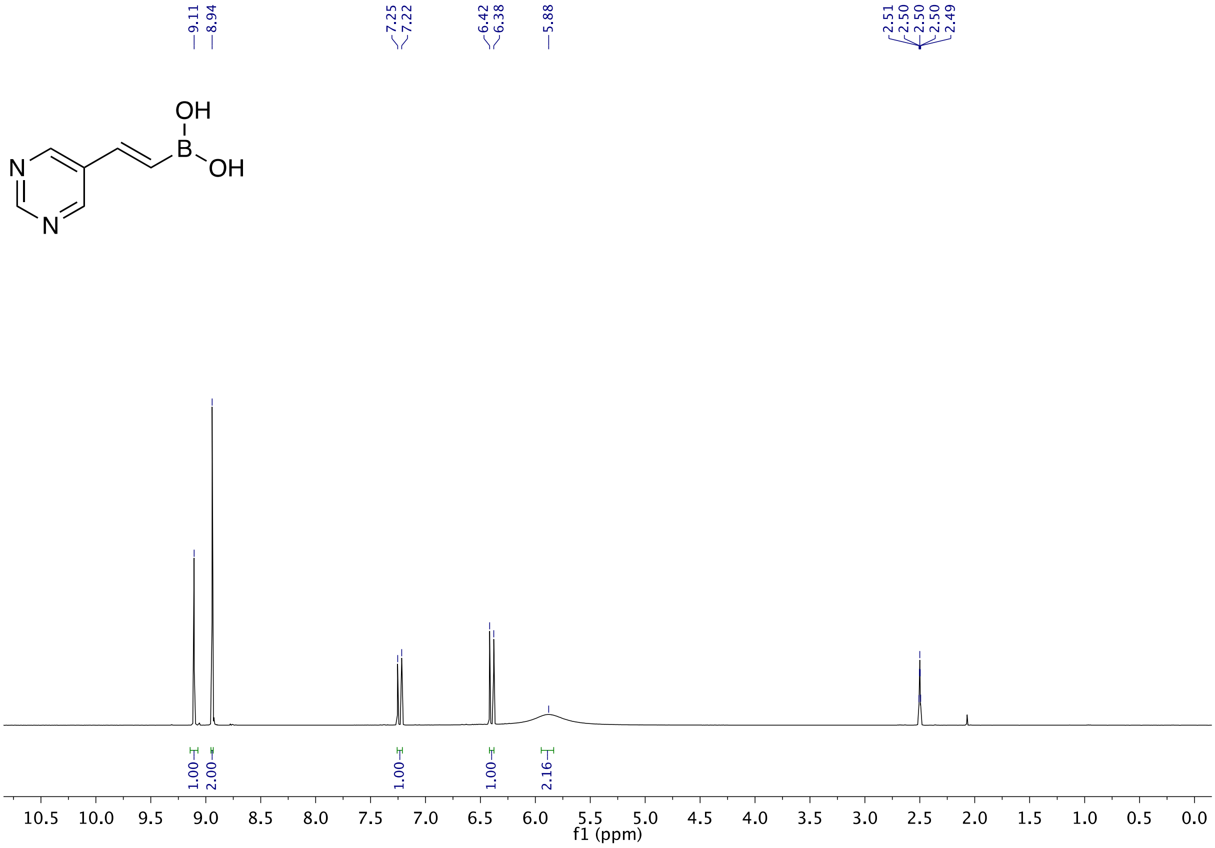

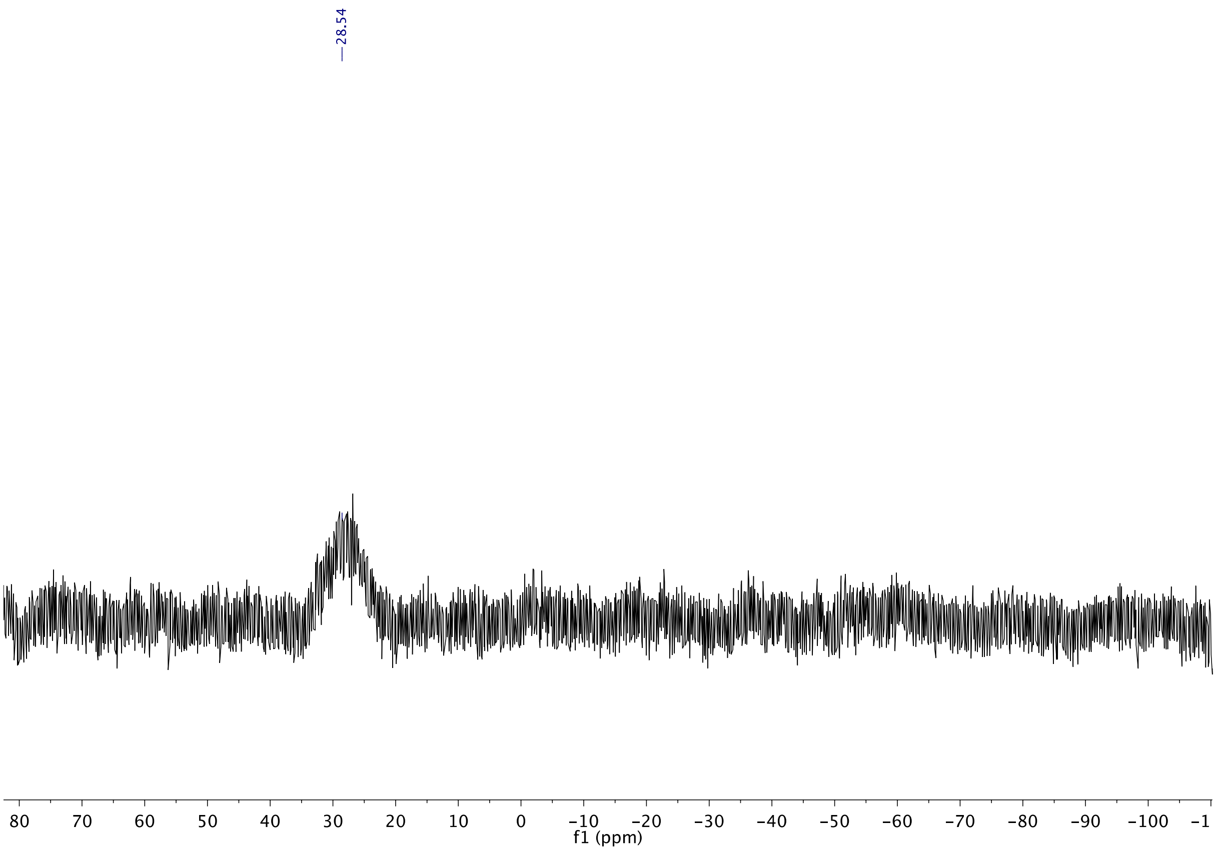
28.54
80
70
60
50
40
30
20
10
0
−10
−20
−30
−40
−50
−60
−70
−80
−90
−100
−1
f1 (ppm)

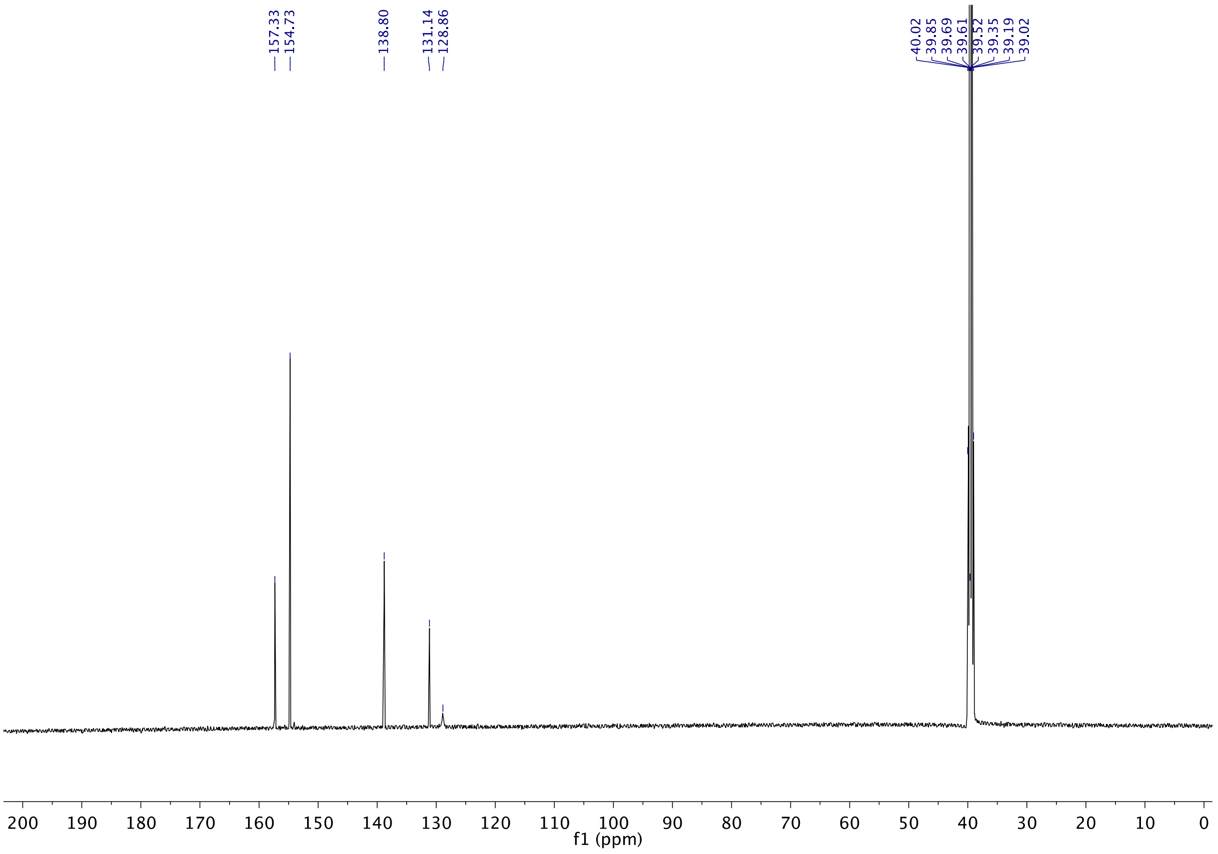
157.33
154.73
138.80
131.14
128.86
40.02
39.85
39.69
39.61
39.52
39.35
39.19
39.02
200
190
180
170
160
150
140
130
120
110
100
90
80
70
60
50
40
30
20
10
0
f1 (ppm)

(*E*)-4,4,5,5-Tetramethyl-2-styryl-1,3,2-dioxaborolane, **S19-int3**

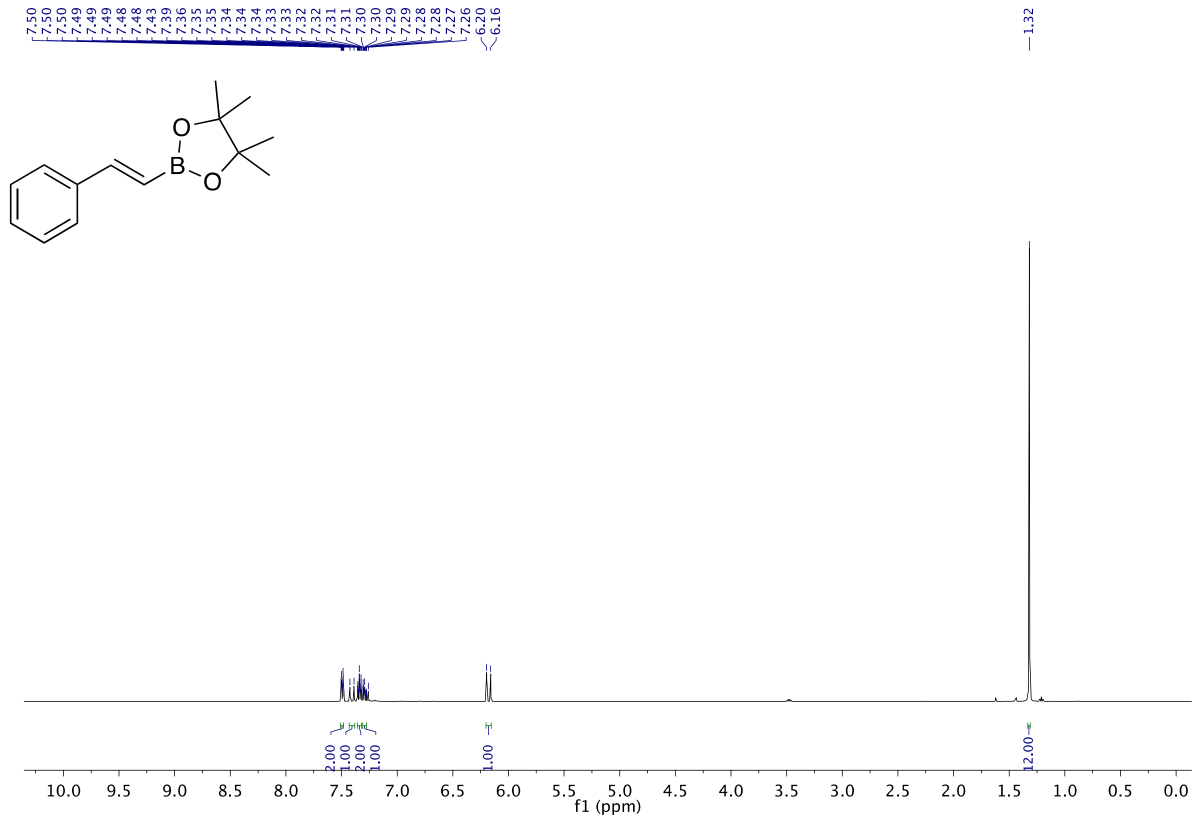

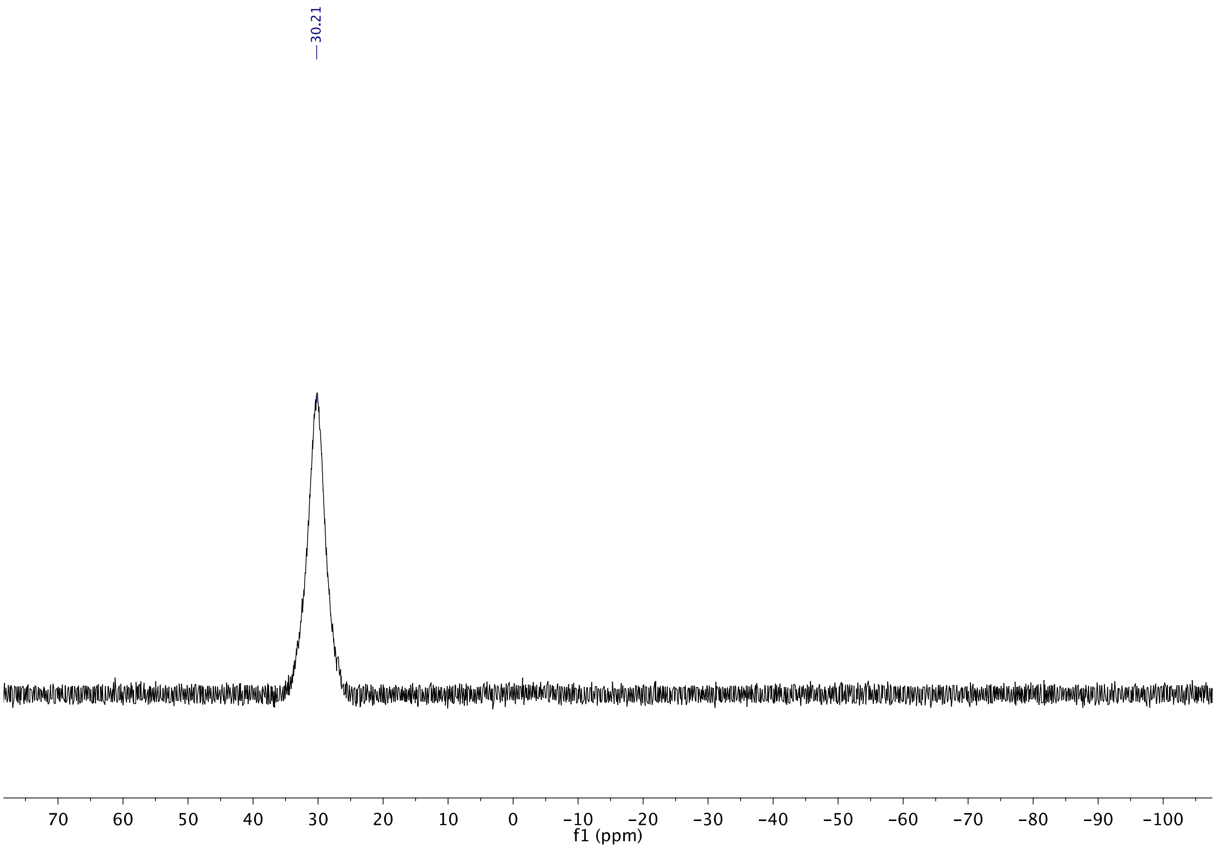

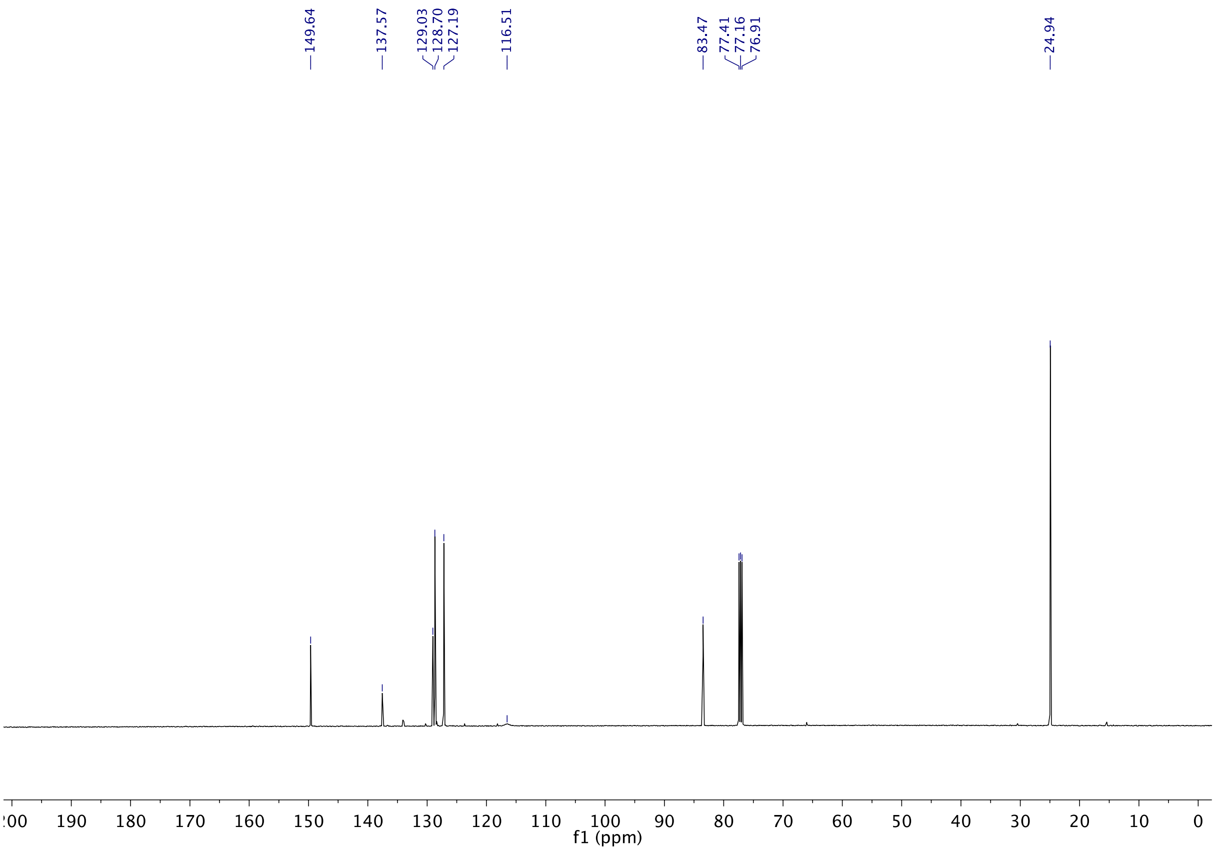

(*Z*)-4,4,5,5-Tetramethyl-2-styryl-1,3,2-dioxaborolane, **S19-int3E:Z**

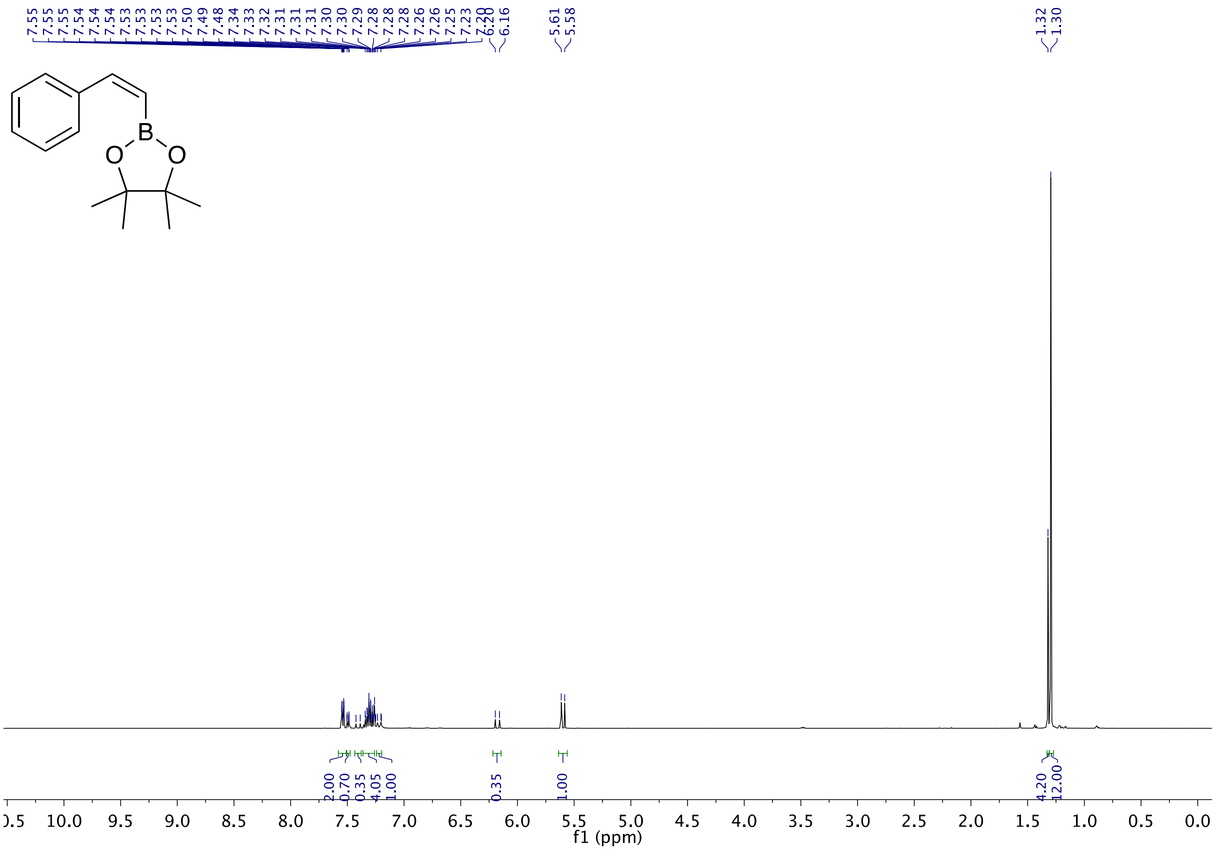

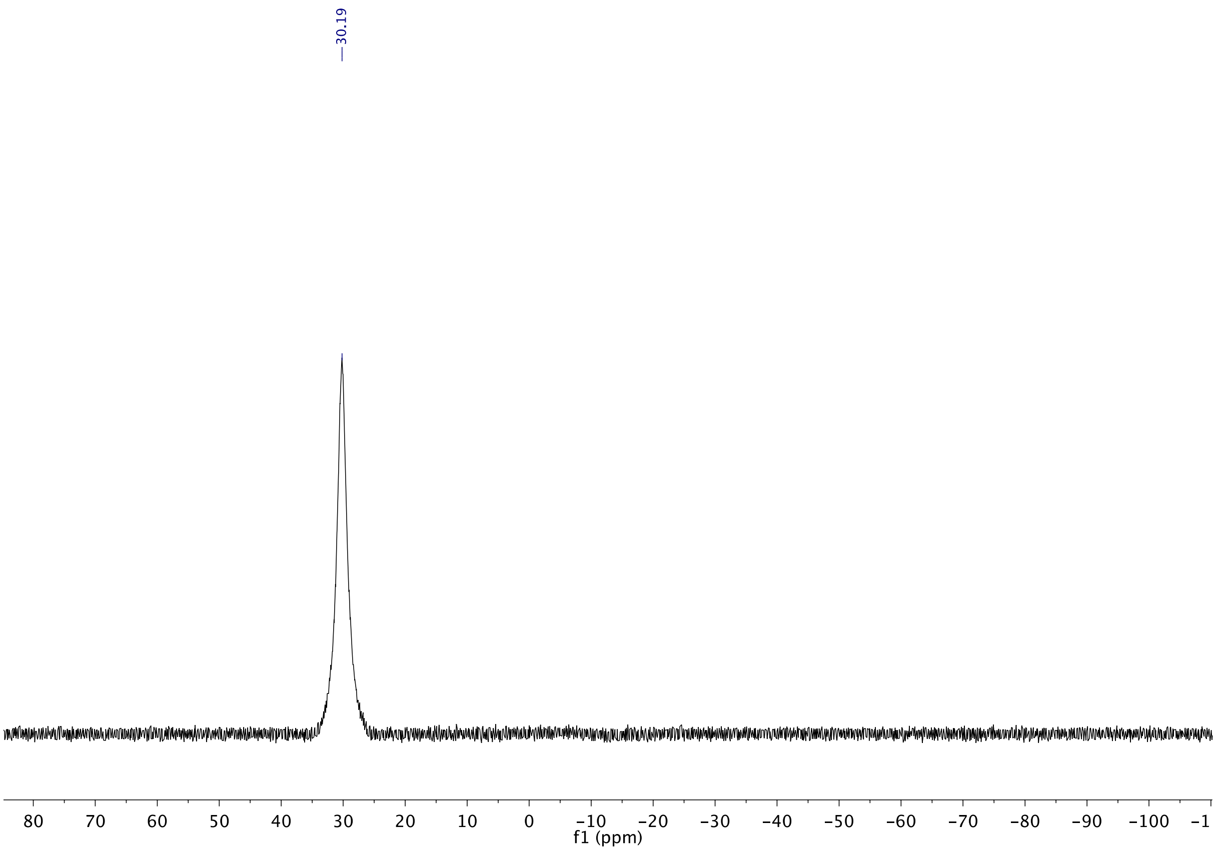
30.19
80 70 60 50 40 30 20 10 0 −10 −20 −30 −40 −50 −60 −70 −80 −90 −100
f1 (ppm)

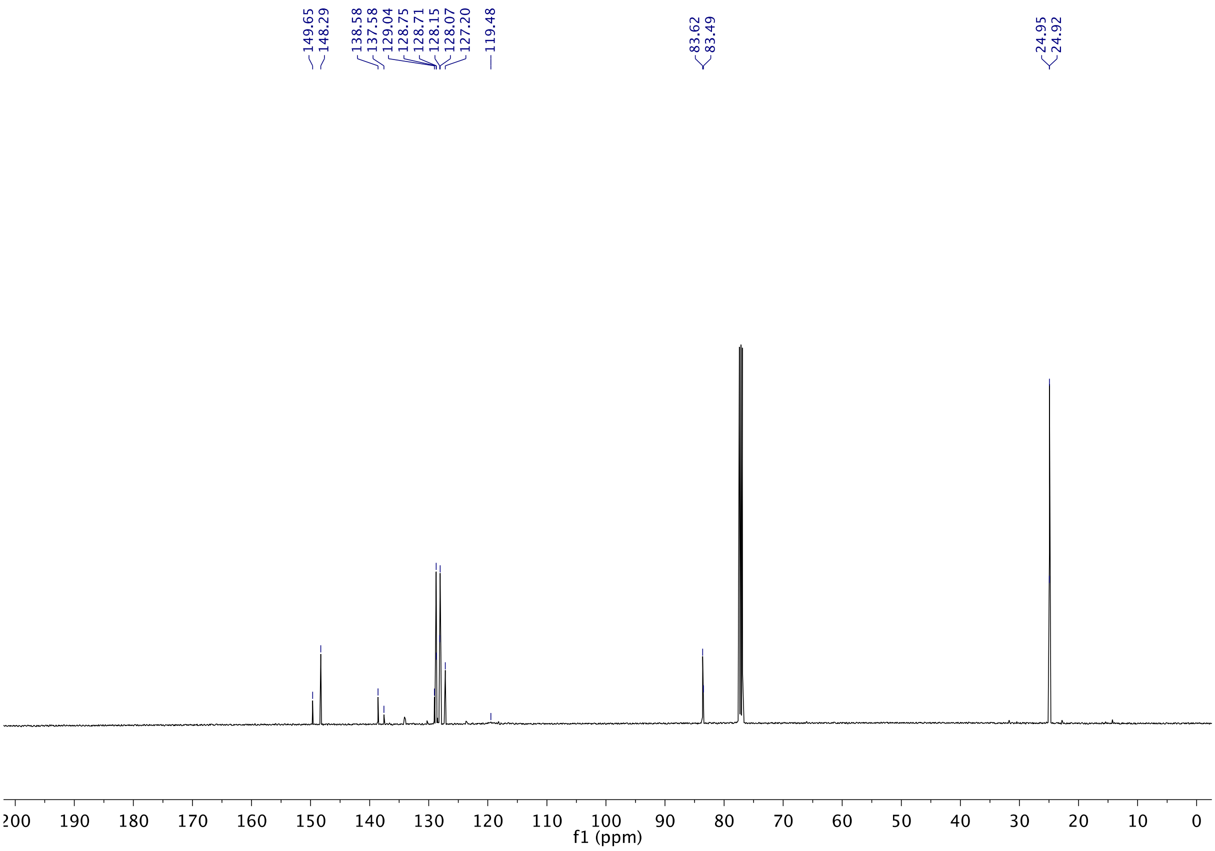
149.65
148.29
138.58
137.58
129.04
128.75
128.71
128.15
128.07
127.20
119.48
83.62
83.49
24.95
24.92
190 180 170 160 150 140 130 120 110 100 90 80 70 60 50 40 30 20 10 0
f1 (ppm)

(*E*) and (*Z*)-trifluoro(styryl)-λ[4]-borane, potassium salt, **S19-int4**

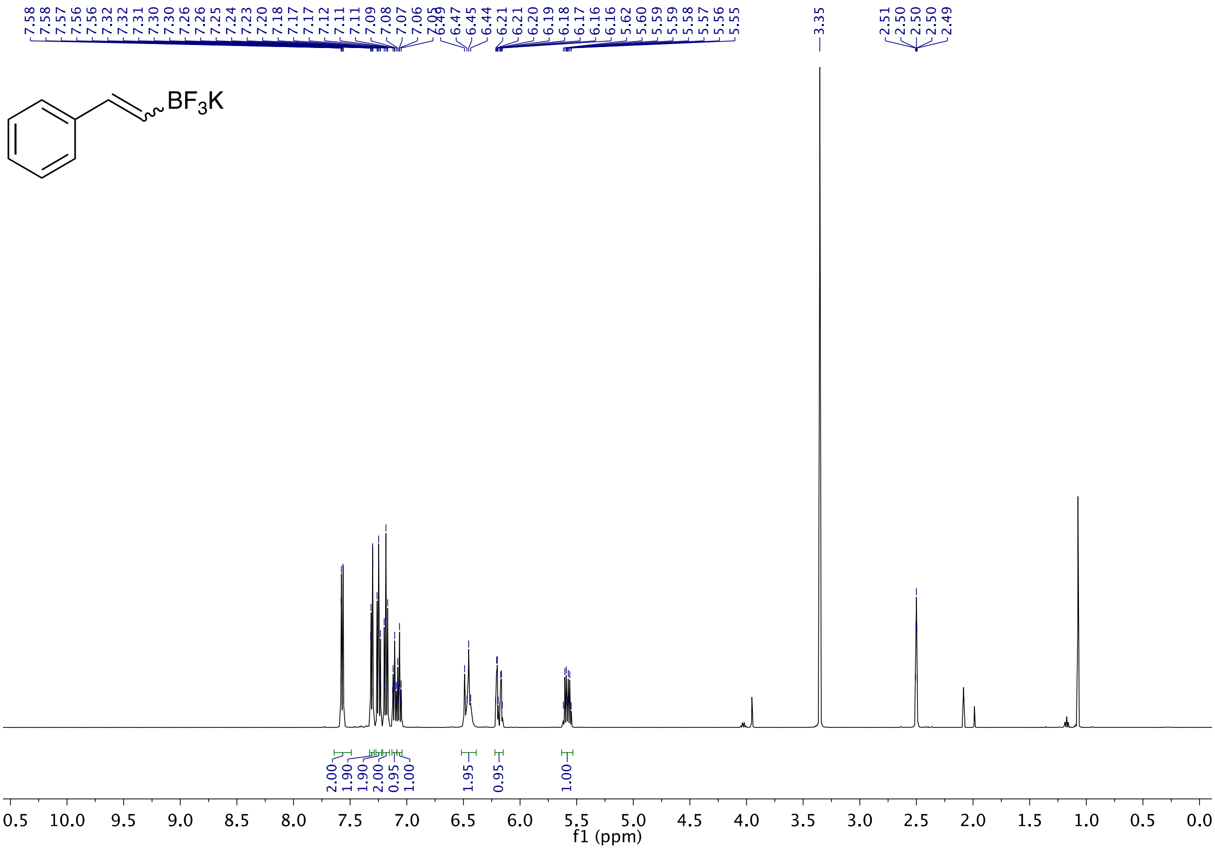

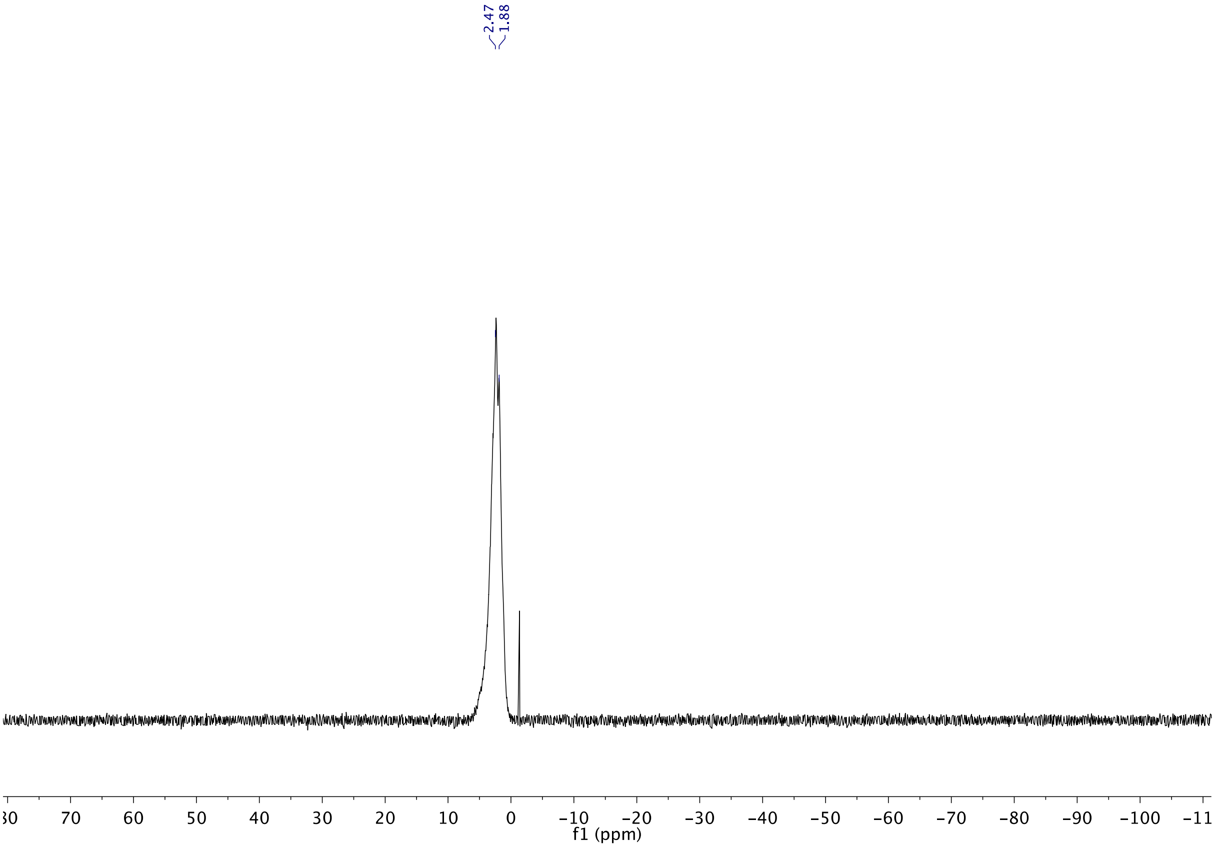

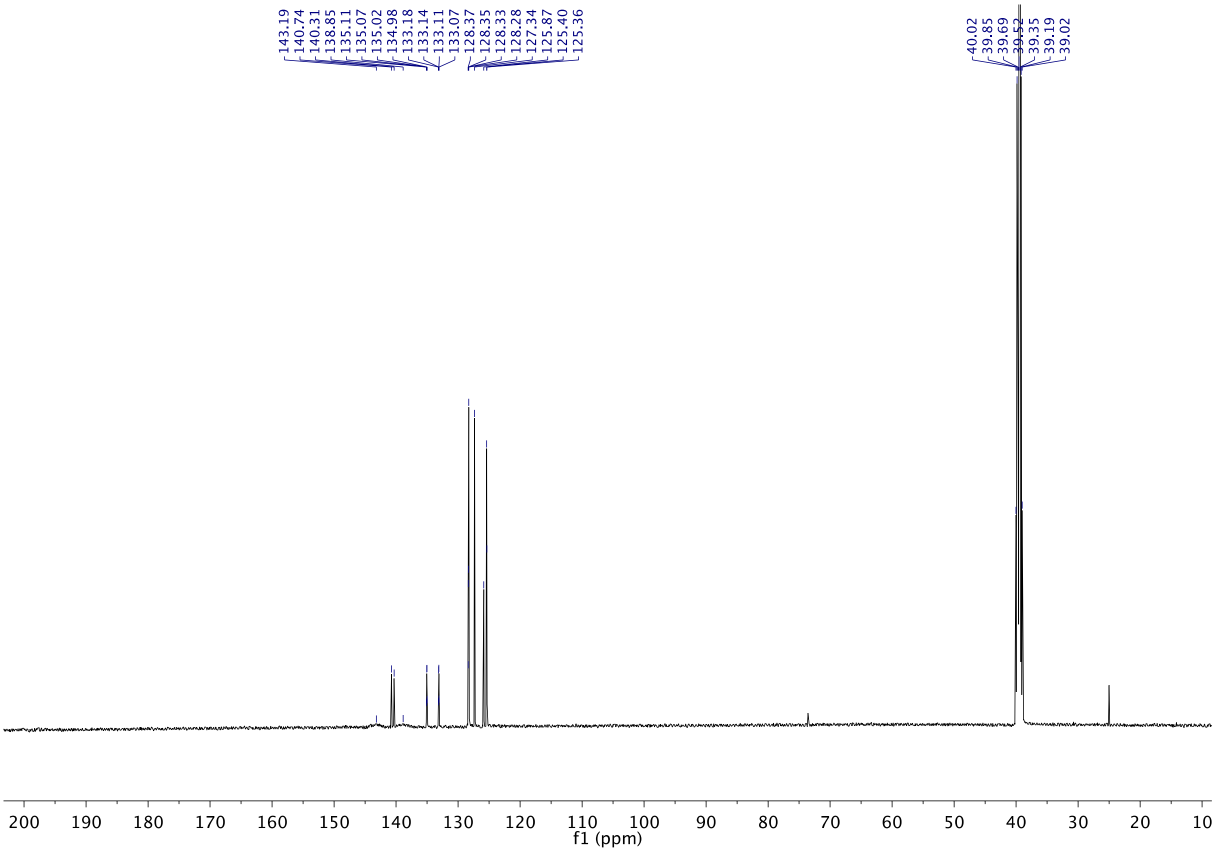
143.19
140.74
140.31
138.85
135.11
135.07
135.02
134.98
133.18
133.14
133.11
133.07
128.37
128.35
128.33
128.28
127.34
125.87
125.40
125.36
40.02
39.85
39.69
39.52
39.35
39.19
39.02
200 190 180 170 160 150 140 130 120 110 100 90 80 70 60 50 40 30 20 10
f1 (ppm)

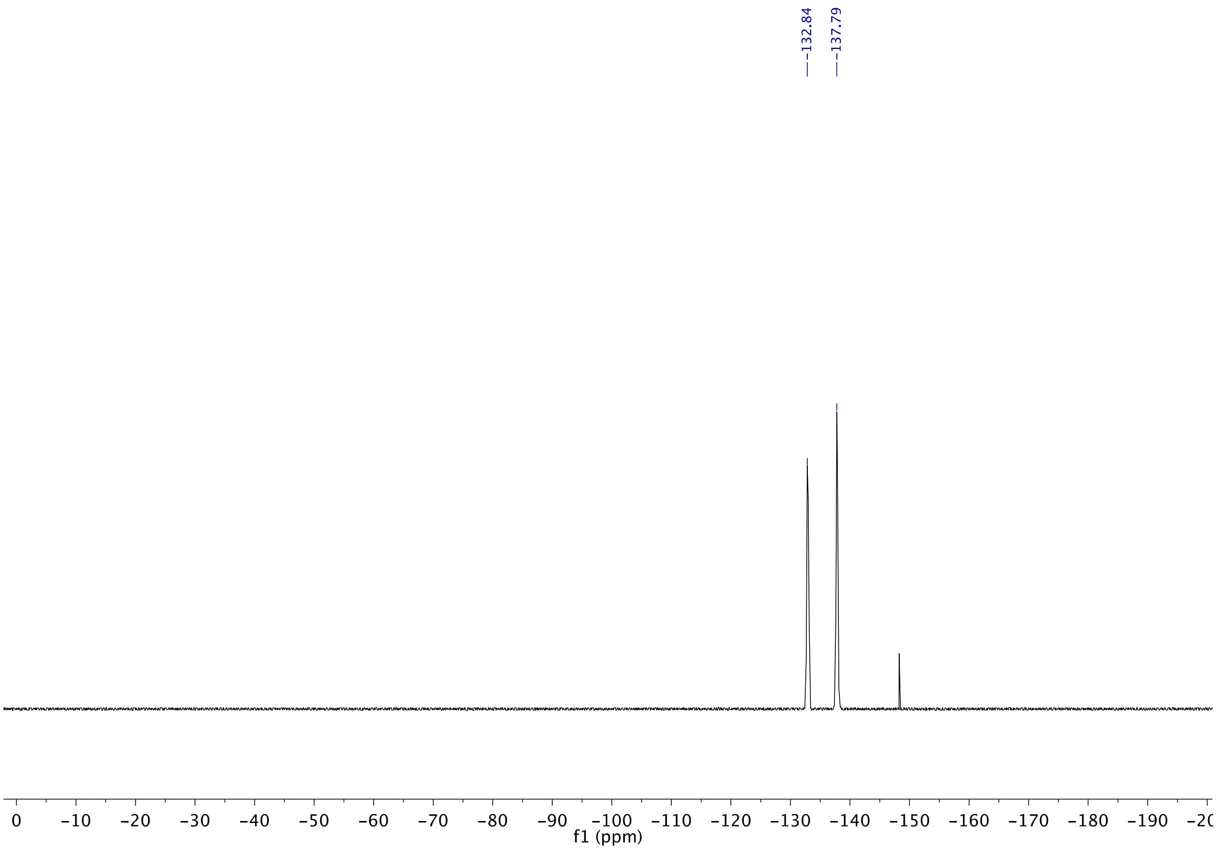
-132.84
-137.79
0 -10 -20 -30 -40 -50 -60 -70 -80 -90 -100 -110 -120 -130 -140 -150 -160 -170 -180 -190
f1 (ppm)

(*E*) and (*Z*)-styrylboronic acid, **S19**

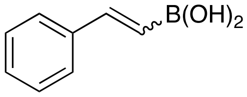

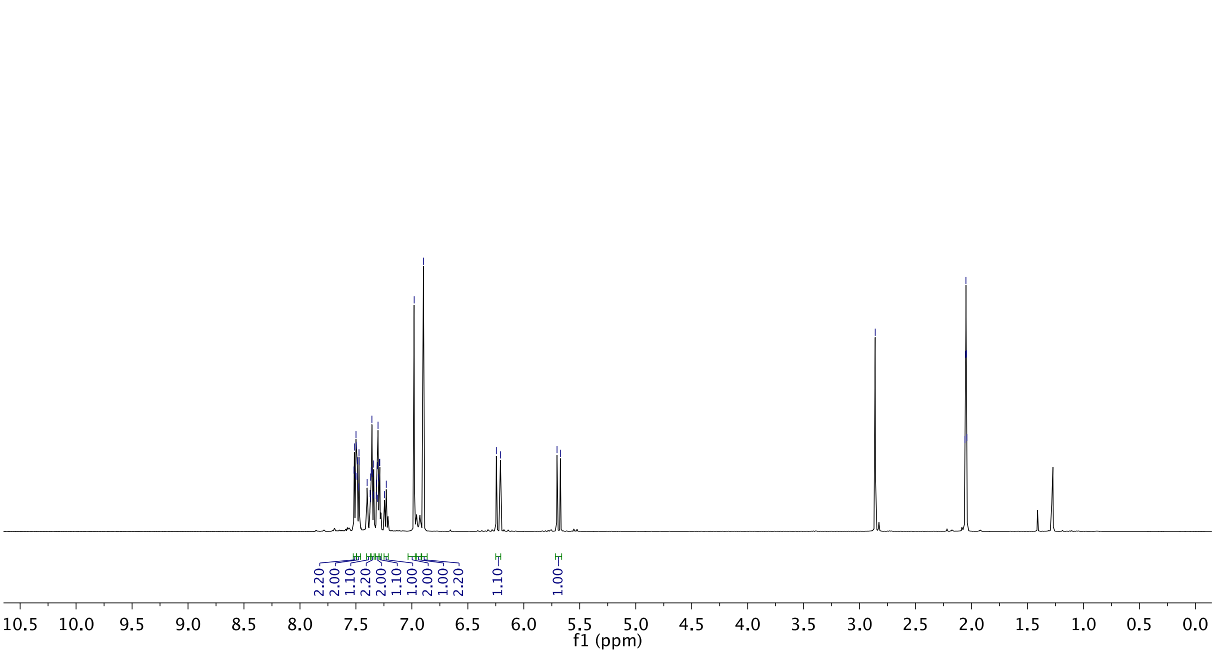

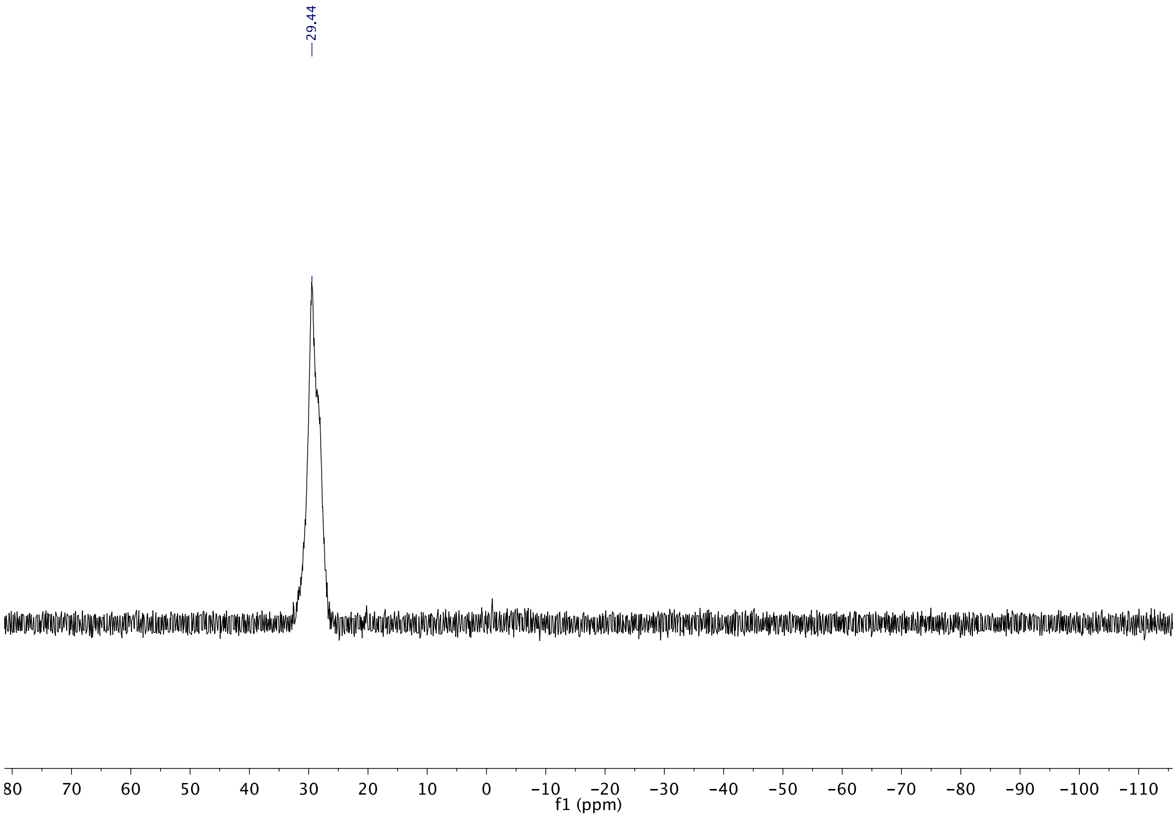

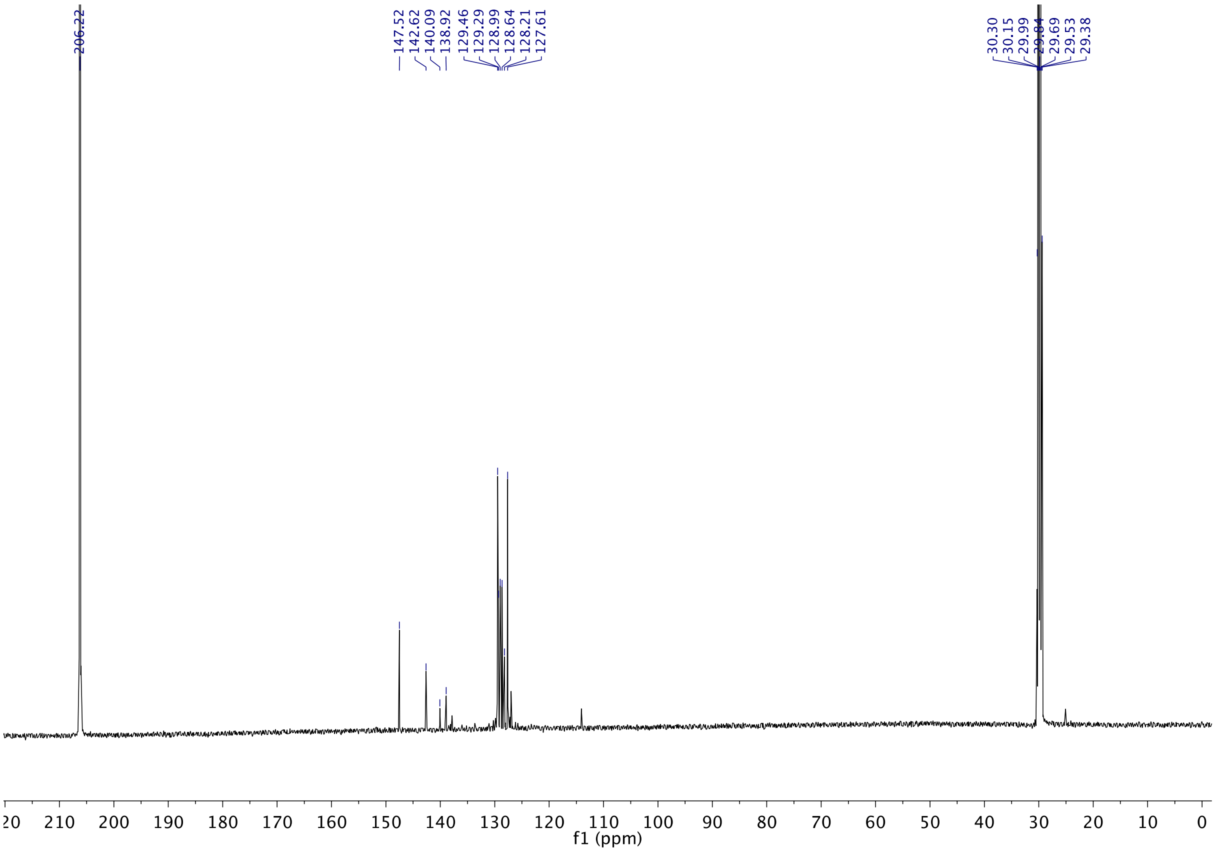

(*E*)-1-Fluoro-4-(4-phenylbut-3-en-1-yl)benzene, **3**

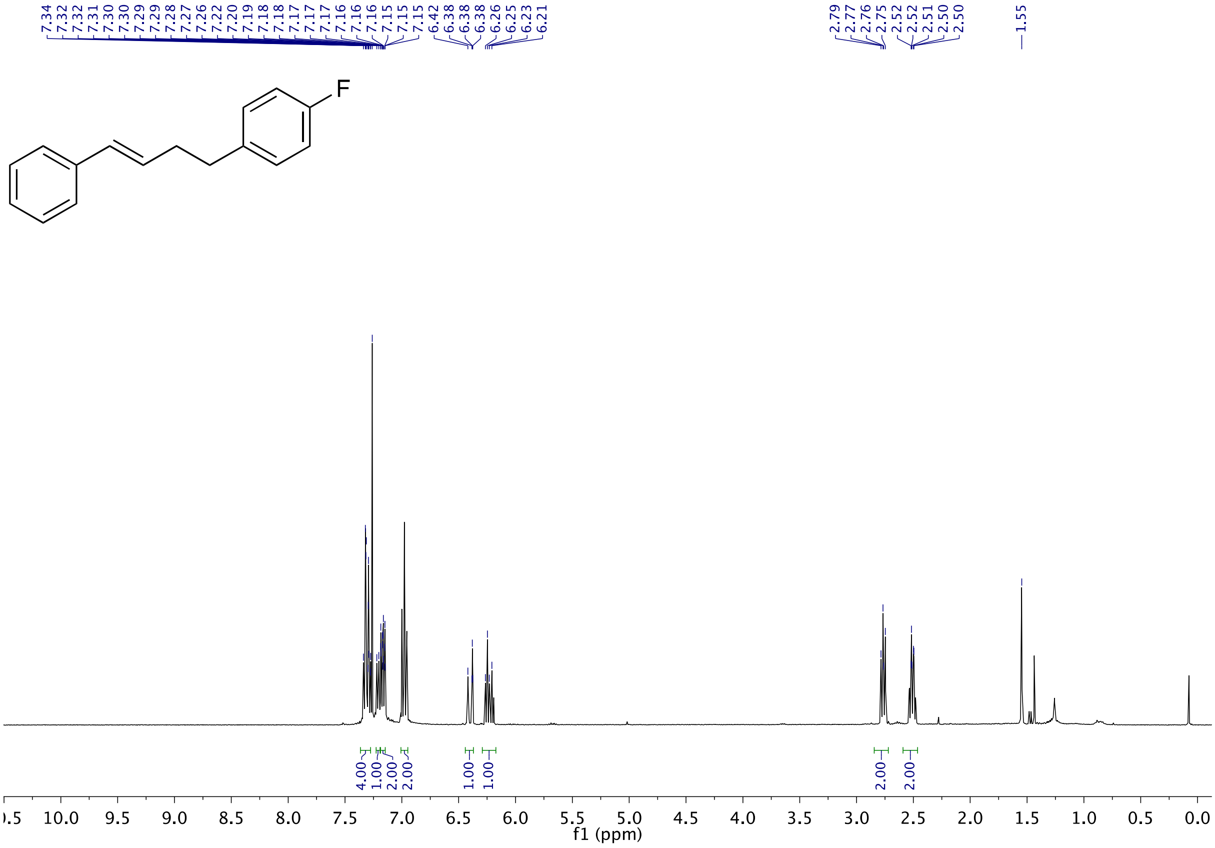

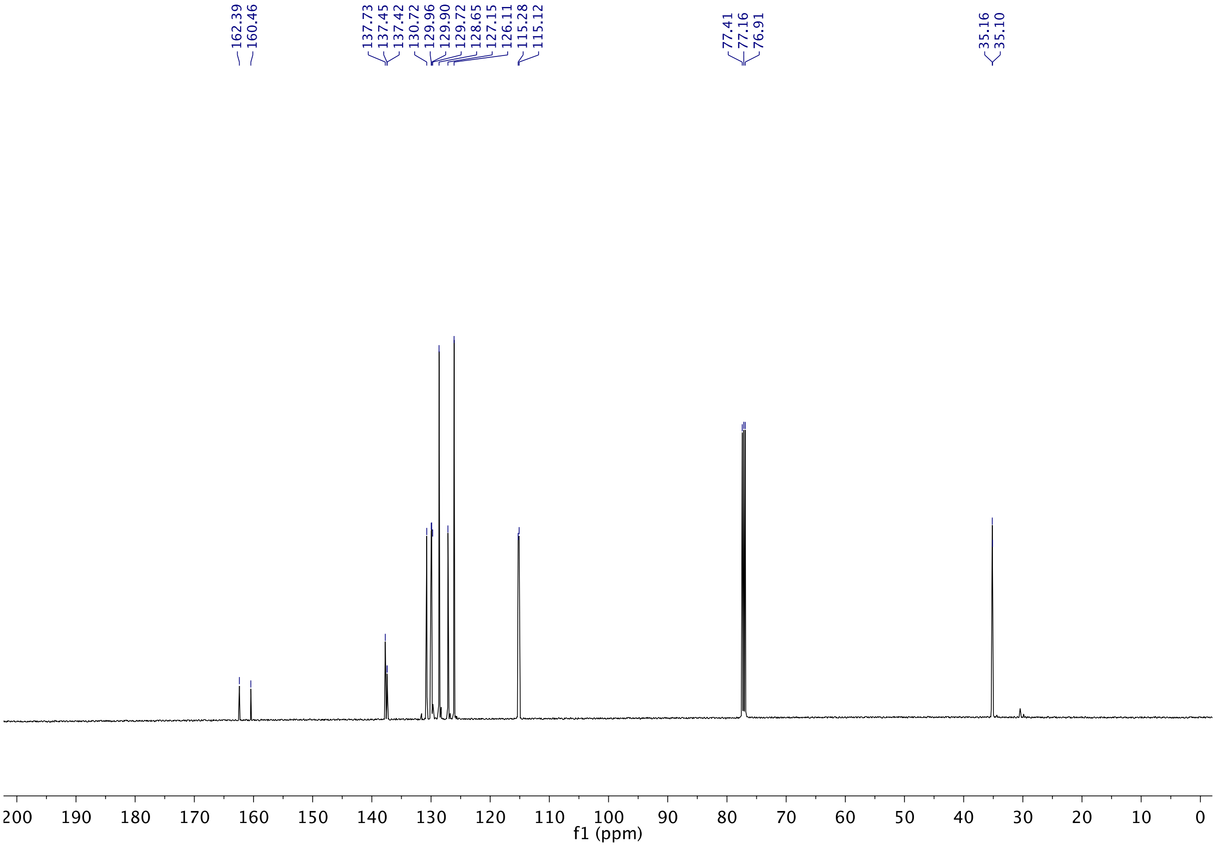
162.39
160.46
137.73
137.45
137.42
130.72
129.96
129.90
129.72
128.65
127.15
126.11
115.28
115.12
77.41
77.16
76.91
35.16
35.10
200 190 180 170 160 150 140 130 120 110 100 90 80 70 60 50 40 30 20 10 0
f1 (ppm)

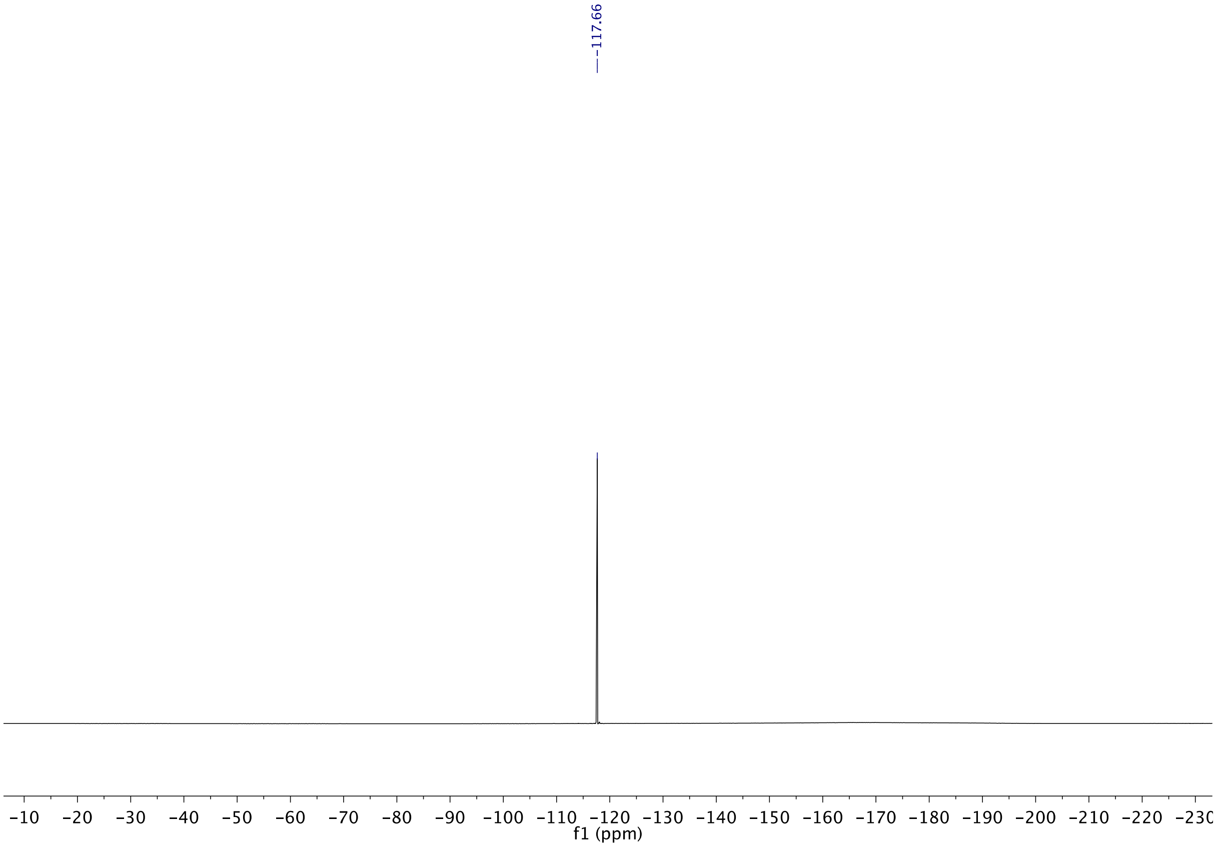
-117.66
-10 -20 -30 -40 -50 -60 -70 -80 -90 -100 -110 -120 -130 -140 -150 -160 -170 -180 -190 -200 -210 -220 -230
f1 (ppm)

(*E*)-(2-Cyclohexylvinyl)benzene, **4**

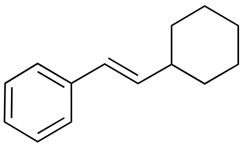

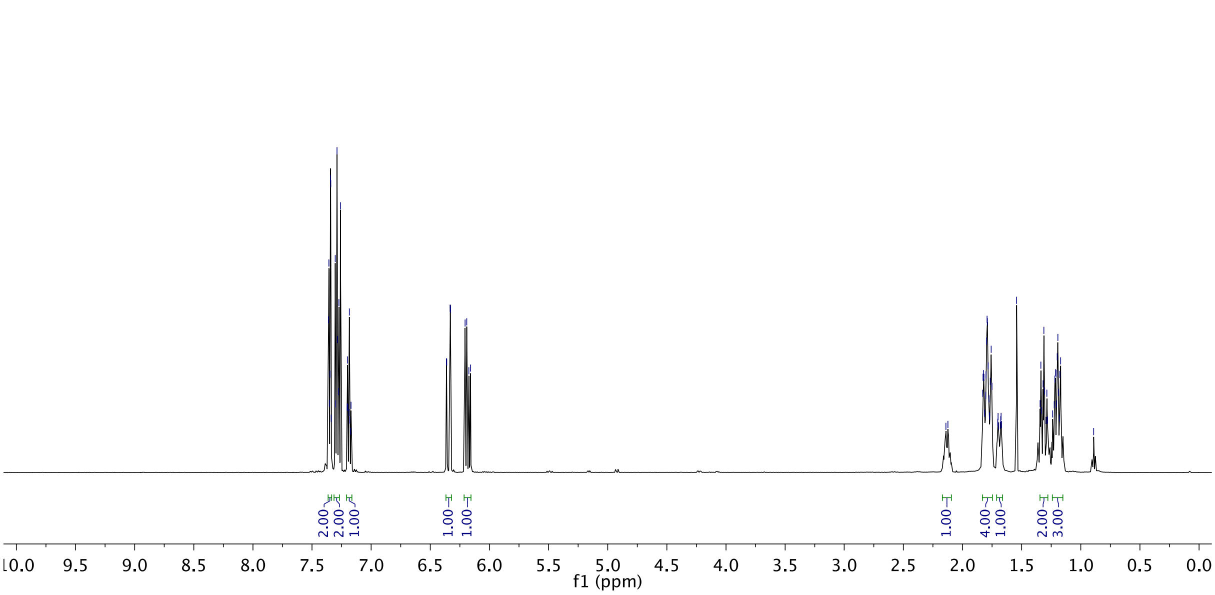

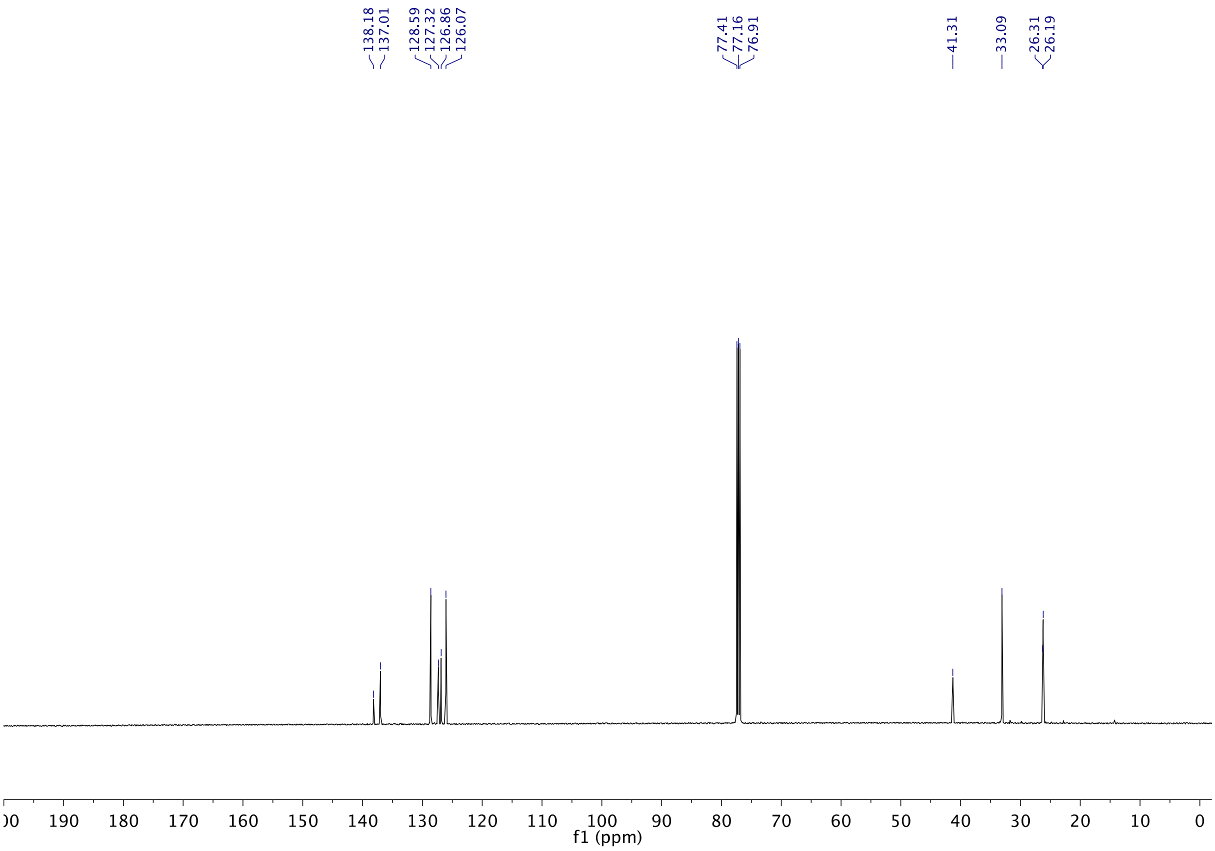

(*E*)-(2-Cyclobutylvinyl)benzene, **6**

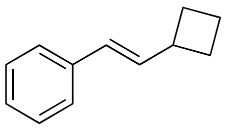

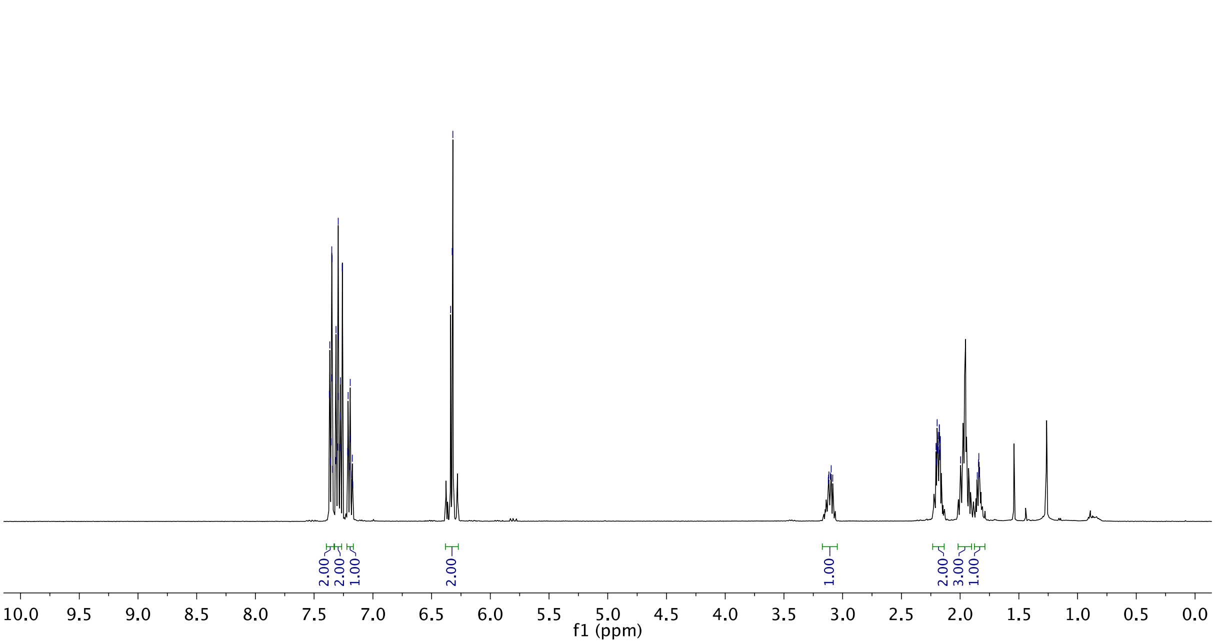

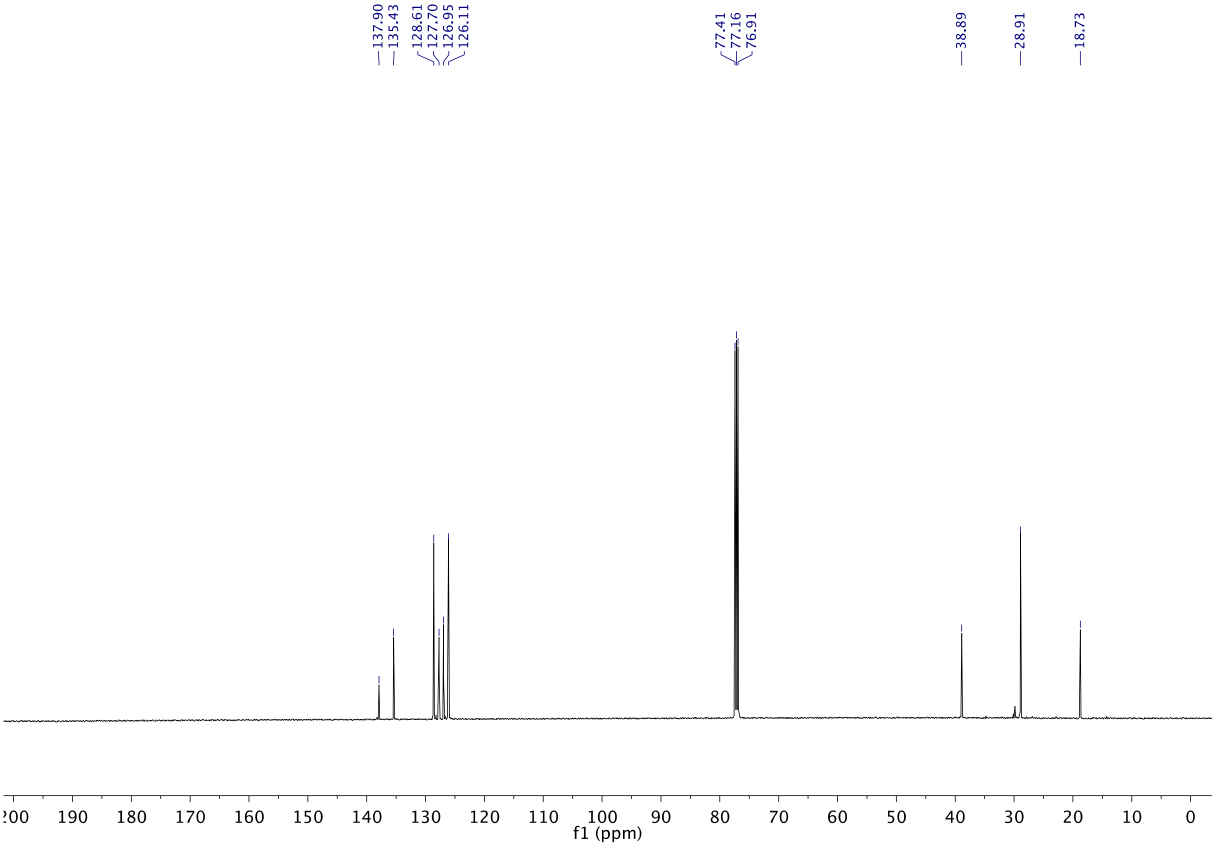

(*E*)-(2-Cyclopentylvinyl)benzene, **7**

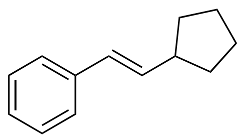

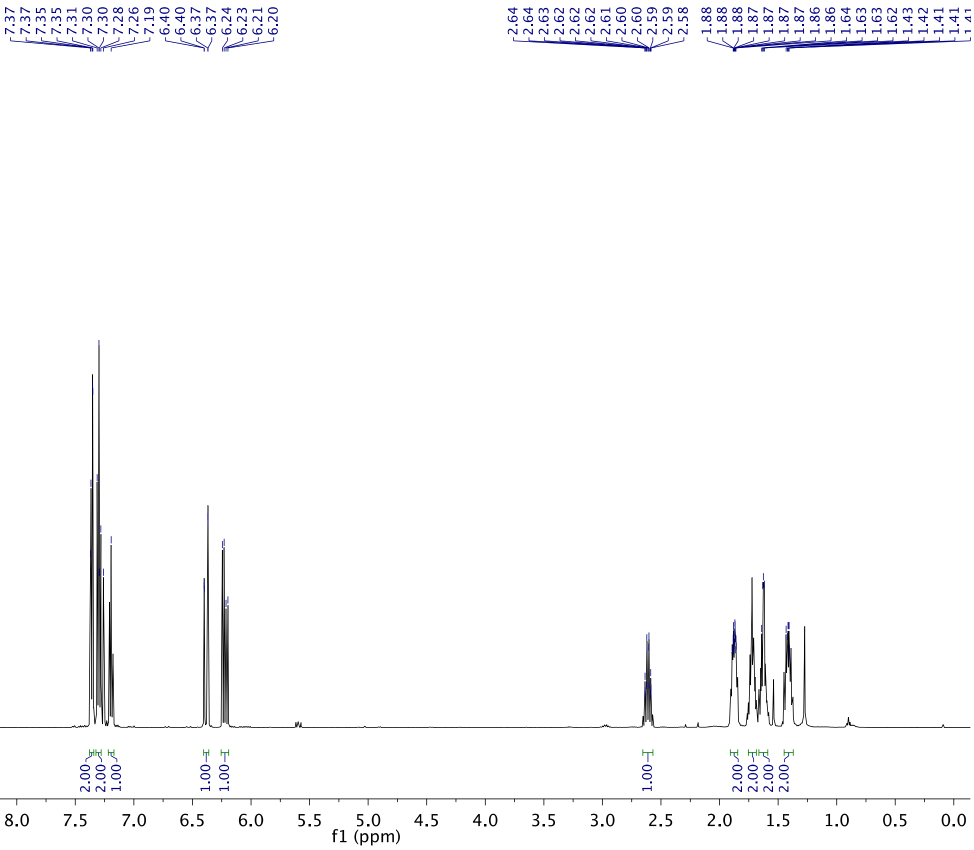

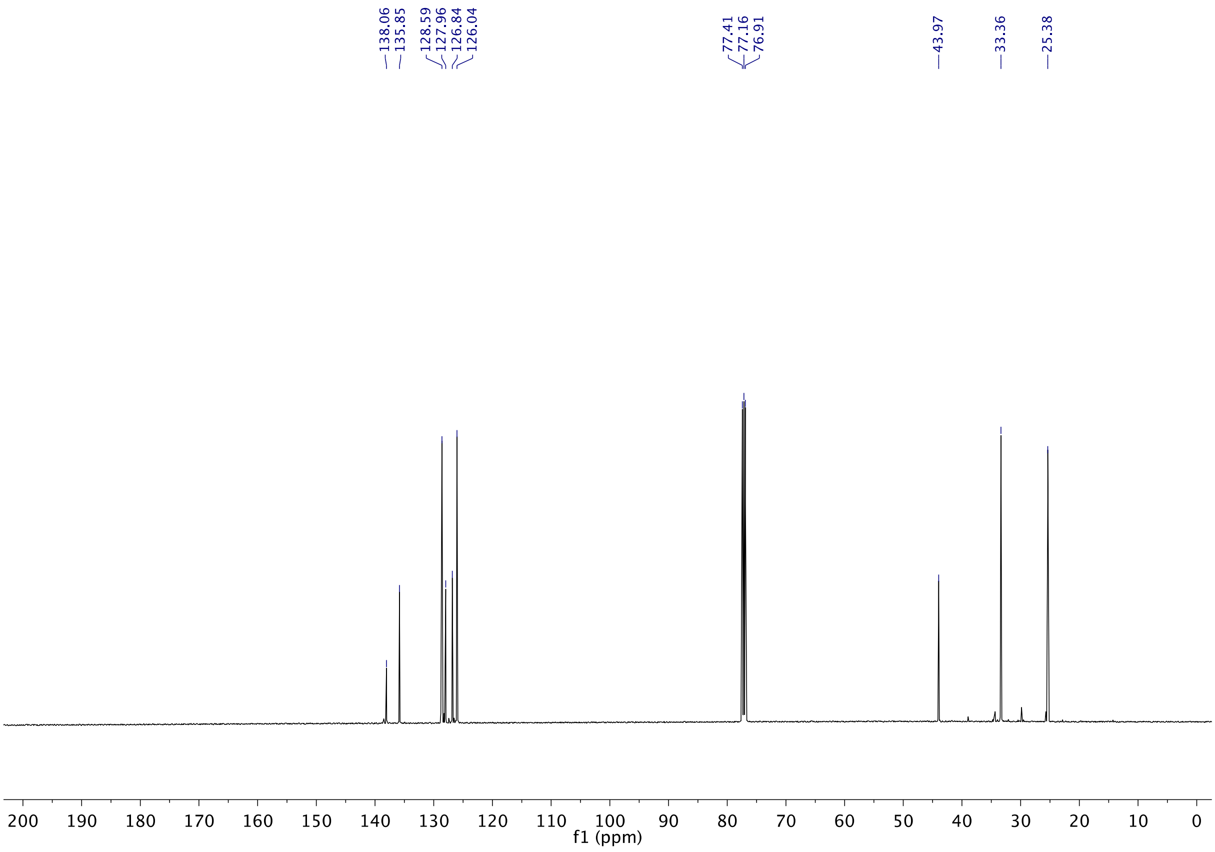

(*E*)-Styrylcycloheptane, **8**

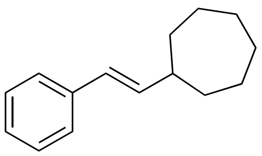

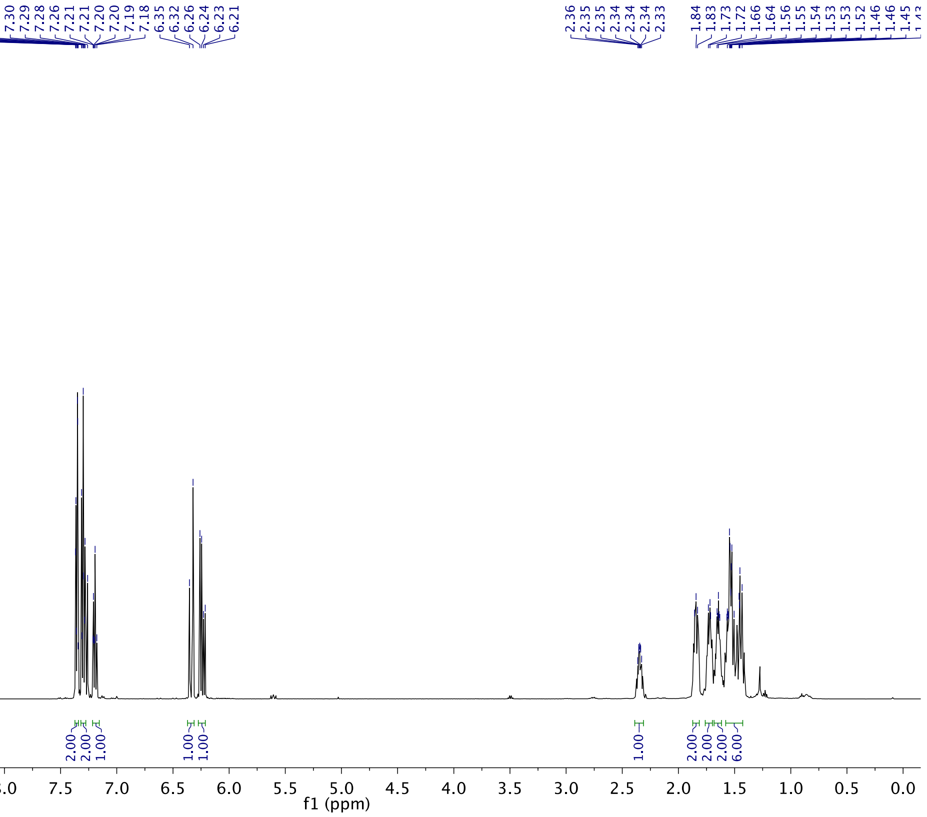

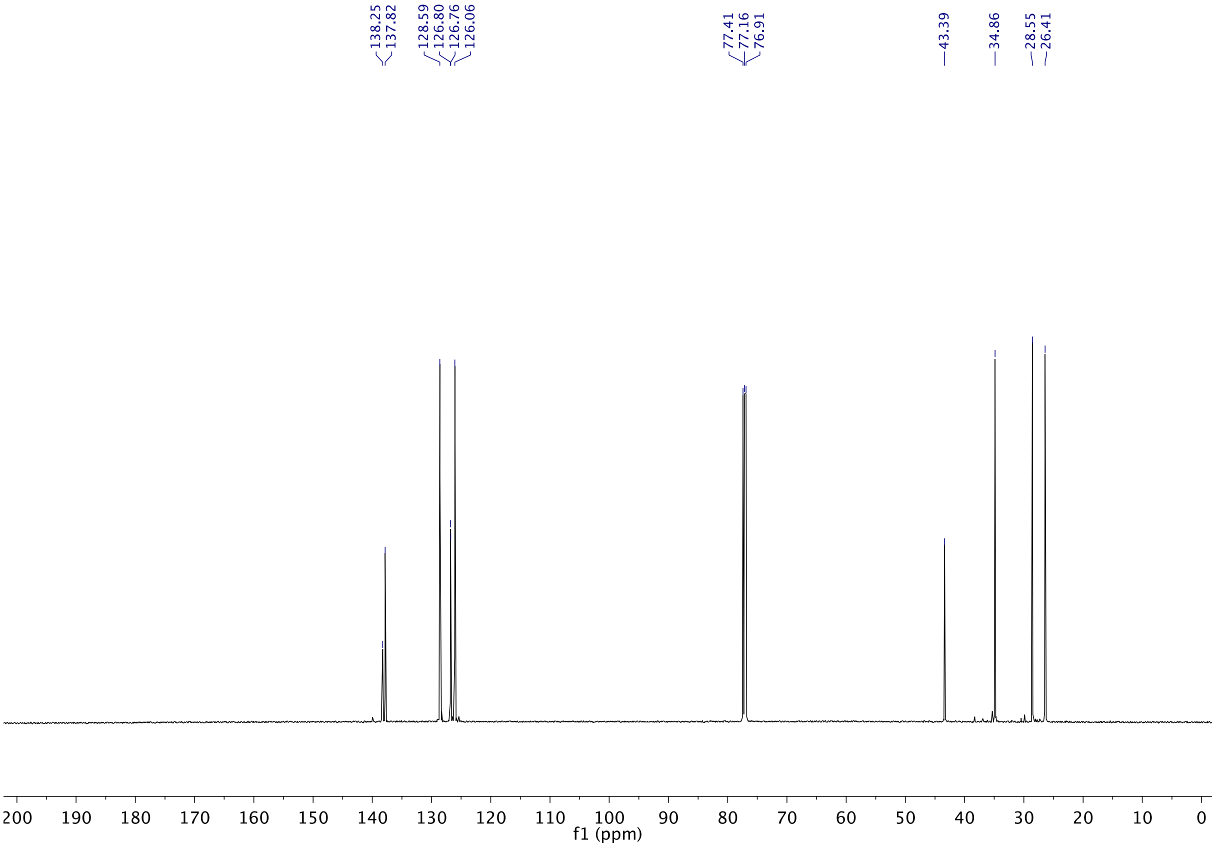

(*E*)-(3,3-Dimethylbut-1-en-1-yl)benzene, **9**

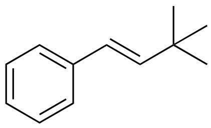

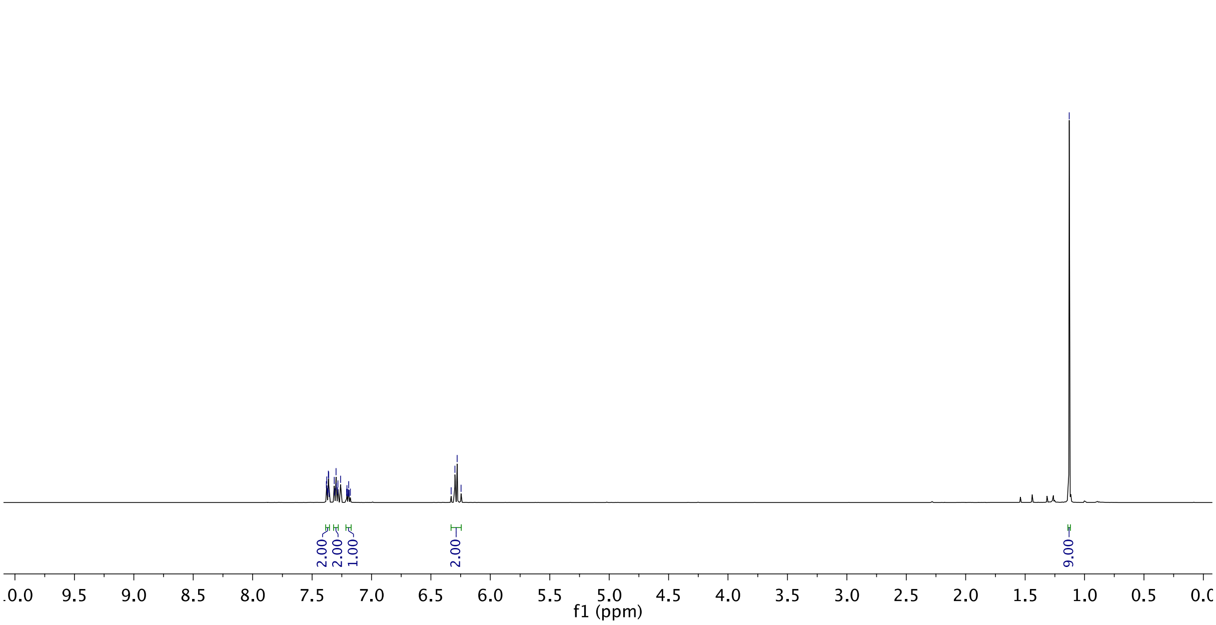

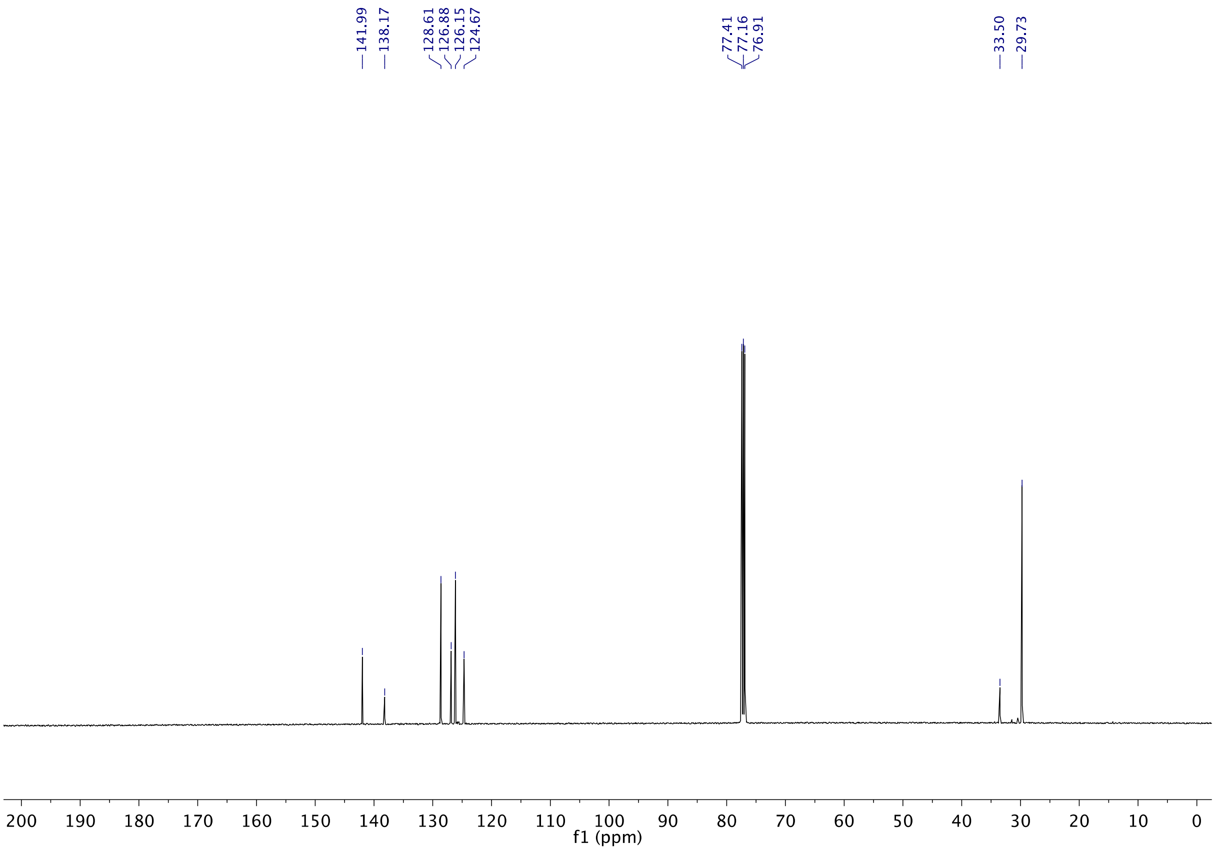

(*E*)-Pent-1-en-1-ylbenzene, **10**

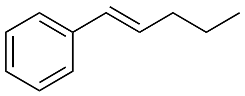

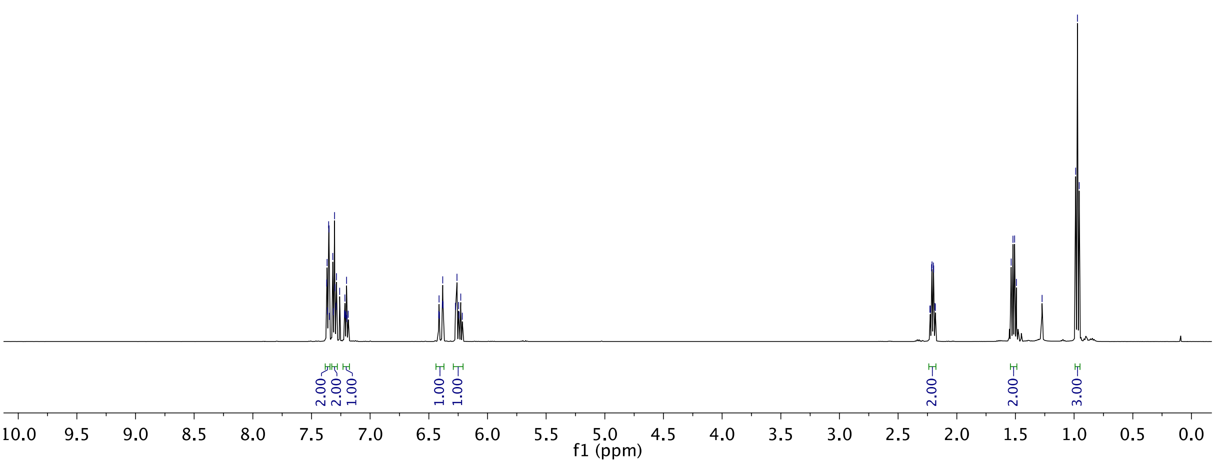

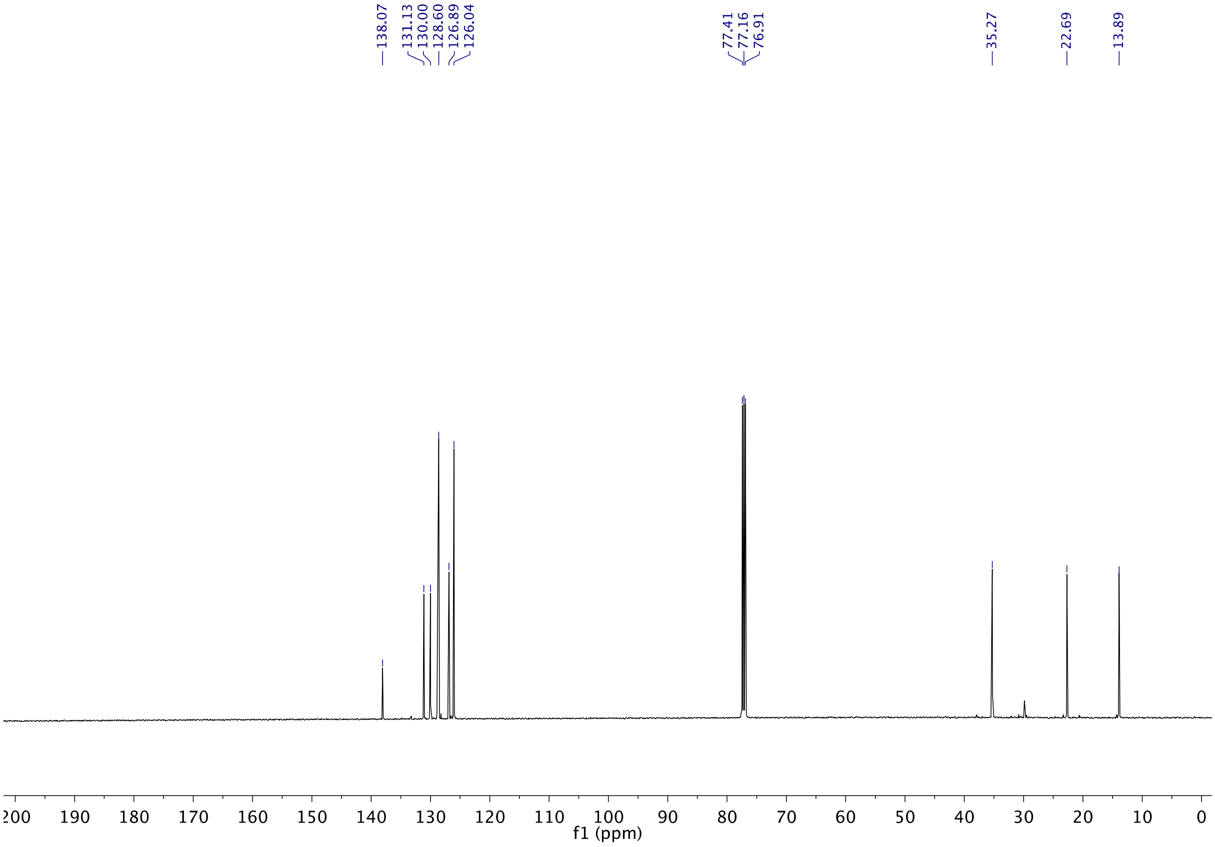

(*E*)-(3-Methylpent-1-en-1-yl)benzene, **11**

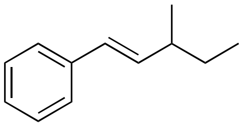

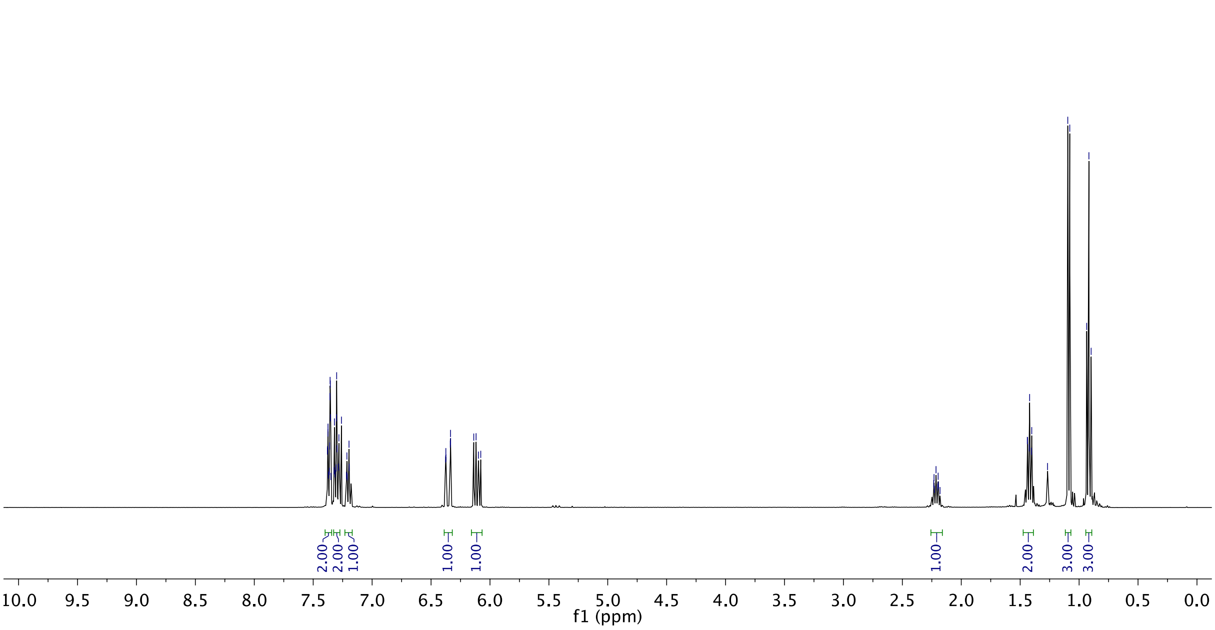

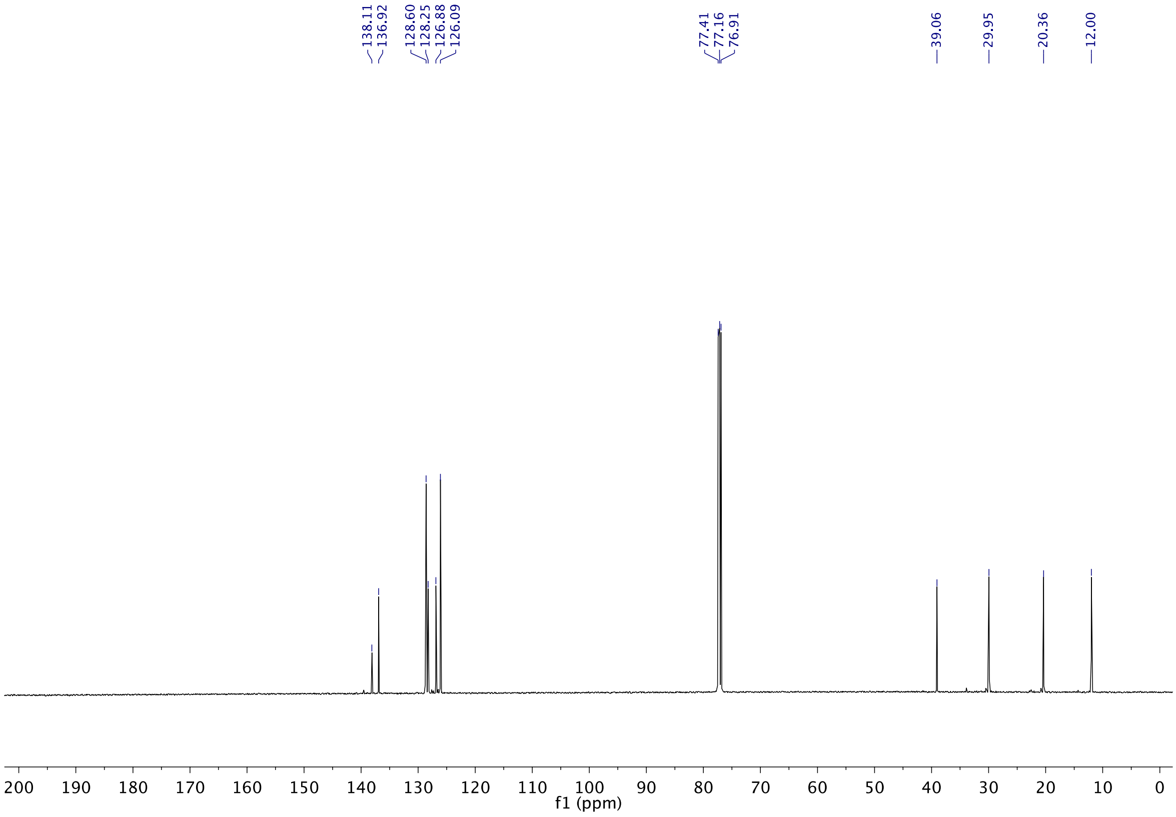

(*E*)-non-1-en-1-ylbenzene, **12**

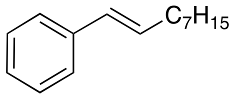

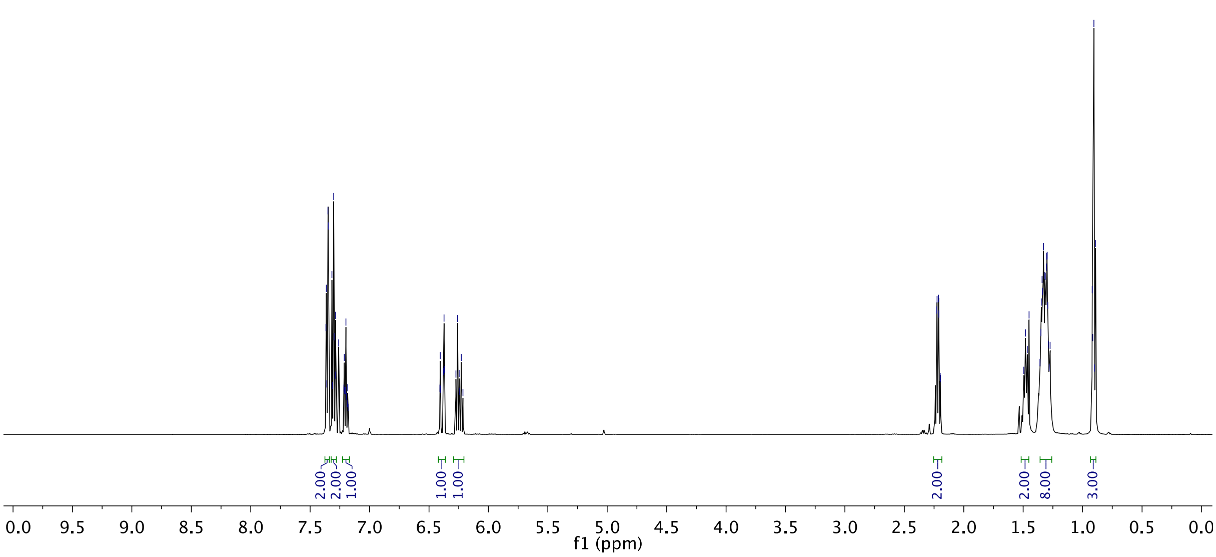

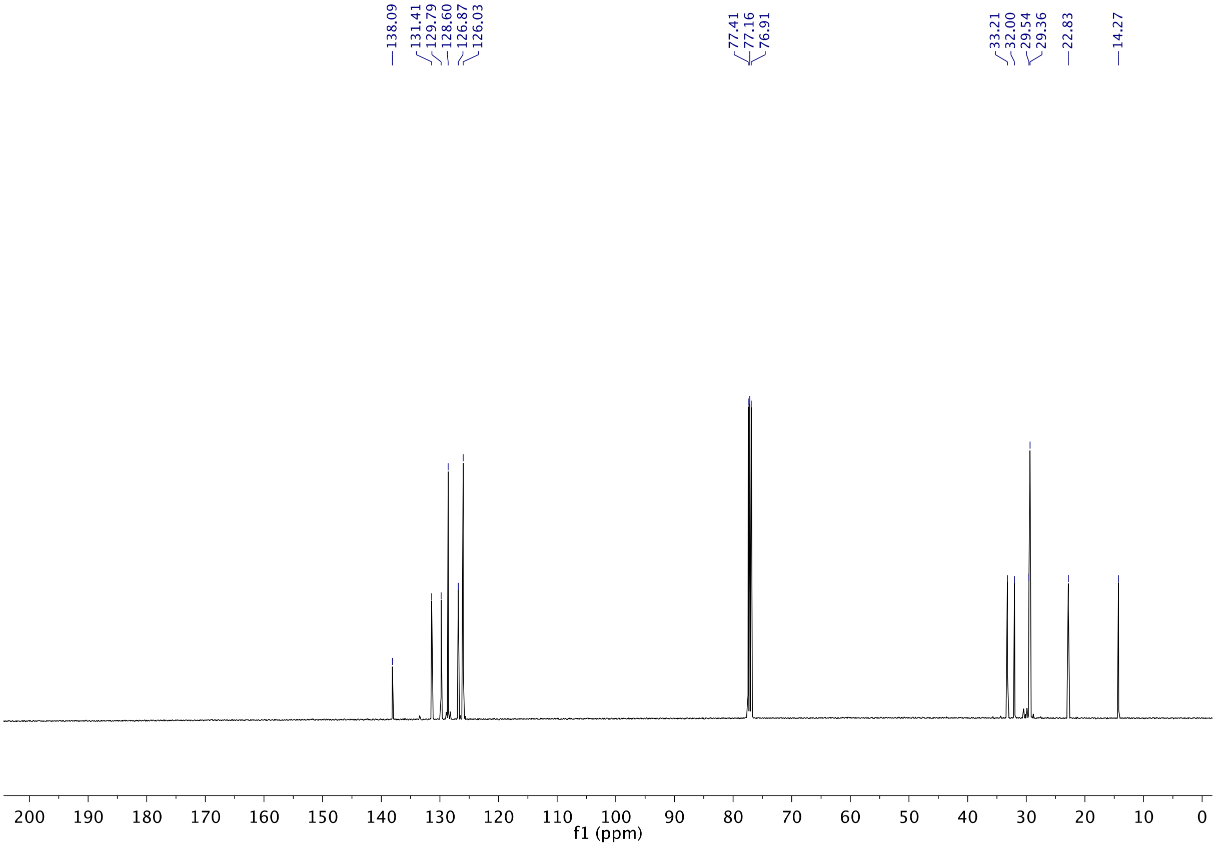

(*E*)-Pentadec-1-en-1-ylbenzene, **13**

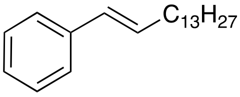

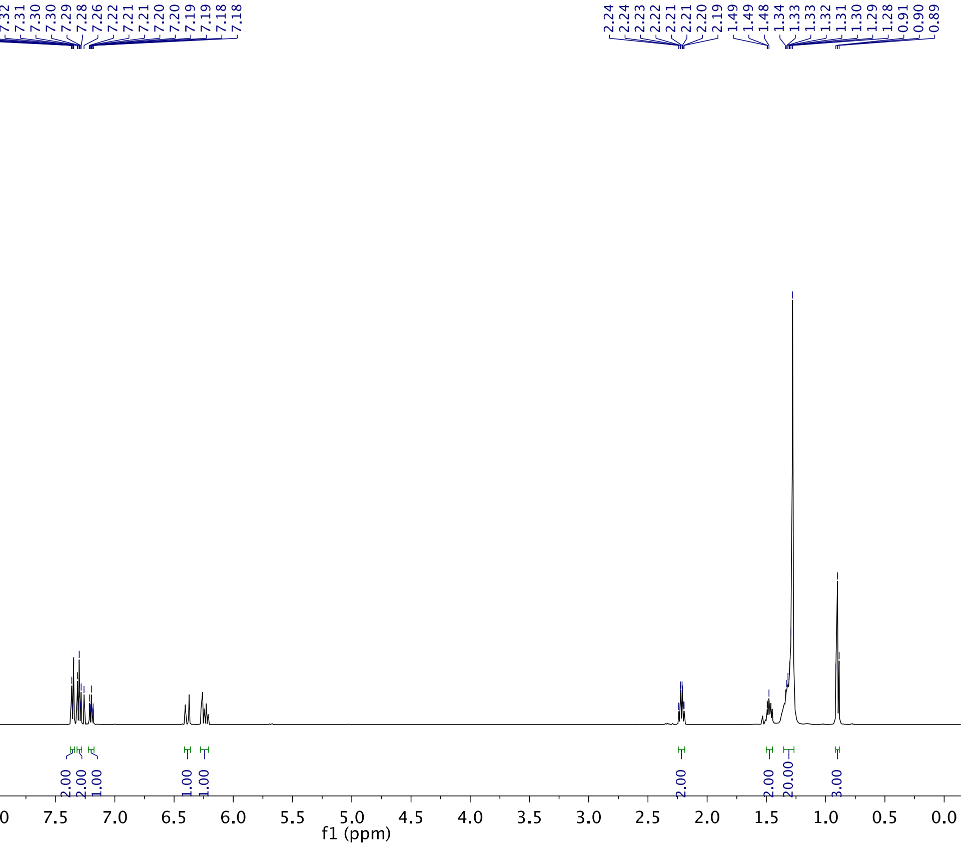

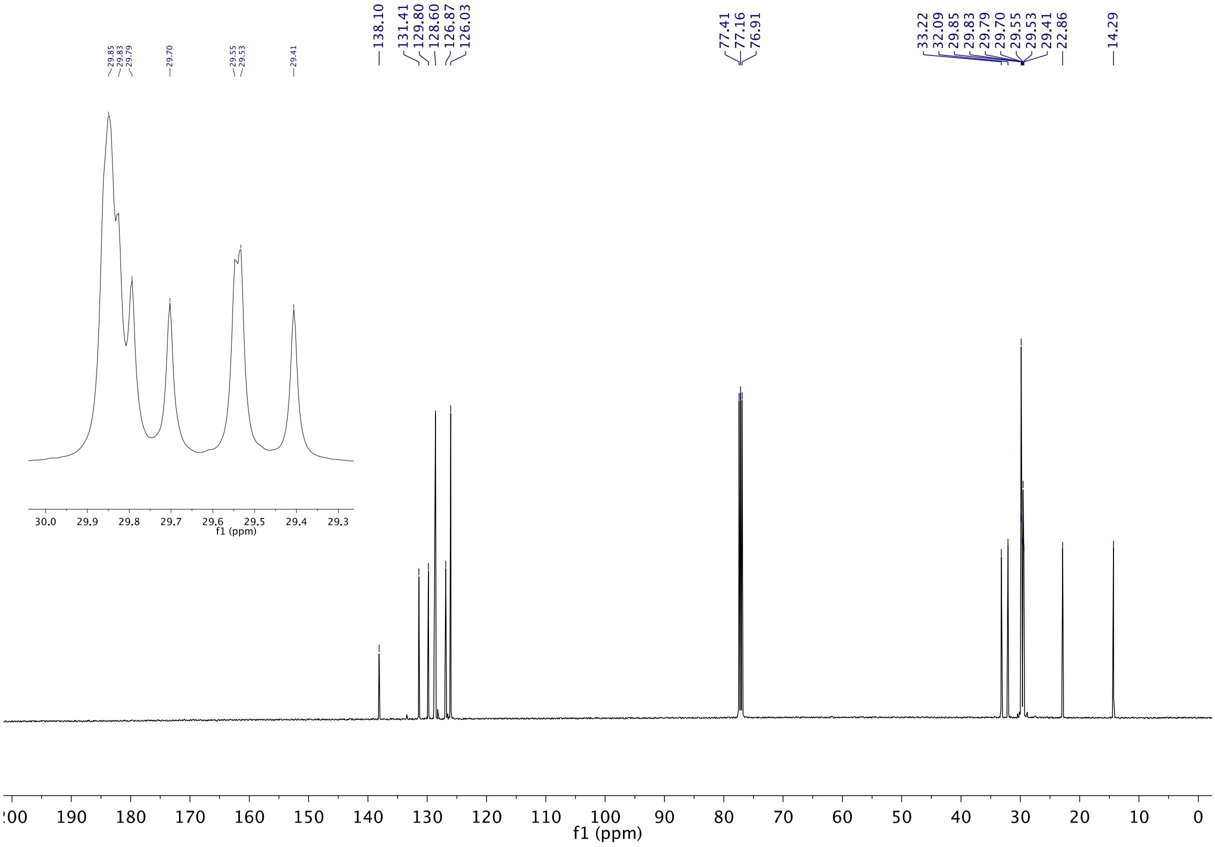

(*E*)-But-1-ene-1,4-diyldibenzene, **14**

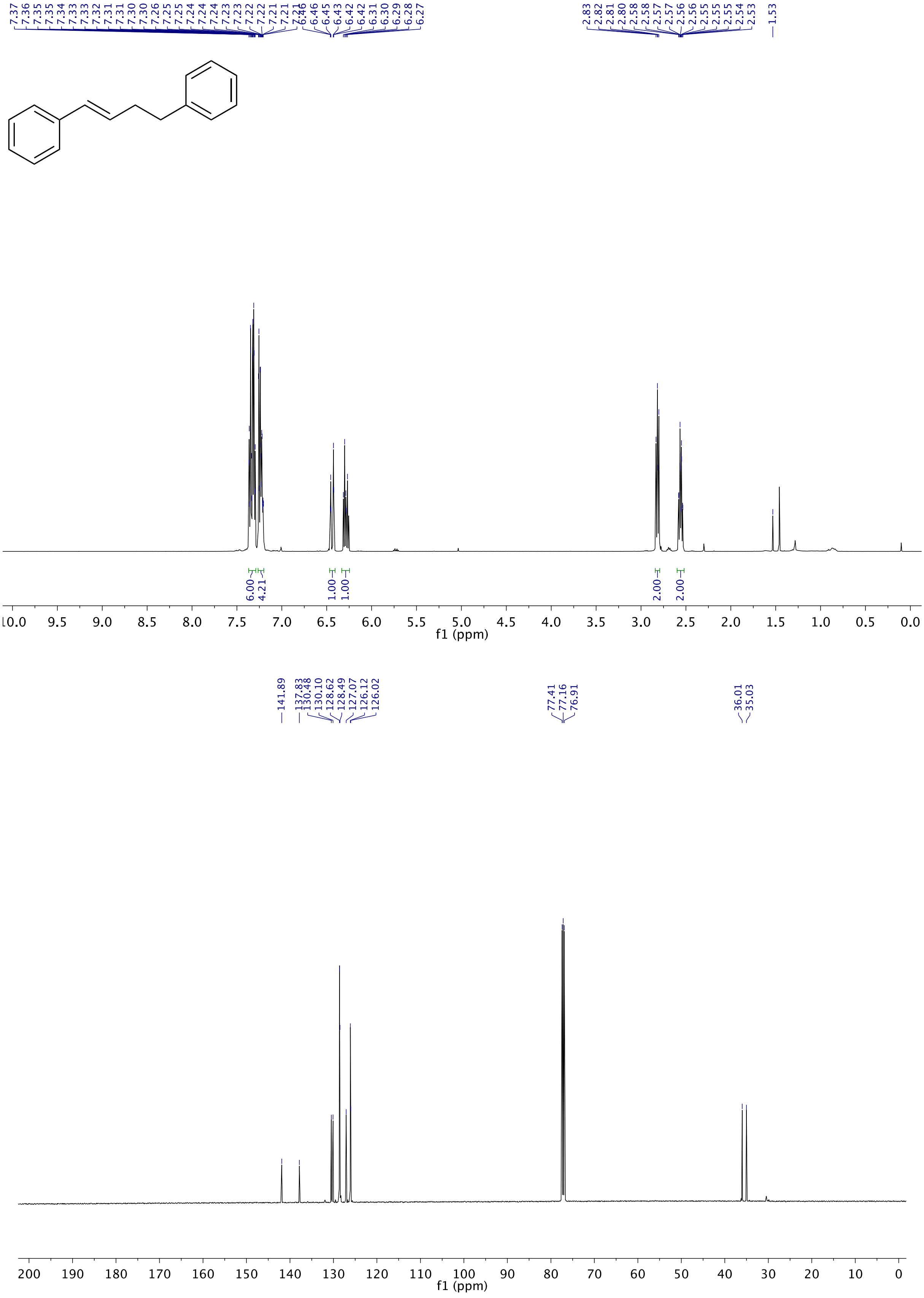

(*E*)-1-Bromo-4-(4-phenylbut-3-en-1-yl)benzene, **15**

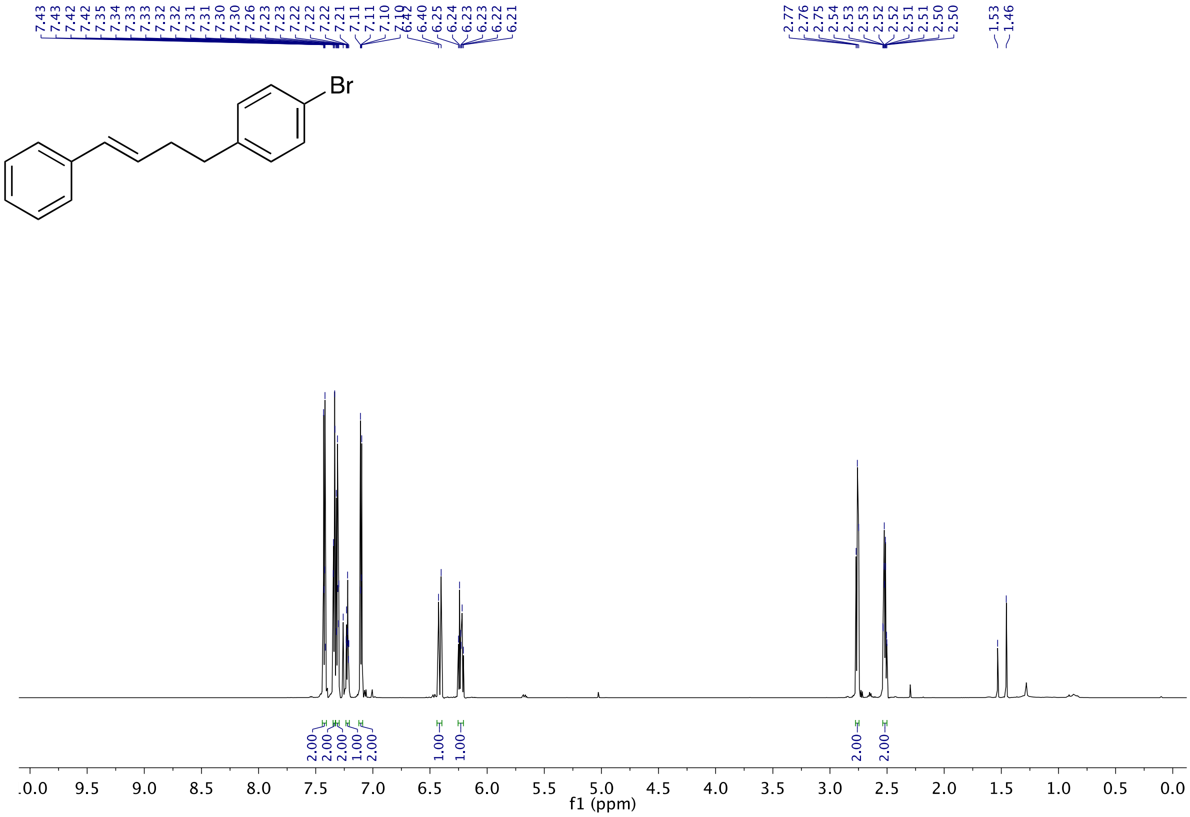

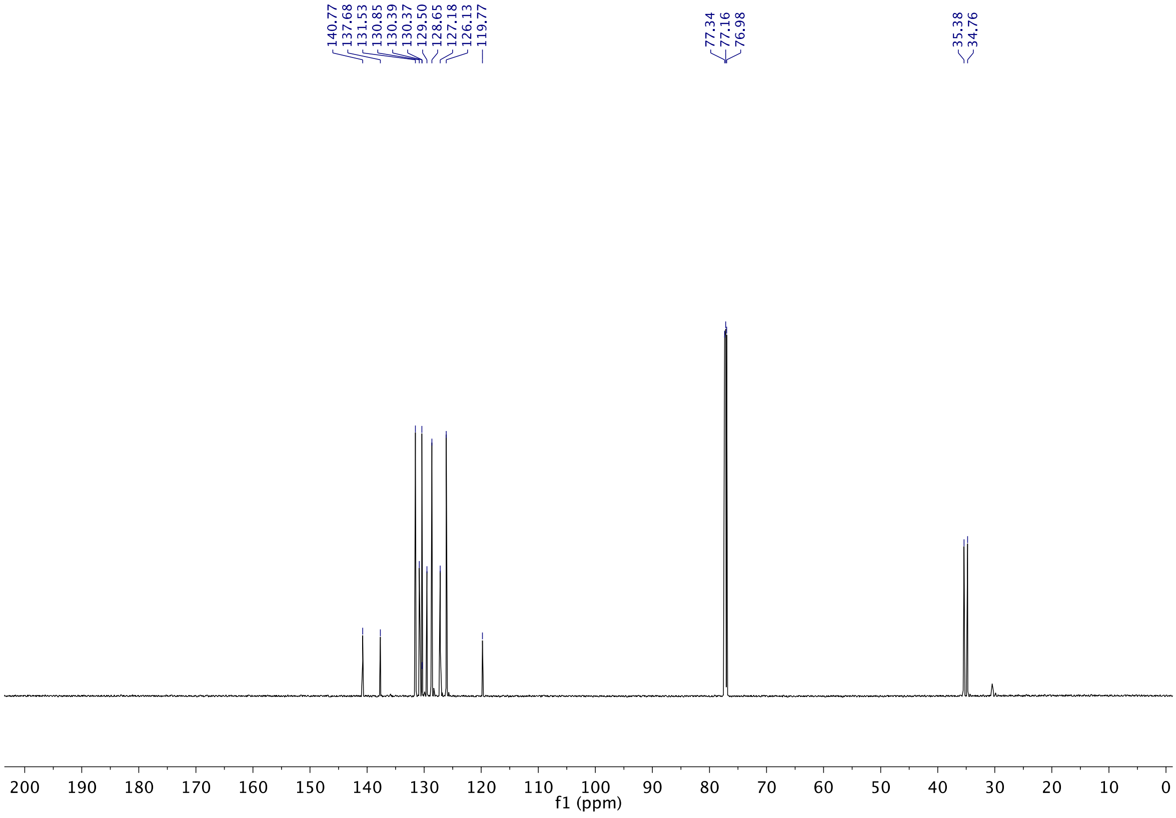

(*E*)-1-Ethynyl-4-(4-phenylbut-3-en-1-yl)benzene, **16**

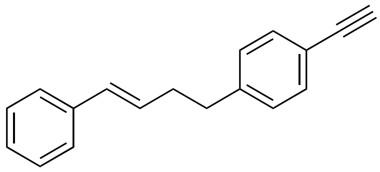

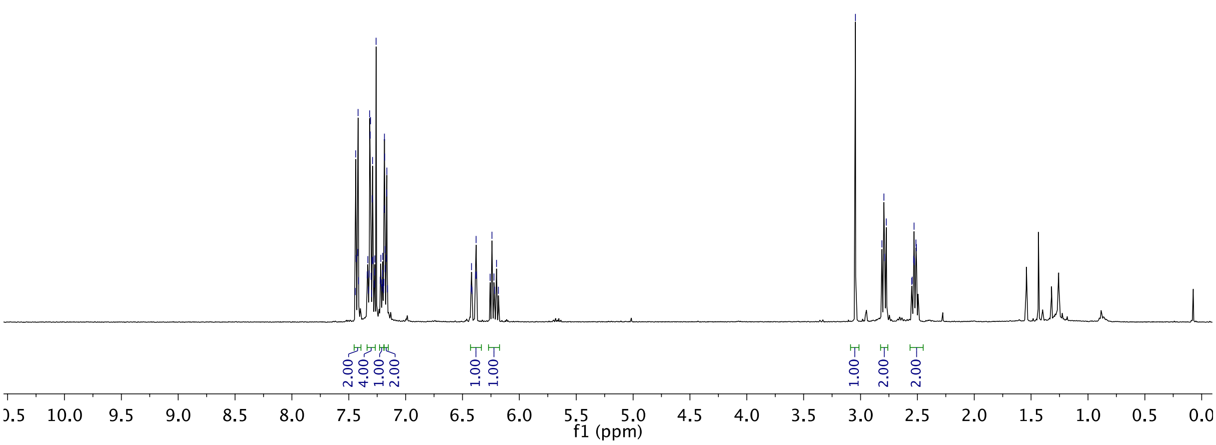

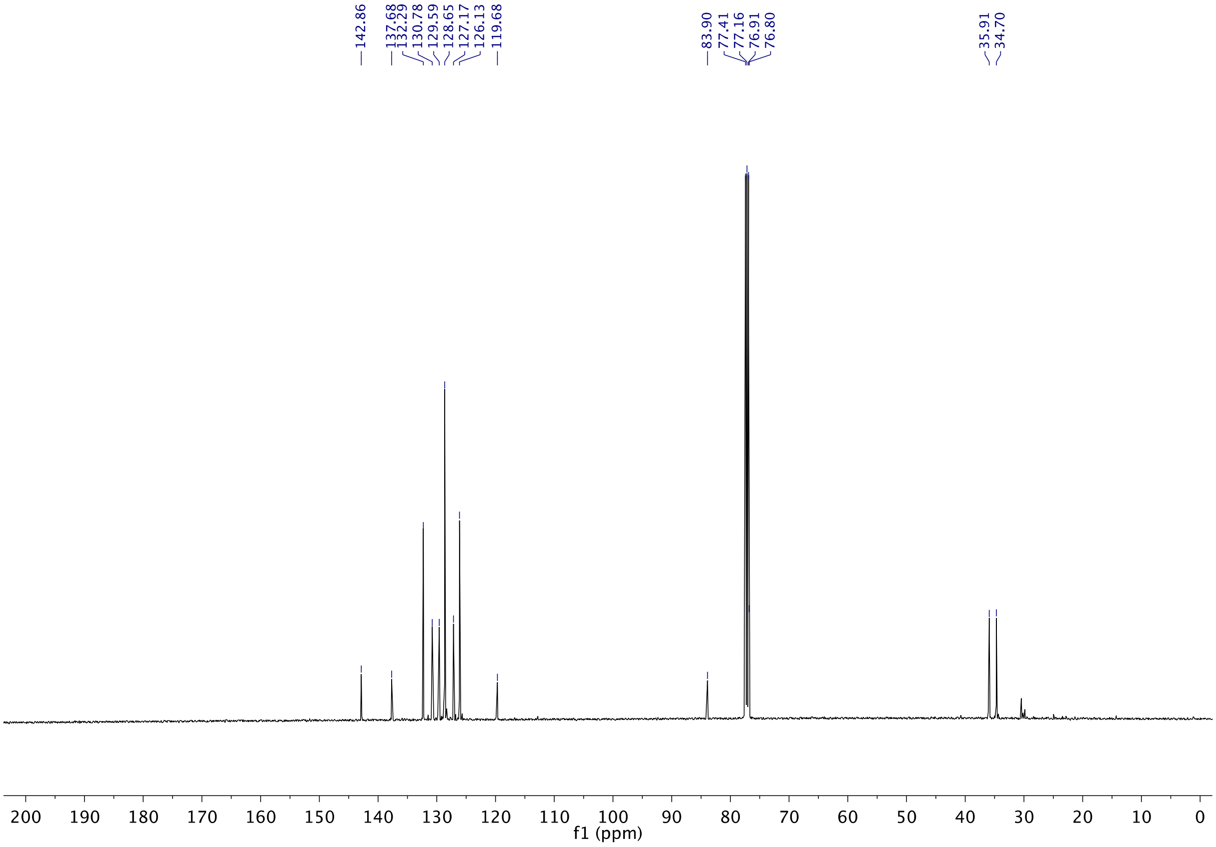

(*E*)-(2-(1-Methylcyclohexyl)vinyl)benzene, **17**

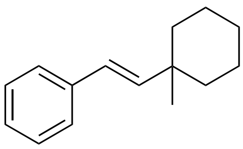

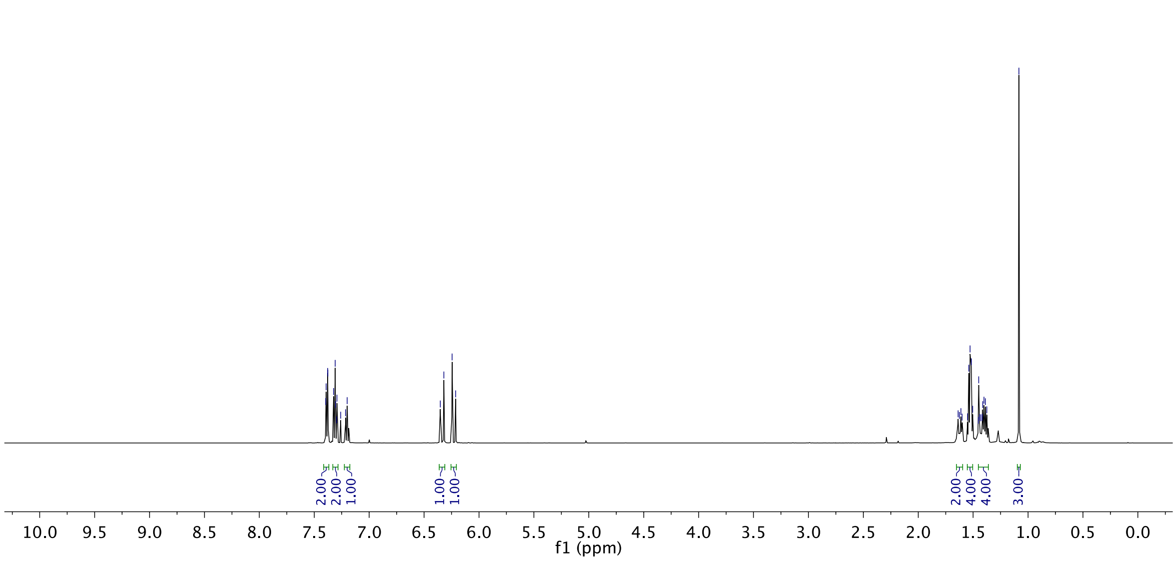

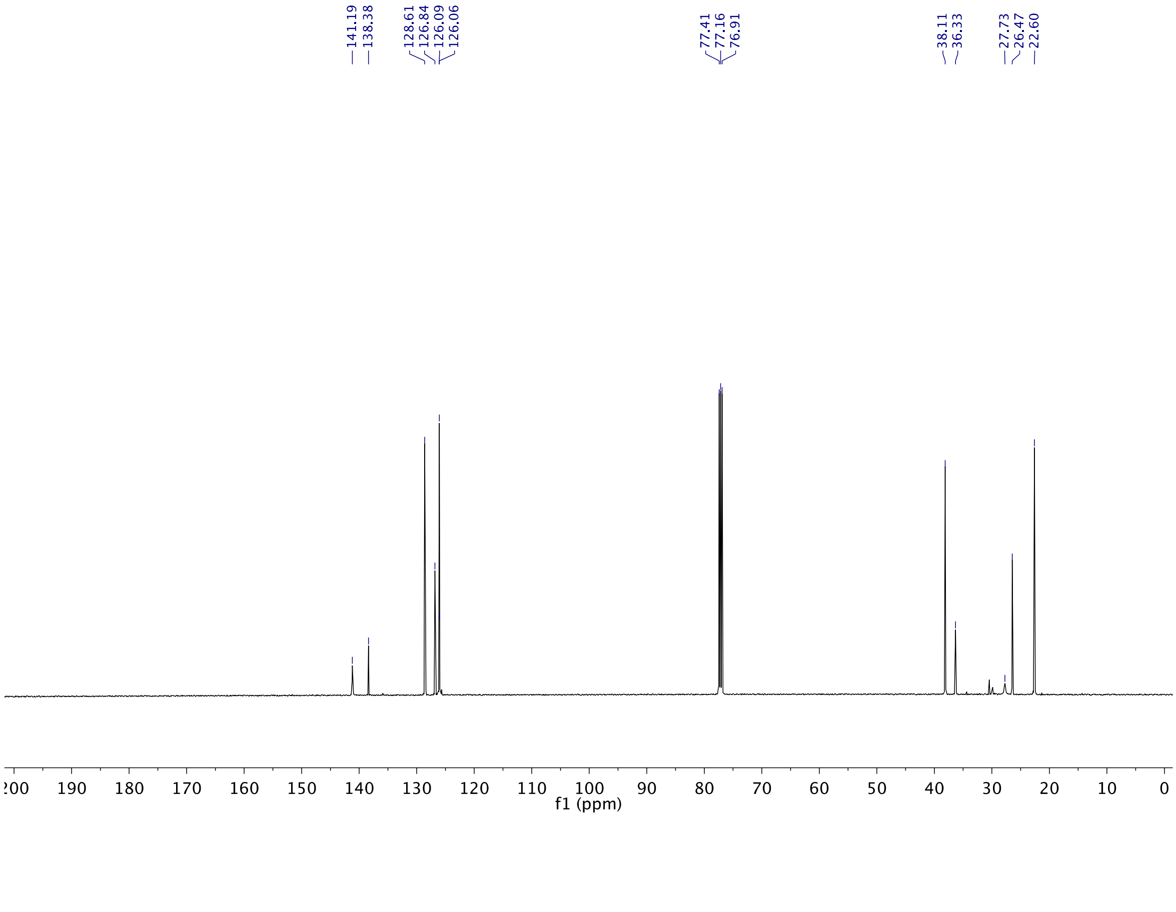

Methyl (*E*)-6-phenylhex-5-enoate, **18**

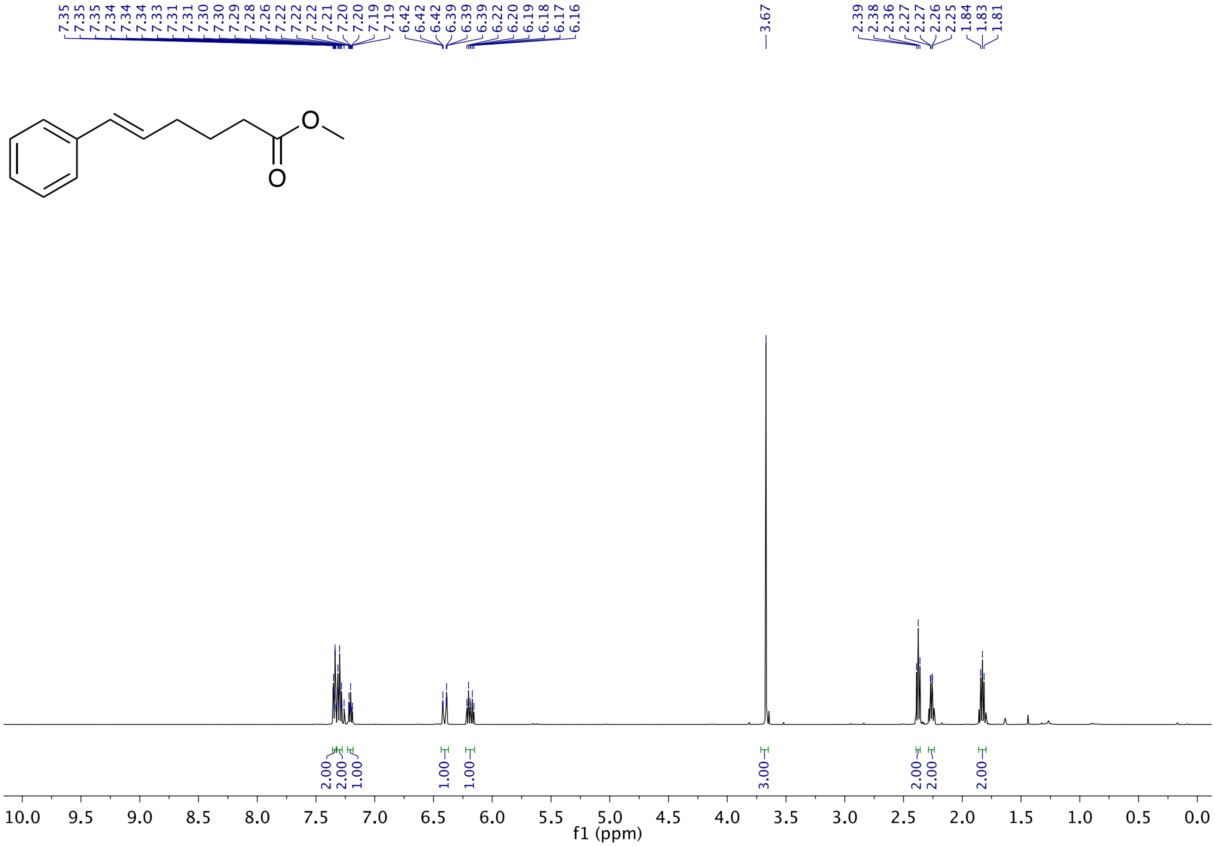

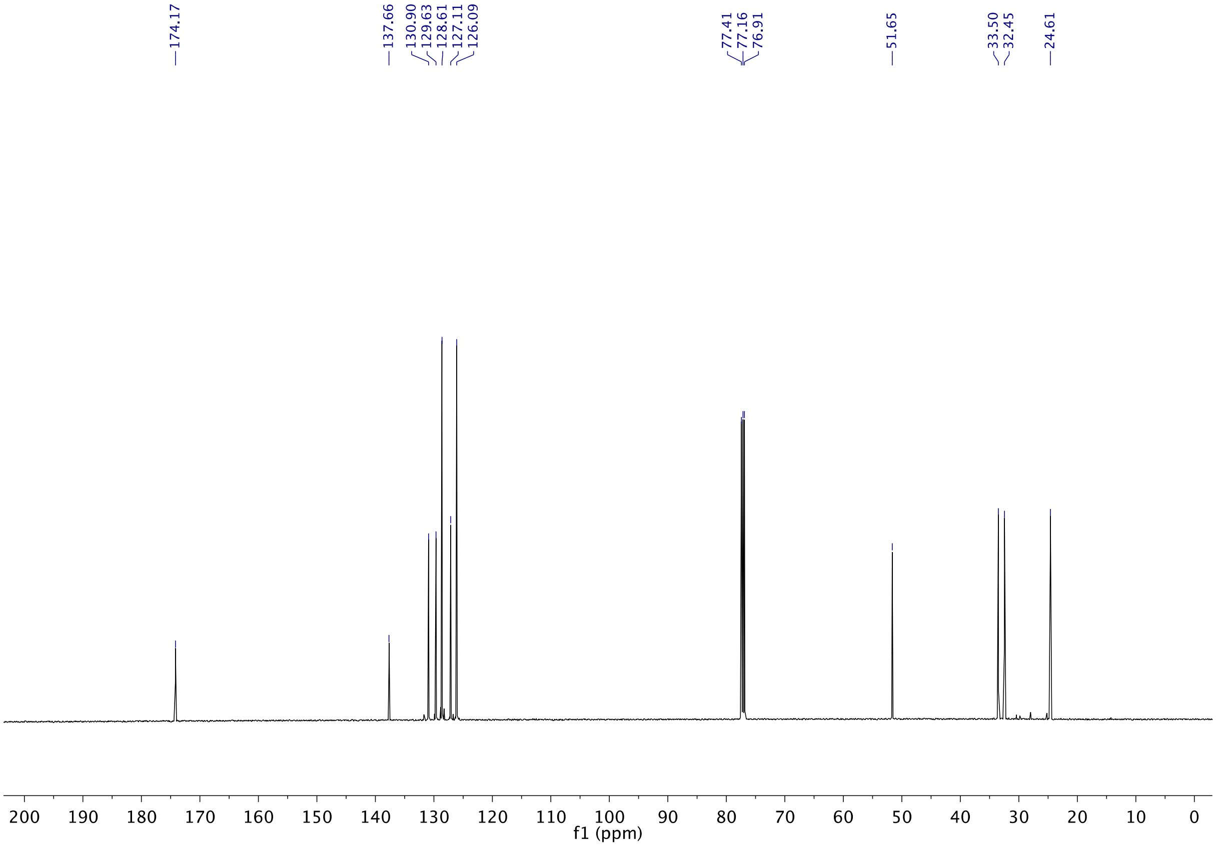

(*E*)-(5-Bromopent-1-en-1-yl)benzene, **19**

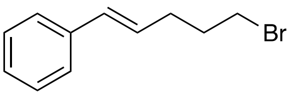

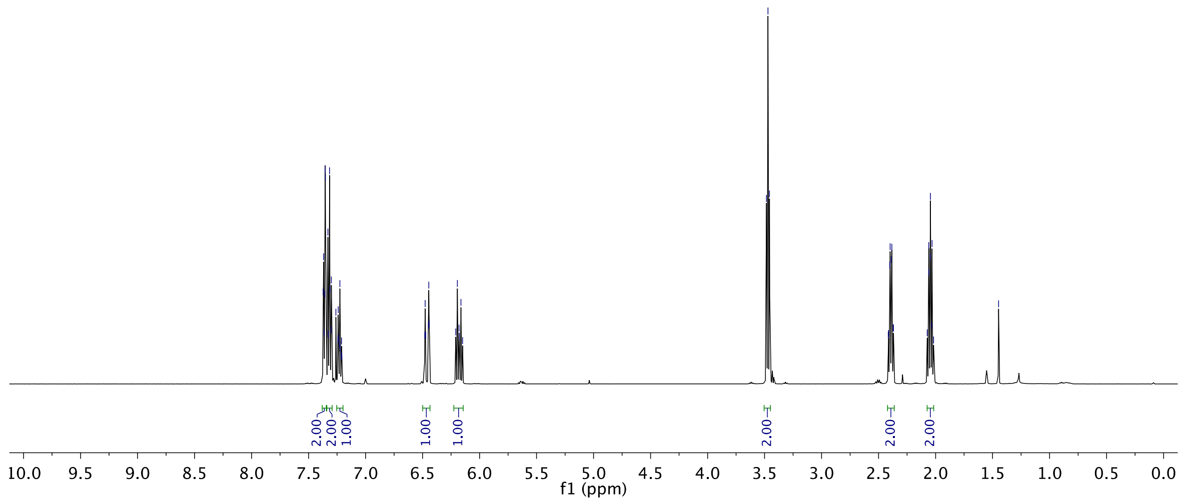

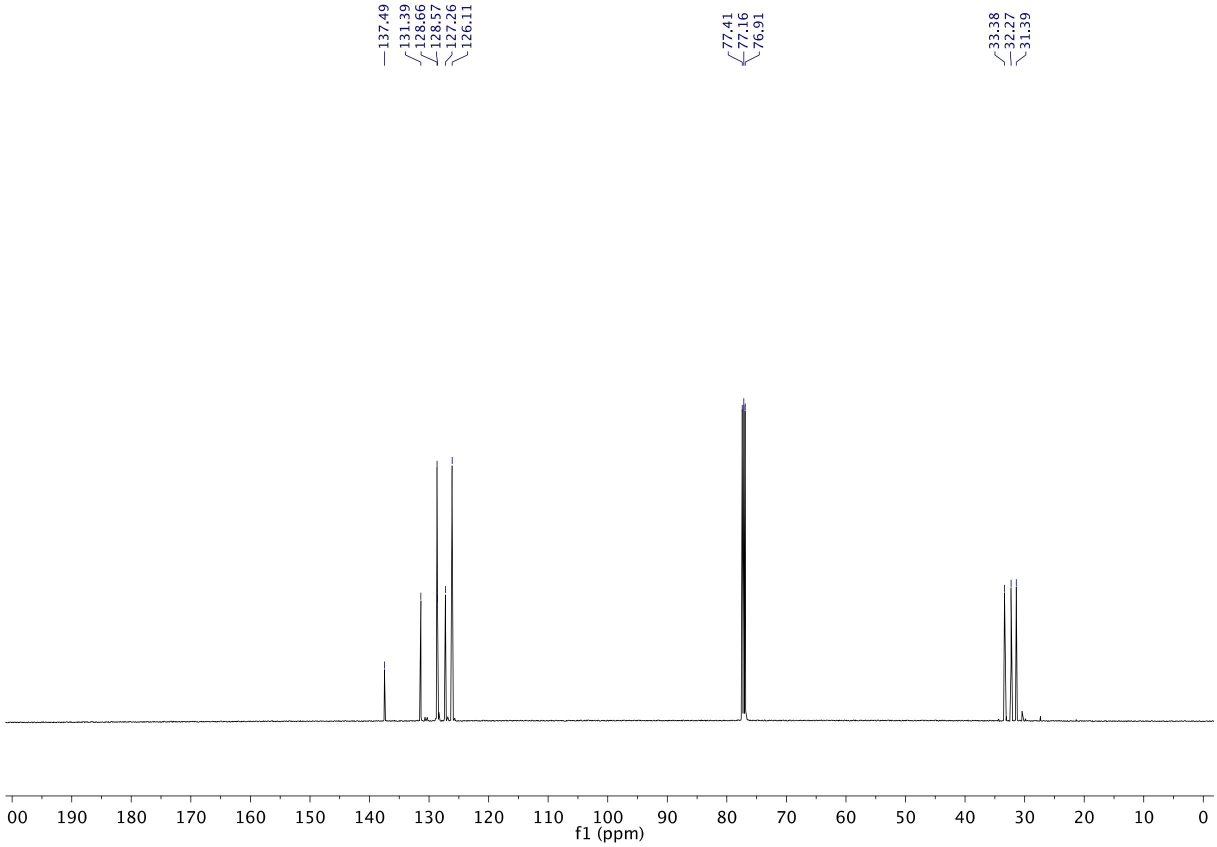

(*E*)-Hexa-1,5-dien-1-ylbenzene, **20**

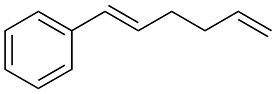

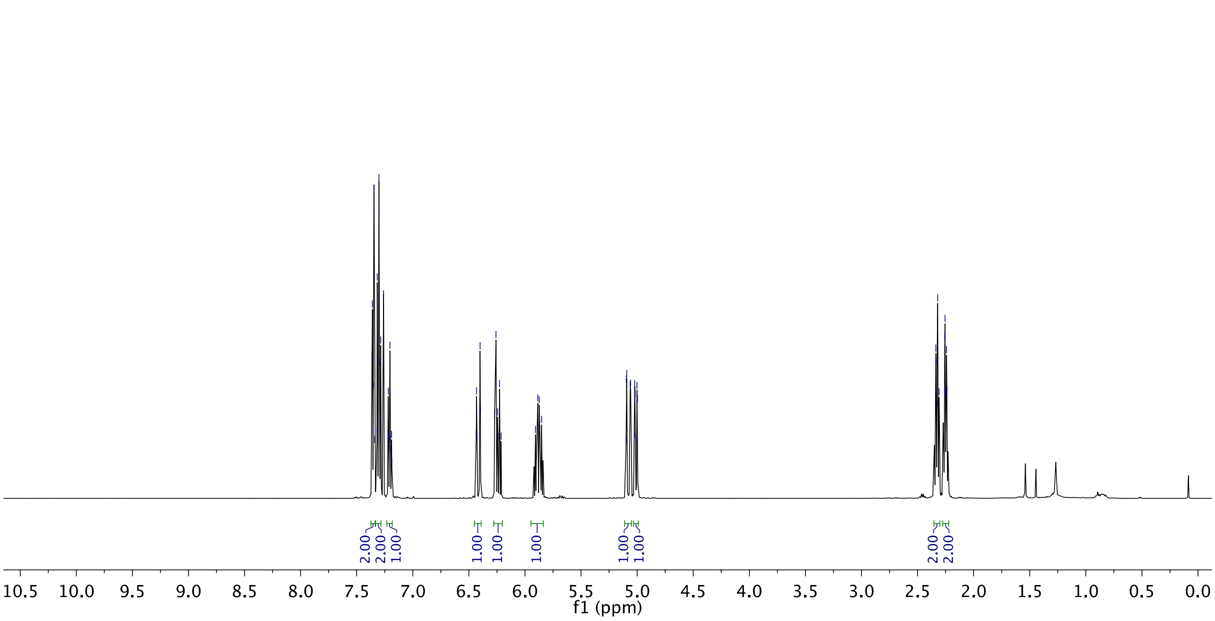

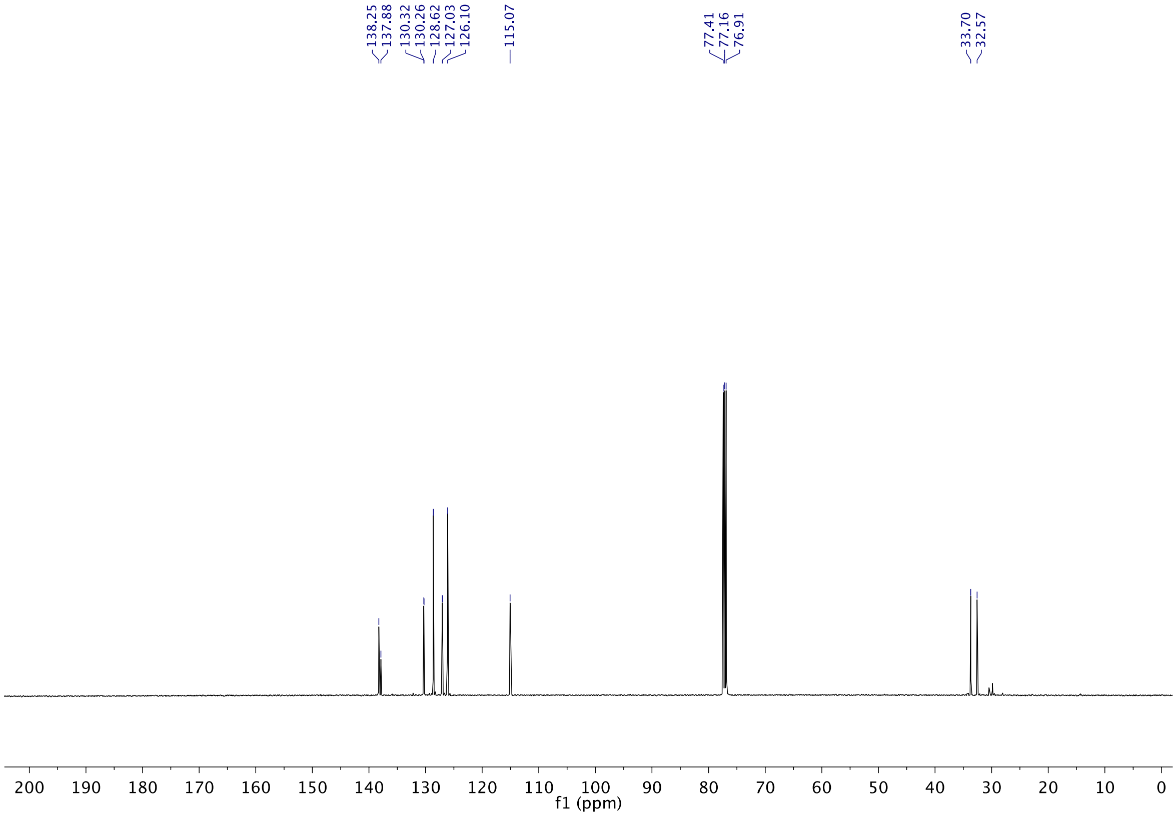

(*E*)-(3-(Cyclopent-2-en-1-yl)prop-1-en-1-yl)benzene, **21**

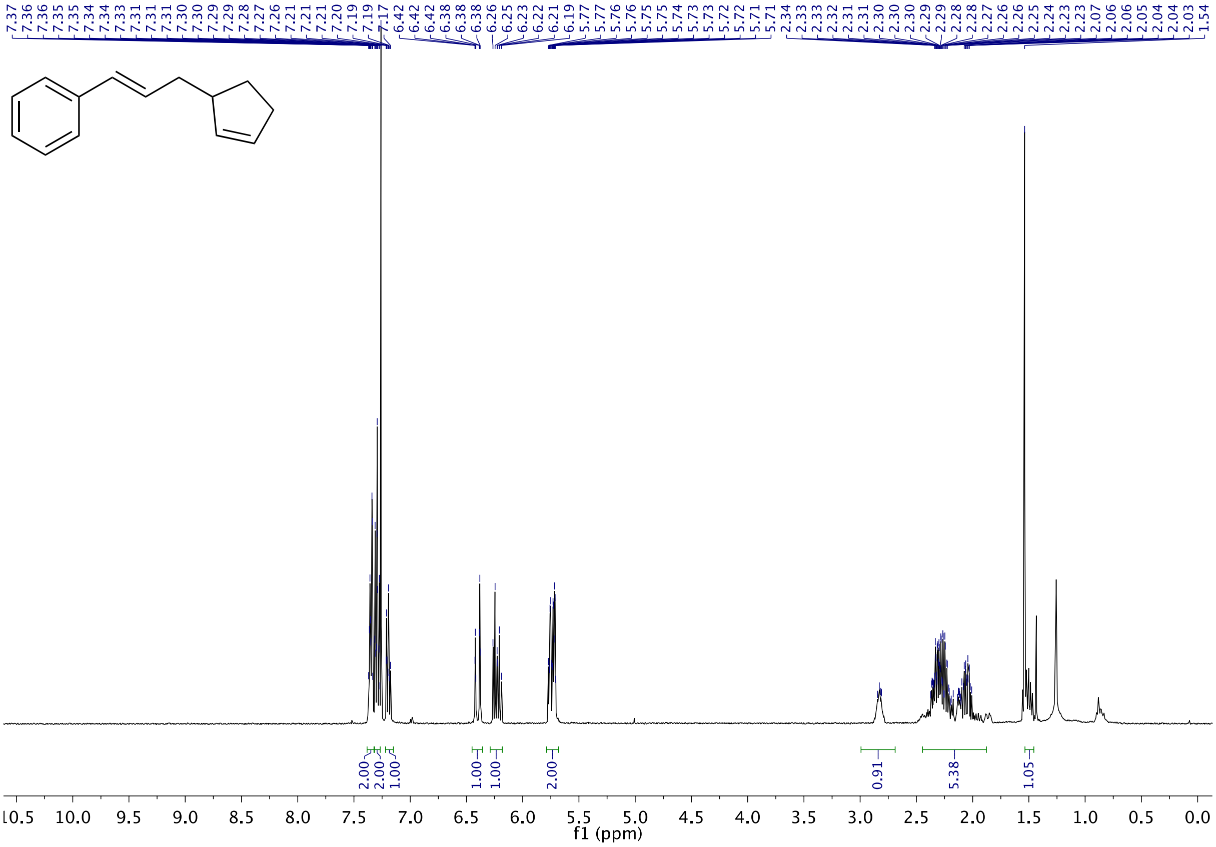

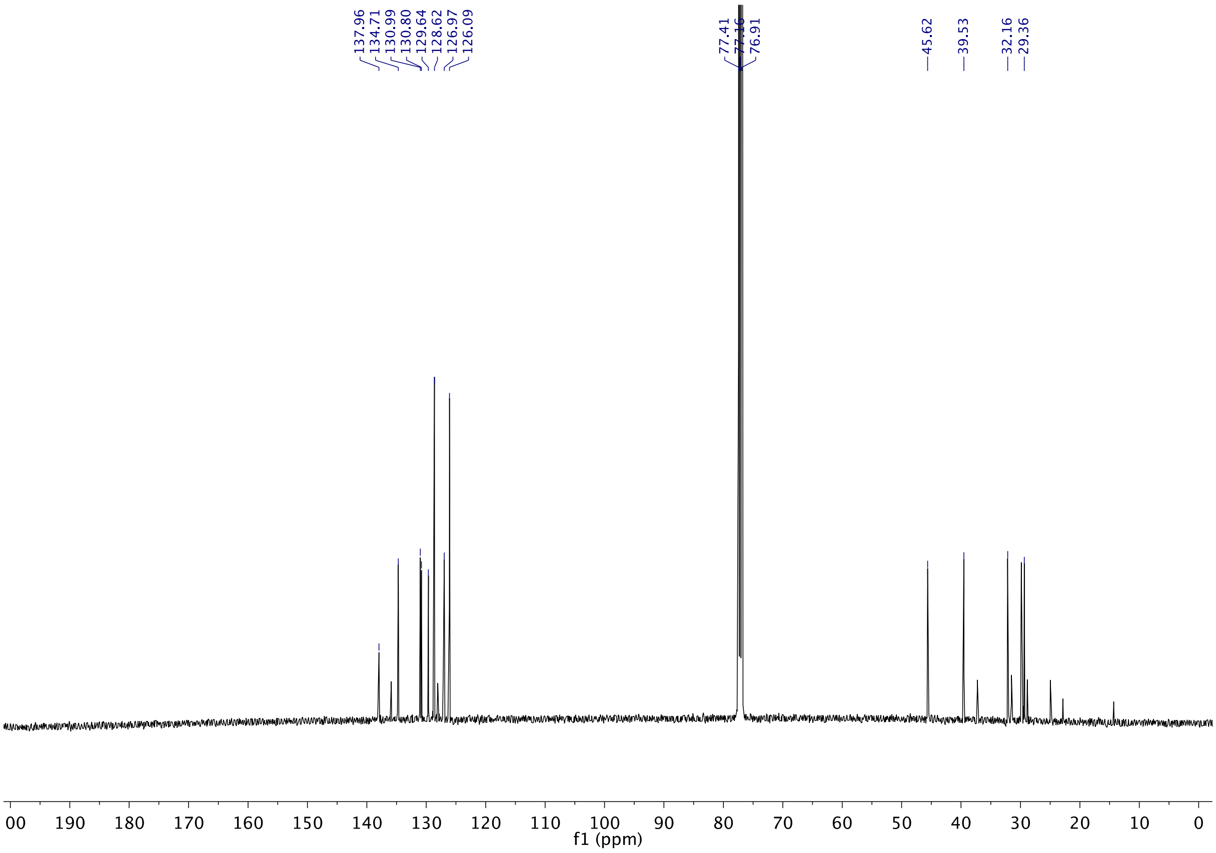

(*E*)-Hex-1-en-5-yn-1-ylbenzene, **22**

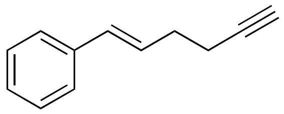

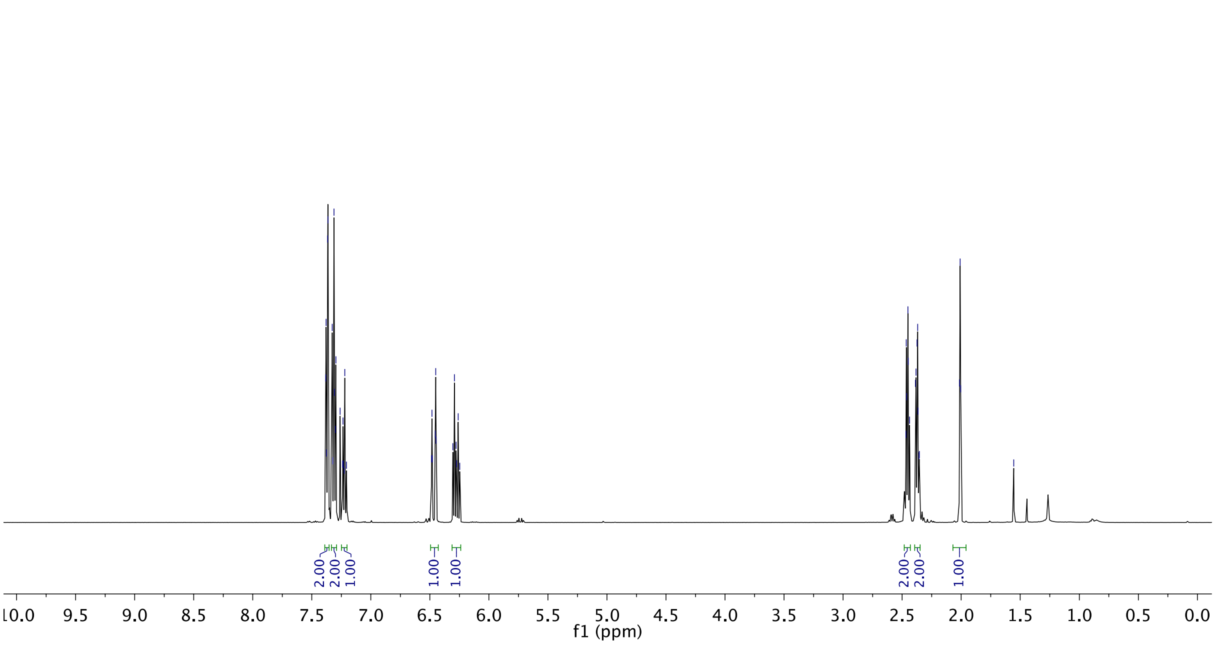

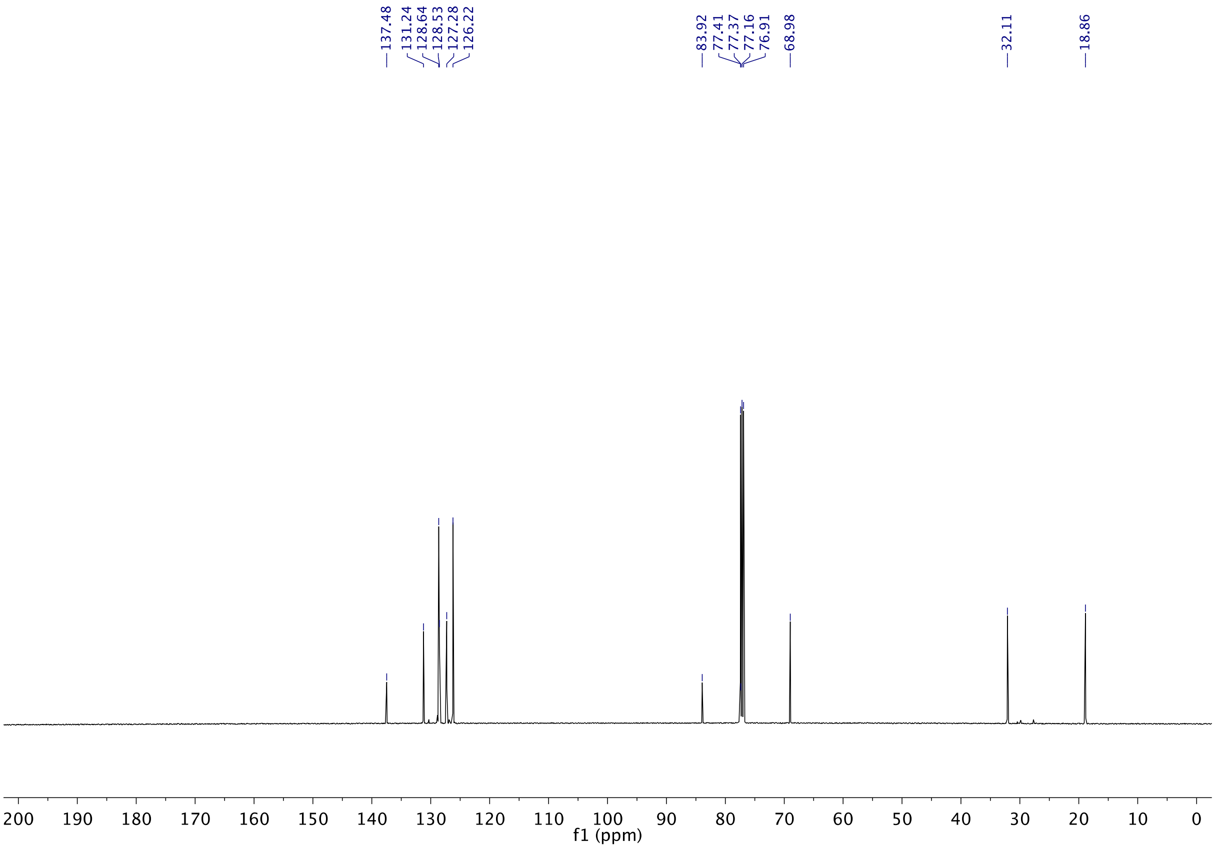

tert-Butyl cinnamylcarbamate, **23**

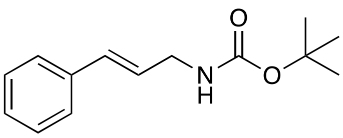

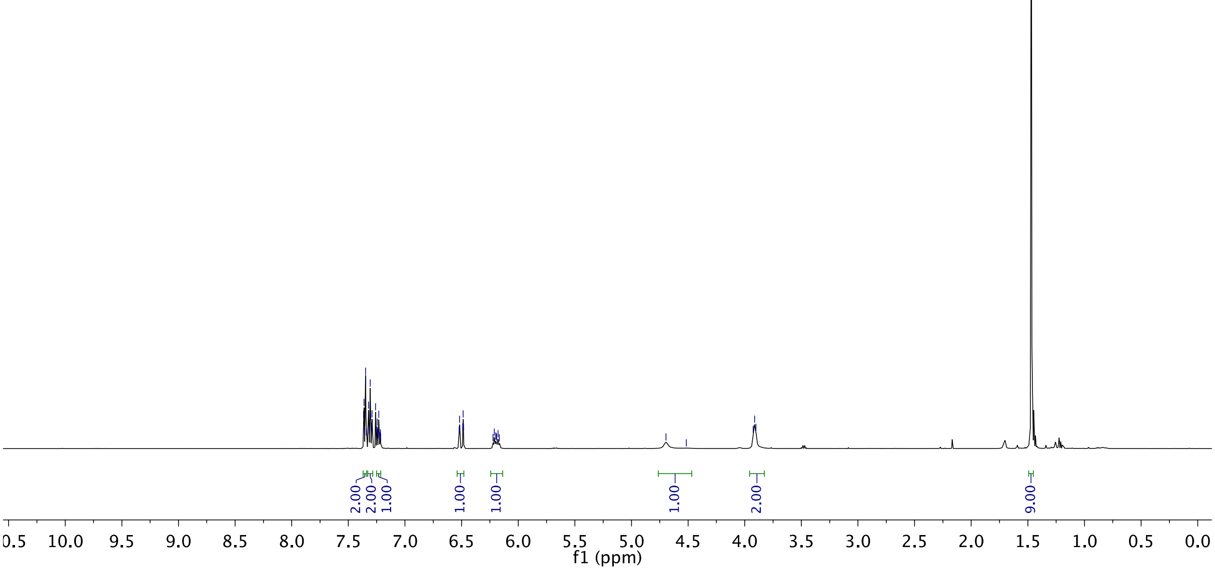

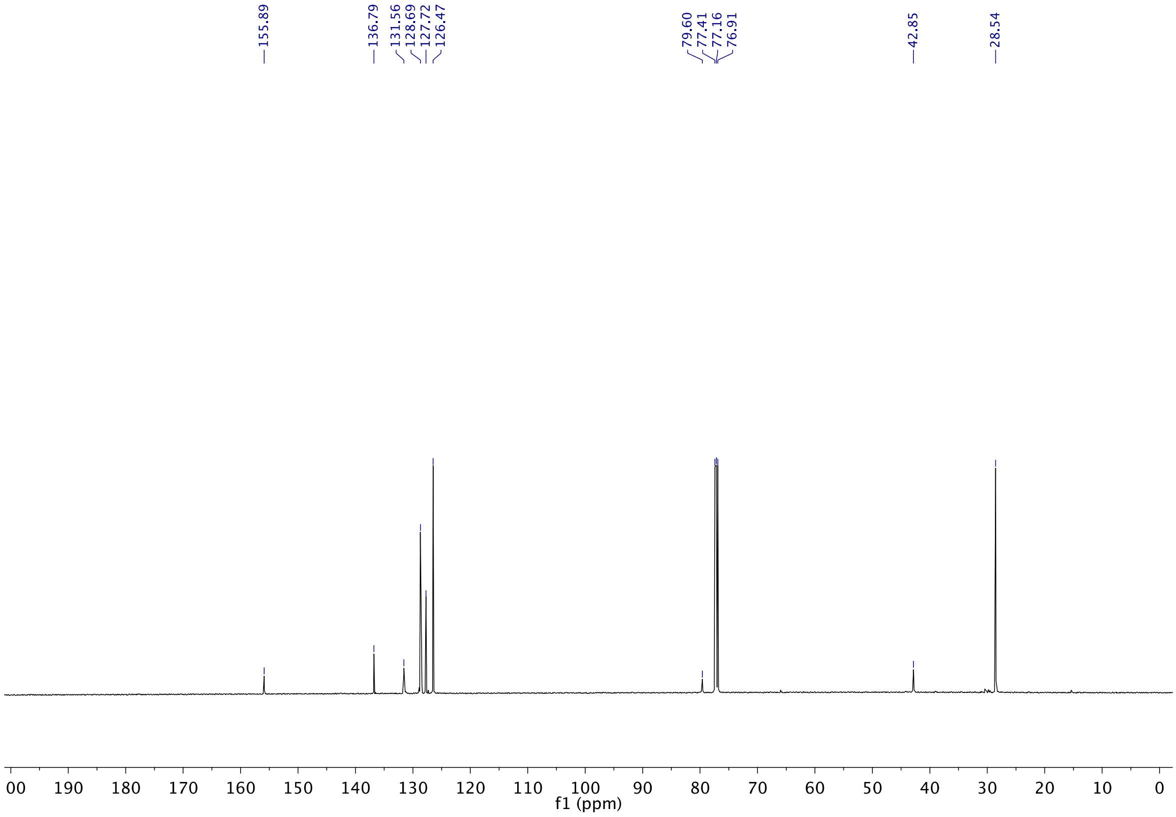

tert-Butyl (*E*)-methyl(4-phenylbut-3-en-2-yl)carbamate, **24**

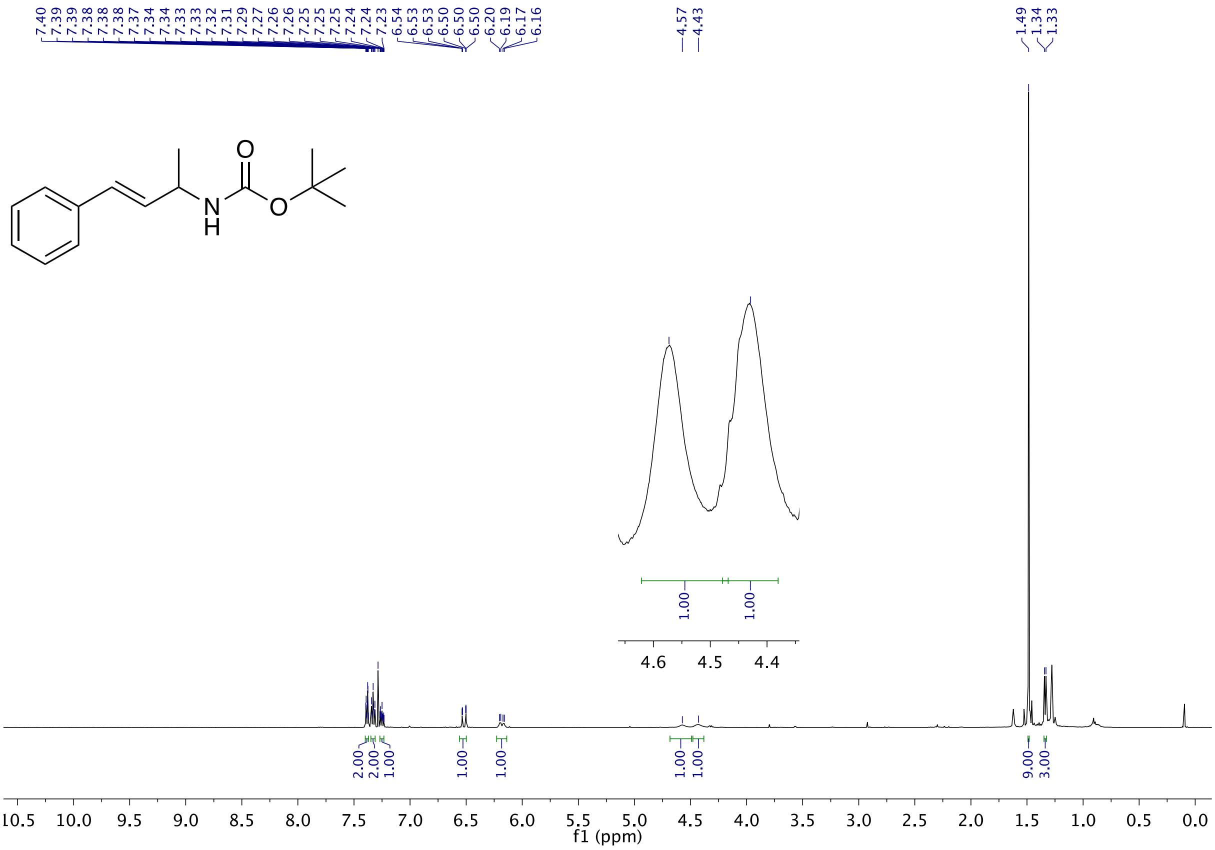

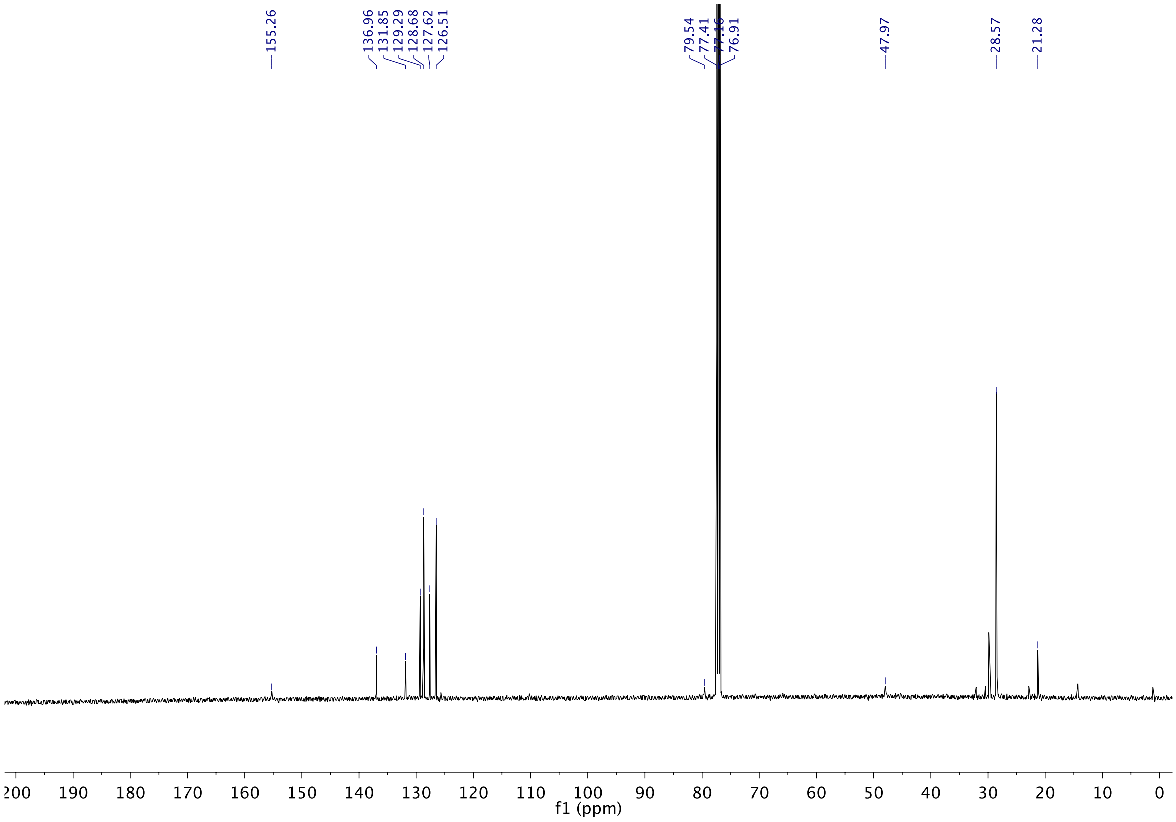

(*E*)-*tert*-Butyl((2,2-dimethyl-4-phenylbut-3-en-1-yl)oxy)dimethylsilane, **25**

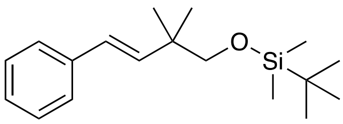

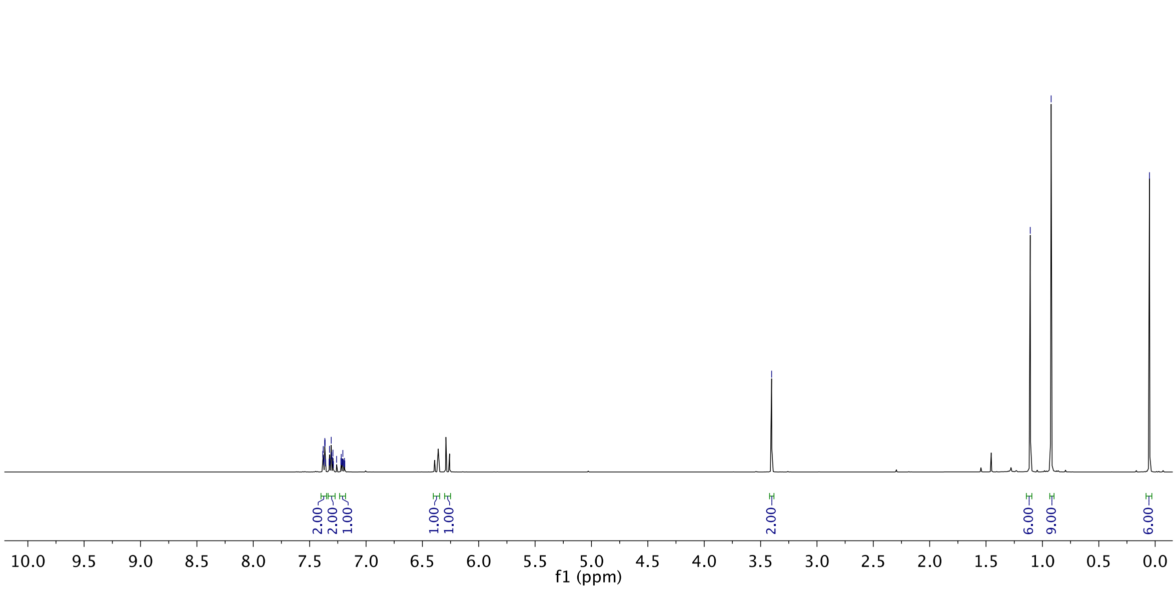

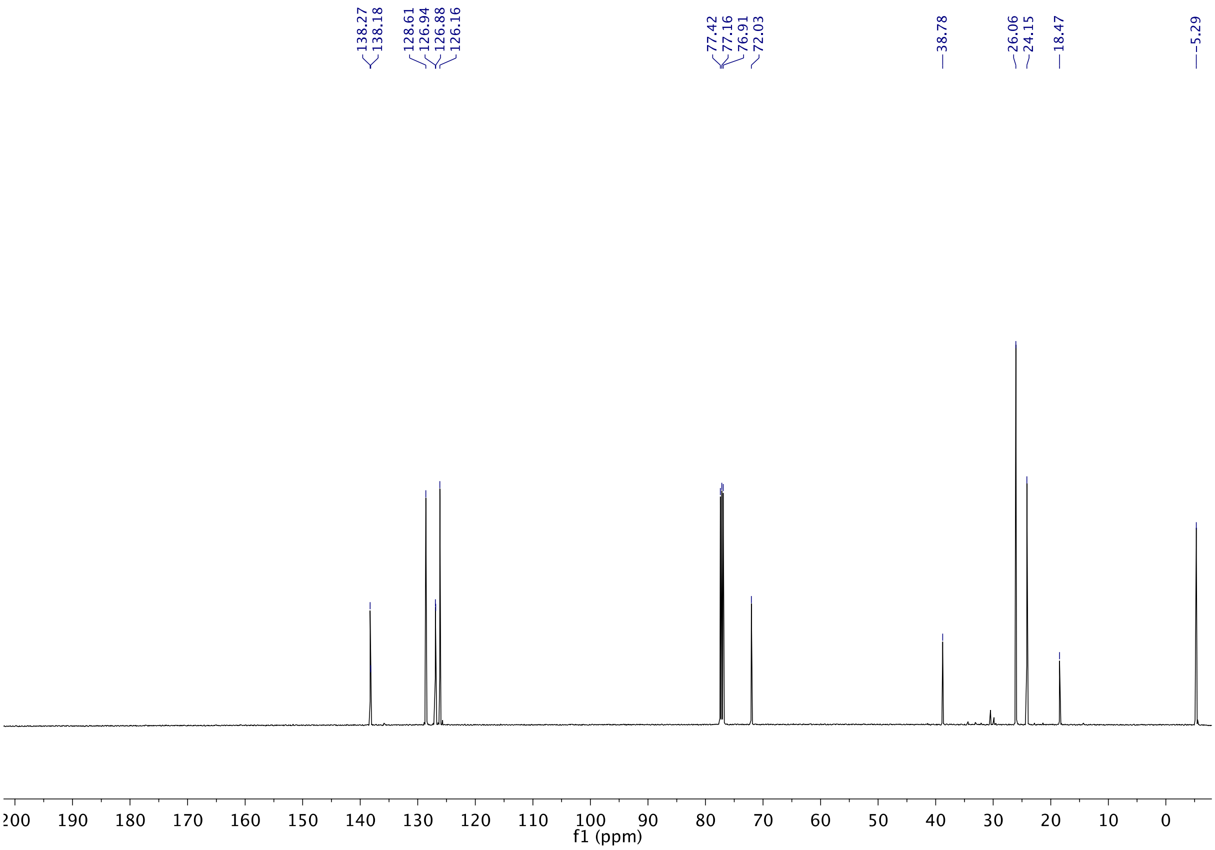

(*E*)-3-Methyl-3-styryloxetane, **26**

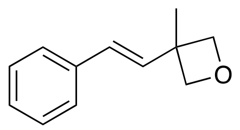

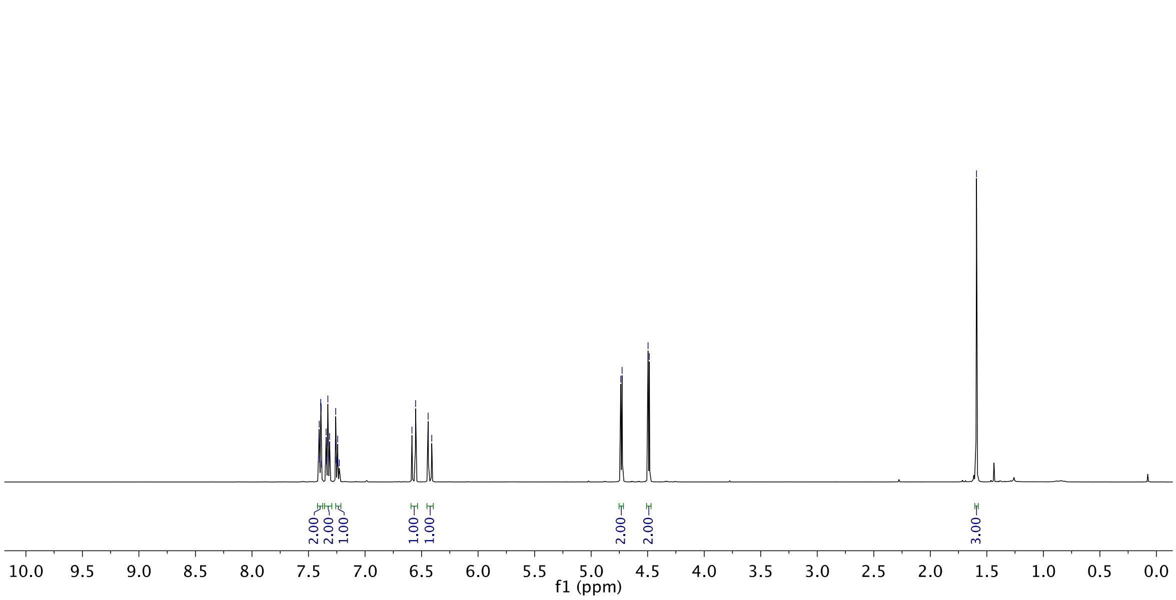

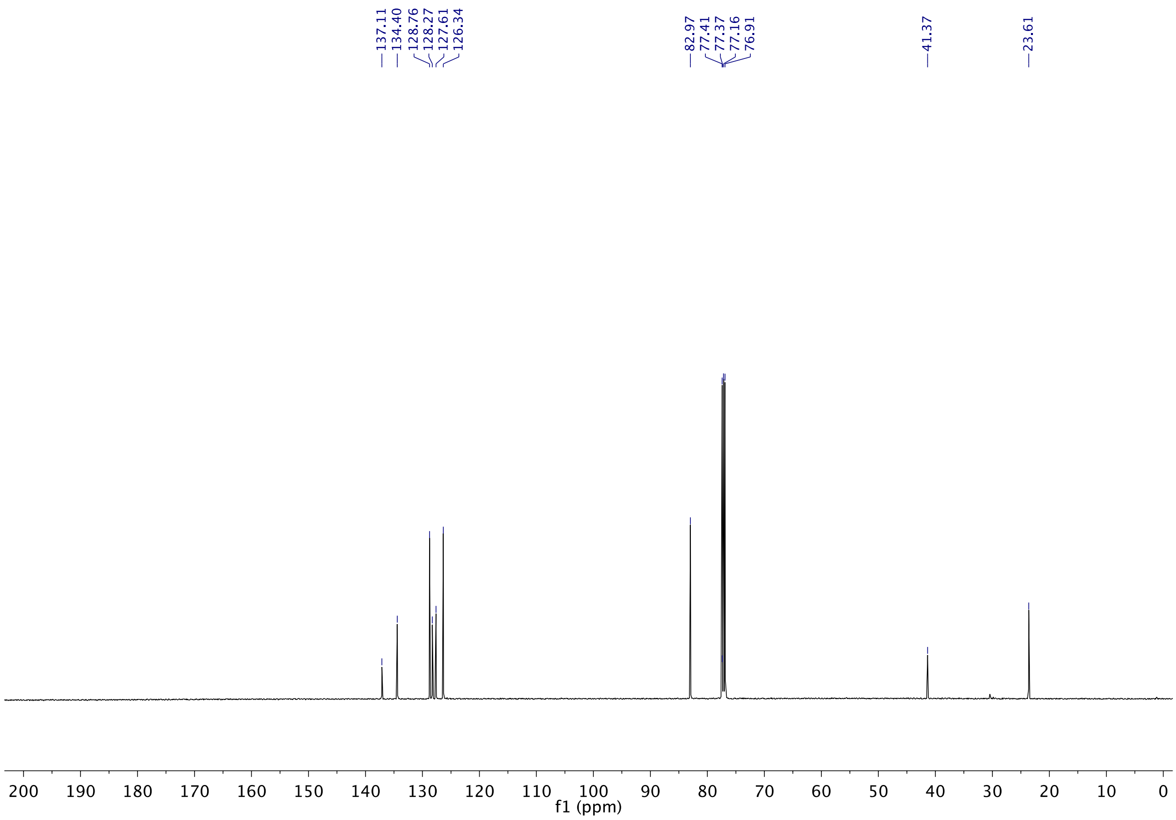

(*E*)-3-Styrylcyclobutan-1-one, **27**

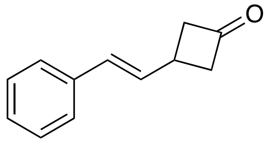

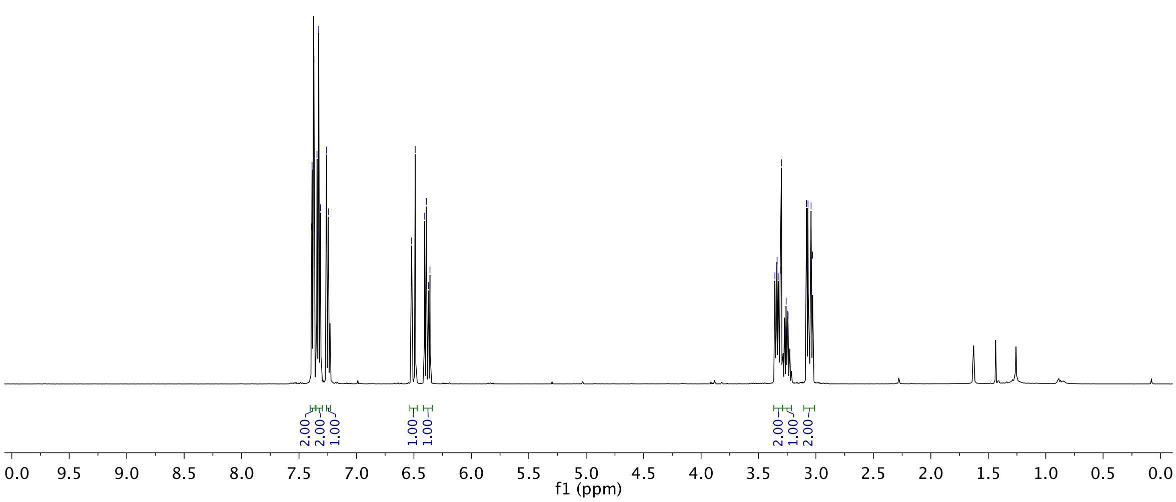

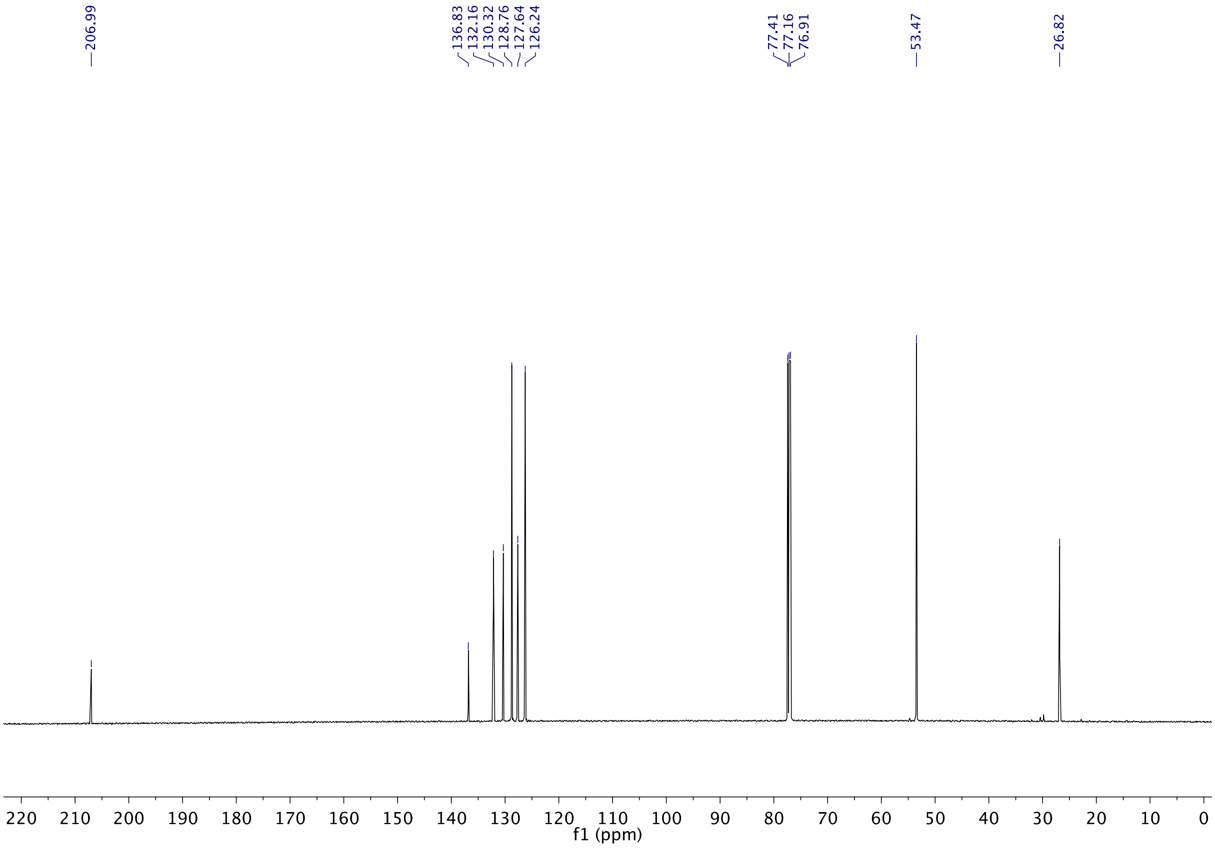

tert-Butyl (*E*)-4-styrylpiperidine-1-carboxylate, **28**

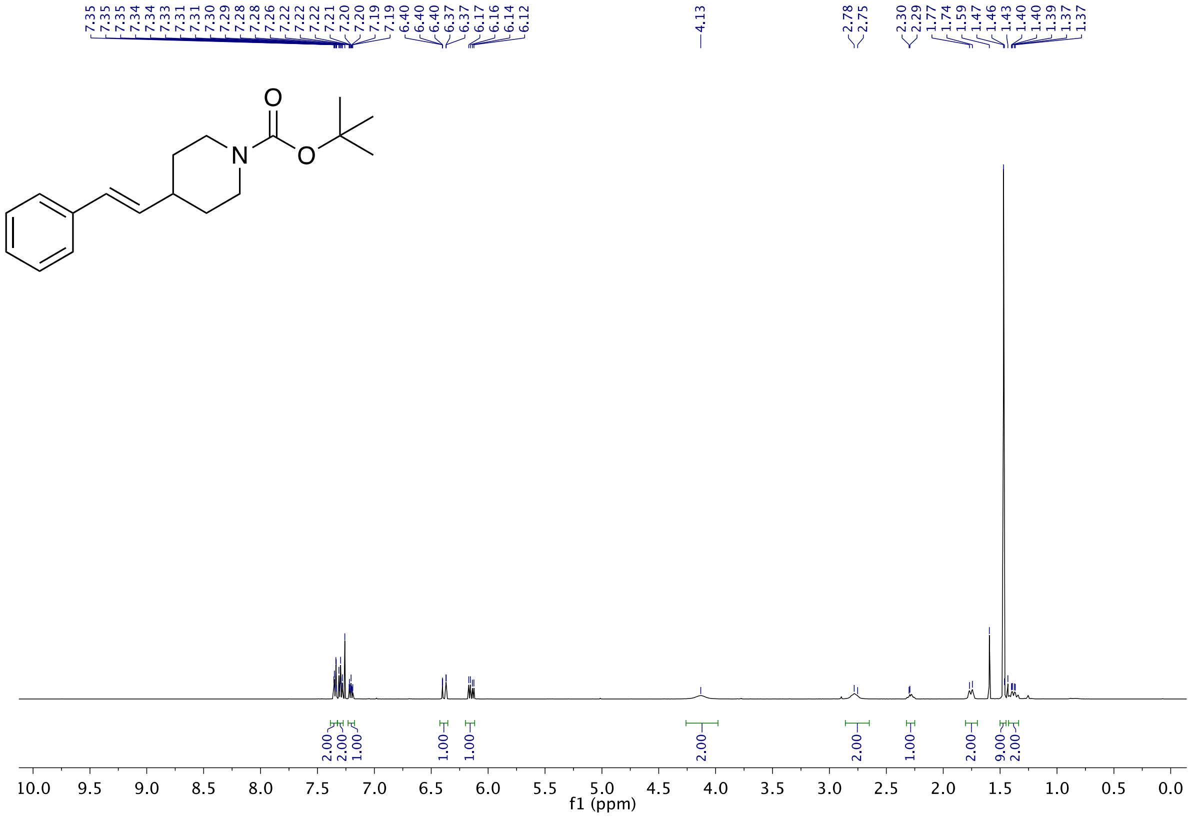

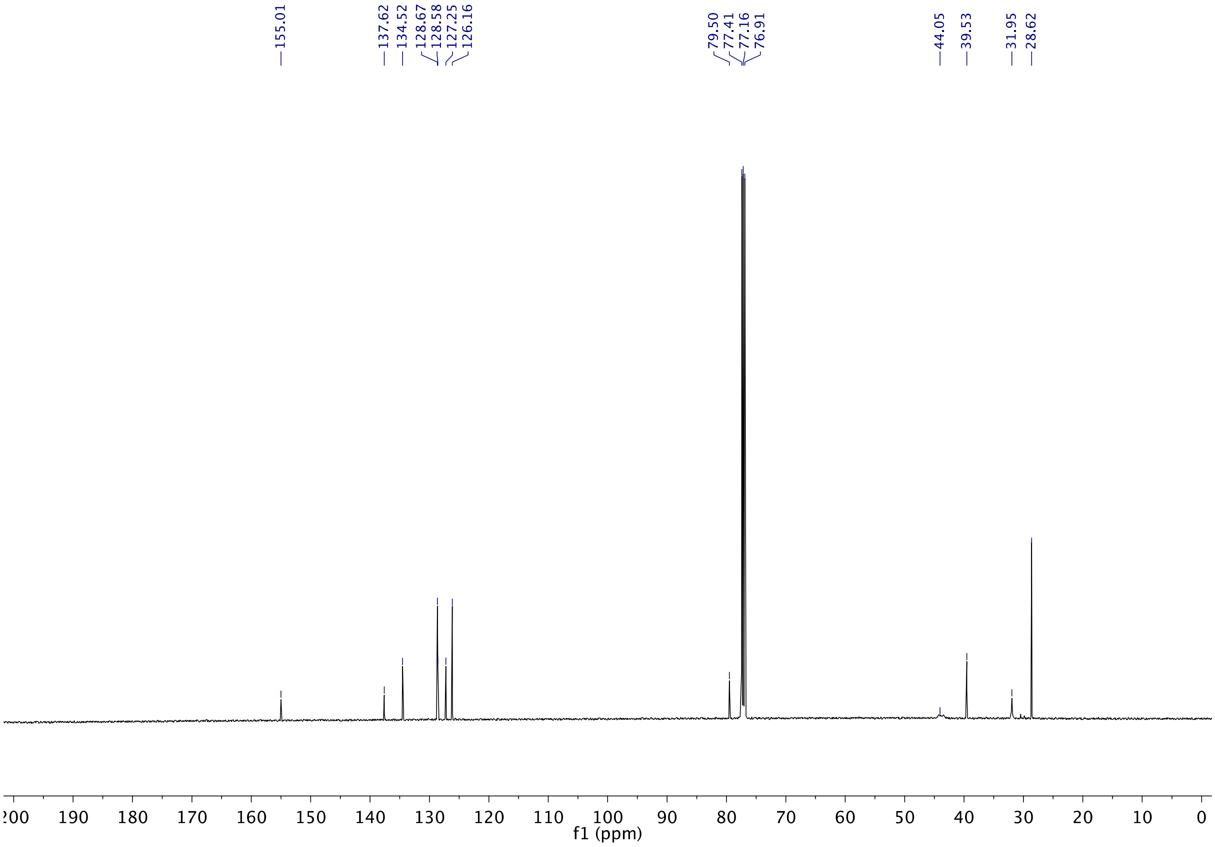

tert-Butyl (*E*)-3-styrylpiperidine-1-carboxylate, **29**

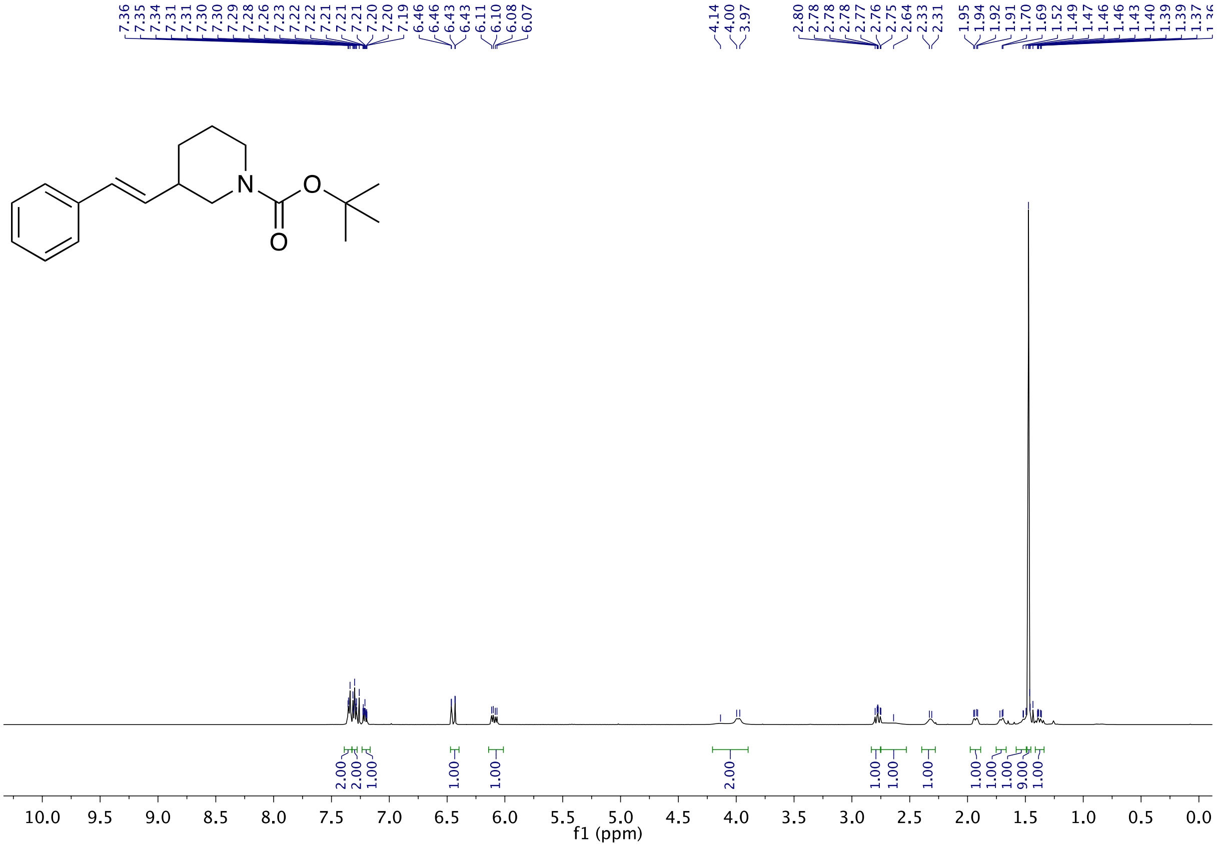

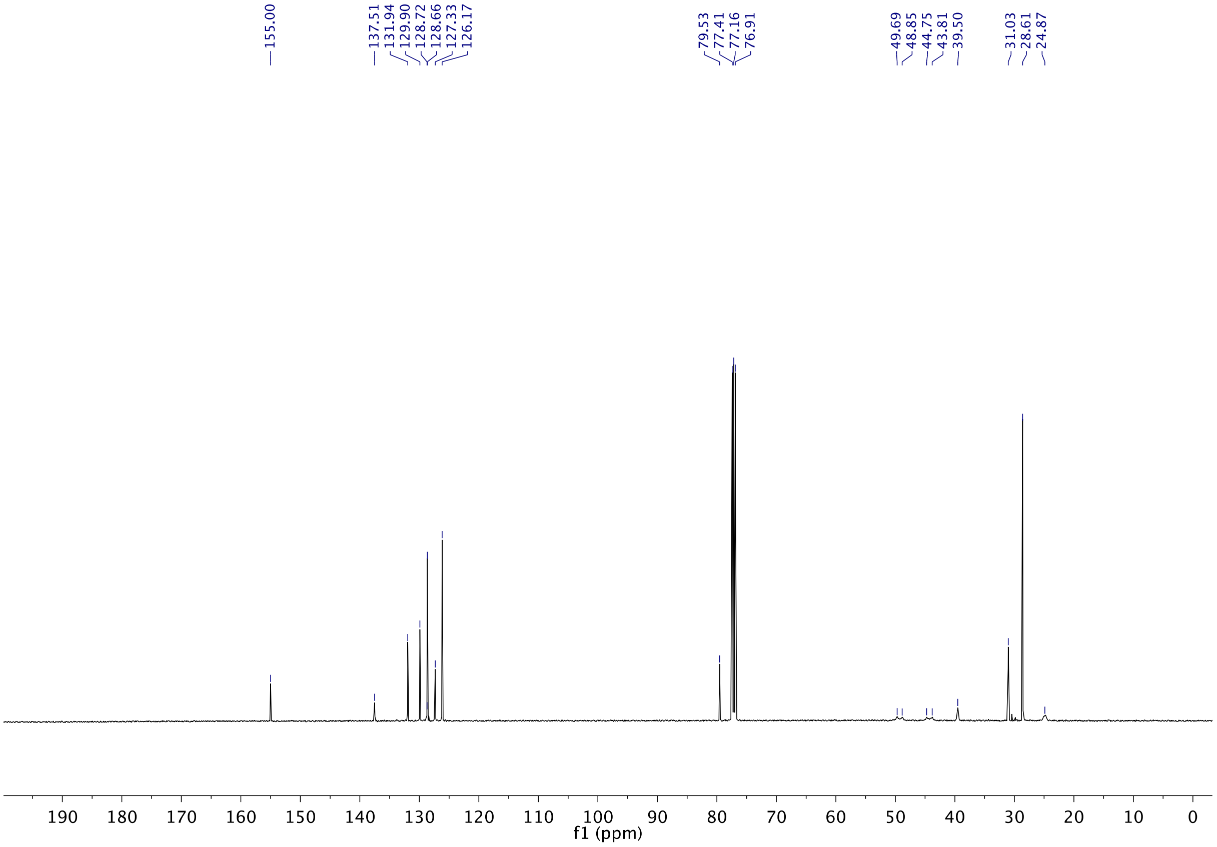

tert-Butyl (*E*)-2-styrylpyrrolidine-1-carboxylate, **30**

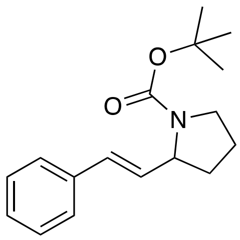

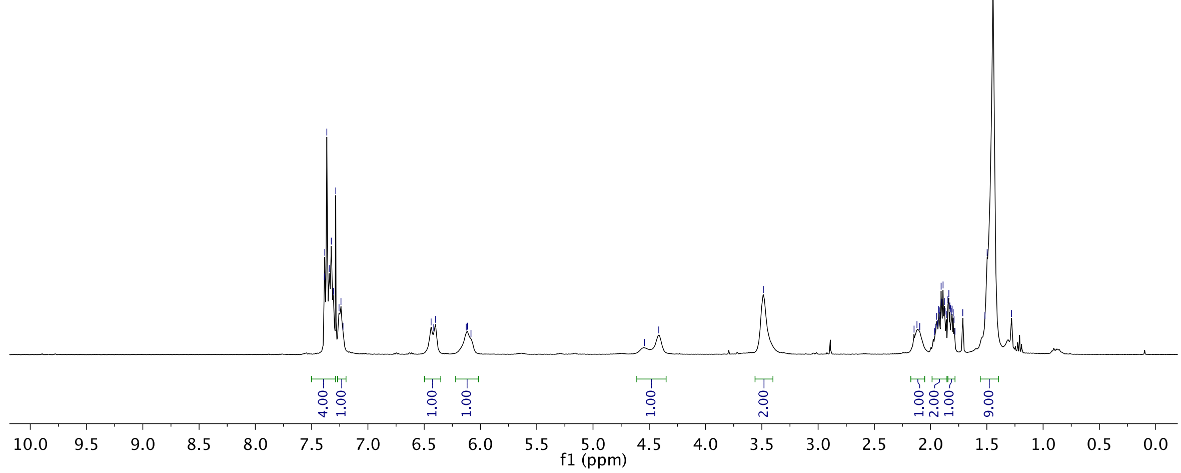

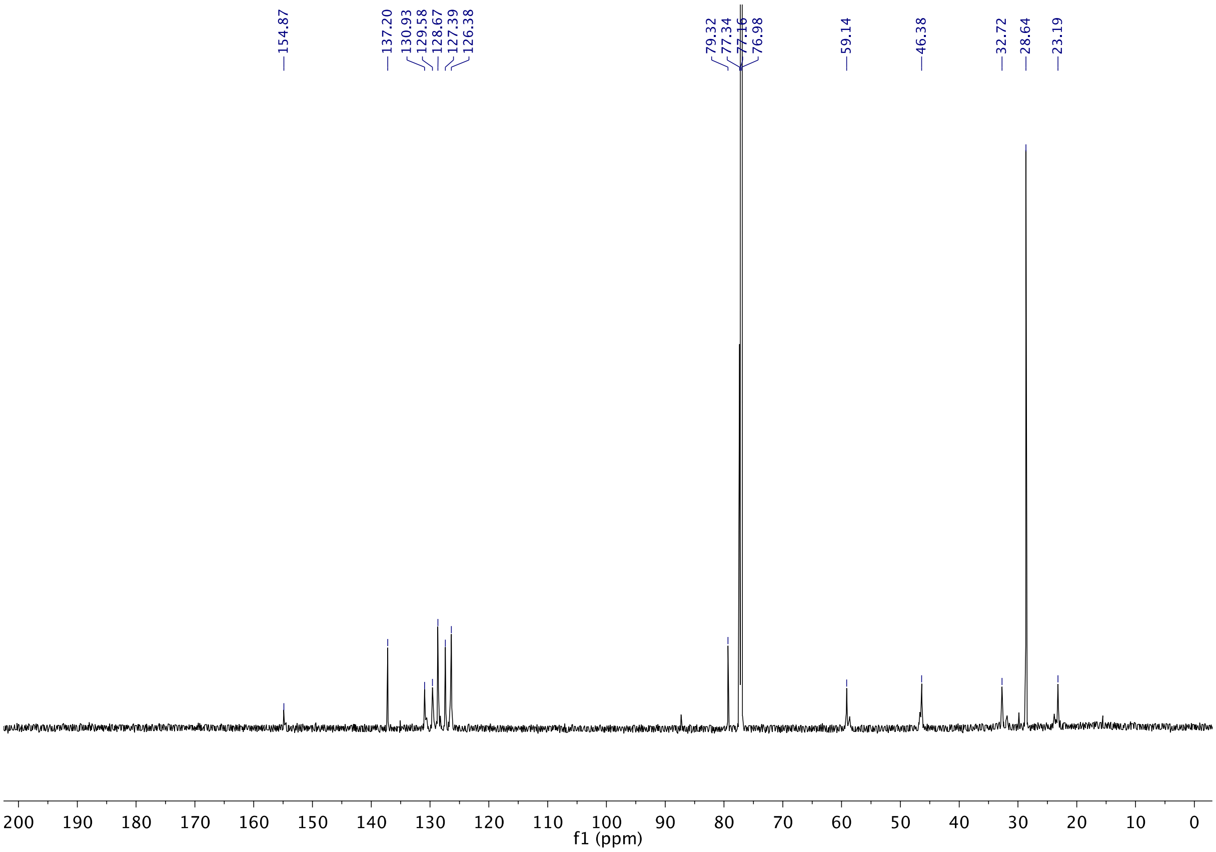

tert-Butyl (*E*)-2-styrylpiperidine-1-carboxylate, **31**

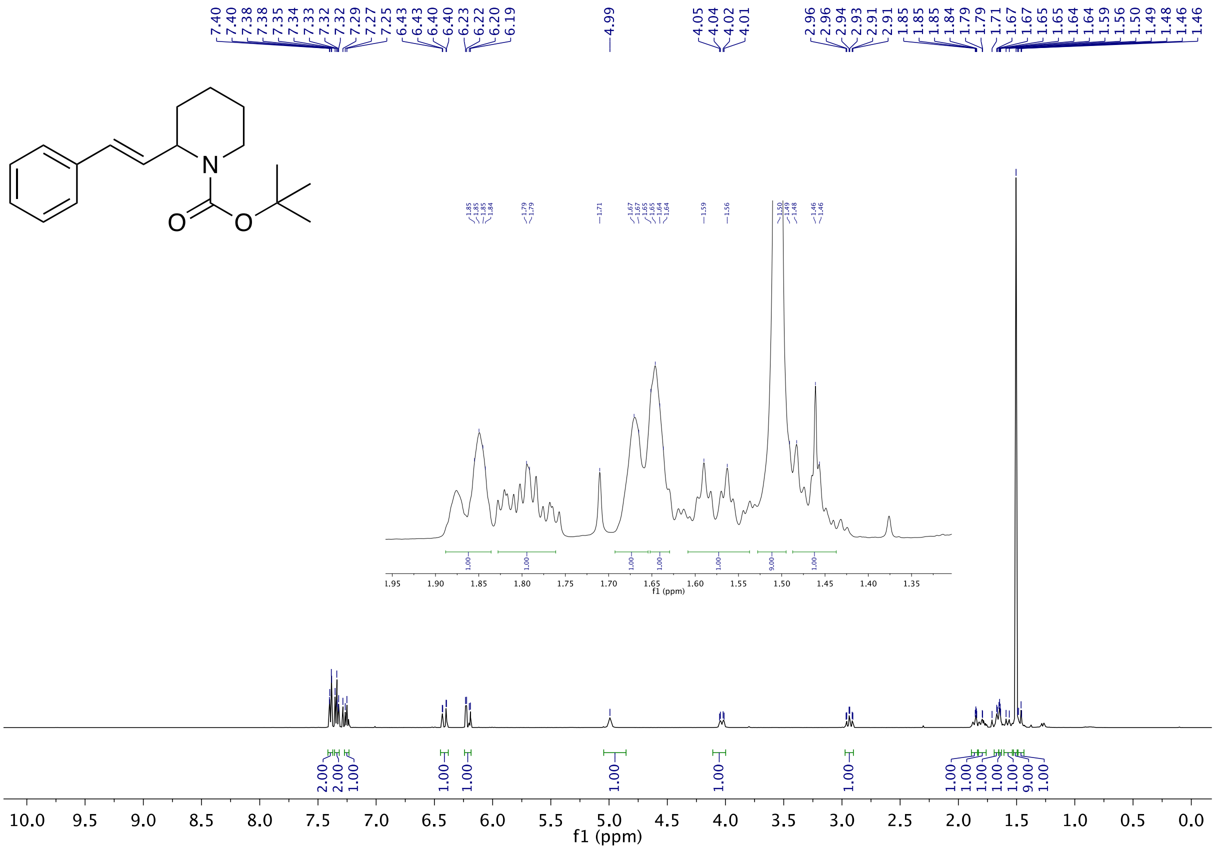

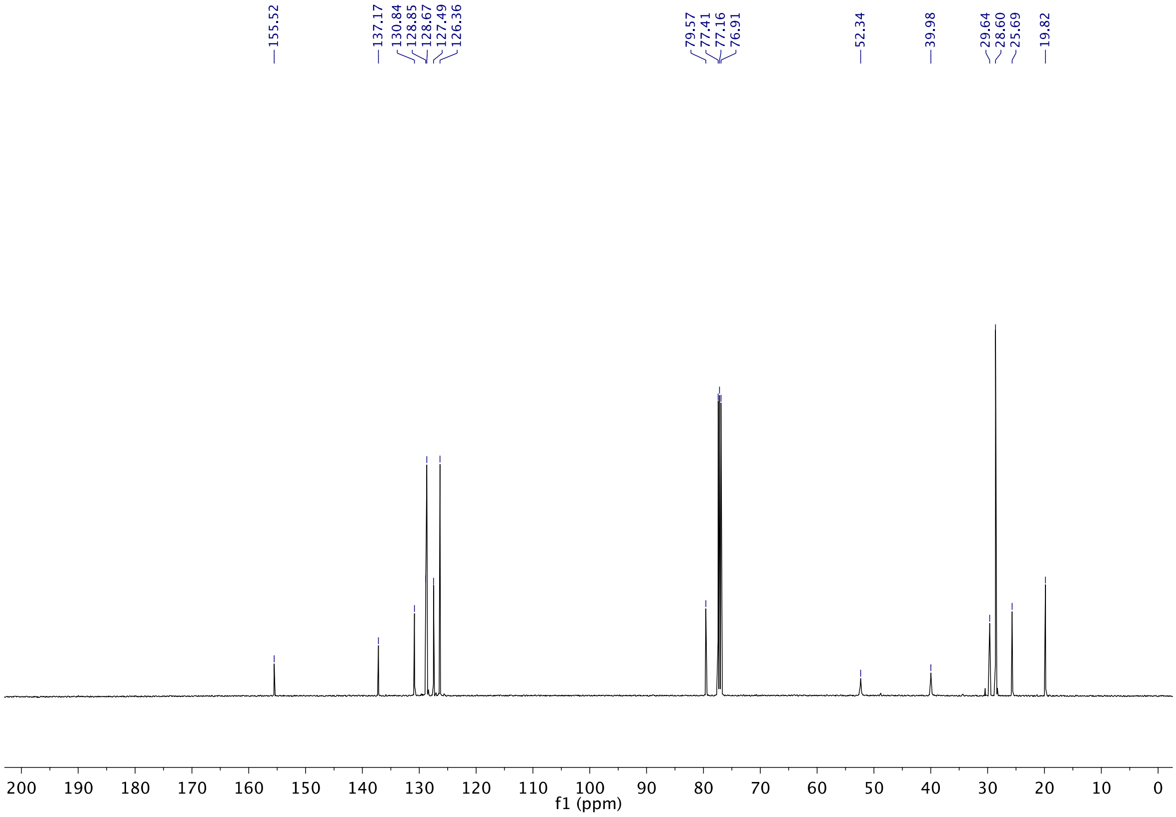

(*E*)-3-Styryltetrahydrofuran, **32**

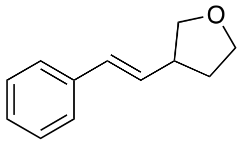

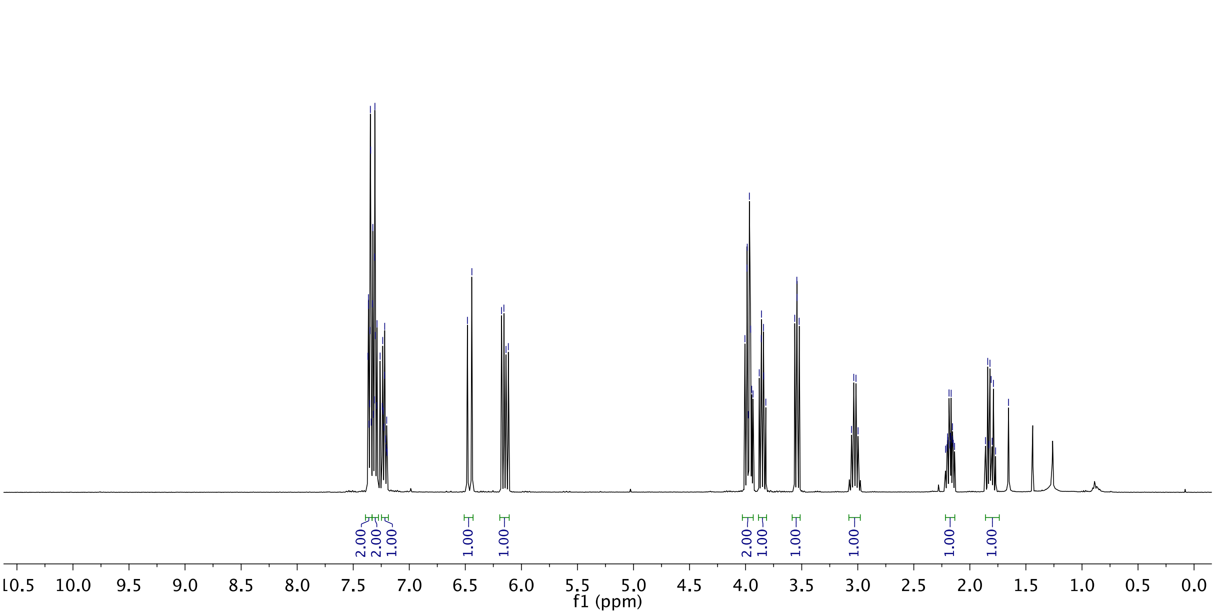

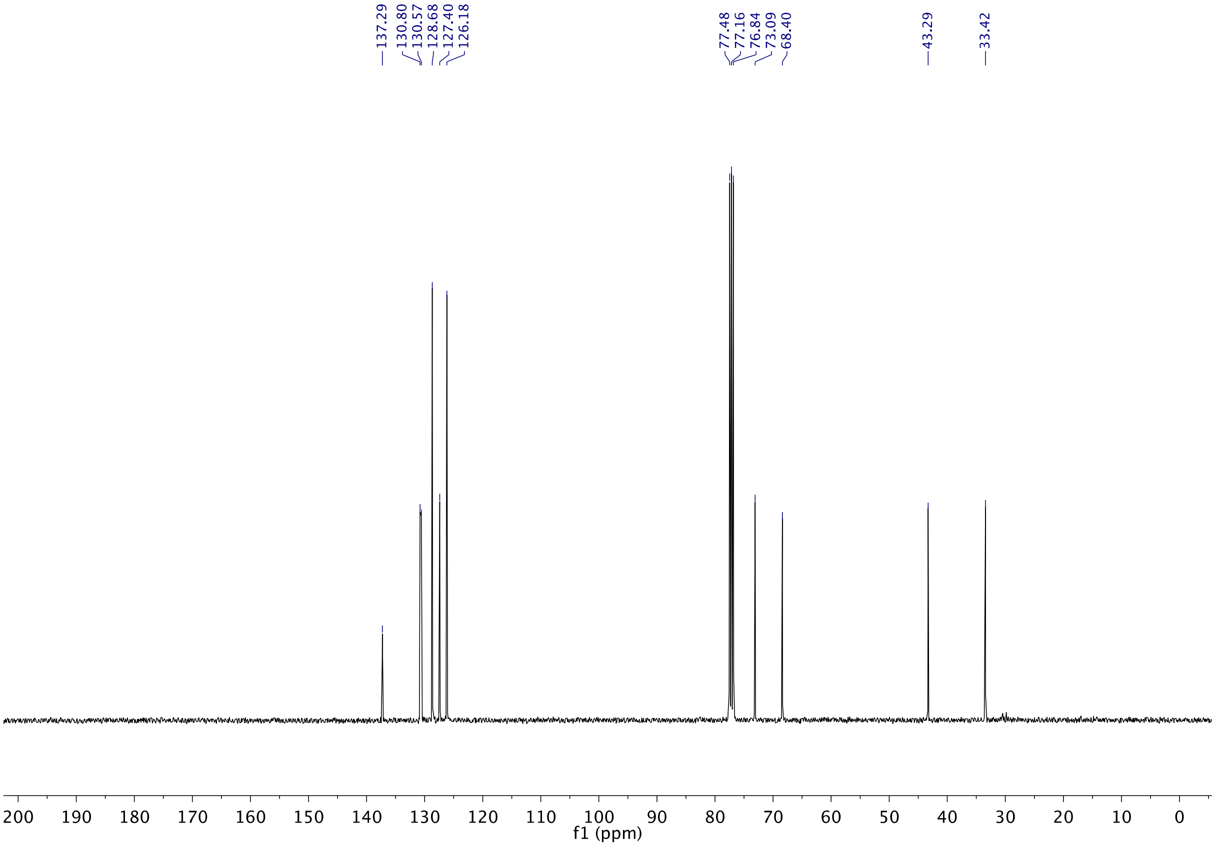

(*E*)-2-Styryltetrahydrofuran, **33**

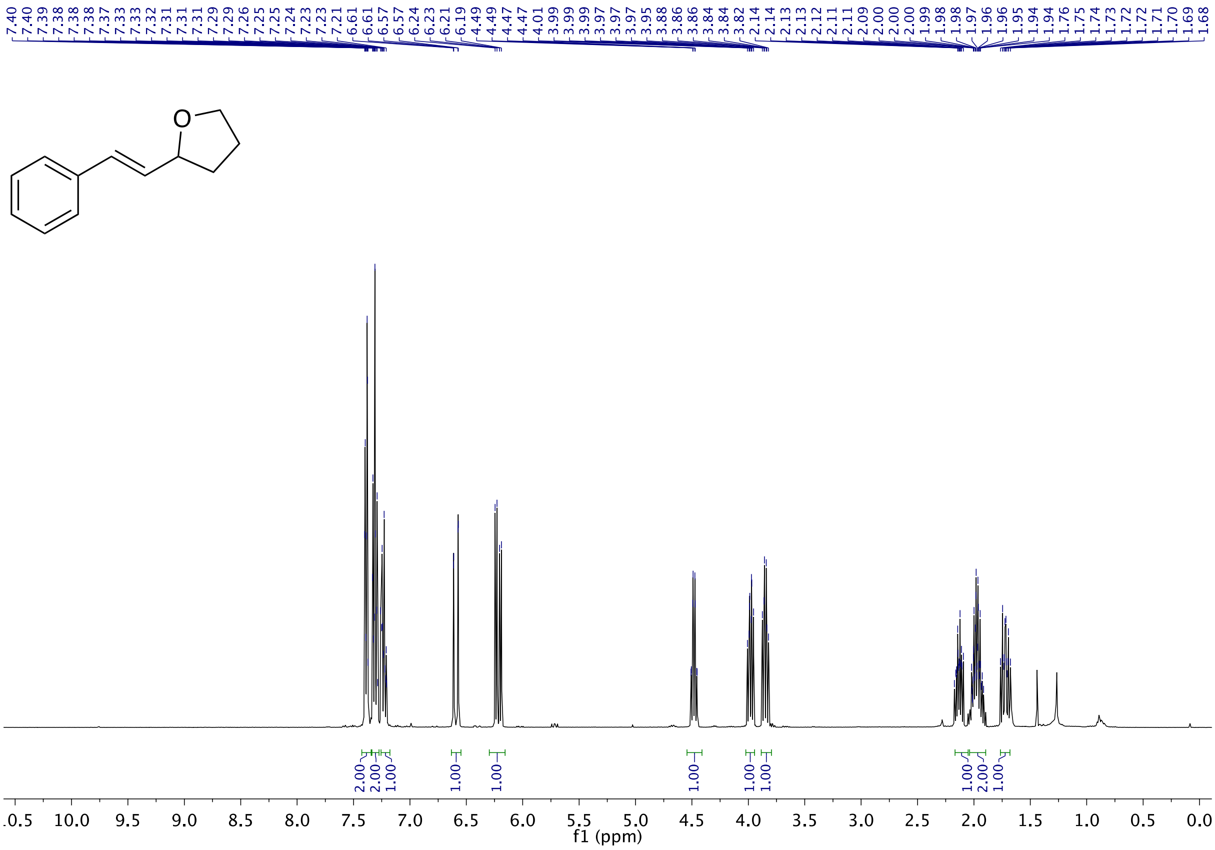

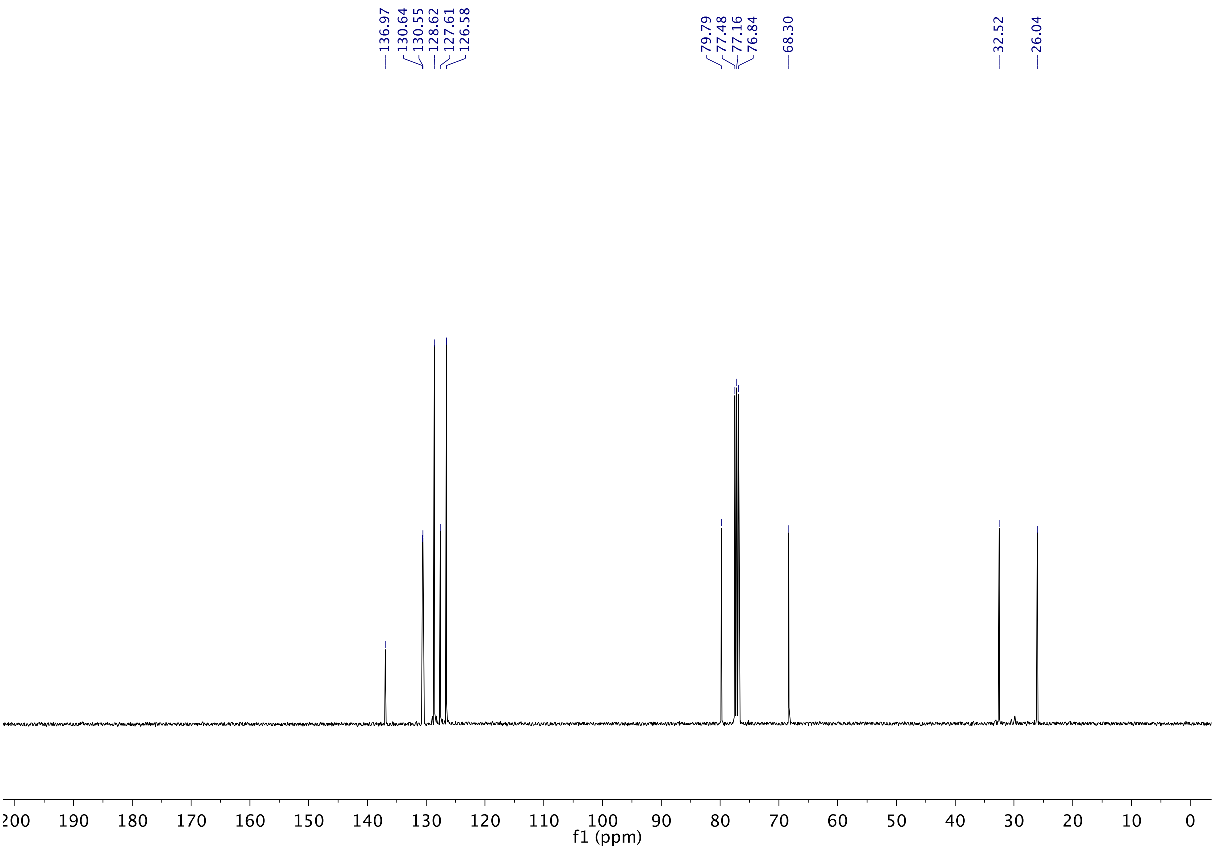

(*E*)-2-Styryltetrahydro-2*H*-pyran, **34**

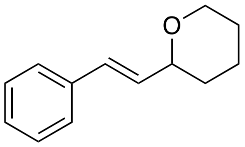

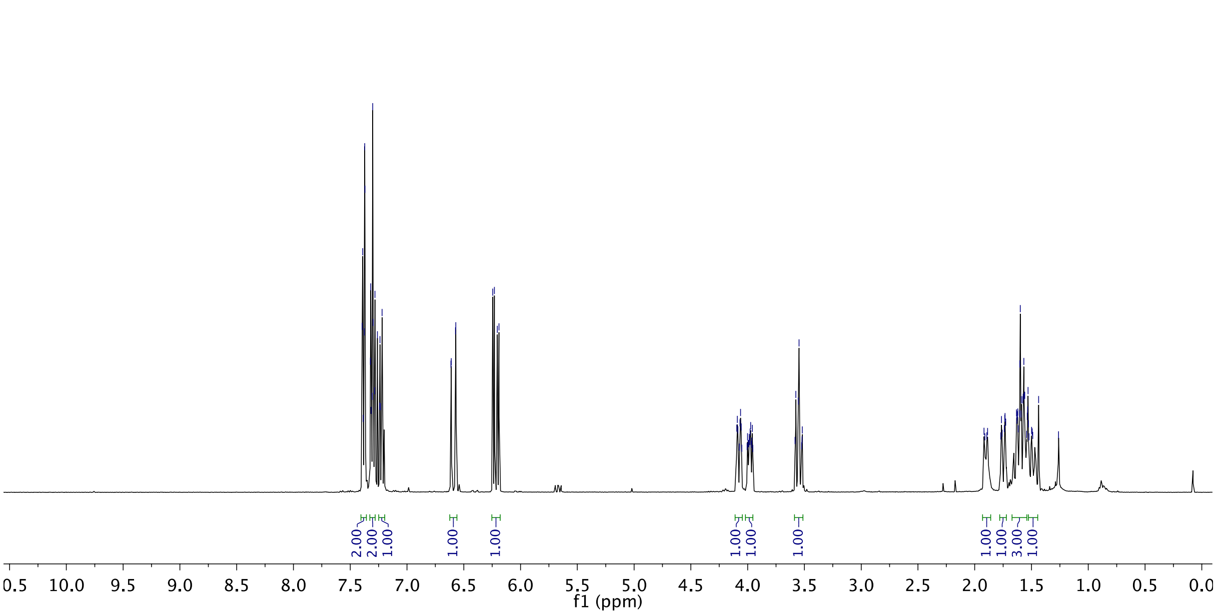

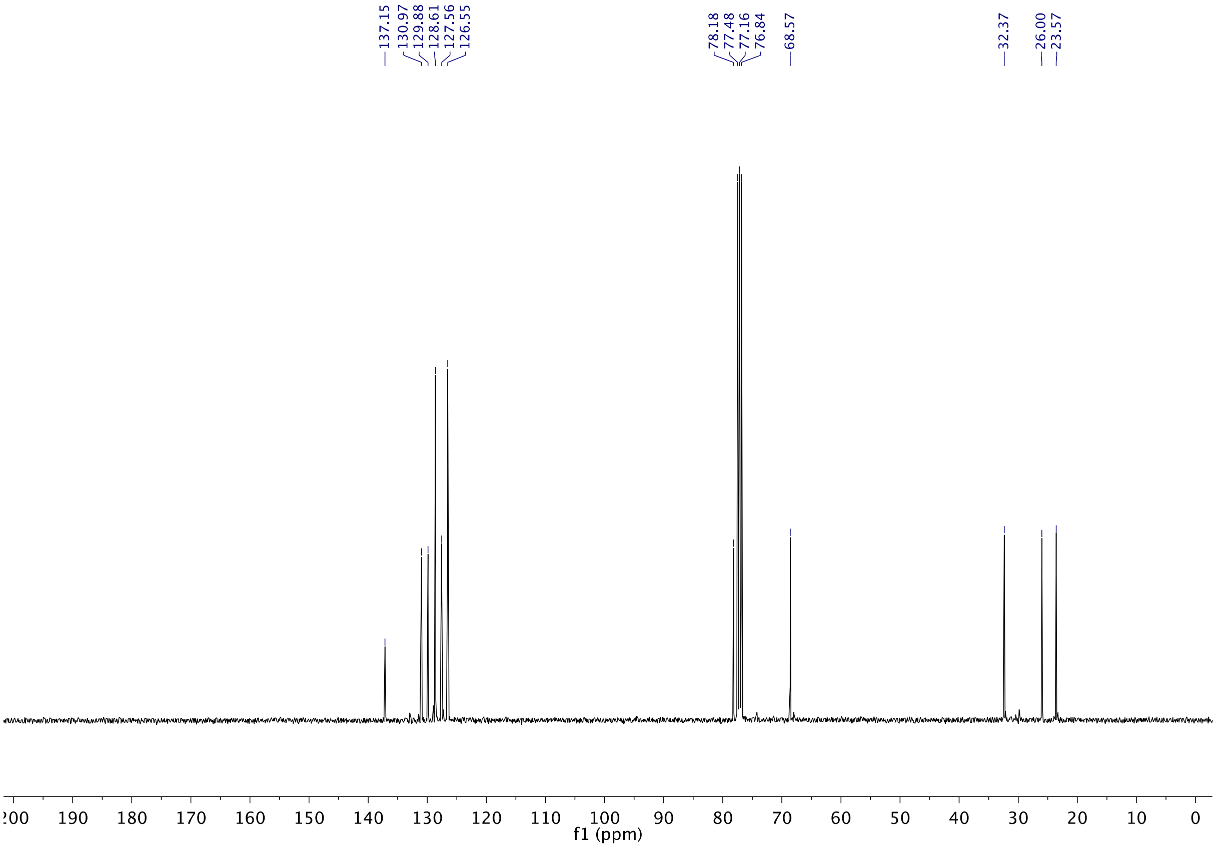

(*E*)-2-(4-Phenylbut-3-en-1-yl)furan, **35**

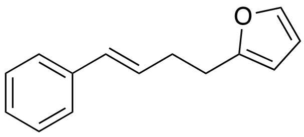

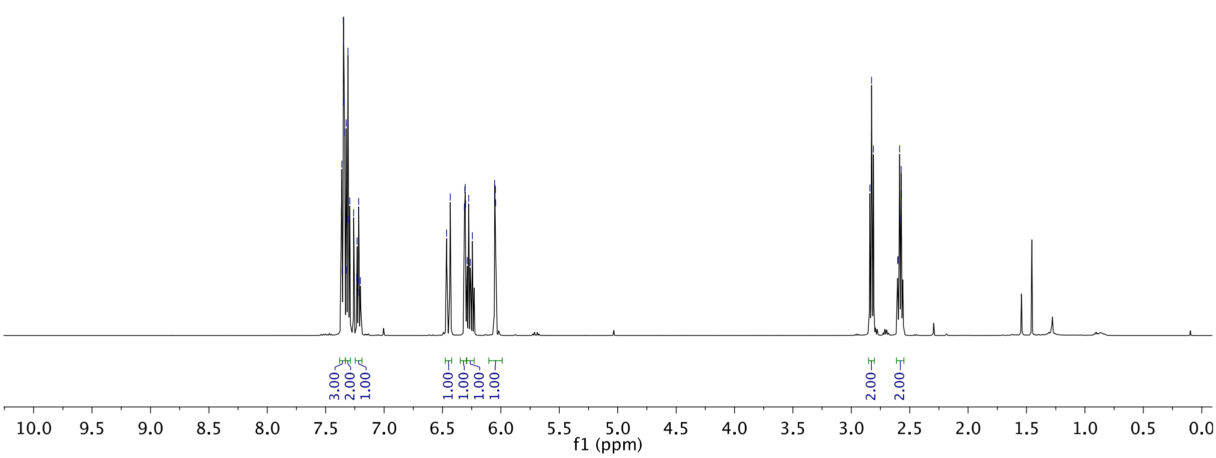

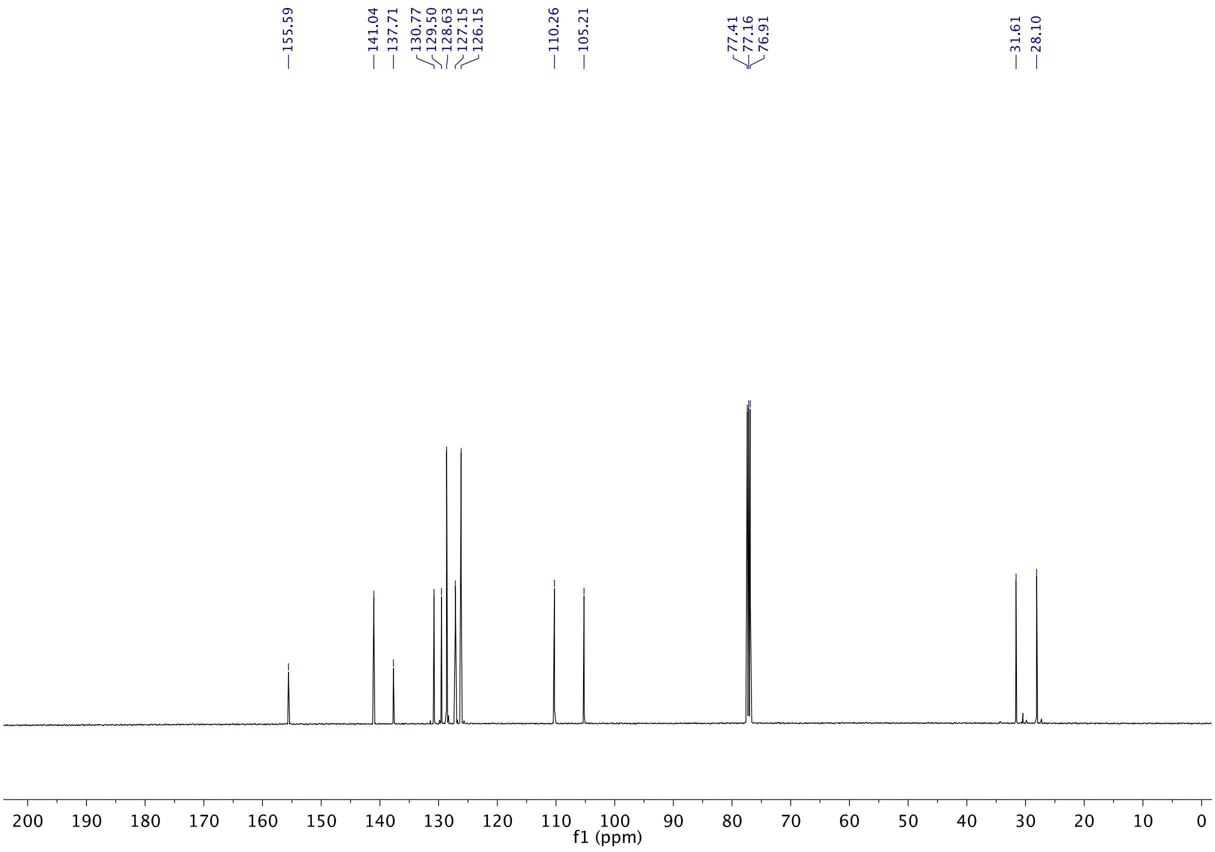

(*E*)-3-(4-Phenylbut-3-en-1-yl)-1*H*-indole, **36**

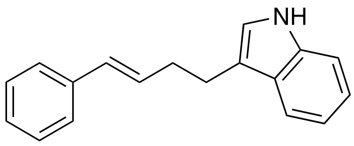

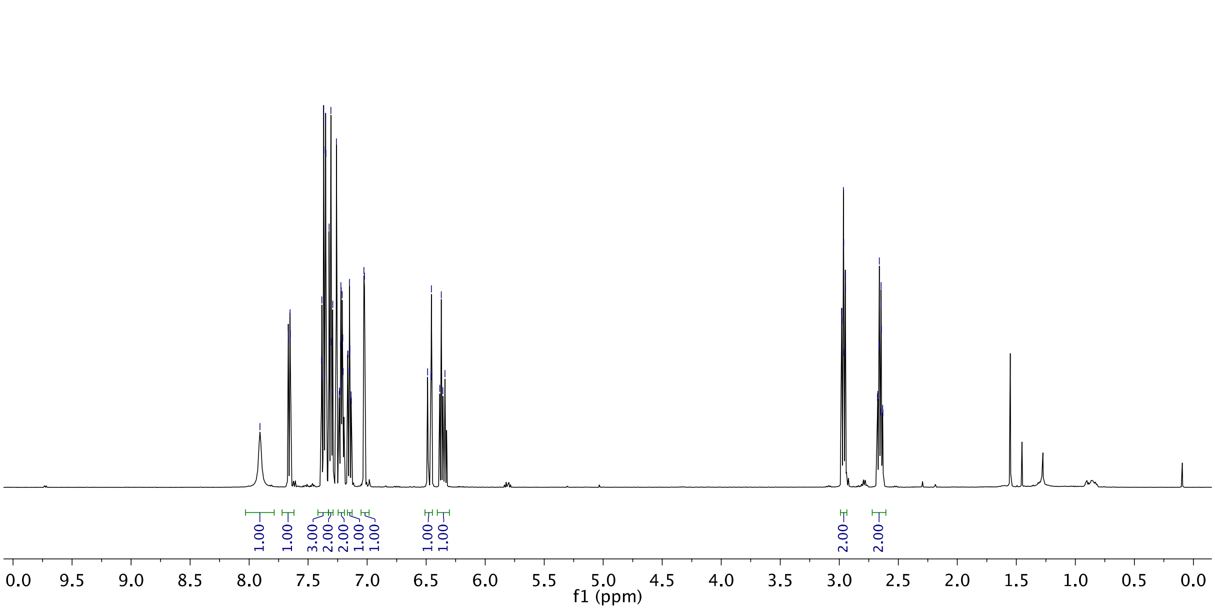

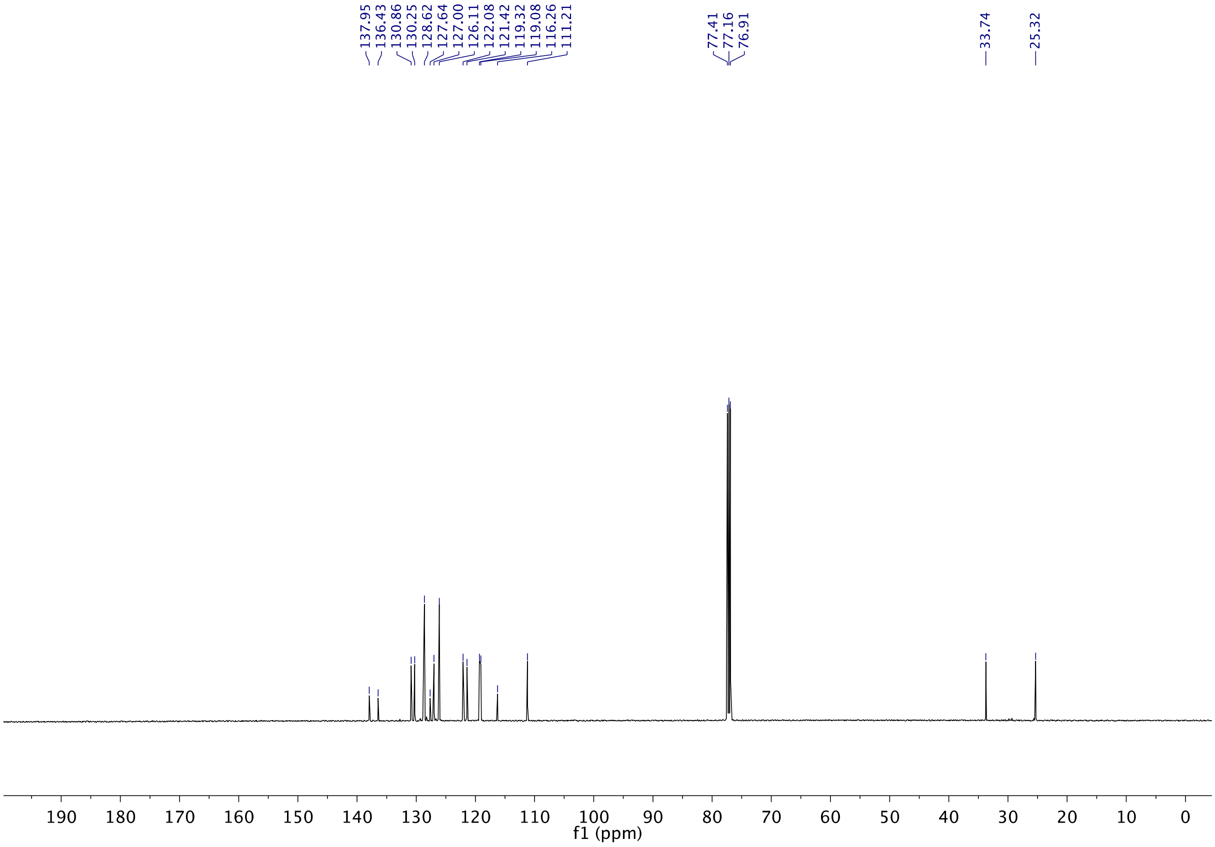

tert-Butyl (*E*)-3-(4-phenylbut-3-en-1-yl)-1*H*-indole-1-carboxylate, **37**

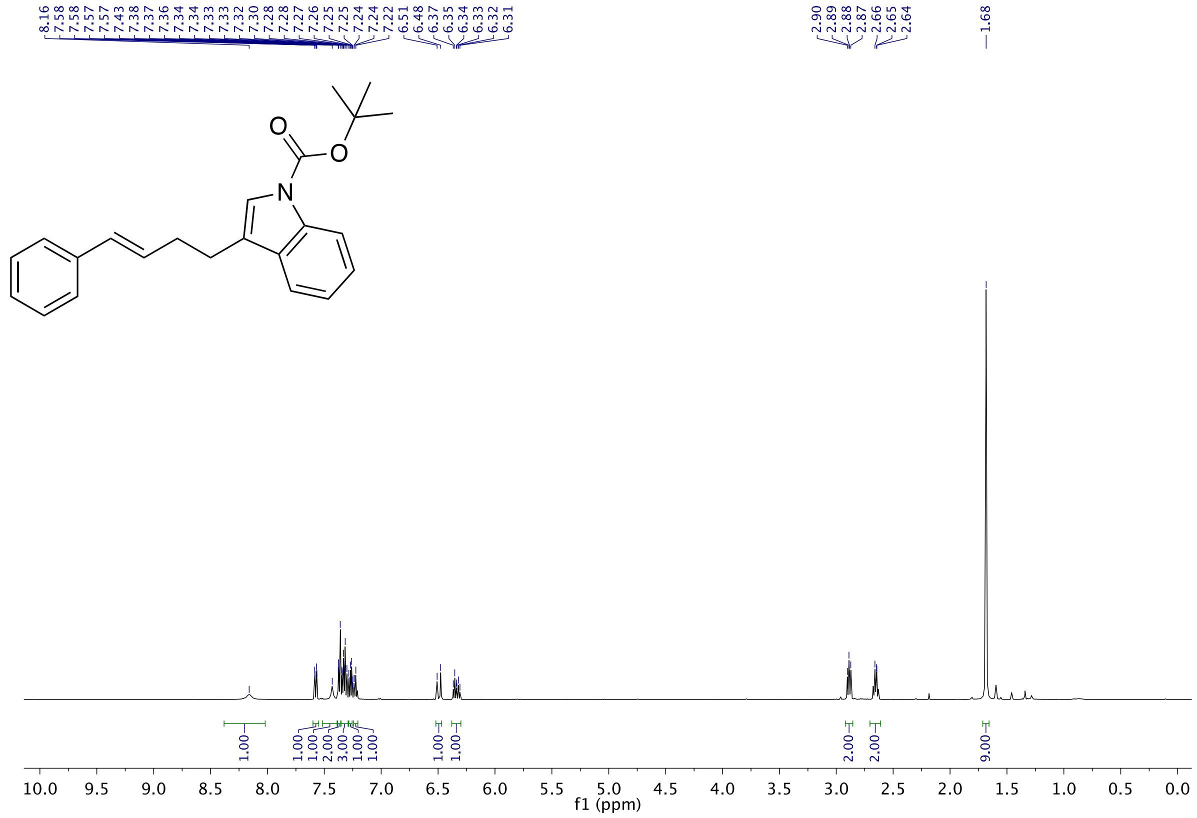

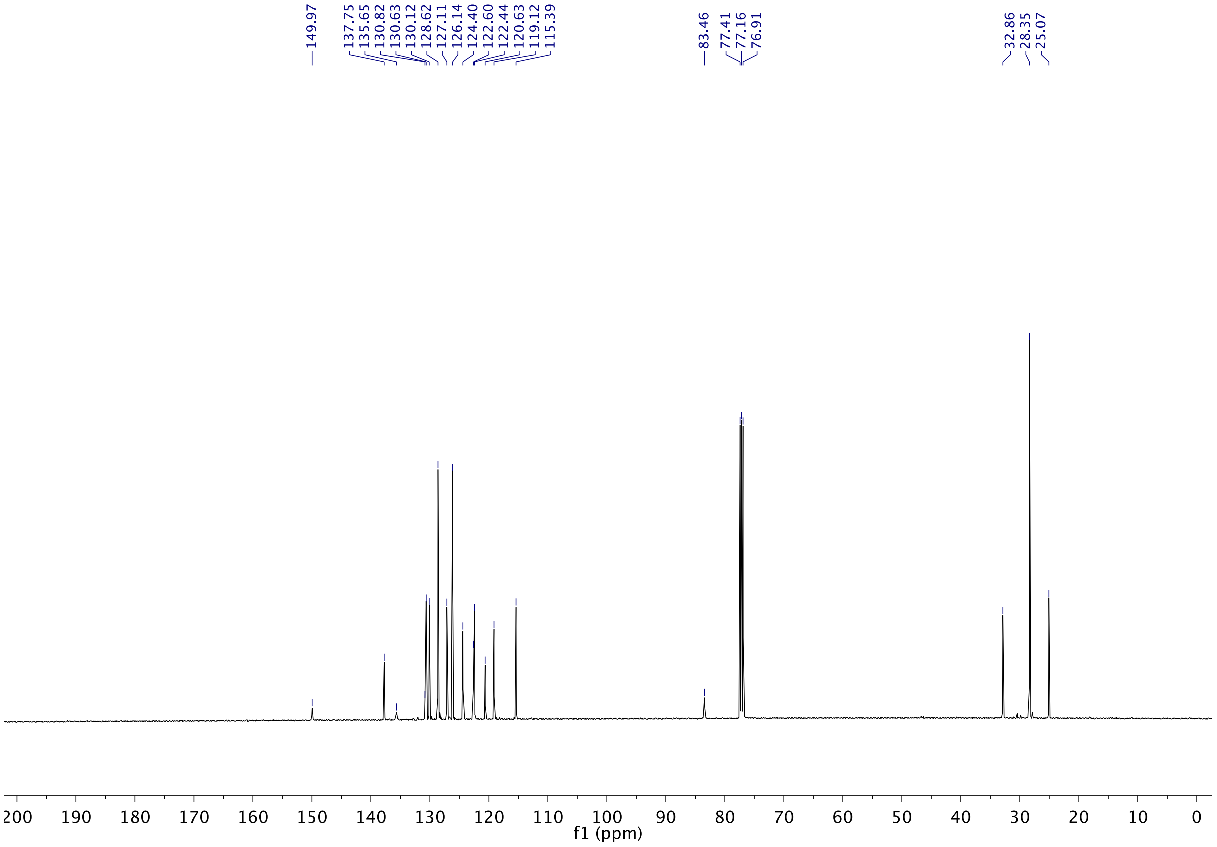

(*E*)-3-(4-Phenylbut-3-en-1-yl)pyridine, **38**

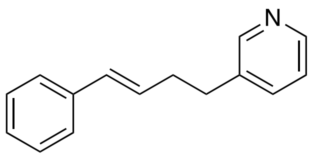

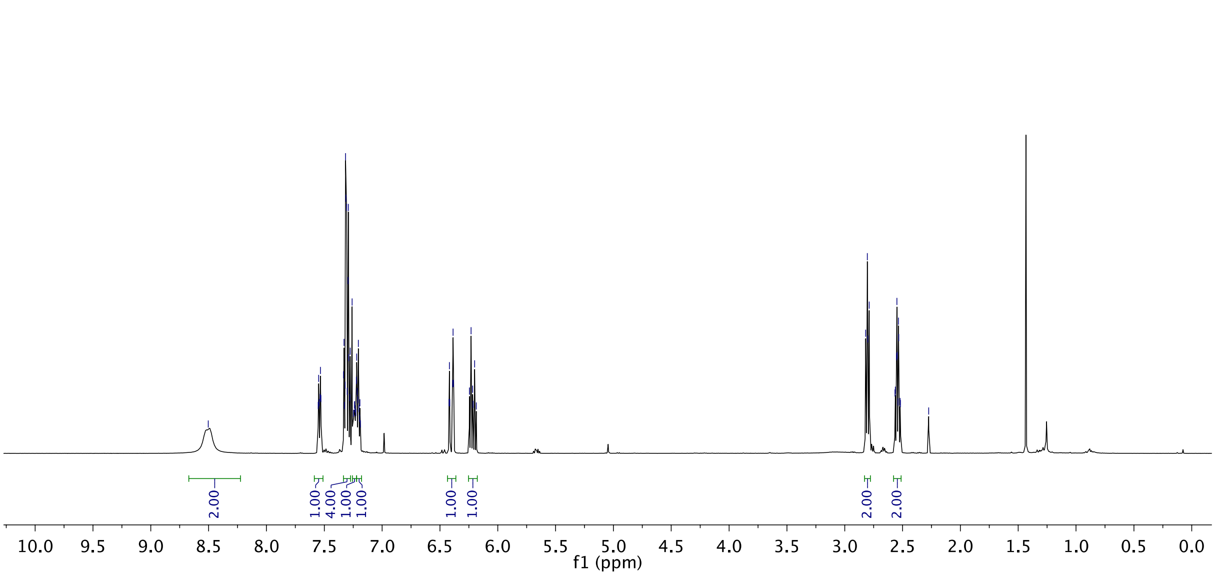

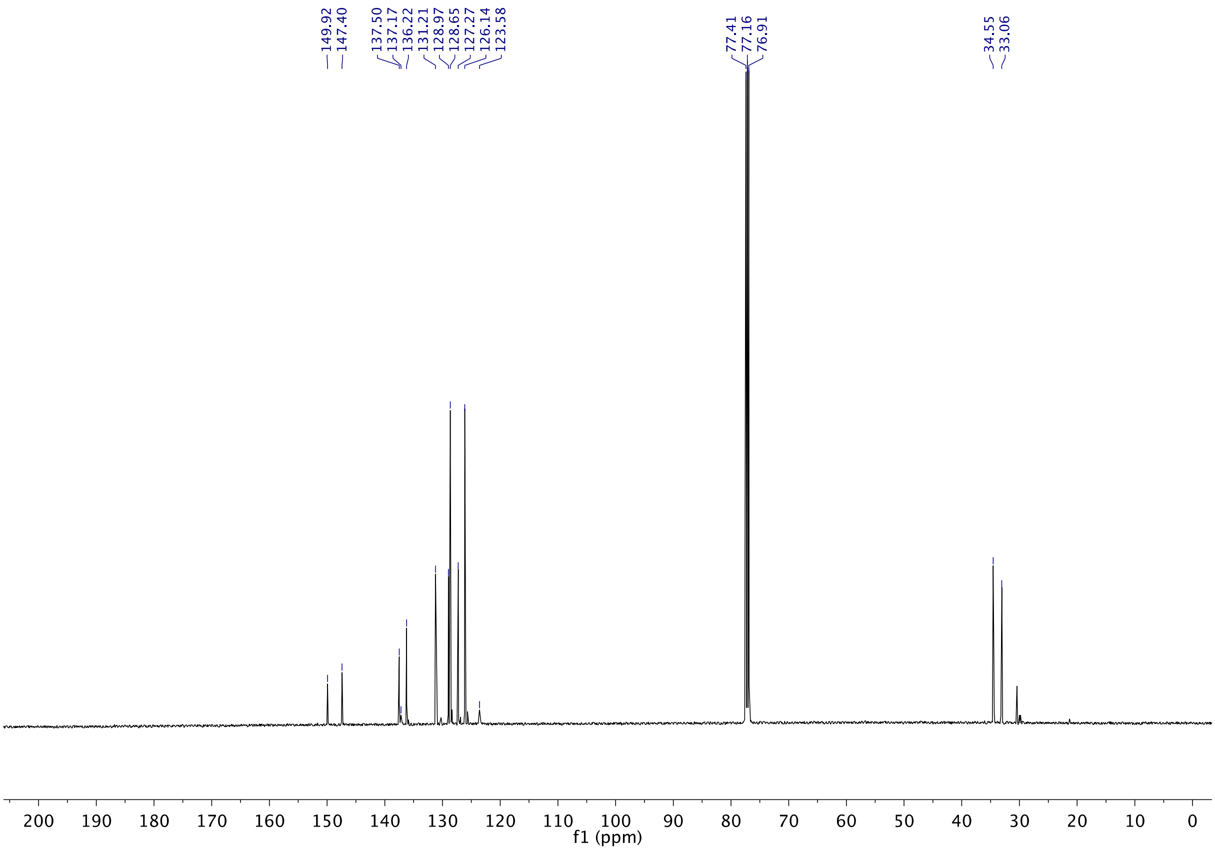

Methyl (*E*)-3-styrylbicyclo[1.1.1]pentane-1-carboxylate, **39**

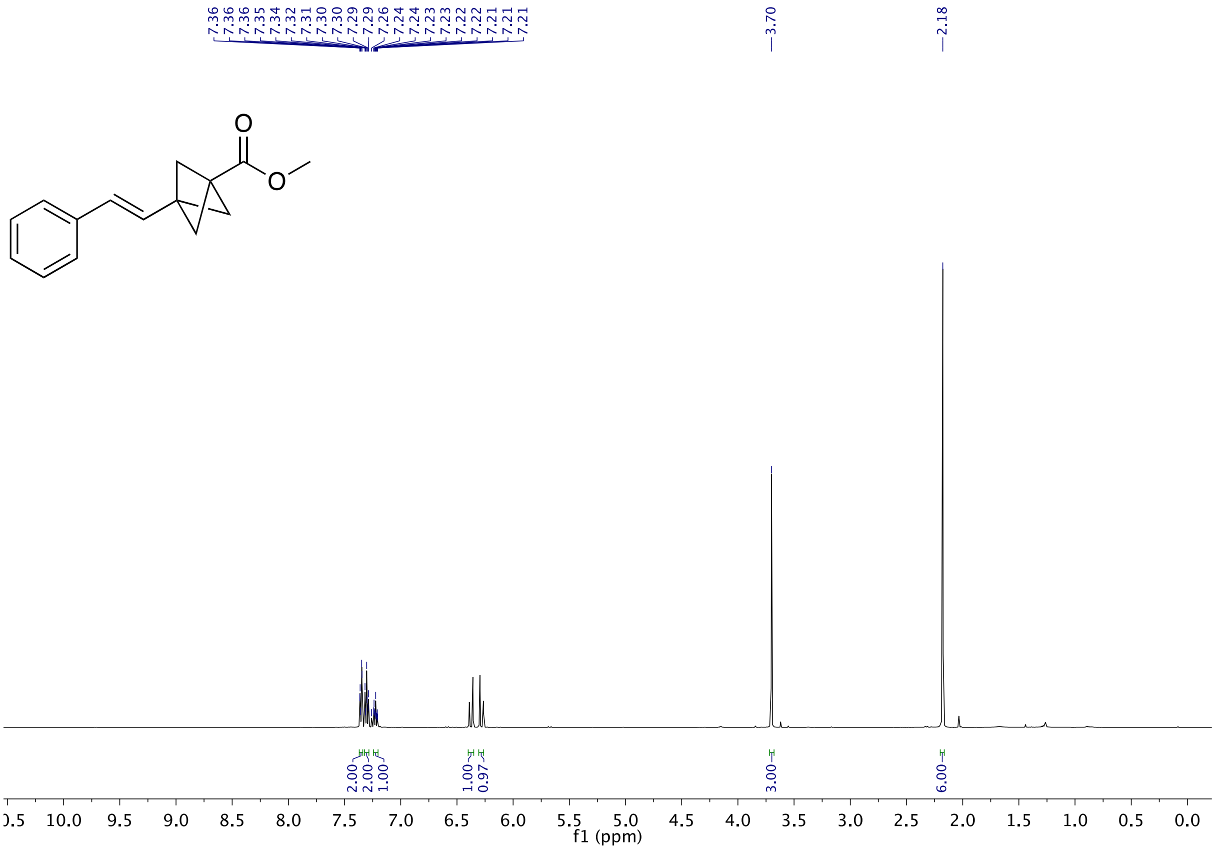

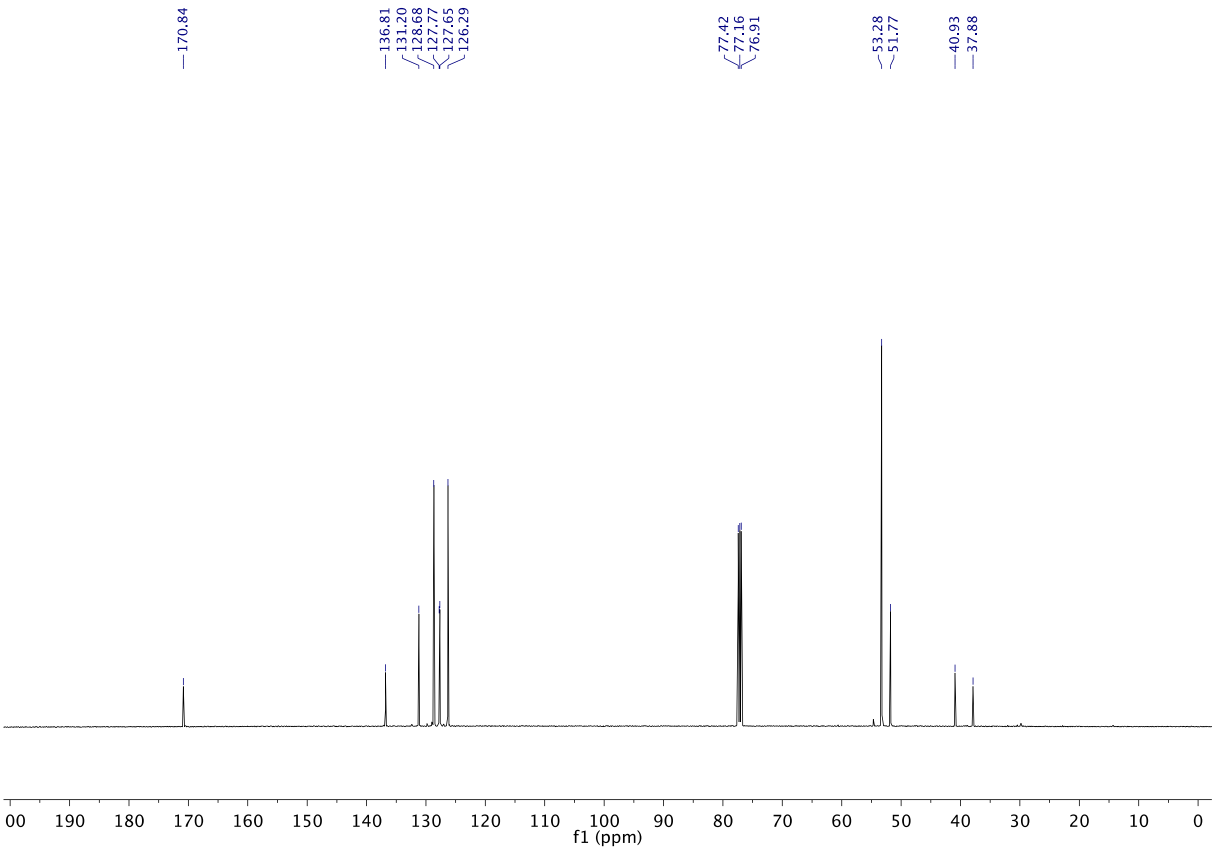

(*E*)-4,4,5,5-Tetramethyl-2-(4-(4-phenylbut-3-en-1-yl)phenyl)-1,3,2-dioxaborolane, **40**

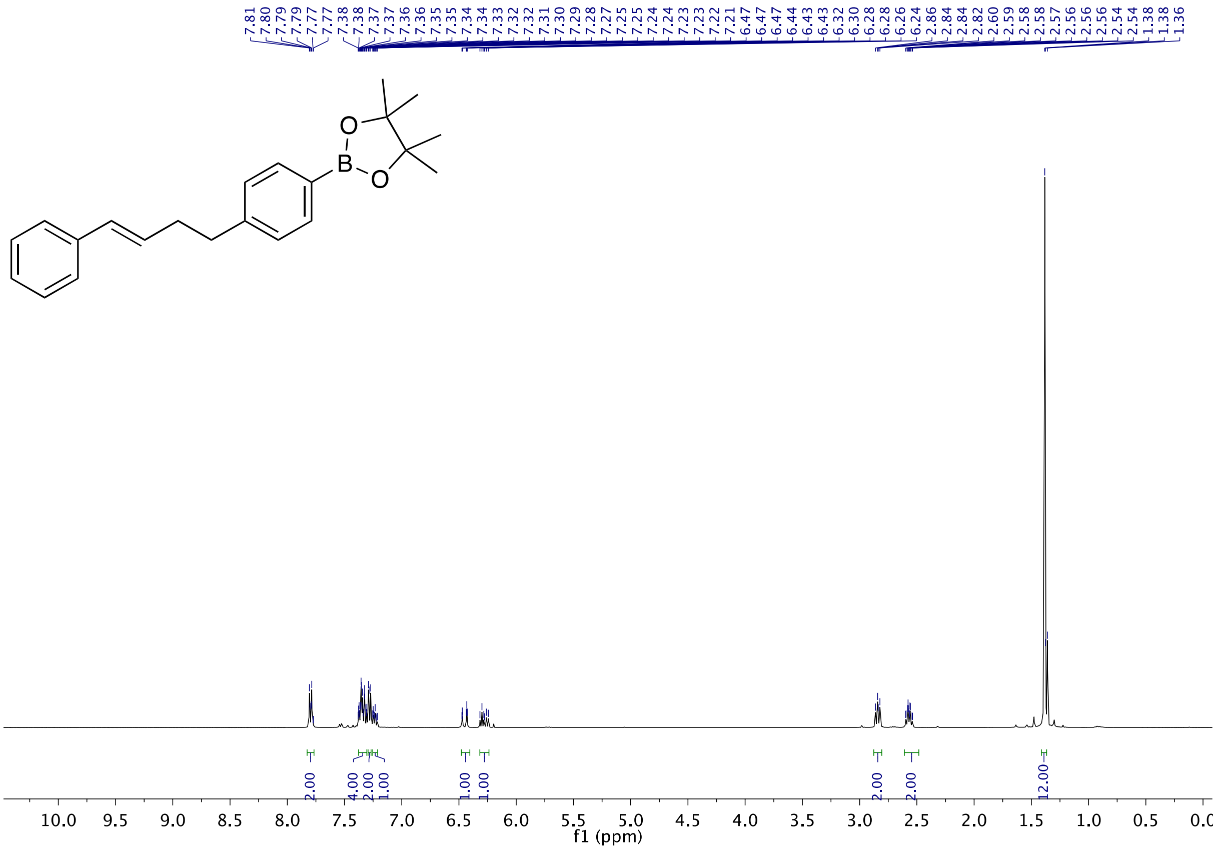

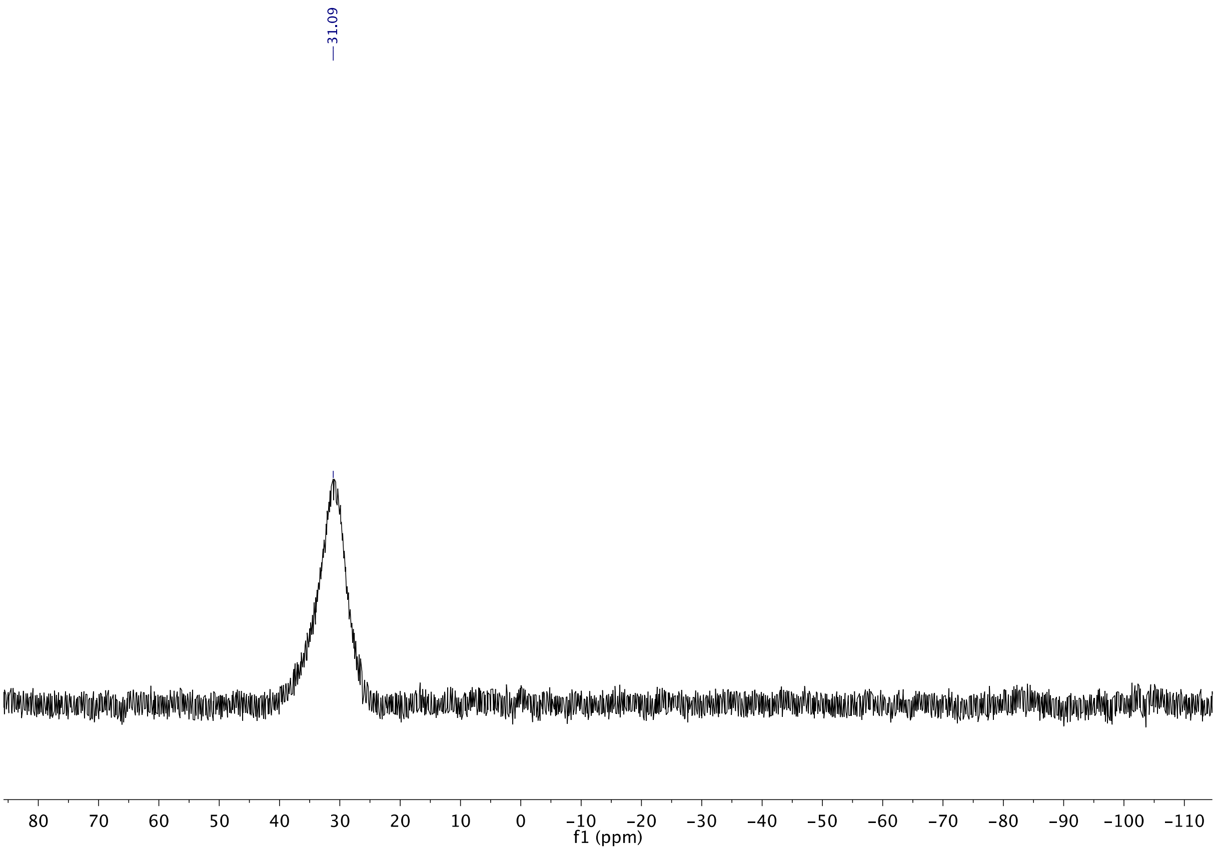

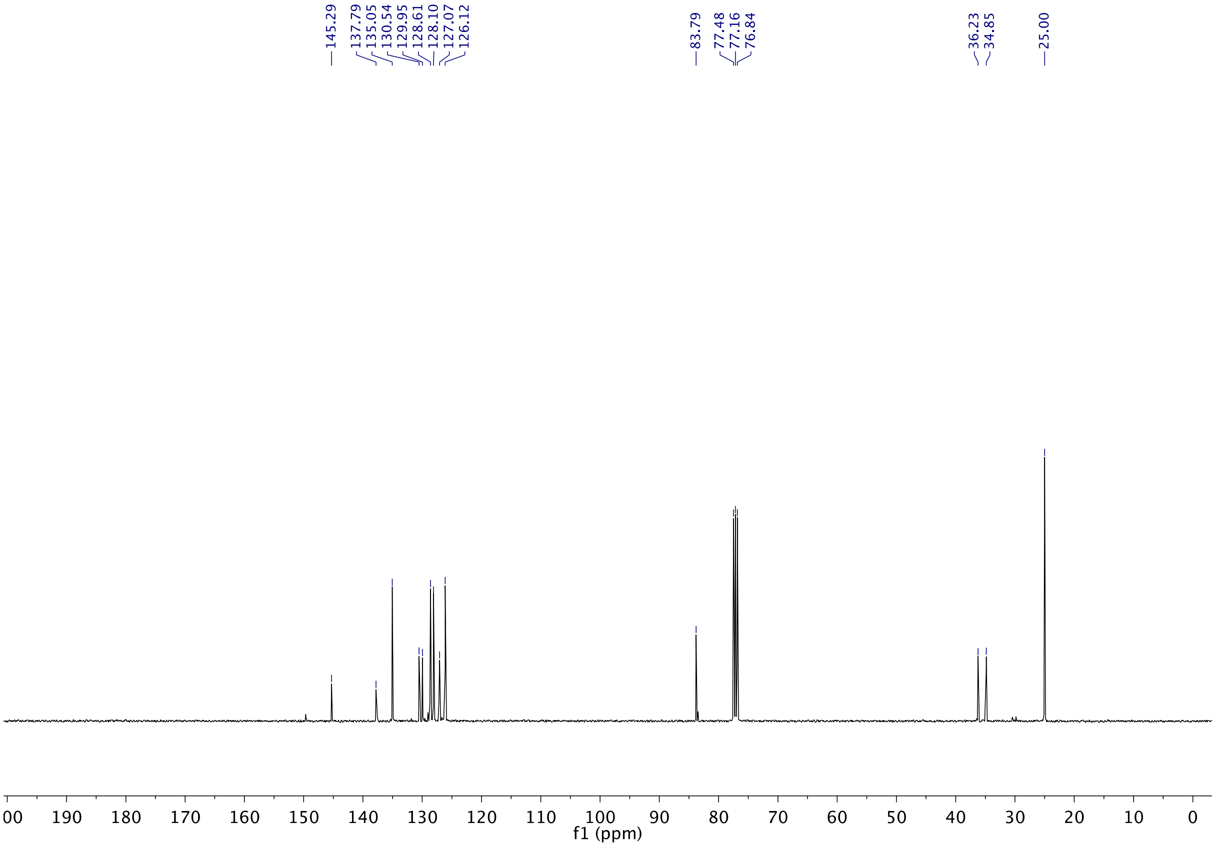

(*E*)-1-(2-cyclohexylvinyl)-4-methylbenzene, **42**

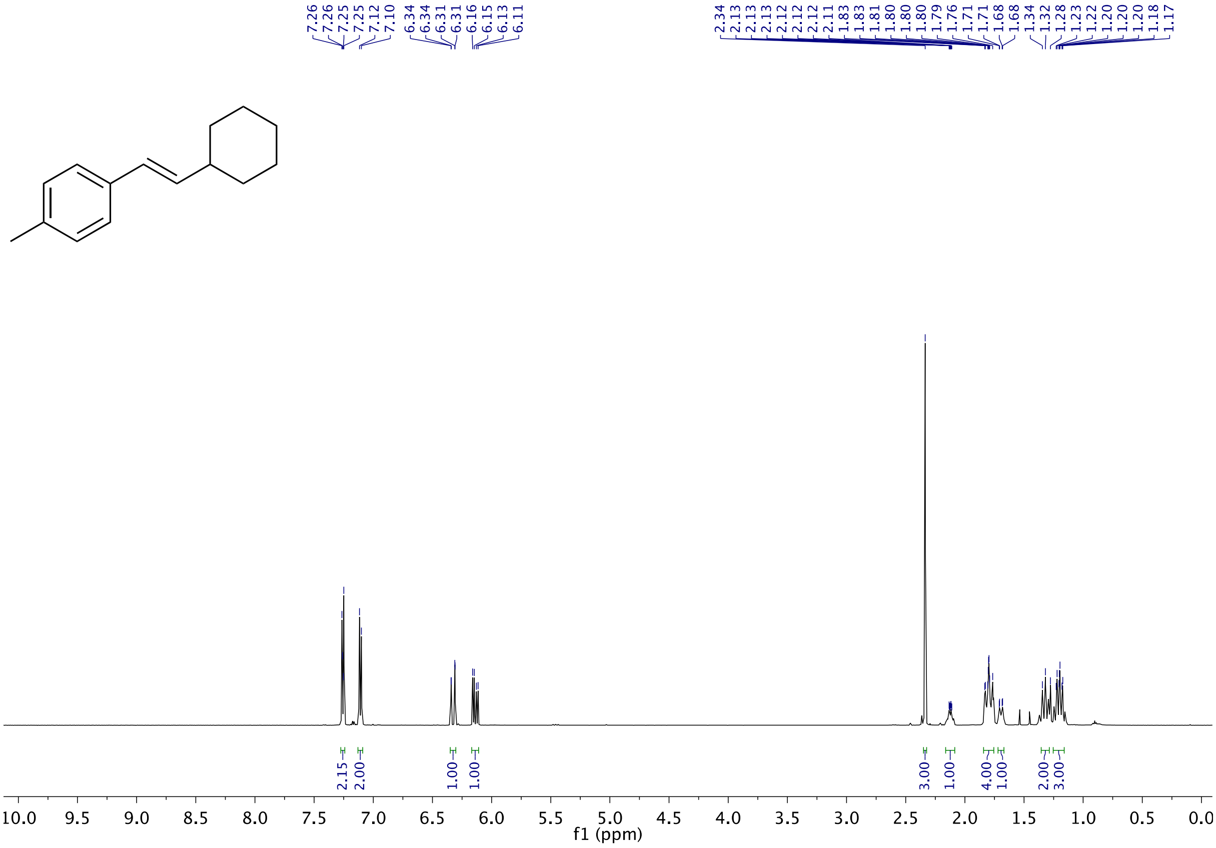

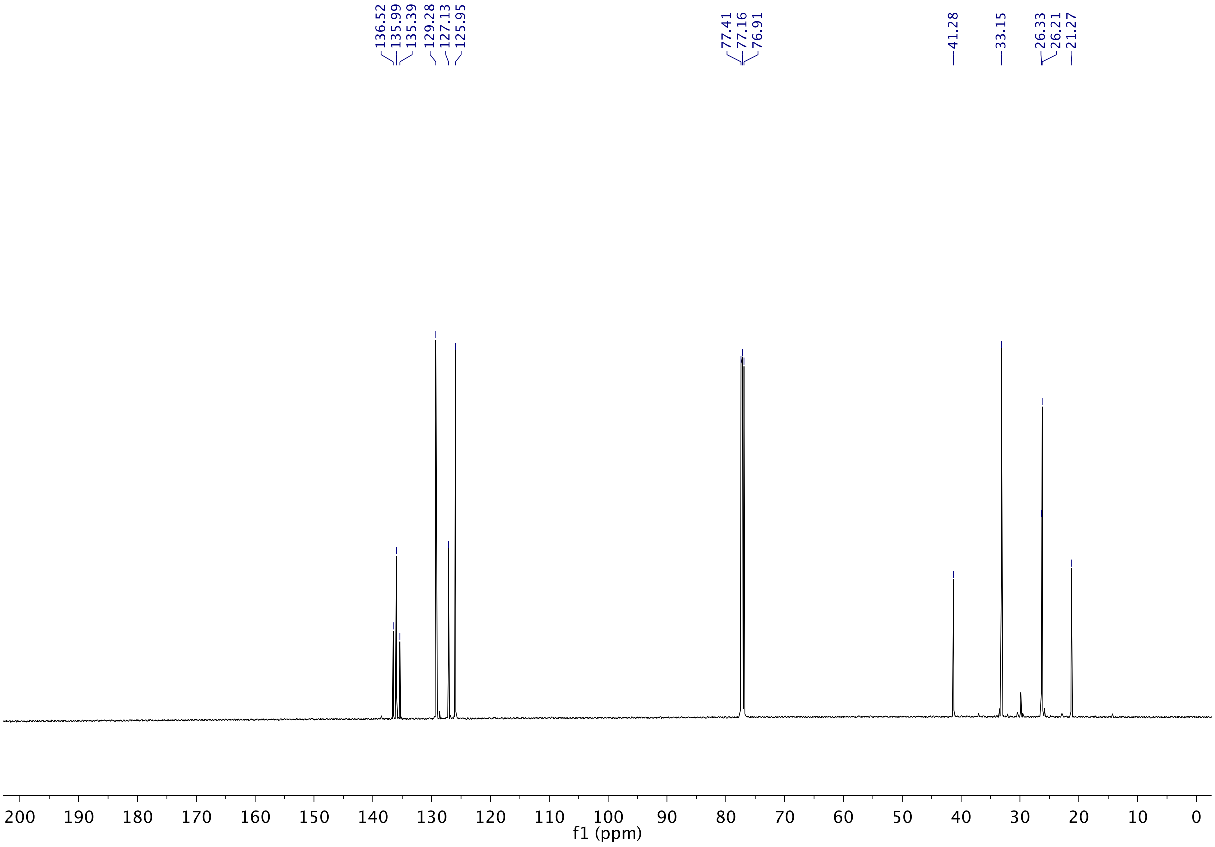

(*E*)-1-chloro-4-(2-cyclohexylvinyl)benzene, **43**

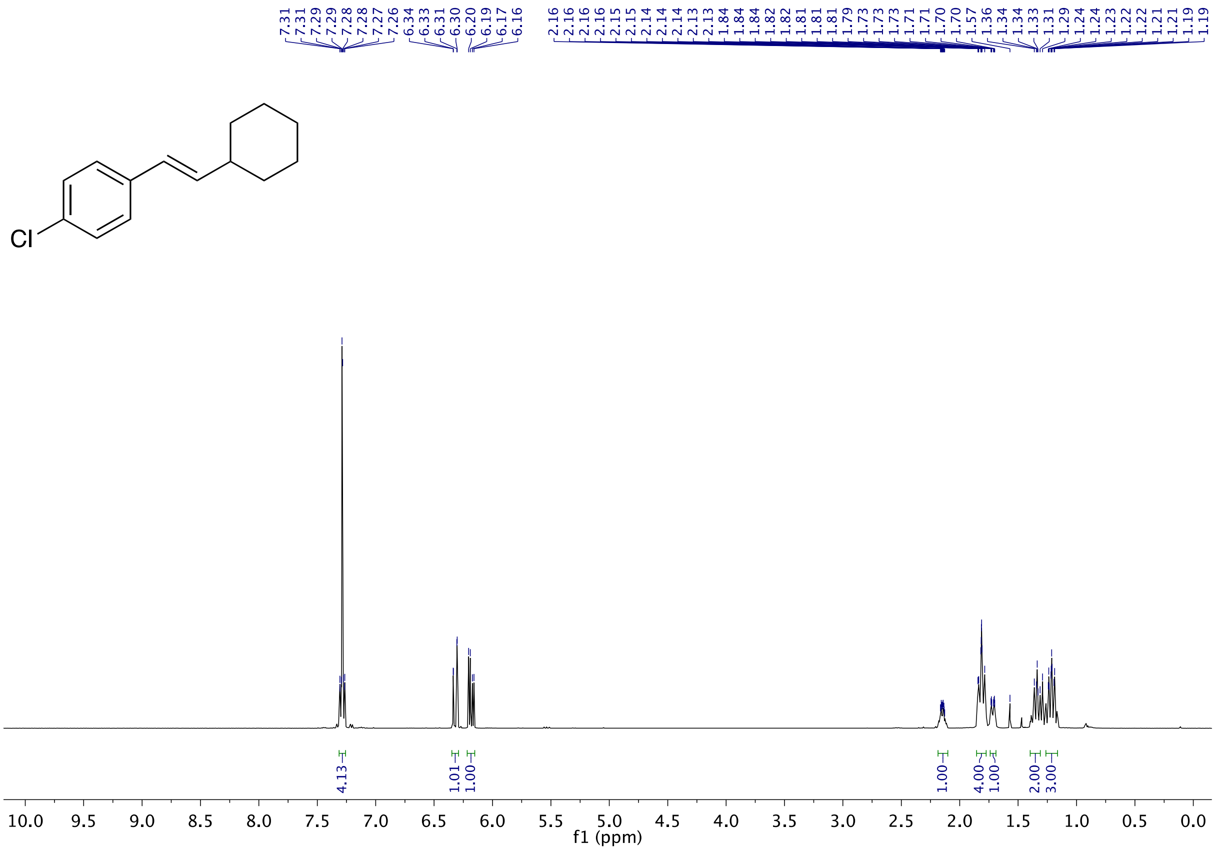

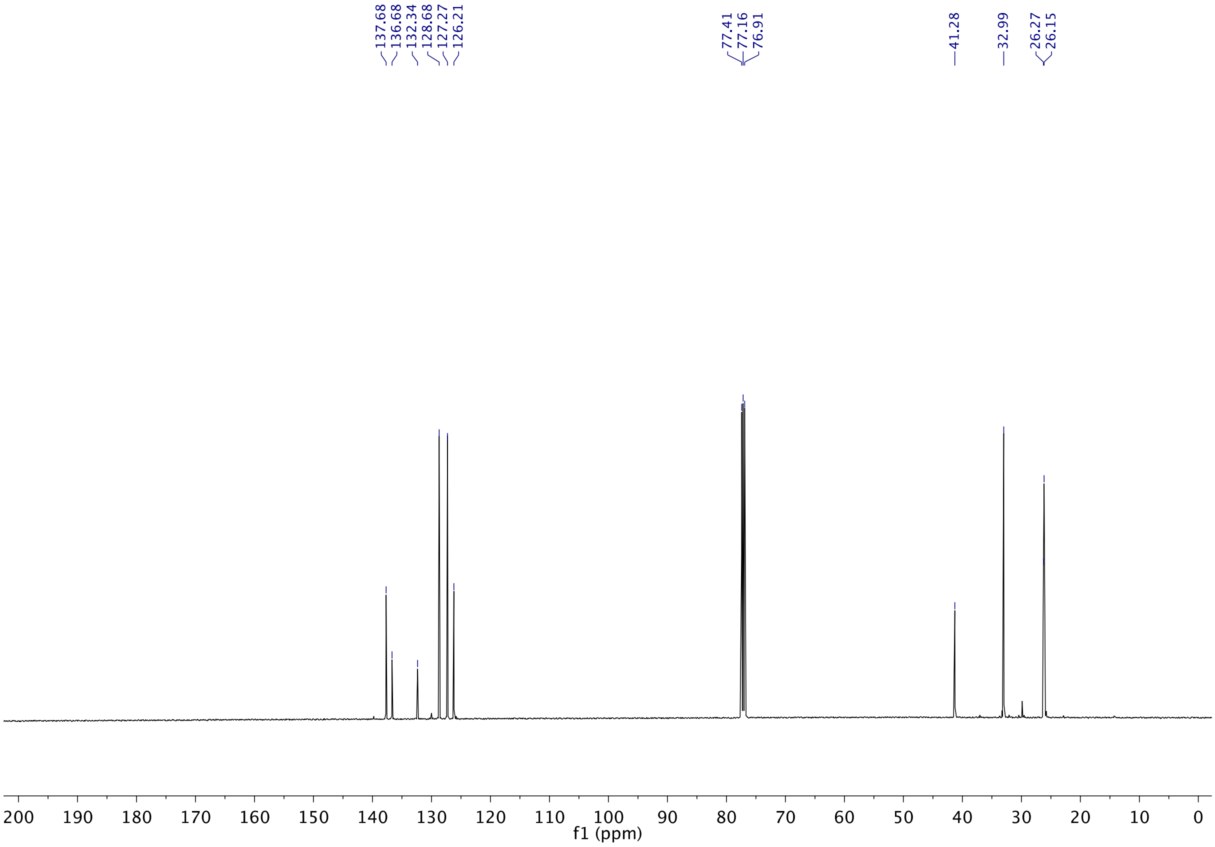

(*E*)-1-(2-cyclohexylvinyl)-4-methoxybenzene, **44**

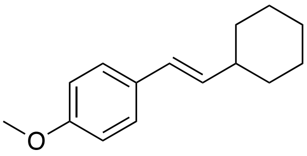

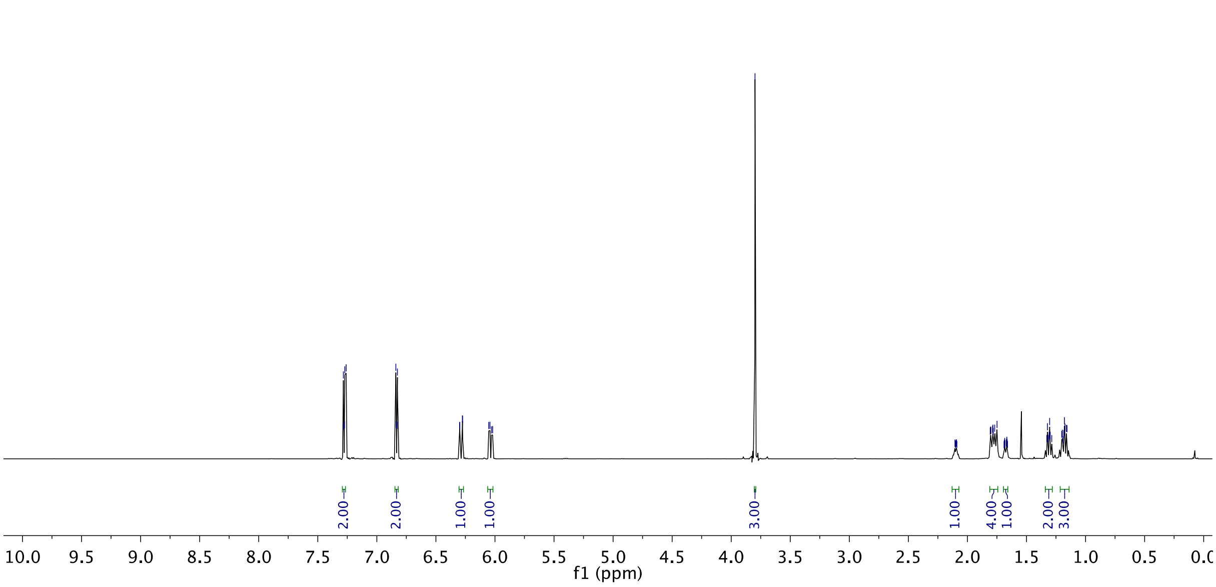

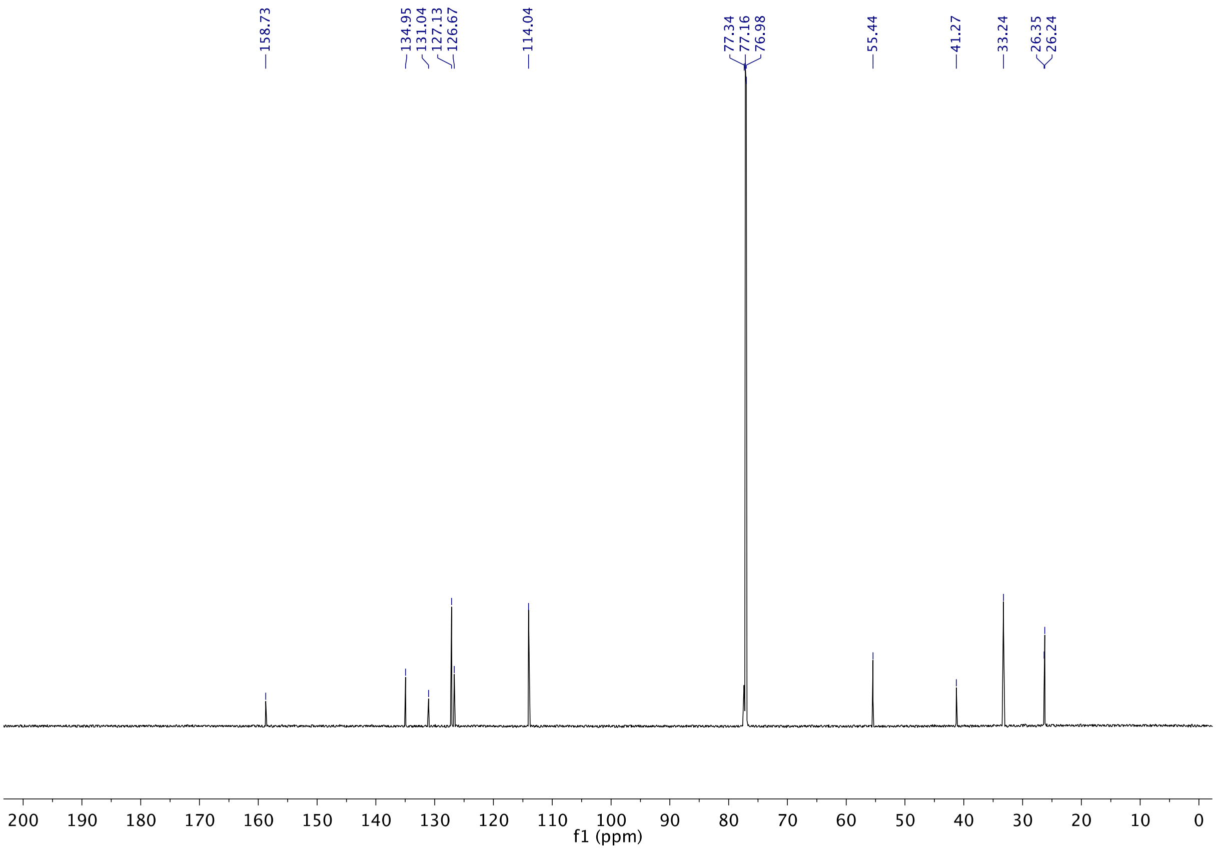

(*E*)-1-(2-cyclohexylvinyl)-4-fluorobenzene, **45**

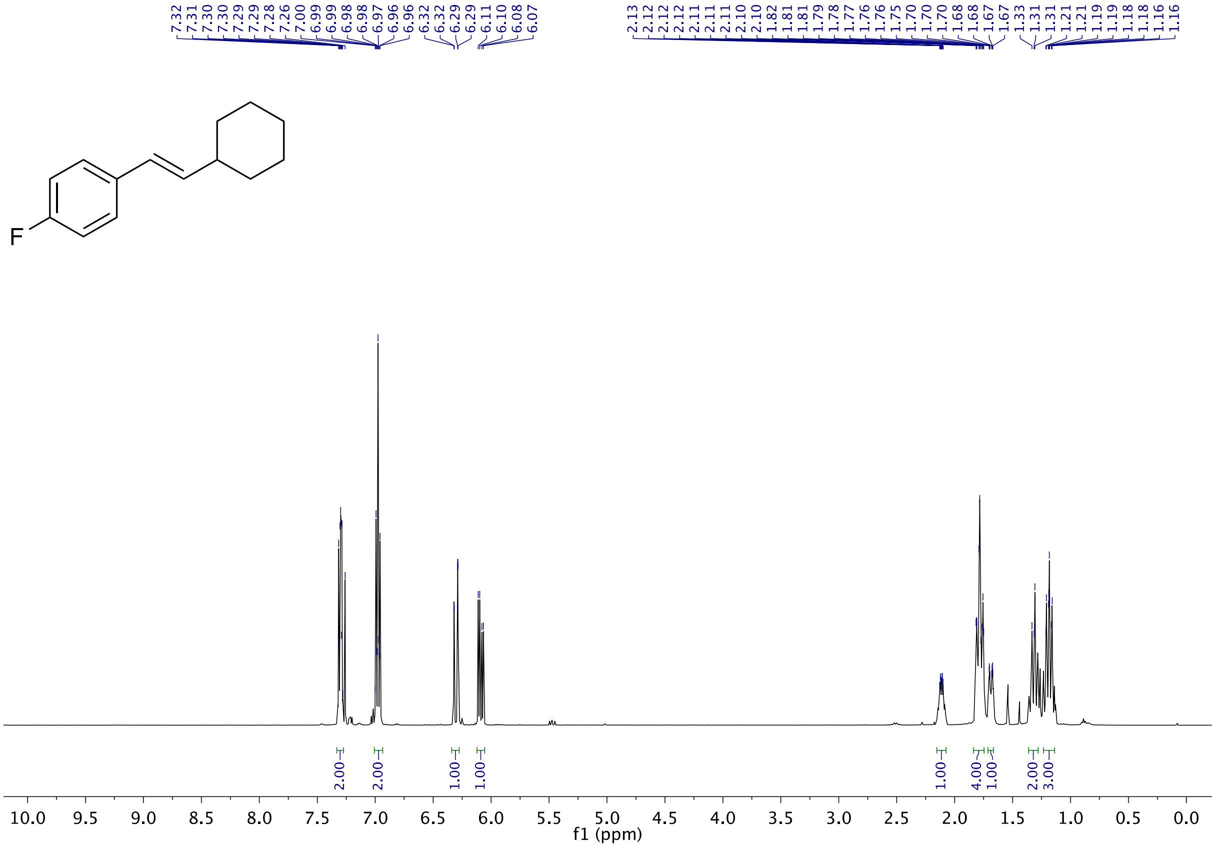

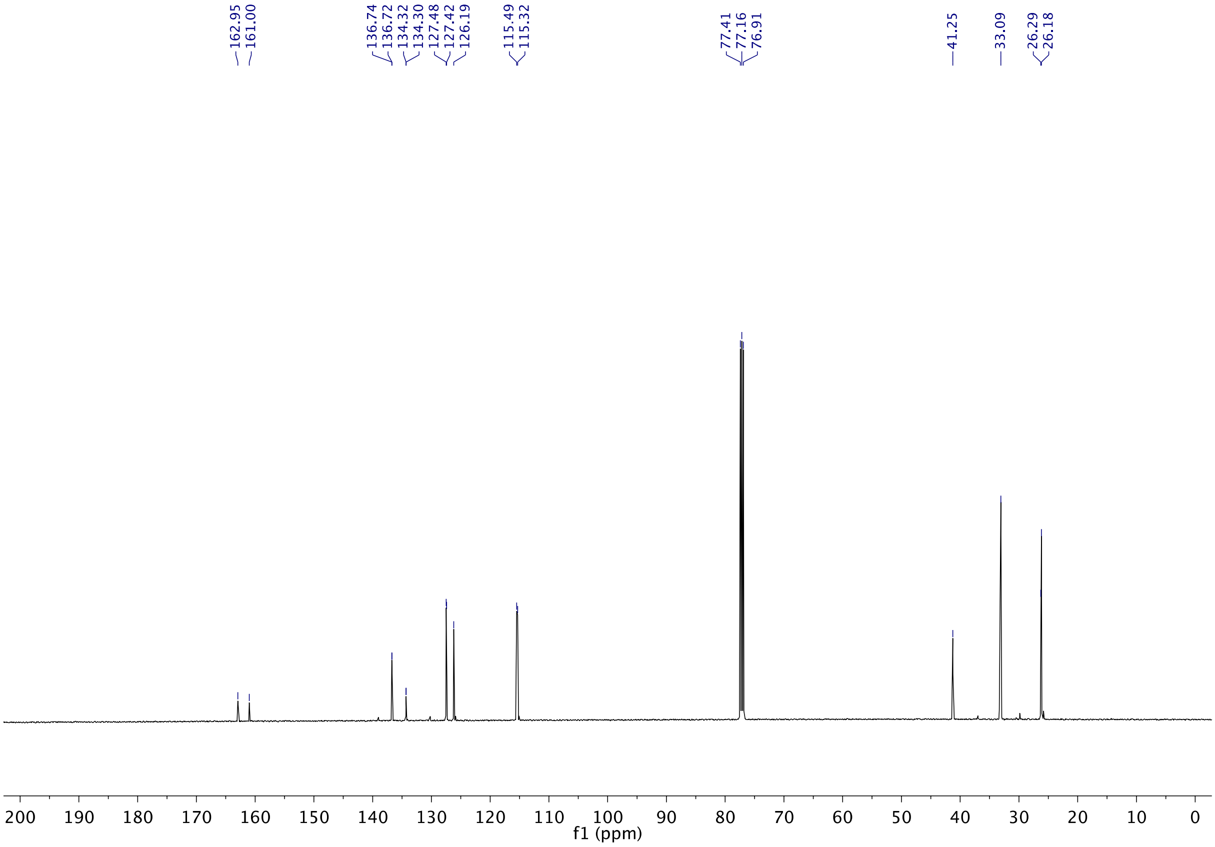
162.95
161.00
136.74
136.72
134.32
134.30
127.48
127.42
126.19
115.49
115.32
77.41
77.16
76.91
41.25
33.09
26.29
26.18
200 190 180 170 160 150 140 130 120 110 100 90 80 70 60 50 40 30 20 10 0
f1 (ppm)

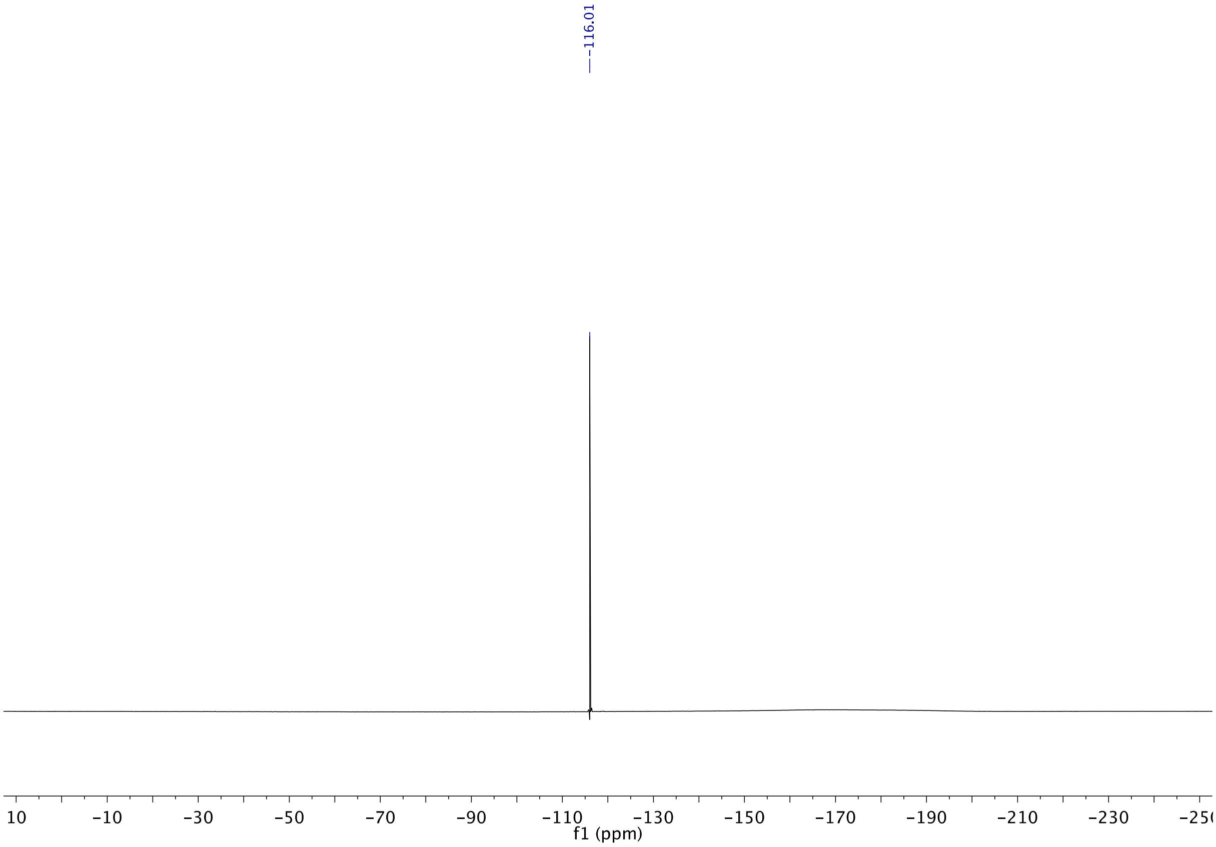
-116.01
10 −10 −30 −50 −70 −90 −110 −130 −150 −170 −190 −210 −230
f1 (ppm)

(*E*)-4-(2-Cyclohexylvinyl)-1,1'-biphenyl, **46**

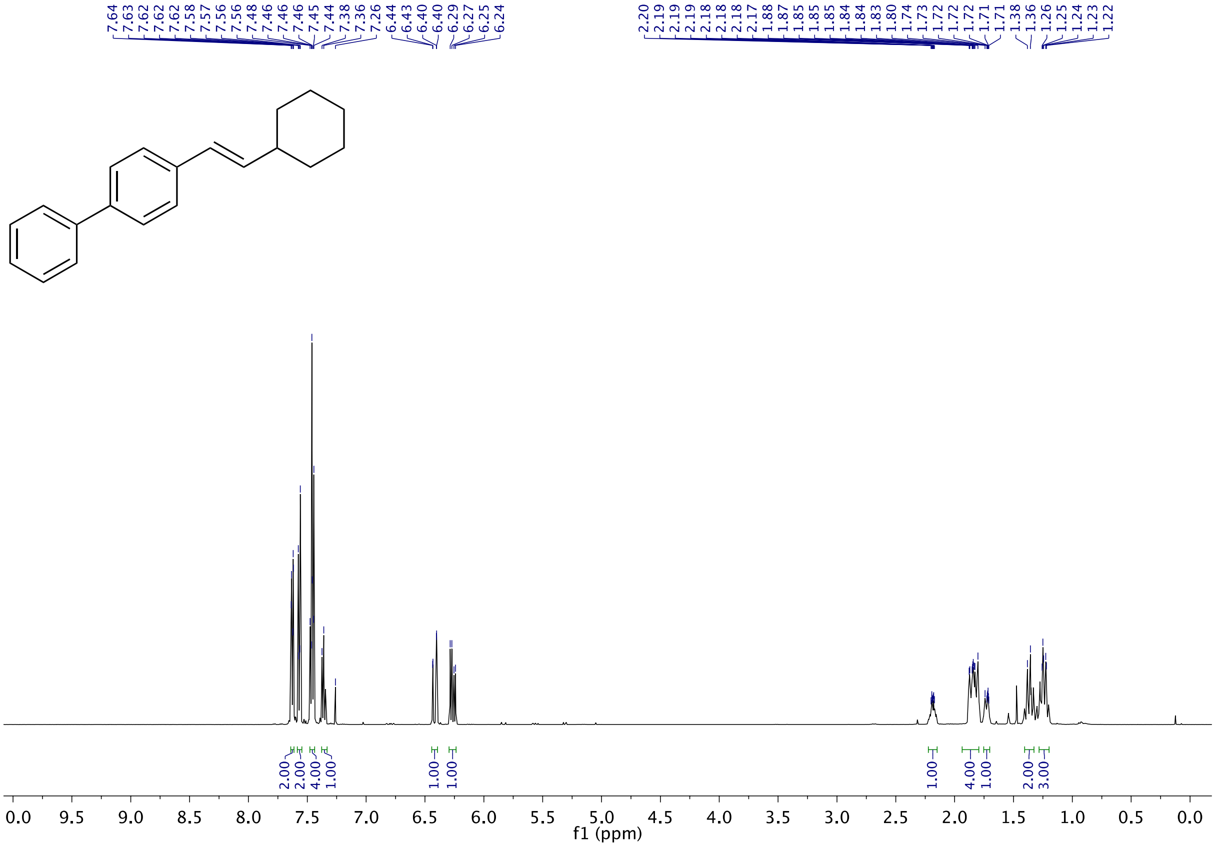

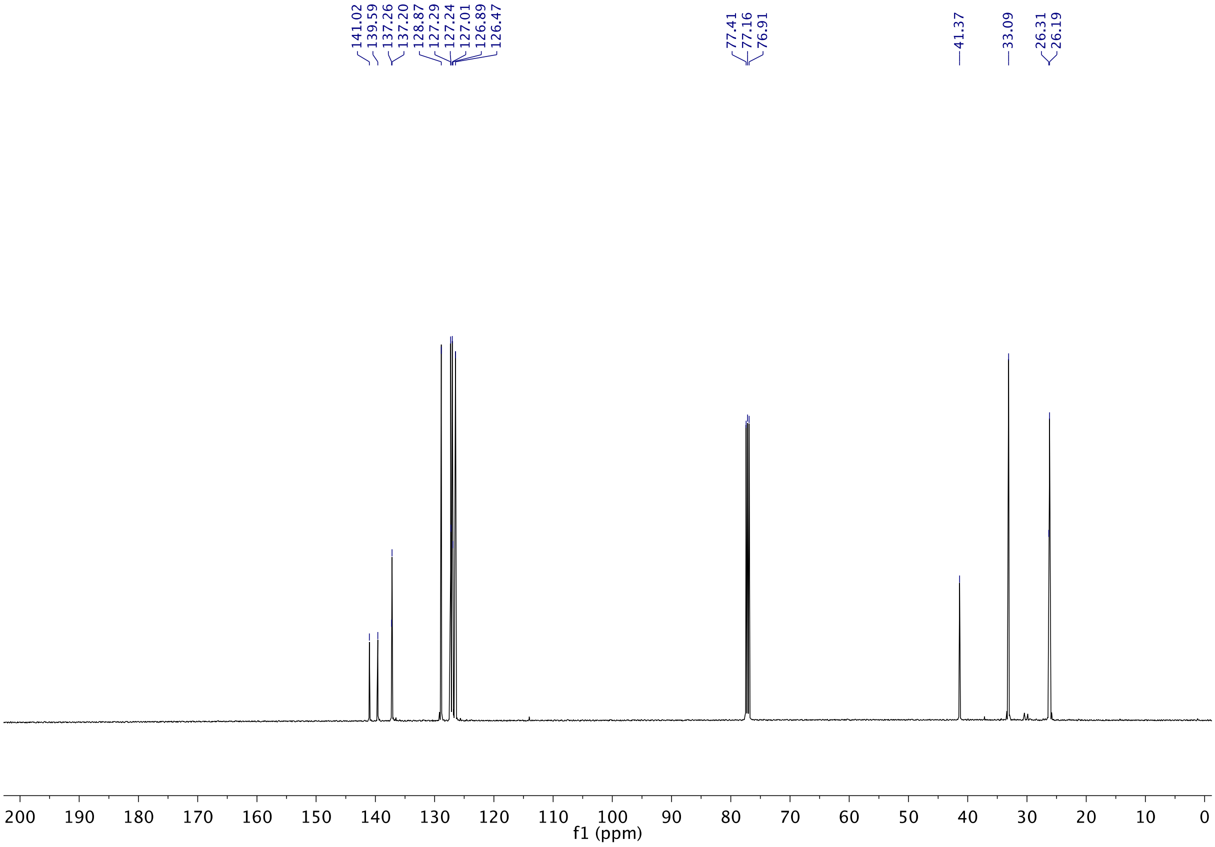

(*E*)-4-(2-Cyclohexylvinyl)benzonitrile, **47**

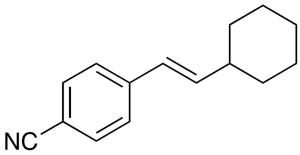

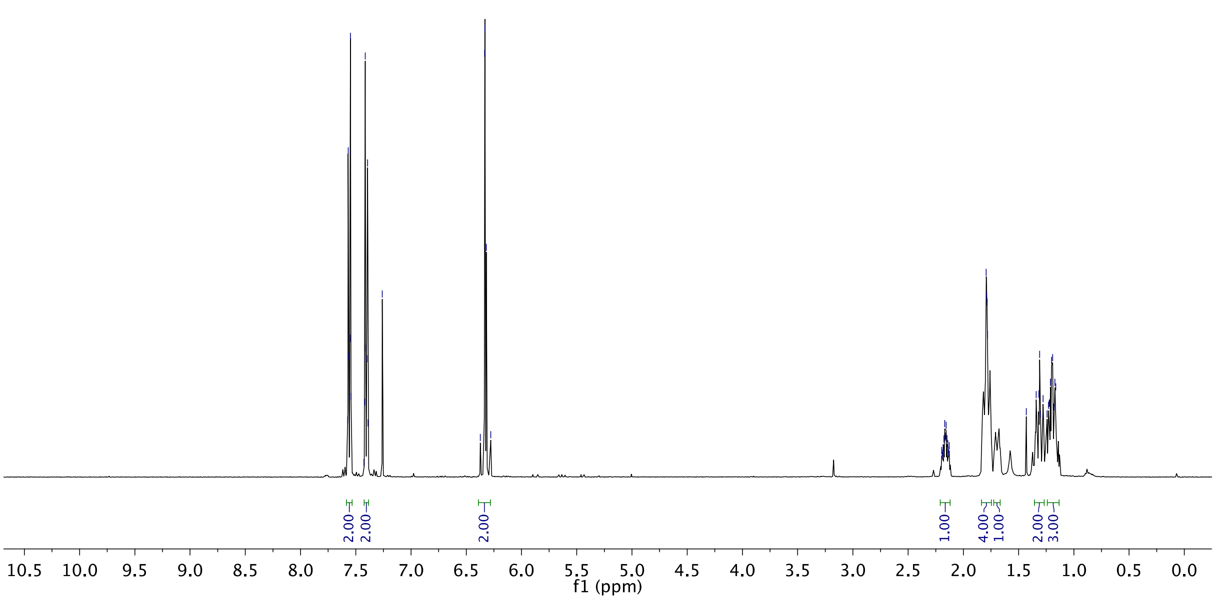

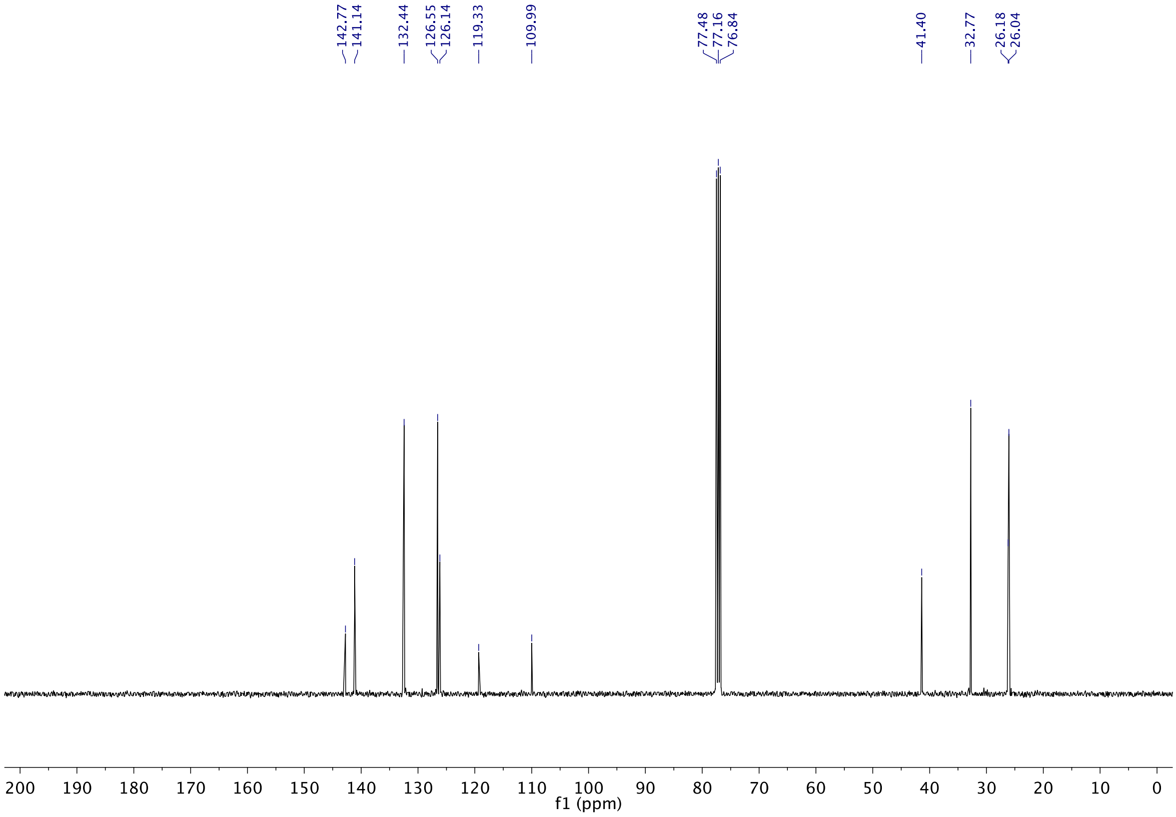

(*E*)-1-(2-Cyclohexylvinyl)-3-methoxybenzene, **48**

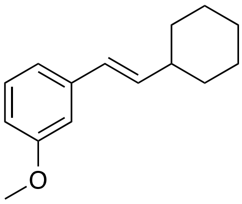

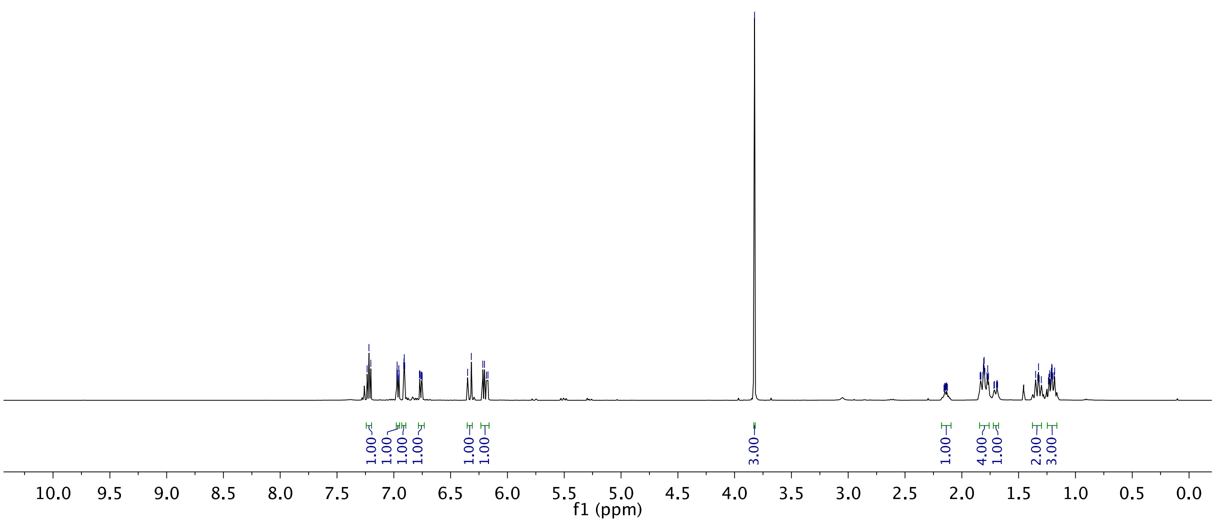

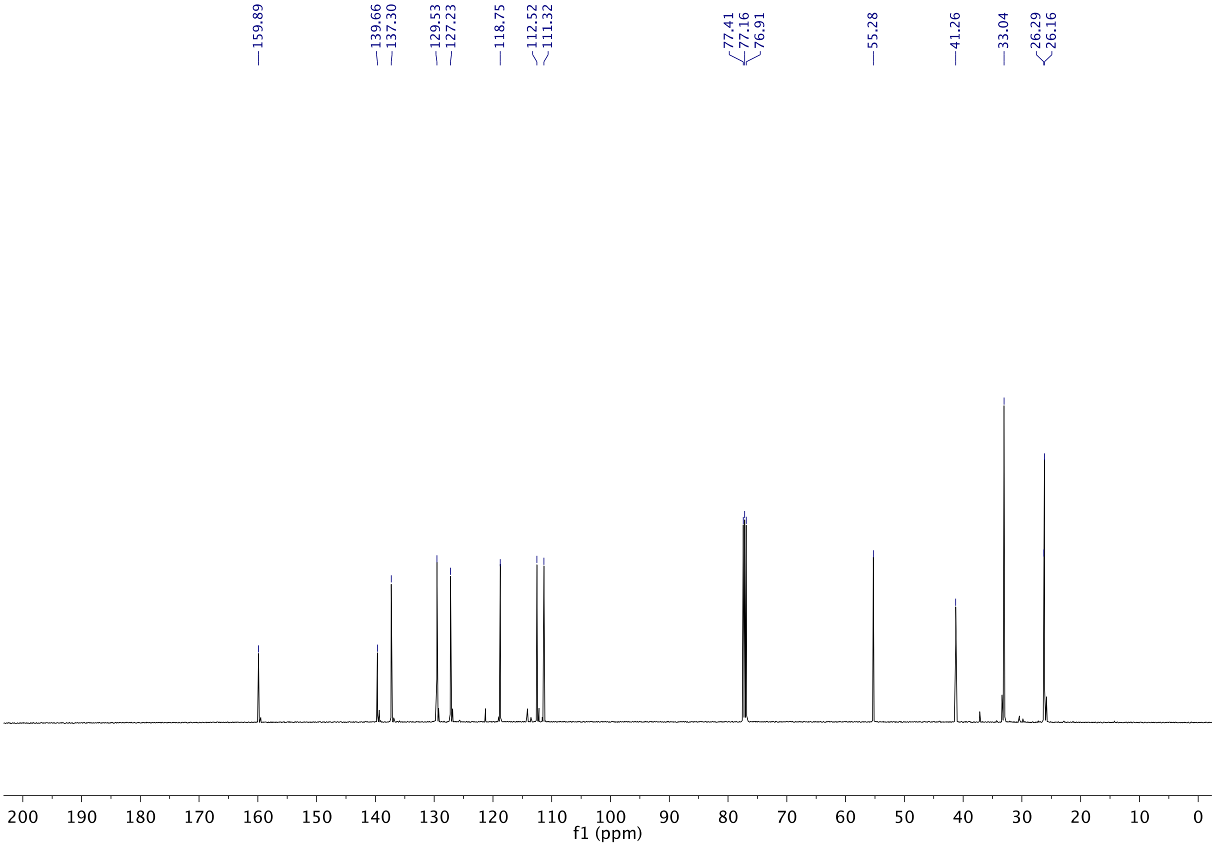

(*E*)-*N*-(3-(2-Cyclohexylvinyl)phenyl)acetamide, **49**

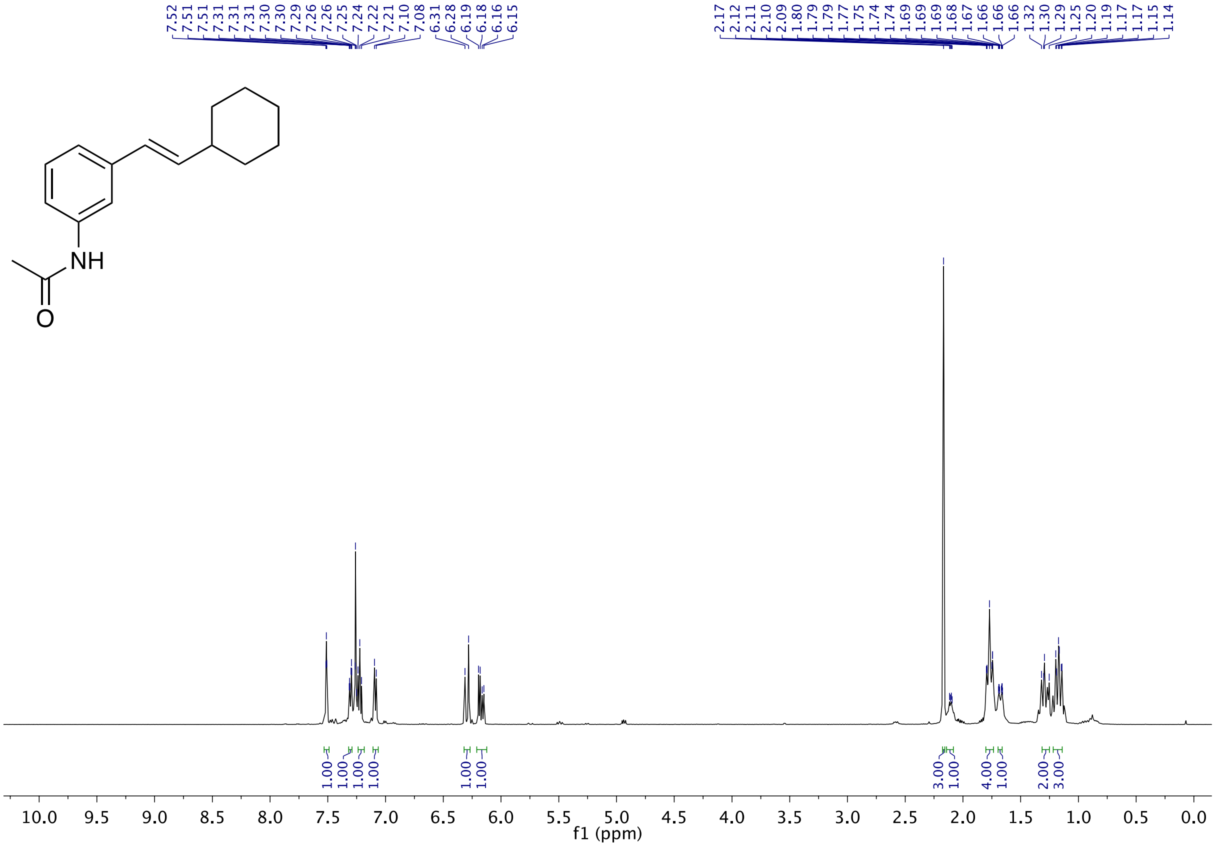

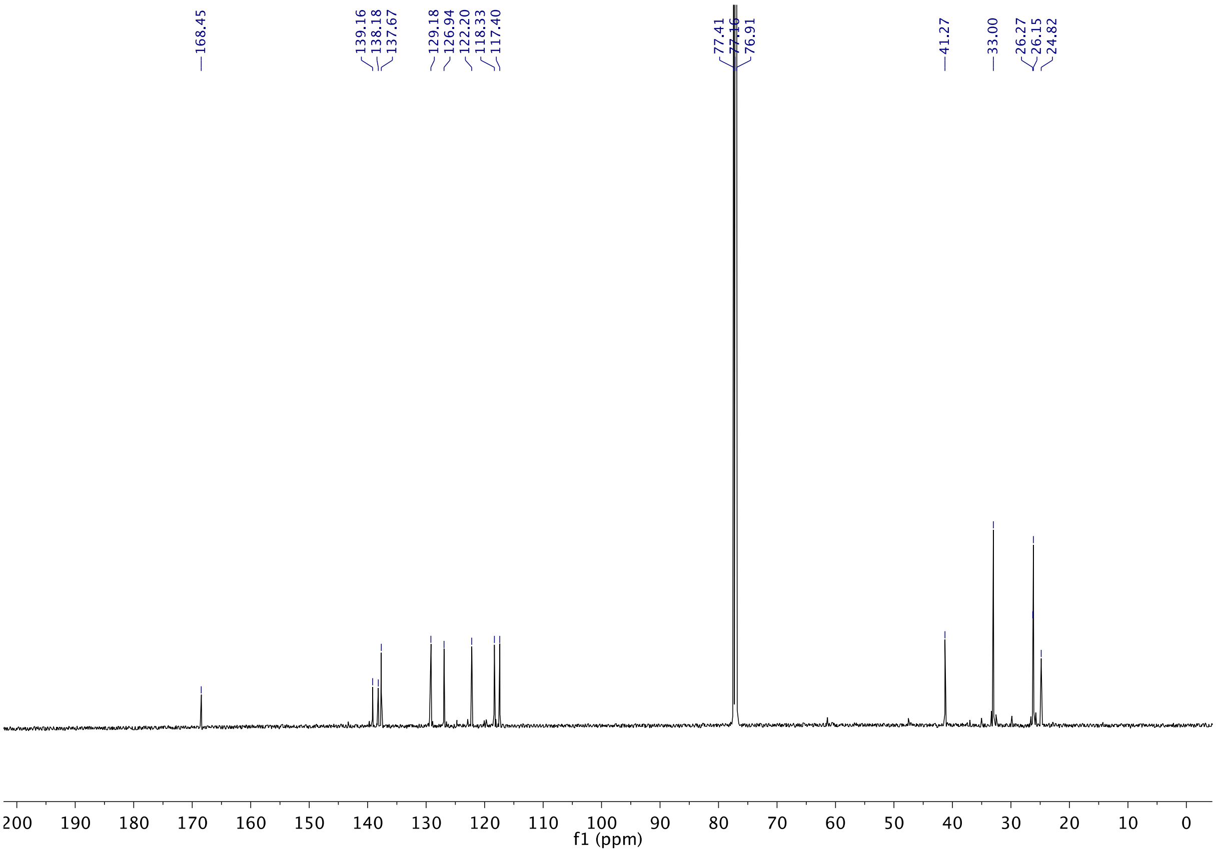

Methyl (*E*)-3-(2-cyclohexylvinyl)benzoate, **50**

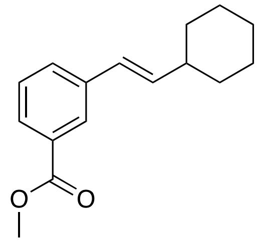

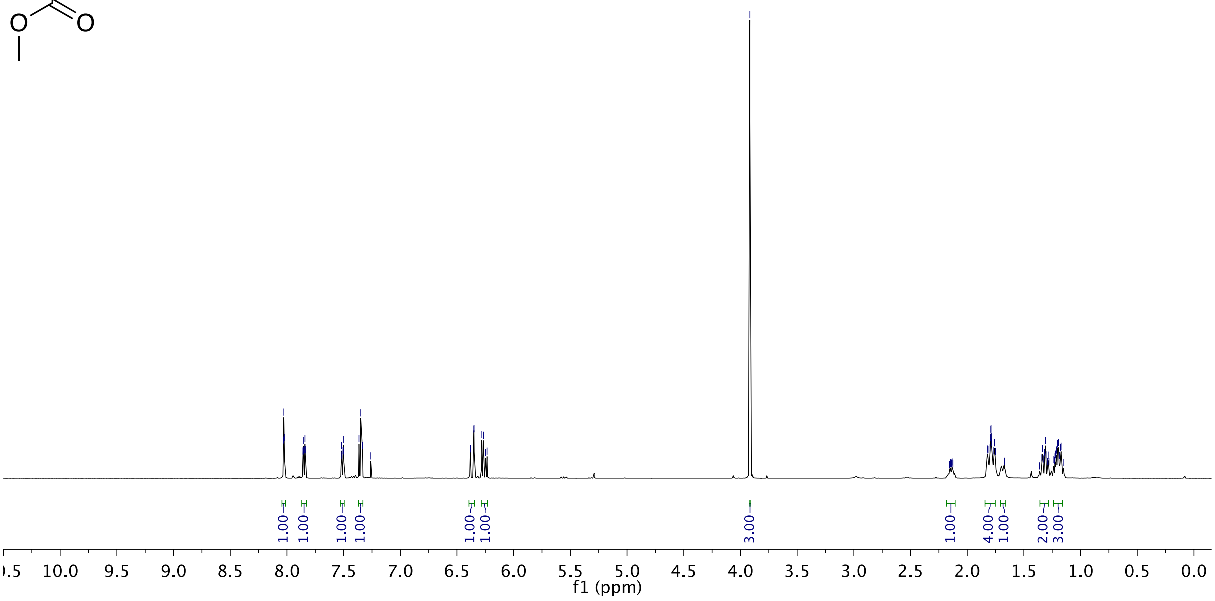

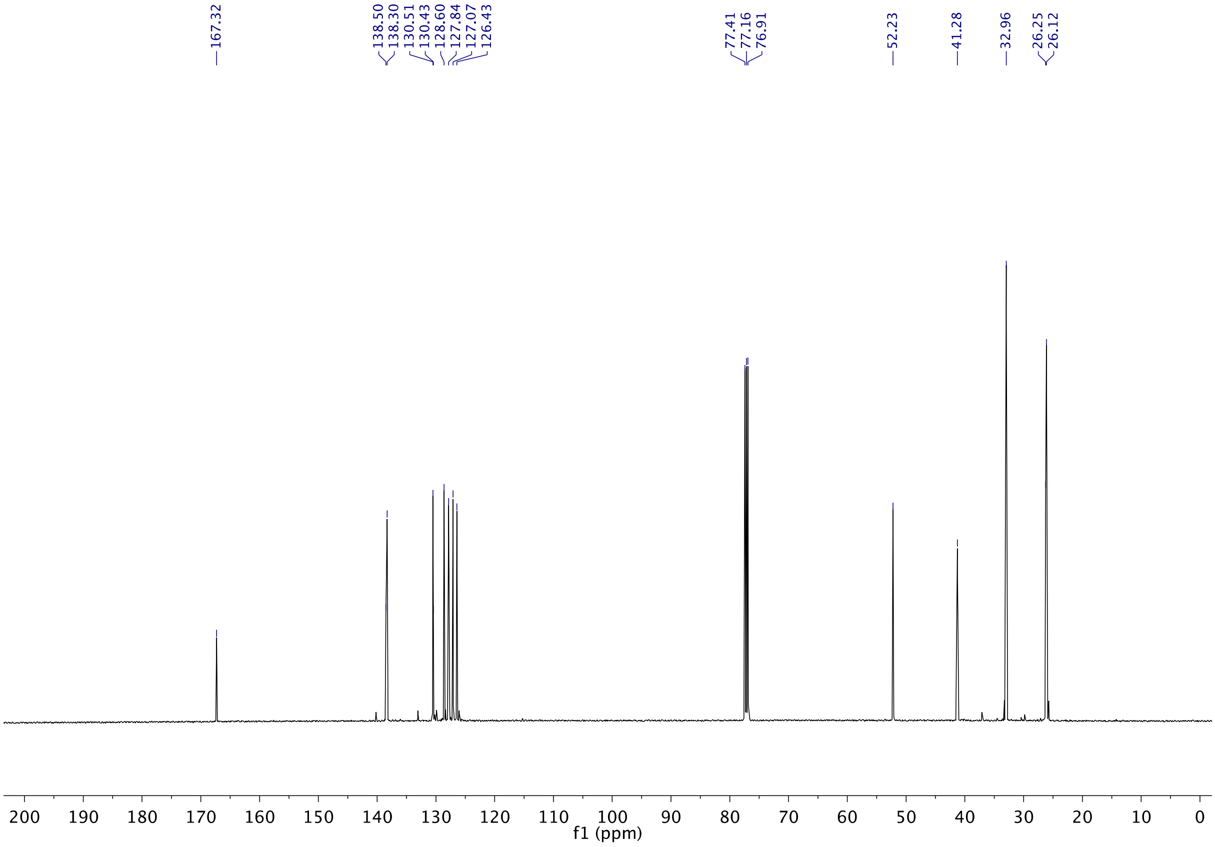

(*E*)-1-(2-(2-Cyclohexylvinyl)phenyl)ethan-1-one, **51**

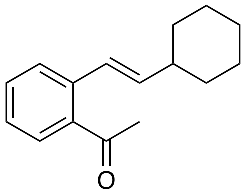

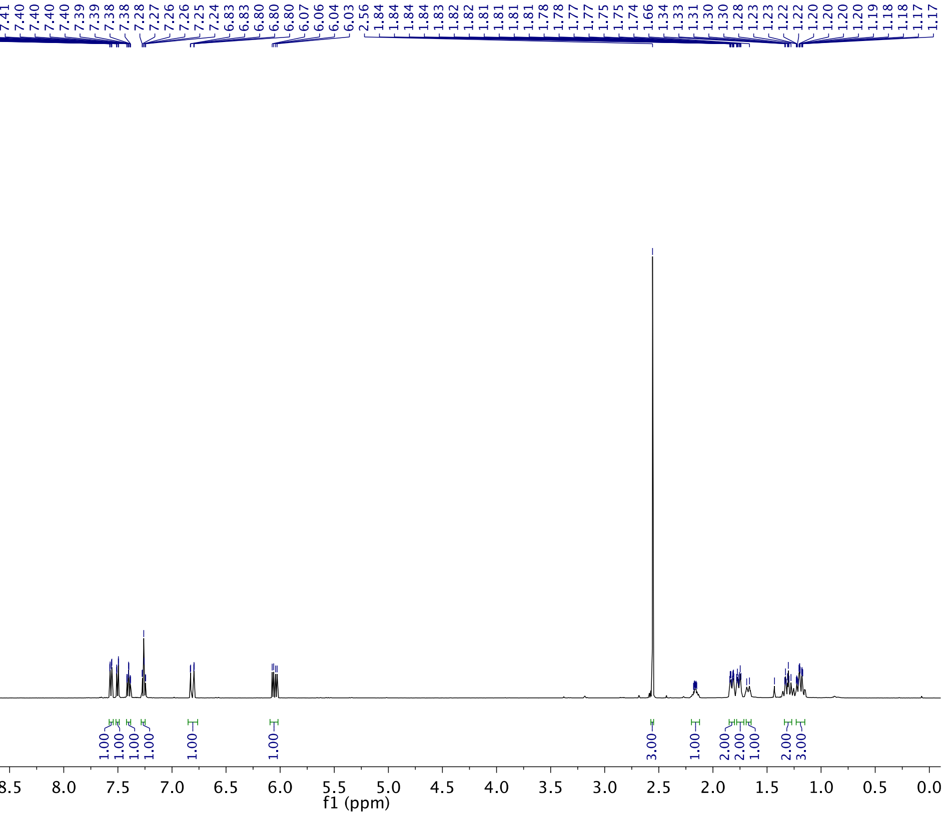

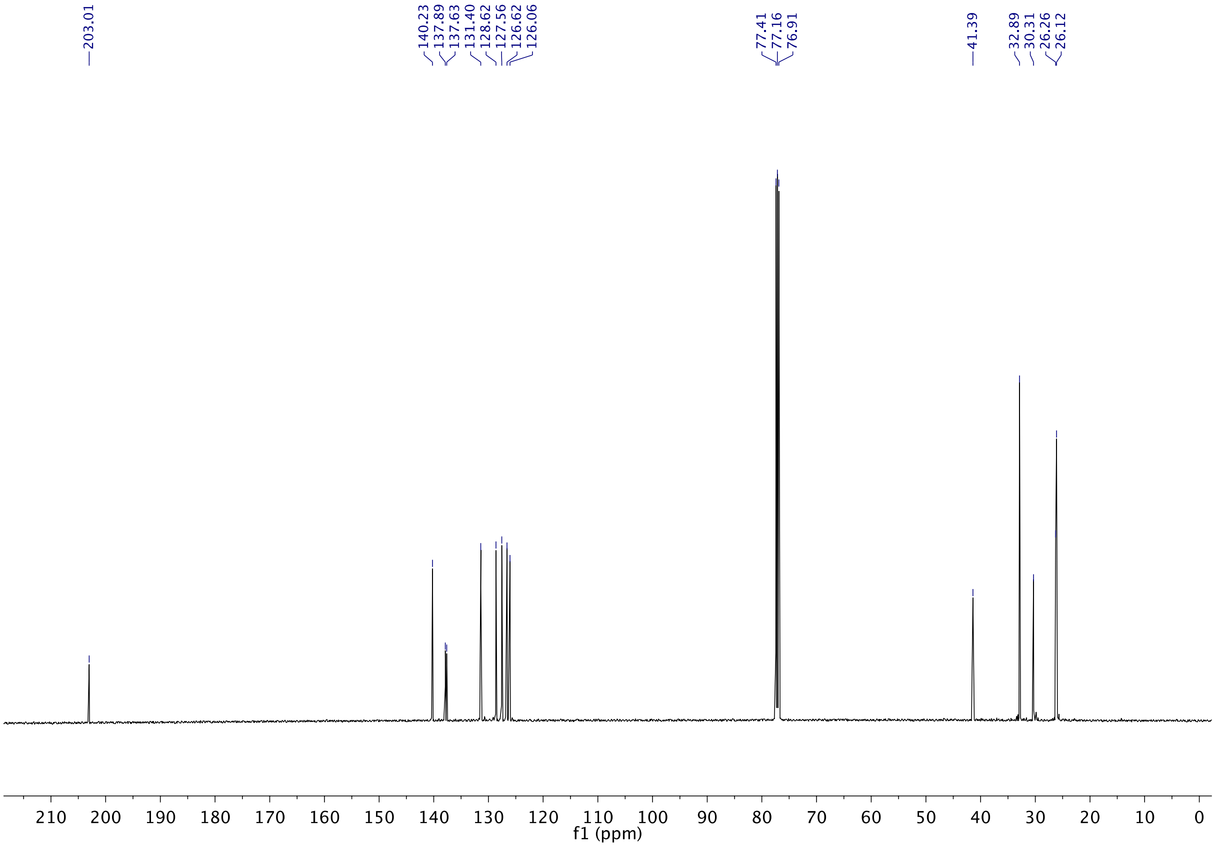

(*E*)-1-Bromo-2-(2-cyclohexylvinyl)benzene, **52**

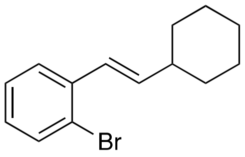

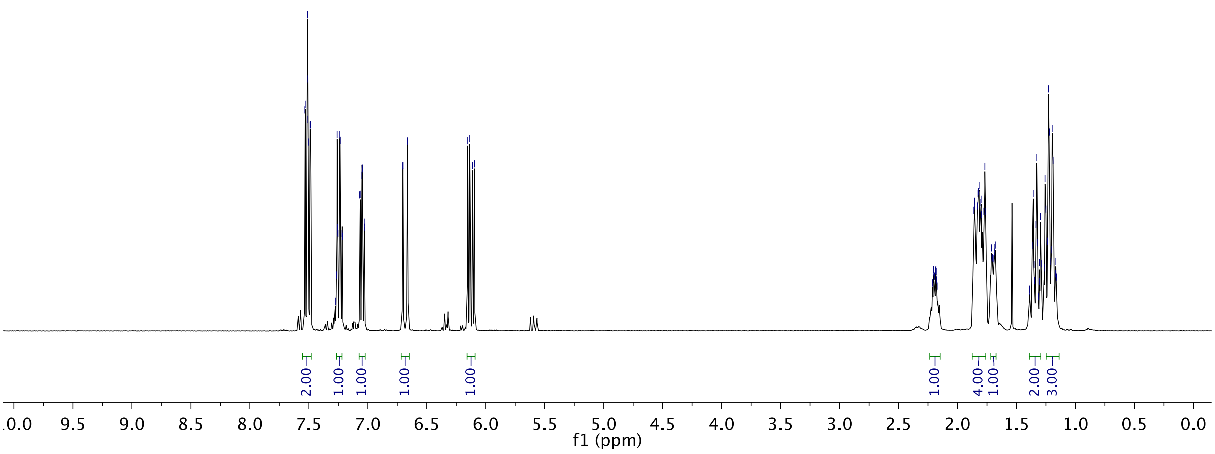

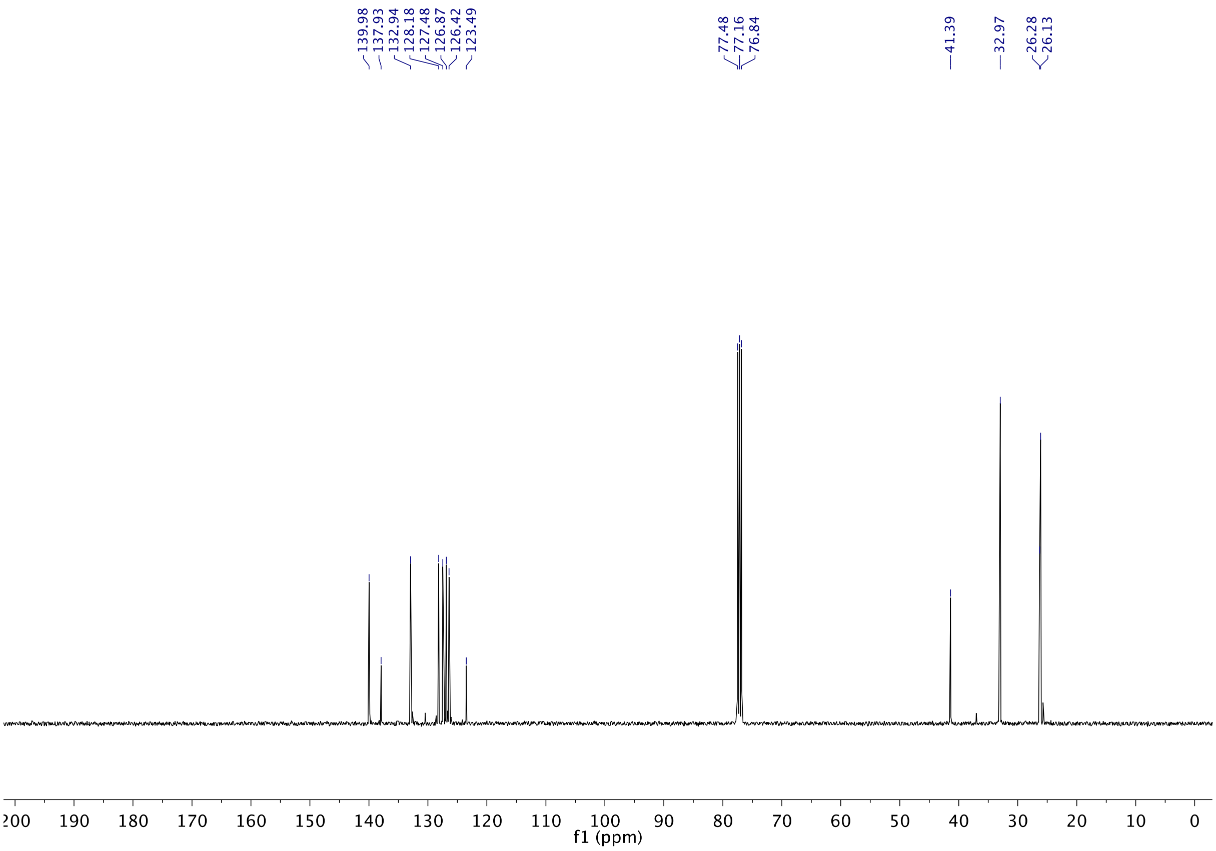

(*E*)-1-(2-Cyclohexylvinyl)naphthalene, **53**

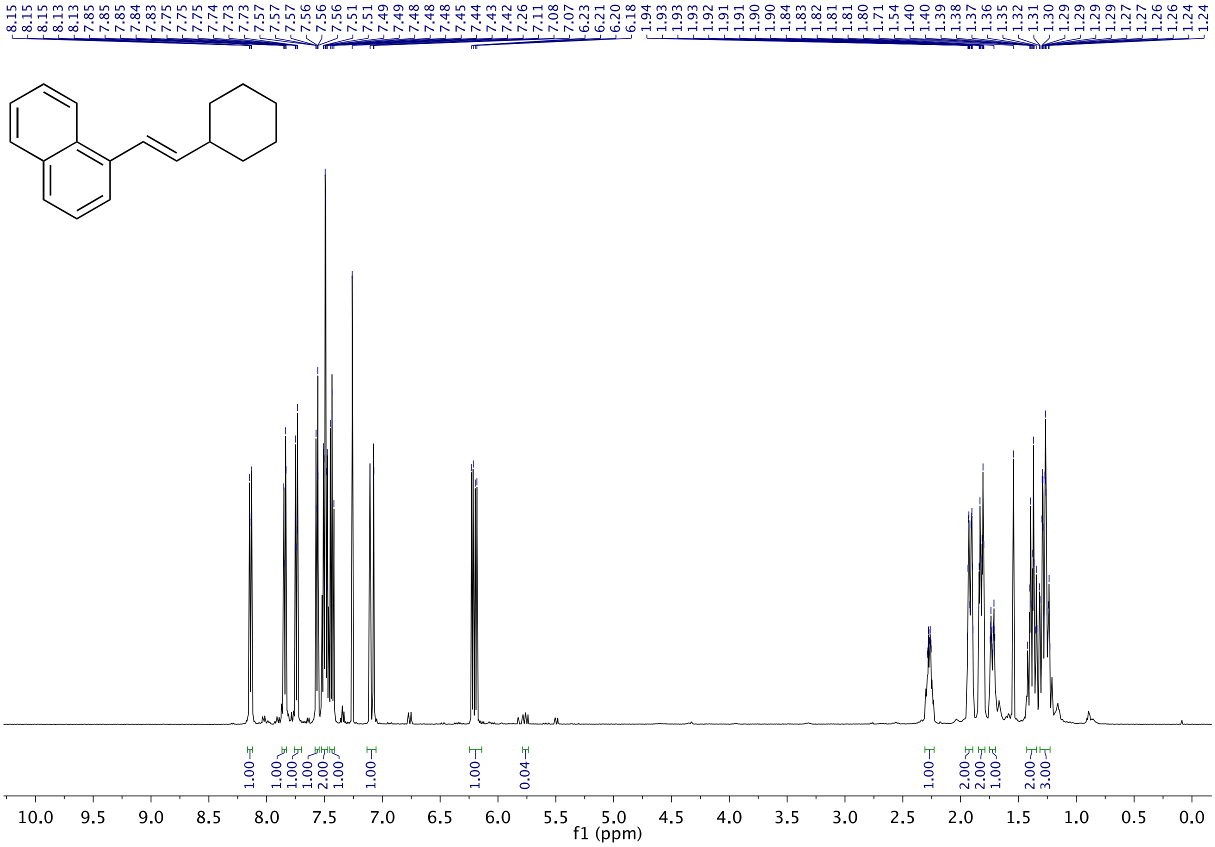

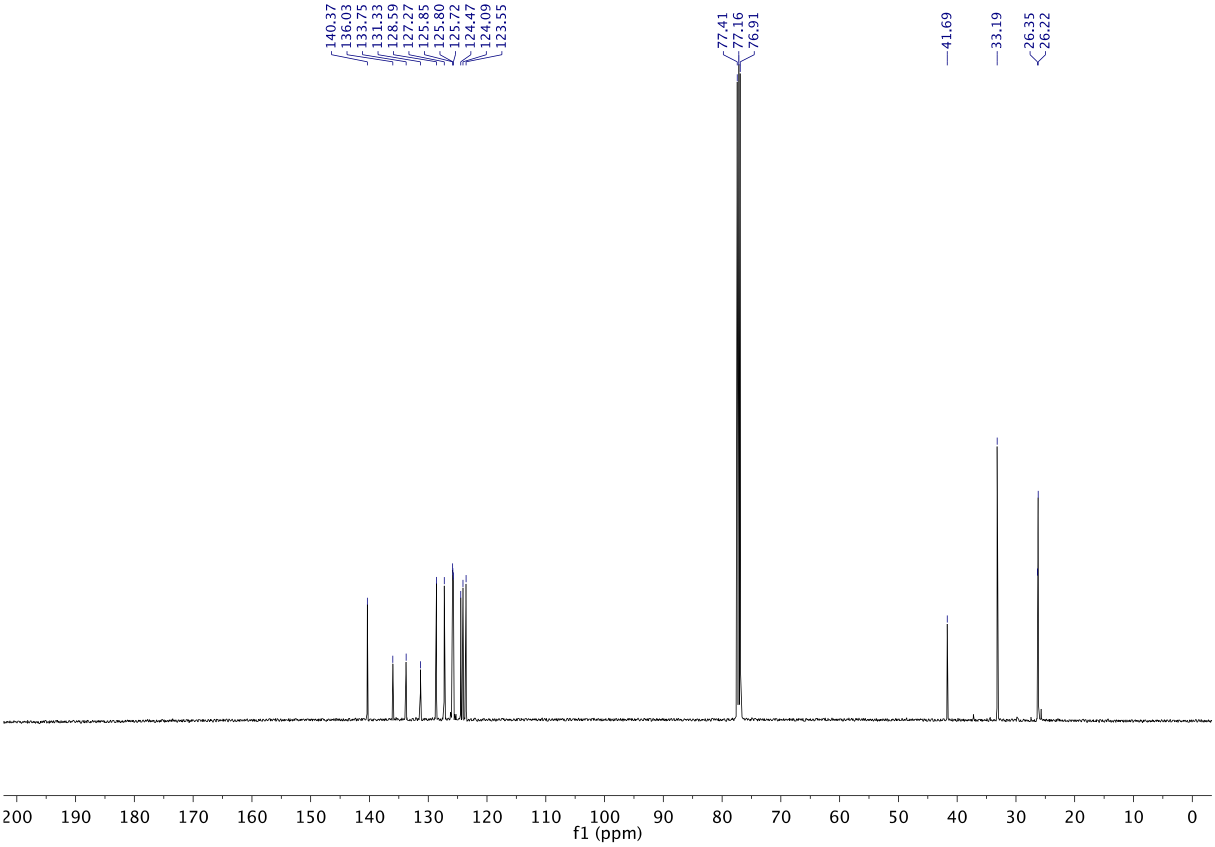

(*E*)-2-(2-Cyclohexylvinyl)pyridine, **54**

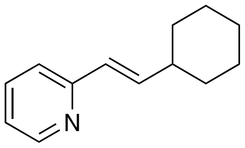

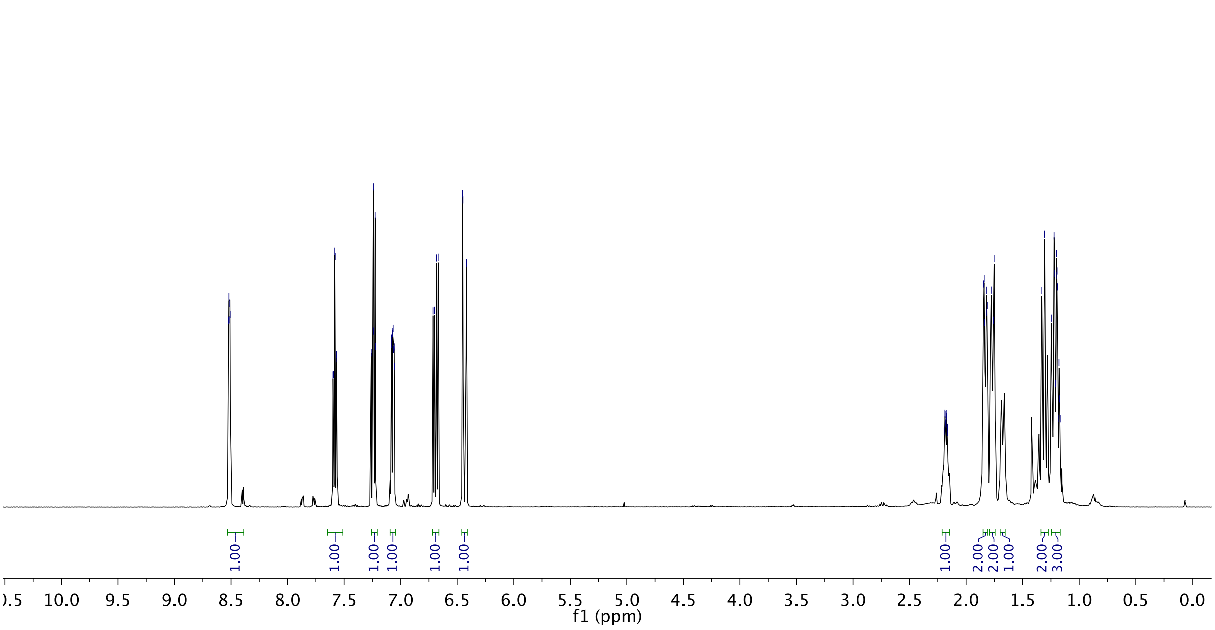

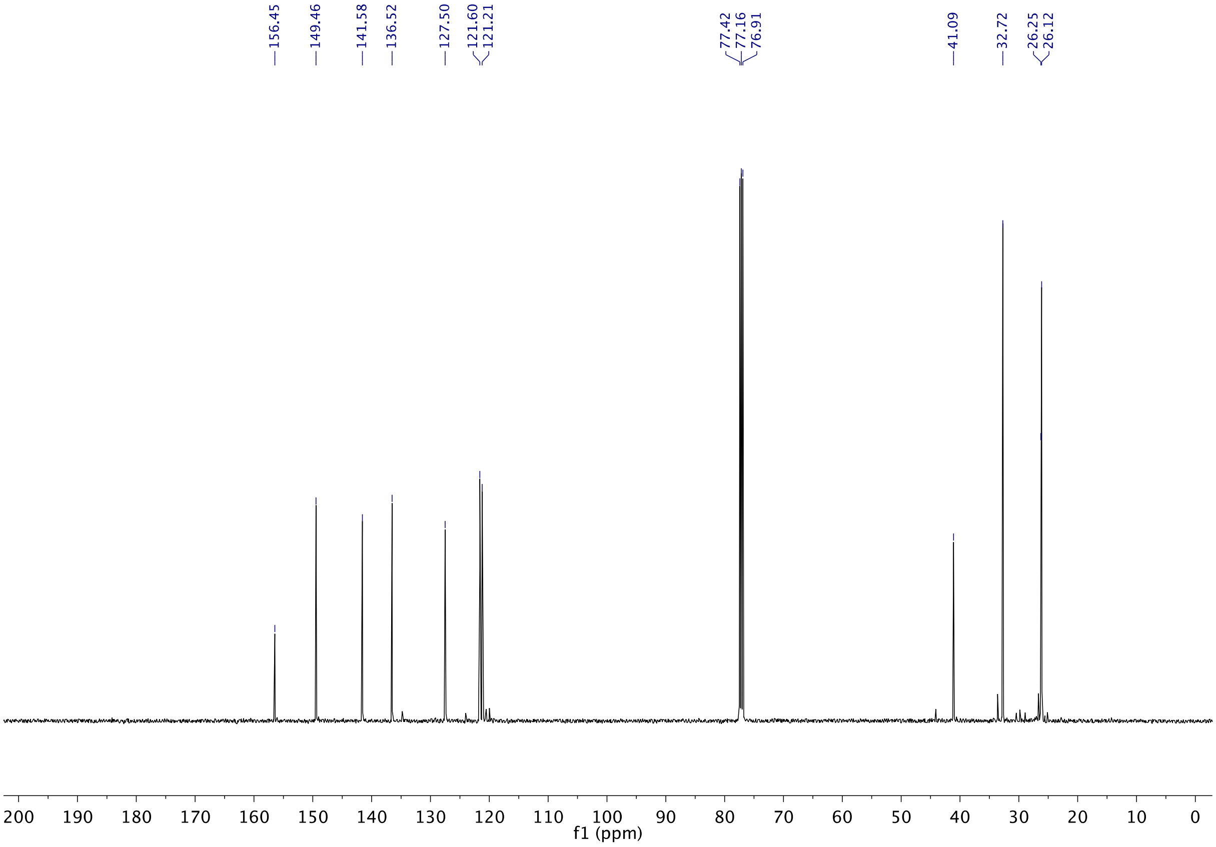

(*E*)-2-(2-Cyclohexylvinyl)thiophene, **55**

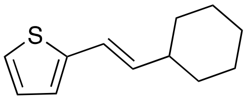

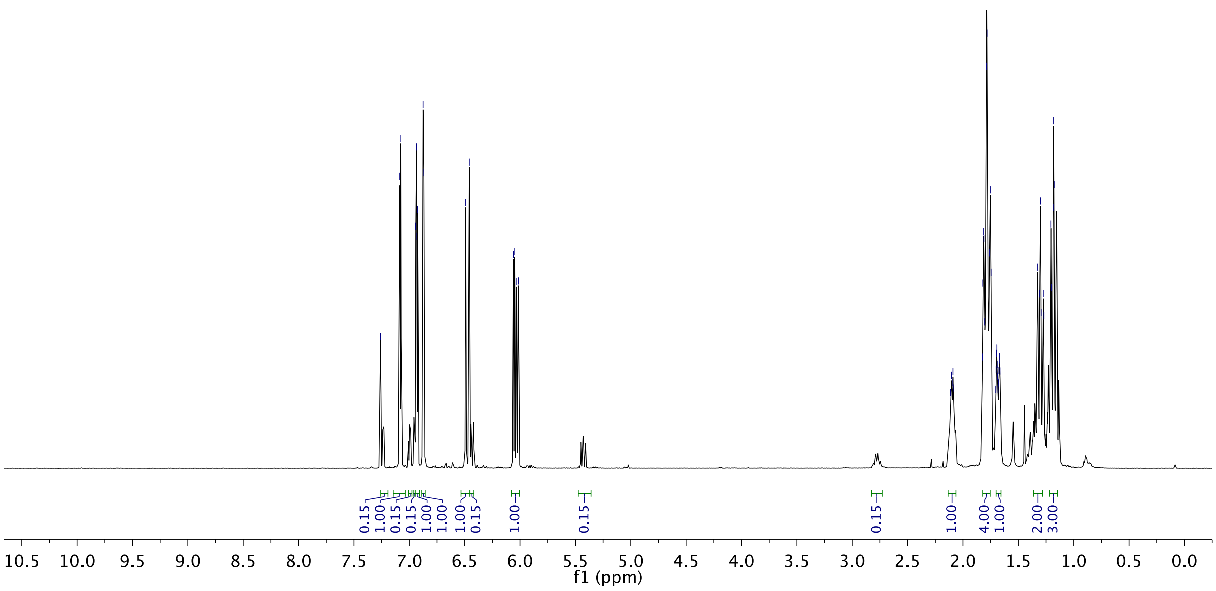

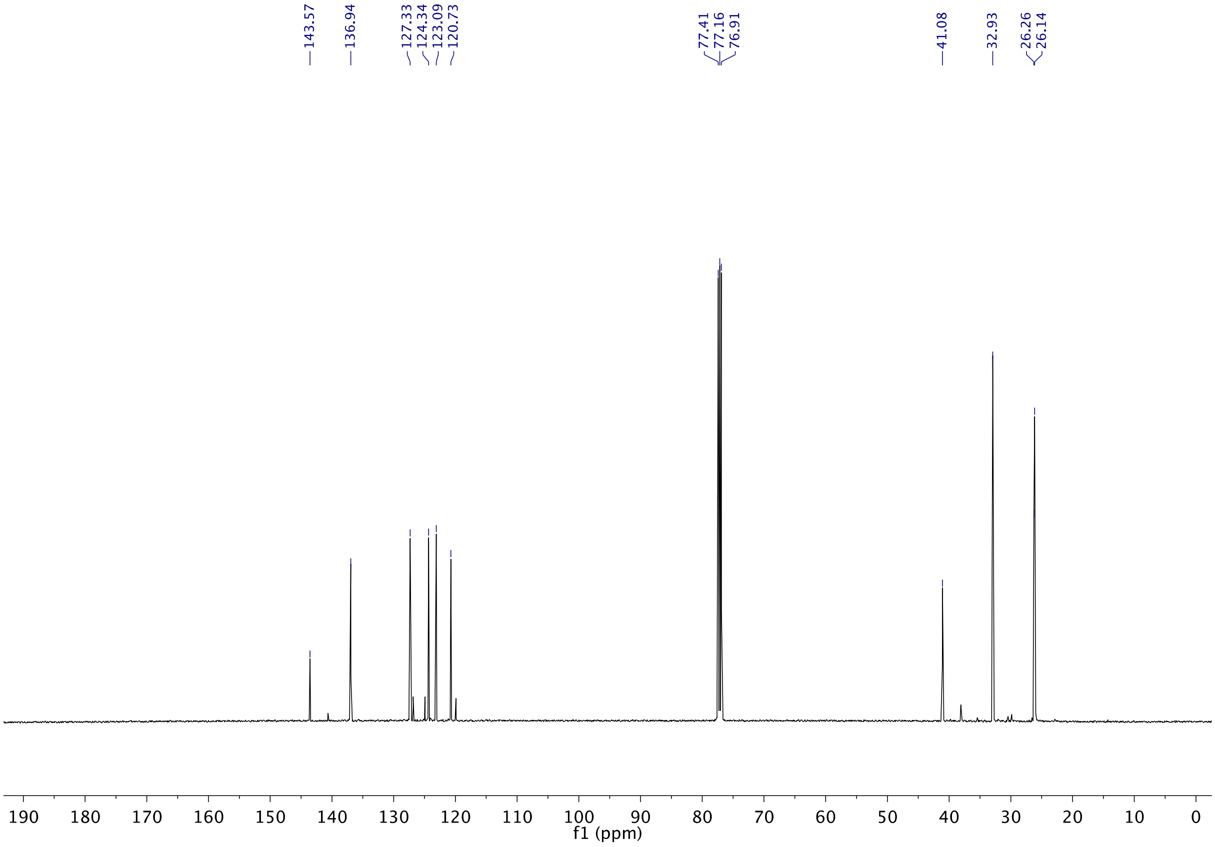

(*E*)-5-(2-Cyclohexylvinyl)benzofuran, **56**

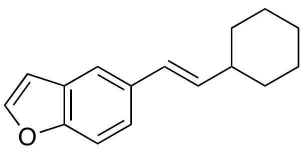

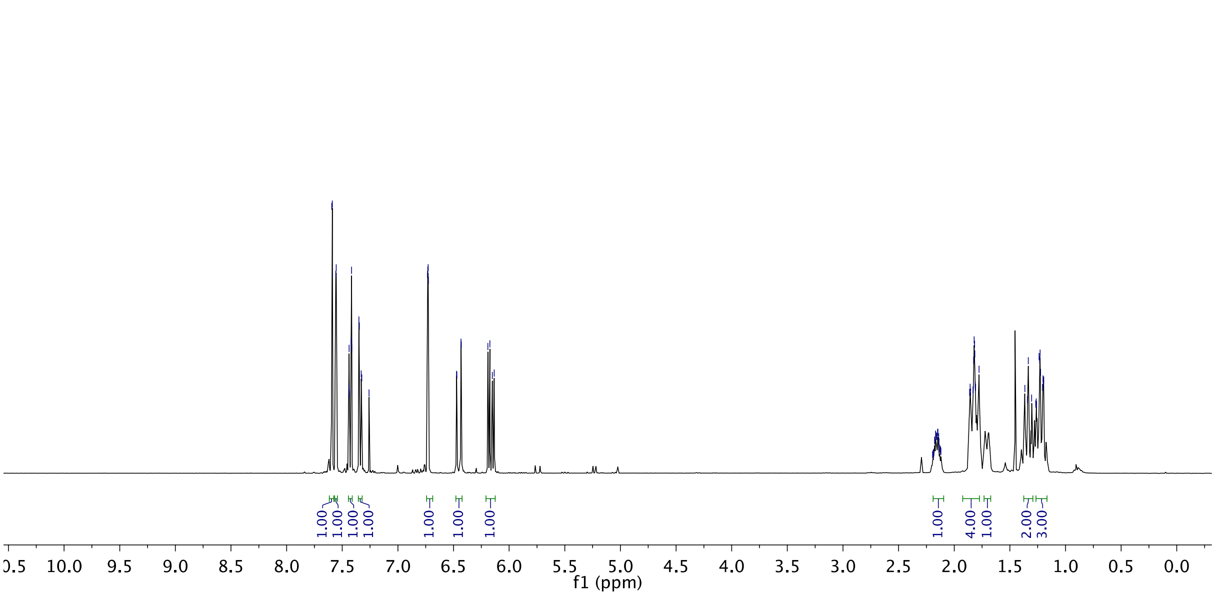

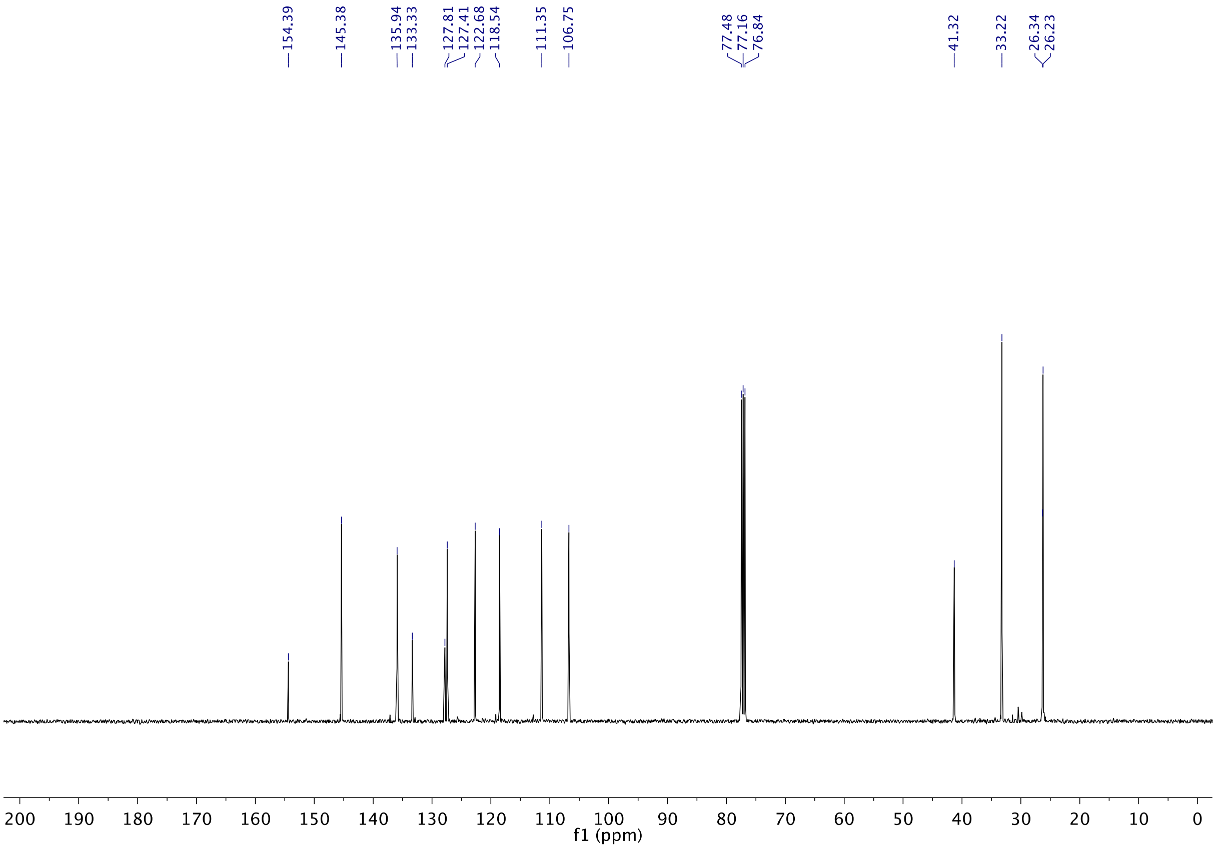

(*E*)-2-Bromo-1-(2-cyclohexylvinyl)-4-(trifluoromethoxy)benzene, **57**

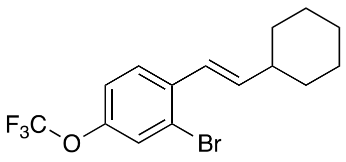

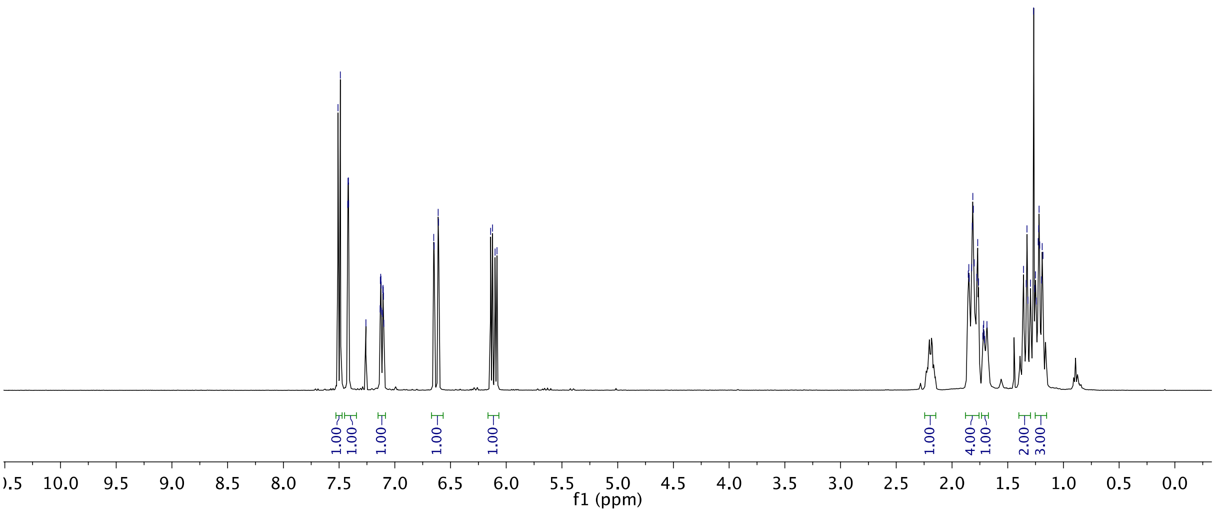

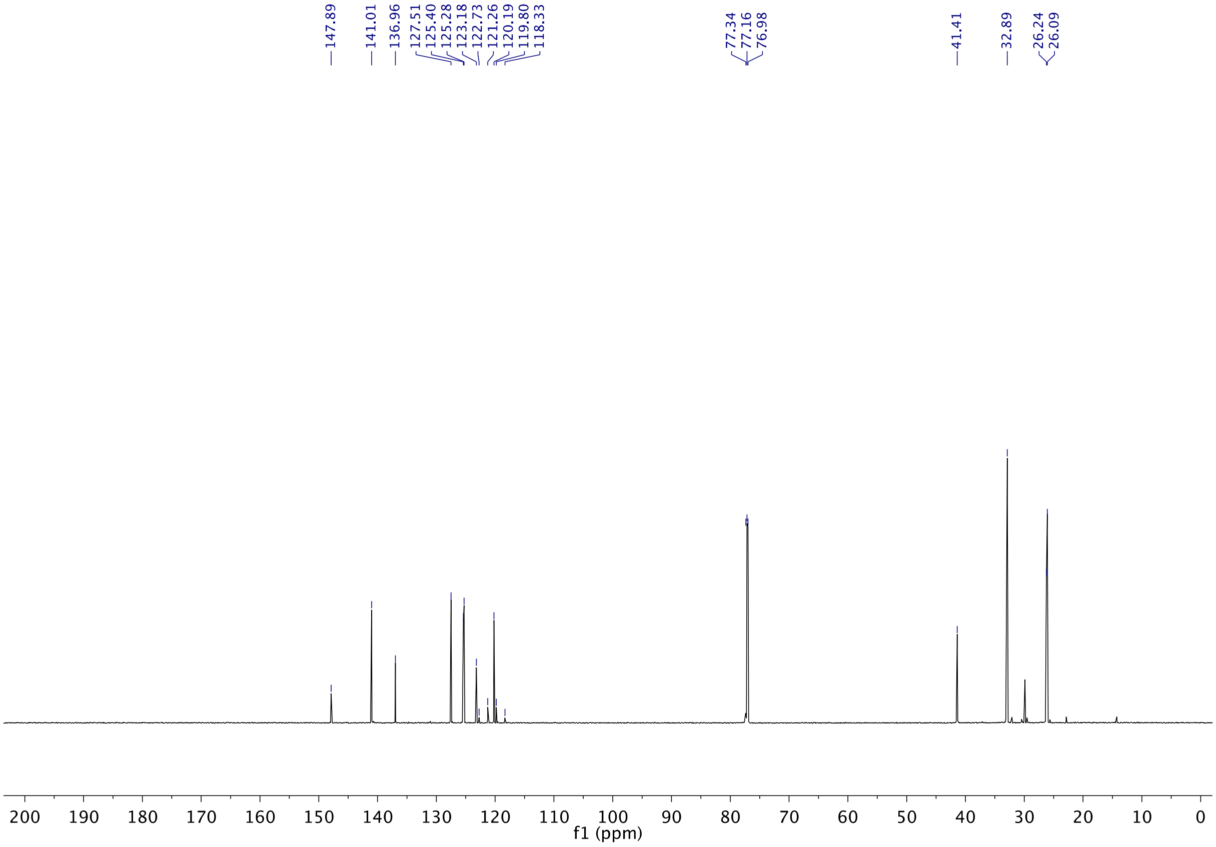

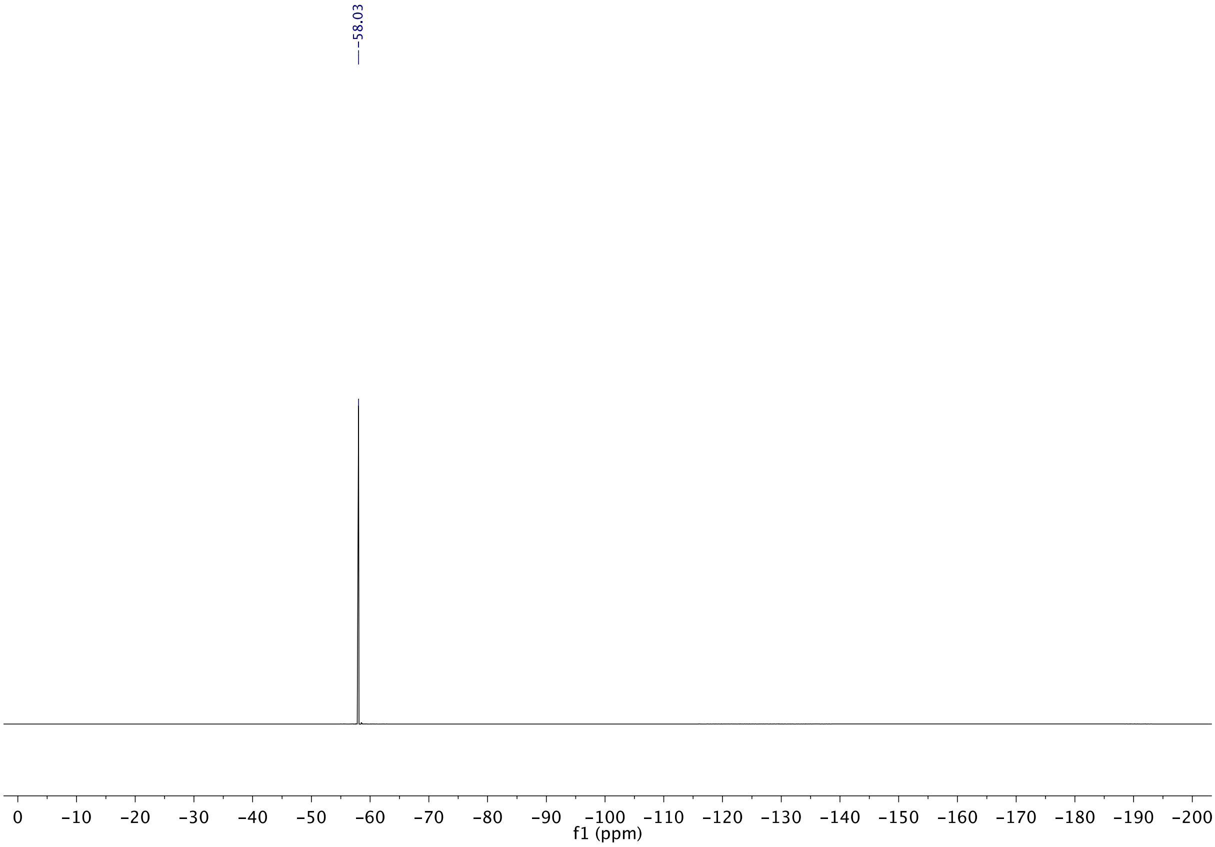

((1*E*,3*E*) and (1*Z*,3*E*)-4-Cyclohexylbuta-1,3-dien-1-yl)benzene, **58**

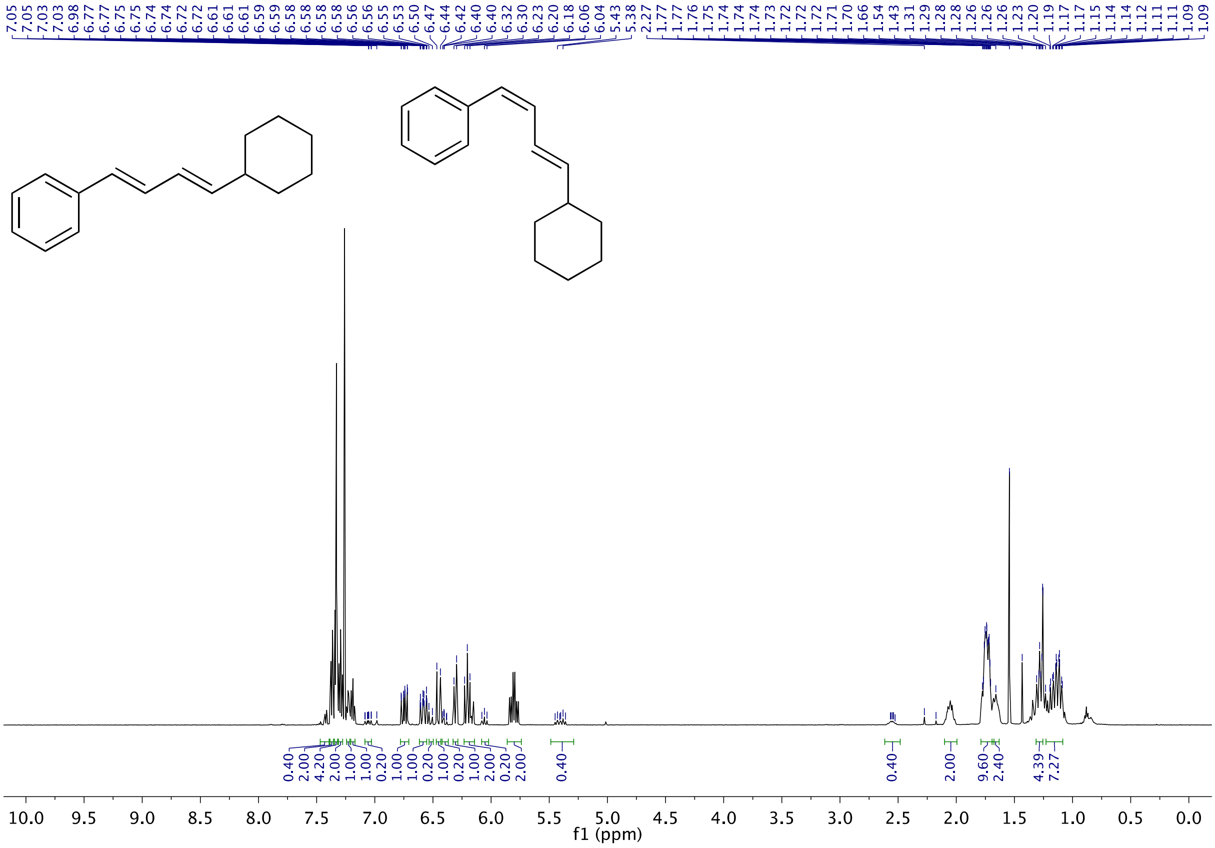

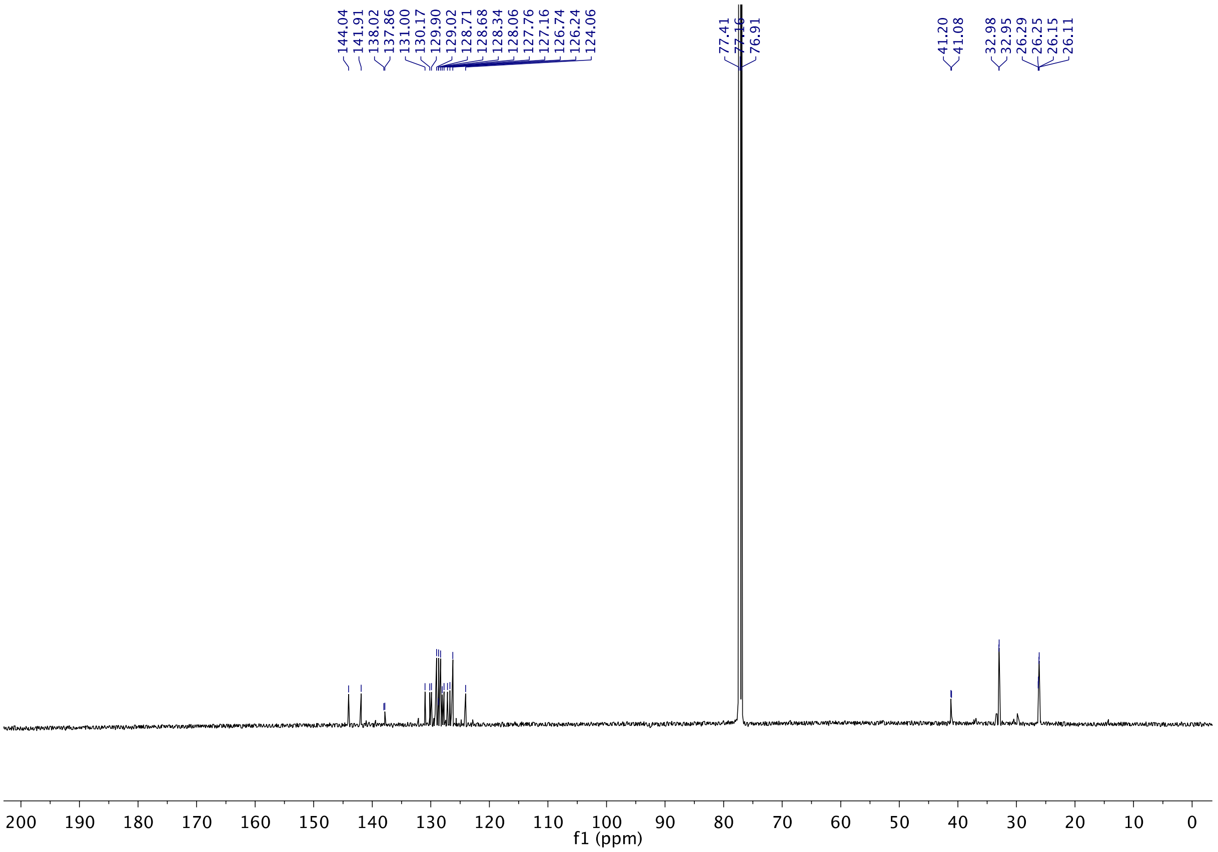

Methyl (*E*)-2-(2-cyclohexylvinyl)benzoate, **59**

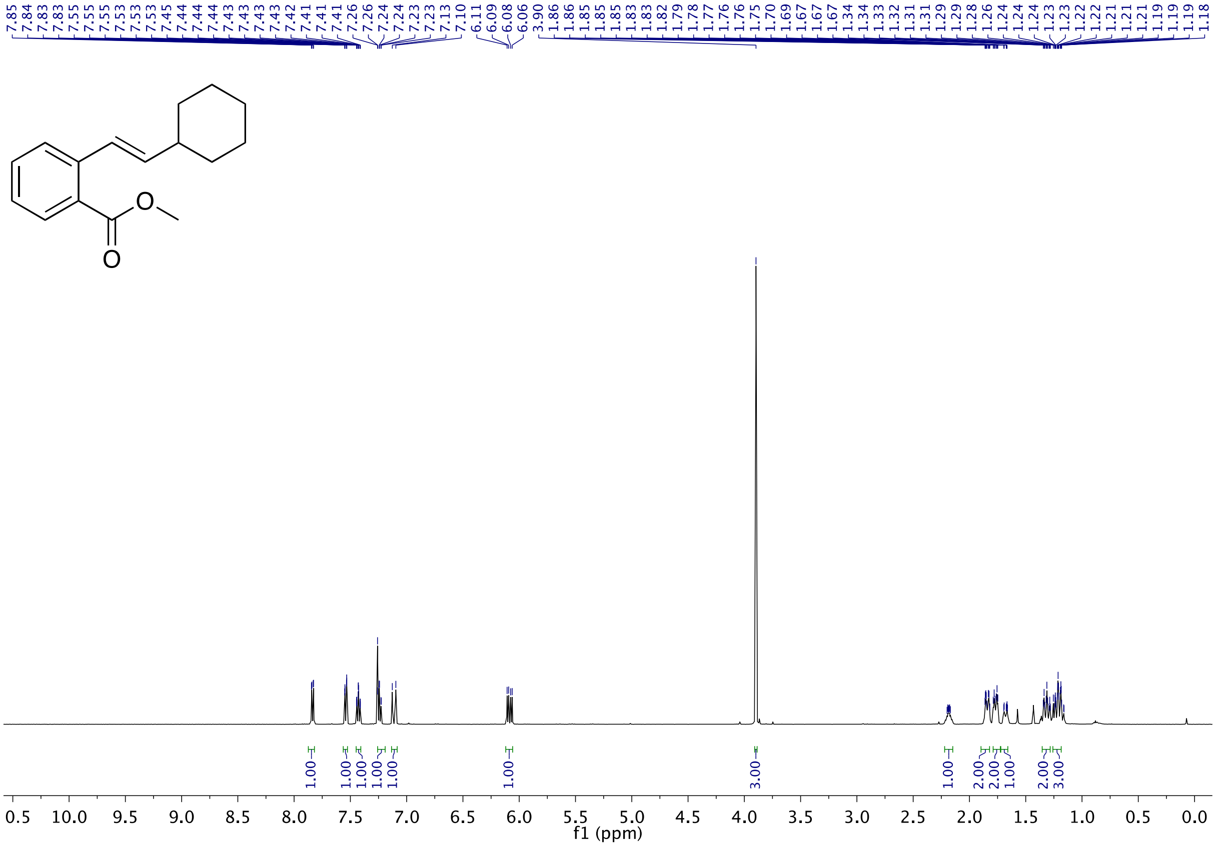

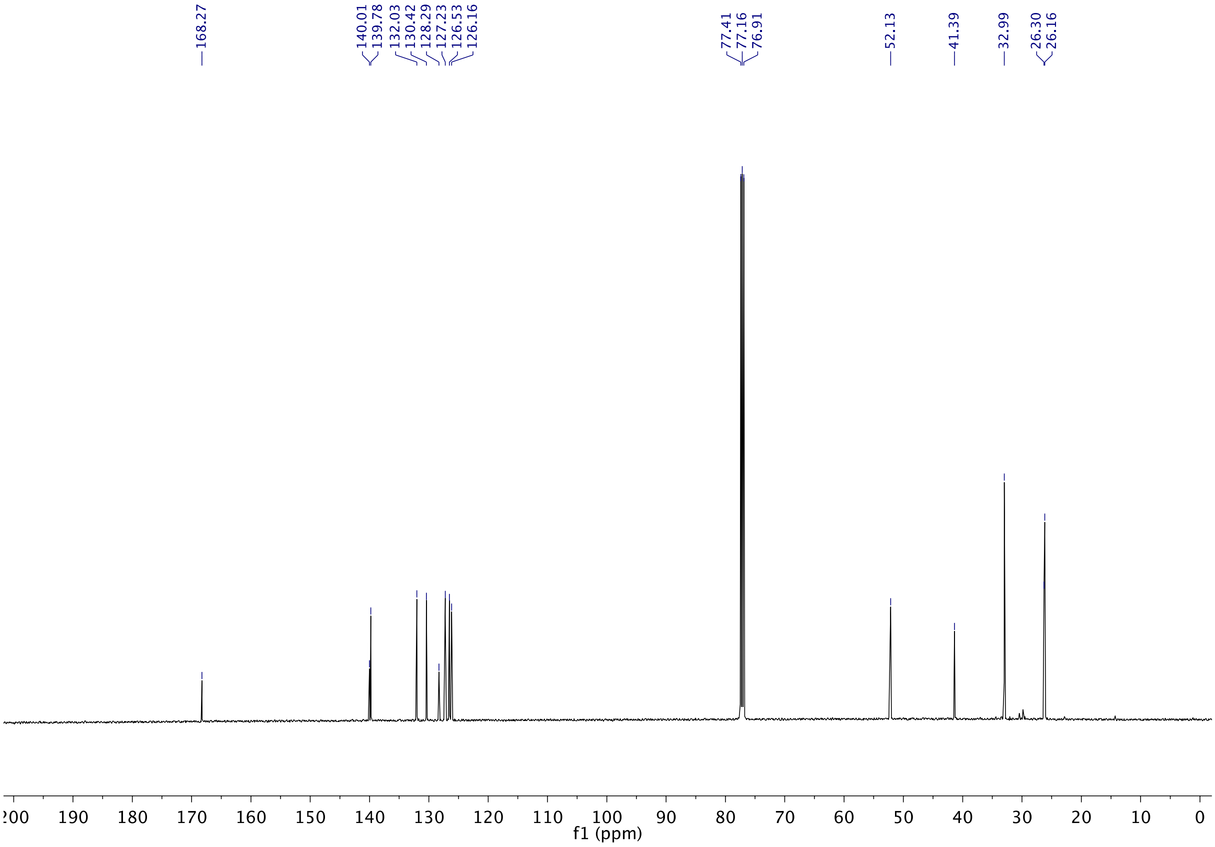

(*E*)-2-(2-Cyclohexylvinyl)-3'-methoxy-1,1'-biphenyl, **60**

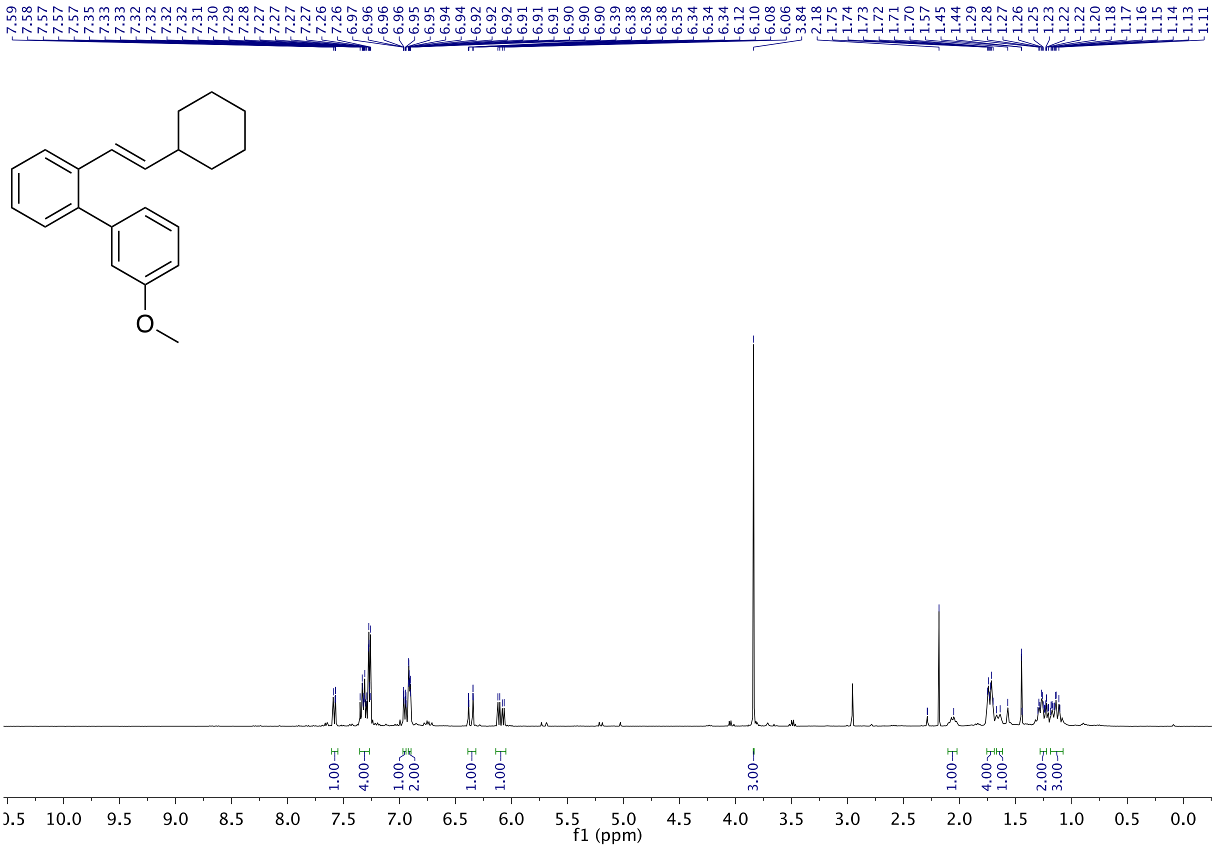

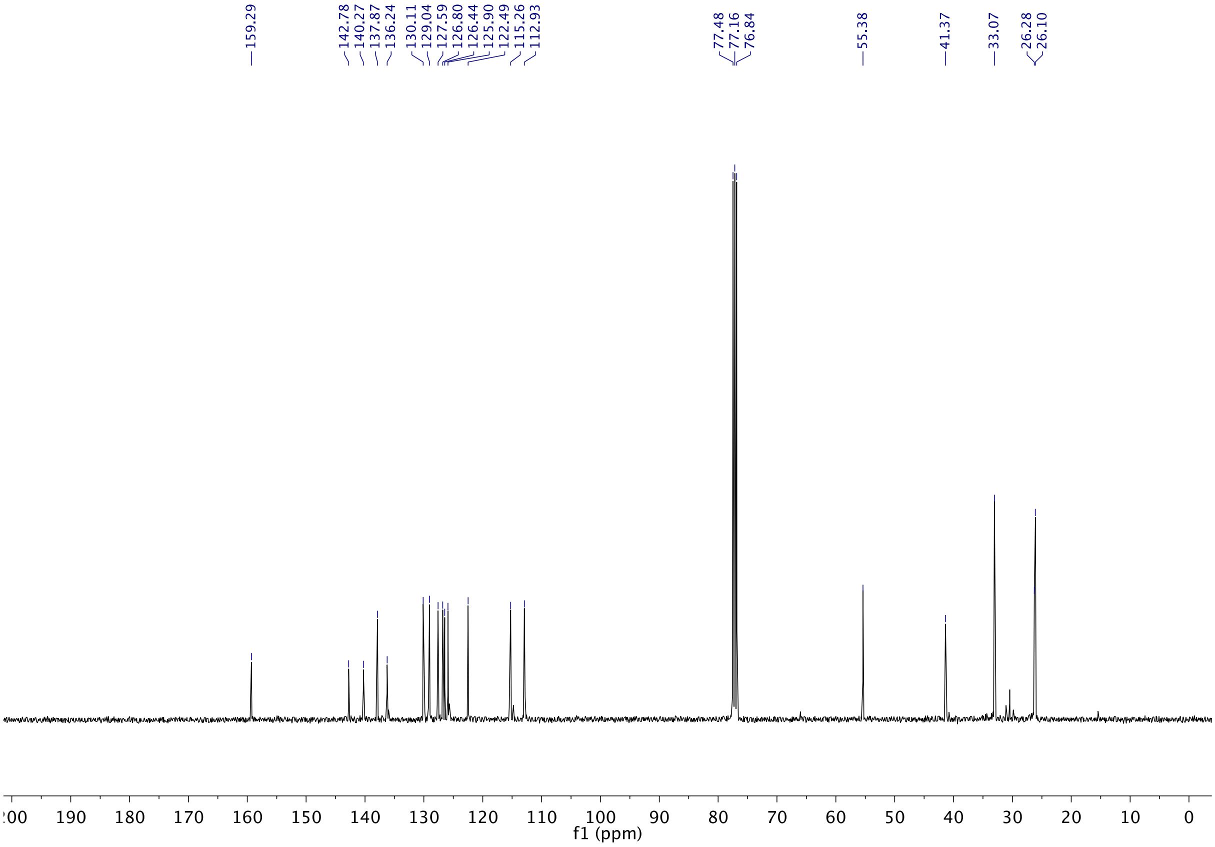

(*E*)-Octa-1,7-dien-1-ylbenzene, **62**

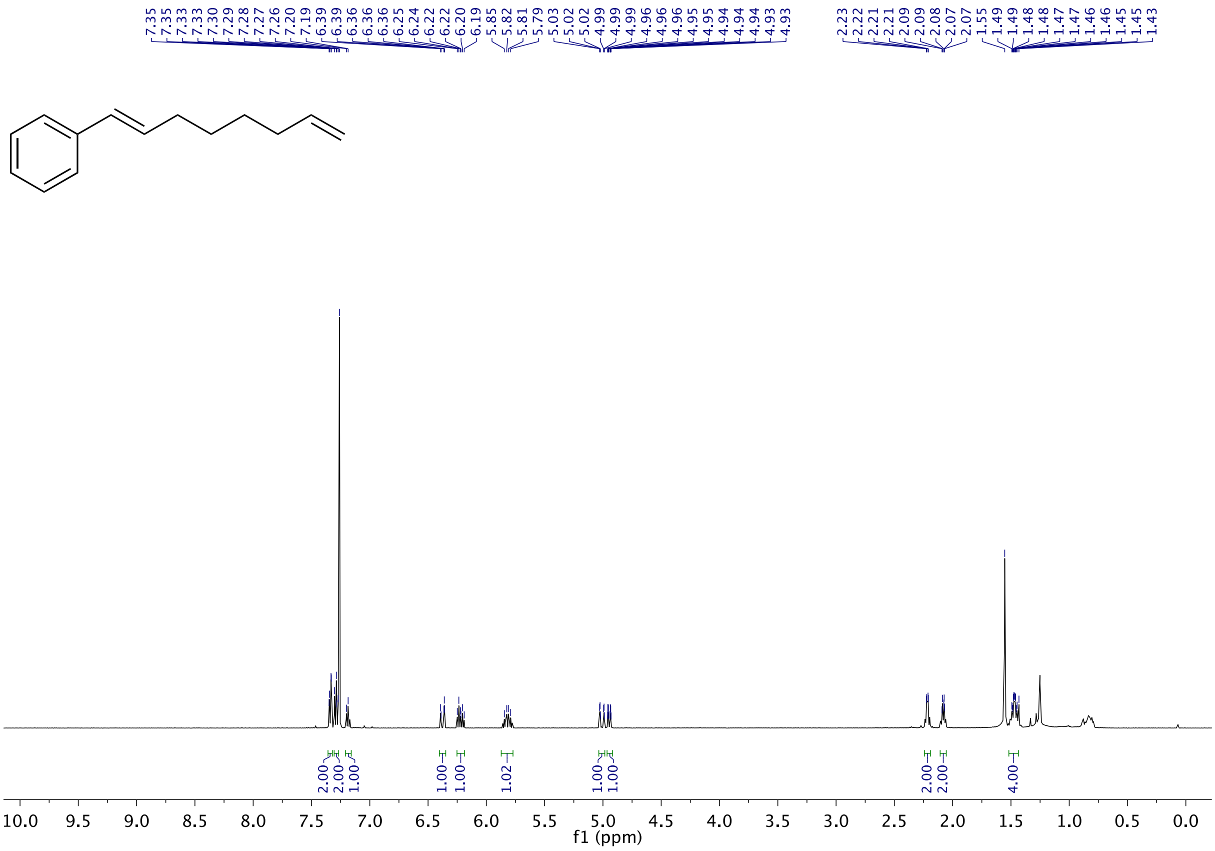

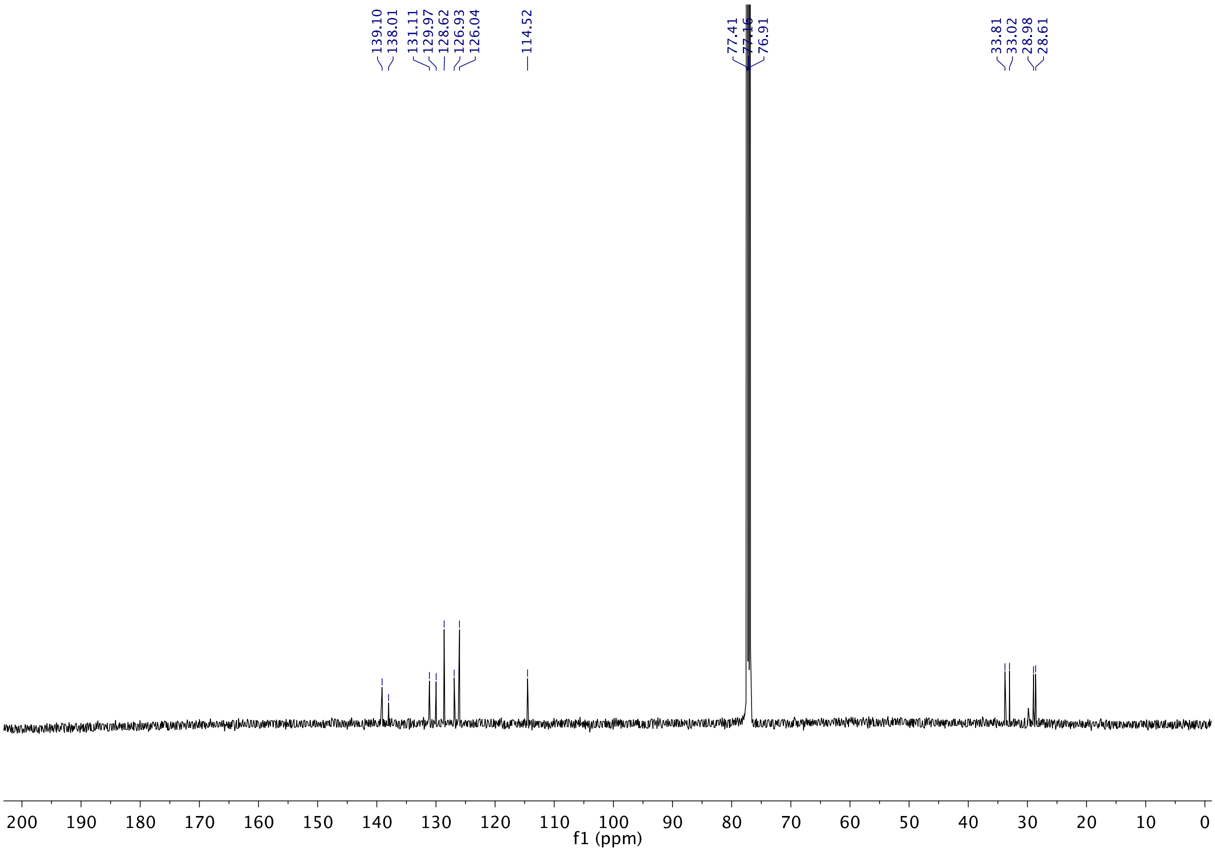

(*E*)-(3-Cyclopentylprop-1-en-1-yl)benzene, **63**

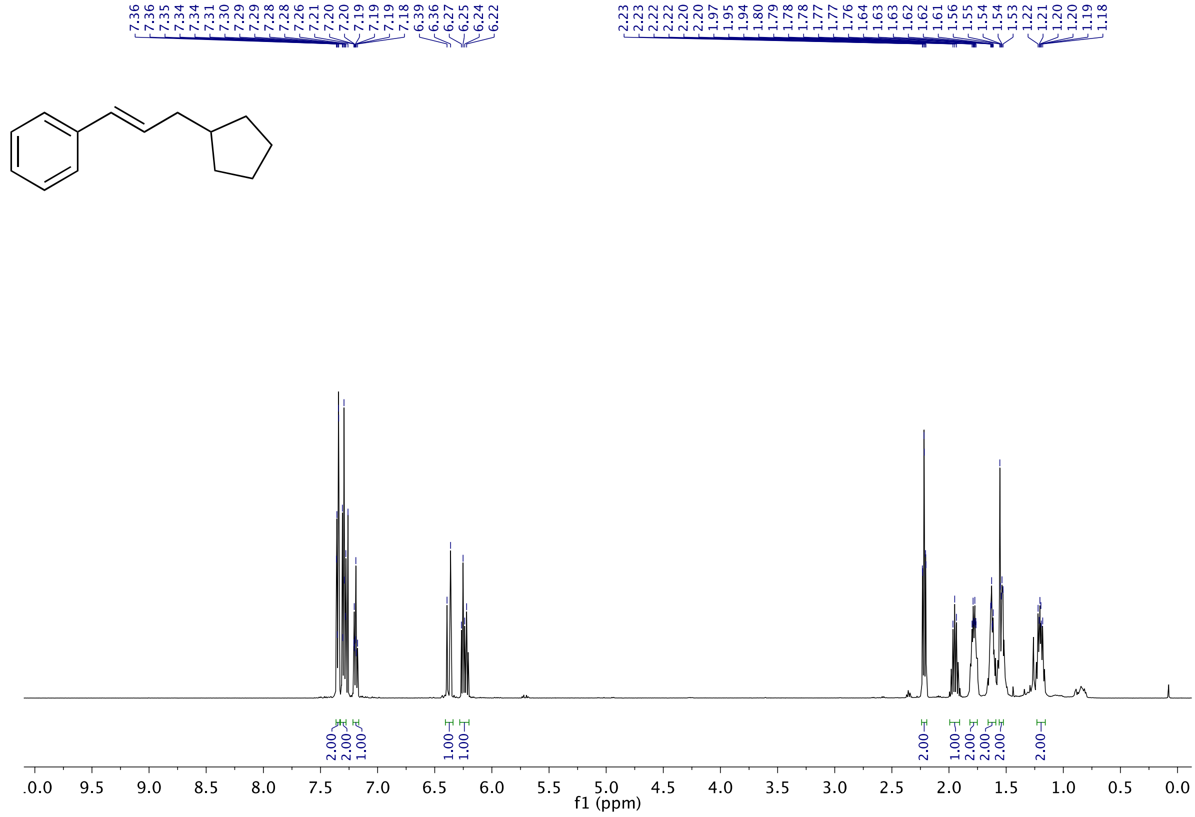

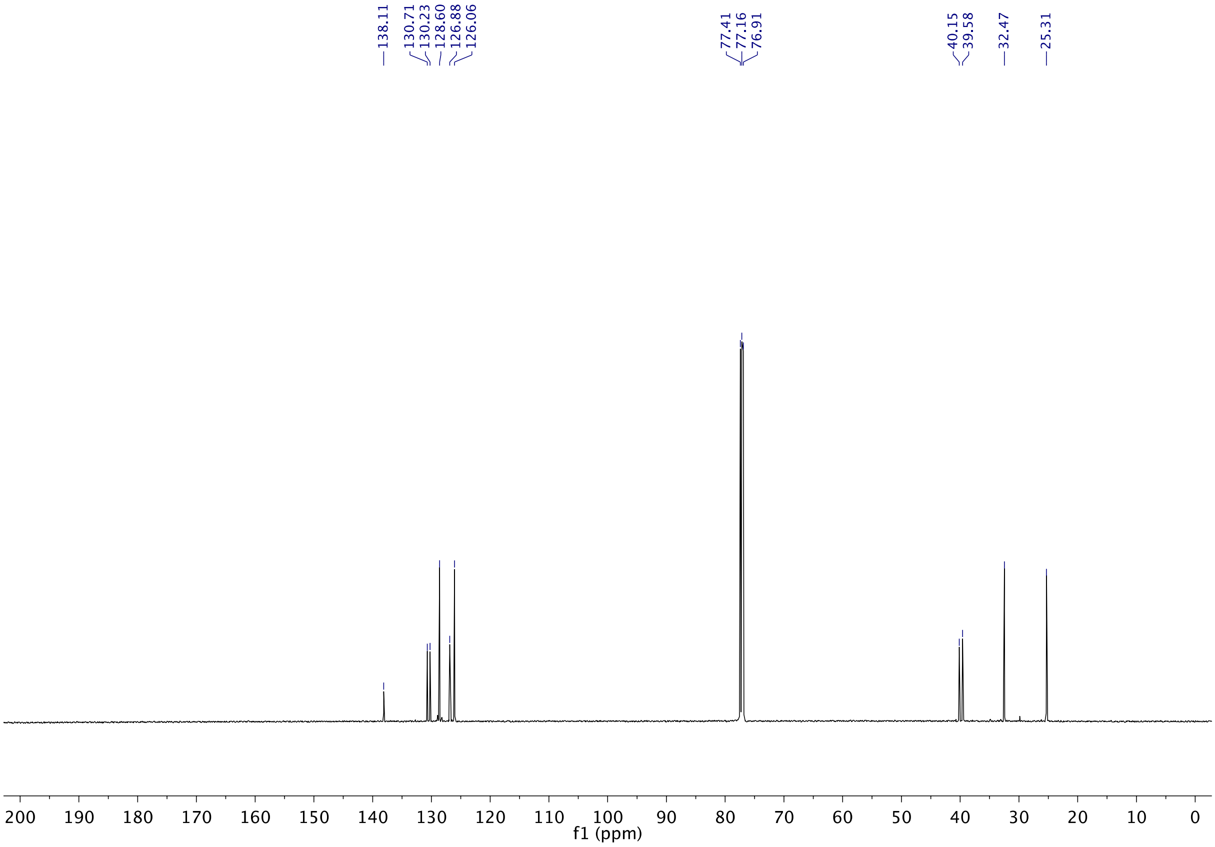

2-cyclohexyl-1-(4-fluorophenyl)ethan-1-one, **75**

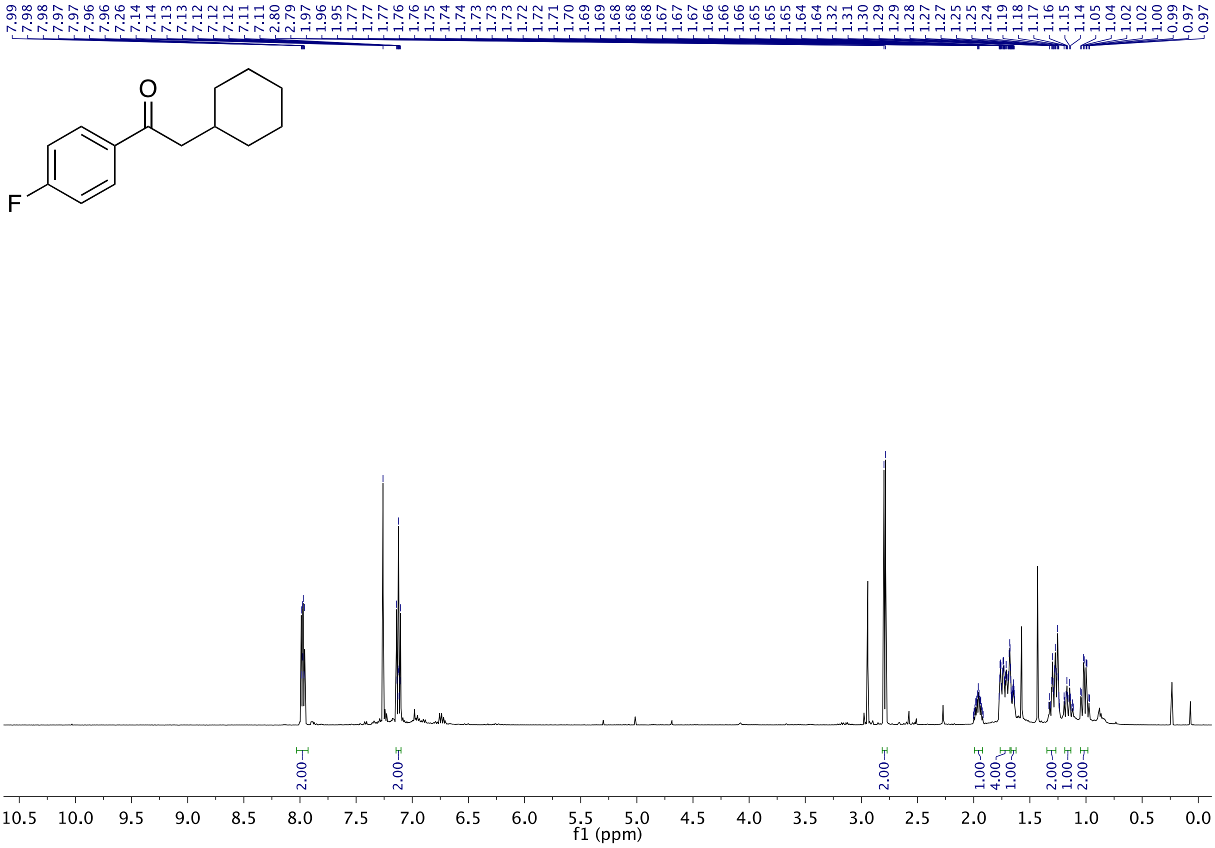

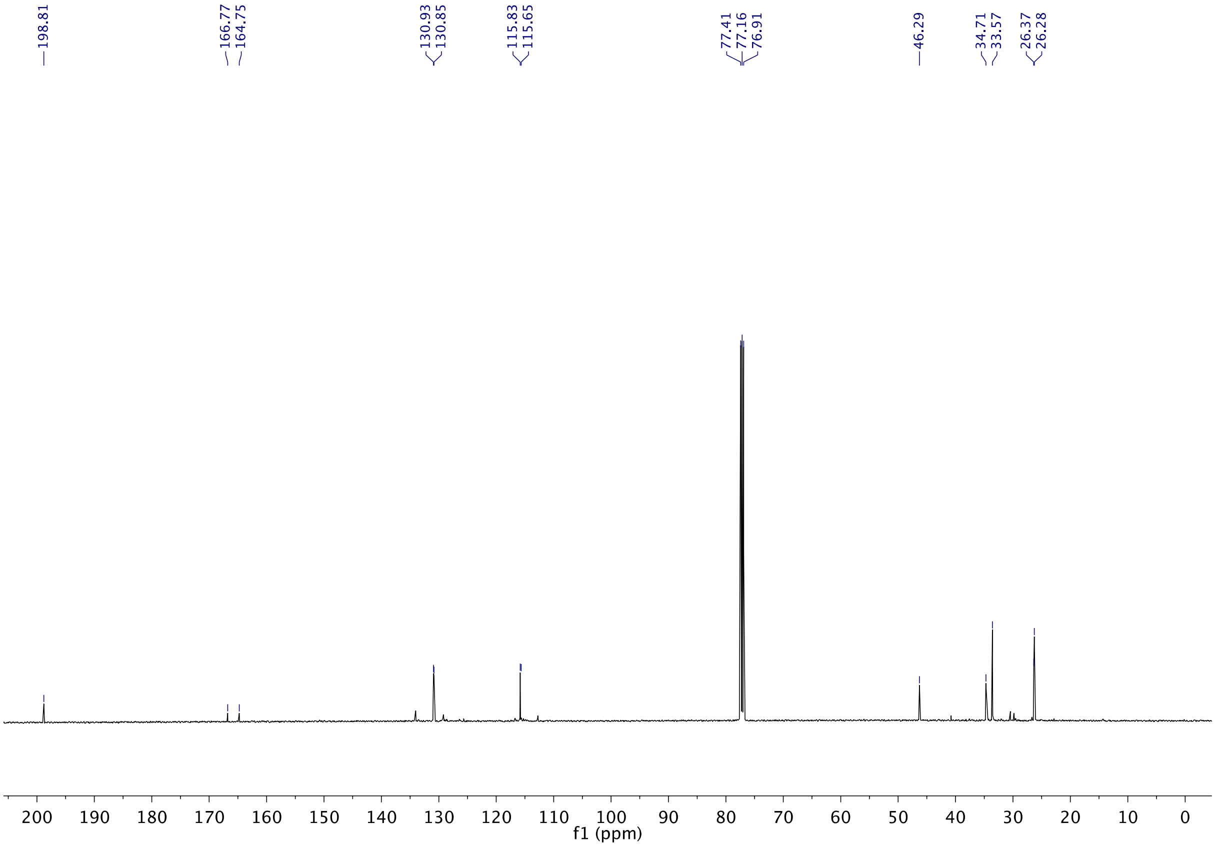
198.81
166.77
164.75
130.93
130.85
115.83
115.65
77.41
77.16
76.91
46.29
34.71
33.57
26.37
26.28
200 190 180 170 160 150 140 130 120 110 100 90 80 70 60 50 40 30 20 10 0
f1 (ppm)

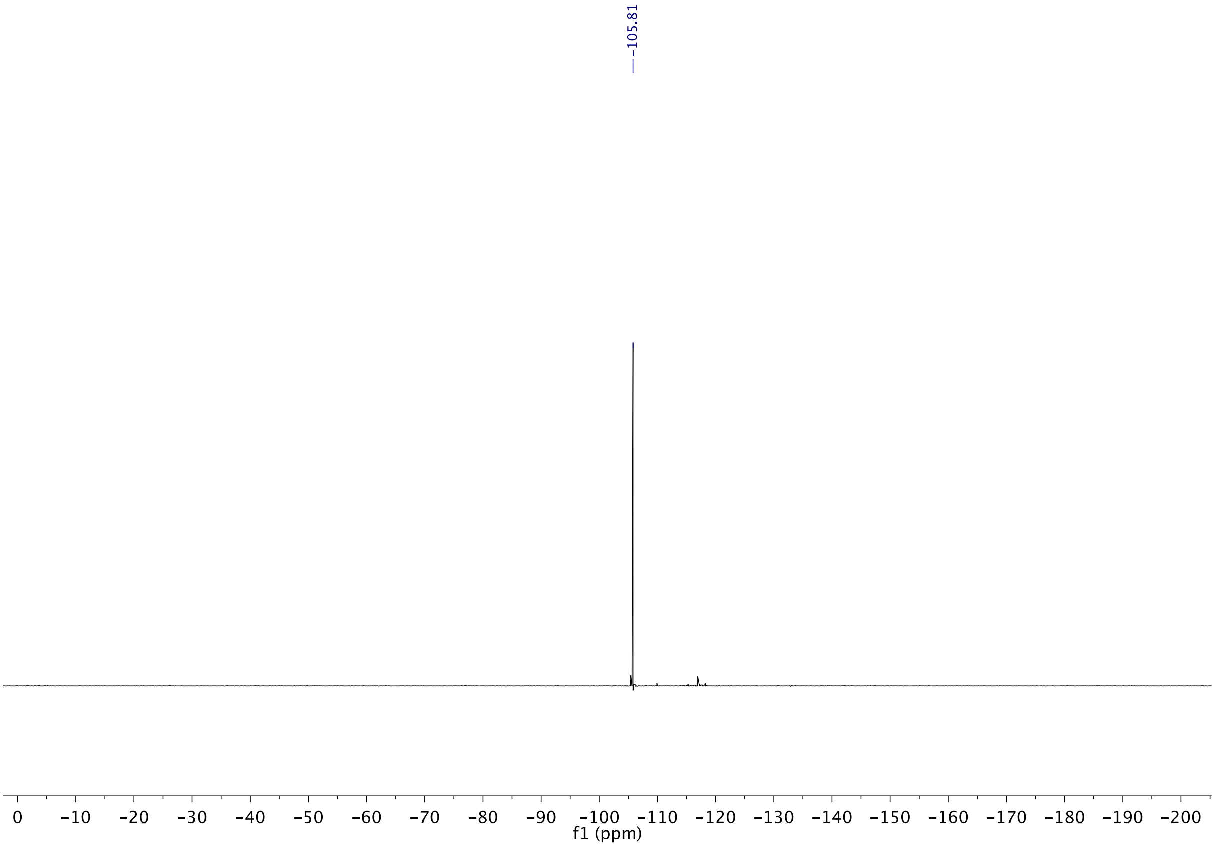
−105.81
0 −10 −20 −30 −40 −50 −60 −70 −80 −90 −100 −110 −120 −130 −140 −150 −160 −170 −180 −190 −200
f1 (ppm)

(*E*)-(1-Cyclohexylprop-1-en-2-yl)benzene, **P1**

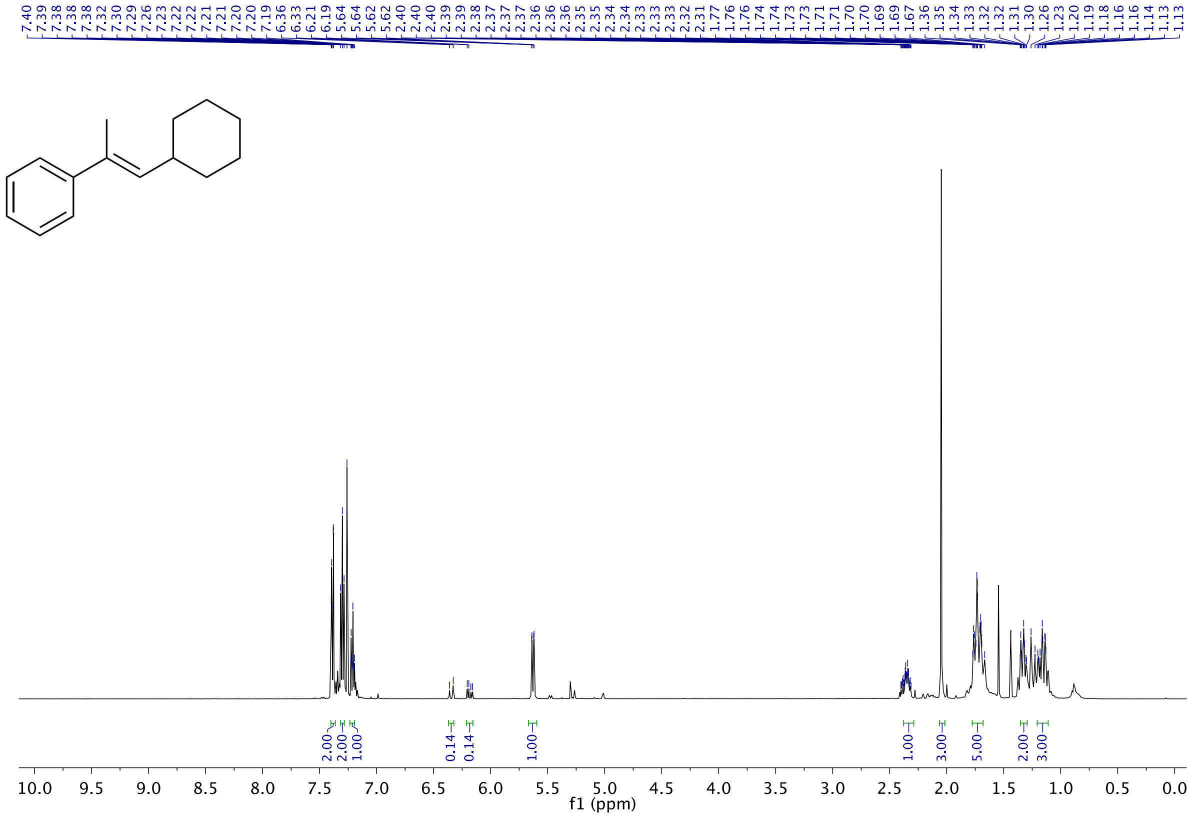

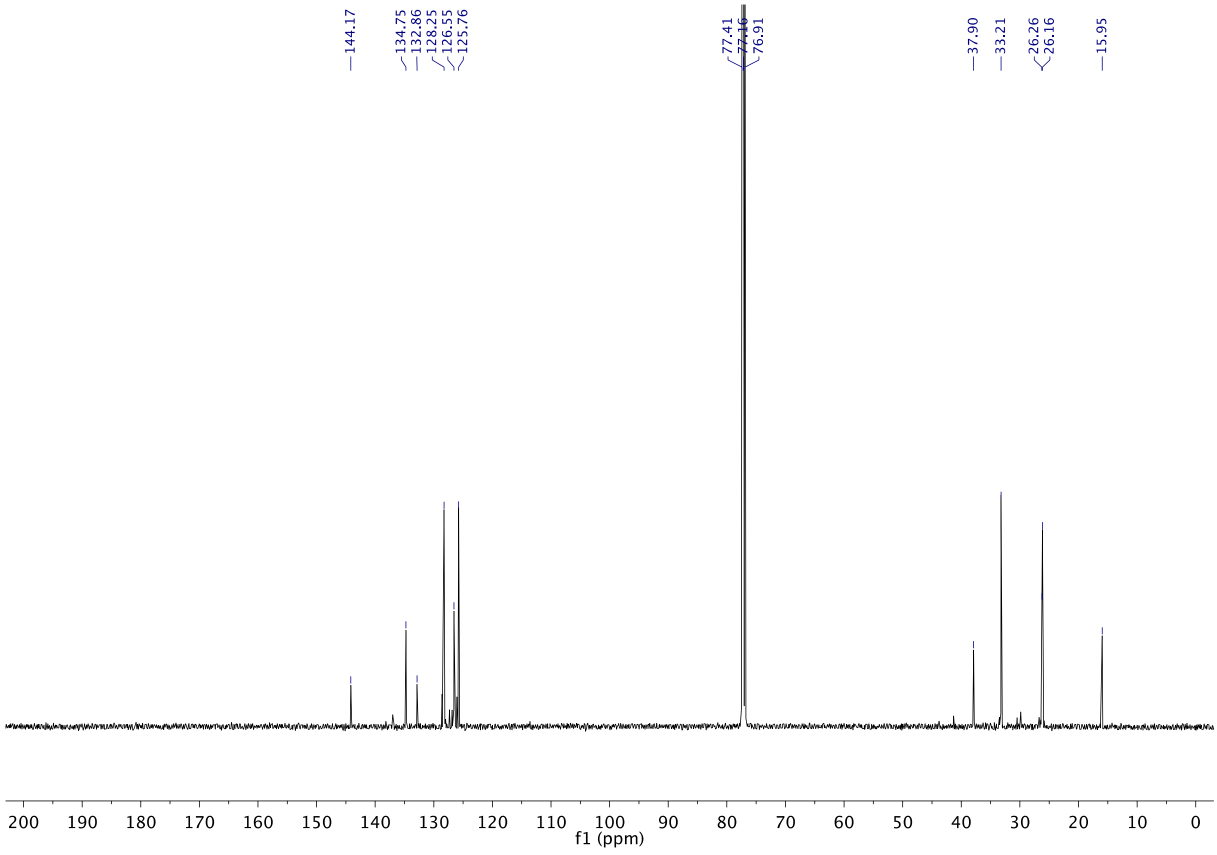

tert-Butyl (*E*)-2-styrylindoline-1-carboxylate, **P3**

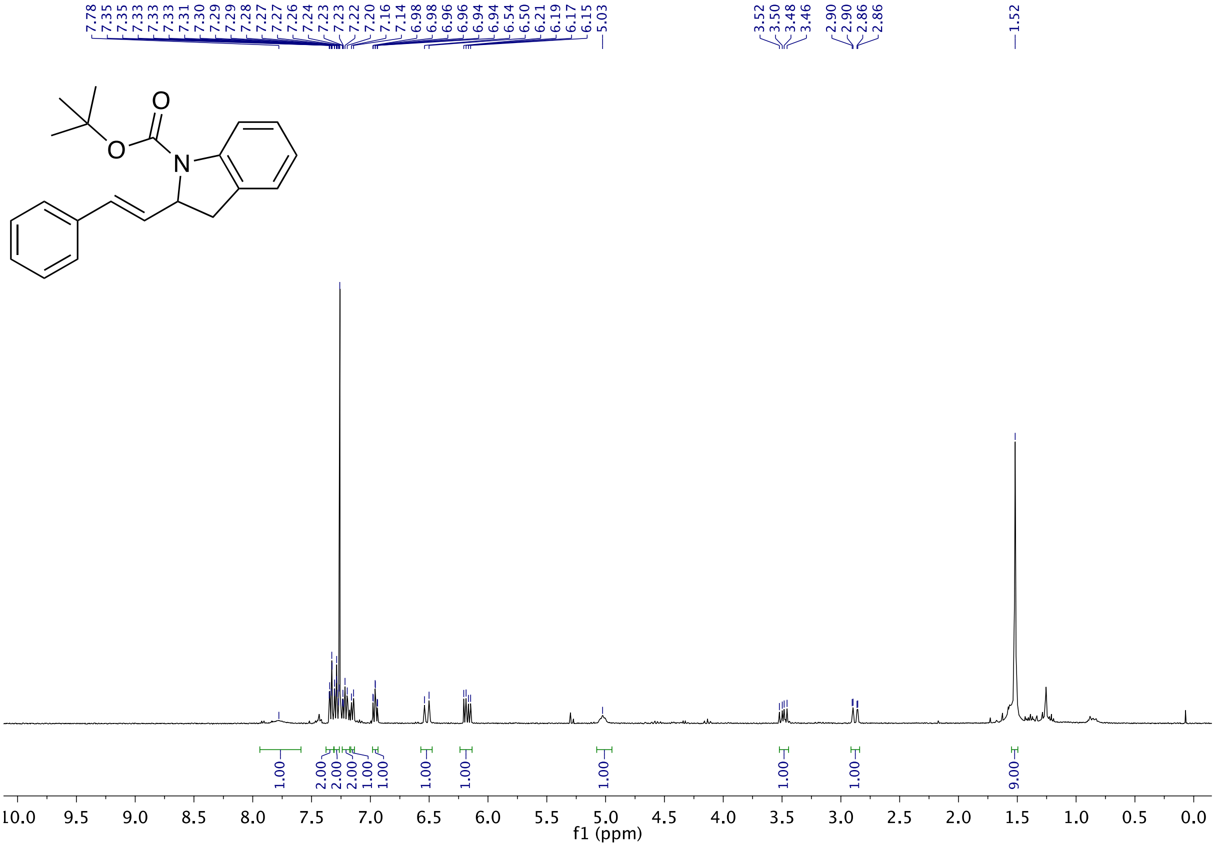

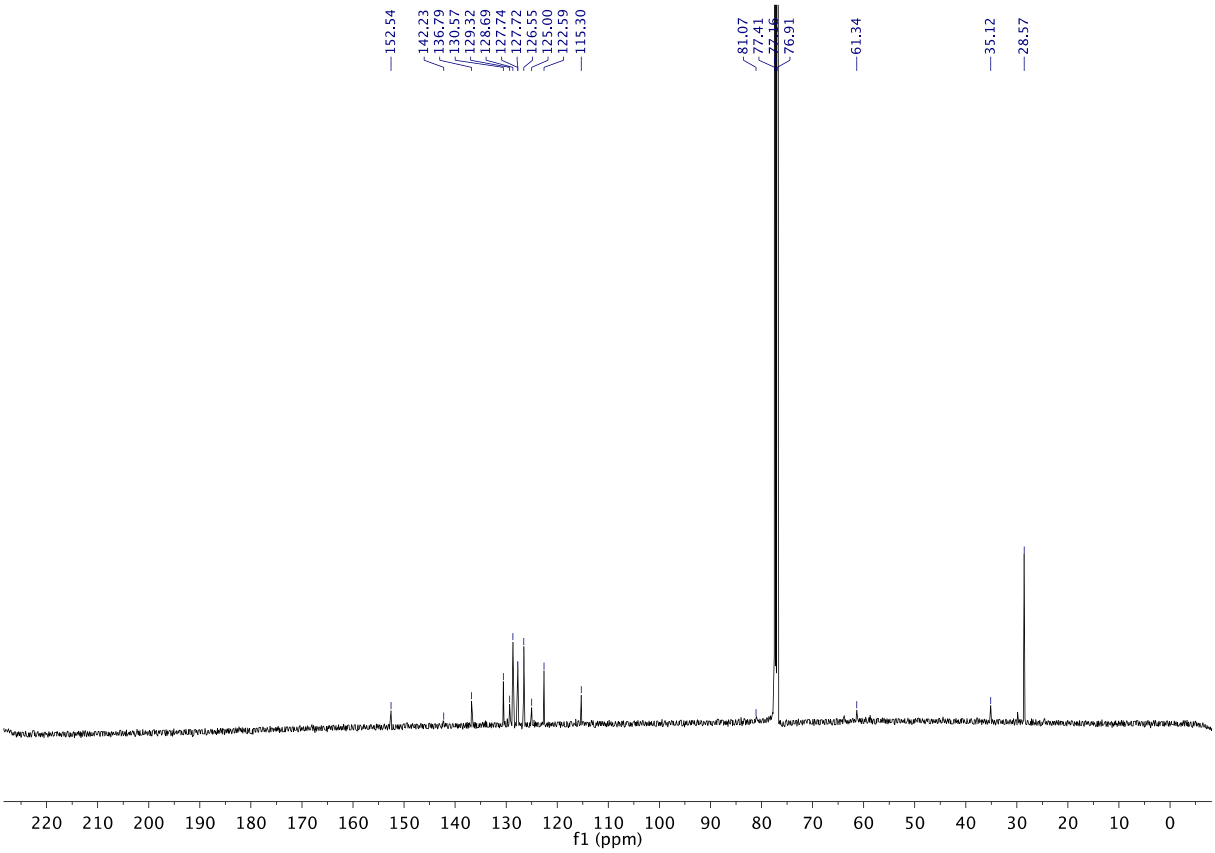

tert-butyl 1*H*-indole-1-carboxylate, **P4**

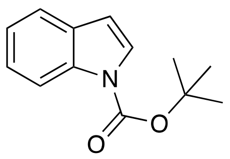

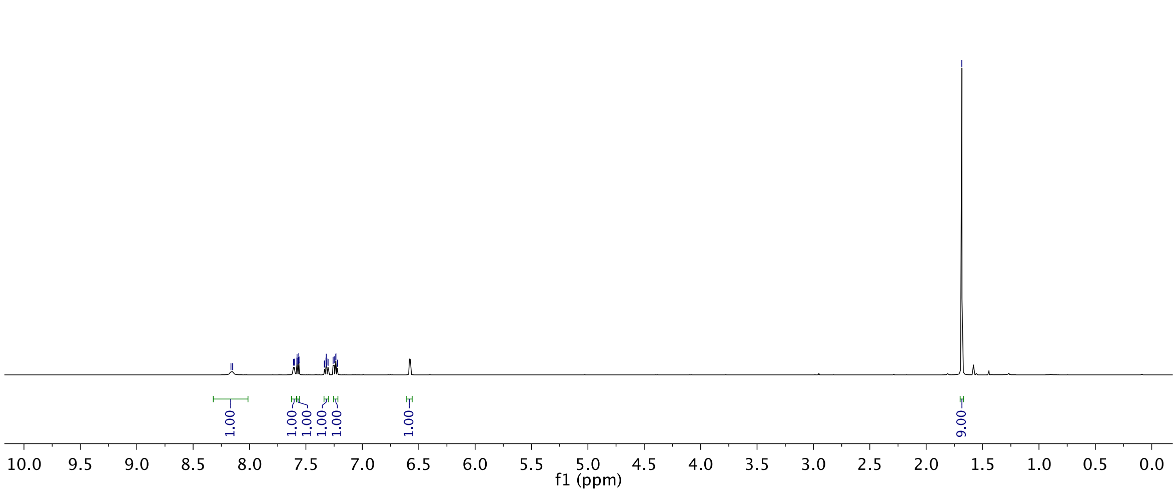

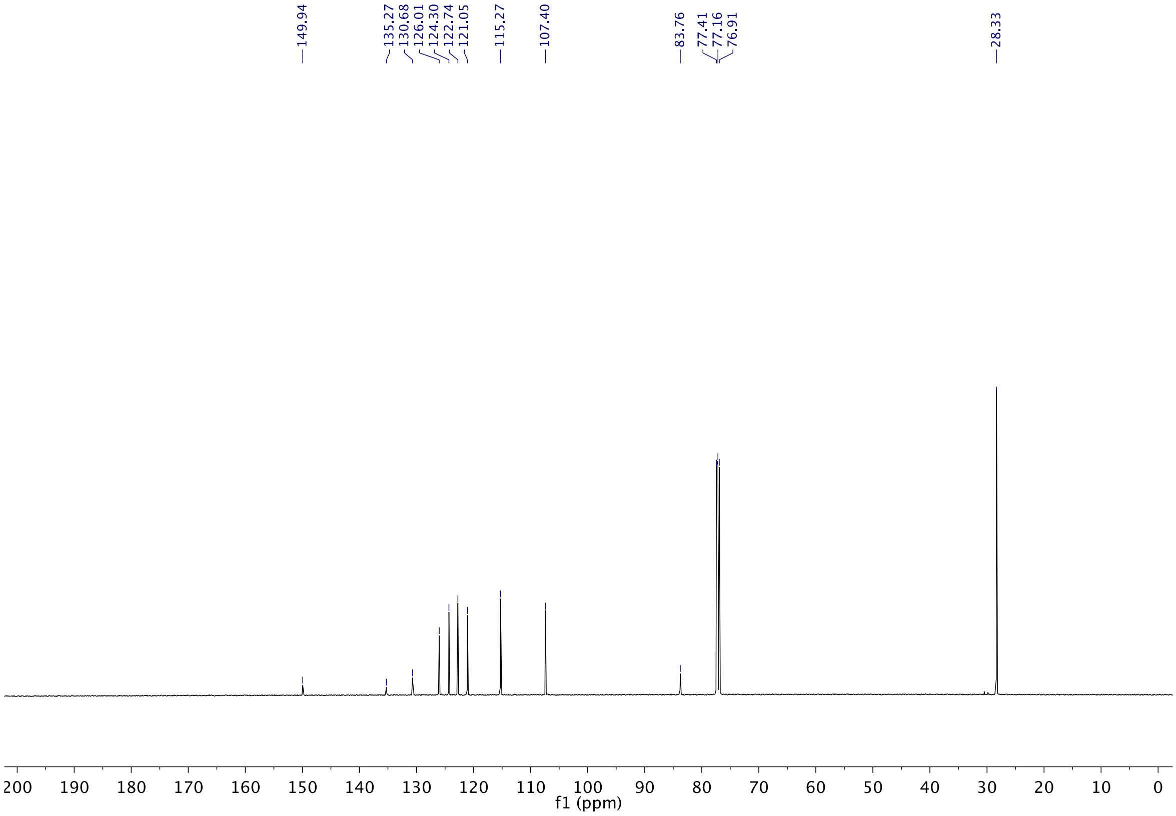

Supplement: Supplementary file 1 — Supporting Information [file ANIE-62-0-s001.pdf]
